# Correction: Beristain-Ruiz et al. Possible Association between Selected Tick-Borne Pathogen Prevalence and *Rhipicephalus sanguineus* sensu lato Infestation in Dogs from Juarez City (Chihuahua), Northwest Mexico–US Border. *Pathogens* 2022, *11*, 552

**DOI:** 10.3390/pathogens14020153

**Published:** 2025-02-05

**Authors:** Diana M. Beristain-Ruiz, Javier A. Garza-Hernández, Julio V. Figueroa-Millán, José J. Lira-Amaya, Andrés Quezada-Casasola, Susana Ordoñez-López, Stephanie Viridiana Laredo-Tiscareño, Beatriz Alvarado-Robles, Oliver R. Castillo-Luna, Adriana Floriano-López, Luis M. Hernández-Triana, Francisco Martínez-Ibáñez, Ramón Rivera-Barreno, Carlos A. Rodríguez-Alarcón

**Affiliations:** 1 Departamento de Ciencias Veterinarias, Universidad Autónoma de Ciudad Juárez, Anillo Envolvente y Estocolmo s/n Colonia Progresista AP 1729-D Cd. Juárez, Chihuahua 32310, Mexico; diana.beristain@uacj.mx (D.M.B.-R.);; 2 Departamento de Ciencias Químico-Biológicas, Universidad Autónoma de Ciudad Juárez, Anillo Envolvente y Estocolmo s/n Colonia Progresista AP 1729-D Cd. Juárez, Chihuahua 32310, Mexico; 3 CENID-Salud Animal e Inocuidad, Instituto Nacional de Investigaciones Forestales, Agrícolas y Pecuarias, Cuernavaca-Cuautla 8534, Progreso, Jiutepec 62574, Mexico; figueroa.julio@inifap.gob.mx (J.V.F.-M.);; 4 Virology Department, Vector Borne Diseases Research Group, Animal and Plant Health Agency (APHA), Woodham Lane, New Haw, Addlestone, Surrey KT15 3NB, UK; luis.hernandez-triana@apha.gov.uk; 5 Departamento de Ectoparásitos y Dípteros, CENAPA-SENASICA, Jiutepec 62550, Mexico

## Figures 1, 3, and 4 Captions

In the original publication [1], there was a mistake in the caption of Figures 1, 3 and 4. The reason for these mistakes is that more than 35 pictures were reviewed for this publication. A caption was written for each of them, and there was a confusion between the figure and the caption. The mistake for Figure 1 is the mention of “stigmata” in the mouth parts. The mistakes in Figure 3 are that both specimens are showing dorsally (so “ventral view” is incorrect) and that the second image (b) corresponds to a female, not a male. The mistakes in Figure 4 are that the inverted “U shape” should correspond to the genital aperture, not the anus, and that the word “stigmata” appears again. The correct captions appear below.

**Figure 1.** Ventral view of the oral apparatus of an *R. sanguineus* s. l. tick. The hypostome shows four rows of teeth as well as the depression of the four joints present in the pedipalp. (**a**) Photography was carried out with an Axio Zoom V6 microscope. (**b**) Photography was carried outobtained with an LSM 700 confocal scanning microscope.

**Figure 3.** Dorsal view of a male tick of *R. sanguineus* s. l. Observe the basis of the hexagonal-shaped gnathostome, with a complete scutum and the ocelli featuring at each end of the scutum. (**a**) *R. sanguineus* s. l. male. Photography was carried out with an Axio Zoom V6 microscope. (**b**) *R. sanguineus* s. l. female. Photography obtained with an LSM 700 confocal scanning microscope.

**Figure 4.** Rear ventral view of a semi-fully engorged female tick of *R. sanguineus* s. l. Observe the inverted U-shaped genital aperture and the spiracles below the fourth pair of legs. Festoons on the back side of the specimen can be seen. (**a**,**b**) Photography obtained with an Axio Zoom V6 microscope. (**c**) Photography obtained with a laser scanning confocal 700 (LSM 700).

The authors state that the scientific conclusions are unaffected. This correction was approved by the Academic Editor. The original publication has also been updated.
