# Supplementary material for: Possible Association between Selected Tick-Borne Pathogen Prevalence and Rhipicephalus sanguineus sensu lato Infestation in Dogs from Juarez City (Chihuahua), Northwest Mexico–US Border
Source: Pathogens. 2022 May 7;11(5):552. doi: 10.3390/pathogens11050552 (PMC9145599; doi:10.3390/pathogens11050552)
Supplement: Supplementary file 1 [file Table S1. Blast diagnostic seq. R. sanguineus.pdf › pathogens-1665365-supplementary/Table S1. Blast diagnostic seq. R. sanguineus.pdf]

RID: 6SEPCXY6016
Job Title:17 seq of Rhipicephalus sanguineus Juarez. Mexico

Program: BLASTN
Database: nt Nucleotide collection (nt)
Query #1: TKCJJ084-19|TKCJ012018G12|Rhipicephalus sanguineus|COI-5P Query ID: lcl|Query_26763 Length: 658

Sequences producing significant alignments:

Description	Scientific Name	Common Name	Taxid	Max Score	Total Score	Query cover	E Value	Per. Ident	Acc. Len	Accession
Rhipicephalus sanguineus sensu lato 'tropical lineage' isolate...	Rhipicephalu...	NA	2138177	1216	1216	100%	0.0	100.00	688	MF425995.1
Rhipicephalus sanguineus sensu lato 'tropical lineage' isolate...	Rhipicephalu...	NA	2138177	1216	1216	100%	0.0	100.00	703	MF425994.1
Rhipicephalus sanguineus sensu lato 'tropical lineage' isolate...	Rhipicephalu...	NA	2138177	1216	1216	100%	0.0	100.00	702	MF425993.1
Rhipicephalus sanguineus sensu lato 'tropical lineage' isolate...	Rhipicephalu...	NA	2138177	1216	1216	100%	0.0	100.00	693	MF425992.1
Rhipicephalus sanguineus haplotype RsanColSinSP cytochrome c...	Rhipicephalu...	brown dog tick	34632	1216	1216	100%	0.0	100.00	690	KT906183.1
Rhipicephalus sanguineus haplotype RsanColSanM cytochrome c...	Rhipicephalu...	brown dog tick	34632	1216	1216	100%	0.0	100.00	690	KT906182.1
Rhipicephalus sanguineus isolate Q3 mitochondrion, complete...	Rhipicephalu...	brown dog tick	34632	1216	1216	100%	0.0	100.00	14711	OM368327.1
Rhipicephalus sanguineus isolate C19 mitochondrion, complete...	Rhipicephalu...	brown dog tick	34632	1216	1216	100%	0.0	100.00	14714	OM368323.1
Rhipicephalus sanguineus isolate A44 mitochondrion, complete...	Rhipicephalu...	brown dog tick	34632	1216	1216	100%	0.0	100.00	14713	OM368322.1
Rhipicephalus sanguineus isolate Guangxi cytochrome oxidase...	Rhipicephalu...	brown dog tick	34632	1216	1216	100%	0.0	100.00	707	JQ737084.1
Rhipicephalus sanguineus cytochrome oxidase subunit 1 (COI)...	Rhipicephalu...	brown dog tick	34632	1216	1216	100%	0.0	100.00	658	KF437543.1
Rhipicephalus linnaei isolate LAO32-2 clone JS5930...	Rhipicephalu...	NA	2138177	1216	1216	100%	0.0	100.00	14715	MW429383.1
Rhipicephalus linnaei mitochondrion, complete sequence	Rhipicephalu...	NA	2138177	1216	1216	100%	0.0	100.00	14711	NC_060409.1

Ixodidae sp. sc_01082 cytochrome oxidase subunit 1 (COI) gene,... Ixodidae sp.... NA 1901042 1210 1210 100% 0.0 99.85 658 KX053537.1
Rhipicephalus sanguineus haplotype RsanColCorL cytochrome c...
Rhipicephalu... brown dog tick 34632 1210 1210 100% 0.0 99.85 690 KT906185.1
Rhipicephalus sanguineus haplotype RsanColTolC cytochrome c...
Rhipicephalu... brown dog tick 34632 1210 1210 100% 0.0 99.85 690 KT906184.1
Rhipicephalus sanguineus voucher CAS-T065 cytochrome oxidase...
Rhipicephalu... brown dog tick 34632 1210 1210 100% 0.0 99.85 658 KF200113.1
Rhipicephalus linnaei isolate SC931-1 clone JS5927...
Rhipicephalu... NA 2138177 1210 1210 100% 0.0 99.85 14717 MW429382.1
Rhipicephalus sanguineus voucher DO-311 cytochrome oxidase...
Rhipicephalu... brown dog tick 34632 1205 1205 99% 0.0 99.85 658 KX360367.1
Rhipicephalus sanguineus haplotype RsanColSamCM cytochrome c...
Rhipicephalu... brown dog tick 34632 1205 1205 100% 0.0 99.70 690 KT906186.1
Rhipicephalus sanguineus voucher GRP5-154 cytochrome oxidase...
Rhipicephalu... brown dog tick 34632 1205 1205 100% 0.0 99.70 658 KF200112.1
Rhipicephalus sanguineus mitochondrion, complete genome
Rhipicephalu... brown dog tick 34632 1205 1205 100% 0.0 99.70 14714 JX416325.1
Rhipicephalus sanguineus isolate T95D15 cytochrome c oxidase...
Rhipicephalu... brown dog tick 34632 1192 1192 98% 0.0 100.00 661 MZ401443.1
Rhipicephalus sanguineus isolate T81D7 cytochrome c oxidase...
Rhipicephalu... brown dog tick 34632 1192 1192 98% 0.0 100.00 661 MZ401441.1
Rhipicephalus sanguineus isolate T79D2 cytochrome c oxidase...
Rhipicephalu... brown dog tick 34632 1192 1192 98% 0.0 99.85 664 MZ401440.1
Rhipicephalus sanguineus isolate WZ004 cytochrome oxidase...
Rhipicephalu... brown dog tick 34632 1190 1190 97% 0.0 100.00 664 MG969507.1
Rhipicephalus sanguineus voucher GRP5-150 cytochrome oxidase...
Rhipicephalu... brown dog tick 34632 1188 1188 98% 0.0 99.69 649 KF200084.1
Rhipicephalus sanguineus isolate LIC6084B cytochrome oxidase...
Rhipicephalu... brown dog tick 34632 1186 1186 97% 0.0 100.00 768 KX383817.1
Rhipicephalus sanguineus isolate T91D13 cytochrome c oxidase...
Rhipicephalu... brown dog tick 34632 1186 1186 97% 0.0 100.00 657 MZ401442.1
Rhipicephalus sanguineus isolate T63D1 cytochrome c oxidase...
Rhipicephalu... brown dog tick 34632 1186 1186 97% 0.0 100.00 661 MZ401438.1
Rhipicephalus sanguineus isolate WZ003 cytochrome oxidase...
Rhipicephalu... brown dog tick 34632 1184 1184 97% 0.0 99.84 664 MG969506.1

Rhipicephalus sanguineus isolate WZ002 cytochrome oxidase...
Rhipicephalu... brown dog tick 34632 1184 1184 97% 0.0 99.84 664
MG969505.1
Rhipicephalus sanguineus isolate WZ001 cytochrome oxidase...
Rhipicephalu... brown dog tick 34632 1184 1184 97% 0.0 99.84 664
MG969504.1
Rhipicephalus sanguineus voucher CAS-T066 cytochrome oxidase...
Rhipicephalu... brown dog tick 34632 1179 1179 97% 0.0 99.69 644
KF200096.1
Rhipicephalus linnaei voucher P1/22_18-4 cytochrome c oxidase...
Rhipicephalu... NA 2138177 1177 1177 100% 0.0 98.94 658
OM984983.1
Rhipicephalus sanguineus isolate GHM1 cytochrome oxidase subun...
Rhipicephalu... brown dog tick 34632 1177 1177 96% 0.0 100.00 802
JX416302.1
Rhipicephalus sanguineus isolate CHM1 cytochrome oxidase subun...
Rhipicephalu... brown dog tick 34632 1177 1177 96% 0.0 100.00 802
JX416298.1
Rhipicephalus sanguineus voucher AMMS-RS-5 cytochrome c oxidas...
Rhipicephalu... brown dog tick 34632 1177 1177 97% 0.0 99.69 670
HM193873.1
Rhipicephalus sanguineus isolate LIC6767D cytochrome oxidase...
Rhipicephalu... brown dog tick 34632 1175 1175 97% 0.0 99.69 768
KX383820.1
Rhipicephalus sanguineus isolate LIC5766G cytochrome oxidase...
Rhipicephalu... brown dog tick 34632 1171 1171 97% 0.0 99.53 724
KX383816.1
Rhipicephalus linnaei voucher P1/22_18-5 cytochrome c oxidase...
Rhipicephalu... NA 2138177 1171 1171 100% 0.0 98.78 658
OM984984.1
Rhipicephalus linnaei voucher P1/22_8-1 cytochrome c oxidase...
Rhipicephalu... NA 2138177 1171 1171 100% 0.0 98.78 658
OM984974.1
Rhipicephalus linnaei voucher P1/22_6-2 cytochrome c oxidase...
Rhipicephalu... NA 2138177 1171 1171 100% 0.0 98.78 658
OM984973.1
Rhipicephalus sanguineus isolate SZM2 cytochrome oxidase subun...
Rhipicephalu... brown dog tick 34632 1171 1171 96% 0.0 99.84 802
JX416308.1
Rhipicephalus sanguineus isolate GTF3 cytochrome oxidase subun...
Rhipicephalu... brown dog tick 34632 1171 1171 96% 0.0 99.84 802
JX416304.1
Rhipicephalus sanguineus isolate GBM1 cytochrome oxidase subun...
Rhipicephalu... brown dog tick 34632 1171 1171 96% 0.0 99.84 802
JX416300.1
Rhipicephalus sanguineus isolate GBF1 cytochrome oxidase subun...
Rhipicephalu... brown dog tick 34632 1171 1171 96% 0.0 99.84 802
JX416299.1
Rhipicephalus sanguineus isolate CHF1 cytochrome oxidase subun...
Rhipicephalu... brown dog tick 34632 1171 1171 96% 0.0 99.84 802
JX416297.1
Rhipicephalus sanguineus isolate DGM1 cytochrome oxidase subun...
Rhipicephalu... brown dog tick 34632 1171 1171 96% 0.0 99.84 802
JX416296.1

```
Rhipicephalus sanguineus isolate FSF1 cytochrome oxidase subun...
Rhipicephalu... brown dog tick  34632      1171   1171  96%   0.0   99.84  802
     JX416293.1
Rhipicephalus sanguineus isolate T78D2 cytochrome c oxidase...
Rhipicephalu... brown dog tick  34632      1171   1171  96%   0.0   100.00 650
     MZ401439.1
Rhipicephalus sanguineus isolate LIC5762 cytochrome oxidase...
Rhipicephalu... brown dog tick  34632      1166   1166  96%   0.0   99.69  763
     KX383802.1
Rhipicephalus linnaei voucher P1/22_16-1 cytochrome c oxidase...
Rhipicephalu... NA              2138177    1166   1166  100%  0.0   98.63  658
     OM984979.1
Rhipicephalus linnaei voucher P1/22_1-1 cytochrome c oxidase...
Rhipicephalu... NA              2138177    1166   1166  100%  0.0   98.63  658
     OM984968.1
Rhipicephalus sanguineus isolate DGF1 cytochrome oxidase subun...
Rhipicephalu... brown dog tick  34632      1166   1166  96%   0.0   99.69  802
     JX416295.1
Rhipicephalus sanguineus isolate FSM1 cytochrome oxidase subun...
Rhipicephalu... brown dog tick  34632      1166   1166  96%   0.0   99.69  802
     JX416294.1
Rhipicephalus sanguineus isolate sanguineus0926 cytochrome...
Rhipicephalu... brown dog tick  34632      1162   1162  99%   0.0   98.77  658
     MH513252.1
Rhipicephalus linnaei voucher P1/22_19-1 cytochrome c oxidase...
Rhipicephalu... NA              2138177    1160   1160  100%  0.0   98.48  658
     OM984985.1
Rhipicephalus linnaei voucher P1/22_14-1 cytochrome c oxidase...
Rhipicephalu... NA              2138177    1160   1160  100%  0.0   98.48  658
     OM984978.1
Rhipicephalus linnaei voucher P1/22_13-1 cytochrome c oxidase...
Rhipicephalu... NA              2138177    1160   1160  100%  0.0   98.48  658
     OM984977.1
Rhipicephalus linnaei voucher P1/22_10-1 cytochrome c oxidase...
Rhipicephalu... NA              2138177    1160   1160  100%  0.0   98.48  658
     OM984975.1
Rhipicephalus linnaei voucher P1/22_5-1 cytochrome c oxidase...
Rhipicephalu... NA              2138177    1160   1160  100%  0.0   98.48  658
     OM984971.1
Rhipicephalus linnaei voucher P1/22_4-1 cytochrome c oxidase...
Rhipicephalu... NA              2138177    1160   1160  100%  0.0   98.48  658
     OM984970.1
Rhipicephalus linnaei voucher P1/22_3-1 cytochrome c oxidase...
Rhipicephalu... NA              2138177    1160   1160  100%  0.0   98.48  658
     OM984969.1
Rhipicephalus sanguineus isolate GHF1 cytochrome oxidase subun...
Rhipicephalu... brown dog tick  34632      1160   1160  96%   0.0   99.53  802
     JX416301.1
Rhipicephalus sanguineus isolate Tropical lineage RJ cytochrom...
Rhipicephalu... brown dog tick  34632      1155   1155  95%   0.0   99.68  642
     MT010523.1
Rhipicephalus linnaei voucher P1/22_21-1 cytochrome c oxidase...
Rhipicephalu... NA              2138177    1155   1155  100%  0.0   98.33  658
     OM984988.1
```

```
Rhipicephalus linnaei voucher P1/22_12-1 cytochrome c oxidase...
Rhipicephalu... NA              2138177    1155   1155  100%  0.0   98.33  658
     OM984976.1
Rhipicephalus sanguineus isolate GTF2 cytochrome oxidase subun...
Rhipicephalu... brown dog tick  34632      1155   1155  96%   0.0   99.37  802
     JX416303.1
Rhipicephalus sanguineus sensu lato 'tropical lineage' isolate...
Rhipicephalu... NA              2138177    1153   1153  98%   0.0   98.77  826
     MF426003.1
Rhipicephalus sanguineus voucher AMMS-RS-4 cytochrome c oxidas...
Rhipicephalu... brown dog tick  34632      1146   1146  96%   0.0   99.21  665
     HM193874.1
Rhipicephalus sanguineus isolate Abidjan cytochrome oxidase...
Rhipicephalu... brown dog tick  34632      1140   1140  94%   0.0   99.84  630
     KX757914.1
Rhipicephalus sanguineus isolate LIC5960 cytochrome oxidase...
Rhipicephalu... brown dog tick  34632      1136   1136  97%   0.0   98.60  768
     KX383800.1
Rhipicephalus sanguineus isolate LIC5682B cytochrome oxidase...
Rhipicephalu... brown dog tick  34632      1131   1131  93%   0.0   99.68  744
     KX383796.1
Rhipicephalus sanguineus isolate LIC5958B cytochrome oxidase...
Rhipicephalu... brown dog tick  34632      1127   1127  96%   0.0   98.59  698
     KX383798.1
Rhipicephalus sanguineus isolate LIC5584D cytochrome oxidase...
Rhipicephalu... brown dog tick  34632      1125   1125  97%   0.0   98.29  768
     KX383814.1
Rhipicephalus sanguineus isolate LIC5755 cytochrome oxidase...
Rhipicephalu... brown dog tick  34632      1122   1122  96%   0.0   98.43  763
     KX383801.1
Rhipicephalus sanguineus isolate 400 cytochrome c oxidase...
Rhipicephalu... brown dog tick  34632      1120   1120  97%   0.0   98.28  657
     MW558150.1
Rhipicephalus linnaei isolate C2-T1 cytochrome c oxidase subun...
Rhipicephalu... NA              2138177    1116   1116  91%   0.0   100.00 604
     MZ726445.1
Rhipicephalus linnaei isolate C1-T1 cytochrome c oxidase subun...
Rhipicephalu... NA              2138177    1116   1116  91%   0.0   100.00 604
     MZ726444.1
Rhipicephalus linnaei isolate B15-T1 cytochrome c oxidase...
Rhipicephalu... NA              2138177    1116   1116  91%   0.0   100.00 604
     MZ726443.1
Rhipicephalus linnaei isolate B12-T1 cytochrome c oxidase...
Rhipicephalu... NA              2138177    1116   1116  91%   0.0   100.00 604
     MZ726442.1
Rhipicephalus linnaei isolate B8-T1 cytochrome c oxidase subun...
Rhipicephalu... NA              2138177    1116   1116  91%   0.0   100.00 604
     MZ726441.1
Rhipicephalus linnaei isolate B7-T3 cytochrome c oxidase subun...
Rhipicephalu... NA              2138177    1116   1116  91%   0.0   100.00 604
     MZ726440.1
Rhipicephalus linnaei isolate B7-T2 cytochrome c oxidase subun...
Rhipicephalu... NA              2138177    1116   1116  91%   0.0   100.00 604
     MZ726439.1
```

```
Rhipicephalus linnaei isolate B7-T1 cytochrome c oxidase subun...
Rhipicephalu... NA             2138177    1116   1116  91%   0.0   100.00 604
     MZ726438.1
Rhipicephalus linnaei isolate B6-T1 cytochrome c oxidase subun...
Rhipicephalu... NA             2138177    1116   1116  91%   0.0   100.00 604
     MZ726437.1
Rhipicephalus linnaei isolate B4-T1 cytochrome c oxidase subun...
Rhipicephalu... NA             2138177    1116   1116  91%   0.0   100.00 604
     MZ726436.1
Rhipicephalus linnaei isolate B3-T1 cytochrome c oxidase subun...
Rhipicephalu... NA             2138177    1116   1116  91%   0.0   100.00 604
     MZ726435.1
Rhipicephalus linnaei isolate B2-T1 cytochrome c oxidase subun...
Rhipicephalu... NA             2138177    1116   1116  91%   0.0   100.00 604
     MZ726434.1
Rhipicephalus linnaei isolate A7-T1 cytochrome c oxidase subun...
Rhipicephalu... NA             2138177    1116   1116  91%   0.0   100.00 604
     MZ726433.1
Rhipicephalus linnaei isolate A6-T1 cytochrome c oxidase subun...
Rhipicephalu... NA             2138177    1116   1116  91%   0.0   100.00 604
     MZ726432.1
Rhipicephalus linnaei isolate A5-T1 cytochrome c oxidase subun...
Rhipicephalu... NA             2138177    1116   1116  91%   0.0   100.00 604
     MZ726431.1
Rhipicephalus linnaei isolate A2-T1 cytochrome c oxidase subun...
Rhipicephalu... NA             2138177    1116   1116  91%   0.0   100.00 604
     MZ726430.1
Rhipicephalus linnaei isolate B9-T1 cytochrome c oxidase subun...
Rhipicephalu... NA             2138177    1110   1110  91%   0.0   99.83  604
     MZ726449.1
Rhipicephalus linnaei isolate B18-T1 cytochrome c oxidase...
Rhipicephalu... NA             2138177    1110   1110  91%   0.0   99.83  604
     MZ726429.1
Rhipicephalus linnaei isolate B17-T1 cytochrome c oxidase...
Rhipicephalu... NA             2138177    1110   1110  91%   0.0   99.83  604
     MZ726428.1
Rhipicephalus linnaei isolate B16-T1 cytochrome c oxidase...
Rhipicephalu... NA             2138177    1110   1110  91%   0.0   99.83  604
     MZ726427.1
Rhipicephalus linnaei isolate B1-T1 cytochrome c oxidase subun...
Rhipicephalu... NA             2138177    1110   1110  91%   0.0   99.83  604
     MZ726426.1
Rhipicephalus sanguineus voucher DO-306 cytochrome oxidase...
Rhipicephalu... brown dog tick  34632     1092   1092  91%   0.0   99.33  638
     KX360338.1
```

Alignments:

>Rhipicephalus sanguineus sensu lato 'tropical lineage' isolate A3021 cytochrome c oxidase subunit I (COI) gene, partial cds; mitochondrial
Sequence ID: MF425995.1 Length: 688
Range 1: 18 to 675

```
Score:1216 bits(658), Expect:0.0,
Identities:658/658(100%),  Gaps:0/658(0%), Strand: Plus/Plus

Query  1    GACAATATACTTAATTTTTGGAGCATGATCCGGAATACTAGGATTAAGAATAAGAATATT  60
            ||||||||||||||||||||||||||||||||||||||||||||||||||||||||||||
Sbjct  18   GACAATATACTTAATTTTTGGAGCATGATCCGGAATACTAGGATTAAGAATAAGAATATT  77

Query  61   AATCCGCATAGAATTAGGTCAACCAGGAACTTTAATTGGAAACGATCAAATTTATAATGT  120
            ||||||||||||||||||||||||||||||||||||||||||||||||||||||||||||
Sbjct  78   AATCCGCATAGAATTAGGTCAACCAGGAACTTTAATTGGAAACGATCAAATTTATAATGT  137

Query  121  AATTGTAACAGCACATGCATTTATTATAAtttttttATAGTTATACCAATTATAATCGG  180
            ||||||||||||||||||||||||||||||||||||||||||||||||||||||||||||
Sbjct  138  AATTGTAACAGCACATGCATTTATTATAATTTTTTTATAGTTATACCAATTATAATCGG  197

Query  181  AGGATTTGGAAATTGATTAGTTCCTATTATACTAGGAGCTCCAGATATAGCATTTCCACG  240
            ||||||||||||||||||||||||||||||||||||||||||||||||||||||||||||
Sbjct  198  AGGATTTGGAAATTGATTAGTTCCTATTATACTAGGAGCTCCAGATATAGCATTTCCACG  257

Query  241  AATAAATAATATAAGATTTTGGTTACTTCCTCCTTCTCTATTTCTACTAATTAATTCTTC  300
            ||||||||||||||||||||||||||||||||||||||||||||||||||||||||||||
Sbjct  258  AATAAATAATATAAGATTTTGGTTACTTCCTCCTTCTCTATTTCTACTAATTAATTCTTC  317

Query  301  ATTAATCGAATCAGGAGCAGGTACAGGGTGAACAGTTTAcccccccTTATCATCAAATTT  360
            ||||||||||||||||||||||||||||||||||||||||||||||||||||||||||||
Sbjct  318  ATTAATCGAATCAGGAGCAGGTACAGGGTGAACAGTTTACCCCCCCTTATCATCAAATTT  377

Query  361  ATCACACTACGGACCATCAGTAGATTTAGCTATTTTTTCTCTGCATCTTGCAGGTGCTTC  420
            ||||||||||||||||||||||||||||||||||||||||||||||||||||||||||||
Sbjct  378  ATCACACTACGGACCATCAGTAGATTTAGCTATTTTTTCTCTGCATCTTGCAGGTGCTTC  437

Query  421  TTCAATTTTAGGTGCAATCAATTTCATTACAACTATTGTAAATATACGATCCATTGGAAT  480
            ||||||||||||||||||||||||||||||||||||||||||||||||||||||||||||
Sbjct  438  TTCAATTTTAGGTGCAATCAATTTCATTACAACTATTGTAAATATACGATCCATTGGAAT  497

Query  481  AACAATAGAACGAATACCATTATTTGTATGATCTGTTTTAATTACTGCAATTTTATTATT  540
            ||||||||||||||||||||||||||||||||||||||||||||||||||||||||||||
Sbjct  498  AACAATAGAACGAATACCATTATTTGTATGATCTGTTTTAATTACTGCAATTTTATTATT  557

Query  541  ATTATCTTTACCAGTCTTAGCTGGCGCAATTACAATATTATTAACTGATCGAAACTTTAA  600
            ||||||||||||||||||||||||||||||||||||||||||||||||||||||||||||
Sbjct  558  ATTATCTTTACCAGTCTTAGCTGGCGCAATTACAATATTATTAACTGATCGAAACTTTAA  617

Query  601  TACATCATTCTTTGACCCTTCAGGAGGAGGTGACCCAATTCTATATCAACATTTATTT  658
            ||||||||||||||||||||||||||||||||||||||||||||||||||||||||||
Sbjct  618  TACATCATTCTTTGACCCTTCAGGAGGAGGTGACCCAATTCTATATCAACATTTATTT  675
```

>Rhipicephalus sanguineus sensu lato 'tropical lineage' isolate A3015 cytochrome c oxidase subunit I (COI) gene, partial cds; mitochondrial
Sequence ID: MF425994.1 Length: 703
>Rhipicephalus sanguineus sensu lato 'tropical lineage' isolate A3022 cytochrome

c oxidase subunit I (COI) gene, partial cds; mitochondrial
Sequence ID: MF425996.1 Length: 703
Range 1: 20 to 677

Score:1216 bits(658), Expect:0.0,
Identities:658/658(100%), Gaps:0/658(0%), Strand: Plus/Plus

```
Query  1    GACAATATACTTAATTTTTGGAGCATGATCCGGAATACTAGGATTAAGAATAAGAATATT  60
            ||||||||||||||||||||||||||||||||||||||||||||||||||||||||||||
Sbjct  20   GACAATATACTTAATTTTTGGAGCATGATCCGGAATACTAGGATTAAGAATAAGAATATT  79

Query  61   AATCCGCATAGAATTAGGTCAACCAGGAACTTTAATTGGAAACGATCAAATTTATAATGT  120
            ||||||||||||||||||||||||||||||||||||||||||||||||||||||||||||
Sbjct  80   AATCCGCATAGAATTAGGTCAACCAGGAACTTTAATTGGAAACGATCAAATTTATAATGT  139

Query  121  AATTGTAACAGCACATGCATTTATTATAAttttttttATAGTTATACCAATTATAATCGG  180
            ||||||||||||||||||||||||||||||||||||||||||||||||||||||||||||
Sbjct  140  AATTGTAACAGCACATGCATTTATTATAATTTTTTTTATAGTTATACCAATTATAATCGG  199

Query  181  AGGATTTGGAAATTGATTAGTTCCTATTATACTAGGAGCTCCAGATATAGCATTTCCACG  240
            ||||||||||||||||||||||||||||||||||||||||||||||||||||||||||||
Sbjct  200  AGGATTTGGAAATTGATTAGTTCCTATTATACTAGGAGCTCCAGATATAGCATTTCCACG  259

Query  241  AATAAATAATATAAGATTTTGGTTACTTCCTCCTTCTCTATTTCTACTAATTAATTCTTC  300
            ||||||||||||||||||||||||||||||||||||||||||||||||||||||||||||
Sbjct  260  AATAAATAATATAAGATTTTGGTTACTTCCTCCTTCTCTATTTCTACTAATTAATTCTTC  319

Query  301  ATTAATCGAATCAGGAGCAGGTACAGGGTGAACAGTTTAcccccccTTATCATCAAATTT  360
            ||||||||||||||||||||||||||||||||||||||||||||||||||||||||||||
Sbjct  320  ATTAATCGAATCAGGAGCAGGTACAGGGTGAACAGTTTACCCCCCCTTATCATCAAATTT  379

Query  361  ATCACACTACGGACCATCAGTAGATTTAGCTATTTTTTCTCTGCATCTTGCAGGTGCTTC  420
            ||||||||||||||||||||||||||||||||||||||||||||||||||||||||||||
Sbjct  380  ATCACACTACGGACCATCAGTAGATTTAGCTATTTTTTCTCTGCATCTTGCAGGTGCTTC  439

Query  421  TTCAATTTTAGGTGCAATCAATTTCATTACAACTATTGTAAATATACGATCCATTGGAAT  480
            ||||||||||||||||||||||||||||||||||||||||||||||||||||||||||||
Sbjct  440  TTCAATTTTAGGTGCAATCAATTTCATTACAACTATTGTAAATATACGATCCATTGGAAT  499

Query  481  AACAATAGAACGAATACCATTATTTGTATGATCTGTTTTAATTACTGCAATTTTATTATT  540
            ||||||||||||||||||||||||||||||||||||||||||||||||||||||||||||
Sbjct  500  AACAATAGAACGAATACCATTATTTGTATGATCTGTTTTAATTACTGCAATTTTATTATT  559

Query  541  ATTATCTTTACCAGTCTTAGCTGGCGCAATTACAATATTATTAACTGATCGAAACTTTAA  600
            ||||||||||||||||||||||||||||||||||||||||||||||||||||||||||||
Sbjct  560  ATTATCTTTACCAGTCTTAGCTGGCGCAATTACAATATTATTAACTGATCGAAACTTTAA  619

Query  601  TACATCATTCTTTGACCCTTCAGGAGGAGGTGACCCAATTCTATATCAACATTTATTT  658
            ||||||||||||||||||||||||||||||||||||||||||||||||||||||||||
Sbjct  620  TACATCATTCTTTGACCCTTCAGGAGGAGGTGACCCAATTCTATATCAACATTTATTT  677
```

```
>Rhipicephalus sanguineus sensu lato 'tropical lineage' isolate A3014 cytochrome
c oxidase subunit I (COI) gene, partial cds; mitochondrial
Sequence ID: MF425993.1 Length: 702
Range 1: 20 to 677

Score:1216 bits(658), Expect:0.0,
Identities:658/658(100%),  Gaps:0/658(0%), Strand: Plus/Plus

Query  1    GACAATATACTTAATTTTTGGAGCATGATCCGGAATACTAGGATTAAGAATAAGAATATT  60
            ||||||||||||||||||||||||||||||||||||||||||||||||||||||||||||
Sbjct  20   GACAATATACTTAATTTTTGGAGCATGATCCGGAATACTAGGATTAAGAATAAGAATATT  79

Query  61   AATCCGCATAGAATTAGGTCAACCAGGAACTTTAATTGGAAACGATCAAATTTATAATGT  120
            ||||||||||||||||||||||||||||||||||||||||||||||||||||||||||||
Sbjct  80   AATCCGCATAGAATTAGGTCAACCAGGAACTTTAATTGGAAACGATCAAATTTATAATGT  139

Query  121  AATTGTAACAGCACATGCATTTATTATAAttttttttATAGTTATACCAATTATAATCGG  180
            ||||||||||||||||||||||||||||||||||||||||||||||||||||||||||||
Sbjct  140  AATTGTAACAGCACATGCATTTATTATAATTTTTTTTATAGTTATACCAATTATAATCGG  199

Query  181  AGGATTTGGAAATTGATTAGTTCCTATTATACTAGGAGCTCCAGATATAGCATTTCCACG  240
            ||||||||||||||||||||||||||||||||||||||||||||||||||||||||||||
Sbjct  200  AGGATTTGGAAATTGATTAGTTCCTATTATACTAGGAGCTCCAGATATAGCATTTCCACG  259

Query  241  AATAAATAATATAAGATTTTGGTTACTTCCTCCTTCTCTATTTCTACTAATTAATTCTTC  300
            ||||||||||||||||||||||||||||||||||||||||||||||||||||||||||||
Sbjct  260  AATAAATAATATAAGATTTTGGTTACTTCCTCCTTCTCTATTTCTACTAATTAATTCTTC  319

Query  301  ATTAATCGAATCAGGAGCAGGTACAGGGTGAACAGTTTAcccccccTTATCATCAAATTT  360
            ||||||||||||||||||||||||||||||||||||||||||||||||||||||||||||
Sbjct  320  ATTAATCGAATCAGGAGCAGGTACAGGGTGAACAGTTTACCCCCCCTTATCATCAAATTT  379

Query  361  ATCACACTACGGACCATCAGTAGATTTAGCTATTTTTTCTCTGCATCTTGCAGGTGCTTC  420
            ||||||||||||||||||||||||||||||||||||||||||||||||||||||||||||
Sbjct  380  ATCACACTACGGACCATCAGTAGATTTAGCTATTTTTTCTCTGCATCTTGCAGGTGCTTC  439

Query  421  TTCAATTTTAGGTGCAATCAATTTCATTACAACTATTGTAAATATACGATCCATTGGAAT  480
            ||||||||||||||||||||||||||||||||||||||||||||||||||||||||||||
Sbjct  440  TTCAATTTTAGGTGCAATCAATTTCATTACAACTATTGTAAATATACGATCCATTGGAAT  499

Query  481  AACAATAGAACGAATACCATTATTTGTATGATCTGTTTTAATTACTGCAATTTTATTATT  540
            ||||||||||||||||||||||||||||||||||||||||||||||||||||||||||||
Sbjct  500  AACAATAGAACGAATACCATTATTTGTATGATCTGTTTTAATTACTGCAATTTTATTATT  559

Query  541  ATTATCTTTACCAGTCTTAGCTGGCGCAATTACAATATTATTAACTGATCGAAACTTTAA  600
            ||||||||||||||||||||||||||||||||||||||||||||||||||||||||||||
Sbjct  560  ATTATCTTTACCAGTCTTAGCTGGCGCAATTACAATATTATTAACTGATCGAAACTTTAA  619

Query  601  TACATCATTCTTTGACCCTTCAGGAGGAGGTGACCCAATTCTATATCAACATTTATTT  658
            ||||||||||||||||||||||||||||||||||||||||||||||||||||||||||
Sbjct  620  TACATCATTCTTTGACCCTTCAGGAGGAGGTGACCCAATTCTATATCAACATTTATTT  677
```

>Rhipicephalus sanguineus sensu lato 'tropical lineage' isolate A3013 cytochrome c oxidase subunit I (COI) gene, partial cds; mitochondrial
Sequence ID: MF425992.1 Length: 693
Range 1: 20 to 677

Score:1216 bits(658), Expect:0.0,
Identities:658/658(100%), Gaps:0/658(0%), Strand: Plus/Plus

```
Query  1    GACAATATACTTAATTTTTGGAGCATGATCCGGAATACTAGGATTAAGAATAAGAATATT  60
            ||||||||||||||||||||||||||||||||||||||||||||||||||||||||||||
Sbjct  20   GACAATATACTTAATTTTTGGAGCATGATCCGGAATACTAGGATTAAGAATAAGAATATT  79

Query  61   AATCCGCATAGAATTAGGTCAACCAGGAACTTTAATTGGAAACGATCAAATTTATAATGT  120
            ||||||||||||||||||||||||||||||||||||||||||||||||||||||||||||
Sbjct  80   AATCCGCATAGAATTAGGTCAACCAGGAACTTTAATTGGAAACGATCAAATTTATAATGT  139

Query  121  AATTGTAACAGCACATGCATTTATTATAAttttttttATAGTTATACCAATTATAATCGG  180
            ||||||||||||||||||||||||||||||||||||||||||||||||||||||||||||
Sbjct  140  AATTGTAACAGCACATGCATTTATTATAATTTTTTTTATAGTTATACCAATTATAATCGG  199

Query  181  AGGATTTGGAAATTGATTAGTTCCTATTATACTAGGAGCTCCAGATATAGCATTTCCACG  240
            ||||||||||||||||||||||||||||||||||||||||||||||||||||||||||||
Sbjct  200  AGGATTTGGAAATTGATTAGTTCCTATTATACTAGGAGCTCCAGATATAGCATTTCCACG  259

Query  241  AATAAATAATATAAGATTTTGGTTACTTCCTCCTTCTCTATTTCTACTAATTAATTCTTC  300
            ||||||||||||||||||||||||||||||||||||||||||||||||||||||||||||
Sbjct  260  AATAAATAATATAAGATTTTGGTTACTTCCTCCTTCTCTATTTCTACTAATTAATTCTTC  319

Query  301  ATTAATCGAATCAGGAGCAGGTACAGGGTGAACAGTTTAcccccccTTATCATCAAATTT  360
            ||||||||||||||||||||||||||||||||||||||||||||||||||||||||||||
Sbjct  320  ATTAATCGAATCAGGAGCAGGTACAGGGTGAACAGTTTACCCCCCCTTATCATCAAATTT  379

Query  361  ATCACACTACGGACCATCAGTAGATTTAGCTATTTTTTCTCTGCATCTTGCAGGTGCTTC  420
            ||||||||||||||||||||||||||||||||||||||||||||||||||||||||||||
Sbjct  380  ATCACACTACGGACCATCAGTAGATTTAGCTATTTTTTCTCTGCATCTTGCAGGTGCTTC  439

Query  421  TTCAATTTTAGGTGCAATCAATTTCATTACAACTATTGTAAATATACGATCCATTGGAAT  480
            ||||||||||||||||||||||||||||||||||||||||||||||||||||||||||||
Sbjct  440  TTCAATTTTAGGTGCAATCAATTTCATTACAACTATTGTAAATATACGATCCATTGGAAT  499

Query  481  AACAATAGAACGAATACCATTATTTGTATGATCTGTTTTAATTACTGCAATTTTATTATT  540
            ||||||||||||||||||||||||||||||||||||||||||||||||||||||||||||
Sbjct  500  AACAATAGAACGAATACCATTATTTGTATGATCTGTTTTAATTACTGCAATTTTATTATT  559

Query  541  ATTATCTTTACCAGTCTTAGCTGGCGCAATTACAATATTATTAACTGATCGAAACTTTAA  600
            ||||||||||||||||||||||||||||||||||||||||||||||||||||||||||||
Sbjct  560  ATTATCTTTACCAGTCTTAGCTGGCGCAATTACAATATTATTAACTGATCGAAACTTTAA  619

Query  601  TACATCATTCTTTGACCCTTCAGGAGGAGGTGACCCAATTCTATATCAACATTTATTT  658
            ||||||||||||||||||||||||||||||||||||||||||||||||||||||||||
Sbjct  620  TACATCATTCTTTGACCCTTCAGGAGGAGGTGACCCAATTCTATATCAACATTTATTT  677
```

>Rhipicephalus sanguineus haplotype RsanColSinSP cytochrome c oxidase subunit I (COX1) gene, partial cds; mitochondrial
Sequence ID: KT906183.1 Length: 690
Range 1: 18 to 675

Score:1216 bits(658), Expect:0.0,
Identities:658/658(100%), Gaps:0/658(0%), Strand: Plus/Plus

```
Query  1    GACAATATACTTAATTTTTGGAGCATGATCCGGAATACTAGGATTAAGAATAAGAATATT  60
            ||||||||||||||||||||||||||||||||||||||||||||||||||||||||||||
Sbjct  18   GACAATATACTTAATTTTTGGAGCATGATCCGGAATACTAGGATTAAGAATAAGAATATT  77

Query  61   AATCCGCATAGAATTAGGTCAACCAGGAACTTTAATTGGAAACGATCAAATTTATAATGT  120
            ||||||||||||||||||||||||||||||||||||||||||||||||||||||||||||
Sbjct  78   AATCCGCATAGAATTAGGTCAACCAGGAACTTTAATTGGAAACGATCAAATTTATAATGT  137

Query  121  AATTGTAACAGCACATGCATTTATTATAAtttttttATAGTTATACCAATTATAATCGG  180
            ||||||||||||||||||||||||||||||||||||||||||||||||||||||||||||
Sbjct  138  AATTGTAACAGCACATGCATTTATTATAATTTTTTTATAGTTATACCAATTATAATCGG  197

Query  181  AGGATTTGGAAATTGATTAGTTCCTATTATACTAGGAGCTCCAGATATAGCATTTCCACG  240
            ||||||||||||||||||||||||||||||||||||||||||||||||||||||||||||
Sbjct  198  AGGATTTGGAAATTGATTAGTTCCTATTATACTAGGAGCTCCAGATATAGCATTTCCACG  257

Query  241  AATAAATAATATAAGATTTTGGTTACTTCCTCCTTCTCTATTTCTACTAATTAATTCTTC  300
            ||||||||||||||||||||||||||||||||||||||||||||||||||||||||||||
Sbjct  258  AATAAATAATATAAGATTTTGGTTACTTCCTCCTTCTCTATTTCTACTAATTAATTCTTC  317

Query  301  ATTAATCGAATCAGGAGCAGGTACAGGGTGAACAGTTTAcccccccTTATCATCAAATTT  360
            ||||||||||||||||||||||||||||||||||||||||||||||||||||||||||||
Sbjct  318  ATTAATCGAATCAGGAGCAGGTACAGGGTGAACAGTTTACCCCCCCTTATCATCAAATTT  377

Query  361  ATCACACTACGGACCATCAGTAGATTTAGCTATTTTTTCTCTGCATCTTGCAGGTGCTTC  420
            ||||||||||||||||||||||||||||||||||||||||||||||||||||||||||||
Sbjct  378  ATCACACTACGGACCATCAGTAGATTTAGCTATTTTTTCTCTGCATCTTGCAGGTGCTTC  437

Query  421  TTCAATTTTAGGTGCAATCAATTTCATTACAACTATTGTAAATATACGATCCATTGGAAT  480
            ||||||||||||||||||||||||||||||||||||||||||||||||||||||||||||
Sbjct  438  TTCAATTTTAGGTGCAATCAATTTCATTACAACTATTGTAAATATACGATCCATTGGAAT  497

Query  481  AACAATAGAACGAATACCATTATTTGTATGATCTGTTTTAATTACTGCAATTTTATTATT  540
            ||||||||||||||||||||||||||||||||||||||||||||||||||||||||||||
Sbjct  498  AACAATAGAACGAATACCATTATTTGTATGATCTGTTTTAATTACTGCAATTTTATTATT  557

Query  541  ATTATCTTTACCAGTCTTAGCTGGCGCAATTACAATATTATTAACTGATCGAAACTTTAA  600
            ||||||||||||||||||||||||||||||||||||||||||||||||||||||||||||
Sbjct  558  ATTATCTTTACCAGTCTTAGCTGGCGCAATTACAATATTATTAACTGATCGAAACTTTAA  617

Query  601  TACATCATTCTTTGACCCTTCAGGAGGAGGTGACCCAATTCTATATCAACATTTATTT  658
            ||||||||||||||||||||||||||||||||||||||||||||||||||||||||||
Sbjct  618  TACATCATTCTTTGACCCTTCAGGAGGAGGTGACCCAATTCTATATCAACATTTATTT  675
```

>Rhipicephalus sanguineus haplotype RsanColSanM cytochrome c oxidase subunit I (COX1) gene, partial cds; mitochondrial
Sequence ID: KT906182.1 Length: 690
Range 1: 18 to 675

Score:1216 bits(658), Expect:0.0,
Identities:658/658(100%), Gaps:0/658(0%), Strand: Plus/Plus

```
Query  1    GACAATATACTTAATTTTTGGAGCATGATCCGGAATACTAGGATTAAGAATAAGAATATT  60
            ||||||||||||||||||||||||||||||||||||||||||||||||||||||||||||
Sbjct  18   GACAATATACTTAATTTTTGGAGCATGATCCGGAATACTAGGATTAAGAATAAGAATATT  77

Query  61   AATCCGCATAGAATTAGGTCAACCAGGAACTTTAATTGGAAACGATCAAATTTATAATGT  120
            ||||||||||||||||||||||||||||||||||||||||||||||||||||||||||||
Sbjct  78   AATCCGCATAGAATTAGGTCAACCAGGAACTTTAATTGGAAACGATCAAATTTATAATGT  137

Query  121  AATTGTAACAGCACATGCATTTATTATAAttttttttATAGTTATACCAATTATAATCGG  180
            ||||||||||||||||||||||||||||||||||||||||||||||||||||||||||||
Sbjct  138  AATTGTAACAGCACATGCATTTATTATAATTTTTTTTATAGTTATACCAATTATAATCGG  197

Query  181  AGGATTTGGAAATTGATTAGTTCCTATTATACTAGGAGCTCCAGATATAGCATTTCCACG  240
            ||||||||||||||||||||||||||||||||||||||||||||||||||||||||||||
Sbjct  198  AGGATTTGGAAATTGATTAGTTCCTATTATACTAGGAGCTCCAGATATAGCATTTCCACG  257

Query  241  AATAAATAATATAAGATTTTGGTTACTTCCTCCTTCTCTATTTCTACTAATTAATTCTTC  300
            ||||||||||||||||||||||||||||||||||||||||||||||||||||||||||||
Sbjct  258  AATAAATAATATAAGATTTTGGTTACTTCCTCCTTCTCTATTTCTACTAATTAATTCTTC  317

Query  301  ATTAATCGAATCAGGAGCAGGTACAGGGTGAACAGTTTAcccccccTTATCATCAAATTT  360
            ||||||||||||||||||||||||||||||||||||||||||||||||||||||||||||
Sbjct  318  ATTAATCGAATCAGGAGCAGGTACAGGGTGAACAGTTTACCCCCCCTTATCATCAAATTT  377

Query  361  ATCACACTACGGACCATCAGTAGATTTAGCTATTTTTTCTCTGCATCTTGCAGGTGCTTC  420
            ||||||||||||||||||||||||||||||||||||||||||||||||||||||||||||
Sbjct  378  ATCACACTACGGACCATCAGTAGATTTAGCTATTTTTTCTCTGCATCTTGCAGGTGCTTC  437

Query  421  TTCAATTTTAGGTGCAATCAATTTCATTACAACTATTGTAAATATACGATCCATTGGAAT  480
            ||||||||||||||||||||||||||||||||||||||||||||||||||||||||||||
Sbjct  438  TTCAATTTTAGGTGCAATCAATTTCATTACAACTATTGTAAATATACGATCCATTGGAAT  497

Query  481  AACAATAGAACGAATACCATTATTTGTATGATCTGTTTTAATTACTGCAATTTTATTATT  540
            ||||||||||||||||||||||||||||||||||||||||||||||||||||||||||||
Sbjct  498  AACAATAGAACGAATACCATTATTTGTATGATCTGTTTTAATTACTGCAATTTTATTATT  557

Query  541  ATTATCTTTACCAGTCTTAGCTGGCGCAATTACAATATTATTAACTGATCGAAACTTTAA  600
            ||||||||||||||||||||||||||||||||||||||||||||||||||||||||||||
Sbjct  558  ATTATCTTTACCAGTCTTAGCTGGCGCAATTACAATATTATTAACTGATCGAAACTTTAA  617

Query  601  TACATCATTCTTTGACCCTTCAGGAGGAGGTGACCCAATTCTATATCAACATTTATTT  658
            ||||||||||||||||||||||||||||||||||||||||||||||||||||||||||
Sbjct  618  TACATCATTCTTTGACCCTTCAGGAGGAGGTGACCCAATTCTATATCAACATTTATTT  675
```

```
>Rhipicephalus sanguineus isolate Q3 mitochondrion, complete genome
Sequence ID: OM368327.1 Length: 14711
Range 1: 1185 to 1842

Score:1216 bits(658), Expect:0.0,
Identities:658/658(100%),  Gaps:0/658(0%), Strand: Plus/Plus

Query  1     GACAATATACTTAATTTTTGGAGCATGATCCGGAATACTAGGATTAAGAATAAGAATATT  60
             ||||||||||||||||||||||||||||||||||||||||||||||||||||||||||||
Sbjct  1185  GACAATATACTTAATTTTTGGAGCATGATCCGGAATACTAGGATTAAGAATAAGAATATT  1244

Query  61    AATCCGCATAGAATTAGGTCAACCAGGAACTTTAATTGGAAACGATCAAATTTATAATGT  120
             ||||||||||||||||||||||||||||||||||||||||||||||||||||||||||||
Sbjct  1245  AATCCGCATAGAATTAGGTCAACCAGGAACTTTAATTGGAAACGATCAAATTTATAATGT  1304

Query  121   AATTGTAACAGCACATGCATTTATTATAAttttttttATAGTTATACCAATTATAATCGG  180
             ||||||||||||||||||||||||||||||||||||||||||||||||||||||||||||
Sbjct  1305  AATTGTAACAGCACATGCATTTATTATAATTTTTTTTATAGTTATACCAATTATAATCGG  1364

Query  181   AGGATTTGGAAATTGATTAGTTCCTATTATACTAGGAGCTCCAGATATAGCATTTCCACG  240
             ||||||||||||||||||||||||||||||||||||||||||||||||||||||||||||
Sbjct  1365  AGGATTTGGAAATTGATTAGTTCCTATTATACTAGGAGCTCCAGATATAGCATTTCCACG  1424

Query  241   AATAAATAATATAAGATTTTGGTTACTTCCTCCTTCTCTATTTCTACTAATTAATTCTTC  300
             ||||||||||||||||||||||||||||||||||||||||||||||||||||||||||||
Sbjct  1425  AATAAATAATATAAGATTTTGGTTACTTCCTCCTTCTCTATTTCTACTAATTAATTCTTC  1484

Query  301   ATTAATCGAATCAGGAGCAGGTACAGGGTGAACAGTTTAcccccccTTATCATCAAATTT  360
             ||||||||||||||||||||||||||||||||||||||||||||||||||||||||||||
Sbjct  1485  ATTAATCGAATCAGGAGCAGGTACAGGGTGAACAGTTTACCCCCCCTTATCATCAAATTT  1544

Query  361   ATCACACTACGGACCATCAGTAGATTTAGCTATTTTTTCTCTGCATCTTGCAGGTGCTTC  420
             ||||||||||||||||||||||||||||||||||||||||||||||||||||||||||||
Sbjct  1545  ATCACACTACGGACCATCAGTAGATTTAGCTATTTTTTCTCTGCATCTTGCAGGTGCTTC  1604

Query  421   TTCAATTTTAGGTGCAATCAATTTCATTACAACTATTGTAAATATACGATCCATTGGAAT  480
             ||||||||||||||||||||||||||||||||||||||||||||||||||||||||||||
Sbjct  1605  TTCAATTTTAGGTGCAATCAATTTCATTACAACTATTGTAAATATACGATCCATTGGAAT  1664

Query  481   AACAATAGAACGAATACCATTATTTGTATGATCTGTTTTAATTACTGCAATTTTATTATT  540
             ||||||||||||||||||||||||||||||||||||||||||||||||||||||||||||
Sbjct  1665  AACAATAGAACGAATACCATTATTTGTATGATCTGTTTTAATTACTGCAATTTTATTATT  1724

Query  541   ATTATCTTTACCAGTCTTAGCTGGCGCAATTACAATATTATTAACTGATCGAAACTTTAA  600
             ||||||||||||||||||||||||||||||||||||||||||||||||||||||||||||
Sbjct  1725  ATTATCTTTACCAGTCTTAGCTGGCGCAATTACAATATTATTAACTGATCGAAACTTTAA  1784

Query  601   TACATCATTCTTTGACCCTTCAGGAGGAGGTGACCCAATTCTATATCAACATTTATTT  658
             ||||||||||||||||||||||||||||||||||||||||||||||||||||||||||
Sbjct  1785  TACATCATTCTTTGACCCTTCAGGAGGAGGTGACCCAATTCTATATCAACATTTATTT  1842
```

```
>Rhipicephalus sanguineus isolate C19 mitochondrion, complete genome
Sequence ID: OM368323.1 Length: 14714
Range 1: 1185 to 1842

Score:1216 bits(658), Expect:0.0,
Identities:658/658(100%),  Gaps:0/658(0%), Strand: Plus/Plus

Query  1     GACAATATACTTAATTTTTGGAGCATGATCCGGAATACTAGGATTAAGAATAAGAATATT  60
             ||||||||||||||||||||||||||||||||||||||||||||||||||||||||||||
Sbjct  1185  GACAATATACTTAATTTTTGGAGCATGATCCGGAATACTAGGATTAAGAATAAGAATATT  1244

Query  61    AATCCGCATAGAATTAGGTCAACCAGGAACTTTAATTGGAAACGATCAAATTTATAATGT  120
             ||||||||||||||||||||||||||||||||||||||||||||||||||||||||||||
Sbjct  1245  AATCCGCATAGAATTAGGTCAACCAGGAACTTTAATTGGAAACGATCAAATTTATAATGT  1304

Query  121   AATTGTAACAGCACATGCATTTATTATAAttttttttATAGTTATACCAATTATAATCGG  180
             ||||||||||||||||||||||||||||||||||||||||||||||||||||||||||||
Sbjct  1305  AATTGTAACAGCACATGCATTTATTATAATTTTTTTTATAGTTATACCAATTATAATCGG  1364

Query  181   AGGATTTGGAAATTGATTAGTTCCTATTATACTAGGAGCTCCAGATATAGCATTTCCACG  240
             ||||||||||||||||||||||||||||||||||||||||||||||||||||||||||||
Sbjct  1365  AGGATTTGGAAATTGATTAGTTCCTATTATACTAGGAGCTCCAGATATAGCATTTCCACG  1424

Query  241   AATAAATAATATAAGATTTTGGTTACTTCCTCCTTCTCTATTTCTACTAATTAATTCTTC  300
             ||||||||||||||||||||||||||||||||||||||||||||||||||||||||||||
Sbjct  1425  AATAAATAATATAAGATTTTGGTTACTTCCTCCTTCTCTATTTCTACTAATTAATTCTTC  1484

Query  301   ATTAATCGAATCAGGAGCAGGTACAGGGTGAACAGTTTAcccccccTTATCATCAAATTT  360
             ||||||||||||||||||||||||||||||||||||||||||||||||||||||||||||
Sbjct  1485  ATTAATCGAATCAGGAGCAGGTACAGGGTGAACAGTTTACCCCCCCTTATCATCAAATTT  1544

Query  361   ATCACACTACGGACCATCAGTAGATTTAGCTATTTTTTCTCTGCATCTTGCAGGTGCTTC  420
             ||||||||||||||||||||||||||||||||||||||||||||||||||||||||||||
Sbjct  1545  ATCACACTACGGACCATCAGTAGATTTAGCTATTTTTTCTCTGCATCTTGCAGGTGCTTC  1604

Query  421   TTCAATTTTAGGTGCAATCAATTTCATTACAACTATTGTAAATATACGATCCATTGGAAT  480
             ||||||||||||||||||||||||||||||||||||||||||||||||||||||||||||
Sbjct  1605  TTCAATTTTAGGTGCAATCAATTTCATTACAACTATTGTAAATATACGATCCATTGGAAT  1664

Query  481   AACAATAGAACGAATACCATTATTTGTATGATCTGTTTTAATTACTGCAATTTTATTATT  540
             ||||||||||||||||||||||||||||||||||||||||||||||||||||||||||||
Sbjct  1665  AACAATAGAACGAATACCATTATTTGTATGATCTGTTTTAATTACTGCAATTTTATTATT  1724

Query  541   ATTATCTTTACCAGTCTTAGCTGGCGCAATTACAATATTATTAACTGATCGAAACTTTAA  600
             ||||||||||||||||||||||||||||||||||||||||||||||||||||||||||||
Sbjct  1725  ATTATCTTTACCAGTCTTAGCTGGCGCAATTACAATATTATTAACTGATCGAAACTTTAA  1784

Query  601   TACATCATTCTTTGACCCTTCAGGAGGAGGTGACCCAATTCTATATCAACATTTATTT  658
             ||||||||||||||||||||||||||||||||||||||||||||||||||||||||||
Sbjct  1785  TACATCATTCTTTGACCCTTCAGGAGGAGGTGACCCAATTCTATATCAACATTTATTT  1842
```

>Rhipicephalus sanguineus isolate A44 mitochondrion, complete genome
Sequence ID: OM368322.1 Length: 14713
Range 1: 1185 to 1842

Score:1216 bits(658), Expect:0.0,
Identities:658/658(100%), Gaps:0/658(0%), Strand: Plus/Plus

```
Query  1     GACAATATACTTAATTTTTGGAGCATGATCCGGAATACTAGGATTAAGAATAAGAATATT  60
             ||||||||||||||||||||||||||||||||||||||||||||||||||||||||||||
Sbjct  1185  GACAATATACTTAATTTTTGGAGCATGATCCGGAATACTAGGATTAAGAATAAGAATATT  1244

Query  61    AATCCGCATAGAATTAGGTCAACCAGGAACTTTAATTGGAAACGATCAAATTTATAATGT  120
             ||||||||||||||||||||||||||||||||||||||||||||||||||||||||||||
Sbjct  1245  AATCCGCATAGAATTAGGTCAACCAGGAACTTTAATTGGAAACGATCAAATTTATAATGT  1304

Query  121   AATTGTAACAGCACATGCATTTATTATAAttttttttATAGTTATACCAATTATAATCGG  180
             ||||||||||||||||||||||||||||||||||||||||||||||||||||||||||||
Sbjct  1305  AATTGTAACAGCACATGCATTTATTATAATTTTTTTTATAGTTATACCAATTATAATCGG  1364

Query  181   AGGATTTGGAAATTGATTAGTTCCTATTATACTAGGAGCTCCAGATATAGCATTTCCACG  240
             ||||||||||||||||||||||||||||||||||||||||||||||||||||||||||||
Sbjct  1365  AGGATTTGGAAATTGATTAGTTCCTATTATACTAGGAGCTCCAGATATAGCATTTCCACG  1424

Query  241   AATAAATAATATAAGATTTTGGTTACTTCCTCCTTCTCTATTTCTACTAATTAATTCTTC  300
             ||||||||||||||||||||||||||||||||||||||||||||||||||||||||||||
Sbjct  1425  AATAAATAATATAAGATTTTGGTTACTTCCTCCTTCTCTATTTCTACTAATTAATTCTTC  1484

Query  301   ATTAATCGAATCAGGAGCAGGTACAGGGTGAACAGTTTAcccccccTTATCATCAAATTT  360
             ||||||||||||||||||||||||||||||||||||||||||||||||||||||||||||
Sbjct  1485  ATTAATCGAATCAGGAGCAGGTACAGGGTGAACAGTTTACCCCCCCTTATCATCAAATTT  1544

Query  361   ATCACACTACGGACCATCAGTAGATTTAGCTATTTTTTCTCTGCATCTTGCAGGTGCTTC  420
             ||||||||||||||||||||||||||||||||||||||||||||||||||||||||||||
Sbjct  1545  ATCACACTACGGACCATCAGTAGATTTAGCTATTTTTTCTCTGCATCTTGCAGGTGCTTC  1604

Query  421   TTCAATTTTAGGTGCAATCAATTTCATTACAACTATTGTAAATATACGATCCATTGGAAT  480
             ||||||||||||||||||||||||||||||||||||||||||||||||||||||||||||
Sbjct  1605  TTCAATTTTAGGTGCAATCAATTTCATTACAACTATTGTAAATATACGATCCATTGGAAT  1664

Query  481   AACAATAGAACGAATACCATTATTTGTATGATCTGTTTTAATTACTGCAATTTTATTATT  540
             ||||||||||||||||||||||||||||||||||||||||||||||||||||||||||||
Sbjct  1665  AACAATAGAACGAATACCATTATTTGTATGATCTGTTTTAATTACTGCAATTTTATTATT  1724

Query  541   ATTATCTTTACCAGTCTTAGCTGGCGCAATTACAATATTATTAACTGATCGAAACTTTAA  600
             ||||||||||||||||||||||||||||||||||||||||||||||||||||||||||||
Sbjct  1725  ATTATCTTTACCAGTCTTAGCTGGCGCAATTACAATATTATTAACTGATCGAAACTTTAA  1784

Query  601   TACATCATTCTTTGACCCTTCAGGAGGAGGTGACCCAATTCTATATCAACATTTATTT  658
             ||||||||||||||||||||||||||||||||||||||||||||||||||||||||||
Sbjct  1785  TACATCATTCTTTGACCCTTCAGGAGGAGGTGACCCAATTCTATATCAACATTTATTT  1842
```

```
>Rhipicephalus sanguineus isolate Guangxi cytochrome oxidase subunit I (COI)
gene, partial cds; mitochondrial
Sequence ID: JQ737084.1 Length: 707
Range 1: 24 to 681

Score:1216 bits(658), Expect:0.0,
Identities:658/658(100%),  Gaps:0/658(0%), Strand: Plus/Plus

Query  1    GACAATATACTTAATTTTTGGAGCATGATCCGGAATACTAGGATTAAGAATAAGAATATT  60
            ||||||||||||||||||||||||||||||||||||||||||||||||||||||||||||
Sbjct  24   GACAATATACTTAATTTTTGGAGCATGATCCGGAATACTAGGATTAAGAATAAGAATATT  83

Query  61   AATCCGCATAGAATTAGGTCAACCAGGAACTTTAATTGGAAACGATCAAATTTATAATGT  120
            ||||||||||||||||||||||||||||||||||||||||||||||||||||||||||||
Sbjct  84   AATCCGCATAGAATTAGGTCAACCAGGAACTTTAATTGGAAACGATCAAATTTATAATGT  143

Query  121  AATTGTAACAGCACATGCATTTATTATAAttttttttATAGTTATACCAATTATAATCGG  180
            ||||||||||||||||||||||||||||||||||||||||||||||||||||||||||||
Sbjct  144  AATTGTAACAGCACATGCATTTATTATAATTTTTTTTATAGTTATACCAATTATAATCGG  203

Query  181  AGGATTTGGAAATTGATTAGTTCCTATTATACTAGGAGCTCCAGATATAGCATTTCCACG  240
            ||||||||||||||||||||||||||||||||||||||||||||||||||||||||||||
Sbjct  204  AGGATTTGGAAATTGATTAGTTCCTATTATACTAGGAGCTCCAGATATAGCATTTCCACG  263

Query  241  AATAAATAATATAAGATTTTGGTTACTTCCTCCTTCTCTATTTCTACTAATTAATTCTTC  300
            ||||||||||||||||||||||||||||||||||||||||||||||||||||||||||||
Sbjct  264  AATAAATAATATAAGATTTTGGTTACTTCCTCCTTCTCTATTTCTACTAATTAATTCTTC  323

Query  301  ATTAATCGAATCAGGAGCAGGTACAGGGTGAACAGTTTAcccccccTTATCATCAAATTT  360
            ||||||||||||||||||||||||||||||||||||||||||||||||||||||||||||
Sbjct  324  ATTAATCGAATCAGGAGCAGGTACAGGGTGAACAGTTTACCCCCCCTTATCATCAAATTT  383

Query  361  ATCACACTACGGACCATCAGTAGATTTAGCTATTTTTTCTCTGCATCTTGCAGGTGCTTC  420
            ||||||||||||||||||||||||||||||||||||||||||||||||||||||||||||
Sbjct  384  ATCACACTACGGACCATCAGTAGATTTAGCTATTTTTTCTCTGCATCTTGCAGGTGCTTC  443

Query  421  TTCAATTTTAGGTGCAATCAATTTCATTACAACTATTGTAAATATACGATCCATTGGAAT  480
            ||||||||||||||||||||||||||||||||||||||||||||||||||||||||||||
Sbjct  444  TTCAATTTTAGGTGCAATCAATTTCATTACAACTATTGTAAATATACGATCCATTGGAAT  503

Query  481  AACAATAGAACGAATACCATTATTTGTATGATCTGTTTTAATTACTGCAATTTTATTATT  540
            ||||||||||||||||||||||||||||||||||||||||||||||||||||||||||||
Sbjct  504  AACAATAGAACGAATACCATTATTTGTATGATCTGTTTTAATTACTGCAATTTTATTATT  563

Query  541  ATTATCTTTACCAGTCTTAGCTGGCGCAATTACAATATTATTAACTGATCGAAACTTTAA  600
            ||||||||||||||||||||||||||||||||||||||||||||||||||||||||||||
Sbjct  564  ATTATCTTTACCAGTCTTAGCTGGCGCAATTACAATATTATTAACTGATCGAAACTTTAA  623

Query  601  TACATCATTCTTTGACCCTTCAGGAGGAGGTGACCCAATTCTATATCAACATTTATTT  658
            ||||||||||||||||||||||||||||||||||||||||||||||||||||||||||
```

```
Sbjct  624  TACATCATTCTTTGACCCTTCAGGAGGAGGTGACCCAATTCTATATCAACATTTATTT  681
```

>Rhipicephalus sanguineus cytochrome oxidase subunit 1 (COI) gene, partial cds; mitochondrial
Sequence ID: KF437543.1 Length: 658
>Ixodidae sp. sc_00964 cytochrome oxidase subunit 1 (COI) gene, partial cds; mitochondrial
Sequence ID: KX053539.1 Length: 658
Range 1: 1 to 658

Score:1216 bits(658), Expect:0.0,
Identities:658/658(100%), Gaps:0/658(0%), Strand: Plus/Plus

```
Query  1    GACAATATACTTAATTTTTGGAGCATGATCCGGAATACTAGGATTAAGAATAAGAATATT  60
            ||||||||||||||||||||||||||||||||||||||||||||||||||||||||||||
Sbjct  1    GACAATATACTTAATTTTTGGAGCATGATCCGGAATACTAGGATTAAGAATAAGAATATT  60

Query  61   AATCCGCATAGAATTAGGTCAACCAGGAACTTTAATTGGAAACGATCAAATTTATAATGT  120
            ||||||||||||||||||||||||||||||||||||||||||||||||||||||||||||
Sbjct  61   AATCCGCATAGAATTAGGTCAACCAGGAACTTTAATTGGAAACGATCAAATTTATAATGT  120

Query  121  AATTGTAACAGCACATGCATTTATTATAAttttttttATAGTTATACCAATTATAATCGG  180
            ||||||||||||||||||||||||||||||||||||||||||||||||||||||||||||
Sbjct  121  AATTGTAACAGCACATGCATTTATTATAATTTTTTTTATAGTTATACCAATTATAATCGG  180

Query  181  AGGATTTGGAAATTGATTAGTTCCTATTATACTAGGAGCTCCAGATATAGCATTTCCACG  240
            ||||||||||||||||||||||||||||||||||||||||||||||||||||||||||||
Sbjct  181  AGGATTTGGAAATTGATTAGTTCCTATTATACTAGGAGCTCCAGATATAGCATTTCCACG  240

Query  241  AATAAATAATATAAGATTTTGGTTACTTCCTCCTTCTCTATTTCTACTAATTAATTCTTC  300
            ||||||||||||||||||||||||||||||||||||||||||||||||||||||||||||
Sbjct  241  AATAAATAATATAAGATTTTGGTTACTTCCTCCTTCTCTATTTCTACTAATTAATTCTTC  300

Query  301  ATTAATCGAATCAGGAGCAGGTACAGGGTGAACAGTTTAcccccccTTATCATCAAATTT  360
            ||||||||||||||||||||||||||||||||||||||||||||||||||||||||||||
Sbjct  301  ATTAATCGAATCAGGAGCAGGTACAGGGTGAACAGTTTACCCCCCCTTATCATCAAATTT  360

Query  361  ATCACACTACGGACCATCAGTAGATTTAGCTATTTTTTCTCTGCATCTTGCAGGTGCTTC  420
            ||||||||||||||||||||||||||||||||||||||||||||||||||||||||||||
Sbjct  361  ATCACACTACGGACCATCAGTAGATTTAGCTATTTTTTCTCTGCATCTTGCAGGTGCTTC  420

Query  421  TTCAATTTTAGGTGCAATCAATTTCATTACAACTATTGTAAATATACGATCCATTGGAAT  480
            ||||||||||||||||||||||||||||||||||||||||||||||||||||||||||||
Sbjct  421  TTCAATTTTAGGTGCAATCAATTTCATTACAACTATTGTAAATATACGATCCATTGGAAT  480

Query  481  AACAATAGAACGAATACCATTATTTGTATGATCTGTTTTAATTACTGCAATTTTATTATT  540
            ||||||||||||||||||||||||||||||||||||||||||||||||||||||||||||
Sbjct  481  AACAATAGAACGAATACCATTATTTGTATGATCTGTTTTAATTACTGCAATTTTATTATT  540

Query  541  ATTATCTTTACCAGTCTTAGCTGGCGCAATTACAATATTATTAACTGATCGAAACTTTAA  600
            ||||||||||||||||||||||||||||||||||||||||||||||||||||||||||||
```

```
Sbjct  541   ATTATCTTTACCAGTCTTAGCTGGCGCAATTACAATATTATTAACTGATCGAAACTTTAA  600

Query  601   TACATCATTCTTTGACCCTTCAGGAGGAGGTGACCCAATTCTATATCAACATTTATTT  658
             ||||||||||||||||||||||||||||||||||||||||||||||||||||||||||
Sbjct  601   TACATCATTCTTTGACCCTTCAGGAGGAGGTGACCCAATTCTATATCAACATTTATTT  658
```

>Rhipicephalus linnaei isolate LAO32-2 clone JS5930 mitochondrion, complete genome
Sequence ID: MW429383.1 Length: 14715
Range 1: 45 to 702

Score:1216 bits(658), Expect:0.0,
Identities:658/658(100%), Gaps:0/658(0%), Strand: Plus/Plus

```
Query  1     GACAATATACTTAATTTTTGGAGCATGATCCGGAATACTAGGATTAAGAATAAGAATATT  60
             ||||||||||||||||||||||||||||||||||||||||||||||||||||||||||||
Sbjct  45    GACAATATACTTAATTTTTGGAGCATGATCCGGAATACTAGGATTAAGAATAAGAATATT  104

Query  61    AATCCGCATAGAATTAGGTCAACCAGGAACTTTAATTGGAAACGATCAAATTTATAATGT  120
             ||||||||||||||||||||||||||||||||||||||||||||||||||||||||||||
Sbjct  105   AATCCGCATAGAATTAGGTCAACCAGGAACTTTAATTGGAAACGATCAAATTTATAATGT  164

Query  121   AATTGTAACAGCACATGCATTTATTATAAttttttttATAGTTATACCAATTATAATCGG  180
             ||||||||||||||||||||||||||||||||||||||||||||||||||||||||||||
Sbjct  165   AATTGTAACAGCACATGCATTTATTATAATTTTTTTTATAGTTATACCAATTATAATCGG  224

Query  181   AGGATTTGGAAATTGATTAGTTCCTATTATACTAGGAGCTCCAGATATAGCATTTCCACG  240
             ||||||||||||||||||||||||||||||||||||||||||||||||||||||||||||
Sbjct  225   AGGATTTGGAAATTGATTAGTTCCTATTATACTAGGAGCTCCAGATATAGCATTTCCACG  284

Query  241   AATAAATAATATAAGATTTTGGTTACTTCCTCCTTCTCTATTTCTACTAATTAATTCTTC  300
             ||||||||||||||||||||||||||||||||||||||||||||||||||||||||||||
Sbjct  285   AATAAATAATATAAGATTTTGGTTACTTCCTCCTTCTCTATTTCTACTAATTAATTCTTC  344

Query  301   ATTAATCGAATCAGGAGCAGGTACAGGGTGAACAGTTTAcccccccTTATCATCAAATTT  360
             ||||||||||||||||||||||||||||||||||||||||||||||||||||||||||||
Sbjct  345   ATTAATCGAATCAGGAGCAGGTACAGGGTGAACAGTTTACCCCCCCTTATCATCAAATTT  404

Query  361   ATCACACTACGGACCATCAGTAGATTTAGCTATTTTTTCTCTGCATCTTGCAGGTGCTTC  420
             ||||||||||||||||||||||||||||||||||||||||||||||||||||||||||||
Sbjct  405   ATCACACTACGGACCATCAGTAGATTTAGCTATTTTTTCTCTGCATCTTGCAGGTGCTTC  464

Query  421   TTCAATTTTAGGTGCAATCAATTTCATTACAACTATTGTAAATATACGATCCATTGGAAT  480
             ||||||||||||||||||||||||||||||||||||||||||||||||||||||||||||
Sbjct  465   TTCAATTTTAGGTGCAATCAATTTCATTACAACTATTGTAAATATACGATCCATTGGAAT  524

Query  481   AACAATAGAACGAATACCATTATTTGTATGATCTGTTTTAATTACTGCAATTTTATTATT  540
             ||||||||||||||||||||||||||||||||||||||||||||||||||||||||||||
Sbjct  525   AACAATAGAACGAATACCATTATTTGTATGATCTGTTTTAATTACTGCAATTTTATTATT  584

Query  541   ATTATCTTTACCAGTCTTAGCTGGCGCAATTACAATATTATTAACTGATCGAAACTTTAA  600
```

```
          ||||||||||||||||||||||||||||||||||||||||||||||||||||||||||||
Sbjct  585  ATTATCTTTACCAGTCTTAGCTGGCGCAATTACAATATTATTAACTGATCGAAACTTTAA  644

Query  601  TACATCATTCTTTGACCCTTCAGGAGGAGGTGACCCAATTCTATATCAACATTTATTT  658
            ||||||||||||||||||||||||||||||||||||||||||||||||||||||||||
Sbjct  645  TACATCATTCTTTGACCCTTCAGGAGGAGGTGACCCAATTCTATATCAACATTTATTT  702
```

>Rhipicephalus linnaei mitochondrion, complete sequence
Sequence ID: NC_060409.1 Length: 14711
>Rhipicephalus linnaei strain Coominya isolate AUS3 clone JS5929 mitochondrion, complete genome
Sequence ID: MW429381.1 Length: 14711
Range 1: 45 to 702

Score:1216 bits(658), Expect:0.0,
Identities:658/658(100%), Gaps:0/658(0%), Strand: Plus/Plus

```
Query  1    GACAATATACTTAATTTTTGGAGCATGATCCGGAATACTAGGATTAAGAATAAGAATATT  60
            ||||||||||||||||||||||||||||||||||||||||||||||||||||||||||||
Sbjct  45   GACAATATACTTAATTTTTGGAGCATGATCCGGAATACTAGGATTAAGAATAAGAATATT  104

Query  61   AATCCGCATAGAATTAGGTCAACCAGGAACTTTAATTGGAAACGATCAAATTTATAATGT  120
            ||||||||||||||||||||||||||||||||||||||||||||||||||||||||||||
Sbjct  105  AATCCGCATAGAATTAGGTCAACCAGGAACTTTAATTGGAAACGATCAAATTTATAATGT  164

Query  121  AATTGTAACAGCACATGCATTTATTATAAttttttttATAGTTATACCAATTATAATCGG  180
            ||||||||||||||||||||||||||||||||||||||||||||||||||||||||||||
Sbjct  165  AATTGTAACAGCACATGCATTTATTATAATTTTTTTTATAGTTATACCAATTATAATCGG  224

Query  181  AGGATTTGGAAATTGATTAGTTCCTATTATACTAGGAGCTCCAGATATAGCATTTCCACG  240
            ||||||||||||||||||||||||||||||||||||||||||||||||||||||||||||
Sbjct  225  AGGATTTGGAAATTGATTAGTTCCTATTATACTAGGAGCTCCAGATATAGCATTTCCACG  284

Query  241  AATAAATAATATAAGATTTTGGTTACTTCCTCCTTCTCTATTTCTACTAATTAATTCTTC  300
            ||||||||||||||||||||||||||||||||||||||||||||||||||||||||||||
Sbjct  285  AATAAATAATATAAGATTTTGGTTACTTCCTCCTTCTCTATTTCTACTAATTAATTCTTC  344

Query  301  ATTAATCGAATCAGGAGCAGGTACAGGGTGAACAGTTTAcccccccTTATCATCAAATTT  360
            ||||||||||||||||||||||||||||||||||||||||||||||||||||||||||||
Sbjct  345  ATTAATCGAATCAGGAGCAGGTACAGGGTGAACAGTTTACCCCCCCTTATCATCAAATTT  404

Query  361  ATCACACTACGGACCATCAGTAGATTTAGCTATTTTTTCTCTGCATCTTGCAGGTGCTTC  420
            ||||||||||||||||||||||||||||||||||||||||||||||||||||||||||||
Sbjct  405  ATCACACTACGGACCATCAGTAGATTTAGCTATTTTTTCTCTGCATCTTGCAGGTGCTTC  464

Query  421  TTCAATTTTAGGTGCAATCAATTTCATTACAACTATTGTAAATATACGATCCATTGGAAT  480
            ||||||||||||||||||||||||||||||||||||||||||||||||||||||||||||
Sbjct  465  TTCAATTTTAGGTGCAATCAATTTCATTACAACTATTGTAAATATACGATCCATTGGAAT  524

Query  481  AACAATAGAACGAATACCATTATTTGTATGATCTGTTTTAATTACTGCAATTTTATTATT  540
            ||||||||||||||||||||||||||||||||||||||||||||||||||||||||||||
```

```
Sbjct  525   AACAATAGAACGAATACCATTATTTGTATGATCTGTTTTAATTACTGCAATTTTATTATT  584

Query  541   ATTATCTTTACCAGTCTTAGCTGGCGCAATTACAATATTATTAACTGATCGAAACTTTAA  600
             ||||||||||||||||||||||||||||||||||||||||||||||||||||||||||||
Sbjct  585   ATTATCTTTACCAGTCTTAGCTGGCGCAATTACAATATTATTAACTGATCGAAACTTTAA  644

Query  601   TACATCATTCTTTGACCCTTCAGGAGGAGGTGACCCAATTCTATATCAACATTTATTT  658
             ||||||||||||||||||||||||||||||||||||||||||||||||||||||||||
Sbjct  645   TACATCATTCTTTGACCCTTCAGGAGGAGGTGACCCAATTCTATATCAACATTTATTT  702
```

>Ixodidae sp. sc_01082 cytochrome oxidase subunit 1 (COI) gene, partial cds; mitochondrial
Sequence ID: KX053537.1 Length: 658
Range 1: 1 to 658

Score:1210 bits(655), Expect:0.0,
Identities:657/658(99%), Gaps:0/658(0%), Strand: Plus/Plus

```
Query  1     GACAATATACTTAATTTTTGGAGCATGATCCGGAATACTAGGATTAAGAATAAGAATATT  60
             ||||||||||||||||||||||||||||||||||||||||||||||||||||||||||||
Sbjct  1     GACAATATACTTAATTTTTGGAGCATGATCCGGAATACTAGGATTAAGAATAAGAATATT  60

Query  61    AATCCGCATAGAATTAGGTCAACCAGGAACTTTAATTGGAAACGATCAAATTTATAATGT  120
             ||||||||||||||||||||||||||||||||||||||||||||||||||||||||||||
Sbjct  61    AATCCGCATAGAATTAGGTCAACCAGGAACTTTAATTGGAAACGATCAAATTTATAATGT  120

Query  121   AATTGTAACAGCACATGCATTTATTATAAtttttttATAGTTATACCAATTATAATCGG  180
             ||||||||||||||||||||||||||||||||||||||||||||||||||||||||||||
Sbjct  121   AATTGTAACAGCACATGCATTTATTATAATTTTTTTATAGTTATACCAATTATAATCGG  180

Query  181   AGGATTTGGAAATTGATTAGTTCCTATTATACTAGGAGCTCCAGATATAGCATTTCCACG  240
             ||||||||||||||||||||||||||||||||||||||||||||||||||||||||||||
Sbjct  181   AGGATTTGGAAATTGATTAGTTCCTATTATACTAGGAGCTCCAGATATAGCATTTCCACG  240

Query  241   AATAAATAATATAAGATTTTGGTTACTTCCTCCTTCTCTATTTCTACTAATTAATTCTTC  300
             ||||||||||||||||||||||||||||||||||||||||||||||||||||||||||||
Sbjct  241   AATAAATAATATAAGATTTTGGTTACTTCCTCCTTCTCTATTTCTACTAATTAATTCTTC  300

Query  301   ATTAATCGAATCAGGAGCAGGTACAGGGTGAACAGTTTAcccccccTTATCATCAAATTT  360
             ||||||||||||||||||||||||||||||||||||||||||||||||||||||||||||
Sbjct  301   ATTAATCGAATCAGGAGCAGGTACAGGGTGAACAGTTTACCCCCCCTTATCATCAAATTT  360

Query  361   ATCACACTACGGACCATCAGTAGATTTAGCTATTTTTTCTCTGCATCTTGCAGGTGCTTC  420
             |||||||||||||||||||||||| |||||||||||||||||||||||||||||||||||
Sbjct  361   ATCACACTACGGACCATCAGTAGATCTAGCTATTTTTTCTCTGCATCTTGCAGGTGCTTC  420

Query  421   TTCAATTTTAGGTGCAATCAATTTCATTACAACTATTGTAAATATACGATCCATTGGAAT  480
             ||||||||||||||||||||||||||||||||||||||||||||||||||||||||||||
Sbjct  421   TTCAATTTTAGGTGCAATCAATTTCATTACAACTATTGTAAATATACGATCCATTGGAAT  480

Query  481   AACAATAGAACGAATACCATTATTTGTATGATCTGTTTTAATTACTGCAATTTTATTATT  540
```

```
          ||||||||||||||||||||||||||||||||||||||||||||||||||||||||||||
Sbjct  481   AACAATAGAACGAATACCATTATTTGTATGATCTGTTTTAATTACTGCAATTTTATTATT  540

Query  541   ATTATCTTTACCAGTCTTAGCTGGCGCAATTACAATATTATTAACTGATCGAAACTTTAA  600
             ||||||||||||||||||||||||||||||||||||||||||||||||||||||||||||
Sbjct  541   ATTATCTTTACCAGTCTTAGCTGGCGCAATTACAATATTATTAACTGATCGAAACTTTAA  600

Query  601   TACATCATTCTTTGACCCTTCAGGAGGAGGTGACCCAATTCTATATCAACATTTATTT  658
             ||||||||||||||||||||||||||||||||||||||||||||||||||||||||||
Sbjct  601   TACATCATTCTTTGACCCTTCAGGAGGAGGTGACCCAATTCTATATCAACATTTATTT  658
```

>Rhipicephalus sanguineus haplotype RsanColCorL cytochrome c oxidase subunit I (COX1) gene, partial cds; mitochondrial
Sequence ID: KT906185.1 Length: 690
Range 1: 18 to 675

Score:1210 bits(655), Expect:0.0,
Identities:657/658(99%), Gaps:0/658(0%), Strand: Plus/Plus

```
Query  1     GACAATATACTTAATTTTTGGAGCATGATCCGGAATACTAGGATTAAGAATAAGAATATT  60
             ||||||||||||||||||||||||||||||||||||||||||||||||||||||||||||
Sbjct  18    GACAATATACTTAATTTTTGGAGCATGATCCGGAATACTAGGATTAAGAATAAGAATATT  77

Query  61    AATCCGCATAGAATTAGGTCAACCAGGAACTTTAATTGGAAACGATCAAATTTATAATGT  120
             ||||||||||||||||||||||||||||||||||||||||||||||||||||||||||||
Sbjct  78    AATCCGCATAGAATTAGGTCAACCAGGAACTTTAATTGGAAACGATCAAATTTATAATGT  137

Query  121   AATTGTAACAGCACATGCATTTATTATAAttttttttATAGTTATACCAATTATAATCGG  180
             ||||||||||||||||||||||||||||||||||||||||||||||||||||||||||||
Sbjct  138   AATTGTAACAGCACATGCATTTATTATAATTTTTTTTATAGTTATACCAATTATAATCGG  197

Query  181   AGGATTTGGAAATTGATTAGTTCCTATTATACTAGGAGCTCCAGATATAGCATTTCCACG  240
             ||||||||||||||||||||||||||||||||| ||||||||||||||||||||||||||
Sbjct  198   AGGATTTGGAAATTGATTAGTTCCTATTATACTGGGAGCTCCAGATATAGCATTTCCACG  257

Query  241   AATAAATAATATAAGATTTTGGTTACTTCCTCCTTCTCTATTTCTACTAATTAATTCTTC  300
             ||||||||||||||||||||||||||||||||||||||||||||||||||||||||||||
Sbjct  258   AATAAATAATATAAGATTTTGGTTACTTCCTCCTTCTCTATTTCTACTAATTAATTCTTC  317

Query  301   ATTAATCGAATCAGGAGCAGGTACAGGGTGAACAGTTTAcccccccTTATCATCAAATTT  360
             ||||||||||||||||||||||||||||||||||||||||||||||||||||||||||||
Sbjct  318   ATTAATCGAATCAGGAGCAGGTACAGGGTGAACAGTTTACCCCCCCTTATCATCAAATTT  377

Query  361   ATCACACTACGGACCATCAGTAGATTTAGCTATTTTTTCTCTGCATCTTGCAGGTGCTTC  420
             ||||||||||||||||||||||||||||||||||||||||||||||||||||||||||||
Sbjct  378   ATCACACTACGGACCATCAGTAGATTTAGCTATTTTTTCTCTGCATCTTGCAGGTGCTTC  437

Query  421   TTCAATTTTAGGTGCAATCAATTTCATTACAACTATTGTAAATATACGATCCATTGGAAT  480
             ||||||||||||||||||||||||||||||||||||||||||||||||||||||||||||
Sbjct  438   TTCAATTTTAGGTGCAATCAATTTCATTACAACTATTGTAAATATACGATCCATTGGAAT  497
```

```
Query  481   AACAATAGAACGAATACCATTATTTGTATGATCTGTTTTAATTACTGCAATTTTATTATT  540
             ||||||||||||||||||||||||||||||||||||||||||||||||||||||||||||
Sbjct  498   AACAATAGAACGAATACCATTATTTGTATGATCTGTTTTAATTACTGCAATTTTATTATT  557

Query  541   ATTATCTTTACCAGTCTTAGCTGGCGCAATTACAATATTATTAACTGATCGAAACTTTAA  600
             ||||||||||||||||||||||||||||||||||||||||||||||||||||||||||||
Sbjct  558   ATTATCTTTACCAGTCTTAGCTGGCGCAATTACAATATTATTAACTGATCGAAACTTTAA  617

Query  601   TACATCATTCTTTGACCCTTCAGGAGGAGGTGACCCAATTCTATATCAACATTTATTT  658
             ||||||||||||||||||||||||||||||||||||||||||||||||||||||||||
Sbjct  618   TACATCATTCTTTGACCCTTCAGGAGGAGGTGACCCAATTCTATATCAACATTTATTT  675
```

>Rhipicephalus sanguineus haplotype RsanColTolC cytochrome c oxidase subunit I (COX1) gene, partial cds; mitochondrial
Sequence ID: KT906184.1 Length: 690
Range 1: 18 to 675

Score:1210 bits(655), Expect:0.0,
Identities:657/658(99%), Gaps:0/658(0%), Strand: Plus/Plus

```
Query  1     GACAATATACTTAATTTTTGGAGCATGATCCGGAATACTAGGATTAAGAATAAGAATATT  60
             |||||||||||||||||||||||||||||||| |||||||||||||||||||||||||||
Sbjct  18    GACAATATACTTAATTTTTGGAGCATGATCCGGGATACTAGGATTAAGAATAAGAATATT  77

Query  61    AATCCGCATAGAATTAGGTCAACCAGGAACTTTAATTGGAAACGATCAAATTTATAATGT  120
             ||||||||||||||||||||||||||||||||||||||||||||||||||||||||||||
Sbjct  78    AATCCGCATAGAATTAGGTCAACCAGGAACTTTAATTGGAAACGATCAAATTTATAATGT  137

Query  121   AATTGTAACAGCACATGCATTTATTATAAttttttttATAGTTATACCAATTATAATCGG  180
             ||||||||||||||||||||||||||||||||||||||||||||||||||||||||||||
Sbjct  138   AATTGTAACAGCACATGCATTTATTATAATTTTTTTTATAGTTATACCAATTATAATCGG  197

Query  181   AGGATTTGGAAATTGATTAGTTCCTATTATACTAGGAGCTCCAGATATAGCATTTCCACG  240
             ||||||||||||||||||||||||||||||||||||||||||||||||||||||||||||
Sbjct  198   AGGATTTGGAAATTGATTAGTTCCTATTATACTAGGAGCTCCAGATATAGCATTTCCACG  257

Query  241   AATAAATAATATAAGATTTTGGTTACTTCCTCCTTCTCTATTTCTACTAATTAATTCTTC  300
             ||||||||||||||||||||||||||||||||||||||||||||||||||||||||||||
Sbjct  258   AATAAATAATATAAGATTTTGGTTACTTCCTCCTTCTCTATTTCTACTAATTAATTCTTC  317

Query  301   ATTAATCGAATCAGGAGCAGGTACAGGGTGAACAGTTTAcccccccTTATCATCAAATTT  360
             ||||||||||||||||||||||||||||||||||||||||||||||||||||||||||||
Sbjct  318   ATTAATCGAATCAGGAGCAGGTACAGGGTGAACAGTTTACCCCCCCTTATCATCAAATTT  377

Query  361   ATCACACTACGGACCATCAGTAGATTTAGCTATTTTTTCTCTGCATCTTGCAGGTGCTTC  420
             ||||||||||||||||||||||||||||||||||||||||||||||||||||||||||||
Sbjct  378   ATCACACTACGGACCATCAGTAGATTTAGCTATTTTTTCTCTGCATCTTGCAGGTGCTTC  437

Query  421   TTCAATTTTAGGTGCAATCAATTTCATTACAACTATTGTAAATATACGATCCATTGGAAT  480
             ||||||||||||||||||||||||||||||||||||||||||||||||||||||||||||
Sbjct  438   TTCAATTTTAGGTGCAATCAATTTCATTACAACTATTGTAAATATACGATCCATTGGAAT  497
```

```
Query  481   AACAATAGAACGAATACCATTATTTGTATGATCTGTTTTAATTACTGCAATTTTATTATT  540
             ||||||||||||||||||||||||||||||||||||||||||||||||||||||||||||
Sbjct  498   AACAATAGAACGAATACCATTATTTGTATGATCTGTTTTAATTACTGCAATTTTATTATT  557

Query  541   ATTATCTTTACCAGTCTTAGCTGGCGCAATTACAATATTATTAACTGATCGAAACTTTAA  600
             ||||||||||||||||||||||||||||||||||||||||||||||||||||||||||||
Sbjct  558   ATTATCTTTACCAGTCTTAGCTGGCGCAATTACAATATTATTAACTGATCGAAACTTTAA  617

Query  601   TACATCATTCTTTGACCCTTCAGGAGGAGGTGACCCAATTCTATATCAACATTTATTT  658
             ||||||||||||||||||||||||||||||||||||||||||||||||||||||||||
Sbjct  618   TACATCATTCTTTGACCCTTCAGGAGGAGGTGACCCAATTCTATATCAACATTTATTT  675
```

>Rhipicephalus sanguineus voucher CAS-T065 cytochrome oxidase subunit 1 (COI) gene, partial cds; mitochondrial
Sequence ID: KF200113.1 Length: 658
Range 1: 1 to 658

Score:1210 bits(655), Expect:0.0,
Identities:657/658(99%), Gaps:0/658(0%), Strand: Plus/Plus

```
Query  1     GACAATATACTTAATTTTTGGAGCATGATCCGGAATACTAGGATTAAGAATAAGAATATT  60
             ||||||||||||||||||||||||||||||||||||||||||||||||||||||||||||
Sbjct  1     GACAATATACTTAATTTTTGGAGCATGATCCGGAATACTAGGATTAAGAATAAGAATATT  60

Query  61    AATCCGCATAGAATTAGGTCAACCAGGAACTTTAATTGGAAACGATCAAATTTATAATGT  120
             ||||||||||||||||||||||||||||||||||||||||||||||||||||||||||||
Sbjct  61    AATCCGCATAGAATTAGGTCAACCAGGAACTTTAATTGGAAACGATCAAATTTATAATGT  120

Query  121   AATTGTAACAGCACATGCATTTATTATAAttttttttATAGTTATACCAATTATAATCGG  180
             ||||||||||||||||||||||||||||||||||||||||||||||||||||||||||||
Sbjct  121   AATTGTAACAGCACATGCATTTATTATAATTTTTTTTATAGTTATACCAATTATAATCGG  180

Query  181   AGGATTTGGAAATTGATTAGTTCCTATTATACTAGGAGCTCCAGATATAGCATTTCCACG  240
             ||||||||||||||||||||||||||||||||||||||||||||||||||||||||||||
Sbjct  181   AGGATTTGGAAATTGATTAGTTCCTATTATACTAGGAGCTCCAGATATAGCATTTCCACG  240

Query  241   AATAAATAATATAAGATTTTGGTTACTTCCTCCTTCTCTATTTCTACTAATTAATTCTTC  300
             ||||||||||||||||||||||||||||||||||||||||||||||||||||||||||||
Sbjct  241   AATAAATAATATAAGATTTTGGTTACTTCCTCCTTCTCTATTTCTACTAATTAATTCTTC  300

Query  301   ATTAATCGAATCAGGAGCAGGTACAGGGTGAACAGTTTAcccccccTTATCATCAAATTT  360
             ||||||||||||||||||||| ||||||||||||||||||||||||||||||||||||||
Sbjct  301   ATTAATCGAATCAGGAGCAGGAACAGGGTGAACAGTTTACCCCCCCTTATCATCAAATTT  360

Query  361   ATCACACTACGGACCATCAGTAGATTTAGCTATTTTTTCTCTGCATCTTGCAGGTGCTTC  420
             ||||||||||||||||||||||||||||||||||||||||||||||||||||||||||||
Sbjct  361   ATCACACTACGGACCATCAGTAGATTTAGCTATTTTTTCTCTGCATCTTGCAGGTGCTTC  420

Query  421   TTCAATTTTAGGTGCAATCAATTTCATTACAACTATTGTAAATATACGATCCATTGGAAT  480
             ||||||||||||||||||||||||||||||||||||||||||||||||||||||||||||
```

```
Sbjct  421  TTCAATTTTAGGTGCAATCAATTTCATTACAACTATTGTAAATATACGATCCATTGGAAT  480

Query  481  AACAATAGAACGAATACCATTATTTGTATGATCTGTTTTAATTACTGCAATTTTATTATT  540
            ||||||||||||||||||||||||||||||||||||||||||||||||||||||||||||
Sbjct  481  AACAATAGAACGAATACCATTATTTGTATGATCTGTTTTAATTACTGCAATTTTATTATT  540

Query  541  ATTATCTTTACCAGTCTTAGCTGGCGCAATTACAATATTATTAACTGATCGAAACTTTAA  600
            ||||||||||||||||||||||||||||||||||||||||||||||||||||||||||||
Sbjct  541  ATTATCTTTACCAGTCTTAGCTGGCGCAATTACAATATTATTAACTGATCGAAACTTTAA  600

Query  601  TACATCATTCTTTGACCCTTCAGGAGGAGGTGACCCAATTCTATATCAACATTTATTT  658
            ||||||||||||||||||||||||||||||||||||||||||||||||||||||||||
Sbjct  601  TACATCATTCTTTGACCCTTCAGGAGGAGGTGACCCAATTCTATATCAACATTTATTT  658
```

>Rhipicephalus linnaei isolate SC931-1 clone JS5927 mitochondrion, complete genome
Sequence ID: MW429382.1 Length: 14717
Range 1: 45 to 702

Score:1210 bits(655), Expect:0.0,
Identities:657/658(99%), Gaps:0/658(0%), Strand: Plus/Plus

```
Query  1    GACAATATACTTAATTTTTGGAGCATGATCCGGAATACTAGGATTAAGAATAAGAATATT  60
            ||||||||||||||||||||||||||||||||||||||||||||||||||||||||||||
Sbjct  45   GACAATATACTTAATTTTTGGAGCATGATCCGGAATACTAGGATTAAGAATAAGAATATT  104

Query  61   AATCCGCATAGAATTAGGTCAACCAGGAACTTTAATTGGAAACGATCAAATTTATAATGT  120
            ||||||||||||||||||||||||||||||||||||||||||||||||||||||||||||
Sbjct  105  AATCCGCATAGAATTAGGTCAACCAGGAACTTTAATTGGAAACGATCAAATTTATAATGT  164

Query  121  AATTGTAACAGCACATGCATTTATTATAAttttttttATAGTTATACCAATTATAATCGG  180
            ||||||||||||||||||||||||||||||||||||||||||||||||||||||||||||
Sbjct  165  AATTGTAACAGCACATGCATTTATTATAATTTTTTTTATAGTTATACCAATTATAATCGG  224

Query  181  AGGATTTGGAAATTGATTAGTTCCTATTATACTAGGAGCTCCAGATATAGCATTTCCACG  240
            ||||||||||||||||||||||||||||||||||||||||||||||||||||||||||||
Sbjct  225  AGGATTTGGAAATTGATTAGTTCCTATTATACTAGGAGCTCCAGATATAGCATTTCCACG  284

Query  241  AATAAATAATATAAGATTTTGGTTACTTCCTCCTTCTCTATTTCTACTAATTAATTCTTC  300
            ||||||||||||||||||||||||||||||||||||||||||||||||||||||||||||
Sbjct  285  AATAAATAATATAAGATTTTGGTTACTTCCTCCTTCTCTATTTCTACTAATTAATTCTTC  344

Query  301  ATTAATCGAATCAGGAGCAGGTACAGGGTGAACAGTTTAcccccccTTATCATCAAATTT  360
            ||||||||||||||||||||||||||||||||||||||||||||||||||||||||||||
Sbjct  345  ATTAATCGAATCAGGAGCAGGTACAGGGTGAACAGTTTACCCCCCCTTATCATCAAATTT  404

Query  361  ATCACACTACGGACCATCAGTAGATTTAGCTATTTTTCTCTGCATCTTGCAGGTGCTTC  420
            ||||||||||||||||||||||||||||||||||||||||| ||||||||||||||||||
Sbjct  405  ATCACACTACGGACCATCAGTAGATTTAGCTATTTTTCTCTACATCTTGCAGGTGCTTC  464

Query  421  TTCAATTTTAGGTGCAATCAATTTCATTACAACTATTGTAAATATACGATCCATTGGAAT  480
```

```
            ||||||||||||||||||||||||||||||||||||||||||||||||||||||||||||
Sbjct  465  TTCAATTTTAGGTGCAATCAATTTCATTACAACTATTGTAAATATACGATCCATTGGAAT  524

Query  481  AACAATAGAACGAATACCATTATTTGTATGATCTGTTTTAATTACTGCAATTTTATTATT  540
            ||||||||||||||||||||||||||||||||||||||||||||||||||||||||||||
Sbjct  525  AACAATAGAACGAATACCATTATTTGTATGATCTGTTTTAATTACTGCAATTTTATTATT  584

Query  541  ATTATCTTTACCAGTCTTAGCTGGCGCAATTACAATATTATTAACTGATCGAAACTTTAA  600
            ||||||||||||||||||||||||||||||||||||||||||||||||||||||||||||
Sbjct  585  ATTATCTTTACCAGTCTTAGCTGGCGCAATTACAATATTATTAACTGATCGAAACTTTAA  644

Query  601  TACATCATTCTTTGACCCTTCAGGAGGAGGTGACCCAATTCTATATCAACATTTATTT  658
            ||||||||||||||||||||||||||||||||||||||||||||||||||||||||||
Sbjct  645  TACATCATTCTTTGACCCTTCAGGAGGAGGTGACCCAATTCTATATCAACATTTATTT  702
```

>Rhipicephalus sanguineus voucher DO-311 cytochrome oxidase subunit 1 (COI) gene, partial cds; mitochondrial
Sequence ID: KX360367.1 Length: 658
Range 1: 4 to 658

Score:1205 bits(652), Expect:0.0,
Identities:654/655(99%), Gaps:0/655(0%), Strand: Plus/Plus

```
Query  4    AATATACTTAATTTTTGGAGCATGATCCGGAATACTAGGATTAAGAATAAGAATATTAAT  63
            ||||||||||||||||||||||||||||||||||||||||||||||||||||||||||||
Sbjct  4    AATATACTTAATTTTTGGAGCATGATCCGGAATACTAGGATTAAGAATAAGAATATTAAT  63

Query  64   CCGCATAGAATTAGGTCAACCAGGAACTTTAATTGGAAACGATCAAATTTATAATGTAAT  123
            ||||||||||||||||||||||||||||||||||||||||||||||||||||||||||||
Sbjct  64   CCGCATAGAATTAGGTCAACCAGGAACTTTAATTGGAAACGATCAAATTTATAATGTAAT  123

Query  124  TGTAACAGCACATGCATTTATTATAAttttttttATAGTTATACCAATTATAATCGGAGG  183
            ||||||||||||||||||||||||||||||||||||||||||||||||||||||||||||
Sbjct  124  TGTAACAGCACATGCATTTATTATAATTTTTTTTATAGTTATACCAATTATAATCGGAGG  183

Query  184  ATTTGGAAATTGATTAGTTCCTATTATACTAGGAGCTCCAGATATAGCATTTCCACGAAT  243
            ||||||||||||||||||||||||||||||||||||||||||||||||||||||||||||
Sbjct  184  ATTTGGAAATTGATTAGTTCCTATTATACTAGGAGCTCCAGATATAGCATTTCCACGAAT  243

Query  244  AAATAATATAAGATTTTGGTTACTTCCTCCTTCTCTATTTCTACTAATTAATTCTTCATT  303
            ||||||||||||||||||||||||||||||||||||||||||||||||||||||||||||
Sbjct  244  AAATAATATAAGATTTTGGTTACTTCCTCCTTCTCTATTTCTACTAATTAATTCTTCATT  303

Query  304  AATCGAATCAGGAGCAGGTACAGGGTGAACAGTTTAcccccccTTATCATCAAATTTATC  363
            ||||||||||||||||||||||||||||||||||||||||||||||||||||||||||||
Sbjct  304  AATCGAATCAGGAGCAGGTACAGGGTGAACAGTTTACCCCCCCTTATCATCAAATTTATC  363

Query  364  ACACTACGGACCATCAGTAGATTTAGCTATTTTTTCTCTGCATCTTGCAGGTGCTTCTTC  423
            ||||||||||||||||||||||||||||||||||||||| ||||||||||||||||||||
Sbjct  364  ACACTACGGACCATCAGTAGATTTAGCTATTTTTTCTCTACATCTTGCAGGTGCTTCTTC  423
```

```
Query  424  AATTTTAGGTGCAATCAATTTCATTACAACTATTGTAAATATACGATCCATTGGAATAAC  483
            ||||||||||||||||||||||||||||||||||||||||||||||||||||||||||||
Sbjct  424  AATTTTAGGTGCAATCAATTTCATTACAACTATTGTAAATATACGATCCATTGGAATAAC  483

Query  484  AATAGAACGAATACCATTATTTGTATGATCTGTTTTAATTACTGCAATTTTATTATTATT  543
            ||||||||||||||||||||||||||||||||||||||||||||||||||||||||||||
Sbjct  484  AATAGAACGAATACCATTATTTGTATGATCTGTTTTAATTACTGCAATTTTATTATTATT  543

Query  544  ATCTTTACCAGTCTTAGCTGGCGCAATTACAATATTATTAACTGATCGAAACTTTAATAC  603
            ||||||||||||||||||||||||||||||||||||||||||||||||||||||||||||
Sbjct  544  ATCTTTACCAGTCTTAGCTGGCGCAATTACAATATTATTAACTGATCGAAACTTTAATAC  603

Query  604  ATCATTCTTTGACCCTTCAGGAGGAGGTGACCCAATTCTATATCAACATTTATTT  658
            |||||||||||||||||||||||||||||||||||||||||||||||||||||||
Sbjct  604  ATCATTCTTTGACCCTTCAGGAGGAGGTGACCCAATTCTATATCAACATTTATTT  658
```

```
>Rhipicephalus sanguineus haplotype RsanColSamCM cytochrome c oxidase subunit I
(COX1) gene, partial cds; mitochondrial
Sequence ID: KT906186.1 Length: 690
Range 1: 18 to 675

Score:1205 bits(652), Expect:0.0,
Identities:656/658(99%),  Gaps:0/658(0%), Strand: Plus/Plus

Query  1    GACAATATACTTAATTTTTGGAGCATGATCCGGAATACTAGGATTAAGAATAAGAATATT  60
            ||||||||||||||||||||||||||||||||||||| ||||||||||||||||||||||
Sbjct  18   GACAATATACTTAATTTTTGGAGCATGATCCGGAATATTAGGATTAAGAATAAGAATATT  77

Query  61   AATCCGCATAGAATTAGGTCAACCAGGAACTTTAATTGGAAACGATCAAATTTATAATGT  120
            ||||||||||||||||||||||||||||||||||||||||||||||||||||||||||||
Sbjct  78   AATCCGCATAGAATTAGGTCAACCAGGAACTTTAATTGGAAACGATCAAATTTATAATGT  137

Query  121  AATTGTAACAGCACATGCATTTATTATAAttttttttATAGTTATACCAATTATAATCGG  180
            ||||||||||||||||||||||||||||||||||||||||||||||||||||||||||||
Sbjct  138  AATTGTAACAGCACATGCATTTATTATAATTTTTTTTATAGTTATACCAATTATAATCGG  197

Query  181  AGGATTTGGAAATTGATTAGTTCCTATTATACTAGGAGCTCCAGATATAGCATTTCCACG  240
            |||||||||||||||||||||||||||||||||||| |||||||||||||||||||||||
Sbjct  198  AGGATTTGGAAATTGATTAGTTCCTATTATACTAGGGGCTCCAGATATAGCATTTCCACG  257

Query  241  AATAAATAATATAAGATTTTGGTTACTTCCTCCTTCTCTATTTCTACTAATTAATTCTTC  300
            ||||||||||||||||||||||||||||||||||||||||||||||||||||||||||||
Sbjct  258  AATAAATAATATAAGATTTTGGTTACTTCCTCCTTCTCTATTTCTACTAATTAATTCTTC  317

Query  301  ATTAATCGAATCAGGAGCAGGTACAGGGTGAACAGTTTAcccccccTTATCATCAAATTT  360
            ||||||||||||||||||||||||||||||||||||||||||||||||||||||||||||
Sbjct  318  ATTAATCGAATCAGGAGCAGGTACAGGGTGAACAGTTTACCCCCCCTTATCATCAAATTT  377

Query  361  ATCACACTACGGACCATCAGTAGATTTAGCTATTTTTTCTCTGCATCTTGCAGGTGCTTC  420
            ||||||||||||||||||||||||||||||||||||||||||||||||||||||||||||
Sbjct  378  ATCACACTACGGACCATCAGTAGATTTAGCTATTTTTTCTCTGCATCTTGCAGGTGCTTC  437
```

```
Query  421   TTCAATTTTAGGTGCAATCAATTTCATTACAACTATTGTAAATATACGATCCATTGGAAT  480
             ||||||||||||||||||||||||||||||||||||||||||||||||||||||||||||
Sbjct  438   TTCAATTTTAGGTGCAATCAATTTCATTACAACTATTGTAAATATACGATCCATTGGAAT  497

Query  481   AACAATAGAACGAATACCATTATTTGTATGATCTGTTTTAATTACTGCAATTTTATTATT  540
             ||||||||||||||||||||||||||||||||||||||||||||||||||||||||||||
Sbjct  498   AACAATAGAACGAATACCATTATTTGTATGATCTGTTTTAATTACTGCAATTTTATTATT  557

Query  541   ATTATCTTTACCAGTCTTAGCTGGCGCAATTACAATATTATTAACTGATCGAAACTTTAA  600
             ||||||||||||||||||||||||||||||||||||||||||||||||||||||||||||
Sbjct  558   ATTATCTTTACCAGTCTTAGCTGGCGCAATTACAATATTATTAACTGATCGAAACTTTAA  617

Query  601   TACATCATTCTTTGACCCTTCAGGAGGAGGTGACCCAATTCTATATCAACATTTATTT  658
             ||||||||||||||||||||||||||||||||||||||||||||||||||||||||||
Sbjct  618   TACATCATTCTTTGACCCTTCAGGAGGAGGTGACCCAATTCTATATCAACATTTATTT  675
```

>Rhipicephalus sanguineus voucher GRP5-154 cytochrome oxidase subunit 1 (COI) gene, partial cds; mitochondrial
Sequence ID: KF200112.1 Length: 658
>Rhipicephalus sanguineus voucher DO-309 cytochrome oxidase subunit 1 (COI) gene, partial cds; mitochondrial
Sequence ID: KX360403.1 Length: 658
Range 1: 1 to 658

Score:1205 bits(652), Expect:0.0,
Identities:656/658(99%), Gaps:0/658(0%), Strand: Plus/Plus

```
Query  1     GACAATATACTTAATTTTTGGAGCATGATCCGGAATACTAGGATTAAGAATAAGAATATT  60
             ||||||||||||||||||||||||||||||||||||| ||||||||||||||||||||||
Sbjct  1     GACAATATACTTAATTTTTGGAGCATGATCCGGAATATTAGGATTAAGAATAAGAATATT  60

Query  61    AATCCGCATAGAATTAGGTCAACCAGGAACTTTAATTGGAAACGATCAAATTTATAATGT  120
             ||||||||||||||||||||||||||||||||||||||||||||||||||||||||||||
Sbjct  61    AATCCGCATAGAATTAGGTCAACCAGGAACTTTAATTGGAAACGATCAAATTTATAATGT  120

Query  121   AATTGTAACAGCACATGCATTTATTATAAtttttttATAGTTATACCAATTATAATCGG  180
             ||||||||||||||||||||||||||||||||||||||||||||||||||||||||||||
Sbjct  121   AATTGTAACAGCACATGCATTTATTATAATTTTTTTATAGTTATACCAATTATAATCGG  180

Query  181   AGGATTTGGAAATTGATTAGTTCCTATTATACTAGGAGCTCCAGATATAGCATTTCCACG  240
             |||||||||||||||||||||||||||||||||||| |||||||||||||||||||||||
Sbjct  181   AGGATTTGGAAATTGATTAGTTCCTATTATACTAGGGGCTCCAGATATAGCATTTCCACG  240

Query  241   AATAAATAATATAAGATTTTGGTTACTTCCTCCTTCTCTATTTCTACTAATTAATTCTTC  300
             ||||||||||||||||||||||||||||||||||||||||||||||||||||||||||||
Sbjct  241   AATAAATAATATAAGATTTTGGTTACTTCCTCCTTCTCTATTTCTACTAATTAATTCTTC  300

Query  301   ATTAATCGAATCAGGAGCAGGTACAGGGTGAACAGTTTAcccccccTTATCATCAAATTT  360
             ||||||||||||||||||||||||||||||||||||||||||||||||||||||||||||
Sbjct  301   ATTAATCGAATCAGGAGCAGGTACAGGGTGAACAGTTTACCCCCCCTTATCATCAAATTT  360
```

```
Query  361   ATCACACTACGGACCATCAGTAGATTTAGCTATTTTTTCTCTGCATCTTGCAGGTGCTTC  420
             ||||||||||||||||||||||||||||||||||||||||||||||||||||||||||||
Sbjct  361   ATCACACTACGGACCATCAGTAGATTTAGCTATTTTTTCTCTGCATCTTGCAGGTGCTTC  420

Query  421   TTCAATTTTAGGTGCAATCAATTTCATTACAACTATTGTAAATATACGATCCATTGGAAT  480
             ||||||||||||||||||||||||||||||||||||||||||||||||||||||||||||
Sbjct  421   TTCAATTTTAGGTGCAATCAATTTCATTACAACTATTGTAAATATACGATCCATTGGAAT  480

Query  481   AACAATAGAACGAATACCATTATTTGTATGATCTGTTTTAATTACTGCAATTTTATTATT  540
             ||||||||||||||||||||||||||||||||||||||||||||||||||||||||||||
Sbjct  481   AACAATAGAACGAATACCATTATTTGTATGATCTGTTTTAATTACTGCAATTTTATTATT  540

Query  541   ATTATCTTTACCAGTCTTAGCTGGCGCAATTACAATATTATTAACTGATCGAAACTTTAA  600
             ||||||||||||||||||||||||||||||||||||||||||||||||||||||||||||
Sbjct  541   ATTATCTTTACCAGTCTTAGCTGGCGCAATTACAATATTATTAACTGATCGAAACTTTAA  600

Query  601   TACATCATTCTTTGACCCTTCAGGAGGAGGTGACCCAATTCTATATCAACATTTATTT  658
             ||||||||||||||||||||||||||||||||||||||||||||||||||||||||||
Sbjct  601   TACATCATTCTTTGACCCTTCAGGAGGAGGTGACCCAATTCTATATCAACATTTATTT  658
```

>Rhipicephalus sanguineus mitochondrion, complete genome
Sequence ID: JX416325.1 Length: 14714
Range 1: 1185 to 1842

Score:1205 bits(652), Expect:0.0,
Identities:656/658(99%), Gaps:0/658(0%), Strand: Plus/Plus

```
Query  1     GACAATATACTTAATTTTTGGAGCATGATCCGGAATACTAGGATTAAGAATAAGAATATT  60
             ||||||||||||||||||||||||||||||||||||||||||||||||||||||||||||
Sbjct  1185  GACAATATACTTAATTTTTGGAGCATGATCCGGAATACTAGGATTAAGAATAAGAATATT  1244

Query  61    AATCCGCATAGAATTAGGTCAACCAGGAACTTTAATTGGAAACGATCAAATTTATAATGT  120
             ||||||||||||||||||||||||||||||||||||||||||||||||||||||||||||
Sbjct  1245  AATCCGCATAGAATTAGGTCAACCAGGAACTTTAATTGGAAACGATCAAATTTATAATGT  1304

Query  121   AATTGTAACAGCACATGCATTTATTATAAttttttttATAGTTATACCAATTATAATCGG  180
             ||||||||||||||||||||||||||||||||||||||||||||||||||||||||||||
Sbjct  1305  AATTGTAACAGCACATGCATTTATTATAATTTTTTTTATAGTTATACCAATTATAATCGG  1364

Query  181   AGGATTTGGAAATTGATTAGTTCCTATTATACTAGGAGCTCCAGATATAGCATTTCCACG  240
             |||||||||||||||||||||||||||||||||||||||||||||| |||||||||||||
Sbjct  1365  AGGATTTGGAAATTGATTAGTTCCTATTATACTAGGAGCTCCAGATGTAGCATTTCCACG  1424

Query  241   AATAAATAATATAAGATTTTGGTTACTTCCTCCTTCTCTATTTCTACTAATTAATTCTTC  300
             ||||||||||||||||||||||||||||||||||||||||||||||||||||||||||||
Sbjct  1425  AATAAATAATATAAGATTTTGGTTACTTCCTCCTTCTCTATTTCTACTAATTAATTCTTC  1484

Query  301   ATTAATCGAATCAGGAGCAGGTACAGGGTGAACAGTTTAcccccccTTATCATCAAATTT  360
             ||||||||||||||||||||||||||||||||||||||||||||||||||||||||||||
Sbjct  1485  ATTAATCGAATCAGGAGCAGGTACAGGGTGAACAGTTTACCCCCCCTTATCATCAAATTT  1544
```

```
Query  361   ATCACACTACGGACCATCAGTAGATTTAGCTATTTTTTCTCTGCATCTTGCAGGTGCTTC  420
             ||||||||||||||||||||||||||||||||||||||||||||||||||||||||||||
Sbjct  1545  ATCACACTACGGACCATCAGTAGATTTAGCTATTTTTTCTCTGCATCTTGCAGGTGCTTC  1604

Query  421   TTCAATTTTAGGTGCAATCAATTTCATTACAACTATTGTAAATATACGATCCATTGGAAT  480
             ||||||||||||||||||||||||||||||||||||||||||||||||||||||||||||
Sbjct  1605  TTCAATTTTAGGTGCAATCAATTTCATTACAACTATTGTAAATATACGATCCATTGGAAT  1664

Query  481   AACAATAGAACGAATACCATTATTTGTATGATCTGTTTTAATTACTGCAATTTTATTATT  540
             ||||||||||||||||||||||||||||||||||||||||||||||||||||||||||||
Sbjct  1665  AACAATAGAACGAATACCATTATTTGTATGATCTGTTTTAATTACTGCAATTTTATTATT  1724

Query  541   ATTATCTTTACCAGTCTTAGCTGGCGCAATTACAATATTATTAACTGATCGAAACTTTAA  600
             ||||||||||||||||||||||||||||||||||||||||||||||||||||||||||||
Sbjct  1725  ATTATCTTTACCAGTCTTAGCTGGCGCAATTACAATATTATTAACTGATCGAAACTTTAA  1784

Query  601   TACATCATTCTTTGACCCTTCAGGAGGAGGTGACCCAATTCTATATCAACATTTATTT  658
             |||||||||||||||||||||||||||||||||||||||||||||||||| |||||||
Sbjct  1785  TACATCATTCTTTGACCCTTCAGGAGGAGGTGACCCAATTCTATATCAACGTTTATTT  1842
```

>Rhipicephalus sanguineus isolate T95D15 cytochrome c oxidase subunit I (COX1) gene, partial cds; mitochondrial
Sequence ID: MZ401443.1 Length: 661
Range 1: 1 to 645

Score:1192 bits(645), Expect:0.0,
Identities:645/645(100%), Gaps:0/645(0%), Strand: Plus/Plus

```
Query  14   ATTTTTGGAGCATGATCCGGAATACTAGGATTAAGAATAAGAATATTAATCCGCATAGAA  73
            ||||||||||||||||||||||||||||||||||||||||||||||||||||||||||||
Sbjct  1    ATTTTTGGAGCATGATCCGGAATACTAGGATTAAGAATAAGAATATTAATCCGCATAGAA  60

Query  74   TTAGGTCAACCAGGAACTTTAATTGGAAACGATCAAATTTATAATGTAATTGTAACAGCA  133
            ||||||||||||||||||||||||||||||||||||||||||||||||||||||||||||
Sbjct  61   TTAGGTCAACCAGGAACTTTAATTGGAAACGATCAAATTTATAATGTAATTGTAACAGCA  120

Query  134  CATGCATTTATTATAAttttttttATAGTTATACCAATTATAATCGGAGGATTTGGAAAT  193
            ||||||||||||||||||||||||||||||||||||||||||||||||||||||||||||
Sbjct  121  CATGCATTTATTATAATTTTTTTTATAGTTATACCAATTATAATCGGAGGATTTGGAAAT  180

Query  194  TGATTAGTTCCTATTATACTAGGAGCTCCAGATATAGCATTTCCACGAATAAATAATATA  253
            ||||||||||||||||||||||||||||||||||||||||||||||||||||||||||||
Sbjct  181  TGATTAGTTCCTATTATACTAGGAGCTCCAGATATAGCATTTCCACGAATAAATAATATA  240

Query  254  AGATTTTGGTTACTTCCTCCTTCTCTATTTCTACTAATTAATTCTTCATTAATCGAATCA  313
            ||||||||||||||||||||||||||||||||||||||||||||||||||||||||||||
Sbjct  241  AGATTTTGGTTACTTCCTCCTTCTCTATTTCTACTAATTAATTCTTCATTAATCGAATCA  300

Query  314  GGAGCAGGTACAGGGTGAACAGTTTAcccccccTTATCATCAAATTTATCACACTACGGA  373
            ||||||||||||||||||||||||||||||||||||||||||||||||||||||||||||
```

```
Sbjct  301   GGAGCAGGTACAGGGTGAACAGTTTACCCCCCCTTATCATCAAATTTATCACACTACGGA  360

Query  374   CCATCAGTAGATTTAGCTATTTTTTCTCTGCATCTTGCAGGTGCTTCTTCAATTTTAGGT  433
             ||||||||||||||||||||||||||||||||||||||||||||||||||||||||||||
Sbjct  361   CCATCAGTAGATTTAGCTATTTTTTCTCTGCATCTTGCAGGTGCTTCTTCAATTTTAGGT  420

Query  434   GCAATCAATTTCATTACAACTATTGTAAATATACGATCCATTGGAATAACAATAGAACGA  493
             ||||||||||||||||||||||||||||||||||||||||||||||||||||||||||||
Sbjct  421   GCAATCAATTTCATTACAACTATTGTAAATATACGATCCATTGGAATAACAATAGAACGA  480

Query  494   ATACCATTATTTGTATGATCTGTTTTAATTACTGCAATTTTATTATTATTATCTTTACCA  553
             ||||||||||||||||||||||||||||||||||||||||||||||||||||||||||||
Sbjct  481   ATACCATTATTTGTATGATCTGTTTTAATTACTGCAATTTTATTATTATTATCTTTACCA  540

Query  554   GTCTTAGCTGGCGCAATTACAATATTATTAACTGATCGAAACTTTAATACATCATTCTTT  613
             ||||||||||||||||||||||||||||||||||||||||||||||||||||||||||||
Sbjct  541   GTCTTAGCTGGCGCAATTACAATATTATTAACTGATCGAAACTTTAATACATCATTCTTT  600

Query  614   GACCCTTCAGGAGGAGGTGACCCAATTCTATATCAACATTTATTT  658
             |||||||||||||||||||||||||||||||||||||||||||||
Sbjct  601   GACCCTTCAGGAGGAGGTGACCCAATTCTATATCAACATTTATTT  645
```

>Rhipicephalus sanguineus isolate T81D7 cytochrome c oxidase subunit I (COX1) gene, partial cds; mitochondrial
Sequence ID: MZ401441.1 Length: 661
Range 1: 1 to 645

Score:1192 bits(645), Expect:0.0,
Identities:645/645(100%), Gaps:0/645(0%), Strand: Plus/Plus

```
Query  14    ATTTTTGGAGCATGATCCGGAATACTAGGATTAAGAATAAGAATATTAATCCGCATAGAA  73
             ||||||||||||||||||||||||||||||||||||||||||||||||||||||||||||
Sbjct  1     ATTTTTGGAGCATGATCCGGAATACTAGGATTAAGAATAAGAATATTAATCCGCATAGAA  60

Query  74    TTAGGTCAACCAGGAACTTTAATTGGAAACGATCAAATTTATAATGTAATTGTAACAGCA  133
             ||||||||||||||||||||||||||||||||||||||||||||||||||||||||||||
Sbjct  61    TTAGGTCAACCAGGAACTTTAATTGGAAACGATCAAATTTATAATGTAATTGTAACAGCA  120

Query  134   CATGCATTTATTATAAttttttttATAGTTATACCAATTATAATCGGAGGATTTGGAAAT  193
             ||||||||||||||||||||||||||||||||||||||||||||||||||||||||||||
Sbjct  121   CATGCATTTATTATAATTTTTTTTATAGTTATACCAATTATAATCGGAGGATTTGGAAAT  180

Query  194   TGATTAGTTCCTATTATACTAGGAGCTCCAGATATAGCATTTCCACGAATAAATAATATA  253
             ||||||||||||||||||||||||||||||||||||||||||||||||||||||||||||
Sbjct  181   TGATTAGTTCCTATTATACTAGGAGCTCCAGATATAGCATTTCCACGAATAAATAATATA  240

Query  254   AGATTTTGGTTACTTCCTCCTTCTCTATTTCTACTAATTAATTCTTCATTAATCGAATCA  313
             ||||||||||||||||||||||||||||||||||||||||||||||||||||||||||||
Sbjct  241   AGATTTTGGTTACTTCCTCCTTCTCTATTTCTACTAATTAATTCTTCATTAATCGAATCA  300

Query  314   GGAGCAGGTACAGGGTGAACAGTTTAcccccccTTATCATCAAATTTATCACACTACGGA  373
```

```
               ||||||||||||||||||||||||||||||||||||||||||||||||||||||||||||
Sbjct  301  GGAGCAGGTACAGGGTGAACAGTTTACCCCCCCTTATCATCAAATTTATCACACTACGGA  360

Query  374  CCATCAGTAGATTTAGCTATTTTTTCTCTGCATCTTGCAGGTGCTTCTTCAATTTTAGGT  433
            ||||||||||||||||||||||||||||||||||||||||||||||||||||||||||||
Sbjct  361  CCATCAGTAGATTTAGCTATTTTTTCTCTGCATCTTGCAGGTGCTTCTTCAATTTTAGGT  420

Query  434  GCAATCAATTTCATTACAACTATTGTAAATATACGATCCATTGGAATAACAATAGAACGA  493
            ||||||||||||||||||||||||||||||||||||||||||||||||||||||||||||
Sbjct  421  GCAATCAATTTCATTACAACTATTGTAAATATACGATCCATTGGAATAACAATAGAACGA  480

Query  494  ATACCATTATTTGTATGATCTGTTTTAATTACTGCAATTTTATTATTATTATCTTTACCA  553
            ||||||||||||||||||||||||||||||||||||||||||||||||||||||||||||
Sbjct  481  ATACCATTATTTGTATGATCTGTTTTAATTACTGCAATTTTATTATTATTATCTTTACCA  540

Query  554  GTCTTAGCTGGCGCAATTACAATATTATTAACTGATCGAAACTTTAATACATCATTCTTT  613
            ||||||||||||||||||||||||||||||||||||||||||||||||||||||||||||
Sbjct  541  GTCTTAGCTGGCGCAATTACAATATTATTAACTGATCGAAACTTTAATACATCATTCTTT  600

Query  614  GACCCTTCAGGAGGAGGTGACCCAATTCTATATCAACATTTATTT  658
            |||||||||||||||||||||||||||||||||||||||||||||
Sbjct  601  GACCCTTCAGGAGGAGGTGACCCAATTCTATATCAACATTTATTT  645
```

>Rhipicephalus sanguineus isolate T79D2 cytochrome c oxidase subunit I (COX1) gene, partial cds; mitochondrial
Sequence ID: MZ401440.1 Length: 664
Range 1: 1 to 648

Score:1192 bits(645), Expect:0.0,
Identities:647/648(99%), Gaps:0/648(0%), Strand: Plus/Plus

```
Query  11   TTAATTTTTGGAGCATGATCCGGAATACTAGGATTAAGAATAAGAATATTAATCCGCATA  70
            ||||||||||||||||||||||||||||||||||||||||||||||||||||||||||||
Sbjct  1    TTAATTTTTGGAGCATGATCCGGAATACTAGGATTAAGAATAAGAATATTAATCCGCATA  60

Query  71   GAATTAGGTCAACCAGGAACTTTAATTGGAAACGATCAAATTTATAATGTAATTGTAACA  130
            ||||||||||||||||||||||||||||||||||||||||||||||||||||||||||||
Sbjct  61   GAATTAGGTCAACCAGGAACTTTAATTGGAAACGATCAAATTTATAATGTAATTGTAACA  120

Query  131  GCACATGCATTTATTATAAtttttttATAGTTATACCAATTATAATCGGAGGATTTGGA  190
            ||||||||||||||||||||||||||||||||||||||||||||||||||||||||||||
Sbjct  121  GCACATGCATTTATTATAATTTTTTTATAGTTATACCAATTATAATCGGAGGATTTGGA  180

Query  191  AATTGATTAGTTCCTATTATACTAGGAGCTCCAGATATAGCATTTCCACGAATAAATAAT  250
            ||||||||||||||||||||||||||||||||||||||||||||||||||||||||||||
Sbjct  181  AATTGATTAGTTCCTATTATACTAGGAGCTCCAGATATAGCATTTCCACGAATAAATAAT  240

Query  251  ATAAGATTTTGGTTACTTCCTCCTTCTCTATTTCTACTAATTAATTCTTCATTAATCGAA  310
            ||||||||||||||||||||||||||||||||||||||||||||||||||||||||||||
Sbjct  241  ATAAGATTTTGGTTACTTCCTCCTTCTCTATTTCTACTAATTAATTCTTCATTAATCGAA  300
```

```
Query  311   TCAGGAGCAGGTACAGGGTGAACAGTTTAcccccccTTATCATCAAATTTATCACACTAC  370
             ||||||||||| ||||||||||||||||||||||||||||||||||||||||||||||||
Sbjct  301   TCAGGAGCAGGAACAGGGTGAACAGTTTACCCCCCCTTATCATCAAATTTATCACACTAC  360

Query  371   GGACCATCAGTAGATTTAGCTATTTTTTCTCTGCATCTTGCAGGTGCTTCTTCAATTTTA  430
             ||||||||||||||||||||||||||||||||||||||||||||||||||||||||||||
Sbjct  361   GGACCATCAGTAGATTTAGCTATTTTTTCTCTGCATCTTGCAGGTGCTTCTTCAATTTTA  420

Query  431   GGTGCAATCAATTTCATTACAACTATTGTAAATATACGATCCATTGGAATAACAATAGAA  490
             ||||||||||||||||||||||||||||||||||||||||||||||||||||||||||||
Sbjct  421   GGTGCAATCAATTTCATTACAACTATTGTAAATATACGATCCATTGGAATAACAATAGAA  480

Query  491   CGAATACCATTATTTGTATGATCTGTTTTAATTACTGCAATTTTATTATTATTATCTTTA  550
             ||||||||||||||||||||||||||||||||||||||||||||||||||||||||||||
Sbjct  481   CGAATACCATTATTTGTATGATCTGTTTTAATTACTGCAATTTTATTATTATTATCTTTA  540

Query  551   CCAGTCTTAGCTGGCGCAATTACAATATTATTAACTGATCGAAACTTTAATACATCATTC  610
             ||||||||||||||||||||||||||||||||||||||||||||||||||||||||||||
Sbjct  541   CCAGTCTTAGCTGGCGCAATTACAATATTATTAACTGATCGAAACTTTAATACATCATTC  600

Query  611   TTTGACCCTTCAGGAGGAGGTGACCCAATTCTATATCAACATTTATTT  658
             ||||||||||||||||||||||||||||||||||||||||||||||||
Sbjct  601   TTTGACCCTTCAGGAGGAGGTGACCCAATTCTATATCAACATTTATTT  648
```

>Rhipicephalus sanguineus isolate WZ004 cytochrome oxidase subunit I gene, partial cds; mitochondrial
Sequence ID: MG969507.1 Length: 664
Range 1: 1 to 644

Score:1190 bits(644), Expect:0.0,
Identities:644/644(100%), Gaps:0/644(0%), Strand: Plus/Plus

```
Query  15    TTTTTGGAGCATGATCCGGAATACTAGGATTAAGAATAAGAATATTAATCCGCATAGAAT  74
             ||||||||||||||||||||||||||||||||||||||||||||||||||||||||||||
Sbjct  1     TTTTTGGAGCATGATCCGGAATACTAGGATTAAGAATAAGAATATTAATCCGCATAGAAT  60

Query  75    TAGGTCAACCAGGAACTTTAATTGGAAACGATCAAATTTATAATGTAATTGTAACAGCAC  134
             ||||||||||||||||||||||||||||||||||||||||||||||||||||||||||||
Sbjct  61    TAGGTCAACCAGGAACTTTAATTGGAAACGATCAAATTTATAATGTAATTGTAACAGCAC  120

Query  135   ATGCATTTATTATAAttttttttATAGTTATACCAATTATAATCGGAGGATTTGGAAATT  194
             ||||||||||||||||||||||||||||||||||||||||||||||||||||||||||||
Sbjct  121   ATGCATTTATTATAATTTTTTTTATAGTTATACCAATTATAATCGGAGGATTTGGAAATT  180

Query  195   GATTAGTTCCTATTATACTAGGAGCTCCAGATATAGCATTTCCACGAATAAATAATATAA  254
             ||||||||||||||||||||||||||||||||||||||||||||||||||||||||||||
Sbjct  181   GATTAGTTCCTATTATACTAGGAGCTCCAGATATAGCATTTCCACGAATAAATAATATAA  240

Query  255   GATTTTGGTTACTTCCTCCTTCTCTATTTCTACTAATTAATTCTTCATTAATCGAATCAG  314
             ||||||||||||||||||||||||||||||||||||||||||||||||||||||||||||
Sbjct  241   GATTTTGGTTACTTCCTCCTTCTCTATTTCTACTAATTAATTCTTCATTAATCGAATCAG  300
```

```
Query  315   GAGCAGGTACAGGGTGAACAGTTTAcccccccTTATCATCAAATTTATCACACTACGGAC  374
             ||||||||||||||||||||||||||||||||||||||||||||||||||||||||||||
Sbjct  301   GAGCAGGTACAGGGTGAACAGTTTACCCCCCCTTATCATCAAATTTATCACACTACGGAC  360

Query  375   CATCAGTAGATTTAGCTATTTTTTCTCTGCATCTTGCAGGTGCTTCTTCAATTTTAGGTG  434
             ||||||||||||||||||||||||||||||||||||||||||||||||||||||||||||
Sbjct  361   CATCAGTAGATTTAGCTATTTTTTCTCTGCATCTTGCAGGTGCTTCTTCAATTTTAGGTG  420

Query  435   CAATCAATTTCATTACAACTATTGTAAATATACGATCCATTGGAATAACAATAGAACGAA  494
             ||||||||||||||||||||||||||||||||||||||||||||||||||||||||||||
Sbjct  421   CAATCAATTTCATTACAACTATTGTAAATATACGATCCATTGGAATAACAATAGAACGAA  480

Query  495   TACCATTATTTGTATGATCTGTTTTAATTACTGCAATTTTATTATTATTATCTTTACCAG  554
             ||||||||||||||||||||||||||||||||||||||||||||||||||||||||||||
Sbjct  481   TACCATTATTTGTATGATCTGTTTTAATTACTGCAATTTTATTATTATTATCTTTACCAG  540

Query  555   TCTTAGCTGGCGCAATTACAATATTATTAACTGATCGAAACTTTAATACATCATTCTTTG  614
             ||||||||||||||||||||||||||||||||||||||||||||||||||||||||||||
Sbjct  541   TCTTAGCTGGCGCAATTACAATATTATTAACTGATCGAAACTTTAATACATCATTCTTTG  600

Query  615   ACCCTTCAGGAGGAGGTGACCCAATTCTATATCAACATTTATTT  658
             ||||||||||||||||||||||||||||||||||||||||||||
Sbjct  601   ACCCTTCAGGAGGAGGTGACCCAATTCTATATCAACATTTATTT  644
```

```
>Rhipicephalus sanguineus voucher GRP5-150 cytochrome oxidase subunit 1 (COI)
gene, partial cds; mitochondrial
Sequence ID: KF200084.1 Length: 649
Range 1: 1 to 649

Score:1188 bits(643), Expect:0.0,
Identities:647/649(99%),  Gaps:0/649(0%), Strand: Plus/Plus

Query  10    CTTAATTTTTGGAGCATGATCCGGAATACTAGGATTAAGAATAAGAATATTAATCCGCAT  69
             |||||||||||||||||||||||||||| |||||||||||||||||||||||||||||||
Sbjct  1     CTTAATTTTTGGAGCATGATCCGGAATATTAGGATTAAGAATAAGAATATTAATCCGCAT  60

Query  70    AGAATTAGGTCAACCAGGAACTTTAATTGGAAACGATCAAATTTATAATGTAATTGTAAC  129
             ||||||||||||||||||||||||||||||||||||||||||||||||||||||||||||
Sbjct  61    AGAATTAGGTCAACCAGGAACTTTAATTGGAAACGATCAAATTTATAATGTAATTGTAAC  120

Query  130   AGCACATGCATTTATTATAAttttttttATAGTTATACCAATTATAATCGGAGGATTTGG  189
             ||||||||||||||||||||||||||||||||||||||||||||||||||||||||||||
Sbjct  121   AGCACATGCATTTATTATAATTTTTTTTATAGTTATACCAATTATAATCGGAGGATTTGG  180

Query  190   AAATTGATTAGTTCCTATTATACTAGGAGCTCCAGATATAGCATTTCCACGAATAAATAA  249
             ||||||||||||||||||||||||||| ||||||||||||||||||||||||||||||||
Sbjct  181   AAATTGATTAGTTCCTATTATACTAGGGGCTCCAGATATAGCATTTCCACGAATAAATAA  240

Query  250   TATAAGATTTTGGTTACTTCCTCCTTCTCTATTTCTACTAATTAATTCTTCATTAATCGA  309
             ||||||||||||||||||||||||||||||||||||||||||||||||||||||||||||
```

```
Sbjct  241   TATAAGATTTTGGTTACTTCCTCCTTCTCTATTTCTACTAATTAATTCTTCATTAATCGA  300

Query  310   ATCAGGAGCAGGTACAGGGTGAACAGTTTAccccccccTTATCATCAAATTTATCACACTA  369
             ||||||||||||||||||||||||||||||||||||||||||||||||||||||||||||
Sbjct  301   ATCAGGAGCAGGTACAGGGTGAACAGTTTACCCCCCCCTTATCATCAAATTTATCACACTA  360

Query  370   CGGACCATCAGTAGATTTAGCTATTTTTTCTCTGCATCTTGCAGGTGCTTCTTCAATTTT  429
             ||||||||||||||||||||||||||||||||||||||||||||||||||||||||||||
Sbjct  361   CGGACCATCAGTAGATTTAGCTATTTTTTCTCTGCATCTTGCAGGTGCTTCTTCAATTTT  420

Query  430   AGGTGCAATCAATTTCATTACAACTATTGTAAATATACGATCCATTGGAATAACAATAGA  489
             ||||||||||||||||||||||||||||||||||||||||||||||||||||||||||||
Sbjct  421   AGGTGCAATCAATTTCATTACAACTATTGTAAATATACGATCCATTGGAATAACAATAGA  480

Query  490   ACGAATACCATTATTTGTATGATCTGTTTTAATTACTGCAATTTTATTATTATTATCTTT  549
             ||||||||||||||||||||||||||||||||||||||||||||||||||||||||||||
Sbjct  481   ACGAATACCATTATTTGTATGATCTGTTTTAATTACTGCAATTTTATTATTATTATCTTT  540

Query  550   ACCAGTCTTAGCTGGCGCAATTACAATATTATTAACTGATCGAAACTTTAATACATCATT  609
             ||||||||||||||||||||||||||||||||||||||||||||||||||||||||||||
Sbjct  541   ACCAGTCTTAGCTGGCGCAATTACAATATTATTAACTGATCGAAACTTTAATACATCATT  600

Query  610   CTTTGACCCTTCAGGAGGAGGTGACCCAATTCTATATCAACATTTATTT  658
             |||||||||||||||||||||||||||||||||||||||||||||||||
Sbjct  601   CTTTGACCCTTCAGGAGGAGGTGACCCAATTCTATATCAACATTTATTT  649
```

>Rhipicephalus sanguineus isolate LIC6084B cytochrome oxidase subunit 1 (COI) gene, partial cds; mitochondrial
Sequence ID: KX383817.1 Length: 768
Range 1: 1 to 642

Score:1186 bits(642), Expect:0.0,
Identities:642/642(100%), Gaps:0/642(0%), Strand: Plus/Plus

```
Query  17    TTTGGAGCATGATCCGGAATACTAGGATTAAGAATAAGAATATTAATCCGCATAGAATTA  76
             ||||||||||||||||||||||||||||||||||||||||||||||||||||||||||||
Sbjct  1     TTTGGAGCATGATCCGGAATACTAGGATTAAGAATAAGAATATTAATCCGCATAGAATTA  60

Query  77    GGTCAACCAGGAACTTTAATTGGAAACGATCAAATTTATAATGTAATTGTAACAGCACAT  136
             ||||||||||||||||||||||||||||||||||||||||||||||||||||||||||||
Sbjct  61    GGTCAACCAGGAACTTTAATTGGAAACGATCAAATTTATAATGTAATTGTAACAGCACAT  120

Query  137   GCATTTATTATAAttttttttATAGTTATACCAATTATAATCGGAGGATTTGGAAATTGA  196
             ||||||||||||||||||||||||||||||||||||||||||||||||||||||||||||
Sbjct  121   GCATTTATTATAATTTTTTTTATAGTTATACCAATTATAATCGGAGGATTTGGAAATTGA  180

Query  197   TTAGTTCCTATTATACTAGGAGCTCCAGATATAGCATTTCCACGAATAAATAATATAAGA  256
             ||||||||||||||||||||||||||||||||||||||||||||||||||||||||||||
Sbjct  181   TTAGTTCCTATTATACTAGGAGCTCCAGATATAGCATTTCCACGAATAAATAATATAAGA  240

Query  257   TTTTGGTTACTTCCTCCTTCTCTATTTCTACTAATTAATTCTTCATTAATCGAATCAGGA  316
```

```
          ||||||||||||||||||||||||||||||||||||||||||||||||||||||||||||
Sbjct  241  TTTTGGTTACTTCCTCCTTCTCTATTTCTACTAATTAATTCTTCATTAATCGAATCAGGA  300

Query  317  GCAGGTACAGGGTGAACAGTTTAcccccccTTATCATCAAATTTATCACACTACGGACCA  376
            ||||||||||||||||||||||||||||||||||||||||||||||||||||||||||||
Sbjct  301  GCAGGTACAGGGTGAACAGTTTACCCCCCCTTATCATCAAATTTATCACACTACGGACCA  360

Query  377  TCAGTAGATTTAGCTATTTTTTCTCTGCATCTTGCAGGTGCTTCTTCAATTTTAGGTGCA  436
            ||||||||||||||||||||||||||||||||||||||||||||||||||||||||||||
Sbjct  361  TCAGTAGATTTAGCTATTTTTTCTCTGCATCTTGCAGGTGCTTCTTCAATTTTAGGTGCA  420

Query  437  ATCAATTTCATTACAACTATTGTAAATATACGATCCATTGGAATAACAATAGAACGAATA  496
            ||||||||||||||||||||||||||||||||||||||||||||||||||||||||||||
Sbjct  421  ATCAATTTCATTACAACTATTGTAAATATACGATCCATTGGAATAACAATAGAACGAATA  480

Query  497  CCATTATTTGTATGATCTGTTTTAATTACTGCAATTTTATTATTATTATCTTTACCAGTC  556
            ||||||||||||||||||||||||||||||||||||||||||||||||||||||||||||
Sbjct  481  CCATTATTTGTATGATCTGTTTTAATTACTGCAATTTTATTATTATTATCTTTACCAGTC  540

Query  557  TTAGCTGGCGCAATTACAATATTATTAACTGATCGAAACTTTAATACATCATTCTTTGAC  616
            ||||||||||||||||||||||||||||||||||||||||||||||||||||||||||||
Sbjct  541  TTAGCTGGCGCAATTACAATATTATTAACTGATCGAAACTTTAATACATCATTCTTTGAC  600

Query  617  CCTTCAGGAGGAGGTGACCCAATTCTATATCAACATTTATTT  658
            ||||||||||||||||||||||||||||||||||||||||||
Sbjct  601  CCTTCAGGAGGAGGTGACCCAATTCTATATCAACATTTATTT  642
```

>Rhipicephalus sanguineus isolate T91D13 cytochrome c oxidase subunit I (COX1) gene, partial cds; mitochondrial
Sequence ID: MZ401442.1 Length: 657
Range 1: 1 to 642

Score:1186 bits(642), Expect:0.0,
Identities:642/642(100%), Gaps:0/642(0%), Strand: Plus/Plus

```
Query  17   TTTGGAGCATGATCCGGAATACTAGGATTAAGAATAAGAATATTAATCCGCATAGAATTA  76
            ||||||||||||||||||||||||||||||||||||||||||||||||||||||||||||
Sbjct  1    TTTGGAGCATGATCCGGAATACTAGGATTAAGAATAAGAATATTAATCCGCATAGAATTA  60

Query  77   GGTCAACCAGGAACTTTAATTGGAAACGATCAAATTTATAATGTAATTGTAACAGCACAT  136
            ||||||||||||||||||||||||||||||||||||||||||||||||||||||||||||
Sbjct  61   GGTCAACCAGGAACTTTAATTGGAAACGATCAAATTTATAATGTAATTGTAACAGCACAT  120

Query  137  GCATTTATTATAAttttttttATAGTTATACCAATTATAATCGGAGGATTTGGAAATTGA  196
            ||||||||||||||||||||||||||||||||||||||||||||||||||||||||||||
Sbjct  121  GCATTTATTATAATTTTTTTTATAGTTATACCAATTATAATCGGAGGATTTGGAAATTGA  180

Query  197  TTAGTTCCTATTATACTAGGAGCTCCAGATATAGCATTTCCACGAATAAATAATATAAGA  256
            ||||||||||||||||||||||||||||||||||||||||||||||||||||||||||||
Sbjct  181  TTAGTTCCTATTATACTAGGAGCTCCAGATATAGCATTTCCACGAATAAATAATATAAGA  240
```

```
Query  257   TTTTGGTTACTTCCTCCTTCTCTATTTCTACTAATTAATTCTTCATTAATCGAATCAGGA  316
             ||||||||||||||||||||||||||||||||||||||||||||||||||||||||||||
Sbjct  241   TTTTGGTTACTTCCTCCTTCTCTATTTCTACTAATTAATTCTTCATTAATCGAATCAGGA  300

Query  317   GCAGGTACAGGGTGAACAGTTTAcccccccTTATCATCAAATTTATCACACTACGGACCA  376
             ||||||||||||||||||||||||||||||||||||||||||||||||||||||||||||
Sbjct  301   GCAGGTACAGGGTGAACAGTTTACCCCCCCTTATCATCAAATTTATCACACTACGGACCA  360

Query  377   TCAGTAGATTTAGCTATTTTTTCTCTGCATCTTGCAGGTGCTTCTTCAATTTTAGGTGCA  436
             ||||||||||||||||||||||||||||||||||||||||||||||||||||||||||||
Sbjct  361   TCAGTAGATTTAGCTATTTTTTCTCTGCATCTTGCAGGTGCTTCTTCAATTTTAGGTGCA  420

Query  437   ATCAATTTCATTACAACTATTGTAAATATACGATCCATTGGAATAACAATAGAACGAATA  496
             ||||||||||||||||||||||||||||||||||||||||||||||||||||||||||||
Sbjct  421   ATCAATTTCATTACAACTATTGTAAATATACGATCCATTGGAATAACAATAGAACGAATA  480

Query  497   CCATTATTTGTATGATCTGTTTTAATTACTGCAATTTTATTATTATTATCTTTACCAGTC  556
             ||||||||||||||||||||||||||||||||||||||||||||||||||||||||||||
Sbjct  481   CCATTATTTGTATGATCTGTTTTAATTACTGCAATTTTATTATTATTATCTTTACCAGTC  540

Query  557   TTAGCTGGCGCAATTACAATATTATTAACTGATCGAAACTTTAATACATCATTCTTTGAC  616
             ||||||||||||||||||||||||||||||||||||||||||||||||||||||||||||
Sbjct  541   TTAGCTGGCGCAATTACAATATTATTAACTGATCGAAACTTTAATACATCATTCTTTGAC  600

Query  617   CCTTCAGGAGGAGGTGACCCAATTCTATATCAACATTTATTT  658
             ||||||||||||||||||||||||||||||||||||||||||
Sbjct  601   CCTTCAGGAGGAGGTGACCCAATTCTATATCAACATTTATTT  642
```

>Rhipicephalus sanguineus isolate T63D1 cytochrome c oxidase subunit I (COX1) gene, partial cds; mitochondrial
Sequence ID: MZ401438.1 Length: 661
Range 1: 1 to 642

Score:1186 bits(642), Expect:0.0,
Identities:642/642(100%), Gaps:0/642(0%), Strand: Plus/Plus

```
Query  17    TTTGGAGCATGATCCGGAATACTAGGATTAAGAATAAGAATATTAATCCGCATAGAATTA  76
             ||||||||||||||||||||||||||||||||||||||||||||||||||||||||||||
Sbjct  1     TTTGGAGCATGATCCGGAATACTAGGATTAAGAATAAGAATATTAATCCGCATAGAATTA  60

Query  77    GGTCAACCAGGAACTTTAATTGGAAACGATCAAATTTATAATGTAATTGTAACAGCACAT  136
             ||||||||||||||||||||||||||||||||||||||||||||||||||||||||||||
Sbjct  61    GGTCAACCAGGAACTTTAATTGGAAACGATCAAATTTATAATGTAATTGTAACAGCACAT  120

Query  137   GCATTTATTATAAtttttttATAGTTATACCAATTATAATCGGAGGATTTGGAAATTGA  196
             ||||||||||||||||||||||||||||||||||||||||||||||||||||||||||||
Sbjct  121   GCATTTATTATAATTTTTTTATAGTTATACCAATTATAATCGGAGGATTTGGAAATTGA  180

Query  197   TTAGTTCCTATTATACTAGGAGCTCCAGATATAGCATTTCCACGAATAAATAATATAAGA  256
             ||||||||||||||||||||||||||||||||||||||||||||||||||||||||||||
Sbjct  181   TTAGTTCCTATTATACTAGGAGCTCCAGATATAGCATTTCCACGAATAAATAATATAAGA  240
```

```
Query  257   TTTTGGTTACTTCCTCCTTCTCTATTTCTACTAATTAATTCTTCATTAATCGAATCAGGA  316
             ||||||||||||||||||||||||||||||||||||||||||||||||||||||||||||
Sbjct  241   TTTTGGTTACTTCCTCCTTCTCTATTTCTACTAATTAATTCTTCATTAATCGAATCAGGA  300

Query  317   GCAGGTACAGGGTGAACAGTTTAcccccccTTATCATCAAATTTATCACACTACGGACCA  376
             ||||||||||||||||||||||||||||||||||||||||||||||||||||||||||||
Sbjct  301   GCAGGTACAGGGTGAACAGTTTACCCCCCCTTATCATCAAATTTATCACACTACGGACCA  360

Query  377   TCAGTAGATTTAGCTATTTTTTCTCTGCATCTTGCAGGTGCTTCTTCAATTTTAGGTGCA  436
             ||||||||||||||||||||||||||||||||||||||||||||||||||||||||||||
Sbjct  361   TCAGTAGATTTAGCTATTTTTTCTCTGCATCTTGCAGGTGCTTCTTCAATTTTAGGTGCA  420

Query  437   ATCAATTTCATTACAACTATTGTAAATATACGATCCATTGGAATAACAATAGAACGAATA  496
             ||||||||||||||||||||||||||||||||||||||||||||||||||||||||||||
Sbjct  421   ATCAATTTCATTACAACTATTGTAAATATACGATCCATTGGAATAACAATAGAACGAATA  480

Query  497   CCATTATTTGTATGATCTGTTTTAATTACTGCAATTTTATTATTATTATCTTTACCAGTC  556
             ||||||||||||||||||||||||||||||||||||||||||||||||||||||||||||
Sbjct  481   CCATTATTTGTATGATCTGTTTTAATTACTGCAATTTTATTATTATTATCTTTACCAGTC  540

Query  557   TTAGCTGGCGCAATTACAATATTATTAACTGATCGAAACTTTAATACATCATTCTTTGAC  616
             ||||||||||||||||||||||||||||||||||||||||||||||||||||||||||||
Sbjct  541   TTAGCTGGCGCAATTACAATATTATTAACTGATCGAAACTTTAATACATCATTCTTTGAC  600

Query  617   CCTTCAGGAGGAGGTGACCCAATTCTATATCAACATTTATTT  658
             ||||||||||||||||||||||||||||||||||||||||||
Sbjct  601   CCTTCAGGAGGAGGTGACCCAATTCTATATCAACATTTATTT  642
```

```
>Rhipicephalus sanguineus isolate WZ003 cytochrome oxidase subunit I gene,
partial cds; mitochondrial
Sequence ID: MG969506.1 Length: 664
Range 1: 1 to 644

Score:1184 bits(641), Expect:0.0,
Identities:643/644(99%),  Gaps:0/644(0%), Strand: Plus/Plus

Query  15    TTTTTGGAGCATGATCCGGAATACTAGGATTAAGAATAAGAATATTAATCCGCATAGAAT  74
             || |||||||||||||||||||||||||||||||||||||||||||||||||||||||||
Sbjct  1     TTGTTGGAGCATGATCCGGAATACTAGGATTAAGAATAAGAATATTAATCCGCATAGAAT  60

Query  75    TAGGTCAACCAGGAACTTTAATTGGAAACGATCAAATTTATAATGTAATTGTAACAGCAC  134
             ||||||||||||||||||||||||||||||||||||||||||||||||||||||||||||
Sbjct  61    TAGGTCAACCAGGAACTTTAATTGGAAACGATCAAATTTATAATGTAATTGTAACAGCAC  120

Query  135   ATGCATTTATTATAAttttttttATAGTTATACCAATTATAATCGGAGGATTTGGAAATT  194
             ||||||||||||||||||||||||||||||||||||||||||||||||||||||||||||
Sbjct  121   ATGCATTTATTATAATTTTTTTTATAGTTATACCAATTATAATCGGAGGATTTGGAAATT  180

Query  195   GATTAGTTCCTATTATACTAGGAGCTCCAGATATAGCATTTCCACGAATAAATAATATAA  254
             ||||||||||||||||||||||||||||||||||||||||||||||||||||||||||||
```

```
Sbjct  181  GATTAGTTCCTATTATACTAGGAGCTCCAGATATAGCATTTCCACGAATAAATAATATAA  240

Query  255  GATTTTGGTTACTTCCTCCTTCTCTATTTCTACTAATTAATTCTTCATTAATCGAATCAG  314
            ||||||||||||||||||||||||||||||||||||||||||||||||||||||||||||
Sbjct  241  GATTTTGGTTACTTCCTCCTTCTCTATTTCTACTAATTAATTCTTCATTAATCGAATCAG  300

Query  315  GAGCAGGTACAGGGTGAACAGTTTAcccccccTTATCATCAAATTTATCACACTACGGAC  374
            ||||||||||||||||||||||||||||||||||||||||||||||||||||||||||||
Sbjct  301  GAGCAGGTACAGGGTGAACAGTTTACCCCCCCTTATCATCAAATTTATCACACTACGGAC  360

Query  375  CATCAGTAGATTTAGCTATTTTTTCTCTGCATCTTGCAGGTGCTTCTTCAATTTTAGGTG  434
            ||||||||||||||||||||||||||||||||||||||||||||||||||||||||||||
Sbjct  361  CATCAGTAGATTTAGCTATTTTTTCTCTGCATCTTGCAGGTGCTTCTTCAATTTTAGGTG  420

Query  435  CAATCAATTTCATTACAACTATTGTAAATATACGATCCATTGGAATAACAATAGAACGAA  494
            ||||||||||||||||||||||||||||||||||||||||||||||||||||||||||||
Sbjct  421  CAATCAATTTCATTACAACTATTGTAAATATACGATCCATTGGAATAACAATAGAACGAA  480

Query  495  TACCATTATTTGTATGATCTGTTTTAATTACTGCAATTTTATTATTATTATCTTTACCAG  554
            ||||||||||||||||||||||||||||||||||||||||||||||||||||||||||||
Sbjct  481  TACCATTATTTGTATGATCTGTTTTAATTACTGCAATTTTATTATTATTATCTTTACCAG  540

Query  555  TCTTAGCTGGCGCAATTACAATATTATTAACTGATCGAAACTTTAATACATCATTCTTTG  614
            ||||||||||||||||||||||||||||||||||||||||||||||||||||||||||||
Sbjct  541  TCTTAGCTGGCGCAATTACAATATTATTAACTGATCGAAACTTTAATACATCATTCTTTG  600

Query  615  ACCCTTCAGGAGGAGGTGACCCAATTCTATATCAACATTTATTT  658
            ||||||||||||||||||||||||||||||||||||||||||||
Sbjct  601  ACCCTTCAGGAGGAGGTGACCCAATTCTATATCAACATTTATTT  644
```

>Rhipicephalus sanguineus isolate WZ002 cytochrome oxidase subunit I gene, partial cds; mitochondrial
Sequence ID: MG969505.1 Length: 664
Range 1: 1 to 644

Score:1184 bits(641), Expect:0.0,
Identities:643/644(99%), Gaps:0/644(0%), Strand: Plus/Plus

```
Query  15   TTTTTGGAGCATGATCCGGAATACTAGGATTAAGAATAAGAATATTAATCCGCATAGAAT  74
            || |||||||||||||||||||||||||||||||||||||||||||||||||||||||||
Sbjct  1    TTATTGGAGCATGATCCGGAATACTAGGATTAAGAATAAGAATATTAATCCGCATAGAAT  60

Query  75   TAGGTCAACCAGGAACTTTAATTGGAAACGATCAAATTTATAATGTAATTGTAACAGCAC  134
            ||||||||||||||||||||||||||||||||||||||||||||||||||||||||||||
Sbjct  61   TAGGTCAACCAGGAACTTTAATTGGAAACGATCAAATTTATAATGTAATTGTAACAGCAC  120

Query  135  ATGCATTTATTATAAttttttttATAGTTATACCAATTATAATCGGAGGATTTGGAAATT  194
            ||||||||||||||||||||||||||||||||||||||||||||||||||||||||||||
Sbjct  121  ATGCATTTATTATAATTTTTTTTATAGTTATACCAATTATAATCGGAGGATTTGGAAATT  180

Query  195  GATTAGTTCCTATTATACTAGGAGCTCCAGATATAGCATTTCCACGAATAAATAATATAA  254
```

```
            ||||||||||||||||||||||||||||||||||||||||||||||||||||||||||||
Sbjct  181  GATTAGTTCCTATTATACTAGGAGCTCCAGATATAGCATTTCCACGAATAAATAATATAA  240

Query  255  GATTTTGGTTACTTCCTCCTTCTCTATTTCTACTAATTAATTCTTCATTAATCGAATCAG  314
            ||||||||||||||||||||||||||||||||||||||||||||||||||||||||||||
Sbjct  241  GATTTTGGTTACTTCCTCCTTCTCTATTTCTACTAATTAATTCTTCATTAATCGAATCAG  300

Query  315  GAGCAGGTACAGGGTGAACAGTTTAcccccccTTATCATCAAATTTATCACACTACGGAC  374
            ||||||||||||||||||||||||||||||||||||||||||||||||||||||||||||
Sbjct  301  GAGCAGGTACAGGGTGAACAGTTTACCCCCCCTTATCATCAAATTTATCACACTACGGAC  360

Query  375  CATCAGTAGATTTAGCTATTTTTTCTCTGCATCTTGCAGGTGCTTCTTCAATTTTAGGTG  434
            ||||||||||||||||||||||||||||||||||||||||||||||||||||||||||||
Sbjct  361  CATCAGTAGATTTAGCTATTTTTTCTCTGCATCTTGCAGGTGCTTCTTCAATTTTAGGTG  420

Query  435  CAATCAATTTCATTACAACTATTGTAAATATACGATCCATTGGAATAACAATAGAACGAA  494
            ||||||||||||||||||||||||||||||||||||||||||||||||||||||||||||
Sbjct  421  CAATCAATTTCATTACAACTATTGTAAATATACGATCCATTGGAATAACAATAGAACGAA  480

Query  495  TACCATTATTTGTATGATCTGTTTTAATTACTGCAATTTTATTATTATTATCTTTACCAG  554
            ||||||||||||||||||||||||||||||||||||||||||||||||||||||||||||
Sbjct  481  TACCATTATTTGTATGATCTGTTTTAATTACTGCAATTTTATTATTATTATCTTTACCAG  540

Query  555  TCTTAGCTGGCGCAATTACAATATTATTAACTGATCGAAACTTTAATACATCATTCTTTG  614
            ||||||||||||||||||||||||||||||||||||||||||||||||||||||||||||
Sbjct  541  TCTTAGCTGGCGCAATTACAATATTATTAACTGATCGAAACTTTAATACATCATTCTTTG  600

Query  615  ACCCTTCAGGAGGAGGTGACCCAATTCTATATCAACATTTATTT  658
            ||||||||||||||||||||||||||||||||||||||||||||
Sbjct  601  ACCCTTCAGGAGGAGGTGACCCAATTCTATATCAACATTTATTT  644
```

>Rhipicephalus sanguineus isolate WZ001 cytochrome oxidase subunit I gene, partial cds; mitochondrial
Sequence ID: MG969504.1 Length: 664
Range 1: 1 to 644

Score:1184 bits(641), Expect:0.0,
Identities:643/644(99%), Gaps:0/644(0%), Strand: Plus/Plus

```
Query  15   TTTTTGGAGCATGATCCGGAATACTAGGATTAAGAATAAGAATATTAATCCGCATAGAAT  74
            || |||||||||||||||||||||||||||||||||||||||||||||||||||||||||
Sbjct  1    TTGTTGGAGCATGATCCGGAATACTAGGATTAAGAATAAGAATATTAATCCGCATAGAAT  60

Query  75   TAGGTCAACCAGGAACTTTAATTGGAAACGATCAAATTTATAATGTAATTGTAACAGCAC  134
            ||||||||||||||||||||||||||||||||||||||||||||||||||||||||||||
Sbjct  61   TAGGTCAACCAGGAACTTTAATTGGAAACGATCAAATTTATAATGTAATTGTAACAGCAC  120

Query  135  ATGCATTTATTATAAttttttttATAGTTATACCAATTATAATCGGAGGATTTGGAAATT  194
            ||||||||||||||||||||||||||||||||||||||||||||||||||||||||||||
Sbjct  121  ATGCATTTATTATAATTTTTTTTATAGTTATACCAATTATAATCGGAGGATTTGGAAATT  180
```

```
Query  195  GATTAGTTCCTATTATACTAGGAGCTCCAGATATAGCATTTCCACGAATAAATAATATAA  254
            ||||||||||||||||||||||||||||||||||||||||||||||||||||||||||||
Sbjct  181  GATTAGTTCCTATTATACTAGGAGCTCCAGATATAGCATTTCCACGAATAAATAATATAA  240

Query  255  GATTTTGGTTACTTCCTCCTTCTCTATTTCTACTAATTAATTCTTCATTAATCGAATCAG  314
            ||||||||||||||||||||||||||||||||||||||||||||||||||||||||||||
Sbjct  241  GATTTTGGTTACTTCCTCCTTCTCTATTTCTACTAATTAATTCTTCATTAATCGAATCAG  300

Query  315  GAGCAGGTACAGGGTGAACAGTTTAcccccccTTATCATCAAATTTATCACACTACGGAC  374
            ||||||||||||||||||||||||||||||||||||||||||||||||||||||||||||
Sbjct  301  GAGCAGGTACAGGGTGAACAGTTTACCCCCCCTTATCATCAAATTTATCACACTACGGAC  360

Query  375  CATCAGTAGATTTAGCTATTTTTTCTCTGCATCTTGCAGGTGCTTCTTCAATTTTAGGTG  434
            ||||||||||||||||||||||||||||||||||||||||||||||||||||||||||||
Sbjct  361  CATCAGTAGATTTAGCTATTTTTTCTCTGCATCTTGCAGGTGCTTCTTCAATTTTAGGTG  420

Query  435  CAATCAATTTCATTACAACTATTGTAAATATACGATCCATTGGAATAACAATAGAACGAA  494
            ||||||||||||||||||||||||||||||||||||||||||||||||||||||||||||
Sbjct  421  CAATCAATTTCATTACAACTATTGTAAATATACGATCCATTGGAATAACAATAGAACGAA  480

Query  495  TACCATTATTTGTATGATCTGTTTTAATTACTGCAATTTTATTATTATTATCTTTACCAG  554
            ||||||||||||||||||||||||||||||||||||||||||||||||||||||||||||
Sbjct  481  TACCATTATTTGTATGATCTGTTTTAATTACTGCAATTTTATTATTATTATCTTTACCAG  540

Query  555  TCTTAGCTGGCGCAATTACAATATTATTAACTGATCGAAACTTTAATACATCATTCTTTG  614
            ||||||||||||||||||||||||||||||||||||||||||||||||||||||||||||
Sbjct  541  TCTTAGCTGGCGCAATTACAATATTATTAACTGATCGAAACTTTAATACATCATTCTTTG  600

Query  615  ACCCTTCAGGAGGAGGTGACCCAATTCTATATCAACATTTATTT  658
            ||||||||||||||||||||||||||||||||||||||||||||
Sbjct  601  ACCCTTCAGGAGGAGGTGACCCAATTCTATATCAACATTTATTT  644
```

>Rhipicephalus sanguineus voucher CAS-T066 cytochrome oxidase subunit 1 (COI) gene, partial cds; mitochondrial
Sequence ID: KF200096.1 Length: 644
Range 1: 1 to 644

Score:1179 bits(638), Expect:0.0,
Identities:642/644(99%), Gaps:0/644(0%), Strand: Plus/Plus

```
Query  6    TATACTTAATTTTTGGAGCATGATCCGGAATACTAGGATTAAGAATAAGAATATTAATCC  65
            |||||||||||||||||||||||||||||||| |||||||||||||||||||||||||||
Sbjct  1    TATACTTAATTTTTGGAGCATGATCCGGAATATTAGGATTAAGAATAAGAATATTAATCC  60

Query  66   GCATAGAATTAGGTCAACCAGGAACTTTAATTGGAAACGATCAAATTTATAATGTAATTG  125
            ||||||||||||||||||||||||||||||||||||||||||||||||||||||||||||
Sbjct  61   GCATAGAATTAGGTCAACCAGGAACTTTAATTGGAAACGATCAAATTTATAATGTAATTG  120

Query  126  TAACAGCACATGCATTTATTATAAttttttttATAGTTATACCAATTATAATCGGAGGAT  185
            ||||||||||||||||||||||||||||||||||||||||||||||||||||||||||||
Sbjct  121  TAACAGCACATGCATTTATTATAATTTTTTTTATAGTTATACCAATTATAATCGGAGGAT  180
```

```
Query  186   TTGGAAATTGATTAGTTCCTATTATACTAGGAGCTCCAGATATAGCATTTCCACGAATAA  245
             ||||||||||||||||||||||||||||||| ||||||||||||||||||||||||||||
Sbjct  181   TTGGAAATTGATTAGTTCCTATTATACTAGGGGCTCCAGATATAGCATTTCCACGAATAA  240

Query  246   ATAATATAAGATTTTGGTTACTTCCTCCTTCTCTATTTCTACTAATTAATTCTTCATTAA  305
             ||||||||||||||||||||||||||||||||||||||||||||||||||||||||||||
Sbjct  241   ATAATATAAGATTTTGGTTACTTCCTCCTTCTCTATTTCTACTAATTAATTCTTCATTAA  300

Query  306   TCGAATCAGGAGCAGGTACAGGGTGAACAGTTTAcccccccTTATCATCAAATTTATCAC  365
             ||||||||||||||||||||||||||||||||||||||||||||||||||||||||||||
Sbjct  301   TCGAATCAGGAGCAGGTACAGGGTGAACAGTTTACCCCCCCTTATCATCAAATTTATCAC  360

Query  366   ACTACGGACCATCAGTAGATTTAGCTATTTTTTCTCTGCATCTTGCAGGTGCTTCTTCAA  425
             ||||||||||||||||||||||||||||||||||||||||||||||||||||||||||||
Sbjct  361   ACTACGGACCATCAGTAGATTTAGCTATTTTTTCTCTGCATCTTGCAGGTGCTTCTTCAA  420

Query  426   TTTTAGGTGCAATCAATTTCATTACAACTATTGTAAATATACGATCCATTGGAATAACAA  485
             ||||||||||||||||||||||||||||||||||||||||||||||||||||||||||||
Sbjct  421   TTTTAGGTGCAATCAATTTCATTACAACTATTGTAAATATACGATCCATTGGAATAACAA  480

Query  486   TAGAACGAATACCATTATTTGTATGATCTGTTTTAATTACTGCAATTTTATTATTATTAT  545
             ||||||||||||||||||||||||||||||||||||||||||||||||||||||||||||
Sbjct  481   TAGAACGAATACCATTATTTGTATGATCTGTTTTAATTACTGCAATTTTATTATTATTAT  540

Query  546   CTTTACCAGTCTTAGCTGGCGCAATTACAATATTATTAACTGATCGAAACTTTAATACAT  605
             ||||||||||||||||||||||||||||||||||||||||||||||||||||||||||||
Sbjct  541   CTTTACCAGTCTTAGCTGGCGCAATTACAATATTATTAACTGATCGAAACTTTAATACAT  600

Query  606   CATTCTTTGACCCTTCAGGAGGAGGTGACCCAATTCTATATCAA  649
             ||||||||||||||||||||||||||||||||||||||||||||
Sbjct  601   CATTCTTTGACCCTTCAGGAGGAGGTGACCCAATTCTATATCAA  644
```

>Rhipicephalus linnaei voucher P1/22_18-4 cytochrome c oxidase subunit I (COX1) gene, partial cds; mitochondrial
Sequence ID: OM984983.1 Length: 658
Range 1: 1 to 658

Score:1177 bits(637), Expect:0.0,
Identities:651/658(99%), Gaps:0/658(0%), Strand: Plus/Plus

```
Query  1     GACAATATACTTAATTTTTGGAGCATGATCCGGAATACTAGGATTAAGAATAAGAATATT  60
             ||||||||||||||||||||||||||||||||||||| ||||||||||||||||||||||
Sbjct  1     GACAATATACTTAATTTTTGGAGCATGATCCGGAATATTAGGATTAAGAATAAGAATATT  60

Query  61    AATCCGCATAGAATTAGGTCAACCAGGAACTTTAATTGGAAACGATCAAATTTATAATGT  120
             ||||||||||||||||||||||||||||||||||||||||||||||||||||||||||||
Sbjct  61    AATCCGCATAGAATTAGGTCAACCAGGAACTTTAATTGGAAACGATCAAATTTATAATGT  120

Query  121   AATTGTAACAGCACATGCATTTATTATAAtttttttATAGTTATACCAATTATAATCGG  180
             ||||||||||||||||||||||||||||||||||||||||||||||||||||||||||||
```

```
Sbjct  121  AATTGTAACAGCACATGCATTTATTATAATTTTTTTTATAGTTATACCAATTATAATCGG  180

Query  181  AGGATTTGGAAATTGATTAGTTCCTATTATACTAGGAGCTCCAGATATAGCATTTCCACG  240
            ||||||||||||||||||||||||||||||||||||||||||||||||||||||||||||
Sbjct  181  AGGATTTGGAAATTGATTAGTTCCTATTATACTAGGAGCTCCAGATATAGCATTTCCACG  240

Query  241  AATAAATAATATAAGATTTTGGTTACTTCCTCCTTCTCTATTTCTACTAATTAATTCTTC  300
            ||||||||||||||||||||||||||||||||||||||||||||||||||||||||||||
Sbjct  241  AATAAATAATATAAGATTTTGGTTACTTCCTCCTTCTCTATTTCTACTAATTAATTCTTC  300

Query  301  ATTAATCGAATCAGGAGCAGGTACAGGGTGAACAGTTTAcccccccTTATCATCAAATTT  360
            ||||||||||||||||||||||||||||||||||||||||||||||||||||||||||||
Sbjct  301  ATTAATCGAATCAGGAGCAGGTACAGGGTGAACAGTTTACCCCCCCTTATCATCAAATTT  360

Query  361  ATCACACTACGGACCATCAGTAGATTTAGCTATTTTTTCTCTGCATCTTGCAGGTGCTTC  420
            |||||||||||| |||||||||||||||||||| ||||||||||||||||| ||||||||
Sbjct  361  ATCACACTACGGGCCATCAGTAGATTTAGCTATCTTTTCTCTGCATCTTGCTGGTGCTTC  420

Query  421  TTCAATTTTAGGTGCAATCAATTTCATTACAACTATTGTAAATATACGATCCATTGGAAT  480
            |||||||||||||||||| |||||||||||||||||||||||||||||||||||||||||
Sbjct  421  TTCAATTTTAGGTGCAATTAATTTCATTACAACTATTGTAAATATACGATCCATTGGAAT  480

Query  481  AACAATAGAACGAATACCATTATTTGTATGATCTGTTTTAATTACTGCAATTTTATTATT  540
            ||||||||||||||||||||||||||||||||||||||||||||||||||||||||||||
Sbjct  481  AACAATAGAACGAATACCATTATTTGTATGATCTGTTTTAATTACTGCAATTTTATTATT  540

Query  541  ATTATCTTTACCAGTCTTAGCTGGCGCAATTACAATATTATTAACTGATCGAAACTTTAA  600
            |||||||||||||||||||||||| |||||||||||||||||||||||||||||||||||
Sbjct  541  ATTATCTTTACCAGTCTTAGCTGGTGCAATTACAATATTATTAACTGATCGAAACTTTAA  600

Query  601  TACATCATTCTTTGACCCTTCAGGAGGAGGTGACCCAATTCTATATCAACATTTATTT  658
            |||||||||||||||||||||||||||||||||||||||||| |||||||||||||||
Sbjct  601  TACATCATTCTTTGACCCTTCAGGAGGAGGTGACCCAATTCTGTATCAACATTTATTT  658
```

>Rhipicephalus sanguineus isolate GHM1 cytochrome oxidase subunit I (cox1) gene, partial cds; mitochondrial
Sequence ID: JX416302.1 Length: 802
>Rhipicephalus sanguineus isolate GPF1 cytochrome oxidase subunit I (cox1) gene, partial cds; mitochondrial
Sequence ID: JX416305.1 Length: 802
>Rhipicephalus sanguineus isolate GPF2 cytochrome oxidase subunit I (cox1) gene, partial cds; mitochondrial
Sequence ID: JX416306.1 Length: 802
>Rhipicephalus sanguineus isolate SZM1 cytochrome oxidase subunit I (cox1) gene, partial cds; mitochondrial
Sequence ID: JX416307.1 Length: 802
Range 1: 1 to 637

Score:1177 bits(637), Expect:0.0,
Identities:637/637(100%), Gaps:0/637(0%), Strand: Plus/Plus

```
Query  22   AGCATGATCCGGAATACTAGGATTAAGAATAAGAATATTAATCCGCATAGAATTAGGTCA  81
            ||||||||||||||||||||||||||||||||||||||||||||||||||||||||||||
Sbjct  1    AGCATGATCCGGAATACTAGGATTAAGAATAAGAATATTAATCCGCATAGAATTAGGTCA  60

Query  82   ACCAGGAACTTTAATTGGAAACGATCAAATTTATAATGTAATTGTAACAGCACATGCATT  141
            ||||||||||||||||||||||||||||||||||||||||||||||||||||||||||||
Sbjct  61   ACCAGGAACTTTAATTGGAAACGATCAAATTTATAATGTAATTGTAACAGCACATGCATT  120

Query  142  TATTATAAtttttttATAGTTATACCAATTATAATCGGAGGATTTGGAAATTGATTAGT  201
            ||||||||||||||||||||||||||||||||||||||||||||||||||||||||||||
Sbjct  121  TATTATAATTTTTTTTATAGTTATACCAATTATAATCGGAGGATTTGGAAATTGATTAGT  180

Query  202  TCCTATTATACTAGGAGCTCCAGATATAGCATTTCCACGAATAAATAATATAAGATTTTG  261
            ||||||||||||||||||||||||||||||||||||||||||||||||||||||||||||
Sbjct  181  TCCTATTATACTAGGAGCTCCAGATATAGCATTTCCACGAATAAATAATATAAGATTTTG  240

Query  262  GTTACTTCCTCCTTCTCTATTTCTACTAATTAATTCTTCATTAATCGAATCAGGAGCAGG  321
            ||||||||||||||||||||||||||||||||||||||||||||||||||||||||||||
Sbjct  241  GTTACTTCCTCCTTCTCTATTTCTACTAATTAATTCTTCATTAATCGAATCAGGAGCAGG  300

Query  322  TACAGGGTGAACAGTTTAccccccTTATCATCAAATTTATCACACTACGGACCATCAGT  381
            ||||||||||||||||||||||||||||||||||||||||||||||||||||||||||||
Sbjct  301  TACAGGGTGAACAGTTTACCCCCCCTTATCATCAAATTTATCACACTACGGACCATCAGT  360

Query  382  AGATTTAGCTATTTTTTCTCTGCATCTTGCAGGTGCTTCTTCAATTTTAGGTGCAATCAA  441
            ||||||||||||||||||||||||||||||||||||||||||||||||||||||||||||
Sbjct  361  AGATTTAGCTATTTTTTCTCTGCATCTTGCAGGTGCTTCTTCAATTTTAGGTGCAATCAA  420

Query  442  TTTCATTACAACTATTGTAAATATACGATCCATTGGAATAACAATAGAACGAATACCATT  501
            ||||||||||||||||||||||||||||||||||||||||||||||||||||||||||||
Sbjct  421  TTTCATTACAACTATTGTAAATATACGATCCATTGGAATAACAATAGAACGAATACCATT  480

Query  502  ATTTGTATGATCTGTTTTAATTACTGCAATTTTATTATTATTATCTTTACCAGTCTTAGC  561
            ||||||||||||||||||||||||||||||||||||||||||||||||||||||||||||
Sbjct  481  ATTTGTATGATCTGTTTTAATTACTGCAATTTTATTATTATTATCTTTACCAGTCTTAGC  540

Query  562  TGGCGCAATTACAATATTATTAACTGATCGAAACTTTAATACATCATTCTTTGACCCTTC  621
            ||||||||||||||||||||||||||||||||||||||||||||||||||||||||||||
Sbjct  541  TGGCGCAATTACAATATTATTAACTGATCGAAACTTTAATACATCATTCTTTGACCCTTC  600

Query  622  AGGAGGAGGTGACCCAATTCTATATCAACATTTATTT  658
            |||||||||||||||||||||||||||||||||||||
Sbjct  601  AGGAGGAGGTGACCCAATTCTATATCAACATTTATTT  637
```

```
>Rhipicephalus sanguineus isolate CHM1 cytochrome oxidase subunit I (cox1) gene,
partial cds; mitochondrial
Sequence ID: JX416298.1 Length: 802
Range 1: 1 to 637

Score:1177 bits(637), Expect:0.0,
Identities:637/637(100%),  Gaps:0/637(0%), Strand: Plus/Plus
```

```
Query  22   AGCATGATCCGGAATACTAGGATTAAGAATAAGAATATTAATCCGCATAGAATTAGGTCA  81
            ||||||||||||||||||||||||||||||||||||||||||||||||||||||||||||
Sbjct  1    AGCATGATCCGGAATACTAGGATTAAGAATAAGAATATTAATCCGCATAGAATTAGGTCA  60

Query  82   ACCAGGAACTTTAATTGGAAACGATCAAATTTATAATGTAATTGTAACAGCACATGCATT  141
            ||||||||||||||||||||||||||||||||||||||||||||||||||||||||||||
Sbjct  61   ACCAGGAACTTTAATTGGAAACGATCAAATTTATAATGTAATTGTAACAGCACATGCATT  120

Query  142  TATTATAAtttttttATAGTTATACCAATTATAATCGGAGGATTTGGAAATTGATTAGT  201
            ||||||||||||||||||||||||||||||||||||||||||||||||||||||||||||
Sbjct  121  TATTATAATTTTTTTATAGTTATACCAATTATAATCGGAGGATTTGGAAATTGATTAGT  180

Query  202  TCCTATTATACTAGGAGCTCCAGATATAGCATTTCCACGAATAAATAATATAAGATTTTG  261
            ||||||||||||||||||||||||||||||||||||||||||||||||||||||||||||
Sbjct  181  TCCTATTATACTAGGAGCTCCAGATATAGCATTTCCACGAATAAATAATATAAGATTTTG  240

Query  262  GTTACTTCCTCCTTCTCTATTTCTACTAATTAATTCTTCATTAATCGAATCAGGAGCAGG  321
            ||||||||||||||||||||||||||||||||||||||||||||||||||||||||||||
Sbjct  241  GTTACTTCCTCCTTCTCTATTTCTACTAATTAATTCTTCATTAATCGAATCAGGAGCAGG  300

Query  322  TACAGGGTGAACAGTTTAcccccccTTATCATCAAATTTATCACACTACGGACCATCAGT  381
            ||||||||||||||||||||||||||||||||||||||||||||||||||||||||||||
Sbjct  301  TACAGGGTGAACAGTTTACCCCCCCTTATCATCAAATTTATCACACTACGGACCATCAGT  360

Query  382  AGATTTAGCTATTTTTTCTCTGCATCTTGCAGGTGCTTCTTCAATTTTAGGTGCAATCAA  441
            ||||||||||||||||||||||||||||||||||||||||||||||||||||||||||||
Sbjct  361  AGATTTAGCTATTTTTTCTCTGCATCTTGCAGGTGCTTCTTCAATTTTAGGTGCAATCAA  420

Query  442  TTTCATTACAACTATTGTAAATATACGATCCATTGGAATAACAATAGAACGAATACCATT  501
            ||||||||||||||||||||||||||||||||||||||||||||||||||||||||||||
Sbjct  421  TTTCATTACAACTATTGTAAATATACGATCCATTGGAATAACAATAGAACGAATACCATT  480

Query  502  ATTTGTATGATCTGTTTTAATTACTGCAATTTTATTATTATTATCTTTACCAGTCTTAGC  561
            ||||||||||||||||||||||||||||||||||||||||||||||||||||||||||||
Sbjct  481  ATTTGTATGATCTGTTTTAATTACTGCAATTTTATTATTATTATCTTTACCAGTCTTAGC  540

Query  562  TGGCGCAATTACAATATTATTAACTGATCGAAACTTTAATACATCATTCTTTGACCCTTC  621
            ||||||||||||||||||||||||||||||||||||||||||||||||||||||||||||
Sbjct  541  TGGCGCAATTACAATATTATTAACTGATCGAAACTTTAATACATCATTCTTTGACCCTTC  600

Query  622  AGGAGGAGGTGACCCAATTCTATATCAACATTTATTT  658
            |||||||||||||||||||||||||||||||||||||
Sbjct  601  AGGAGGAGGTGACCCAATTCTATATCAACATTTATTT  637
```

>Rhipicephalus sanguineus voucher AMMS-RS-5 cytochrome c oxidase subunit I-like (COI) gene, partial sequence; mitochondrial
Sequence ID: HM193873.1 Length: 670
Range 1: 1 to 642

Score:1177 bits(637), Expect:0.0,

```
Identities:642/644(99%),  Gaps:2/644(0%), Strand: Plus/Plus

Query  15    TTTTTGGAGCATGATCCGGAATACTAGGATTAAGAATAAGAATATTAATCCGCATAGAAT  74
             |||||||| |||||||||| ||||||||||||||||||||||||||||||||||||||||
Sbjct  1     TTTTTGGA-CATGATCCGG-ATACTAGGATTAAGAATAAGAATATTAATCCGCATAGAAT  58

Query  75    TAGGTCAACCAGGAACTTTAATTGGAAACGATCAAATTTATAATGTAATTGTAACAGCAC  134
             ||||||||||||||||||||||||||||||||||||||||||||||||||||||||||||
Sbjct  59    TAGGTCAACCAGGAACTTTAATTGGAAACGATCAAATTTATAATGTAATTGTAACAGCAC  118

Query  135   ATGCATTTATTATAAttttttttATAGTTATACCAATTATAATCGGAGGATTTGGAAATT  194
             ||||||||||||||||||||||||||||||||||||||||||||||||||||||||||||
Sbjct  119   ATGCATTTATTATAATTTTTTTTATAGTTATACCAATTATAATCGGAGGATTTGGAAATT  178

Query  195   GATTAGTTCCTATTATACTAGGAGCTCCAGATATAGCATTTCCACGAATAAATAATATAA  254
             ||||||||||||||||||||||||||||||||||||||||||||||||||||||||||||
Sbjct  179   GATTAGTTCCTATTATACTAGGAGCTCCAGATATAGCATTTCCACGAATAAATAATATAA  238

Query  255   GATTTTGGTTACTTCCTCCTTCTCTATTTCTACTAATTAATTCTTCATTAATCGAATCAG  314
             ||||||||||||||||||||||||||||||||||||||||||||||||||||||||||||
Sbjct  239   GATTTTGGTTACTTCCTCCTTCTCTATTTCTACTAATTAATTCTTCATTAATCGAATCAG  298

Query  315   GAGCAGGTACAGGGTGAACAGTTTAcccccccTTATCATCAAATTTATCACACTACGGAC  374
             ||||||||||||||||||||||||||||||||||||||||||||||||||||||||||||
Sbjct  299   GAGCAGGTACAGGGTGAACAGTTTACCCCCCCTTATCATCAAATTTATCACACTACGGAC  358

Query  375   CATCAGTAGATTTAGCTATTTTTTCTCTGCATCTTGCAGGTGCTTCTTCAATTTTAGGTG  434
             ||||||||||||||||||||||||||||||||||||||||||||||||||||||||||||
Sbjct  359   CATCAGTAGATTTAGCTATTTTTTCTCTGCATCTTGCAGGTGCTTCTTCAATTTTAGGTG  418

Query  435   CAATCAATTTCATTACAACTATTGTAAATATACGATCCATTGGAATAACAATAGAACGAA  494
             ||||||||||||||||||||||||||||||||||||||||||||||||||||||||||||
Sbjct  419   CAATCAATTTCATTACAACTATTGTAAATATACGATCCATTGGAATAACAATAGAACGAA  478

Query  495   TACCATTATTTGTATGATCTGTTTTAATTACTGCAATTTTATTATTATTATCTTTACCAG  554
             ||||||||||||||||||||||||||||||||||||||||||||||||||||||||||||
Sbjct  479   TACCATTATTTGTATGATCTGTTTTAATTACTGCAATTTTATTATTATTATCTTTACCAG  538

Query  555   TCTTAGCTGGCGCAATTACAATATTATTAACTGATCGAAACTTTAATACATCATTCTTTG  614
             ||||||||||||||||||||||||||||||||||||||||||||||||||||||||||||
Sbjct  539   TCTTAGCTGGCGCAATTACAATATTATTAACTGATCGAAACTTTAATACATCATTCTTTG  598

Query  615   ACCCTTCAGGAGGAGGTGACCCAATTCTATATCAACATTTATTT  658
             ||||||||||||||||||||||||||||||||||||||||||||
Sbjct  599   ACCCTTCAGGAGGAGGTGACCCAATTCTATATCAACATTTATTT  642
```

>Rhipicephalus sanguineus isolate LIC6767D cytochrome oxidase subunit 1 (COI) gene, partial cds; mitochondrial
Sequence ID: KX383820.1 Length: 768
Range 1: 1 to 642

```
Score:1175 bits(636), Expect:0.0,
Identities:640/642(99%),  Gaps:0/642(0%), Strand: Plus/Plus

Query  17   TTTGGAGCATGATCCGGAATACTAGGATTAAGAATAAGAATATTAATCCGCATAGAATTA  76
            ||||||||||||||||||||||||||||||||||||||||||||||||||||||||||||
Sbjct  1    TTTGGAGCATGATCCGGAATACTAGGATTAAGAATAAGAATATTAATCCGCATAGAATTA  60

Query  77   GGTCAACCAGGAACTTTAATTGGAAACGATCAAATTTATAATGTAATTGTAACAGCACAT  136
            ||||||||||||||||||||||||||||||||||||||||||||||||||||||||||||
Sbjct  61   GGTCAACCAGGAACTTTAATTGGAAACGATCAAATTTATAATGTAATTGTAACAGCACAT  120

Query  137  GCATTTATTATAAttttttttATAGTTATACCAATTATAATCGGAGGATTTGGAAATTGA  196
            ||||||||||||||||||||||||||||||||||||||||||||||||||||||||||||
Sbjct  121  GCATTTATTATAATTTTTTTTATAGTTATACCAATTATAATCGGAGGATTTGGAAATTGA  180

Query  197  TTAGTTCCTATTATACTAGGAGCTCCAGATATAGCATTTCCACGAATAAATAATATAAGA  256
            ||||||||||||||||||||||||||||||||||||||||||||||||||||||||||||
Sbjct  181  TTAGTTCCTATTATACTAGGAGCTCCAGATATAGCATTTCCACGAATAAATAATATAAGA  240

Query  257  TTTTGGTTACTTCCTCCTTCTCTATTTCTACTAATTAATTCTTCATTAATCGAATCAGGA  316
            ||||||||||||||||||||||||||||||||||||||||||||||||||||||||||||
Sbjct  241  TTTTGGTTACTTCCTCCTTCTCTATTTCTACTAATTAATTCTTCATTAATCGAATCAGGA  300

Query  317  GCAGGTACAGGGTGAACAGTTTAcccccccTTATCATCAAATTTATCACACTACGGACCA  376
            ||||||||||||||||||||||||||||||||||||||||||||||||||||||||||||
Sbjct  301  GCAGGTACAGGGTGAACAGTTTACCCCCCCTTATCATCAAATTTATCACACTACGGACCA  360

Query  377  TCAGTAGATTTAGCTATTTTTTCTCTGCATCTTGCAGGTGCTTCTTCAATTTTAGGTGCA  436
            |||||||||||||||||||||||||| |||||||||||||||||||||||||||||||||
Sbjct  361  TCAGTAGATTTAGCTATTTTTTCTCTACATCTTGCAGGTGCTTCTTCAATTTTAGGTGCA  420

Query  437  ATCAATTTCATTACAACTATTGTAAATATACGATCCATTGGAATAACAATAGAACGAATA  496
            ||||||||||||||||||||||||||||||||||||||||||||||||||||||||||||
Sbjct  421  ATCAATTTCATTACAACTATTGTAAATATACGATCCATTGGAATAACAATAGAACGAATA  480

Query  497  CCATTATTTGTATGATCTGTTTTAATTACTGCAATTTTATTATTATTATCTTTACCAGTC  556
            ||||||||||||||||||||||||||||||||||||||||||||||||||||||||||||
Sbjct  481  CCATTATTTGTATGATCTGTTTTAATTACTGCAATTTTATTATTATTATCTTTACCAGTC  540

Query  557  TTAGCTGGCGCAATTACAATATTATTAACTGATCGAAACTTTAATACATCATTCTTTGAC  616
            ||||||||||||||||||||||||||||||||||||||||||||||||||||||||||||
Sbjct  541  TTAGCTGGCGCAATTACAATATTATTAACTGATCGAAACTTTAATACATCATTCTTTGAC  600

Query  617  CCTTCAGGAGGAGGTGACCCAATTCTATATCAACATTTATTT  658
            ||||||||||| ||||||||||||||||||||||||||||||
Sbjct  601  CCTTCAGGAGGGGGTGACCCAATTCTATATCAACATTTATTT  642
```

```
>Rhipicephalus sanguineus isolate LIC5766G cytochrome oxidase subunit 1 (COI)
gene, partial cds; mitochondrial
Sequence ID: KX383816.1 Length: 724
Range 1: 1 to 642
```

```
Score:1171 bits(634), Expect:0.0,
Identities:639/642(99%),  Gaps:0/642(0%), Strand: Plus/Plus

Query  17   TTTGGAGCATGATCCGGAATACTAGGATTAAGAATAAGAATATTAATCCGCATAGAATTA  76
            ||||||||||||||||||||||||||||||||||||||||||||||||||||||||||||
Sbjct  1    TTTGGAGCATGATCCGGAATACTAGGATTAAGAATAAGAATATTAATCCGCATAGAATTA  60

Query  77   GGTCAACCAGGAACTTTAATTGGAAACGATCAAATTTATAATGTAATTGTAACAGCACAT  136
            |||||||||||||||||||||||||||||| |||||||||||||||||||||||||||||
Sbjct  61   GGTCAACCAGGAACTTTAATTGGAAACGATSAAATTTATAATGTAATTGTAACAGCACAT  120

Query  137  GCATTTATTATAAttttttttATAGTTATACCAATTATAATCGGAGGATTTGGAAATTGA  196
            ||||||||||||||||||||||||||||||||||||||||||||||||||||||||||||
Sbjct  121  GCATTTATTATAATTTTTTTTATAGTTATACCAATTATAATCGGAGGATTTGGAAATTGA  180

Query  197  TTAGTTCCTATTATACTAGGAGCTCCAGATATAGCATTTCCACGAATAAATAATATAAGA  256
            ||||||||||||||||||||||||||||||||||||||||||||||||||||||||||||
Sbjct  181  TTAGTTCCTATTATACTAGGAGCTCCAGATATAGCATTTCCACGAATAAATAATATAAGA  240

Query  257  TTTTGGTTACTTCCTCCTTCTCTATTTCTACTAATTAATTCTTCATTAATCGAATCAGGA  316
            ||||||||||||||||||||||||||||||||||||||||||||||||||||||||||||
Sbjct  241  TTTTGGTTACTTCCTCCTTCTCTATTTCTACTAATTAATTCTTCATTAATCGAATCAGGA  300

Query  317  GCAGGTACAGGGTGAACAGTTTAcccccccTTATCATCAAATTTATCACACTACGGACCA  376
            ||||||||||||||||||||||||||||||||||||||||||||||||||||||||||||
Sbjct  301  GCAGGTACAGGGTGAACAGTTTACCCCCCCTTATCATCAAATTTATCACACTACGGACCA  360

Query  377  TCAGTAGATTTAGCTATTTTTTCTCTGCATCTTGCAGGTGCTTCTTCAATTTTAGGTGCA  436
            |||||||||||||||||||||||||| |||||||||||||||||||||||||||||||||
Sbjct  361  TCAGTAGATTTAGCTATTTTTTCTCTACATCTTGCAGGTGCTTCTTCAATTTTAGGTGCA  420

Query  437  ATCAATTTCATTACAACTATTGTAAATATACGATCCATTGGAATAACAATAGAACGAATA  496
            ||||||||||||||||||||||||||||||||||||||||||||||||||||||||||||
Sbjct  421  ATCAATTTCATTACAACTATTGTAAATATACGATCCATTGGAATAACAATAGAACGAATA  480

Query  497  CCATTATTTGTATGATCTGTTTTAATTACTGCAATTTTATTATTATTATCTTTACCAGTC  556
            ||||||||||||||||||||||||||||||||||||||||||||||||||||||||||||
Sbjct  481  CCATTATTTGTATGATCTGTTTTAATTACTGCAATTTTATTATTATTATCTTTACCAGTC  540

Query  557  TTAGCTGGCGCAATTACAATATTATTAACTGATCGAAACTTTAATACATCATTCTTTGAC  616
            ||||||||||||||||||||||||||||||||||||||||||||||||||||||||||||
Sbjct  541  TTAGCTGGCGCAATTACAATATTATTAACTGATCGAAACTTTAATACATCATTCTTTGAC  600

Query  617  CCTTCAGGAGGAGGTGACCCAATTCTATATCAACATTTATTT  658
            ||||||||||| ||||||||||||||||||||||||||||||
Sbjct  601  CCTTCAGGAGGGGGTGACCCAATTCTATATCAACATTTATTT  642
```

>Rhipicephalus linnaei voucher P1/22_18-5 cytochrome c oxidase subunit I (COX1) gene, partial cds; mitochondrial
Sequence ID: OM984984.1 Length: 658

Range 1: 1 to 658

Score:1171 bits(634), Expect:0.0,
Identities:650/658(99%), Gaps:0/658(0%), Strand: Plus/Plus

```
Query  1    GACAATATACTTAATTTTTGGAGCATGATCCGGAATACTAGGATTAAGAATAAGAATATT  60
            ||||||||||||||||||||||||||||||||||||| ||||||||||||||||||||||
Sbjct  1    GACAATATACTTAATTTTTGGAGCATGATCCGGAATATTAGGATTAAGAATAAGAATATT  60

Query  61   AATCCGCATAGAATTAGGTCAACCAGGAACTTTAATTGGAAACGATCAAATTTATAATGT  120
            ||||||||||||||||||||||||||||||||||||||||||||||||||||||||||||
Sbjct  61   AATCCGCATAGAATTAGGTCAACCAGGAACTTTAATTGGAAACGATCAAATTTATAATGT  120

Query  121  AATTGTAACAGCACATGCATTTATTATAAttttttttATAGTTATACCAATTATAATCGG  180
            ||||||||||||||||||||||||||||||||||||||||||||||||||||||||||||
Sbjct  121  AATTGTAACAGCACATGCATTTATTATAATTTTTTTTATAGTTATACCAATTATAATCGG  180

Query  181  AGGATTTGGAAATTGATTAGTTCCTATTATACTAGGAGCTCCAGATATAGCATTTCCACG  240
            ||||||||||||||||||||||||||||||||||||||||||||||||||||||||||||
Sbjct  181  AGGATTTGGAAATTGATTAGTTCCTATTATACTAGGAGCTCCAGATATAGCATTTCCACG  240

Query  241  AATAAATAATATAAGATTTTGGTTACTTCCTCCTTCTCTATTTCTACTAATTAATTCTTC  300
            ||||||||||||||||||||||||||||||||||||||||||||||||||||||||||||
Sbjct  241  AATAAATAATATAAGATTTTGGTTACTTCCTCCTTCTCTATTTCTACTAATTAATTCTTC  300

Query  301  ATTAATCGAATCAGGAGCAGGTACAGGGTGAACAGTTTAcccccccTTATCATCAAATTT  360
            ||||||||||||||||||||||||||||||||||||||||||||||||||||||||||||
Sbjct  301  ATTAATCGAATCAGGAGCAGGTACAGGGTGAACAGTTTACCCCCCCTTATCATCAAATTT  360

Query  361  ATCACACTACGGACCATCAGTAGATTTAGCTATTTTTTCTCTGCATCTTGCAGGTGCTTC  420
            |||||||||||| |||||||||||||||||||| ||||||||||||||||| ||||||||
Sbjct  361  ATCACACTACGGGCCATCAGTAGATTTAGCTATCTTTTCTCTGCATCTTGCTGGTGCTTC  420

Query  421  TTCAATTTTAGGTGCAATCAATTTCATTACAACTATTGTAAATATACGATCCATTGGAAT  480
            |||||||||||||||||| |||||||||||||||||||||||||||||||||||||||||
Sbjct  421  TTCAATTTTAGGTGCAATTAATTTCATTACAACTATTGTAAATATACGATCCATTGGAAT  480

Query  481  AACAATAGAACGAATACCATTATTTGTATGATCTGTTTTAATTACTGCAATTTTATTATT  540
            ||||||||||||||||||||||||||||||||||||||||||||||||||||||||||||
Sbjct  481  AACAATAGAACGAATACCATTATTTGTATGATCTGTTTTAATTACTGCAATTTTATTATT  540

Query  541  ATTATCTTTACCAGTCTTAGCTGGCGCAATTACAATATTATTAACTGATCGAAACTTTAA  600
            |||||||||||||||||||||||| |||||||||||||||||||||||||||||||||||
Sbjct  541  ATTATCTTTACCAGTCTTAGCTGGTGCAATTACAATATTATTAACTGATCGAAACTTTAA  600

Query  601  TACATCATTCTTTGACCCTTCAGGAGGAGGTGACCCAATTCTATATCAACATTTATTT  658
            ||||||||||||||||||||||||||| |||||||||||||| |||||||||||||||
Sbjct  601  TACATCATTCTTTGACCCTTCAGGAGGGGGTGACCCAATTCTGTATCAACATTTATTT  658
```

>Rhipicephalus linnaei voucher P1/22_8-1 cytochrome c oxidase subunit I (COX1) gene, partial cds; mitochondrial

```
Sequence ID: OM984974.1 Length: 658
Range 1: 1 to 658

Score:1171 bits(634), Expect:0.0,
Identities:650/658(99%),  Gaps:0/658(0%), Strand: Plus/Plus

Query  1    GACAATATACTTAATTTTTGGAGCATGATCCGGAATACTAGGATTAAGAATAAGAATATT  60
            |||||||||||||||||||||||||||||||||||| |||||||||||||||||||||||
Sbjct  1    GACAATATACTTAATTTTTGGAGCATGATCCGGAATATTAGGATTAAGAATAAGAATATT  60

Query  61   AATCCGCATAGAATTAGGTCAACCAGGAACTTTAATTGGAAACGATCAAATTTATAATGT  120
            ||||||||||||||||||||||||||||||||||||||||||||||||||||||||||||
Sbjct  61   AATCCGCATAGAATTAGGTCAACCAGGAACTTTAATTGGAAACGATCAAATTTATAATGT  120

Query  121  AATTGTAACAGCACATGCATTTATTATAAttttttttATAGTTATACCAATTATAATCGG  180
            ||||||||||||||||||||||||||||||||||||||||||||||||||||||||||||
Sbjct  121  AATTGTAACAGCACATGCATTTATTATAATTTTTTTTATAGTTATACCAATTATAATCGG  180

Query  181  AGGATTTGGAAATTGATTAGTTCCTATTATACTAGGAGCTCCAGATATAGCATTTCCACG  240
            ||||||||||||||||||||||||||||||||||||||||||||||||||||||||||||
Sbjct  181  AGGATTTGGAAATTGATTAGTTCCTATTATACTAGGAGCTCCAGATATAGCATTTCCACG  240

Query  241  AATAAATAATATAAGATTTTGGTTACTTCCTCCTTCTCTATTTCTACTAATTAATTCTTC  300
            ||||||||||||||||||||||||||||||||||||||||||||||||||||||||||||
Sbjct  241  AATAAATAATATAAGATTTTGGTTACTTCCTCCTTCTCTATTTCTACTAATTAATTCTTC  300

Query  301  ATTAATCGAATCAGGAGCAGGTACAGGGTGAACAGTTTAcccccccTTATCATCAAATTT  360
            ||||||||||||||||||||||||||||||||||||||||||||||||||||||||||||
Sbjct  301  ATTAATCGAATCAGGAGCAGGTACAGGGTGAACAGTTTACCCCCCCTTATCATCAAATTT  360

Query  361  ATCACACTACGGACCATCAGTAGATTTAGCTATTTTTTCTCTGCATCTTGCAGGTGCTTC  420
            |||||||||||| ||||||||||||||||||||| ||||||||||||||||| ||||||||
Sbjct  361  ATCACACTACGGGCCATCAGTAGATTTAGCTATCTTTTCTCTGCATCTTGCTGGTGCTTC  420

Query  421  TTCAATTTTAGGTGCAATCAATTTCATTACAACTATTGTAAATATACGATCCATTGGAAT  480
            |||||||||||||||||| |||||||||||||||||||||||||||||||||||||||||
Sbjct  421  TTCAATTTTAGGTGCAATTAATTTCATTACAACTATTGTAAATATACGATCCATTGGAAT  480

Query  481  AACAATAGAACGAATACCATTATTTGTATGATCTGTTTTAATTACTGCAATTTTATTATT  540
            ||||||||||||||||||||||||||||||||||||||||||||||||||||||||||||
Sbjct  481  AACAATAGAACGAATACCATTATTTGTATGATCTGTTTTAATTACTGCAATTTTATTATT  540

Query  541  ATTATCTTTACCAGTCTTAGCTGGCGCAATTACAATATTATTAACTGATCGAAACTTTAA  600
            ||||||||||||||||||||||||| ||||||||||||||||||||||||||||||||||
Sbjct  541  ATTATCTTTACCAGTCTTAGCTGGTGCAATTACAATATTATTAACTGATCGAAACTTTAA  600

Query  601  TACATCATTCTTTGACCCTTCAGGAGGAGGTGACCCAATTCTATATCAACATTTATTT  658
            |||||||||||||||||||||||||||| |||||||||||||| |||||||||||||||
Sbjct  601  TACATCATTCTTTGACCCTTCAGGAGGGGGTGACCCAATTCTGTATCAACATTTATTT  658
```

>Rhipicephalus linnaei voucher P1/22_6-2 cytochrome c oxidase subunit I (COX1)

gene, partial cds; mitochondrial
Sequence ID: OM984973.1 Length: 658
Range 1: 1 to 658

Score:1171 bits(634), Expect:0.0,
Identities:650/658(99%), Gaps:0/658(0%), Strand: Plus/Plus

```
Query  1    GACAATATACTTAATTTTTGGAGCATGATCCGGAATACTAGGATTAAGAATAAGAATATT  60
            ||||||||||||||||||||||||||||||||||||| ||||||||||||||||||||||
Sbjct  1    GACAATATACTTAATTTTTGGAGCATGATCCGGAATATTAGGATTAAGAATAAGAATATT  60

Query  61   AATCCGCATAGAATTAGGTCAACCAGGAACTTTAATTGGAAACGATCAAATTTATAATGT  120
            ||||||||||||||||||||||||||||||||||||||||||||||||||||||||||||
Sbjct  61   AATCCGCATAGAATTAGGTCAACCAGGAACTTTAATTGGAAACGATCAAATTTATAATGT  120

Query  121  AATTGTAACAGCACATGCATTTATTATAAttttttttATAGTTATACCAATTATAATCGG  180
            ||||||||||||||||||||||||||||||||||||||||||||||||||||||||||||
Sbjct  121  AATTGTAACAGCACATGCATTTATTATAATTTTTTTTATAGTTATACCAATTATAATCGG  180

Query  181  AGGATTTGGAAATTGATTAGTTCCTATTATACTAGGAGCTCCAGATATAGCATTTCCACG  240
            ||||||||||||||||||||||||||||||||||||||||||||||||||||||||||||
Sbjct  181  AGGATTTGGAAATTGATTAGTTCCTATTATACTAGGAGCTCCAGATATAGCATTTCCACG  240

Query  241  AATAAATAATATAAGATTTTGGTTACTTCCTCCTTCTCTATTTCTACTAATTAATTCTTC  300
            ||||||||||||||||||||||||||||||||||||||||||||||||||||||||||||
Sbjct  241  AATAAATAATATAAGATTTTGGTTACTTCCTCCTTCTCTATTTCTACTAATTAATTCTTC  300

Query  301  ATTAATCGAATCAGGAGCAGGTACAGGGTGAACAGTTTAcccccccTTATCATCAAATTT  360
            ||||||||||||||||||||||||||||||||||||||||||||||||||||||||||||
Sbjct  301  ATTAATCGAATCAGGAGCAGGTACAGGGTGAACAGTTTACCCCCCCTTATCATCAAATTT  360

Query  361  ATCACACTACGGACCATCAGTAGATTTAGCTATTTTTTCTCTGCATCTTGCAGGTGCTTC  420
            |||||||||||| |||||||||||||||||||| ||||||||||||||||| ||||||||
Sbjct  361  ATCACACTACGGGCCATCAGTAGATTTAGCTATCTTTTCTCTGCATCTTGCTGGTGCTTC  420

Query  421  TTCAATTTTAGGTGCAATCAATTTCATTACAACTATTGTAAATATACGATCCATTGGAAT  480
            |||||||||||||||||| |||||||||||||||||||||||||||||||||||||||||
Sbjct  421  TTCAATTTTAGGTGCAATTAATTTCATTACAACTATTGTAAATATACGATCCATTGGAAT  480

Query  481  AACAATAGAACGAATACCATTATTTGTATGATCTGTTTTAATTACTGCAATTTTATTATT  540
            ||||||||||||||||||||||||||||||||||||||||||||||||||||||||||||
Sbjct  481  AACAATAGAACGAATACCATTATTTGTATGATCTGTTTTAATTACTGCAATTTTATTATT  540

Query  541  ATTATCTTTACCAGTCTTAGCTGGCGCAATTACAATATTATTAACTGATCGAAACTTTAA  600
            |||||||||||||||||||||||| |||||||||||||||||||||||||||||||||||
Sbjct  541  ATTATCTTTACCAGTCTTAGCTGGTGCAATTACAATATTATTAACTGATCGAAACTTTAA  600

Query  601  TACATCATTCTTTGACCCTTCAGGAGGAGGTGACCCAATTCTATATCAACATTTATTT  658
            ||||||||||||||||||||||||||| |||||||||||||| |||||||||||||||
Sbjct  601  TACATCATTCTTTGACCCTTCAGGAGGGGGTGACCCAATTCTGTATCAACATTTATTT  658
```

```
>Rhipicephalus sanguineus isolate SZM2 cytochrome oxidase subunit I (cox1) gene,
partial cds; mitochondrial
Sequence ID: JX416308.1 Length: 802
Range 1: 1 to 637

Score:1171 bits(634), Expect:0.0,
Identities:636/637(99%),  Gaps:0/637(0%), Strand: Plus/Plus

Query  22   AGCATGATCCGGAATACTAGGATTAAGAATAAGAATATTAATCCGCATAGAATTAGGTCA  81
            ||||||||||||||||||||||||||||||||||||||||||||||||||||||||||||
Sbjct  1    AGCATGATCCGGAATACTAGGATTAAGAATAAGAATATTAATCCGCATAGAATTAGGTCA  60

Query  82   ACCAGGAACTTTAATTGGAAACGATCAAATTTATAATGTAATTGTAACAGCACATGCATT  141
            ||||||||||||||||||||||||||||||||||||||||||||||||||||||||||||
Sbjct  61   ACCAGGAACTTTAATTGGAAACGATCAAATTTATAATGTAATTGTAACAGCACATGCATT  120

Query  142  TATTATAAttttttttATAGTTATACCAATTATAATCGGAGGATTTGGAAATTGATTAGT  201
            ||||||||||||||||||||||||||||||||||||||||||||||||||||||||||||
Sbjct  121  TATTATAATTTTTTTTATAGTTATACCAATTATAATCGGAGGATTTGGAAATTGATTAGT  180

Query  202  TCCTATTATACTAGGAGCTCCAGATATAGCATTTCCACGAATAAATAATATAAGATTTTG  261
            ||||||||||||||||||||||||||||||||||||||||||||||||||||||||||||
Sbjct  181  TCCTATTATACTAGGAGCTCCAGATATAGCATTTCCACGAATAAATAATATAAGATTTTG  240

Query  262  GTTACTTCCTCCTTCTCTATTTCTACTAATTAATTCTTCATTAATCGAATCAGGAGCAGG  321
            ||||||||||||||||||||||||||||||||||||||||||||||||||||||||||||
Sbjct  241  GTTACTTCCTCCTTCTCTATTTCTACTAATTAATTCTTCATTAATCGAATCAGGAGCAGG  300

Query  322  TACAGGGTGAACAGTTTAccccccccTTATCATCAAATTTATCACACTACGGACCATCAGT  381
            ||||||||||||||||||||||||||||||||||||||||||||||||||||||||||||
Sbjct  301  TACAGGGTGAACAGTTTACCCCCCCTTATCATCAAATTTATCACACTACGGACCATCAGT  360

Query  382  AGATTTAGCTATTTTTTCTCTGCATCTTGCAGGTGCTTCTTCAATTTTAGGTGCAATCAA  441
            ||||||||||||||||||||||||||||||||||||||||||||||||||||||||||||
Sbjct  361  AGATTTAGCTATTTTTTCTCTGCATCTTGCAGGTGCTTCTTCAATTTTAGGTGCAATCAA  420

Query  442  TTTCATTACAACTATTGTAAATATACGATCCATTGGAATAACAATAGAACGAATACCATT  501
            ||||||||||||||||||||||||||||||||||||||||||||||||||||||||||||
Sbjct  421  TTTCATTACAACTATTGTAAATATACGATCCATTGGAATAACAATAGAACGAATACCATT  480

Query  502  ATTTGTATGATCTGTTTTAATTACTGCAATTTTATTATTATTATCTTTACCAGTCTTAGC  561
            ||||||||||||||||||||||||||||||||||||||||||||||||||||||||||||
Sbjct  481  ATTTGTATGATCTGTTTTAATTACTGCAATTTTATTATTATTATCTTTACCAGTCTTAGC  540

Query  562  TGGCGCAATTACAATATTATTAACTGATCGAAACTTTAATACATCATTCTTTGACCCTTC  621
            |||||||||||||||||||||||||||||||||||||||||||| |||||||||||||||
Sbjct  541  TGGCGCAATTACAATATTATTAACTGATCGAAACTTTAATACAACATTCTTTGACCCTTC  600

Query  622  AGGAGGAGGTGACCCAATTCTATATCAACATTTATTT  658
            |||||||||||||||||||||||||||||||||||||
Sbjct  601  AGGAGGAGGTGACCCAATTCTATATCAACATTTATTT  637
```

```
>Rhipicephalus sanguineus isolate GTF3 cytochrome oxidase subunit I (cox1) gene,
partial cds; mitochondrial
Sequence ID: JX416304.1 Length: 802
Range 1: 1 to 637

Score:1171 bits(634), Expect:0.0,
Identities:636/637(99%),  Gaps:0/637(0%), Strand: Plus/Plus

Query  22   AGCATGATCCGGAATACTAGGATTAAGAATAAGAATATTAATCCGCATAGAATTAGGTCA  81
            ||||||||||||||||||||||||||||||||||||||||||||||||||||||||||||
Sbjct  1    AGCATGATCCGGAATACTAGGATTAAGAATAAGAATATTAATCCGCATAGAATTAGGTCA  60

Query  82   ACCAGGAACTTTAATTGGAAACGATCAAATTTATAATGTAATTGTAACAGCACATGCATT  141
            ||||||||||||||||||||||||||||||||||||||||||||||||||| ||||||||
Sbjct  61   ACCAGGAACTTTAATTGGAAACGATCAAATTTATAATGTAATTGTAACAGCTCATGCATT  120

Query  142  TATTATAAtttttttATAGTTATACCAATTATAATCGGAGGATTTGGAAATTGATTAGT  201
            ||||||||||||||||||||||||||||||||||||||||||||||||||||||||||||
Sbjct  121  TATTATAATTTTTTTATAGTTATACCAATTATAATCGGAGGATTTGGAAATTGATTAGT  180

Query  202  TCCTATTATACTAGGAGCTCCAGATATAGCATTTCCACGAATAAATAATATAAGATTTTG  261
            ||||||||||||||||||||||||||||||||||||||||||||||||||||||||||||
Sbjct  181  TCCTATTATACTAGGAGCTCCAGATATAGCATTTCCACGAATAAATAATATAAGATTTTG  240

Query  262  GTTACTTCCTCCTTCTCTATTTCTACTAATTAATTCTTCATTAATCGAATCAGGAGCAGG  321
            ||||||||||||||||||||||||||||||||||||||||||||||||||||||||||||
Sbjct  241  GTTACTTCCTCCTTCTCTATTTCTACTAATTAATTCTTCATTAATCGAATCAGGAGCAGG  300

Query  322  TACAGGGTGAACAGTTTAcccccccTTATCATCAAATTTATCACACTACGGACCATCAGT  381
            ||||||||||||||||||||||||||||||||||||||||||||||||||||||||||||
Sbjct  301  TACAGGGTGAACAGTTTACCCCCCCTTATCATCAAATTTATCACACTACGGACCATCAGT  360

Query  382  AGATTTAGCTATTTTTTCTCTGCATCTTGCAGGTGCTTCTTCAATTTTAGGTGCAATCAA  441
            ||||||||||||||||||||||||||||||||||||||||||||||||||||||||||||
Sbjct  361  AGATTTAGCTATTTTTTCTCTGCATCTTGCAGGTGCTTCTTCAATTTTAGGTGCAATCAA  420

Query  442  TTTCATTACAACTATTGTAAATATACGATCCATTGGAATAACAATAGAACGAATACCATT  501
            ||||||||||||||||||||||||||||||||||||||||||||||||||||||||||||
Sbjct  421  TTTCATTACAACTATTGTAAATATACGATCCATTGGAATAACAATAGAACGAATACCATT  480

Query  502  ATTTGTATGATCTGTTTTAATTACTGCAATTTTATTATTATTATCTTTACCAGTCTTAGC  561
            ||||||||||||||||||||||||||||||||||||||||||||||||||||||||||||
Sbjct  481  ATTTGTATGATCTGTTTTAATTACTGCAATTTTATTATTATTATCTTTACCAGTCTTAGC  540

Query  562  TGGCGCAATTACAATATTATTAACTGATCGAAACTTTAATACATCATTCTTTGACCCTTC  621
            ||||||||||||||||||||||||||||||||||||||||||||||||||||||||||||
Sbjct  541  TGGCGCAATTACAATATTATTAACTGATCGAAACTTTAATACATCATTCTTTGACCCTTC  600

Query  622  AGGAGGAGGTGACCCAATTCTATATCAACATTTATTT  658
            |||||||||||||||||||||||||||||||||||||
Sbjct  601  AGGAGGAGGTGACCCAATTCTATATCAACATTTATTT  637
```

```
>Rhipicephalus sanguineus isolate GBM1 cytochrome oxidase subunit I (cox1) gene,
partial cds; mitochondrial
Sequence ID: JX416300.1 Length: 802
Range 1: 1 to 637

Score:1171 bits(634), Expect:0.0,
Identities:636/637(99%),  Gaps:0/637(0%), Strand: Plus/Plus

Query  22   AGCATGATCCGGAATACTAGGATTAAGAATAAGAATATTAATCCGCATAGAATTAGGTCA  81
            ||||||||||||||||||||||||||||||||||||||||||||||||||||||||||||
Sbjct  1    AGCATGATCCGGAATACTAGGATTAAGAATAAGAATATTAATCCGCATAGAATTAGGTCA  60

Query  82   ACCAGGAACTTTAATTGGAAACGATCAAATTTATAATGTAATTGTAACAGCACATGCATT  141
            ||||||||||||||||||||||||||||||||||||||||||||||||||||||||||||
Sbjct  61   ACCAGGAACTTTAATTGGAAACGATCAAATTTATAATGTAATTGTAACAGCACATGCATT  120

Query  142  TATTATAAttttttttATAGTTATACCAATTATAATCGGAGGATTTGGAAATTGATTAGT  201
            ||||||||||||||||||||||||||||||||||||||||||||||| ||||||||||||
Sbjct  121  TATTATAATTTTTTTTATAGTTATACCAATTATAATCGGAGGATTTGGGAATTGATTAGT  180

Query  202  TCCTATTATACTAGGAGCTCCAGATATAGCATTTCCACGAATAAATAATATAAGATTTTG  261
            ||||||||||||||||||||||||||||||||||||||||||||||||||||||||||||
Sbjct  181  TCCTATTATACTAGGAGCTCCAGATATAGCATTTCCACGAATAAATAATATAAGATTTTG  240

Query  262  GTTACTTCCTCCTTCTCTATTTCTACTAATTAATTCTTCATTAATCGAATCAGGAGCAGG  321
            ||||||||||||||||||||||||||||||||||||||||||||||||||||||||||||
Sbjct  241  GTTACTTCCTCCTTCTCTATTTCTACTAATTAATTCTTCATTAATCGAATCAGGAGCAGG  300

Query  322  TACAGGGTGAACAGTTTAccccccccTTATCATCAAATTTATCACACTACGGACCATCAGT  381
            ||||||||||||||||||||||||||||||||||||||||||||||||||||||||||||
Sbjct  301  TACAGGGTGAACAGTTTACCCCCCCCTTATCATCAAATTTATCACACTACGGACCATCAGT  360

Query  382  AGATTTAGCTATTTTTTCTCTGCATCTTGCAGGTGCTTCTTCAATTTTAGGTGCAATCAA  441
            ||||||||||||||||||||||||||||||||||||||||||||||||||||||||||||
Sbjct  361  AGATTTAGCTATTTTTTCTCTGCATCTTGCAGGTGCTTCTTCAATTTTAGGTGCAATCAA  420

Query  442  TTTCATTACAACTATTGTAAATATACGATCCATTGGAATAACAATAGAACGAATACCATT  501
            ||||||||||||||||||||||||||||||||||||||||||||||||||||||||||||
Sbjct  421  TTTCATTACAACTATTGTAAATATACGATCCATTGGAATAACAATAGAACGAATACCATT  480

Query  502  ATTTGTATGATCTGTTTTAATTACTGCAATTTTATTATTATTATCTTTACCAGTCTTAGC  561
            ||||||||||||||||||||||||||||||||||||||||||||||||||||||||||||
Sbjct  481  ATTTGTATGATCTGTTTTAATTACTGCAATTTTATTATTATTATCTTTACCAGTCTTAGC  540

Query  562  TGGCGCAATTACAATATTATTAACTGATCGAAACTTTAATACATCATTCTTTGACCCTTC  621
            ||||||||||||||||||||||||||||||||||||||||||||||||||||||||||||
Sbjct  541  TGGCGCAATTACAATATTATTAACTGATCGAAACTTTAATACATCATTCTTTGACCCTTC  600

Query  622  AGGAGGAGGTGACCCAATTCTATATCAACATTTATTT  658
            |||||||||||||||||||||||||||||||||||||
Sbjct  601  AGGAGGAGGTGACCCAATTCTATATCAACATTTATTT  637
```

```
>Rhipicephalus sanguineus isolate GBF1 cytochrome oxidase subunit I (cox1) gene,
partial cds; mitochondrial
Sequence ID: JX416299.1 Length: 802
Range 1: 1 to 637

Score:1171 bits(634), Expect:0.0,
Identities:636/637(99%),  Gaps:0/637(0%), Strand: Plus/Plus

Query  22   AGCATGATCCGGAATACTAGGATTAAGAATAAGAATATTAATCCGCATAGAATTAGGTCA  81
            |||||||||||| |||||||||||||||||||||||||||||||||||||||||||||||
Sbjct  1    AGCATGATCCGGGATACTAGGATTAAGAATAAGAATATTAATCCGCATAGAATTAGGTCA  60

Query  82   ACCAGGAACTTTAATTGGAAACGATCAAATTTATAATGTAATTGTAACAGCACATGCATT  141
            ||||||||||||||||||||||||||||||||||||||||||||||||||||||||||||
Sbjct  61   ACCAGGAACTTTAATTGGAAACGATCAAATTTATAATGTAATTGTAACAGCACATGCATT  120

Query  142  TATTATAAtttttttATAGTTATACCAATTATAATCGGAGGATTTGGAAATTGATTAGT  201
            ||||||||||||||||||||||||||||||||||||||||||||||||||||||||||||
Sbjct  121  TATTATAATTTTTTTATAGTTATACCAATTATAATCGGAGGATTTGGAAATTGATTAGT  180

Query  202  TCCTATTATACTAGGAGCTCCAGATATAGCATTTCCACGAATAAATAATATAAGATTTTG  261
            ||||||||||||||||||||||||||||||||||||||||||||||||||||||||||||
Sbjct  181  TCCTATTATACTAGGAGCTCCAGATATAGCATTTCCACGAATAAATAATATAAGATTTTG  240

Query  262  GTTACTTCCTCCTTCTCTATTTCTACTAATTAATTCTTCATTAATCGAATCAGGAGCAGG  321
            ||||||||||||||||||||||||||||||||||||||||||||||||||||||||||||
Sbjct  241  GTTACTTCCTCCTTCTCTATTTCTACTAATTAATTCTTCATTAATCGAATCAGGAGCAGG  300

Query  322  TACAGGGTGAACAGTTTAcccccccTTATCATCAAATTTATCACACTACGGACCATCAGT  381
            ||||||||||||||||||||||||||||||||||||||||||||||||||||||||||||
Sbjct  301  TACAGGGTGAACAGTTTACCCCCCCTTATCATCAAATTTATCACACTACGGACCATCAGT  360

Query  382  AGATTTAGCTATTTTTTCTCTGCATCTTGCAGGTGCTTCTTCAATTTTAGGTGCAATCAA  441
            ||||||||||||||||||||||||||||||||||||||||||||||||||||||||||||
Sbjct  361  AGATTTAGCTATTTTTTCTCTGCATCTTGCAGGTGCTTCTTCAATTTTAGGTGCAATCAA  420

Query  442  TTTCATTACAACTATTGTAAATATACGATCCATTGGAATAACAATAGAACGAATACCATT  501
            ||||||||||||||||||||||||||||||||||||||||||||||||||||||||||||
Sbjct  421  TTTCATTACAACTATTGTAAATATACGATCCATTGGAATAACAATAGAACGAATACCATT  480

Query  502  ATTTGTATGATCTGTTTTAATTACTGCAATTTTATTATTATTATCTTTACCAGTCTTAGC  561
            ||||||||||||||||||||||||||||||||||||||||||||||||||||||||||||
Sbjct  481  ATTTGTATGATCTGTTTTAATTACTGCAATTTTATTATTATTATCTTTACCAGTCTTAGC  540

Query  562  TGGCGCAATTACAATATTATTAACTGATCGAAACTTTAATACATCATTCTTTGACCCTTC  621
            ||||||||||||||||||||||||||||||||||||||||||||||||||||||||||||
Sbjct  541  TGGCGCAATTACAATATTATTAACTGATCGAAACTTTAATACATCATTCTTTGACCCTTC  600

Query  622  AGGAGGAGGTGACCCAATTCTATATCAACATTTATTT  658
            |||||||||||||||||||||||||||||||||||||
Sbjct  601  AGGAGGAGGTGACCCAATTCTATATCAACATTTATTT  637
```

>Rhipicephalus sanguineus isolate CHF1 cytochrome oxidase subunit I (cox1) gene, partial cds; mitochondrial
Sequence ID: JX416297.1 Length: 802
Range 1: 1 to 637

Score:1171 bits(634), Expect:0.0,
Identities:636/637(99%), Gaps:0/637(0%), Strand: Plus/Plus

```
Query  22   AGCATGATCCGGAATACTAGGATTAAGAATAAGAATATTAATCCGCATAGAATTAGGTCA  81
            ||||||||||||||||||||||||||||||||||||||||||||||||||||||||||||
Sbjct  1    AGCATGATCCGGAATACTAGGATTAAGAATAAGAATATTAATCCGCATAGAATTAGGTCA  60

Query  82   ACCAGGAACTTTAATTGGAAACGATCAAATTTATAATGTAATTGTAACAGCACATGCATT  141
            ||||||||||||||||||||||||||||||||||||||||||||||||||||||||||||
Sbjct  61   ACCAGGAACTTTAATTGGAAACGATCAAATTTATAATGTAATTGTAACAGCACATGCATT  120

Query  142  TATTATAAtttttttATAGTTATACCAATTATAATCGGAGGATTTGGAAATTGATTAGT  201
            ||||||||||||||||||||||||||||||||||||||||||||||||||||||||||||
Sbjct  121  TATTATAATTTTTTTTATAGTTATACCAATTATAATCGGAGGATTTGGAAATTGATTAGT  180

Query  202  TCCTATTATACTAGGAGCTCCAGATATAGCATTTCCACGAATAAATAATATAAGATTTTG  261
            ||||||||||||||||||||||||||||||||||||||||||||||||||||||||||||
Sbjct  181  TCCTATTATACTAGGAGCTCCAGATATAGCATTTCCACGAATAAATAATATAAGATTTTG  240

Query  262  GTTACTTCCTCCTTCTCTATTTCTACTAATTAATTCTTCATTAATCGAATCAGGAGCAGG  321
            ||||||||||||||||||||||||||||||||||||||||||||||||||||||||||||
Sbjct  241  GTTACTTCCTCCTTCTCTATTTCTACTAATTAATTCTTCATTAATCGAATCAGGAGCAGG  300

Query  322  TACAGGGTGAACAGTTTAcccccccTTATCATCAAATTTATCACACTACGGACCATCAGT  381
            ||||||||||||||||||||||||||||||||||||||||||||||||||||||||||||
Sbjct  301  TACAGGGTGAACAGTTTACCCCCCCTTATCATCAAATTTATCACACTACGGACCATCAGT  360

Query  382  AGATTTAGCTATTTTTTCTCTGCATCTTGCAGGTGCTTCTTCAATTTTAGGTGCAATCAA  441
            ||||||||||||||||||||||||||||||||||||||||||||||||||||||||||||
Sbjct  361  AGATTTAGCTATTTTTTCTCTGCATCTTGCAGGTGCTTCTTCAATTTTAGGTGCAATCAA  420

Query  442  TTTCATTACAACTATTGTAAATATACGATCCATTGGAATAACAATAGAACGAATACCATT  501
            ||||||||||||||||||||||||||||||||||||||||||||||||||||||||||||
Sbjct  421  TTTCATTACAACTATTGTAAATATACGATCCATTGGAATAACAATAGAACGAATACCATT  480

Query  502  ATTTGTATGATCTGTTTTAATTACTGCAATTTTATTATTATTATCTTTACCAGTCTTAGC  561
            ||||||||||||||||||||||||||||||||||||||||| ||||||||||||||||||
Sbjct  481  ATTTGTATGATCTGTTTTAATTACTGCAATTTTATTATTACTATCTTTACCAGTCTTAGC  540

Query  562  TGGCGCAATTACAATATTATTAACTGATCGAAACTTTAATACATCATTCTTTGACCCTTC  621
            ||||||||||||||||||||||||||||||||||||||||||||||||||||||||||||
Sbjct  541  TGGCGCAATTACAATATTATTAACTGATCGAAACTTTAATACATCATTCTTTGACCCTTC  600

Query  622  AGGAGGAGGTGACCCAATTCTATATCAACATTTATTT  658
            |||||||||||||||||||||||||||||||||||||
```

```
Sbjct  601  AGGAGGAGGTGACCCAATTCTATATCAACATTTATTT  637
```

```
>Rhipicephalus sanguineus isolate DGM1 cytochrome oxidase subunit I (cox1) gene,
partial cds; mitochondrial
Sequence ID: JX416296.1 Length: 802
Range 1: 1 to 637

Score:1171 bits(634), Expect:0.0,
Identities:636/637(99%),  Gaps:0/637(0%), Strand: Plus/Plus

Query  22   AGCATGATCCGGAATACTAGGATTAAGAATAAGAATATTAATCCGCATAGAATTAGGTCA  81
            ||||||||||||||||||||||||||||||||||||||||||||||||||||||||||||
Sbjct  1    AGCATGATCCGGAATACTAGGATTAAGAATAAGAATATTAATCCGCATAGAATTAGGTCA  60

Query  82   ACCAGGAACTTTAATTGGAAACGATCAAATTTATAATGTAATTGTAACAGCACATGCATT  141
            ||||||||||||||||||||||||||||||||||||||||||||||||||||||||||||
Sbjct  61   ACCAGGAACTTTAATTGGAAACGATCAAATTTATAATGTAATTGTAACAGCACATGCATT  120

Query  142  TATTATAAttttttttATAGTTATACCAATTATAATCGGAGGATTTGGAAATTGATTAGT  201
            |||||| |||||||||||||||||||||||||||||||||||||||||||||||||||||
Sbjct  121  TATTATGATTTTTTTTATAGTTATACCAATTATAATCGGAGGATTTGGAAATTGATTAGT  180

Query  202  TCCTATTATACTAGGAGCTCCAGATATAGCATTTCCACGAATAAATAATATAAGATTTTG  261
            ||||||||||||||||||||||||||||||||||||||||||||||||||||||||||||
Sbjct  181  TCCTATTATACTAGGAGCTCCAGATATAGCATTTCCACGAATAAATAATATAAGATTTTG  240

Query  262  GTTACTTCCTCCTTCTCTATTTCTACTAATTAATTCTTCATTAATCGAATCAGGAGCAGG  321
            ||||||||||||||||||||||||||||||||||||||||||||||||||||||||||||
Sbjct  241  GTTACTTCCTCCTTCTCTATTTCTACTAATTAATTCTTCATTAATCGAATCAGGAGCAGG  300

Query  322  TACAGGGTGAACAGTTTAcccccccTTATCATCAAATTTATCACACTACGGACCATCAGT  381
            ||||||||||||||||||||||||||||||||||||||||||||||||||||||||||||
Sbjct  301  TACAGGGTGAACAGTTTACCCCCCCTTATCATCAAATTTATCACACTACGGACCATCAGT  360

Query  382  AGATTTAGCTATTTTTTCTCTGCATCTTGCAGGTGCTTCTTCAATTTTAGGTGCAATCAA  441
            ||||||||||||||||||||||||||||||||||||||||||||||||||||||||||||
Sbjct  361  AGATTTAGCTATTTTTTCTCTGCATCTTGCAGGTGCTTCTTCAATTTTAGGTGCAATCAA  420

Query  442  TTTCATTACAACTATTGTAAATATACGATCCATTGGAATAACAATAGAACGAATACCATT  501
            ||||||||||||||||||||||||||||||||||||||||||||||||||||||||||||
Sbjct  421  TTTCATTACAACTATTGTAAATATACGATCCATTGGAATAACAATAGAACGAATACCATT  480

Query  502  ATTTGTATGATCTGTTTTAATTACTGCAATTTTATTATTATTATCTTTACCAGTCTTAGC  561
            ||||||||||||||||||||||||||||||||||||||||||||||||||||||||||||
Sbjct  481  ATTTGTATGATCTGTTTTAATTACTGCAATTTTATTATTATTATCTTTACCAGTCTTAGC  540

Query  562  TGGCGCAATTACAATATTATTAACTGATCGAAACTTTAATACATCATTCTTTGACCCTTC  621
            ||||||||||||||||||||||||||||||||||||||||||||||||||||||||||||
Sbjct  541  TGGCGCAATTACAATATTATTAACTGATCGAAACTTTAATACATCATTCTTTGACCCTTC  600

Query  622  AGGAGGAGGTGACCCAATTCTATATCAACATTTATTT  658
```

```
                 |||||||||||||||||||||||||||||||||||||
Sbjct  601  AGGAGGAGGTGACCCAATTCTATATCAACATTTATTT  637
```

>Rhipicephalus sanguineus isolate FSF1 cytochrome oxidase subunit I (cox1) gene, partial cds; mitochondrial
Sequence ID: JX416293.1 Length: 802
Range 1: 1 to 637

Score:1171 bits(634), Expect:0.0,
Identities:636/637(99%), Gaps:0/637(0%), Strand: Plus/Plus

```
Query  22   AGCATGATCCGGAATACTAGGATTAAGAATAAGAATATTAATCCGCATAGAATTAGGTCA  81
            ||||||||||||||||||||||||| ||||||||||||||||||||||||||||||||||
Sbjct  1    AGCATGATCCGGAATACTAGGATTACGAATAAGAATATTAATCCGCATAGAATTAGGTCA  60

Query  82   ACCAGGAACTTTAATTGGAAACGATCAAATTTATAATGTAATTGTAACAGCACATGCATT  141
            ||||||||||||||||||||||||||||||||||||||||||||||||||||||||||||
Sbjct  61   ACCAGGAACTTTAATTGGAAACGATCAAATTTATAATGTAATTGTAACAGCACATGCATT  120

Query  142  TATTATAAtttttttATAGTTATACCAATTATAATCGGAGGATTTGGAAATTGATTAGT  201
            ||||||||||||||||||||||||||||||||||||||||||||||||||||||||||||
Sbjct  121  TATTATAATTTTTTTATAGTTATACCAATTATAATCGGAGGATTTGGAAATTGATTAGT  180

Query  202  TCCTATTATACTAGGAGCTCCAGATATAGCATTTCCACGAATAAATAATATAAGATTTTG  261
            ||||||||||||||||||||||||||||||||||||||||||||||||||||||||||||
Sbjct  181  TCCTATTATACTAGGAGCTCCAGATATAGCATTTCCACGAATAAATAATATAAGATTTTG  240

Query  262  GTTACTTCCTCCTTCTCTATTTCTACTAATTAATTCTTCATTAATCGAATCAGGAGCAGG  321
            ||||||||||||||||||||||||||||||||||||||||||||||||||||||||||||
Sbjct  241  GTTACTTCCTCCTTCTCTATTTCTACTAATTAATTCTTCATTAATCGAATCAGGAGCAGG  300

Query  322  TACAGGGTGAACAGTTTAcccccccTTATCATCAAATTTATCACACTACGGACCATCAGT  381
            ||||||||||||||||||||||||||||||||||||||||||||||||||||||||||||
Sbjct  301  TACAGGGTGAACAGTTTACCCCCCCTTATCATCAAATTTATCACACTACGGACCATCAGT  360

Query  382  AGATTTAGCTATTTTTTCTCTGCATCTTGCAGGTGCTTCTTCAATTTTAGGTGCAATCAA  441
            ||||||||||||||||||||||||||||||||||||||||||||||||||||||||||||
Sbjct  361  AGATTTAGCTATTTTTTCTCTGCATCTTGCAGGTGCTTCTTCAATTTTAGGTGCAATCAA  420

Query  442  TTTCATTACAACTATTGTAAATATACGATCCATTGGAATAACAATAGAACGAATACCATT  501
            ||||||||||||||||||||||||||||||||||||||||||||||||||||||||||||
Sbjct  421  TTTCATTACAACTATTGTAAATATACGATCCATTGGAATAACAATAGAACGAATACCATT  480

Query  502  ATTTGTATGATCTGTTTTAATTACTGCAATTTTATTATTATTATCTTTACCAGTCTTAGC  561
            ||||||||||||||||||||||||||||||||||||||||||||||||||||||||||||
Sbjct  481  ATTTGTATGATCTGTTTTAATTACTGCAATTTTATTATTATTATCTTTACCAGTCTTAGC  540

Query  562  TGGCGCAATTACAATATTATTAACTGATCGAAACTTTAATACATCATTCTTTGACCCTTC  621
            ||||||||||||||||||||||||||||||||||||||||||||||||||||||||||||
Sbjct  541  TGGCGCAATTACAATATTATTAACTGATCGAAACTTTAATACATCATTCTTTGACCCTTC  600
```

```
Query  622  AGGAGGAGGTGACCCAATTCTATATCAACATTTATTT  658
            |||||||||||||||||||||||||||||||||||||
Sbjct  601  AGGAGGAGGTGACCCAATTCTATATCAACATTTATTT  637
```

>Rhipicephalus sanguineus isolate T78D2 cytochrome c oxidase subunit I (COX1) gene, partial cds; mitochondrial
Sequence ID: MZ401439.1 Length: 650
Range 1: 1 to 634

Score:1171 bits(634), Expect:0.0,
Identities:634/634(100%), Gaps:0/634(0%), Strand: Plus/Plus

```
Query  25   ATGATCCGGAATACTAGGATTAAGAATAAGAATATTAATCCGCATAGAATTAGGTCAACC  84
            ||||||||||||||||||||||||||||||||||||||||||||||||||||||||||||
Sbjct  1    ATGATCCGGAATACTAGGATTAAGAATAAGAATATTAATCCGCATAGAATTAGGTCAACC  60

Query  85   AGGAACTTTAATTGGAAACGATCAAATTTATAATGTAATTGTAACAGCACATGCATTTAT  144
            ||||||||||||||||||||||||||||||||||||||||||||||||||||||||||||
Sbjct  61   AGGAACTTTAATTGGAAACGATCAAATTTATAATGTAATTGTAACAGCACATGCATTTAT  120

Query  145  TATAAtttttttATAGTTATACCAATTATAATCGGAGGATTTGGAAATTGATTAGTTCC  204
            ||||||||||||||||||||||||||||||||||||||||||||||||||||||||||||
Sbjct  121  TATAATTTTTTTATAGTTATACCAATTATAATCGGAGGATTTGGAAATTGATTAGTTCC  180

Query  205  TATTATACTAGGAGCTCCAGATATAGCATTTCCACGAATAAATAATATAAGATTTTGGTT  264
            ||||||||||||||||||||||||||||||||||||||||||||||||||||||||||||
Sbjct  181  TATTATACTAGGAGCTCCAGATATAGCATTTCCACGAATAAATAATATAAGATTTTGGTT  240

Query  265  ACTTCCTCCTTCTCTATTTCTACTAATTAATTCTTCATTAATCGAATCAGGAGCAGGTAC  324
            ||||||||||||||||||||||||||||||||||||||||||||||||||||||||||||
Sbjct  241  ACTTCCTCCTTCTCTATTTCTACTAATTAATTCTTCATTAATCGAATCAGGAGCAGGTAC  300

Query  325  AGGGTGAACAGTTTAcccccccTTATCATCAAATTTATCACACTACGGACCATCAGTAGA  384
            ||||||||||||||||||||||||||||||||||||||||||||||||||||||||||||
Sbjct  301  AGGGTGAACAGTTTACCCCCCCTTATCATCAAATTTATCACACTACGGACCATCAGTAGA  360

Query  385  TTTAGCTATTTTTTCTCTGCATCTTGCAGGTGCTTCTTCAATTTTAGGTGCAATCAATTT  444
            ||||||||||||||||||||||||||||||||||||||||||||||||||||||||||||
Sbjct  361  TTTAGCTATTTTTTCTCTGCATCTTGCAGGTGCTTCTTCAATTTTAGGTGCAATCAATTT  420

Query  445  CATTACAACTATTGTAAATATACGATCCATTGGAATAACAATAGAACGAATACCATTATT  504
            ||||||||||||||||||||||||||||||||||||||||||||||||||||||||||||
Sbjct  421  CATTACAACTATTGTAAATATACGATCCATTGGAATAACAATAGAACGAATACCATTATT  480

Query  505  TGTATGATCTGTTTTAATTACTGCAATTTTATTATTATTATCTTTACCAGTCTTAGCTGG  564
            ||||||||||||||||||||||||||||||||||||||||||||||||||||||||||||
Sbjct  481  TGTATGATCTGTTTTAATTACTGCAATTTTATTATTATTATCTTTACCAGTCTTAGCTGG  540

Query  565  CGCAATTACAATATTATTAACTGATCGAAACTTTAATACATCATTCTTTGACCCTTCAGG  624
            ||||||||||||||||||||||||||||||||||||||||||||||||||||||||||||
Sbjct  541  CGCAATTACAATATTATTAACTGATCGAAACTTTAATACATCATTCTTTGACCCTTCAGG  600
```

```
Query  625  AGGAGGTGACCCAATTCTATATCAACATTTATTT  658
            ||||||||||||||||||||||||||||||||||
Sbjct  601  AGGAGGTGACCCAATTCTATATCAACATTTATTT  634
```

>Rhipicephalus sanguineus isolate LIC5762 cytochrome oxidase subunit 1 (COI) gene, partial cds; mitochondrial
Sequence ID: KX383802.1 Length: 763
>Rhipicephalus sanguineus isolate LIC5767C cytochrome oxidase subunit 1 (COI) gene, partial cds; mitochondrial
Sequence ID: KX383803.1 Length: 763
>Rhipicephalus sanguineus isolate LIC5767D cytochrome oxidase subunit 1 (COI) gene, partial cds; mitochondrial
Sequence ID: KX383804.1 Length: 763
Range 1: 1 to 637

Score:1166 bits(631), Expect:0.0,
Identities:635/637(99%), Gaps:0/637(0%), Strand: Plus/Plus

```
Query  22   AGCATGATCCGGAATACTAGGATTAAGAATAAGAATATTAATCCGCATAGAATTAGGTCA  81
            ||||||||||||||||||||||||||||||||||||||||||||||||||||||||||||
Sbjct  1    AGCATGATCCGGAATACTAGGATTAAGAATAAGAATATTAATCCGCATAGAATTAGGTCA  60

Query  82   ACCAGGAACTTTAATTGGAAACGATCAAATTTATAATGTAATTGTAACAGCACATGCATT  141
            ||||||||||||||||||||||||||||||||||||||||||||||||||||||||||||
Sbjct  61   ACCAGGAACTTTAATTGGAAACGATCAAATTTATAATGTAATTGTAACAGCACATGCATT  120

Query  142  TATTATAAttttttttATAGTTATACCAATTATAATCGGAGGATTTGGAAATTGATTAGT  201
            ||||||||||||||||||||||||||||||||||||||||||||||||||||||||||||
Sbjct  121  TATTATAATTTTTTTTATAGTTATACCAATTATAATCGGAGGATTTGGAAATTGATTAGT  180

Query  202  TCCTATTATACTAGGAGCTCCAGATATAGCATTTCCACGAATAAATAATATAAGATTTTG  261
            ||||||||||||||||||||||||||||||||||||||||||||||||||||||||||||
Sbjct  181  TCCTATTATACTAGGAGCTCCAGATATAGCATTTCCACGAATAAATAATATAAGATTTTG  240

Query  262  GTTACTTCCTCCTTCTCTATTTCTACTAATTAATTCTTCATTAATCGAATCAGGAGCAGG  321
            ||||||||||||||||||||||||||||||||||||||||||||||||||||||||||||
Sbjct  241  GTTACTTCCTCCTTCTCTATTTCTACTAATTAATTCTTCATTAATCGAATCAGGAGCAGG  300

Query  322  TACAGGGTGAACAGTTTAcccccccTTATCATCAAATTTATCACACTACGGACCATCAGT  381
            ||||||||||||||||||||||||||||||||||||||||||||||||||||||||||||
Sbjct  301  TACAGGGTGAACAGTTTACCCCCCCTTATCATCAAATTTATCACACTACGGACCATCAGT  360

Query  382  AGATTTAGCTATTTTTTCTCTGCATCTTGCAGGTGCTTCTTCAATTTTAGGTGCAATCAA  441
            ||||||||||||||||||||| ||||||||||||||||||||||||||||||||||||||
Sbjct  361  AGATTTAGCTATTTTTTCTCTACATCTTGCAGGTGCTTCTTCAATTTTAGGTGCAATCAA  420

Query  442  TTTCATTACAACTATTGTAAATATACGATCCATTGGAATAACAATAGAACGAATACCATT  501
            ||||||||||||||||||||||||||||||||||||||||||||||||||||||||||||
Sbjct  421  TTTCATTACAACTATTGTAAATATACGATCCATTGGAATAACAATAGAACGAATACCATT  480
```

```
Query  502  ATTTGTATGATCTGTTTTAATTACTGCAATTTTATTATTATTATCTTTACCAGTCTTAGC  561
            ||||||||||||||||||||||||||||||||||||||||||||||||||||||||||||
Sbjct  481  ATTTGTATGATCTGTTTTAATTACTGCAATTTTATTATTATTATCTTTACCAGTCTTAGC  540

Query  562  TGGCGCAATTACAATATTATTAACTGATCGAAACTTTAATACATCATTCTTTGACCCTTC  621
            ||||||||||||||||||||||||||||||||||||||||||||||||||||||||||||
Sbjct  541  TGGCGCAATTACAATATTATTAACTGATCGAAACTTTAATACATCATTCTTTGACCCTTC  600

Query  622  AGGAGGAGGTGACCCAATTCTATATCAACATTTATTT  658
            |||||| ||||||||||||||||||||||||||||||
Sbjct  601  AGGAGGGGGTGACCCAATTCTATATCAACATTTATTT  637
```

>Rhipicephalus linnaei voucher P1/22_16-1 cytochrome c oxidase subunit I (COX1) gene, partial cds; mitochondrial
Sequence ID: OM984979.1 Length: 658
Range 1: 1 to 658

Score:1166 bits(631), Expect:0.0,
Identities:649/658(99%), Gaps:0/658(0%), Strand: Plus/Plus

```
Query  1    GACAATATACTTAATTTTTGGAGCATGATCCGGAATACTAGGATTAAGAATAAGAATATT  60
            ||||||||||||||||||||||||||||||||||||| ||||||||||||||||||||||
Sbjct  1    GACAATATACTTAATTTTTGGAGCATGATCCGGAATATTAGGATTAAGAATAAGAATATT  60

Query  61   AATCCGCATAGAATTAGGTCAACCAGGAACTTTAATTGGAAACGATCAAATTTATAATGT  120
            ||||||||||||||||||||||||||||||||||||||||||||||||||||||||||||
Sbjct  61   AATCCGCATAGAATTAGGTCAACCAGGAACTTTAATTGGAAACGATCAAATTTATAATGT  120

Query  121  AATTGTAACAGCACATGCATTTATTATAAttttttttATAGTTATACCAATTATAATCGG  180
            ||||||||||||||||||||||||||||||||||||||||||||||||||||||||||||
Sbjct  121  AATTGTAACAGCACATGCATTTATTATAATTTTTTTTATAGTTATACCAATTATAATCGG  180

Query  181  AGGATTTGGAAATTGATTAGTTCCTATTATACTAGGAGCTCCAGATATAGCATTTCCACG  240
            |||||||||||||||||||||||||||||| |||||||||||||||||||||||||||||
Sbjct  181  AGGATTTGGAAATTGATTAGTTCCTATTATCCTAGGAGCTCCAGATATAGCATTTCCACG  240

Query  241  AATAAATAATATAAGATTTTGGTTACTTCCTCCTTCTCTATTTCTACTAATTAATTCTTC  300
            ||||||||||||||||||||||||||||||||||||||||||||||||||||||||||||
Sbjct  241  AATAAATAATATAAGATTTTGGTTACTTCCTCCTTCTCTATTTCTACTAATTAATTCTTC  300

Query  301  ATTAATCGAATCAGGAGCAGGTACAGGGTGAACAGTTTAcccccccTTATCATCAAATTT  360
            ||||||||||||||||||||||||||||||||||||||||||||||||||||||||||||
Sbjct  301  ATTAATCGAATCAGGAGCAGGTACAGGGTGAACAGTTTACCCCCCCTTATCATCAAATTT  360

Query  361  ATCACACTACGGACCATCAGTAGATTTAGCTATTTTTTCTCTGCATCTTGCAGGTGCTTC  420
            |||||||||||| |||||||||||||||||||| ||||||||||||||||| ||||||||
Sbjct  361  ATCACACTACGGGCCATCAGTAGATTTAGCTATCTTTTCTCTGCATCTTGCCGGTGCTTC  420

Query  421  TTCAATTTTAGGTGCAATCAATTTCATTACAACTATTGTAAATATACGATCCATTGGAAT  480
            |||||||||||||||||| |||||||||||||||||||||||||||||||||||||| ||
Sbjct  421  TTCAATTTTAGGTGCAATTAATTTCATTACAACTATTGTAAATATACGATCCATTGGGAT  480
```

```
Query  481   AACAATAGAACGAATACCATTATTTGTATGATCTGTTTTAATTACTGCAATTTTATTATT  540
             ||||||||||||||||||||||||||||||||||||||||||||||||||||||||||||
Sbjct  481   AACAATAGAACGAATACCATTATTTGTATGATCTGTTTTAATTACTGCAATTTTATTATT  540

Query  541   ATTATCTTTACCAGTCTTAGCTGGCGCAATTACAATATTATTAACTGATCGAAACTTTAA  600
             ||||||||||||||||||||||||||||||||||||||||||||||||||||||||||||
Sbjct  541   ATTATCTTTACCAGTCTTAGCTGGCGCAATTACAATATTATTAACTGATCGAAACTTTAA  600

Query  601   TACATCATTCTTTGACCCTTCAGGAGGAGGTGACCCAATTCTATATCAACATTTATTT  658
             ||||||||||||||||||||||||||| |||||||||||||| |||||||||||||||
Sbjct  601   TACATCATTCTTTGACCCTTCAGGAGGGGGTGACCCAATTCTGTATCAACATTTATTT  658
```

>Rhipicephalus linnaei voucher P1/22_1-1 cytochrome c oxidase subunit I (COX1) gene, partial cds; mitochondrial
Sequence ID: OM984968.1 Length: 658
Range 1: 1 to 658

Score:1166 bits(631), Expect:0.0,
Identities:649/658(99%), Gaps:0/658(0%), Strand: Plus/Plus

```
Query  1     GACAATATACTTAATTTTTGGAGCATGATCCGGAATACTAGGATTAAGAATAAGAATATT  60
             ||||||||||||||||||||||||||||||||||||| ||||||||||||||||||||||
Sbjct  1     GACAATATACTTAATTTTTGGAGCATGATCCGGAATATTAGGATTAAGAATAAGAATATT  60

Query  61    AATCCGCATAGAATTAGGTCAACCAGGAACTTTAATTGGAAACGATCAAATTTATAATGT  120
             ||||||||||||||||||||||||||||||||||||||||||||||||||||||||||||
Sbjct  61    AATCCGCATAGAATTAGGTCAACCAGGAACTTTAATTGGAAACGATCAAATTTATAATGT  120

Query  121   AATTGTAACAGCACATGCATTTATTATAAtttttttATAGTTATACCAATTATAATCGG  180
             ||||||||||||||||||||||||||||||||||||||||||||||||||||||||||||
Sbjct  121   AATTGTAACAGCACATGCATTTATTATAATTTTTTTATAGTTATACCAATTATAATCGG  180

Query  181   AGGATTTGGAAATTGATTAGTTCCTATTATACTAGGAGCTCCAGATATAGCATTTCCACG  240
             |||||||||||||||||||||||||||||| |||||||||||||||||||||||||||||
Sbjct  181   AGGATTTGGAAATTGATTAGTTCCTATTATGCTAGGAGCTCCAGATATAGCATTTCCACG  240

Query  241   AATAAATAATATAAGATTTTGGTTACTTCCTCCTTCTCTATTTCTACTAATTAATTCTTC  300
             ||||||||||||||||||||||||||||||||||||||||||||||||||||||||||||
Sbjct  241   AATAAATAATATAAGATTTTGGTTACTTCCTCCTTCTCTATTTCTACTAATTAATTCTTC  300

Query  301   ATTAATCGAATCAGGAGCAGGTACAGGGTGAACAGTTTAcccccccTTATCATCAAATTT  360
             |||||||||||||||||||||||||||||||||||||||||| |||||||||||||||||
Sbjct  301   ATTAATCGAATCAGGAGCAGGTACAGGGTGAACAGTTTACCCTCCCTTATCATCAAATTT  360

Query  361   ATCACACTACGGACCATCAGTAGATTTAGCTATTTTTTCTCTGCATCTTGCAGGTGCTTC  420
             |||||||||||| ||||||||||||||||||||||| |||||||||||||| ||||||||
Sbjct  361   ATCACACTACGGGCCATCAGTAGATTTAGCTATTTTCTCTCTGCATCTTGCCGGTGCTTC  420

Query  421   TTCAATTTTAGGTGCAATCAATTTCATTACAACTATTGTAAATATACGATCCATTGGAAT  480
             |||||||||||||||||| |||||||||||||||||||||||||||||||||||||||||
```

```
Sbjct  421   TTCAATTTTAGGTGCAATTAATTTCATTACAACTATTGTAAATATACGATCCATTGGAAT  480

Query  481   AACAATAGAACGAATACCATTATTTGTATGATCTGTTTTAATTACTGCAATTTTATTATT  540
             ||||||||||||||||||||||||||||||||||||||||||||||||||||||||||||
Sbjct  481   AACAATAGAACGAATACCATTATTTGTATGATCTGTTTTAATTACTGCAATTTTATTATT  540

Query  541   ATTATCTTTACCAGTCTTAGCTGGCGCAATTACAATATTATTAACTGATCGAAACTTTAA  600
             |||||||||||||||||||||||| |||||||||||||||||||||||||||||||||||
Sbjct  541   ATTATCTTTACCAGTCTTAGCTGGTGCAATTACAATATTATTAACTGATCGAAACTTTAA  600

Query  601   TACATCATTCTTTGACCCTTCAGGAGGAGGTGACCCAATTCTATATCAACATTTATTT  658
             |||||||||||||||||||||||||||||||||||||||||| |||||||||||||||
Sbjct  601   TACATCATTCTTTGACCCTTCAGGAGGAGGTGACCCAATTCTGTATCAACATTTATTT  658
```

>Rhipicephalus sanguineus isolate DGF1 cytochrome oxidase subunit I (cox1) gene, partial cds; mitochondrial
Sequence ID: JX416295.1 Length: 802
Range 1: 1 to 637

Score:1166 bits(631), Expect:0.0,
Identities:635/637(99%), Gaps:0/637(0%), Strand: Plus/Plus

```
Query  22    AGCATGATCCGGAATACTAGGATTAAGAATAAGAATATTAATCCGCATAGAATTAGGTCA  81
             ||||||||||||||||||||||||||||||||||||||||||||||||||||||||||||
Sbjct  1     AGCATGATCCGGAATACTAGGATTAAGAATAAGAATATTAATCCGCATAGAATTAGGTCA  60

Query  82    ACCAGGAACTTTAATTGGAAACGATCAAATTTATAATGTAATTGTAACAGCACATGCATT  141
             ||||||||||||||||||||||||||||||||||||||||||||||||||||||||||||
Sbjct  61    ACCAGGAACTTTAATTGGAAACGATCAAATTTATAATGTAATTGTAACAGCACATGCATT  120

Query  142   TATTATAAttttttttATAGTTATACCAATTATAATCGGAGGATTTGGAAATTGATTAGT  201
             ||||||||||||||||||||||||||||||||||||||||||||||||||||||||||||
Sbjct  121   TATTATAATTTTTTTTATAGTTATACCAATTATAATCGGAGGATTTGGAAATTGATTAGT  180

Query  202   TCCTATTATACTAGGAGCTCCAGATATAGCATTTCCACGAATAAATAATATAAGATTTTG  261
             ||||||||||||||||||||||||||||||||||||||||||||||||||||||||||||
Sbjct  181   TCCTATTATACTAGGAGCTCCAGATATAGCATTTCCACGAATAAATAATATAAGATTTTG  240

Query  262   GTTACTTCCTCCTTCTCTATTTCTACTAATTAATTCTTCATTAATCGAATCAGGAGCAGG  321
             ||||||||||||||||||||||||||||||||||||||||||||||||||||||||||||
Sbjct  241   GTTACTTCCTCCTTCTCTATTTCTACTAATTAATTCTTCATTAATCGAATCAGGAGCAGG  300

Query  322   TACAGGGTGAACAGTTTAcccccccTTATCATCAAATTTATCACACTACGGACCATCAGT  381
             |||||||||||| |||||||||||||||||||||||||||||||||||||||||||||||
Sbjct  301   TACAGGGTGAACGGTTTACCCCCCCTTATCATCAAATTTATCACACTACGGACCATCAGT  360

Query  382   AGATTTAGCTATTTTTTCTCTGCATCTTGCAGGTGCTTCTTCAATTTTAGGTGCAATCAA  441
             ||||||||||||||||||||||||||||||||||||||||||||||||||||||||||||
Sbjct  361   AGATTTAGCTATTTTTTCTCTGCATCTTGCAGGTGCTTCTTCAATTTTAGGTGCAATCAA  420

Query  442   TTTCATTACAACTATTGTAAATATACGATCCATTGGAATAACAATAGAACGAATACCATT  501
```

```
            ||||||||||||||||||||||||||||||||||||||||||||||||||||||||||||
Sbjct  421  TTTCATTACAACTATTGTAAATATACGATCCATTGGAATAACAATAGAACGAATACCATT  480

Query  502  ATTTGTATGATCTGTTTTAATTACTGCAATTTTATTATTATTATCTTTACCAGTCTTAGC  561
            ||||||||||||||||||||||||||||||||||||||||||||||||||||||||||||
Sbjct  481  ATTTGTATGATCTGTTTTAATTACTGCAATTTTATTATTATTATCTTTACCAGTCTTAGC  540

Query  562  TGGCGCAATTACAATATTATTAACTGATCGAAACTTTAATACATCATTCTTTGACCCTTC  621
            ||||||||||||||||||||||||||||||||||||||||||||||||||||||||||||
Sbjct  541  TGGCGCAATTACAATATTATTAACTGATCGAAACTTTAATACATCATTCTTTGACCCTTC  600

Query  622  AGGAGGAGGTGACCCAATTCTATATCAACATTTATTT  658
            |||||||||||||||||||||||||| ||||||||||
Sbjct  601  AGGAGGAGGTGACCCAATTCTATATCGACATTTATTT  637
```

```
>Rhipicephalus sanguineus isolate FSM1 cytochrome oxidase subunit I (cox1) gene,
partial cds; mitochondrial
Sequence ID: JX416294.1 Length: 802
Range 1: 1 to 637

Score:1166 bits(631), Expect:0.0,
Identities:635/637(99%),  Gaps:0/637(0%), Strand: Plus/Plus

Query  22   AGCATGATCCGGAATACTAGGATTAAGAATAAGAATATTAATCCGCATAGAATTAGGTCA  81
            |||||||||||||||||||||||||||||||||||||||||||||||||| |||||||||
Sbjct  1    AGCATGATCCGGAATACTAGGATTAAGAATAAGAATATTAATCCGCATAGTATTAGGTCA  60

Query  82   ACCAGGAACTTTAATTGGAAACGATCAAATTTATAATGTAATTGTAACAGCACATGCATT  141
            ||||||||||||||||||||||||||||||||||||||||||||||||||||||||||||
Sbjct  61   ACCAGGAACTTTAATTGGAAACGATCAAATTTATAATGTAATTGTAACAGCACATGCATT  120

Query  142  TATTATAAtttttttATAGTTATACCAATTATAATCGGAGGATTTGGAAATTGATTAGT  201
            |||||||||||||||||||||||||| |||||||||||||||||||||||||||||||||
Sbjct  121  TATTATAATTTTTTTATAGTTATACCTATTATAATCGGAGGATTTGGAAATTGATTAGT  180

Query  202  TCCTATTATACTAGGAGCTCCAGATATAGCATTTCCACGAATAAATAATATAAGATTTTG  261
            ||||||||||||||||||||||||||||||||||||||||||||||||||||||||||||
Sbjct  181  TCCTATTATACTAGGAGCTCCAGATATAGCATTTCCACGAATAAATAATATAAGATTTTG  240

Query  262  GTTACTTCCTCCTTCTCTATTTCTACTAATTAATTCTTCATTAATCGAATCAGGAGCAGG  321
            ||||||||||||||||||||||||||||||||||||||||||||||||||||||||||||
Sbjct  241  GTTACTTCCTCCTTCTCTATTTCTACTAATTAATTCTTCATTAATCGAATCAGGAGCAGG  300

Query  322  TACAGGGTGAACAGTTTAcccccccTTATCATCAAATTTATCACACTACGGACCATCAGT  381
            ||||||||||||||||||||||||||||||||||||||||||||||||||||||||||||
Sbjct  301  TACAGGGTGAACAGTTTACCCCCCCTTATCATCAAATTTATCACACTACGGACCATCAGT  360

Query  382  AGATTTAGCTATTTTTTCTCTGCATCTTGCAGGTGCTTCTTCAATTTTAGGTGCAATCAA  441
            ||||||||||||||||||||||||||||||||||||||||||||||||||||||||||||
Sbjct  361  AGATTTAGCTATTTTTTCTCTGCATCTTGCAGGTGCTTCTTCAATTTTAGGTGCAATCAA  420
```

```
Query  442   TTTCATTACAACTATTGTAAATATACGATCCATTGGAATAACAATAGAACGAATACCATT  501
             ||||||||||||||||||||||||||||||||||||||||||||||||||||||||||||
Sbjct  421   TTTCATTACAACTATTGTAAATATACGATCCATTGGAATAACAATAGAACGAATACCATT  480

Query  502   ATTTGTATGATCTGTTTTAATTACTGCAATTTTATTATTATTATCTTTACCAGTCTTAGC  561
             ||||||||||||||||||||||||||||||||||||||||||||||||||||||||||||
Sbjct  481   ATTTGTATGATCTGTTTTAATTACTGCAATTTTATTATTATTATCTTTACCAGTCTTAGC  540

Query  562   TGGCGCAATTACAATATTATTAACTGATCGAAACTTTAATACATCATTCTTTGACCCTTC  621
             ||||||||||||||||||||||||||||||||||||||||||||||||||||||||||||
Sbjct  541   TGGCGCAATTACAATATTATTAACTGATCGAAACTTTAATACATCATTCTTTGACCCTTC  600

Query  622   AGGAGGAGGTGACCCAATTCTATATCAACATTTATTT  658
             |||||||||||||||||||||||||||||||||||||
Sbjct  601   AGGAGGAGGTGACCCAATTCTATATCAACATTTATTT  637
```

```
>Rhipicephalus sanguineus isolate sanguineus0926 cytochrome oxidase subunit 1
(COX1) gene, partial cds; mitochondrial
Sequence ID: MH513252.1 Length: 658
Range 1: 6 to 658

Score:1162 bits(629), Expect:0.0,
Identities:645/653(99%),  Gaps:0/653(0%), Strand: Plus/Plus

Query  1     GACAATATACTTAATTTTTGGAGCATGATCCGGAATACTAGGATTAAGAATAAGAATATT  60
             ||||||||||||||||||||||||||||||||||||||||||||||||||||||||||||
Sbjct  6     GACAATATACTTAATTTTTGGAGCATGATCCGGAATACTAGGATTAAGAATAAGAATATT  65

Query  61    AATCCGCATAGAATTAGGTCAACCAGGAACTTTAATTGGAAACGATCAAATTTATAATGT  120
             ||| ||||||||||||||||||||||||||||||||||||||||||||||||||||||||
Sbjct  66    AATTCGCATAGAATTAGGTCAACCAGGAACTTTAATTGGAAACGATCAAATTTATAATGT  125

Query  121   AATTGTAACAGCACATGCATTTATTATAAttttttttATAGTTATACCAATTATAATCGG  180
             ||||||||||||||||||||||||||||||||||||||||||||||||||||||||||||
Sbjct  126   AATTGTAACAGCACATGCATTTATTATAATTTTTTTTATAGTTATACCAATTATAATCGG  185

Query  181   AGGATTTGGAAATTGATTAGTTCCTATTATACTAGGAGCTCCAGATATAGCATTTCCACG  240
             ||||||||||||||||||||||||||||||||||||||||||||||||||||||||||||
Sbjct  186   AGGATTTGGAAATTGATTAGTTCCTATTATACTAGGAGCTCCAGATATAGCATTTCCACG  245

Query  241   AATAAATAATATAAGATTTTGGTTACTTCCTCCTTCTCTATTTCTACTAATTAATTCTTC  300
             |||||| |||||||||||||||||||||||||||||||||||||||||||||||||||||
Sbjct  246   AATAAACAATATAAGATTTTGGTTACTTCCTCCTTCTCTATTTCTACTAATTAATTCTTC  305

Query  301   ATTAATCGAATCAGGAGCAGGTACAGGGTGAACAGTTTAcccccccTTATCATCAAATTT  360
             |||||||||||||||||| |||||||||||||||||||||||||||||||||||||||||
Sbjct  306   ATTAATCGAATCAGGAGCGGGTACAGGGTGAACAGTTTACCCCCCCTTATCATCAAATTT  365

Query  361   ATCACACTACGGACCATCAGTAGATTTAGCTATTTTTTCTCTGCATCTTGCAGGTGCTTC  420
             ||||||||||||||||||||||||||||||||| ||||||||||||||||||||||||||
Sbjct  366   ATCACACTACGGACCATCAGTAGATTTAGCTATCTTTTCTCTGCATCTTGCAGGTGCTTC  425
```

```
Query  421   TTCAATTTTAGGTGCAATCAATTTCATTACAACTATTGTAAATATACGATCCATTGGAAT  480
             |||||||||||| |||||||||||||||||||||||||||||||||||||||||||||||
Sbjct  426   TTCAATTTTAGGCGCAATCAATTTCATTACAACTATTGTAAATATACGATCCATTGGAAT  485

Query  481   AACAATAGAACGAATACCATTATTTGTATGATCTGTTTTAATTACTGCAATTTTATTATT  540
             ||||||||||||||||||||||||||||||||||||||||||||||||||||||||||||
Sbjct  486   AACAATAGAACGAATACCATTATTTGTATGATCTGTTTTAATTACTGCAATTTTATTATT  545

Query  541   ATTATCTTTACCAGTCTTAGCTGGCGCAATTACAATATTATTAACTGATCGAAACTTTAA  600
             |||||||||||||||||||||||| |||||||||||||| ||||||||||||||||||||
Sbjct  546   ATTATCTTTACCAGTCTTAGCTGGTGCAATTACAATATTGTTAACTGATCGAAACTTTAA  605

Query  601   TACATCATTCTTTGACCCTTCAGGAGGAGGTGACCCAATTCTATATCAACATT  653
             |||||||||||||||||||||||||||||||||||||||||| ||||||||||
Sbjct  606   TACATCATTCTTTGACCCTTCAGGAGGAGGTGACCCAATTCTGTATCAACATT  658
```

>Rhipicephalus linnaei voucher P1/22_19-1 cytochrome c oxidase subunit I (COX1) gene, partial cds; mitochondrial
Sequence ID: OM984985.1 Length: 658
Range 1: 1 to 658

Score:1160 bits(628), Expect:0.0,
Identities:648/658(98%), Gaps:0/658(0%), Strand: Plus/Plus

```
Query  1     GACAATATACTTAATTTTTGGAGCATGATCCGGAATACTAGGATTAAGAATAAGAATATT  60
             ||||||||||||||||||||||||||||||||||||| ||||||||||||||||||||||
Sbjct  1     GACAATATACTTAATTTTTGGAGCATGATCCGGAATATTAGGATTAAGAATAAGAATATT  60

Query  61    AATCCGCATAGAATTAGGTCAACCAGGAACTTTAATTGGAAACGATCAAATTTATAATGT  120
             ||||||||||||||||||||||||||||||||||||||||||||||||||||||||||||
Sbjct  61    AATCCGCATAGAATTAGGTCAACCAGGAACTTTAATTGGAAACGATCAAATTTATAATGT  120

Query  121   AATTGTAACAGCACATGCATTTATTATAAttttttttATAGTTATACCAATTATAATCGG  180
             ||||||||||||||||||||||||||||||||||||||||||||||||||||||||||||
Sbjct  121   AATTGTAACAGCACATGCATTTATTATAATTTTTTTTATAGTTATACCAATTATAATCGG  180

Query  181   AGGATTTGGAAATTGATTAGTTCCTATTATACTAGGAGCTCCAGATATAGCATTTCCACG  240
             |||||||||||||||||||||||||||||| |||||||||||||||||||||||||||||
Sbjct  181   AGGATTTGGAAATTGATTAGTTCCTATTATGCTAGGAGCTCCAGATATAGCATTTCCACG  240

Query  241   AATAAATAATATAAGATTTTGGTTACTTCCTCCTTCTCTATTTCTACTAATTAATTCTTC  300
             ||||||||||||||||||||||||||||||||||||||||||||||||||||||||||||
Sbjct  241   AATAAATAATATAAGATTTTGGTTACTTCCTCCTTCTCTATTTCTACTAATTAATTCTTC  300

Query  301   ATTAATCGAATCAGGAGCAGGTACAGGGTGAACAGTTTAcccccccTTATCATCAAATTT  360
             |||||||||||||||||||||||||||||||||||||||||| |||||||||||||||||
Sbjct  301   ATTAATCGAATCAGGAGCAGGTACAGGGTGAACAGTTTACCCTCCCTTATCATCAAATTT  360

Query  361   ATCACACTACGGACCATCAGTAGATTTAGCTATTTTTTCTCTGCATCTTGCAGGTGCTTC  420
             |||||||||||| |||||||||||||||||||| || |||||||||||||| ||||||||
```

```
Sbjct  361  ATCACACTACGGGCCATCAGTAGATTTAGCTATCTTCTCTCTGCATCTTGCCGGTGCTTC  420

Query  421  TTCAATTTTAGGTGCAATCAATTTCATTACAACTATTGTAAATATACGATCCATTGGAAT  480
            |||||||||||||||||| |||||||||||||||||||||||||||||||||||||||||
Sbjct  421  TTCAATTTTAGGTGCAATTAATTTCATTACAACTATTGTAAATATACGATCCATTGGAAT  480

Query  481  AACAATAGAACGAATACCATTATTTGTATGATCTGTTTTAATTACTGCAATTTTATTATT  540
            ||||||||||||||||||||||||||||||||||||||||||||||||||||||||||||
Sbjct  481  AACAATAGAACGAATACCATTATTTGTATGATCTGTTTTAATTACTGCAATTTTATTATT  540

Query  541  ATTATCTTTACCAGTCTTAGCTGGCGCAATTACAATATTATTAACTGATCGAAACTTTAA  600
            |||||||||||||||||||||||| |||||||||||||||||||||||||||||||||||
Sbjct  541  ATTATCTTTACCAGTCTTAGCTGGTGCAATTACAATATTATTAACTGATCGAAACTTTAA  600

Query  601  TACATCATTCTTTGACCCTTCAGGAGGAGGTGACCCAATTCTATATCAACATTTATTT  658
            |||||||||||||||||||||||||||||||||||||||||| |||||||||||||||
Sbjct  601  TACATCATTCTTTGACCCTTCAGGAGGAGGTGACCCAATTCTGTATCAACATTTATTT  658
```

>Rhipicephalus linnaei voucher P1/22_14-1 cytochrome c oxidase subunit I (COX1) gene, partial cds; mitochondrial
Sequence ID: OM984978.1 Length: 658
Range 1: 1 to 658

Score:1160 bits(628), Expect:0.0,
Identities:648/658(98%), Gaps:0/658(0%), Strand: Plus/Plus

```
Query  1    GACAATATACTTAATTTTTGGAGCATGATCCGGAATACTAGGATTAAGAATAAGAATATT  60
            ||||||||||||||||||||||||||||||||||||| ||||||||||||||||||||||
Sbjct  1    GACAATATACTTAATTTTTGGAGCATGATCCGGAATATTAGGATTAAGAATAAGAATATT  60

Query  61   AATCCGCATAGAATTAGGTCAACCAGGAACTTTAATTGGAAACGATCAAATTTATAATGT  120
            ||||||||||||||||||||||||||||||||||||||||||||||||||||||||||||
Sbjct  61   AATCCGCATAGAATTAGGTCAACCAGGAACTTTAATTGGAAACGATCAAATTTATAATGT  120

Query  121  AATTGTAACAGCACATGCATTTATTATAAtttttttATAGTTATACCAATTATAATCGG  180
            ||||||||||||||||||||||||||||||||||||||||||||||||||||||||||||
Sbjct  121  AATTGTAACAGCACATGCATTTATTATAATTTTTTTATAGTTATACCAATTATAATCGG  180

Query  181  AGGATTTGGAAATTGATTAGTTCCTATTATACTAGGAGCTCCAGATATAGCATTTCCACG  240
            |||||||||||||||||||||||||||||| |||||||||||||||||||||||||||||
Sbjct  181  AGGATTTGGAAATTGATTAGTTCCTATTATGCTAGGAGCTCCAGATATAGCATTTCCACG  240

Query  241  AATAAATAATATAAGATTTTGGTTACTTCCTCCTTCTCTATTTCTACTAATTAATTCTTC  300
            ||||||||||||||||||||||||||||||||||||||||||||||||||||||||||||
Sbjct  241  AATAAATAATATAAGATTTTGGTTACTTCCTCCTTCTCTATTTCTACTAATTAATTCTTC  300

Query  301  ATTAATCGAATCAGGAGCAGGTACAGGGTGAACAGTTTAcccccccTTATCATCAAATTT  360
            ||||||||||||||||||||||||||||||||||||||||||| ||||||||||||||||
Sbjct  301  ATTAATCGAATCAGGAGCAGGTACAGGGTGAACAGTTTACCCTCCCTTATCATCAAATTT  360

Query  361  ATCACACTACGGACCATCAGTAGATTTAGCTATTTTTTCTCTGCATCTTGCAGGTGCTTC  420
```

```
                ||||||||||||| |||||||||||||||||||| || |||||||||||||| ||||||||
Sbjct  361   ATCACACTACGGGCCATCAGTAGATTTAGCTATCTTCTCTCTGCATCTTGCCGGTGCTTC  420

Query  421   TTCAATTTTAGGTGCAATCAATTTCATTACAACTATTGTAAATATACGATCCATTGGAAT  480
             |||||||||||||||||| |||||||||||||||||||||||||||||||||||||||||
Sbjct  421   TTCAATTTTAGGTGCAATTAATTTCATTACAACTATTGTAAATATACGATCCATTGGAAT  480

Query  481   AACAATAGAACGAATACCATTATTTGTATGATCTGTTTTAATTACTGCAATTTTATTATT  540
             ||||||||||||||||||||||||||||||||||||||||||||||||||||||||||||
Sbjct  481   AACAATAGAACGAATACCATTATTTGTATGATCTGTTTTAATTACTGCAATTTTATTATT  540

Query  541   ATTATCTTTACCAGTCTTAGCTGGCGCAATTACAATATTATTAACTGATCGAAACTTTAA  600
             |||||||||||||||||||||||| |||||||||||||||||||||||||||||||||||
Sbjct  541   ATTATCTTTACCAGTCTTAGCTGGTGCAATTACAATATTATTAACTGATCGAAACTTTAA  600

Query  601   TACATCATTCTTTGACCCTTCAGGAGGAGGTGACCCAATTCTATATCAACATTTATTT  658
             |||||||||||||||||||||||||||||||||||||||||| |||||||||||||||
Sbjct  601   TACATCATTCTTTGACCCTTCAGGAGGAGGTGACCCAATTCTGTATCAACATTTATTT  658
```

>Rhipicephalus linnaei voucher P1/22_13-1 cytochrome c oxidase subunit I (COX1) gene, partial cds; mitochondrial
Sequence ID: OM984977.1 Length: 658
Range 1: 1 to 658

Score:1160 bits(628), Expect:0.0,
Identities:648/658(98%), Gaps:0/658(0%), Strand: Plus/Plus

```
Query  1     GACAATATACTTAATTTTTGGAGCATGATCCGGAATACTAGGATTAAGAATAAGAATATT  60
             ||||||||||||||||||||||||||||||||||||| ||||||||||||||||||||||
Sbjct  1     GACAATATACTTAATTTTTGGAGCATGATCCGGAATATTAGGATTAAGAATAAGAATATT  60

Query  61    AATCCGCATAGAATTAGGTCAACCAGGAACTTTAATTGGAAACGATCAAATTTATAATGT  120
             ||||||||||||||||||||||||||||||||||||||||||||||||||||||||||||
Sbjct  61    AATCCGCATAGAATTAGGTCAACCAGGAACTTTAATTGGAAACGATCAAATTTATAATGT  120

Query  121   AATTGTAACAGCACATGCATTTATTATAAtttttttATAGTTATACCAATTATAATCGG  180
             ||||||||||||||||||||||||||||||||||||||||||||||||||||||||||||
Sbjct  121   AATTGTAACAGCACATGCATTTATTATAATTTTTTTATAGTTATACCAATTATAATCGG  180

Query  181   AGGATTTGGAAATTGATTAGTTCCTATTATACTAGGAGCTCCAGATATAGCATTTCCACG  240
             |||||||||||||||||||||||||||||| |||||||||||||||||||||||||||||
Sbjct  181   AGGATTTGGAAATTGATTAGTTCCTATTATGCTAGGAGCTCCAGATATAGCATTTCCACG  240

Query  241   AATAAATAATATAAGATTTTGGTTACTTCCTCCTTCTCTATTTCTACTAATTAATTCTTC  300
             ||||||||||||||||||||||||||||||||||||||||||||||||||||||||||||
Sbjct  241   AATAAATAATATAAGATTTTGGTTACTTCCTCCTTCTCTATTTCTACTAATTAATTCTTC  300

Query  301   ATTAATCGAATCAGGAGCAGGTACAGGGTGAACAGTTTAcccccccTTATCATCAAATTT  360
             |||||||||||||||||||||||||||||||||||||||||| |||||||||||||||||
Sbjct  301   ATTAATCGAATCAGGAGCAGGTACAGGGTGAACAGTTTACCCTCCCTTATCATCAAATTT  360
```

```
Query  361   ATCACACTACGGACCATCAGTAGATTTAGCTATTTTTTCTCTGCATCTTGCAGGTGCTTC  420
             |||||||||||| |||||||||||||||||||| || |||||||||||||| ||||||||
Sbjct  361   ATCACACTACGGGCCATCAGTAGATTTAGCTATCTTCTCTCTGCATCTTGCCGGTGCTTC  420

Query  421   TTCAATTTAGGTGCAATCAATTTCATTACAACTATTGTAAATATACGATCCATTGGAAT  480
             ||||||||||||||||| ||||||||||||||||||||||||||||||||||||||||||
Sbjct  421   TTCAATTTAGGTGCAATTAATTTCATTACAACTATTGTAAATATACGATCCATTGGAAT  480

Query  481   AACAATAGAACGAATACCATTATTTGTATGATCTGTTTTAATTACTGCAATTTTATTATT  540
             ||||||||||||||||||||||||||||||||||||||||||||||||||||||||||||
Sbjct  481   AACAATAGAACGAATACCATTATTTGTATGATCTGTTTTAATTACTGCAATTTTATTATT  540

Query  541   ATTATCTTTACCAGTCTTAGCTGGCGCAATTACAATATTATTAACTGATCGAAACTTTAA  600
             |||||||||||||||||||||||| |||||||||||||||||||||||||||||||||||
Sbjct  541   ATTATCTTTACCAGTCTTAGCTGGTGCAATTACAATATTATTAACTGATCGAAACTTTAA  600

Query  601   TACATCATTCTTTGACCCTTCAGGAGGAGGTGACCCAATTCTATATCAACATTTATTT  658
             |||||||||||||||||||||||||||||||||||||||||| |||||||||||||||
Sbjct  601   TACATCATTCTTTGACCCTTCAGGAGGAGGTGACCCAATTCTGTATCAACATTTATTT  658
```

>Rhipicephalus linnaei voucher P1/22_10-1 cytochrome c oxidase subunit I (COX1) gene, partial cds; mitochondrial
Sequence ID: OM984975.1 Length: 658
Range 1: 1 to 658

Score:1160 bits(628), Expect:0.0,
Identities:648/658(98%), Gaps:0/658(0%), Strand: Plus/Plus

```
Query  1     GACAATATACTTAATTTTTGGAGCATGATCCGGAATACTAGGATTAAGAATAAGAATATT  60
             ||||||||||||||||||||||||||||||||||||| ||||||||||||||||||||||
Sbjct  1     GACAATATACTTAATTTTTGGAGCATGATCCGGAATATTAGGATTAAGAATAAGAATATT  60

Query  61    AATCCGCATAGAATTAGGTCAACCAGGAACTTTAATTGGAAACGATCAAATTTATAATGT  120
             ||||||||||||||||||||||||||||||||||||||||||||||||||||||||||||
Sbjct  61    AATCCGCATAGAATTAGGTCAACCAGGAACTTTAATTGGAAACGATCAAATTTATAATGT  120

Query  121   AATTGTAACAGCACATGCATTTATTATAAtttttttATAGTTATACCAATTATAATCGG  180
             ||||||||||||||||||||||||||||||||||||||||||||||||||||||||||||
Sbjct  121   AATTGTAACAGCACATGCATTTATTATAATTTTTTTTATAGTTATACCAATTATAATCGG  180

Query  181   AGGATTTGGAAATTGATTAGTTCCTATTATACTAGGAGCTCCAGATATAGCATTTCCACG  240
             |||||||||||||||||||||||||||||| |||||||||||||||||||||||||||||
Sbjct  181   AGGATTTGGAAATTGATTAGTTCCTATTATGCTAGGAGCTCCAGATATAGCATTTCCACG  240

Query  241   AATAAATAATATAAGATTTTGGTTACTTCCTCCTTCTCTATTTCTACTAATTAATTCTTC  300
             ||||||||||||||||||||||||||||||||||||||||||||||||||||||||||||
Sbjct  241   AATAAATAATATAAGATTTTGGTTACTTCCTCCTTCTCTATTTCTACTAATTAATTCTTC  300

Query  301   ATTAATCGAATCAGGAGCAGGTACAGGGTGAACAGTTTAcccccccTTATCATCAAATTT  360
             |||||||||||||||||||||||||||||||||||||||||| |||||||||||||||||
Sbjct  301   ATTAATCGAATCAGGAGCAGGTACAGGGTGAACAGTTTACCCTCCCTTATCATCAAATTT  360
```

```
Query  361  ATCACACTACGGACCATCAGTAGATTTAGCTATTTTTTCTCTGCATCTTGCAGGTGCTTC  420
            |||||||||||| |||||||||||||||||||| || |||||||||||||| ||||||||
Sbjct  361  ATCACACTACGGGCCATCAGTAGATTTAGCTATCTTCTCTCTGCATCTTGCCGGTGCTTC  420

Query  421  TTCAATTTTAGGTGCAATCAATTTCATTACAACTATTGTAAATATACGATCCATTGGAAT  480
            |||||||||||||||||| |||||||||||||||||||||||||||||||||||||||||
Sbjct  421  TTCAATTTTAGGTGCAATTAATTTCATTACAACTATTGTAAATATACGATCCATTGGAAT  480

Query  481  AACAATAGAACGAATACCATTATTTGTATGATCTGTTTTAATTACTGCAATTTTATTATT  540
            ||||||||||||||||||||||||||||||||||||||||||||||||||||||||||||
Sbjct  481  AACAATAGAACGAATACCATTATTTGTATGATCTGTTTTAATTACTGCAATTTTATTATT  540

Query  541  ATTATCTTTACCAGTCTTAGCTGGCGCAATTACAATATTATTAACTGATCGAAACTTTAA  600
            |||||||||||||||||||||||| |||||||||||||||||||||||||||||||||||
Sbjct  541  ATTATCTTTACCAGTCTTAGCTGGTGCAATTACAATATTATTAACTGATCGAAACTTTAA  600

Query  601  TACATCATTCTTTGACCCTTCAGGAGGAGGTGACCCAATTCTATATCAACATTTATTT  658
            |||||||||||||||||||||||||||||||||||||||||| |||||||||||||||
Sbjct  601  TACATCATTCTTTGACCCTTCAGGAGGAGGTGACCCAATTCTGTATCAACATTTATTT  658
```

```
>Rhipicephalus linnaei voucher P1/22_5-1 cytochrome c oxidase subunit I (COX1)
gene, partial cds; mitochondrial
Sequence ID: OM984971.1 Length: 658
Range 1: 1 to 658

Score:1160 bits(628), Expect:0.0,
Identities:648/658(98%),  Gaps:0/658(0%), Strand: Plus/Plus

Query  1    GACAATATACTTAATTTTTGGAGCATGATCCGGAATACTAGGATTAAGAATAAGAATATT  60
            ||||||||||||||||||||||||||||||||||||| ||||||||||||||||||||||
Sbjct  1    GACAATATACTTAATTTTTGGAGCATGATCCGGAATATTAGGATTAAGAATAAGAATATT  60

Query  61   AATCCGCATAGAATTAGGTCAACCAGGAACTTTAATTGGAAACGATCAAATTTATAATGT  120
            ||||||||||||||||||||||||||||||||||||||||||||||||||||||||||||
Sbjct  61   AATCCGCATAGAATTAGGTCAACCAGGAACTTTAATTGGAAACGATCAAATTTATAATGT  120

Query  121  AATTGTAACAGCACATGCATTTATTATAAttttttttATAGTTATACCAATTATAATCGG  180
            ||||||||||||||||||||||||||||||||||||||||||||||||||||||||||||
Sbjct  121  AATTGTAACAGCACATGCATTTATTATAATTTTTTTTATAGTTATACCAATTATAATCGG  180

Query  181  AGGATTTGGAAATTGATTAGTTCCTATTATACTAGGAGCTCCAGATATAGCATTTCCACG  240
            |||||||||||||||||||||||||||||| |||||||||||||||||||||||||||||
Sbjct  181  AGGATTTGGAAATTGATTAGTTCCTATTATGCTAGGAGCTCCAGATATAGCATTTCCACG  240

Query  241  AATAAATAATATAAGATTTTGGTTACTTCCTCCTTCTCTATTTCTACTAATTAATTCTTC  300
            ||||||||||||||||||||||||||||||||||||||||||||||||||||||||||||
Sbjct  241  AATAAATAATATAAGATTTTGGTTACTTCCTCCTTCTCTATTTCTACTAATTAATTCTTC  300

Query  301  ATTAATCGAATCAGGAGCAGGTACAGGGTGAACAGTTTAcccccccTTATCATCAAATTT  360
            ||||||||||||||||||||||||||||||||||||||||||||||||||||||||||||
```

```
Sbjct  301   ATTAATCGAATCAGGAGCAGGTACAGGGTGAACAGTTTACCCCCCCTTATCATCAAATTT  360

Query  361   ATCACACTACGGACCATCAGTAGATTTAGCTATTTTTTCTCTGCATCTTGCAGGTGCTTC  420
             |||||||||||| |||||||||||||||||||| ||||||||||||||||| ||||||||
Sbjct  361   ATCACACTACGGGCCATCAGTAGATTTAGCTATCTTTTCTCTGCATCTTGCCGGTGCTTC  420

Query  421   TTCAATTTTAGGTGCAATCAATTTCATTACAACTATTGTAAATATACGATCCATTGGAAT  480
             |||||||||||||||||| ||||||||||||||||| |||||||||||||||||||| ||
Sbjct  421   TTCAATTTTAGGTGCAATTAATTTCATTACAACTATGGTAAATATACGATCCATTGGGAT  480

Query  481   AACAATAGAACGAATACCATTATTTGTATGATCTGTTTTAATTACTGCAATTTTATTATT  540
             ||||||||||||||||||||||||||||||||||||||||||||||||||||||||||||
Sbjct  481   AACAATAGAACGAATACCATTATTTGTATGATCTGTTTTAATTACTGCAATTTTATTATT  540

Query  541   ATTATCTTTACCAGTCTTAGCTGGCGCAATTACAATATTATTAACTGATCGAAACTTTAA  600
             ||||||||||||||||||||||||||||||||||||||||||||||||||||||||||||
Sbjct  541   ATTATCTTTACCAGTCTTAGCTGGCGCAATTACAATATTATTAACTGATCGAAACTTTAA  600

Query  601   TACATCATTCTTTGACCCTTCAGGAGGAGGTGACCCAATTCTATATCAACATTTATTT  658
             ||||||||||||||||||||||||||| |||||||||||||| |||||||||||||||
Sbjct  601   TACATCATTCTTTGACCCTTCAGGAGGGGGTGACCCAATTCTGTATCAACATTTATTT  658
```

>Rhipicephalus linnaei voucher P1/22_4-1 cytochrome c oxidase subunit I (COX1) gene, partial cds; mitochondrial
Sequence ID: OM984970.1 Length: 658
Range 1: 1 to 658

Score:1160 bits(628), Expect:0.0,
Identities:648/658(98%), Gaps:0/658(0%), Strand: Plus/Plus

```
Query  1     GACAATATACTTAATTTTTGGAGCATGATCCGGAATACTAGGATTAAGAATAAGAATATT  60
             ||||||||||||||||||||||||||||||||||||| ||||||||||||||||||||||
Sbjct  1     GACAATATACTTAATTTTTGGAGCATGATCCGGAATATTAGGATTAAGAATAAGAATATT  60

Query  61    AATCCGCATAGAATTAGGTCAACCAGGAACTTTAATTGGAAACGATCAAATTTATAATGT  120
             ||||||||||||||||||||||||||||||||||||||||||||||||||||||||||||
Sbjct  61    AATCCGCATAGAATTAGGTCAACCAGGAACTTTAATTGGAAACGATCAAATTTATAATGT  120

Query  121   AATTGTAACAGCACATGCATTTATTATAAttttttttATAGTTATACCAATTATAATCGG  180
             ||||||||||||||||||||||||||||||||||||||||||||||||||||||||||||
Sbjct  121   AATTGTAACAGCACATGCATTTATTATAATTTTTTTTATAGTTATACCAATTATAATCGG  180

Query  181   AGGATTTGGAAATTGATTAGTTCCTATTATACTAGGAGCTCCAGATATAGCATTTCCACG  240
             |||||||||||||||||||||||||||||| |||||||||||||||||||||||||||||
Sbjct  181   AGGATTTGGAAATTGATTAGTTCCTATTATGCTAGGAGCTCCAGATATAGCATTTCCACG  240

Query  241   AATAAATAATATAAGATTTTGGTTACTTCCTCCTTCTCTATTTCTACTAATTAATTCTTC  300
             ||||||||||||||||||||||||||||||||||||||||||||||||||||||||||||
Sbjct  241   AATAAATAATATAAGATTTTGGTTACTTCCTCCTTCTCTATTTCTACTAATTAATTCTTC  300

Query  301   ATTAATCGAATCAGGAGCAGGTACAGGGTGAACAGTTTAcccccccTTATCATCAAATTT  360
```

```
                ||||||||||||||||||||||||||||||||||||||||||| |||||||||||||||||
Sbjct  301   ATTAATCGAATCAGGAGCAGGTACAGGGTGAACAGTTTACCCTCCCTTATCATCAAATTT  360

Query  361   ATCACACTACGGACCATCAGTAGATTTAGCTATTTTTTCTCTGCATCTTGCAGGTGCTTC  420
             |||||||||||| |||||||||||||||||||| || |||||||||||||| ||||||||
Sbjct  361   ATCACACTACGGGCCATCAGTAGATTTAGCTATCTTCTCTCTGCATCTTGCCGGTGCTTC  420

Query  421   TTCAATTTTAGGTGCAATCAATTTCATTACAACTATTGTAAATATACGATCCATTGGAAT  480
             |||||||||||||||||| ||||||||||||||||||||||||||||||||||||||||||
Sbjct  421   TTCAATTTTAGGTGCAATTAATTTCATTACAACTATTGTAAATATACGATCCATTGGAAT  480

Query  481   AACAATAGAACGAATACCATTATTTGTATGATCTGTTTTAATTACTGCAATTTTATTATT  540
             ||||||||||||||||||||||||||||||||||||||||||||||||||||||||||||
Sbjct  481   AACAATAGAACGAATACCATTATTTGTATGATCTGTTTTAATTACTGCAATTTTATTATT  540

Query  541   ATTATCTTTACCAGTCTTAGCTGGCGCAATTACAATATTATTAACTGATCGAAACTTTAA  600
             |||||||||||||||||||||||| |||||||||||||||||||||||||||||||||||
Sbjct  541   ATTATCTTTACCAGTCTTAGCTGGTGCAATTACAATATTATTAACTGATCGAAACTTTAA  600

Query  601   TACATCATTCTTTGACCCTTCAGGAGGAGGTGACCCAATTCTATATCAACATTTATTT  658
             |||||||||||||||||||||||||||||||||||||||||| |||||||||||||||
Sbjct  601   TACATCATTCTTTGACCCTTCAGGAGGAGGTGACCCAATTCTGTATCAACATTTATTT  658
```

>Rhipicephalus linnaei voucher P1/22_3-1 cytochrome c oxidase subunit I (COX1) gene, partial cds; mitochondrial
Sequence ID: OM984969.1 Length: 658
Range 1: 1 to 658

Score:1160 bits(628), Expect:0.0,
Identities:648/658(98%), Gaps:0/658(0%), Strand: Plus/Plus

```
Query  1     GACAATATACTTAATTTTTGGAGCATGATCCGGAATACTAGGATTAAGAATAAGAATATT  60
             ||||||||||||||||||||||||||||||||||||| ||||||||||||||||||||||
Sbjct  1     GACAATATACTTAATTTTTGGAGCATGATCCGGAATATTAGGATTAAGAATAAGAATATT  60

Query  61    AATCCGCATAGAATTAGGTCAACCAGGAACTTTAATTGGAAACGATCAAATTTATAATGT  120
             ||||||||||||||||||||||||||||||||||||||||||||||||||||||||||||
Sbjct  61    AATCCGCATAGAATTAGGTCAACCAGGAACTTTAATTGGAAACGATCAAATTTATAATGT  120

Query  121   AATTGTAACAGCACATGCATTTATTATAAttttttttATAGTTATACCAATTATAATCGG  180
             ||||||||||||||||||||||||||||||||||||||||||||||||||||||||||||
Sbjct  121   AATTGTAACAGCACATGCATTTATTATAATTTTTTTTATAGTTATACCAATTATAATCGG  180

Query  181   AGGATTTGGAAATTGATTAGTTCCTATTATACTAGGAGCTCCAGATATAGCATTTCCACG  240
             |||||||||||||||||||||||||||||| |||||||||||||||||||||||||||||
Sbjct  181   AGGATTTGGAAATTGATTAGTTCCTATTATGCTAGGAGCTCCAGATATAGCATTTCCACG  240

Query  241   AATAAATAATATAAGATTTTGGTTACTTCCTCCTTCTCTATTTCTACTAATTAATTCTTC  300
             ||||||||||||||||||||||||||||||||||||||||||||||||||||||||||||
Sbjct  241   AATAAATAATATAAGATTTTGGTTACTTCCTCCTTCTCTATTTCTACTAATTAATTCTTC  300
```

```
Query  301  ATTAATCGAATCAGGAGCAGGTACAGGGTGAACAGTTTAcccccccTTATCATCAAATTT  360
            |||||||||||||||||||||||||||||||||||||||||| |||||||||||||||||
Sbjct  301  ATTAATCGAATCAGGAGCAGGTACAGGGTGAACAGTTTACCCTCCCTTATCATCAAATTT  360

Query  361  ATCACACTACGGACCATCAGTAGATTTAGCTATTTTTTCTCTGCATCTTGCAGGTGCTTC  420
            |||||||||||| |||||||||||||||||||| || |||||||||||||| ||||||||
Sbjct  361  ATCACACTACGGGCCATCAGTAGATTTAGCTATCTTCTCTCTGCATCTTGCCGGTGCTTC  420

Query  421  TTCAATTTTAGGTGCAATCAATTTCATTACAACTATTGTAAATATACGATCCATTGGAAT  480
            |||||||||||||||||| |||||||||||||||||||||||||||||||||||||||||
Sbjct  421  TTCAATTTTAGGTGCAATTAATTTCATTACAACTATTGTAAATATACGATCCATTGGAAT  480

Query  481  AACAATAGAACGAATACCATTATTTGTATGATCTGTTTTAATTACTGCAATTTTATTATT  540
            ||||||||||||||||||||||||||||||||||||||||||||||||||||||||||||
Sbjct  481  AACAATAGAACGAATACCATTATTTGTATGATCTGTTTTAATTACTGCAATTTTATTATT  540

Query  541  ATTATCTTTACCAGTCTTAGCTGGCGCAATTACAATATTATTAACTGATCGAAACTTTAA  600
            |||||||||||||||||||||||| |||||||||||||||||||||||||||||||||||
Sbjct  541  ATTATCTTTACCAGTCTTAGCTGGTGCAATTACAATATTATTAACTGATCGAAACTTTAA  600

Query  601  TACATCATTCTTTGACCCTTCAGGAGGAGGTGACCCAATTCTATATCAACATTTATTT  658
            |||||||||||||||||||||||||||||||||||||||||| |||||||||||||||
Sbjct  601  TACATCATTCTTTGACCCTTCAGGAGGAGGTGACCCAATTCTGTATCAACATTTATTT  658
```

>Rhipicephalus sanguineus isolate GHF1 cytochrome oxidase subunit I (cox1) gene, partial cds; mitochondrial
Sequence ID: JX416301.1 Length: 802
Range 1: 1 to 637

Score:1160 bits(628), Expect:0.0,
Identities:634/637(99%), Gaps:0/637(0%), Strand: Plus/Plus

```
Query  22   AGCATGATCCGGAATACTAGGATTAAGAATAAGAATATTAATCCGCATAGAATTAGGTCA  81
            ||||||||||||||||||||||||||||||||||||||||||||||||||||||||||||
Sbjct  1    AGCATGATCCGGAATACTAGGATTAAGAATAAGAATATTAATCCGCATAGAATTAGGTCA  60

Query  82   ACCAGGAACTTTAATTGGAAACGATCAAATTTATAATGTAATTGTAACAGCACATGCATT  141
            ||||||||||||||||||||||||||||||||||||||||||||||||||||||||||||
Sbjct  61   ACCAGGAACTTTAATTGGAAACGATCAAATTTATAATGTAATTGTAACAGCACATGCATT  120

Query  142  TATTATAAtttttttATAGTTATACCAATTATAATCGGAGGATTTGGAAATTGATTAGT  201
            ||||||||||||||||||||||||||||||||||||||||||||||||||||||||||||
Sbjct  121  TATTATAATTTTTTTTATAGTTATACCAATTATAATCGGAGGATTTGGAAATTGATTAGT  180

Query  202  TCCTATTATACTAGGAGCTCCAGATATAGCATTTCCACGAATAAATAATATAAGATTTTG  261
            ||||||||||||||||||||||||||||||||||||||||||||||||||||||||||||
Sbjct  181  TCCTATTATACTAGGAGCTCCAGATATAGCATTTCCACGAATAAATAATATAAGATTTTG  240

Query  262  GTTACTTCCTCCTTCTCTATTTCTACTAATTAATTCTTCATTAATCGAATCAGGAGCAGG  321
            ||||||||||||||||||| ||||||||||||||||||||||||||||||||||||||||
Sbjct  241  GTTACTTCCTCCTTCTCTAATTCTACTAATTAATTCTTCATTAATCGAATCAGGAGCAGG  300
```

```
Query  322  TACAGGGTGAACAGTTTAcccccccTTATCATCAAATTTATCACACTACGGACCATCAGT  381
            ||||||||||||||||||||||||||||||||||||||||||||||||||||||||||||
Sbjct  301  TACAGGGTGAACAGTTTACCCCCCCTTATCATCAAATTTATCACACTACGGACCATCAGT  360

Query  382  AGATTTAGCTATTTTTTCTCTGCATCTTGCAGGTGCTTCTTCAATTTTAGGTGCAATCAA  441
            ||||||||||||||||||||||||||||||||||||||||||||||||||||||||||||
Sbjct  361  AGATTTAGCTATTTTTTCTCTGCATCTTGCAGGTGCTTCTTCAATTTTAGGTGCAATCAA  420

Query  442  TTTCATTACAACTATTGTAAATATACGATCCATTGGAATAACAATAGAACGAATACCATT  501
            ||||||||||||||||||||||||||||||||||||||||||||||||||||||||||||
Sbjct  421  TTTCATTACAACTATTGTAAATATACGATCCATTGGAATAACAATAGAACGAATACCATT  480

Query  502  ATTTGTATGATCTGTTTTAATTACTGCAATTTTATTATTATTATCTTTACCAGTCTTAGC  561
            ||||||||||||||||||||||||||||||||||||||||||||||||||||||||||||
Sbjct  481  ATTTGTATGATCTGTTTTAATTACTGCAATTTTATTATTATTATCTTTACCAGTCTTAGC  540

Query  562  TGGCGCAATTACAATATTATTAACTGATCGAAACTTTAATACATCATTCTTTGACCCTTC  621
            |||||| ||||||||||||||||||||||||||||||||||||| |||||||||||||||
Sbjct  541  TGGCGCTATTACAATATTATTAACTGATCGAAACTTTAATACATTATTCTTTGACCCTTC  600

Query  622  AGGAGGAGGTGACCCAATTCTATATCAACATTTATTT  658
            |||||||||||||||||||||||||||||||||||||
Sbjct  601  AGGAGGAGGTGACCCAATTCTATATCAACATTTATTT  637
```

>Rhipicephalus sanguineus isolate Tropical lineage RJ cytochrome c oxidase subunit I (COX1) gene, partial cds; mitochondrial
Sequence ID: MT010523.1 Length: 642
Range 1: 1 to 631

Score:1155 bits(625), Expect:0.0,
Identities:629/631(99%), Gaps:0/631(0%), Strand: Plus/Plus

```
Query  28   ATCCGGAATACTAGGATTAAGAATAAGAATATTAATCCGCATAGAATTAGGTCAACCAGG  87
            ||||||||||||||||||||||||||||||||||||||||||||||||||||||||||||
Sbjct  1    ATCCGGAATACTAGGATTAAGAATAAGAATATTAATCCGCATAGAATTAGGTCAACCAGG  60

Query  88   AACTTTAATTGGAAACGATCAAATTTATAATGTAATTGTAACAGCACATGCATTTATTAT  147
            ||||||||||||||||||||||||||||||||||||||||||||||||||||||||||||
Sbjct  61   AACTTTAATTGGAAACGATCAAATTTATAATGTAATTGTAACAGCACATGCATTTATTAT  120

Query  148  AAttttttttATAGTTATACCAATTATAATCGGAGGATTTGGAAATTGATTAGTTCCTAT  207
            ||||||||||||||||||||||||||||||||||||||||||||||||||||||||||||
Sbjct  121  AATTTTTTTTATAGTTATACCAATTATAATCGGAGGATTTGGAAATTGATTAGTTCCTAT  180

Query  208  TATACTAGGAGCTCCAGATATAGCATTTCCACGAATAAATAATATAAGATTTTGGTTACT  267
            ||||||||||||||||||||||||||||||||||||||||||||||||||||||||||||
Sbjct  181  TATACTAGGAGCTCCAGATATAGCATTTCCACGAATAAATAATATAAGATTTTGGTTACT  240

Query  268  TCCTCCTTCTCTATTTCTACTAATTAATTCTTCATTAATCGAATCAGGAGCAGGTACAGG  327
            ||||||||||||||||||||||||||||||||||||||||||||||||||||||||||||
```

```
Sbjct  241  TCCTCCTTCTCTATTTCTACTAATTAATTCTTCATTAATCGAATCAGGAGCAGGTACAGG  300

Query  328  GTGAACAGTTTAcccccccTTATCATCAAATTTATCACACTACGGACCATCAGTAGATTT  387
            ||||||||||||||||||||||||||||||||||||||||||||||||||||||||||||
Sbjct  301  GTGAACAGTTTACCCCCCCTTATCATCAAATTTATCACACTACGGACCATCAGTAGATTT  360

Query  388  AGCTATTTTTTCTCTGCATCTTGCAGGTGCTTCTTCAATTTTAGGTGCAATCAATTTCAT  447
            ||||||||||||||| ||||||||||||||||||||||||||||||||||||||||||||
Sbjct  361  AGCTATTTTTTCTCTACATCTTGCAGGTGCTTCTTCAATTTTAGGTGCAATCAATTTCAT  420

Query  448  TACAACTATTGTAAATATACGATCCATTGGAATAACAATAGAACGAATACCATTATTTGT  507
            ||||||||||||||||||||||||||||||||||||||||||||||||||||||||||||
Sbjct  421  TACAACTATTGTAAATATACGATCCATTGGAATAACAATAGAACGAATACCATTATTTGT  480

Query  508  ATGATCTGTTTTAATTACTGCAATTTTATTATTATTATCTTTACCAGTCTTAGCTGGCGC  567
            ||||||||||||||||||||||||||||||||||||||||||||||||||||||||||||
Sbjct  481  ATGATCTGTTTTAATTACTGCAATTTTATTATTATTATCTTTACCAGTCTTAGCTGGCGC  540

Query  568  AATTACAATATTATTAACTGATCGAAACTTTAATACATCATTCTTTGACCCTTCAGGAGG  627
            ||||||||||||||||||||||||||||||||||||||||||||||||||||||||||||
Sbjct  541  AATTACAATATTATTAACTGATCGAAACTTTAATACATCATTCTTTGACCCTTCAGGAGG  600

Query  628  AGGTGACCCAATTCTATATCAACATTTATTT  658
             ||||||||||||||||||||||||||||||
Sbjct  601  GGGTGACCCAATTCTATATCAACATTTATTT  631
```

>Rhipicephalus linnaei voucher P1/22_21-1 cytochrome c oxidase subunit I (COX1) gene, partial cds; mitochondrial
Sequence ID: OM984988.1 Length: 658
Range 1: 1 to 658

Score:1155 bits(625), Expect:0.0,
Identities:647/658(98%), Gaps:0/658(0%), Strand: Plus/Plus

```
Query  1    GACAATATACTTAATTTTTGGAGCATGATCCGGAATACTAGGATTAAGAATAAGAATATT  60
            ||||||||||||||||||||||||||||||||||||| ||||||||||||||||||||||
Sbjct  1    GACAATATACTTAATTTTTGGAGCATGATCCGGAATATTAGGATTAAGAATAAGAATATT  60

Query  61   AATCCGCATAGAATTAGGTCAACCAGGAACTTTAATTGGAAACGATCAAATTTATAATGT  120
            ||||||||||||||||||||||||||||||||||||||||||||||||||||||||||||
Sbjct  61   AATCCGCATAGAATTAGGTCAACCAGGAACTTTAATTGGAAACGATCAAATTTATAATGT  120

Query  121  AATTGTAACAGCACATGCATTTATTATAAttttttttATAGTTATACCAATTATAATCGG  180
            ||||||||||||||||||||||||||||||||||||||||||||||||||||||||||||
Sbjct  121  AATTGTAACAGCACATGCATTTATTATAATTTTTTTTATAGTTATACCAATTATAATCGG  180

Query  181  AGGATTTGGAAATTGATTAGTTCCTATTATACTAGGAGCTCCAGATATAGCATTTCCACG  240
            |||||||||||||||||||||||||||||| |||||||||||||||||||||||||||||
Sbjct  181  AGGATTTGGAAATTGATTAGTTCCTATTATGCTAGGAGCTCCAGATATAGCATTTCCACG  240

Query  241  AATAAATAATATAAGATTTTGGTTACTTCCTCCTTCTCTATTTCTACTAATTAATTCTTC  300
```

```
               ||||||||||||||||||||||||||||||||||||||||||||||||||||||||||||
Sbjct  241    AATAAATAATATAAGATTTTGGTTACTTCCTCCTTCTCTATTTCTACTAATTAATTCTTC  300

Query  301    ATTAATCGAATCAGGAGCAGGTACAGGGTGAACAGTTTAccccccTTATCATCAAATTT  360
               |||||||||||||||||||||||||||||||||||||||||| |||||||||||||||||
Sbjct  301    ATTAATCGAATCAGGAGCAGGTACAGGGTGAACAGTTTACCCTCCCTTATCATCAAATTT  360

Query  361    ATCACACTACGGACCATCAGTAGATTTAGCTATTTTTTCTCTGCATCTTGCAGGTGCTTC  420
               |||||||||||| |||||||||||||||||||| || |||||||||||||| ||||||||
Sbjct  361    ATCACACTACGGGCCATCAGTAGATTTAGCTATCTTCTCTCTGCATCTTGCCGGTGCTTC  420

Query  421    TTCAATTTTAGGTGCAATCAATTTCATTACAACTATTGTAAATATACGATCCATTGGAAT  480
               |||||||||||||||||| |||||||||||||||||||||||||||||||||||||||||
Sbjct  421    TTCAATTTTAGGTGCAATTAATTTCATTACAACTATTGTAAATATACGATCCATTGGAAT  480

Query  481    AACAATAGAACGAATACCATTATTTGTATGATCTGTTTTAATTACTGCAATTTTATTATT  540
               ||||||||||||||||||||||||||||||||||||||||||||||||||||||||||||
Sbjct  481    AACAATAGAACGAATACCATTATTTGTATGATCTGTTTTAATTACTGCAATTTTATTATT  540

Query  541    ATTATCTTTACCAGTCTTAGCTGGCGCAATTACAATATTATTAACTGATCGAAACTTTAA  600
               |||||||||||||||||||||||| |||||||||||||||||||||||||||||||||||
Sbjct  541    ATTATCTTTACCAGTCTTAGCTGGTGCAATTACAATATTATTAACTGATCGAAACTTTAA  600

Query  601    TACATCATTCTTTGACCCTTCAGGAGGAGGTGACCCAATTCTATATCAACATTTATTT  658
               ||||||||| |||||||||||||||||||||||||||||||| |||||||||||||||
Sbjct  601    TACATCATTTTTTGACCCTTCAGGAGGAGGTGACCCAATTCTGTATCAACATTTATTT  658
```

>Rhipicephalus linnaei voucher P1/22_12-1 cytochrome c oxidase subunit I (COX1) gene, partial cds; mitochondrial
Sequence ID: OM984976.1 Length: 658
Range 1: 1 to 658

Score:1155 bits(625), Expect:0.0,
Identities:647/658(98%), Gaps:0/658(0%), Strand: Plus/Plus

```
Query  1      GACAATATACTTAATTTTTGGAGCATGATCCGGAATACTAGGATTAAGAATAAGAATATT  60
               ||||||||||||||||||||||||||||||||||||| ||||||||||||||||||||||
Sbjct  1      GACAATATACTTAATTTTTGGAGCATGATCCGGAATATTAGGATTAAGAATAAGAATATT  60

Query  61     AATCCGCATAGAATTAGGTCAACCAGGAACTTTAATTGGAAACGATCAAATTTATAATGT  120
               ||||||||||||||||||||||||||||||||||||||||||||||||||||||||||||
Sbjct  61     AATCCGCATAGAATTAGGTCAACCAGGAACTTTAATTGGAAACGATCAAATTTATAATGT  120

Query  121    AATTGTAACAGCACATGCATTTATTATAAttttttttATAGTTATACCAATTATAATCGG  180
               ||||||||||||||||||||||||||||||||||||||||||||||||||||||||||||
Sbjct  121    AATTGTAACAGCACATGCATTTATTATAATTTTTTTTATAGTTATACCAATTATAATCGG  180

Query  181    AGGATTTGGAAATTGATTAGTTCCTATTATACTAGGAGCTCCAGATATAGCATTTCCACG  240
               |||||||||||||||||||||||||||||| |||||||||||||||||||||||||||||
Sbjct  181    AGGATTTGGAAATTGATTAGTTCCTATTATGCTAGGAGCTCCAGATATAGCATTTCCACG  240
```

```
Query  241  AATAAATAATATAAGATTTTGGTTACTTCCTCCTTCTCTATTTCTACTAATTAATTCTTC  300
            ||||||||||||||||||||||||||||||||||||||||||||||||||||||||||||
Sbjct  241  AATAAATAATATAAGATTTTGGTTACTTCCTCCTTCTCTATTTCTACTAATTAATTCTTC  300

Query  301  ATTAATCGAATCAGGAGCAGGTACAGGGTGAACAGTTTAcccccccTTATCATCAAATTT  360
            |||||||||||||||||||||||||||||||||||||||||| |||||||||||||||||
Sbjct  301  ATTAATCGAATCAGGAGCAGGTACAGGGTGAACAGTTTACCCTCCCTTATCATCAAATTT  360

Query  361  ATCACACTACGGACCATCAGTAGATTTAGCTATTTTTTCTCTGCATCTTGCAGGTGCTTC  420
            |||||||||||| |||||||||||||||||||| || |||||||||||||| ||||||||
Sbjct  361  ATCACACTACGGGCCATCAGTAGATTTAGCTATCTTCTCTCTGCATCTTGCCGGTGCTTC  420

Query  421  TTCAATTTAGGTGCAATCAATTTCATTACAACTATTGTAAATATACGATCCATTGGAAT  480
            ||||||||||||||||| ||||||||||||||||||||||||||||||||||||||||||
Sbjct  421  TTCAATTTAGGTGCAATTAATTTCATTACAACTATTGTAAATATACGATCCATTGGAAT  480

Query  481  AACAATAGAACGAATACCATTATTTGTATGATCTGTTTTAATTACTGCAATTTTATTATT  540
            ||||||||||||||||||||||||||||||||||||||||||||||||||||||||||||
Sbjct  481  AACAATAGAACGAATACCATTATTTGTATGATCTGTTTTAATTACTGCAATTTTATTATT  540

Query  541  ATTATCTTTACCAGTCTTAGCTGGCGCAATTACAATATTATTAACTGATCGAAACTTTAA  600
            |||||||||||||||||||||||| |||||||||||||||||||||||||||||||||||
Sbjct  541  ATTATCTTTACCAGTCTTAGCTGGTGCAATTACAATATTATTAACTGATCGAAACTTTAA  600

Query  601  TACATCATTCTTTGACCCTTCAGGAGGAGGTGACCCAATTCTATATCAACATTTATTT  658
            ||||||||| |||||||||||||||||||||||||||||||| |||||||||||||||
Sbjct  601  TACATCATTTTTTGACCCTTCAGGAGGAGGTGACCCAATTCTGTATCAACATTTATTT  658
```

>Rhipicephalus sanguineus isolate GTF2 cytochrome oxidase subunit I (cox1) gene, partial cds; mitochondrial
Sequence ID: JX416303.1 Length: 802
Range 1: 1 to 637

Score:1155 bits(625), Expect:0.0,
Identities:633/637(99%), Gaps:0/637(0%), Strand: Plus/Plus

```
Query  22   AGCATGATCCGGAATACTAGGATTAAGAATAAGAATATTAATCCGCATAGAATTAGGTCA  81
            ||||||||||||||||||||||||||||||||||||||||||||||||||||||||||||
Sbjct  1    AGCATGATCCGGAATACTAGGATTAAGAATAAGAATATTAATCCGCATAGAATTAGGTCA  60

Query  82   ACCAGGAACTTTAATTGGAAACGATCAAATTTATAATGTAATTGTAACAGCACATGCATT  141
            ||||||||||||||||||||||||||||||||||||||||||||||||||||||||||||
Sbjct  61   ACCAGGAACTTTAATTGGAAACGATCAAATTTATAATGTAATTGTAACAGCACATGCATT  120

Query  142  TATTATAAtttttttATAGTTATACCAATTATAATCGGAGGATTTGGAAATTGATTAGT  201
            |||||||||||||||||||||||||||||||||||||||||||||||| |||||||||||
Sbjct  121  TATTATAATTTTTTTATAGTTATACCAATTATAATCGGAGGATTTGGAGATTGATTAGT  180

Query  202  TCCTATTATACTAGGAGCTCCAGATATAGCATTTCCACGAATAAATAATATAAGATTTTG  261
            ||||||||||||||||||||||||||||||||||||||||||||||||||||||||||||
Sbjct  181  TCCTATTATACTAGGAGCTCCAGATATAGCATTTCCACGAATAAATAATATAAGATTTTG  240
```

```
Query  262   GTTACTTCCTCCTTCTCTATTTCTACTAATTAATTCTTCATTAATCGAATCAGGAGCAGG  321
             ||||||||||||||||||||||||||||||||||||||||||||||||||||||||||||
Sbjct  241   GTTACTTCCTCCTTCTCTATTTCTACTAATTAATTCTTCATTAATCGAATCAGGAGCAGG  300

Query  322   TACAGGGTGAACAGTTTAcccccccTTATCATCAAATTTATCACACTACGGACCATCAGT  381
             ||||||||||||||||||||||||||||||||||||||||||||||||||||||||||||
Sbjct  301   TACAGGGTGAACAGTTTACCCCCCCTTATCATCAAATTTATCACACTACGGACCATCAGT  360

Query  382   AGATTTAGCTATTTTTTCTCTGCATCTTGCAGGTGCTTCTTCAATTTTAGGTGCAATCAA  441
             ||||||||||||||||||||||||||||||||||||||||||||| ||||||||||||||
Sbjct  361   AGATTTAGCTATTTTTTCTCTGCATCTTGCAGGTGCTTCTTCAATCTTAGGTGCAATCAA  420

Query  442   TTTCATTACAACTATTGTAAATATACGATCCATTGGAATAACAATAGAACGAATACCATT  501
             ||||||||||||||||||||||| ||||||||||||||||||||||||||||||||||||
Sbjct  421   TTTCATTACAACTATTGTAAATACACGATCCATTGGAATAACAATAGAACGAATACCATT  480

Query  502   ATTTGTATGATCTGTTTTAATTACTGCAATTTTATTATTATTATCTTTACCAGTCTTAGC  561
             |||||||||||||||||||||||||||||||||||||||||||| |||||||||||||||
Sbjct  481   ATTTGTATGATCTGTTTTAATTACTGCAATTTTATTATTATTATTTTTACCAGTCTTAGC  540

Query  562   TGGCGCAATTACAATATTATTAACTGATCGAAACTTTAATACATCATTCTTTGACCCTTC  621
             ||||||||||||||||||||||||||||||||||||||||||||||||||||||||||||
Sbjct  541   TGGCGCAATTACAATATTATTAACTGATCGAAACTTTAATACATCATTCTTTGACCCTTC  600

Query  622   AGGAGGAGGTGACCCAATTCTATATCAACATTTATTT  658
             |||||||||||||||||||||||||||||||||||||
Sbjct  601   AGGAGGAGGTGACCCAATTCTATATCAACATTTATTT  637
```

>Rhipicephalus sanguineus sensu lato 'tropical lineage' isolate CV3042
cytochrome c oxidase subunit I (COI) gene, partial cds; mitochondrial
Sequence ID: MF426003.1 Length: 826
Range 1: 3 to 650

Score:1153 bits(624), Expect:0.0,
Identities:640/648(99%), Gaps:0/648(0%), Strand: Plus/Plus

```
Query  11    TTAATTTTTGGAGCATGATCCGGAATACTAGGATTAAGAATAAGAATATTAATCCGCATA  70
             ||||||||||||||||||||||||||||||||||||||||||||||||||||| ||||||
Sbjct  3     TTAATTTTTGGAGCATGATCCGGAATACTAGGATTAAGAATAAGAATATTAATTCGCATA  62

Query  71    GAATTAGGTCAACCAGGAACTTTAATTGGAAACGATCAAATTTATAATGTAATTGTAACA  130
             ||||||||||||||||||||||||||||||||||||||||||||||||||||||||||||
Sbjct  63    GAATTAGGTCAACCAGGAACTTTAATTGGAAACGATCAAATTTATAATGTAATTGTAACA  122

Query  131   GCACATGCATTTATTATAAttttttttATAGTTATACCAATTATAATCGGAGGATTTGGA  190
             ||||||||||||||||||||||||||||||||||||||||||||||||||||||||||||
Sbjct  123   GCACATGCATTTATTATAATTTTTTTTATAGTTATACCAATTATAATCGGAGGATTTGGA  182

Query  191   AATTGATTAGTTCCTATTATACTAGGAGCTCCAGATATAGCATTTCCACGAATAAATAAT  250
             |||||||||||||||||||||||||||||||||||||||||||||||||||||||| |||
```

```
Sbjct  183   AATTGATTAGTTCCTATTATACTAGGAGCTCCAGATATAGCATTTCCACGAATAAACAAT  242

Query  251   ATAAGATTTTGGTTACTTCCTCCTTCTCTATTTCTACTAATTAATTCTTCATTAATCGAA  310
             ||||||||||||||||||||||||||||||||||||||||||||||||||||||||||||
Sbjct  243   ATAAGATTTTGGTTACTTCCTCCTTCTCTATTTCTACTAATTAATTCTTCATTAATCGAA  302

Query  311   TCAGGAGCAGGTACAGGGTGAACAGTTTAcccccccTTATCATCAAATTTATCACACTAC  370
             |||||||| |||||||||||||||||||||||||||||||||||||||||||||||||||
Sbjct  303   TCAGGAGCGGGTACAGGGTGAACAGTTTACCCCCCCTTATCATCAAATTTATCACACTAC  362

Query  371   GGACCATCAGTAGATTTAGCTATTTTTCTCTGCATCTTGCAGGTGCTTCTTCAATTTTA  430
             ||||||||||||||||||||||| ||||||||||||||||||||||||||||||||||||
Sbjct  363   GGACCATCAGTAGATTTAGCTATCTTTCTCTGCATCTTGCAGGTGCTTCTTCAATTTTA  422

Query  431   GGTGCAATCAATTTCATTACAACTATTGTAAATATACGATCCATTGGAATAACAATAGAA  490
             || |||||||||||||||||||||||||||||||||||||||||||||||||||||||||
Sbjct  423   GGCGCAATCAATTTCATTACAACTATTGTAAATATACGATCCATTGGAATAACAATAGAA  482

Query  491   CGAATACCATTATTTGTATGATCTGTTTTAATTACTGCAATTTTATTATTATTATCTTTA  550
             ||||||||||||||||||||||||||||||||||||||||||||||||||||||||||||
Sbjct  483   CGAATACCATTATTTGTATGATCTGTTTTAATTACTGCAATTTTATTATTATTATCTTTA  542

Query  551   CCAGTCTTAGCTGGCGCAATTACAATATTATTAACTGATCGAAACTTTAATACATCATTC  610
             |||||||||||||| |||||||||||||| ||||||||||||||||||||||||||||||
Sbjct  543   CCAGTCTTAGCTGGTGCAATTACAATATTGTTAACTGATCGAAACTTTAATACATCATTC  602

Query  611   TTTGACCCTTCAGGAGGAGGTGACCCAATTCTATATCAACATTTATTT  658
             |||||||||||||||||||||||||||||||| |||||||||||||||
Sbjct  603   TTTGACCCTTCAGGAGGAGGTGACCCAATTCTGTATCAACATTTATTT  650
```

>Rhipicephalus sanguineus voucher AMMS-RS-4 cytochrome c oxidase subunit I-like (COI) gene, partial sequence; mitochondrial
Sequence ID: HM193874.1 Length: 665
Range 1: 31 to 665

Score:1146 bits(620), Expect:0.0,
Identities:631/636(99%), Gaps:1/636(0%), Strand: Plus/Plus

```
Query  1     GACAATATACTTAATTTTTGGAGCATGATCCGGAATACTAGGATTAAGAATAAGAATATT  60
             ||||||||||||||||||||||||||||||||||||||||||||||||||||||||||||
Sbjct  31    GACAATATACTTAATTTTTGGAGCATGATCCGGAATACTAGGATTAAGAATAAGAATATT  90

Query  61    AATCCGCATAGAATTAGGTCAACCAGGAACTTTAATTGGAAACGATCAAATTTATAATGT  120
             ||||||||||||||||||||||||||||||||||||||||||||||||||||||||||||
Sbjct  91    AATCCGCATAGAATTAGGTCAACCAGGAACTTTAATTGGAAACGATCAAATTTATAATGT  150

Query  121   AATTGTAACAGCACATGCATTTATTATAAttttttttATAGTTATACCAATTATAATCGG  180
             ||||||||||||||||||||||||||||||||||||||||||||||||||||||||||||
Sbjct  151   AATTGTAACAGCACATGCATTTATTATAATTTTTTTTATAGTTATACCAATTATAATCGG  210

Query  181   AGGATTTGGAAATTGATTAGTTCCTATTATACTAGGAGCTCCAGATATAGCATTTCCACG  240
```

```
                ||||||||||||||||||||||||||||||||||||||||||||||||||||||||||||
Sbjct  211  AGGATTTGGAAATTGATTAGTTCCTATTATACTAGGAGCTCCAGATATAGCATTTCCACG  270

Query  241  AATAAATAATATAAGATTTTGGTTACTTCCTCCTTCTCTATTTCTACTAATTAATTCTTC  300
            ||||||||||||||||||||||||||||||||||||||||||||||||||||||||||||
Sbjct  271  AATAAATAATATAAGATTTTGGTTACTTCCTCCTTCTCTATTTCTACTAATTAATTCTTC  330

Query  301  ATTAATCGAATCAGGAGCAGGTACAGGGTGAACAGTTTAccccccTTATCATCAAATTT  360
            ||||||||||||||||||||||||||||||||||||||||||||||||||||||||||||
Sbjct  331  ATTAATCGAATCAGGAGCAGGTACAGGGTGAACAGTTTACCCCCCTTATCATCAAATTT  390

Query  361  ATCACACTACGGACCATCAGTAGATTTAGCTATTTTTTCTCTGCATCTTGCAGGTGCTTC  420
            ||||||||||||||||||||||||||||||||||||||||||||||||||||||||||||
Sbjct  391  ATCACACTACGGACCATCAGTAGATTTAGCTATTTTTTCTCTGCATCTTGCAGGTGCTTC  450

Query  421  TTCAATTTTAGGTGCAATCAATTTCATTACAACTATTGTAAATATACGATCCATTGGAAT  480
            ||||||||||||||||||||||||||||||||||||||||||||||||||||||||||||
Sbjct  451  TTCAATTTTAGGTGCAATCAATTTCATTACAACTATTGTAAATATACGATCCATTGGAAT  510

Query  481  AACAATAGAACGAATACCATTATTTGTATGATCTGTTTTAATTACTGCAATTTTATTATT  540
            ||||||||||||||| ||||||||||||||||||||||||||||||||||||||||||||
Sbjct  511  AACAATAGAACGAATCCCATTATTTGTATGATCTGTTTTAATTACTGCAATTTTATTATT  570

Query  541  ATTATCTTTACCAGTCTTAGCTGGCGCAATTACAATATTATTAACTGATCGAAACTTTAA  600
            ||||||||||||||||||||||||||||||||||||| ||||||||||||||||||||||
Sbjct  571  ATTATCTTTACCAGTCTTAGCTGGCGCAATTACAATAATATTAACTGATCGAAACTTTAA  630

Query  601  TACATCATTCTTTGACCCTTCAGGAGGAGGTGACCC  636
            ||||||||||||||||||  |||||||||| |||||
Sbjct  631  TACATCATTCTTTGACCCAGCAGGAGGAGG-GACCC  665
```

>Rhipicephalus sanguineus isolate Abidjan cytochrome oxidase subunit I (COI)
gene, partial cds; mitochondrial
Sequence ID: KX757914.1 Length: 630
Range 1: 1 to 620

Score:1140 bits(617), Expect:0.0,
Identities:619/620(99%), Gaps:0/620(0%), Strand: Plus/Plus

```
Query  39   TAGGATTAAGAATAAGAATATTAATCCGCATAGAATTAGGTCAACCAGGAACTTTAATTG  98
            ||||||||||||||||||||||||||||||||||||||||||||||||||||||||||||
Sbjct  1    TAGGATTAAGAATAAGAATATTAATCCGCATAGAATTAGGTCAACCAGGAACTTTAATTG  60

Query  99   GAAACGATCAAATTTATAATGTAATTGTAACAGCACATGCATTTATTATAAtttttttA  158
            ||||||||||||||||||||||||||||||||||||||||||||||||||||||||||||
Sbjct  61   GAAACGATCAAATTTATAATGTAATTGTAACAGCACATGCATTTATTATAATTTTTTTA  120

Query  159  TAGTTATACCAATTATAATCGGAGGATTTGGAAATTGATTAGTTCCTATTATACTAGGAG  218
            |||||||||||||||||||||||||||||||||||||||||||||||||||||||||| |
Sbjct  121  TAGTTATACCAATTATAATCGGAGGATTTGGAAATTGATTAGTTCCTATTATACTAGGGG  180
```

```
Query  219   CTCCAGATATAGCATTTCCACGAATAAATAATATAAGATTTTGGTTACTTCCTCCTTCTC  278
             ||||||||||||||||||||||||||||||||||||||||||||||||||||||||||||
Sbjct  181   CTCCAGATATAGCATTTCCACGAATAAATAATATAAGATTTTGGTTACTTCCTCCTTCTC  240

Query  279   TATTTCTACTAATTAATTCTTCATTAATCGAATCAGGAGCAGGTACAGGGTGAACAGTTT  338
             ||||||||||||||||||||||||||||||||||||||||||||||||||||||||||||
Sbjct  241   TATTTCTACTAATTAATTCTTCATTAATCGAATCAGGAGCAGGTACAGGGTGAACAGTTT  300

Query  339   AcccccccTTATCATCAAATTTATCACACTACGGACCATCAGTAGATTTAGCTATTTTTT  398
             ||||||||||||||||||||||||||||||||||||||||||||||||||||||||||||
Sbjct  301   ACCCCCCCTTATCATCAAATTTATCACACTACGGACCATCAGTAGATTTAGCTATTTTTT  360

Query  399   CTCTGCATCTTGCAGGTGCTTCTTCAATTTTAGGTGCAATCAATTTCATTACAACTATTG  458
             ||||||||||||||||||||||||||||||||||||||||||||||||||||||||||||
Sbjct  361   CTCTGCATCTTGCAGGTGCTTCTTCAATTTTAGGTGCAATCAATTTCATTACAACTATTG  420

Query  459   TAAATATACGATCCATTGGAATAACAATAGAACGAATACCATTATTTGTATGATCTGTTT  518
             ||||||||||||||||||||||||||||||||||||||||||||||||||||||||||||
Sbjct  421   TAAATATACGATCCATTGGAATAACAATAGAACGAATACCATTATTTGTATGATCTGTTT  480

Query  519   TAATTACTGCAATTTTATTATTATTATCTTTACCAGTCTTAGCTGGCGCAATTACAATAT  578
             ||||||||||||||||||||||||||||||||||||||||||||||||||||||||||||
Sbjct  481   TAATTACTGCAATTTTATTATTATTATCTTTACCAGTCTTAGCTGGCGCAATTACAATAT  540

Query  579   TATTAACTGATCGAAACTTTAATACATCATTCTTTGACCCTTCAGGAGGAGGTGACCCAA  638
             ||||||||||||||||||||||||||||||||||||||||||||||||||||||||||||
Sbjct  541   TATTAACTGATCGAAACTTTAATACATCATTCTTTGACCCTTCAGGAGGAGGTGACCCAA  600

Query  639   TTCTATATCAACATTTATTT  658
             ||||||||||||||||||||
Sbjct  601   TTCTATATCAACATTTATTT  620
```

```
>Rhipicephalus sanguineus isolate LIC5960 cytochrome oxidase subunit 1 (COI)
gene, partial cds; mitochondrial
Sequence ID: KX383800.1 Length: 768
>Rhipicephalus sanguineus isolate LIC5766D cytochrome oxidase subunit 1 (COI)
gene, partial cds; mitochondrial
Sequence ID: KX383815.1 Length: 768
Range 1: 1 to 642

Score:1136 bits(615), Expect:0.0,
Identities:633/642(99%),  Gaps:0/642(0%), Strand: Plus/Plus

Query  17    TTTGGAGCATGATCCGGAATACTAGGATTAAGAATAAGAATATTAATCCGCATAGAATTA  76
             ||||||||||||||||||||||||||||||||||||||||||||||| ||||||||||||
Sbjct  1     TTTGGAGCATGATCCGGAATACTAGGATTAAGAATAAGAATATTAATTCGCATAGAATTA  60

Query  77    GGTCAACCAGGAACTTTAATTGGAAACGATCAAATTTATAATGTAATTGTAACAGCACAT  136
             |||||||||||||||||||||||||||||||||||||||||||||||||| |||||||||
Sbjct  61    GGTCAACCAGGAACTTTAATTGGAAACGATCAAATTTATAATGTAATTGTGACAGCACAT  120
```

```
Query  137  GCATTTATTATAAtttttttATAGTTATACCAATTATAATCGGAGGATTTGGAAATTGA  196
            |||||||||||||| |||||||||||||||||||||||||||||||||||||||||||||
Sbjct  121  GCATTTATTATAATCTTTTTTATAGTTATACCAATTATAATCGGAGGATTTGGAAATTGA  180

Query  197  TTAGTTCCTATTATACTAGGAGCTCCAGATATAGCATTTCCACGAATAAATAATATAAGA  256
            |||||||||||||||||||||||||||||||||||||||||||||||||| |||||||||
Sbjct  181  TTAGTTCCTATTATACTAGGAGCTCCAGATATAGCATTTCCACGAATAAACAATATAAGA  240

Query  257  TTTTGGTTACTTCCTCCTTCTCTATTTCTACTAATTAATTCTTCATTAATCGAATCAGGA  316
            ||||||||||||||||||||||||||||||||||||||||||||||||||||||||||||
Sbjct  241  TTTTGGTTACTTCCTCCTTCTCTATTTCTACTAATTAATTCTTCATTAATCGAATCAGGA  300

Query  317  GCAGGTACAGGGTGAACAGTTTAcccccccTTATCATCAAATTTATCACACTACGGACCA  376
            ||||||||||||||||||||||||||||||||||||||||||||||||||||||||||||
Sbjct  301  GCAGGTACAGGGTGAACAGTTTACCCCCCCTTATCATCAAATTTATCACACTACGGACCA  360

Query  377  TCAGTAGATTTAGCTATTTTTTCTCTGCATCTTGCAGGTGCTTCTTCAATTTTAGGTGCA  436
            ||||||||||||||||| |||||||||||||||||||||||||||||||||||||||| |||
Sbjct  361  TCAGTAGATTTAGCTATCTTTTCTCTGCATCTTGCAGGTGCTTCTTCAATTTTAGGCGCA  420

Query  437  ATCAATTTCATTACAACTATTGTAAATATACGATCCATTGGAATAACAATAGAACGAATA  496
            ||||||||||||||||||||||||||||||||||||||||||||||||||||||||||||
Sbjct  421  ATCAATTTCATTACAACTATTGTAAATATACGATCCATTGGAATAACAATAGAACGAATA  480

Query  497  CCATTATTTGTATGATCTGTTTTAATTACTGCAATTTTATTATTATTATCTTTACCAGTC  556
            |||||||||||||||||||||||||||||||||||||||||||| |||||||||||||||
Sbjct  481  CCATTATTTGTATGATCTGTTTTAATTACTGCAATTTTATTATTGTTATCTTTACCAGTC  540

Query  557  TTAGCTGGCGCAATTACAATATTATTAACTGATCGAAACTTTAATACATCATTCTTTGAC  616
            |||||||| |||||||||||||||||||||||||||||||||||||||||||||||||||
Sbjct  541  TTAGCTGGTGCAATTACAATATTATTAACTGATCGAAACTTTAATACATCATTCTTTGAC  600

Query  617  CCTTCAGGAGGAGGTGACCCAATTCTATATCAACATTTATTT  658
            |||||||||||||||||||||||||| |||||||||||||||
Sbjct  601  CCTTCAGGAGGAGGTGACCCAATTCTGTATCAACATTTATTT  642
```

>Rhipicephalus sanguineus isolate LIC5682B cytochrome oxidase subunit 1 (COI) gene, partial cds; mitochondrial
Sequence ID: KX383796.1 Length: 744
Range 1: 1 to 618

Score:1131 bits(612), Expect:0.0,
Identities:616/618(99%), Gaps:0/618(0%), Strand: Plus/Plus

```
Query  41   GGATTAAGAATAAGAATATTAATCCGCATAGAATTAGGTCAACCAGGAACTTTAATTGGA  100
            ||||||||||||||||||||||||||||||||||||||||||||||||||||||||||||
Sbjct  1    GGATTAAGAATAAGAATATTAATCCGCATAGAATTAGGTCAACCAGGAACTTTAATTGGA  60

Query  101  AACGATCAAATTTATAATGTAATTGTAACAGCACATGCATTTATTATAAtttttttATA  160
            ||||||||||||||||||||||||||||||||||||||||||||||||||||||||||||
Sbjct  61   AACGATCAAATTTATAATGTAATTGTAACAGCACATGCATTTATTATAATTTTTTTTATA  120
```

```
Query  161  GTTATACCAATTATAATCGGAGGATTTGGAAATTGATTAGTTCCTATTATACTAGGAGCT  220
            ||||||||||||||||||||||||||||||||||||||||||||||||||||||||||||
Sbjct  121  GTTATACCAATTATAATCGGAGGATTTGGAAATTGATTAGTTCCTATTATACTAGGAGCT  180

Query  221  CCAGATATAGCATTTCCACGAATAAATAATATAAGATTTTGGTTACTTCCTCCTTCTCTA  280
            ||||||||||||||||||||||||||||||||||||||||||||||||||||||||||||
Sbjct  181  CCAGATATAGCATTTCCACGAATAAATAATATAAGATTTTGGTTACTTCCTCCTTCTCTA  240

Query  281  TTTCTACTAATTAATTCTTCATTAATCGAATCAGGAGCAGGTACAGGGTGAACAGTTTAc  340
            ||||||||||||||||||||||||||||||||||||||||||||||||||||||||||||
Sbjct  241  TTTCTACTAATTAATTCTTCATTAATCGAATCAGGAGCAGGTACAGGGTGAACAGTTTAC  300

Query  341  ccccccTTATCATCAAATTTATCACACTACGGACCATCAGTAGATTTAGCTATTTTTTCT  400
            ||||||||||||||||||||||||||||||||||||||||||||||||||||||||||||
Sbjct  301  CCCCCCTTATCATCAAATTTATCACACTACGGACCATCAGTAGATTTAGCTATTTTTTCT  360

Query  401  CTGCATCTTGCAGGTGCTTCTTCAATTTTAGGTGCAATCAATTTCATTACAACTATTGTA  460
            || |||||||||||||||||||||||||||||||||||||||||||||||||||||||||
Sbjct  361  CTACATCTTGCAGGTGCTTCTTCAATTTTAGGTGCAATCAATTTCATTACAACTATTGTA  420

Query  461  AATATACGATCCATTGGAATAACAATAGAACGAATACCATTATTTGTATGATCTGTTTTA  520
            ||||||||||||||||||||||||||||||||||||||||||||||||||||||||||||
Sbjct  421  AATATACGATCCATTGGAATAACAATAGAACGAATACCATTATTTGTATGATCTGTTTTA  480

Query  521  ATTACTGCAATTTTATTATTATTATCTTTACCAGTCTTAGCTGGCGCAATTACAATATTA  580
            ||||||||||||||||||||||||||||||||||||||||||||||||||||||||||||
Sbjct  481  ATTACTGCAATTTTATTATTATTATCTTTACCAGTCTTAGCTGGCGCAATTACAATATTA  540

Query  581  TTAACTGATCGAAACTTTAATACATCATTCTTTGACCCTTCAGGAGGAGGTGACCCAATT  640
            ||||||||||||||||||||||||||||||||||||||||||||||| ||||||||||||
Sbjct  541  TTAACTGATCGAAACTTTAATACATCATTCTTTGACCCTTCAGGAGGGGGTGACCCAATT  600

Query  641  CTATATCAACATTTATTT  658
            ||||||||||||||||||
Sbjct  601  CTATATCAACATTTATTT  618
```

>Rhipicephalus sanguineus isolate LIC5958B cytochrome oxidase subunit 1 (COI) gene, partial cds; mitochondrial
Sequence ID: KX383798.1 Length: 698
Range 1: 1 to 637

Score:1127 bits(610), Expect:0.0,
Identities:628/637(99%), Gaps:0/637(0%), Strand: Plus/Plus

```
Query  22   AGCATGATCCGGAATACTAGGATTAAGAATAAGAATATTAATCCGCATAGAATTAGGTCA  81
            |||||||||||||||||||||||||||||||||||||||||| |||||||||||||||||
Sbjct  1    AGCATGATCCGGAATACTAGGATTAAGAATAAGAATATTAATTCGCATAGAATTAGGTCA  60

Query  82   ACCAGGAACTTTAATTGGAAACGATCAAATTTATAATGTAATTGTAACAGCACATGCATT  141
            ||||||||||||||||||||||||||||||||||||||||||||| ||||||||||||||
```

```
Sbjct  61   ACCAGGAACTTTAATTGGAAACGATCAAATTTATAATGTAATTGTGACAGCACATGCATT  120

Query  142  TATTATAAtttttttATAGTTATACCAATTATAATCGGAGGATTTGGAAATTGATTAGT  201
            ||||||||| ||||||||||||||||||||||||||||||||||||||||||||||||||
Sbjct  121  TATTATAATCTTTTTTATAGTTATACCAATTATAATCGGAGGATTTGGAAATTGATTAGT  180

Query  202  TCCTATTATACTAGGAGCTCCAGATATAGCATTTCCACGAATAAATAATATAAGATTTTG  261
            ||||||||||||||||||||||||||||||||||||||||||||| ||||||||||||||
Sbjct  181  TCCTATTATACTAGGAGCTCCAGATATAGCATTTCCACGAATAAACAATATAAGATTTTG  240

Query  262  GTTACTTCCTCCTTCTCTATTTCTACTAATTAATTCTTCATTAATCGAATCAGGAGCAGG  321
            ||||||||||||||||||||||||||||||||||||||||||||||||||||||||||||
Sbjct  241  GTTACTTCCTCCTTCTCTATTTCTACTAATTAATTCTTCATTAATCGAATCAGGAGCAGG  300

Query  322  TACAGGGTGAACAGTTTAcccccccTTATCATCAAATTTATCACACTACGGACCATCAGT  381
            ||||||||||||||||||||||||||||||||||||||||||||||||||||||||||||
Sbjct  301  TACAGGGTGAACAGTTTACCCCCCCTTATCATCAAATTTATCACACTACGGACCATCAGT  360

Query  382  AGATTTAGCTATTTTTTCTCTGCATCTTGCAGGTGCTTCTTCAATTTTAGGTGCAATCAA  441
            |||||||||||| |||||||||||||||||||||||||||||||||||||| ||||||||
Sbjct  361  AGATTTAGCTATCTTTTCTCTGCATCTTGCAGGTGCTTCTTCAATTTTAGGCGCAATCAA  420

Query  442  TTTCATTACAACTATTGTAAATATACGATCCATTGGAATAACAATAGAACGAATACCATT  501
            ||||||||||||||||||||||||||||||||||||||||||||||||||||||||||||
Sbjct  421  TTTCATTACAACTATTGTAAATATACGATCCATTGGAATAACAATAGAACGAATACCATT  480

Query  502  ATTTGTATGATCTGTTTTAATTACTGCAATTTTATTATTATTATCTTTACCAGTCTTAGC  561
            ||||||||||||||||||||||||||||||||||||||| ||||||||||||||||||||
Sbjct  481  ATTTGTATGATCTGTTTTAATTACTGCAATTTTATTATTGTTATCTTTACCAGTCTTAGC  540

Query  562  TGGCGCAATTACAATATTATTAACTGATCGAAACTTTAATACATCATTCTTTGACCCTTC  621
            ||| ||||||||||||||||||||||||||||||||||||||||||||||||||||||||
Sbjct  541  TGGTGCAATTACAATATTATTAACTGATCGAAACTTTAATACATCATTCTTTGACCCTTC  600

Query  622  AGGAGGAGGTGACCCAATTCTATATCAACATTTATTT  658
            ||||||||||||||||||||| |||||||||||||||
Sbjct  601  AGGAGGAGGTGACCCAATTCTGTATCAACATTTATTT  637
```

>Rhipicephalus sanguineus isolate LIC5584D cytochrome oxidase subunit 1 (COI) gene, partial cds; mitochondrial
Sequence ID: KX383814.1 Length: 768
Range 1: 1 to 642

Score:1125 bits(609), Expect:0.0,
Identities:631/642(98%), Gaps:0/642(0%), Strand: Plus/Plus

```
Query  17   TTTGGAGCATGATCCGGAATACTAGGATTAAGAATAAGAATATTAATCCGCATAGAATTA  76
            |||||||||||||||||||||||||||||||||||||||||||||| |||||||||||||
Sbjct  1    TTTGGAGCATGATCCGGAATACTAGGATTAAGAATAAGAATATTAATTCGCATAGAATTA  60

Query  77   GGTCAACCAGGAACTTTAATTGGAAACGATCAAATTTATAATGTAATTGTAACAGCACAT  136
```

```
                ||||||||||||||||||||||||||||||||||||||||||||||||||| |||||||||
Sbjct  61       GGTCAACCAGGAACTTTAATTGGAAACGATCAAATTTATAATGTAATTGTGACAGCACAT  120

Query  137      GCATTTATTATAAttttttttATAGTTATACCAATTATAATCGGAGGATTTGGAAATTGA  196
                |||||||||||||| |||||||||||||||||||||||||||||||||||||||||||||
Sbjct  121      GCATTTATTATAATCTTTTTTATAGTTATACCAATTATAATCGGAGGATTTGGAAATTGA  180

Query  197      TTAGTTCCTATTATACTAGGAGCTCCAGATATAGCATTTCCACGAATAAATAATATAAGA  256
                |||||||||||||||||||||||||||||||||||||||||||||||||| |||||||||
Sbjct  181      TTAGTTCCTATTATACTAGGAGCTCCAGATATAGCATTTCCACGAATAAACAATATAAGA  240

Query  257      TTTTGGTTACTTCCTCCTTCTCTATTTCTACTAATTAATTCTTCATTAATCGAATCAGGA  316
                ||||||||||||||||||||||||||||||||||||||||||||||||||||||||||||
Sbjct  241      TTTTGGTTACTTCCTCCTTCTCTATTTCTACTAATTAATTCTTCATTAATCGAATCAGGA  300

Query  317      GCAGGTACAGGGTGAACAGTTTAcccccccTTATCATCAAATTTATCACACTACGGACCA  376
                ||||||||||||||||||||||||||||||||||||||||||||||||||||||||| ||
Sbjct  301      GCAGGTACAGGGTGAACAGTTTACCCCCCCTTATCATCAAATTTATCACACTACGGATCA  360

Query  377      TCAGTAGATTTAGCTATTTTTTCTCTGCATCTTGCAGGTGCTTCTTCAATTTTAGGTGCA  436
                ||||||||||||||||| |||||||||||||||||||||||||||||||||||||| |||
Sbjct  361      TCAGTAGATTTAGCTATCTTTTCTCTGCATCTTGCAGGTGCTTCTTCAATTTTAGGCGCA  420

Query  437      ATCAATTTCATTACAACTATTGTAAATATACGATCCATTGGAATAACAATAGAACGAATA  496
                ||||||||||||||||||||||||||||||||||||||||||||||||||||||||||||
Sbjct  421      ATCAATTTCATTACAACTATTGTAAATATACGATCCATTGGAATAACAATAGAACGAATA  480

Query  497      CCATTATTTGTATGATCTGTTTTAATTACTGCAATTTTATTATTATTATCTTTACCAGTC  556
                |||||||||||||||||||||||||||||||||||||||||||| ||||||||||||||
Sbjct  481      CCATTATTTGTATGATCTGTTTTAATTACTGCAATTTTATTATTGTTATCTTTACCAGTT  540

Query  557      TTAGCTGGCGCAATTACAATATTATTAACTGATCGAAACTTTAATACATCATTCTTTGAC  616
                |||||||| |||||||||||||||||||||||||||||||||||||||||||||||||||
Sbjct  541      TTAGCTGGTGCAATTACAATATTATTAACTGATCGAAACTTTAATACATCATTCTTTGAC  600

Query  617      CCTTCAGGAGGAGGTGACCCAATTCTATATCAACATTTATTT  658
                |||||||||||||||||||||||||| |||||||||||||||
Sbjct  601      CCTTCAGGAGGAGGTGACCCAATTCTGTATCAACATTTATTT  642
```

>Rhipicephalus sanguineus isolate LIC5755 cytochrome oxidase subunit 1 (COI)
gene, partial cds; mitochondrial
Sequence ID: KX383801.1 Length: 763
Range 1: 1 to 637

Score:1122 bits(607), Expect:0.0,
Identities:627/637(98%), Gaps:0/637(0%), Strand: Plus/Plus

```
Query  22       AGCATGATCCGGAATACTAGGATTAAGAATAAGAATATTAATCCGCATAGAATTAGGTCA  81
                |||||||||||||||||||||||||||||||||||||||||| |||||||||||||||||
Sbjct  1        AGCATGATCCGGAATACTAGGATTAAGAATAAGAATATTAATTCGCATAGAATTAGGTCA  60
```

```
Query  82   ACCAGGAACTTTAATTGGAAACGATCAAATTTATAATGTAATTGTAACAGCACATGCATT  141
            ||||||||||||||||||||||||||||||||||||||||||||| ||||||||||||||
Sbjct  61   ACCAGGAACTTTAATTGGAAACGATCAAATTTATAATGTAATTGTGACAGCACATGCATT  120

Query  142  TATTATAAtttttttATAGTTATACCAATTATAATCGGAGGATTTGGAAATTGATTAGT  201
            ||||||||| ||||||||||||||||||||||||||||||||||||||||||||||||||
Sbjct  121  TATTATAATCTTTTTTATAGTTATACCAATTATAATCGGAGGATTTGGAAATTGATTAGT  180

Query  202  TCCTATTATACTAGGAGCTCCAGATATAGCATTTCCACGAATAAATAATATAAGATTTTG  261
            ||||||||||||||||||||||||||||||||||||||||||||| ||||||||||||||
Sbjct  181  TCCTATTATACTAGGAGCTCCAGATATAGCATTTCCACGAATAAACAATATAAGATTTTG  240

Query  262  GTTACTTCCTCCTTCTCTATTTCTACTAATTAATTCTTCATTAATCGAATCAGGAGCAGG  321
            ||||||||||||||||||||||||||||||||||||||||||||||||||||||||||||
Sbjct  241  GTTACTTCCTCCTTCTCTATTTCTACTAATTAATTCTTCATTAATCGAATCAGGAGCAGG  300

Query  322  TACAGGGTGAACAGTTTAcccccccTTATCATCAAATTTATCACACTACGGACCATCAGT  381
            |||||||||||||||||||||||||||||||||||||||||||||||||||| |||||||
Sbjct  301  TACAGGGTGAACAGTTTACCCCCCCTTATCATCAAATTTATCACACTACGGATCATCAGT  360

Query  382  AGATTTAGCTATTTTTTCTCTGCATCTTGCAGGTGCTTCTTCAATTTTAGGTGCAATCAA  441
            |||||||||||| |||||||||||||||||||||||||||||||||||||||| ||||||||
Sbjct  361  AGATTTAGCTATCTTTTCTCTGCATCTTGCAGGTGCTTCTTCAATTTTAGGCGCAATCAA  420

Query  442  TTTCATTACAACTATTGTAAATATACGATCCATTGGAATAACAATAGAACGAATACCATT  501
            ||||||||||||||||||||||||||||||||||||||||||||||||||||||||||||
Sbjct  421  TTTCATTACAACTATTGTAAATATACGATCCATTGGAATAACAATAGAACGAATACCATT  480

Query  502  ATTTGTATGATCTGTTTTAATTACTGCAATTTTATTATTATTATCTTTACCAGTCTTAGC  561
            ||||||||||||||||||||||||||||||||||||||| ||||||||||||||||||||
Sbjct  481  ATTTGTATGATCTGTTTTAATTACTGCAATTTTATTATTGTTATCTTTACCAGTCTTAGC  540

Query  562  TGGCGCAATTACAATATTATTAACTGATCGAAACTTTAATACATCATTCTTTGACCCTTC  621
            ||| ||||||||||||||||||||||||||||||||||||||||||||||||||||||||
Sbjct  541  TGGTGCAATTACAATATTATTAACTGATCGAAACTTTAATACATCATTCTTTGACCCTTC  600

Query  622  AGGAGGAGGTGACCCAATTCTATATCAACATTTATTT  658
            ||||||||||||||||||||| |||||||||||||||
Sbjct  601  AGGAGGAGGTGACCCAATTCTGTATCAACATTTATTT  637
```

>Rhipicephalus sanguineus isolate 400 cytochrome c oxidase subunit I (COX1) gene, partial cds; mitochondrial
Sequence ID: MW558150.1 Length: 657
Range 1: 1 to 639

Score:1120 bits(606), Expect:0.0,
Identities:628/639(98%), Gaps:0/639(0%), Strand: Plus/Plus

```
Query  20   GGAGCATGATCCGGAATACTAGGATTAAGAATAAGAATATTAATCCGCATAGAATTAGGT  79
            |||||| ||||||||||| |||||||||||||||||||||||||||||||||||||||||
Sbjct  1    GGAGCAGGATCCGGAATATTAGGATTAAGAATAAGAATATTAATCCGCATAGAATTAGGT  60
```

```
Query  80   CAACCAGGAACTTTAATTGGAAACGATCAAATTTATAATGTAATTGTAACAGCACATGCA  139
            ||||||||||||||||||||||||||||||||||||||||||||||||||||||||||||
Sbjct  61   CAACCAGGAACTTTAATTGGAAACGATCAAATTTATAATGTAATTGTAACAGCACATGCA  120

Query  140  TTTATTATAAttttttttATAGTTATACCAATTATAATCGGAGGATTTGGAAATTGATTA  199
            |||||||||||||||||||||||||||||||||||||||||||||||||||||| |||||
Sbjct  121  TTTATTATAATTTTTTTTATAGTTATACCAATTATAATCGGAGGATTTGGAAATGGATTA  180

Query  200  GTTCCTATTATACTAGGAGCTCCAGATATAGCATTTCCACGAATAAATAATATAAGATTT  259
            ||||||||||| ||||||||||||||||||||||||||||||||||||||||||||||||
Sbjct  181  GTTCCTATTATGCTAGGAGCTCCAGATATAGCATTTCCACGAATAAATAATATAAGATTT  240

Query  260  TGGTTACTTCCTCCTTCTCTATTTCTACTAATTAATTCTTCATTAATCGAATCAGGAGCA  319
            ||||||||||||||||||||||||||||||||||||||||||||||||||||||||||||
Sbjct  241  TGGTTACTTCCTCCTTCTCTATTTCTACTAATTAATTCTTCATTAATCGAATCAGGAGCA  300

Query  320  GGTACAGGGTGAACAGTTAcccccccTTATCATCAAATTTATCACACTACGGACCATCA  379
            ||||||||| ||||||||||||||||||||||||||||||||||||||||||| ||||||
Sbjct  301  GGTACAGGGGGAACAGTTACCCCCCCTTATCATCAAATTTATCACACTACGGGCCATCA  360

Query  380  GTAGATTTAGCTATTTTTTCTCTGCATCTTGCAGGTGCTTCTTCAATTTTAGGTGCAATC  439
            |||||||||||||| ||||||||||||||||| |||||||||||||||||||||||||||
Sbjct  361  GTAGATTTAGCTATCTTTTCTCTGCATCTTGCCGGTGCTTCTTCAATTTTAGGTGCAATC  420

Query  440  AATTTCATTACAACTATTGTAAATATACGATCCATTGGAATAACAATAGAACGAATACCA  499
            ||||||||||||||||||||||||||||||||||||||||||||||||||||||||||||
Sbjct  421  AATTTCATTACAACTATTGTAAATATACGATCCATTGGAATAACAATAGAACGAATACCA  480

Query  500  TTATTTGTATGATCTGTTTTAATTACTGCAATTTTATTATTATTATCTTTACCAGTCTTA  559
            ||||||||| ||||||||||||||||||||||||||||||||||||||||||||||||||
Sbjct  481  TTATTTGTAGGATCTGTTTTAATTACTGCAATTTTATTATTATTATCTTTACCAGTCTTA  540

Query  560  GCTGGCGCAATTACAATATTATTAACTGATCGAAACTTTAATACATCATTCTTTGACCCT  619
            ||||| ||||||||||||||||||||||||||||||||||||||||||||||||||||||
Sbjct  541  GCTGGTGCAATTACAATATTATTAACTGATCGAAACTTTAATACATCATTCTTTGACCCT  600

Query  620  TCAGGAGGAGGTGACCCAATTCTATATCAACATTTATTT  658
            |||||||| ||||||||||||||||||||||||||||||
Sbjct  601  TCAGGAGGGGGTGACCCAATTCTATATCAACATTTATTT  639
```

>Rhipicephalus linnaei isolate C2-T1 cytochrome c oxidase subunit I (COX1) gene, partial cds; mitochondrial
Sequence ID: MZ726445.1 Length: 604
Range 1: 1 to 604

Score:1116 bits(604), Expect:0.0,
Identities:604/604(100%), Gaps:0/604(0%), Strand: Plus/Plus

```
Query  1    GACAATATACTTAATTTTTGGAGCATGATCCGGAATACTAGGATTAAGAATAAGAATATT  60
            ||||||||||||||||||||||||||||||||||||||||||||||||||||||||||||
```

```
Sbjct  1    GACAATATACTTAATTTTTGGAGCATGATCCGGAATACTAGGATTAAGAATAAGAATATT  60

Query  61   AATCCGCATAGAATTAGGTCAACCAGGAACTTTAATTGGAAACGATCAAATTTATAATGT  120
            ||||||||||||||||||||||||||||||||||||||||||||||||||||||||||||
Sbjct  61   AATCCGCATAGAATTAGGTCAACCAGGAACTTTAATTGGAAACGATCAAATTTATAATGT  120

Query  121  AATTGTAACAGCACATGCATTTATTATAAttttttttATAGTTATACCAATTATAATCGG  180
            ||||||||||||||||||||||||||||||||||||||||||||||||||||||||||||
Sbjct  121  AATTGTAACAGCACATGCATTTATTATAATTTTTTTTATAGTTATACCAATTATAATCGG  180

Query  181  AGGATTTGGAAATTGATTAGTTCCTATTATACTAGGAGCTCCAGATATAGCATTTCCACG  240
            ||||||||||||||||||||||||||||||||||||||||||||||||||||||||||||
Sbjct  181  AGGATTTGGAAATTGATTAGTTCCTATTATACTAGGAGCTCCAGATATAGCATTTCCACG  240

Query  241  AATAAATAATATAAGATTTTGGTTACTTCCTCCTTCTCTATTTCTACTAATTAATTCTTC  300
            ||||||||||||||||||||||||||||||||||||||||||||||||||||||||||||
Sbjct  241  AATAAATAATATAAGATTTTGGTTACTTCCTCCTTCTCTATTTCTACTAATTAATTCTTC  300

Query  301  ATTAATCGAATCAGGAGCAGGTACAGGGTGAACAGTTTAcccccccTTATCATCAAATTT  360
            ||||||||||||||||||||||||||||||||||||||||||||||||||||||||||||
Sbjct  301  ATTAATCGAATCAGGAGCAGGTACAGGGTGAACAGTTTACCCCCCCTTATCATCAAATTT  360

Query  361  ATCACACTACGGACCATCAGTAGATTTAGCTATTTTTTCTCTGCATCTTGCAGGTGCTTC  420
            ||||||||||||||||||||||||||||||||||||||||||||||||||||||||||||
Sbjct  361  ATCACACTACGGACCATCAGTAGATTTAGCTATTTTTTCTCTGCATCTTGCAGGTGCTTC  420

Query  421  TTCAATTTTAGGTGCAATCAATTTCATTACAACTATTGTAAATATACGATCCATTGGAAT  480
            ||||||||||||||||||||||||||||||||||||||||||||||||||||||||||||
Sbjct  421  TTCAATTTTAGGTGCAATCAATTTCATTACAACTATTGTAAATATACGATCCATTGGAAT  480

Query  481  AACAATAGAACGAATACCATTATTTGTATGATCTGTTTTAATTACTGCAATTTTATTATT  540
            ||||||||||||||||||||||||||||||||||||||||||||||||||||||||||||
Sbjct  481  AACAATAGAACGAATACCATTATTTGTATGATCTGTTTTAATTACTGCAATTTTATTATT  540

Query  541  ATTATCTTTACCAGTCTTAGCTGGCGCAATTACAATATTATTAACTGATCGAAACTTTAA  600
            ||||||||||||||||||||||||||||||||||||||||||||||||||||||||||||
Sbjct  541  ATTATCTTTACCAGTCTTAGCTGGCGCAATTACAATATTATTAACTGATCGAAACTTTAA  600

Query  601  TACA  604
            ||||
Sbjct  601  TACA  604
```

>Rhipicephalus linnaei isolate C1-T1 cytochrome c oxidase subunit I (COX1) gene, partial cds; mitochondrial
Sequence ID: MZ726444.1 Length: 604
Range 1: 1 to 604

Score:1116 bits(604), Expect:0.0,
Identities:604/604(100%), Gaps:0/604(0%), Strand: Plus/Plus

```
Query  1    GACAATATACTTAATTTTTGGAGCATGATCCGGAATACTAGGATTAAGAATAAGAATATT  60
```

```
            ||||||||||||||||||||||||||||||||||||||||||||||||||||||||||||
Sbjct  1    GACAATATACTTAATTTTTGGAGCATGATCCGGAATACTAGGATTAAGAATAAGAATATT  60

Query  61   AATCCGCATAGAATTAGGTCAACCAGGAACTTTAATTGGAAACGATCAAATTTATAATGT  120
            ||||||||||||||||||||||||||||||||||||||||||||||||||||||||||||
Sbjct  61   AATCCGCATAGAATTAGGTCAACCAGGAACTTTAATTGGAAACGATCAAATTTATAATGT  120

Query  121  AATTGTAACAGCACATGCATTTATTATAAttttttttATAGTTATACCAATTATAATCGG  180
            ||||||||||||||||||||||||||||||||||||||||||||||||||||||||||||
Sbjct  121  AATTGTAACAGCACATGCATTTATTATAATTTTTTTTATAGTTATACCAATTATAATCGG  180

Query  181  AGGATTTGGAAATTGATTAGTTCCTATTATACTAGGAGCTCCAGATATAGCATTTCCACG  240
            ||||||||||||||||||||||||||||||||||||||||||||||||||||||||||||
Sbjct  181  AGGATTTGGAAATTGATTAGTTCCTATTATACTAGGAGCTCCAGATATAGCATTTCCACG  240

Query  241  AATAAATAATATAAGATTTTGGTTACTTCCTCCTTCTCTATTTCTACTAATTAATTCTTC  300
            ||||||||||||||||||||||||||||||||||||||||||||||||||||||||||||
Sbjct  241  AATAAATAATATAAGATTTTGGTTACTTCCTCCTTCTCTATTTCTACTAATTAATTCTTC  300

Query  301  ATTAATCGAATCAGGAGCAGGTACAGGGTGAACAGTTTAcccccccTTATCATCAAATTT  360
            ||||||||||||||||||||||||||||||||||||||||||||||||||||||||||||
Sbjct  301  ATTAATCGAATCAGGAGCAGGTACAGGGTGAACAGTTTACCCCCCCTTATCATCAAATTT  360

Query  361  ATCACACTACGGACCATCAGTAGATTTAGCTATTTTTTCTCTGCATCTTGCAGGTGCTTC  420
            ||||||||||||||||||||||||||||||||||||||||||||||||||||||||||||
Sbjct  361  ATCACACTACGGACCATCAGTAGATTTAGCTATTTTTTCTCTGCATCTTGCAGGTGCTTC  420

Query  421  TTCAATTTTAGGTGCAATCAATTTCATTACAACTATTGTAAATATACGATCCATTGGAAT  480
            ||||||||||||||||||||||||||||||||||||||||||||||||||||||||||||
Sbjct  421  TTCAATTTTAGGTGCAATCAATTTCATTACAACTATTGTAAATATACGATCCATTGGAAT  480

Query  481  AACAATAGAACGAATACCATTATTTGTATGATCTGTTTTAATTACTGCAATTTTATTATT  540
            ||||||||||||||||||||||||||||||||||||||||||||||||||||||||||||
Sbjct  481  AACAATAGAACGAATACCATTATTTGTATGATCTGTTTTAATTACTGCAATTTTATTATT  540

Query  541  ATTATCTTTACCAGTCTTAGCTGGCGCAATTACAATATTATTAACTGATCGAAACTTTAA  600
            ||||||||||||||||||||||||||||||||||||||||||||||||||||||||||||
Sbjct  541  ATTATCTTTACCAGTCTTAGCTGGCGCAATTACAATATTATTAACTGATCGAAACTTTAA  600

Query  601  TACA  604
            ||||
Sbjct  601  TACA  604
```

>Rhipicephalus linnaei isolate B15-T1 cytochrome c oxidase subunit I (COX1) gene, partial cds; mitochondrial
Sequence ID: MZ726443.1 Length: 604
Range 1: 1 to 604

Score:1116 bits(604), Expect:0.0,
Identities:604/604(100%), Gaps:0/604(0%), Strand: Plus/Plus

```
Query  1    GACAATATACTTAATTTTTGGAGCATGATCCGGAATACTAGGATTAAGAATAAGAATATT  60
            ||||||||||||||||||||||||||||||||||||||||||||||||||||||||||||
Sbjct  1    GACAATATACTTAATTTTTGGAGCATGATCCGGAATACTAGGATTAAGAATAAGAATATT  60

Query  61   AATCCGCATAGAATTAGGTCAACCAGGAACTTTAATTGGAAACGATCAAATTTATAATGT  120
            ||||||||||||||||||||||||||||||||||||||||||||||||||||||||||||
Sbjct  61   AATCCGCATAGAATTAGGTCAACCAGGAACTTTAATTGGAAACGATCAAATTTATAATGT  120

Query  121  AATTGTAACAGCACATGCATTTATTATAAtttttttATAGTTATACCAATTATAATCGG  180
            ||||||||||||||||||||||||||||||||||||||||||||||||||||||||||||
Sbjct  121  AATTGTAACAGCACATGCATTTATTATAATTTTTTTATAGTTATACCAATTATAATCGG  180

Query  181  AGGATTTGGAAATTGATTAGTTCCTATTATACTAGGAGCTCCAGATATAGCATTTCCACG  240
            ||||||||||||||||||||||||||||||||||||||||||||||||||||||||||||
Sbjct  181  AGGATTTGGAAATTGATTAGTTCCTATTATACTAGGAGCTCCAGATATAGCATTTCCACG  240

Query  241  AATAAATAATATAAGATTTTGGTTACTTCCTCCTTCTCTATTTCTACTAATTAATTCTTC  300
            ||||||||||||||||||||||||||||||||||||||||||||||||||||||||||||
Sbjct  241  AATAAATAATATAAGATTTTGGTTACTTCCTCCTTCTCTATTTCTACTAATTAATTCTTC  300

Query  301  ATTAATCGAATCAGGAGCAGGTACAGGGTGAACAGTTTAcccccccTTATCATCAAATTT  360
            ||||||||||||||||||||||||||||||||||||||||||||||||||||||||||||
Sbjct  301  ATTAATCGAATCAGGAGCAGGTACAGGGTGAACAGTTTACCCCCCCTTATCATCAAATTT  360

Query  361  ATCACACTACGGACCATCAGTAGATTTAGCTATTTTTTCTCTGCATCTTGCAGGTGCTTC  420
            ||||||||||||||||||||||||||||||||||||||||||||||||||||||||||||
Sbjct  361  ATCACACTACGGACCATCAGTAGATTTAGCTATTTTTTCTCTGCATCTTGCAGGTGCTTC  420

Query  421  TTCAATTTTAGGTGCAATCAATTTCATTACAACTATTGTAAATATACGATCCATTGGAAT  480
            ||||||||||||||||||||||||||||||||||||||||||||||||||||||||||||
Sbjct  421  TTCAATTTTAGGTGCAATCAATTTCATTACAACTATTGTAAATATACGATCCATTGGAAT  480

Query  481  AACAATAGAACGAATACCATTATTTGTATGATCTGTTTTAATTACTGCAATTTTATTATT  540
            ||||||||||||||||||||||||||||||||||||||||||||||||||||||||||||
Sbjct  481  AACAATAGAACGAATACCATTATTTGTATGATCTGTTTTAATTACTGCAATTTTATTATT  540

Query  541  ATTATCTTTACCAGTCTTAGCTGGCGCAATTACAATATTATTAACTGATCGAAACTTTAA  600
            ||||||||||||||||||||||||||||||||||||||||||||||||||||||||||||
Sbjct  541  ATTATCTTTACCAGTCTTAGCTGGCGCAATTACAATATTATTAACTGATCGAAACTTTAA  600

Query  601  TACA  604
            ||||
Sbjct  601  TACA  604
```

>Rhipicephalus linnaei isolate B12-T1 cytochrome c oxidase subunit I (COX1)
gene, partial cds; mitochondrial
Sequence ID: MZ726442.1 Length: 604
Range 1: 1 to 604

Score:1116 bits(604), Expect:0.0,
Identities:604/604(100%), Gaps:0/604(0%), Strand: Plus/Plus

```
Query  1    GACAATATACTTAATTTTTGGAGCATGATCCGGAATACTAGGATTAAGAATAAGAATATT  60
            ||||||||||||||||||||||||||||||||||||||||||||||||||||||||||||
Sbjct  1    GACAATATACTTAATTTTTGGAGCATGATCCGGAATACTAGGATTAAGAATAAGAATATT  60

Query  61   AATCCGCATAGAATTAGGTCAACCAGGAACTTTAATTGGAAACGATCAAATTTATAATGT  120
            ||||||||||||||||||||||||||||||||||||||||||||||||||||||||||||
Sbjct  61   AATCCGCATAGAATTAGGTCAACCAGGAACTTTAATTGGAAACGATCAAATTTATAATGT  120

Query  121  AATTGTAACAGCACATGCATTTATTATAAttttttttATAGTTATACCAATTATAATCGG  180
            ||||||||||||||||||||||||||||||||||||||||||||||||||||||||||||
Sbjct  121  AATTGTAACAGCACATGCATTTATTATAATTTTTTTTATAGTTATACCAATTATAATCGG  180

Query  181  AGGATTTGGAAATTGATTAGTTCCTATTATACTAGGAGCTCCAGATATAGCATTTCCACG  240
            ||||||||||||||||||||||||||||||||||||||||||||||||||||||||||||
Sbjct  181  AGGATTTGGAAATTGATTAGTTCCTATTATACTAGGAGCTCCAGATATAGCATTTCCACG  240

Query  241  AATAAATAATATAAGATTTTGGTTACTTCCTCCTTCTCTATTTCTACTAATTAATTCTTC  300
            ||||||||||||||||||||||||||||||||||||||||||||||||||||||||||||
Sbjct  241  AATAAATAATATAAGATTTTGGTTACTTCCTCCTTCTCTATTTCTACTAATTAATTCTTC  300

Query  301  ATTAATCGAATCAGGAGCAGGTACAGGGTGAACAGTTTAcccccccTTATCATCAAATTT  360
            ||||||||||||||||||||||||||||||||||||||||||||||||||||||||||||
Sbjct  301  ATTAATCGAATCAGGAGCAGGTACAGGGTGAACAGTTTACCCCCCCTTATCATCAAATTT  360

Query  361  ATCACACTACGGACCATCAGTAGATTTAGCTATTTTTTCTCTGCATCTTGCAGGTGCTTC  420
            ||||||||||||||||||||||||||||||||||||||||||||||||||||||||||||
Sbjct  361  ATCACACTACGGACCATCAGTAGATTTAGCTATTTTTTCTCTGCATCTTGCAGGTGCTTC  420

Query  421  TTCAATTTTAGGTGCAATCAATTTCATTACAACTATTGTAAATATACGATCCATTGGAAT  480
            ||||||||||||||||||||||||||||||||||||||||||||||||||||||||||||
Sbjct  421  TTCAATTTTAGGTGCAATCAATTTCATTACAACTATTGTAAATATACGATCCATTGGAAT  480

Query  481  AACAATAGAACGAATACCATTATTTGTATGATCTGTTTTAATTACTGCAATTTTATTATT  540
            ||||||||||||||||||||||||||||||||||||||||||||||||||||||||||||
Sbjct  481  AACAATAGAACGAATACCATTATTTGTATGATCTGTTTTAATTACTGCAATTTTATTATT  540

Query  541  ATTATCTTTACCAGTCTTAGCTGGCGCAATTACAATATTATTAACTGATCGAAACTTTAA  600
            ||||||||||||||||||||||||||||||||||||||||||||||||||||||||||||
Sbjct  541  ATTATCTTTACCAGTCTTAGCTGGCGCAATTACAATATTATTAACTGATCGAAACTTTAA  600

Query  601  TACA  604
            ||||
Sbjct  601  TACA  604
```

>Rhipicephalus linnaei isolate B8-T1 cytochrome c oxidase subunit I (COX1) gene, partial cds; mitochondrial
Sequence ID: MZ726441.1 Length: 604
Range 1: 1 to 604

Score:1116 bits(604), Expect:0.0,

```
Identities:604/604(100%),  Gaps:0/604(0%), Strand: Plus/Plus

Query  1    GACAATATACTTAATTTTTGGAGCATGATCCGGAATACTAGGATTAAGAATAAGAATATT  60
            ||||||||||||||||||||||||||||||||||||||||||||||||||||||||||||
Sbjct  1    GACAATATACTTAATTTTTGGAGCATGATCCGGAATACTAGGATTAAGAATAAGAATATT  60

Query  61   AATCCGCATAGAATTAGGTCAACCAGGAACTTTAATTGGAAACGATCAAATTTATAATGT  120
            ||||||||||||||||||||||||||||||||||||||||||||||||||||||||||||
Sbjct  61   AATCCGCATAGAATTAGGTCAACCAGGAACTTTAATTGGAAACGATCAAATTTATAATGT  120

Query  121  AATTGTAACAGCACATGCATTTATTATAAttttttttATAGTTATACCAATTATAATCGG  180
            ||||||||||||||||||||||||||||||||||||||||||||||||||||||||||||
Sbjct  121  AATTGTAACAGCACATGCATTTATTATAATTTTTTTTATAGTTATACCAATTATAATCGG  180

Query  181  AGGATTTGGAAATTGATTAGTTCCTATTATACTAGGAGCTCCAGATATAGCATTTCCACG  240
            ||||||||||||||||||||||||||||||||||||||||||||||||||||||||||||
Sbjct  181  AGGATTTGGAAATTGATTAGTTCCTATTATACTAGGAGCTCCAGATATAGCATTTCCACG  240

Query  241  AATAAATAATATAAGATTTTGGTTACTTCCTCCTTCTCTATTTCTACTAATTAATTCTTC  300
            ||||||||||||||||||||||||||||||||||||||||||||||||||||||||||||
Sbjct  241  AATAAATAATATAAGATTTTGGTTACTTCCTCCTTCTCTATTTCTACTAATTAATTCTTC  300

Query  301  ATTAATCGAATCAGGAGCAGGTACAGGGTGAACAGTTTAcccccccTTATCATCAAATTT  360
            ||||||||||||||||||||||||||||||||||||||||||||||||||||||||||||
Sbjct  301  ATTAATCGAATCAGGAGCAGGTACAGGGTGAACAGTTTACCCCCCCTTATCATCAAATTT  360

Query  361  ATCACACTACGGACCATCAGTAGATTTAGCTATTTTTTCTCTGCATCTTGCAGGTGCTTC  420
            ||||||||||||||||||||||||||||||||||||||||||||||||||||||||||||
Sbjct  361  ATCACACTACGGACCATCAGTAGATTTAGCTATTTTTTCTCTGCATCTTGCAGGTGCTTC  420

Query  421  TTCAATTTTAGGTGCAATCAATTTCATTACAACTATTGTAAATATACGATCCATTGGAAT  480
            ||||||||||||||||||||||||||||||||||||||||||||||||||||||||||||
Sbjct  421  TTCAATTTTAGGTGCAATCAATTTCATTACAACTATTGTAAATATACGATCCATTGGAAT  480

Query  481  AACAATAGAACGAATACCATTATTTGTATGATCTGTTTTAATTACTGCAATTTTATTATT  540
            ||||||||||||||||||||||||||||||||||||||||||||||||||||||||||||
Sbjct  481  AACAATAGAACGAATACCATTATTTGTATGATCTGTTTTAATTACTGCAATTTTATTATT  540

Query  541  ATTATCTTTACCAGTCTTAGCTGGCGCAATTACAATATTATTAACTGATCGAAACTTTAA  600
            ||||||||||||||||||||||||||||||||||||||||||||||||||||||||||||
Sbjct  541  ATTATCTTTACCAGTCTTAGCTGGCGCAATTACAATATTATTAACTGATCGAAACTTTAA  600

Query  601  TACA  604
            ||||
Sbjct  601  TACA  604
```

```
>Rhipicephalus linnaei isolate B7-T3 cytochrome c oxidase subunit I (COX1) gene,
partial cds; mitochondrial
Sequence ID: MZ726440.1 Length: 604
Range 1: 1 to 604
```

Score:1116 bits(604), Expect:0.0,
Identities:604/604(100%), Gaps:0/604(0%), Strand: Plus/Plus

```
Query  1    GACAATATACTTAATTTTTGGAGCATGATCCGGAATACTAGGATTAAGAATAAGAATATT  60
            ||||||||||||||||||||||||||||||||||||||||||||||||||||||||||||
Sbjct  1    GACAATATACTTAATTTTTGGAGCATGATCCGGAATACTAGGATTAAGAATAAGAATATT  60

Query  61   AATCCGCATAGAATTAGGTCAACCAGGAACTTTAATTGGAAACGATCAAATTTATAATGT  120
            ||||||||||||||||||||||||||||||||||||||||||||||||||||||||||||
Sbjct  61   AATCCGCATAGAATTAGGTCAACCAGGAACTTTAATTGGAAACGATCAAATTTATAATGT  120

Query  121  AATTGTAACAGCACATGCATTTATTATAAtttttttATAGTTATACCAATTATAATCGG  180
            ||||||||||||||||||||||||||||||||||||||||||||||||||||||||||||
Sbjct  121  AATTGTAACAGCACATGCATTTATTATAATTTTTTTATAGTTATACCAATTATAATCGG  180

Query  181  AGGATTTGGAAATTGATTAGTTCCTATTATACTAGGAGCTCCAGATATAGCATTTCCACG  240
            ||||||||||||||||||||||||||||||||||||||||||||||||||||||||||||
Sbjct  181  AGGATTTGGAAATTGATTAGTTCCTATTATACTAGGAGCTCCAGATATAGCATTTCCACG  240

Query  241  AATAAATAATATAAGATTTTGGTTACTTCCTCCTTCTCTATTTCTACTAATTAATTCTTC  300
            ||||||||||||||||||||||||||||||||||||||||||||||||||||||||||||
Sbjct  241  AATAAATAATATAAGATTTTGGTTACTTCCTCCTTCTCTATTTCTACTAATTAATTCTTC  300

Query  301  ATTAATCGAATCAGGAGCAGGTACAGGGTGAACAGTTTAcccccccTTATCATCAAATTT  360
            ||||||||||||||||||||||||||||||||||||||||||||||||||||||||||||
Sbjct  301  ATTAATCGAATCAGGAGCAGGTACAGGGTGAACAGTTTACCCCCCCTTATCATCAAATTT  360

Query  361  ATCACACTACGGACCATCAGTAGATTTAGCTATTTTTTCTCTGCATCTTGCAGGTGCTTC  420
            ||||||||||||||||||||||||||||||||||||||||||||||||||||||||||||
Sbjct  361  ATCACACTACGGACCATCAGTAGATTTAGCTATTTTTTCTCTGCATCTTGCAGGTGCTTC  420

Query  421  TTCAATTTTAGGTGCAATCAATTTCATTACAACTATTGTAAATATACGATCCATTGGAAT  480
            ||||||||||||||||||||||||||||||||||||||||||||||||||||||||||||
Sbjct  421  TTCAATTTTAGGTGCAATCAATTTCATTACAACTATTGTAAATATACGATCCATTGGAAT  480

Query  481  AACAATAGAACGAATACCATTATTTGTATGATCTGTTTTAATTACTGCAATTTTATTATT  540
            ||||||||||||||||||||||||||||||||||||||||||||||||||||||||||||
Sbjct  481  AACAATAGAACGAATACCATTATTTGTATGATCTGTTTTAATTACTGCAATTTTATTATT  540

Query  541  ATTATCTTTACCAGTCTTAGCTGGCGCAATTACAATATTATTAACTGATCGAAACTTTAA  600
            ||||||||||||||||||||||||||||||||||||||||||||||||||||||||||||
Sbjct  541  ATTATCTTTACCAGTCTTAGCTGGCGCAATTACAATATTATTAACTGATCGAAACTTTAA  600

Query  601  TACA  604
            ||||
Sbjct  601  TACA  604
```

>Rhipicephalus linnaei isolate B7-T2 cytochrome c oxidase subunit I (COX1) gene, partial cds; mitochondrial
Sequence ID: MZ726439.1 Length: 604
Range 1: 1 to 604

```
Score:1116 bits(604), Expect:0.0,
Identities:604/604(100%),  Gaps:0/604(0%), Strand: Plus/Plus

Query  1    GACAATATACTTAATTTTTGGAGCATGATCCGGAATACTAGGATTAAGAATAAGAATATT  60
            ||||||||||||||||||||||||||||||||||||||||||||||||||||||||||||
Sbjct  1    GACAATATACTTAATTTTTGGAGCATGATCCGGAATACTAGGATTAAGAATAAGAATATT  60

Query  61   AATCCGCATAGAATTAGGTCAACCAGGAACTTTAATTGGAAACGATCAAATTTATAATGT  120
            ||||||||||||||||||||||||||||||||||||||||||||||||||||||||||||
Sbjct  61   AATCCGCATAGAATTAGGTCAACCAGGAACTTTAATTGGAAACGATCAAATTTATAATGT  120

Query  121  AATTGTAACAGCACATGCATTTATTATAAttttttttATAGTTATACCAATTATAATCGG  180
            ||||||||||||||||||||||||||||||||||||||||||||||||||||||||||||
Sbjct  121  AATTGTAACAGCACATGCATTTATTATAATTTTTTTTATAGTTATACCAATTATAATCGG  180

Query  181  AGGATTTGGAAATTGATTAGTTCCTATTATACTAGGAGCTCCAGATATAGCATTTCCACG  240
            ||||||||||||||||||||||||||||||||||||||||||||||||||||||||||||
Sbjct  181  AGGATTTGGAAATTGATTAGTTCCTATTATACTAGGAGCTCCAGATATAGCATTTCCACG  240

Query  241  AATAAATAATATAAGATTTTGGTTACTTCCTCCTTCTCTATTTCTACTAATTAATTCTTC  300
            ||||||||||||||||||||||||||||||||||||||||||||||||||||||||||||
Sbjct  241  AATAAATAATATAAGATTTTGGTTACTTCCTCCTTCTCTATTTCTACTAATTAATTCTTC  300

Query  301  ATTAATCGAATCAGGAGCAGGTACAGGGTGAACAGTTTAcccccccTTATCATCAAATTT  360
            ||||||||||||||||||||||||||||||||||||||||||||||||||||||||||||
Sbjct  301  ATTAATCGAATCAGGAGCAGGTACAGGGTGAACAGTTTACCCCCCCTTATCATCAAATTT  360

Query  361  ATCACACTACGGACCATCAGTAGATTTAGCTATTTTTTCTCTGCATCTTGCAGGTGCTTC  420
            ||||||||||||||||||||||||||||||||||||||||||||||||||||||||||||
Sbjct  361  ATCACACTACGGACCATCAGTAGATTTAGCTATTTTTTCTCTGCATCTTGCAGGTGCTTC  420

Query  421  TTCAATTTTAGGTGCAATCAATTTCATTACAACTATTGTAAATATACGATCCATTGGAAT  480
            ||||||||||||||||||||||||||||||||||||||||||||||||||||||||||||
Sbjct  421  TTCAATTTTAGGTGCAATCAATTTCATTACAACTATTGTAAATATACGATCCATTGGAAT  480

Query  481  AACAATAGAACGAATACCATTATTTGTATGATCTGTTTTAATTACTGCAATTTTATTATT  540
            ||||||||||||||||||||||||||||||||||||||||||||||||||||||||||||
Sbjct  481  AACAATAGAACGAATACCATTATTTGTATGATCTGTTTTAATTACTGCAATTTTATTATT  540

Query  541  ATTATCTTTACCAGTCTTAGCTGGCGCAATTACAATATTATTAACTGATCGAAACTTTAA  600
            ||||||||||||||||||||||||||||||||||||||||||||||||||||||||||||
Sbjct  541  ATTATCTTTACCAGTCTTAGCTGGCGCAATTACAATATTATTAACTGATCGAAACTTTAA  600

Query  601  TACA  604
            ||||
Sbjct  601  TACA  604
```

>Rhipicephalus linnaei isolate B7-T1 cytochrome c oxidase subunit I (COX1) gene, partial cds; mitochondrial
Sequence ID: MZ726438.1 Length: 604

```
Range 1: 1 to 604

Score:1116 bits(604), Expect:0.0,
Identities:604/604(100%),  Gaps:0/604(0%), Strand: Plus/Plus

Query  1    GACAATATACTTAATTTTTGGAGCATGATCCGGAATACTAGGATTAAGAATAAGAATATT  60
            ||||||||||||||||||||||||||||||||||||||||||||||||||||||||||||
Sbjct  1    GACAATATACTTAATTTTTGGAGCATGATCCGGAATACTAGGATTAAGAATAAGAATATT  60

Query  61   AATCCGCATAGAATTAGGTCAACCAGGAACTTTAATTGGAAACGATCAAATTTATAATGT  120
            ||||||||||||||||||||||||||||||||||||||||||||||||||||||||||||
Sbjct  61   AATCCGCATAGAATTAGGTCAACCAGGAACTTTAATTGGAAACGATCAAATTTATAATGT  120

Query  121  AATTGTAACAGCACATGCATTTATTATAAttttttttATAGTTATACCAATTATAATCGG  180
            ||||||||||||||||||||||||||||||||||||||||||||||||||||||||||||
Sbjct  121  AATTGTAACAGCACATGCATTTATTATAATTTTTTTTATAGTTATACCAATTATAATCGG  180

Query  181  AGGATTTGGAAATTGATTAGTTCCTATTATACTAGGAGCTCCAGATATAGCATTTCCACG  240
            ||||||||||||||||||||||||||||||||||||||||||||||||||||||||||||
Sbjct  181  AGGATTTGGAAATTGATTAGTTCCTATTATACTAGGAGCTCCAGATATAGCATTTCCACG  240

Query  241  AATAAATAATATAAGATTTTGGTTACTTCCTCCTTCTCTATTTCTACTAATTAATTCTTC  300
            ||||||||||||||||||||||||||||||||||||||||||||||||||||||||||||
Sbjct  241  AATAAATAATATAAGATTTTGGTTACTTCCTCCTTCTCTATTTCTACTAATTAATTCTTC  300

Query  301  ATTAATCGAATCAGGAGCAGGTACAGGGTGAACAGTTTAcccccccTTATCATCAAATTT  360
            ||||||||||||||||||||||||||||||||||||||||||||||||||||||||||||
Sbjct  301  ATTAATCGAATCAGGAGCAGGTACAGGGTGAACAGTTTACCCCCCCTTATCATCAAATTT  360

Query  361  ATCACACTACGGACCATCAGTAGATTTAGCTATTTTTTCTCTGCATCTTGCAGGTGCTTC  420
            ||||||||||||||||||||||||||||||||||||||||||||||||||||||||||||
Sbjct  361  ATCACACTACGGACCATCAGTAGATTTAGCTATTTTTTCTCTGCATCTTGCAGGTGCTTC  420

Query  421  TTCAATTTTAGGTGCAATCAATTTCATTACAACTATTGTAAATATACGATCCATTGGAAT  480
            ||||||||||||||||||||||||||||||||||||||||||||||||||||||||||||
Sbjct  421  TTCAATTTTAGGTGCAATCAATTTCATTACAACTATTGTAAATATACGATCCATTGGAAT  480

Query  481  AACAATAGAACGAATACCATTATTTGTATGATCTGTTTTAATTACTGCAATTTTATTATT  540
            ||||||||||||||||||||||||||||||||||||||||||||||||||||||||||||
Sbjct  481  AACAATAGAACGAATACCATTATTTGTATGATCTGTTTTAATTACTGCAATTTTATTATT  540

Query  541  ATTATCTTTACCAGTCTTAGCTGGCGCAATTACAATATTATTAACTGATCGAAACTTTAA  600
            ||||||||||||||||||||||||||||||||||||||||||||||||||||||||||||
Sbjct  541  ATTATCTTTACCAGTCTTAGCTGGCGCAATTACAATATTATTAACTGATCGAAACTTTAA  600

Query  601  TACA  604
            ||||
Sbjct  601  TACA  604
```

>Rhipicephalus linnaei isolate B6-T1 cytochrome c oxidase subunit I (COX1) gene, partial cds; mitochondrial

```
Sequence ID: MZ726437.1 Length: 604
Range 1: 1 to 604

Score:1116 bits(604), Expect:0.0,
Identities:604/604(100%),  Gaps:0/604(0%), Strand: Plus/Plus

Query  1    GACAATATACTTAATTTTTGGAGCATGATCCGGAATACTAGGATTAAGAATAAGAATATT  60
            ||||||||||||||||||||||||||||||||||||||||||||||||||||||||||||
Sbjct  1    GACAATATACTTAATTTTTGGAGCATGATCCGGAATACTAGGATTAAGAATAAGAATATT  60

Query  61   AATCCGCATAGAATTAGGTCAACCAGGAACTTTAATTGGAAACGATCAAATTTATAATGT  120
            ||||||||||||||||||||||||||||||||||||||||||||||||||||||||||||
Sbjct  61   AATCCGCATAGAATTAGGTCAACCAGGAACTTTAATTGGAAACGATCAAATTTATAATGT  120

Query  121  AATTGTAACAGCACATGCATTTATTATAAttttttttATAGTTATACCAATTATAATCGG  180
            ||||||||||||||||||||||||||||||||||||||||||||||||||||||||||||
Sbjct  121  AATTGTAACAGCACATGCATTTATTATAATTTTTTTTATAGTTATACCAATTATAATCGG  180

Query  181  AGGATTTGGAAATTGATTAGTTCCTATTATACTAGGAGCTCCAGATATAGCATTTCCACG  240
            ||||||||||||||||||||||||||||||||||||||||||||||||||||||||||||
Sbjct  181  AGGATTTGGAAATTGATTAGTTCCTATTATACTAGGAGCTCCAGATATAGCATTTCCACG  240

Query  241  AATAAATAATATAAGATTTTGGTTACTTCCTCCTTCTCTATTTCTACTAATTAATTCTTC  300
            ||||||||||||||||||||||||||||||||||||||||||||||||||||||||||||
Sbjct  241  AATAAATAATATAAGATTTTGGTTACTTCCTCCTTCTCTATTTCTACTAATTAATTCTTC  300

Query  301  ATTAATCGAATCAGGAGCAGGTACAGGGTGAACAGTTTAcccccccTTATCATCAAATTT  360
            ||||||||||||||||||||||||||||||||||||||||||||||||||||||||||||
Sbjct  301  ATTAATCGAATCAGGAGCAGGTACAGGGTGAACAGTTTACCCCCCCTTATCATCAAATTT  360

Query  361  ATCACACTACGGACCATCAGTAGATTTAGCTATTTTTTCTCTGCATCTTGCAGGTGCTTC  420
            ||||||||||||||||||||||||||||||||||||||||||||||||||||||||||||
Sbjct  361  ATCACACTACGGACCATCAGTAGATTTAGCTATTTTTTCTCTGCATCTTGCAGGTGCTTC  420

Query  421  TTCAATTTTAGGTGCAATCAATTTCATTACAACTATTGTAAATATACGATCCATTGGAAT  480
            ||||||||||||||||||||||||||||||||||||||||||||||||||||||||||||
Sbjct  421  TTCAATTTTAGGTGCAATCAATTTCATTACAACTATTGTAAATATACGATCCATTGGAAT  480

Query  481  AACAATAGAACGAATACCATTATTTGTATGATCTGTTTTAATTACTGCAATTTTATTATT  540
            ||||||||||||||||||||||||||||||||||||||||||||||||||||||||||||
Sbjct  481  AACAATAGAACGAATACCATTATTTGTATGATCTGTTTTAATTACTGCAATTTTATTATT  540

Query  541  ATTATCTTTACCAGTCTTAGCTGGCGCAATTACAATATTATTAACTGATCGAAACTTTAA  600
            ||||||||||||||||||||||||||||||||||||||||||||||||||||||||||||
Sbjct  541  ATTATCTTTACCAGTCTTAGCTGGCGCAATTACAATATTATTAACTGATCGAAACTTTAA  600

Query  601  TACA  604
            ||||
Sbjct  601  TACA  604
```

>Rhipicephalus linnaei isolate B4-T1 cytochrome c oxidase subunit I (COX1) gene,

partial cds; mitochondrial
Sequence ID: MZ726436.1 Length: 604
Range 1: 1 to 604

Score:1116 bits(604), Expect:0.0,
Identities:604/604(100%), Gaps:0/604(0%), Strand: Plus/Plus

```
Query  1    GACAATATACTTAATTTTTGGAGCATGATCCGGAATACTAGGATTAAGAATAAGAATATT  60
            ||||||||||||||||||||||||||||||||||||||||||||||||||||||||||||
Sbjct  1    GACAATATACTTAATTTTTGGAGCATGATCCGGAATACTAGGATTAAGAATAAGAATATT  60

Query  61   AATCCGCATAGAATTAGGTCAACCAGGAACTTTAATTGGAAACGATCAAATTTATAATGT  120
            ||||||||||||||||||||||||||||||||||||||||||||||||||||||||||||
Sbjct  61   AATCCGCATAGAATTAGGTCAACCAGGAACTTTAATTGGAAACGATCAAATTTATAATGT  120

Query  121  AATTGTAACAGCACATGCATTTATTATAAtttttttATAGTTATACCAATTATAATCGG  180
            ||||||||||||||||||||||||||||||||||||||||||||||||||||||||||||
Sbjct  121  AATTGTAACAGCACATGCATTTATTATAATTTTTTTATAGTTATACCAATTATAATCGG  180

Query  181  AGGATTTGGAAATTGATTAGTTCCTATTATACTAGGAGCTCCAGATATAGCATTTCCACG  240
            ||||||||||||||||||||||||||||||||||||||||||||||||||||||||||||
Sbjct  181  AGGATTTGGAAATTGATTAGTTCCTATTATACTAGGAGCTCCAGATATAGCATTTCCACG  240

Query  241  AATAAATAATATAAGATTTTGGTTACTTCCTCCTTCTCTATTTCTACTAATTAATTCTTC  300
            ||||||||||||||||||||||||||||||||||||||||||||||||||||||||||||
Sbjct  241  AATAAATAATATAAGATTTTGGTTACTTCCTCCTTCTCTATTTCTACTAATTAATTCTTC  300

Query  301  ATTAATCGAATCAGGAGCAGGTACAGGGTGAACAGTTTAcccccccTTATCATCAAATTT  360
            ||||||||||||||||||||||||||||||||||||||||||||||||||||||||||||
Sbjct  301  ATTAATCGAATCAGGAGCAGGTACAGGGTGAACAGTTTACCCCCCCTTATCATCAAATTT  360

Query  361  ATCACACTACGGACCATCAGTAGATTTAGCTATTTTTTCTCTGCATCTTGCAGGTGCTTC  420
            ||||||||||||||||||||||||||||||||||||||||||||||||||||||||||||
Sbjct  361  ATCACACTACGGACCATCAGTAGATTTAGCTATTTTTTCTCTGCATCTTGCAGGTGCTTC  420

Query  421  TTCAATTTTAGGTGCAATCAATTTCATTACAACTATTGTAAATATACGATCCATTGGAAT  480
            ||||||||||||||||||||||||||||||||||||||||||||||||||||||||||||
Sbjct  421  TTCAATTTTAGGTGCAATCAATTTCATTACAACTATTGTAAATATACGATCCATTGGAAT  480

Query  481  AACAATAGAACGAATACCATTATTTGTATGATCTGTTTTAATTACTGCAATTTTATTATT  540
            ||||||||||||||||||||||||||||||||||||||||||||||||||||||||||||
Sbjct  481  AACAATAGAACGAATACCATTATTTGTATGATCTGTTTTAATTACTGCAATTTTATTATT  540

Query  541  ATTATCTTTACCAGTCTTAGCTGGCGCAATTACAATATTATTAACTGATCGAAACTTTAA  600
            ||||||||||||||||||||||||||||||||||||||||||||||||||||||||||||
Sbjct  541  ATTATCTTTACCAGTCTTAGCTGGCGCAATTACAATATTATTAACTGATCGAAACTTTAA  600

Query  601  TACA  604
            ||||
Sbjct  601  TACA  604
```

>Rhipicephalus linnaei isolate B3-T1 cytochrome c oxidase subunit I (COX1) gene, partial cds; mitochondrial
Sequence ID: MZ726435.1 Length: 604
Range 1: 1 to 604

Score:1116 bits(604), Expect:0.0,
Identities:604/604(100%), Gaps:0/604(0%), Strand: Plus/Plus

```
Query  1    GACAATATACTTAATTTTTGGAGCATGATCCGGAATACTAGGATTAAGAATAAGAATATT  60
            ||||||||||||||||||||||||||||||||||||||||||||||||||||||||||||
Sbjct  1    GACAATATACTTAATTTTTGGAGCATGATCCGGAATACTAGGATTAAGAATAAGAATATT  60

Query  61   AATCCGCATAGAATTAGGTCAACCAGGAACTTTAATTGGAAACGATCAAATTTATAATGT  120
            ||||||||||||||||||||||||||||||||||||||||||||||||||||||||||||
Sbjct  61   AATCCGCATAGAATTAGGTCAACCAGGAACTTTAATTGGAAACGATCAAATTTATAATGT  120

Query  121  AATTGTAACAGCACATGCATTTATTATAAttttttttATAGTTATACCAATTATAATCGG  180
            ||||||||||||||||||||||||||||||||||||||||||||||||||||||||||||
Sbjct  121  AATTGTAACAGCACATGCATTTATTATAATTTTTTTTATAGTTATACCAATTATAATCGG  180

Query  181  AGGATTTGGAAATTGATTAGTTCCTATTATACTAGGAGCTCCAGATATAGCATTTCCACG  240
            ||||||||||||||||||||||||||||||||||||||||||||||||||||||||||||
Sbjct  181  AGGATTTGGAAATTGATTAGTTCCTATTATACTAGGAGCTCCAGATATAGCATTTCCACG  240

Query  241  AATAAATAATATAAGATTTTGGTTACTTCCTCCTTCTCTATTTCTACTAATTAATTCTTC  300
            ||||||||||||||||||||||||||||||||||||||||||||||||||||||||||||
Sbjct  241  AATAAATAATATAAGATTTTGGTTACTTCCTCCTTCTCTATTTCTACTAATTAATTCTTC  300

Query  301  ATTAATCGAATCAGGAGCAGGTACAGGGTGAACAGTTTAcccccccTTATCATCAAATTT  360
            ||||||||||||||||||||||||||||||||||||||||||||||||||||||||||||
Sbjct  301  ATTAATCGAATCAGGAGCAGGTACAGGGTGAACAGTTTACCCCCCCTTATCATCAAATTT  360

Query  361  ATCACACTACGGACCATCAGTAGATTTAGCTATTTTTTCTCTGCATCTTGCAGGTGCTTC  420
            ||||||||||||||||||||||||||||||||||||||||||||||||||||||||||||
Sbjct  361  ATCACACTACGGACCATCAGTAGATTTAGCTATTTTTTCTCTGCATCTTGCAGGTGCTTC  420

Query  421  TTCAATTTTAGGTGCAATCAATTTCATTACAACTATTGTAAATATACGATCCATTGGAAT  480
            ||||||||||||||||||||||||||||||||||||||||||||||||||||||||||||
Sbjct  421  TTCAATTTTAGGTGCAATCAATTTCATTACAACTATTGTAAATATACGATCCATTGGAAT  480

Query  481  AACAATAGAACGAATACCATTATTTGTATGATCTGTTTTAATTACTGCAATTTTATTATT  540
            ||||||||||||||||||||||||||||||||||||||||||||||||||||||||||||
Sbjct  481  AACAATAGAACGAATACCATTATTTGTATGATCTGTTTTAATTACTGCAATTTTATTATT  540

Query  541  ATTATCTTTACCAGTCTTAGCTGGCGCAATTACAATATTATTAACTGATCGAAACTTTAA  600
            ||||||||||||||||||||||||||||||||||||||||||||||||||||||||||||
Sbjct  541  ATTATCTTTACCAGTCTTAGCTGGCGCAATTACAATATTATTAACTGATCGAAACTTTAA  600

Query  601  TACA  604
            ||||
Sbjct  601  TACA  604
```

>Rhipicephalus linnaei isolate B2-T1 cytochrome c oxidase subunit I (COX1) gene, partial cds; mitochondrial
Sequence ID: MZ726434.1 Length: 604
Range 1: 1 to 604

Score:1116 bits(604), Expect:0.0,
Identities:604/604(100%), Gaps:0/604(0%), Strand: Plus/Plus

```
Query  1    GACAATATACTTAATTTTTGGAGCATGATCCGGAATACTAGGATTAAGAATAAGAATATT  60
            ||||||||||||||||||||||||||||||||||||||||||||||||||||||||||||
Sbjct  1    GACAATATACTTAATTTTTGGAGCATGATCCGGAATACTAGGATTAAGAATAAGAATATT  60

Query  61   AATCCGCATAGAATTAGGTCAACCAGGAACTTTAATTGGAAACGATCAAATTTATAATGT  120
            ||||||||||||||||||||||||||||||||||||||||||||||||||||||||||||
Sbjct  61   AATCCGCATAGAATTAGGTCAACCAGGAACTTTAATTGGAAACGATCAAATTTATAATGT  120

Query  121  AATTGTAACAGCACATGCATTTATTATAAttttttttATAGTTATACCAATTATAATCGG  180
            ||||||||||||||||||||||||||||||||||||||||||||||||||||||||||||
Sbjct  121  AATTGTAACAGCACATGCATTTATTATAATTTTTTTTATAGTTATACCAATTATAATCGG  180

Query  181  AGGATTTGGAAATTGATTAGTTCCTATTATACTAGGAGCTCCAGATATAGCATTTCCACG  240
            ||||||||||||||||||||||||||||||||||||||||||||||||||||||||||||
Sbjct  181  AGGATTTGGAAATTGATTAGTTCCTATTATACTAGGAGCTCCAGATATAGCATTTCCACG  240

Query  241  AATAAATAATATAAGATTTTGGTTACTTCCTCCTTCTCTATTTCTACTAATTAATTCTTC  300
            ||||||||||||||||||||||||||||||||||||||||||||||||||||||||||||
Sbjct  241  AATAAATAATATAAGATTTTGGTTACTTCCTCCTTCTCTATTTCTACTAATTAATTCTTC  300

Query  301  ATTAATCGAATCAGGAGCAGGTACAGGGTGAACAGTTTAcccccccTTATCATCAAATTT  360
            ||||||||||||||||||||||||||||||||||||||||||||||||||||||||||||
Sbjct  301  ATTAATCGAATCAGGAGCAGGTACAGGGTGAACAGTTTACCCCCCCTTATCATCAAATTT  360

Query  361  ATCACACTACGGACCATCAGTAGATTTAGCTATTTTTTCTCTGCATCTTGCAGGTGCTTC  420
            ||||||||||||||||||||||||||||||||||||||||||||||||||||||||||||
Sbjct  361  ATCACACTACGGACCATCAGTAGATTTAGCTATTTTTTCTCTGCATCTTGCAGGTGCTTC  420

Query  421  TTCAATTTTAGGTGCAATCAATTTCATTACAACTATTGTAAATATACGATCCATTGGAAT  480
            ||||||||||||||||||||||||||||||||||||||||||||||||||||||||||||
Sbjct  421  TTCAATTTTAGGTGCAATCAATTTCATTACAACTATTGTAAATATACGATCCATTGGAAT  480

Query  481  AACAATAGAACGAATACCATTATTTGTATGATCTGTTTTAATTACTGCAATTTTATTATT  540
            ||||||||||||||||||||||||||||||||||||||||||||||||||||||||||||
Sbjct  481  AACAATAGAACGAATACCATTATTTGTATGATCTGTTTTAATTACTGCAATTTTATTATT  540

Query  541  ATTATCTTTACCAGTCTTAGCTGGCGCAATTACAATATTATTAACTGATCGAAACTTTAA  600
            ||||||||||||||||||||||||||||||||||||||||||||||||||||||||||||
Sbjct  541  ATTATCTTTACCAGTCTTAGCTGGCGCAATTACAATATTATTAACTGATCGAAACTTTAA  600

Query  601  TACA  604
            ||||
Sbjct  601  TACA  604
```

>Rhipicephalus linnaei isolate A7-T1 cytochrome c oxidase subunit I (COX1) gene, partial cds; mitochondrial
Sequence ID: MZ726433.1 Length: 604
Range 1: 1 to 604

Score:1116 bits(604), Expect:0.0,
Identities:604/604(100%), Gaps:0/604(0%), Strand: Plus/Plus

```
Query  1    GACAATATACTTAATTTTTGGAGCATGATCCGGAATACTAGGATTAAGAATAAGAATATT  60
            ||||||||||||||||||||||||||||||||||||||||||||||||||||||||||||
Sbjct  1    GACAATATACTTAATTTTTGGAGCATGATCCGGAATACTAGGATTAAGAATAAGAATATT  60

Query  61   AATCCGCATAGAATTAGGTCAACCAGGAACTTTAATTGGAAACGATCAAATTTATAATGT  120
            ||||||||||||||||||||||||||||||||||||||||||||||||||||||||||||
Sbjct  61   AATCCGCATAGAATTAGGTCAACCAGGAACTTTAATTGGAAACGATCAAATTTATAATGT  120

Query  121  AATTGTAACAGCACATGCATTTATTATAAttttttttATAGTTATACCAATTATAATCGG  180
            ||||||||||||||||||||||||||||||||||||||||||||||||||||||||||||
Sbjct  121  AATTGTAACAGCACATGCATTTATTATAATTTTTTTTATAGTTATACCAATTATAATCGG  180

Query  181  AGGATTTGGAAATTGATTAGTTCCTATTATACTAGGAGCTCCAGATATAGCATTTCCACG  240
            ||||||||||||||||||||||||||||||||||||||||||||||||||||||||||||
Sbjct  181  AGGATTTGGAAATTGATTAGTTCCTATTATACTAGGAGCTCCAGATATAGCATTTCCACG  240

Query  241  AATAAATAATATAAGATTTTGGTTACTTCCTCCTTCTCTATTTCTACTAATTAATTCTTC  300
            ||||||||||||||||||||||||||||||||||||||||||||||||||||||||||||
Sbjct  241  AATAAATAATATAAGATTTTGGTTACTTCCTCCTTCTCTATTTCTACTAATTAATTCTTC  300

Query  301  ATTAATCGAATCAGGAGCAGGTACAGGGTGAACAGTTTAcccccccTTATCATCAAATTT  360
            ||||||||||||||||||||||||||||||||||||||||||||||||||||||||||||
Sbjct  301  ATTAATCGAATCAGGAGCAGGTACAGGGTGAACAGTTTACCCCCCCTTATCATCAAATTT  360

Query  361  ATCACACTACGGACCATCAGTAGATTTAGCTATTTTTTCTCTGCATCTTGCAGGTGCTTC  420
            ||||||||||||||||||||||||||||||||||||||||||||||||||||||||||||
Sbjct  361  ATCACACTACGGACCATCAGTAGATTTAGCTATTTTTTCTCTGCATCTTGCAGGTGCTTC  420

Query  421  TTCAATTTTAGGTGCAATCAATTTCATTACAACTATTGTAAATATACGATCCATTGGAAT  480
            ||||||||||||||||||||||||||||||||||||||||||||||||||||||||||||
Sbjct  421  TTCAATTTTAGGTGCAATCAATTTCATTACAACTATTGTAAATATACGATCCATTGGAAT  480

Query  481  AACAATAGAACGAATACCATTATTTGTATGATCTGTTTTAATTACTGCAATTTTATTATT  540
            ||||||||||||||||||||||||||||||||||||||||||||||||||||||||||||
Sbjct  481  AACAATAGAACGAATACCATTATTTGTATGATCTGTTTTAATTACTGCAATTTTATTATT  540

Query  541  ATTATCTTTACCAGTCTTAGCTGGCGCAATTACAATATTATTAACTGATCGAAACTTTAA  600
            ||||||||||||||||||||||||||||||||||||||||||||||||||||||||||||
Sbjct  541  ATTATCTTTACCAGTCTTAGCTGGCGCAATTACAATATTATTAACTGATCGAAACTTTAA  600

Query  601  TACA  604
            ||||
Sbjct  601  TACA  604
```

>Rhipicephalus linnaei isolate A6-T1 cytochrome c oxidase subunit I (COX1) gene, partial cds; mitochondrial
Sequence ID: MZ726432.1 Length: 604
Range 1: 1 to 604

Score:1116 bits(604), Expect:0.0,
Identities:604/604(100%), Gaps:0/604(0%), Strand: Plus/Plus

```
Query  1    GACAATATACTTAATTTTTGGAGCATGATCCGGAATACTAGGATTAAGAATAAGAATATT  60
            ||||||||||||||||||||||||||||||||||||||||||||||||||||||||||||
Sbjct  1    GACAATATACTTAATTTTTGGAGCATGATCCGGAATACTAGGATTAAGAATAAGAATATT  60

Query  61   AATCCGCATAGAATTAGGTCAACCAGGAACTTTAATTGGAAACGATCAAATTTATAATGT  120
            ||||||||||||||||||||||||||||||||||||||||||||||||||||||||||||
Sbjct  61   AATCCGCATAGAATTAGGTCAACCAGGAACTTTAATTGGAAACGATCAAATTTATAATGT  120

Query  121  AATTGTAACAGCACATGCATTTATTATAAttttttttATAGTTATACCAATTATAATCGG  180
            ||||||||||||||||||||||||||||||||||||||||||||||||||||||||||||
Sbjct  121  AATTGTAACAGCACATGCATTTATTATAATTTTTTTTATAGTTATACCAATTATAATCGG  180

Query  181  AGGATTTGGAAATTGATTAGTTCCTATTATACTAGGAGCTCCAGATATAGCATTTCCACG  240
            ||||||||||||||||||||||||||||||||||||||||||||||||||||||||||||
Sbjct  181  AGGATTTGGAAATTGATTAGTTCCTATTATACTAGGAGCTCCAGATATAGCATTTCCACG  240

Query  241  AATAAATAATATAAGATTTTGGTTACTTCCTCCTTCTCTATTTCTACTAATTAATTCTTC  300
            ||||||||||||||||||||||||||||||||||||||||||||||||||||||||||||
Sbjct  241  AATAAATAATATAAGATTTTGGTTACTTCCTCCTTCTCTATTTCTACTAATTAATTCTTC  300

Query  301  ATTAATCGAATCAGGAGCAGGTACAGGGTGAACAGTTTAcccccccTTATCATCAAATTT  360
            ||||||||||||||||||||||||||||||||||||||||||||||||||||||||||||
Sbjct  301  ATTAATCGAATCAGGAGCAGGTACAGGGTGAACAGTTTACCCCCCCTTATCATCAAATTT  360

Query  361  ATCACACTACGGACCATCAGTAGATTTAGCTATTTTTTCTCTGCATCTTGCAGGTGCTTC  420
            ||||||||||||||||||||||||||||||||||||||||||||||||||||||||||||
Sbjct  361  ATCACACTACGGACCATCAGTAGATTTAGCTATTTTTTCTCTGCATCTTGCAGGTGCTTC  420

Query  421  TTCAATTTTAGGTGCAATCAATTTCATTACAACTATTGTAAATATACGATCCATTGGAAT  480
            ||||||||||||||||||||||||||||||||||||||||||||||||||||||||||||
Sbjct  421  TTCAATTTTAGGTGCAATCAATTTCATTACAACTATTGTAAATATACGATCCATTGGAAT  480

Query  481  AACAATAGAACGAATACCATTATTTGTATGATCTGTTTTAATTACTGCAATTTTATTATT  540
            ||||||||||||||||||||||||||||||||||||||||||||||||||||||||||||
Sbjct  481  AACAATAGAACGAATACCATTATTTGTATGATCTGTTTTAATTACTGCAATTTTATTATT  540

Query  541  ATTATCTTTACCAGTCTTAGCTGGCGCAATTACAATATTATTAACTGATCGAAACTTTAA  600
            ||||||||||||||||||||||||||||||||||||||||||||||||||||||||||||
Sbjct  541  ATTATCTTTACCAGTCTTAGCTGGCGCAATTACAATATTATTAACTGATCGAAACTTTAA  600

Query  601  TACA  604
            ||||
Sbjct  601  TACA  604
```

>Rhipicephalus linnaei isolate A5-T1 cytochrome c oxidase subunit I (COX1) gene, partial cds; mitochondrial
Sequence ID: MZ726431.1 Length: 604
Range 1: 1 to 604

Score:1116 bits(604), Expect:0.0,
Identities:604/604(100%), Gaps:0/604(0%), Strand: Plus/Plus

```
Query  1    GACAATATACTTAATTTTTGGAGCATGATCCGGAATACTAGGATTAAGAATAAGAATATT  60
            ||||||||||||||||||||||||||||||||||||||||||||||||||||||||||||
Sbjct  1    GACAATATACTTAATTTTTGGAGCATGATCCGGAATACTAGGATTAAGAATAAGAATATT  60

Query  61   AATCCGCATAGAATTAGGTCAACCAGGAACTTTAATTGGAAACGATCAAATTTATAATGT  120
            ||||||||||||||||||||||||||||||||||||||||||||||||||||||||||||
Sbjct  61   AATCCGCATAGAATTAGGTCAACCAGGAACTTTAATTGGAAACGATCAAATTTATAATGT  120

Query  121  AATTGTAACAGCACATGCATTTATTATAAttttttttATAGTTATACCAATTATAATCGG  180
            ||||||||||||||||||||||||||||||||||||||||||||||||||||||||||||
Sbjct  121  AATTGTAACAGCACATGCATTTATTATAATTTTTTTTATAGTTATACCAATTATAATCGG  180

Query  181  AGGATTTGGAAATTGATTAGTTCCTATTATACTAGGAGCTCCAGATATAGCATTTCCACG  240
            ||||||||||||||||||||||||||||||||||||||||||||||||||||||||||||
Sbjct  181  AGGATTTGGAAATTGATTAGTTCCTATTATACTAGGAGCTCCAGATATAGCATTTCCACG  240

Query  241  AATAAATAATATAAGATTTTGGTTACTTCCTCCTTCTCTATTTCTACTAATTAATTCTTC  300
            ||||||||||||||||||||||||||||||||||||||||||||||||||||||||||||
Sbjct  241  AATAAATAATATAAGATTTTGGTTACTTCCTCCTTCTCTATTTCTACTAATTAATTCTTC  300

Query  301  ATTAATCGAATCAGGAGCAGGTACAGGGTGAACAGTTTAcccccccTTATCATCAAATTT  360
            ||||||||||||||||||||||||||||||||||||||||||||||||||||||||||||
Sbjct  301  ATTAATCGAATCAGGAGCAGGTACAGGGTGAACAGTTTACCCCCCCTTATCATCAAATTT  360

Query  361  ATCACACTACGGACCATCAGTAGATTTAGCTATTTTTTCTCTGCATCTTGCAGGTGCTTC  420
            ||||||||||||||||||||||||||||||||||||||||||||||||||||||||||||
Sbjct  361  ATCACACTACGGACCATCAGTAGATTTAGCTATTTTTTCTCTGCATCTTGCAGGTGCTTC  420

Query  421  TTCAATTTTAGGTGCAATCAATTTCATTACAACTATTGTAAATATACGATCCATTGGAAT  480
            ||||||||||||||||||||||||||||||||||||||||||||||||||||||||||||
Sbjct  421  TTCAATTTTAGGTGCAATCAATTTCATTACAACTATTGTAAATATACGATCCATTGGAAT  480

Query  481  AACAATAGAACGAATACCATTATTTGTATGATCTGTTTTAATTACTGCAATTTTATTATT  540
            ||||||||||||||||||||||||||||||||||||||||||||||||||||||||||||
Sbjct  481  AACAATAGAACGAATACCATTATTTGTATGATCTGTTTTAATTACTGCAATTTTATTATT  540

Query  541  ATTATCTTTACCAGTCTTAGCTGGCGCAATTACAATATTATTAACTGATCGAAACTTTAA  600
            ||||||||||||||||||||||||||||||||||||||||||||||||||||||||||||
Sbjct  541  ATTATCTTTACCAGTCTTAGCTGGCGCAATTACAATATTATTAACTGATCGAAACTTTAA  600

Query  601  TACA  604
            ||||
```

```
Sbjct  601  TACA  604
```

>Rhipicephalus linnaei isolate A2-T1 cytochrome c oxidase subunit I (COX1) gene, partial cds; mitochondrial
Sequence ID: MZ726430.1 Length: 604
Range 1: 1 to 604

Score:1116 bits(604), Expect:0.0,
Identities:604/604(100%), Gaps:0/604(0%), Strand: Plus/Plus

```
Query  1    GACAATATACTTAATTTTTGGAGCATGATCCGGAATACTAGGATTAAGAATAAGAATATT  60
            ||||||||||||||||||||||||||||||||||||||||||||||||||||||||||||
Sbjct  1    GACAATATACTTAATTTTTGGAGCATGATCCGGAATACTAGGATTAAGAATAAGAATATT  60

Query  61   AATCCGCATAGAATTAGGTCAACCAGGAACTTTAATTGGAAACGATCAAATTTATAATGT  120
            ||||||||||||||||||||||||||||||||||||||||||||||||||||||||||||
Sbjct  61   AATCCGCATAGAATTAGGTCAACCAGGAACTTTAATTGGAAACGATCAAATTTATAATGT  120

Query  121  AATTGTAACAGCACATGCATTTATTATAAtttttttATAGTTATACCAATTATAATCGG  180
            ||||||||||||||||||||||||||||||||||||||||||||||||||||||||||||
Sbjct  121  AATTGTAACAGCACATGCATTTATTATAATTTTTTTATAGTTATACCAATTATAATCGG  180

Query  181  AGGATTTGGAAATTGATTAGTTCCTATTATACTAGGAGCTCCAGATATAGCATTTCCACG  240
            ||||||||||||||||||||||||||||||||||||||||||||||||||||||||||||
Sbjct  181  AGGATTTGGAAATTGATTAGTTCCTATTATACTAGGAGCTCCAGATATAGCATTTCCACG  240

Query  241  AATAAATAATATAAGATTTTGGTTACTTCCTCCTTCTCTATTTCTACTAATTAATTCTTC  300
            ||||||||||||||||||||||||||||||||||||||||||||||||||||||||||||
Sbjct  241  AATAAATAATATAAGATTTTGGTTACTTCCTCCTTCTCTATTTCTACTAATTAATTCTTC  300

Query  301  ATTAATCGAATCAGGAGCAGGTACAGGGTGAACAGTTTAcccccccTTATCATCAAATTT  360
            ||||||||||||||||||||||||||||||||||||||||||||||||||||||||||||
Sbjct  301  ATTAATCGAATCAGGAGCAGGTACAGGGTGAACAGTTTACCCCCCCTTATCATCAAATTT  360

Query  361  ATCACACTACGGACCATCAGTAGATTTAGCTATTTTTTCTCTGCATCTTGCAGGTGCTTC  420
            ||||||||||||||||||||||||||||||||||||||||||||||||||||||||||||
Sbjct  361  ATCACACTACGGACCATCAGTAGATTTAGCTATTTTTTCTCTGCATCTTGCAGGTGCTTC  420

Query  421  TTCAATTTTAGGTGCAATCAATTTCATTACAACTATTGTAAATATACGATCCATTGGAAT  480
            ||||||||||||||||||||||||||||||||||||||||||||||||||||||||||||
Sbjct  421  TTCAATTTTAGGTGCAATCAATTTCATTACAACTATTGTAAATATACGATCCATTGGAAT  480

Query  481  AACAATAGAACGAATACCATTATTTGTATGATCTGTTTTAATTACTGCAATTTTATTATT  540
            ||||||||||||||||||||||||||||||||||||||||||||||||||||||||||||
Sbjct  481  AACAATAGAACGAATACCATTATTTGTATGATCTGTTTTAATTACTGCAATTTTATTATT  540

Query  541  ATTATCTTTACCAGTCTTAGCTGGCGCAATTACAATATTATTAACTGATCGAAACTTTAA  600
            ||||||||||||||||||||||||||||||||||||||||||||||||||||||||||||
Sbjct  541  ATTATCTTTACCAGTCTTAGCTGGCGCAATTACAATATTATTAACTGATCGAAACTTTAA  600

Query  601  TACA  604
```

```
             ||||
Sbjct  601  TACA  604
```

>Rhipicephalus linnaei isolate B9-T1 cytochrome c oxidase subunit I (COX1) gene, partial cds; mitochondrial
Sequence ID: MZ726449.1 Length: 604
Range 1: 1 to 604

Score:1110 bits(601), Expect:0.0,
Identities:603/604(99%), Gaps:0/604(0%), Strand: Plus/Plus

```
Query  1    GACAATATACTTAATTTTTGGAGCATGATCCGGAATACTAGGATTAAGAATAAGAATATT  60
            ||||||||||||||||||||||||||||||||||||||||||||||||||||||||||||
Sbjct  1    GACAATATACTTAATTTTTGGAGCATGATCCGGAATACTAGGATTAAGAATAAGAATATT  60

Query  61   AATCCGCATAGAATTAGGTCAACCAGGAACTTTAATTGGAAACGATCAAATTTATAATGT  120
            ||||||||||||||||||||||||||||||||||||||||||||||||||||||||||||
Sbjct  61   AATCCGCATAGAATTAGGTCAACCAGGAACTTTAATTGGAAACGATCAAATTTATAATGT  120

Query  121  AATTGTAACAGCACATGCATTTATTATAAttttttttATAGTTATACCAATTATAATCGG  180
            ||||||||||||||||||||||||||||||||||||||||||||||||||||||||||||
Sbjct  121  AATTGTAACAGCACATGCATTTATTATAATTTTTTTTATAGTTATACCAATTATAATCGG  180

Query  181  AGGATTTGGAAATTGATTAGTTCCTATTATACTAGGAGCTCCAGATATAGCATTTCCACG  240
            ||||||||||||||||||||||||||||||||||||||||||||||||||||||||||||
Sbjct  181  AGGATTTGGAAATTGATTAGTTCCTATTATACTAGGAGCTCCAGATATAGCATTTCCACG  240

Query  241  AATAAATAATATAAGATTTTGGTTACTTCCTCCTTCTCTATTTCTACTAATTAATTCTTC  300
            ||||||||||||||||||||||||||||||||||||||||||||||||||||||||||||
Sbjct  241  AATAAATAATATAAGATTTTGGTTACTTCCTCCTTCTCTATTTCTACTAATTAATTCTTC  300

Query  301  ATTAATCGAATCAGGAGCAGGTACAGGGTGAACAGTTTAcccccccTTATCATCAAATTT  360
            |||||||||||||||||||||||| |||||||||||||||||||||||||||||||||||
Sbjct  301  ATTAATCGAATCAGGAGCAGGTACGGGGTGAACAGTTTACCCCCCCTTATCATCAAATTT  360

Query  361  ATCACACTACGGACCATCAGTAGATTTAGCTATTTTTTCTCTGCATCTTGCAGGTGCTTC  420
            ||||||||||||||||||||||||||||||||||||||||||||||||||||||||||||
Sbjct  361  ATCACACTACGGACCATCAGTAGATTTAGCTATTTTTTCTCTGCATCTTGCAGGTGCTTC  420

Query  421  TTCAATTTTAGGTGCAATCAATTTCATTACAACTATTGTAAATATACGATCCATTGGAAT  480
            ||||||||||||||||||||||||||||||||||||||||||||||||||||||||||||
Sbjct  421  TTCAATTTTAGGTGCAATCAATTTCATTACAACTATTGTAAATATACGATCCATTGGAAT  480

Query  481  AACAATAGAACGAATACCATTATTTGTATGATCTGTTTTAATTACTGCAATTTTATTATT  540
            ||||||||||||||||||||||||||||||||||||||||||||||||||||||||||||
Sbjct  481  AACAATAGAACGAATACCATTATTTGTATGATCTGTTTTAATTACTGCAATTTTATTATT  540

Query  541  ATTATCTTTACCAGTCTTAGCTGGCGCAATTACAATATTATTAACTGATCGAAACTTTAA  600
            ||||||||||||||||||||||||||||||||||||||||||||||||||||||||||||
Sbjct  541  ATTATCTTTACCAGTCTTAGCTGGCGCAATTACAATATTATTAACTGATCGAAACTTTAA  600
```

```
Query  601  TACA  604
            ||||
Sbjct  601  TACA  604
```

>Rhipicephalus linnaei isolate B18-T1 cytochrome c oxidase subunit I (COX1) gene, partial cds; mitochondrial
Sequence ID: MZ726429.1 Length: 604
Range 1: 1 to 604

Score:1110 bits(601), Expect:0.0,
Identities:603/604(99%), Gaps:0/604(0%), Strand: Plus/Plus

```
Query  1    GACAATATACTTAATTTTTGGAGCATGATCCGGAATACTAGGATTAAGAATAAGAATATT  60
            ||||||||||||||||||||||||||||||||||||||||||||||||||||||||||||
Sbjct  1    GACAATATACTTAATTTTTGGAGCATGATCCGGAATACTAGGATTAAGAATAAGAATATT  60

Query  61   AATCCGCATAGAATTAGGTCAACCAGGAACTTTAATTGGAAACGATCAAATTTATAATGT  120
            ||||||||||||||||||||||||||||||||||||||||||||||||||||||||||||
Sbjct  61   AATCCGCATAGAATTAGGTCAACCAGGAACTTTAATTGGAAACGATCAAATTTATAATGT  120

Query  121  AATTGTAACAGCACATGCATTTATTATAAttttttttATAGTTATACCAATTATAATCGG  180
            ||||||||||||||||||||||||||||||||||||||||||||||||||||||||||||
Sbjct  121  AATTGTAACAGCACATGCATTTATTATAATTTTTTTTATAGTTATACCAATTATAATCGG  180

Query  181  AGGATTTGGAAATTGATTAGTTCCTATTATACTAGGAGCTCCAGATATAGCATTTCCACG  240
            ||||||||||||||||||||||||||||||||||||||||||||||||||||||||||||
Sbjct  181  AGGATTTGGAAATTGATTAGTTCCTATTATACTAGGAGCTCCAGATATAGCATTTCCACG  240

Query  241  AATAAATAATATAAGATTTTGGTTACTTCCTCCTTCTCTATTTCTACTAATTAATTCTTC  300
            ||||||||||||||||||||||||||||||||||||||||||||||||||||||||||||
Sbjct  241  AATAAATAATATAAGATTTTGGTTACTTCCTCCTTCTCTATTTCTACTAATTAATTCTTC  300

Query  301  ATTAATCGAATCAGGAGCAGGTACAGGGTGAACAGTTTAcccccccTTATCATCAAATTT  360
            ||||||||||||||||||||||||||||||||||||||||||||||||||||||||||||
Sbjct  301  ATTAATCGAATCAGGAGCAGGTACAGGGTGAACAGTTTACCCCCCCTTATCATCAAATTT  360

Query  361  ATCACACTACGGACCATCAGTAGATTTAGCTATTTTTTCTCTGCATCTTGCAGGTGCTTC  420
            |||||||||||||||||||||||||||||||||||||||||| |||||||||||||||||
Sbjct  361  ATCACACTACGGACCATCAGTAGATTTAGCTATTTTTTCTCTACATCTTGCAGGTGCTTC  420

Query  421  TTCAATTTTAGGTGCAATCAATTTCATTACAACTATTGTAAATATACGATCCATTGGAAT  480
            ||||||||||||||||||||||||||||||||||||||||||||||||||||||||||||
Sbjct  421  TTCAATTTTAGGTGCAATCAATTTCATTACAACTATTGTAAATATACGATCCATTGGAAT  480

Query  481  AACAATAGAACGAATACCATTATTTGTATGATCTGTTTTAATTACTGCAATTTTATTATT  540
            ||||||||||||||||||||||||||||||||||||||||||||||||||||||||||||
Sbjct  481  AACAATAGAACGAATACCATTATTTGTATGATCTGTTTTAATTACTGCAATTTTATTATT  540

Query  541  ATTATCTTTACCAGTCTTAGCTGGCGCAATTACAATATTATTAACTGATCGAAACTTTAA  600
            ||||||||||||||||||||||||||||||||||||||||||||||||||||||||||||
Sbjct  541  ATTATCTTTACCAGTCTTAGCTGGCGCAATTACAATATTATTAACTGATCGAAACTTTAA  600
```

```
Query  601  TACA  604
            ||||
Sbjct  601  TACA  604
```

>Rhipicephalus linnaei isolate B17-T1 cytochrome c oxidase subunit I (COX1) gene, partial cds; mitochondrial
Sequence ID: MZ726428.1 Length: 604
Range 1: 1 to 604

Score:1110 bits(601), Expect:0.0,
Identities:603/604(99%), Gaps:0/604(0%), Strand: Plus/Plus

```
Query  1    GACAATATACTTAATTTTTGGAGCATGATCCGGAATACTAGGATTAAGAATAAGAATATT  60
            ||||||||||||||||||||||||||||||||||||||||||||||||||||||||||||
Sbjct  1    GACAATATACTTAATTTTTGGAGCATGATCCGGAATACTAGGATTAAGAATAAGAATATT  60

Query  61   AATCCGCATAGAATTAGGTCAACCAGGAACTTTAATTGGAAACGATCAAATTTATAATGT  120
            ||||||||||||||||||||||||||||||||||||||||||||||||||||||||||||
Sbjct  61   AATCCGCATAGAATTAGGTCAACCAGGAACTTTAATTGGAAACGATCAAATTTATAATGT  120

Query  121  AATTGTAACAGCACATGCATTTATTATAAttttttttATAGTTATACCAATTATAATCGG  180
            ||||||||||||||||||||||||||||||||||||||||||||||||||||||||||||
Sbjct  121  AATTGTAACAGCACATGCATTTATTATAATTTTTTTTATAGTTATACCAATTATAATCGG  180

Query  181  AGGATTTGGAAATTGATTAGTTCCTATTATACTAGGAGCTCCAGATATAGCATTTCCACG  240
            ||||||||||||||||||||||||||||||||||||||||||||||||||||||||||||
Sbjct  181  AGGATTTGGAAATTGATTAGTTCCTATTATACTAGGAGCTCCAGATATAGCATTTCCACG  240

Query  241  AATAAATAATATAAGATTTTGGTTACTTCCTCCTTCTCTATTTCTACTAATTAATTCTTC  300
            ||||||||||||||||||||||||||||||||||||||||||||||||||||||||||||
Sbjct  241  AATAAATAATATAAGATTTTGGTTACTTCCTCCTTCTCTATTTCTACTAATTAATTCTTC  300

Query  301  ATTAATCGAATCAGGAGCAGGTACAGGGTGAACAGTTTAcccccccTTATCATCAAATTT  360
            ||||||||||||||||||||||||||||||||||||||||||||||||||||||||||||
Sbjct  301  ATTAATCGAATCAGGAGCAGGTACAGGGTGAACAGTTTACCCCCCCTTATCATCAAATTT  360

Query  361  ATCACACTACGGACCATCAGTAGATTTAGCTATTTTTTCTCTGCATCTTGCAGGTGCTTC  420
            |||||||||||||||||||||||||||||||||||||||||| |||||||||||||||||
Sbjct  361  ATCACACTACGGACCATCAGTAGATTTAGCTATTTTTTCTCTACATCTTGCAGGTGCTTC  420

Query  421  TTCAATTTTAGGTGCAATCAATTTCATTACAACTATTGTAAATATACGATCCATTGGAAT  480
            ||||||||||||||||||||||||||||||||||||||||||||||||||||||||||||
Sbjct  421  TTCAATTTTAGGTGCAATCAATTTCATTACAACTATTGTAAATATACGATCCATTGGAAT  480

Query  481  AACAATAGAACGAATACCATTATTTGTATGATCTGTTTTAATTACTGCAATTTTATTATT  540
            ||||||||||||||||||||||||||||||||||||||||||||||||||||||||||||
Sbjct  481  AACAATAGAACGAATACCATTATTTGTATGATCTGTTTTAATTACTGCAATTTTATTATT  540

Query  541  ATTATCTTTACCAGTCTTAGCTGGCGCAATTACAATATTATTAACTGATCGAAACTTTAA  600
            ||||||||||||||||||||||||||||||||||||||||||||||||||||||||||||
```

```
Sbjct  541  ATTATCTTTACCAGTCTTAGCTGGCGCAATTACAATATTATTAACTGATCGAAACTTTAA  600

Query  601  TACA  604
            ||||
Sbjct  601  TACA  604
```

>Rhipicephalus linnaei isolate B16-T1 cytochrome c oxidase subunit I (COX1) gene, partial cds; mitochondrial
Sequence ID: MZ726427.1 Length: 604
Range 1: 1 to 604

Score:1110 bits(601), Expect:0.0,
Identities:603/604(99%), Gaps:0/604(0%), Strand: Plus/Plus

```
Query  1    GACAATATACTTAATTTTTGGAGCATGATCCGGAATACTAGGATTAAGAATAAGAATATT  60
            ||||||||||||||||||||||||||||||||||||||||||||||||||||||||||||
Sbjct  1    GACAATATACTTAATTTTTGGAGCATGATCCGGAATACTAGGATTAAGAATAAGAATATT  60

Query  61   AATCCGCATAGAATTAGGTCAACCAGGAACTTTAATTGGAAACGATCAAATTTATAATGT  120
            ||||||||||||||||||||||||||||||||||||||||||||||||||||||||||||
Sbjct  61   AATCCGCATAGAATTAGGTCAACCAGGAACTTTAATTGGAAACGATCAAATTTATAATGT  120

Query  121  AATTGTAACAGCACATGCATTTATTATAAtttttttATAGTTATACCAATTATAATCGG  180
            ||||||||||||||||||||||||||||||||||||||||||||||||||||||||||||
Sbjct  121  AATTGTAACAGCACATGCATTTATTATAATTTTTTTATAGTTATACCAATTATAATCGG  180

Query  181  AGGATTTGGAAATTGATTAGTTCCTATTATACTAGGAGCTCCAGATATAGCATTTCCACG  240
            ||||||||||||||||||||||||||||||||||||||||||||||||||||||||||||
Sbjct  181  AGGATTTGGAAATTGATTAGTTCCTATTATACTAGGAGCTCCAGATATAGCATTTCCACG  240

Query  241  AATAAATAATATAAGATTTTGGTTACTTCCTCCTTCTCTATTTCTACTAATTAATTCTTC  300
            ||||||||||||||||||||||||||||||||||||||||||||||||||||||||||||
Sbjct  241  AATAAATAATATAAGATTTTGGTTACTTCCTCCTTCTCTATTTCTACTAATTAATTCTTC  300

Query  301  ATTAATCGAATCAGGAGCAGGTACAGGGTGAACAGTTTAcccccccTTATCATCAAATTT  360
            ||||||||||||||||||||||||||||||||||||||||||||||||||||||||||||
Sbjct  301  ATTAATCGAATCAGGAGCAGGTACAGGGTGAACAGTTTACCCCCCCTTATCATCAAATTT  360

Query  361  ATCACACTACGGACCATCAGTAGATTTAGCTATTTTTTCTCTGCATCTTGCAGGTGCTTC  420
            |||||||||||||||||||||||||||||||||||||||||| |||||||||||||||||
Sbjct  361  ATCACACTACGGACCATCAGTAGATTTAGCTATTTTTTCTCTACATCTTGCAGGTGCTTC  420

Query  421  TTCAATTTTAGGTGCAATCAATTTCATTACAACTATTGTAAATATACGATCCATTGGAAT  480
            ||||||||||||||||||||||||||||||||||||||||||||||||||||||||||||
Sbjct  421  TTCAATTTTAGGTGCAATCAATTTCATTACAACTATTGTAAATATACGATCCATTGGAAT  480

Query  481  AACAATAGAACGAATACCATTATTTGTATGATCTGTTTTAATTACTGCAATTTTATTATT  540
            ||||||||||||||||||||||||||||||||||||||||||||||||||||||||||||
Sbjct  481  AACAATAGAACGAATACCATTATTTGTATGATCTGTTTTAATTACTGCAATTTTATTATT  540

Query  541  ATTATCTTTACCAGTCTTAGCTGGCGCAATTACAATATTATTAACTGATCGAAACTTTAA  600
```

```
          ||||||||||||||||||||||||||||||||||||||||||||||||||||||||||||
Sbjct  541  ATTATCTTTACCAGTCTTAGCTGGCGCAATTACAATATTATTAACTGATCGAAACTTTAA  600

Query  601  TACA  604
            ||||
Sbjct  601  TACA  604
```

>Rhipicephalus linnaei isolate B1-T1 cytochrome c oxidase subunit I (COX1) gene, partial cds; mitochondrial
Sequence ID: MZ726426.1 Length: 604
Range 1: 1 to 604

Score:1110 bits(601), Expect:0.0,
Identities:603/604(99%), Gaps:0/604(0%), Strand: Plus/Plus

```
Query  1    GACAATATACTTAATTTTTGGAGCATGATCCGGAATACTAGGATTAAGAATAAGAATATT  60
            ||||||||||||||||||||||||||||||||||||||||||||||||||||||||||||
Sbjct  1    GACAATATACTTAATTTTTGGAGCATGATCCGGAATACTAGGATTAAGAATAAGAATATT  60

Query  61   AATCCGCATAGAATTAGGTCAACCAGGAACTTTAATTGGAAACGATCAAATTTATAATGT  120
            ||||||||||||||||||||||||||||||||||||||||||||||||||||||||||||
Sbjct  61   AATCCGCATAGAATTAGGTCAACCAGGAACTTTAATTGGAAACGATCAAATTTATAATGT  120

Query  121  AATTGTAACAGCACATGCATTTATTATAAttttttttATAGTTATACCAATTATAATCGG  180
            ||||||||||||||||||||||||||||||||||||||||||||||||||||||||||||
Sbjct  121  AATTGTAACAGCACATGCATTTATTATAATTTTTTTTATAGTTATACCAATTATAATCGG  180

Query  181  AGGATTTGGAAATTGATTAGTTCCTATTATACTAGGAGCTCCAGATATAGCATTTCCACG  240
            ||||||||||||||||||||||||||||||||||||||||||||||||||||||||||||
Sbjct  181  AGGATTTGGAAATTGATTAGTTCCTATTATACTAGGAGCTCCAGATATAGCATTTCCACG  240

Query  241  AATAAATAATATAAGATTTTGGTTACTTCCTCCTTCTCTATTTCTACTAATTAATTCTTC  300
            ||||||||||||||||||||||||||||||||||||||||||||||||||||||||||||
Sbjct  241  AATAAATAATATAAGATTTTGGTTACTTCCTCCTTCTCTATTTCTACTAATTAATTCTTC  300

Query  301  ATTAATCGAATCAGGAGCAGGTACAGGGTGAACAGTTTAcccccccTTATCATCAAATTT  360
            ||||||||||||||||||||||||||||||||||||||||||||||||||||||||||||
Sbjct  301  ATTAATCGAATCAGGAGCAGGTACAGGGTGAACAGTTTACCCCCCCTTATCATCAAATTT  360

Query  361  ATCACACTACGGACCATCAGTAGATTTAGCTATTTTTTCTCTGCATCTTGCAGGTGCTTC  420
            |||||||||||||||||||||||||||||||||||||||||| |||||||||||||||||
Sbjct  361  ATCACACTACGGACCATCAGTAGATTTAGCTATTTTTTCTCTACATCTTGCAGGTGCTTC  420

Query  421  TTCAATTTTAGGTGCAATCAATTTCATTACAACTATTGTAAATATACGATCCATTGGAAT  480
            ||||||||||||||||||||||||||||||||||||||||||||||||||||||||||||
Sbjct  421  TTCAATTTTAGGTGCAATCAATTTCATTACAACTATTGTAAATATACGATCCATTGGAAT  480

Query  481  AACAATAGAACGAATACCATTATTTGTATGATCTGTTTTAATTACTGCAATTTTATTATT  540
            ||||||||||||||||||||||||||||||||||||||||||||||||||||||||||||
Sbjct  481  AACAATAGAACGAATACCATTATTTGTATGATCTGTTTTAATTACTGCAATTTTATTATT  540
```

```
Query  541  ATTATCTTTACCAGTCTTAGCTGGCGCAATTACAATATTATTAACTGATCGAAACTTTAA  600
            ||||||||||||||||||||||||||||||||||||||||||||||||||||||||||||
Sbjct  541  ATTATCTTTACCAGTCTTAGCTGGCGCAATTACAATATTATTAACTGATCGAAACTTTAA  600

Query  601  TACA  604
            ||||
Sbjct  601  TACA  604
```

>Rhipicephalus sanguineus voucher DO-306 cytochrome oxidase subunit 1 (COI) gene, partial cds; mitochondrial
Sequence ID: KX360338.1 Length: 638
Range 1: 1 to 601

Score:1092 bits(591), Expect:0.0,
Identities:597/601(99%), Gaps:0/601(0%), Strand: Plus/Plus

```
Query  20   GGAGCATGATCCGGAATACTAGGATTAAGAATAAGAATATTAATCCGCATAGAATTAGGT  79
            ||||||||||||||||| ||||||||||||||||||||||||||||||||||||||||||
Sbjct  1    GGAGCATGATCCGGAATATTAGGATTAAGAATAAGAATATTAATCCGCATAGAATTAGGT  60

Query  80   CAACCAGGAACTTTAATTGGAAACGATCAAATTTATAATGTAATTGTAACAGCACATGCA  139
            ||||||||||||||||||||||||||||||||||||||||||||||||||||||||||||
Sbjct  61   CAACCAGGAACTTTAATTGGAAACGATCAAATTTATAATGTAATTGTAACAGCACATGCA  120

Query  140  TTTATTATAAttttttttATAGTTATACCAATTATAATCGGAGGATTTGGAAATTGATTA  199
            ||||||||||||||||||||||||||||||||||||||||||||||||||||||||||||
Sbjct  121  TTTATTATAATTTTTTTTATAGTTATACCAATTATAATCGGAGGATTTGGAAATTGATTA  180

Query  200  GTTCCTATTATACTAGGAGCTCCAGATATAGCATTTCCACGAATAAATAATATAAGATTT  259
            ||||||||||||||||| ||||||||||||||||||||||||||||||||||||||||||
Sbjct  181  GTTCCTATTATACTAGGGGCTCCAGATATAGCATTTCCACGAATAAATAATATAAGATTT  240

Query  260  TGGTTACTTCCTCCTTCTCTATTTCTACTAATTAATTCTTCATTAATCGAATCAGGAGCA  319
            ||||||||||||||||||||||||||||||||||||||||||||||||||||||||||||
Sbjct  241  TGGTTACTTCCTCCTTCTCTATTTCTACTAATTAATTCTTCATTAATCGAATCAGGAGCA  300

Query  320  GGTACAGGGTGAACAGTTTAcccccccTTATCATCAAATTTATCACACTACGGACCATCA  379
            || |||||||||||||||||||||||||||||||||||||||||||||||||||||||||
Sbjct  301  GGNACAGGGTGAACAGTTTACCCCCCCTTATCATCAAATTTATCACACTACGGACCATCA  360

Query  380  GTAGATTTAGCTATTTTTTCTCTGCATCTTGCAGGTGCTTCTTCAATTTTAGGTGCAATC  439
            ||||||||||||||||||||||||||||||||||||||||||||||||||||||||||||
Sbjct  361  GTAGATTTAGCTATTTTTTCTCTGCATCTTGCAGGTGCTTCTTCAATTTTAGGTGCAATC  420

Query  440  AATTTCATTACAACTATTGTAAATATACGATCCATTGGAATAACAATAGAACGAATACCA  499
            ||||||||||||||||||||||||||||||||||||||||||||||||||||||||||||
Sbjct  421  AATTTCATTACAACTATTGTAAATATACGATCCATTGGAATAACAATAGAACGAATACCA  480

Query  500  TTATTTGTATGATCTGTTTTAATTACTGCAATTTTATTATTATTATCTTTACCAGTCTTA  559
            |||||||||||||||||||||||||| |||||||||||||||||||||||||||||||||
Sbjct  481  TTATTTGTATGATCTGTTTTAATTACNGCAATTTTATTATTATTATCTTTACCAGTCTTA  540
```

```
Query  560  GCTGGCGCAATTACAATATTATTAACTGATCGAAACTTTAATACATCATTCTTTGACCCT  619
            ||||||||||||||||||||||||||||||||||||||||||||||||||||||||||||
Sbjct  541  GCTGGCGCAATTACAATATTATTAACTGATCGAAACTTTAATACATCATTCTTTGACCCT  600

Query  620  T  620
            |
Sbjct  601  T  601
```

Query #2: TKCJJ091-19|TKCJ012018H07|Rhipicephalus sanguineus|COI-5P Query ID: lcl|Query_26764 Length: 658

```
Sequences producing significant alignments:
                                                                           Scientific
 Common                     Max    Total Query   E   Per.   Acc.

Description                                                                Name
 Name             Taxid      Score  Score cover Value Ident  Len        Accession

Rhipicephalus sanguineus sensu lato 'temperate lineage' isolat...
Rhipicephalu... brown dog tick  34632      1216   1216  100%  0.0   100.00 697
     MF426019.1
Rhipicephalus sanguineus haplotype III cytochrome oxidase...
Rhipicephalu... brown dog tick  34632      1216   1216  100%  0.0   100.00 713
     KU556745.1
Rhipicephalus sanguineus isolate Arizona-1 cytochrome c oxidas...
Rhipicephalu... brown dog tick  34632      1216   1216  100%  0.0   100.00 710
     MN593344.1
Rhipicephalus sanguineus isolate Arizona-2 cytochrome c oxidas...
Rhipicephalu... brown dog tick  34632      1216   1216  100%  0.0   100.00 710
     MN593343.1
Rhipicephalus sanguineus isolate 11N cytochrome oxidase subuni...
Rhipicephalu... brown dog tick  34632      1216   1216  100%  0.0   100.00 703
     MN585197.1
Rhipicephalus sanguineus cytochrome c oxidase I (COI) gene,...
Rhipicephalu... brown dog tick  34632      1216   1216  100%  0.0   100.00 793
     AF132839.1
Rhipicephalus sanguineus sensu lato 'temperate lineage' isolat...
Rhipicephalu... brown dog tick  34632      1210   1210  100%  0.0   99.85  675
     MF426018.1
Rhipicephalus sanguineus sensu lato 'temperate lineage' isolat...
Rhipicephalu... brown dog tick  34632      1210   1210  100%  0.0   99.85  680
     MF426016.1
Rhipicephalus sanguineus sensu lato 'temperate lineage' isolat...
Rhipicephalu... brown dog tick  34632      1210   1210  100%  0.0   99.85  693
     MF426013.1
Rhipicephalus sanguineus sensu lato 'temperate lineage' isolat...
Rhipicephalu... brown dog tick  34632      1210   1210  100%  0.0   99.85  679
     MF426009.1
Rhipicephalus sanguineus sensu lato 'temperate lineage' isolat...
```

Rhipicephalu... brown dog tick 34632 1210 1210 100% 0.0 99.85 690
MF426008.1
Rhipicephalus sanguineus sensu lato 'temperate lineage' isolat...
Rhipicephalu... brown dog tick 34632 1210 1210 100% 0.0 99.85 686
MF426007.1
Rhipicephalus sanguineus sensu lato 'temperate lineage' isolat...
Rhipicephalu... brown dog tick 34632 1210 1210 100% 0.0 99.85 684
MF426001.1
Rhipicephalus sanguineus sensu lato 'temperate lineage' isolat...
Rhipicephalu... brown dog tick 34632 1210 1210 100% 0.0 99.85 695
MF425997.1
Rhipicephalus sanguineus voucher CROBB813 cytochrome oxidase...
Rhipicephalu... brown dog tick 34632 1210 1210 100% 0.0 99.85 658
MZ305546.1
Rhipicephalus sanguineus voucher CROBB810 cytochrome oxidase...
Rhipicephalu... brown dog tick 34632 1210 1210 100% 0.0 99.85 658
MZ305545.1
Rhipicephalus sanguineus isolate 5MSK cytochrome c oxidase...
Rhipicephalu... brown dog tick 34632 1210 1210 100% 0.0 99.85 676
MW152145.1
Rhipicephalus sanguineus isolate 1MSK cytochrome c oxidase...
Rhipicephalu... brown dog tick 34632 1210 1210 100% 0.0 99.85 675
MW152144.1
Rhipicephalus sanguineus isolate RSN2SK cytochrome c oxidase...
Rhipicephalu... brown dog tick 34632 1210 1210 100% 0.0 99.85 676
MW152143.1
Rhipicephalus sanguineus isolate RSN11SK cytochrome c oxidase...
Rhipicephalu... brown dog tick 34632 1210 1210 100% 0.0 99.85 669
MW152142.1
Rhipicephalus sanguineus sensu lato 'temperate lineage' isolat...
Rhipicephalu... brown dog tick 34632 1205 1205 100% 0.0 99.70 692
MF426015.1
Rhipicephalus sanguineus sensu lato 'temperate lineage' isolat...
Rhipicephalu... brown dog tick 34632 1205 1205 100% 0.0 99.70 705
MF426006.1
Rhipicephalus sanguineus sensu lato 'temperate lineage' isolat...
Rhipicephalu... brown dog tick 34632 1199 1199 100% 0.0 99.54 680
MF426017.1
Rhipicephalus sanguineus sensu lato 'temperate lineage' isolat...
Rhipicephalu... brown dog tick 34632 1199 1199 100% 0.0 99.54 689
MF425998.1
Rhipicephalus sanguineus haplotype IV cytochrome oxidase subun...
Rhipicephalu... brown dog tick 34632 1199 1257 100% 0.0 99.54 771
KU556746.1
Rhipicephalus sanguineus sensu lato 'temperate lineage' isolat...
Rhipicephalu... brown dog tick 34632 1194 1194 100% 0.0 99.39 684
MF426012.1
Rhipicephalus sanguineus sensu lato 'temperate lineage' isolat...
Rhipicephalu... brown dog tick 34632 1192 1192 98% 0.0 99.85 824
MF426010.1
Rhipicephalus sanguineus haplotype II cytochrome oxidase subun...
Rhipicephalu... brown dog tick 34632 1190 1190 100% 0.0 99.24 787
KU556744.1
Rhipicephalus sanguineus haplotype I cytochrome oxidase subuni...

```
Rhipicephalu... brown dog tick  34632      1186   1186  98%   0.0   99.69  659
     KU556743.1
Rhipicephalus sanguineus sensu lato 'temperate lineage' isolat...
Rhipicephalu... brown dog tick  34632      1182   1182  97%   0.0   99.84  812
     MF426020.1
Rhipicephalus sanguineus isolate LIC5424B cytochrome oxidase...
Rhipicephalu... brown dog tick  34632      1175   1175  97%   0.0   99.69  768
     KX383818.1
Rhipicephalus sanguineus isolate LIC5533B cytochrome oxidase...
Rhipicephalu... brown dog tick  34632      1168   1168  96%   0.0   99.69  764
     KX383811.1
Rhipicephalus sanguineus isolate LIC4736B cytochrome oxidase...
Rhipicephalu... brown dog tick  34632      1166   1166  96%   0.0   99.69  763
     KX383807.1
Rhipicephalus sanguineus sensu lato 'temperate lineage' isolat...
Rhipicephalu... brown dog tick  34632      1164   1164  97%   0.0   99.38  809
     MF426011.1
Rhipicephalus sanguineus isolate LIC4736D cytochrome oxidase...
Rhipicephalu... brown dog tick  34632      1164   1164  96%   0.0   99.69  762
     KX383809.1
Rhipicephalus sanguineus isolate LIC4727D cytochrome oxidase...
Rhipicephalu... brown dog tick  34632      1164   1164  96%   0.0   99.69  714
     KX383806.1
Rhipicephalus sanguineus isolate LIC4727C cytochrome oxidase...
Rhipicephalu... brown dog tick  34632      1162   1162  96%   0.0   99.69  761
     KX383805.1
Rhipicephalus sanguineus sensu lato 'temperate lineage' isolat...
Rhipicephalu... brown dog tick  34632      1160   1160  97%   0.0   99.38  654
     MF425988.1
Rhipicephalus sanguineus clone #7 cytochrome oxidase subunit I...
Rhipicephalu... brown dog tick  34632      1157   1157  95%   0.0   100.00 654
     KX519712.1
Rhipicephalus sanguineus sensu lato 'temperate lineage' isolat...
Rhipicephalu... brown dog tick  34632      1155   1155  95%   0.0   99.84  639
     MF426002.1
Rhipicephalus sanguineus isolate Rsan-ML4 cytochrome oxidase...
Rhipicephalu... brown dog tick  34632      1146   1146  94%   0.0   100.00 630
     MG855658.1
Rhipicephalus sanguineus isolate Rsan-ML3 cytochrome oxidase...
Rhipicephalu... brown dog tick  34632      1146   1146  94%   0.0   100.00 630
     MG855657.1
Rhipicephalus sanguineus isolate Rsan-ML2 cytochrome oxidase...
Rhipicephalu... brown dog tick  34632      1146   1146  94%   0.0   100.00 630
     MG855656.1
Rhipicephalus sanguineus isolate Kehf-Lagareb-A cytochrome...
Rhipicephalu... brown dog tick  34632      1146   1146  94%   0.0   100.00 630
     KX757910.1
Rhipicephalus sanguineus isolate Italy-B cytochrome oxidase...
Rhipicephalu... brown dog tick  34632      1146   1146  94%   0.0   100.00 630
     KX757904.1
Rhipicephalus sanguineus isolate Malta-A cytochrome oxidase...
Rhipicephalu... brown dog tick  34632      1146   1146  94%   0.0   100.00 630
     KX757902.1
Rhipicephalus sanguineus isolate Zagreb-C cytochrome oxidase...
```

Rhipicephalu... brown dog tick 34632   1146   1146 94%   0.0   100.00 630
     KX757896.1
Rhipicephalus sanguineus isolate Pula-B cytochrome oxidase...
Rhipicephalu... brown dog tick 34632   1146   1146 94%   0.0   100.00 630
     KX757889.1
Rhipicephalus sanguineus isolate Rovind cytochrome oxidase...
Rhipicephalu... brown dog tick 34632   1146   1146 94%   0.0   100.00 630
     KX757887.1
Rhipicephalus sanguineus isolate Kajtasovo cytochrome oxidase...
Rhipicephalu... brown dog tick 34632   1146   1146 94%   0.0   100.00 630
     KX757879.1
Rhipicephalus sanguineus clone #6 cytochrome oxidase subunit I...
Rhipicephalu... brown dog tick 34632   1146   1146 94%   0.0   100.00 636
     KX519711.1
Rhipicephalus sanguineus isolate SV4 cytochrome c oxidase...
Rhipicephalu... brown dog tick 34632   1146   1146 94%   0.0   100.00 630
     MW135448.1
Rhipicephalus sanguineus isolate Samont2 cytochrome oxidase...
Rhipicephalu... brown dog tick 34632   1144   1144 94%   0.0   99.84 644
     MH630347.1
Rhipicephalus sanguineus isolate Samont1 cytochrome oxidase...
Rhipicephalu... brown dog tick 34632   1144   1144 94%   0.0   99.84 644
     MH630346.1
Rhipicephalus sanguineus sensu lato 'temperate lineage' isolat...
Rhipicephalu... brown dog tick 34632   1144   1144 94%   0.0   99.84 635
     MF426000.1
Rhipicephalus sanguineus isolate Boka-A cytochrome oxidase...
Rhipicephalu... brown dog tick 34632   1140   1140 94%   0.0   99.84 630
     KX757905.1
Rhipicephalus sanguineus isolate Sibenik cytochrome oxidase...
Rhipicephalu... brown dog tick 34632   1140   1140 94%   0.0   99.84 630
     KX757895.1
Rhipicephalus sanguineus isolate Zadar cytochrome oxidase...
Rhipicephalu... brown dog tick 34632   1140   1140 94%   0.0   99.84 630
     KX757892.1
Rhipicephalus sanguineus isolate Zagreb-A cytochrome oxidase...
Rhipicephalu... brown dog tick 34632   1140   1140 94%   0.0   99.84 630
     KX757890.1
Rhipicephalus sanguineus isolate Pula-A cytochrome oxidase...
Rhipicephalu... brown dog tick 34632   1140   1140 94%   0.0   99.84 630
     KX757888.1
Rhipicephalus sanguineus isolate Petnica cytochrome oxidase...
Rhipicephalu... brown dog tick 34632   1140   1140 94%   0.0   99.84 630
     KX757883.1
Rhipicephalus sanguineus clone #3 cytochrome oxidase subunit I...
Rhipicephalu... brown dog tick 34632   1140   1140 93%   0.0   100.00 629
     KX519708.1
Rhipicephalus sanguineus isolate SV2 cytochrome c oxidase...
Rhipicephalu... brown dog tick 34632   1140   1140 94%   0.0   99.84 630
     MW135447.1
Rhipicephalus sanguineus sensu lato 'temperate lineage' isolat...
Rhipicephalu... brown dog tick 34632   1138   1138 94%   0.0   99.68 649
     MF426014.1
Rhipicephalus sanguineus clone #2 cytochrome oxidase subunit I...

Rhipicephalu... brown dog tick 34632   1138   1138 93%   0.0   100.00 626
KX519707.1
Rhipicephalus sanguineus isolate Szekszard cytochrome oxidase...
Rhipicephalu... brown dog tick 34632   1134   1134 94%   0.0   99.68 630
KX757901.1
Rhipicephalus sanguineus voucher Rs7 cytochrome oxidase subuni...
Rhipicephalu... brown dog tick 34632   1131   1131 95%   0.0   99.05 656
KU568516.1
Rhipicephalus sanguineus clone #4 cytochrome oxidase subunit I...
Rhipicephalu... brown dog tick 34632   1129   1129 94%   0.0   99.52 635
KX519709.1
Rhipicephalus sanguineus isolate LIC4723B cytochrome oxidase...
Rhipicephalu... brown dog tick 34632   1092   1092 90%   0.0   99.66 723
KX714599.1
Rhipicephalus sanguineus clone #8 cytochrome oxidase subunit I...
Rhipicephalu... brown dog tick 34632   1072   1072 88%   0.0   100.00 580
KX519713.1
Rhipicephalus sanguineus mitochondrial DNA, complete genome
Rhipicephalu... brown dog tick 34632   1061   1061 100% 0.0   95.74 14710
AF081829.1
Rhipicephalus sanguineus clone #1 cytochrome oxidase subunit I...
Rhipicephalu... brown dog tick 34632   1053   1053 86%   0.0   100.00 595
KX519706.1
Rhipicephalus sanguineus sensu lato 'temperate lineage' isolat...
Rhipicephalu... brown dog tick 34632   1014   1014 85%   0.0   99.29 574
MF425987.1
Rhipicephalus sanguineus clone #5 cytochrome oxidase subunit I...
Rhipicephalu... brown dog tick 34632   989   989   83%   0.0   99.27 553
KX519710.1
Rhipicephalus sanguineus isolate Italy-A cytochrome oxidase...
Rhipicephalu... brown dog tick 34632   985   985   94%   0.0   95.32 630
KX757903.1
Rhipicephalus sanguineus isolate Zagreb-B cytochrome oxidase...
Rhipicephalu... brown dog tick 34632   985   985   94%   0.0   95.32 629
KX757893.1
Rhipicephalus sanguineus isolate Al-Hosema-A cytochrome oxidas...
Rhipicephalu... brown dog tick 34632   979   979   94%   0.0   95.16 630
KX757909.1
Rhipicephalus sanguineus isolate FT157 cytochrome c oxidase...
Rhipicephalu... brown dog tick 34632   963   963   79%   0.0   99.81 535
MN223690.1
Rhipicephalus sanguineus isolate FT154 cytochrome c oxidase...
Rhipicephalu... brown dog tick 34632   957   957   79%   0.0   99.62 535
MN223688.1
Rhipicephalus sanguineus isolate CE67 cytochrome c oxidase...
Rhipicephalu... brown dog tick 34632   957   957   79%   0.0   99.62 535
MN223686.1
Rhipicephalus sanguineus isolate CE61 cytochrome c oxidase...
Rhipicephalu... brown dog tick 34632   952   952   79%   0.0   99.43 535
MN223689.1
Rhipicephalus sanguineus isolate RI46 cytochrome c oxidase...
Rhipicephalu... brown dog tick 34632   952   952   79%   0.0   99.43 535
MN223687.1
Rhipicephalus sanguineus isolate RI08 cytochrome c oxidase...

Rhipicephalu... brown dog tick 34632 948 948 79% 0.0 99.43 535
MN223684.1
Rhipicephalus sanguineus isolate RI54 cytochrome c oxidase...
Rhipicephalu... brown dog tick 34632 946 946 79% 0.0 99.24 535
MN223685.1
Rhipicephalus sanguineus isolate FT044 cytochrome c oxidase...
Rhipicephalu... brown dog tick 34632 946 946 79% 0.0 99.24 535
MN223683.1
Rhipicephalus sanguineus isolate Rsang2 cytochrome oxidase...
Rhipicephalu... brown dog tick 34632 900 900 100% 0.0 91.34 790
KY678135.1
Rhipicephalus sanguineus cytochrome oxidase subunit 1 (CO1)...
Rhipicephalu... brown dog tick 34632 898 898 98% 0.0 91.67 829
KU214592.1
Rhipicephalus sanguineus isolate Gilan-e Gharb cytochrome...
Rhipicephalu... brown dog tick 34632 889 889 100% 0.0 91.03 1539
KM494916.1
Rhipicephalus sanguineus isolate Tehran cytochrome oxidase...
Rhipicephalu... brown dog tick 34632 889 889 100% 0.0 91.03 1539
KM494915.1
Rhipicephalus camicasi isolate SC0126 mitochondrion, complete...
Rhipicephalu... NA 669981 889 889 100% 0.0 91.03 14725
NC_061616.1
Rhipicephalus turanicus isolate COX1-225-Goat cytochrome c...
Rhipicephalu... NA 34633 883 883 100% 0.0 90.88 673
MT800313.1
Rhipicephalus turanicus isolate COX1-18-Sheep cytochrome c...
Rhipicephalu... NA 34633 881 881 99% 0.0 90.87 673
MT800314.1
Rhipicephalus turanicus isolate 31X cytochrome oxidase subunit...
Rhipicephalu... NA 34633 872 872 100% 0.0 90.58 873
KY606303.1
Rhipicephalus turanicus isolate 19X cytochrome oxidase subunit...
Rhipicephalu... NA 34633 872 872 100% 0.0 90.58 873
KY606302.1
Rhipicephalus turanicus isolate 19C cytochrome oxidase subunit...
Rhipicephalu... NA 34633 872 872 100% 0.0 90.58 873
KY606301.1
Rhipicephalus turanicus isolate 12X cytochrome oxidase subunit...
Rhipicephalu... NA 34633 872 872 100% 0.0 90.58 873
KY606300.1
Rhipicephalus turanicus isolate 12C cytochrome oxidase subunit...
Rhipicephalu... NA 34633 872 872 100% 0.0 90.58 873
KY606299.1
Rhipicephalus turanicus isolate COX1-7-Sheep cytochrome c...
Rhipicephalu... NA 34633 865 865 99% 0.0 90.41 673
MT800312.1
Rhipicephalus turanicus isolate GY34-1 cytochrome c oxidase...
Rhipicephalu... NA 34633 861 861 99% 0.0 90.49 678
MT079206.1
Rhipicephalus turanicus voucher CROBB808 cytochrome oxidase...
Rhipicephalu... NA 34633 861 861 100% 0.0 90.27 658
MZ305547.1

Alignments:

>Rhipicephalus sanguineus sensu lato 'temperate lineage' isolate S827 cytochrome c oxidase subunit I (COI) gene, partial cds; mitochondrial
Sequence ID: MF426019.1 Length: 697
Range 1: 19 to 676

Score:1216 bits(658), Expect:0.0,
Identities:658/658(100%), Gaps:0/658(0%), Strand: Plus/Plus

```
Query  1    AACAATATATTTAATTTTTGGAGCATGATCAGGTATATTAGGACTTAGTATAAGAATATT  60
            ||||||||||||||||||||||||||||||||||||||||||||||||||||||||||||
Sbjct  19   AACAATATATTTAATTTTTGGAGCATGATCAGGTATATTAGGACTTAGTATAAGAATATT  78

Query  61   AATTCGTATAGAATTAGGACAACCTGGAACTTTAATTGGAAATGATCAGATTTATAATGT  120
            ||||||||||||||||||||||||||||||||||||||||||||||||||||||||||||
Sbjct  79   AATTCGTATAGAATTAGGACAACCTGGAACTTTAATTGGAAATGATCAGATTTATAATGT  138

Query  121  AATCGTAACAGCACATGCATTTATTATAAtttttttATAGTTATACCAATCATAATCGG  180
            ||||||||||||||||||||||||||||||||||||||||||||||||||||||||||||
Sbjct  139  AATCGTAACAGCACATGCATTTATTATAATTTTTTTATAGTTATACCAATCATAATCGG  198

Query  181  AGGATTTGGAAATTGATTAGTCCCTATCATATTGGGTGCCCCAGATATAGCATTTCCACG  240
            ||||||||||||||||||||||||||||||||||||||||||||||||||||||||||||
Sbjct  199  AGGATTTGGAAATTGATTAGTCCCTATCATATTGGGTGCCCCAGATATAGCATTTCCACG  258

Query  241  AATAAATAATATAAGATTTTGACTTTTACCTCCTTCACTATTTTTATTAATTAACTCTTC  300
            ||||||||||||||||||||||||||||||||||||||||||||||||||||||||||||
Sbjct  259  AATAAATAATATAAGATTTTGACTTTTACCTCCTTCACTATTTTTATTAATTAACTCTTC  318

Query  301  ATTAATTGAATCTGGAGCTGGTACAGGATGAACAGTTTACCCTCCTCTATCTTCAAATTT  360
            ||||||||||||||||||||||||||||||||||||||||||||||||||||||||||||
Sbjct  319  ATTAATTGAATCTGGAGCTGGTACAGGATGAACAGTTTACCCTCCTCTATCTTCAAATTT  378

Query  361  ATCACATTATGGCCCTTCAGTAGATTTAGCTATTTTTTCTCTTCATCTTGCTGGTGCTTC  420
            ||||||||||||||||||||||||||||||||||||||||||||||||||||||||||||
Sbjct  379  ATCACATTATGGCCCTTCAGTAGATTTAGCTATTTTTTCTCTTCATCTTGCTGGTGCTTC  438

Query  421  TTCAATTTTAGGTGCAATTAACTTTATTACAACTATTGTAAACATACGATCAATTGGAAT  480
            ||||||||||||||||||||||||||||||||||||||||||||||||||||||||||||
Sbjct  439  TTCAATTTTAGGTGCAATTAACTTTATTACAACTATTGTAAACATACGATCAATTGGAAT  498

Query  481  AACAATAGAACGTATACCATTATTTGTTTGATCTGTTTTAATTACAGCTATTTTATTACT  540
            ||||||||||||||||||||||||||||||||||||||||||||||||||||||||||||
Sbjct  499  AACAATAGAACGTATACCATTATTTGTTTGATCTGTTTTAATTACAGCTATTTTATTACT  558

Query  541  TTTATCTTTACCTGTATTAGCAGGTGCCATTACAATATTGTTAACTGATCGAAACTTTAA  600
            ||||||||||||||||||||||||||||||||||||||||||||||||||||||||||||
Sbjct  559  TTTATCTTTACCTGTATTAGCAGGTGCCATTACAATATTGTTAACTGATCGAAACTTTAA  618

Query  601  TACATCATTTTTTGATCCTTCAGGGGGAGGAGATCCAATTTTATATCAACATTTATTT  658
            ||||||||||||||||||||||||||||||||||||||||||||||||||||||||||
Sbjct  619  TACATCATTTTTTGATCCTTCAGGGGGAGGAGATCCAATTTTATATCAACATTTATTT  676
```

>Rhipicephalus sanguineus haplotype III cytochrome oxidase subunit I (COI) gene, partial cds; mitochondrial
Sequence ID: KU556745.1 Length: 713
Range 1: 27 to 684

Score:1216 bits(658), Expect:0.0,
Identities:658/658(100%), Gaps:0/658(0%), Strand: Plus/Plus

```
Query  1    AACAATATATTTAATTTTTGGAGCATGATCAGGTATATTAGGACTTAGTATAAGAATATT  60
            ||||||||||||||||||||||||||||||||||||||||||||||||||||||||||||
Sbjct  27   AACAATATATTTAATTTTTGGAGCATGATCAGGTATATTAGGACTTAGTATAAGAATATT  86

Query  61   AATTCGTATAGAATTAGGACAACCTGGAACTTTAATTGGAAATGATCAGATTTATAATGT  120
            ||||||||||||||||||||||||||||||||||||||||||||||||||||||||||||
Sbjct  87   AATTCGTATAGAATTAGGACAACCTGGAACTTTAATTGGAAATGATCAGATTTATAATGT  146

Query  121  AATCGTAACAGCACATGCATTTATTATAAttttttttATAGTTATACCAATCATAATCGG  180
            ||||||||||||||||||||||||||||||||||||||||||||||||||||||||||||
Sbjct  147  AATCGTAACAGCACATGCATTTATTATAATTTTTTTTATAGTTATACCAATCATAATCGG  206

Query  181  AGGATTTGGAAATTGATTAGTCCCTATCATATTGGGTGCCCCAGATATAGCATTTCCACG  240
            ||||||||||||||||||||||||||||||||||||||||||||||||||||||||||||
Sbjct  207  AGGATTTGGAAATTGATTAGTCCCTATCATATTGGGTGCCCCAGATATAGCATTTCCACG  266

Query  241  AATAAATAATATAAGATTTTGACTTTTACCTCCTTCACTATTTTTATTAATTAACTCTTC  300
            ||||||||||||||||||||||||||||||||||||||||||||||||||||||||||||
Sbjct  267  AATAAATAATATAAGATTTTGACTTTTACCTCCTTCACTATTTTTATTAATTAACTCTTC  326

Query  301  ATTAATTGAATCTGGAGCTGGTACAGGATGAACAGTTTACCCTCCTCTATCTTCAAATTT  360
            ||||||||||||||||||||||||||||||||||||||||||||||||||||||||||||
Sbjct  327  ATTAATTGAATCTGGAGCTGGTACAGGATGAACAGTTTACCCTCCTCTATCTTCAAATTT  386

Query  361  ATCACATTATGGCCCTTCAGTAGATTTAGCTATTTTTTCTCTTCATCTTGCTGGTGCTTC  420
            ||||||||||||||||||||||||||||||||||||||||||||||||||||||||||||
Sbjct  387  ATCACATTATGGCCCTTCAGTAGATTTAGCTATTTTTTCTCTTCATCTTGCTGGTGCTTC  446

Query  421  TTCAATTTTAGGTGCAATTAACTTTATTACAACTATTGTAAACATACGATCAATTGGAAT  480
            ||||||||||||||||||||||||||||||||||||||||||||||||||||||||||||
Sbjct  447  TTCAATTTTAGGTGCAATTAACTTTATTACAACTATTGTAAACATACGATCAATTGGAAT  506

Query  481  AACAATAGAACGTATACCATTATTTGTTTGATCTGTTTTAATTACAGCTATTTTATTACT  540
            ||||||||||||||||||||||||||||||||||||||||||||||||||||||||||||
Sbjct  507  AACAATAGAACGTATACCATTATTTGTTTGATCTGTTTTAATTACAGCTATTTTATTACT  566

Query  541  TTTATCTTTACCTGTATTAGCAGGTGCCATTACAATATTGTTAACTGATCGAAACTTTAA  600
            ||||||||||||||||||||||||||||||||||||||||||||||||||||||||||||
Sbjct  567  TTTATCTTTACCTGTATTAGCAGGTGCCATTACAATATTGTTAACTGATCGAAACTTTAA  626

Query  601  TACATCATTTTTTGATCCTTCAGGGGGAGGAGATCCAATTTTATATCAACATTTATTT  658
            ||||||||||||||||||||||||||||||||||||||||||||||||||||||||||
```

```
Sbjct  627  TACATCATTTTTTGATCCTTCAGGGGGAGGAGATCCAATTTTATATCAACATTTATTT  684
```

>Rhipicephalus sanguineus isolate Arizona-1 cytochrome c oxidase subunit 1 (COI) gene, partial cds; mitochondrial
Sequence ID: MN593344.1 Length: 710
Range 1: 27 to 684

Score:1216 bits(658), Expect:0.0,
Identities:658/658(100%), Gaps:0/658(0%), Strand: Plus/Minus

```
Query  1    AACAATATATTTAATTTTTGGAGCATGATCAGGTATATTAGGACTTAGTATAAGAATATT  60
            ||||||||||||||||||||||||||||||||||||||||||||||||||||||||||||
Sbjct  684  AACAATATATTTAATTTTTGGAGCATGATCAGGTATATTAGGACTTAGTATAAGAATATT  625

Query  61   AATTCGTATAGAATTAGGACAACCTGGAACTTTAATTGGAAATGATCAGATTTATAATGT  120
            ||||||||||||||||||||||||||||||||||||||||||||||||||||||||||||
Sbjct  624  AATTCGTATAGAATTAGGACAACCTGGAACTTTAATTGGAAATGATCAGATTTATAATGT  565

Query  121  AATCGTAACAGCACATGCATTTATTATAAttttttttATAGTTATACCAATCATAATCGG  180
            ||||||||||||||||||||||||||||||||||||||||||||||||||||||||||||
Sbjct  564  AATCGTAACAGCACATGCATTTATTATAATTTTTTTTATAGTTATACCAATCATAATCGG  505

Query  181  AGGATTTGGAAATTGATTAGTCCCTATCATATTGGGTGCCCCAGATATAGCATTTCCACG  240
            ||||||||||||||||||||||||||||||||||||||||||||||||||||||||||||
Sbjct  504  AGGATTTGGAAATTGATTAGTCCCTATCATATTGGGTGCCCCAGATATAGCATTTCCACG  445

Query  241  AATAAATAATATAAGATTTTGACTTTTACCTCCTTCACTATTTTTATTAATTAACTCTTC  300
            ||||||||||||||||||||||||||||||||||||||||||||||||||||||||||||
Sbjct  444  AATAAATAATATAAGATTTTGACTTTTACCTCCTTCACTATTTTTATTAATTAACTCTTC  385

Query  301  ATTAATTGAATCTGGAGCTGGTACAGGATGAACAGTTTACCCTCCTCTATCTTCAAATTT  360
            ||||||||||||||||||||||||||||||||||||||||||||||||||||||||||||
Sbjct  384  ATTAATTGAATCTGGAGCTGGTACAGGATGAACAGTTTACCCTCCTCTATCTTCAAATTT  325

Query  361  ATCACATTATGGCCCTTCAGTAGATTTAGCTATTTTTTCTCTTCATCTTGCTGGTGCTTC  420
            ||||||||||||||||||||||||||||||||||||||||||||||||||||||||||||
Sbjct  324  ATCACATTATGGCCCTTCAGTAGATTTAGCTATTTTTTCTCTTCATCTTGCTGGTGCTTC  265

Query  421  TTCAATTTTAGGTGCAATTAACTTTATTACAACTATTGTAAACATACGATCAATTGGAAT  480
            ||||||||||||||||||||||||||||||||||||||||||||||||||||||||||||
Sbjct  264  TTCAATTTTAGGTGCAATTAACTTTATTACAACTATTGTAAACATACGATCAATTGGAAT  205

Query  481  AACAATAGAACGTATACCATTATTTGTTTGATCTGTTTTAATTACAGCTATTTTATTACT  540
            ||||||||||||||||||||||||||||||||||||||||||||||||||||||||||||
Sbjct  204  AACAATAGAACGTATACCATTATTTGTTTGATCTGTTTTAATTACAGCTATTTTATTACT  145

Query  541  TTTATCTTTACCTGTATTAGCAGGTGCCATTACAATATTGTTAACTGATCGAAACTTTAA  600
            ||||||||||||||||||||||||||||||||||||||||||||||||||||||||||||
Sbjct  144  TTTATCTTTACCTGTATTAGCAGGTGCCATTACAATATTGTTAACTGATCGAAACTTTAA  85

Query  601  TACATCATTTTTTGATCCTTCAGGGGGAGGAGATCCAATTTTATATCAACATTTATTT  658
```

```
            ||||||||||||||||||||||||||||||||||||||||||||||||||||||||
Sbjct  84   TACATCATTTTTTGATCCTTCAGGGGGAGGAGATCCAATTTTATATCAACATTTATTT  27
```

>Rhipicephalus sanguineus isolate Arizona-2 cytochrome c oxidase subunit 1 (COI) gene, partial cds; mitochondrial
Sequence ID: MN593343.1 Length: 710
Range 1: 27 to 684

Score:1216 bits(658), Expect:0.0,
Identities:658/658(100%), Gaps:0/658(0%), Strand: Plus/Minus

```
Query  1    AACAATATATTTAATTTTTGGAGCATGATCAGGTATATTAGGACTTAGTATAAGAATATT  60
            ||||||||||||||||||||||||||||||||||||||||||||||||||||||||||||
Sbjct  684  AACAATATATTTAATTTTTGGAGCATGATCAGGTATATTAGGACTTAGTATAAGAATATT  625

Query  61   AATTCGTATAGAATTAGGACAACCTGGAACTTTAATTGGAAATGATCAGATTTATAATGT  120
            ||||||||||||||||||||||||||||||||||||||||||||||||||||||||||||
Sbjct  624  AATTCGTATAGAATTAGGACAACCTGGAACTTTAATTGGAAATGATCAGATTTATAATGT  565

Query  121  AATCGTAACAGCACATGCATTTATTATAAtttttttATAGTTATACCAATCATAATCGG  180
            ||||||||||||||||||||||||||||||||||||||||||||||||||||||||||||
Sbjct  564  AATCGTAACAGCACATGCATTTATTATAATTTTTTTATAGTTATACCAATCATAATCGG  505

Query  181  AGGATTTGGAAATTGATTAGTCCCTATCATATTGGGTGCCCCAGATATAGCATTTCCACG  240
            ||||||||||||||||||||||||||||||||||||||||||||||||||||||||||||
Sbjct  504  AGGATTTGGAAATTGATTAGTCCCTATCATATTGGGTGCCCCAGATATAGCATTTCCACG  445

Query  241  AATAAATAATATAAGATTTTGACTTTTACCTCCTTCACTATTTTTATTAATTAACTCTTC  300
            ||||||||||||||||||||||||||||||||||||||||||||||||||||||||||||
Sbjct  444  AATAAATAATATAAGATTTTGACTTTTACCTCCTTCACTATTTTTATTAATTAACTCTTC  385

Query  301  ATTAATTGAATCTGGAGCTGGTACAGGATGAACAGTTTACCCTCCTCTATCTTCAAATTT  360
            ||||||||||||||||||||||||||||||||||||||||||||||||||||||||||||
Sbjct  384  ATTAATTGAATCTGGAGCTGGTACAGGATGAACAGTTTACCCTCCTCTATCTTCAAATTT  325

Query  361  ATCACATTATGGCCCTTCAGTAGATTTAGCTATTTTTTCTCTTCATCTTGCTGGTGCTTC  420
            ||||||||||||||||||||||||||||||||||||||||||||||||||||||||||||
Sbjct  324  ATCACATTATGGCCCTTCAGTAGATTTAGCTATTTTTTCTCTTCATCTTGCTGGTGCTTC  265

Query  421  TTCAATTTTAGGTGCAATTAACTTTATTACAACTATTGTAAACATACGATCAATTGGAAT  480
            ||||||||||||||||||||||||||||||||||||||||||||||||||||||||||||
Sbjct  264  TTCAATTTTAGGTGCAATTAACTTTATTACAACTATTGTAAACATACGATCAATTGGAAT  205

Query  481  AACAATAGAACGTATACCATTATTTGTTTGATCTGTTTTAATTACAGCTATTTTATTACT  540
            ||||||||||||||||||||||||||||||||||||||||||||||||||||||||||||
Sbjct  204  AACAATAGAACGTATACCATTATTTGTTTGATCTGTTTTAATTACAGCTATTTTATTACT  145

Query  541  TTTATCTTTACCTGTATTAGCAGGTGCCATTACAATATTGTTAACTGATCGAAACTTTAA  600
            ||||||||||||||||||||||||||||||||||||||||||||||||||||||||||||
Sbjct  144  TTTATCTTTACCTGTATTAGCAGGTGCCATTACAATATTGTTAACTGATCGAAACTTTAA  85
```

```
Query  601  TACATCATTTTTTGATCCTTCAGGGGGAGGAGATCCAATTTTATATCAACATTTATTT  658
            ||||||||||||||||||||||||||||||||||||||||||||||||||||||||||
Sbjct  84   TACATCATTTTTTGATCCTTCAGGGGGAGGAGATCCAATTTTATATCAACATTTATTT  27
```

>Rhipicephalus sanguineus isolate 11N cytochrome oxidase subunit I (COX1) gene, partial cds; mitochondrial
Sequence ID: MN585197.1 Length: 703
Range 1: 22 to 679

Score:1216 bits(658), Expect:0.0,
Identities:658/658(100%), Gaps:0/658(0%), Strand: Plus/Minus

```
Query  1    AACAATATATTTAATTTTTGGAGCATGATCAGGTATATTAGGACTTAGTATAAGAATATT  60
            ||||||||||||||||||||||||||||||||||||||||||||||||||||||||||||
Sbjct  679  AACAATATATTTAATTTTTGGAGCATGATCAGGTATATTAGGACTTAGTATAAGAATATT  620

Query  61   AATTCGTATAGAATTAGGACAACCTGGAACTTTAATTGGAAATGATCAGATTTATAATGT  120
            ||||||||||||||||||||||||||||||||||||||||||||||||||||||||||||
Sbjct  619  AATTCGTATAGAATTAGGACAACCTGGAACTTTAATTGGAAATGATCAGATTTATAATGT  560

Query  121  AATCGTAACAGCACATGCATTTATTATAAtttttttATAGTTATACCAATCATAATCGG  180
            ||||||||||||||||||||||||||||||||||||||||||||||||||||||||||||
Sbjct  559  AATCGTAACAGCACATGCATTTATTATAATTTTTTTATAGTTATACCAATCATAATCGG  500

Query  181  AGGATTTGGAAATTGATTAGTCCCTATCATATTGGGTGCCCCAGATATAGCATTTCCACG  240
            ||||||||||||||||||||||||||||||||||||||||||||||||||||||||||||
Sbjct  499  AGGATTTGGAAATTGATTAGTCCCTATCATATTGGGTGCCCCAGATATAGCATTTCCACG  440

Query  241  AATAAATAATATAAGATTTTGACTTTTACCTCCTTCACTATTTTTATTAATTAACTCTTC  300
            ||||||||||||||||||||||||||||||||||||||||||||||||||||||||||||
Sbjct  439  AATAAATAATATAAGATTTTGACTTTTACCTCCTTCACTATTTTTATTAATTAACTCTTC  380

Query  301  ATTAATTGAATCTGGAGCTGGTACAGGATGAACAGTTTACCCTCCTCTATCTTCAAATTT  360
            ||||||||||||||||||||||||||||||||||||||||||||||||||||||||||||
Sbjct  379  ATTAATTGAATCTGGAGCTGGTACAGGATGAACAGTTTACCCTCCTCTATCTTCAAATTT  320

Query  361  ATCACATTATGGCCCTTCAGTAGATTTAGCTATTTTTTCTCTTCATCTTGCTGGTGCTTC  420
            ||||||||||||||||||||||||||||||||||||||||||||||||||||||||||||
Sbjct  319  ATCACATTATGGCCCTTCAGTAGATTTAGCTATTTTTTCTCTTCATCTTGCTGGTGCTTC  260

Query  421  TTCAATTTTAGGTGCAATTAACTTTATTACAACTATTGTAAACATACGATCAATTGGAAT  480
            ||||||||||||||||||||||||||||||||||||||||||||||||||||||||||||
Sbjct  259  TTCAATTTTAGGTGCAATTAACTTTATTACAACTATTGTAAACATACGATCAATTGGAAT  200

Query  481  AACAATAGAACGTATACCATTATTTGTTTGATCTGTTTTAATTACAGCTATTTTATTACT  540
            ||||||||||||||||||||||||||||||||||||||||||||||||||||||||||||
Sbjct  199  AACAATAGAACGTATACCATTATTTGTTTGATCTGTTTTAATTACAGCTATTTTATTACT  140

Query  541  TTTATCTTTACCTGTATTAGCAGGTGCCATTACAATATTGTTAACTGATCGAAACTTTAA  600
            ||||||||||||||||||||||||||||||||||||||||||||||||||||||||||||
Sbjct  139  TTTATCTTTACCTGTATTAGCAGGTGCCATTACAATATTGTTAACTGATCGAAACTTTAA  80
```

```
Query  601   TACATCATTTTTTGATCCTTCAGGGGGAGGAGATCCAATTTTATATCAACATTTATTT  658
             ||||||||||||||||||||||||||||||||||||||||||||||||||||||||||
Sbjct  79    TACATCATTTTTTGATCCTTCAGGGGGAGGAGATCCAATTTTATATCAACATTTATTT  22
```

>Rhipicephalus sanguineus cytochrome c oxidase I (COI) gene, partial cds;
mitochondrial gene for mitochondrial product
Sequence ID: AF132839.1 Length: 793
Range 1: 42 to 699

Score:1216 bits(658), Expect:0.0,
Identities:658/658(100%), Gaps:0/658(0%), Strand: Plus/Plus

```
Query  1     AACAATATATTTAATTTTTGGAGCATGATCAGGTATATTAGGACTTAGTATAAGAATATT  60
             ||||||||||||||||||||||||||||||||||||||||||||||||||||||||||||
Sbjct  42    AACAATATATTTAATTTTTGGAGCATGATCAGGTATATTAGGACTTAGTATAAGAATATT  101

Query  61    AATTCGTATAGAATTAGGACAACCTGGAACTTTAATTGGAAATGATCAGATTTATAATGT  120
             ||||||||||||||||||||||||||||||||||||||||||||||||||||||||||||
Sbjct  102   AATTCGTATAGAATTAGGACAACCTGGAACTTTAATTGGAAATGATCAGATTTATAATGT  161

Query  121   AATCGTAACAGCACATGCATTTATTATAAttttttttATAGTTATACCAATCATAATCGG  180
             ||||||||||||||||||||||||||||||||||||||||||||||||||||||||||||
Sbjct  162   AATCGTAACAGCACATGCATTTATTATAATTTTTTTTATAGTTATACCAATCATAATCGG  221

Query  181   AGGATTTGGAAATTGATTAGTCCCTATCATATTGGGTGCCCCAGATATAGCATTTCCACG  240
             ||||||||||||||||||||||||||||||||||||||||||||||||||||||||||||
Sbjct  222   AGGATTTGGAAATTGATTAGTCCCTATCATATTGGGTGCCCCAGATATAGCATTTCCACG  281

Query  241   AATAAATAATATAAGATTTTGACTTTTACCTCCTTCACTATTTTTATTAATTAACTCTTC  300
             ||||||||||||||||||||||||||||||||||||||||||||||||||||||||||||
Sbjct  282   AATAAATAATATAAGATTTTGACTTTTACCTCCTTCACTATTTTTATTAATTAACTCTTC  341

Query  301   ATTAATTGAATCTGGAGCTGGTACAGGATGAACAGTTTACCCTCCTCTATCTTCAAATTT  360
             ||||||||||||||||||||||||||||||||||||||||||||||||||||||||||||
Sbjct  342   ATTAATTGAATCTGGAGCTGGTACAGGATGAACAGTTTACCCTCCTCTATCTTCAAATTT  401

Query  361   ATCACATTATGGCCCTTCAGTAGATTTAGCTATTTTTTCTCTTCATCTTGCTGGTGCTTC  420
             ||||||||||||||||||||||||||||||||||||||||||||||||||||||||||||
Sbjct  402   ATCACATTATGGCCCTTCAGTAGATTTAGCTATTTTTTCTCTTCATCTTGCTGGTGCTTC  461

Query  421   TTCAATTTTAGGTGCAATTAACTTTATTACAACTATTGTAAACATACGATCAATTGGAAT  480
             ||||||||||||||||||||||||||||||||||||||||||||||||||||||||||||
Sbjct  462   TTCAATTTTAGGTGCAATTAACTTTATTACAACTATTGTAAACATACGATCAATTGGAAT  521

Query  481   AACAATAGAACGTATACCATTATTTGTTTGATCTGTTTTAATTACAGCTATTTTATTACT  540
             ||||||||||||||||||||||||||||||||||||||||||||||||||||||||||||
Sbjct  522   AACAATAGAACGTATACCATTATTTGTTTGATCTGTTTTAATTACAGCTATTTTATTACT  581

Query  541   TTTATCTTTACCTGTATTAGCAGGTGCCATTACAATATTGTTAACTGATCGAAACTTTAA  600
             ||||||||||||||||||||||||||||||||||||||||||||||||||||||||||||
```

```
Sbjct  582  TTTATCTTTACCTGTATTAGCAGGTGCCATTACAATATTGTTAACTGATCGAAACTTTAA  641

Query  601  TACATCATTTTTTGATCCTTCAGGGGGAGGAGATCCAATTTTATATCAACATTTATTT  658
            ||||||||||||||||||||||||||||||||||||||||||||||||||||||||||
Sbjct  642  TACATCATTTTTTGATCCTTCAGGGGGAGGAGATCCAATTTTATATCAACATTTATTT  699
```

```
>Rhipicephalus sanguineus sensu lato 'temperate lineage' isolate S58 cytochrome
c oxidase subunit I (COI) gene, partial cds; mitochondrial
Sequence ID: MF426018.1 Length: 675
Range 1: 11 to 668

Score:1210 bits(655), Expect:0.0,
Identities:657/658(99%),  Gaps:0/658(0%), Strand: Plus/Plus

Query  1    AACAATATATTTAATTTTTGGAGCATGATCAGGTATATTAGGACTTAGTATAAGAATATT  60
            ||||||||||||||||||||||||||||||||||||||||||||||||||||||||||||
Sbjct  11   AACAATATATTTAATTTTTGGAGCATGATCAGGTATATTAGGACTTAGTATAAGAATATT  70

Query  61   AATTCGTATAGAATTAGGACAACCTGGAACTTTAATTGGAAATGATCAGATTTATAATGT  120
            ||||||||||||||||||||||||||||||||||||||||||||||||||||||||||||
Sbjct  71   AATTCGTATAGAATTAGGACAACCTGGAACTTTAATTGGAAATGATCAGATTTATAATGT  130

Query  121  AATCGTAACAGCACATGCATTTATTATAAtttttttATAGTTATACCAATCATAATCGG  180
            ||||||||||||||||||||||||||||||||||||||||||||||||||||||||||||
Sbjct  131  AATCGTAACAGCACATGCATTTATTATAATTTTTTTATAGTTATACCAATCATAATCGG  190

Query  181  AGGATTTGGAAATTGATTAGTCCCTATCATATTGGGTGCCCCAGATATAGCATTTCCACG  240
            ||||||||||||||||||||||||||||||||||||||||||||||||||||||||||||
Sbjct  191  AGGATTTGGAAATTGATTAGTCCCTATCATATTGGGTGCCCCAGATATAGCATTTCCACG  250

Query  241  AATAAATAATATAAGATTTTGACTTTTACCTCCTTCACTATTTTTATTAATTAACTCTTC  300
            ||||||||||||||||||||||||||||||||||||||||||||||||||||||||||||
Sbjct  251  AATAAATAATATAAGATTTTGACTTTTACCTCCTTCACTATTTTTATTAATTAACTCTTC  310

Query  301  ATTAATTGAATCTGGAGCTGGTACAGGATGAACAGTTTACCCTCCTCTATCTTCAAATTT  360
            ||||||||||||||||||||||||||||||||||||||||||||||||||||||||||||
Sbjct  311  ATTAATTGAATCTGGAGCTGGTACAGGATGAACAGTTTACCCTCCTCTATCTTCAAATTT  370

Query  361  ATCACATTATGGCCCTTCAGTAGATTTAGCTATTTTTTCTCTTCATCTTGCTGGTGCTTC  420
            |||||||||||| |||||||||||||||||||||||||||||||||||||||||||||||
Sbjct  371  ATCACATTATGGTCCTTCAGTAGATTTAGCTATTTTTTCTCTTCATCTTGCTGGTGCTTC  430

Query  421  TTCAATTTTAGGTGCAATTAACTTTATTACAACTATTGTAAACATACGATCAATTGGAAT  480
            ||||||||||||||||||||||||||||||||||||||||||||||||||||||||||||
Sbjct  431  TTCAATTTTAGGTGCAATTAACTTTATTACAACTATTGTAAACATACGATCAATTGGAAT  490

Query  481  AACAATAGAACGTATACCATTATTTGTTTGATCTGTTTTAATTACAGCTATTTTATTACT  540
            ||||||||||||||||||||||||||||||||||||||||||||||||||||||||||||
Sbjct  491  AACAATAGAACGTATACCATTATTTGTTTGATCTGTTTTAATTACAGCTATTTTATTACT  550

Query  541  TTTATCTTTACCTGTATTAGCAGGTGCCATTACAATATTGTTAACTGATCGAAACTTTAA  600
```

```
                ||||||||||||||||||||||||||||||||||||||||||||||||||||||||||||
Sbjct  551  TTTATCTTTACCTGTATTAGCAGGTGCCATTACAATATTGTTAACTGATCGAAACTTTAA  610

Query  601  TACATCATTTTTTGATCCTTCAGGGGGAGGAGATCCAATTTTATATCAACATTTATTT  658
            ||||||||||||||||||||||||||||||||||||||||||||||||||||||||||
Sbjct  611  TACATCATTTTTTGATCCTTCAGGGGGAGGAGATCCAATTTTATATCAACATTTATTT  668
```

>Rhipicephalus sanguineus sensu lato 'temperate lineage' isolate S52 cytochrome c oxidase subunit I (COI) gene, partial cds; mitochondrial
Sequence ID: MF426016.1 Length: 680
Range 1: 12 to 669

Score:1210 bits(655), Expect:0.0,
Identities:657/658(99%), Gaps:0/658(0%), Strand: Plus/Plus

```
Query  1    AACAATATATTTAATTTTTGGAGCATGATCAGGTATATTAGGACTTAGTATAAGAATATT  60
            ||||||||||||||||||||||||||||||||||||||||||||||||||||||||||||
Sbjct  12   AACAATATATTTAATTTTTGGAGCATGATCAGGTATATTAGGACTTAGTATAAGAATATT  71

Query  61   AATTCGTATAGAATTAGGACAACCTGGAACTTTAATTGGAAATGATCAGATTTATAATGT  120
            ||||||||||||||||||||||||||||||||||||||||||||||||||||||||||||
Sbjct  72   AATTCGTATAGAATTAGGACAACCTGGAACTTTAATTGGAAATGATCAGATTTATAATGT  131

Query  121  AATCGTAACAGCACATGCATTTATTATAAttttttttATAGTTATACCAATCATAATCGG  180
            ||||||||||||||||||||||||||||||||||||||||||||||||||||||||||||
Sbjct  132  AATCGTAACAGCACATGCATTTATTATAATTTTTTTTATAGTTATACCAATCATAATCGG  191

Query  181  AGGATTTGGAAATTGATTAGTCCCTATCATATTGGGTGCCCCAGATATAGCATTTCCACG  240
            ||||||||||||||||||||||||||||||||||||||||||||||||||||||||||||
Sbjct  192  AGGATTTGGAAATTGATTAGTCCCTATCATATTGGGTGCCCCAGATATAGCATTTCCACG  251

Query  241  AATAAATAATATAAGATTTTGACTTTTACCTCCTTCACTATTTTTATTAATTAACTCTTC  300
            ||||||||||||||||||||||||||||||||||||||||||||||||||||||||||||
Sbjct  252  AATAAATAATATAAGATTTTGACTTTTACCTCCTTCACTATTTTTATTAATTAACTCTTC  311

Query  301  ATTAATTGAATCTGGAGCTGGTACAGGATGAACAGTTTACCCTCCTCTATCTTCAAATTT  360
            ||||||||||||||||||||||||||||||||||||||||||||||||||||||||||||
Sbjct  312  ATTAATTGAATCTGGAGCTGGTACAGGATGAACAGTTTACCCTCCTCTATCTTCAAATTT  371

Query  361  ATCACATTATGGCCCTTCAGTAGATTTAGCTATTTTTTCTCTTCATCTTGCTGGTGCTTC  420
            |||||||||||| |||||||||||||||||||||||||||||||||||||||||||||||
Sbjct  372  ATCACATTATGGTCCTTCAGTAGATTTAGCTATTTTTTCTCTTCATCTTGCTGGTGCTTC  431

Query  421  TTCAATTTTAGGTGCAATTAACTTTATTACAACTATTGTAAACATACGATCAATTGGAAT  480
            ||||||||||||||||||||||||||||||||||||||||||||||||||||||||||||
Sbjct  432  TTCAATTTTAGGTGCAATTAACTTTATTACAACTATTGTAAACATACGATCAATTGGAAT  491

Query  481  AACAATAGAACGTATACCATTATTTGTTTGATCTGTTTTAATTACAGCTATTTTATTACT  540
            ||||||||||||||||||||||||||||||||||||||||||||||||||||||||||||
Sbjct  492  AACAATAGAACGTATACCATTATTTGTTTGATCTGTTTTAATTACAGCTATTTTATTACT  551
```

```
Query  541   TTTATCTTTACCTGTATTAGCAGGTGCCATTACAATATTGTTAACTGATCGAAACTTTAA  600
             ||||||||||||||||||||||||||||||||||||||||||||||||||||||||||||
Sbjct  552   TTTATCTTTACCTGTATTAGCAGGTGCCATTACAATATTGTTAACTGATCGAAACTTTAA  611

Query  601   TACATCATTTTTTGATCCTTCAGGGGGAGGAGATCCAATTTTATATCAACATTTATTT  658
             ||||||||||||||||||||||||||||||||||||||||||||||||||||||||||
Sbjct  612   TACATCATTTTTTGATCCTTCAGGGGGAGGAGATCCAATTTTATATCAACATTTATTT  669
```

>Rhipicephalus sanguineus sensu lato 'temperate lineage' isolate S37 cytochrome c oxidase subunit I (COI) gene, partial cds; mitochondrial
Sequence ID: MF426013.1 Length: 693
Range 1: 20 to 677

Score:1210 bits(655), Expect:0.0,
Identities:657/658(99%), Gaps:0/658(0%), Strand: Plus/Plus

```
Query  1     AACAATATATTTAATTTTTGGAGCATGATCAGGTATATTAGGACTTAGTATAAGAATATT  60
             ||||||||||||||||||||||||||||||||||||||||||||||||||||||||||||
Sbjct  20    AACAATATATTTAATTTTTGGAGCATGATCAGGTATATTAGGACTTAGTATAAGAATATT  79

Query  61    AATTCGTATAGAATTAGGACAACCTGGAACTTTAATTGGAAATGATCAGATTTATAATGT  120
             ||||||||||||||||||||||||||||||||||||||||||||||||||||||||||||
Sbjct  80    AATTCGTATAGAATTAGGACAACCTGGAACTTTAATTGGAAATGATCAGATTTATAATGT  139

Query  121   AATCGTAACAGCACATGCATTTATTATAAttttttttATAGTTATACCAATCATAATCGG  180
             ||||||||||||||||||||||||||||||||||||||||||||||||||||||||||||
Sbjct  140   AATCGTAACAGCACATGCATTTATTATAATTTTTTTTATAGTTATACCAATCATAATCGG  199

Query  181   AGGATTTGGAAATTGATTAGTCCCTATCATATTGGGTGCCCCAGATATAGCATTTCCACG  240
             ||||||||||||||||||||||||||||||||||||||||||||||||||||||||||||
Sbjct  200   AGGATTTGGAAATTGATTAGTCCCTATCATATTGGGTGCCCCAGATATAGCATTTCCACG  259

Query  241   AATAAATAATATAAGATTTTGACTTTTACCTCCTTCACTATTTTTATTAATTAACTCTTC  300
             ||||||||||||||||||||||||||||||||||||||||||||||||||||||||||||
Sbjct  260   AATAAATAATATAAGATTTTGACTTTTACCTCCTTCACTATTTTTATTAATTAACTCTTC  319

Query  301   ATTAATTGAATCTGGAGCTGGTACAGGATGAACAGTTTACCCTCCTCTATCTTCAAATTT  360
             ||||||||||||||||||||||||||||||||||||||||||||||||||||||||||||
Sbjct  320   ATTAATTGAATCTGGAGCTGGTACAGGATGAACAGTTTACCCTCCTCTATCTTCAAATTT  379

Query  361   ATCACATTATGGCCCTTCAGTAGATTTAGCTATTTTTTCTCTTCATCTTGCTGGTGCTTC  420
             |||||||||||| |||||||||||||||||||||||||||||||||||||||||||||||
Sbjct  380   ATCACATTATGGTCCTTCAGTAGATTTAGCTATTTTTTCTCTTCATCTTGCTGGTGCTTC  439

Query  421   TTCAATTTTAGGTGCAATTAACTTTATTACAACTATTGTAAACATACGATCAATTGGAAT  480
             ||||||||||||||||||||||||||||||||||||||||||||||||||||||||||||
Sbjct  440   TTCAATTTTAGGTGCAATTAACTTTATTACAACTATTGTAAACATACGATCAATTGGAAT  499

Query  481   AACAATAGAACGTATACCATTATTTGTTTGATCTGTTTTAATTACAGCTATTTTATTACT  540
             ||||||||||||||||||||||||||||||||||||||||||||||||||||||||||||
Sbjct  500   AACAATAGAACGTATACCATTATTTGTTTGATCTGTTTTAATTACAGCTATTTTATTACT  559
```

```
Query  541   TTTATCTTTACCTGTATTAGCAGGTGCCATTACAATATTGTTAACTGATCGAAACTTTAA  600
             ||||||||||||||||||||||||||||||||||||||||||||||||||||||||||||
Sbjct  560   TTTATCTTTACCTGTATTAGCAGGTGCCATTACAATATTGTTAACTGATCGAAACTTTAA  619

Query  601   TACATCATTTTTTGATCCTTCAGGGGGAGGAGATCCAATTTTATATCAACATTTATTT  658
             ||||||||||||||||||||||||||||||||||||||||||||||||||||||||||
Sbjct  620   TACATCATTTTTTGATCCTTCAGGGGGAGGAGATCCAATTTTATATCAACATTTATTT  677
```

>Rhipicephalus sanguineus sensu lato 'temperate lineage' isolate S33 cytochrome c oxidase subunit I (COI) gene, partial cds; mitochondrial
Sequence ID: MF426009.1 Length: 679
Range 1: 11 to 668

Score:1210 bits(655), Expect:0.0,
Identities:657/658(99%), Gaps:0/658(0%), Strand: Plus/Plus

```
Query  1     AACAATATATTTAATTTTTGGAGCATGATCAGGTATATTAGGACTTAGTATAAGAATATT  60
             ||||||||||||||||||||||||||||||||||||||||||||||||||||||||||||
Sbjct  11    AACAATATATTTAATTTTTGGAGCATGATCAGGTATATTAGGACTTAGTATAAGAATATT  70

Query  61    AATTCGTATAGAATTAGGACAACCTGGAACTTTAATTGGAAATGATCAGATTTATAATGT  120
             ||||||||||||||||||||||||||||||||||||||||||||||||||||||||||||
Sbjct  71    AATTCGTATAGAATTAGGACAACCTGGAACTTTAATTGGAAATGATCAGATTTATAATGT  130

Query  121   AATCGTAACAGCACATGCATTTATTATAAttttttttATAGTTATACCAATCATAATCGG  180
             ||||||||||||||||||||||||||||||||||||||||||||||||||||||||||||
Sbjct  131   AATCGTAACAGCACATGCATTTATTATAATTTTTTTTATAGTTATACCAATCATAATCGG  190

Query  181   AGGATTTGGAAATTGATTAGTCCCTATCATATTGGGTGCCCCAGATATAGCATTTCCACG  240
             ||||||||||||||||||||||||||||||||||||||||||||||||||||||||||||
Sbjct  191   AGGATTTGGAAATTGATTAGTCCCTATCATATTGGGTGCCCCAGATATAGCATTTCCACG  250

Query  241   AATAAATAATATAAGATTTTGACTTTTACCTCCTTCACTATTTTTATTAATTAACTCTTC  300
             ||||||||||||||||||||||||||||||||||||||||||||||||||||||||||||
Sbjct  251   AATAAATAATATAAGATTTTGACTTTTACCTCCTTCACTATTTTTATTAATTAACTCTTC  310

Query  301   ATTAATTGAATCTGGAGCTGGTACAGGATGAACAGTTTACCCTCCTCTATCTTCAAATTT  360
             ||||||||||||||||||||||||||||||||||||||||||||||||||||||||||||
Sbjct  311   ATTAATTGAATCTGGAGCTGGTACAGGATGAACAGTTTACCCTCCTCTATCTTCAAATTT  370

Query  361   ATCACATTATGGCCCTTCAGTAGATTTAGCTATTTTTTCTCTTCATCTTGCTGGTGCTTC  420
             |||||||||||| |||||||||||||||||||||||||||||||||||||||||||||||
Sbjct  371   ATCACATTATGGTCCTTCAGTAGATTTAGCTATTTTTTCTCTTCATCTTGCTGGTGCTTC  430

Query  421   TTCAATTTTAGGTGCAATTAACTTTATTACAACTATTGTAAACATACGATCAATTGGAAT  480
             ||||||||||||||||||||||||||||||||||||||||||||||||||||||||||||
Sbjct  431   TTCAATTTTAGGTGCAATTAACTTTATTACAACTATTGTAAACATACGATCAATTGGAAT  490

Query  481   AACAATAGAACGTATACCATTATTTGTTTGATCTGTTTTAATTACAGCTATTTTATTACT  540
             ||||||||||||||||||||||||||||||||||||||||||||||||||||||||||||
```

```
Sbjct  491   AACAATAGAACGTATACCATTATTTGTTTGATCTGTTTTAATTACAGCTATTTTATTACT  550

Query  541   TTTATCTTTACCTGTATTAGCAGGTGCCATTACAATATTGTTAACTGATCGAAACTTTAA  600
             ||||||||||||||||||||||||||||||||||||||||||||||||||||||||||||
Sbjct  551   TTTATCTTTACCTGTATTAGCAGGTGCCATTACAATATTGTTAACTGATCGAAACTTTAA  610

Query  601   TACATCATTTTTTGATCCTTCAGGGGGAGGAGATCCAATTTTATATCAACATTTATTT  658
             ||||||||||||||||||||||||||||||||||||||||||||||||||||||||||
Sbjct  611   TACATCATTTTTTGATCCTTCAGGGGGAGGAGATCCAATTTTATATCAACATTTATTT  668
```

```
>Rhipicephalus sanguineus sensu lato 'temperate lineage' isolate S32 cytochrome
c oxidase subunit I (COI) gene, partial cds; mitochondrial
Sequence ID: MF426008.1 Length: 690
Range 1: 14 to 671

Score:1210 bits(655), Expect:0.0,
Identities:657/658(99%),  Gaps:0/658(0%), Strand: Plus/Plus

Query  1     AACAATATATTTAATTTTTGGAGCATGATCAGGTATATTAGGACTTAGTATAAGAATATT  60
             ||||||||||||||||||||||||||||||||||||||||||||||||||||||||||||
Sbjct  14    AACAATATATTTAATTTTTGGAGCATGATCAGGTATATTAGGACTTAGTATAAGAATATT  73

Query  61    AATTCGTATAGAATTAGGACAACCTGGAACTTTAATTGGAAATGATCAGATTTATAATGT  120
             ||||||||||||||||||||||||||||||||||||||||||||||||||||||||||||
Sbjct  74    AATTCGTATAGAATTAGGACAACCTGGAACTTTAATTGGAAATGATCAGATTTATAATGT  133

Query  121   AATCGTAACAGCACATGCATTTATTATAAtttttttATAGTTATACCAATCATAATCGG  180
             ||||||||||||||||||||||||||||||||||||||||||||||||||||||||||||
Sbjct  134   AATCGTAACAGCACATGCATTTATTATAATTTTTTTATAGTTATACCAATCATAATCGG  193

Query  181   AGGATTTGGAAATTGATTAGTCCCTATCATATTGGGTGCCCCAGATATAGCATTTCCACG  240
             ||||||||||||||||||||||||||||||||||||||||||||||||||||||||||||
Sbjct  194   AGGATTTGGAAATTGATTAGTCCCTATCATATTGGGTGCCCCAGATATAGCATTTCCACG  253

Query  241   AATAAATAATATAAGATTTTGACTTTTACCTCCTTCACTATTTTTATTAATTAACTCTTC  300
             ||||||||||||||||||||||||||||||||||||||||||||||||||||||||||||
Sbjct  254   AATAAATAATATAAGATTTTGACTTTTACCTCCTTCACTATTTTTATTAATTAACTCTTC  313

Query  301   ATTAATTGAATCTGGAGCTGGTACAGGATGAACAGTTTACCCTCCTCTATCTTCAAATTT  360
             ||||||||||||||||||||||||||||||||||||||||||||||||||||||||||||
Sbjct  314   ATTAATTGAATCTGGAGCTGGTACAGGATGAACAGTTTACCCTCCTCTATCTTCAAATTT  373

Query  361   ATCACATTATGGCCCTTCAGTAGATTTAGCTATTTTTTCTCTTCATCTTGCTGGTGCTTC  420
             |||||||||||||||||||||||||||||||||| |||||||||||||||||||||||||
Sbjct  374   ATCACATTATGGCCCTTCAGTAGATTTAGCTATCTTTTCTCTTCATCTTGCTGGTGCTTC  433

Query  421   TTCAATTTTAGGTGCAATTAACTTTATTACAACTATTGTAAACATACGATCAATTGGAAT  480
             ||||||||||||||||||||||||||||||||||||||||||||||||||||||||||||
Sbjct  434   TTCAATTTTAGGTGCAATTAACTTTATTACAACTATTGTAAACATACGATCAATTGGAAT  493

Query  481   AACAATAGAACGTATACCATTATTTGTTTGATCTGTTTTAATTACAGCTATTTTATTACT  540
```

```
           ||||||||||||||||||||||||||||||||||||||||||||||||||||||||||||
Sbjct  494  AACAATAGAACGTATACCATTATTTGTTTGATCTGTTTTAATTACAGCTATTTTATTACT  553

Query  541  TTTATCTTTACCTGTATTAGCAGGTGCCATTACAATATTGTTAACTGATCGAAACTTTAA  600
           ||||||||||||||||||||||||||||||||||||||||||||||||||||||||||||
Sbjct  554  TTTATCTTTACCTGTATTAGCAGGTGCCATTACAATATTGTTAACTGATCGAAACTTTAA  613

Query  601  TACATCATTTTTTGATCCTTCAGGGGGAGGAGATCCAATTTTATATCAACATTTATTT  658
           ||||||||||||||||||||||||||||||||||||||||||||||||||||||||||
Sbjct  614  TACATCATTTTTTGATCCTTCAGGGGGAGGAGATCCAATTTTATATCAACATTTATTT  671
```

>Rhipicephalus sanguineus sensu lato 'temperate lineage' isolate S29 cytochrome c oxidase subunit I (COI) gene, partial cds; mitochondrial
Sequence ID: MF426007.1 Length: 686
Range 1: 14 to 671

Score:1210 bits(655), Expect:0.0,
Identities:657/658(99%), Gaps:0/658(0%), Strand: Plus/Plus

```
Query  1    AACAATATATTTAATTTTTGGAGCATGATCAGGTATATTAGGACTTAGTATAAGAATATT  60
           ||||||||||||||||||||||||||||||||||||||||||||||||||||||||||||
Sbjct  14   AACAATATATTTAATTTTTGGAGCATGATCAGGTATATTAGGACTTAGTATAAGAATATT  73

Query  61   AATTCGTATAGAATTAGGACAACCTGGAACTTTAATTGGAAATGATCAGATTTATAATGT  120
           ||||||||||||||||||||||||||||||||||||||||||||||||||||||||||||
Sbjct  74   AATTCGTATAGAATTAGGACAACCTGGAACTTTAATTGGAAATGATCAGATTTATAATGT  133

Query  121  AATCGTAACAGCACATGCATTTATTATAAttttttttATAGTTATACCAATCATAATCGG  180
           ||||||||||||||||||||||||||||||||||||||||||||||||||||||||||||
Sbjct  134  AATCGTAACAGCACATGCATTTATTATAATTTTTTTTATAGTTATACCAATCATAATCGG  193

Query  181  AGGATTTGGAAATTGATTAGTCCCTATCATATTGGGTGCCCCAGATATAGCATTTCCACG  240
           ||||||||||||||||||||||||||||||||||||||||||||||||||||||||||||
Sbjct  194  AGGATTTGGAAATTGATTAGTCCCTATCATATTGGGTGCCCCAGATATAGCATTTCCACG  253

Query  241  AATAAATAATATAAGATTTTGACTTTTACCTCCTTCACTATTTTTATTAATTAACTCTTC  300
           ||||||||||||||||||||||||||||||||||||||||||||||||||||||||||||
Sbjct  254  AATAAATAATATAAGATTTTGACTTTTACCTCCTTCACTATTTTTATTAATTAACTCTTC  313

Query  301  ATTAATTGAATCTGGAGCTGGTACAGGATGAACAGTTTACCCTCCTCTATCTTCAAATTT  360
           ||||||||||||||||||||||||||||||||||||||||||||||||||||||||||||
Sbjct  314  ATTAATTGAATCTGGAGCTGGTACAGGATGAACAGTTTACCCTCCTCTATCTTCAAATTT  373

Query  361  ATCACATTATGGCCCTTCAGTAGATTTAGCTATTTTTTCTCTTCATCTTGCTGGTGCTTC  420
           |||||||||||| |||||||||||||||||||||||||||||||||||||||||||||||
Sbjct  374  ATCACATTATGGTCCTTCAGTAGATTTAGCTATTTTTTCTCTTCATCTTGCTGGTGCTTC  433

Query  421  TTCAATTTTAGGTGCAATTAACTTTATTACAACTATTGTAAACATACGATCAATTGGAAT  480
           ||||||||||||||||||||||||||||||||||||||||||||||||||||||||||||
Sbjct  434  TTCAATTTTAGGTGCAATTAACTTTATTACAACTATTGTAAACATACGATCAATTGGAAT  493
```

```
Query  481   AACAATAGAACGTATACCATTATTTGTTTGATCTGTTTTAATTACAGCTATTTTATTACT  540
             ||||||||||||||||||||||||||||||||||||||||||||||||||||||||||||
Sbjct  494   AACAATAGAACGTATACCATTATTTGTTTGATCTGTTTTAATTACAGCTATTTTATTACT  553

Query  541   TTTATCTTTACCTGTATTAGCAGGTGCCATTACAATATTGTTAACTGATCGAAACTTTAA  600
             ||||||||||||||||||||||||||||||||||||||||||||||||||||||||||||
Sbjct  554   TTTATCTTTACCTGTATTAGCAGGTGCCATTACAATATTGTTAACTGATCGAAACTTTAA  613

Query  601   TACATCATTTTTTGATCCTTCAGGGGGAGGAGATCCAATTTTATATCAACATTTATTT  658
             ||||||||||||||||||||||||||||||||||||||||||||||||||||||||||
Sbjct  614   TACATCATTTTTTGATCCTTCAGGGGGAGGAGATCCAATTTTATATCAACATTTATTT  671
```

>Rhipicephalus sanguineus sensu lato 'temperate lineage' isolate CR1570 cytochrome c oxidase subunit I (COI) gene, partial cds; mitochondrial
Sequence ID: MF426001.1 Length: 684
Range 1: 14 to 671

Score:1210 bits(655), Expect:0.0,
Identities:657/658(99%), Gaps:0/658(0%), Strand: Plus/Plus

```
Query  1     AACAATATATTTAATTTTTGGAGCATGATCAGGTATATTAGGACTTAGTATAAGAATATT  60
             ||||||||||||||||||||||||||||||||||||||||||||||||||||||||||||
Sbjct  14    AACAATATATTTAATTTTTGGAGCATGATCAGGTATATTAGGACTTAGTATAAGAATATT  73

Query  61    AATTCGTATAGAATTAGGACAACCTGGAACTTTAATTGGAAATGATCAGATTTATAATGT  120
             ||||||||||||||||||||||||||||||||||||||||||||||||||||||||||||
Sbjct  74    AATTCGTATAGAATTAGGACAACCTGGAACTTTAATTGGAAATGATCAGATTTATAATGT  133

Query  121   AATCGTAACAGCACATGCATTTATTATAAttttttttATAGTTATACCAATCATAATCGG  180
             ||||||||||||||||||||||||||||||||||||||||||||||||||||||||||||
Sbjct  134   AATCGTAACAGCACATGCATTTATTATAATTTTTTTTATAGTTATACCAATCATAATCGG  193

Query  181   AGGATTTGGAAATTGATTAGTCCCTATCATATTGGGTGCCCCAGATATAGCATTTCCACG  240
             ||||||||||||||||||||||||||||||||||||||||||||||||||||||||||||
Sbjct  194   AGGATTTGGAAATTGATTAGTCCCTATCATATTGGGTGCCCCAGATATAGCATTTCCACG  253

Query  241   AATAAATAATATAAGATTTTGACTTTTACCTCCTTCACTATTTTTATTAATTAACTCTTC  300
             ||||||||||||||||||||||||||||||||||||||||||||||||||||||||||||
Sbjct  254   AATAAATAATATAAGATTTTGACTTTTACCTCCTTCACTATTTTTATTAATTAACTCTTC  313

Query  301   ATTAATTGAATCTGGAGCTGGTACAGGATGAACAGTTTACCCTCCTCTATCTTCAAATTT  360
             ||||||||||||||||||||||||||||||||||||||||||||||||||||||||||||
Sbjct  314   ATTAATTGAATCTGGAGCTGGTACAGGATGAACAGTTTACCCTCCTCTATCTTCAAATTT  373

Query  361   ATCACATTATGGCCCTTCAGTAGATTTAGCTATTTTTTCTCTTCATCTTGCTGGTGCTTC  420
             ||||||||||||||||||||||||||||||||| ||||||||||||||||||||||||||
Sbjct  374   ATCACATTATGGCCCTTCAGTAGATTTAGCTATCTTTTCTCTTCATCTTGCTGGTGCTTC  433

Query  421   TTCAATTTTAGGTGCAATTAACTTTATTACAACTATTGTAAACATACGATCAATTGGAAT  480
             ||||||||||||||||||||||||||||||||||||||||||||||||||||||||||||
Sbjct  434   TTCAATTTTAGGTGCAATTAACTTTATTACAACTATTGTAAACATACGATCAATTGGAAT  493
```

```
Query  481   AACAATAGAACGTATACCATTATTTGTTTGATCTGTTTTAATTACAGCTATTTTATTACT  540
             ||||||||||||||||||||||||||||||||||||||||||||||||||||||||||||
Sbjct  494   AACAATAGAACGTATACCATTATTTGTTTGATCTGTTTTAATTACAGCTATTTTATTACT  553

Query  541   TTTATCTTTACCTGTATTAGCAGGTGCCATTACAATATTGTTAACTGATCGAAACTTTAA  600
             ||||||||||||||||||||||||||||||||||||||||||||||||||||||||||||
Sbjct  554   TTTATCTTTACCTGTATTAGCAGGTGCCATTACAATATTGTTAACTGATCGAAACTTTAA  613

Query  601   TACATCATTTTTTGATCCTTCAGGGGGAGGAGATCCAATTTTATATCAACATTTATTT  658
             ||||||||||||||||||||||||||||||||||||||||||||||||||||||||||
Sbjct  614   TACATCATTTTTTGATCCTTCAGGGGGAGGAGATCCAATTTTATATCAACATTTATTT  671
```

>Rhipicephalus sanguineus sensu lato 'temperate lineage' isolate CR1551 cytochrome c oxidase subunit I (COI) gene, partial cds; mitochondrial
Sequence ID: MF425997.1 Length: 695
Range 1: 22 to 679

Score:1210 bits(655), Expect:0.0,
Identities:657/658(99%), Gaps:0/658(0%), Strand: Plus/Plus

```
Query  1     AACAATATATTTAATTTTTGGAGCATGATCAGGTATATTAGGACTTAGTATAAGAATATT  60
             ||||||||||||||||||||||||||||||||||||||||||||||||||||||||||||
Sbjct  22    AACAATATATTTAATTTTTGGAGCATGATCAGGTATATTAGGACTTAGTATAAGAATATT  81

Query  61    AATTCGTATAGAATTAGGACAACCTGGAACTTTAATTGGAAATGATCAGATTTATAATGT  120
             ||||||||||||||||||||||||||||||||||||||||||||||||||||||||||||
Sbjct  82    AATTCGTATAGAATTAGGACAACCTGGAACTTTAATTGGAAATGATCAGATTTATAATGT  141

Query  121   AATCGTAACAGCACATGCATTTATTATAAttttttttATAGTTATACCAATCATAATCGG  180
             ||||||||||||||||||||||||||||||||||||||||||||||||||||||||||||
Sbjct  142   AATCGTAACAGCACATGCATTTATTATAATTTTTTTTATAGTTATACCAATCATAATCGG  201

Query  181   AGGATTTGGAAATTGATTAGTCCCTATCATATTGGGTGCCCCAGATATAGCATTTCCACG  240
             ||||||||||||||||||||||||||||||||||||||||||||||||||||||||||||
Sbjct  202   AGGATTTGGAAATTGATTAGTCCCTATCATATTGGGTGCCCCAGATATAGCATTTCCACG  261

Query  241   AATAAATAATATAAGATTTTGACTTTTACCTCCTTCACTATTTTTATTAATTAACTCTTC  300
             ||||||||||||||||||||||||||||||||||||||||||||||||||||||||||||
Sbjct  262   AATAAATAATATAAGATTTTGACTTTTACCTCCTTCACTATTTTTATTAATTAACTCTTC  321

Query  301   ATTAATTGAATCTGGAGCTGGTACAGGATGAACAGTTTACCCTCCTCTATCTTCAAATTT  360
             ||||||||||||||||||||||||||||||||||||||||||||||||||||||||||||
Sbjct  322   ATTAATTGAATCTGGAGCTGGTACAGGATGAACAGTTTACCCTCCTCTATCTTCAAATTT  381

Query  361   ATCACATTATGGCCCTTCAGTAGATTTAGCTATTTTTTCTCTTCATCTTGCTGGTGCTTC  420
             |||||||||||||||||||||||||||||||||| |||||||||||||||||||||||||
Sbjct  382   ATCACATTATGGCCCTTCAGTAGATTTAGCTATCTTTTCTCTTCATCTTGCTGGTGCTTC  441

Query  421   TTCAATTTTAGGTGCAATTAACTTTATTACAACTATTGTAAACATACGATCAATTGGAAT  480
             ||||||||||||||||||||||||||||||||||||||||||||||||||||||||||||
```

```
Sbjct  442   TTCAATTTTAGGTGCAATTAACTTTATTACAACTATTGTAAACATACGATCAATTGGAAT  501

Query  481   AACAATAGAACGTATACCATTATTTGTTTGATCTGTTTTAATTACAGCTATTTTATTACT  540
             ||||||||||||||||||||||||||||||||||||||||||||||||||||||||||||
Sbjct  502   AACAATAGAACGTATACCATTATTTGTTTGATCTGTTTTAATTACAGCTATTTTATTACT  561

Query  541   TTTATCTTTACCTGTATTAGCAGGTGCCATTACAATATTGTTAACTGATCGAAACTTTAA  600
             ||||||||||||||||||||||||||||||||||||||||||||||||||||||||||||
Sbjct  562   TTTATCTTTACCTGTATTAGCAGGTGCCATTACAATATTGTTAACTGATCGAAACTTTAA  621

Query  601   TACATCATTTTTTGATCCTTCAGGGGGAGGAGATCCAATTTTATATCAACATTTATTT  658
             ||||||||||||||||||||||||||||||||||||||||||||||||||||||||||
Sbjct  622   TACATCATTTTTTGATCCTTCAGGGGGAGGAGATCCAATTTTATATCAACATTTATTT  679
```

>Rhipicephalus sanguineus voucher CROBB813 cytochrome oxidase subunit 1 (COI) gene, partial cds; mitochondrial
Sequence ID: MZ305546.1 Length: 658
Range 1: 1 to 658

Score:1210 bits(655), Expect:0.0,
Identities:657/658(99%), Gaps:0/658(0%), Strand: Plus/Plus

```
Query  1     AACAATATATTTAATTTTTGGAGCATGATCAGGTATATTAGGACTTAGTATAAGAATATT  60
             ||||||||||||||||||||||||||||||||||||||||||||||||||||||||||||
Sbjct  1     AACAATATATTTAATTTTTGGAGCATGATCAGGTATATTAGGACTTAGTATAAGAATATT  60

Query  61    AATTCGTATAGAATTAGGACAACCTGGAACTTTAATTGGAAATGATCAGATTTATAATGT  120
             |||||||||||| |||||||||||||||||||||||||||||||||||||||||||||||
Sbjct  61    AATTCGTATAGAGTTAGGACAACCTGGAACTTTAATTGGAAATGATCAGATTTATAATGT  120

Query  121   AATCGTAACAGCACATGCATTTATTATAAttttttttATAGTTATACCAATCATAATCGG  180
             ||||||||||||||||||||||||||||||||||||||||||||||||||||||||||||
Sbjct  121   AATCGTAACAGCACATGCATTTATTATAATTTTTTTTATAGTTATACCAATCATAATCGG  180

Query  181   AGGATTTGGAAATTGATTAGTCCCTATCATATTGGGTGCCCCAGATATAGCATTTCCACG  240
             ||||||||||||||||||||||||||||||||||||||||||||||||||||||||||||
Sbjct  181   AGGATTTGGAAATTGATTAGTCCCTATCATATTGGGTGCCCCAGATATAGCATTTCCACG  240

Query  241   AATAAATAATATAAGATTTTGACTTTTACCTCCTTCACTATTTTTATTAATTAACTCTTC  300
             ||||||||||||||||||||||||||||||||||||||||||||||||||||||||||||
Sbjct  241   AATAAATAATATAAGATTTTGACTTTTACCTCCTTCACTATTTTTATTAATTAACTCTTC  300

Query  301   ATTAATTGAATCTGGAGCTGGTACAGGATGAACAGTTTACCCTCCTCTATCTTCAAATTT  360
             ||||||||||||||||||||||||||||||||||||||||||||||||||||||||||||
Sbjct  301   ATTAATTGAATCTGGAGCTGGTACAGGATGAACAGTTTACCCTCCTCTATCTTCAAATTT  360

Query  361   ATCACATTATGGCCCTTCAGTAGATTTAGCTATTTTTTCTCTTCATCTTGCTGGTGCTTC  420
             ||||||||||||||||||||||||||||||||||||||||||||||||||||||||||||
Sbjct  361   ATCACATTATGGCCCTTCAGTAGATTTAGCTATTTTTTCTCTTCATCTTGCTGGTGCTTC  420

Query  421   TTCAATTTTAGGTGCAATTAACTTTATTACAACTATTGTAAACATACGATCAATTGGAAT  480
```

```
             ||||||||||||||||||||||||||||||||||||||||||||||||||||||||||||
Sbjct  421   TTCAATTTTAGGTGCAATTAACTTTATTACAACTATTGTAAACATACGATCAATTGGAAT  480

Query  481   AACAATAGAACGTATACCATTATTTGTTTGATCTGTTTTAATTACAGCTATTTTATTACT  540
             ||||||||||||||||||||||||||||||||||||||||||||||||||||||||||||
Sbjct  481   AACAATAGAACGTATACCATTATTTGTTTGATCTGTTTTAATTACAGCTATTTTATTACT  540

Query  541   TTTATCTTTACCTGTATTAGCAGGTGCCATTACAATATTGTTAACTGATCGAAACTTTAA  600
             ||||||||||||||||||||||||||||||||||||||||||||||||||||||||||||
Sbjct  541   TTTATCTTTACCTGTATTAGCAGGTGCCATTACAATATTGTTAACTGATCGAAACTTTAA  600

Query  601   TACATCATTTTTTGATCCTTCAGGGGGAGGAGATCCAATTTTATATCAACATTTATTT  658
             ||||||||||||||||||||||||||||||||||||||||||||||||||||||||||
Sbjct  601   TACATCATTTTTTGATCCTTCAGGGGGAGGAGATCCAATTTTATATCAACATTTATTT  658
```

>Rhipicephalus sanguineus voucher CROBB810 cytochrome oxidase subunit 1 (COI) gene, partial cds; mitochondrial
Sequence ID: MZ305545.1 Length: 658
Range 1: 1 to 658

Score:1210 bits(655), Expect:0.0,
Identities:657/658(99%), Gaps:0/658(0%), Strand: Plus/Plus

```
Query  1     AACAATATATTTAATTTTTGGAGCATGATCAGGTATATTAGGACTTAGTATAAGAATATT  60
             ||||||||||||||||||||||||||||||||||||||||||||||||||||||||||||
Sbjct  1     AACAATATATTTAATTTTTGGAGCATGATCAGGTATATTAGGACTTAGTATAAGAATATT  60

Query  61    AATTCGTATAGAATTAGGACAACCTGGAACTTTAATTGGAAATGATCAGATTTATAATGT  120
             ||||||||||||||||||||||||||||||||||||||||||||||||||||||||||||
Sbjct  61    AATTCGTATAGAATTAGGACAACCTGGAACTTTAATTGGAAATGATCAGATTTATAATGT  120

Query  121   AATCGTAACAGCACATGCATTTATTATAAttttttttATAGTTATACCAATCATAATCGG  180
             ||||||||||||||||||||||||||||||||||||||||||||||||||||||||||||
Sbjct  121   AATCGTAACAGCACATGCATTTATTATAATTTTTTTTATAGTTATACCAATCATAATCGG  180

Query  181   AGGATTTGGAAATTGATTAGTCCCTATCATATTGGGTGCCCCAGATATAGCATTTCCACG  240
             ||||||||||||||||||||||||||||||||||||||||||||||||||||||||||||
Sbjct  181   AGGATTTGGAAATTGATTAGTCCCTATCATATTGGGTGCCCCAGATATAGCATTTCCACG  240

Query  241   AATAAATAATATAAGATTTTGACTTTTACCTCCTTCACTATTTTTATTAATTAACTCTTC  300
             ||||||||||||||||||||||||||||||||||||||||||||||||||||||| |||||
Sbjct  241   AATAAATAATATAAGATTTTGACTTTTACCTCCTTCACTATTTTTATTAATTAATTCTTC  300

Query  301   ATTAATTGAATCTGGAGCTGGTACAGGATGAACAGTTTACCCTCCTCTATCTTCAAATTT  360
             ||||||||||||||||||||||||||||||||||||||||||||||||||||||||||||
Sbjct  301   ATTAATTGAATCTGGAGCTGGTACAGGATGAACAGTTTACCCTCCTCTATCTTCAAATTT  360

Query  361   ATCACATTATGGCCCTTCAGTAGATTTAGCTATTTTTTCTCTTCATCTTGCTGGTGCTTC  420
             ||||||||||||||||||||||||||||||||||||||||||||||||||||||||||||
Sbjct  361   ATCACATTATGGCCCTTCAGTAGATTTAGCTATTTTTTCTCTTCATCTTGCTGGTGCTTC  420
```

```
Query  421   TTCAATTTTAGGTGCAATTAACTTTATTACAACTATTGTAAACATACGATCAATTGGAAT  480
             ||||||||||||||||||||||||||||||||||||||||||||||||||||||||||||
Sbjct  421   TTCAATTTTAGGTGCAATTAACTTTATTACAACTATTGTAAACATACGATCAATTGGAAT  480

Query  481   AACAATAGAACGTATACCATTATTTGTTTGATCTGTTTTAATTACAGCTATTTTATTACT  540
             ||||||||||||||||||||||||||||||||||||||||||||||||||||||||||||
Sbjct  481   AACAATAGAACGTATACCATTATTTGTTTGATCTGTTTTAATTACAGCTATTTTATTACT  540

Query  541   TTTATCTTTACCTGTATTAGCAGGTGCCATTACAATATTGTTAACTGATCGAAACTTTAA  600
             ||||||||||||||||||||||||||||||||||||||||||||||||||||||||||||
Sbjct  541   TTTATCTTTACCTGTATTAGCAGGTGCCATTACAATATTGTTAACTGATCGAAACTTTAA  600

Query  601   TACATCATTTTTTGATCCTTCAGGGGGAGGAGATCCAATTTTATATCAACATTTATTT  658
             ||||||||||||||||||||||||||||||||||||||||||||||||||||||||||
Sbjct  601   TACATCATTTTTTGATCCTTCAGGGGGAGGAGATCCAATTTTATATCAACATTTATTT  658
```

>Rhipicephalus sanguineus isolate 5MSK cytochrome c oxidase subunit I (COX1) gene, partial cds; mitochondrial
Sequence ID: MW152145.1 Length: 676
Range 1: 1 to 658

Score:1210 bits(655), Expect:0.0,
Identities:657/658(99%), Gaps:0/658(0%), Strand: Plus/Plus

```
Query  1     AACAATATATTTAATTTTTGGAGCATGATCAGGTATATTAGGACTTAGTATAAGAATATT  60
             ||||||||||||||||||||||||||||||||||||||||||||||||||||||||||||
Sbjct  1     AACAATATATTTAATTTTTGGAGCATGATCAGGTATATTAGGACTTAGTATAAGAATATT  60

Query  61    AATTCGTATAGAATTAGGACAACCTGGAACTTTAATTGGAAATGATCAGATTTATAATGT  120
             ||||||||||||||||||||||||||||||||||||||||||||||||||||||||||||
Sbjct  61    AATTCGTATAGAATTAGGACAACCTGGAACTTTAATTGGAAATGATCAGATTTATAATGT  120

Query  121   AATCGTAACAGCACATGCATTTATTATAAttttttttATAGTTATACCAATCATAATCGG  180
             ||||||||||||||||||||||||||||||||||||||||||||||||||||||||||||
Sbjct  121   AATCGTAACAGCACATGCATTTATTATAATTTTTTTTATAGTTATACCAATCATAATCGG  180

Query  181   AGGATTTGGAAATTGATTAGTCCCTATCATATTGGGTGCCCCAGATATAGCATTTCCACG  240
             ||||||||||||||||||||||||||||||||||||||||||||||||||||||||||||
Sbjct  181   AGGATTTGGAAATTGATTAGTCCCTATCATATTGGGTGCCCCAGATATAGCATTTCCACG  240

Query  241   AATAAATAATATAAGATTTTGACTTTTACCTCCTTCACTATTTTTATTAATTAACTCTTC  300
             |||||||||||||||||||||||||||||||||||||||||||||||||||||| |||||
Sbjct  241   AATAAATAATATAAGATTTTGACTTTTACCTCCTTCACTATTTTTATTAATTAATTCTTC  300

Query  301   ATTAATTGAATCTGGAGCTGGTACAGGATGAACAGTTTACCCTCCTCTATCTTCAAATTT  360
             ||||||||||||||||||||||||||||||||||||||||||||||||||||||||||||
Sbjct  301   ATTAATTGAATCTGGAGCTGGTACAGGATGAACAGTTTACCCTCCTCTATCTTCAAATTT  360

Query  361   ATCACATTATGGCCCTTCAGTAGATTTAGCTATTTTTTCTCTTCATCTTGCTGGTGCTTC  420
             ||||||||||||||||||||||||||||||||||||||||||||||||||||||||||||
Sbjct  361   ATCACATTATGGCCCTTCAGTAGATTTAGCTATTTTTTCTCTTCATCTTGCTGGTGCTTC  420
```

```
Query  421   TTCAATTTTAGGTGCAATTAACTTTATTACAACTATTGTAAACATACGATCAATTGGAAT  480
             ||||||||||||||||||||||||||||||||||||||||||||||||||||||||||||
Sbjct  421   TTCAATTTTAGGTGCAATTAACTTTATTACAACTATTGTAAACATACGATCAATTGGAAT  480

Query  481   AACAATAGAACGTATACCATTATTTGTTTGATCTGTTTTAATTACAGCTATTTTATTACT  540
             ||||||||||||||||||||||||||||||||||||||||||||||||||||||||||||
Sbjct  481   AACAATAGAACGTATACCATTATTTGTTTGATCTGTTTTAATTACAGCTATTTTATTACT  540

Query  541   TTTATCTTTACCTGTATTAGCAGGTGCCATTACAATATTGTTAACTGATCGAAACTTTAA  600
             ||||||||||||||||||||||||||||||||||||||||||||||||||||||||||||
Sbjct  541   TTTATCTTTACCTGTATTAGCAGGTGCCATTACAATATTGTTAACTGATCGAAACTTTAA  600

Query  601   TACATCATTTTTTGATCCTTCAGGGGGAGGAGATCCAATTTTATATCAACATTTATTT  658
             ||||||||||||||||||||||||||||||||||||||||||||||||||||||||||
Sbjct  601   TACATCATTTTTTGATCCTTCAGGGGGAGGAGATCCAATTTTATATCAACATTTATTT  658
```

>Rhipicephalus sanguineus isolate 1MSK cytochrome c oxidase subunit I (COX1) gene, partial cds; mitochondrial
Sequence ID: MW152144.1 Length: 675
Range 1: 1 to 658

Score:1210 bits(655), Expect:0.0,
Identities:657/658(99%), Gaps:0/658(0%), Strand: Plus/Plus

```
Query  1     AACAATATATTTAATTTTTGGAGCATGATCAGGTATATTAGGACTTAGTATAAGAATATT  60
             ||||||||||||||||||||||||||||||||||||||||||||||||||||||||||||
Sbjct  1     AACAATATATTTAATTTTTGGAGCATGATCAGGTATATTAGGACTTAGTATAAGAATATT  60

Query  61    AATTCGTATAGAATTAGGACAACCTGGAACTTTAATTGGAAATGATCAGATTTATAATGT  120
             ||||||||||||||||||||||||||||||||||||||||||||||||||||||||||||
Sbjct  61    AATTCGTATAGAATTAGGACAACCTGGAACTTTAATTGGAAATGATCAGATTTATAATGT  120

Query  121   AATCGTAACAGCACATGCATTTATTATAAttttttttATAGTTATACCAATCATAATCGG  180
             ||||||||||||||||||||||||||||||||||||||||||||||||||||||||||||
Sbjct  121   AATCGTAACAGCACATGCATTTATTATAATTTTTTTTATAGTTATACCAATCATAATCGG  180

Query  181   AGGATTTGGAAATTGATTAGTCCCTATCATATTGGGTGCCCCAGATATAGCATTTCCACG  240
             ||||||||||||||||||||||||||||||||||||||||||||||||||||||||||||
Sbjct  181   AGGATTTGGAAATTGATTAGTCCCTATCATATTGGGTGCCCCAGATATAGCATTTCCACG  240

Query  241   AATAAATAATATAAGATTTTGACTTTTACCTCCTTCACTATTTTTATTAATTAACTCTTC  300
             |||||||||||||||||||||||||||||||||||||||||||||||||||||| |||||
Sbjct  241   AATAAATAATATAAGATTTTGACTTTTACCTCCTTCACTATTTTTATTAATTAATTCTTC  300

Query  301   ATTAATTGAATCTGGAGCTGGTACAGGATGAACAGTTTACCCTCCTCTATCTTCAAATTT  360
             ||||||||||||||||||||||||||||||||||||||||||||||||||||||||||||
Sbjct  301   ATTAATTGAATCTGGAGCTGGTACAGGATGAACAGTTTACCCTCCTCTATCTTCAAATTT  360

Query  361   ATCACATTATGGCCCTTCAGTAGATTTAGCTATTTTTTCTCTTCATCTTGCTGGTGCTTC  420
             ||||||||||||||||||||||||||||||||||||||||||||||||||||||||||||
```

```
Sbjct  361   ATCACATTATGGCCCTTCAGTAGATTTAGCTATTTTTTCTCTTCATCTTGCTGGTGCTTC  420

Query  421   TTCAATTTTAGGTGCAATTAACTTTATTACAACTATTGTAAACATACGATCAATTGGAAT  480
             ||||||||||||||||||||||||||||||||||||||||||||||||||||||||||||
Sbjct  421   TTCAATTTTAGGTGCAATTAACTTTATTACAACTATTGTAAACATACGATCAATTGGAAT  480

Query  481   AACAATAGAACGTATACCATTATTTGTTTGATCTGTTTTAATTACAGCTATTTTATTACT  540
             ||||||||||||||||||||||||||||||||||||||||||||||||||||||||||||
Sbjct  481   AACAATAGAACGTATACCATTATTTGTTTGATCTGTTTTAATTACAGCTATTTTATTACT  540

Query  541   TTTATCTTTACCTGTATTAGCAGGTGCCATTACAATATTGTTAACTGATCGAAACTTTAA  600
             ||||||||||||||||||||||||||||||||||||||||||||||||||||||||||||
Sbjct  541   TTTATCTTTACCTGTATTAGCAGGTGCCATTACAATATTGTTAACTGATCGAAACTTTAA  600

Query  601   TACATCATTTTTTGATCCTTCAGGGGGAGGAGATCCAATTTTATATCAACATTTATTT  658
             ||||||||||||||||||||||||||||||||||||||||||||||||||||||||||
Sbjct  601   TACATCATTTTTTGATCCTTCAGGGGGAGGAGATCCAATTTTATATCAACATTTATTT  658
```

>Rhipicephalus sanguineus isolate RSN2SK cytochrome c oxidase subunit I (COX1) gene, partial cds; mitochondrial
Sequence ID: MW152143.1 Length: 676
Range 1: 1 to 658

Score:1210 bits(655), Expect:0.0,
Identities:657/658(99%), Gaps:0/658(0%), Strand: Plus/Plus

```
Query  1     AACAATATATTTAATTTTTGGAGCATGATCAGGTATATTAGGACTTAGTATAAGAATATT  60
             ||||||||||||||||||||||||||||||||||||||||||||||||||||||||||||
Sbjct  1     AACAATATATTTAATTTTTGGAGCATGATCAGGTATATTAGGACTTAGTATAAGAATATT  60

Query  61    AATTCGTATAGAATTAGGACAACCTGGAACTTTAATTGGAAATGATCAGATTTATAATGT  120
             ||||||||||||||||||||||||||||||||||||||||||||||||||||||||||||
Sbjct  61    AATTCGTATAGAATTAGGACAACCTGGAACTTTAATTGGAAATGATCAGATTTATAATGT  120

Query  121   AATCGTAACAGCACATGCATTTATTATAAtttttttATAGTTATACCAATCATAATCGG  180
             ||||||||||||||||||||||||||||||||||||||||||||||||||||||||||||
Sbjct  121   AATCGTAACAGCACATGCATTTATTATAATTTTTTTATAGTTATACCAATCATAATCGG  180

Query  181   AGGATTTGGAAATTGATTAGTCCCTATCATATTGGGTGCCCCAGATATAGCATTTCCACG  240
             ||||||||||||||||||||||||||||||||||||||||||||||||||||||||||||
Sbjct  181   AGGATTTGGAAATTGATTAGTCCCTATCATATTGGGTGCCCCAGATATAGCATTTCCACG  240

Query  241   AATAAATAATATAAGATTTTGACTTTTACCTCCTTCACTATTTTTATTAATTAACTCTTC  300
             |||||||||||||||||||||||||||||||||||||||||||||||||||||| |||||
Sbjct  241   AATAAATAATATAAGATTTTGACTTTTACCTCCTTCACTATTTTTATTAATTAATTCTTC  300

Query  301   ATTAATTGAATCTGGAGCTGGTACAGGATGAACAGTTTACCCTCCTCTATCTTCAAATTT  360
             ||||||||||||||||||||||||||||||||||||||||||||||||||||||||||||
Sbjct  301   ATTAATTGAATCTGGAGCTGGTACAGGATGAACAGTTTACCCTCCTCTATCTTCAAATTT  360

Query  361   ATCACATTATGGCCCTTCAGTAGATTTAGCTATTTTTTCTCTTCATCTTGCTGGTGCTTC  420
```

```
                ||||||||||||||||||||||||||||||||||||||||||||||||||||||||||||
Sbjct  361   ATCACATTATGGCCCTTCAGTAGATTTAGCTATTTTTTCTCTTCATCTTGCTGGTGCTTC  420

Query  421   TTCAATTTTAGGTGCAATTAACTTTATTACAACTATTGTAAACATACGATCAATTGGAAT  480
             ||||||||||||||||||||||||||||||||||||||||||||||||||||||||||||
Sbjct  421   TTCAATTTTAGGTGCAATTAACTTTATTACAACTATTGTAAACATACGATCAATTGGAAT  480

Query  481   AACAATAGAACGTATACCATTATTTGTTTGATCTGTTTTAATTACAGCTATTTTATTACT  540
             ||||||||||||||||||||||||||||||||||||||||||||||||||||||||||||
Sbjct  481   AACAATAGAACGTATACCATTATTTGTTTGATCTGTTTTAATTACAGCTATTTTATTACT  540

Query  541   TTTATCTTTACCTGTATTAGCAGGTGCCATTACAATATTGTTAACTGATCGAAACTTTAA  600
             ||||||||||||||||||||||||||||||||||||||||||||||||||||||||||||
Sbjct  541   TTTATCTTTACCTGTATTAGCAGGTGCCATTACAATATTGTTAACTGATCGAAACTTTAA  600

Query  601   TACATCATTTTTTGATCCTTCAGGGGGAGGAGATCCAATTTTATATCAACATTTATTT  658
             ||||||||||||||||||||||||||||||||||||||||||||||||||||||||||
Sbjct  601   TACATCATTTTTTGATCCTTCAGGGGGAGGAGATCCAATTTTATATCAACATTTATTT  658
```

>Rhipicephalus sanguineus isolate RSN11SK cytochrome c oxidase subunit I (COX1) gene, partial cds; mitochondrial
Sequence ID: MW152142.1 Length: 669
Range 1: 1 to 658

Score:1210 bits(655), Expect:0.0,
Identities:657/658(99%), Gaps:0/658(0%), Strand: Plus/Plus

```
Query  1     AACAATATATTTAATTTTTGGAGCATGATCAGGTATATTAGGACTTAGTATAAGAATATT  60
             ||||||||||||||||||||||||||||||||||||||||||||||||||||||||||||
Sbjct  1     AACAATATATTTAATTTTTGGAGCATGATCAGGTATATTAGGACTTAGTATAAGAATATT  60

Query  61    AATTCGTATAGAATTAGGACAACCTGGAACTTTAATTGGAAATGATCAGATTTATAATGT  120
             ||||||||||||||||||||||||||||||||||||||||||||||||||||||||||||
Sbjct  61    AATTCGTATAGAATTAGGACAACCTGGAACTTTAATTGGAAATGATCAGATTTATAATGT  120

Query  121   AATCGTAACAGCACATGCATTTATTATAAttttttttATAGTTATACCAATCATAATCGG  180
             ||||||||||||||||||||||||||||||||||||||||||||||||||||||||||||
Sbjct  121   AATCGTAACAGCACATGCATTTATTATAATTTTTTTTATAGTTATACCAATCATAATCGG  180

Query  181   AGGATTTGGAAATTGATTAGTCCCTATCATATTGGGTGCCCCAGATATAGCATTTCCACG  240
             ||||||||||||||||||||||||||||||||||||||||||||||||||||||||||||
Sbjct  181   AGGATTTGGAAATTGATTAGTCCCTATCATATTGGGTGCCCCAGATATAGCATTTCCACG  240

Query  241   AATAAATAATATAAGATTTTGACTTTTACCTCCTTCACTATTTTTATTAATTAACTCTTC  300
             |||||||||||||||||||||||||||||||||||||||||||||||||||||| |||||
Sbjct  241   AATAAATAATATAAGATTTTGACTTTTACCTCCTTCACTATTTTTATTAATTAATTCTTC  300

Query  301   ATTAATTGAATCTGGAGCTGGTACAGGATGAACAGTTTACCCTCCTCTATCTTCAAATTT  360
             ||||||||||||||||||||||||||||||||||||||||||||||||||||||||||||
Sbjct  301   ATTAATTGAATCTGGAGCTGGTACAGGATGAACAGTTTACCCTCCTCTATCTTCAAATTT  360
```

```
Query  361   ATCACATTATGGCCCTTCAGTAGATTTAGCTATTTTTTCTCTTCATCTTGCTGGTGCTTC  420
             ||||||||||||||||||||||||||||||||||||||||||||||||||||||||||||
Sbjct  361   ATCACATTATGGCCCTTCAGTAGATTTAGCTATTTTTTCTCTTCATCTTGCTGGTGCTTC  420

Query  421   TTCAATTTTAGGTGCAATTAACTTTATTACAACTATTGTAAACATACGATCAATTGGAAT  480
             ||||||||||||||||||||||||||||||||||||||||||||||||||||||||||||
Sbjct  421   TTCAATTTTAGGTGCAATTAACTTTATTACAACTATTGTAAACATACGATCAATTGGAAT  480

Query  481   AACAATAGAACGTATACCATTATTTGTTTGATCTGTTTTAATTACAGCTATTTTATTACT  540
             ||||||||||||||||||||||||||||||||||||||||||||||||||||||||||||
Sbjct  481   AACAATAGAACGTATACCATTATTTGTTTGATCTGTTTTAATTACAGCTATTTTATTACT  540

Query  541   TTTATCTTTACCTGTATTAGCAGGTGCCATTACAATATTGTTAACTGATCGAAACTTTAA  600
             ||||||||||||||||||||||||||||||||||||||||||||||||||||||||||||
Sbjct  541   TTTATCTTTACCTGTATTAGCAGGTGCCATTACAATATTGTTAACTGATCGAAACTTTAA  600

Query  601   TACATCATTTTTTGATCCTTCAGGGGGAGGAGATCCAATTTTATATCAACATTTATTT  658
             ||||||||||||||||||||||||||||||||||||||||||||||||||||||||||
Sbjct  601   TACATCATTTTTTGATCCTTCAGGGGGAGGAGATCCAATTTTATATCAACATTTATTT  658
```

>Rhipicephalus sanguineus sensu lato 'temperate lineage' isolate S48 cytochrome c oxidase subunit I (COI) gene, partial cds; mitochondrial
Sequence ID: MF426015.1 Length: 692
Range 1: 19 to 676

Score:1205 bits(652), Expect:0.0,
Identities:656/658(99%), Gaps:0/658(0%), Strand: Plus/Plus

```
Query  1     AACAATATATTTAATTTTTGGAGCATGATCAGGTATATTAGGACTTAGTATAAGAATATT  60
             ||||||||||||||||||||||||||||||||||||||||||||||||||||||||||||
Sbjct  19    AACAATATATTTAATTTTTGGAGCATGATCAGGTATATTAGGACTTAGTATAAGAATATT  78

Query  61    AATTCGTATAGAATTAGGACAACCTGGAACTTTAATTGGAAATGATCAGATTTATAATGT  120
             ||||||||||||||||||||||||||||||||||||||||||||||||||||||||||||
Sbjct  79    AATTCGTATAGAATTAGGACAACCTGGAACTTTAATTGGAAATGATCAGATTTATAATGT  138

Query  121   AATCGTAACAGCACATGCATTTATTATAAttttttttATAGTTATACCAATCATAATCGG  180
             ||||||||||||||||||||||||||||||||||||||||||||||||||||||||||||
Sbjct  139   AATCGTAACAGCACATGCATTTATTATAATTTTTTTTATAGTTATACCAATCATAATCGG  198

Query  181   AGGATTTGGAAATTGATTAGTCCCTATCATATTGGGTGCCCCAGATATAGCATTTCCACG  240
             ||||||||||||||||||||||||||||||||||||||||||||||||||||||||||||
Sbjct  199   AGGATTTGGAAATTGATTAGTCCCTATCATATTGGGTGCCCCAGATATAGCATTTCCACG  258

Query  241   AATAAATAATATAAGATTTTGACTTTTACCTCCTTCACTATTTTTATTAATTAACTCTTC  300
             ||||||||||||||||||||||||||||||||||||||||||||||||||||||||||||
Sbjct  259   AATAAATAATATAAGATTTTGACTTTTACCTCCTTCACTATTTTTATTAATTAACTCTTC  318

Query  301   ATTAATTGAATCTGGAGCTGGTACAGGATGAACAGTTTACCCTCCTCTATCTTCAAATTT  360
             ||||||||||||||||||||||||||||||||||||||||||||||||||||||||||||
Sbjct  319   ATTAATTGAATCTGGAGCTGGTACAGGATGAACAGTTTACCCTCCTCTATCTTCAAATTT  378
```

```
Query  361   ATCACATTATGGCCCTTCAGTAGATTTAGCTATTTTTTCTCTTCATCTTGCTGGTGCTTC  420
             |||||||||||| |||||||||||||||||||||||||||||||||||||||||||||||
Sbjct  379   ATCACATTATGGTCCTTCAGTAGATTTAGCTATTTTTTCTCTTCATCTTGCTGGTGCTTC  438

Query  421   TTCAATTTTAGGTGCAATTAACTTTATTACAACTATTGTAAACATACGATCAATTGGAAT  480
             ||||||||||||||||||||||||||||||||||||||||||||||||||||||||||||
Sbjct  439   TTCAATTTTAGGTGCAATTAACTTTATTACAACTATTGTAAACATACGATCAATTGGAAT  498

Query  481   AACAATAGAACGTATACCATTATTTGTTTGATCTGTTTTAATTACAGCTATTTTATTACT  540
             ||||||||||||||||||||||||||||||||||||||||||||||||||||||| ||||
Sbjct  499   AACAATAGAACGTATACCATTATTTGTTTGATCTGTTTTAATTACAGCTATTTTACTACT  558

Query  541   TTTATCTTTACCTGTATTAGCAGGTGCCATTACAATATTGTTAACTGATCGAAACTTTAA  600
             ||||||||||||||||||||||||||||||||||||||||||||||||||||||||||||
Sbjct  559   TTTATCTTTACCTGTATTAGCAGGTGCCATTACAATATTGTTAACTGATCGAAACTTTAA  618

Query  601   TACATCATTTTTTGATCCTTCAGGGGGAGGAGATCCAATTTTATATCAACATTTATTT  658
             ||||||||||||||||||||||||||||||||||||||||||||||||||||||||||
Sbjct  619   TACATCATTTTTTGATCCTTCAGGGGGAGGAGATCCAATTTTATATCAACATTTATTT  676
```

```
>Rhipicephalus sanguineus sensu lato 'temperate lineage' isolate S1060
cytochrome c oxidase subunit I (COI) gene, partial cds; mitochondrial
Sequence ID: MF426006.1 Length: 705
Range 1: 28 to 685

Score:1205 bits(652), Expect:0.0,
Identities:656/658(99%),  Gaps:0/658(0%), Strand: Plus/Plus

Query  1     AACAATATATTTAATTTTTGGAGCATGATCAGGTATATTAGGACTTAGTATAAGAATATT  60
             ||||||||||||||||||||||||||||||||||||||||||||||||||||||||||||
Sbjct  28    AACAATATATTTAATTTTTGGAGCATGATCAGGTATATTAGGACTTAGTATAAGAATATT  87

Query  61    AATTCGTATAGAATTAGGACAACCTGGAACTTTAATTGGAAATGATCAGATTTATAATGT  120
             ||||||||||||||||||||||||||||||||||||||||||||||||||||||||||||
Sbjct  88    AATTCGTATAGAATTAGGACAACCTGGAACTTTAATTGGAAATGATCAGATTTATAATGT  147

Query  121   AATCGTAACAGCACATGCATTTATTATAAttttttttATAGTTATACCAATCATAATCGG  180
             ||||||||||||||||||||||||||||||||||||||||||||||||||||||||||||
Sbjct  148   AATCGTAACAGCACATGCATTTATTATAATTTTTTTTATAGTTATACCAATCATAATCGG  207

Query  181   AGGATTTGGAAATTGATTAGTCCCTATCATATTGGGTGCCCCAGATATAGCATTTCCACG  240
             ||||||||||||||||||||||||||||||||||||||||||||||||||||||||||||
Sbjct  208   AGGATTTGGAAATTGATTAGTCCCTATCATATTGGGTGCCCCAGATATAGCATTTCCACG  267

Query  241   AATAAATAATATAAGATTTTGACTTTTACCTCCTTCACTATTTTTATTAATTAACTCTTC  300
             ||||||||||||||||||||||||||||||||||||||||||||||||||||||||||||
Sbjct  268   AATAAATAATATAAGATTTTGACTTTTACCTCCTTCACTATTTTTATTAATTAACTCTTC  327

Query  301   ATTAATTGAATCTGGAGCTGGTACAGGATGAACAGTTTACCCTCCTCTATCTTCAAATTT  360
             ||||||||||||||||||||||||||||||||||||||||||||||||||||||||||||
```

```
Sbjct  328   ATTAATTGAATCTGGAGCTGGTACAGGATGAACAGTTTACCCTCCTCTATCTTCAAATTT  387

Query  361   ATCACATTATGGCCCTTCAGTAGATTTAGCTATTTTTTCTCTTCATCTTGCTGGTGCTTC  420
             |||||||||||| |||||||||||||||||||||||||||||||||||||||||||||||
Sbjct  388   ATCACATTATGGTCCTTCAGTAGATTTAGCTATTTTTTCTCTTCATCTTGCTGGTGCTTC  447

Query  421   TTCAATTTTAGGTGCAATTAACTTTATTACAACTATTGTAAACATACGATCAATTGGAAT  480
             |||||||||||||||||||||||||||||||||||||||||||||||||| |||||||||
Sbjct  448   TTCAATTTTAGGTGCAATTAACTTTATTACAACTATTGTAAACATACGATTAATTGGAAT  507

Query  481   AACAATAGAACGTATACCATTATTTGTTTGATCTGTTTTAATTACAGCTATTTTATTACT  540
             ||||||||||||||||||||||||||||||||||||||||||||||||||||||||||||
Sbjct  508   AACAATAGAACGTATACCATTATTTGTTTGATCTGTTTTAATTACAGCTATTTTATTACT  567

Query  541   TTTATCTTTACCTGTATTAGCAGGTGCCATTACAATATTGTTAACTGATCGAAACTTTAA  600
             ||||||||||||||||||||||||||||||||||||||||||||||||||||||||||||
Sbjct  568   TTTATCTTTACCTGTATTAGCAGGTGCCATTACAATATTGTTAACTGATCGAAACTTTAA  627

Query  601   TACATCATTTTTTGATCCTTCAGGGGGAGGAGATCCAATTTTATATCAACATTTATTT  658
             ||||||||||||||||||||||||||||||||||||||||||||||||||||||||||
Sbjct  628   TACATCATTTTTTGATCCTTCAGGGGGAGGAGATCCAATTTTATATCAACATTTATTT  685
```

>Rhipicephalus sanguineus sensu lato 'temperate lineage' isolate S56 cytochrome c oxidase subunit I (COI) gene, partial cds; mitochondrial
Sequence ID: MF426017.1 Length: 680
Range 1: 11 to 668

Score:1199 bits(649), Expect:0.0,
Identities:655/658(99%), Gaps:0/658(0%), Strand: Plus/Plus

```
Query  1     AACAATATATTTAATTTTTGGAGCATGATCAGGTATATTAGGACTTAGTATAAGAATATT  60
             ||||||||||||||||||||||||||||||||||||||||||||||||||||||||||||
Sbjct  11    AACAATATATTTAATTTTTGGAGCATGATCAGGTATATTAGGACTTAGTATAAGAATATT  70

Query  61    AATTCGTATAGAATTAGGACAACCTGGAACTTTAATTGGAAATGATCAGATTTATAATGT  120
             ||||||||||||||||||||||||||||||||||||||||||||||||||||||||||||
Sbjct  71    AATTCGTATAGAATTAGGACAACCTGGAACTTTAATTGGAAATGATCAGATTTATAATGT  130

Query  121   AATCGTAACAGCACATGCATTTATTATAAttttttttATAGTTATACCAATCATAATCGG  180
             ||||||||||||||||||||||||||||||||||||||||||||||||||||||||||||
Sbjct  131   AATCGTAACAGCACATGCATTTATTATAATTTTTTTTATAGTTATACCAATCATAATCGG  190

Query  181   AGGATTTGGAAATTGATTAGTCCCTATCATATTGGGTGCCCCAGATATAGCATTTCCACG  240
             ||||||||||||||||||||||||||||||||||||||||||||||||||||||||||||
Sbjct  191   AGGATTTGGAAATTGATTAGTCCCTATCATATTGGGTGCCCCAGATATAGCATTTCCACG  250

Query  241   AATAAATAATATAAGATTTTGACTTTTACCTCCTTCACTATTTTTATTAATTAACTCTTC  300
             ||||||||||||||||||||||||||||||||||||||||||||||||||||||||||||
Sbjct  251   AATAAATAATATAAGATTTTGACTTTTACCTCCTTCACTATTTTTATTAATTAACTCTTC  310

Query  301   ATTAATTGAATCTGGAGCTGGTACAGGATGAACAGTTTACCCTCCTCTATCTTCAAATTT  360
```

```
                ||||||||||||||||||||||||||||||||||||||| ||||||||||||||||||||
Sbjct  311   ATTAATTGAATCTGGAGCTGGTACAGGATGAACAGTTTATCCTCCTCTATCTTCAAATTT  370

Query  361   ATCACATTATGGCCCTTCAGTAGATTTAGCTATTTTTTCTCTTCATCTTGCTGGTGCTTC  420
             ||||||||||||||||||||||||||||||||| ||||||||||||||||||||||||||
Sbjct  371   ATCACATTATGGCCCTTCAGTAGATTTAGCTATCTTTTCTCTTCATCTTGCTGGTGCTTC  430

Query  421   TTCAATTTTAGGTGCAATTAACTTTATTACAACTATTGTAAACATACGATCAATTGGAAT  480
             |||||||||||| |||||||||||||||||||||||||||||||||||||||||||||||
Sbjct  431   TTCAATTTTAGGAGCAATTAACTTTATTACAACTATTGTAAACATACGATCAATTGGAAT  490

Query  481   AACAATAGAACGTATACCATTATTTGTTTGATCTGTTTTAATTACAGCTATTTTATTACT  540
             ||||||||||||||||||||||||||||||||||||||||||||||||||||||||||||
Sbjct  491   AACAATAGAACGTATACCATTATTTGTTTGATCTGTTTTAATTACAGCTATTTTATTACT  550

Query  541   TTTATCTTTACCTGTATTAGCAGGTGCCATTACAATATTGTTAACTGATCGAAACTTTAA  600
             ||||||||||||||||||||||||||||||||||||||||||||||||||||||||||||
Sbjct  551   TTTATCTTTACCTGTATTAGCAGGTGCCATTACAATATTGTTAACTGATCGAAACTTTAA  610

Query  601   TACATCATTTTTTGATCCTTCAGGGGGAGGAGATCCAATTTTATATCAACATTTATTT  658
             ||||||||||||||||||||||||||||||||||||||||||||||||||||||||||
Sbjct  611   TACATCATTTTTTGATCCTTCAGGGGGAGGAGATCCAATTTTATATCAACATTTATTT  668
```

>Rhipicephalus sanguineus sensu lato 'temperate lineage' isolate CR1553
cytochrome c oxidase subunit I (COI) gene, partial cds; mitochondrial
Sequence ID: MF425998.1 Length: 689
Range 1: 20 to 677

Score:1199 bits(649), Expect:0.0,
Identities:655/658(99%), Gaps:0/658(0%), Strand: Plus/Plus

```
Query  1     AACAATATATTTAATTTTTGGAGCATGATCAGGTATATTAGGACTTAGTATAAGAATATT  60
             ||||||||||||||||||||||||||||||||||||||||||||||||||||||||||||
Sbjct  20    AACAATATATTTAATTTTTGGAGCATGATCAGGTATATTAGGACTTAGTATAAGAATATT  79

Query  61    AATTCGTATAGAATTAGGACAACCTGGAACTTTAATTGGAAATGATCAGATTTATAATGT  120
             ||||||||||||||||||||||||||||||||||||||||||||||||||||||||||||
Sbjct  80    AATTCGTATAGAATTAGGACAACCTGGAACTTTAATTGGAAATGATCAGATTTATAATGT  139

Query  121   AATCGTAACAGCACATGCATTTATTATAAttttttttATAGTTATACCAATCATAATCGG  180
             ||||||||||||||||||||||||||||||||||||||||||||||||||||||||||||
Sbjct  140   AATCGTAACAGCACATGCATTTATTATAATTTTTTTTATAGTTATACCAATCATAATCGG  199

Query  181   AGGATTTGGAAATTGATTAGTCCCTATCATATTGGGTGCCCCAGATATAGCATTTCCACG  240
             ||||||||||||||||||||||||||||||||| ||||||||||||||||||||||||||
Sbjct  200   AGGATTTGGAAATTGATTAGTCCCTATCATATTAGGTGCCCCAGATATAGCATTTCCACG  259

Query  241   AATAAATAATATAAGATTTTGACTTTTACCTCCTTCACTATTTTTATTAATTAACTCTTC  300
             ||||||||||||||||||||||||||||||||||||||||||||||||||||||||||||
Sbjct  260   AATAAATAATATAAGATTTTGACTTTTACCTCCTTCACTATTTTTATTAATTAACTCTTC  319
```

```
Query  301  ATTAATTGAATCTGGAGCTGGTACAGGATGAACAGTTTACCCTCCTCTATCTTCAAATTT  360
            ||||||||||||||||||||||||||||||||||||||||||||||||||||||||||||
Sbjct  320  ATTAATTGAATCTGGAGCTGGTACAGGATGAACAGTTTACCCTCCTCTATCTTCAAATTT  379

Query  361  ATCACATTATGGCCCTTCAGTAGATTTAGCTATTTTTTCTCTTCATCTTGCTGGTGCTTC  420
            ||||||||||||||||||||||||||||||||| ||||||||||||||||||||||||||
Sbjct  380  ATCACATTATGGCCCTTCAGTAGATTTAGCTATCTTTTCTCTTCATCTTGCTGGTGCTTC  439

Query  421  TTCAATTTTAGGTGCAATTAACTTTATTACAACTATTGTAAACATACGATCAATTGGAAT  480
            ||||||||||||||||||||||||||||||||||||||||||||||||||||||||||||
Sbjct  440  TTCAATTTTAGGTGCAATTAACTTTATTACAACTATTGTAAACATACGATCAATTGGAAT  499

Query  481  AACAATAGAACGTATACCATTATTTGTTTGATCTGTTTTAATTACAGCTATTTTATTACT  540
            ||||||||||||||||||||||||||||||||||||||||||||||||||||||||||||
Sbjct  500  AACAATAGAACGTATACCATTATTTGTTTGATCTGTTTTAATTACAGCTATTTTATTACT  559

Query  541  TTTATCTTTACCTGTATTAGCAGGTGCCATTACAATATTGTTAACTGATCGAAACTTTAA  600
             |||||||||||||||||||||||||||||||||||||||||||||||||||||||||||
Sbjct  560  CTTATCTTTACCTGTATTAGCAGGTGCCATTACAATATTGTTAACTGATCGAAACTTTAA  619

Query  601  TACATCATTTTTTGATCCTTCAGGGGGAGGAGATCCAATTTTATATCAACATTTATTT  658
            ||||||||||||||||||||||||||||||||||||||||||||||||||||||||||
Sbjct  620  TACATCATTTTTTGATCCTTCAGGGGGAGGAGATCCAATTTTATATCAACATTTATTT  677
```

>Rhipicephalus sanguineus haplotype IV cytochrome oxidase subunit I (COI) gene, partial cds; mitochondrial
Sequence ID: KU556746.1 Length: 771
Range 1: 90 to 747

Score:1199 bits(649), Expect:0.0,
Identities:655/658(99%), Gaps:0/658(0%), Strand: Plus/Plus

```
Query  1    AACAATATATTTAATTTTTGGAGCATGATCAGGTATATTAGGACTTAGTATAAGAATATT  60
            ||||||||||||||||||||||||||||||||||||||||||||||||||||||||||||
Sbjct  90   AACAATATATTTAATTTTTGGAGCATGATCAGGTATATTAGGACTTAGTATAAGAATATT  149

Query  61   AATTCGTATAGAATTAGGACAACCTGGAACTTTAATTGGAAATGATCAGATTTATAATGT  120
            ||||||||||||||||||||||||||||||||||||||||||||||||||||||||||||
Sbjct  150  AATTCGTATAGAATTAGGACAACCTGGAACTTTAATTGGAAATGATCAGATTTATAATGT  209

Query  121  AATCGTAACAGCACATGCATTTATTATAAttttttttATAGTTATACCAATCATAATCGG  180
            ||||||||||||||||||||||||||||||||||||||||||||||||||||||||||||
Sbjct  210  AATCGTAACAGCACATGCATTTATTATAATTTTTTTTATAGTTATACCAATCATAATCGG  269

Query  181  AGGATTTGGAAATTGATTAGTCCCTATCATATTGGGTGCCCCAGATATAGCATTTCCACG  240
            ||||||||||||||||||||||||||||||||||||||||||||||||||||||||||||
Sbjct  270  AGGATTTGGAAATTGATTAGTCCCTATCATATTGGGTGCCCCAGATATAGCATTTCCACG  329

Query  241  AATAAATAATATAAGATTTTGACTTTTACCTCCTTCACTATTTTTATTAATTAACTCTTC  300
            ||||||||||||||||||||||||||||||||||||||||||||||||||||||||||||
Sbjct  330  AATAAATAATATAAGATTTTGACTTTTACCTCCTTCACTATTTTTATTAATTAACTCTTC  389
```

```
Query  301  ATTAATTGAATCTGGAGCTGGTACAGGATGAACAGTTTACCCTCCTCTATCTTCAAATTT  360
            ||||||||||||||||||||||||||||||||||||||| ||||||||||||||||||||
Sbjct  390  ATTAATTGAATCTGGAGCTGGTACAGGATGAACAGTTTATCCTCCTCTATCTTCAAATTT  449

Query  361  ATCACATTATGGCCCTTCAGTAGATTTAGCTATTTTTTCTCTTCATCTTGCTGGTGCTTC  420
            ||||||||||||||||||||||||||||||||| ||||||||||||||||||||||||||
Sbjct  450  ATCACATTATGGCCCTTCAGTAGATTTAGCTATCTTTTCTCTTCATCTTGCTGGTGCTTC  509

Query  421  TTCAATTTAGGTGCAATTAACTTTATTACAACTATTGTAAACATACGATCAATTGGAAT  480
            ||||||||||| ||||||||||||||||||||||||||||||||||||||||||||||||
Sbjct  510  TTCAATTTAGGAGCAATTAACTTTATTACAACTATTGTAAACATACGATCAATTGGAAT  569

Query  481  AACAATAGAACGTATACCATTATTTGTTTGATCTGTTTTAATTACAGCTATTTTATTACT  540
            ||||||||||||||||||||||||||||||||||||||||||||||||||||||||||||
Sbjct  570  AACAATAGAACGTATACCATTATTTGTTTGATCTGTTTTAATTACAGCTATTTTATTACT  629

Query  541  TTTATCTTTACCTGTATTAGCAGGTGCCATTACAATATTGTTAACTGATCGAAACTTTAA  600
            ||||||||||||||||||||||||||||||||||||||||||||||||||||||||||||
Sbjct  630  TTTATCTTTACCTGTATTAGCAGGTGCCATTACAATATTGTTAACTGATCGAAACTTTAA  689

Query  601  TACATCATTTTTTGATCCTTCAGGGGGAGGAGATCCAATTTTATATCAACATTTATTT  658
            ||||||||||||||||||||||||||||||||||||||||||||||||||||||||||
Sbjct  690  TACATCATTTTTTGATCCTTCAGGGGGAGGAGATCCAATTTTATATCAACATTTATTT  747
```

Range 2: 30 to 64

Score:58.4 bits(31), Expect:0.001,
Identities:34/35(97%), Gaps:1/35(2%), Strand: Plus/Plus

```
Query  1   AACAATATATTTAATTTTTGGAGCATGATCA-GGT  34
           ||||||||||||||||||||||||||||||| |||
Sbjct  30  AACAATATATTTAATTTTTGGAGCATGATCATGGT  64
```

>Rhipicephalus sanguineus sensu lato 'temperate lineage' isolate S358 cytochrome c oxidase subunit I (COI) gene, partial cds; mitochondrial
Sequence ID: MF426012.1 Length: 684
Range 1: 14 to 671

Score:1194 bits(646), Expect:0.0,
Identities:654/658(99%), Gaps:0/658(0%), Strand: Plus/Plus

```
Query  1    AACAATATATTTAATTTTTGGAGCATGATCAGGTATATTAGGACTTAGTATAAGAATATT  60
            ||||||||||||||||||||||||||||||||||||||||||||||||||||||||||||
Sbjct  14   AACAATATATTTAATTTTTGGAGCATGATCAGGTATATTAGGACTTAGTATAAGAATATT  73

Query  61   AATTCGTATAGAATTAGGACAACCTGGAACTTTAATTGGAAATGATCAGATTTATAATGT  120
            ||||||||||||||||||||||||||||||||||||||||||||||||||||||||||||
```

```
Sbjct  74   AATTCGTATAGAATTAGGACAACCTGGAACTTTAATTGGAAATGATCAGATTTATAATGT  133

Query  121  AATCGTAACAGCACATGCATTTATTATAAttttttttATAGTTATACCAATCATAATCGG  180
            ||||||||||||||||||||||||||||||||||||||||||||||||||||||||||||
Sbjct  134  AATCGTAACAGCACATGCATTTATTATAATTTTTTTTATAGTTATACCAATCATAATCGG  193

Query  181  AGGATTTGGAAATTGATTAGTCCCTATCATATTGGGTGCCCCAGATATAGCATTTCCACG  240
            ||||||||||||||||||||||||||||||||| ||||||||||||||||||||||||||
Sbjct  194  AGGATTTGGAAATTGATTAGTCCCTATCATATTAGGTGCCCCAGATATAGCATTTCCACG  253

Query  241  AATAAATAATATAAGATTTTGACTTTTACCTCCTTCACTATTTTTATTAATTAACTCTTC  300
            ||||||||||||||||||||||||||||||||||||||||||||||||||||||||||||
Sbjct  254  AATAAATAATATAAGATTTTGACTTTTACCTCCTTCACTATTTTTATTAATTAACTCTTC  313

Query  301  ATTAATTGAATCTGGAGCTGGTACAGGATGAACAGTTTACCCTCCTCTATCTTCAAATTT  360
            ||||||||||||||||||||||||||||||||||||||||||||||||||||||||||||
Sbjct  314  ATTAATTGAATCTGGAGCTGGTACAGGATGAACAGTTTACCCTCCTCTATCTTCAAATTT  373

Query  361  ATCACATTATGGCCCTTCAGTAGATTTAGCTATTTTTTCTCTTCATCTTGCTGGTGCTTC  420
            ||||||||||||||||||||||||||||||||| ||||||||||||||||||||||||||
Sbjct  374  ATCACATTATGGCCCTTCAGTAGATTTAGCTATCTTTTCTCTTCATCTTGCTGGTGCTTC  433

Query  421  TTCAATTTTAGGTGCAATTAACTTTATTACAACTATTGTAAACATACGATCAATTGGAAT  480
            ||||||||||||||||||||||||||||||||||||| ||||||||||||||||||||||
Sbjct  434  TTCAATTTTAGGTGCAATTAACTTTATTACAACTATTATAAACATACGATCAATTGGAAT  493

Query  481  AACAATAGAACGTATACCATTATTTGTTTGATCTGTTTTAATTACAGCTATTTTATTACT  540
            ||||||||||||||||||||||||||||||||||||||||||||||||||||||||||||
Sbjct  494  AACAATAGAACGTATACCATTATTTGTTTGATCTGTTTTAATTACAGCTATTTTATTACT  553

Query  541  TTTATCTTTACCTGTATTAGCAGGTGCCATTACAATATTGTTAACTGATCGAAACTTTAA  600
             |||||||||||||||||||||||||||||||||||||||||||||||||||||||||||
Sbjct  554  CTTATCTTTACCTGTATTAGCAGGTGCCATTACAATATTGTTAACTGATCGAAACTTTAA  613

Query  601  TACATCATTTTTTGATCCTTCAGGGGGAGGAGATCCAATTTTATATCAACATTTATTT  658
            ||||||||||||||||||||||||||||||||||||||||||||||||||||||||||
Sbjct  614  TACATCATTTTTTGATCCTTCAGGGGGAGGAGATCCAATTTTATATCAACATTTATTT  671
```

>Rhipicephalus sanguineus sensu lato 'temperate lineage' isolate S337 cytochrome c oxidase subunit I (COI) gene, partial cds; mitochondrial
Sequence ID: MF426010.1 Length: 824
Range 1: 1 to 648

Score:1192 bits(645), Expect:0.0,
Identities:647/648(99%), Gaps:0/648(0%), Strand: Plus/Plus

```
Query  11   TTAATTTTTGGAGCATGATCAGGTATATTAGGACTTAGTATAAGAATATTAATTCGTATA  70
            ||||||||||||||||||||||||||||||||||||||||||||||||||||||||||||
Sbjct  1    TTAATTTTTGGAGCATGATCAGGTATATTAGGACTTAGTATAAGAATATTAATTCGTATA  60

Query  71   GAATTAGGACAACCTGGAACTTTAATTGGAAATGATCAGATTTATAATGTAATCGTAACA  130
```

```
             ||||||||||||||||||||||||||||||||||||||||||||||||||||||||||||
Sbjct  61    GAATTAGGACAACCTGGAACTTTAATTGGAAATGATCAGATTTATAATGTAATCGTAACA  120

Query  131   GCACATGCATTTATTATAAttttttttATAGTTATACCAATCATAATCGGAGGATTTGGA  190
             ||||||||||||||||||||||||||||||||||||||||||||||||||||||||||||
Sbjct  121   GCACATGCATTTATTATAATTTTTTTTATAGTTATACCAATCATAATCGGAGGATTTGGA  180

Query  191   AATTGATTAGTCCCTATCATATTGGGTGCCCCAGATATAGCATTTCCACGAATAAATAAT  250
             ||||||||||||||||||||||||||||||||||||||||||||||||||||||||||||
Sbjct  181   AATTGATTAGTCCCTATCATATTGGGTGCCCCAGATATAGCATTTCCACGAATAAATAAT  240

Query  251   ATAAGATTTTGACTTTTACCTCCTTCACTATTTTTATTAATTAACTCTTCATTAATTGAA  310
             ||||||||||||||||||||||||||||||||||||||||||||||||||||||||||||
Sbjct  241   ATAAGATTTTGACTTTTACCTCCTTCACTATTTTTATTAATTAACTCTTCATTAATTGAA  300

Query  311   TCTGGAGCTGGTACAGGATGAACAGTTTACCCTCCTCTATCTTCAAATTTATCACATTAT  370
             ||||||||||||||||||||||||||||||||||||||||||||||||||||||||||||
Sbjct  301   TCTGGAGCTGGTACAGGATGAACAGTTTACCCTCCTCTATCTTCAAATTTATCACATTAT  360

Query  371   GGCCCTTCAGTAGATTTAGCTATTTTTTCTCTTCATCTTGCTGGTGCTTCTTCAATTTTA  430
             ||||||||||||||||||||||| ||||||||||||||||||||||||||||||||||||
Sbjct  361   GGCCCTTCAGTAGATTTAGCTATCTTTTCTCTTCATCTTGCTGGTGCTTCTTCAATTTTA  420

Query  431   GGTGCAATTAACTTTATTACAACTATTGTAAACATACGATCAATTGGAATAACAATAGAA  490
             ||||||||||||||||||||||||||||||||||||||||||||||||||||||||||||
Sbjct  421   GGTGCAATTAACTTTATTACAACTATTGTAAACATACGATCAATTGGAATAACAATAGAA  480

Query  491   CGTATACCATTATTTGTTTGATCTGTTTTAATTACAGCTATTTTATTACTTTTATCTTTA  550
             ||||||||||||||||||||||||||||||||||||||||||||||||||||||||||||
Sbjct  481   CGTATACCATTATTTGTTTGATCTGTTTTAATTACAGCTATTTTATTACTTTTATCTTTA  540

Query  551   CCTGTATTAGCAGGTGCCATTACAATATTGTTAACTGATCGAAACTTTAATACATCATTT  610
             ||||||||||||||||||||||||||||||||||||||||||||||||||||||||||||
Sbjct  541   CCTGTATTAGCAGGTGCCATTACAATATTGTTAACTGATCGAAACTTTAATACATCATTT  600

Query  611   TTTGATCCTTCAGGGGGAGGAGATCCAATTTTATATCAACATTTATTT  658
             ||||||||||||||||||||||||||||||||||||||||||||||||
Sbjct  601   TTTGATCCTTCAGGGGGAGGAGATCCAATTTTATATCAACATTTATTT  648
```

```
>Rhipicephalus sanguineus haplotype II cytochrome oxidase subunit I (COI) gene,
partial cds; mitochondrial
Sequence ID: KU556744.1 Length: 787
Range 1: 29 to 688

Score:1190 bits(644), Expect:0.0,
Identities:655/660(99%),  Gaps:2/660(0%), Strand: Plus/Plus

Query  1     AACAATATATTTAATTTTTGGAGCATGATCAGGTATATTAGGACTTAGTATAAGAATATT  60
             ||||||||||||||||||||||||||||||||||||||||||||||||||||||||||||
Sbjct  29    AACAATATATTTAATTTTTGGAGCATGATCAGGTATATTAGGACTTAGTATAAGAATATT  88
```

```
Query  61    AATTCGTATAGAATTAGGACAACCTGGAACTTTAATTGGAAATGATCAGATTTATAATGT  120
             ||||||||||||||||||||||||||||||||||||||||||||||||||||||||||||
Sbjct  89    AATTCGTATAGAATTAGGACAACCTGGAACTTTAATTGGAAATGATCAGATTTATAATGT  148

Query  121   AATCGTAACAGCACATGCATTTATTATAAttttttttATAGTTATACCAATCATAATCGG  180
             ||||||||||||||||||||||||||||||||||||||||||||||||||||||||||||
Sbjct  149   AATCGTAACAGCACATGCATTTATTATAATTTTTTTTATAGTTATACCAATCATAATCGG  208

Query  181   AGGATTTGGAAATTGATTAGTCCCTATCATATTGGGTGCCCCAGATATAGCATTTCCACG  240
             |||||||||||| |||||||||||||||||||||||||||||||||||||||||||||||
Sbjct  209   AGGATTTGGAAACTGATTAGTCCCTATCATATTGGGTGCCCCAGATATAGCATTTCCACG  268

Query  241   AATAAATAATATAAGATTTTGACTTTTACCTCCTTCACTATTTTTATTAATTAACTCTTC  300
             ||||||||||||||||||||||||||||||||||||||||||||||||||||||||||||
Sbjct  269   AATAAATAATATAAGATTTTGACTTTTACCTCCTTCACTATTTTTATTAATTAACTCTTC  328

Query  301   ATTAATTGAATCTGGAGCTGGTACAGGATGAACAGTTTACCCTCCTCTATCTTCAAATTT  360
             ||||||||||||||||||||||||||||||||||||||||||||||||||||||||||||
Sbjct  329   ATTAATTGAATCTGGAGCTGGTACAGGATGAACAGTTTACCCTCCTCTATCTTCAAATTT  388

Query  361   ATCACATTATGGCCCTTCAGTAGATTTAGCTATTTTTTCTCTTCATCTTGCTGGTGCTTC  420
             |||||||||||| |||||||||||||||||||||||||||||||||||||||||||||||
Sbjct  389   ATCACATTATGGTCCTTCAGTAGATTTAGCTATTTTTTCTCTTCATCTTGCTGGTGCTTC  448

Query  421   TTCAATTTTAGGTGCAATTAACTTTATTACAACTATTGTAAACATACGATCAATTGGAAT  480
             ||||||||||||||||||||||||||||||||||||||||||||||||||||||||||||
Sbjct  449   TTCAATTTTAGGTGCAATTAACTTTATTACAACTATTGTAAACATACGATCAATTGGAAT  508

Query  481   AACAATAGAACGTATACCATTATTTGTTTGATCTGTTTTAATTACAGCTATTTTATTACT  540
             ||||||||||||||||||||||||||||||||||||||||||||||||||||||||||||
Sbjct  509   AACAATAGAACGTATACCATTATTTGTTTGATCTGTTTTAATTACAGCTATTTTATTACT  568

Query  541   TTTATCTTTACCTGTATTAGCAGGTGCCATTACAATATTGTTAACTGATCGAAACTTTAA  600
             ||||||||||||||||||||||||||||||||||||||||||||||||||||||||||||
Sbjct  569   TTTATCTTTACCTGTATTAGCAGGTGCCATTACAATATTGTTAACTGATCGAAACTTTAA  628

Query  601   TACATCATTTTTTGATCCTTCAGGGGG-AGGAGATCC-AATTTTATATCAACATTTATTT  658
             ||||||||||||||||||||||||||| ||||||||| |||||||||||||| |||||||
Sbjct  629   TACATCATTTTTTGATCCTTCAGGGGGGAGGAGATCCAAATTTTATATCAACTTTTATTT  688
```

>Rhipicephalus sanguineus haplotype I cytochrome oxidase subunit I (COI) gene, partial cds; mitochondrial
Sequence ID: KU556743.1 Length: 659
Range 1: 12 to 659

Score:1186 bits(642), Expect:0.0,
Identities:646/648(99%), Gaps:0/648(0%), Strand: Plus/Plus

```
Query  1     AACAATATATTTAATTTTTGGAGCATGATCAGGTATATTAGGACTTAGTATAAGAATATT  60
             ||||||||||||||||||||||||||||||||||||||||||||||||||||||||||||
Sbjct  12    AACAATATATTTAATTTTTGGAGCATGATCAGGTATATTAGGACTTAGTATAAGAATATT  71
```

```
Query  61   AATTCGTATAGAATTAGGACAACCTGGAACTTTAATTGGAAATGATCAGATTTATAATGT  120
            ||||||||||||||||||||||||||||||||||||||||||||||||||||||||||||
Sbjct  72   AATTCGTATAGAATTAGGACAACCTGGAACTTTAATTGGAAATGATCAGATTTATAATGT  131

Query  121  AATCGTAACAGCACATGCATTTATTATAAtttttttATAGTTATACCAATCATAATCGG  180
            ||||||||||||||||||||||||||||||||||||||||||||||||||||||||||||
Sbjct  132  AATCGTAACAGCACATGCATTTATTATAATTTTTTTATAGTTATACCAATCATAATCGG  191

Query  181  AGGATTTGGAAATTGATTAGTCCCTATCATATTGGGTGCCCCAGATATAGCATTTCCACG  240
            ||||||||||||||||||| ||||||||||||||||||||||||||||||||||||||||
Sbjct  192  AGGATTTGGAAATTGATTAATCCCTATCATATTGGGTGCCCCAGATATAGCATTTCCACG  251

Query  241  AATAAATAATATAAGATTTTGACTTTTACCTCCTTCACTATTTTTATTAATTAACTCTTC  300
            ||||||||||||||||||||||||||||||||||||||||||||||||||||||||||||
Sbjct  252  AATAAATAATATAAGATTTTGACTTTTACCTCCTTCACTATTTTTATTAATTAACTCTTC  311

Query  301  ATTAATTGAATCTGGAGCTGGTACAGGATGAACAGTTTACCCTCCTCTATCTTCAAATTT  360
            ||||||||||||||||||||||||||||||||||||||||||||||||||||||||||||
Sbjct  312  ATTAATTGAATCTGGAGCTGGTACAGGATGAACAGTTTACCCTCCTCTATCTTCAAATTT  371

Query  361  ATCACATTATGGCCCTTCAGTAGATTTAGCTATTTTTTCTCTTCATCTTGCTGGTGCTTC  420
            |||||||||||| |||||||||||||||||||||||||||||||||||||||||||||||
Sbjct  372  ATCACATTATGGTCCTTCAGTAGATTTAGCTATTTTTTCTCTTCATCTTGCTGGTGCTTC  431

Query  421  TTCAATTTTAGGTGCAATTAACTTTATTACAACTATTGTAAACATACGATCAATTGGAAT  480
            ||||||||||||||||||||||||||||||||||||||||||||||||||||||||||||
Sbjct  432  TTCAATTTTAGGTGCAATTAACTTTATTACAACTATTGTAAACATACGATCAATTGGAAT  491

Query  481  AACAATAGAACGTATACCATTATTTGTTTGATCTGTTTTAATTACAGCTATTTTATTACT  540
            ||||||||||||||||||||||||||||||||||||||||||||||||||||||||||||
Sbjct  492  AACAATAGAACGTATACCATTATTTGTTTGATCTGTTTTAATTACAGCTATTTTATTACT  551

Query  541  TTTATCTTTACCTGTATTAGCAGGTGCCATTACAATATTGTTAACTGATCGAAACTTTAA  600
            ||||||||||||||||||||||||||||||||||||||||||||||||||||||||||||
Sbjct  552  TTTATCTTTACCTGTATTAGCAGGTGCCATTACAATATTGTTAACTGATCGAAACTTTAA  611

Query  601  TACATCATTTTTTGATCCTTCAGGGGGAGGAGATCCAATTTTATATCA  648
            ||||||||||||||||||||||||||||||||||||||||||||||||
Sbjct  612  TACATCATTTTTTGATCCTTCAGGGGGAGGAGATCCAATTTTATATCA  659
```

>Rhipicephalus sanguineus sensu lato 'temperate lineage' isolate S943 cytochrome c oxidase subunit I (COI) gene, partial cds; mitochondrial
Sequence ID: MF426020.1 Length: 812
Range 1: 2 to 644

Score:1182 bits(640), Expect:0.0,
Identities:642/643(99%), Gaps:0/643(0%), Strand: Plus/Plus

```
Query  16   TTTTGGAGCATGATCAGGTATATTAGGACTTAGTATAAGAATATTAATTCGTATAGAATT  75
            ||||||||||||||||||||||||||||||||||||||||||||||||||||||||||||
```

```
Sbjct  2    TTTTGGAGCATGATCAGGTATATTAGGACTTAGTATAAGAATATTAATTCGTATAGAATT  61

Query  76   AGGACAACCTGGAACTTTAATTGGAAATGATCAGATTTATAATGTAATCGTAACAGCACA  135
            ||||||||||||||||||||||||||||||||||||||||||||||||||||||||||||
Sbjct  62   AGGACAACCTGGAACTTTAATTGGAAATGATCAGATTTATAATGTAATCGTAACAGCACA  121

Query  136  TGCATTTATTATAAttttttttATAGTTATACCAATCATAATCGGAGGATTTGGAAATTG  195
            ||||||||||||||||||||||||||||||||||||||||||||||||||||||||||||
Sbjct  122  TGCATTTATTATAATTTTTTTTATAGTTATACCAATCATAATCGGAGGATTTGGAAATTG  181

Query  196  ATTAGTCCCTATCATATTGGGTGCCCCAGATATAGCATTTCCACGAATAAATAATATAAG  255
            ||||||||||||||||||||||||||||||||||||||||||||||||||||||||||||
Sbjct  182  ATTAGTCCCTATCATATTGGGTGCCCCAGATATAGCATTTCCACGAATAAATAATATAAG  241

Query  256  ATTTTGACTTTTACCTCCTTCACTATTTTTATTAATTAACTCTTCATTAATTGAATCTGG  315
            ||||||||||||||||||||||||||||||||||||||||||||||||||||||||||||
Sbjct  242  ATTTTGACTTTTACCTCCTTCACTATTTTTATTAATTAACTCTTCATTAATTGAATCTGG  301

Query  316  AGCTGGTACAGGATGAACAGTTTACCCTCCTCTATCTTCAAATTTATCACATTATGGCCC  375
            ||||||||||||||||||||||||||||||||||||||||||||||||||||||||| ||
Sbjct  302  AGCTGGTACAGGATGAACAGTTTACCCTCCTCTATCTTCAAATTTATCACATTATGGTCC  361

Query  376  TTCAGTAGATTTAGCTATTTTTTCTCTTCATCTTGCTGGTGCTTCTTCAATTTTAGGTGC  435
            ||||||||||||||||||||||||||||||||||||||||||||||||||||||||||||
Sbjct  362  TTCAGTAGATTTAGCTATTTTTTCTCTTCATCTTGCTGGTGCTTCTTCAATTTTAGGTGC  421

Query  436  AATTAACTTTATTACAACTATTGTAAACATACGATCAATTGGAATAACAATAGAACGTAT  495
            ||||||||||||||||||||||||||||||||||||||||||||||||||||||||||||
Sbjct  422  AATTAACTTTATTACAACTATTGTAAACATACGATCAATTGGAATAACAATAGAACGTAT  481

Query  496  ACCATTATTTGTTTGATCTGTTTTAATTACAGCTATTTTATTACTTTTATCTTTACCTGT  555
            ||||||||||||||||||||||||||||||||||||||||||||||||||||||||||||
Sbjct  482  ACCATTATTTGTTTGATCTGTTTTAATTACAGCTATTTTATTACTTTTATCTTTACCTGT  541

Query  556  ATTAGCAGGTGCCATTACAATATTGTTAACTGATCGAAACTTTAATACATCATTTTTTGA  615
            ||||||||||||||||||||||||||||||||||||||||||||||||||||||||||||
Sbjct  542  ATTAGCAGGTGCCATTACAATATTGTTAACTGATCGAAACTTTAATACATCATTTTTTGA  601

Query  616  TCCTTCAGGGGGAGGAGATCCAATTTTATATCAACATTTATTT  658
            |||||||||||||||||||||||||||||||||||||||||||
Sbjct  602  TCCTTCAGGGGGAGGAGATCCAATTTTATATCAACATTTATTT  644
```

>Rhipicephalus sanguineus isolate LIC5424B cytochrome oxidase subunit 1 (COI) gene, partial cds; mitochondrial
Sequence ID: KX383818.1 Length: 768
>Rhipicephalus sanguineus isolate LIC5554A cytochrome oxidase subunit 1 (COI) gene, partial cds; mitochondrial
Sequence ID: KX383819.1 Length: 768
Range 1: 1 to 642

Score:1175 bits(636), Expect:0.0,

```
Identities:640/642(99%),  Gaps:0/642(0%), Strand: Plus/Plus

Query  17    TTTGGAGCATGATCAGGTATATTAGGACTTAGTATAAGAATATTAATTCGTATAGAATTA  76
             ||||||||||||||||||||||||||||||||||||||||||||||||||||||||||||
Sbjct  1     TTTGGAGCATGATCAGGTATATTAGGACTTAGTATAAGAATATTAATTCGTATAGAATTA  60

Query  77    GGACAACCTGGAACTTTAATTGGAAATGATCAGATTTATAATGTAATCGTAACAGCACAT  136
             ||||||||||||||||||||||||||||||||||||||||||||||||||||||||||||
Sbjct  61    GGACAACCTGGAACTTTAATTGGAAATGATCAGATTTATAATGTAATCGTAACAGCACAT  120

Query  137   GCATTTATTATAAttttttttATAGTTATACCAATCATAATCGGAGGATTTGGAAATTGA  196
             ||||||||||||||||||||||||||||||||||||||||||||||||||||||||||||
Sbjct  121   GCATTTATTATAATTTTTTTTATAGTTATACCAATCATAATCGGAGGATTTGGAAATTGA  180

Query  197   TTAGTCCCTATCATATTGGGTGCCCCAGATATAGCATTTCCACGAATAAATAATATAAGA  256
             ||||||||||||||||||||||||||||||||||||||||||||||||||||||||||||
Sbjct  181   TTAGTCCCTATCATATTGGGTGCCCCAGATATAGCATTTCCACGAATAAATAATATAAGA  240

Query  257   TTTTGACTTTTACCTCCTTCACTATTTTTATTAATTAACTCTTCATTAATTGAATCTGGA  316
             ||||||||||||||||||||||||||||||||||||||||||||||||||||||||||||
Sbjct  241   TTTTGACTTTTACCTCCTTCACTATTTTTATTAATTAACTCTTCATTAATTGAATCTGGA  300

Query  317   GCTGGTACAGGATGAACAGTTTACCCTCCTCTATCTTCAAATTTATCACATTATGGCCCT  376
             ||||||||||||||||||||||||||||||||||||||||||||||||||||||||||||
Sbjct  301   GCTGGTACAGGATGAACAGTTTACCCTCCTCTATCTTCAAATTTATCACATTATGGCCCT  360

Query  377   TCAGTAGATTTAGCTATTTTTTCTCTTCATCTTGCTGGTGCTTCTTCAATTTTAGGTGCA  436
             ||||||||||||||||| ||||||||||||||||||||||||||||||||||||||||||
Sbjct  361   TCAGTAGATTTAGCTATCTTTTCTCTTCATCTTGCTGGTGCTTCTTCAATTTTAGGTGCA  420

Query  437   ATTAACTTTATTACAACTATTGTAAACATACGATCAATTGGAATAACAATAGAACGTATA  496
             |||||||||||||||||||||||||||||||| |||||||||||||||||||||||||||
Sbjct  421   ATTAACTTTATTACAACTATTGTAAACATACGGTCAATTGGAATAACAATAGAACGTATA  480

Query  497   CCATTATTTGTTTGATCTGTTTTAATTACAGCTATTTTATTACTTTTATCTTTACCTGTA  556
             ||||||||||||||||||||||||||||||||||||||||||||||||||||||||||||
Sbjct  481   CCATTATTTGTTTGATCTGTTTTAATTACAGCTATTTTATTACTTTTATCTTTACCTGTA  540

Query  557   TTAGCAGGTGCCATTACAATATTGTTAACTGATCGAAACTTTAATACATCATTTTTTGAT  616
             ||||||||||||||||||||||||||||||||||||||||||||||||||||||||||||
Sbjct  541   TTAGCAGGTGCCATTACAATATTGTTAACTGATCGAAACTTTAATACATCATTTTTTGAT  600

Query  617   CCTTCAGGGGGAGGAGATCCAATTTTATATCAACATTTATTT  658
             ||||||||||||||||||||||||||||||||||||||||||
Sbjct  601   CCTTCAGGGGGAGGAGATCCAATTTTATATCAACATTTATTT  642
```

>Rhipicephalus sanguineus isolate LIC5533B cytochrome oxidase subunit 1 (COI) gene, partial cds; mitochondrial
Sequence ID: KX383811.1 Length: 764
>Rhipicephalus sanguineus isolate LIC5533C cytochrome oxidase subunit 1 (COI) gene, partial cds; mitochondrial

Sequence ID: KX383812.1 Length: 764
Range 1: 1 to 638

Score:1168 bits(632), Expect:0.0,
Identities:636/638(99%), Gaps:0/638(0%), Strand: Plus/Plus

```
Query  21   GAGCATGATCAGGTATATTAGGACTTAGTATAAGAATATTAATTCGTATAGAATTAGGAC  80
            ||||||||||||||||||||||||||||||||||||||||||||||||||||||||||||
Sbjct  1    GAGCATGATCAGGTATATTAGGACTTAGTATAAGAATATTAATTCGTATAGAATTAGGAC  60

Query  81   AACCTGGAACTTTAATTGGAAATGATCAGATTTATAATGTAATCGTAACAGCACATGCAT  140
            ||||||||||||||||||||||||||||||||||||||||||||||||||||||||||||
Sbjct  61   AACCTGGAACTTTAATTGGAAATGATCAGATTTATAATGTAATCGTAACAGCACATGCAT  120

Query  141  TTATTATAAttttttttATAGTTATACCAATCATAATCGGAGGATTTGGAAATTGATTAG  200
            ||||||||||||||||||||||||||||||||||||||||||||||||||||||||||||
Sbjct  121  TTATTATAATTTTTTTTATAGTTATACCAATCATAATCGGAGGATTTGGAAATTGATTAG  180

Query  201  TCCCTATCATATTGGGTGCCCCAGATATAGCATTTCCACGAATAAATAATATAAGATTTT  260
            ||||||||||||||||||||||||||||||||||||||||||||||||||||||||||||
Sbjct  181  TCCCTATCATATTGGGTGCCCCAGATATAGCATTTCCACGAATAAATAATATAAGATTTT  240

Query  261  GACTTTTACCTCCTTCACTATTTTTATTAATTAACTCTTCATTAATTGAATCTGGAGCTG  320
            ||||||||||||||||||||||||||||||||||||||||||||||||||||||||||||
Sbjct  241  GACTTTTACCTCCTTCACTATTTTTATTAATTAACTCTTCATTAATTGAATCTGGAGCTG  300

Query  321  GTACAGGATGAACAGTTTACCCTCCTCTATCTTCAAATTTATCACATTATGGCCCTTCAG  380
            ||||||||||||||||||||||||||||||||||||||||||||||||||||||||||||
Sbjct  301  GTACAGGATGAACAGTTTACCCTCCTCTATCTTCAAATTTATCACATTATGGCCCTTCAG  360

Query  381  TAGATTTAGCTATTTTTTCTCTTCATCTTGCTGGTGCTTCTTCAATTTTAGGTGCAATTA  440
            ||||||||||||| ||||||||||||||||||||||||||||||||||||||||||||||
Sbjct  361  TAGATTTAGCTATCTTTTCTCTTCATCTTGCTGGTGCTTCTTCAATTTTAGGTGCAATTA  420

Query  441  ACTTTATTACAACTATTGTAAACATACGATCAATTGGAATAACAATAGAACGTATACCAT  500
            |||||||||||||||||||||||||||| |||||||||||||||||||||||||||||||
Sbjct  421  ACTTTATTACAACTATTGTAAACATACGGTCAATTGGAATAACAATAGAACGTATACCAT  480

Query  501  TATTTGTTTGATCTGTTTTAATTACAGCTATTTTATTACTTTTATCTTTACCTGTATTAG  560
            ||||||||||||||||||||||||||||||||||||||||||||||||||||||||||||
Sbjct  481  TATTTGTTTGATCTGTTTTAATTACAGCTATTTTATTACTTTTATCTTTACCTGTATTAG  540

Query  561  CAGGTGCCATTACAATATTGTTAACTGATCGAAACTTTAATACATCATTTTTTGATCCTT  620
            ||||||||||||||||||||||||||||||||||||||||||||||||||||||||||||
Sbjct  541  CAGGTGCCATTACAATATTGTTAACTGATCGAAACTTTAATACATCATTTTTTGATCCTT  600

Query  621  CAGGGGGAGGAGATCCAATTTTATATCAACATTTATTT  658
            ||||||||||||||||||||||||||||||||||||||
Sbjct  601  CAGGGGGAGGAGATCCAATTTTATATCAACATTTATTT  638
```

>Rhipicephalus sanguineus isolate LIC4736B cytochrome oxidase subunit 1 (COI)

gene, partial cds; mitochondrial
Sequence ID: KX383807.1 Length: 763
>Rhipicephalus sanguineus isolate LIC4736C cytochrome oxidase subunit 1 (COI) gene, partial cds; mitochondrial
Sequence ID: KX383808.1 Length: 763
>Rhipicephalus sanguineus isolate LIC4736F cytochrome oxidase subunit 1 (COI) gene, partial cds; mitochondrial
Sequence ID: KX383810.1 Length: 763
>Rhipicephalus sanguineus isolate LIC5535C cytochrome oxidase subunit 1 (COI) gene, partial cds; mitochondrial
Sequence ID: KX383813.1 Length: 763
Range 1: 1 to 637

Score:1166 bits(631), Expect:0.0,
Identities:635/637(99%), Gaps:0/637(0%), Strand: Plus/Plus

```
Query  22   AGCATGATCAGGTATATTAGGACTTAGTATAAGAATATTAATTCGTATAGAATTAGGACA  81
            ||||||||||||||||||||||||||||||||||||||||||||||||||||||||||||
Sbjct  1    AGCATGATCAGGTATATTAGGACTTAGTATAAGAATATTAATTCGTATAGAATTAGGACA  60

Query  82   ACCTGGAACTTTAATTGGAAATGATCAGATTTATAATGTAATCGTAACAGCACATGCATT  141
            ||||||||||||||||||||||||||||||||||||||||||||||||||||||||||||
Sbjct  61   ACCTGGAACTTTAATTGGAAATGATCAGATTTATAATGTAATCGTAACAGCACATGCATT  120

Query  142  TATTATAAttttttttATAGTTATACCAATCATAATCGGAGGATTTGGAAATTGATTAGT  201
            ||||||||||||||||||||||||||||||||||||||||||||||||||||||||||||
Sbjct  121  TATTATAATTTTTTTTATAGTTATACCAATCATAATCGGAGGATTTGGAAATTGATTAGT  180

Query  202  CCCTATCATATTGGGTGCCCCAGATATAGCATTTCCACGAATAAATAATATAAGATTTTG  261
            ||||||||||||||||||||||||||||||||||||||||||||||||||||||||||||
Sbjct  181  CCCTATCATATTGGGTGCCCCAGATATAGCATTTCCACGAATAAATAATATAAGATTTTG  240

Query  262  ACTTTTACCTCCTTCACTATTTTTATTAATTAACTCTTCATTAATTGAATCTGGAGCTGG  321
            ||||||||||||||||||||||||||||||||||||||||||||||||||||||||||||
Sbjct  241  ACTTTTACCTCCTTCACTATTTTTATTAATTAACTCTTCATTAATTGAATCTGGAGCTGG  300

Query  322  TACAGGATGAACAGTTTACCCTCCTCTATCTTCAAATTTATCACATTATGGCCCTTCAGT  381
            ||||||||||||||||||||||||||||||||||||||||||||||||||||||||||||
Sbjct  301  TACAGGATGAACAGTTTACCCTCCTCTATCTTCAAATTTATCACATTATGGCCCTTCAGT  360

Query  382  AGATTTAGCTATTTTTTCTCTTCATCTTGCTGGTGCTTCTTCAATTTTAGGTGCAATTAA  441
            |||||||||||| |||||||||||||||||||||||||||||||||||||||||||||||
Sbjct  361  AGATTTAGCTATCTTTTCTCTTCATCTTGCTGGTGCTTCTTCAATTTTAGGTGCAATTAA  420

Query  442  CTTTATTACAACTATTGTAAACATACGATCAATTGGAATAACAATAGAACGTATACCATT  501
            |||||||||||||||||||||||||||| |||||||||||||||||||||||||||||||
Sbjct  421  CTTTATTACAACTATTGTAAACATACGGTCAATTGGAATAACAATAGAACGTATACCATT  480

Query  502  ATTTGTTTGATCTGTTTTAATTACAGCTATTTTATTACTTTTATCTTTACCTGTATTAGC  561
            ||||||||||||||||||||||||||||||||||||||||||||||||||||||||||||
Sbjct  481  ATTTGTTTGATCTGTTTTAATTACAGCTATTTTATTACTTTTATCTTTACCTGTATTAGC  540

Query  562  AGGTGCCATTACAATATTGTTAACTGATCGAAACTTTAATACATCATTTTTTGATCCTTC  621
            ||||||||||||||||||||||||||||||||||||||||||||||||||||||||||||
```

```
Sbjct  541  AGGTGCCATTACAATATTGTTAACTGATCGAAACTTTAATACATCATTTTTTGATCCTTC  600

Query  622  AGGGGGAGGAGATCCAATTTTATATCAACATTTATTT  658
            |||||||||||||||||||||||||||||||||||||
Sbjct  601  AGGGGGAGGAGATCCAATTTTATATCAACATTTATTT  637
```

```
>Rhipicephalus sanguineus sensu lato 'temperate lineage' isolate S344 cytochrome
c oxidase subunit I (COI) gene, partial cds; mitochondrial
Sequence ID: MF426011.1 Length: 809
Range 1: 3 to 644

Score:1164 bits(630), Expect:0.0,
Identities:638/642(99%),  Gaps:0/642(0%), Strand: Plus/Plus

Query  17   TTTGGAGCATGATCAGGTATATTAGGACTTAGTATAAGAATATTAATTCGTATAGAATTA  76
            ||||||||||||||||||||||||||||||||||||||||||||||||||||||||||||
Sbjct  3    TTTGGAGCATGATCAGGTATATTAGGACTTAGTATAAGAATATTAATTCGTATAGAATTA  62

Query  77   GGACAACCTGGAACTTTAATTGGAAATGATCAGATTTATAATGTAATCGTAACAGCACAT  136
            ||||||||||||||||||||||||||||||||||||||||||||||||||||||||||| 
Sbjct  63   GGACAACCTGGAACTTTAATTGGAAATGATCAGATTTATAATGTAATCGTAACAGCACAC  122

Query  137  GCATTTATTATAAtttttttATAGTTATACCAATCATAATCGGAGGATTTGGAAATTGA  196
            ||||||||||||||||||||||||||||||||||||||||||||||||||||||||||||
Sbjct  123  GCATTTATTATAATTTTTTTATAGTTATACCAATCATAATCGGAGGATTTGGAAATTGA  182

Query  197  TTAGTCCCTATCATATTGGGTGCCCCAGATATAGCATTTCCACGAATAAATAATATAAGA  256
            ||||||||||| ||||| ||||||||||||||||||||||||||||||||||||||||||
Sbjct  183  TTAGTCCCTATTATATTAGGTGCCCCAGATATAGCATTTCCACGAATAAATAATATAAGA  242

Query  257  TTTTGACTTTTACCTCCTTCACTATTTTTATTAATTAACTCTTCATTAATTGAATCTGGA  316
            ||||||||||||||||||||||||||||||||||||||||||||||||||||||||||||
Sbjct  243  TTTTGACTTTTACCTCCTTCACTATTTTTATTAATTAACTCTTCATTAATTGAATCTGGA  302

Query  317  GCTGGTACAGGATGAACAGTTTACCCTCCTCTATCTTCAAATTTATCACATTATGGCCCT  376
            ||||||||||||||||||||||||||||||||||||||||||||||||||||||||||||
Sbjct  303  GCTGGTACAGGATGAACAGTTTACCCTCCTCTATCTTCAAATTTATCACATTATGGCCCT  362

Query  377  TCAGTAGATTTAGCTATTTTTTCTCTTCATCTTGCTGGTGCTTCTTCAATTTTAGGTGCA  436
            ||||||||||||||||| ||||||||||||||||||||||||||||||||||||||||||
Sbjct  363  TCAGTAGATTTAGCTATCTTTTCTCTTCATCTTGCTGGTGCTTCTTCAATTTTAGGTGCA  422

Query  437  ATTAACTTTATTACAACTATTGTAAACATACGATCAATTGGAATAACAATAGAACGTATA  496
            ||||||||||||||||||||||||||||||||||||||||||||||||||||||||||||
Sbjct  423  ATTAACTTTATTACAACTATTGTAAACATACGATCAATTGGAATAACAATAGAACGTATA  482

Query  497  CCATTATTTGTTTGATCTGTTTTAATTACAGCTATTTTATTACTTTTATCTTTACCTGTA  556
            ||||||||||||||||||||||||||||||||||||||||||||||||||||||||||||
Sbjct  483  CCATTATTTGTTTGATCTGTTTTAATTACAGCTATTTTATTACTTTTATCTTTACCTGTA  542

Query  557  TTAGCAGGTGCCATTACAATATTGTTAACTGATCGAAACTTTAATACATCATTTTTTGAT  616
```

```
           ||||||||||||||||||||||||||||||||||||||||||||||||||||||||||||
Sbjct  543  TTAGCAGGTGCCATTACAATATTGTTAACTGATCGAAACTTTAATACATCATTTTTTGAT  602

Query  617  CCTTCAGGGGGAGGAGATCCAATTTTATATCAACATTTATTT  658
            ||||||||||||||||||||||||||||||||||||||||||
Sbjct  603  CCTTCAGGGGGAGGAGATCCAATTTTATATCAACATTTATTT  644
```

>Rhipicephalus sanguineus isolate LIC4736D cytochrome oxidase subunit 1 (COI) gene, partial cds; mitochondrial
Sequence ID: KX383809.1 Length: 762
Range 1: 1 to 636

Score:1164 bits(630), Expect:0.0,
Identities:634/636(99%), Gaps:0/636(0%), Strand: Plus/Plus

```
Query  23   GCATGATCAGGTATATTAGGACTTAGTATAAGAATATTAATTCGTATAGAATTAGGACAA  82
            ||||||||||||||||||||||||||||||||||||||||||||||||||||||||||||
Sbjct  1    GCATGATCAGGTATATTAGGACTTAGTATAAGAATATTAATTCGTATAGAATTAGGACAA  60

Query  83   CCTGGAACTTTAATTGGAAATGATCAGATTTATAATGTAATCGTAACAGCACATGCATTT  142
            ||||||||||||||||||||||||||||||||||||||||||||||||||||||||||||
Sbjct  61   CCTGGAACTTTAATTGGAAATGATCAGATTTATAATGTAATCGTAACAGCACATGCATTT  120

Query  143  ATTATAAtttttttATAGTTATACCAATCATAATCGGAGGATTTGGAAATTGATTAGTC  202
            ||||||||||||||||||||||||||||||||||||||||||||||||||||||||||||
Sbjct  121  ATTATAATTTTTTTATAGTTATACCAATCATAATCGGAGGATTTGGAAATTGATTAGTC  180

Query  203  CCTATCATATTGGGTGCCCCAGATATAGCATTTCCACGAATAAATAATATAAGATTTTGA  262
            ||||||||||||||||||||||||||||||||||||||||||||||||||||||||||||
Sbjct  181  CCTATCATATTGGGTGCCCCAGATATAGCATTTCCACGAATAAATAATATAAGATTTTGA  240

Query  263  CTTTTACCTCCTTCACTATTTTTATTAATTAACTCTTCATTAATTGAATCTGGAGCTGGT  322
            ||||||||||||||||||||||||||||||||||||||||||||||||||||||||||||
Sbjct  241  CTTTTACCTCCTTCACTATTTTTATTAATTAACTCTTCATTAATTGAATCTGGAGCTGGT  300

Query  323  ACAGGATGAACAGTTTACCCTCCTCTATCTTCAAATTTATCACATTATGGCCCTTCAGTA  382
            ||||||||||||||||||||||||||||||||||||||||||||||||||||||||||||
Sbjct  301  ACAGGATGAACAGTTTACCCTCCTCTATCTTCAAATTTATCACATTATGGCCCTTCAGTA  360

Query  383  GATTTAGCTATTTTTTCTCTTCATCTTGCTGGTGCTTCTTCAATTTTAGGTGCAATTAAC  442
            ||||||||||| ||||||||||||||||||||||||||||||||||||||||||||||||
Sbjct  361  GATTTAGCTATCTTTTCTCTTCATCTTGCTGGTGCTTCTTCAATTTTAGGTGCAATTAAC  420

Query  443  TTTATTACAACTATTGTAAACATACGATCAATTGGAATAACAATAGAACGTATACCATTA  502
            |||||||||||||||||||||||||| |||||||||||||||||||||||||||||||||
Sbjct  421  TTTATTACAACTATTGTAAACATACGGTCAATTGGAATAACAATAGAACGTATACCATTA  480

Query  503  TTTGTTTGATCTGTTTTAATTACAGCTATTTTATTACTTTTATCTTTACCTGTATTAGCA  562
            ||||||||||||||||||||||||||||||||||||||||||||||||||||||||||||
Sbjct  481  TTTGTTTGATCTGTTTTAATTACAGCTATTTTATTACTTTTATCTTTACCTGTATTAGCA  540
```

```
Query  563  GGTGCCATTACAATATTGTTAACTGATCGAAACTTTAATACATCATTTTTTGATCCTTCA  622
            ||||||||||||||||||||||||||||||||||||||||||||||||||||||||||||
Sbjct  541  GGTGCCATTACAATATTGTTAACTGATCGAAACTTTAATACATCATTTTTTGATCCTTCA  600

Query  623  GGGGGAGGAGATCCAATTTTATATCAACATTTATTT  658
            ||||||||||||||||||||||||||||||||||||
Sbjct  601  GGGGGAGGAGATCCAATTTTATATCAACATTTATTT  636
```

>Rhipicephalus sanguineus isolate LIC4727D cytochrome oxidase subunit 1 (COI) gene, partial cds; mitochondrial
Sequence ID: KX383806.1 Length: 714
Range 1: 1 to 636

Score:1164 bits(630), Expect:0.0,
Identities:634/636(99%), Gaps:0/636(0%), Strand: Plus/Plus

```
Query  23   GCATGATCAGGTATATTAGGACTTAGTATAAGAATATTAATTCGTATAGAATTAGGACAA  82
            ||||||||||||||||||||||||||||||||||||||||||||||||||||||||||||
Sbjct  1    GCATGATCAGGTATATTAGGACTTAGTATAAGAATATTAATTCGTATAGAATTAGGACAA  60

Query  83   CCTGGAACTTTAATTGGAAATGATCAGATTTATAATGTAATCGTAACAGCACATGCATTT  142
            ||||||||||||||||||||||||||||||||||||||||||||||||||||||||||||
Sbjct  61   CCTGGAACTTTAATTGGAAATGATCAGATTTATAATGTAATCGTAACAGCACATGCATTT  120

Query  143  ATTATAAtttttttATAGTTATACCAATCATAATCGGAGGATTTGGAAATTGATTAGTC  202
            ||||||||||||||||||||||||||||||||||||||||||||||||||||||||||||
Sbjct  121  ATTATAATTTTTTTATAGTTATACCAATCATAATCGGAGGATTTGGAAATTGATTAGTC  180

Query  203  CCTATCATATTGGGTGCCCCAGATATAGCATTTCCACGAATAAATAATATAAGATTTTGA  262
            ||||||||||||||||||||||||||||||||||||||||||||||||||||||||||||
Sbjct  181  CCTATCATATTGGGTGCCCCAGATATAGCATTTCCACGAATAAATAATATAAGATTTTGA  240

Query  263  CTTTTACCTCCTTCACTATTTTTATTAATTAACTCTTCATTAATTGAATCTGGAGCTGGT  322
            ||||||||||||||||||||||||||||||||||||||||||||||||||||||||||||
Sbjct  241  CTTTTACCTCCTTCACTATTTTTATTAATTAACTCTTCATTAATTGAATCTGGAGCTGGT  300

Query  323  ACAGGATGAACAGTTTACCCTCCTCTATCTTCAAATTTATCACATTATGGCCCTTCAGTA  382
            ||||||||||||||||||||||||||||||||||||||||||||||||||||||||||||
Sbjct  301  ACAGGATGAACAGTTTACCCTCCTCTATCTTCAAATTTATCACATTATGGCCCTTCAGTA  360

Query  383  GATTTAGCTATTTTTTCTCTTCATCTTGCTGGTGCTTCTTCAATTTTAGGTGCAATTAAC  442
            ||||||||||| ||||||||||||||||||||||||||||||||||||||||||||||||
Sbjct  361  GATTTAGCTATCTTTTCTCTTCATCTTGCTGGTGCTTCTTCAATTTTAGGTGCAATTAAC  420

Query  443  TTTATTACAACTATTGTAAACATACGATCAATTGGAATAACAATAGAACGTATACCATTA  502
            |||||||||||||||||||||||||| |||||||||||||||||||||||||||||||||
Sbjct  421  TTTATTACAACTATTGTAAACATACGGTCAATTGGAATAACAATAGAACGTATACCATTA  480

Query  503  TTTGTTTGATCTGTTTTAATTACAGCTATTTTATTACTTTTATCTTTACCTGTATTAGCA  562
            ||||||||||||||||||||||||||||||||||||||||||||||||||||||||||||
Sbjct  481  TTTGTTTGATCTGTTTTAATTACAGCTATTTTATTACTTTTATCTTTACCTGTATTAGCA  540
```

```
Query  563  GGTGCCATTACAATATTGTTAACTGATCGAAACTTTAATACATCATTTTTTGATCCTTCA  622
            ||||||||||||||||||||||||||||||||||||||||||||||||||||||||||||
Sbjct  541  GGTGCCATTACAATATTGTTAACTGATCGAAACTTTAATACATCATTTTTTGATCCTTCA  600

Query  623  GGGGGAGGAGATCCAATTTTATATCAACATTTATTT  658
            ||||||||||||||||||||||||||||||||||||
Sbjct  601  GGGGGAGGAGATCCAATTTTATATCAACATTTATTT  636
```

>Rhipicephalus sanguineus isolate LIC4727C cytochrome oxidase subunit 1 (COI) gene, partial cds; mitochondrial
Sequence ID: KX383805.1 Length: 761
Range 1: 1 to 635

Score:1162 bits(629), Expect:0.0,
Identities:633/635(99%), Gaps:0/635(0%), Strand: Plus/Plus

```
Query  24   CATGATCAGGTATATTAGGACTTAGTATAAGAATATTAATTCGTATAGAATTAGGACAAC  83
            ||||||||||||||||||||||||||||||||||||||||||||||||||||||||||||
Sbjct  1    CATGATCAGGTATATTAGGACTTAGTATAAGAATATTAATTCGTATAGAATTAGGACAAC  60

Query  84   CTGGAACTTTAATTGGAAATGATCAGATTTATAATGTAATCGTAACAGCACATGCATTTA  143
            ||||||||||||||||||||||||||||||||||||||||||||||||||||||||||||
Sbjct  61   CTGGAACTTTAATTGGAAATGATCAGATTTATAATGTAATCGTAACAGCACATGCATTTA  120

Query  144  TTATAAttttttttATAGTTATACCAATCATAATCGGAGGATTTGGAAATTGATTAGTCC  203
            ||||||||||||||||||||||||||||||||||||||||||||||||||||||||||||
Sbjct  121  TTATAATTTTTTTTATAGTTATACCAATCATAATCGGAGGATTTGGAAATTGATTAGTCC  180

Query  204  CTATCATATTGGGTGCCCCAGATATAGCATTTCCACGAATAAATAATATAAGATTTTGAC  263
            ||||||||||||||||||||||||||||||||||||||||||||||||||||||||||||
Sbjct  181  CTATCATATTGGGTGCCCCAGATATAGCATTTCCACGAATAAATAATATAAGATTTTGAC  240

Query  264  TTTTACCTCCTTCACTATTTTTATTAATTAACTCTTCATTAATTGAATCTGGAGCTGGTA  323
            ||||||||||||||||||||||||||||||||||||||||||||||||||||||||||||
Sbjct  241  TTTTACCTCCTTCACTATTTTTATTAATTAACTCTTCATTAATTGAATCTGGAGCTGGTA  300

Query  324  CAGGATGAACAGTTTACCCTCCTCTATCTTCAAATTTATCACATTATGGCCCTTCAGTAG  383
            ||||||||||||||||||||||||||||||||||||||||||||||||||||||||||||
Sbjct  301  CAGGATGAACAGTTTACCCTCCTCTATCTTCAAATTTATCACATTATGGCCCTTCAGTAG  360

Query  384  ATTTAGCTATTTTTTCTCTTCATCTTGCTGGTGCTTCTTCAATTTTAGGTGCAATTAACT  443
            |||||||||| |||||||||||||||||||||||||||||||||||||||||||||||||
Sbjct  361  ATTTAGCTATCTTTTCTCTTCATCTTGCTGGTGCTTCTTCAATTTTAGGTGCAATTAACT  420

Query  444  TTATTACAACTATTGTAAACATACGATCAATTGGAATAACAATAGAACGTATACCATTAT  503
            ||||||||||||||||||||||||| ||||||||||||||||||||||||||||||||||
Sbjct  421  TTATTACAACTATTGTAAACATACGGTCAATTGGAATAACAATAGAACGTATACCATTAT  480

Query  504  TTGTTTGATCTGTTTTAATTACAGCTATTTTATTACTTTTATCTTTACCTGTATTAGCAG  563
            ||||||||||||||||||||||||||||||||||||||||||||||||||||||||||||
```

```
Sbjct  481   TTGTTTGATCTGTTTTAATTACAGCTATTTTATTACTTTTATCTTTACCTGTATTAGCAG  540

Query  564   GTGCCATTACAATATTGTTAACTGATCGAAACTTTAATACATCATTTTTTGATCCTTCAG  623
             ||||||||||||||||||||||||||||||||||||||||||||||||||||||||||||
Sbjct  541   GTGCCATTACAATATTGTTAACTGATCGAAACTTTAATACATCATTTTTTGATCCTTCAG  600

Query  624   GGGGAGGAGATCCAATTTTATATCAACATTTATTT  658
             |||||||||||||||||||||||||||||||||||
Sbjct  601   GGGGAGGAGATCCAATTTTATATCAACATTTATTT  635
```

>Rhipicephalus sanguineus sensu lato 'temperate lineage' isolate SF3003
cytochrome c oxidase subunit I (COI) gene, partial cds; mitochondrial
Sequence ID: MF425988.1 Length: 654
Range 1: 1 to 640

Score:1160 bits(628), Expect:0.0,
Identities:636/640(99%), Gaps:0/640(0%), Strand: Plus/Plus

```
Query  17    TTTGGAGCATGATCAGGTATATTAGGACTTAGTATAAGAATATTAATTCGTATAGAATTA  76
             ||||||||||||||||||||||||||||||||||||||||||||||||||||||||||||
Sbjct  1     TTTGGAGCATGATCAGGTATATTAGGACTTAGTATAAGAATATTAATTCGTATAGAATTA  60

Query  77    GGACAACCTGGAACTTTAATTGGAAATGATCAGATTTATAATGTAATCGTAACAGCACAT  136
             ||||||||||||||||||||||||||||||||||||||||||||||||||||||||||||
Sbjct  61    GGACAACCTGGAACTTTAATTGGAAATGATCAGATTTATAATGTAATCGTAACAGCACAT  120

Query  137   GCATTTATTATAAtttttttATAGTTATACCAATCATAATCGGAGGATTTGGAAATTGA  196
             ||||||||||||||||||||||||||||||||||||||||||||||||||||||||||||
Sbjct  121   GCATTTATTATAATTTTTTTATAGTTATACCAATCATAATCGGAGGATTTGGAAATTGA  180

Query  197   TTAGTCCCTATCATATTGGGTGCCCCAGATATAGCATTTCCACGAATAAATAATATAAGA  256
             ||||||||||||||||| ||||||||||||||||||||||||||||||||||||||||||
Sbjct  181   TTAGTCCCTATCATATTAGGTGCCCCAGATATAGCATTTCCACGAATAAATAATATAAGA  240

Query  257   TTTTGACTTTTACCTCCTTCACTATTTTTATTAATTAACTCTTCATTAATTGAATCTGGA  316
             ||||||||||||||||||||||||||||||||||||||||||||||||||||||||||||
Sbjct  241   TTTTGACTTTTACCTCCTTCACTATTTTTATTAATTAACTCTTCATTAATTGAATCTGGA  300

Query  317   GCTGGTACAGGATGAACAGTTTACCCTCCTCTATCTTCAAATTTATCACATTATGGCCCT  376
             ||||||||||||||||||||||||||||||||||||||||||||||||||||||||||||
Sbjct  301   GCTGGTACAGGATGAACAGTTTACCCTCCTCTATCTTCAAATTTATCACATTATGGCCCT  360

Query  377   TCAGTAGATTTAGCTATTTTTTTCTCTTCATCTTGCTGGTGCTTCTTCAATTTTAGGTGCA  436
             ||||||||||||||||| ||||||||||||||||||||||||||||||||||||||||||
Sbjct  361   TCAGTAGATTTAGCTATCTTTTTCTCTTCATCTTGCTGGTGCTTCTTCAATTTTAGGTGCA  420

Query  437   ATTAACTTTATTACAACTATTGTAAACATACGATCAATTGGAATAACAATAGAACGTATA  496
             ||||||||||||||||||||||||||||||||||||||||||||||||||||||||||||
Sbjct  421   ATTAACTTTATTACAACTATTGTAAACATACGATCAATTGGAATAACAATAGAACGTATA  480

Query  497   CCATTATTTGTTTGATCTGTTTTAATTACAGCTATTTTATTACTTTTATCTTTACCTGTA  556
```

```
                ||||||||||||||||||||||||||||||||||||||||||||||||||||||||||||
Sbjct  481   CCATTATTTGTTTGATCTGTTTTAATTACAGCTATTTTATTACTTTTATCTTTACCTGTA  540

Query  557   TTAGCAGGTGCCATTACAATATTGTTAACTGATCGAAACTTTAATACATCATTTTTTGAT  616
             ||||||||||||||||||||||||||||||||||||||||||||||||||||||||||||
Sbjct  541   TTAGCAGGTGCCATTACAATATTGTTAACTGATCGAAACTTTAATACATCATTTTTTGAT  600

Query  617   CCTTCAGGGGGAGGAGATCCAATTTTATATCAACATTTAT  656
             ||||||||||||||| |||||||||||||||| |||||||
Sbjct  601   CCTTCAGGGGGAGGACATCCAATTTTATATCACCATTTAT  640
```

```
>Rhipicephalus sanguineus clone #7 cytochrome oxidase subunit I (COI) gene,
partial cds; mitochondrial
Sequence ID: KX519712.1 Length: 654
Range 1: 3 to 628

Score:1157 bits(626), Expect:0.0,
Identities:626/626(100%),  Gaps:0/626(0%), Strand: Plus/Plus

Query  33    GTATATTAGGACTTAGTATAAGAATATTAATTCGTATAGAATTAGGACAACCTGGAACTT  92
             ||||||||||||||||||||||||||||||||||||||||||||||||||||||||||||
Sbjct  3     GTATATTAGGACTTAGTATAAGAATATTAATTCGTATAGAATTAGGACAACCTGGAACTT  62

Query  93    TAATTGGAAATGATCAGATTTATAATGTAATCGTAACAGCACATGCATTTATTATAAttt  152
             ||||||||||||||||||||||||||||||||||||||||||||||||||||||||||||
Sbjct  63    TAATTGGAAATGATCAGATTTATAATGTAATCGTAACAGCACATGCATTTATTATAATTT  122

Query  153   tttttATAGTTATACCAATCATAATCGGAGGATTTGGAAATTGATTAGTCCCTATCATAT  212
             ||||||||||||||||||||||||||||||||||||||||||||||||||||||||||||
Sbjct  123   TTTTTATAGTTATACCAATCATAATCGGAGGATTTGGAAATTGATTAGTCCCTATCATAT  182

Query  213   TGGGTGCCCCAGATATAGCATTTCCACGAATAAATAATATAAGATTTTGACTTTTACCTC  272
             ||||||||||||||||||||||||||||||||||||||||||||||||||||||||||||
Sbjct  183   TGGGTGCCCCAGATATAGCATTTCCACGAATAAATAATATAAGATTTTGACTTTTACCTC  242

Query  273   CTTCACTATTTTTATTAATTAACTCTTCATTAATTGAATCTGGAGCTGGTACAGGATGAA  332
             ||||||||||||||||||||||||||||||||||||||||||||||||||||||||||||
Sbjct  243   CTTCACTATTTTTATTAATTAACTCTTCATTAATTGAATCTGGAGCTGGTACAGGATGAA  302

Query  333   CAGTTTACCCTCCTCTATCTTCAAATTTATCACATTATGGCCCTTCAGTAGATTTAGCTA  392
             ||||||||||||||||||||||||||||||||||||||||||||||||||||||||||||
Sbjct  303   CAGTTTACCCTCCTCTATCTTCAAATTTATCACATTATGGCCCTTCAGTAGATTTAGCTA  362

Query  393   TTTTTTCTCTTCATCTTGCTGGTGCTTCTTCAATTTTAGGTGCAATTAACTTTATTACAA  452
             ||||||||||||||||||||||||||||||||||||||||||||||||||||||||||||
Sbjct  363   TTTTTTCTCTTCATCTTGCTGGTGCTTCTTCAATTTTAGGTGCAATTAACTTTATTACAA  422

Query  453   CTATTGTAAACATACGATCAATTGGAATAACAATAGAACGTATACCATTATTTGTTTGAT  512
             ||||||||||||||||||||||||||||||||||||||||||||||||||||||||||||
Sbjct  423   CTATTGTAAACATACGATCAATTGGAATAACAATAGAACGTATACCATTATTTGTTTGAT  482
```

```
Query  513  CTGTTTTAATTACAGCTATTTTATTACTTTTATCTTTACCTGTATTAGCAGGTGCCATTA  572
            ||||||||||||||||||||||||||||||||||||||||||||||||||||||||||||
Sbjct  483  CTGTTTTAATTACAGCTATTTTATTACTTTTATCTTTACCTGTATTAGCAGGTGCCATTA  542

Query  573  CAATATTGTTAACTGATCGAAACTTTAATACATCATTTTTTGATCCTTCAGGGGGAGGAG  632
            ||||||||||||||||||||||||||||||||||||||||||||||||||||||||||||
Sbjct  543  CAATATTGTTAACTGATCGAAACTTTAATACATCATTTTTTGATCCTTCAGGGGGAGGAG  602

Query  633  ATCCAATTTTATATCAACATTTATTT  658
            ||||||||||||||||||||||||||
Sbjct  603  ATCCAATTTTATATCAACATTTATTT  628
```

>Rhipicephalus sanguineus sensu lato 'temperate lineage' isolate CR1575 cytochrome c oxidase subunit I (COI) gene, partial cds; mitochondrial
Sequence ID: MF426002.1 Length: 639
Range 1: 1 to 628

Score:1155 bits(625), Expect:0.0,
Identities:627/628(99%), Gaps:0/628(0%), Strand: Plus/Plus

```
Query  31   AGGTATATTAGGACTTAGTATAAGAATATTAATTCGTATAGAATTAGGACAACCTGGAAC  90
            ||||||||||||||||||||||||||||||||||||||||||||||||||||||||||||
Sbjct  1    AGGTATATTAGGACTTAGTATAAGAATATTAATTCGTATAGAATTAGGACAACCTGGAAC  60

Query  91   TTTAATTGGAAATGATCAGATTTATAATGTAATCGTAACAGCACATGCATTTATTATAAt  150
            ||||||||||||||||||||||||||||||||||||||||||||||||||||||||||||
Sbjct  61   TTTAATTGGAAATGATCAGATTTATAATGTAATCGTAACAGCACATGCATTTATTATAAT  120

Query  151  ttttttATAGTTATACCAATCATAATCGGAGGATTTGGAAATTGATTAGTCCCTATCAT  210
            ||||||||||||||||||||||||||||||||||||||||||||||||||||||||||||
Sbjct  121  TTTTTTATAGTTATACCAATCATAATCGGAGGATTTGGAAATTGATTAGTCCCTATCAT  180

Query  211  ATTGGGTGCCCCAGATATAGCATTTCCACGAATAAATAATATAAGATTTTGACTTTTACC  270
            ||||||||||||||||||||||||||||||||||||||||||||||||||||||||||||
Sbjct  181  ATTGGGTGCCCCAGATATAGCATTTCCACGAATAAATAATATAAGATTTTGACTTTTACC  240

Query  271  TCCTTCACTATTTTTATTAATTAACTCTTCATTAATTGAATCTGGAGCTGGTACAGGATG  330
            ||||||||||||||||||||||||||||||||||||||||||||||||||||||||||||
Sbjct  241  TCCTTCACTATTTTTATTAATTAACTCTTCATTAATTGAATCTGGAGCTGGTACAGGATG  300

Query  331  AACAGTTTACCCTCCTCTATCTTCAAATTTATCACATTATGGCCCTTCAGTAGATTTAGC  390
            ||||||||||||||||||||||||||||||||||||||||||||||||||||||||||||
Sbjct  301  AACAGTTTACCCTCCTCTATCTTCAAATTTATCACATTATGGCCCTTCAGTAGATTTAGC  360

Query  391  TATTTTTTCTCTTCATCTTGCTGGTGCTTCTTCAATTTTAGGTGCAATTAACTTTATTAC  450
            ||| ||||||||||||||||||||||||||||||||||||||||||||||||||||||||
Sbjct  361  TATCTTTTCTCTTCATCTTGCTGGTGCTTCTTCAATTTTAGGTGCAATTAACTTTATTAC  420

Query  451  AACTATTGTAAACATACGATCAATTGGAATAACAATAGAACGTATACCATTATTTGTTTG  510
            ||||||||||||||||||||||||||||||||||||||||||||||||||||||||||||
Sbjct  421  AACTATTGTAAACATACGATCAATTGGAATAACAATAGAACGTATACCATTATTTGTTTG  480
```

```
Query  511  ATCTGTTTTAATTACAGCTATTTTATTACTTTTATCTTTACCTGTATTAGCAGGTGCCAT  570
            ||||||||||||||||||||||||||||||||||||||||||||||||||||||||||||
Sbjct  481  ATCTGTTTTAATTACAGCTATTTTATTACTTTTATCTTTACCTGTATTAGCAGGTGCCAT  540

Query  571  TACAATATTGTTAACTGATCGAAACTTTAATACATCATTTTTTGATCCTTCAGGGGGAGG  630
            ||||||||||||||||||||||||||||||||||||||||||||||||||||||||||||
Sbjct  541  TACAATATTGTTAACTGATCGAAACTTTAATACATCATTTTTTGATCCTTCAGGGGGAGG  600

Query  631  AGATCCAATTTTATATCAACATTTATTT  658
            ||||||||||||||||||||||||||||
Sbjct  601  AGATCCAATTTTATATCAACATTTATTT  628
```

>Rhipicephalus sanguineus isolate Rsan-ML4 cytochrome oxidase subunit I (cox1) gene, partial cds; mitochondrial
Sequence ID: MG855658.1 Length: 630
Range 1: 1 to 620

Score:1146 bits(620), Expect:0.0,
Identities:620/620(100%), Gaps:0/620(0%), Strand: Plus/Plus

```
Query  39   TAGGACTTAGTATAAGAATATTAATTCGTATAGAATTAGGACAACCTGGAACTTTAATTG  98
            ||||||||||||||||||||||||||||||||||||||||||||||||||||||||||||
Sbjct  1    TAGGACTTAGTATAAGAATATTAATTCGTATAGAATTAGGACAACCTGGAACTTTAATTG  60

Query  99   GAAATGATCAGATTTATAATGTAATCGTAACAGCACATGCATTTATTATAAtttttttA  158
            ||||||||||||||||||||||||||||||||||||||||||||||||||||||||||||
Sbjct  61   GAAATGATCAGATTTATAATGTAATCGTAACAGCACATGCATTTATTATAATTTTTTTTA  120

Query  159  TAGTTATACCAATCATAATCGGAGGATTTGGAAATTGATTAGTCCCTATCATATTGGGTG  218
            ||||||||||||||||||||||||||||||||||||||||||||||||||||||||||||
Sbjct  121  TAGTTATACCAATCATAATCGGAGGATTTGGAAATTGATTAGTCCCTATCATATTGGGTG  180

Query  219  CCCCAGATATAGCATTTCCACGAATAAATAATATAAGATTTTGACTTTTACCTCCTTCAC  278
            ||||||||||||||||||||||||||||||||||||||||||||||||||||||||||||
Sbjct  181  CCCCAGATATAGCATTTCCACGAATAAATAATATAAGATTTTGACTTTTACCTCCTTCAC  240

Query  279  TATTTTTATTAATTAACTCTTCATTAATTGAATCTGGAGCTGGTACAGGATGAACAGTTT  338
            ||||||||||||||||||||||||||||||||||||||||||||||||||||||||||||
Sbjct  241  TATTTTTATTAATTAACTCTTCATTAATTGAATCTGGAGCTGGTACAGGATGAACAGTTT  300

Query  339  ACCCTCCTCTATCTTCAAATTTATCACATTATGGCCCTTCAGTAGATTTAGCTATTTTTT  398
            ||||||||||||||||||||||||||||||||||||||||||||||||||||||||||||
Sbjct  301  ACCCTCCTCTATCTTCAAATTTATCACATTATGGCCCTTCAGTAGATTTAGCTATTTTTT  360

Query  399  CTCTTCATCTTGCTGGTGCTTCTTCAATTTTAGGTGCAATTAACTTTATTACAACTATTG  458
            ||||||||||||||||||||||||||||||||||||||||||||||||||||||||||||
Sbjct  361  CTCTTCATCTTGCTGGTGCTTCTTCAATTTTAGGTGCAATTAACTTTATTACAACTATTG  420

Query  459  TAAACATACGATCAATTGGAATAACAATAGAACGTATACCATTATTTGTTTGATCTGTTT  518
            ||||||||||||||||||||||||||||||||||||||||||||||||||||||||||||
```

```
Sbjct  421   TAAACATACGATCAATTGGAATAACAATAGAACGTATACCATTATTTGTTTGATCTGTTT  480

Query  519   TAATTACAGCTATTTTATTACTTTTATCTTTACCTGTATTAGCAGGTGCCATTACAATAT  578
             ||||||||||||||||||||||||||||||||||||||||||||||||||||||||||||
Sbjct  481   TAATTACAGCTATTTTATTACTTTTATCTTTACCTGTATTAGCAGGTGCCATTACAATAT  540

Query  579   TGTTAACTGATCGAAACTTTAATACATCATTTTTTGATCCTTCAGGGGGAGGAGATCCAA  638
             ||||||||||||||||||||||||||||||||||||||||||||||||||||||||||||
Sbjct  541   TGTTAACTGATCGAAACTTTAATACATCATTTTTTGATCCTTCAGGGGGAGGAGATCCAA  600

Query  639   TTTTATATCAACATTTATTT  658
             ||||||||||||||||||||
Sbjct  601   TTTTATATCAACATTTATTT  620
```

>Rhipicephalus sanguineus isolate Rsan-ML3 cytochrome oxidase subunit I (cox1) gene, partial cds; mitochondrial
Sequence ID: MG855657.1 Length: 630
Range 1: 1 to 620

Score:1146 bits(620), Expect:0.0,
Identities:620/620(100%), Gaps:0/620(0%), Strand: Plus/Plus

```
Query  39    TAGGACTTAGTATAAGAATATTAATTCGTATAGAATTAGGACAACCTGGAACTTTAATTG  98
             ||||||||||||||||||||||||||||||||||||||||||||||||||||||||||||
Sbjct  1     TAGGACTTAGTATAAGAATATTAATTCGTATAGAATTAGGACAACCTGGAACTTTAATTG  60

Query  99    GAAATGATCAGATTTATAATGTAATCGTAACAGCACATGCATTTATTATAAtttttttA  158
             ||||||||||||||||||||||||||||||||||||||||||||||||||||||||||||
Sbjct  61    GAAATGATCAGATTTATAATGTAATCGTAACAGCACATGCATTTATTATAATTTTTTTTA  120

Query  159   TAGTTATACCAATCATAATCGGAGGATTTGGAAATTGATTAGTCCCTATCATATTGGGTG  218
             ||||||||||||||||||||||||||||||||||||||||||||||||||||||||||||
Sbjct  121   TAGTTATACCAATCATAATCGGAGGATTTGGAAATTGATTAGTCCCTATCATATTGGGTG  180

Query  219   CCCCAGATATAGCATTTCCACGAATAAATAATATAAGATTTTGACTTTTACCTCCTTCAC  278
             ||||||||||||||||||||||||||||||||||||||||||||||||||||||||||||
Sbjct  181   CCCCAGATATAGCATTTCCACGAATAAATAATATAAGATTTTGACTTTTACCTCCTTCAC  240

Query  279   TATTTTTATTAATTAACTCTTCATTAATTGAATCTGGAGCTGGTACAGGATGAACAGTTT  338
             ||||||||||||||||||||||||||||||||||||||||||||||||||||||||||||
Sbjct  241   TATTTTTATTAATTAACTCTTCATTAATTGAATCTGGAGCTGGTACAGGATGAACAGTTT  300

Query  339   ACCCTCCTCTATCTTCAAATTTATCACATTATGGCCCTTCAGTAGATTTAGCTATTTTTT  398
             ||||||||||||||||||||||||||||||||||||||||||||||||||||||||||||
Sbjct  301   ACCCTCCTCTATCTTCAAATTTATCACATTATGGCCCTTCAGTAGATTTAGCTATTTTTT  360

Query  399   CTCTTCATCTTGCTGGTGCTTCTTCAATTTTAGGTGCAATTAACTTTATTACAACTATTG  458
             ||||||||||||||||||||||||||||||||||||||||||||||||||||||||||||
Sbjct  361   CTCTTCATCTTGCTGGTGCTTCTTCAATTTTAGGTGCAATTAACTTTATTACAACTATTG  420

Query  459   TAAACATACGATCAATTGGAATAACAATAGAACGTATACCATTATTTGTTTGATCTGTTT  518
```

```
                |||||||||||||||||||||||||||||||||||||||||||||||||||||||||||||
Sbjct  421  TAAACATACGATCAATTGGAATAACAATAGAACGTATACCATTATTTGTTTGATCTGTTT  480

Query  519  TAATTACAGCTATTTTATTACTTTTATCTTTACCTGTATTAGCAGGTGCCATTACAATAT  578
            |||||||||||||||||||||||||||||||||||||||||||||||||||||||||||||
Sbjct  481  TAATTACAGCTATTTTATTACTTTTATCTTTACCTGTATTAGCAGGTGCCATTACAATAT  540

Query  579  TGTTAACTGATCGAAACTTTAATACATCATTTTTTGATCCTTCAGGGGGAGGAGATCCAA  638
            |||||||||||||||||||||||||||||||||||||||||||||||||||||||||||||
Sbjct  541  TGTTAACTGATCGAAACTTTAATACATCATTTTTTGATCCTTCAGGGGGAGGAGATCCAA  600

Query  639  TTTTATATCAACATTTATTT  658
            ||||||||||||||||||||
Sbjct  601  TTTTATATCAACATTTATTT  620
```

>Rhipicephalus sanguineus isolate Rsan-ML2 cytochrome oxidase subunit I (cox1) gene, partial cds; mitochondrial
Sequence ID: MG855656.1 Length: 630
Range 1: 1 to 620

Score:1146 bits(620), Expect:0.0,
Identities:620/620(100%), Gaps:0/620(0%), Strand: Plus/Plus

```
Query  39   TAGGACTTAGTATAAGAATATTAATTCGTATAGAATTAGGACAACCTGGAACTTTAATTG  98
            |||||||||||||||||||||||||||||||||||||||||||||||||||||||||||||
Sbjct  1    TAGGACTTAGTATAAGAATATTAATTCGTATAGAATTAGGACAACCTGGAACTTTAATTG  60

Query  99   GAAATGATCAGATTTATAATGTAATCGTAACAGCACATGCATTTATTATAAttttttttA  158
            |||||||||||||||||||||||||||||||||||||||||||||||||||||||||||||
Sbjct  61   GAAATGATCAGATTTATAATGTAATCGTAACAGCACATGCATTTATTATAATTTTTTTTA  120

Query  159  TAGTTATACCAATCATAATCGGAGGATTTGGAAATTGATTAGTCCCTATCATATTGGGTG  218
            |||||||||||||||||||||||||||||||||||||||||||||||||||||||||||||
Sbjct  121  TAGTTATACCAATCATAATCGGAGGATTTGGAAATTGATTAGTCCCTATCATATTGGGTG  180

Query  219  CCCCAGATATAGCATTTCCACGAATAAATAATATAAGATTTTGACTTTTACCTCCTTCAC  278
            |||||||||||||||||||||||||||||||||||||||||||||||||||||||||||||
Sbjct  181  CCCCAGATATAGCATTTCCACGAATAAATAATATAAGATTTTGACTTTTACCTCCTTCAC  240

Query  279  TATTTTTATTAATTAACTCTTCATTAATTGAATCTGGAGCTGGTACAGGATGAACAGTTT  338
            |||||||||||||||||||||||||||||||||||||||||||||||||||||||||||||
Sbjct  241  TATTTTTATTAATTAACTCTTCATTAATTGAATCTGGAGCTGGTACAGGATGAACAGTTT  300

Query  339  ACCCTCCTCTATCTTCAAATTTATCACATTATGGCCCTTCAGTAGATTTAGCTATTTTTT  398
            |||||||||||||||||||||||||||||||||||||||||||||||||||||||||||||
Sbjct  301  ACCCTCCTCTATCTTCAAATTTATCACATTATGGCCCTTCAGTAGATTTAGCTATTTTTT  360

Query  399  CTCTTCATCTTGCTGGTGCTTCTTCAATTTTAGGTGCAATTAACTTTATTACAACTATTG  458
            |||||||||||||||||||||||||||||||||||||||||||||||||||||||||||||
Sbjct  361  CTCTTCATCTTGCTGGTGCTTCTTCAATTTTAGGTGCAATTAACTTTATTACAACTATTG  420
```

```
Query  459   TAAACATACGATCAATTGGAATAACAATAGAACGTATACCATTATTTGTTTGATCTGTTT  518
             ||||||||||||||||||||||||||||||||||||||||||||||||||||||||||||
Sbjct  421   TAAACATACGATCAATTGGAATAACAATAGAACGTATACCATTATTTGTTTGATCTGTTT  480

Query  519   TAATTACAGCTATTTTATTACTTTTATCTTTACCTGTATTAGCAGGTGCCATTACAATAT  578
             ||||||||||||||||||||||||||||||||||||||||||||||||||||||||||||
Sbjct  481   TAATTACAGCTATTTTATTACTTTTATCTTTACCTGTATTAGCAGGTGCCATTACAATAT  540

Query  579   TGTTAACTGATCGAAACTTTAATACATCATTTTTTGATCCTTCAGGGGGAGGAGATCCAA  638
             ||||||||||||||||||||||||||||||||||||||||||||||||||||||||||||
Sbjct  541   TGTTAACTGATCGAAACTTTAATACATCATTTTTTGATCCTTCAGGGGGAGGAGATCCAA  600

Query  639   TTTTATATCAACATTTATTT  658
             ||||||||||||||||||||
Sbjct  601   TTTTATATCAACATTTATTT  620
```

>Rhipicephalus sanguineus isolate Kehf-Lagareb-A cytochrome oxidase subunit I (COI) gene, partial cds; mitochondrial
Sequence ID: KX757910.1 Length: 630
Range 1: 1 to 620

Score:1146 bits(620), Expect:0.0,
Identities:620/620(100%), Gaps:0/620(0%), Strand: Plus/Plus

```
Query  39    TAGGACTTAGTATAAGAATATTAATTCGTATAGAATTAGGACAACCTGGAACTTTAATTG  98
             ||||||||||||||||||||||||||||||||||||||||||||||||||||||||||||
Sbjct  1     TAGGACTTAGTATAAGAATATTAATTCGTATAGAATTAGGACAACCTGGAACTTTAATTG  60

Query  99    GAAATGATCAGATTTATAATGTAATCGTAACAGCACATGCATTTATTATAAtttttttA  158
             ||||||||||||||||||||||||||||||||||||||||||||||||||||||||||||
Sbjct  61    GAAATGATCAGATTTATAATGTAATCGTAACAGCACATGCATTTATTATAATTTTTTTTA  120

Query  159   TAGTTATACCAATCATAATCGGAGGATTTGGAAATTGATTAGTCCCTATCATATTGGGTG  218
             ||||||||||||||||||||||||||||||||||||||||||||||||||||||||||||
Sbjct  121   TAGTTATACCAATCATAATCGGAGGATTTGGAAATTGATTAGTCCCTATCATATTGGGTG  180

Query  219   CCCCAGATATAGCATTTCCACGAATAAATAATATAAGATTTTGACTTTTACCTCCTTCAC  278
             ||||||||||||||||||||||||||||||||||||||||||||||||||||||||||||
Sbjct  181   CCCCAGATATAGCATTTCCACGAATAAATAATATAAGATTTTGACTTTTACCTCCTTCAC  240

Query  279   TATTTTTATTAATTAACTCTTCATTAATTGAATCTGGAGCTGGTACAGGATGAACAGTTT  338
             ||||||||||||||||||||||||||||||||||||||||||||||||||||||||||||
Sbjct  241   TATTTTTATTAATTAACTCTTCATTAATTGAATCTGGAGCTGGTACAGGATGAACAGTTT  300

Query  339   ACCCTCCTCTATCTTCAAATTTATCACATTATGGCCCTTCAGTAGATTTAGCTATTTTTT  398
             ||||||||||||||||||||||||||||||||||||||||||||||||||||||||||||
Sbjct  301   ACCCTCCTCTATCTTCAAATTTATCACATTATGGCCCTTCAGTAGATTTAGCTATTTTTT  360

Query  399   CTCTTCATCTTGCTGGTGCTTCTTCAATTTTAGGTGCAATTAACTTTATTACAACTATTG  458
             ||||||||||||||||||||||||||||||||||||||||||||||||||||||||||||
Sbjct  361   CTCTTCATCTTGCTGGTGCTTCTTCAATTTTAGGTGCAATTAACTTTATTACAACTATTG  420
```

```
Query  459   TAAACATACGATCAATTGGAATAACAATAGAACGTATACCATTATTTGTTTGATCTGTTT  518
             ||||||||||||||||||||||||||||||||||||||||||||||||||||||||||||
Sbjct  421   TAAACATACGATCAATTGGAATAACAATAGAACGTATACCATTATTTGTTTGATCTGTTT  480

Query  519   TAATTACAGCTATTTTATTACTTTTATCTTTACCTGTATTAGCAGGTGCCATTACAATAT  578
             ||||||||||||||||||||||||||||||||||||||||||||||||||||||||||||
Sbjct  481   TAATTACAGCTATTTTATTACTTTTATCTTTACCTGTATTAGCAGGTGCCATTACAATAT  540

Query  579   TGTTAACTGATCGAAACTTTAATACATCATTTTTTGATCCTTCAGGGGGAGGAGATCCAA  638
             ||||||||||||||||||||||||||||||||||||||||||||||||||||||||||||
Sbjct  541   TGTTAACTGATCGAAACTTTAATACATCATTTTTTGATCCTTCAGGGGGAGGAGATCCAA  600

Query  639   TTTTATATCAACATTTATTT  658
             ||||||||||||||||||||
Sbjct  601   TTTTATATCAACATTTATTT  620
```

>Rhipicephalus sanguineus isolate Italy-B cytochrome oxidase subunit I (COI) gene, partial cds; mitochondrial
Sequence ID: KX757904.1 Length: 630
Range 1: 1 to 620

Score:1146 bits(620), Expect:0.0,
Identities:620/620(100%), Gaps:0/620(0%), Strand: Plus/Plus

```
Query  39    TAGGACTTAGTATAAGAATATTAATTCGTATAGAATTAGGACAACCTGGAACTTTAATTG  98
             ||||||||||||||||||||||||||||||||||||||||||||||||||||||||||||
Sbjct  1     TAGGACTTAGTATAAGAATATTAATTCGTATAGAATTAGGACAACCTGGAACTTTAATTG  60

Query  99    GAAATGATCAGATTTATAATGTAATCGTAACAGCACATGCATTTATTATAAttttttttA  158
             ||||||||||||||||||||||||||||||||||||||||||||||||||||||||||||
Sbjct  61    GAAATGATCAGATTTATAATGTAATCGTAACAGCACATGCATTTATTATAATTTTTTTTA  120

Query  159   TAGTTATACCAATCATAATCGGAGGATTTGGAAATTGATTAGTCCCTATCATATTGGGTG  218
             ||||||||||||||||||||||||||||||||||||||||||||||||||||||||||||
Sbjct  121   TAGTTATACCAATCATAATCGGAGGATTTGGAAATTGATTAGTCCCTATCATATTGGGTG  180

Query  219   CCCCAGATATAGCATTTCCACGAATAAATAATATAAGATTTTGACTTTTACCTCCTTCAC  278
             ||||||||||||||||||||||||||||||||||||||||||||||||||||||||||||
Sbjct  181   CCCCAGATATAGCATTTCCACGAATAAATAATATAAGATTTTGACTTTTACCTCCTTCAC  240

Query  279   TATTTTTATTAATTAACTCTTCATTAATTGAATCTGGAGCTGGTACAGGATGAACAGTTT  338
             ||||||||||||||||||||||||||||||||||||||||||||||||||||||||||||
Sbjct  241   TATTTTTATTAATTAACTCTTCATTAATTGAATCTGGAGCTGGTACAGGATGAACAGTTT  300

Query  339   ACCCTCCTCTATCTTCAAATTTATCACATTATGGCCCTTCAGTAGATTTAGCTATTTTTT  398
             ||||||||||||||||||||||||||||||||||||||||||||||||||||||||||||
Sbjct  301   ACCCTCCTCTATCTTCAAATTTATCACATTATGGCCCTTCAGTAGATTTAGCTATTTTTT  360

Query  399   CTCTTCATCTTGCTGGTGCTTCTTCAATTTTAGGTGCAATTAACTTTATTACAACTATTG  458
             ||||||||||||||||||||||||||||||||||||||||||||||||||||||||||||
```

```
Sbjct  361   CTCTTCATCTTGCTGGTGCTTCTTCAATTTTAGGTGCAATTAACTTTATTACAACTATTG  420

Query  459   TAAACATACGATCAATTGGAATAACAATAGAACGTATACCATTATTTGTTTGATCTGTTT  518
             ||||||||||||||||||||||||||||||||||||||||||||||||||||||||||||
Sbjct  421   TAAACATACGATCAATTGGAATAACAATAGAACGTATACCATTATTTGTTTGATCTGTTT  480

Query  519   TAATTACAGCTATTTTATTACTTTTATCTTTACCTGTATTAGCAGGTGCCATTACAATAT  578
             ||||||||||||||||||||||||||||||||||||||||||||||||||||||||||||
Sbjct  481   TAATTACAGCTATTTTATTACTTTTATCTTTACCTGTATTAGCAGGTGCCATTACAATAT  540

Query  579   TGTTAACTGATCGAAACTTTAATACATCATTTTTTGATCCTTCAGGGGGAGGAGATCCAA  638
             ||||||||||||||||||||||||||||||||||||||||||||||||||||||||||||
Sbjct  541   TGTTAACTGATCGAAACTTTAATACATCATTTTTTGATCCTTCAGGGGGAGGAGATCCAA  600

Query  639   TTTTATATCAACATTTATTT  658
             ||||||||||||||||||||
Sbjct  601   TTTTATATCAACATTTATTT  620
```

>Rhipicephalus sanguineus isolate Malta-A cytochrome oxidase subunit I (COI) gene, partial cds; mitochondrial
Sequence ID: KX757902.1 Length: 630
Range 1: 1 to 620

Score:1146 bits(620), Expect:0.0,
Identities:620/620(100%), Gaps:0/620(0%), Strand: Plus/Plus

```
Query  39    TAGGACTTAGTATAAGAATATTAATTCGTATAGAATTAGGACAACCTGGAACTTTAATTG  98
             ||||||||||||||||||||||||||||||||||||||||||||||||||||||||||||
Sbjct  1     TAGGACTTAGTATAAGAATATTAATTCGTATAGAATTAGGACAACCTGGAACTTTAATTG  60

Query  99    GAAATGATCAGATTTATAATGTAATCGTAACAGCACATGCATTTATTATAAtttttttA  158
             ||||||||||||||||||||||||||||||||||||||||||||||||||||||||||||
Sbjct  61    GAAATGATCAGATTTATAATGTAATCGTAACAGCACATGCATTTATTATAATTTTTTTA  120

Query  159   TAGTTATACCAATCATAATCGGAGGATTTGGAAATTGATTAGTCCCTATCATATTGGGTG  218
             ||||||||||||||||||||||||||||||||||||||||||||||||||||||||||||
Sbjct  121   TAGTTATACCAATCATAATCGGAGGATTTGGAAATTGATTAGTCCCTATCATATTGGGTG  180

Query  219   CCCCAGATATAGCATTTCCACGAATAAATAATATAAGATTTTGACTTTTACCTCCTTCAC  278
             ||||||||||||||||||||||||||||||||||||||||||||||||||||||||||||
Sbjct  181   CCCCAGATATAGCATTTCCACGAATAAATAATATAAGATTTTGACTTTTACCTCCTTCAC  240

Query  279   TATTTTTATTAATTAACTCTTCATTAATTGAATCTGGAGCTGGTACAGGATGAACAGTTT  338
             ||||||||||||||||||||||||||||||||||||||||||||||||||||||||||||
Sbjct  241   TATTTTTATTAATTAACTCTTCATTAATTGAATCTGGAGCTGGTACAGGATGAACAGTTT  300

Query  339   ACCCTCCTCTATCTTCAAATTTATCACATTATGGCCCTTCAGTAGATTTAGCTATTTTTT  398
             ||||||||||||||||||||||||||||||||||||||||||||||||||||||||||||
Sbjct  301   ACCCTCCTCTATCTTCAAATTTATCACATTATGGCCCTTCAGTAGATTTAGCTATTTTTT  360

Query  399   CTCTTCATCTTGCTGGTGCTTCTTCAATTTTAGGTGCAATTAACTTTATTACAACTATTG  458
```

```
          ||||||||||||||||||||||||||||||||||||||||||||||||||||||||||||
Sbjct  361  CTCTTCATCTTGCTGGTGCTTCTTCAATTTTAGGTGCAATTAACTTTATTACAACTATTG  420

Query  459  TAAACATACGATCAATTGGAATAACAATAGAACGTATACCATTATTTGTTTGATCTGTTT  518
            ||||||||||||||||||||||||||||||||||||||||||||||||||||||||||||
Sbjct  421  TAAACATACGATCAATTGGAATAACAATAGAACGTATACCATTATTTGTTTGATCTGTTT  480

Query  519  TAATTACAGCTATTTTATTACTTTTATCTTTACCTGTATTAGCAGGTGCCATTACAATAT  578
            ||||||||||||||||||||||||||||||||||||||||||||||||||||||||||||
Sbjct  481  TAATTACAGCTATTTTATTACTTTTATCTTTACCTGTATTAGCAGGTGCCATTACAATAT  540

Query  579  TGTTAACTGATCGAAACTTTAATACATCATTTTTTGATCCTTCAGGGGGAGGAGATCCAA  638
            ||||||||||||||||||||||||||||||||||||||||||||||||||||||||||||
Sbjct  541  TGTTAACTGATCGAAACTTTAATACATCATTTTTTGATCCTTCAGGGGGAGGAGATCCAA  600

Query  639  TTTTATATCAACATTTATTT  658
            ||||||||||||||||||||
Sbjct  601  TTTTATATCAACATTTATTT  620
```

>Rhipicephalus sanguineus isolate Zagreb-C cytochrome oxidase subunit I (COI) gene, partial cds; mitochondrial
Sequence ID: KX757896.1 Length: 630
Range 1: 1 to 620

Score:1146 bits(620), Expect:0.0,
Identities:620/620(100%), Gaps:0/620(0%), Strand: Plus/Plus

```
Query  39   TAGGACTTAGTATAAGAATATTAATTCGTATAGAATTAGGACAACCTGGAACTTTAATTG  98
            ||||||||||||||||||||||||||||||||||||||||||||||||||||||||||||
Sbjct  1    TAGGACTTAGTATAAGAATATTAATTCGTATAGAATTAGGACAACCTGGAACTTTAATTG  60

Query  99   GAAATGATCAGATTTATAATGTAATCGTAACAGCACATGCATTTATTATAAttttttttA  158
            ||||||||||||||||||||||||||||||||||||||||||||||||||||||||||||
Sbjct  61   GAAATGATCAGATTTATAATGTAATCGTAACAGCACATGCATTTATTATAATTTTTTTTA  120

Query  159  TAGTTATACCAATCATAATCGGAGGATTTGGAAATTGATTAGTCCCTATCATATTGGGTG  218
            ||||||||||||||||||||||||||||||||||||||||||||||||||||||||||||
Sbjct  121  TAGTTATACCAATCATAATCGGAGGATTTGGAAATTGATTAGTCCCTATCATATTGGGTG  180

Query  219  CCCCAGATATAGCATTTCCACGAATAAATAATATAAGATTTTGACTTTTACCTCCTTCAC  278
            ||||||||||||||||||||||||||||||||||||||||||||||||||||||||||||
Sbjct  181  CCCCAGATATAGCATTTCCACGAATAAATAATATAAGATTTTGACTTTTACCTCCTTCAC  240

Query  279  TATTTTTATTAATTAACTCTTCATTAATTGAATCTGGAGCTGGTACAGGATGAACAGTTT  338
            ||||||||||||||||||||||||||||||||||||||||||||||||||||||||||||
Sbjct  241  TATTTTTATTAATTAACTCTTCATTAATTGAATCTGGAGCTGGTACAGGATGAACAGTTT  300

Query  339  ACCCTCCTCTATCTTCAAATTTATCACATTATGGCCCTTCAGTAGATTTAGCTATTTTTT  398
            ||||||||||||||||||||||||||||||||||||||||||||||||||||||||||||
Sbjct  301  ACCCTCCTCTATCTTCAAATTTATCACATTATGGCCCTTCAGTAGATTTAGCTATTTTTT  360
```

```
Query  399   CTCTTCATCTTGCTGGTGCTTCTTCAATTTTAGGTGCAATTAACTTTATTACAACTATTG  458
             ||||||||||||||||||||||||||||||||||||||||||||||||||||||||||||
Sbjct  361   CTCTTCATCTTGCTGGTGCTTCTTCAATTTTAGGTGCAATTAACTTTATTACAACTATTG  420

Query  459   TAAACATACGATCAATTGGAATAACAATAGAACGTATACCATTATTTGTTTGATCTGTTT  518
             ||||||||||||||||||||||||||||||||||||||||||||||||||||||||||||
Sbjct  421   TAAACATACGATCAATTGGAATAACAATAGAACGTATACCATTATTTGTTTGATCTGTTT  480

Query  519   TAATTACAGCTATTTTATTACTTTTATCTTTACCTGTATTAGCAGGTGCCATTACAATAT  578
             ||||||||||||||||||||||||||||||||||||||||||||||||||||||||||||
Sbjct  481   TAATTACAGCTATTTTATTACTTTTATCTTTACCTGTATTAGCAGGTGCCATTACAATAT  540

Query  579   TGTTAACTGATCGAAACTTTAATACATCATTTTTTGATCCTTCAGGGGGAGGAGATCCAA  638
             ||||||||||||||||||||||||||||||||||||||||||||||||||||||||||||
Sbjct  541   TGTTAACTGATCGAAACTTTAATACATCATTTTTTGATCCTTCAGGGGGAGGAGATCCAA  600

Query  639   TTTTATATCAACATTTATTT  658
             ||||||||||||||||||||
Sbjct  601   TTTTATATCAACATTTATTT  620



>Rhipicephalus sanguineus isolate Pula-B cytochrome oxidase subunit I (COI)
gene, partial cds; mitochondrial
Sequence ID: KX757889.1 Length: 630
Range 1: 1 to 620

Score:1146 bits(620), Expect:0.0,
Identities:620/620(100%),  Gaps:0/620(0%), Strand: Plus/Plus

Query  39    TAGGACTTAGTATAAGAATATTAATTCGTATAGAATTAGGACAACCTGGAACTTTAATTG  98
             ||||||||||||||||||||||||||||||||||||||||||||||||||||||||||||
Sbjct  1     TAGGACTTAGTATAAGAATATTAATTCGTATAGAATTAGGACAACCTGGAACTTTAATTG  60

Query  99    GAAATGATCAGATTTATAATGTAATCGTAACAGCACATGCATTTATTATAAtttttttA  158
             ||||||||||||||||||||||||||||||||||||||||||||||||||||||||||||
Sbjct  61    GAAATGATCAGATTTATAATGTAATCGTAACAGCACATGCATTTATTATAATTTTTTTTA  120

Query  159   TAGTTATACCAATCATAATCGGAGGATTTGGAAATTGATTAGTCCCTATCATATTGGGTG  218
             ||||||||||||||||||||||||||||||||||||||||||||||||||||||||||||
Sbjct  121   TAGTTATACCAATCATAATCGGAGGATTTGGAAATTGATTAGTCCCTATCATATTGGGTG  180

Query  219   CCCCAGATATAGCATTTCCACGAATAAATAATATAAGATTTTGACTTTTACCTCCTTCAC  278
             ||||||||||||||||||||||||||||||||||||||||||||||||||||||||||||
Sbjct  181   CCCCAGATATAGCATTTCCACGAATAAATAATATAAGATTTTGACTTTTACCTCCTTCAC  240

Query  279   TATTTTTATTAATTAACTCTTCATTAATTGAATCTGGAGCTGGTACAGGATGAACAGTTT  338
             ||||||||||||||||||||||||||||||||||||||||||||||||||||||||||||
Sbjct  241   TATTTTTATTAATTAACTCTTCATTAATTGAATCTGGAGCTGGTACAGGATGAACAGTTT  300

Query  339   ACCCTCCTCTATCTTCAAATTTATCACATTATGGCCCTTCAGTAGATTTAGCTATTTTTT  398
             ||||||||||||||||||||||||||||||||||||||||||||||||||||||||||||
Sbjct  301   ACCCTCCTCTATCTTCAAATTTATCACATTATGGCCCTTCAGTAGATTTAGCTATTTTTT  360
```

```
Query  399   CTCTTCATCTTGCTGGTGCTTCTTCAATTTTAGGTGCAATTAACTTTATTACAACTATTG  458
             ||||||||||||||||||||||||||||||||||||||||||||||||||||||||||||
Sbjct  361   CTCTTCATCTTGCTGGTGCTTCTTCAATTTTAGGTGCAATTAACTTTATTACAACTATTG  420

Query  459   TAAACATACGATCAATTGGAATAACAATAGAACGTATACCATTATTTGTTTGATCTGTTT  518
             ||||||||||||||||||||||||||||||||||||||||||||||||||||||||||||
Sbjct  421   TAAACATACGATCAATTGGAATAACAATAGAACGTATACCATTATTTGTTTGATCTGTTT  480

Query  519   TAATTACAGCTATTTTATTACTTTTATCTTTACCTGTATTAGCAGGTGCCATTACAATAT  578
             ||||||||||||||||||||||||||||||||||||||||||||||||||||||||||||
Sbjct  481   TAATTACAGCTATTTTATTACTTTTATCTTTACCTGTATTAGCAGGTGCCATTACAATAT  540

Query  579   TGTTAACTGATCGAAACTTTAATACATCATTTTTTGATCCTTCAGGGGGAGGAGATCCAA  638
             ||||||||||||||||||||||||||||||||||||||||||||||||||||||||||||
Sbjct  541   TGTTAACTGATCGAAACTTTAATACATCATTTTTTGATCCTTCAGGGGGAGGAGATCCAA  600

Query  639   TTTTATATCAACATTTATTT  658
             ||||||||||||||||||||
Sbjct  601   TTTTATATCAACATTTATTT  620
```

>Rhipicephalus sanguineus isolate Rovind cytochrome oxidase subunit I (COI) gene, partial cds; mitochondrial
Sequence ID: KX757887.1 Length: 630
Range 1: 1 to 620

Score:1146 bits(620), Expect:0.0,
Identities:620/620(100%), Gaps:0/620(0%), Strand: Plus/Plus

```
Query  39    TAGGACTTAGTATAAGAATATTAATTCGTATAGAATTAGGACAACCTGGAACTTTAATTG  98
             ||||||||||||||||||||||||||||||||||||||||||||||||||||||||||||
Sbjct  1     TAGGACTTAGTATAAGAATATTAATTCGTATAGAATTAGGACAACCTGGAACTTTAATTG  60

Query  99    GAAATGATCAGATTTATAATGTAATCGTAACAGCACATGCATTTATTATAAtttttttA  158
             ||||||||||||||||||||||||||||||||||||||||||||||||||||||||||||
Sbjct  61    GAAATGATCAGATTTATAATGTAATCGTAACAGCACATGCATTTATTATAATTTTTTTA  120

Query  159   TAGTTATACCAATCATAATCGGAGGATTTGGAAATTGATTAGTCCCTATCATATTGGGTG  218
             ||||||||||||||||||||||||||||||||||||||||||||||||||||||||||||
Sbjct  121   TAGTTATACCAATCATAATCGGAGGATTTGGAAATTGATTAGTCCCTATCATATTGGGTG  180

Query  219   CCCCAGATATAGCATTTCCACGAATAAATAATATAAGATTTTGACTTTTACCTCCTTCAC  278
             ||||||||||||||||||||||||||||||||||||||||||||||||||||||||||||
Sbjct  181   CCCCAGATATAGCATTTCCACGAATAAATAATATAAGATTTTGACTTTTACCTCCTTCAC  240

Query  279   TATTTTTATTAATTAACTCTTCATTAATTGAATCTGGAGCTGGTACAGGATGAACAGTTT  338
             ||||||||||||||||||||||||||||||||||||||||||||||||||||||||||||
Sbjct  241   TATTTTTATTAATTAACTCTTCATTAATTGAATCTGGAGCTGGTACAGGATGAACAGTTT  300

Query  339   ACCCTCCTCTATCTTCAAATTTATCACATTATGGCCCTTCAGTAGATTTAGCTATTTTTT  398
             ||||||||||||||||||||||||||||||||||||||||||||||||||||||||||||
```

```
Sbjct  301   ACCCTCCTCTATCTTCAAATTTATCACATTATGGCCCTTCAGTAGATTTAGCTATTTTTT  360

Query  399   CTCTTCATCTTGCTGGTGCTTCTTCAATTTTAGGTGCAATTAACTTTATTACAACTATTG  458
             ||||||||||||||||||||||||||||||||||||||||||||||||||||||||||||
Sbjct  361   CTCTTCATCTTGCTGGTGCTTCTTCAATTTTAGGTGCAATTAACTTTATTACAACTATTG  420

Query  459   TAAACATACGATCAATTGGAATAACAATAGAACGTATACCATTATTTGTTTGATCTGTTT  518
             ||||||||||||||||||||||||||||||||||||||||||||||||||||||||||||
Sbjct  421   TAAACATACGATCAATTGGAATAACAATAGAACGTATACCATTATTTGTTTGATCTGTTT  480

Query  519   TAATTACAGCTATTTTATTACTTTTATCTTTACCTGTATTAGCAGGTGCCATTACAATAT  578
             ||||||||||||||||||||||||||||||||||||||||||||||||||||||||||||
Sbjct  481   TAATTACAGCTATTTTATTACTTTTATCTTTACCTGTATTAGCAGGTGCCATTACAATAT  540

Query  579   TGTTAACTGATCGAAACTTTAATACATCATTTTTTGATCCTTCAGGGGGAGGAGATCCAA  638
             ||||||||||||||||||||||||||||||||||||||||||||||||||||||||||||
Sbjct  541   TGTTAACTGATCGAAACTTTAATACATCATTTTTTGATCCTTCAGGGGGAGGAGATCCAA  600

Query  639   TTTTATATCAACATTTATTT  658
             ||||||||||||||||||||
Sbjct  601   TTTTATATCAACATTTATTT  620
```

>Rhipicephalus sanguineus isolate Kajtasovo cytochrome oxidase subunit I (COI) gene, partial cds; mitochondrial
Sequence ID: KX757879.1 Length: 630
Range 1: 1 to 620

Score:1146 bits(620), Expect:0.0,
Identities:620/620(100%), Gaps:0/620(0%), Strand: Plus/Plus

```
Query  39    TAGGACTTAGTATAAGAATATTAATTCGTATAGAATTAGGACAACCTGGAACTTTAATTG  98
             ||||||||||||||||||||||||||||||||||||||||||||||||||||||||||||
Sbjct  1     TAGGACTTAGTATAAGAATATTAATTCGTATAGAATTAGGACAACCTGGAACTTTAATTG  60

Query  99    GAAATGATCAGATTTATAATGTAATCGTAACAGCACATGCATTTATTATAAtttttttA  158
             ||||||||||||||||||||||||||||||||||||||||||||||||||||||||||||
Sbjct  61    GAAATGATCAGATTTATAATGTAATCGTAACAGCACATGCATTTATTATAATTTTTTTA  120

Query  159   TAGTTATACCAATCATAATCGGAGGATTTGGAAATTGATTAGTCCCTATCATATTGGGTG  218
             ||||||||||||||||||||||||||||||||||||||||||||||||||||||||||||
Sbjct  121   TAGTTATACCAATCATAATCGGAGGATTTGGAAATTGATTAGTCCCTATCATATTGGGTG  180

Query  219   CCCCAGATATAGCATTTCCACGAATAAATAATATAAGATTTTGACTTTTACCTCCTTCAC  278
             ||||||||||||||||||||||||||||||||||||||||||||||||||||||||||||
Sbjct  181   CCCCAGATATAGCATTTCCACGAATAAATAATATAAGATTTTGACTTTTACCTCCTTCAC  240

Query  279   TATTTTTATTAATTAACTCTTCATTAATTGAATCTGGAGCTGGTACAGGATGAACAGTTT  338
             ||||||||||||||||||||||||||||||||||||||||||||||||||||||||||||
Sbjct  241   TATTTTTATTAATTAACTCTTCATTAATTGAATCTGGAGCTGGTACAGGATGAACAGTTT  300

Query  339   ACCCTCCTCTATCTTCAAATTTATCACATTATGGCCCTTCAGTAGATTTAGCTATTTTTT  398
```

```
            ||||||||||||||||||||||||||||||||||||||||||||||||||||||||||||
Sbjct  301  ACCCTCCTCTATCTTCAAATTTATCACATTATGGCCCTTCAGTAGATTTAGCTATTTTTT  360

Query  399  CTCTTCATCTTGCTGGTGCTTCTTCAATTTTAGGTGCAATTAACTTTATTACAACTATTG  458
            ||||||||||||||||||||||||||||||||||||||||||||||||||||||||||||
Sbjct  361  CTCTTCATCTTGCTGGTGCTTCTTCAATTTTAGGTGCAATTAACTTTATTACAACTATTG  420

Query  459  TAAACATACGATCAATTGGAATAACAATAGAACGTATACCATTATTTGTTTGATCTGTTT  518
            ||||||||||||||||||||||||||||||||||||||||||||||||||||||||||||
Sbjct  421  TAAACATACGATCAATTGGAATAACAATAGAACGTATACCATTATTTGTTTGATCTGTTT  480

Query  519  TAATTACAGCTATTTTATTACTTTTATCTTTACCTGTATTAGCAGGTGCCATTACAATAT  578
            ||||||||||||||||||||||||||||||||||||||||||||||||||||||||||||
Sbjct  481  TAATTACAGCTATTTTATTACTTTTATCTTTACCTGTATTAGCAGGTGCCATTACAATAT  540

Query  579  TGTTAACTGATCGAAACTTTAATACATCATTTTTTGATCCTTCAGGGGGAGGAGATCCAA  638
            ||||||||||||||||||||||||||||||||||||||||||||||||||||||||||||
Sbjct  541  TGTTAACTGATCGAAACTTTAATACATCATTTTTTGATCCTTCAGGGGGAGGAGATCCAA  600

Query  639  TTTTATATCAACATTTATTT  658
            ||||||||||||||||||||
Sbjct  601  TTTTATATCAACATTTATTT  620
```

>Rhipicephalus sanguineus clone #6 cytochrome oxidase subunit I (COI) gene, partial cds; mitochondrial
Sequence ID: KX519711.1 Length: 636
Range 1: 1 to 620

Score:1146 bits(620), Expect:0.0,
Identities:620/620(100%), Gaps:0/620(0%), Strand: Plus/Plus

```
Query  39   TAGGACTTAGTATAAGAATATTAATTCGTATAGAATTAGGACAACCTGGAACTTTAATTG  98
            ||||||||||||||||||||||||||||||||||||||||||||||||||||||||||||
Sbjct  1    TAGGACTTAGTATAAGAATATTAATTCGTATAGAATTAGGACAACCTGGAACTTTAATTG  60

Query  99   GAAATGATCAGATTTATAATGTAATCGTAACAGCACATGCATTTATTATAAtttttttA  158
            ||||||||||||||||||||||||||||||||||||||||||||||||||||||||||||
Sbjct  61   GAAATGATCAGATTTATAATGTAATCGTAACAGCACATGCATTTATTATAATTTTTTTA  120

Query  159  TAGTTATACCAATCATAATCGGAGGATTTGGAAATTGATTAGTCCCTATCATATTGGGTG  218
            ||||||||||||||||||||||||||||||||||||||||||||||||||||||||||||
Sbjct  121  TAGTTATACCAATCATAATCGGAGGATTTGGAAATTGATTAGTCCCTATCATATTGGGTG  180

Query  219  CCCCAGATATAGCATTTCCACGAATAAATAATATAAGATTTTGACTTTTACCTCCTTCAC  278
            ||||||||||||||||||||||||||||||||||||||||||||||||||||||||||||
Sbjct  181  CCCCAGATATAGCATTTCCACGAATAAATAATATAAGATTTTGACTTTTACCTCCTTCAC  240

Query  279  TATTTTTATTAATTAACTCTTCATTAATTGAATCTGGAGCTGGTACAGGATGAACAGTTT  338
            ||||||||||||||||||||||||||||||||||||||||||||||||||||||||||||
Sbjct  241  TATTTTTATTAATTAACTCTTCATTAATTGAATCTGGAGCTGGTACAGGATGAACAGTTT  300
```

```
Query  339  ACCCTCCTCTATCTTCAAATTTATCACATTATGGCCCTTCAGTAGATTTAGCTATTTTTT  398
            ||||||||||||||||||||||||||||||||||||||||||||||||||||||||||||
Sbjct  301  ACCCTCCTCTATCTTCAAATTTATCACATTATGGCCCTTCAGTAGATTTAGCTATTTTTT  360

Query  399  CTCTTCATCTTGCTGGTGCTTCTTCAATTTTAGGTGCAATTAACTTTATTACAACTATTG  458
            ||||||||||||||||||||||||||||||||||||||||||||||||||||||||||||
Sbjct  361  CTCTTCATCTTGCTGGTGCTTCTTCAATTTTAGGTGCAATTAACTTTATTACAACTATTG  420

Query  459  TAAACATACGATCAATTGGAATAACAATAGAACGTATACCATTATTTGTTTGATCTGTTT  518
            ||||||||||||||||||||||||||||||||||||||||||||||||||||||||||||
Sbjct  421  TAAACATACGATCAATTGGAATAACAATAGAACGTATACCATTATTTGTTTGATCTGTTT  480

Query  519  TAATTACAGCTATTTTATTACTTTTATCTTTACCTGTATTAGCAGGTGCCATTACAATAT  578
            ||||||||||||||||||||||||||||||||||||||||||||||||||||||||||||
Sbjct  481  TAATTACAGCTATTTTATTACTTTTATCTTTACCTGTATTAGCAGGTGCCATTACAATAT  540

Query  579  TGTTAACTGATCGAAACTTTAATACATCATTTTTTGATCCTTCAGGGGGAGGAGATCCAA  638
            ||||||||||||||||||||||||||||||||||||||||||||||||||||||||||||
Sbjct  541  TGTTAACTGATCGAAACTTTAATACATCATTTTTTGATCCTTCAGGGGGAGGAGATCCAA  600

Query  639  TTTTATATCAACATTTATTT  658
            ||||||||||||||||||||
Sbjct  601  TTTTATATCAACATTTATTT  620
```

>Rhipicephalus sanguineus isolate SV4 cytochrome c oxidase subunit I (COX1) gene, partial cds; mitochondrial
Sequence ID: MW135448.1 Length: 630
Range 1: 1 to 620

Score:1146 bits(620), Expect:0.0,
Identities:620/620(100%), Gaps:0/620(0%), Strand: Plus/Plus

```
Query  39   TAGGACTTAGTATAAGAATATTAATTCGTATAGAATTAGGACAACCTGGAACTTTAATTG  98
            ||||||||||||||||||||||||||||||||||||||||||||||||||||||||||||
Sbjct  1    TAGGACTTAGTATAAGAATATTAATTCGTATAGAATTAGGACAACCTGGAACTTTAATTG  60

Query  99   GAAATGATCAGATTTATAATGTAATCGTAACAGCACATGCATTTATTATAAtttttttA  158
            ||||||||||||||||||||||||||||||||||||||||||||||||||||||||||||
Sbjct  61   GAAATGATCAGATTTATAATGTAATCGTAACAGCACATGCATTTATTATAATTTTTTTA  120

Query  159  TAGTTATACCAATCATAATCGGAGGATTTGGAAATTGATTAGTCCCTATCATATTGGGTG  218
            ||||||||||||||||||||||||||||||||||||||||||||||||||||||||||||
Sbjct  121  TAGTTATACCAATCATAATCGGAGGATTTGGAAATTGATTAGTCCCTATCATATTGGGTG  180

Query  219  CCCCAGATATAGCATTTCCACGAATAAATAATATAAGATTTTGACTTTTACCTCCTTCAC  278
            ||||||||||||||||||||||||||||||||||||||||||||||||||||||||||||
Sbjct  181  CCCCAGATATAGCATTTCCACGAATAAATAATATAAGATTTTGACTTTTACCTCCTTCAC  240

Query  279  TATTTTTATTAATTAACTCTTCATTAATTGAATCTGGAGCTGGTACAGGATGAACAGTTT  338
            ||||||||||||||||||||||||||||||||||||||||||||||||||||||||||||
Sbjct  241  TATTTTTATTAATTAACTCTTCATTAATTGAATCTGGAGCTGGTACAGGATGAACAGTTT  300
```

```
Query  339   ACCCTCCTCTATCTTCAAATTTATCACATTATGGCCCTTCAGTAGATTTAGCTATTTTTT  398
             ||||||||||||||||||||||||||||||||||||||||||||||||||||||||||||
Sbjct  301   ACCCTCCTCTATCTTCAAATTTATCACATTATGGCCCTTCAGTAGATTTAGCTATTTTTT  360

Query  399   CTCTTCATCTTGCTGGTGCTTCTTCAATTTTAGGTGCAATTAACTTTATTACAACTATTG  458
             ||||||||||||||||||||||||||||||||||||||||||||||||||||||||||||
Sbjct  361   CTCTTCATCTTGCTGGTGCTTCTTCAATTTTAGGTGCAATTAACTTTATTACAACTATTG  420

Query  459   TAAACATACGATCAATTGGAATAACAATAGAACGTATACCATTATTTGTTTGATCTGTTT  518
             ||||||||||||||||||||||||||||||||||||||||||||||||||||||||||||
Sbjct  421   TAAACATACGATCAATTGGAATAACAATAGAACGTATACCATTATTTGTTTGATCTGTTT  480

Query  519   TAATTACAGCTATTTTATTACTTTTATCTTTACCTGTATTAGCAGGTGCCATTACAATAT  578
             ||||||||||||||||||||||||||||||||||||||||||||||||||||||||||||
Sbjct  481   TAATTACAGCTATTTTATTACTTTTATCTTTACCTGTATTAGCAGGTGCCATTACAATAT  540

Query  579   TGTTAACTGATCGAAACTTTAATACATCATTTTTTGATCCTTCAGGGGGAGGAGATCCAA  638
             ||||||||||||||||||||||||||||||||||||||||||||||||||||||||||||
Sbjct  541   TGTTAACTGATCGAAACTTTAATACATCATTTTTTGATCCTTCAGGGGGAGGAGATCCAA  600

Query  639   TTTTATATCAACATTTATTT  658
             ||||||||||||||||||||
Sbjct  601   TTTTATATCAACATTTATTT  620
```

>Rhipicephalus sanguineus isolate Samont2 cytochrome oxidase subunit I (cox1) gene, partial cds; mitochondrial
Sequence ID: MH630347.1 Length: 644
Range 1: 20 to 641

Score:1144 bits(619), Expect:0.0,
Identities:621/622(99%), Gaps:0/622(0%), Strand: Plus/Plus

```
Query  1     AACAATATATTTAATTTTTGGAGCATGATCAGGTATATTAGGACTTAGTATAAGAATATT  60
             ||||||||||||||||||||||||||||||||||||||||||||||||||||||||||||
Sbjct  20    AACAATATATTTAATTTTTGGAGCATGATCAGGTATATTAGGACTTAGTATAAGAATATT  79

Query  61    AATTCGTATAGAATTAGGACAACCTGGAACTTTAATTGGAAATGATCAGATTTATAATGT  120
             |||| |||||||||||||||||||||||||||||||||||||||||||||||||||||||
Sbjct  80    AATTGGTATAGAATTAGGACAACCTGGAACTTTAATTGGAAATGATCAGATTTATAATGT  139

Query  121   AATCGTAACAGCACATGCATTTATTATAAttttttttATAGTTATACCAATCATAATCGG  180
             ||||||||||||||||||||||||||||||||||||||||||||||||||||||||||||
Sbjct  140   AATCGTAACAGCACATGCATTTATTATAATTTTTTTTATAGTTATACCAATCATAATCGG  199

Query  181   AGGATTTGGAAATTGATTAGTCCCTATCATATTGGGTGCCCCAGATATAGCATTTCCACG  240
             ||||||||||||||||||||||||||||||||||||||||||||||||||||||||||||
Sbjct  200   AGGATTTGGAAATTGATTAGTCCCTATCATATTGGGTGCCCCAGATATAGCATTTCCACG  259

Query  241   AATAAATAATATAAGATTTTGACTTTTACCTCCTTCACTATTTTTATTAATTAACTCTTC  300
             ||||||||||||||||||||||||||||||||||||||||||||||||||||||||||||
```

```
Sbjct  260  AATAAATAATATAAGATTTTGACTTTTACCTCCTTCACTATTTTTATTAATTAACTCTTC  319

Query  301  ATTAATTGAATCTGGAGCTGGTACAGGATGAACAGTTTACCCTCCTCTATCTTCAAATTT  360
            ||||||||||||||||||||||||||||||||||||||||||||||||||||||||||||
Sbjct  320  ATTAATTGAATCTGGAGCTGGTACAGGATGAACAGTTTACCCTCCTCTATCTTCAAATTT  379

Query  361  ATCACATTATGGCCCTTCAGTAGATTTAGCTATTTTTTCTCTTCATCTTGCTGGTGCTTC  420
            ||||||||||||||||||||||||||||||||||||||||||||||||||||||||||||
Sbjct  380  ATCACATTATGGCCCTTCAGTAGATTTAGCTATTTTTTCTCTTCATCTTGCTGGTGCTTC  439

Query  421  TTCAATTTTAGGTGCAATTAACTTTATTACAACTATTGTAAACATACGATCAATTGGAAT  480
            ||||||||||||||||||||||||||||||||||||||||||||||||||||||||||||
Sbjct  440  TTCAATTTTAGGTGCAATTAACTTTATTACAACTATTGTAAACATACGATCAATTGGAAT  499

Query  481  AACAATAGAACGTATACCATTATTTGTTTGATCTGTTTTAATTACAGCTATTTTATTACT  540
            ||||||||||||||||||||||||||||||||||||||||||||||||||||||||||||
Sbjct  500  AACAATAGAACGTATACCATTATTTGTTTGATCTGTTTTAATTACAGCTATTTTATTACT  559

Query  541  TTTATCTTTACCTGTATTAGCAGGTGCCATTACAATATTGTTAACTGATCGAAACTTTAA  600
            ||||||||||||||||||||||||||||||||||||||||||||||||||||||||||||
Sbjct  560  TTTATCTTTACCTGTATTAGCAGGTGCCATTACAATATTGTTAACTGATCGAAACTTTAA  619

Query  601  TACATCATTTTTTGATCCTTCA  622
            ||||||||||||||||||||||
Sbjct  620  TACATCATTTTTTGATCCTTCA  641
```

>Rhipicephalus sanguineus isolate Samont1 cytochrome oxidase subunit I (cox1) gene, partial cds; mitochondrial
Sequence ID: MH630346.1 Length: 644
Range 1: 20 to 641

Score:1144 bits(619), Expect:0.0,
Identities:621/622(99%), Gaps:0/622(0%), Strand: Plus/Plus

```
Query  1    AACAATATATTTAATTTTTGGAGCATGATCAGGTATATTAGGACTTAGTATAAGAATATT  60
            ||||||||||||||||||||||||||||||||||||||||||||||||||||||||||||
Sbjct  20   AACAATATATTTAATTTTTGGAGCATGATCAGGTATATTAGGACTTAGTATAAGAATATT  79

Query  61   AATTCGTATAGAATTAGGACAACCTGGAACTTTAATTGGAAATGATCAGATTTATAATGT  120
            |||| |||||||||||||||||||||||||||||||||||||||||||||||||||||||
Sbjct  80   AATTGGTATAGAATTAGGACAACCTGGAACTTTAATTGGAAATGATCAGATTTATAATGT  139

Query  121  AATCGTAACAGCACATGCATTTATTATAAtttttttATAGTTATACCAATCATAATCGG  180
            ||||||||||||||||||||||||||||||||||||||||||||||||||||||||||||
Sbjct  140  AATCGTAACAGCACATGCATTTATTATAATTTTTTTATAGTTATACCAATCATAATCGG  199

Query  181  AGGATTTGGAAATTGATTAGTCCCTATCATATTGGGTGCCCCAGATATAGCATTTCCACG  240
            ||||||||||||||||||||||||||||||||||||||||||||||||||||||||||||
Sbjct  200  AGGATTTGGAAATTGATTAGTCCCTATCATATTGGGTGCCCCAGATATAGCATTTCCACG  259

Query  241  AATAAATAATATAAGATTTTGACTTTTACCTCCTTCACTATTTTTATTAATTAACTCTTC  300
```

```
            ||||||||||||||||||||||||||||||||||||||||||||||||||||||||||||
Sbjct  260  AATAAATAATATAAGATTTTGACTTTTACCTCCTTCACTATTTTTATTAATTAACTCTTC  319

Query  301  ATTAATTGAATCTGGAGCTGGTACAGGATGAACAGTTTACCCTCCTCTATCTTCAAATTT  360
            ||||||||||||||||||||||||||||||||||||||||||||||||||||||||||||
Sbjct  320  ATTAATTGAATCTGGAGCTGGTACAGGATGAACAGTTTACCCTCCTCTATCTTCAAATTT  379

Query  361  ATCACATTATGGCCCTTCAGTAGATTTAGCTATTTTTTCTCTTCATCTTGCTGGTGCTTC  420
            ||||||||||||||||||||||||||||||||||||||||||||||||||||||||||||
Sbjct  380  ATCACATTATGGCCCTTCAGTAGATTTAGCTATTTTTTCTCTTCATCTTGCTGGTGCTTC  439

Query  421  TTCAATTTTAGGTGCAATTAACTTTATTACAACTATTGTAAACATACGATCAATTGGAAT  480
            ||||||||||||||||||||||||||||||||||||||||||||||||||||||||||||
Sbjct  440  TTCAATTTTAGGTGCAATTAACTTTATTACAACTATTGTAAACATACGATCAATTGGAAT  499

Query  481  AACAATAGAACGTATACCATTATTTGTTTGATCTGTTTTAATTACAGCTATTTTATTACT  540
            ||||||||||||||||||||||||||||||||||||||||||||||||||||||||||||
Sbjct  500  AACAATAGAACGTATACCATTATTTGTTTGATCTGTTTTAATTACAGCTATTTTATTACT  559

Query  541  TTTATCTTTACCTGTATTAGCAGGTGCCATTACAATATTGTTAACTGATCGAAACTTTAA  600
            ||||||||||||||||||||||||||||||||||||||||||||||||||||||||||||
Sbjct  560  TTTATCTTTACCTGTATTAGCAGGTGCCATTACAATATTGTTAACTGATCGAAACTTTAA  619

Query  601  TACATCATTTTTTGATCCTTCA  622
            ||||||||||||||||||||||
Sbjct  620  TACATCATTTTTTGATCCTTCA  641
```

>Rhipicephalus sanguineus sensu lato 'temperate lineage' isolate CR1568 cytochrome c oxidase subunit I (COI) gene, partial cds; mitochondrial
Sequence ID: MF426000.1 Length: 635
Range 1: 14 to 635

Score:1144 bits(619), Expect:0.0,
Identities:621/622(99%), Gaps:0/622(0%), Strand: Plus/Plus

```
Query  1    AACAATATATTTAATTTTTGGAGCATGATCAGGTATATTAGGACTTAGTATAAGAATATT  60
            ||||||||||||||||||||||||||||||||||||||||||||||||||||||||||||
Sbjct  14   AACAATATATTTAATTTTTGGAGCATGATCAGGTATATTAGGACTTAGTATAAGAATATT  73

Query  61   AATTCGTATAGAATTAGGACAACCTGGAACTTTAATTGGAAATGATCAGATTTATAATGT  120
            ||||||||||||||||||||||||||||||||||||||||||||||||||||||||||||
Sbjct  74   AATTCGTATAGAATTAGGACAACCTGGAACTTTAATTGGAAATGATCAGATTTATAATGT  133

Query  121  AATCGTAACAGCACATGCATTTATTATAAttttttttATAGTTATACCAATCATAATCGG  180
            ||||||||||||||||||||||||||||||||||||||||||||||||||||||||||||
Sbjct  134  AATCGTAACAGCACATGCATTTATTATAATTTTTTTTATAGTTATACCAATCATAATCGG  193

Query  181  AGGATTTGGAAATTGATTAGTCCCTATCATATTGGGTGCCCCAGATATAGCATTTCCACG  240
            ||||||||||||||||||||||||||||||||||||||||||||||||||||||||||||
Sbjct  194  AGGATTTGGAAATTGATTAGTCCCTATCATATTGGGTGCCCCAGATATAGCATTTCCACG  253
```

```
Query  241  AATAAATAATATAAGATTTTGACTTTTACCTCCTTCACTATTTTTATTAATTAACTCTTC  300
            ||||||||||||||||||||||||||||||||||||||||||||||||||||||||||||
Sbjct  254  AATAAATAATATAAGATTTTGACTTTTACCTCCTTCACTATTTTTATTAATTAACTCTTC  313

Query  301  ATTAATTGAATCTGGAGCTGGTACAGGATGAACAGTTTACCCTCCTCTATCTTCAAATTT  360
            ||||||||||||||||||||||||||||||||||||||||||||||||||||||||||||
Sbjct  314  ATTAATTGAATCTGGAGCTGGTACAGGATGAACAGTTTACCCTCCTCTATCTTCAAATTT  373

Query  361  ATCACATTATGGCCCTTCAGTAGATTTAGCTATTTTTTCTCTTCATCTTGCTGGTGCTTC  420
            |||||||||||| |||||||||||||||||||||||||||||||||||||||||||||||
Sbjct  374  ATCACATTATGGTCCTTCAGTAGATTTAGCTATTTTTTCTCTTCATCTTGCTGGTGCTTC  433

Query  421  TTCAATTTTAGGTGCAATTAACTTTATTACAACTATTGTAAACATACGATCAATTGGAAT  480
            ||||||||||||||||||||||||||||||||||||||||||||||||||||||||||||
Sbjct  434  TTCAATTTTAGGTGCAATTAACTTTATTACAACTATTGTAAACATACGATCAATTGGAAT  493

Query  481  AACAATAGAACGTATACCATTATTTGTTTGATCTGTTTTAATTACAGCTATTTTATTACT  540
            ||||||||||||||||||||||||||||||||||||||||||||||||||||||||||||
Sbjct  494  AACAATAGAACGTATACCATTATTTGTTTGATCTGTTTTAATTACAGCTATTTTATTACT  553

Query  541  TTTATCTTTACCTGTATTAGCAGGTGCCATTACAATATTGTTAACTGATCGAAACTTTAA  600
            ||||||||||||||||||||||||||||||||||||||||||||||||||||||||||||
Sbjct  554  TTTATCTTTACCTGTATTAGCAGGTGCCATTACAATATTGTTAACTGATCGAAACTTTAA  613

Query  601  TACATCATTTTTTGATCCTTCA  622
            ||||||||||||||||||||||
Sbjct  614  TACATCATTTTTTGATCCTTCA  635
```

>Rhipicephalus sanguineus isolate Boka-A cytochrome oxidase subunit I (COI) gene, partial cds; mitochondrial
Sequence ID: KX757905.1 Length: 630
Range 1: 1 to 620

Score:1140 bits(617), Expect:0.0,
Identities:619/620(99%), Gaps:0/620(0%), Strand: Plus/Plus

```
Query  39   TAGGACTTAGTATAAGAATATTAATTCGTATAGAATTAGGACAACCTGGAACTTTAATTG  98
            ||||||||||||||||||||||||||||||||||||||||||||||||||||||||||||
Sbjct  1    TAGGACTTAGTATAAGAATATTAATTCGTATAGAATTAGGACAACCTGGAACTTTAATTG  60

Query  99   GAAATGATCAGATTTATAATGTAATCGTAACAGCACATGCATTTATTATAAtttttttA  158
            ||||||||||||||||||||||||||||||||||||||||||||||||||||||||||||
Sbjct  61   GAAATGATCAGATTTATAATGTAATCGTAACAGCACATGCATTTATTATAATTTTTTTA  120

Query  159  TAGTTATACCAATCATAATCGGAGGATTTGGAAATTGATTAGTCCCTATCATATTGGGTG  218
            ||||||||||||||||||||||||||||||||||||||||||||||||||||||||||||
Sbjct  121  TAGTTATACCAATCATAATCGGAGGATTTGGAAATTGATTAGTCCCTATCATATTGGGTG  180

Query  219  CCCCAGATATAGCATTTCCACGAATAAATAATATAAGATTTTGACTTTTACCTCCTTCAC  278
            ||||||||||||||||||||||||||||||||||||||||||||||||||||||||||||
Sbjct  181  CCCCAGATATAGCATTTCCACGAATAAATAATATAAGATTTTGACTTTTACCTCCTTCAC  240
```

```
Query  279   TATTTTTATTAATTAACTCTTCATTAATTGAATCTGGAGCTGGTACAGGATGAACAGTTT  338
             |||||||||||||||| |||||||||||||||||||||||||||||||||||||||||||
Sbjct  241   TATTTTTATTAATTAATTCTTCATTAATTGAATCTGGAGCTGGTACAGGATGAACAGTTT  300

Query  339   ACCCTCCTCTATCTTCAAATTTATCACATTATGGCCCTTCAGTAGATTTAGCTATTTTTT  398
             ||||||||||||||||||||||||||||||||||||||||||||||||||||||||||||
Sbjct  301   ACCCTCCTCTATCTTCAAATTTATCACATTATGGCCCTTCAGTAGATTTAGCTATTTTTT  360

Query  399   CTCTTCATCTTGCTGGTGCTTCTTCAATTTTAGGTGCAATTAACTTTATTACAACTATTG  458
             ||||||||||||||||||||||||||||||||||||||||||||||||||||||||||||
Sbjct  361   CTCTTCATCTTGCTGGTGCTTCTTCAATTTTAGGTGCAATTAACTTTATTACAACTATTG  420

Query  459   TAAACATACGATCAATTGGAATAACAATAGAACGTATACCATTATTTGTTTGATCTGTTT  518
             ||||||||||||||||||||||||||||||||||||||||||||||||||||||||||||
Sbjct  421   TAAACATACGATCAATTGGAATAACAATAGAACGTATACCATTATTTGTTTGATCTGTTT  480

Query  519   TAATTACAGCTATTTTATTACTTTTATCTTTACCTGTATTAGCAGGTGCCATTACAATAT  578
             ||||||||||||||||||||||||||||||||||||||||||||||||||||||||||||
Sbjct  481   TAATTACAGCTATTTTATTACTTTTATCTTTACCTGTATTAGCAGGTGCCATTACAATAT  540

Query  579   TGTTAACTGATCGAAACTTTAATACATCATTTTTTGATCCTTCAGGGGGAGGAGATCCAA  638
             ||||||||||||||||||||||||||||||||||||||||||||||||||||||||||||
Sbjct  541   TGTTAACTGATCGAAACTTTAATACATCATTTTTTGATCCTTCAGGGGGAGGAGATCCAA  600

Query  639   TTTTATATCAACATTTATTT  658
             ||||||||||||||||||||
Sbjct  601   TTTTATATCAACATTTATTT  620



>Rhipicephalus sanguineus isolate Sibenik cytochrome oxidase subunit I (COI)
gene, partial cds; mitochondrial
Sequence ID: KX757895.1 Length: 630
Range 1: 1 to 620

Score:1140 bits(617), Expect:0.0,
Identities:619/620(99%),  Gaps:0/620(0%), Strand: Plus/Plus

Query  39    TAGGACTTAGTATAAGAATATTAATTCGTATAGAATTAGGACAACCTGGAACTTTAATTG  98
             ||||||||||||||||||||||||||||||||| ||||||||||||||||||||||||||
Sbjct  1     TAGGACTTAGTATAAGAATATTAATTCGTATAGAGTTAGGACAACCTGGAACTTTAATTG  60

Query  99    GAAATGATCAGATTTATAATGTAATCGTAACAGCACATGCATTTATTATAAtttttttA  158
             ||||||||||||||||||||||||||||||||||||||||||||||||||||||||||||
Sbjct  61    GAAATGATCAGATTTATAATGTAATCGTAACAGCACATGCATTTATTATAATTTTTTTA  120

Query  159   TAGTTATACCAATCATAATCGGAGGATTTGGAAATTGATTAGTCCCTATCATATTGGGTG  218
             ||||||||||||||||||||||||||||||||||||||||||||||||||||||||||||
Sbjct  121   TAGTTATACCAATCATAATCGGAGGATTTGGAAATTGATTAGTCCCTATCATATTGGGTG  180

Query  219   CCCCAGATATAGCATTTCCACGAATAAATAATATAAGATTTTGACTTTTACCTCCTTCAC  278
             ||||||||||||||||||||||||||||||||||||||||||||||||||||||||||||
```

```
Sbjct  181   CCCCAGATATAGCATTTCCACGAATAAATAATATAAGATTTTGACTTTTACCTCCTTCAC  240

Query  279   TATTTTTATTAATTAACTCTTCATTAATTGAATCTGGAGCTGGTACAGGATGAACAGTTT  338
             ||||||||||||||||||||||||||||||||||||||||||||||||||||||||||||
Sbjct  241   TATTTTTATTAATTAACTCTTCATTAATTGAATCTGGAGCTGGTACAGGATGAACAGTTT  300

Query  339   ACCCTCCTCTATCTTCAAATTTATCACATTATGGCCCTTCAGTAGATTTAGCTATTTTTT  398
             ||||||||||||||||||||||||||||||||||||||||||||||||||||||||||||
Sbjct  301   ACCCTCCTCTATCTTCAAATTTATCACATTATGGCCCTTCAGTAGATTTAGCTATTTTTT  360

Query  399   CTCTTCATCTTGCTGGTGCTTCTTCAATTTTAGGTGCAATTAACTTTATTACAACTATTG  458
             ||||||||||||||||||||||||||||||||||||||||||||||||||||||||||||
Sbjct  361   CTCTTCATCTTGCTGGTGCTTCTTCAATTTTAGGTGCAATTAACTTTATTACAACTATTG  420

Query  459   TAAACATACGATCAATTGGAATAACAATAGAACGTATACCATTATTTGTTTGATCTGTTT  518
             ||||||||||||||||||||||||||||||||||||||||||||||||||||||||||||
Sbjct  421   TAAACATACGATCAATTGGAATAACAATAGAACGTATACCATTATTTGTTTGATCTGTTT  480

Query  519   TAATTACAGCTATTTTATTACTTTTATCTTTACCTGTATTAGCAGGTGCCATTACAATAT  578
             ||||||||||||||||||||||||||||||||||||||||||||||||||||||||||||
Sbjct  481   TAATTACAGCTATTTTATTACTTTTATCTTTACCTGTATTAGCAGGTGCCATTACAATAT  540

Query  579   TGTTAACTGATCGAAACTTTAATACATCATTTTTTGATCCTTCAGGGGGAGGAGATCCAA  638
             ||||||||||||||||||||||||||||||||||||||||||||||||||||||||||||
Sbjct  541   TGTTAACTGATCGAAACTTTAATACATCATTTTTTGATCCTTCAGGGGGAGGAGATCCAA  600

Query  639   TTTTATATCAACATTTATTT  658
             ||||||||||||||||||||
Sbjct  601   TTTTATATCAACATTTATTT  620
```

>Rhipicephalus sanguineus isolate Zadar cytochrome oxidase subunit I (COI) gene, partial cds; mitochondrial
Sequence ID: KX757892.1 Length: 630
Range 1: 1 to 620

Score:1140 bits(617), Expect:0.0,
Identities:619/620(99%), Gaps:0/620(0%), Strand: Plus/Plus

```
Query  39    TAGGACTTAGTATAAGAATATTAATTCGTATAGAATTAGGACAACCTGGAACTTTAATTG  98
             |||||||||||||||||||||||||||||||||| |||||||||||||||||||||||||
Sbjct  1     TAGGACTTAGTATAAGAATATTAATTCGTATAGAGTTAGGACAACCTGGAACTTTAATTG  60

Query  99    GAAATGATCAGATTTATAATGTAATCGTAACAGCACATGCATTTATTATAAttttttttA  158
             ||||||||||||||||||||||||||||||||||||||||||||||||||||||||||||
Sbjct  61    GAAATGATCAGATTTATAATGTAATCGTAACAGCACATGCATTTATTATAATTTTTTTTA  120

Query  159   TAGTTATACCAATCATAATCGGAGGATTTGGAAATTGATTAGTCCCTATCATATTGGGTG  218
             ||||||||||||||||||||||||||||||||||||||||||||||||||||||||||||
Sbjct  121   TAGTTATACCAATCATAATCGGAGGATTTGGAAATTGATTAGTCCCTATCATATTGGGTG  180

Query  219   CCCCAGATATAGCATTTCCACGAATAAATAATATAAGATTTTGACTTTTACCTCCTTCAC  278
```

```
                ||||||||||||||||||||||||||||||||||||||||||||||||||||||||||||
Sbjct  181  CCCCAGATATAGCATTTCCACGAATAAATAATATAAGATTTTGACTTTTACCTCCTTCAC  240

Query  279  TATTTTTATTAATTAACTCTTCATTAATTGAATCTGGAGCTGGTACAGGATGAACAGTTT  338
            ||||||||||||||||||||||||||||||||||||||||||||||||||||||||||||
Sbjct  241  TATTTTTATTAATTAACTCTTCATTAATTGAATCTGGAGCTGGTACAGGATGAACAGTTT  300

Query  339  ACCCTCCTCTATCTTCAAATTTATCACATTATGGCCCTTCAGTAGATTTAGCTATTTTTT  398
            ||||||||||||||||||||||||||||||||||||||||||||||||||||||||||||
Sbjct  301  ACCCTCCTCTATCTTCAAATTTATCACATTATGGCCCTTCAGTAGATTTAGCTATTTTTT  360

Query  399  CTCTTCATCTTGCTGGTGCTTCTTCAATTTTAGGTGCAATTAACTTTATTACAACTATTG  458
            ||||||||||||||||||||||||||||||||||||||||||||||||||||||||||||
Sbjct  361  CTCTTCATCTTGCTGGTGCTTCTTCAATTTTAGGTGCAATTAACTTTATTACAACTATTG  420

Query  459  TAAACATACGATCAATTGGAATAACAATAGAACGTATACCATTATTTGTTTGATCTGTTT  518
            ||||||||||||||||||||||||||||||||||||||||||||||||||||||||||||
Sbjct  421  TAAACATACGATCAATTGGAATAACAATAGAACGTATACCATTATTTGTTTGATCTGTTT  480

Query  519  TAATTACAGCTATTTTATTACTTTTATCTTTACCTGTATTAGCAGGTGCCATTACAATAT  578
            ||||||||||||||||||||||||||||||||||||||||||||||||||||||||||||
Sbjct  481  TAATTACAGCTATTTTATTACTTTTATCTTTACCTGTATTAGCAGGTGCCATTACAATAT  540

Query  579  TGTTAACTGATCGAAACTTTAATACATCATTTTTTGATCCTTCAGGGGGAGGAGATCCAA  638
            ||||||||||||||||||||||||||||||||||||||||||||||||||||||||||||
Sbjct  541  TGTTAACTGATCGAAACTTTAATACATCATTTTTTGATCCTTCAGGGGGAGGAGATCCAA  600

Query  639  TTTTATATCAACATTTATTT  658
            ||||||||||||||||||||
Sbjct  601  TTTTATATCAACATTTATTT  620
```

```
>Rhipicephalus sanguineus isolate Zagreb-A cytochrome oxidase subunit I (COI)
gene, partial cds; mitochondrial
Sequence ID: KX757890.1 Length: 630
Range 1: 1 to 620

Score:1140 bits(617), Expect:0.0,
Identities:619/620(99%),  Gaps:0/620(0%), Strand: Plus/Plus

Query  39   TAGGACTTAGTATAAGAATATTAATTCGTATAGAATTAGGACAACCTGGAACTTTAATTG  98
            |||||||||||||||||||||||||||||||||| |||||||||||||||||||||||||
Sbjct  1    TAGGACTTAGTATAAGAATATTAATTCGTATAGAGTTAGGACAACCTGGAACTTTAATTG  60

Query  99   GAAATGATCAGATTTATAATGTAATCGTAACAGCACATGCATTTATTATAAtttttttA  158
            ||||||||||||||||||||||||||||||||||||||||||||||||||||||||||||
Sbjct  61   GAAATGATCAGATTTATAATGTAATCGTAACAGCACATGCATTTATTATAATTTTTTTTA  120

Query  159  TAGTTATACCAATCATAATCGGAGGATTTGGAAATTGATTAGTCCCTATCATATTGGGTG  218
            ||||||||||||||||||||||||||||||||||||||||||||||||||||||||||||
Sbjct  121  TAGTTATACCAATCATAATCGGAGGATTTGGAAATTGATTAGTCCCTATCATATTGGGTG  180
```

```
Query  219  CCCCAGATATAGCATTTCCACGAATAAATAATATAAGATTTTGACTTTTACCTCCTTCAC  278
            ||||||||||||||||||||||||||||||||||||||||||||||||||||||||||||
Sbjct  181  CCCCAGATATAGCATTTCCACGAATAAATAATATAAGATTTTGACTTTTACCTCCTTCAC  240

Query  279  TATTTTTATTAATTAACTCTTCATTAATTGAATCTGGAGCTGGTACAGGATGAACAGTTT  338
            ||||||||||||||||||||||||||||||||||||||||||||||||||||||||||||
Sbjct  241  TATTTTTATTAATTAACTCTTCATTAATTGAATCTGGAGCTGGTACAGGATGAACAGTTT  300

Query  339  ACCCTCCTCTATCTTCAAATTTATCACATTATGGCCCTTCAGTAGATTTAGCTATTTTTT  398
            ||||||||||||||||||||||||||||||||||||||||||||||||||||||||||||
Sbjct  301  ACCCTCCTCTATCTTCAAATTTATCACATTATGGCCCTTCAGTAGATTTAGCTATTTTTT  360

Query  399  CTCTTCATCTTGCTGGTGCTTCTTCAATTTTAGGTGCAATTAACTTTATTACAACTATTG  458
            ||||||||||||||||||||||||||||||||||||||||||||||||||||||||||||
Sbjct  361  CTCTTCATCTTGCTGGTGCTTCTTCAATTTTAGGTGCAATTAACTTTATTACAACTATTG  420

Query  459  TAAACATACGATCAATTGGAATAACAATAGAACGTATACCATTATTTGTTTGATCTGTTT  518
            ||||||||||||||||||||||||||||||||||||||||||||||||||||||||||||
Sbjct  421  TAAACATACGATCAATTGGAATAACAATAGAACGTATACCATTATTTGTTTGATCTGTTT  480

Query  519  TAATTACAGCTATTTTATTACTTTTATCTTTACCTGTATTAGCAGGTGCCATTACAATAT  578
            ||||||||||||||||||||||||||||||||||||||||||||||||||||||||||||
Sbjct  481  TAATTACAGCTATTTTATTACTTTTATCTTTACCTGTATTAGCAGGTGCCATTACAATAT  540

Query  579  TGTTAACTGATCGAAACTTTAATACATCATTTTTTGATCCTTCAGGGGGAGGAGATCCAA  638
            ||||||||||||||||||||||||||||||||||||||||||||||||||||||||||||
Sbjct  541  TGTTAACTGATCGAAACTTTAATACATCATTTTTTGATCCTTCAGGGGGAGGAGATCCAA  600

Query  639  TTTTATATCAACATTTATTT  658
            ||||||||||||||||||||
Sbjct  601  TTTTATATCAACATTTATTT  620
```

>Rhipicephalus sanguineus isolate Pula-A cytochrome oxidase subunit I (COI) gene, partial cds; mitochondrial
Sequence ID: KX757888.1 Length: 630
Range 1: 1 to 620

Score:1140 bits(617), Expect:0.0,
Identities:619/620(99%), Gaps:0/620(0%), Strand: Plus/Plus

```
Query  39   TAGGACTTAGTATAAGAATATTAATTCGTATAGAATTAGGACAACCTGGAACTTTAATTG  98
            ||||||||||||||||||||||||||||||||||||||||||||||||||||||||||||
Sbjct  1    TAGGACTTAGTATAAGAATATTAATTCGTATAGAATTAGGACAACCTGGAACTTTAATTG  60

Query  99   GAAATGATCAGATTTATAATGTAATCGTAACAGCACATGCATTTATTATAAttttttttA  158
            ||||||||||||||||||||||||||||||||||||||||||||||||||||||||||||
Sbjct  61   GAAATGATCAGATTTATAATGTAATCGTAACAGCACATGCATTTATTATAATTTTTTTTA  120

Query  159  TAGTTATACCAATCATAATCGGAGGATTTGGAAATTGATTAGTCCCTATCATATTGGGTG  218
            ||||||||||||||||||||||||||||||||||||||||||||||||||||||||||||
Sbjct  121  TAGTTATACCAATCATAATCGGAGGATTTGGAAATTGATTAGTCCCTATCATATTGGGTG  180
```

```
Query  219   CCCCAGATATAGCATTTCCACGAATAAATAATATAAGATTTTGACTTTTACCTCCTTCAC  278
             ||||||||||||||||||||||||||||||||||||||||||||||||||||||||||||
Sbjct  181   CCCCAGATATAGCATTTCCACGAATAAATAATATAAGATTTTGACTTTTACCTCCTTCAC  240

Query  279   TATTTTTATTAATTAACTCTTCATTAATTGAATCTGGAGCTGGTACAGGATGAACAGTTT  338
             |||||||||||||||| |||||||||||||||||||||||||||||||||||||||||||
Sbjct  241   TATTTTTATTAATTAATTCTTCATTAATTGAATCTGGAGCTGGTACAGGATGAACAGTTT  300

Query  339   ACCCTCCTCTATCTTCAAATTTATCACATTATGGCCCTTCAGTAGATTTAGCTATTTTTT  398
             ||||||||||||||||||||||||||||||||||||||||||||||||||||||||||||
Sbjct  301   ACCCTCCTCTATCTTCAAATTTATCACATTATGGCCCTTCAGTAGATTTAGCTATTTTTT  360

Query  399   CTCTTCATCTTGCTGGTGCTTCTTCAATTTTAGGTGCAATTAACTTTATTACAACTATTG  458
             ||||||||||||||||||||||||||||||||||||||||||||||||||||||||||||
Sbjct  361   CTCTTCATCTTGCTGGTGCTTCTTCAATTTTAGGTGCAATTAACTTTATTACAACTATTG  420

Query  459   TAAACATACGATCAATTGGAATAACAATAGAACGTATACCATTATTTGTTTGATCTGTTT  518
             ||||||||||||||||||||||||||||||||||||||||||||||||||||||||||||
Sbjct  421   TAAACATACGATCAATTGGAATAACAATAGAACGTATACCATTATTTGTTTGATCTGTTT  480

Query  519   TAATTACAGCTATTTTATTACTTTTATCTTTACCTGTATTAGCAGGTGCCATTACAATAT  578
             ||||||||||||||||||||||||||||||||||||||||||||||||||||||||||||
Sbjct  481   TAATTACAGCTATTTTATTACTTTTATCTTTACCTGTATTAGCAGGTGCCATTACAATAT  540

Query  579   TGTTAACTGATCGAAACTTTAATACATCATTTTTTGATCCTTCAGGGGGAGGAGATCCAA  638
             ||||||||||||||||||||||||||||||||||||||||||||||||||||||||||||
Sbjct  541   TGTTAACTGATCGAAACTTTAATACATCATTTTTTGATCCTTCAGGGGGAGGAGATCCAA  600

Query  639   TTTTATATCAACATTTATTT  658
             ||||||||||||||||||||
Sbjct  601   TTTTATATCAACATTTATTT  620
```

>Rhipicephalus sanguineus isolate Petnica cytochrome oxidase subunit I (COI) gene, partial cds; mitochondrial
Sequence ID: KX757883.1 Length: 630
Range 1: 1 to 620

Score:1140 bits(617), Expect:0.0,
Identities:619/620(99%), Gaps:0/620(0%), Strand: Plus/Plus

```
Query  39    TAGGACTTAGTATAAGAATATTAATTCGTATAGAATTAGGACAACCTGGAACTTTAATTG  98
             |||||||||||||||||||||||||||||||||| |||||||||||||||||||||||||
Sbjct  1     TAGGACTTAGTATAAGAATATTAATTCGTATAGAGTTAGGACAACCTGGAACTTTAATTG  60

Query  99    GAAATGATCAGATTTATAATGTAATCGTAACAGCACATGCATTTATTATAAtttttttA  158
             ||||||||||||||||||||||||||||||||||||||||||||||||||||||||||||
Sbjct  61    GAAATGATCAGATTTATAATGTAATCGTAACAGCACATGCATTTATTATAATTTTTTTTA  120

Query  159   TAGTTATACCAATCATAATCGGAGGATTTGGAAATTGATTAGTCCCTATCATATTGGGTG  218
             ||||||||||||||||||||||||||||||||||||||||||||||||||||||||||||
```

```
Sbjct  121   TAGTTATACCAATCATAATCGGAGGATTTGGAAATTGATTAGTCCCTATCATATTGGGTG  180

Query  219   CCCCAGATATAGCATTTCCACGAATAAATAATATAAGATTTTGACTTTTACCTCCTTCAC  278
             ||||||||||||||||||||||||||||||||||||||||||||||||||||||||||||
Sbjct  181   CCCCAGATATAGCATTTCCACGAATAAATAATATAAGATTTTGACTTTTACCTCCTTCAC  240

Query  279   TATTTTTATTAATTAACTCTTCATTAATTGAATCTGGAGCTGGTACAGGATGAACAGTTT  338
             ||||||||||||||||||||||||||||||||||||||||||||||||||||||||||||
Sbjct  241   TATTTTTATTAATTAACTCTTCATTAATTGAATCTGGAGCTGGTACAGGATGAACAGTTT  300

Query  339   ACCCTCCTCTATCTTCAAATTTATCACATTATGGCCCTTCAGTAGATTTAGCTATTTTTT  398
             ||||||||||||||||||||||||||||||||||||||||||||||||||||||||||||
Sbjct  301   ACCCTCCTCTATCTTCAAATTTATCACATTATGGCCCTTCAGTAGATTTAGCTATTTTTT  360

Query  399   CTCTTCATCTTGCTGGTGCTTCTTCAATTTTAGGTGCAATTAACTTTATTACAACTATTG  458
             ||||||||||||||||||||||||||||||||||||||||||||||||||||||||||||
Sbjct  361   CTCTTCATCTTGCTGGTGCTTCTTCAATTTTAGGTGCAATTAACTTTATTACAACTATTG  420

Query  459   TAAACATACGATCAATTGGAATAACAATAGAACGTATACCATTATTTGTTTGATCTGTTT  518
             ||||||||||||||||||||||||||||||||||||||||||||||||||||||||||||
Sbjct  421   TAAACATACGATCAATTGGAATAACAATAGAACGTATACCATTATTTGTTTGATCTGTTT  480

Query  519   TAATTACAGCTATTTTATTACTTTTATCTTTACCTGTATTAGCAGGTGCCATTACAATAT  578
             ||||||||||||||||||||||||||||||||||||||||||||||||||||||||||||
Sbjct  481   TAATTACAGCTATTTTATTACTTTTATCTTTACCTGTATTAGCAGGTGCCATTACAATAT  540

Query  579   TGTTAACTGATCGAAACTTTAATACATCATTTTTTGATCCTTCAGGGGGAGGAGATCCAA  638
             ||||||||||||||||||||||||||||||||||||||||||||||||||||||||||||
Sbjct  541   TGTTAACTGATCGAAACTTTAATACATCATTTTTTGATCCTTCAGGGGGAGGAGATCCAA  600

Query  639   TTTTATATCAACATTTATTT  658
             ||||||||||||||||||||
Sbjct  601   TTTTATATCAACATTTATTT  620
```

>Rhipicephalus sanguineus clone #3 cytochrome oxidase subunit I (COI) gene, partial cds; mitochondrial
Sequence ID: KX519708.1 Length: 629
Range 1: 4 to 620

Score:1140 bits(617), Expect:0.0,
Identities:617/617(100%), Gaps:0/617(0%), Strand: Plus/Plus

```
Query  42    GACTTAGTATAAGAATATTAATTCGTATAGAATTAGGACAACCTGGAACTTTAATTGGAA  101
             ||||||||||||||||||||||||||||||||||||||||||||||||||||||||||||
Sbjct  4     GACTTAGTATAAGAATATTAATTCGTATAGAATTAGGACAACCTGGAACTTTAATTGGAA  63

Query  102   ATGATCAGATTTATAATGTAATCGTAACAGCACATGCATTTATTATAAttttttttATAG  161
             ||||||||||||||||||||||||||||||||||||||||||||||||||||||||||||
Sbjct  64    ATGATCAGATTTATAATGTAATCGTAACAGCACATGCATTTATTATAATTTTTTTTATAG  123

Query  162   TTATACCAATCATAATCGGAGGATTTGGAAATTGATTAGTCCCTATCATATTGGGTGCCC  221
```

```
                ||||||||||||||||||||||||||||||||||||||||||||||||||||||||||||
Sbjct  124  TTATACCAATCATAATCGGAGGATTTGGAAATTGATTAGTCCCTATCATATTGGGTGCCC  183

Query  222  CAGATATAGCATTTCCACGAATAAATAATATAAGATTTTGACTTTTACCTCCTTCACTAT  281
            ||||||||||||||||||||||||||||||||||||||||||||||||||||||||||||
Sbjct  184  CAGATATAGCATTTCCACGAATAAATAATATAAGATTTTGACTTTTACCTCCTTCACTAT  243

Query  282  TTTTATTAATTAACTCTTCATTAATTGAATCTGGAGCTGGTACAGGATGAACAGTTTACC  341
            ||||||||||||||||||||||||||||||||||||||||||||||||||||||||||||
Sbjct  244  TTTTATTAATTAACTCTTCATTAATTGAATCTGGAGCTGGTACAGGATGAACAGTTTACC  303

Query  342  CTCCTCTATCTTCAAATTTATCACATTATGGCCCTTCAGTAGATTTAGCTATTTTTTCTC  401
            ||||||||||||||||||||||||||||||||||||||||||||||||||||||||||||
Sbjct  304  CTCCTCTATCTTCAAATTTATCACATTATGGCCCTTCAGTAGATTTAGCTATTTTTTCTC  363

Query  402  TTCATCTTGCTGGTGCTTCTTCAATTTTAGGTGCAATTAACTTTATTACAACTATTGTAA  461
            ||||||||||||||||||||||||||||||||||||||||||||||||||||||||||||
Sbjct  364  TTCATCTTGCTGGTGCTTCTTCAATTTTAGGTGCAATTAACTTTATTACAACTATTGTAA  423

Query  462  ACATACGATCAATTGGAATAACAATAGAACGTATACCATTATTTGTTTGATCTGTTTTAA  521
            ||||||||||||||||||||||||||||||||||||||||||||||||||||||||||||
Sbjct  424  ACATACGATCAATTGGAATAACAATAGAACGTATACCATTATTTGTTTGATCTGTTTTAA  483

Query  522  TTACAGCTATTTTATTACTTTTATCTTTACCTGTATTAGCAGGTGCCATTACAATATTGT  581
            ||||||||||||||||||||||||||||||||||||||||||||||||||||||||||||
Sbjct  484  TTACAGCTATTTTATTACTTTTATCTTTACCTGTATTAGCAGGTGCCATTACAATATTGT  543

Query  582  TAACTGATCGAAACTTTAATACATCATTTTTTGATCCTTCAGGGGGAGGAGATCCAATTT  641
            ||||||||||||||||||||||||||||||||||||||||||||||||||||||||||||
Sbjct  544  TAACTGATCGAAACTTTAATACATCATTTTTTGATCCTTCAGGGGGAGGAGATCCAATTT  603

Query  642  TATATCAACATTTATTT  658
            |||||||||||||||||
Sbjct  604  TATATCAACATTTATTT  620
```

>Rhipicephalus sanguineus isolate SV2 cytochrome c oxidase subunit I (COX1) gene, partial cds; mitochondrial
Sequence ID: MW135447.1 Length: 630
Range 1: 1 to 620

Score:1140 bits(617), Expect:0.0,
Identities:619/620(99%), Gaps:0/620(0%), Strand: Plus/Plus

```
Query  39   TAGGACTTAGTATAAGAATATTAATTCGTATAGAATTAGGACAACCTGGAACTTTAATTG  98
            ||||||||||||||||||||||||||||||||||||||||||||||||||||||||||||
Sbjct  1    TAGGACTTAGTATAAGAATATTAATTCGTATAGAATTAGGACAACCTGGAACTTTAATTG  60

Query  99   GAAATGATCAGATTTATAATGTAATCGTAACAGCACATGCATTTATTATAAttttttttA  158
            ||||||||||||||||||||||||||||||||||||||||||||||||||||||||||||
Sbjct  61   GAAATGATCAGATTTATAATGTAATCGTAACAGCACATGCATTTATTATAATTTTTTTTA  120
```

```
Query  159   TAGTTATACCAATCATAATCGGAGGATTTGGAAATTGATTAGTCCCTATCATATTGGGTG  218
             ||||||||||||||||||||||||||||||||||||||||||||||||||||||||||||
Sbjct  121   TAGTTATACCAATCATAATCGGAGGATTTGGAAATTGATTAGTCCCTATCATATTGGGTG  180

Query  219   CCCCAGATATAGCATTTCCACGAATAAATAATATAAGATTTTGACTTTTACCTCCTTCAC  278
             ||||||||||||||||||||||||||||||||||||||||||||||||||||||||||||
Sbjct  181   CCCCAGATATAGCATTTCCACGAATAAATAATATAAGATTTTGACTTTTACCTCCTTCAC  240

Query  279   TATTTTTATTAATTAACTCTTCATTAATTGAATCTGGAGCTGGTACAGGATGAACAGTTT  338
             ||||||||||||||||||||||||||||||||||||||||||||||||||||||||||||
Sbjct  241   TATTTTTATTAATTAACTCTTCATTAATTGAATCTGGAGCTGGTACAGGATGAACAGTTT  300

Query  339   ACCCTCCTCTATCTTCAAATTTATCACATTATGGCCCTTCAGTAGATTTAGCTATTTTTT  398
             ||||||||||||||||||||||||||||||||||||||||||||||||||||||||||||
Sbjct  301   ACCCTCCTCTATCTTCAAATTTATCACATTATGGCCCTTCAGTAGATTTAGCTATTTTTT  360

Query  399   CTCTTCATCTTGCTGGTGCTTCTTCAATTTTAGGTGCAATTAACTTTATTACAACTATTG  458
             ||||||||||||||||||||||||||||||||||||||||||||||||||||||||||||
Sbjct  361   CTCTTCATCTTGCTGGTGCTTCTTCAATTTTAGGTGCAATTAACTTTATTACAACTATTG  420

Query  459   TAAACATACGATCAATTGGAATAACAATAGAACGTATACCATTATTTGTTTGATCTGTTT  518
             ||||||||||||||||||||||||||||||||||||||||||||||||||||||||||||
Sbjct  421   TAAACATACGATCAATTGGAATAACAATAGAACGTATACCATTATTTGTTTGATCTGTTT  480

Query  519   TAATTACAGCTATTTTATTACTTTTATCTTTACCTGTATTAGCAGGTGCCATTACAATAT  578
             ||||||||||||||||||||||||||||||||||||||||||||||||||||||||||||
Sbjct  481   TAATTACAGCTATTTTATTACTTTTATCTTTACCTGTATTAGCAGGTGCCATTACAATAT  540

Query  579   TGTTAACTGATCGAAACTTTAATACATCATTTTTTGATCCTTCAGGGGGAGGAGATCCAA  638
             ||||||||||||||||||||||||||||||||||||||||||||||||||||||| ||||
Sbjct  541   TGTTAACTGATCGAAACTTTAATACATCATTTTTTGATCCTTCAGGGGGAGGAGACCCAA  600

Query  639   TTTTATATCAACATTTATTT  658
             ||||||||||||||||||||
Sbjct  601   TTTTATATCAACATTTATTT  620
```

>Rhipicephalus sanguineus sensu lato 'temperate lineage' isolate S475 cytochrome c oxidase subunit I (COI) gene, partial cds; mitochondrial
Sequence ID: MF426014.1 Length: 649
Range 1: 28 to 649

Score:1138 bits(616), Expect:0.0,
Identities:620/622(99%), Gaps:0/622(0%), Strand: Plus/Plus

```
Query  1     AACAATATATTTAATTTTTGGAGCATGATCAGGTATATTAGGACTTAGTATAAGAATATT  60
             ||||||||||||||||||||||||||||||||||||||||||||||||||||||||||||
Sbjct  28    AACAATATATTTAATTTTTGGAGCATGATCAGGTATATTAGGACTTAGTATAAGAATATT  87

Query  61    AATTCGTATAGAATTAGGACAACCTGGAACTTTAATTGGAAATGATCAGATTTATAATGT  120
             ||||||||||||||||||||||||||||||||||||||||||||||||||||||||||||
Sbjct  88    AATTCGTATAGAATTAGGACAACCTGGAACTTTAATTGGAAATGATCAGATTTATAATGT  147
```

```
Query  121  AATCGTAACAGCACATGCATTTATTATAAttttttttATAGTTATACCAATCATAATCGG  180
            ||||||||||||||||||||||||||||||||||||||||||||||||||||||||||||
Sbjct  148  AATCGTAACAGCACATGCATTTATTATAATTTTTTTTATAGTTATACCAATCATAATCGG  207

Query  181  AGGATTTGGAAATTGATTAGTCCCTATCATATTGGGTGCCCCAGATATAGCATTTCCACG  240
            ||||||||||||||||||||||||||||||||||||||||||||||||||||||||||||
Sbjct  208  AGGATTTGGAAATTGATTAGTCCCTATCATATTGGGTGCCCCAGATATAGCATTTCCACG  267

Query  241  AATAAATAATATAAGATTTTGACTTTTACCTCCTTCACTATTTTTATTAATTAACTCTTC  300
            ||||||||||||||||||||||||||||||||||||||||||||||||||||||||||||
Sbjct  268  AATAAATAATATAAGATTTTGACTTTTACCTCCTTCACTATTTTTATTAATTAACTCTTC  327

Query  301  ATTAATTGAATCTGGAGCTGGTACAGGATGAACAGTTTACCCTCCTCTATCTTCAAATTT  360
            ||||||||||||||||||||||||||||||||||||||||||||||||||||||||||||
Sbjct  328  ATTAATTGAATCTGGAGCTGGTACAGGATGAACAGTTTACCCTCCTCTATCTTCAAATTT  387

Query  361  ATCACATTATGGCCCTTCAGTAGATTTAGCTATTTTTTCTCTTCATCTTGCTGGTGCTTC  420
            |||||||||||| |||||||||||||||||||||||||||||||||||||||||||||||
Sbjct  388  ATCACATTATGGTCCTTCAGTAGATTTAGCTATTTTTTCTCTTCATCTTGCTGGTGCTTC  447

Query  421  TTCAATTTTAGGTGCAATTAACTTTATTACAACTATTGTAAACATACGATCAATTGGAAT  480
            ||||||||||||||||||||||||||||||||||||||||||||||||||||||||||||
Sbjct  448  TTCAATTTTAGGTGCAATTAACTTTATTACAACTATTGTAAACATACGATCAATTGGAAT  507

Query  481  AACAATAGAACGTATACCATTATTTGTTTGATCTGTTTTAATTACAGCTATTTTATTACT  540
            ||||||||||||||||||||||||||||||||||||||||||||||||||||||| ||||
Sbjct  508  AACAATAGAACGTATACCATTATTTGTTTGATCTGTTTTAATTACAGCTATTTTACTACT  567

Query  541  TTTATCTTTACCTGTATTAGCAGGTGCCATTACAATATTGTTAACTGATCGAAACTTTAA  600
            ||||||||||||||||||||||||||||||||||||||||||||||||||||||||||||
Sbjct  568  TTTATCTTTACCTGTATTAGCAGGTGCCATTACAATATTGTTAACTGATCGAAACTTTAA  627

Query  601  TACATCATTTTTTGATCCTTCA  622
            ||||||||||||||||||||||
Sbjct  628  TACATCATTTTTTGATCCTTCA  649
```

>Rhipicephalus sanguineus clone #2 cytochrome oxidase subunit I (COI) gene, partial cds; mitochondrial
Sequence ID: KX519707.1 Length: 626
Range 1: 1 to 616

Score:1138 bits(616), Expect:0.0,
Identities:616/616(100%), Gaps:0/616(0%), Strand: Plus/Plus

```
Query  43   ACTTAGTATAAGAATATTAATTCGTATAGAATTAGGACAACCTGGAACTTTAATTGGAAA  102
            ||||||||||||||||||||||||||||||||||||||||||||||||||||||||||||
Sbjct  1    ACTTAGTATAAGAATATTAATTCGTATAGAATTAGGACAACCTGGAACTTTAATTGGAAA  60

Query  103  TGATCAGATTTATAATGTAATCGTAACAGCACATGCATTTATTATAAttttttttATAGT  162
            ||||||||||||||||||||||||||||||||||||||||||||||||||||||||||||
```

```
Sbjct  61    TGATCAGATTTATAATGTAATCGTAACAGCACATGCATTTATTATAATTTTTTTTATAGT  120

Query  163   TATACCAATCATAATCGGAGGATTTGGAAATTGATTAGTCCCTATCATATTGGGTGCCCC  222
             ||||||||||||||||||||||||||||||||||||||||||||||||||||||||||||
Sbjct  121   TATACCAATCATAATCGGAGGATTTGGAAATTGATTAGTCCCTATCATATTGGGTGCCCC  180

Query  223   AGATATAGCATTTCCACGAATAAATAATATAAGATTTTGACTTTTACCTCCTTCACTATT  282
             ||||||||||||||||||||||||||||||||||||||||||||||||||||||||||||
Sbjct  181   AGATATAGCATTTCCACGAATAAATAATATAAGATTTTGACTTTTACCTCCTTCACTATT  240

Query  283   TTTATTAATTAACTCTTCATTAATTGAATCTGGAGCTGGTACAGGATGAACAGTTTACCC  342
             ||||||||||||||||||||||||||||||||||||||||||||||||||||||||||||
Sbjct  241   TTTATTAATTAACTCTTCATTAATTGAATCTGGAGCTGGTACAGGATGAACAGTTTACCC  300

Query  343   TCCTCTATCTTCAAATTTATCACATTATGGCCCTTCAGTAGATTTAGCTATTTTTTCTCT  402
             ||||||||||||||||||||||||||||||||||||||||||||||||||||||||||||
Sbjct  301   TCCTCTATCTTCAAATTTATCACATTATGGCCCTTCAGTAGATTTAGCTATTTTTTCTCT  360

Query  403   TCATCTTGCTGGTGCTTCTTCAATTTTAGGTGCAATTAACTTTATTACAACTATTGTAAA  462
             ||||||||||||||||||||||||||||||||||||||||||||||||||||||||||||
Sbjct  361   TCATCTTGCTGGTGCTTCTTCAATTTTAGGTGCAATTAACTTTATTACAACTATTGTAAA  420

Query  463   CATACGATCAATTGGAATAACAATAGAACGTATACCATTATTTGTTTGATCTGTTTTAAT  522
             ||||||||||||||||||||||||||||||||||||||||||||||||||||||||||||
Sbjct  421   CATACGATCAATTGGAATAACAATAGAACGTATACCATTATTTGTTTGATCTGTTTTAAT  480

Query  523   TACAGCTATTTTATTACTTTTATCTTTACCTGTATTAGCAGGTGCCATTACAATATTGTT  582
             ||||||||||||||||||||||||||||||||||||||||||||||||||||||||||||
Sbjct  481   TACAGCTATTTTATTACTTTTATCTTTACCTGTATTAGCAGGTGCCATTACAATATTGTT  540

Query  583   AACTGATCGAAACTTTAATACATCATTTTTTGATCCTTCAGGGGGAGGAGATCCAATTTT  642
             ||||||||||||||||||||||||||||||||||||||||||||||||||||||||||||
Sbjct  541   AACTGATCGAAACTTTAATACATCATTTTTTGATCCTTCAGGGGGAGGAGATCCAATTTT  600

Query  643   ATATCAACATTTATTT  658
             ||||||||||||||||
Sbjct  601   ATATCAACATTTATTT  616
```

>Rhipicephalus sanguineus isolate Szekszard cytochrome oxidase subunit I (COI) gene, partial cds; mitochondrial
Sequence ID: KX757901.1 Length: 630
Range 1: 1 to 620

Score:1134 bits(614), Expect:0.0,
Identities:618/620(99%), Gaps:0/620(0%), Strand: Plus/Plus

```
Query  39    TAGGACTTAGTATAAGAATATTAATTCGTATAGAATTAGGACAACCTGGAACTTTAATTG  98
             ||||||||||||||||||||||||||||||||||||||||||||||||||||||||||||
Sbjct  1     TAGGACTTAGTATAAGAATATTAATTCGTATAGAATTAGGACAACCTGGAACTTTAATTG  60

Query  99    GAAATGATCAGATTTATAATGTAATCGTAACAGCACATGCATTTATTATAAttttttttA  158
```

```
            ||||||||||||||||||||||||||||||||||||||||||||||||||||||||||||
Sbjct  61   GAAATGATCAGATTTATAATGTAATCGTAACAGCACATGCATTTATTATAATTTTTTTTA  120

Query  159  TAGTTATACCAATCATAATCGGAGGATTTGGAAATTGATTAGTCCCTATCATATTGGGTG  218
            ||||||||||||||||||||||||||||||||||||||||||||||||||||||||||||
Sbjct  121  TAGTTATACCAATCATAATCGGAGGATTTGGAAATTGATTAGTCCCTATCATATTGGGTG  180

Query  219  CCCCAGATATAGCATTTCCACGAATAAATAATATAAGATTTTGACTTTTACCTCCTTCAC  278
            ||||||||||||||||||||||||||||||||||||||||||||||||||||||||||||
Sbjct  181  CCCCAGATATAGCATTTCCACGAATAAATAATATAAGATTTTGACTTTTACCTCCTTCAC  240

Query  279  TATTTTTATTAATTAACTCTTCATTAATTGAATCTGGAGCTGGTACAGGATGAACAGTTT  338
            ||||||||||||||||||||||||||||||||||||||||||||||||||||||||||||
Sbjct  241  TATTTTTATTAATTAACTCTTCATTAATTGAATCTGGAGCTGGTACAGGATGAACAGTTT  300

Query  339  ACCCTCCTCTATCTTCAAATTTATCACATTATGGCCCTTCAGTAGATTTAGCTATTTTTT  398
            ||||||||||||||||||||||||||||||||||||||||||||||||||||||| ||||
Sbjct  301  ACCCTCCTCTATCTTCAAATTTATCACATTATGGCCCTTCAGTAGATTTAGCTATCTTTT  360

Query  399  CTCTTCATCTTGCTGGTGCTTCTTCAATTTTAGGTGCAATTAACTTTATTACAACTATTG  458
            ||||||||||||||||||||||||||||||||||||||||||||||||||||||||||||
Sbjct  361  CTCTTCATCTTGCTGGTGCTTCTTCAATTTTAGGTGCAATTAACTTTATTACAACTATTG  420

Query  459  TAAACATACGATCAATTGGAATAACAATAGAACGTATACCATTATTTGTTTGATCTGTTT  518
            |||||||||| |||||||||||||||||||||||||||||||||||||||||||||||||
Sbjct  421  TAAACATACGGTCAATTGGAATAACAATAGAACGTATACCATTATTTGTTTGATCTGTTT  480

Query  519  TAATTACAGCTATTTTATTACTTTTATCTTTACCTGTATTAGCAGGTGCCATTACAATAT  578
            ||||||||||||||||||||||||||||||||||||||||||||||||||||||||||||
Sbjct  481  TAATTACAGCTATTTTATTACTTTTATCTTTACCTGTATTAGCAGGTGCCATTACAATAT  540

Query  579  TGTTAACTGATCGAAACTTTAATACATCATTTTTTGATCCTTCAGGGGGAGGAGATCCAA  638
            ||||||||||||||||||||||||||||||||||||||||||||||||||||||||||||
Sbjct  541  TGTTAACTGATCGAAACTTTAATACATCATTTTTTGATCCTTCAGGGGGAGGAGATCCAA  600

Query  639  TTTTATATCAACATTTATTT  658
            ||||||||||||||||||||
Sbjct  601  TTTTATATCAACATTTATTT  620
```

>Rhipicephalus sanguineus voucher Rs7 cytochrome oxidase subunit I (COI) gene, partial cds; mitochondrial
Sequence ID: KU568516.1 Length: 656
Range 1: 27 to 656

Score:1131 bits(612), Expect:0.0,
Identities:624/630(99%), Gaps:0/630(0%), Strand: Plus/Plus

```
Query  1    AACAATATATTTAATTTTTGGAGCATGATCAGGTATATTAGGACTTAGTATAAGAATATT  60
            ||||||||||||||||||||||||||||||||||||||||||||||||||||||||||||
Sbjct  27   AACAATATATTTAATTTTTGGAGCATGATCAGGTATATTAGGACTTAGTATAAGAATATT  86
```

```
Query  61    AATTCGTATAGAATTAGGACAACCTGGAACTTTAATTGGAAATGATCAGATTTATAATGT  120
             ||||||||||||||||||||||||||||||||||||||||||||||||||||||||||||
Sbjct  87    AATTCGTATAGAATTAGGACAACCTGGAACTTTAATTGGAAATGATCAGATTTATAATGT  146

Query  121   AATCGTAACAGCACATGCATTTATTATAAttttttttATAGTTATACCAATCATAATCGG  180
             ||||||||||||||||||||||||||||||||||||||||||||||||||||||||||||
Sbjct  147   AATCGTAACAGCACATGCATTTATTATAATTTTTTTTATAGTTATACCAATCATAATCGG  206

Query  181   AGGATTTGGAAATTGATTAGTCCCTATCATATTGGGTGCCCCAGATATAGCATTTCCACG  240
             ||||||||||||||||||| ||||||||||||| ||||||||||||||||||||||||||
Sbjct  207   AGGATTTGGAAATTGATTAATCCCTATCATATTAGGTGCCCCAGATATAGCATTTCCACG  266

Query  241   AATAAATAATATAAGATTTTGACTTTTACCTCCTTCACTATTTTTATTAATTAACTCTTC  300
             ||||||||||||||||||||||||||||||||||||||||||||||||||||||||||||
Sbjct  267   AATAAATAATATAAGATTTTGACTTTTACCTCCTTCACTATTTTTATTAATTAACTCTTC  326

Query  301   ATTAATTGAATCTGGAGCTGGTACAGGATGAACAGTTTACCCTCCTCTATCTTCAAATTT  360
             ||||||||||||||||||||||||||||||||||||||||||||||||||||||||||||
Sbjct  327   ATTAATTGAATCTGGAGCTGGTACAGGATGAACAGTTTACCCTCCTCTATCTTCAAATTT  386

Query  361   ATCACATTATGGCCCTTCAGTAGATTTAGCTATTTTTTCTCTTCATCTTGCTGGTGCTTC  420
             ||||||||||||||||||||||||||||||||| ||||||||||||||||||||||||||
Sbjct  387   ATCACATTATGGCCCTTCAGTAGATTTAGCTATCTTTTCTCTTCATCTTGCTGGTGCTTC  446

Query  421   TTCAATTTTAGGTGCAATTAACTTTATTACAACTATTGTAAACATACGATCAATTGGAAT  480
             ||||||||||||||||||||||||||||||||||||||||||||||||||||||||||||
Sbjct  447   TTCAATTTTAGGTGCAATTAACTTTATTACAACTATTGTAAACATACGATCAATTGGAAT  506

Query  481   AACAATAGAACGTATACCATTATTTGTTTGATCTGTTTTAATTACAGCTATTTTATTACT  540
             ||||||||||||||||||||||||||||||||||||||||||||||||||||||||||||
Sbjct  507   AACAATAGAACGTATACCATTATTTGTTTGATCTGTTTTAATTACAGCTATTTTATTACT  566

Query  541   TTTATCTTTACCTGTATTAGCAGGTGCCATTACAATATTGTTAACTGATCGAAACTTTAA  600
              |||||||||||||||||||||||||||||||||||||||||||||||||||||||||||
Sbjct  567   CTTATCTTTACCTGTATTAGCAGGTGCCATTACAATATTGTTAACTGATCGAAACTTTAA  626

Query  601   TACATCATTTTTTGATCCTTCAGGGGGAGG  630
             ||||||||||||||| |||||||| |||||
Sbjct  627   TACATCATTTTTTGACCCTTCAGGAGGAGG  656
```

>Rhipicephalus sanguineus clone #4 cytochrome oxidase subunit I (COI) gene, partial cds; mitochondrial
Sequence ID: KX519709.1 Length: 635
Range 1: 2 to 621

Score:1129 bits(611), Expect:0.0,
Identities:617/620(99%), Gaps:0/620(0%), Strand: Plus/Plus

```
Query  39    TAGGACTTAGTATAAGAATATTAATTCGTATAGAATTAGGACAACCTGGAACTTTAATTG  98
             ||||||||||||||||||||||||||||||||||||||||||||||||||||||||||||
Sbjct  2     TAGGACTTAGTATAAGAATATTAATTCGTATAGAATTAGGACAACCTGGAACTTTAATTG  61
```

```
Query  99   GAAATGATCAGATTTATAATGTAATCGTAACAGCACATGCATTTATTATAAttttttttA  158
            |||||||||||||||||||||||||||||||||||||||| |||||||||||||||||||
Sbjct  62   GAAATGATCAGATTTATAATGTAATCGTAACAGCACATGCTTTTATTATAATTTTTTTTA  121

Query  159  TAGTTATACCAATCATAATCGGAGGATTTGGAAATTGATTAGTCCCTATCATATTGGGTG  218
            ||||||||||||||||||||||||||||||||||||||||||||||||||||||||||||
Sbjct  122  TAGTTATACCAATCATAATCGGAGGATTTGGAAATTGATTAGTCCCTATCATATTGGGTG  181

Query  219  CCCCAGATATAGCATTTCCACGAATAAATAATATAAGATTTTGACTTTTACCTCCTTCAC  278
            |||| |||||||||||||||||||||||||||||||||||||||||||||||||||||||
Sbjct  182  CCCCGGATATAGCATTTCCACGAATAAATAATATAAGATTTTGACTTTTACCTCCTTCAC  241

Query  279  TATTTTTATTAATTAACTCTTCATTAATTGAATCTGGAGCTGGTACAGGATGAACAGTTT  338
            ||||||||||||||||||||||||||||||||||||||||||||||||||||||||||||
Sbjct  242  TATTTTTATTAATTAACTCTTCATTAATTGAATCTGGAGCTGGTACAGGATGAACAGTTT  301

Query  339  ACCCTCCTCTATCTTCAAATTTATCACATTATGGCCCTTCAGTAGATTTAGCTATTTTTT  398
            |||||||||||||||||||||||||||||||||||||||||| |||||||||||||||||
Sbjct  302  ACCCTCCTCTATCTTCAAATTTATCACATTATGGCCCTTCAGGAGATTTAGCTATTTTTT  361

Query  399  CTCTTCATCTTGCTGGTGCTTCTTCAATTTTAGGTGCAATTAACTTTATTACAACTATTG  458
            ||||||||||||||||||||||||||||||||||||||||||||||||||||||||||||
Sbjct  362  CTCTTCATCTTGCTGGTGCTTCTTCAATTTTAGGTGCAATTAACTTTATTACAACTATTG  421

Query  459  TAAACATACGATCAATTGGAATAACAATAGAACGTATACCATTATTTGTTTGATCTGTTT  518
            ||||||||||||||||||||||||||||||||||||||||||||||||||||||||||||
Sbjct  422  TAAACATACGATCAATTGGAATAACAATAGAACGTATACCATTATTTGTTTGATCTGTTT  481

Query  519  TAATTACAGCTATTTTATTACTTTTATCTTTACCTGTATTAGCAGGTGCCATTACAATAT  578
            ||||||||||||||||||||||||||||||||||||||||||||||||||||||||||||
Sbjct  482  TAATTACAGCTATTTTATTACTTTTATCTTTACCTGTATTAGCAGGTGCCATTACAATAT  541

Query  579  TGTTAACTGATCGAAACTTTAATACATCATTTTTTGATCCTTCAGGGGGAGGAGATCCAA  638
            ||||||||||||||||||||||||||||||||||||||||||||||||||||||||||||
Sbjct  542  TGTTAACTGATCGAAACTTTAATACATCATTTTTTGATCCTTCAGGGGGAGGAGATCCAA  601

Query  639  TTTTATATCAACATTTATTT  658
            ||||||||||||||||||||
Sbjct  602  TTTTATATCAACATTTATTT  621
```

>Rhipicephalus sanguineus isolate LIC4723B cytochrome oxidase subunit 1 (COI) gene, partial cds; mitochondrial
Sequence ID: KX714599.1 Length: 723
>Rhipicephalus sanguineus isolate LIC4750B cytochrome oxidase subunit 1 (COI) gene, partial cds; mitochondrial
Sequence ID: KX714600.1 Length: 723
Range 1: 1 to 597

Score:1092 bits(591), Expect:0.0,
Identities:595/597(99%), Gaps:0/597(0%), Strand: Plus/Plus

```
Query  62    ATTCGTATAGAATTAGGACAACCTGGAACTTTAATTGGAAATGATCAGATTTATAATGTA  121
             ||||||||||||||||||||||||||||||||||||||||||||||||||||||||||||
Sbjct  1     ATTCGTATAGAATTAGGACAACCTGGAACTTTAATTGGAAATGATCAGATTTATAATGTA  60

Query  122   ATCGTAACAGCACATGCATTTATTATAAttttttttATAGTTATACCAATCATAATCGGA  181
             |||||||||||||||||||||||||||||||||||||||||||||||||||||||| |||
Sbjct  61    ATCGTAACAGCACATGCATTTATTATAATTTTTTTTATAGTTATACCAATCATAATTGGA  120

Query  182   GGATTTGGAAATTGATTAGTCCCTATCATATTGGGTGCCCCAGATATAGCATTTCCACGA  241
             ||||||||||||||||||||||||||||||||||||||||||||||||||||||||||||
Sbjct  121   GGATTTGGAAATTGATTAGTCCCTATCATATTGGGTGCCCCAGATATAGCATTTCCACGA  180

Query  242   ATAAATAATATAAGATTTTGACTTTTACCTCCTTCACTATTTTTATTAATTAACTCTTCA  301
             ||||||||||||||||||||||||||||||||||||||||||||||||||||||||||||
Sbjct  181   ATAAATAATATAAGATTTTGACTTTTACCTCCTTCACTATTTTTATTAATTAACTCTTCA  240

Query  302   TTAATTGAATCTGGAGCTGGTACAGGATGAACAGTTTACCCTCCTCTATCTTCAAATTTA  361
             ||||||||||||||||||||||||||||||||||||||||||||||||||||||||||||
Sbjct  241   TTAATTGAATCTGGAGCTGGTACAGGATGAACAGTTTACCCTCCTCTATCTTCAAATTTA  300

Query  362   TCACATTATGGCCCTTCAGTAGATTTAGCTATTTTTTCTCTTCATCTTGCTGGTGCTTCT  421
             |||||||||||||||||||||||||||||||| |||||||||||||||||||||||||||
Sbjct  301   TCACATTATGGCCCTTCAGTAGATTTAGCTATCTTTTCTCTTCATCTTGCTGGTGCTTCT  360

Query  422   TCAATTTTAGGTGCAATTAACTTTATTACAACTATTGTAAACATACGATCAATTGGAATA  481
             ||||||||||||||||||||||||||||||||||||||||||||||||||||||||||||
Sbjct  361   TCAATTTTAGGTGCAATTAACTTTATTACAACTATTGTAAACATACGATCAATTGGAATA  420

Query  482   ACAATAGAACGTATACCATTATTTGTTTGATCTGTTTTAATTACAGCTATTTTATTACTT  541
             ||||||||||||||||||||||||||||||||||||||||||||||||||||||||||||
Sbjct  421   ACAATAGAACGTATACCATTATTTGTTTGATCTGTTTTAATTACAGCTATTTTATTACTT  480

Query  542   TTATCTTTACCTGTATTAGCAGGTGCCATTACAATATTGTTAACTGATCGAAACTTTAAT  601
             ||||||||||||||||||||||||||||||||||||||||||||||||||||||||||||
Sbjct  481   TTATCTTTACCTGTATTAGCAGGTGCCATTACAATATTGTTAACTGATCGAAACTTTAAT  540

Query  602   ACATCATTTTTTGATCCTTCAGGGGGAGGAGATCCAATTTTATATCAACATTTATTT  658
             |||||||||||||||||||||||||||||||||||||||||||||||||||||||||
Sbjct  541   ACATCATTTTTTGATCCTTCAGGGGGAGGAGATCCAATTTTATATCAACATTTATTT  597
```

>Rhipicephalus sanguineus clone #8 cytochrome oxidase subunit I (COI) gene, partial cds; mitochondrial
Sequence ID: KX519713.1 Length: 580
Range 1: 1 to 580

Score:1072 bits(580), Expect:0.0,
Identities:580/580(100%), Gaps:0/580(0%), Strand: Plus/Plus

```
Query  43    ACTTAGTATAAGAATATTAATTCGTATAGAATTAGGACAACCTGGAACTTTAATTGGAAA  102
             ||||||||||||||||||||||||||||||||||||||||||||||||||||||||||||
```

```
Sbjct  1     ACTTAGTATAAGAATATTAATTCGTATAGAATTAGGACAACCTGGAACTTTAATTGGAAA  60

Query  103   TGATCAGATTTATAATGTAATCGTAACAGCACATGCATTTATTATAAttttttttATAGT  162
             ||||||||||||||||||||||||||||||||||||||||||||||||||||||||||||
Sbjct  61    TGATCAGATTTATAATGTAATCGTAACAGCACATGCATTTATTATAATTTTTTTTATAGT  120

Query  163   TATACCAATCATAATCGGAGGATTTGGAAATTGATTAGTCCCTATCATATTGGGTGCCCC  222
             ||||||||||||||||||||||||||||||||||||||||||||||||||||||||||||
Sbjct  121   TATACCAATCATAATCGGAGGATTTGGAAATTGATTAGTCCCTATCATATTGGGTGCCCC  180

Query  223   AGATATAGCATTTCCACGAATAAATAATATAAGATTTTGACTTTTACCTCCTTCACTATT  282
             ||||||||||||||||||||||||||||||||||||||||||||||||||||||||||||
Sbjct  181   AGATATAGCATTTCCACGAATAAATAATATAAGATTTTGACTTTTACCTCCTTCACTATT  240

Query  283   TTTATTAATTAACTCTTCATTAATTGAATCTGGAGCTGGTACAGGATGAACAGTTTACCC  342
             ||||||||||||||||||||||||||||||||||||||||||||||||||||||||||||
Sbjct  241   TTTATTAATTAACTCTTCATTAATTGAATCTGGAGCTGGTACAGGATGAACAGTTTACCC  300

Query  343   TCCTCTATCTTCAAATTTATCACATTATGGCCCTTCAGTAGATTTAGCTATTTTTTCTCT  402
             ||||||||||||||||||||||||||||||||||||||||||||||||||||||||||||
Sbjct  301   TCCTCTATCTTCAAATTTATCACATTATGGCCCTTCAGTAGATTTAGCTATTTTTTCTCT  360

Query  403   TCATCTTGCTGGTGCTTCTTCAATTTTAGGTGCAATTAACTTTATTACAACTATTGTAAA  462
             ||||||||||||||||||||||||||||||||||||||||||||||||||||||||||||
Sbjct  361   TCATCTTGCTGGTGCTTCTTCAATTTTAGGTGCAATTAACTTTATTACAACTATTGTAAA  420

Query  463   CATACGATCAATTGGAATAACAATAGAACGTATACCATTATTTGTTTGATCTGTTTTAAT  522
             ||||||||||||||||||||||||||||||||||||||||||||||||||||||||||||
Sbjct  421   CATACGATCAATTGGAATAACAATAGAACGTATACCATTATTTGTTTGATCTGTTTTAAT  480

Query  523   TACAGCTATTTTATTACTTTTATCTTTACCTGTATTAGCAGGTGCCATTACAATATTGTT  582
             ||||||||||||||||||||||||||||||||||||||||||||||||||||||||||||
Sbjct  481   TACAGCTATTTTATTACTTTTATCTTTACCTGTATTAGCAGGTGCCATTACAATATTGTT  540

Query  583   AACTGATCGAAACTTTAATACATCATTTTTTGATCCTTCA  622
             ||||||||||||||||||||||||||||||||||||||||
Sbjct  541   AACTGATCGAAACTTTAATACATCATTTTTTGATCCTTCA  580
```

>Rhipicephalus sanguineus mitochondrial DNA, complete genome
Sequence ID: AF081829.1 Length: 14710
Range 1: 1191 to 1848

Score:1061 bits(574), Expect:0.0,
Identities:630/658(96%), Gaps:0/658(0%), Strand: Plus/Plus

```
Query  1      AACAATATATTTAATTTTTGGAGCATGATCAGGTATATTAGGACTTAGTATAAGAATATT  60
              ||||||||||||||||||||||||||||||||||||||||||||||||||||||||||||
Sbjct  1191   AACAATATATTTAATTTTTGGAGCATGATCAGGTATATTAGGACTTAGTATAAGAATATT  1250

Query  61     AATTCGTATAGAATTAGGACAACCTGGAACTTTAATTGGAAATGATCAGATTTATAATGT  120
              ||||||||||||||||||||||||||||||||||||||||||||||||||||||||||||
```

```
Sbjct  1251  AATTCGTATAGAATTAGGACAACCTGGAACTTTAATTGGAAATGATCAGATTTATAATGT  1310

Query  121   AATCGTAACAGCACATGCATTTATTATAAttttttttATAGTTATACCAATCATAATCGG  180
             ||||||||||||||||||||||||||||||||| ||||||||||||||||||||||||||
Sbjct  1311  AATCGTAACAGCACATGCATTTATTATAATTTTCTTTATAGTTATACCAATCATAATCGG  1370

Query  181   AGGATTTGGAAATTGATTAGTCCCTATCATATTGGGTGCCCCAGATATAGCATTTCCACG  240
             ||| || |||||||||||||| ||||| ||||| ||||||||||||||||||||||||||
Sbjct  1371  AGGGTTCGGAAATTGATTAGTTCCTATTATATTAGGTGCCCCAGATATAGCATTTCCACG  1430

Query  241   AATAAATAATATAAGATTTTGACTTTTACCTCCTTCACTATTTTTATTAATTAACTCTTC  300
             |||||||||||||||||||||||||||||| || ||| |||||||||| |||||||||||
Sbjct  1431  AATAAATAATATAAGATTTTGACTTTTACCCCCTCATTATTTTTATTGATTAACTCTTC  1490

Query  301   ATTAATTGAATCTGGAGCTGGTACAGGATGAACAGTTTACCCTCCTCTATCTTCAAATTT  360
             |||||||||||| |||||||| ||||||||||||||||| || |||||||||||||||||
Sbjct  1491  ATTAATTGAATCCGGAGCTGGAACAGGATGAACAGTTTATCCCCCTCTATCTTCAAATTT  1550

Query  361   ATCACATTATGGCCCTTCAGTAGATTTAGCTATTTTTTCTCTTCATCTTGCTGGTGCTTC  420
             |||||||||||| || ||||||||||||||||||||||||||||| ||||||||||||||
Sbjct  1551  ATCACATTATGGTCCATCAGTAGATTTAGCTATTTTTTCTCTTCACCTTGCTGGTGCTTC  1610

Query  421   TTCAATTTTAGGTGCAATTAACTTTATTACAACTATTGTAAACATACGATCAATTGGAAT  480
             |||||||||||||||||||||||| || |||||||||||||| |||||||||||||||||
Sbjct  1611  TTCAATTTTAGGTGCAATTAACTTCATCACAACTATTGTAAATATACGATCAATTGGAAT  1670

Query  481   AACAATAGAACGTATACCATTATTTGTTTGATCTGTTTTAATTACAGCTATTTTATTACT  540
             |||||||||||| |||||||||||||||||||||||||||||||||||||||||| ||||
Sbjct  1671  AACAATAGAACGAATACCATTATTTGTTTGATCTGTTTTAATTACAGCTATTTTACTACT  1730

Query  541   TTTATCTTTACCTGTATTAGCAGGTGCCATTACAATATTGTTAACTGATCGAAACTTTAA  600
             | ||||||||||||||||||||||||||||||||||||| ||||||||||||||||||||
Sbjct  1731  TCTATCTTTACCTGTATTAGCAGGTGCCATTACAATATTATTAACTGATCGAAACTTTAA  1790

Query  601   TACATCATTTTTTGATCCTTCAGGGGGAGGAGATCCAATTTTATATCAACATTTATTT  658
              ||||||||||||||||||||||| ||||||||||| |||||||||||||| ||||||
Sbjct  1791  CACATCATTTTTTGATCCTTCAGGAGGAGGAGATCCCATTTTATATCAACACTTATTT  1848
```

>Rhipicephalus sanguineus clone #1 cytochrome oxidase subunit I (COI) gene, partial cds; mitochondrial
Sequence ID: KX519706.1 Length: 595
Range 1: 1 to 570

Score:1053 bits(570), Expect:0.0,
Identities:570/570(100%), Gaps:0/570(0%), Strand: Plus/Plus

```
Query  89   ACTTTAATTGGAAATGATCAGATTTATAATGTAATCGTAACAGCACATGCATTTATTATA  148
            ||||||||||||||||||||||||||||||||||||||||||||||||||||||||||||
Sbjct  1    ACTTTAATTGGAAATGATCAGATTTATAATGTAATCGTAACAGCACATGCATTTATTATA  60

Query  149  AttttttttATAGTTATACCAATCATAATCGGAGGATTTGGAAATTGATTAGTCCCTATC  208
```

```
            ||||||||||||||||||||||||||||||||||||||||||||||||||||||||||||
Sbjct  61   ATTTTTTTTATAGTTATACCAATCATAATCGGAGGATTTGGAAATTGATTAGTCCCTATC  120

Query  209  ATATTGGGTGCCCCAGATATAGCATTTCCACGAATAAATAATATAAGATTTTGACTTTTA  268
            ||||||||||||||||||||||||||||||||||||||||||||||||||||||||||||
Sbjct  121  ATATTGGGTGCCCCAGATATAGCATTTCCACGAATAAATAATATAAGATTTTGACTTTTA  180

Query  269  CCTCCTTCACTATTTTTATTAATTAACTCTTCATTAATTGAATCTGGAGCTGGTACAGGA  328
            ||||||||||||||||||||||||||||||||||||||||||||||||||||||||||||
Sbjct  181  CCTCCTTCACTATTTTTATTAATTAACTCTTCATTAATTGAATCTGGAGCTGGTACAGGA  240

Query  329  TGAACAGTTTACCCTCCTCTATCTTCAAATTTATCACATTATGGCCCTTCAGTAGATTTA  388
            ||||||||||||||||||||||||||||||||||||||||||||||||||||||||||||
Sbjct  241  TGAACAGTTTACCCTCCTCTATCTTCAAATTTATCACATTATGGCCCTTCAGTAGATTTA  300

Query  389  GCTATTTTTTCTCTTCATCTTGCTGGTGCTTCTTCAATTTTAGGTGCAATTAACTTTATT  448
            ||||||||||||||||||||||||||||||||||||||||||||||||||||||||||||
Sbjct  301  GCTATTTTTTCTCTTCATCTTGCTGGTGCTTCTTCAATTTTAGGTGCAATTAACTTTATT  360

Query  449  ACAACTATTGTAAACATACGATCAATTGGAATAACAATAGAACGTATACCATTATTTGTT  508
            ||||||||||||||||||||||||||||||||||||||||||||||||||||||||||||
Sbjct  361  ACAACTATTGTAAACATACGATCAATTGGAATAACAATAGAACGTATACCATTATTTGTT  420

Query  509  TGATCTGTTTTAATTACAGCTATTTTATTACTTTTATCTTTACCTGTATTAGCAGGTGCC  568
            ||||||||||||||||||||||||||||||||||||||||||||||||||||||||||||
Sbjct  421  TGATCTGTTTTAATTACAGCTATTTTATTACTTTTATCTTTACCTGTATTAGCAGGTGCC  480

Query  569  ATTACAATATTGTTAACTGATCGAAACTTTAATACATCATTTTTTGATCCTTCAGGGGGA  628
            ||||||||||||||||||||||||||||||||||||||||||||||||||||||||||||
Sbjct  481  ATTACAATATTGTTAACTGATCGAAACTTTAATACATCATTTTTTGATCCTTCAGGGGGA  540

Query  629  GGAGATCCAATTTTATATCAACATTTATTT  658
            ||||||||||||||||||||||||||||||
Sbjct  541  GGAGATCCAATTTTATATCAACATTTATTT  570
```

>Rhipicephalus sanguineus sensu lato 'temperate lineage' isolate SC3005 cytochrome c oxidase subunit I (COI) gene, partial cds; mitochondrial
Sequence ID: MF425987.1 Length: 574
Range 1: 1 to 561

Score:1014 bits(549), Expect:0.0,
Identities:557/561(99%), Gaps:0/561(0%), Strand: Plus/Plus

```
Query  98   GGAAATGATCAGATTTATAATGTAATCGTAACAGCACATGCATTTATTATAAtttttttt  157
            ||||||||||||||||||||||||||||||||||||||| ||||||||||||||||||||
Sbjct  1    GGAAATGATCAGATTTATAATGTAATCGTAACAGCACACGCATTTATTATAATTTTTTTT  60

Query  158  ATAGTTATACCAATCATAATCGGAGGATTTGGAAATTGATTAGTCCCTATCATATTGGGT  217
            |||||||||||||||||||||||||||||||||||||||||||||||||| ||||| |||
Sbjct  61   ATAGTTATACCAATCATAATCGGAGGATTTGGAAATTGATTAGTCCCTATTATATTAGGT  120
```

```
Query  218  GCCCCAGATATAGCATTTCCACGAATAAATAATATAAGATTTTGACTTTTACCTCCTTCA  277
            ||||||||||||||||||||||||||||||||||||||||||||||||||||||||||||
Sbjct  121  GCCCCAGATATAGCATTTCCACGAATAAATAATATAAGATTTTGACTTTTACCTCCTTCA  180

Query  278  CTATTTTTATTAATTAACTCTTCATTAATTGAATCTGGAGCTGGTACAGGATGAACAGTT  337
            ||||||||||||||||||||||||||||||||||||||||||||||||||||||||||||
Sbjct  181  CTATTTTTATTAATTAACTCTTCATTAATTGAATCTGGAGCTGGTACAGGATGAACAGTT  240

Query  338  TACCCTCCTCTATCTTCAAATTTATCACATTATGGCCCTTCAGTAGATTTAGCTATTTTT  397
            |||||||||||||||||||||||||||||||||||||||||||||||||||||||| |||
Sbjct  241  TACCCTCCTCTATCTTCAAATTTATCACATTATGGCCCTTCAGTAGATTTAGCTATCTTT  300

Query  398  TCTCTTCATCTTGCTGGTGCTTCTTCAATTTTAGGTGCAATTAACTTTATTACAACTATT  457
            ||||||||||||||||||||||||||||||||||||||||||||||||||||||||||||
Sbjct  301  TCTCTTCATCTTGCTGGTGCTTCTTCAATTTTAGGTGCAATTAACTTTATTACAACTATT  360

Query  458  GTAAACATACGATCAATTGGAATAACAATAGAACGTATACCATTATTTGTTTGATCTGTT  517
            ||||||||||||||||||||||||||||||||||||||||||||||||||||||||||||
Sbjct  361  GTAAACATACGATCAATTGGAATAACAATAGAACGTATACCATTATTTGTTTGATCTGTT  420

Query  518  TTAATTACAGCTATTTTATTACTTTTATCTTTACCTGTATTAGCAGGTGCCATTACAATA  577
            ||||||||||||||||||||||||||||||||||||||||||||||||||||||||||||
Sbjct  421  TTAATTACAGCTATTTTATTACTTTTATCTTTACCTGTATTAGCAGGTGCCATTACAATA  480

Query  578  TTGTTAACTGATCGAAACTTTAATACATCATTTTTTGATCCTTCAGGGGGAGGAGATCCA  637
            ||||||||||||||||||||||||||||||||||||||||||||||||||||||||||||
Sbjct  481  TTGTTAACTGATCGAAACTTTAATACATCATTTTTTGATCCTTCAGGGGGAGGAGATCCA  540

Query  638  ATTTTATATCAACATTTATTT  658
            |||||||||||||||||||||
Sbjct  541  ATTTTATATCAACATTTATTT  561
```

>Rhipicephalus sanguineus clone #5 cytochrome oxidase subunit I (COI) gene, partial cds; mitochondrial
Sequence ID: KX519710.1 Length: 553
Range 1: 1 to 547

Score:989 bits(535), Expect:0.0,
Identities:543/547(99%), Gaps:0/547(0%), Strand: Plus/Plus

```
Query  77   GGACAACCTGGAACTTTAATTGGAAATGATCAGATTTATAATGTAATCGTAACAGCACAT  136
            ||||||||||||||||||||||||||||||||||||||||||| ||||||||||||||||
Sbjct  1    GGACAACCTGGAACTTTAATTGGAAATGATCAGATTTATAATGGAATCGTAACAGCACAT  60

Query  137  GCATTTATTATAAtttttttATAGTTATACCAATCATAATCGGAGGATTTGGAAATTGA  196
            || |||||||||||||||||||||||||||||||||||||||||||||||||||||||||
Sbjct  61   GCTTTTATTATAATTTTTTTATAGTTATACCAATCATAATCGGAGGATTTGGAAATTGA  120

Query  197  TTAGTCCCTATCATATTGGGTGCCCCAGATATAGCATTTCCACGAATAAATAATATAAGA  256
            ||||||||||||||||||||||||||||||||||||||||||||||||||||||||||||
Sbjct  121  TTAGTCCCTATCATATTGGGTGCCCCAGATATAGCATTTCCACGAATAAATAATATAAGA  180
```

```
Query  257   TTTTGACTTTTACCTCCTTCACTATTTTTATTAATTAACTCTTCATTAATTGAATCTGGA  316
             ||||||||||||||||||||||||||||||||||||||||||||||||||||||||||||
Sbjct  181   TTTTGACTTTTACCTCCTTCACTATTTTTATTAATTAACTCTTCATTAATTGAATCTGGA  240

Query  317   GCTGGTACAGGATGAACAGTTTACCCTCCTCTATCTTCAAATTTATCACATTATGGCCCT  376
             ||||||||||||||||||||||||||||||||||||||||||||||||||||||||||||
Sbjct  241   GCTGGTACAGGATGAACAGTTTACCCTCCTCTATCTTCAAATTTATCACATTATGGCCCT  300

Query  377   TCAGTAGATTTAGCTATTTTTTCTCTTCATCTTGCTGGTGCTTCTTCAATTTTAGGTGCA  436
             |||| |||||||||||||||||||||||||||||||||||||||||||||||||||||||
Sbjct  301   TCAGGAGATTTAGCTATTTTTTCTCTTCATCTTGCTGGTGCTTCTTCAATTTTAGGTGCA  360

Query  437   ATTAACTTTATTACAACTATTGTAAACATACGATCAATTGGAATAACAATAGAACGTATA  496
             ||||||||||||||||||||||||||||||||||||||||||||||||||||||||||||
Sbjct  361   ATTAACTTTATTACAACTATTGTAAACATACGATCAATTGGAATAACAATAGAACGTATA  420

Query  497   CCATTATTTGTTTGATCTGTTTTAATTACAGCTATTTTATTACTTTTATCTTTACCTGTA  556
             ||||||||||||||||||||||||||||||||||||||||||||||||||||||||||||
Sbjct  421   CCATTATTTGTTTGATCTGTTTTAATTACAGCTATTTTATTACTTTTATCTTTACCTGTA  480

Query  557   TTAGCAGGTGCCATTACAATATTGTTAACTGATCGAAACTTTAATACATCATTTTTTGAT  616
             |||||||||||||||||||||||||||||||||||| |||||||||||||||||||||||
Sbjct  481   TTAGCAGGTGCCATTACAATATTGTTAACTGATCGATACTTTAATACATCATTTTTTGAT  540

Query  617   CCTTCAG  623
             |||||||
Sbjct  541   CCTTCAG  547
```

>Rhipicephalus sanguineus isolate Italy-A cytochrome oxidase subunit I (COI) gene, partial cds; mitochondrial
Sequence ID: KX757903.1 Length: 630
Range 1: 1 to 620

Score:985 bits(533), Expect:0.0,
Identities:591/620(95%), Gaps:0/620(0%), Strand: Plus/Plus

```
Query  39    TAGGACTTAGTATAAGAATATTAATTCGTATAGAATTAGGACAACCTGGAACTTTAATTG  98
             ||||||||||||||||||||||||||||||||||||||||||||||||||||||||||||
Sbjct  1     TAGGACTTAGTATAAGAATATTAATTCGTATAGAATTAGGACAACCTGGAACTTTAATTG  60

Query  99    GAAATGATCAGATTTATAATGTAATCGTAACAGCACATGCATTTATTATAAttttttttA  158
             ||||||||||||||||||||||||||||||||||||||||||||||||||||||| ||||
Sbjct  61    GAAATGATCAGATTTATAATGTAATCGTAACAGCACATGCATTTATTATAATTTTCTTTA  120

Query  159   TAGTTATACCAATCATAATCGGAGGATTTGGAAATTGATTAGTCCCTATCATATTGGGTG  218
             ||||||||||||||||||||||||| || |||||||||||||| ||||| ||||| ||||
Sbjct  121   TAGTTATACCAATCATAATCGGAGGGTTCGGAAATTGATTAGTTCCTATTATATTAGGTG  180

Query  219   CCCCAGATATAGCATTTCCACGAATAAATAATATAAGATTTTGACTTTTACCTCCTTCAC  278
             |||||||||||||||||||||||||||||||||||||||||||||||||||| || |||
```

```
Sbjct  181   CCCCAGATATAGCATTTCCACGAATAAATAATATAAGATTTTGACTTTTACCCCCCTCAT  240

Query  279   TATTTTTATTAATTAACTCTTCATTAATTGAATCTGGAGCTGGTACAGGATGAACAGTTT  338
             |||||||||| ||||| ||||||||||||||||| |||||||| ||||||||||||||||
Sbjct  241   TATTTTTATTGATTAATTCTTCATTAATTGAATCCGGAGCTGGAACAGGATGAACAGTTT  300

Query  339   ACCCTCCTCTATCTTCAAATTTATCACATTATGGCCCTTCAGTAGATTTAGCTATTTTTT  398
             | || ||||||||||||||||||||||||||||| || ||||||||||||||||||||||
Sbjct  301   ATCCCCCTCTATCTTCAAATTTATCACATTATGGTCCATCAGTAGATTTAGCTATTTTTT  360

Query  399   CTCTTCATCTTGCTGGTGCTTCTTCAATTTAGGTGCAATTAACTTTATTACAACTATTG  458
             ||||||| ||||||||||||||||||||||||||||||||||||| ||||||||||||||
Sbjct  361   CTCTTCACCTTGCTGGTGCTTCTTCAATTTAGGTGCAATTAACTTCATTACAACTATTG  420

Query  459   TAAACATACGATCAATTGGAATAACAATAGAACGTATACCATTATTTGTTTGATCTGTTT  518
             |||| ||||||||||||||||||||||||||||| |||||||||||||||||||||||||
Sbjct  421   TAAATATACGATCAATTGGAATAACAATAGAACGAATACCATTATTTGTTTGATCTGTTT  480

Query  519   TAATTACAGCTATTTTATTACTTTTATCTTTACCTGTATTAGCAGGTGCCATTACAATAT  578
             ||||||||||||||||| ||||| ||||||||||||||||||||||||||||||||||||
Sbjct  481   TAATTACAGCTATTTTACTACTTCTATCTTTACCTGTATTAGCAGGTGCCATTACAATAT  540

Query  579   TGTTAACTGATCGAAACTTTAATACATCATTTTTTGATCCTTCAGGGGGAGGAGATCCAA  638
             | |||||||||||||||||||| |||||||||||||| |||||||| ||||||||||| |
Sbjct  541   TATTAACTGATCGAAACTTTAACACATCATTTTTTGACCCTTCAGGAGGAGGAGATCCCA  600

Query  639   TTTTATATCAACATTTATTT  658
             ||||||||||||| ||||||
Sbjct  601   TTTTATATCAACACTTATTT  620



>Rhipicephalus sanguineus isolate Zagreb-B cytochrome oxidase subunit I (COI)
gene, partial cds; mitochondrial
Sequence ID: KX757893.1 Length: 629
Range 1: 1 to 620

Score:985 bits(533), Expect:0.0,
Identities:591/620(95%),  Gaps:0/620(0%), Strand: Plus/Plus

Query  39    TAGGACTTAGTATAAGAATATTAATTCGTATAGAATTAGGACAACCTGGAACTTTAATTG  98
             ||||||||||||||||||||||||||||||||||||||||||||||||||||||||||||
Sbjct  1     TAGGACTTAGTATAAGAATATTAATTCGTATAGAATTAGGACAACCTGGAACTTTAATTG  60

Query  99    GAAATGATCAGATTTATAATGTAATCGTAACAGCACATGCATTTATTATAAttttttttA  158
             ||||||||||||||||||||||||||||||||||||||||||||||||||||||| ||||
Sbjct  61    GAAATGATCAGATTTATAATGTAATCGTAACAGCACATGCATTTATTATAATTTTCTTTA  120

Query  159   TAGTTATACCAATCATAATCGGAGGATTTGGAAATTGATTAGTCCCTATCATATTGGGTG  218
             ||||||||||||||||||||||||| || ||||||||||||| |||||| ||||| ||||
Sbjct  121   TAGTTATACCAATCATAATCGGAGGGTTCGGAAATTGATTAGTTCCTATTATATTAGGTG  180

Query  219   CCCCAGATATAGCATTTCCACGAATAAATAATATAAGATTTTGACTTTTACCTCCTTCAC  278
```

```
                ||||||||||||||||||||||||||||||||||||||||||||||||||||| || |||
Sbjct  181  CCCCAGATATAGCATTTCCACGAATAAATAATATAAGATTTTGACTTTTACCCCCCTCAT  240

Query  279  TATTTTTATTAATTAACTCTTCATTAATTGAATCTGGAGCTGGTACAGGATGAACAGTTT  338
            |||||||||| ||||||||||||||||||||||| |||||||| ||||||||||||||||
Sbjct  241  TATTTTTATTGATTAACTCTTCATTAATTGAATCCGGAGCTGGAACAGGATGAACAGTTT  300

Query  339  ACCCTCCTCTATCTTCAAATTTATCACATTATGGCCCTTCAGTAGATTTAGCTATTTTTT  398
            | || ||||||||||||||||||||||||||||| || ||||||||||||||||||||||
Sbjct  301  ATCCCCCTCTATCTTCAAATTTATCACATTATGGTCCATCAGTAGATTTAGCTATTTTTT  360

Query  399  CTCTTCATCTTGCTGGTGCTTCTTCAATTTTAGGTGCAATTAACTTTATTACAACTATTG  458
            ||||||| |||||||||||||||||||||||||||||||||||||| || ||||||||||
Sbjct  361  CTCTTCACCTTGCTGGTGCTTCTTCAATTTTAGGTGCAATTAACTTCATCACAACTATTG  420

Query  459  TAAACATACGATCAATTGGAATAACAATAGAACGTATACCATTATTTGTTTGATCTGTTT  518
            |||| ||||||||||||||||||||||||||||| |||||||||||||||||||||||||
Sbjct  421  TAAATATACGATCAATTGGAATAACAATAGAACGAATACCATTATTTGTTTGATCTGTTT  480

Query  519  TAATTACAGCTATTTTATTACTTTTATCTTTACCTGTATTAGCAGGTGCCATTACAATAT  578
            ||||||||||||||||| ||||| ||||||||||||||||||||||||||||||||||||
Sbjct  481  TAATTACAGCTATTTTACTACTTCTATCTTTACCTGTATTAGCAGGTGCCATTACAATAT  540

Query  579  TGTTAACTGATCGAAACTTTAATACATCATTTTTTGATCCTTCAGGGGGAGGAGATCCAA  638
            | |||||||||||||||||||| ||||||||||||| ||||||||| ||||||||||| |
Sbjct  541  TATTAACTGATCGAAACTTTAACACATCATTTTTTGACCCTTCAGGAGGAGGAGATCCCA  600

Query  639  TTTTATATCAACATTTATTT  658
            ||||||||||||| ||||||
Sbjct  601  TTTTATATCAACACTTATTT  620
```

>Rhipicephalus sanguineus isolate Al-Hosema-A cytochrome oxidase subunit I (COI) gene, partial cds; mitochondrial
Sequence ID: KX757909.1 Length: 630
Range 1: 1 to 620

Score:979 bits(530), Expect:0.0,
Identities:590/620(95%), Gaps:0/620(0%), Strand: Plus/Plus

```
Query  39   TAGGACTTAGTATAAGAATATTAATTCGTATAGAATTAGGACAACCTGGAACTTTAATTG  98
            ||||||||||||||||||||||||||||||||||||||||||||||||||||||||||||
Sbjct  1    TAGGACTTAGTATAAGAATATTAATTCGTATAGAATTAGGACAACCTGGAACTTTAATTG  60

Query  99   GAAATGATCAGATTTATAATGTAATCGTAACAGCACATGCATTTATTATAAttttttttA  158
            ||||||||||||||||||||||||||||||||||||||||||||||||||||||| ||||
Sbjct  61   GAAATGATCAGATTTATAATGTAATCGTAACAGCACATGCATTTATTATAATTTTCTTTA  120

Query  159  TAGTTATACCAATCATAATCGGAGGATTTGGAAATTGATTAGTCCCTATCATATTGGGTG  218
            ||||||||||||| ||||||||||| || |||||||||||||| ||||| ||||| ||||
Sbjct  121  TAGTTATACCAATAATAATCGGAGGGTTCGGAAATTGATTAGTTCCTATTATATTAGGTG  180
```

```
Query  219  CCCCAGATATAGCATTTCCACGAATAAATAATATAAGATTTTGACTTTTACCTCCTTCAC  278
            |||||||||||||||||||||||||||||||||||||||||||||||||||| || |||
Sbjct  181  CCCCAGATATAGCATTTCCACGAATAAATAATATAAGATTTTGACTTTTACCCCCCTCAT  240

Query  279  TATTTTTATTAATTAACTCTTCATTAATTGAATCTGGAGCTGGTACAGGATGAACAGTTT  338
            |||||||||| |||||||||||||||||||||||||||||||| ||||||||||||||||
Sbjct  241  TATTTTTATTGATTAACTCTTCATTAATTGAATCTGGAGCTGGAACAGGATGAACAGTTT  300

Query  339  ACCCTCCTCTATCTTCAAATTTATCACATTATGGCCCTTCAGTAGATTTAGCTATTTTTT  398
            | || |||||||||||||||||||||||||||||||| ||||||||||||||||||||||
Sbjct  301  ATCCCCCTCTATCTTCAAATTTATCACATTATGGCCCATCAGTAGATTTAGCTATTTTTT  360

Query  399  CTCTTCATCTTGCTGGTGCTTCTTCAATTTTAGGTGCAATTAACTTTATTACAACTATTG  458
            ||||||| |||||||||||||||||||||||||||||||||||||| || ||||||||||
Sbjct  361  CTCTTCACCTTGCTGGTGCTTCTTCAATTTTAGGTGCAATTAACTTCATCACAACTATTG  420

Query  459  TAAACATACGATCAATTGGAATAACAATAGAACGTATACCATTATTTGTTTGATCTGTTT  518
            |||| ||||||||||||||||||||||||||||| |||||||||||||||||||||||||
Sbjct  421  TAAATATACGATCAATTGGAATAACAATAGAACGAATACCATTATTTGTTTGATCTGTTT  480

Query  519  TAATTACAGCTATTTTATTACTTTTATCTTTACCTGTATTAGCAGGTGCCATTACAATAT  578
            ||||||||||||||||| ||||| ||||||||||||||||||||||||||||||||||||
Sbjct  481  TAATTACAGCTATTTTACTACTTCTATCTTTACCTGTATTAGCAGGTGCCATTACAATAT  540

Query  579  TGTTAACTGATCGAAACTTTAATACATCATTTTTTGATCCTTCAGGGGGAGGAGATCCAA  638
            | || |||||||||||||||||| ||||||||||||| |||||||| ||||| ||||| |
Sbjct  541  TATTGACTGATCGAAACTTTAACACATCATTTTTTGACCCTTCAGGAGGAGGGGATCCCA  600

Query  639  TTTTATATCAACATTTATTT  658
            ||||||||||||| ||||||
Sbjct  601  TTTTATATCAACACTTATTT  620
```

>Rhipicephalus sanguineus isolate FT157 cytochrome c oxidase subunit I (COI) gene, partial cds; mitochondrial
Sequence ID: MN223690.1 Length: 535
Range 1: 1 to 524

Score:963 bits(521), Expect:0.0,
Identities:523/524(99%), Gaps:0/524(0%), Strand: Plus/Plus

```
Query  135  ATGCATTTATTATAAttttttttATAGTTATACCAATCATAATCGGAGGATTTGGAAATT  194
            ||||||||||||||||||||||||||||||||||||||||||||||||||||||||||||
Sbjct  1    ATGCATTTATTATAATTTTTTTTATAGTTATACCAATCATAATCGGAGGATTTGGAAATT  60

Query  195  GATTAGTCCCTATCATATTGGGTGCCCCAGATATAGCATTTCCACGAATAAATAATATAA  254
            ||||||||||||||||||||||||||||||||||||||||||||||||||||||||||||
Sbjct  61   GATTAGTCCCTATCATATTGGGTGCCCCAGATATAGCATTTCCACGAATAAATAATATAA  120

Query  255  GATTTTGACTTTTACCTCCTTCACTATTTTTATTAATTAACTCTTCATTAATTGAATCTG  314
            ||||||||||||||||||||||||||||||||||||||||||||||||||||||||||||
Sbjct  121  GATTTTGACTTTTACCTCCTTCACTATTTTTATTAATTAACTCTTCATTAATTGAATCTG  180
```

```
Query  315  GAGCTGGTACAGGATGAACAGTTTACCCTCCTCTATCTTCAAATTTATCACATTATGGCC  374
            |||||||||||||||||||||||||||||||| |||||||||||||||||||||||||||
Sbjct  181  GAGCTGGTACAGGATGAACAGTTTACCCTCCTTTATCTTCAAATTTATCACATTATGGCC  240

Query  375  CTTCAGTAGATTTAGCTATTTTTTCTCTTCATCTTGCTGGTGCTTCTTCAATTTTAGGTG  434
            ||||||||||||||||||||||||||||||||||||||||||||||||||||||||||||
Sbjct  241  CTTCAGTAGATTTAGCTATTTTTTCTCTTCATCTTGCTGGTGCTTCTTCAATTTTAGGTG  300

Query  435  CAATTAACTTTATTACAACTATTGTAAACATACGATCAATTGGAATAACAATAGAACGTA  494
            ||||||||||||||||||||||||||||||||||||||||||||||||||||||||||||
Sbjct  301  CAATTAACTTTATTACAACTATTGTAAACATACGATCAATTGGAATAACAATAGAACGTA  360

Query  495  TACCATTATTTGTTTGATCTGTTTTAATTACAGCTATTTTATTACTTTTATCTTTACCTG  554
            ||||||||||||||||||||||||||||||||||||||||||||||||||||||||||||
Sbjct  361  TACCATTATTTGTTTGATCTGTTTTAATTACAGCTATTTTATTACTTTTATCTTTACCTG  420

Query  555  TATTAGCAGGTGCCATTACAATATTGTTAACTGATCGAAACTTTAATACATCATTTTTTG  614
            ||||||||||||||||||||||||||||||||||||||||||||||||||||||||||||
Sbjct  421  TATTAGCAGGTGCCATTACAATATTGTTAACTGATCGAAACTTTAATACATCATTTTTTG  480

Query  615  ATCCTTCAGGGGGAGGAGATCCAATTTTATATCAACATTTATTT  658
            ||||||||||||||||||||||||||||||||||||||||||||
Sbjct  481  ATCCTTCAGGGGGAGGAGATCCAATTTTATATCAACATTTATTT  524
```

>Rhipicephalus sanguineus isolate FT154 cytochrome c oxidase subunit I (COI) gene, partial cds; mitochondrial
Sequence ID: MN223688.1 Length: 535
Range 1: 1 to 524

Score:957 bits(518), Expect:0.0,
Identities:522/524(99%), Gaps:0/524(0%), Strand: Plus/Plus

```
Query  135  ATGCATTTATTATAAtttttttATAGTTATACCAATCATAATCGGAGGATTTGGAAATT  194
            ||||||||||||||||||||||||||||||||||||||||||||||||||||||||||||
Sbjct  1    ATGCATTTATTATAATTTTTTTTATAGTTATACCAATCATAATCGGAGGATTTGGAAATT  60

Query  195  GATTAGTCCCTATCATATTGGGTGCCCCAGATATAGCATTTCCACGAATAAATAATATAA  254
            ||||||||||||||||||||||||||||||||||||||||||||||||||||||||||||
Sbjct  61   GATTAGTCCCTATCATATTGGGTGCCCCAGATATAGCATTTCCACGAATAAATAATATAA  120

Query  255  GATTTTGACTTTTACCTCCTTCACTATTTTTATTAATTAACTCTTCATTAATTGAATCTG  314
            ||||||||||||||||||||||||||||||||||||||||||||||| ||||||||||||
Sbjct  121  GATTTTGACTTTTACCTCCTTCACTATTTTTATTAATTAACTCTTCACTAATTGAATCTG  180

Query  315  GAGCTGGTACAGGATGAACAGTTTACCCTCCTCTATCTTCAAATTTATCACATTATGGCC  374
            ||||||||||||||||||||||||||||||||||||||||||| ||||||||||||||||
Sbjct  181  GAGCTGGTACAGGATGAACAGTTTACCCTCCTCTATCTTCAAACTTATCACATTATGGCC  240

Query  375  CTTCAGTAGATTTAGCTATTTTTTCTCTTCATCTTGCTGGTGCTTCTTCAATTTTAGGTG  434
            ||||||||||||||||||||||||||||||||||||||||||||||||||||||||||||
```

```
Sbjct  241   CTTCAGTAGATTTAGCTATTTTTTCTCTTCATCTTGCTGGTGCTTCTTCAATTTTAGGTG  300

Query  435   CAATTAACTTTATTACAACTATTGTAAACATACGATCAATTGGAATAACAATAGAACGTA  494
             ||||||||||||||||||||||||||||||||||||||||||||||||||||||||||||
Sbjct  301   CAATTAACTTTATTACAACTATTGTAAACATACGATCAATTGGAATAACAATAGAACGTA  360

Query  495   TACCATTATTTGTTTGATCTGTTTTAATTACAGCTATTTTATTACTTTTATCTTTACCTG  554
             ||||||||||||||||||||||||||||||||||||||||||||||||||||||||||||
Sbjct  361   TACCATTATTTGTTTGATCTGTTTTAATTACAGCTATTTTATTACTTTTATCTTTACCTG  420

Query  555   TATTAGCAGGTGCCATTACAATATTGTTAACTGATCGAAACTTTAATACATCATTTTTTG  614
             ||||||||||||||||||||||||||||||||||||||||||||||||||||||||||||
Sbjct  421   TATTAGCAGGTGCCATTACAATATTGTTAACTGATCGAAACTTTAATACATCATTTTTTG  480

Query  615   ATCCTTCAGGGGGAGGAGATCCAATTTTATATCAACATTTATTT  658
             ||||||||||||||||||||||||||||||||||||||||||||
Sbjct  481   ATCCTTCAGGGGGAGGAGATCCAATTTTATATCAACATTTATTT  524
```

>Rhipicephalus sanguineus isolate CE67 cytochrome c oxidase subunit I (COI) gene, partial cds; mitochondrial
Sequence ID: MN223686.1 Length: 535
Range 1: 1 to 524

Score:957 bits(518), Expect:0.0,
Identities:522/524(99%), Gaps:0/524(0%), Strand: Plus/Plus

```
Query  135   ATGCATTTATTATAAtttttttATAGTTATACCAATCATAATCGGAGGATTTGGAAATT  194
             ||||||||||||||||||||||||||||||||||||||||||||||||||||||||||||
Sbjct  1     ATGCATTTATTATAATTTTTTTATAGTTATACCAATCATAATCGGAGGATTTGGAAATT  60

Query  195   GATTAGTCCCTATCATATTGGGTGCCCCAGATATAGCATTTCCACGAATAAATAATATAA  254
             ||||||||||||||||||||||||||||||||||||||||||||||||||||||||||||
Sbjct  61    GATTAGTCCCTATCATATTGGGTGCCCCAGATATAGCATTTCCACGAATAAATAATATAA  120

Query  255   GATTTTGACTTTTACCTCCTTCACTATTTTTATTAATTAACTCTTCATTAATTGAATCTG  314
             ||||||||||||||||||||||||||||||||||||||||||||||||||||||||||||
Sbjct  121   GATTTTGACTTTTACCTCCTTCACTATTTTTATTAATTAACTCTTCATTAATTGAATCTG  180

Query  315   GAGCTGGTACAGGATGAACAGTTTACCCTCCTCTATCTTCAAATTTATCACATTATGGCC  374
             ||||||||||||||||||||||||||||||||||||||||||||||||||||||||||||
Sbjct  181   GAGCTGGTACAGGATGAACAGTTTACCCTCCTCTATCTTCAAATTTATCACATTATGGCC  240

Query  375   CTTCAGTAGATTTAGCTATTTTTTCTCTTCATCTTGCTGGTGCTTCTTCAATTTTAGGTG  434
             ||||||||||||||||||| ||||||||||||||||||||||||||||||||||||||||
Sbjct  241   CTTCAGTAGATTTAGCTATCTTTTCTCTTCATCTTGCTGGTGCTTCTTCAATTTTAGGTG  300

Query  435   CAATTAACTTTATTACAACTATTGTAAACATACGATCAATTGGAATAACAATAGAACGTA  494
             ||||||||||||||||||||||||||||||||||||||||||||||||||||||||||| 
Sbjct  301   CAATTAACTTTATTACAACTATTGTAAACATACGATCAATTGGAATAACAATAGAACGTG  360

Query  495   TACCATTATTTGTTTGATCTGTTTTAATTACAGCTATTTTATTACTTTTATCTTTACCTG  554
```

```
                ||||||||||||||||||||||||||||||||||||||||||||||||||||||||||||
Sbjct  361  TACCATTATTTGTTTGATCTGTTTTAATTACAGCTATTTTATTACTTTTATCTTTACCTG  420

Query  555  TATTAGCAGGTGCCATTACAATATTGTTAACTGATCGAAACTTTAATACATCATTTTTTG  614
            ||||||||||||||||||||||||||||||||||||||||||||||||||||||||||||
Sbjct  421  TATTAGCAGGTGCCATTACAATATTGTTAACTGATCGAAACTTTAATACATCATTTTTTG  480

Query  615  ATCCTTCAGGGGGAGGAGATCCAATTTTATATCAACATTTATTT  658
            ||||||||||||||||||||||||||||||||||||||||||||
Sbjct  481  ATCCTTCAGGGGGAGGAGATCCAATTTTATATCAACATTTATTT  524
```

```
>Rhipicephalus sanguineus isolate CE61 cytochrome c oxidase subunit I (COI)
gene, partial cds; mitochondrial
Sequence ID: MN223689.1 Length: 535
Range 1: 1 to 524

Score:952 bits(515), Expect:0.0,
Identities:521/524(99%),  Gaps:0/524(0%), Strand: Plus/Plus

Query  135  ATGCATTTATTATAAtttttttATAGTTATACCAATCATAATCGGAGGATTTGGAAATT  194
            ||||||||||||||||||||||||||||||||||||||||||||||||||||||||||||
Sbjct  1    ATGCATTTATTATAATTTTTTTTATAGTTATACCAATCATAATCGGAGGATTTGGAAATT  60

Query  195  GATTAGTCCCTATCATATTGGGTGCCCCAGATATAGCATTTCCACGAATAAATAATATAA  254
            ||||||||||||||||||||||||||||||||||||||||||||||||||||||||||||
Sbjct  61   GATTAGTCCCTATCATATTGGGTGCCCCAGATATAGCATTTCCACGAATAAATAATATAA  120

Query  255  GATTTTGACTTTTACCTCCTTCACTATTTTTATTAATTAACTCTTCATTAATTGAATCTG  314
            ||||||||||||||||||||||||||||||||||||||||||||||||||||||||||||
Sbjct  121  GATTTTGACTTTTACCTCCTTCACTATTTTTATTAATTAACTCTTCATTAATTGAATCTG  180

Query  315  GAGCTGGTACAGGATGAACAGTTTACCCTCCTCTATCTTCAAATTTATCACATTATGGCC  374
            |||||||||||||||||||||||||||||||||||||||||||||||||||||||||| |
Sbjct  181  GAGCTGGTACAGGATGAACAGTTTACCCTCCTCTATCTTCAAATTTATCACATTATGGTC  240

Query  375  CTTCAGTAGATTTAGCTATTTTTTCTCTTCATCTTGCTGGTGCTTCTTCAATTTTAGGTG  434
            |||||||||||||| |||||||||||||||||||||||||||||||||||||||||||||
Sbjct  241  CTTCAGTAGATTTAACTATTTTTTCTCTTCATCTTGCTGGTGCTTCTTCAATTTTAGGTG  300

Query  435  CAATTAACTTTATTACAACTATTGTAAACATACGATCAATTGGAATAACAATAGAACGTA  494
            ||||||||||||||||||||||||||||||||||||||||||||||||||||||||||||
Sbjct  301  CAATTAACTTTATTACAACTATTGTAAACATACGATCAATTGGAATAACAATAGAACGTA  360

Query  495  TACCATTATTTGTTTGATCTGTTTTAATTACAGCTATTTTATTACTTTTATCTTTACCTG  554
            ||||||||||||||||||||||||||||||||||||||||| ||||||||||||||||||
Sbjct  361  TACCATTATTTGTTTGATCTGTTTTAATTACAGCTATTTTACTACTTTTATCTTTACCTG  420

Query  555  TATTAGCAGGTGCCATTACAATATTGTTAACTGATCGAAACTTTAATACATCATTTTTTG  614
            ||||||||||||||||||||||||||||||||||||||||||||||||||||||||||||
Sbjct  421  TATTAGCAGGTGCCATTACAATATTGTTAACTGATCGAAACTTTAATACATCATTTTTTG  480
```

```
Query  615  ATCCTTCAGGGGGAGGAGATCCAATTTTATATCAACATTTATTT  658
            ||||||||||||||||||||||||||||||||||||||||||||
Sbjct  481  ATCCTTCAGGGGGAGGAGATCCAATTTTATATCAACATTTATTT  524
```

>Rhipicephalus sanguineus isolate RI46 cytochrome c oxidase subunit I (COI) gene, partial cds; mitochondrial
Sequence ID: MN223687.1 Length: 535
Range 1: 1 to 524

Score:952 bits(515), Expect:0.0,
Identities:521/524(99%), Gaps:0/524(0%), Strand: Plus/Plus

```
Query  135  ATGCATTTATTATAAtttttttATAGTTATACCAATCATAATCGGAGGATTTGGAAATT  194
            ||||||||||||||||||||||||||||||||||||||||||||||||||||||||||||
Sbjct  1    ATGCATTTATTATAATTTTTTTATAGTTATACCAATCATAATCGGAGGATTTGGAAATT  60

Query  195  GATTAGTCCCTATCATATTGGGTGCCCCAGATATAGCATTTCCACGAATAAATAATATAA  254
            ||||||||||||| ||||||||||||||||||||||||||||||||||||||||||||||
Sbjct  61   GATTAGTCCCTATTATATTGGGTGCCCCAGATATAGCATTTCCACGAATAAATAATATAA  120

Query  255  GATTTTGACTTTTACCTCCTTCACTATTTTTATTAATTAACTCTTCATTAATTGAATCTG  314
            ||||||||||||||||||||||||||||||||||||||||||||||||||||||||||||
Sbjct  121  GATTTTGACTTTTACCTCCTTCACTATTTTTATTAATTAACTCTTCATTAATTGAATCTG  180

Query  315  GAGCTGGTACAGGATGAACAGTTTACCCTCCTCTATCTTCAAATTTATCACATTATGGCC  374
            |||||||||||||||||||||||||||||||||||||||||||||||||||| |||||||
Sbjct  181  GAGCTGGTACAGGATGAACAGTTTACCCTCCTCTATCTTCAAATTTATCACACTATGGCC  240

Query  375  CTTCAGTAGATTTAGCTATTTTTTCTCTTCATCTTGCTGGTGCTTCTTCAATTTTAGGTG  434
            ||||||||||||||||||||||||||||||||||||||||||||||||||||||||||||
Sbjct  241  CTTCAGTAGATTTAGCTATTTTTTCTCTTCATCTTGCTGGTGCTTCTTCAATTTTAGGTG  300

Query  435  CAATTAACTTTATTACAACTATTGTAAACATACGATCAATTGGAATAACAATAGAACGTA  494
            ||||||||||||||||||||||||||||||||||||||||||||||||||||||||||||
Sbjct  301  CAATTAACTTTATTACAACTATTGTAAACATACGATCAATTGGAATAACAATAGAACGTA  360

Query  495  TACCATTATTTGTTTGATCTGTTTTAATTACAGCTATTTTATTACTTTTATCTTTACCTG  554
            ||||| ||||||||||||||||||||||||||||||||||||||||||||||||||||||
Sbjct  361  TACCACTATTTGTTTGATCTGTTTTAATTACAGCTATTTTATTACTTTTATCTTTACCTG  420

Query  555  TATTAGCAGGTGCCATTACAATATTGTTAACTGATCGAAACTTTAATACATCATTTTTTG  614
            ||||||||||||||||||||||||||||||||||||||||||||||||||||||||||||
Sbjct  421  TATTAGCAGGTGCCATTACAATATTGTTAACTGATCGAAACTTTAATACATCATTTTTTG  480

Query  615  ATCCTTCAGGGGGAGGAGATCCAATTTTATATCAACATTTATTT  658
            ||||||||||||||||||||||||||||||||||||||||||||
Sbjct  481  ATCCTTCAGGGGGAGGAGATCCAATTTTATATCAACATTTATTT  524
```

>Rhipicephalus sanguineus isolate RI08 cytochrome c oxidase subunit I (COI) gene, partial cds; mitochondrial
Sequence ID: MN223684.1 Length: 535
Range 1: 3 to 524

Score:948 bits(513), Expect:0.0,
Identities:519/522(99%), Gaps:0/522(0%), Strand: Plus/Plus

```
Query  137  GCATTTATTATAAttttttttATAGTTATACCAATCATAATCGGAGGATTTGGAAATTGA  196
            ||||||||||||||||||||||||||||||||||||||||||||||||||||||||||||
Sbjct  3    GCATTTATTATAATTTTTTTTATAGTTATACCAATCATAATCGGAGGATTTGGAAATTGA  62

Query  197  TTAGTCCCTATCATATTGGGTGCCCCAGATATAGCATTTCCACGAATAAATAATATAAGA  256
            ||||||||||| ||||| ||||||||||||||||||||||||||||||||||||||||||
Sbjct  63   TTAGTCCCTATTATATTAGGTGCCCCAGATATAGCATTTCCACGAATAAATAATATAAGA  122

Query  257  TTTTGACTTTTACCTCCTTCACTATTTTTATTAATTAACTCTTCATTAATTGAATCTGGA  316
            ||||||||||||||||||||||||||||||||||||||||||||||||||||||||||||
Sbjct  123  TTTTGACTTTTACCTCCTTCACTATTTTTATTAATTAACTCTTCATTAATTGAATCTGGA  182

Query  317  GCTGGTACAGGATGAACAGTTTACCCTCCTCTATCTTCAAATTTATCACATTATGGCCCT  376
            ||||||||||||||||||||||||||||||||||||||||||||||||||||||||||||
Sbjct  183  GCTGGTACAGGATGAACAGTTTACCCTCCTCTATCTTCAAATTTATCACATTATGGCCCT  242

Query  377  TCAGTAGATTTAGCTATTTTTTCTCTTCATCTTGCTGGTGCTTCTTCAATTTTAGGTGCA  436
            ||||||||||||||||| ||||||||||||||||||||||||||||||||||||||||||
Sbjct  243  TCAGTAGATTTAGCTATCTTTTCTCTTCATCTTGCTGGTGCTTCTTCAATTTTAGGTGCA  302

Query  437  ATTAACTTTATTACAACTATTGTAAACATACGATCAATTGGAATAACAATAGAACGTATA  496
            ||||||||||||||||||||||||||||||||||||||||||||||||||||||||||||
Sbjct  303  ATTAACTTTATTACAACTATTGTAAACATACGATCAATTGGAATAACAATAGAACGTATA  362

Query  497  CCATTATTTGTTTGATCTGTTTTAATTACAGCTATTTTATTACTTTTATCTTTACCTGTA  556
            ||||||||||||||||||||||||||||||||||||||||||||||||||||||||||||
Sbjct  363  CCATTATTTGTTTGATCTGTTTTAATTACAGCTATTTTATTACTTTTATCTTTACCTGTA  422

Query  557  TTAGCAGGTGCCATTACAATATTGTTAACTGATCGAAACTTTAATACATCATTTTTTGAT  616
            ||||||||||||||||||||||||||||||||||||||||||||||||||||||||||||
Sbjct  423  TTAGCAGGTGCCATTACAATATTGTTAACTGATCGAAACTTTAATACATCATTTTTTGAT  482

Query  617  CCTTCAGGGGGAGGAGATCCAATTTTATATCAACATTTATTT  658
            ||||||||||||||||||||||||||||||||||||||||||
Sbjct  483  CCTTCAGGGGGAGGAGATCCAATTTTATATCAACATTTATTT  524
```

>Rhipicephalus sanguineus isolate RI54 cytochrome c oxidase subunit I (COI) gene, partial cds; mitochondrial
Sequence ID: MN223685.1 Length: 535
Range 1: 1 to 524

Score:946 bits(512), Expect:0.0,
Identities:520/524(99%), Gaps:0/524(0%), Strand: Plus/Plus

```
Query  135  ATGCATTTATTATAAttttttttATAGTTATACCAATCATAATCGGAGGATTTGGAAATT  194
            |||||||||||||||||||||||||||||||||| |||||||||||||||||||||||||
Sbjct  1    ATGCATTTATTATAATTTTTTTTATAGTTATACCGATCATAATCGGAGGATTTGGAAATT  60

Query  195  GATTAGTCCCTATCATATTGGGTGCCCCAGATATAGCATTTCCACGAATAAATAATATAA  254
            ||||||||||||||||||| ||||||||||||||||||||||||||||||||||||||||
Sbjct  61   GATTAGTCCCTATCATATTAGGTGCCCCAGATATAGCATTTCCACGAATAAATAATATAA  120

Query  255  GATTTTGACTTTTACCTCCTTCACTATTTTTATTAATTAACTCTTCATTAATTGAATCTG  314
            ||||||||||||||||||||||||||||||||||||||||||||||||||||||||||||
Sbjct  121  GATTTTGACTTTTACCTCCTTCACTATTTTTATTAATTAACTCTTCATTAATTGAATCTG  180

Query  315  GAGCTGGTACAGGATGAACAGTTTACCCTCCTCTATCTTCAAATTTATCACATTATGGCC  374
            ||||||||||||||||||||||||||||||||||||||||||||||||||||||||||||
Sbjct  181  GAGCTGGTACAGGATGAACAGTTTACCCTCCTCTATCTTCAAATTTATCACATTATGGCC  240

Query  375  CTTCAGTAGATTTAGCTATTTTTTCTCTTCATCTTGCTGGTGCTTCTTCAATTTTAGGTG  434
            ||||||||||||||||||| ||||||||||||||||||||||||||||||||||||||||
Sbjct  241  CTTCAGTAGATTTAGCTATCTTTTCTCTTCATCTTGCTGGTGCTTCTTCAATTTTAGGTG  300

Query  435  CAATTAACTTTATTACAACTATTGTAAACATACGATCAATTGGAATAACAATAGAACGTA  494
            ||||||||||||||||||||||||||||||||||||||||||||||||||||||||||||
Sbjct  301  CAATTAACTTTATTACAACTATTGTAAACATACGATCAATTGGAATAACAATAGAACGTA  360

Query  495  TACCATTATTTGTTTGATCTGTTTTAATTACAGCTATTTTATTACTTTTATCTTTACCTG  554
            |||||||||||||||||||||||||||||||||||||||||||||| |||||||||||||
Sbjct  361  TACCATTATTTGTTTGATCTGTTTTAATTACAGCTATTTTATTACTCTTATCTTTACCTG  420

Query  555  TATTAGCAGGTGCCATTACAATATTGTTAACTGATCGAAACTTTAATACATCATTTTTTG  614
            ||||||||||||||||||||||||||||||||||||||||||||||||||||||||||||
Sbjct  421  TATTAGCAGGTGCCATTACAATATTGTTAACTGATCGAAACTTTAATACATCATTTTTTG  480

Query  615  ATCCTTCAGGGGGAGGAGATCCAATTTTATATCAACATTTATTT  658
            ||||||||||||||||||||||||||||||||||||||||||||
Sbjct  481  ATCCTTCAGGGGGAGGAGATCCAATTTTATATCAACATTTATTT  524
```

>Rhipicephalus sanguineus isolate FT044 cytochrome c oxidase subunit I (COI) gene, partial cds; mitochondrial
Sequence ID: MN223683.1 Length: 535
Range 1: 1 to 524

Score:946 bits(512), Expect:0.0,
Identities:520/524(99%), Gaps:0/524(0%), Strand: Plus/Plus

```
Query  135  ATGCATTTATTATAAttttttttATAGTTATACCAATCATAATCGGAGGATTTGGAAATT  194
            ||||||||||||||||||||||||||||||||||||||||||||||||||||||||||||
Sbjct  1    ATGCATTTATTATAATTTTTTTTATAGTTATACCAATCATAATCGGAGGATTTGGAAATT  60

Query  195  GATTAGTCCCTATCATATTGGGTGCCCCAGATATAGCATTTCCACGAATAAATAATATAA  254
            ||||||| ||||||||||| ||||||||||||||||||||||||||||||||||||||||
```

```
Sbjct  61   GATTAGTTCCTATCATATTAGGTGCCCCAGATATAGCATTTCCACGAATAAATAATATAA  120

Query  255  GATTTTGACTTTTACCTCCTTCACTATTTTTATTAATTAACTCTTCATTAATTGAATCTG  314
            ||||||||||||||||||||||||||||||||||||||||||||||||||||||||||||
Sbjct  121  GATTTTGACTTTTACCTCCTTCACTATTTTTATTAATTAACTCTTCATTAATTGAATCTG  180

Query  315  GAGCTGGTACAGGATGAACAGTTTACCCTCCTCTATCTTCAAATTTATCACATTATGGCC  374
            ||||||||||||||||||||||||||||||||||||||||||||||||||||||||||||
Sbjct  181  GAGCTGGTACAGGATGAACAGTTTACCCTCCTCTATCTTCAAATTTATCACATTATGGCC  240

Query  375  CTTCAGTAGATTTAGCTATTTTTTCTCTTCATCTTGCTGGTGCTTCTTCAATTTTAGGTG  434
            ||||||||||||||||||| ||||||||||||||||||||||||||||||||||||||||
Sbjct  241  CTTCAGTAGATTTAGCTATCTTTTCTCTTCATCTTGCTGGTGCTTCTTCAATTTTAGGTG  300

Query  435  CAATTAACTTTATTACAACTATTGTAAACATACGATCAATTGGAATAACAATAGAACGTA  494
            ||||||||||||||||||||||||||||||||||||||||||||||||||||||||||||
Sbjct  301  CAATTAACTTTATTACAACTATTGTAAACATACGATCAATTGGAATAACAATAGAACGTA  360

Query  495  TACCATTATTTGTTTGATCTGTTTTAATTACAGCTATTTTATTACTTTTATCTTTACCTG  554
            ||||||||||||||||||||||||||||||||||||||||||||| ||||||||||||||
Sbjct  361  TACCATTATTTGTTTGATCTGTTTTAATTACAGCTATTTTATTACTGTTATCTTTACCTG  420

Query  555  TATTAGCAGGTGCCATTACAATATTGTTAACTGATCGAAACTTTAATACATCATTTTTTG  614
            ||||||||||||||||||||||||||||||||||||||||||||||||||||||||||||
Sbjct  421  TATTAGCAGGTGCCATTACAATATTGTTAACTGATCGAAACTTTAATACATCATTTTTTG  480

Query  615  ATCCTTCAGGGGGAGGAGATCCAATTTTATATCAACATTTATTT  658
            ||||||||||||||||||||||||||||||||||||||||||||
Sbjct  481  ATCCTTCAGGGGGAGGAGATCCAATTTTATATCAACATTTATTT  524
```

>Rhipicephalus sanguineus isolate Rsang2 cytochrome oxidase subunit 1 gene, partial cds; mitochondrial
Sequence ID: KY678135.1 Length: 790
Range 1: 18 to 675

Score:900 bits(487), Expect:0.0,
Identities:601/658(91%), Gaps:0/658(0%), Strand: Plus/Plus

```
Query  1    AACAATATATTTAATTTTTGGAGCATGATCAGGTATATTAGGACTTAGTATAAGAATATT  60
            |||||||||||||||||||||||||||||| || ||||||||| | || ||||||||| |
Sbjct  18   AACAATATATTTAATTTTTGGAGCATGATCCGGAATATTAGGATTAAGAATAAGAATACT  77

Query  61   AATTCGTATAGAATTAGGACAACCTGGAACTTTAATTGGAAATGATCAGATTTATAATGT  120
            |||||||||||||||||| |||||||||||  ||||||| |||||||| |||||||||||
Sbjct  78   AATTCGTATAGAATTAGGCCAACCTGGAACCCTAATTGGTAATGATCAAATTTATAATGT  137

Query  121  AATCGTAACAGCACATGCATTTATTATAAtttttttATAGTTATACCAATCATAATCGG  180
            ||| || ||||| ||||||||||||||||||||||||||||| |||||||||||||| ||
Sbjct  138  AATTGTTACAGCTCATGCATTTATTATAATTTTTTTTATAGTAATACCAATCATAATTGG  197

Query  181  AGGATTTGGAAATTGATTAGTCCCTATCATATTGGGTGCCCCAGATATAGCATTTCCACG  240
```

```
               ||||||||||| |||||||| ||||| ||| | || || |||||||||||||| |||||
Sbjct  198    TGGATTTGGAAACTGATTAGTTCCTATTATACTAGGAGCTCCAGATATAGCATTCCCACG  257

Query  241    AATAAATAATATAAGATTTTGACTTTTACCTCCTTCACTATTTTTATTAATTAACTCTTC  300
              ||||||||||||||||||||||||| | ||||| ||| ||||| |||||||||| |||||
Sbjct  258    AATAAATAATATAAGATTTTGACTTCTTCCTCCCTCATTATTTATATTAATTAATTCTTC  317

Query  301    ATTAATTGAATCTGGAGCTGGTACAGGATGAACAGTTTACCCTCCTCTATCTTCAAATTT  360
              ||||||||| || ||||| |||||||||||||||||||| |||||  |||| ||||||||
Sbjct  318    ATTAATTGAGTCAGGAGCAGGTACAGGATGAACAGTTTATCCTCCCTTATCCTCAAATTT  377

Query  361    ATCACATTATGGCCCTTCAGTAGATTTAGCTATTTTTTCTCTTCATCTTGCTGGTGCTTC  420
              |||||||||||| || ||||||||||||||||||||||||||||||||||||||||||||
Sbjct  378    ATCACATTATGGGCCATCAGTAGATTTAGCTATTTTTTCTCTTCATCTTGCTGGTGCTTC  437

Query  421    TTCAATTTTAGGTGCAATTAACTTTATTACAACTATTGTAAACATACGATCAATTGGAAT  480
              ||||||||||||||||||||| |||||||||||||||||||| |||||||| ||||||||
Sbjct  438    TTCAATTTTAGGTGCAATTAATTTTATTACAACTATTGTAAATATACGATCTATTGGAAT  497

Query  481    AACAATAGAACGTATACCATTATTTGTTTGATCTGTTTTAATTACAGCTATTTTATTACT  540
              |||||||||||| |||||||||||||| ||||||||||||||||| || |||||||||||
Sbjct  498    AACAATAGAACGAATACCATTATTTGTATGATCTGTTTTAATTACTGCAATTTTATTACT  557

Query  541    TTTATCTTTACCTGTATTAGCAGGTGCCATTACAATATTGTTAACTGATCGAAACTTTAA  600
               |||||||||||||| ||||||||||||||||||||| | ||||| |||||||| |||||
Sbjct  558    ATTATCTTTACCTGTTTTAGCAGGTGCCATTACAATACTATTAACCGATCGAAATTTTAA  617

Query  601    TACATCATTTTTTGATCCTTCAGGGGGAGGAGATCCAATTTTATATCAACATTTATTT  658
               || ||||||||||| |||||||||||||| |||||||||||||||||||||||||||
Sbjct  618    CACTTCATTTTTTGACCCTTCAGGGGGAGGGGATCCAATTTTATATCAACATTTATTT  675
```

>Rhipicephalus sanguineus cytochrome oxidase subunit 1 (CO1) gene, partial cds; mitochondrial
Sequence ID: KU214592.1 Length: 829
Range 1: 1 to 648

Score:898 bits(486), Expect:0.0,
Identities:594/648(92%), Gaps:0/648(0%), Strand: Plus/Plus

```
Query  11     TTAATTTTTGGAGCATGATCAGGTATATTAGGACTTAGTATAAGAATATTAATTCGTATA  70
              |||||||||||||||||||| || ||||||||| | || ||||||||| |||||||||||
Sbjct  1      TTAATTTTTGGAGCATGATCCGGAATATTAGGATTAAGAATAAGAATACTAATTCGTATA  60

Query  71     GAATTAGGACAACCTGGAACTTTAATTGGAAATGATCAGATTTATAATGTAATCGTAACA  130
              |||||||| |||||||||||| ||||||| |||||||| |||||||||||||| || |||
Sbjct  61     GAATTAGGCCAACCTGGAACTCTAATTGGTAATGATCAAATTTATAATGTAATTGTTACA  120

Query  131    GCACATGCATTTATTATAAttttttttATAGTTATACCAATCATAATCGGAGGATTTGGA  190
              || ||||||||||||||||||||||||||||| |||||||||||||| || |||||||||
Sbjct  121    GCTCATGCATTTATTATAATTTTTTTTATAGTAATACCAATCATAATTGGTGGATTTGGA  180
```

```
Query  191  AATTGATTAGTCCCTATCATATTGGGTGCCCCAGATATAGCATTTCCACGAATAAATAAT  250
            || |||||||| ||||| ||| | || || ||||||||||||||||||||||||||||||
Sbjct  181  AACTGATTAGTTCCTATTATACTAGGAGCTCCAGATATAGCATTTCCACGAATAAATAAT  240

Query  251  ATAAGATTTTGACTTTTACCTCCTTCACTATTTTTATTAATTAACTCTTCATTAATTGAA  310
            ||||||||||||||| | ||||| ||| ||||| |||||||||| |||||||||||||| 
Sbjct  241  ATAAGATTTTGACTTCTTCCTCCCTCATTATTTATATTAATTAATTCTTCATTAATTGAG  300

Query  311  TCTGGAGCTGGTACAGGATGAACAGTTTACCCTCCTCTATCTTCAAATTTATCACATTAT  370
            || ||||| ||||||||||| |||||||| ||||| ||||| ||||||||||||||||||
Sbjct  301  TCAGGAGCAGGTACAGGATGGACAGTTTATCCTCCCCTATCCTCAAATTTATCACATTAT  360

Query  371  GGCCCTTCAGTAGATTTAGCTATTTTTTCTCTTCATCTTGCTGGTGCTTCTTCAATTTTA  430
            || || ||||||||||||||||||||||||||||||||||||||||||||||||||||||
Sbjct  361  GGGCCATCAGTAGATTTAGCTATTTTTTCTCTTCATCTTGCTGGTGCTTCTTCAATTTTA  420

Query  431  GGTGCAATTAACTTTATTACAACTATTGTAAACATACGATCAATTGGAATAACAATAGAA  490
            ||||||||||| |||||||||||||||||||| |||||||| ||||||||||||||||||
Sbjct  421  GGTGCAATTAATTTTATTACAACTATTGTAAATATACGATCTATTGGAATAACAATAGAA  480

Query  491  CGTATACCATTATTTGTTTGATCTGTTTTAATTACAGCTATTTTATTACTTTTATCTTTA  550
            || |||||||||||||| ||||||||||||||||| || ||||||||||| |||||||||
Sbjct  481  CGAATACCATTATTTGTATGATCTGTTTTAATTACTGCAATTTTATTACTATTATCTTTA  540

Query  551  CCTGTATTAGCAGGTGCCATTACAATATTGTTAACTGATCGAAACTTTAATACATCATTT  610
            ||||| ||||||||||||||||||||||| ||||| |||||||| ||||| || ||||||
Sbjct  541  CCTGTTTTAGCAGGTGCCATTACAATATTATTAACCGATCGAAATTTTAACACTTCATTT  600

Query  611  TTTGATCCTTCAGGGGGAGGAGATCCAATTTTATATCAACATTTATTT  658
            ||||| |||||||||||||| |||||||||||||||||||||||||||
Sbjct  601  TTTGACCCTTCAGGGGGAGGGGATCCAATTTTATATCAACATTTATTT  648
```

>Rhipicephalus sanguineus isolate Gilan-e Gharb cytochrome oxidase subunit 1 gene, complete cds; mitochondrial
Sequence ID: KM494916.1 Length: 1539
Range 1: 45 to 702

Score:889 bits(481), Expect:0.0,
Identities:599/658(91%), Gaps:0/658(0%), Strand: Plus/Plus

```
Query  1    AACAATATATTTAATTTTTGGAGCATGATCAGGTATATTAGGACTTAGTATAAGAATATT  60
            |||||||||||||||||||||||||||||| || ||||||||| | || ||||||||| |
Sbjct  45   AACAATATATTTAATTTTTGGAGCATGATCCGGAATATTAGGATTAAGAATAAGAATACT  104

Query  61   AATTCGTATAGAATTAGGACAACCTGGAACTTTAATTGGAAATGATCAGATTTATAATGT  120
            |||||||||||||||||| |||||||||||| ||||||| |||||||| |||||||||||
Sbjct  105  AATTCGTATAGAATTAGGTCAACCTGGAACTCTAATTGGTAATGATCAAATTTATAATGT  164

Query  121  AATCGTAACAGCACATGCATTTATTATAAttttttttATAGTTATACCAATCATAATCGG  180
            ||| || ||||| ||||||||||||||||||||||||||||| |||||||| ||||| ||
Sbjct  165  AATTGTTACAGCTCATGCATTTATTATAATTTTTTTTATAGTAATACCAATTATAATTGG  224
```

```
Query  181  AGGATTTGGAAATTGATTAGTCCCTATCATATTGGGTGCCCCAGATATAGCATTTCCACG  240
             ||||||||||| |||||||| ||||| ||| | || || |||||||||||||| |||||
Sbjct  225  TGGATTTGGAAACTGATTAGTACCTATTATACTAGGAGCTCCAGATATAGCATTCCCACG  284

Query  241  AATAAATAATATAAGATTTTGACTTTTACCTCCTTCACTATTTTTATTAATTAACTCTTC  300
            ||||||||||||||||||||||||| | ||||| ||| ||||| |||||||||| |||||
Sbjct  285  AATAAATAATATAAGATTTTGACTTCTTCCTCCCTCATTATTTATATTAATTAATTCTTC  344

Query  301  ATTAATTGAATCTGGAGCTGGTACAGGATGAACAGTTTACCCTCCTCTATCTTCAAATTT  360
            ||||||||| || ||||| |||||||||||||||||||| ||||| ||||| ||||||||
Sbjct  345  ATTAATTGAGTCAGGAGCAGGTACAGGATGAACAGTTTATCCTCCCCTATCCTCAAATTT  404

Query  361  ATCACATTATGGCCCTTCAGTAGATTTAGCTATTTTTTCTCTTCATCTTGCTGGTGCTTC  420
            |||||||||||| || ||||||||||||||||||||||||||||||||||||||||||||
Sbjct  405  ATCACATTATGGGCCATCAGTAGATTTAGCTATTTTTTCTCTTCATCTTGCTGGTGCTTC  464

Query  421  TTCAATTTTAGGTGCAATTAACTTTATTACAACTATTGTAAACATACGATCAATTGGAAT  480
            ||||||||||||||||||||| ||||||||||||||||| || |||||||| ||||||||
Sbjct  465  TTCAATTTTAGGTGCAATTAATTTTATTACAACTATTGTGAATATACGATCTATTGGAAT  524

Query  481  AACAATAGAACGTATACCATTATTTGTTTGATCTGTTTTAATTACAGCTATTTTATTACT  540
            |||||||||||| |||||||||||||| ||||||||||||||||| || |||||||||||
Sbjct  525  AACAATAGAACGAATACCATTATTTGTATGATCTGTTTTAATTACTGCAATTTTATTACT  584

Query  541  TTTATCTTTACCTGTATTAGCAGGTGCCATTACAATATTGTTAACTGATCGAAACTTTAA  600
             |||||||||||||| ||||||||||| ||||||||| | ||||| |||||||| |||||
Sbjct  585  ATTATCTTTACCTGTTTTAGCAGGTGCTATTACAATACTATTAACCGATCGAAATTTTAA  644

Query  601  TACATCATTTTTTGATCCTTCAGGGGGAGGAGATCCAATTTATATCAACATTTATTT  658
             || ||||||||||| |||||||| ||||| |||||||||||||||||||||||||||
Sbjct  645  CACTTCATTTTTTGACCCTTCAGGAGGAGGGGATCCAATTTATATCAACATTTATTT  702
```

>Rhipicephalus sanguineus isolate Tehran cytochrome oxidase subunit 1 gene, complete cds; mitochondrial
Sequence ID: KM494915.1 Length: 1539
Range 1: 45 to 702

Score:889 bits(481), Expect:0.0,
Identities:599/658(91%), Gaps:0/658(0%), Strand: Plus/Plus

```
Query  1    AACAATATATTTAATTTTTGGAGCATGATCAGGTATATTAGGACTTAGTATAAGAATATT  60
            |||||||||||||||||||||||||||||| || ||||||||| | || ||||||||| |
Sbjct  45   AACAATATATTTAATTTTTGGAGCATGATCCGGAATATTAGGATTAAGAATAAGAATACT  104

Query  61   AATTCGTATAGAATTAGGACAACCTGGAACTTTAATTGGAAATGATCAGATTTATAATGT  120
            |||||||||||||||||| |||||||||||| ||||||| |||||||| |||||||||||
Sbjct  105  AATTCGTATAGAATTAGGTCAACCTGGAACTCTAATTGGTAATGATCAAATTTATAATGT  164

Query  121  AATCGTAACAGCACATGCATTTATTATAAttttttttATAGTTATACCAATCATAATCGG  180
            ||| || ||||| ||||||||||||||||||||||||||||| |||||||| ||||| ||
```

```
Sbjct  165  AATTGTTACAGCTCATGCATTTATTATAATTTTTTTATAGTAATACCAATTATAATTGG  224

Query  181  AGGATTTGGAAATTGATTAGTCCCTATCATATTGGGTGCCCCAGATATAGCATTTCCACG  240
             ||||||||||| |||||||| ||||| ||| | || || |||||||||||||| |||||
Sbjct  225  TGGATTTGGAAACTGATTAGTACCTATTATACTAGGAGCTCCAGATATAGCATTCCCACG  284

Query  241  AATAAATAATATAAGATTTTGACTTTTACCTCCTTCACTATTTTTATTAATTAACTCTTC  300
            ||||||||||||||||||||||||| | ||||| ||| ||||| |||||||||| |||||
Sbjct  285  AATAAATAATATAAGATTTTGACTTCTTCCTCCCTCATTATTTATATTAATTAATTCTTC  344

Query  301  ATTAATTGAATCTGGAGCTGGTACAGGATGAACAGTTTACCCTCCTCTATCTTCAAATTT  360
            ||||||||| || ||||| |||||||||||||||||||| ||||| ||||| ||||||||
Sbjct  345  ATTAATTGAGTCAGGAGCAGGTACAGGATGAACAGTTTATCCTCCCCTATCCTCAAATTT  404

Query  361  ATCACATTATGGCCCTTCAGTAGATTTAGCTATTTTTCTCTTCATCTTGCTGGTGCTTC  420
            |||||||||||| || ||||||||||||||||||||||||||||||||||||||||||||
Sbjct  405  ATCACATTATGGGCCATCAGTAGATTTAGCTATTTTTCTCTTCATCTTGCTGGTGCTTC  464

Query  421  TTCAATTTTAGGTGCAATTAACTTTATTACAACTATTGTAAACATACGATCAATTGGAAT  480
            ||||||||||||||||||||| ||||||||||||||||| || |||||||| ||||||||
Sbjct  465  TTCAATTTTAGGTGCAATTAATTTTATTACAACTATTGTGAATATACGATCTATTGGAAT  524

Query  481  AACAATAGAACGTATACCATTATTTGTTTGATCTGTTTTAATTACAGCTATTTTATTACT  540
            |||||||||||| |||||||||||||| ||||||||||||||||| || |||||||||||
Sbjct  525  AACAATAGAACGAATACCATTATTTGTATGATCTGTTTTAATTACTGCAATTTTATTACT  584

Query  541  TTTATCTTTACCTGTATTAGCAGGTGCCATTACAATATTGTTAACTGATCGAAACTTTAA  600
             |||||||||||||| ||||||||||| ||||||||| | ||||| |||||||| |||||
Sbjct  585  ATTATCTTTACCTGTTTTAGCAGGTGCTATTACAATACTATTAACCGATCGAAATTTTAA  644

Query  601  TACATCATTTTTTGATCCTTCAGGGGGAGGAGATCCAATTTTATATCAACATTTATTT  658
             || ||||||||||| |||||||| ||||| |||||||||||||||||||||||||||
Sbjct  645  CACTTCATTTTTTGACCCTTCAGGAGGAGGGGATCCAATTTTATATCAACATTTATTT  702
```

>Rhipicephalus camicasi isolate SC0126 mitochondrion, complete genome
Sequence ID: NC_061616.1 Length: 14725
>Rhipicephalus camicasi isolate SC0126 mitochondrion, complete genome
Sequence ID: MZ323229.1 Length: 14725
Range 1: 45 to 702

Score:889 bits(481), Expect:0.0,
Identities:599/658(91%), Gaps:0/658(0%), Strand: Plus/Plus

```
Query  1    AACAATATATTTAATTTTTGGAGCATGATCAGGTATATTAGGACTTAGTATAAGAATATT  60
            ||||||||| |||||||||||||||||||| || ||||||||| | || ||||||||| |
Sbjct  45   AACAATATACTTAATTTTTGGAGCATGATCTGGAATATTAGGATTAAGAATAAGAATACT  104

Query  61   AATTCGTATAGAATTAGGACAACCTGGAACTTTAATTGGAAATGATCAGATTTATAATGT  120
            |||||||||||||||||| |||||||||||||||||||||||||| || |||||||||||
Sbjct  105  AATTCGTATAGAATTAGGCCAACCTGGAACTTTAATTGGAAATGACCAAATTTATAATGT  164
```

```
Query  121  AATCGTAACAGCACATGCATTTATTATAAttttttttATAGTTATACCAATCATAATCGG  180
            ||| |||||||||||||||||||||||||||||||||||||| |||||||| || || ||
Sbjct  165  AATTGTAACAGCACATGCATTTATTATAATTTTTTTTATAGTAATACCAATTATGATTGG  224

Query  181  AGGATTTGGAAATTGATTAGTCCCTATCATATTGGGTGCCCCAGATATAGCATTTCCACG  240
            |||||| |||||||||||||||||||| ||||| || || ||||||||||||||||||||
Sbjct  225  AGGATTCGGAAATTGATTAGTCCCTATTATATTAGGGGCTCCAGATATAGCATTTCCACG  284

Query  241  AATAAATAATATAAGATTTTGACTTTTACCTCCTTCACTATTTTTATTAATTAACTCTTC  300
            |||||||||||||||||||||| |  | ||||||||| |||||||||||||||| |||||
Sbjct  285  AATAAATAATATAAGATTTTGATTACTTCCTCCTTCATTATTTTTATTAATTAATTCTTC  344

Query  301  ATTAATTGAATCTGGAGCTGGTACAGGATGAACAGTTTACCCTCCTCTATCTTCAAATTT  360
            |||||||||||| ||||| || |||||||||||||||||||||||  |||| ||||||||
Sbjct  345  ATTAATTGAATCAGGAGCAGGGACAGGATGAACAGTTTACCCTCCCTTATCATCAAATTT  404

Query  361  ATCACATTATGGCCCTTCAGTAGATTTAGCTATTTTTTCTCTTCATCTTGCTGGTGCTTC  420
            |||||| ||||| || ||||||||| ||||||||||||||||||||||||||||||||||
Sbjct  405  ATCACACTATGGTCCATCAGTAGATCTAGCTATTTTTTCTCTTCATCTTGCTGGTGCTTC  464

Query  421  TTCAATTTTAGGTGCAATTAACTTTATTACAACTATTGTAAACATACGATCAATTGGAAT  480
            |||||||||||| |||||||| || |||||||| ||||||||||||||||| ||||||||
Sbjct  465  TTCAATTTTAGGCGCAATTAATTTCATTACAACCATTGTAAACATACGATCTATTGGAAT  524

Query  481  AACAATAGAACGTATACCATTATTTGTTTGATCTGTTTTAATTACAGCTATTTTATTACT  540
            |||||||||||| ||||| |||||||| ||||||||||||||||| || ||||||||| |
Sbjct  525  AACAATAGAACGAATACCTTTATTTGTATGATCTGTTTTAATTACTGCAATTTTATTATT  584

Query  541  TTTATCTTTACCTGTATTAGCAGGTGCCATTACAATATTGTTAACTGATCGAAACTTTAA  600
             ||||||||||| || ||||| ||||| ||||||||||| |||||||||||||| |||||
Sbjct  585  ATTATCTTTACCAGTTTTAGCTGGTGCAATTACAATATTATTAACTGATCGAAATTTTAA  644

Query  601  TACATCATTTTTTGATCCTTCAGGGGGAGGAGATCCAATTTTATATCAACATTTATTT  658
            ||| || || |||||||||||||| ||||| |||||||||||||||||||||||||||
Sbjct  645  TACCTCGTTCTTTGATCCTTCAGGAGGAGGGGATCCAATTTTATATCAACATTTATTT  702
```

>Rhipicephalus turanicus isolate COX1-225-Goat cytochrome c oxidase subunit I (COX1) gene, partial cds; mitochondrial
Sequence ID: MT800313.1 Length: 673
Range 1: 8 to 665

Score:883 bits(478), Expect:0.0,
Identities:598/658(91%), Gaps:0/658(0%), Strand: Plus/Plus

```
Query  1    AACAATATATTTAATTTTTGGAGCATGATCAGGTATATTAGGACTTAGTATAAGAATATT  60
            ||||||||||||||||||||| ||||||||||| ||||||||||| || ||||||||| |
Sbjct  8    AACAATATATTTAATTTTTGGGGCATGATCAGGAATATTAGGACTGAGAATAAGAATACT  67

Query  61   AATTCGTATAGAATTAGGACAACCTGGAACTTTAATTGGAAATGATCAGATTTATAATGT  120
            |||||| ||||||||||||||||||||||||||||||||||||||||| |||||||| ||
Sbjct  68   AATTCGCATAGAATTAGGACAACCTGGAACTTTAATTGGAAATGATCAAATTTATAACGT  127
```

```
Query  121  AATCGTAACAGCACATGCATTTATTATAAttttttttATAGTTATACCAATCATAATCGG  180
            ||| |||||||| |||||||||||||||||||||||||| || ||||| || ||||| ||
Sbjct  128  AATTGTAACAGCTCATGCATTTATTATAATTTTTTTTATGGTAATACCTATTATAATTGG  187

Query  181  AGGATTTGGAAATTGATTAGTCCCTATCATATTGGGTGCCCCAGATATAGCATTTCCACG  240
             |||||||||||||||||||| ||||| ||||| || || |||||||||||||| |||||
Sbjct  188  TGGATTTGGAAATTGATTAGTTCCTATTATATTAGGGGCTCCAGATATAGCATTCCCACG  247

Query  241  AATAAATAATATAAGATTTTGACTTTTACCTCCTTCACTATTTTTATTAATTAACTCTTC  300
             |||||||| |||||||||||| |  | ||||||||| ||||| ||||||||||||||||
Sbjct  248  TATAAATAACATAAGATTTTGATTACTTCCTCCTTCATTATTTCTATTAATTAACTCTTC  307

Query  301  ATTAATTGAATCTGGAGCTGGTACAGGATGAACAGTTTACCCTCCTCTATCTTCAAATTT  360
            |||||||||||| ||||| || ||||||||||| ||||| || ||| |||| ||||||||
Sbjct  308  ATTAATTGAATCAGGAGCAGGAACAGGATGAACTGTTTATCCCCCTTTATCCTCAAATTT  367

Query  361  ATCACATTATGGCCCTTCAGTAGATTTAGCTATTTTTCTCTTCATCTTGCTGGTGCTTC  420
            |||||||||||| || ||||||||||||||||||||||||||||||||||||||||||||
Sbjct  368  ATCACATTATGGACCATCAGTAGATTTAGCTATTTTTCTCTTCATCTTGCTGGTGCTTC  427

Query  421  TTCAATTTTAGGTGCAATTAACTTTATTACAACTATTGTAAACATACGATCAATTGGAAT  480
            ||||||| ||||||||||||| |||||||||||||||||||| |||||||| ||||||||
Sbjct  428  TTCAATTCTAGGTGCAATTAATTTTATTACAACTATTGTAAATATACGATCTATTGGAAT  487

Query  481  AACAATAGAACGTATACCATTATTTGTTTGATCTGTTTTAATTACAGCTATTTTATTACT  540
            |||||||||||| |||||||||||||||||||||||||||||||| || || ||| | ||
Sbjct  488  AACAATAGAACGAATACCATTATTTGTTTGATCTGTTTTAATTACCGCCATCTTACTTCT  547

Query  541  TTTATCTTTACCTGTATTAGCAGGTGCCATTACAATATTGTTAACTGATCGAAACTTTAA  600
             |||||||||||||| ||||||||||| ||||||||||| |||||||||||||| || ||
Sbjct  548  CTTATCTTTACCTGTTTTAGCAGGTGCTATTACAATATTATTAACTGATCGAAATTTCAA  607

Query  601  TACATCATTTTTTGATCCTTCAGGGGAGGAGATCCAATTTTATATCAACATTTATT  658
            ||| ||||||||||||||||||||||||| |||||||||||||| ||||| |||||||
Sbjct  608  TACTTCATTTTTTGATCCTTCAGGGGAGGTGATCCAATTTTATACCAACACTTATT  665
```

>Rhipicephalus turanicus isolate COX1-18-Sheep cytochrome c oxidase subunit I (COX1) gene, partial cds; mitochondrial
Sequence ID: MT800314.1 Length: 673
Range 1: 8 to 664

Score:881 bits(477), Expect:0.0,
Identities:597/657(91%), Gaps:0/657(0%), Strand: Plus/Plus

```
Query  1    AACAATATATTTAATTTTTGGAGCATGATCAGGTATATTAGGACTTAGTATAAGAATATT  60
            ||||||||||||||||||||| ||||||||||| ||||||||||| || ||||||||| |
Sbjct  8    AACAATATATTTAATTTTTGGGGCATGATCAGGAATATTAGGACTGAGAATAAGAATACT  67

Query  61   AATTCGTATAGAATTAGGACAACCTGGAACTTTAATTGGAAATGATCAGATTTATAATGT  120
            |||||| ||||||||||||||||||||||||||||||||||||||||| |||||||| ||
```

```
Sbjct  68   AATTCGCATAGAATTAGGACAACCTGGAACTTTAATTGGAAATGATCAAATTTATAACGT  127

Query  121  AATCGTAACAGCACATGCATTTATTATAAtttttttATAGTTATACCAATCATAATCGG  180
            ||| |||||||| ||||||||||||||||||||||||| || ||||| || ||||| ||
Sbjct  128  AATTGTAACAGCTCATGCATTTATTATAATTTTTTTATGGTAATACCCATTATAATTGG  187

Query  181  AGGATTTGGAAATTGATTAGTCCCTATCATATTGGGTGCCCCAGATATAGCATTTCCACG  240
             |||||||||||||||||||| ||||| ||||| || || |||||||||||||| |||||
Sbjct  188  TGGATTTGGAAATTGATTAGTTCCTATTATATTAGGGGCTCCAGATATAGCATTCCCACG  247

Query  241  AATAAATAATATAAGATTTTGACTTTTACCTCCTTCACTATTTTTATTAATTAACTCTTC  300
             |||||||| |||||||||||| |  | ||||||||| ||||| ||||||||||||| ||
Sbjct  248  TATAAATAACATAAGATTTTGATTACTTCCTCCTTCATTATTTCTATTAATTAACTCCTC  307

Query  301  ATTAATTGAATCTGGAGCTGGTACAGGATGAACAGTTTACCCTCCTCTATCTTCAAATTT  360
            |||||||||||| ||||| || ||||||||||| ||||| || ||| |||| ||||||||
Sbjct  308  ATTAATTGAATCGGGAGCAGGAACAGGATGAACTGTTTATCCCCCTTTATCCTCAAATTT  367

Query  361  ATCACATTATGGCCCTTCAGTAGATTTAGCTATTTTTCTCTTCATCTTGCTGGTGCTTC  420
            |||||||||||| || ||||||||||||||||||||||||||||||||||||||||||||
Sbjct  368  ATCACATTATGGACCATCAGTAGATTTAGCTATTTTTCTCTTCATCTTGCTGGTGCTTC  427

Query  421  TTCAATTTTAGGTGCAATTAACTTTATTACAACTATTGTAAACATACGATCAATTGGAAT  480
            ||||||| ||||||||||||| ||||||||||||||||||||||||||||| ||||||||
Sbjct  428  TTCAATTCTAGGTGCAATTAATTTTATTACAACTATTGTAAACATACGATCTATTGGAAT  487

Query  481  AACAATAGAACGTATACCATTATTTGTTTGATCTGTTTTAATTACAGCTATTTTATTACT  540
            |||||||||||| |||||||||||||||||||||||||||||||| || || ||| | ||
Sbjct  488  AACAATAGAACGAATACCATTATTTGTTTGATCTGTTTTAATTACCGCCATCTTACTTCT  547

Query  541  TTTATCTTTACCTGTATTAGCAGGTGCCATTACAATATTGTTAACTGATCGAAACTTTAA  600
             |||||||||||||| ||||||||||| ||||||||||| |||||||||||||| || ||
Sbjct  548  CTTATCTTTACCTGTTTTAGCAGGTGCTATTACAATATTATTAACTGATCGAAATTTCAA  607

Query  601  TACATCATTTTTTGATCCTTCAGGGGGAGGAGATCCAATTTTATATCAACATTTATT  657
            ||| |||||||||||||||||||||||||| |||||||||||||| ||||| |||||
Sbjct  608  TACTTCATTTTTTGATCCTTCAGGGGGAGGTGATCCAATTTTATACCAACACTTATT  664
```

>Rhipicephalus turanicus isolate 31X cytochrome oxidase subunit 1 (COI) gene, partial cds; mitochondrial
Sequence ID: KY606303.1 Length: 873
Range 1: 66 to 723

Score:872 bits(472), Expect:0.0,
Identities:596/658(91%), Gaps:0/658(0%), Strand: Plus/Plus

```
Query  1    AACAATATATTTAATTTTTGGAGCATGATCAGGTATATTAGGACTTAGTATAAGAATATT  60
            |||||||||||||||||||||||||||||| || ||||||||| | || |||||||||||
Sbjct  66   AACAATATATTTAATTTTTGGAGCATGATCCGGGATATTAGGATTAAGAATAAGAATATT  125

Query  61   AATTCGTATAGAATTAGGACAACCTGGAACTTTAATTGGAAATGATCAGATTTATAATGT  120
```

```
               |||||| ||||| |||||||||||||||||||||||||||||||||||| |||||||||||
Sbjct  126   AATTCGAATAGAGTTAGGACAACCTGGAACTTTAATTGGAAATGATCAAATTTATAATGT  185

Query  121   AATCGTAACAGCACATGCATTTATTATAAttttttttATAGTTATACCAATCATAATCGG  180
             ||| |||||||| ||||||||||||||||||||||||||||| |||||||||||||| ||
Sbjct  186   AATTGTAACAGCTCATGCATTTATTATAATTTTTTTTATAGTAATACCAATCATAATTGG  245

Query  181   AGGATTTGGAAATTGATTAGTCCCTATCATATTGGGTGCCCCAGATATAGCATTTCCACG  240
             |||||||||||| |||||||| || || || || || || ||||| ||||||||||||||
Sbjct  246   AGGATTTGGAAACTGATTAGTGCCCATTATGTTAGGAGCTCCAGACATAGCATTTCCACG  305

Query  241   AATAAATAATATAAGATTTTGACTTTTACCTCCTTCACTATTTTTATTAATTAACTCTTC  300
             |||||||||||||||||||||| |  | ||||| ||| |||| ||||||||||| |||||
Sbjct  306   AATAAATAATATAAGATTTTGATTACTTCCTCCCTCATTATTCTTATTAATTAATTCTTC  365

Query  301   ATTAATTGAATCTGGAGCTGGTACAGGATGAACAGTTTACCCTCCTCTATCTTCAAATTT  360
             | | |||||||||||||| || ||||||||||| || ||||||||| |||||||||||||
Sbjct  366   ACTGATTGAATCTGGAGCAGGGACAGGATGAACTGTCTACCCTCCTTTATCTTCAAATTT  425

Query  361   ATCACATTATGGCCCTTCAGTAGATTTAGCTATTTTTTCTCTTCATCTTGCTGGTGCTTC  420
             ||| |||||||| || ||||||||||||||||| || ||||||||||| |||||||||||
Sbjct  426   ATCGCATTATGGACCATCAGTAGATTTAGCTATCTTCTCTCTTCATCTCGCTGGTGCTTC  485

Query  421   TTCAATTTTAGGTGCAATTAACTTTATTACAACTATTGTAAACATACGATCAATTGGAAT  480
             ||||||||||||||||||||| || |||||||||||||||||||||||||| ||||||||
Sbjct  486   TTCAATTTTAGGTGCAATTAATTTCATTACAACTATTGTAAACATACGATCTATTGGAAT  545

Query  481   AACAATAGAACGTATACCATTATTTGTTTGATCTGTTTTAATTACAGCTATTTTATTACT  540
             |||||||||||| ||||||||||| ||||||||||||||||| || ||||||||||| ||
Sbjct  546   AACAATAGAACGAATACCATTATTCGTTTGATCTGTTTTAATCACCGCTATTTTATTGCT  605

Query  541   TTTATCTTTACCTGTATTAGCAGGTGCCATTACAATATTGTTAACTGATCGAAACTTTAA  600
             | ||||||||||||| ||||||||||||||||||||||| |||||||||||||| || ||
Sbjct  606   TCTATCTTTACCTGTTTTAGCAGGTGCCATTACAATATTATTAACTGATCGAAATTTCAA  665

Query  601   TACATCATTTTTGATCCTTCAGGGGGAGGAGATCCAATTTTATATCAACATTTATTT  658
              || |||||||||| |||||||| ||||| || || ||||||||||||||||||||||
Sbjct  666   CACTTCATTTTTGACCCTTCAGGAGGAGGTGACCCTATTTTATATCAACATTTATTT  723
```

>Rhipicephalus turanicus isolate 19X cytochrome oxidase subunit 1 (COI) gene, partial cds; mitochondrial
Sequence ID: KY606302.1 Length: 873
Range 1: 66 to 723

Score:872 bits(472), Expect:0.0,
Identities:596/658(91%), Gaps:0/658(0%), Strand: Plus/Plus

```
Query  1     AACAATATATTTAATTTTTGGAGCATGATCAGGTATATTAGGACTTAGTATAAGAATATT  60
             |||||||||||||||||||||||||||||| || ||||||||| | || |||||||||||
Sbjct  66    AACAATATATTTAATTTTTGGAGCATGATCCGGGATATTAGGATTAAGAATAAGAATATT  125
```

```
Query  61    AATTCGTATAGAATTAGGACAACCTGGAACTTTAATTGGAAATGATCAGATTTATAATGT  120
             |||||| ||||| ||||||||||||||||||||||||||||||||||| |||||||||||
Sbjct  126   AATTCGAATAGAGTTAGGACAACCTGGAACTTTAATTGGAAATGATCAAATTTATAATGT  185

Query  121   AATCGTAACAGCACATGCATTTATTATAAttttttttATAGTTATACCAATCATAATCGG  180
             ||| |||||||| ||||||||||||||||||||||||||||| |||||||||||||| ||
Sbjct  186   AATTGTAACAGCTCATGCATTTATTATAATTTTTTTTATAGTAATACCAATCATAATTGG  245

Query  181   AGGATTTGGAAATTGATTAGTCCCTATCATATTGGGTGCCCCAGATATAGCATTTCCACG  240
             |||||||||||| |||||||| || || || || || || ||||| ||||||||||||||
Sbjct  246   AGGATTTGGAAACTGATTAGTGCCCATTATGTTAGGAGCTCCAGACATAGCATTTCCACG  305

Query  241   AATAAATAATATAAGATTTTGACTTTTACCTCCTTCACTATTTTTATTAATTAACTCTTC  300
             |||||||||||||||||||||| |  | ||||| ||| |||| ||||||||||| |||||
Sbjct  306   AATAAATAATATAAGATTTTGATTACTTCCTCCCTCATTATTCTTATTAATTAATTCTTC  365

Query  301   ATTAATTGAATCTGGAGCTGGTACAGGATGAACAGTTTACCCTCCTCTATCTTCAAATTT  360
             | | |||||||||||||| || ||||||||||| || ||||||||| |||||||||||||
Sbjct  366   ACTGATTGAATCTGGAGCAGGGACAGGATGAACTGTCTACCCTCCTTTATCTTCAAATTT  425

Query  361   ATCACATTATGGCCCTTCAGTAGATTTAGCTATTTTTTCTCTTCATCTTGCTGGTGCTTC  420
             ||| |||||||| || ||||||||||||||||| || ||||||||||| |||||||||||
Sbjct  426   ATCGCATTATGGACCATCAGTAGATTTAGCTATCTTCTCTCTTCATCTCGCTGGTGCTTC  485

Query  421   TTCAATTTTAGGTGCAATTAACTTTATTACAACTATTGTAAACATACGATCAATTGGAAT  480
             ||||||||||||||||||||| || |||||||||||||||||||||||||| ||||||||
Sbjct  486   TTCAATTTTAGGTGCAATTAATTTCATTACAACTATTGTAAACATACGATCTATTGGAAT  545

Query  481   AACAATAGAACGTATACCATTATTTGTTTGATCTGTTTTAATTACAGCTATTTTATTACT  540
             |||||||||||| ||||||||||| ||||||||||||||||| || ||||||||||| ||
Sbjct  546   AACAATAGAACGAATACCATTATTCGTTTGATCTGTTTTAATCACCGCTATTTTATTGCT  605

Query  541   TTTATCTTTACCTGTATTAGCAGGTGCCATTACAATATTGTTAACTGATCGAAACTTTAA  600
             | ||||||||||||| ||||||||||||||||||||||| |||||||||||||| || ||
Sbjct  606   TCTATCTTTACCTGTTTTAGCAGGTGCCATTACAATATTATTAACTGATCGAAATTTCAA  665

Query  601   TACATCATTTTTTGATCCTTCAGGGGGAGGAGATCCAATTTTATATCAACATTTATTT  658
              || ||||||||||| |||||||| ||||| || || |||||||||||||||||||||
Sbjct  666   CACTTCATTTTTTGACCCTTCAGGAGGAGGTGACCCTATTTTATATCAACATTTATTT  723
```

>Rhipicephalus turanicus isolate 19C cytochrome oxidase subunit 1 (COI) gene, partial cds; mitochondrial
Sequence ID: KY606301.1 Length: 873
Range 1: 66 to 723

Score:872 bits(472), Expect:0.0,
Identities:596/658(91%), Gaps:0/658(0%), Strand: Plus/Plus

```
Query  1     AACAATATATTTAATTTTTGGAGCATGATCAGGTATATTAGGACTTAGTATAAGAATATT  60
             |||||||||||||||||||||||||||||| || ||||||||| | || |||||||||||
Sbjct  66    AACAATATATTTAATTTTTGGAGCATGATCCGGGATATTAGGATTAAGAATAAGAATATT  125
```

```
Query  61   AATTCGTATAGAATTAGGACAACCTGGAACTTTAATTGGAAATGATCAGATTTATAATGT  120
            |||||| ||||| ||||||||||||||||||||||||||||||||||| |||||||||||
Sbjct  126  AATTCGAATAGAGTTAGGACAACCTGGAACTTTAATTGGAAATGATCAAATTTATAATGT  185

Query  121  AATCGTAACAGCACATGCATTTATTATAAttttttttATAGTTATACCAATCATAATCGG  180
            ||| |||||||| ||||||||||||||||||||||||||||| |||||||||||||| ||
Sbjct  186  AATTGTAACAGCTCATGCATTTATTATAATTTTTTTTATAGTAATACCAATCATAATTGG  245

Query  181  AGGATTTGGAAATTGATTAGTCCCTATCATATTGGGTGCCCCAGATATAGCATTTCCACG  240
            |||||||||||| |||||||| || || || || || || ||||| ||||||||||||||
Sbjct  246  AGGATTTGGAAACTGATTAGTGCCCATTATGTTAGGAGCTCCAGACATAGCATTTCCACG  305

Query  241  AATAAATAATATAAGATTTTGACTTTTACCTCCTTCACTATTTTTATTAATTAACTCTTC  300
            |||||||||||||||||||||| |  | ||||| ||| |||| ||||||||||| |||||
Sbjct  306  AATAAATAATATAAGATTTTGATTACTTCCTCCCTCATTATTCTTATTAATTAATTCTTC  365

Query  301  ATTAATTGAATCTGGAGCTGGTACAGGATGAACAGTTTACCCTCCTCTATCTTCAAATTT  360
            | | |||||||||||||| || ||||||||||| || ||||||||| |||||||||||||
Sbjct  366  ACTGATTGAATCTGGAGCAGGGACAGGATGAACTGTCTACCCTCCTTTATCTTCAAATTT  425

Query  361  ATCACATTATGGCCCTTCAGTAGATTTAGCTATTTTTTCTCTTCATCTTGCTGGTGCTTC  420
            ||| |||||||| || ||||||||||||||||| || ||||||||||| |||||||||||
Sbjct  426  ATCGCATTATGGACCATCAGTAGATTTAGCTATCTTCTCTCTTCATCTCGCTGGTGCTTC  485

Query  421  TTCAATTTTAGGTGCAATTAACTTTATTACAACTATTGTAAACATACGATCAATTGGAAT  480
            ||||||||||||||||||||| || |||||||||||||||||||||||||| ||||||||
Sbjct  486  TTCAATTTTAGGTGCAATTAATTTCATTACAACTATTGTAAACATACGATCTATTGGAAT  545

Query  481  AACAATAGAACGTATACCATTATTTGTTTGATCTGTTTTAATTACAGCTATTTTATTACT  540
            |||||||||||| ||||||||||| ||||||||||||||||| || ||||||||||| ||
Sbjct  546  AACAATAGAACGAATACCATTATTCGTTTGATCTGTTTTAATCACCGCTATTTTATTGCT  605

Query  541  TTTATCTTTACCTGTATTAGCAGGTGCCATTACAATATTGTTAACTGATCGAAACTTTAA  600
            | ||||||||||||| ||||||||||||||||||||||| |||||||||||||| || ||
Sbjct  606  TCTATCTTTACCTGTTTTAGCAGGTGCCATTACAATATTATTAACTGATCGAAATTTCAA  665

Query  601  TACATCATTTTTTGATCCTTCAGGGGGAGGAGATCCAATTTTATATCAACATTTATTT  658
             || ||||||||||| |||||||| ||||| || || |||||||||||||||||||||
Sbjct  666  CACTTCATTTTTTGACCCTTCAGGAGGAGGTGACCCTATTTTATATCAACATTTATTT  723
```

>Rhipicephalus turanicus isolate 12X cytochrome oxidase subunit 1 (COI) gene, partial cds; mitochondrial
Sequence ID: KY606300.1 Length: 873
Range 1: 66 to 723

Score:872 bits(472), Expect:0.0,
Identities:596/658(91%), Gaps:0/658(0%), Strand: Plus/Plus

```
Query  1    AACAATATATTTAATTTTTGGAGCATGATCAGGTATATTAGGACTTAGTATAAGAATATT  60
            |||||||||||||||||||||||||||||| || ||||||||| | || |||||||||||
```

```
Sbjct  66   AACAATATATTTAATTTTTGGAGCATGATCCGGGATATTAGGATTAAGAATAAGAATATT  125

Query  61   AATTCGTATAGAATTAGGACAACCTGGAACTTTAATTGGAAATGATCAGATTTATAATGT  120
            |||||| ||||| ||||||||||||||||||||||||||||||||||| |||||||||||
Sbjct  126  AATTCGAATAGAGTTAGGACAACCTGGAACTTTAATTGGAAATGATCAAATTTATAATGT  185

Query  121  AATCGTAACAGCACATGCATTTATTATAAttttttttATAGTTATACCAATCATAATCGG  180
            ||| |||||||| ||||||||||||||||||||||||||||| |||||||||||||| ||
Sbjct  186  AATTGTAACAGCTCATGCATTTATTATAATTTTTTTTATAGTAATACCAATCATAATTGG  245

Query  181  AGGATTTGGAAATTGATTAGTCCCTATCATATTGGGTGCCCCAGATATAGCATTTCCACG  240
            |||||||||||| |||||||| || || || || || || ||||| ||||||||||||||
Sbjct  246  AGGATTTGGAAACTGATTAGTGCCCATTATGTTAGGAGCTCCAGACATAGCATTTCCACG  305

Query  241  AATAAATAATATAAGATTTTGACTTTTACCTCCTTCACTATTTTTATTAATTAACTCTTC  300
            |||||||||||||||||||||| |  | ||||| ||| |||| ||||||||||| |||||
Sbjct  306  AATAAATAATATAAGATTTTGATTACTTCCTCCCTCATTATTCTTATTAATTAATTCTTC  365

Query  301  ATTAATTGAATCTGGAGCTGGTACAGGATGAACAGTTTACCCTCCTCTATCTTCAAATTT  360
            | | |||||||||||||| || ||||||||||| || ||||||||| |||||||||||||
Sbjct  366  ACTGATTGAATCTGGAGCAGGGACAGGATGAACTGTCTACCCTCCTTTATCTTCAAATTT  425

Query  361  ATCACATTATGGCCCTTCAGTAGATTTAGCTATTTTTTCTCTTCATCTTGCTGGTGCTTC  420
            ||| |||||||| || ||||||||||||||||| || |||||||||| ||||||||||||
Sbjct  426  ATCGCATTATGGACCATCAGTAGATTTAGCTATCTTCTCTCTTCATCTCGCTGGTGCTTC  485

Query  421  TTCAATTTTAGGTGCAATTAACTTTATTACAACTATTGTAAACATACGATCAATTGGAAT  480
            ||||||||||||||||||||| || |||||||||||||||||||||||||| ||||||||
Sbjct  486  TTCAATTTTAGGTGCAATTAATTTCATTACAACTATTGTAAACATACGATCTATTGGAAT  545

Query  481  AACAATAGAACGTATACCATTATTTGTTTGATCTGTTTTAATTACAGCTATTTTATTACT  540
            |||||||||||| ||||||||||| ||||||||||||||||| || ||||||||||| ||
Sbjct  546  AACAATAGAACGAATACCATTATTCGTTTGATCTGTTTTAATCACCGCTATTTTATTGCT  605

Query  541  TTTATCTTTACCTGTATTAGCAGGTGCCATTACAATATTGTTAACTGATCGAAACTTTAA  600
            | ||||||||||||| ||||||||||||||||||||||| |||||||||||||| || ||
Sbjct  606  TCTATCTTTACCTGTTTTAGCAGGTGCCATTACAATATTATTAACTGATCGAAATTTCAA  665

Query  601  TACATCATTTTTTGATCCTTCAGGGGGAGGAGATCCAATTTTATATCAACATTTATATTT  658
             || ||||||||||| |||||||| ||||| || || |||||||||||||||||||||
Sbjct  666  CACTTCATTTTTTGACCCTTCAGGAGGAGGTGACCCTATTTTATATCAACATTTATATTT  723
```

>Rhipicephalus turanicus isolate 12C cytochrome oxidase subunit 1 (COI) gene, partial cds; mitochondrial
Sequence ID: KY606299.1 Length: 873
Range 1: 66 to 723

Score:872 bits(472), Expect:0.0,
Identities:596/658(91%), Gaps:0/658(0%), Strand: Plus/Plus

```
Query  1    AACAATATATTTAATTTTTGGAGCATGATCAGGTATATTAGGACTTAGTATAAGAATATT  60
```

```
             ||||||||||||||||||||||||||||||| || ||||||||| | || |||||||||||
Sbjct  66    AACAATATATTTAATTTTTGGAGCATGATCCGGGATATTAGGATTAAGAATAAGAATATT  125

Query  61    AATTCGTATAGAATTAGGACAACCTGGAACTTTAATTGGAAATGATCAGATTTATAATGT  120
             |||||| ||||| ||||||||||||||||||||||||||||||||||| |||||||||||
Sbjct  126   AATTCGAATAGAGTTAGGACAACCTGGAACTTTAATTGGAAATGATCAAATTTATAATGT  185

Query  121   AATCGTAACAGCACATGCATTTATTATAAttttttttATAGTTATACCAATCATAATCGG  180
             ||| |||||||| ||||||||||||||||||||||||||||| |||||||||||||| ||
Sbjct  186   AATTGTAACAGCTCATGCATTTATTATAATTTTTTTTATAGTAATACCAATCATAATTGG  245

Query  181   AGGATTTGGAAATTGATTAGTCCCTATCATATTGGGTGCCCCAGATATAGCATTTCCACG  240
             |||||||||||| |||||||| || || || || || || ||||| ||||||||||||||
Sbjct  246   AGGATTTGGAAACTGATTAGTGCCCATTATGTTAGGAGCTCCAGACATAGCATTTCCACG  305

Query  241   AATAAATAATATAAGATTTTGACTTTTACCTCCTTCACTATTTTTATTAATTAACTCTTC  300
             |||||||||||||||||||||| |  | ||||| ||| |||| ||||||||||| |||||
Sbjct  306   AATAAATAATATAAGATTTTGATTACTTCCTCCCTCATTATTCTTATTAATTAATTCTTC  365

Query  301   ATTAATTGAATCTGGAGCTGGTACAGGATGAACAGTTTACCCTCCTCTATCTTCAAATTT  360
             | | |||||||||||||| || ||||||||||| || ||||||||| |||||||||||||
Sbjct  366   ACTGATTGAATCTGGAGCAGGGACAGGATGAACTGTCTACCCTCCTTTATCTTCAAATTT  425

Query  361   ATCACATTATGGCCCTTCAGTAGATTTAGCTATTTTTTCTCTTCATCTTGCTGGTGCTTC  420
             ||| |||||||| || ||||||||||||||||| || ||||||||||| |||||||||||
Sbjct  426   ATCGCATTATGGACCATCAGTAGATTTAGCTATCTTCTCTCTTCATCTCGCTGGTGCTTC  485

Query  421   TTCAATTTTAGGTGCAATTAACTTTATTACAACTATTGTAAACATACGATCAATTGGAAT  480
             ||||||||||||||||||||| || |||||||||||||||||||||||||| ||||||||
Sbjct  486   TTCAATTTTAGGTGCAATTAATTTCATTACAACTATTGTAAACATACGATCTATTGGAAT  545

Query  481   AACAATAGAACGTATACCATTATTTGTTTGATCTGTTTTAATTACAGCTATTTTATTACT  540
             |||||||||||| ||||||||||| ||||||||||||||||| || ||||||||||| ||
Sbjct  546   AACAATAGAACGAATACCATTATTCGTTTGATCTGTTTTAATCACCGCTATTTTATTGCT  605

Query  541   TTTATCTTTACCTGTATTAGCAGGTGCCATTACAATATTGTTAACTGATCGAAACTTTAA  600
             | ||||||||||||| ||||||||||||||||||||||| |||||||||||||| || ||
Sbjct  606   TCTATCTTTACCTGTTTTAGCAGGTGCCATTACAATATTATTAACTGATCGAAATTTCAA  665

Query  601   TACATCATTTTTTGATCCTTCAGGGGGAGGAGATCCAATTTTATATCAACATTTATTT  658
              || ||||||||||| |||||||| ||||| || || |||||||||||||||||||||
Sbjct  666   CACTTCATTTTTTGACCCTTCAGGAGGAGGTGACCCTATTTTATATCAACATTTATTT  723
```

>Rhipicephalus turanicus isolate COX1-7-Sheep cytochrome c oxidase subunit I (COX1) gene, partial cds; mitochondrial
Sequence ID: MT800312.1 Length: 673
Range 1: 8 to 664

Score:865 bits(468), Expect:0.0,
Identities:594/657(90%), Gaps:0/657(0%), Strand: Plus/Plus

```
Query  1    AACAATATATTTAATTTTTGGAGCATGATCAGGTATATTAGGACTTAGTATAAGAATATT  60
            ||||||||||||||||||||| ||||||||||| ||||||||||| || ||||||||| |
Sbjct  8    AACAATATATTTAATTTTTGGGGCATGATCAGGAATATTAGGACTGAGAATAAGAATACT  67

Query  61   AATTCGTATAGAATTAGGACAACCTGGAACTTTAATTGGAAATGATCAGATTTATAATGT  120
            |||||| |||||||||||||||||||||||||||||||||||||| || |||||||| ||
Sbjct  68   AATTCGCATAGAATTAGGACAACCTGGAACTTTAATTGGAAATGACCAAATTTATAACGT  127

Query  121  AATCGTAACAGCACATGCATTTATTATAAtttttttATAGTTATACCAATCATAATCGG  180
            ||| |||||||| ||||||||||||||||||||||||| || ||||| || ||||| ||
Sbjct  128  AATTGTAACAGCTCATGCATTTATTATAATTTTTTTATGGTAATACCTATTATAATTGG  187

Query  181  AGGATTTGGAAATTGATTAGTCCCTATCATATTGGGTGCCCCAGATATAGCATTTCCACG  240
             |||||||||||||||||||| ||||||||||| || || ||||||||||||||||||||
Sbjct  188  TGGATTTGGAAATTGATTAGTTCCTATCATATTAGGGGCTCCAGATATAGCATTTCCACG  247

Query  241  AATAAATAATATAAGATTTTGACTTTTACCTCCTTCACTATTTTTATTAATTAACTCTTC  300
             |||||||| |||||||||||| |  | ||||||||| ||||| ||||||||||||| ||
Sbjct  248  TATAAATAACATAAGATTTTGATTACTTCCTCCTTCATTATTTCTATTAATTAACTCCTC  307

Query  301  ATTAATTGAATCTGGAGCTGGTACAGGATGAACAGTTTACCCTCCTCTATCTTCAAATTT  360
            |||||||||||| ||||| || || |||||||| ||||| || ||| |||| ||||||||
Sbjct  308  ATTAATTGAATCGGGAGCAGGAACGGGATGAACTGTTTATCCCCCTTTATCCTCAAATTT  367

Query  361  ATCACATTATGGCCCTTCAGTAGATTTAGCTATTTTTCTCTTCATCTTGCTGGTGCTTC  420
            |||||||||||| || ||||||||||||||||||||||||||||||||||||||||||||
Sbjct  368  ATCACATTATGGACCATCAGTAGATTTAGCTATTTTTCTCTTCATCTTGCTGGTGCTTC  427

Query  421  TTCAATTTTAGGTGCAATTAACTTTATTACAACTATTGTAAACATACGATCAATTGGAAT  480
            ||||||| ||||||||||||| ||||||||||||||| ||||||||||||| ||||||||
Sbjct  428  TTCAATTCTAGGTGCAATTAATTTTATTACAACTATTCTAAACATACGATCTATTGGAAT  487

Query  481  AACAATAGAACGTATACCATTATTTGTTTGATCTGTTTTAATTACAGCTATTTTATTACT  540
            |||||||||||| |||||||||||||| |||||||||||||||||  | || ||| | ||
Sbjct  488  AACAATAGAACGAATACCATTATTTGTCTGATCTGTTTTAATTACCACCATCTTACTTCT  547

Query  541  TTTATCTTTACCTGTATTAGCAGGTGCCATTACAATATTGTTAACTGATCGAAACTTTAA  600
             |||||||||||||| ||||||||||| ||||||||||| |||||||||||||| || ||
Sbjct  548  CTTATCTTTACCTGTTTTAGCAGGTGCTATTACAATATTATTAACTGATCGAAATTTCAA  607

Query  601  TACATCATTTTTTGATCCTTCAGGGGGAGGAGATCCAATTTTATATCAACATTTATT  657
            ||| |||||||||||||||||||||||||| |||||||||||||| ||||| |||||
Sbjct  608  TACCTCATTTTTTGATCCTTCAGGGGGAGGTGATCCAATTTTATACCAACACTTATT  664
```

>Rhipicephalus turanicus isolate GY34-1 cytochrome c oxidase subunit I (COX1) gene, partial cds; mitochondrial
Sequence ID: MT079206.1 Length: 678
Range 1: 1 to 652

Score:861 bits(466), Expect:0.0,
Identities:590/652(90%), Gaps:0/652(0%), Strand: Plus/Plus

```
Query  7    ATATTTAATTTTTGGAGCATGATCAGGTATATTAGGACTTAGTATAAGAATATTAATTCG  66
            ||||||||||||||||||||||||||||||||||||| | || |||||||||||||||||
Sbjct  1    ATATTTAATTTTTGGAGCATGATCAGGTATATTAGGATTAAGAATAAGAATATTAATTCG  60

Query  67   TATAGAATTAGGACAACCTGGAACTTTAATTGGAAATGATCAGATTTATAATGTAATCGT  126
             ||||||||||||||||||||||||||||||||||||||||| |||||||||||||| ||
Sbjct  61   AATAGAATTAGGACAACCTGGAACTTTAATTGGAAATGATCAAATTTATAATGTAATTGT  120

Query  127  AACAGCACATGCATTTATTATAAttttttttATAGTTATACCAATCATAATCGGAGGATT  186
            |||||| ||||||||||||||||||||||||||||| |||||||||||||| ||||||||
Sbjct  121  AACAGCTCATGCATTTATTATAATTTTTTTTATAGTAATACCAATCATAATTGGAGGATT  180

Query  187  TGGAAATTGATTAGTCCCTATCATATTGGGTGCCCCAGATATAGCATTTCCACGAATAAA  246
            |||||| || ||||| || || ||||| || || ||||| ||||||||||||||||||||
Sbjct  181  TGGAAACTGGTTAGTTCCAATTATATTAGGAGCTCCAGACATAGCATTTCCACGAATAAA  240

Query  247  TAATATAAGATTTTGACTTTTACCTCCTTCACTATTTTTATTAATTAACTCTTCATTAAT  306
            |||||||||||||||| |  | || || ||| |||| ||||||||||| |||||| | ||
Sbjct  241  TAATATAAGATTTTGATTACTTCCCCCCTCATTATTCTTATTAATTAATTCTTCACTGAT  300

Query  307  TGAATCTGGAGCTGGTACAGGATGAACAGTTTACCCTCCTCTATCTTCAAATTTATCACA  366
            ||||||||| || || ||||| ||||| ||||| |||||| |||| ||||||||||| ||
Sbjct  301  TGAATCTGGGGCAGGGACAGGGTGAACTGTTTATCCTCCTTTATCCTCAAATTTATCCCA  360

Query  367  TTATGGCCCTTCAGTAGATTTAGCTATTTTTTCTCTTCATCTTGCTGGTGCTTCTTCAAT  426
            |||||| || ||||||||||||||||| || |||||||||||||||||||||||||||||
Sbjct  361  TTATGGACCATCAGTAGATTTAGCTATCTTCTCTCTTCATCTTGCTGGTGCTTCTTCAAT  420

Query  427  TTTAGGTGCAATTAACTTTATTACAACTATTGTAAACATACGATCAATTGGAATAACAAT  486
            |||||| |||||||| || |||||||||||||||||||||||||| ||||||||||||||
Sbjct  421  TTTAGGCGCAATTAATTTCATTACAACTATTGTAAACATACGATCTATTGGAATAACAAT  480

Query  487  AGAACGTATACCATTATTTGTTTGATCTGTTTTAATTACAGCTATTTTATTACTTTTATC  546
            |||||| ||||||||||| ||||||||||||||||| || ||||||||||| ||| ||||
Sbjct  481  AGAACGAATACCATTATTCGTTTGATCTGTTTTAATCACCGCTATTTTATTGCTTCTATC  540

Query  547  TTTACCTGTATTAGCAGGTGCCATTACAATATTGTTAACTGATCGAAACTTTAATACATC  606
            ||||||||| ||||||||||||||||||||| | |||||||||||||| ||||| || ||
Sbjct  541  TTTACCTGTTTTAGCAGGTGCCATTACAATACTATTAACTGATCGAAATTTTAACACTTC  600

Query  607  ATTTTTTGATCCTTCAGGGGGAGGAGATCCAATTTTATATCAACATTTATTT  658
            ||||||||| |||||||| ||||| || ||||||||||||||||||||||||
Sbjct  601  ATTTTTTGACCCTTCAGGAGGAGGTGACCCAATTTTATATCAACATTTATTT  652
```

>Rhipicephalus turanicus voucher CROBB808 cytochrome oxidase subunit 1 (COI) gene, partial cds; mitochondrial

Sequence ID: MZ305547.1 Length: 658

Range 1: 1 to 658

Score:861 bits(466), Expect:0.0,

```
Identities:594/658(90%),  Gaps:0/658(0%), Strand: Plus/Plus

Query  1    AACAATATATTTAATTTTTGGAGCATGATCAGGTATATTAGGACTTAGTATAAGAATATT  60
            |||||||||||||||||||||||||||||| || ||||||||| | || |||||||||||
Sbjct  1    AACAATATATTTAATTTTTGGAGCATGATCCGGGATATTAGGATTAAGAATAAGAATATT  60

Query  61   AATTCGTATAGAATTAGGACAACCTGGAACTTTAATTGGAAATGATCAGATTTATAATGT  120
            |||||| ||||| |||||||||||||| |||||||||||||||||||| |||||||||||
Sbjct  61   AATTCGAATAGAGTTAGGACAACCTGGGACTTTAATTGGAAATGATCAAATTTATAATGT  120

Query  121  AATCGTAACAGCACATGCATTTATTATAAttttttttATAGTTATACCAATCATAATCGG  180
            ||| |||||||| ||||||||||||||||||||||||||||| |||||||||||||| ||
Sbjct  121  AATTGTAACAGCTCATGCATTTATTATAATTTTTTTTATAGTAATACCAATCATAATTGG  180

Query  181  AGGATTTGGAAATTGATTAGTCCCTATCATATTGGGTGCCCCAGATATAGCATTTCCACG  240
            |||||||||||| |||||||| || || || || || || ||||| ||||||||||||||
Sbjct  181  AGGATTTGGAAACTGATTAGTGCCCATTATGTTAGGAGCTCCAGACATAGCATTTCCACG  240

Query  241  AATAAATAATATAAGATTTTGACTTTTACCTCCTTCACTATTTTTATTAATTAACTCTTC  300
            |||||||||||||||||||||| |  | ||||| ||| |||| ||||||||||| |||||
Sbjct  241  AATAAATAATATAAGATTTTGATTACTTCCTCCCTCATTATTCTTATTAATTAATTCTTC  300

Query  301  ATTAATTGAATCTGGAGCTGGTACAGGATGAACAGTTTACCCTCCTCTATCTTCAAATTT  360
            | | |||||||||||||| || ||||||||||| || ||||||||| |||||||||||||
Sbjct  301  ACTGATTGAATCTGGAGCAGGGACAGGATGAACTGTCTACCCTCCTTTATCTTCAAATTT  360

Query  361  ATCACATTATGGCCCTTCAGTAGATTTAGCTATTTTTTCTCTTCATCTTGCTGGTGCTTC  420
            ||| |||||||| || ||||||||||||||||| || ||||||||||| |||||||||||
Sbjct  361  ATCGCATTATGGACCATCAGTAGATTTAGCTATCTTCTCTCTTCATCTCGCTGGTGCTTC  420

Query  421  TTCAATTTTAGGTGCAATTAACTTTATTACAACTATTGTAAACATACGATCAATTGGAAT  480
            ||||||||||||||||||||| || ||||||||||||||||| |||||||| ||||||||
Sbjct  421  TTCAATTTTAGGTGCAATTAATTTCATTACAACTATTGTAAATATACGATCTATTGGAAT  480

Query  481  AACAATAGAACGTATACCATTATTTGTTTGATCTGTTTTAATTACAGCTATTTTATTACT  540
            |||||||||||| ||||||||||| ||||||||||||||||| || ||||||||||| ||
Sbjct  481  AACAATAGAACGAATACCATTATTCGTTTGATCTGTTTTAATCACCGCTATTTTATTGCT  540

Query  541  TTTATCTTTACCTGTATTAGCAGGTGCCATTACAATATTGTTAACTGATCGAAACTTTAA  600
            | ||||||||||||| ||||||||||||||||||||||| |||||||||||||| || ||
Sbjct  541  TCTATCTTTACCTGTTTTAGCAGGTGCCATTACAATATTATTAACTGATCGAAATTTCAA  600

Query  601  TACATCATTTTTTGATCCTTCAGGGGGAGGAGATCCAATTTTATATCAACATTTATTT  658
             || ||||||||||| |||||||| ||||| || || |||||||||||||||||||||
Sbjct  601  CACTTCATTTTTTGACCCTTCAGGAGGAGGTGACCCTATTTTATATCAACATTTATTT  658
```

Query #3: TKCJJ092-19|TKCJ012018H08|Rhipicephalus sanguineus|COI-5P Query ID: lcl|Query_26765 Length: 658

Sequences producing significant alignments:

Description	Scientific Name	Common Name	Taxid	Max Score	Total Score	Query cover	E Value	Per. Ident	Acc. Len	Accession
Rhipicephalus sanguineus mitochondrial DNA, complete genome	Rhipicephalu...	brown dog tick	34632	1216	1216	100%	0.0	100.00	14710	AF081829.1
Rhipicephalus sanguineus isolate Zagreb-B cytochrome oxidase...	Rhipicephalu...	brown dog tick	34632	1140	1140	94%	0.0	99.84	629	KX757893.1
Rhipicephalus sanguineus isolate Italy-A cytochrome oxidase...	Rhipicephalu...	brown dog tick	34632	1129	1129	94%	0.0	99.52	630	KX757903.1
Rhipicephalus sanguineus isolate Al-Hosema-A cytochrome oxidas...	Rhipicephalu...	brown dog tick	34632	1112	1112	94%	0.0	99.03	630	KX757909.1
Rhipicephalus sanguineus sensu lato 'temperate lineage' isolat...	Rhipicephalu...	brown dog tick	34632	1072	1072	100%	0.0	96.05	692	MF426015.1
Rhipicephalus sanguineus sensu lato 'temperate lineage' isolat...	Rhipicephalu...	brown dog tick	34632	1066	1066	100%	0.0	95.90	675	MF426018.1
Rhipicephalus sanguineus sensu lato 'temperate lineage' isolat...	Rhipicephalu...	brown dog tick	34632	1066	1066	100%	0.0	95.90	680	MF426016.1
Rhipicephalus sanguineus sensu lato 'temperate lineage' isolat...	Rhipicephalu...	brown dog tick	34632	1066	1066	100%	0.0	95.90	693	MF426013.1
Rhipicephalus sanguineus sensu lato 'temperate lineage' isolat...	Rhipicephalu...	brown dog tick	34632	1066	1066	100%	0.0	95.90	679	MF426009.1
Rhipicephalus sanguineus sensu lato 'temperate lineage' isolat...	Rhipicephalu...	brown dog tick	34632	1066	1066	100%	0.0	95.90	686	MF426007.1
Rhipicephalus sanguineus sensu lato 'temperate lineage' isolat...	Rhipicephalu...	brown dog tick	34632	1061	1061	100%	0.0	95.74	697	MF426019.1
Rhipicephalus sanguineus sensu lato 'temperate lineage' isolat...	Rhipicephalu...	brown dog tick	34632	1061	1061	100%	0.0	95.74	705	MF426006.1
Rhipicephalus sanguineus haplotype III cytochrome oxidase...	Rhipicephalu...	brown dog tick	34632	1061	1061	100%	0.0	95.74	713	KU556745.1
Rhipicephalus sanguineus isolate Arizona-1 cytochrome c oxidas...	Rhipicephalu...	brown dog tick	34632	1061	1061	100%	0.0	95.74	710	MN593344.1
Rhipicephalus sanguineus isolate Arizona-2 cytochrome c oxidas...	Rhipicephalu...	brown dog tick	34632	1061	1061	100%	0.0	95.74	710	MN593343.1
Rhipicephalus sanguineus isolate 11N cytochrome oxidase subuni...	Rhipicephalu...	brown dog tick	34632	1061	1061	100%	0.0	95.74	703	MN585197.1

```
Rhipicephalus sanguineus cytochrome c oxidase I (COI) gene,...
Rhipicephalu... brown dog tick  34632      1061   1061  100%  0.0   95.74  793
     AF132839.1
Rhipicephalus sanguineus sensu lato 'temperate lineage' isolat...
Rhipicephalu... brown dog tick  34632      1055   1055  100%  0.0   95.59  680
     MF426017.1
Rhipicephalus sanguineus sensu lato 'temperate lineage' isolat...
Rhipicephalu... brown dog tick  34632      1055   1055  100%  0.0   95.59  690
     MF426008.1
Rhipicephalus sanguineus sensu lato 'temperate lineage' isolat...
Rhipicephalu... brown dog tick  34632      1055   1055  100%  0.0   95.59  684
     MF426001.1
Rhipicephalus sanguineus sensu lato 'temperate lineage' isolat...
Rhipicephalu... brown dog tick  34632      1055   1055  100%  0.0   95.59  689
     MF425998.1
Rhipicephalus sanguineus sensu lato 'temperate lineage' isolat...
Rhipicephalu... brown dog tick  34632      1055   1055  100%  0.0   95.59  695
     MF425997.1
Rhipicephalus sanguineus haplotype IV cytochrome oxidase subun...
Rhipicephalu... brown dog tick  34632      1055   1113  100%  0.0   95.59  771
     KU556746.1
Rhipicephalus sanguineus voucher CROBB813 cytochrome oxidase...
Rhipicephalu... brown dog tick  34632      1055   1055  100%  0.0   95.59  658
     MZ305546.1
Rhipicephalus sanguineus voucher CROBB810 cytochrome oxidase...
Rhipicephalu... brown dog tick  34632      1055   1055  100%  0.0   95.59  658
     MZ305545.1
Rhipicephalus sanguineus isolate 5MSK cytochrome c oxidase...
Rhipicephalu... brown dog tick  34632      1055   1055  100%  0.0   95.59  676
     MW152145.1
Rhipicephalus sanguineus isolate 1MSK cytochrome c oxidase...
Rhipicephalu... brown dog tick  34632      1055   1055  100%  0.0   95.59  675
     MW152144.1
Rhipicephalus sanguineus isolate RSN2SK cytochrome c oxidase...
Rhipicephalu... brown dog tick  34632      1055   1055  100%  0.0   95.59  676
     MW152143.1
Rhipicephalus sanguineus isolate RSN11SK cytochrome c oxidase...
Rhipicephalu... brown dog tick  34632      1055   1055  100%  0.0   95.59  669
     MW152142.1
Rhipicephalus sanguineus sensu lato 'temperate lineage' isolat...
Rhipicephalu... brown dog tick  34632      1050   1050  100%  0.0   95.44  684
     MF426012.1
Rhipicephalus sanguineus haplotype I cytochrome oxidase subuni...
Rhipicephalu... brown dog tick  34632      1048   1048  98%   0.0   95.83  659
     KU556743.1
Rhipicephalus sanguineus haplotype II cytochrome oxidase subun...
Rhipicephalu... brown dog tick  34632      1046   1046  100%  0.0   95.30  787
     KU556744.1
Rhipicephalus sanguineus sensu lato 'temperate lineage' isolat...
Rhipicephalu... brown dog tick  34632      1038   1038  97%   0.0   95.80  812
     MF426020.1
Rhipicephalus sanguineus sensu lato 'temperate lineage' isolat...
Rhipicephalu... brown dog tick  34632      1037   1037  98%   0.0   95.52  824
     MF426010.1
```

Rhipicephalus sanguineus sensu lato 'temperate lineage' isolat...
Rhipicephalu... brown dog tick 34632 1031 1031 97% 0.0 95.64 809
MF426011.1
Rhipicephalus sanguineus sensu lato 'temperate lineage' isolat...
Rhipicephalu... brown dog tick 34632 1022 1022 94% 0.0 96.30 649
MF426014.1
Rhipicephalus sanguineus isolate LIC5424B cytochrome oxidase...
Rhipicephalu... brown dog tick 34632 1020 1020 97% 0.0 95.33 768
KX383818.1
Rhipicephalus sanguineus sensu lato 'temperate lineage' isolat...
Rhipicephalu... brown dog tick 34632 1016 1016 94% 0.0 96.14 635
MF426000.1
Rhipicephalus sanguineus sensu lato 'temperate lineage' isolat...
Rhipicephalu... brown dog tick 34632 1016 1016 97% 0.0 95.31 654
MF425988.1
Rhipicephalus sanguineus isolate LIC5533B cytochrome oxidase...
Rhipicephalu... brown dog tick 34632 1013 1013 96% 0.0 95.30 764
KX383811.1
Rhipicephalus sanguineus isolate LIC4736B cytochrome oxidase...
Rhipicephalu... brown dog tick 34632 1011 1011 96% 0.0 95.29 763
KX383807.1
Rhipicephalus sanguineus voucher Rs7 cytochrome oxidase subuni...
Rhipicephalu... brown dog tick 34632 1009 1009 95% 0.0 95.56 656
KU568516.1
Rhipicephalus sanguineus isolate LIC4736D cytochrome oxidase...
Rhipicephalu... brown dog tick 34632 1009 1009 96% 0.0 95.28 762
KX383809.1
Rhipicephalus sanguineus isolate LIC4727D cytochrome oxidase...
Rhipicephalu... brown dog tick 34632 1009 1009 96% 0.0 95.28 714
KX383806.1
Rhipicephalus sanguineus isolate LIC4727C cytochrome oxidase...
Rhipicephalu... brown dog tick 34632 1007 1007 96% 0.0 95.28 761
KX383805.1
Rhipicephalus sanguineus isolate Samont2 cytochrome oxidase...
Rhipicephalu... brown dog tick 34632 1005 1005 94% 0.0 95.82 644
MH630347.1
Rhipicephalus sanguineus isolate Samont1 cytochrome oxidase...
Rhipicephalu... brown dog tick 34632 1005 1005 94% 0.0 95.82 644
MH630346.1
Rhipicephalus sanguineus clone #7 cytochrome oxidase subunit I...
Rhipicephalu... brown dog tick 34632 1002 1002 95% 0.0 95.53 654
KX519712.1
Rhipicephalus sanguineus sensu lato 'temperate lineage' isolat...
Rhipicephalu... brown dog tick 34632 1000 1000 95% 0.0 95.38 639
MF426002.1
Rhipicephalus sanguineus isolate Rsan-ML4 cytochrome oxidase...
Rhipicephalu... brown dog tick 34632 990 990 94% 0.0 95.48 630
MG855658.1
Rhipicephalus sanguineus isolate Rsan-ML3 cytochrome oxidase...
Rhipicephalu... brown dog tick 34632 990 990 94% 0.0 95.48 630
MG855657.1
Rhipicephalus sanguineus isolate Rsan-ML2 cytochrome oxidase...
Rhipicephalu... brown dog tick 34632 990 990 94% 0.0 95.48 630
MG855656.1

Rhipicephalus sanguineus isolate Kehf-Lagareb-A cytochrome...
Rhipicephalu... brown dog tick 34632 990 990 94% 0.0 95.48 630
KX757910.1
Rhipicephalus sanguineus isolate Italy-B cytochrome oxidase...
Rhipicephalu... brown dog tick 34632 990 990 94% 0.0 95.48 630
KX757904.1
Rhipicephalus sanguineus isolate Malta-A cytochrome oxidase...
Rhipicephalu... brown dog tick 34632 990 990 94% 0.0 95.48 630
KX757902.1
Rhipicephalus sanguineus isolate Zagreb-C cytochrome oxidase...
Rhipicephalu... brown dog tick 34632 990 990 94% 0.0 95.48 630
KX757896.1
Rhipicephalus sanguineus isolate Pula-B cytochrome oxidase...
Rhipicephalu... brown dog tick 34632 990 990 94% 0.0 95.48 630
KX757889.1
Rhipicephalus sanguineus isolate Rovind cytochrome oxidase...
Rhipicephalu... brown dog tick 34632 990 990 94% 0.0 95.48 630
KX757887.1
Rhipicephalus sanguineus isolate Kajtasovo cytochrome oxidase...
Rhipicephalu... brown dog tick 34632 990 990 94% 0.0 95.48 630
KX757879.1
Rhipicephalus sanguineus clone #6 cytochrome oxidase subunit I...
Rhipicephalu... brown dog tick 34632 990 990 94% 0.0 95.48 636
KX519711.1
Rhipicephalus sanguineus isolate SV4 cytochrome c oxidase...
Rhipicephalu... brown dog tick 34632 990 990 94% 0.0 95.48 630
MW135448.1
Rhipicephalus sanguineus isolate Boka-A cytochrome oxidase...
Rhipicephalu... brown dog tick 34632 985 985 94% 0.0 95.32 630
KX757905.1
Rhipicephalus sanguineus isolate Sibenik cytochrome oxidase...
Rhipicephalu... brown dog tick 34632 985 985 94% 0.0 95.32 630
KX757895.1
Rhipicephalus sanguineus isolate Zadar cytochrome oxidase...
Rhipicephalu... brown dog tick 34632 985 985 94% 0.0 95.32 630
KX757892.1
Rhipicephalus sanguineus isolate Zagreb-A cytochrome oxidase...
Rhipicephalu... brown dog tick 34632 985 985 94% 0.0 95.32 630
KX757890.1
Rhipicephalus sanguineus isolate Pula-A cytochrome oxidase...
Rhipicephalu... brown dog tick 34632 985 985 94% 0.0 95.32 630
KX757888.1
Rhipicephalus sanguineus isolate Petnica cytochrome oxidase...
Rhipicephalu... brown dog tick 34632 985 985 94% 0.0 95.32 630
KX757883.1
Rhipicephalus sanguineus clone #3 cytochrome oxidase subunit I...
Rhipicephalu... brown dog tick 34632 985 985 93% 0.0 95.46 629
KX519708.1
Rhipicephalus sanguineus isolate SV2 cytochrome c oxidase...
Rhipicephalu... brown dog tick 34632 985 985 94% 0.0 95.32 630
MW135447.1
Rhipicephalus sanguineus clone #2 cytochrome oxidase subunit I...
Rhipicephalu... brown dog tick 34632 983 983 93% 0.0 95.45 626
KX519707.1

Rhipicephalus sanguineus isolate Szekszard cytochrome oxidase...
Rhipicephalu... brown dog tick 34632 979 979 94% 0.0 95.16 630
KX757901.1
Rhipicephalus sanguineus clone #4 cytochrome oxidase subunit I...
Rhipicephalu... brown dog tick 34632 974 974 94% 0.0 95.00 635
KX519709.1
Rhipicephalus sanguineus isolate LIC4723B cytochrome oxidase...
Rhipicephalu... brown dog tick 34632 937 937 90% 0.0 94.97 723
KX714599.1
Rhipicephalus sanguineus clone #8 cytochrome oxidase subunit I...
Rhipicephalu... brown dog tick 34632 933 933 88% 0.0 95.69 580
KX519713.1
Rhipicephalus sanguineus clone #1 cytochrome oxidase subunit I...
Rhipicephalu... brown dog tick 34632 898 898 86% 0.0 95.09 595
KX519706.1
Rhipicephalus sanguineus isolate Rsang2 cytochrome oxidase...
Rhipicephalu... brown dog tick 34632 883 883 100% 0.0 90.90 790
KY678135.1
Rhipicephalus turanicus isolate COX1-225-Goat cytochrome c...
Rhipicephalu... NA 34633 883 883 100% 0.0 90.88 673
MT800313.1
Rhipicephalus sanguineus sensu lato 'temperate lineage' isolat...
Rhipicephalu... brown dog tick 34632 881 881 85% 0.0 95.01 574
MF425987.1
Rhipicephalus sanguineus cytochrome oxidase subunit 1 (CO1)...
Rhipicephalu... brown dog tick 34632 876 876 98% 0.0 91.05 829
KU214592.1
Rhipicephalus turanicus isolate GY34-1 cytochrome c oxidase...
Rhipicephalu... NA 34633 872 872 99% 0.0 90.80 678
MT079206.1
Rhipicephalus sanguineus isolate GY34 cytochrome c oxidase...
Rhipicephalu... brown dog tick 34632 872 872 99% 0.0 90.80 678
MN862754.1
Rhipicephalus sanguineus isolate Gilan-e Gharb cytochrome...
Rhipicephalu... brown dog tick 34632 872 872 100% 0.0 90.58 1539
KM494916.1
Rhipicephalus sanguineus isolate Tehran cytochrome oxidase...
Rhipicephalu... brown dog tick 34632 872 872 100% 0.0 90.58 1539
KM494915.1
Rhipicephalus turanicus isolate COX1-18-Sheep cytochrome c...
Rhipicephalu... NA 34633 870 870 99% 0.0 90.56 673
MT800314.1
Rhipicephalus turanicus isolate 31X cytochrome oxidase subunit...
Rhipicephalu... NA 34633 861 861 100% 0.0 90.27 873
KY606303.1
Rhipicephalus turanicus isolate 19X cytochrome oxidase subunit...
Rhipicephalu... NA 34633 861 861 100% 0.0 90.27 873
KY606302.1
Rhipicephalus turanicus isolate 19C cytochrome oxidase subunit...
Rhipicephalu... NA 34633 861 861 100% 0.0 90.27 873
KY606301.1
Rhipicephalus turanicus isolate 12X cytochrome oxidase subunit...
Rhipicephalu... NA 34633 861 861 100% 0.0 90.27 873
KY606300.1

```
Rhipicephalus turanicus isolate 12C cytochrome oxidase subunit...
Rhipicephalu... NA              34633      861    861   100%  0.0   90.27  873
     KY606299.1
Rhipicephalus camicasi isolate SC0126 mitochondrion, complete...
Rhipicephalu... NA              669981     861    861   100%  0.0   90.29  14725
     NC_061616.1
Rhipicephalus turanicus voucher CROBB808 cytochrome oxidase...
Rhipicephalu... NA              34633      861    861   100%  0.0   90.27  658
     MZ305547.1
Rhipicephalus turanicus isolate Xinjiang-WQIA cytochrome oxida...
Rhipicephalu... NA              34633      850    850   100%  0.0   89.97  889
     MF002581.1
Rhipicephalus turanicus isolate Xinjiang-WS cytochrome oxidase...
Rhipicephalu... NA              34633      850    850   100%  0.0   89.97  889
     MF002580.1
Rhipicephalus turanicus isolate Xinjiang-BC cytochrome oxidase...
Rhipicephalu... NA              34633      850    850   100%  0.0   89.97  889
     MF002579.1
Rhipicephalus turanicus isolate Xinjiang-YC cytochrome oxidase...
Rhipicephalu... NA              34633      850    850   100%  0.0   89.97  889
     MF002578.1
Rhipicephalus turanicus isolate Xinjiang-PS cytochrome oxidase...
Rhipicephalu... NA              34633      850    850   100%  0.0   89.97  889
     MF002577.1
Rhipicephalus turanicus isolate SHZ cytochrome oxidase subunit...
Rhipicephalu... NA              34633      850    850   100%  0.0   89.97  889
     KY069271.1
Rhipicephalus turanicus isolate FK-1 cytochrome oxidase subuni...
Rhipicephalu... NA              34633      850    850   100%  0.0   89.97  889
     KY606291.1
Rhipicephalus turanicus isolate YN3 cytochrome oxidase subunit...
Rhipicephalu... NA              34633      850    850   100%  0.0   89.97  889
     KY606290.1
Rhipicephalus turanicus isolate YN2 cytochrome oxidase subunit...
Rhipicephalu... NA              34633      850    850   100%  0.0   89.97  889
     KY606289.1


Alignments:

>Rhipicephalus sanguineus mitochondrial DNA, complete genome
Sequence ID: AF081829.1 Length: 14710
Range 1: 1191 to 1848

Score:1216 bits(658), Expect:0.0,
Identities:658/658(100%),  Gaps:0/658(0%), Strand: Plus/Plus

Query  1     AACAATATATTTAATTTTTGGAGCATGATCAGGTATATTAGGACTTAGTATAAGAATATT  60
             ||||||||||||||||||||||||||||||||||||||||||||||||||||||||||||
Sbjct  1191  AACAATATATTTAATTTTTGGAGCATGATCAGGTATATTAGGACTTAGTATAAGAATATT  1250

Query  61    AATTCGTATAGAATTAGGACAACCTGGAACTTTAATTGGAAATGATCAGATTTATAATGT  120
             ||||||||||||||||||||||||||||||||||||||||||||||||||||||||||||
Sbjct  1251  AATTCGTATAGAATTAGGACAACCTGGAACTTTAATTGGAAATGATCAGATTTATAATGT  1310
```

```
Query  121   AATCGTAACAGCACATGCATTTATTATAATTTTCTTTATAGTTATACCAATCATAATCGG  180
             ||||||||||||||||||||||||||||||||||||||||||||||||||||||||||||
Sbjct  1311  AATCGTAACAGCACATGCATTTATTATAATTTTCTTTATAGTTATACCAATCATAATCGG  1370

Query  181   AGGGTTCGGAAATTGATTAGTTCCTATTATATTAGGTGCCCCAGATATAGCATTTCCACG  240
             ||||||||||||||||||||||||||||||||||||||||||||||||||||||||||||
Sbjct  1371  AGGGTTCGGAAATTGATTAGTTCCTATTATATTAGGTGCCCCAGATATAGCATTTCCACG  1430

Query  241   AATAAATAATATAAGATTTTGACTTTTACCCCCCTCATTATTTTTATTGATTAACTCTTC  300
             ||||||||||||||||||||||||||||||||||||||||||||||||||||||||||||
Sbjct  1431  AATAAATAATATAAGATTTTGACTTTTACCCCCCTCATTATTTTTATTGATTAACTCTTC  1490

Query  301   ATTAATTGAATCCGGAGCTGGAACAGGATGAACAGTTTATCCCCCTCTATCTTCAAATTT  360
             ||||||||||||||||||||||||||||||||||||||||||||||||||||||||||||
Sbjct  1491  ATTAATTGAATCCGGAGCTGGAACAGGATGAACAGTTTATCCCCCTCTATCTTCAAATTT  1550

Query  361   ATCACATTATGGTCCATCAGTAGATTTAGCTATTTTTTCTCTTCACCTTGCTGGTGCTTC  420
             ||||||||||||||||||||||||||||||||||||||||||||||||||||||||||||
Sbjct  1551  ATCACATTATGGTCCATCAGTAGATTTAGCTATTTTTTCTCTTCACCTTGCTGGTGCTTC  1610

Query  421   TTCAATTTTAGGTGCAATTAACTTCATCACAACTATTGTAAATATACGATCAATTGGAAT  480
             ||||||||||||||||||||||||||||||||||||||||||||||||||||||||||||
Sbjct  1611  TTCAATTTTAGGTGCAATTAACTTCATCACAACTATTGTAAATATACGATCAATTGGAAT  1670

Query  481   AACAATAGAACGAATACCATTATTTGTTTGATCTGTTTTAATTACAGCTATTTTACTACT  540
             ||||||||||||||||||||||||||||||||||||||||||||||||||||||||||||
Sbjct  1671  AACAATAGAACGAATACCATTATTTGTTTGATCTGTTTTAATTACAGCTATTTTACTACT  1730

Query  541   TCTATCTTTACCTGTATTAGCAGGTGCCATTACAATATTATTAACTGATCGAAACTTTAA  600
             ||||||||||||||||||||||||||||||||||||||||||||||||||||||||||||
Sbjct  1731  TCTATCTTTACCTGTATTAGCAGGTGCCATTACAATATTATTAACTGATCGAAACTTTAA  1790

Query  601   CACATCATTTTTTGATCCTTCAGGAGGAGGAGATCCCATTTTATATCAACACTTATTT  658
             ||||||||||||||||||||||||||||||||||||||||||||||||||||||||||
Sbjct  1791  CACATCATTTTTTGATCCTTCAGGAGGAGGAGATCCCATTTTATATCAACACTTATTT  1848
```

>Rhipicephalus sanguineus isolate Zagreb-B cytochrome oxidase subunit I (COI) gene, partial cds; mitochondrial
Sequence ID: KX757893.1 Length: 629
Range 1: 1 to 620

Score:1140 bits(617), Expect:0.0,
Identities:619/620(99%), Gaps:0/620(0%), Strand: Plus/Plus

```
Query  39   TAGGACTTAGTATAAGAATATTAATTCGTATAGAATTAGGACAACCTGGAACTTTAATTG  98
            ||||||||||||||||||||||||||||||||||||||||||||||||||||||||||||
Sbjct  1    TAGGACTTAGTATAAGAATATTAATTCGTATAGAATTAGGACAACCTGGAACTTTAATTG  60

Query  99   GAAATGATCAGATTTATAATGTAATCGTAACAGCACATGCATTTATTATAATTTTCTTTA  158
            ||||||||||||||||||||||||||||||||||||||||||||||||||||||||||||
```

```
Sbjct  61   GAAATGATCAGATTTATAATGTAATCGTAACAGCACATGCATTTATTATAATTTTCTTTA  120

Query  159  TAGTTATACCAATCATAATCGGAGGGTTCGGAAATTGATTAGTTCCTATTATATTAGGTG  218
            ||||||||||||||||||||||||||||||||||||||||||||||||||||||||||||
Sbjct  121  TAGTTATACCAATCATAATCGGAGGGTTCGGAAATTGATTAGTTCCTATTATATTAGGTG  180

Query  219  CCCCAGATATAGCATTTCCACGAATAAATAATATAAGATTTTGACTTTTACCCCCCTCAT  278
            ||||||||||||||||||||||||||||||||||||||||||||||||||||||||||||
Sbjct  181  CCCCAGATATAGCATTTCCACGAATAAATAATATAAGATTTTGACTTTTACCCCCCTCAT  240

Query  279  TATTTTTATTGATTAACTCTTCATTAATTGAATCCGGAGCTGGAACAGGATGAACAGTTT  338
            ||||||||||||||||||||||||||||||||||||||||||||||||||||||||||||
Sbjct  241  TATTTTTATTGATTAACTCTTCATTAATTGAATCCGGAGCTGGAACAGGATGAACAGTTT  300

Query  339  ATCCCCCTCTATCTTCAAATTTATCACATTATGGTCCATCAGTAGATTTAGCTATTTTTT  398
            ||||||||||||||||||||||||||||||||||||||||||||||||||||||||||||
Sbjct  301  ATCCCCCTCTATCTTCAAATTTATCACATTATGGTCCATCAGTAGATTTAGCTATTTTTT  360

Query  399  CTCTTCACCTTGCTGGTGCTTCTTCAATTTTAGGTGCAATTAACTTCATCACAACTATTG  458
            ||||||||||||||||||||||||||||||||||||||||||||||||||||||||||||
Sbjct  361  CTCTTCACCTTGCTGGTGCTTCTTCAATTTTAGGTGCAATTAACTTCATCACAACTATTG  420

Query  459  TAAATATACGATCAATTGGAATAACAATAGAACGAATACCATTATTTGTTTGATCTGTTT  518
            ||||||||||||||||||||||||||||||||||||||||||||||||||||||||||||
Sbjct  421  TAAATATACGATCAATTGGAATAACAATAGAACGAATACCATTATTTGTTTGATCTGTTT  480

Query  519  TAATTACAGCTATTTTACTACTTCTATCTTTACCTGTATTAGCAGGTGCCATTACAATAT  578
            ||||||||||||||||||||||||||||||||||||||||||||||||||||||||||||
Sbjct  481  TAATTACAGCTATTTTACTACTTCTATCTTTACCTGTATTAGCAGGTGCCATTACAATAT  540

Query  579  TATTAACTGATCGAAACTTTAACACATCATTTTTTGATCCTTCAGGAGGAGGAGATCCCA  638
            ||||||||||||||||||||||||||||||||||||| ||||||||||||||||||||||
Sbjct  541  TATTAACTGATCGAAACTTTAACACATCATTTTTTGACCCTTCAGGAGGAGGAGATCCCA  600

Query  639  TTTTATATCAACACTTATTT  658
            ||||||||||||||||||||
Sbjct  601  TTTTATATCAACACTTATTT  620
```

>Rhipicephalus sanguineus isolate Italy-A cytochrome oxidase subunit I (COI) gene, partial cds; mitochondrial
Sequence ID: KX757903.1 Length: 630
Range 1: 1 to 620

Score:1129 bits(611), Expect:0.0,
Identities:617/620(99%), Gaps:0/620(0%), Strand: Plus/Plus

```
Query  39   TAGGACTTAGTATAAGAATATTAATTCGTATAGAATTAGGACAACCTGGAACTTTAATTG  98
            ||||||||||||||||||||||||||||||||||||||||||||||||||||||||||||
Sbjct  1    TAGGACTTAGTATAAGAATATTAATTCGTATAGAATTAGGACAACCTGGAACTTTAATTG  60

Query  99   GAAATGATCAGATTTATAATGTAATCGTAACAGCACATGCATTTATTATAATTTTCTTTA  158
```

```
                ||||||||||||||||||||||||||||||||||||||||||||||||||||||||||||
Sbjct  61       GAAATGATCAGATTTATAATGTAATCGTAACAGCACATGCATTTATTATAATTTTCTTTA  120

Query  159      TAGTTATACCAATCATAATCGGAGGGTTCGGAAATTGATTAGTTCCTATTATATTAGGTG  218
                ||||||||||||||||||||||||||||||||||||||||||||||||||||||||||||
Sbjct  121      TAGTTATACCAATCATAATCGGAGGGTTCGGAAATTGATTAGTTCCTATTATATTAGGTG  180

Query  219      CCCCAGATATAGCATTTCCACGAATAAATAATATAAGATTTTGACTTTTACCCCCCTCAT  278
                ||||||||||||||||||||||||||||||||||||||||||||||||||||||||||||
Sbjct  181      CCCCAGATATAGCATTTCCACGAATAAATAATATAAGATTTTGACTTTTACCCCCCTCAT  240

Query  279      TATTTTTATTGATTAACTCTTCATTAATTGAATCCGGAGCTGGAACAGGATGAACAGTTT  338
                |||||||||||||||| |||||||||||||||||||||||||||||||||||||||||||
Sbjct  241      TATTTTTATTGATTAATTCTTCATTAATTGAATCCGGAGCTGGAACAGGATGAACAGTTT  300

Query  339      ATCCCCCTCTATCTTCAAATTTATCACATTATGGTCCATCAGTAGATTTAGCTATTTTTT  398
                ||||||||||||||||||||||||||||||||||||||||||||||||||||||||||||
Sbjct  301      ATCCCCCTCTATCTTCAAATTTATCACATTATGGTCCATCAGTAGATTTAGCTATTTTTT  360

Query  399      CTCTTCACCTTGCTGGTGCTTCTTCAATTTTAGGTGCAATTAACTTCATCACAACTATTG  458
                ||||||||||||||||||||||||||||||||||||||||||||||||| ||||||||||
Sbjct  361      CTCTTCACCTTGCTGGTGCTTCTTCAATTTTAGGTGCAATTAACTTCATTACAACTATTG  420

Query  459      TAAATATACGATCAATTGGAATAACAATAGAACGAATACCATTATTTGTTTGATCTGTTT  518
                ||||||||||||||||||||||||||||||||||||||||||||||||||||||||||||
Sbjct  421      TAAATATACGATCAATTGGAATAACAATAGAACGAATACCATTATTTGTTTGATCTGTTT  480

Query  519      TAATTACAGCTATTTTACTACTTCTATCTTTACCTGTATTAGCAGGTGCCATTACAATAT  578
                ||||||||||||||||||||||||||||||||||||||||||||||||||||||||||||
Sbjct  481      TAATTACAGCTATTTTACTACTTCTATCTTTACCTGTATTAGCAGGTGCCATTACAATAT  540

Query  579      TATTAACTGATCGAAACTTTAACACATCATTTTTTGATCCTTCAGGAGGAGGAGATCCCA  638
                ||||||||||||||||||||||||||||||||||||| ||||||||||||||||||||||
Sbjct  541      TATTAACTGATCGAAACTTTAACACATCATTTTTTGACCCTTCAGGAGGAGGAGATCCCA  600

Query  639      TTTTATATCAACACTTATTT  658
                ||||||||||||||||||||
Sbjct  601      TTTTATATCAACACTTATTT  620
```

>Rhipicephalus sanguineus isolate Al-Hosema-A cytochrome oxidase subunit I (COI) gene, partial cds; mitochondrial
Sequence ID: KX757909.1 Length: 630
Range 1: 1 to 620

Score:1112 bits(602), Expect:0.0,
Identities:614/620(99%), Gaps:0/620(0%), Strand: Plus/Plus

```
Query  39       TAGGACTTAGTATAAGAATATTAATTCGTATAGAATTAGGACAACCTGGAACTTTAATTG  98
                ||||||||||||||||||||||||||||||||||||||||||||||||||||||||||||
Sbjct  1        TAGGACTTAGTATAAGAATATTAATTCGTATAGAATTAGGACAACCTGGAACTTTAATTG  60
```

```
Query  99   GAAATGATCAGATTTATAATGTAATCGTAACAGCACATGCATTTATTATAATTTTCTTTA  158
            ||||||||||||||||||||||||||||||||||||||||||||||||||||||||||||
Sbjct  61   GAAATGATCAGATTTATAATGTAATCGTAACAGCACATGCATTTATTATAATTTTCTTTA  120

Query  159  TAGTTATACCAATCATAATCGGAGGGTTCGGAAATTGATTAGTTCCTATTATATTAGGTG  218
            ||||||||||||| ||||||||||||||||||||||||||||||||||||||||||||||
Sbjct  121  TAGTTATACCAATAATAATCGGAGGGTTCGGAAATTGATTAGTTCCTATTATATTAGGTG  180

Query  219  CCCCAGATATAGCATTTCCACGAATAAATAATATAAGATTTTGACTTTTACCCCCCTCAT  278
            ||||||||||||||||||||||||||||||||||||||||||||||||||||||||||||
Sbjct  181  CCCCAGATATAGCATTTCCACGAATAAATAATATAAGATTTTGACTTTTACCCCCCTCAT  240

Query  279  TATTTTTATTGATTAACTCTTCATTAATTGAATCCGGAGCTGGAACAGGATGAACAGTTT  338
            |||||||||||||||||||||||||||||||||| |||||||||||||||||||||||||
Sbjct  241  TATTTTTATTGATTAACTCTTCATTAATTGAATCTGGAGCTGGAACAGGATGAACAGTTT  300

Query  339  ATCCCCCTCTATCTTCAAATTTATCACATTATGGTCCATCAGTAGATTTAGCTATTTTTT  398
            |||||||||||||||||||||||||||||||||| |||||||||||||||||||||||||
Sbjct  301  ATCCCCCTCTATCTTCAAATTTATCACATTATGGCCCATCAGTAGATTTAGCTATTTTTT  360

Query  399  CTCTTCACCTTGCTGGTGCTTCTTCAATTTTAGGTGCAATTAACTTCATCACAACTATTG  458
            ||||||||||||||||||||||||||||||||||||||||||||||||||||||||||||
Sbjct  361  CTCTTCACCTTGCTGGTGCTTCTTCAATTTTAGGTGCAATTAACTTCATCACAACTATTG  420

Query  459  TAAATATACGATCAATTGGAATAACAATAGAACGAATACCATTATTTGTTTGATCTGTTT  518
            ||||||||||||||||||||||||||||||||||||||||||||||||||||||||||||
Sbjct  421  TAAATATACGATCAATTGGAATAACAATAGAACGAATACCATTATTTGTTTGATCTGTTT  480

Query  519  TAATTACAGCTATTTTACTACTTCTATCTTTACCTGTATTAGCAGGTGCCATTACAATAT  578
            ||||||||||||||||||||||||||||||||||||||||||||||||||||||||||||
Sbjct  481  TAATTACAGCTATTTTACTACTTCTATCTTTACCTGTATTAGCAGGTGCCATTACAATAT  540

Query  579  TATTAACTGATCGAAACTTTAACACATCATTTTTGATCCTTCAGGAGGAGGAGATCCCA  638
            |||| ||||||||||||||||||||||||||||||| |||||||||||||| |||||||
Sbjct  541  TATTGACTGATCGAAACTTTAACACATCATTTTTGACCCTTCAGGAGGAGGGGATCCCA  600

Query  639  TTTTATATCAACACTTATTT  658
            ||||||||||||||||||||
Sbjct  601  TTTTATATCAACACTTATTT  620
```

>Rhipicephalus sanguineus sensu lato 'temperate lineage' isolate S48 cytochrome c oxidase subunit I (COI) gene, partial cds; mitochondrial
Sequence ID: MF426015.1 Length: 692
Range 1: 19 to 676

Score:1072 bits(580), Expect:0.0,
Identities:632/658(96%), Gaps:0/658(0%), Strand: Plus/Plus

```
Query  1    AACAATATATTTAATTTTTGGAGCATGATCAGGTATATTAGGACTTAGTATAAGAATATT  60
            ||||||||||||||||||||||||||||||||||||||||||||||||||||||||||||
Sbjct  19   AACAATATATTTAATTTTTGGAGCATGATCAGGTATATTAGGACTTAGTATAAGAATATT  78
```

```
Query  61    AATTCGTATAGAATTAGGACAACCTGGAACTTTAATTGGAAATGATCAGATTTATAATGT  120
             ||||||||||||||||||||||||||||||||||||||||||||||||||||||||||||
Sbjct  79    AATTCGTATAGAATTAGGACAACCTGGAACTTTAATTGGAAATGATCAGATTTATAATGT  138

Query  121   AATCGTAACAGCACATGCATTTATTATAATTTTCTTTATAGTTATACCAATCATAATCGG  180
             ||||||||||||||||||||||||||||||||| ||||||||||||||||||||||||||
Sbjct  139   AATCGTAACAGCACATGCATTTATTATAATTTTTTTTATAGTTATACCAATCATAATCGG  198

Query  181   AGGGTTCGGAAATTGATTAGTTCCTATTATATTAGGTGCCCCAGATATAGCATTTCCACG  240
             ||| || |||||||||||||| ||||| ||||| ||||||||||||||||||||||||||
Sbjct  199   AGGATTTGGAAATTGATTAGTCCCTATCATATTGGGTGCCCCAGATATAGCATTTCCACG  258

Query  241   AATAAATAATATAAGATTTTGACTTTTACCCCCCTCATTATTTTTATTGATTAACTCTTC  300
             |||||||||||||||||||||||||||||| || ||| |||||||||| |||||||||||
Sbjct  259   AATAAATAATATAAGATTTTGACTTTTACCTCCTTCACTATTTTTATTAATTAACTCTTC  318

Query  301   ATTAATTGAATCCGGAGCTGGAACAGGATGAACAGTTTATCCCCCTCTATCTTCAAATTT  360
             |||||||||||| |||||||| ||||||||||||||||| || |||||||||||||||||
Sbjct  319   ATTAATTGAATCTGGAGCTGGTACAGGATGAACAGTTTACCCTCCTCTATCTTCAAATTT  378

Query  361   ATCACATTATGGTCCATCAGTAGATTTAGCTATTTTTTCTCTTCACCTTGCTGGTGCTTC  420
             ||||||||||||||| ||||||||||||||||||||||||||||| ||||||||||||||
Sbjct  379   ATCACATTATGGTCCTTCAGTAGATTTAGCTATTTTTTCTCTTCATCTTGCTGGTGCTTC  438

Query  421   TTCAATTTTAGGTGCAATTAACTTCATCACAACTATTGTAAATATACGATCAATTGGAAT  480
             ||||||||||||||||||||||| || ||||||||||||||| |||||||||||||||||
Sbjct  439   TTCAATTTTAGGTGCAATTAACTTTATTACAACTATTGTAAACATACGATCAATTGGAAT  498

Query  481   AACAATAGAACGAATACCATTATTTGTTTGATCTGTTTTAATTACAGCTATTTTACTACT  540
             |||||||||||| |||||||||||||||||||||||||||||||||||||||||||||||
Sbjct  499   AACAATAGAACGTATACCATTATTTGTTTGATCTGTTTTAATTACAGCTATTTTACTACT  558

Query  541   TCTATCTTTACCTGTATTAGCAGGTGCCATTACAATATTATTAACTGATCGAAACTTTAA  600
             | ||||||||||||||||||||||||||||||||||||| ||||||||||||||||||||
Sbjct  559   TTTATCTTTACCTGTATTAGCAGGTGCCATTACAATATTGTTAACTGATCGAAACTTTAA  618

Query  601   CACATCATTTTTTGATCCTTCAGGAGGAGGAGATCCCATTTTATATCAACACTTATTT  658
              ||||||||||||||||||||||| ||||||||||| |||||||||||||| ||||||
Sbjct  619   TACATCATTTTTTGATCCTTCAGGGGGAGGAGATCCAATTTTATATCAACATTTATTT  676
```

>Rhipicephalus sanguineus sensu lato 'temperate lineage' isolate S58 cytochrome c oxidase subunit I (COI) gene, partial cds; mitochondrial
Sequence ID: MF426018.1 Length: 675
Range 1: 11 to 668

Score:1066 bits(577), Expect:0.0,
Identities:631/658(96%), Gaps:0/658(0%), Strand: Plus/Plus

```
Query  1     AACAATATATTTAATTTTTGGAGCATGATCAGGTATATTAGGACTTAGTATAAGAATATT  60
             ||||||||||||||||||||||||||||||||||||||||||||||||||||||||||||
```

```
Sbjct  11    AACAATATATTTAATTTTTGGAGCATGATCAGGTATATTAGGACTTAGTATAAGAATATT  70

Query  61    AATTCGTATAGAATTAGGACAACCTGGAACTTTAATTGGAAATGATCAGATTTATAATGT  120
             ||||||||||||||||||||||||||||||||||||||||||||||||||||||||||||
Sbjct  71    AATTCGTATAGAATTAGGACAACCTGGAACTTTAATTGGAAATGATCAGATTTATAATGT  130

Query  121   AATCGTAACAGCACATGCATTTATTATAATTTTCTTTATAGTTATACCAATCATAATCGG  180
             ||||||||||||||||||||||||||||||||| ||||||||||||||||||||||||||
Sbjct  131   AATCGTAACAGCACATGCATTTATTATAATTTTTTTTATAGTTATACCAATCATAATCGG  190

Query  181   AGGGTTCGGAAATTGATTAGTTCCTATTATATTAGGTGCCCCAGATATAGCATTTCCACG  240
             ||| || |||||||||||||| ||||| ||||| ||||||||||||||||||||||||||
Sbjct  191   AGGATTTGGAAATTGATTAGTCCCTATCATATTGGGTGCCCCAGATATAGCATTTCCACG  250

Query  241   AATAAATAATATAAGATTTTGACTTTTACCCCCCTCATTATTTTTATTGATTAACTCTTC  300
             |||||||||||||||||||||||||||||| || ||| |||||||||| |||||||||||
Sbjct  251   AATAAATAATATAAGATTTTGACTTTTACCTCCTTCACTATTTTTATTAATTAACTCTTC  310

Query  301   ATTAATTGAATCCGGAGCTGGAACAGGATGAACAGTTTATCCCCCTCTATCTTCAAATTT  360
             |||||||||||| |||||||| ||||||||||||||||| || |||||||||||||||||
Sbjct  311   ATTAATTGAATCTGGAGCTGGTACAGGATGAACAGTTTACCCTCCTCTATCTTCAAATTT  370

Query  361   ATCACATTATGGTCCATCAGTAGATTTAGCTATTTTTTCTCTTCACCTTGCTGGTGCTTC  420
             ||||||||||||||| ||||||||||||||||||||||||||||| ||||||||||||||
Sbjct  371   ATCACATTATGGTCCTTCAGTAGATTTAGCTATTTTTTCTCTTCATCTTGCTGGTGCTTC  430

Query  421   TTCAATTTTAGGTGCAATTAACTTCATCACAACTATTGTAAATATACGATCAATTGGAAT  480
             |||||||||||||||||||||||| || |||||||||||||| |||||||||||||||||
Sbjct  431   TTCAATTTTAGGTGCAATTAACTTTATTACAACTATTGTAAACATACGATCAATTGGAAT  490

Query  481   AACAATAGAACGAATACCATTATTTGTTTGATCTGTTTTAATTACAGCTATTTTACTACT  540
             |||||||||||| |||||||||||||||||||||||||||||||||||||||||| ||||
Sbjct  491   AACAATAGAACGTATACCATTATTTGTTTGATCTGTTTTAATTACAGCTATTTTATTACT  550

Query  541   TCTATCTTTACCTGTATTAGCAGGTGCCATTACAATATTATTAACTGATCGAAACTTTAA  600
             | ||||||||||||||||||||||||||||||||||||| ||||||||||||||||||||
Sbjct  551   TTTATCTTTACCTGTATTAGCAGGTGCCATTACAATATTGTTAACTGATCGAAACTTTAA  610

Query  601   CACATCATTTTTTGATCCTTCAGGAGGAGGAGATCCCATTTTATATCAACACTTATTT  658
              ||||||||||||||||||||||| ||||||||||| |||||||||||||| ||||||
Sbjct  611   TACATCATTTTTTGATCCTTCAGGGGGAGGAGATCCAATTTTATATCAACATTTATTT  668
```

>Rhipicephalus sanguineus sensu lato 'temperate lineage' isolate S52 cytochrome c oxidase subunit I (COI) gene, partial cds; mitochondrial
Sequence ID: MF426016.1 Length: 680
Range 1: 12 to 669

Score:1066 bits(577), Expect:0.0,
Identities:631/658(96%), Gaps:0/658(0%), Strand: Plus/Plus

```
Query  1     AACAATATATTTAATTTTTGGAGCATGATCAGGTATATTAGGACTTAGTATAAGAATATT  60
```

```
              ||||||||||||||||||||||||||||||||||||||||||||||||||||||||||||
Sbjct  12     AACAATATATTTAATTTTTGGAGCATGATCAGGTATATTAGGACTTAGTATAAGAATATT  71

Query  61     AATTCGTATAGAATTAGGACAACCTGGAACTTTAATTGGAAATGATCAGATTTATAATGT  120
              ||||||||||||||||||||||||||||||||||||||||||||||||||||||||||||
Sbjct  72     AATTCGTATAGAATTAGGACAACCTGGAACTTTAATTGGAAATGATCAGATTTATAATGT  131

Query  121    AATCGTAACAGCACATGCATTTATTATAATTTTCTTTATAGTTATACCAATCATAATCGG  180
              ||||||||||||||||||||||||||||||||| ||||||||||||||||||||||||||
Sbjct  132    AATCGTAACAGCACATGCATTTATTATAATTTTTTTTATAGTTATACCAATCATAATCGG  191

Query  181    AGGGTTCGGAAATTGATTAGTTCCTATTATATTAGGTGCCCCAGATATAGCATTTCCACG  240
              ||| || |||||||||||||| ||||| ||||| ||||||||||||||||||||||||||
Sbjct  192    AGGATTTGGAAATTGATTAGTCCCTATCATATTGGGTGCCCCAGATATAGCATTTCCACG  251

Query  241    AATAAATAATATAAGATTTTGACTTTTACCCCCCTCATTATTTTTATTGATTAACTCTTC  300
              |||||||||||||||||||||||||||||| || ||| |||||||||| |||||||||||
Sbjct  252    AATAAATAATATAAGATTTTGACTTTTACCTCCTTCACTATTTTTATTAATTAACTCTTC  311

Query  301    ATTAATTGAATCCGGAGCTGGAACAGGATGAACAGTTTATCCCCCTCTATCTTCAAATTT  360
              |||||||||||| |||||||| ||||||||||||||||| || |||||||||||||||||
Sbjct  312    ATTAATTGAATCTGGAGCTGGTACAGGATGAACAGTTTACCCTCCTCTATCTTCAAATTT  371

Query  361    ATCACATTATGGTCCATCAGTAGATTTAGCTATTTTTTCTCTTCACCTTGCTGGTGCTTC  420
              ||||||||||||||| ||||||||||||||||||||||||||||| ||||||||||||||
Sbjct  372    ATCACATTATGGTCCTTCAGTAGATTTAGCTATTTTTTCTCTTCATCTTGCTGGTGCTTC  431

Query  421    TTCAATTTTAGGTGCAATTAACTTCATCACAACTATTGTAAATATACGATCAATTGGAAT  480
              |||||||||||||||||||||||| || |||||||||||||| |||||||||||||||||
Sbjct  432    TTCAATTTTAGGTGCAATTAACTTTATTACAACTATTGTAAACATACGATCAATTGGAAT  491

Query  481    AACAATAGAACGAATACCATTATTTGTTTGATCTGTTTTAATTACAGCTATTTTACTACT  540
              |||||||||||| |||||||||||||||||||||||||||||||||||||||||| ||||
Sbjct  492    AACAATAGAACGTATACCATTATTTGTTTGATCTGTTTTAATTACAGCTATTTTATTACT  551

Query  541    TCTATCTTTACCTGTATTAGCAGGTGCCATTACAATATTATTAACTGATCGAAACTTTAA  600
              | |||||||||||||||||||||||||||||||||||||| |||||||||||||||||||
Sbjct  552    TTTATCTTTACCTGTATTAGCAGGTGCCATTACAATATTGTTAACTGATCGAAACTTTAA  611

Query  601    CACATCATTTTTTGATCCTTCAGGAGGAGGAGATCCCATTTTATATCAACACTTATTT  658
               ||||||||||||||||||||||| ||||||||||| |||||||||||||| ||||||
Sbjct  612    TACATCATTTTTTGATCCTTCAGGGGGAGGAGATCCAATTTTATATCAACATTTATTT  669
```

>Rhipicephalus sanguineus sensu lato 'temperate lineage' isolate S37 cytochrome c oxidase subunit I (COI) gene, partial cds; mitochondrial
Sequence ID: MF426013.1 Length: 693
Range 1: 20 to 677

Score:1066 bits(577), Expect:0.0,
Identities:631/658(96%), Gaps:0/658(0%), Strand: Plus/Plus

```
Query  1    AACAATATATTTAATTTTTGGAGCATGATCAGGTATATTAGGACTTAGTATAAGAATATT  60
            ||||||||||||||||||||||||||||||||||||||||||||||||||||||||||||
Sbjct  20   AACAATATATTTAATTTTTGGAGCATGATCAGGTATATTAGGACTTAGTATAAGAATATT  79

Query  61   AATTCGTATAGAATTAGGACAACCTGGAACTTTAATTGGAAATGATCAGATTTATAATGT  120
            ||||||||||||||||||||||||||||||||||||||||||||||||||||||||||||
Sbjct  80   AATTCGTATAGAATTAGGACAACCTGGAACTTTAATTGGAAATGATCAGATTTATAATGT  139

Query  121  AATCGTAACAGCACATGCATTTATTATAATTTTCTTTATAGTTATACCAATCATAATCGG  180
            ||||||||||||||||||||||||||||||||| ||||||||||||||||||||||||||
Sbjct  140  AATCGTAACAGCACATGCATTTATTATAATTTTTTTTATAGTTATACCAATCATAATCGG  199

Query  181  AGGGTTCGGAAATTGATTAGTTCCTATTATATTAGGTGCCCCAGATATAGCATTTCCACG  240
            ||| || |||||||||||||| ||||| ||||| ||||||||||||||||||||||||||
Sbjct  200  AGGATTTGGAAATTGATTAGTCCCTATCATATTGGGTGCCCCAGATATAGCATTTCCACG  259

Query  241  AATAAATAATATAAGATTTTGACTTTTACCCCCCTCATTATTTTTATTGATTAACTCTTC  300
            |||||||||||||||||||||||||||||| || ||| |||||||||| |||||||||||
Sbjct  260  AATAAATAATATAAGATTTTGACTTTTACCTCCTTCACTATTTTTATTAATTAACTCTTC  319

Query  301  ATTAATTGAATCCGGAGCTGGAACAGGATGAACAGTTTATCCCCCTCTATCTTCAAATTT  360
            |||||||||||| |||||||| ||||||||||||||||| || |||||||||||||||||
Sbjct  320  ATTAATTGAATCTGGAGCTGGTACAGGATGAACAGTTTACCCTCCTCTATCTTCAAATTT  379

Query  361  ATCACATTATGGTCCATCAGTAGATTTAGCTATTTTTCTCTTCACCTTGCTGGTGCTTC  420
            ||||||||||||||| |||||||||||||||||||||||||||| |||||||||||||||
Sbjct  380  ATCACATTATGGTCCTTCAGTAGATTTAGCTATTTTTCTCTTCATCTTGCTGGTGCTTC  439

Query  421  TTCAATTTTAGGTGCAATTAACTTCATCACAACTATTGTAAATATACGATCAATTGGAAT  480
            |||||||||||||||||||||||| || |||||||||||||| |||||||||||||||||
Sbjct  440  TTCAATTTTAGGTGCAATTAACTTTATTACAACTATTGTAAACATACGATCAATTGGAAT  499

Query  481  AACAATAGAACGAATACCATTATTTGTTTGATCTGTTTTAATTACAGCTATTTTACTACT  540
            |||||||||||| |||||||||||||||||||||||||||||||||||||||||| ||||
Sbjct  500  AACAATAGAACGTATACCATTATTTGTTTGATCTGTTTTAATTACAGCTATTTTATTACT  559

Query  541  TCTATCTTTACCTGTATTAGCAGGTGCCATTACAATATTATTAACTGATCGAAACTTTAA  600
            | ||||||||||||||||||||||||||||||||||||| ||||||||||||||||||||
Sbjct  560  TTTATCTTTACCTGTATTAGCAGGTGCCATTACAATATTGTTAACTGATCGAAACTTTAA  619

Query  601  CACATCATTTTTTGATCCTTCAGGAGGAGGAGATCCCATTTTATATCAACACTTATTT  658
             ||||||||||||||||||||||| ||||||||||| |||||||||||||| ||||||
Sbjct  620  TACATCATTTTTTGATCCTTCAGGGGGAGGAGATCCAATTTTATATCAACATTTATTT  677
```

>Rhipicephalus sanguineus sensu lato 'temperate lineage' isolate S33 cytochrome c oxidase subunit I (COI) gene, partial cds; mitochondrial
Sequence ID: MF426009.1 Length: 679
Range 1: 11 to 668

Score:1066 bits(577), Expect:0.0,
Identities:631/658(96%), Gaps:0/658(0%), Strand: Plus/Plus

```
Query  1    AACAATATATTTAATTTTTGGAGCATGATCAGGTATATTAGGACTTAGTATAAGAATATT  60
            ||||||||||||||||||||||||||||||||||||||||||||||||||||||||||||
Sbjct  11   AACAATATATTTAATTTTTGGAGCATGATCAGGTATATTAGGACTTAGTATAAGAATATT  70

Query  61   AATTCGTATAGAATTAGGACAACCTGGAACTTTAATTGGAAATGATCAGATTTATAATGT  120
            ||||||||||||||||||||||||||||||||||||||||||||||||||||||||||||
Sbjct  71   AATTCGTATAGAATTAGGACAACCTGGAACTTTAATTGGAAATGATCAGATTTATAATGT  130

Query  121  AATCGTAACAGCACATGCATTTATTATAATTTTCTTTATAGTTATACCAATCATAATCGG  180
            ||||||||||||||||||||||||||||||||| ||||||||||||||||||||||||||
Sbjct  131  AATCGTAACAGCACATGCATTTATTATAATTTTTTTTATAGTTATACCAATCATAATCGG  190

Query  181  AGGGTTCGGAAATTGATTAGTTCCTATTATATTAGGTGCCCCAGATATAGCATTTCCACG  240
            ||| || |||||||||||||| ||||| ||||| ||||||||||||||||||||||||||
Sbjct  191  AGGATTTGGAAATTGATTAGTCCCTATCATATTGGGTGCCCCAGATATAGCATTTCCACG  250

Query  241  AATAAATAATATAAGATTTTGACTTTTACCCCCCTCATTATTTTTATTGATTAACTCTTC  300
            |||||||||||||||||||||||||||||| || ||| |||||||||| |||||||||||
Sbjct  251  AATAAATAATATAAGATTTTGACTTTTACCTCCTTCACTATTTTTATTAATTAACTCTTC  310

Query  301  ATTAATTGAATCCGGAGCTGGAACAGGATGAACAGTTTATCCCCCTCTATCTTCAAATTT  360
            |||||||||||| |||||||| ||||||||||||||||| || |||||||||||||||||
Sbjct  311  ATTAATTGAATCTGGAGCTGGTACAGGATGAACAGTTTACCCTCCTCTATCTTCAAATTT  370

Query  361  ATCACATTATGGTCCATCAGTAGATTTAGCTATTTTTTCTCTTCACCTTGCTGGTGCTTC  420
            ||||||||||||||| ||||||||||||||||||||||||||||| ||||||||||||||
Sbjct  371  ATCACATTATGGTCCTTCAGTAGATTTAGCTATTTTTTCTCTTCATCTTGCTGGTGCTTC  430

Query  421  TTCAATTTTAGGTGCAATTAACTTCATCACAACTATTGTAAATATACGATCAATTGGAAT  480
            |||||||||||||||||||||||| || |||||||||||||| |||||||||||||||||
Sbjct  431  TTCAATTTTAGGTGCAATTAACTTTATTACAACTATTGTAAACATACGATCAATTGGAAT  490

Query  481  AACAATAGAACGAATACCATTATTTGTTTGATCTGTTTTAATTACAGCTATTTTACTACT  540
            |||||||||||| |||||||||||||||||||||||||||||||||||||||||| ||||
Sbjct  491  AACAATAGAACGTATACCATTATTTGTTTGATCTGTTTTAATTACAGCTATTTTATTACT  550

Query  541  TCTATCTTTACCTGTATTAGCAGGTGCCATTACAATATTATTAACTGATCGAAACTTTAA  600
            | ||||||||||||||||||||||||||||||||||||| ||||||||||||||||||||
Sbjct  551  TTTATCTTTACCTGTATTAGCAGGTGCCATTACAATATTGTTAACTGATCGAAACTTTAA  610

Query  601  CACATCATTTTTTGATCCTTCAGGAGGAGGAGATCCCATTTTATATCAACACTTATTT  658
             ||||||||||||||||||||||| ||||||||||| |||||||||||||| ||||||
Sbjct  611  TACATCATTTTTTGATCCTTCAGGGGGAGGAGATCCAATTTTATATCAACATTTATTT  668
```

>Rhipicephalus sanguineus sensu lato 'temperate lineage' isolate S29 cytochrome c oxidase subunit I (COI) gene, partial cds; mitochondrial
Sequence ID: MF426007.1 Length: 686
Range 1: 14 to 671

Score:1066 bits(577), Expect:0.0,

```
Identities:631/658(96%),  Gaps:0/658(0%), Strand: Plus/Plus

Query  1    AACAATATATTTAATTTTTGGAGCATGATCAGGTATATTAGGACTTAGTATAAGAATATT  60
            ||||||||||||||||||||||||||||||||||||||||||||||||||||||||||||
Sbjct  14   AACAATATATTTAATTTTTGGAGCATGATCAGGTATATTAGGACTTAGTATAAGAATATT  73

Query  61   AATTCGTATAGAATTAGGACAACCTGGAACTTTAATTGGAAATGATCAGATTTATAATGT  120
            ||||||||||||||||||||||||||||||||||||||||||||||||||||||||||||
Sbjct  74   AATTCGTATAGAATTAGGACAACCTGGAACTTTAATTGGAAATGATCAGATTTATAATGT  133

Query  121  AATCGTAACAGCACATGCATTTATTATAATTTTCTTTATAGTTATACCAATCATAATCGG  180
            ||||||||||||||||||||||||||||||||| ||||||||||||||||||||||||||
Sbjct  134  AATCGTAACAGCACATGCATTTATTATAATTTTTTTTATAGTTATACCAATCATAATCGG  193

Query  181  AGGGTTCGGAAATTGATTAGTTCCTATTATATTAGGTGCCCCAGATATAGCATTTCCACG  240
            ||| || |||||||||||||| ||||| ||||| ||||||||||||||||||||||||||
Sbjct  194  AGGATTTGGAAATTGATTAGTCCCTATCATATTGGGTGCCCCAGATATAGCATTTCCACG  253

Query  241  AATAAATAATATAAGATTTTGACTTTTACCCCCCTCATTATTTTTATTGATTAACTCTTC  300
            |||||||||||||||||||||||||||||| || ||| |||||||||| |||||||||||
Sbjct  254  AATAAATAATATAAGATTTTGACTTTTACCTCCTTCACTATTTTTATTAATTAACTCTTC  313

Query  301  ATTAATTGAATCCGGAGCTGGAACAGGATGAACAGTTTATCCCCCTCTATCTTCAAATTT  360
            |||||||||||| |||||||| ||||||||||||||||| || |||||||||||||||||
Sbjct  314  ATTAATTGAATCTGGAGCTGGTACAGGATGAACAGTTTACCCTCCTCTATCTTCAAATTT  373

Query  361  ATCACATTATGGTCCATCAGTAGATTTAGCTATTTTTTCTCTTCACCTTGCTGGTGCTTC  420
            ||||||||||||||| ||||||||||||||||||||||||||||| ||||||||||||||
Sbjct  374  ATCACATTATGGTCCTTCAGTAGATTTAGCTATTTTTTCTCTTCATCTTGCTGGTGCTTC  433

Query  421  TTCAATTTTAGGTGCAATTAACTTCATCACAACTATTGTAAATATACGATCAATTGGAAT  480
            ||||||||||||||||||||||| || |||||||||||||| ||||||||||||||||||
Sbjct  434  TTCAATTTTAGGTGCAATTAACTTTATTACAACTATTGTAAACATACGATCAATTGGAAT  493

Query  481  AACAATAGAACGAATACCATTATTTGTTTGATCTGTTTTAATTACAGCTATTTTACTACT  540
            |||||||||||| |||||||||||||||||||||||||||||||||||||||||| ||||
Sbjct  494  AACAATAGAACGTATACCATTATTTGTTTGATCTGTTTTAATTACAGCTATTTTATTACT  553

Query  541  TCTATCTTTACCTGTATTAGCAGGTGCCATTACAATATTATTAACTGATCGAAACTTTAA  600
            | ||||||||||||||||||||||||||||||||||||| ||||||||||||||||||||
Sbjct  554  TTTATCTTTACCTGTATTAGCAGGTGCCATTACAATATTGTTAACTGATCGAAACTTTAA  613

Query  601  CACATCATTTTTTGATCCTTCAGGAGGAGGAGATCCCATTTTATATCAACACTTATTT  658
             ||||||||||||||||||||||| ||||||||||| |||||||||||||| ||||||
Sbjct  614  TACATCATTTTTTGATCCTTCAGGGGGAGGAGATCCAATTTTATATCAACATTTATTT  671
```

>Rhipicephalus sanguineus sensu lato 'temperate lineage' isolate S827 cytochrome c oxidase subunit I (COI) gene, partial cds; mitochondrial
Sequence ID: MF426019.1 Length: 697
Range 1: 19 to 676

```
Score:1061 bits(574), Expect:0.0,
Identities:630/658(96%),  Gaps:0/658(0%), Strand: Plus/Plus

Query  1    AACAATATATTTAATTTTTGGAGCATGATCAGGTATATTAGGACTTAGTATAAGAATATT  60
            ||||||||||||||||||||||||||||||||||||||||||||||||||||||||||||
Sbjct  19   AACAATATATTTAATTTTTGGAGCATGATCAGGTATATTAGGACTTAGTATAAGAATATT  78

Query  61   AATTCGTATAGAATTAGGACAACCTGGAACTTTAATTGGAAATGATCAGATTTATAATGT  120
            ||||||||||||||||||||||||||||||||||||||||||||||||||||||||||||
Sbjct  79   AATTCGTATAGAATTAGGACAACCTGGAACTTTAATTGGAAATGATCAGATTTATAATGT  138

Query  121  AATCGTAACAGCACATGCATTTATTATAATTTTCTTTATAGTTATACCAATCATAATCGG  180
            ||||||||||||||||||||||||||||||||| ||||||||||||||||||||||||||
Sbjct  139  AATCGTAACAGCACATGCATTTATTATAATTTTTTTTATAGTTATACCAATCATAATCGG  198

Query  181  AGGGTTCGGAAATTGATTAGTTCCTATTATATTAGGTGCCCCAGATATAGCATTTCCACG  240
            ||| || |||||||||||||| ||||| ||||| ||||||||||||||||||||||||||
Sbjct  199  AGGATTTGGAAATTGATTAGTCCCTATCATATTGGGTGCCCCAGATATAGCATTTCCACG  258

Query  241  AATAAATAATATAAGATTTTGACTTTTACCCCCCTCATTATTTTTATTGATTAACTCTTC  300
            |||||||||||||||||||||||||||||| || ||| |||||||||| |||||||||||
Sbjct  259  AATAAATAATATAAGATTTTGACTTTTACCTCCTTCACTATTTTTATTAATTAACTCTTC  318

Query  301  ATTAATTGAATCCGGAGCTGGAACAGGATGAACAGTTTATCCCCCTCTATCTTCAAATTT  360
            |||||||||||| |||||||| ||||||||||||||||| || |||||||||||||||||
Sbjct  319  ATTAATTGAATCTGGAGCTGGTACAGGATGAACAGTTTACCCTCCTCTATCTTCAAATTT  378

Query  361  ATCACATTATGGTCCATCAGTAGATTTAGCTATTTTTTCTCTTCACCTTGCTGGTGCTTC  420
            |||||||||||| || ||||||||||||||||||||||||||||| ||||||||||||||
Sbjct  379  ATCACATTATGGCCCTTCAGTAGATTTAGCTATTTTTTCTCTTCATCTTGCTGGTGCTTC  438

Query  421  TTCAATTTTAGGTGCAATTAACTTCATCACAACTATTGTAAATATACGATCAATTGGAAT  480
            ||||||||||||||||||||||| || ||||||||||||||| |||||||||||||||||
Sbjct  439  TTCAATTTTAGGTGCAATTAACTTTATTACAACTATTGTAAACATACGATCAATTGGAAT  498

Query  481  AACAATAGAACGAATACCATTATTTGTTTGATCTGTTTTAATTACAGCTATTTTACTACT  540
            |||||||||||| ||||||||||||||||||||||||||||||||||||||||||| ||||
Sbjct  499  AACAATAGAACGTATACCATTATTTGTTTGATCTGTTTTAATTACAGCTATTTTATTACT  558

Query  541  TCTATCTTTACCTGTATTAGCAGGTGCCATTACAATATTATTAACTGATCGAAACTTTAA  600
            | ||||||||||||||||||||||||||||||||||||| ||||||||||||||||||||
Sbjct  559  TTTATCTTTACCTGTATTAGCAGGTGCCATTACAATATTGTTAACTGATCGAAACTTTAA  618

Query  601  CACATCATTTTTTGATCCTTCAGGAGGAGGAGATCCCATTTTATATCAACACTTATTT  658
             ||||||||||||||||||||||| ||||||||||| |||||||||||||| ||||||
Sbjct  619  TACATCATTTTTTGATCCTTCAGGGGGAGGAGATCCAATTTTATATCAACATTTATTT  676


>Rhipicephalus sanguineus sensu lato 'temperate lineage' isolate S1060
cytochrome c oxidase subunit I (COI) gene, partial cds; mitochondrial
Sequence ID: MF426006.1 Length: 705
Range 1: 28 to 685
```

```
Score:1061 bits(574), Expect:0.0,
Identities:630/658(96%),  Gaps:0/658(0%), Strand: Plus/Plus

Query  1    AACAATATATTTAATTTTTGGAGCATGATCAGGTATATTAGGACTTAGTATAAGAATATT  60
            ||||||||||||||||||||||||||||||||||||||||||||||||||||||||||||
Sbjct  28   AACAATATATTTAATTTTTGGAGCATGATCAGGTATATTAGGACTTAGTATAAGAATATT  87

Query  61   AATTCGTATAGAATTAGGACAACCTGGAACTTTAATTGGAAATGATCAGATTTATAATGT  120
            ||||||||||||||||||||||||||||||||||||||||||||||||||||||||||||
Sbjct  88   AATTCGTATAGAATTAGGACAACCTGGAACTTTAATTGGAAATGATCAGATTTATAATGT  147

Query  121  AATCGTAACAGCACATGCATTTATTATAATTTCTTTATAGTTATACCAATCATAATCGG  180
            |||||||||||||||||||||||||||||||| |||||||||||||||||||||||||||
Sbjct  148  AATCGTAACAGCACATGCATTTATTATAATTTTTTTATAGTTATACCAATCATAATCGG  207

Query  181  AGGGTTCGGAAATTGATTAGTTCCTATTATATTAGGTGCCCCAGATATAGCATTTCCACG  240
            ||| || |||||||||||||| ||||| ||||| ||||||||||||||||||||||||||
Sbjct  208  AGGATTTGGAAATTGATTAGTCCCTATCATATTGGGTGCCCCAGATATAGCATTTCCACG  267

Query  241  AATAAATAATATAAGATTTTGACTTTTACCCCCCTCATTATTTTTATTGATTAACTCTTC  300
            |||||||||||||||||||||||||||||| || ||| |||||||||| |||||||||||
Sbjct  268  AATAAATAATATAAGATTTTGACTTTTACCTCCTTCACTATTTTTATTAATTAACTCTTC  327

Query  301  ATTAATTGAATCCGGAGCTGGAACAGGATGAACAGTTTATCCCCCTCTATCTTCAAATTT  360
            |||||||||||| |||||||| ||||||||||||||||| || |||||||||||||||||
Sbjct  328  ATTAATTGAATCTGGAGCTGGTACAGGATGAACAGTTTACCCTCCTCTATCTTCAAATTT  387

Query  361  ATCACATTATGGTCCATCAGTAGATTTAGCTATTTTTCTCTTCACCTTGCTGGTGCTTC  420
            ||||||||||||||| |||||||||||||||||||||||||||| |||||||||||||||
Sbjct  388  ATCACATTATGGTCCTTCAGTAGATTTAGCTATTTTTCTCTTCATCTTGCTGGTGCTTC  447

Query  421  TTCAATTTTAGGTGCAATTAACTTCATCACAACTATTGTAAATATACGATCAATTGGAAT  480
            |||||||||||||||||||||||| || |||||||||||||| ||||||| |||||||||
Sbjct  448  TTCAATTTTAGGTGCAATTAACTTTATTACAACTATTGTAAACATACGATTAATTGGAAT  507

Query  481  AACAATAGAACGAATACCATTATTTGTTTGATCTGTTTTAATTACAGCTATTTTACTACT  540
            |||||||||||| |||||||||||||||||||||||||||||||||||||||||| ||||
Sbjct  508  AACAATAGAACGTATACCATTATTTGTTTGATCTGTTTTAATTACAGCTATTTTATTACT  567

Query  541  TCTATCTTTACCTGTATTAGCAGGTGCCATTACAATATTATTAACTGATCGAAACTTTAA  600
            | ||||||||||||||||||||||||||||||||||||| ||||||||||||||||||||
Sbjct  568  TTTATCTTTACCTGTATTAGCAGGTGCCATTACAATATTGTTAACTGATCGAAACTTTAA  627

Query  601  CACATCATTTTTTGATCCTTCAGGAGGAGGAGATCCCATTTTATATCAACACTTATTT  658
             ||||||||||||||||||||||| ||||||||||| |||||||||||||| ||||||
Sbjct  628  TACATCATTTTTTGATCCTTCAGGGGGAGGAGATCCAATTTTATATCAACATTTATTT  685
```

>Rhipicephalus sanguineus haplotype III cytochrome oxidase subunit I (COI) gene, partial cds; mitochondrial
Sequence ID: KU556745.1 Length: 713

```
Range 1: 27 to 684

Score:1061 bits(574), Expect:0.0,
Identities:630/658(96%),  Gaps:0/658(0%), Strand: Plus/Plus

Query  1    AACAATATATTTAATTTTTGGAGCATGATCAGGTATATTAGGACTTAGTATAAGAATATT  60
            ||||||||||||||||||||||||||||||||||||||||||||||||||||||||||||
Sbjct  27   AACAATATATTTAATTTTTGGAGCATGATCAGGTATATTAGGACTTAGTATAAGAATATT  86

Query  61   AATTCGTATAGAATTAGGACAACCTGGAACTTTAATTGGAAATGATCAGATTTATAATGT  120
            ||||||||||||||||||||||||||||||||||||||||||||||||||||||||||||
Sbjct  87   AATTCGTATAGAATTAGGACAACCTGGAACTTTAATTGGAAATGATCAGATTTATAATGT  146

Query  121  AATCGTAACAGCACATGCATTTATTATAATTTTCTTTATAGTTATACCAATCATAATCGG  180
            ||||||||||||||||||||||||||||||||| ||||||||||||||||||||||||||
Sbjct  147  AATCGTAACAGCACATGCATTTATTATAATTTTTTTTATAGTTATACCAATCATAATCGG  206

Query  181  AGGGTTCGGAAATTGATTAGTTCCTATTATATTAGGTGCCCCAGATATAGCATTTCCACG  240
            ||| || |||||||||||||| ||||| ||||| ||||||||||||||||||||||||||
Sbjct  207  AGGATTTGGAAATTGATTAGTCCCTATCATATTGGGTGCCCCAGATATAGCATTTCCACG  266

Query  241  AATAAATAATATAAGATTTTGACTTTTACCCCCCTCATTATTTTTATTGATTAACTCTTC  300
            |||||||||||||||||||||||||||||| || ||| |||||||||| |||||||||||
Sbjct  267  AATAAATAATATAAGATTTTGACTTTTACCTCCTTCACTATTTTTATTAATTAACTCTTC  326

Query  301  ATTAATTGAATCCGGAGCTGGAACAGGATGAACAGTTTATCCCCCTCTATCTTCAAATTT  360
            |||||||||||| |||||||| ||||||||||||||||| || |||||||||||||||||
Sbjct  327  ATTAATTGAATCTGGAGCTGGTACAGGATGAACAGTTTACCCTCCTCTATCTTCAAATTT  386

Query  361  ATCACATTATGGTCCATCAGTAGATTTAGCTATTTTTTCTCTTCACCTTGCTGGTGCTTC  420
            |||||||||||| || ||||||||||||||||||||||||||||| ||||||||||||||
Sbjct  387  ATCACATTATGGCCCTTCAGTAGATTTAGCTATTTTTTCTCTTCATCTTGCTGGTGCTTC  446

Query  421  TTCAATTTTAGGTGCAATTAACTTCATCACAACTATTGTAAATATACGATCAATTGGAAT  480
            |||||||||||||||||||||||| || |||||||||||||| |||||||||||||||||
Sbjct  447  TTCAATTTTAGGTGCAATTAACTTTATTACAACTATTGTAAACATACGATCAATTGGAAT  506

Query  481  AACAATAGAACGAATACCATTATTTGTTTGATCTGTTTTAATTACAGCTATTTTACTACT  540
            |||||||||||| |||||||||||||||||||||||||||||||||||||||||| ||||
Sbjct  507  AACAATAGAACGTATACCATTATTTGTTTGATCTGTTTTAATTACAGCTATTTTATTACT  566

Query  541  TCTATCTTTACCTGTATTAGCAGGTGCCATTACAATATTATTAACTGATCGAAACTTTAA  600
            | ||||||||||||||||||||||||||||||||||||| ||||||||||||||||||||
Sbjct  567  TTTATCTTTACCTGTATTAGCAGGTGCCATTACAATATTGTTAACTGATCGAAACTTTAA  626

Query  601  CACATCATTTTTTGATCCTTCAGGAGGAGGAGATCCCATTTTATATCAACACTTATTT  658
             ||||||||||||||||||||||| ||||||||||| |||||||||||||| ||||||
Sbjct  627  TACATCATTTTTTGATCCTTCAGGGGGAGGAGATCCAATTTTATATCAACATTTATTT  684
```

>Rhipicephalus sanguineus isolate Arizona-1 cytochrome c oxidase subunit 1 (COI) gene, partial cds; mitochondrial

Sequence ID: MN593344.1 Length: 710
Range 1: 27 to 684

Score:1061 bits(574), Expect:0.0,
Identities:630/658(96%), Gaps:0/658(0%), Strand: Plus/Minus

```
Query  1    AACAATATATTTAATTTTTGGAGCATGATCAGGTATATTAGGACTTAGTATAAGAATATT  60
            ||||||||||||||||||||||||||||||||||||||||||||||||||||||||||||
Sbjct  684  AACAATATATTTAATTTTTGGAGCATGATCAGGTATATTAGGACTTAGTATAAGAATATT  625

Query  61   AATTCGTATAGAATTAGGACAACCTGGAACTTTAATTGGAAATGATCAGATTTATAATGT  120
            ||||||||||||||||||||||||||||||||||||||||||||||||||||||||||||
Sbjct  624  AATTCGTATAGAATTAGGACAACCTGGAACTTTAATTGGAAATGATCAGATTTATAATGT  565

Query  121  AATCGTAACAGCACATGCATTTATTATAATTTTCTTTATAGTTATACCAATCATAATCGG  180
            ||||||||||||||||||||||||||||||||| ||||||||||||||||||||||||||
Sbjct  564  AATCGTAACAGCACATGCATTTATTATAATTTTTTTTATAGTTATACCAATCATAATCGG  505

Query  181  AGGGTTCGGAAATTGATTAGTTCCTATTATATTAGGTGCCCCAGATATAGCATTTCCACG  240
            ||| || |||||||||||||| ||||| ||||| ||||||||||||||||||||||||||
Sbjct  504  AGGATTTGGAAATTGATTAGTCCCTATCATATTGGGTGCCCCAGATATAGCATTTCCACG  445

Query  241  AATAAATAATATAAGATTTTGACTTTTACCCCCCTCATTATTTTTATTGATTAACTCTTC  300
            |||||||||||||||||||||||||||||| || ||| |||||||||| |||||||||||
Sbjct  444  AATAAATAATATAAGATTTTGACTTTTACCTCCTTCACTATTTTTATTAATTAACTCTTC  385

Query  301  ATTAATTGAATCCGGAGCTGGAACAGGATGAACAGTTTATCCCCCTCTATCTTCAAATTT  360
            |||||||||||| |||||||| ||||||||||||||||| || |||||||||||||||||
Sbjct  384  ATTAATTGAATCTGGAGCTGGTACAGGATGAACAGTTTACCCTCCTCTATCTTCAAATTT  325

Query  361  ATCACATTATGGTCCATCAGTAGATTTAGCTATTTTTTCTCTTCACCTTGCTGGTGCTTC  420
            |||||||||||| || ||||||||||||||||||||||||||||| ||||||||||||||
Sbjct  324  ATCACATTATGGCCCTTCAGTAGATTTAGCTATTTTTTCTCTTCATCTTGCTGGTGCTTC  265

Query  421  TTCAATTTTAGGTGCAATTAACTTCATCACAACTATTGTAAATATACGATCAATTGGAAT  480
            |||||||||||||||||||||||| || |||||||||||||| |||||||||||||||||
Sbjct  264  TTCAATTTTAGGTGCAATTAACTTTATTACAACTATTGTAAACATACGATCAATTGGAAT  205

Query  481  AACAATAGAACGAATACCATTATTTGTTTGATCTGTTTTAATTACAGCTATTTTACTACT  540
            |||||||||||| |||||||||||||||||||||||||||||||||||||||||| ||||
Sbjct  204  AACAATAGAACGTATACCATTATTTGTTTGATCTGTTTTAATTACAGCTATTTTATTACT  145

Query  541  TCTATCTTTACCTGTATTAGCAGGTGCCATTACAATATTATTAACTGATCGAAACTTTAA  600
            | ||||||||||||||||||||||||||||||||||||| ||||||||||||||||||||
Sbjct  144  TTTATCTTTACCTGTATTAGCAGGTGCCATTACAATATTGTTAACTGATCGAAACTTTAA  85

Query  601  CACATCATTTTTTGATCCTTCAGGAGGAGGAGATCCCATTTTATATCAACACTTATTT  658
             ||||||||||||||||||||||| ||||||||||| |||||||||||||| ||||||
Sbjct  84   TACATCATTTTTTGATCCTTCAGGGGGAGGAGATCCAATTTTATATCAACATTTATTT  27
```

>Rhipicephalus sanguineus isolate Arizona-2 cytochrome c oxidase subunit 1 (COI)

```
gene, partial cds; mitochondrial
Sequence ID: MN593343.1 Length: 710
Range 1: 27 to 684

Score:1061 bits(574), Expect:0.0,
Identities:630/658(96%),  Gaps:0/658(0%), Strand: Plus/Minus

Query  1    AACAATATATTTAATTTTTGGAGCATGATCAGGTATATTAGGACTTAGTATAAGAATATT  60
            ||||||||||||||||||||||||||||||||||||||||||||||||||||||||||||
Sbjct  684  AACAATATATTTAATTTTTGGAGCATGATCAGGTATATTAGGACTTAGTATAAGAATATT  625

Query  61   AATTCGTATAGAATTAGGACAACCTGGAACTTTAATTGGAAATGATCAGATTTATAATGT  120
            ||||||||||||||||||||||||||||||||||||||||||||||||||||||||||||
Sbjct  624  AATTCGTATAGAATTAGGACAACCTGGAACTTTAATTGGAAATGATCAGATTTATAATGT  565

Query  121  AATCGTAACAGCACATGCATTTATTATAATTTTCTTTATAGTTATACCAATCATAATCGG  180
            ||||||||||||||||||||||||||||||||| ||||||||||||||||||||||||||
Sbjct  564  AATCGTAACAGCACATGCATTTATTATAATTTTTTTTATAGTTATACCAATCATAATCGG  505

Query  181  AGGGTTCGGAAATTGATTAGTTCCTATTATATTAGGTGCCCCAGATATAGCATTTCCACG  240
            ||| || |||||||||||||| ||||| ||||| ||||||||||||||||||||||||||
Sbjct  504  AGGATTTGGAAATTGATTAGTCCCTATCATATTGGGTGCCCCAGATATAGCATTTCCACG  445

Query  241  AATAAATAATATAAGATTTTGACTTTTACCCCCCTCATTATTTTTATTGATTAACTCTTC  300
            |||||||||||||||||||||||||||||| || ||| |||||||||| |||||||||||
Sbjct  444  AATAAATAATATAAGATTTTGACTTTTACCTCCTTCACTATTTTTATTAATTAACTCTTC  385

Query  301  ATTAATTGAATCCGGAGCTGGAACAGGATGAACAGTTTATCCCCCTCTATCTTCAAATTT  360
            |||||||||||| |||||||| ||||||||||||||||| || |||||||||||||||||
Sbjct  384  ATTAATTGAATCTGGAGCTGGTACAGGATGAACAGTTTACCCTCCTCTATCTTCAAATTT  325

Query  361  ATCACATTATGGTCCATCAGTAGATTTAGCTATTTTTTCTCTTCACCTTGCTGGTGCTTC  420
            |||||||||||| || ||||||||||||||||||||||||||||| ||||||||||||||
Sbjct  324  ATCACATTATGGCCCTTCAGTAGATTTAGCTATTTTTTCTCTTCATCTTGCTGGTGCTTC  265

Query  421  TTCAATTTTAGGTGCAATTAACTTCATCACAACTATTGTAAATATACGATCAATTGGAAT  480
            |||||||||||||||||||||||| || |||||||||||||| |||||||||||||||||
Sbjct  264  TTCAATTTTAGGTGCAATTAACTTTATTACAACTATTGTAAACATACGATCAATTGGAAT  205

Query  481  AACAATAGAACGAATACCATTATTTGTTTGATCTGTTTTAATTACAGCTATTTTACTACT  540
            |||||||||||| |||||||||||||||||||||||||||||||||||||||||| ||||
Sbjct  204  AACAATAGAACGTATACCATTATTTGTTTGATCTGTTTTAATTACAGCTATTTTATTACT  145

Query  541  TCTATCTTTACCTGTATTAGCAGGTGCCATTACAATATTATTAACTGATCGAAACTTTAA  600
            | ||||||||||||||||||||||||||||||||||||| ||||||||||||||||||||
Sbjct  144  TTTATCTTTACCTGTATTAGCAGGTGCCATTACAATATTGTTAACTGATCGAAACTTTAA  85

Query  601  CACATCATTTTTTGATCCTTCAGGAGGAGGAGATCCCATTTTATATCAACACTTATTT  658
             ||||||||||||||||||||||| ||||||||||| |||||||||||||| ||||||
Sbjct  84   TACATCATTTTTTGATCCTTCAGGGGGAGGAGATCCAATTTTATATCAACATTTATTT  27
```

>Rhipicephalus sanguineus isolate 11N cytochrome oxidase subunit I (COX1) gene, partial cds; mitochondrial
Sequence ID: MN585197.1 Length: 703
Range 1: 22 to 679

Score:1061 bits(574), Expect:0.0,
Identities:630/658(96%), Gaps:0/658(0%), Strand: Plus/Minus

```
Query  1    AACAATATATTTAATTTTTGGAGCATGATCAGGTATATTAGGACTTAGTATAAGAATATT  60
            ||||||||||||||||||||||||||||||||||||||||||||||||||||||||||||
Sbjct  679  AACAATATATTTAATTTTTGGAGCATGATCAGGTATATTAGGACTTAGTATAAGAATATT  620

Query  61   AATTCGTATAGAATTAGGACAACCTGGAACTTTAATTGGAAATGATCAGATTTATAATGT  120
            ||||||||||||||||||||||||||||||||||||||||||||||||||||||||||||
Sbjct  619  AATTCGTATAGAATTAGGACAACCTGGAACTTTAATTGGAAATGATCAGATTTATAATGT  560

Query  121  AATCGTAACAGCACATGCATTTATTATAATTTTCTTTATAGTTATACCAATCATAATCGG  180
            ||||||||||||||||||||||||||||||||| ||||||||||||||||||||||||||
Sbjct  559  AATCGTAACAGCACATGCATTTATTATAATTTTTTTTATAGTTATACCAATCATAATCGG  500

Query  181  AGGGTTCGGAAATTGATTAGTTCCTATTATATTAGGTGCCCCAGATATAGCATTTCCACG  240
            ||| || |||||||||||||| ||||| ||||| ||||||||||||||||||||||||||
Sbjct  499  AGGATTTGGAAATTGATTAGTCCCTATCATATTGGGTGCCCCAGATATAGCATTTCCACG  440

Query  241  AATAAATAATATAAGATTTTGACTTTTACCCCCCTCATTATTTTTATTGATTAACTCTTC  300
            |||||||||||||||||||||||||||||| || ||| |||||||||| |||||||||||
Sbjct  439  AATAAATAATATAAGATTTTGACTTTTACCTCCTTCACTATTTTTATTAATTAACTCTTC  380

Query  301  ATTAATTGAATCCGGAGCTGGAACAGGATGAACAGTTTATCCCCCTCTATCTTCAAATTT  360
            |||||||||||| |||||||| ||||||||||||||||| || |||||||||||||||||
Sbjct  379  ATTAATTGAATCTGGAGCTGGTACAGGATGAACAGTTTACCCTCCTCTATCTTCAAATTT  320

Query  361  ATCACATTATGGTCCATCAGTAGATTTAGCTATTTTTTCTCTTCACCTTGCTGGTGCTTC  420
            |||||||||||| || ||||||||||||||||||||||||||||| ||||||||||||||
Sbjct  319  ATCACATTATGGCCCTTCAGTAGATTTAGCTATTTTTTCTCTTCATCTTGCTGGTGCTTC  260

Query  421  TTCAATTTTAGGTGCAATTAACTTCATCACAACTATTGTAAATATACGATCAATTGGAAT  480
            |||||||||||||||||||||||| || |||||||||||||| |||||||||||||||||
Sbjct  259  TTCAATTTTAGGTGCAATTAACTTTATTACAACTATTGTAAACATACGATCAATTGGAAT  200

Query  481  AACAATAGAACGAATACCATTATTTGTTTGATCTGTTTTAATTACAGCTATTTTACTACT  540
            |||||||||||| |||||||||||||||||||||||||||||||||||||||||| ||||
Sbjct  199  AACAATAGAACGTATACCATTATTTGTTTGATCTGTTTTAATTACAGCTATTTTATTACT  140

Query  541  TCTATCTTTACCTGTATTAGCAGGTGCCATTACAATATTATTAACTGATCGAAACTTTAA  600
            | ||||||||||||||||||||||||||||||||||||| ||||||||||||||||||||
Sbjct  139  TTTATCTTTACCTGTATTAGCAGGTGCCATTACAATATTGTTAACTGATCGAAACTTTAA  80

Query  601  CACATCATTTTTTGATCCTTCAGGAGGAGGAGATCCCATTTTATATCAACACTTATTT  658
             ||||||||||||||||||||||| ||||||||||| |||||||||||||| ||||||
Sbjct  79   TACATCATTTTTTGATCCTTCAGGGGGAGGAGATCCAATTTTATATCAACATTTATTT  22
```

>Rhipicephalus sanguineus cytochrome c oxidase I (COI) gene, partial cds; mitochondrial gene for mitochondrial product
Sequence ID: AF132839.1 Length: 793
Range 1: 42 to 699

Score:1061 bits(574), Expect:0.0,
Identities:630/658(96%), Gaps:0/658(0%), Strand: Plus/Plus

```
Query  1    AACAATATATTTAATTTTTGGAGCATGATCAGGTATATTAGGACTTAGTATAAGAATATT  60
            ||||||||||||||||||||||||||||||||||||||||||||||||||||||||||||
Sbjct  42   AACAATATATTTAATTTTTGGAGCATGATCAGGTATATTAGGACTTAGTATAAGAATATT  101

Query  61   AATTCGTATAGAATTAGGACAACCTGGAACTTTAATTGGAAATGATCAGATTTATAATGT  120
            ||||||||||||||||||||||||||||||||||||||||||||||||||||||||||||
Sbjct  102  AATTCGTATAGAATTAGGACAACCTGGAACTTTAATTGGAAATGATCAGATTTATAATGT  161

Query  121  AATCGTAACAGCACATGCATTTATTATAATTTTCTTTATAGTTATACCAATCATAATCGG  180
            ||||||||||||||||||||||||||||||||| ||||||||||||||||||||||||||
Sbjct  162  AATCGTAACAGCACATGCATTTATTATAATTTTTTTTATAGTTATACCAATCATAATCGG  221

Query  181  AGGGTTCGGAAATTGATTAGTTCCTATTATATTAGGTGCCCCAGATATAGCATTTCCACG  240
            ||| || |||||||||||||| ||||| ||||| ||||||||||||||||||||||||||
Sbjct  222  AGGATTTGGAAATTGATTAGTCCCTATCATATTGGGTGCCCCAGATATAGCATTTCCACG  281

Query  241  AATAAATAATATAAGATTTTGACTTTTACCCCCCTCATTATTTTTATTGATTAACTCTTC  300
            |||||||||||||||||||||||||||||| || ||| |||||||||| |||||||||||
Sbjct  282  AATAAATAATATAAGATTTTGACTTTTACCTCCTTCACTATTTTTATTAATTAACTCTTC  341

Query  301  ATTAATTGAATCCGGAGCTGGAACAGGATGAACAGTTTATCCCCCTCTATCTTCAAATTT  360
            |||||||||||| |||||||| ||||||||||||||||| || |||||||||||||||||
Sbjct  342  ATTAATTGAATCTGGAGCTGGTACAGGATGAACAGTTTACCCTCCTCTATCTTCAAATTT  401

Query  361  ATCACATTATGGTCCATCAGTAGATTTAGCTATTTTTTCTCTTCACCTTGCTGGTGCTTC  420
            |||||||||||| || ||||||||||||||||||||||||||||| ||||||||||||||
Sbjct  402  ATCACATTATGGCCCTTCAGTAGATTTAGCTATTTTTTCTCTTCATCTTGCTGGTGCTTC  461

Query  421  TTCAATTTAGGTGCAATTAACTTCATCACAACTATTGTAAATATACGATCAATTGGAAT  480
            ||||||||||||||||||||||| || |||||||||||||| ||||||||||||||||||
Sbjct  462  TTCAATTTAGGTGCAATTAACTTTATTACAACTATTGTAAACATACGATCAATTGGAAT  521

Query  481  AACAATAGAACGAATACCATTATTTGTTTGATCTGTTTTAATTACAGCTATTTTACTACT  540
            |||||||||||| |||||||||||||||||||||||||||||||||||||||||| ||||
Sbjct  522  AACAATAGAACGTATACCATTATTTGTTTGATCTGTTTTAATTACAGCTATTTTATTACT  581

Query  541  TCTATCTTTACCTGTATTAGCAGGTGCCATTACAATATTATTAACTGATCGAAACTTTAA  600
            | ||||||||||||||||||||||||||||||||||||| ||||||||||||||||||||
Sbjct  582  TTTATCTTTACCTGTATTAGCAGGTGCCATTACAATATTGTTAACTGATCGAAACTTTAA  641

Query  601  CACATCATTTTTTGATCCTTCAGGAGGAGGAGATCCCATTTTATATCAACACTTATTT  658
             ||||||||||||||||||||||| ||||||||||| |||||||||||||| ||||||
Sbjct  642  TACATCATTTTTTGATCCTTCAGGGGGAGGAGATCCAATTTTATATCAACATTTATTT  699
```

>Rhipicephalus sanguineus sensu lato 'temperate lineage' isolate S56 cytochrome c oxidase subunit I (COI) gene, partial cds; mitochondrial
Sequence ID: MF426017.1 Length: 680
Range 1: 11 to 668

Score:1055 bits(571), Expect:0.0,
Identities:629/658(96%), Gaps:0/658(0%), Strand: Plus/Plus

```
Query  1    AACAATATATTTAATTTTTGGAGCATGATCAGGTATATTAGGACTTAGTATAAGAATATT  60
            ||||||||||||||||||||||||||||||||||||||||||||||||||||||||||||
Sbjct  11   AACAATATATTTAATTTTTGGAGCATGATCAGGTATATTAGGACTTAGTATAAGAATATT  70

Query  61   AATTCGTATAGAATTAGGACAACCTGGAACTTTAATTGGAAATGATCAGATTTATAATGT  120
            ||||||||||||||||||||||||||||||||||||||||||||||||||||||||||||
Sbjct  71   AATTCGTATAGAATTAGGACAACCTGGAACTTTAATTGGAAATGATCAGATTTATAATGT  130

Query  121  AATCGTAACAGCACATGCATTTATTATAATTTTCTTTATAGTTATACCAATCATAATCGG  180
            ||||||||||||||||||||||||||||||||| ||||||||||||||||||||||||||
Sbjct  131  AATCGTAACAGCACATGCATTTATTATAATTTTTTTTATAGTTATACCAATCATAATCGG  190

Query  181  AGGGTTCGGAAATTGATTAGTTCCTATTATATTAGGTGCCCCAGATATAGCATTTCCACG  240
            ||| || |||||||||||||| ||||| ||||| ||||||||||||||||||||||||||
Sbjct  191  AGGATTTGGAAATTGATTAGTCCCTATCATATTGGGTGCCCCAGATATAGCATTTCCACG  250

Query  241  AATAAATAATATAAGATTTTGACTTTTACCCCCCTCATTATTTTTATTGATTAACTCTTC  300
            |||||||||||||||||||||||||||||| || ||| |||||||||| |||||||||||
Sbjct  251  AATAAATAATATAAGATTTTGACTTTTACCTCCTTCACTATTTTTATTAATTAACTCTTC  310

Query  301  ATTAATTGAATCCGGAGCTGGAACAGGATGAACAGTTTATCCCCCTCTATCTTCAAATTT  360
            |||||||||||| |||||||| |||||||||||||||||||| |||||||||||||||||
Sbjct  311  ATTAATTGAATCTGGAGCTGGTACAGGATGAACAGTTTATCCTCCTCTATCTTCAAATTT  370

Query  361  ATCACATTATGGTCCATCAGTAGATTTAGCTATTTTTTCTCTTCACCTTGCTGGTGCTTC  420
            |||||||||||| || ||||||||||||||||| ||||||||||| ||||||||||||||
Sbjct  371  ATCACATTATGGCCCTTCAGTAGATTTAGCTATCTTTTCTCTTCATCTTGCTGGTGCTTC  430

Query  421  TTCAATTTTAGGTGCAATTAACTTCATCACAACTATTGTAAATATACGATCAATTGGAAT  480
            |||||||||||| ||||||||||| || |||||||||||||| |||||||||||||||||
Sbjct  431  TTCAATTTTAGGAGCAATTAACTTTATTACAACTATTGTAAACATACGATCAATTGGAAT  490

Query  481  AACAATAGAACGAATACCATTATTTGTTTGATCTGTTTTAATTACAGCTATTTTACTACT  540
            |||||||||||| ||||||||||||||||||||||||||||||||||||||||||| ||||
Sbjct  491  AACAATAGAACGTATACCATTATTTGTTTGATCTGTTTTAATTACAGCTATTTTATTACT  550

Query  541  TCTATCTTTACCTGTATTAGCAGGTGCCATTACAATATTATTAACTGATCGAAACTTTAA  600
            | ||||||||||||||||||||||||||||||||||||| ||||||||||||||||||||
Sbjct  551  TTTATCTTTACCTGTATTAGCAGGTGCCATTACAATATTGTTAACTGATCGAAACTTTAA  610

Query  601  CACATCATTTTTTGATCCTTCAGGAGGAGGAGATCCCATTTTATATCAACACTTATTT  658
             ||||||||||||||||||||||| ||||||||||| |||||||||||||| ||||||
Sbjct  611  TACATCATTTTTTGATCCTTCAGGGGGAGGAGATCCAATTTTATATCAACATTTATTT  668
```

>Rhipicephalus sanguineus sensu lato 'temperate lineage' isolate S32 cytochrome c oxidase subunit I (COI) gene, partial cds; mitochondrial
Sequence ID: MF426008.1 Length: 690
Range 1: 14 to 671

Score:1055 bits(571), Expect:0.0,
Identities:629/658(96%), Gaps:0/658(0%), Strand: Plus/Plus

```
Query  1    AACAATATATTTAATTTTTGGAGCATGATCAGGTATATTAGGACTTAGTATAAGAATATT  60
            ||||||||||||||||||||||||||||||||||||||||||||||||||||||||||||
Sbjct  14   AACAATATATTTAATTTTTGGAGCATGATCAGGTATATTAGGACTTAGTATAAGAATATT  73

Query  61   AATTCGTATAGAATTAGGACAACCTGGAACTTTAATTGGAAATGATCAGATTTATAATGT  120
            ||||||||||||||||||||||||||||||||||||||||||||||||||||||||||||
Sbjct  74   AATTCGTATAGAATTAGGACAACCTGGAACTTTAATTGGAAATGATCAGATTTATAATGT  133

Query  121  AATCGTAACAGCACATGCATTTATTATAATTTTCTTTATAGTTATACCAATCATAATCGG  180
            ||||||||||||||||||||||||||||||||| ||||||||||||||||||||||||||
Sbjct  134  AATCGTAACAGCACATGCATTTATTATAATTTTTTTTATAGTTATACCAATCATAATCGG  193

Query  181  AGGGTTCGGAAATTGATTAGTTCCTATTATATTAGGTGCCCCAGATATAGCATTTCCACG  240
            ||| || |||||||||||||| ||||| ||||| ||||||||||||||||||||||||||
Sbjct  194  AGGATTTGGAAATTGATTAGTCCCTATCATATTGGGTGCCCCAGATATAGCATTTCCACG  253

Query  241  AATAAATAATATAAGATTTTGACTTTTACCCCCCTCATTATTTTTATTGATTAACTCTTC  300
            |||||||||||||||||||||||||||||| || ||| |||||||||| |||||||||||
Sbjct  254  AATAAATAATATAAGATTTTGACTTTTACCTCCTTCACTATTTTTATTAATTAACTCTTC  313

Query  301  ATTAATTGAATCCGGAGCTGGAACAGGATGAACAGTTTATCCCCCTCTATCTTCAAATTT  360
            |||||||||||| |||||||| ||||||||||||||||| || |||||||||||||||||
Sbjct  314  ATTAATTGAATCTGGAGCTGGTACAGGATGAACAGTTTACCCTCCTCTATCTTCAAATTT  373

Query  361  ATCACATTATGGTCCATCAGTAGATTTAGCTATTTTTTCTCTTCACCTTGCTGGTGCTTC  420
            |||||||||||| || ||||||||||||||||| ||||||||||| ||||||||||||||
Sbjct  374  ATCACATTATGGCCCTTCAGTAGATTTAGCTATCTTTTCTCTTCATCTTGCTGGTGCTTC  433

Query  421  TTCAATTTTAGGTGCAATTAACTTCATCACAACTATTGTAAATATACGATCAATTGGAAT  480
            |||||||||||||||||||||||| || |||||||||||||| |||||||||||||||||
Sbjct  434  TTCAATTTTAGGTGCAATTAACTTTATTACAACTATTGTAAACATACGATCAATTGGAAT  493

Query  481  AACAATAGAACGAATACCATTATTTGTTTGATCTGTTTTAATTACAGCTATTTTACTACT  540
            |||||||||||| |||||||||||||||||||||||||||||||||||||||||| ||||
Sbjct  494  AACAATAGAACGTATACCATTATTTGTTTGATCTGTTTTAATTACAGCTATTTTATTACT  553

Query  541  TCTATCTTTACCTGTATTAGCAGGTGCCATTACAATATTATTAACTGATCGAAACTTTAA  600
            | ||||||||||||||||||||||||||||||||||||| ||||||||||||||||||||
Sbjct  554  TTTATCTTTACCTGTATTAGCAGGTGCCATTACAATATTGTTAACTGATCGAAACTTTAA  613

Query  601  CACATCATTTTTTGATCCTTCAGGAGGAGGAGATCCCATTTATATCAACACTTATTT  658
             ||||||||||||||||||||||| ||||||||||| ||||||||||||| ||||||
Sbjct  614  TACATCATTTTTTGATCCTTCAGGGGGAGGAGATCCAATTTATATCAACATTTATTT  671
```

```
>Rhipicephalus sanguineus sensu lato 'temperate lineage' isolate CR1570
cytochrome c oxidase subunit I (COI) gene, partial cds; mitochondrial
Sequence ID: MF426001.1 Length: 684
Range 1: 14 to 671

Score:1055 bits(571), Expect:0.0,
Identities:629/658(96%),  Gaps:0/658(0%), Strand: Plus/Plus

Query  1    AACAATATATTTAATTTTTGGAGCATGATCAGGTATATTAGGACTTAGTATAAGAATATT  60
            ||||||||||||||||||||||||||||||||||||||||||||||||||||||||||||
Sbjct  14   AACAATATATTTAATTTTTGGAGCATGATCAGGTATATTAGGACTTAGTATAAGAATATT  73

Query  61   AATTCGTATAGAATTAGGACAACCTGGAACTTTAATTGGAAATGATCAGATTTATAATGT  120
            ||||||||||||||||||||||||||||||||||||||||||||||||||||||||||||
Sbjct  74   AATTCGTATAGAATTAGGACAACCTGGAACTTTAATTGGAAATGATCAGATTTATAATGT  133

Query  121  AATCGTAACAGCACATGCATTTATTATAATTTTCTTTATAGTTATACCAATCATAATCGG  180
            ||||||||||||||||||||||||||||||||| ||||||||||||||||||||||||||
Sbjct  134  AATCGTAACAGCACATGCATTTATTATAATTTTTTTTATAGTTATACCAATCATAATCGG  193

Query  181  AGGGTTCGGAAATTGATTAGTTCCTATTATATTAGGTGCCCCAGATATAGCATTTCCACG  240
            ||| || |||||||||||||| ||||| ||||| ||||||||||||||||||||||||||
Sbjct  194  AGGATTTGGAAATTGATTAGTCCCTATCATATTGGGTGCCCCAGATATAGCATTTCCACG  253

Query  241  AATAAATAATATAAGATTTTGACTTTTACCCCCCTCATTATTTTTATTGATTAACTCTTC  300
            |||||||||||||||||||||||||||||| || ||| |||||||||| |||||||||||
Sbjct  254  AATAAATAATATAAGATTTTGACTTTTACCTCCTTCACTATTTTTATTAATTAACTCTTC  313

Query  301  ATTAATTGAATCCGGAGCTGGAACAGGATGAACAGTTTATCCCCCTCTATCTTCAAATTT  360
            |||||||||||| |||||||| ||||||||||||||||| || |||||||||||||||||
Sbjct  314  ATTAATTGAATCTGGAGCTGGTACAGGATGAACAGTTTACCCTCCTCTATCTTCAAATTT  373

Query  361  ATCACATTATGGTCCATCAGTAGATTTAGCTATTTTTTCTCTTCACCTTGCTGGTGCTTC  420
            |||||||||||| || ||||||||||||||||| ||||||||||| ||||||||||||||
Sbjct  374  ATCACATTATGGCCCTTCAGTAGATTTAGCTATCTTTTCTCTTCATCTTGCTGGTGCTTC  433

Query  421  TTCAATTTTAGGTGCAATTAACTTCATCACAACTATTGTAAATATACGATCAATTGGAAT  480
            |||||||||||||||||||||||| || |||||||||||||| |||||||||||||||||
Sbjct  434  TTCAATTTTAGGTGCAATTAACTTTATTACAACTATTGTAAACATACGATCAATTGGAAT  493

Query  481  AACAATAGAACGAATACCATTATTTGTTTGATCTGTTTTAATTACAGCTATTTTACTACT  540
            |||||||||||| ||||||||||||||||||||||||||||||||||||||||||| ||||
Sbjct  494  AACAATAGAACGTATACCATTATTTGTTTGATCTGTTTTAATTACAGCTATTTTATTACT  553

Query  541  TCTATCTTTACCTGTATTAGCAGGTGCCATTACAATATTATTAACTGATCGAAACTTTAA  600
            | ||||||||||||||||||||||||||||||||||||| ||||||||||||||||||||
Sbjct  554  TTTATCTTTACCTGTATTAGCAGGTGCCATTACAATATTGTTAACTGATCGAAACTTTAA  613

Query  601  CACATCATTTTTTGATCCTTCAGGAGGAGGAGATCCCATTTTATATCAACACTTATTT  658
             ||||||||||||||||||||||| ||||||||||| |||||||||||||| ||||||
```

```
Sbjct  614  TACATCATTTTTTGATCCTTCAGGGGGAGGAGATCCAATTTTATATCAACATTTATTT  671
```

>Rhipicephalus sanguineus sensu lato 'temperate lineage' isolate CR1553 cytochrome c oxidase subunit I (COI) gene, partial cds; mitochondrial
Sequence ID: MF425998.1 Length: 689
Range 1: 20 to 677

Score:1055 bits(571), Expect:0.0,
Identities:629/658(96%), Gaps:0/658(0%), Strand: Plus/Plus

```
Query  1    AACAATATATTTAATTTTTGGAGCATGATCAGGTATATTAGGACTTAGTATAAGAATATT  60
            ||||||||||||||||||||||||||||||||||||||||||||||||||||||||||||
Sbjct  20   AACAATATATTTAATTTTTGGAGCATGATCAGGTATATTAGGACTTAGTATAAGAATATT  79

Query  61   AATTCGTATAGAATTAGGACAACCTGGAACTTTAATTGGAAATGATCAGATTTATAATGT  120
            ||||||||||||||||||||||||||||||||||||||||||||||||||||||||||||
Sbjct  80   AATTCGTATAGAATTAGGACAACCTGGAACTTTAATTGGAAATGATCAGATTTATAATGT  139

Query  121  AATCGTAACAGCACATGCATTTATTATAATTTCTTTATAGTTATACCAATCATAATCGG  180
            |||||||||||||||||||||||||||||||| |||||||||||||||||||||||||||
Sbjct  140  AATCGTAACAGCACATGCATTTATTATAATTTTTTTATAGTTATACCAATCATAATCGG  199

Query  181  AGGGTTCGGAAATTGATTAGTTCCTATTATATTAGGTGCCCCAGATATAGCATTTCCACG  240
            ||| || |||||||||||||| ||||| ||||||||||||||||||||||||||||||||
Sbjct  200  AGGATTTGGAAATTGATTAGTCCCTATCATATTAGGTGCCCCAGATATAGCATTTCCACG  259

Query  241  AATAAATAATATAAGATTTTGACTTTTACCCCCCTCATTATTTTTATTGATTAACTCTTC  300
            |||||||||||||||||||||||||||||| || ||| |||||||||| |||||||||||
Sbjct  260  AATAAATAATATAAGATTTTGACTTTTACCTCCTTCACTATTTTTATTAATTAACTCTTC  319

Query  301  ATTAATTGAATCCGGAGCTGGAACAGGATGAACAGTTTATCCCCCTCTATCTTCAAATTT  360
            |||||||||||| |||||||| ||||||||||||||||| || |||||||||||||||||
Sbjct  320  ATTAATTGAATCTGGAGCTGGTACAGGATGAACAGTTTACCCTCCTCTATCTTCAAATTT  379

Query  361  ATCACATTATGGTCCATCAGTAGATTTAGCTATTTTTTCTCTTCACCTTGCTGGTGCTTC  420
            |||||||||||| || ||||||||||||||||| ||||||||||| ||||||||||||||
Sbjct  380  ATCACATTATGGCCCTTCAGTAGATTTAGCTATCTTTTCTCTTCATCTTGCTGGTGCTTC  439

Query  421  TTCAATTTTAGGTGCAATTAACTTCATCACAACTATTGTAAATATACGATCAATTGGAAT  480
            |||||||||||||||||||||||| || |||||||||||||| |||||||||||||||||
Sbjct  440  TTCAATTTTAGGTGCAATTAACTTTATTACAACTATTGTAAACATACGATCAATTGGAAT  499

Query  481  AACAATAGAACGAATACCATTATTTGTTTGATCTGTTTTAATTACAGCTATTTTACTACT  540
            |||||||||||| |||||||||||||||||||||||||||||||||||||||||| ||||
Sbjct  500  AACAATAGAACGTATACCATTATTTGTTTGATCTGTTTTAATTACAGCTATTTTATTACT  559

Query  541  TCTATCTTTACCTGTATTAGCAGGTGCCATTACAATATTATTAACTGATCGAAACTTTAA  600
              ||||||||||||||||||||||||||||||||||||| ||||||||||||||||||||
Sbjct  560  CTTATCTTTACCTGTATTAGCAGGTGCCATTACAATATTGTTAACTGATCGAAACTTTAA  619

Query  601  CACATCATTTTTTGATCCTTCAGGAGGAGGAGATCCCATTTTATATCAACACTTATTT  658
```

```
                ||||||||||||||||||||||| ||||||||||| |||||||||||||| ||||||
Sbjct  620  TACATCATTTTTTGATCCTTCAGGGGGAGGAGATCCAATTTTATATCAACATTTATTT  677
```

>Rhipicephalus sanguineus sensu lato 'temperate lineage' isolate CR1551 cytochrome c oxidase subunit I (COI) gene, partial cds; mitochondrial
Sequence ID: MF425997.1 Length: 695
Range 1: 22 to 679

Score:1055 bits(571), Expect:0.0,
Identities:629/658(96%), Gaps:0/658(0%), Strand: Plus/Plus

```
Query  1    AACAATATATTTAATTTTTGGAGCATGATCAGGTATATTAGGACTTAGTATAAGAATATT  60
            ||||||||||||||||||||||||||||||||||||||||||||||||||||||||||||
Sbjct  22   AACAATATATTTAATTTTTGGAGCATGATCAGGTATATTAGGACTTAGTATAAGAATATT  81

Query  61   AATTCGTATAGAATTAGGACAACCTGGAACTTTAATTGGAAATGATCAGATTTATAATGT  120
            ||||||||||||||||||||||||||||||||||||||||||||||||||||||||||||
Sbjct  82   AATTCGTATAGAATTAGGACAACCTGGAACTTTAATTGGAAATGATCAGATTTATAATGT  141

Query  121  AATCGTAACAGCACATGCATTTATTATAATTTTCTTTATAGTTATACCAATCATAATCGG  180
            ||||||||||||||||||||||||||||||||| ||||||||||||||||||||||||||
Sbjct  142  AATCGTAACAGCACATGCATTTATTATAATTTTTTTTATAGTTATACCAATCATAATCGG  201

Query  181  AGGGTTCGGAAATTGATTAGTTCCTATTATATTAGGTGCCCCAGATATAGCATTTCCACG  240
            ||| || |||||||||||||| ||||| ||||| ||||||||||||||||||||||||||
Sbjct  202  AGGATTTGGAAATTGATTAGTCCCTATCATATTGGGTGCCCCAGATATAGCATTTCCACG  261

Query  241  AATAAATAATATAAGATTTTGACTTTTACCCCCCTCATTATTTTTATTGATTAACTCTTC  300
            |||||||||||||||||||||||||||||| || ||| |||||||||| |||||||||||
Sbjct  262  AATAAATAATATAAGATTTTGACTTTTACCTCCTTCACTATTTTTATTAATTAACTCTTC  321

Query  301  ATTAATTGAATCCGGAGCTGGAACAGGATGAACAGTTTATCCCCCTCTATCTTCAAATTT  360
            |||||||||||| |||||||| |||||||||||||||| || ||||||||||||||||||
Sbjct  322  ATTAATTGAATCTGGAGCTGGTACAGGATGAACAGTTTACCCTCCTCTATCTTCAAATTT  381

Query  361  ATCACATTATGGTCCATCAGTAGATTTAGCTATTTTTTCTCTTCACCTTGCTGGTGCTTC  420
            |||||||||||| || ||||||||||||||||| ||||||||||| ||||||||||||||
Sbjct  382  ATCACATTATGGCCCTTCAGTAGATTTAGCTATCTTTTCTCTTCATCTTGCTGGTGCTTC  441

Query  421  TTCAATTTTAGGTGCAATTAACTTCATCACAACTATTGTAAATATACGATCAATTGGAAT  480
            |||||||||||||||||||||||| || |||||||||||||| |||||||||||||||||
Sbjct  442  TTCAATTTTAGGTGCAATTAACTTTATTACAACTATTGTAAACATACGATCAATTGGAAT  501

Query  481  AACAATAGAACGAATACCATTATTTGTTTGATCTGTTTTAATTACAGCTATTTTACTACT  540
            |||||||||||| ||||||||||||||||||||||||||||||||||||||||||| ||||
Sbjct  502  AACAATAGAACGTATACCATTATTTGTTTGATCTGTTTTAATTACAGCTATTTTATTACT  561

Query  541  TCTATCTTTACCTGTATTAGCAGGTGCCATTACAATATTATTAACTGATCGAAACTTTAA  600
            | |||||||||||||||||||||||||||||||||||| |||||||||||||||||||||
Sbjct  562  TTTATCTTTACCTGTATTAGCAGGTGCCATTACAATATTGTTAACTGATCGAAACTTTAA  621
```

```
Query  601  CACATCATTTTTTGATCCTTCAGGAGGAGGAGATCCCATTTTATATCAACACTTATTT  658
             ||||||||||||||||||||||| ||||||||||| |||||||||||||| ||||||
Sbjct  622  TACATCATTTTTTGATCCTTCAGGGGGAGGAGATCCAATTTTATATCAACATTTATTT  679
```

>Rhipicephalus sanguineus haplotype IV cytochrome oxidase subunit I (COI) gene, partial cds; mitochondrial
Sequence ID: KU556746.1 Length: 771
Range 1: 90 to 747

Score:1055 bits(571), Expect:0.0,
Identities:629/658(96%), Gaps:0/658(0%), Strand: Plus/Plus

```
Query  1    AACAATATATTTAATTTTTGGAGCATGATCAGGTATATTAGGACTTAGTATAAGAATATT  60
            ||||||||||||||||||||||||||||||||||||||||||||||||||||||||||||
Sbjct  90   AACAATATATTTAATTTTTGGAGCATGATCAGGTATATTAGGACTTAGTATAAGAATATT  149

Query  61   AATTCGTATAGAATTAGGACAACCTGGAACTTTAATTGGAAATGATCAGATTTATAATGT  120
            ||||||||||||||||||||||||||||||||||||||||||||||||||||||||||||
Sbjct  150  AATTCGTATAGAATTAGGACAACCTGGAACTTTAATTGGAAATGATCAGATTTATAATGT  209

Query  121  AATCGTAACAGCACATGCATTTATTATAATTTTCTTTATAGTTATACCAATCATAATCGG  180
            ||||||||||||||||||||||||||||||||| ||||||||||||||||||||||||||
Sbjct  210  AATCGTAACAGCACATGCATTTATTATAATTTTTTTTATAGTTATACCAATCATAATCGG  269

Query  181  AGGGTTCGGAAATTGATTAGTTCCTATTATATTAGGTGCCCCAGATATAGCATTTCCACG  240
            ||| || |||||||||||||| ||||| ||||| ||||||||||||||||||||||||||
Sbjct  270  AGGATTTGGAAATTGATTAGTCCCTATCATATTGGGTGCCCCAGATATAGCATTTCCACG  329

Query  241  AATAAATAATATAAGATTTTGACTTTTACCCCCCTCATTATTTTTATTGATTAACTCTTC  300
            |||||||||||||||||||||||||||||| || ||| |||||||||| |||||||||||
Sbjct  330  AATAAATAATATAAGATTTTGACTTTTACCTCCTTCACTATTTTTATTAATTAACTCTTC  389

Query  301  ATTAATTGAATCCGGAGCTGGAACAGGATGAACAGTTTATCCCCCTCTATCTTCAAATTT  360
            |||||||||||| |||||||| |||||||||||||||||||| |||||||||||||||||
Sbjct  390  ATTAATTGAATCTGGAGCTGGTACAGGATGAACAGTTTATCCTCCTCTATCTTCAAATTT  449

Query  361  ATCACATTATGGTCCATCAGTAGATTTAGCTATTTTTTCTCTTCACCTTGCTGGTGCTTC  420
            |||||||||||| || ||||||||||||||||| ||||||||||| ||||||||||||||
Sbjct  450  ATCACATTATGGCCCTTCAGTAGATTTAGCTATCTTTTCTCTTCATCTTGCTGGTGCTTC  509

Query  421  TTCAATTTTAGGTGCAATTAACTTCATCACAACTATTGTAAATATACGATCAATTGGAAT  480
            |||||||||||| ||||||||||| || |||||||||||||| |||||||||||||||||
Sbjct  510  TTCAATTTTAGGAGCAATTAACTTTATTACAACTATTGTAAACATACGATCAATTGGAAT  569

Query  481  AACAATAGAACGAATACCATTATTTGTTTGATCTGTTTTAATTACAGCTATTTTACTACT  540
            |||||||||||| |||||||||||||||||||||||||||||||||||||||||| ||||
Sbjct  570  AACAATAGAACGTATACCATTATTTGTTTGATCTGTTTTAATTACAGCTATTTTATTACT  629

Query  541  TCTATCTTTACCTGTATTAGCAGGTGCCATTACAATATTATTAACTGATCGAAACTTTAA  600
            | ||||||||||||||||||||||||||||||||||||| ||||||||||||||||||||
Sbjct  630  TTTATCTTTACCTGTATTAGCAGGTGCCATTACAATATTGTTAACTGATCGAAACTTTAA  689
```

```
Query  601  CACATCATTTTTTGATCCTTCAGGAGGAGGAGATCCCATTTTATATCAACACTTATTT  658
             ||||||||||||||||||||||| ||||||||||| |||||||||||||| ||||||
Sbjct  690  TACATCATTTTTTGATCCTTCAGGGGGAGGAGATCCAATTTTATATCAACATTTATTT  747
```

Range 2: 30 to 64

Score:58.4 bits(31), Expect:0.001,
Identities:34/35(97%), Gaps:1/35(2%), Strand: Plus/Plus

```
Query  1   AACAATATATTTAATTTTTGGAGCATGATCA-GGT  34
           ||||||||||||||||||||||||||||||| |||
Sbjct  30  AACAATATATTTAATTTTTGGAGCATGATCATGGT  64
```

>Rhipicephalus sanguineus voucher CROBB813 cytochrome oxidase subunit 1 (COI) gene, partial cds; mitochondrial
Sequence ID: MZ305546.1 Length: 658
Range 1: 1 to 658

Score:1055 bits(571), Expect:0.0,
Identities:629/658(96%), Gaps:0/658(0%), Strand: Plus/Plus

```
Query  1    AACAATATATTTAATTTTTGGAGCATGATCAGGTATATTAGGACTTAGTATAAGAATATT  60
            ||||||||||||||||||||||||||||||||||||||||||||||||||||||||||||
Sbjct  1    AACAATATATTTAATTTTTGGAGCATGATCAGGTATATTAGGACTTAGTATAAGAATATT  60

Query  61   AATTCGTATAGAATTAGGACAACCTGGAACTTTAATTGGAAATGATCAGATTTATAATGT  120
            |||||||||||| |||||||||||||||||||||||||||||||||||||||||||||||
Sbjct  61   AATTCGTATAGAGTTAGGACAACCTGGAACTTTAATTGGAAATGATCAGATTTATAATGT  120

Query  121  AATCGTAACAGCACATGCATTTATTATAATTTTCTTTATAGTTATACCAATCATAATCGG  180
            ||||||||||||||||||||||||||||||||| ||||||||||||||||||||||||||
Sbjct  121  AATCGTAACAGCACATGCATTTATTATAATTTTTTTTATAGTTATACCAATCATAATCGG  180

Query  181  AGGGTTCGGAAATTGATTAGTTCCTATTATATTAGGTGCCCCAGATATAGCATTTCCACG  240
            ||| || |||||||||||||| ||||| ||||| ||||||||||||||||||||||||||
Sbjct  181  AGGATTTGGAAATTGATTAGTCCCTATCATATTGGGTGCCCCAGATATAGCATTTCCACG  240

Query  241  AATAAATAATATAAGATTTTGACTTTTACCCCCCTCATTATTTTTATTGATTAACTCTTC  300
            |||||||||||||||||||||||||||||| || ||| |||||||||| |||||||||||
Sbjct  241  AATAAATAATATAAGATTTTGACTTTTACCTCCTTCACTATTTTTATTAATTAACTCTTC  300

Query  301  ATTAATTGAATCCGGAGCTGGAACAGGATGAACAGTTTATCCCCCTCTATCTTCAAATTT  360
            |||||||||||| |||||||| ||||||||||||||||| || |||||||||||||||||
Sbjct  301  ATTAATTGAATCTGGAGCTGGTACAGGATGAACAGTTTACCCTCCTCTATCTTCAAATTT  360

Query  361  ATCACATTATGGTCCATCAGTAGATTTAGCTATTTTTCTCTTCACCTTGCTGGTGCTTC  420
            |||||||||||| || ||||||||||||||||||||||||||||| ||||||||||||||
```

```
Sbjct  361  ATCACATTATGGCCCTTCAGTAGATTTAGCTATTTTTTCTCTTCATCTTGCTGGTGCTTC  420

Query  421  TTCAATTTTAGGTGCAATTAACTTCATCACAACTATTGTAAATATACGATCAATTGGAAT  480
            |||||||||||||||||||||||| || |||||||||||||| |||||||||||||||||
Sbjct  421  TTCAATTTTAGGTGCAATTAACTTTATTACAACTATTGTAAACATACGATCAATTGGAAT  480

Query  481  AACAATAGAACGAATACCATTATTTGTTTGATCTGTTTTAATTACAGCTATTTTACTACT  540
            |||||||||||| |||||||||||||||||||||||||||||||||||||||||| ||||
Sbjct  481  AACAATAGAACGTATACCATTATTTGTTTGATCTGTTTTAATTACAGCTATTTTATTACT  540

Query  541  TCTATCTTTACCTGTATTAGCAGGTGCCATTACAATATTATTAACTGATCGAAACTTTAA  600
            | ||||||||||||||||||||||||||||||||||||| ||||||||||||||||||||
Sbjct  541  TTTATCTTTACCTGTATTAGCAGGTGCCATTACAATATTGTTAACTGATCGAAACTTTAA  600

Query  601  CACATCATTTTTTGATCCTTCAGGAGGAGGAGATCCCATTTTATATCAACACTTATTT  658
             ||||||||||||||||||||||| ||||||||||| |||||||||||||| ||||||
Sbjct  601  TACATCATTTTTTGATCCTTCAGGGGGAGGAGATCCAATTTTATATCAACATTTATTT  658



>Rhipicephalus sanguineus voucher CROBB810 cytochrome oxidase subunit 1 (COI)
gene, partial cds; mitochondrial
Sequence ID: MZ305545.1 Length: 658
Range 1: 1 to 658

Score:1055 bits(571), Expect:0.0,
Identities:629/658(96%),  Gaps:0/658(0%), Strand: Plus/Plus

Query  1    AACAATATATTTAATTTTTGGAGCATGATCAGGTATATTAGGACTTAGTATAAGAATATT  60
            ||||||||||||||||||||||||||||||||||||||||||||||||||||||||||||
Sbjct  1    AACAATATATTTAATTTTTGGAGCATGATCAGGTATATTAGGACTTAGTATAAGAATATT  60

Query  61   AATTCGTATAGAATTAGGACAACCTGGAACTTTAATTGGAAATGATCAGATTTATAATGT  120
            ||||||||||||||||||||||||||||||||||||||||||||||||||||||||||||
Sbjct  61   AATTCGTATAGAATTAGGACAACCTGGAACTTTAATTGGAAATGATCAGATTTATAATGT  120

Query  121  AATCGTAACAGCACATGCATTTATTATAATTTTCTTTATAGTTATACCAATCATAATCGG  180
            ||||||||||||||||||||||||||||||||| ||||||||||||||||||||||||||
Sbjct  121  AATCGTAACAGCACATGCATTTATTATAATTTTTTTTATAGTTATACCAATCATAATCGG  180

Query  181  AGGGTTCGGAAATTGATTAGTTCCTATTATATTAGGTGCCCCAGATATAGCATTTCCACG  240
            ||| || |||||||||||||| ||||| ||||| ||||||||||||||||||||||||||
Sbjct  181  AGGATTTGGAAATTGATTAGTCCCTATCATATTGGGTGCCCCAGATATAGCATTTCCACG  240

Query  241  AATAAATAATATAAGATTTTGACTTTTACCCCCCTCATTATTTTTATTGATTAACTCTTC  300
            |||||||||||||||||||||||||||||| || ||| |||||||||| ||||| |||||
Sbjct  241  AATAAATAATATAAGATTTTGACTTTTACCTCCTTCACTATTTTTATTAATTAATTCTTC  300

Query  301  ATTAATTGAATCCGGAGCTGGAACAGGATGAACAGTTTATCCCCCTCTATCTTCAAATTT  360
            |||||||||||| |||||||| ||||||||||||||||| || |||||||||||||||||
Sbjct  301  ATTAATTGAATCTGGAGCTGGTACAGGATGAACAGTTTACCCTCCTCTATCTTCAAATTT  360

Query  361  ATCACATTATGGTCCATCAGTAGATTTAGCTATTTTTTCTCTTCACCTTGCTGGTGCTTC  420
```

```
                ||||||||||||| || ||||||||||||||||||||||||||||| ||||||||||||||
Sbjct  361   ATCACATTATGGCCCTTCAGTAGATTTAGCTATTTTTTCTCTTCATCTTGCTGGTGCTTC  420

Query  421   TTCAATTTTAGGTGCAATTAACTTCATCACAACTATTGTAAATATACGATCAATTGGAAT  480
             |||||||||||||||||||||||| || |||||||||||||| |||||||||||||||||
Sbjct  421   TTCAATTTTAGGTGCAATTAACTTTATTACAACTATTGTAAACATACGATCAATTGGAAT  480

Query  481   AACAATAGAACGAATACCATTATTTGTTTGATCTGTTTTAATTACAGCTATTTTACTACT  540
             |||||||||||| |||||||||||||||||||||||||||||||||||||||||| ||||
Sbjct  481   AACAATAGAACGTATACCATTATTTGTTTGATCTGTTTTAATTACAGCTATTTTATTACT  540

Query  541   TCTATCTTTACCTGTATTAGCAGGTGCCATTACAATATTATTAACTGATCGAAACTTTAA  600
             | ||||||||||||||||||||||||||||||||||||| ||||||||||||||||||||
Sbjct  541   TTTATCTTTACCTGTATTAGCAGGTGCCATTACAATATTGTTAACTGATCGAAACTTTAA  600

Query  601   CACATCATTTTTTGATCCTTCAGGAGGAGGAGATCCCATTTTATATCAACACTTATTT  658
              ||||||||||||||||||||||| ||||||||||| |||||||||||||| ||||||
Sbjct  601   TACATCATTTTTTGATCCTTCAGGGGGAGGAGATCCAATTTTATATCAACATTTATTT  658
```

>Rhipicephalus sanguineus isolate 5MSK cytochrome c oxidase subunit I (COX1) gene, partial cds; mitochondrial
Sequence ID: MW152145.1 Length: 676
Range 1: 1 to 658

Score:1055 bits(571), Expect:0.0,
Identities:629/658(96%), Gaps:0/658(0%), Strand: Plus/Plus

```
Query  1     AACAATATATTTAATTTTTGGAGCATGATCAGGTATATTAGGACTTAGTATAAGAATATT  60
             ||||||||||||||||||||||||||||||||||||||||||||||||||||||||||||
Sbjct  1     AACAATATATTTAATTTTTGGAGCATGATCAGGTATATTAGGACTTAGTATAAGAATATT  60

Query  61    AATTCGTATAGAATTAGGACAACCTGGAACTTTAATTGGAAATGATCAGATTTATAATGT  120
             ||||||||||||||||||||||||||||||||||||||||||||||||||||||||||||
Sbjct  61    AATTCGTATAGAATTAGGACAACCTGGAACTTTAATTGGAAATGATCAGATTTATAATGT  120

Query  121   AATCGTAACAGCACATGCATTTATTATAATTTTCTTTATAGTTATACCAATCATAATCGG  180
             ||||||||||||||||||||||||||||||||| ||||||||||||||||||||||||||
Sbjct  121   AATCGTAACAGCACATGCATTTATTATAATTTTTTTTATAGTTATACCAATCATAATCGG  180

Query  181   AGGGTTCGGAAATTGATTAGTTCCTATTATATTAGGTGCCCCAGATATAGCATTTCCACG  240
             ||| || |||||||||||||| ||||| ||||| ||||||||||||||||||||||||||
Sbjct  181   AGGATTTGGAAATTGATTAGTCCCTATCATATTGGGTGCCCCAGATATAGCATTTCCACG  240

Query  241   AATAAATAATATAAGATTTTGACTTTTACCCCCCTCATTATTTTTATTGATTAACTCTTC  300
             |||||||||||||||||||||||||||||| || ||| |||||||||| ||||| |||||
Sbjct  241   AATAAATAATATAAGATTTTGACTTTTACCTCCTTCACTATTTTTATTAATTAATTCTTC  300

Query  301   ATTAATTGAATCCGGAGCTGGAACAGGATGAACAGTTTATCCCCCTCTATCTTCAAATTT  360
             |||||||||||| |||||||| |||||||||||||||||| || ||||||||||||||||
Sbjct  301   ATTAATTGAATCTGGAGCTGGTACAGGATGAACAGTTTACCCTCCTCTATCTTCAAATTT  360
```

```
Query  361   ATCACATTATGGTCCATCAGTAGATTTAGCTATTTTTTCTCTTCACCTTGCTGGTGCTTC  420
             |||||||||||| || ||||||||||||||||||||||||||||| ||||||||||||||
Sbjct  361   ATCACATTATGGCCCTTCAGTAGATTTAGCTATTTTTTCTCTTCATCTTGCTGGTGCTTC  420

Query  421   TTCAATTTTAGGTGCAATTAACTTCATCACAACTATTGTAAATATACGATCAATTGGAAT  480
             |||||||||||||||||||||||| || ||||||||||||||| ||||||||||||||||
Sbjct  421   TTCAATTTTAGGTGCAATTAACTTTATTACAACTATTGTAAACATACGATCAATTGGAAT  480

Query  481   AACAATAGAACGAATACCATTATTTGTTTGATCTGTTTTAATTACAGCTATTTTACTACT  540
             |||||||||||| |||||||||||||||||||||||||||||||||||||||||| ||||
Sbjct  481   AACAATAGAACGTATACCATTATTTGTTTGATCTGTTTTAATTACAGCTATTTTATTACT  540

Query  541   TCTATCTTTACCTGTATTAGCAGGTGCCATTACAATATTATTAACTGATCGAAACTTTAA  600
             | ||||||||||||||||||||||||||||||||||||| ||||||||||||||||||||
Sbjct  541   TTTATCTTTACCTGTATTAGCAGGTGCCATTACAATATTGTTAACTGATCGAAACTTTAA  600

Query  601   CACATCATTTTTTGATCCTTCAGGAGGAGGAGATCCCATTTATATCAACACTTATTT  658
              |||||||||||||||||||||||| ||||||||||| ||||||||||||| ||||||
Sbjct  601   TACATCATTTTTTGATCCTTCAGGGGGAGGAGATCCAATTTATATCAACATTTATTT  658
```

>Rhipicephalus sanguineus isolate 1MSK cytochrome c oxidase subunit I (COX1) gene, partial cds; mitochondrial
Sequence ID: MW152144.1 Length: 675
Range 1: 1 to 658

Score:1055 bits(571), Expect:0.0,
Identities:629/658(96%), Gaps:0/658(0%), Strand: Plus/Plus

```
Query  1     AACAATATATTTAATTTTTGGAGCATGATCAGGTATATTAGGACTTAGTATAAGAATATT  60
             ||||||||||||||||||||||||||||||||||||||||||||||||||||||||||||
Sbjct  1     AACAATATATTTAATTTTTGGAGCATGATCAGGTATATTAGGACTTAGTATAAGAATATT  60

Query  61    AATTCGTATAGAATTAGGACAACCTGGAACTTTAATTGGAAATGATCAGATTTATAATGT  120
             ||||||||||||||||||||||||||||||||||||||||||||||||||||||||||||
Sbjct  61    AATTCGTATAGAATTAGGACAACCTGGAACTTTAATTGGAAATGATCAGATTTATAATGT  120

Query  121   AATCGTAACAGCACATGCATTTATTATAATTTTCTTTATAGTTATACCAATCATAATCGG  180
             ||||||||||||||||||||||||||||||||| ||||||||||||||||||||||||||
Sbjct  121   AATCGTAACAGCACATGCATTTATTATAATTTTTTTTATAGTTATACCAATCATAATCGG  180

Query  181   AGGGTTCGGAAATTGATTAGTTCCTATTATATTAGGTGCCCCAGATATAGCATTTCCACG  240
             ||| || |||||||||||||| ||||| ||||| ||||||||||||||||||||||||||
Sbjct  181   AGGATTTGGAAATTGATTAGTCCCTATCATATTGGGTGCCCCAGATATAGCATTTCCACG  240

Query  241   AATAAATAATATAAGATTTTGACTTTTACCCCCCTCATTATTTTTATTGATTAACTCTTC  300
             |||||||||||||||||||||||||||||| || ||| |||||||||| ||||| |||||
Sbjct  241   AATAAATAATATAAGATTTTGACTTTTACCTCCTTCACTATTTTTATTAATTAATTCTTC  300

Query  301   ATTAATTGAATCCGGAGCTGGAACAGGATGAACAGTTTATCCCCCTCTATCTTCAAATTT  360
             |||||||||||| |||||||| ||||||||||||||||| || |||||||||||||||||
Sbjct  301   ATTAATTGAATCTGGAGCTGGTACAGGATGAACAGTTTACCCTCCTCTATCTTCAAATTT  360
```

```
Query  361  ATCACATTATGGTCCATCAGTAGATTTAGCTATTTTTTCTCTTCACCTTGCTGGTGCTTC  420
            |||||||||||| || |||||||||||||||||||||||||||| |||||||||||||||
Sbjct  361  ATCACATTATGGCCCTTCAGTAGATTTAGCTATTTTTTCTCTTCATCTTGCTGGTGCTTC  420

Query  421  TTCAATTTTAGGTGCAATTAACTTCATCACAACTATTGTAAATATACGATCAATTGGAAT  480
            |||||||||||||||||||||||| || |||||||||||||| |||||||||||||||||
Sbjct  421  TTCAATTTTAGGTGCAATTAACTTTATTACAACTATTGTAAACATACGATCAATTGGAAT  480

Query  481  AACAATAGAACGAATACCATTATTTGTTTGATCTGTTTTAATTACAGCTATTTTACTACT  540
            |||||||||||| |||||||||||||||||||||||||||||||||||||||||| ||||
Sbjct  481  AACAATAGAACGTATACCATTATTTGTTTGATCTGTTTTAATTACAGCTATTTTATTACT  540

Query  541  TCTATCTTTACCTGTATTAGCAGGTGCCATTACAATATTATTAACTGATCGAAACTTTAA  600
            | ||||||||||||||||||||||||||||||||||||| ||||||||||||||||||||
Sbjct  541  TTTATCTTTACCTGTATTAGCAGGTGCCATTACAATATTGTTAACTGATCGAAACTTTAA  600

Query  601  CACATCATTTTTTGATCCTTCAGGAGGAGGAGATCCCATTTTATATCAACACTTATTT  658
             ||||||||||||||||||||||| ||||||||||| |||||||||||||| ||||||
Sbjct  601  TACATCATTTTTTGATCCTTCAGGGGGAGGAGATCCAATTTTATATCAACATTTATTT  658
```

```
>Rhipicephalus sanguineus isolate RSN2SK cytochrome c oxidase subunit I (COX1)
gene, partial cds; mitochondrial
Sequence ID: MW152143.1 Length: 676
Range 1: 1 to 658

Score:1055 bits(571), Expect:0.0,
Identities:629/658(96%),  Gaps:0/658(0%), Strand: Plus/Plus

Query  1    AACAATATATTTAATTTTTGGAGCATGATCAGGTATATTAGGACTTAGTATAAGAATATT  60
            ||||||||||||||||||||||||||||||||||||||||||||||||||||||||||||
Sbjct  1    AACAATATATTTAATTTTTGGAGCATGATCAGGTATATTAGGACTTAGTATAAGAATATT  60

Query  61   AATTCGTATAGAATTAGGACAACCTGGAACTTTAATTGGAAATGATCAGATTTATAATGT  120
            ||||||||||||||||||||||||||||||||||||||||||||||||||||||||||||
Sbjct  61   AATTCGTATAGAATTAGGACAACCTGGAACTTTAATTGGAAATGATCAGATTTATAATGT  120

Query  121  AATCGTAACAGCACATGCATTTATTATAATTTTCTTTATAGTTATACCAATCATAATCGG  180
            ||||||||||||||||||||||||||||||||| ||||||||||||||||||||||||||
Sbjct  121  AATCGTAACAGCACATGCATTTATTATAATTTTTTTTATAGTTATACCAATCATAATCGG  180

Query  181  AGGGTTCGGAAATTGATTAGTTCCTATTATATTAGGTGCCCCAGATATAGCATTTCCACG  240
            ||| || |||||||||||||| ||||| ||||| ||||||||||||||||||||||||||
Sbjct  181  AGGATTTGGAAATTGATTAGTCCCTATCATATTGGGTGCCCCAGATATAGCATTTCCACG  240

Query  241  AATAAATAATATAAGATTTTGACTTTTACCCCCCTCATTATTTTTATTGATTAACTCTTC  300
            |||||||||||||||||||||||||||||| || ||| |||||||||| ||||| |||||
Sbjct  241  AATAAATAATATAAGATTTTGACTTTTACCTCCTTCACTATTTTTATTAATTAATTCTTC  300

Query  301  ATTAATTGAATCCGGAGCTGGAACAGGATGAACAGTTTATCCCCCTCTATCTTCAAATTT  360
            |||||||||||| |||||||| ||||||||||||||||| || |||||||||||||||||
```

```
Sbjct  301   ATTAATTGAATCTGGAGCTGGTACAGGATGAACAGTTTACCCTCCTCTATCTTCAAATTT  360

Query  361   ATCACATTATGGTCCATCAGTAGATTTAGCTATTTTTTCTCTTCACCTTGCTGGTGCTTC  420
             |||||||||||| || ||||||||||||||||||||||||||||| ||||||||||||||
Sbjct  361   ATCACATTATGGCCCTTCAGTAGATTTAGCTATTTTTTCTCTTCATCTTGCTGGTGCTTC  420

Query  421   TTCAATTTTAGGTGCAATTAACTTCATCACAACTATTGTAAATATACGATCAATTGGAAT  480
             |||||||||||||||||||||||| || |||||||||||||| |||||||||||||||||
Sbjct  421   TTCAATTTTAGGTGCAATTAACTTTATTACAACTATTGTAAACATACGATCAATTGGAAT  480

Query  481   AACAATAGAACGAATACCATTATTTGTTTGATCTGTTTTAATTACAGCTATTTTACTACT  540
             |||||||||||| |||||||||||||||||||||||||||||||||||||||||| ||||
Sbjct  481   AACAATAGAACGTATACCATTATTTGTTTGATCTGTTTTAATTACAGCTATTTTATTACT  540

Query  541   TCTATCTTTACCTGTATTAGCAGGTGCCATTACAATATTATTAACTGATCGAAACTTTAA  600
             | ||||||||||||||||||||||||||||||||||||| ||||||||||||||||||||
Sbjct  541   TTTATCTTTACCTGTATTAGCAGGTGCCATTACAATATTGTTAACTGATCGAAACTTTAA  600

Query  601   CACATCATTTTTTGATCCTTCAGGAGGAGGAGATCCCATTTTATATCAACACTTATTT  658
              ||||||||||||||||||||||| ||||||||||| |||||||||||||| ||||||
Sbjct  601   TACATCATTTTTTGATCCTTCAGGGGGAGGAGATCCAATTTTATATCAACATTTATTT  658
```

>Rhipicephalus sanguineus isolate RSN11SK cytochrome c oxidase subunit I (COX1) gene, partial cds; mitochondrial
Sequence ID: MW152142.1 Length: 669
Range 1: 1 to 658

Score:1055 bits(571), Expect:0.0,
Identities:629/658(96%), Gaps:0/658(0%), Strand: Plus/Plus

```
Query  1     AACAATATATTTAATTTTTGGAGCATGATCAGGTATATTAGGACTTAGTATAAGAATATT  60
             ||||||||||||||||||||||||||||||||||||||||||||||||||||||||||||
Sbjct  1     AACAATATATTTAATTTTTGGAGCATGATCAGGTATATTAGGACTTAGTATAAGAATATT  60

Query  61    AATTCGTATAGAATTAGGACAACCTGGAACTTTAATTGGAAATGATCAGATTTATAATGT  120
             ||||||||||||||||||||||||||||||||||||||||||||||||||||||||||||
Sbjct  61    AATTCGTATAGAATTAGGACAACCTGGAACTTTAATTGGAAATGATCAGATTTATAATGT  120

Query  121   AATCGTAACAGCACATGCATTTATTATAATTTCTTTATAGTTATACCAATCATAATCGG  180
             |||||||||||||||||||||||||||||||||| |||||||||||||||||||||||||
Sbjct  121   AATCGTAACAGCACATGCATTTATTATAATTTTTTTATAGTTATACCAATCATAATCGG  180

Query  181   AGGGTTCGGAAATTGATTAGTTCCTATTATATTAGGTGCCCCAGATATAGCATTTCCACG  240
             ||| || |||||||||||||| ||||| ||||| ||||||||||||||||||||||||||
Sbjct  181   AGGATTTGGAAATTGATTAGTCCCTATCATATTGGGTGCCCCAGATATAGCATTTCCACG  240

Query  241   AATAAATAATATAAGATTTTGACTTTTACCCCCCTCATTATTTTTATTGATTAACTCTTC  300
             |||||||||||||||||||||||||||||| || ||| |||||||||| ||||| |||||
Sbjct  241   AATAAATAATATAAGATTTTGACTTTTACCTCCTTCACTATTTTTATTAATTAATTCTTC  300

Query  301   ATTAATTGAATCCGGAGCTGGAACAGGATGAACAGTTTATCCCCCTCTATCTTCAAATTT  360
```

```
            |||||||||||| |||||||| ||||||||||||||||| || |||||||||||||||||
Sbjct  301  ATTAATTGAATCTGGAGCTGGTACAGGATGAACAGTTTACCCTCCTCTATCTTCAAATTT  360

Query  361  ATCACATTATGGTCCATCAGTAGATTTAGCTATTTTTTCTCTTCACCTTGCTGGTGCTTC  420
            |||||||||||| || ||||||||||||||||||||||||||||| ||||||||||||||
Sbjct  361  ATCACATTATGGCCCTTCAGTAGATTTAGCTATTTTTTCTCTTCATCTTGCTGGTGCTTC  420

Query  421  TTCAATTTTAGGTGCAATTAACTTCATCACAACTATTGTAAATATACGATCAATTGGAAT  480
            |||||||||||||||||||||||| || |||||||||||||| |||||||||||||||||
Sbjct  421  TTCAATTTTAGGTGCAATTAACTTTATTACAACTATTGTAAACATACGATCAATTGGAAT  480

Query  481  AACAATAGAACGAATACCATTATTTGTTTGATCTGTTTTAATTACAGCTATTTTACTACT  540
            |||||||||||| |||||||||||||||||||||||||||||||||||||||||| ||||
Sbjct  481  AACAATAGAACGTATACCATTATTTGTTTGATCTGTTTTAATTACAGCTATTTTATTACT  540

Query  541  TCTATCTTTACCTGTATTAGCAGGTGCCATTACAATATTATTAACTGATCGAAACTTTAA  600
            | ||||||||||||||||||||||||||||||||||||| ||||||||||||||||||||
Sbjct  541  TTTATCTTTACCTGTATTAGCAGGTGCCATTACAATATTGTTAACTGATCGAAACTTTAA  600

Query  601  CACATCATTTTTTGATCCTTCAGGAGGAGGAGATCCCATTTTATATCAACACTTATTT  658
             ||||||||||||||||||||||| ||||||||||| |||||||||||||| ||||||
Sbjct  601  TACATCATTTTTTGATCCTTCAGGGGGAGGAGATCCAATTTTATATCAACATTTATTT  658
```

>Rhipicephalus sanguineus sensu lato 'temperate lineage' isolate S358 cytochrome c oxidase subunit I (COI) gene, partial cds; mitochondrial
Sequence ID: MF426012.1 Length: 684
Range 1: 14 to 671

Score:1050 bits(568), Expect:0.0,
Identities:628/658(95%), Gaps:0/658(0%), Strand: Plus/Plus

```
Query  1    AACAATATATTTAATTTTTGGAGCATGATCAGGTATATTAGGACTTAGTATAAGAATATT  60
            ||||||||||||||||||||||||||||||||||||||||||||||||||||||||||||
Sbjct  14   AACAATATATTTAATTTTTGGAGCATGATCAGGTATATTAGGACTTAGTATAAGAATATT  73

Query  61   AATTCGTATAGAATTAGGACAACCTGGAACTTTAATTGGAAATGATCAGATTTATAATGT  120
            ||||||||||||||||||||||||||||||||||||||||||||||||||||||||||||
Sbjct  74   AATTCGTATAGAATTAGGACAACCTGGAACTTTAATTGGAAATGATCAGATTTATAATGT  133

Query  121  AATCGTAACAGCACATGCATTTATTATAATTTTCTTTATAGTTATACCAATCATAATCGG  180
            ||||||||||||||||||||||||||||||||| ||||||||||||||||||||||||||
Sbjct  134  AATCGTAACAGCACATGCATTTATTATAATTTTTTTTATAGTTATACCAATCATAATCGG  193

Query  181  AGGGTTCGGAAATTGATTAGTTCCTATTATATTAGGTGCCCCAGATATAGCATTTCCACG  240
            ||| || |||||||||||||| ||||| ||||||||||||||||||||||||||||||||
Sbjct  194  AGGATTTGGAAATTGATTAGTCCCTATCATATTAGGTGCCCCAGATATAGCATTTCCACG  253

Query  241  AATAAATAATATAAGATTTTGACTTTTACCCCCCTCATTATTTTTATTGATTAACTCTTC  300
            |||||||||||||||||||||||||||||| || ||| |||||||||| |||||||||||
Sbjct  254  AATAAATAATATAAGATTTTGACTTTTACCTCCTTCACTATTTTTATTAATTAACTCTTC  313
```

```
Query  301  ATTAATTGAATCCGGAGCTGGAACAGGATGAACAGTTTATCCCCCTCTATCTTCAAATTT  360
            |||||||||||| |||||||| ||||||||||||||||| || |||||||||||||||||
Sbjct  314  ATTAATTGAATCTGGAGCTGGTACAGGATGAACAGTTTACCCTCCTCTATCTTCAAATTT  373

Query  361  ATCACATTATGGTCCATCAGTAGATTTAGCTATTTTTCTCTTCACCTTGCTGGTGCTTC  420
            |||||||||||| || ||||||||||||||||| |||||||||| |||||||||||||||
Sbjct  374  ATCACATTATGGCCCTTCAGTAGATTTAGCTATCTTTCTCTTCATCTTGCTGGTGCTTC  433

Query  421  TTCAATTTTAGGTGCAATTAACTTCATCACAACTATTGTAAATATACGATCAATTGGAAT  480
            |||||||||||||||||||||||| || ||||||||| |||| |||||||||||||||||
Sbjct  434  TTCAATTTTAGGTGCAATTAACTTTATTACAACTATTATAAACATACGATCAATTGGAAT  493

Query  481  AACAATAGAACGAATACCATTATTTGTTTGATCTGTTTTAATTACAGCTATTTTACTACT  540
            |||||||||||| |||||||||||||||||||||||||||||||||||||||||| ||||
Sbjct  494  AACAATAGAACGTATACCATTATTTGTTTGATCTGTTTTAATTACAGCTATTTTATTACT  553

Query  541  TCTATCTTTACCTGTATTAGCAGGTGCCATTACAATATTATTAACTGATCGAAACTTTAA  600
              ||||||||||||||||||||||||||||||||||||| ||||||||||||||||||||
Sbjct  554  CTTATCTTTACCTGTATTAGCAGGTGCCATTACAATATTGTTAACTGATCGAAACTTTAA  613

Query  601  CACATCATTTTTTGATCCTTCAGGAGGAGGAGATCCCATTTTATATCAACACTTATTT  658
             ||||||||||||||||||||||| ||||||||||| |||||||||||||| ||||||
Sbjct  614  TACATCATTTTTTGATCCTTCAGGGGGAGGAGATCCAATTTTATATCAACATTTATTT  671
```

>Rhipicephalus sanguineus haplotype I cytochrome oxidase subunit I (COI) gene, partial cds; mitochondrial
Sequence ID: KU556743.1 Length: 659
Range 1: 12 to 659

Score:1048 bits(567), Expect:0.0,
Identities:621/648(96%), Gaps:0/648(0%), Strand: Plus/Plus

```
Query  1    AACAATATATTTAATTTTTGGAGCATGATCAGGTATATTAGGACTTAGTATAAGAATATT  60
            ||||||||||||||||||||||||||||||||||||||||||||||||||||||||||||
Sbjct  12   AACAATATATTTAATTTTTGGAGCATGATCAGGTATATTAGGACTTAGTATAAGAATATT  71

Query  61   AATTCGTATAGAATTAGGACAACCTGGAACTTTAATTGGAAATGATCAGATTTATAATGT  120
            ||||||||||||||||||||||||||||||||||||||||||||||||||||||||||||
Sbjct  72   AATTCGTATAGAATTAGGACAACCTGGAACTTTAATTGGAAATGATCAGATTTATAATGT  131

Query  121  AATCGTAACAGCACATGCATTTATTATAATTTTCTTTATAGTTATACCAATCATAATCGG  180
            ||||||||||||||||||||||||||||||||| ||||||||||||||||||||||||||
Sbjct  132  AATCGTAACAGCACATGCATTTATTATAATTTTTTTTATAGTTATACCAATCATAATCGG  191

Query  181  AGGGTTCGGAAATTGATTAGTTCCTATTATATTAGGTGCCCCAGATATAGCATTTCCACG  240
            ||| || ||||||||||| | ||||| ||||| |||||||||||||||||||||||||||
Sbjct  192  AGGATTTGGAAATTGATTAATCCCTATCATATTGGGTGCCCCAGATATAGCATTTCCACG  251

Query  241  AATAAATAATATAAGATTTTGACTTTTACCCCCCTCATTATTTTTATTGATTAACTCTTC  300
            |||||||||||||||||||||||||||||| || ||| |||||||||| |||||||||||
Sbjct  252  AATAAATAATATAAGATTTTGACTTTTACCTCCTTCACTATTTTTATTAATTAACTCTTC  311
```

```
Query  301  ATTAATTGAATCCGGAGCTGGAACAGGATGAACAGTTTATCCCCCTCTATCTTCAAATTT  360
            |||||||||||| |||||||| ||||||||||||||||| || |||||||||||||||||
Sbjct  312  ATTAATTGAATCTGGAGCTGGTACAGGATGAACAGTTTACCCTCCTCTATCTTCAAATTT  371

Query  361  ATCACATTATGGTCCATCAGTAGATTTAGCTATTTTTTCTCTTCACCTTGCTGGTGCTTC  420
            ||||||||||||||| ||||||||||||||||||||||||||||| ||||||||||||||
Sbjct  372  ATCACATTATGGTCCTTCAGTAGATTTAGCTATTTTTTCTCTTCATCTTGCTGGTGCTTC  431

Query  421  TTCAATTTTAGGTGCAATTAACTTCATCACAACTATTGTAAATATACGATCAATTGGAAT  480
            |||||||||||||||||||||||| || |||||||||||||| |||||||||||||||||
Sbjct  432  TTCAATTTTAGGTGCAATTAACTTTATTACAACTATTGTAAACATACGATCAATTGGAAT  491

Query  481  AACAATAGAACGAATACCATTATTTGTTTGATCTGTTTTAATTACAGCTATTTTACTACT  540
            |||||||||||| |||||||||||||||||||||||||||||||||||||||||| ||||
Sbjct  492  AACAATAGAACGTATACCATTATTTGTTTGATCTGTTTTAATTACAGCTATTTTATTACT  551

Query  541  TCTATCTTTACCTGTATTAGCAGGTGCCATTACAATATTATTAACTGATCGAAACTTTAA  600
            | ||||||||||||||||||||||||||||||||||||| ||||||||||||||||||||
Sbjct  552  TTTATCTTTACCTGTATTAGCAGGTGCCATTACAATATTGTTAACTGATCGAAACTTTAA  611

Query  601  CACATCATTTTTTGATCCTTCAGGAGGAGGAGATCCCATTTTATATCA  648
             ||||||||||||||||||||||| ||||||||||| |||||||||||
Sbjct  612  TACATCATTTTTTGATCCTTCAGGGGGAGGAGATCCAATTTTATATCA  659
```

>Rhipicephalus sanguineus haplotype II cytochrome oxidase subunit I (COI) gene, partial cds; mitochondrial
Sequence ID: KU556744.1 Length: 787
Range 1: 29 to 688

Score:1046 bits(566), Expect:0.0,
Identities:629/660(95%), Gaps:2/660(0%), Strand: Plus/Plus

```
Query  1    AACAATATATTTAATTTTTGGAGCATGATCAGGTATATTAGGACTTAGTATAAGAATATT  60
            ||||||||||||||||||||||||||||||||||||||||||||||||||||||||||||
Sbjct  29   AACAATATATTTAATTTTTGGAGCATGATCAGGTATATTAGGACTTAGTATAAGAATATT  88

Query  61   AATTCGTATAGAATTAGGACAACCTGGAACTTTAATTGGAAATGATCAGATTTATAATGT  120
            ||||||||||||||||||||||||||||||||||||||||||||||||||||||||||||
Sbjct  89   AATTCGTATAGAATTAGGACAACCTGGAACTTTAATTGGAAATGATCAGATTTATAATGT  148

Query  121  AATCGTAACAGCACATGCATTTATTATAATTTTCTTTATAGTTATACCAATCATAATCGG  180
            |||||||||||||||||||||||||||||||||| |||||||||||||||||||||||||
Sbjct  149  AATCGTAACAGCACATGCATTTATTATAATTTTTTTTATAGTTATACCAATCATAATCGG  208

Query  181  AGGGTTCGGAAATTGATTAGTTCCTATTATATTAGGTGCCCCAGATATAGCATTTCCACG  240
            ||| || ||||| |||||||| ||||| ||||| ||||||||||||||||||||||||||
Sbjct  209  AGGATTTGGAAACTGATTAGTCCCTATCATATTGGGTGCCCCAGATATAGCATTTCCACG  268

Query  241  AATAAATAATATAAGATTTTGACTTTTACCCCCCTCATTATTTTTATTGATTAACTCTTC  300
            |||||||||||||||||||||||||||||| || ||| |||||||||| |||||||||||
```

```
Sbjct  269   AATAAATAATATAAGATTTTGACTTTTACCTCCTTCACTATTTTTATTAATTAACTCTTC  328

Query  301   ATTAATTGAATCCGGAGCTGGAACAGGATGAACAGTTTATCCCCCTCTATCTTCAAATTT  360
             |||||||||||| |||||||| ||||||||||||||||| || |||||||||||||||||
Sbjct  329   ATTAATTGAATCTGGAGCTGGTACAGGATGAACAGTTTACCCTCCTCTATCTTCAAATTT  388

Query  361   ATCACATTATGGTCCATCAGTAGATTTAGCTATTTTTTCTCTTCACCTTGCTGGTGCTTC  420
             ||||||||||||||| ||||||||||||||||||||||||||||| ||||||||||||||
Sbjct  389   ATCACATTATGGTCCTTCAGTAGATTTAGCTATTTTTTCTCTTCATCTTGCTGGTGCTTC  448

Query  421   TTCAATTTTAGGTGCAATTAACTTCATCACAACTATTGTAAATATACGATCAATTGGAAT  480
             |||||||||||||||||||||||| || |||||||||||||| |||||||||||||||||
Sbjct  449   TTCAATTTTAGGTGCAATTAACTTTATTACAACTATTGTAAACATACGATCAATTGGAAT  508

Query  481   AACAATAGAACGAATACCATTATTTGTTTGATCTGTTTTAATTACAGCTATTTTACTACT  540
             |||||||||||| |||||||||||||||||||||||||||||||||||||||||| ||||
Sbjct  509   AACAATAGAACGTATACCATTATTTGTTTGATCTGTTTTAATTACAGCTATTTTATTACT  568

Query  541   TCTATCTTTACCTGTATTAGCAGGTGCCATTACAATATTATTAACTGATCGAAACTTTAA  600
             | ||||||||||||||||||||||||||||||||||||| ||||||||||||||||||||
Sbjct  569   TTTATCTTTACCTGTATTAGCAGGTGCCATTACAATATTGTTAACTGATCGAAACTTTAA  628

Query  601   CACATCATTTTTTGATCCTTCAGGAGG-AGGAGATCCC-ATTTTATATCAACACTTATTT  658
              ||||||||||||||||||||||| || |||||||||  |||||||||||||  ||||||
Sbjct  629   TACATCATTTTTTGATCCTTCAGGGGGGAGGAGATCCAAATTTTATATCAACTTTTATTT  688
```

>Rhipicephalus sanguineus sensu lato 'temperate lineage' isolate S943 cytochrome c oxidase subunit I (COI) gene, partial cds; mitochondrial
Sequence ID: MF426020.1 Length: 812
Range 1: 2 to 644

Score:1038 bits(562), Expect:0.0,
Identities:616/643(96%), Gaps:0/643(0%), Strand: Plus/Plus

```
Query  16    TTTTGGAGCATGATCAGGTATATTAGGACTTAGTATAAGAATATTAATTCGTATAGAATT  75
             ||||||||||||||||||||||||||||||||||||||||||||||||||||||||||||
Sbjct  2     TTTTGGAGCATGATCAGGTATATTAGGACTTAGTATAAGAATATTAATTCGTATAGAATT  61

Query  76    AGGACAACCTGGAACTTTAATTGGAAATGATCAGATTTATAATGTAATCGTAACAGCACA  135
             ||||||||||||||||||||||||||||||||||||||||||||||||||||||||||||
Sbjct  62    AGGACAACCTGGAACTTTAATTGGAAATGATCAGATTTATAATGTAATCGTAACAGCACA  121

Query  136   TGCATTTATTATAATTTTCTTTATAGTTATACCAATCATAATCGGAGGGTTCGGAAATTG  195
             |||||||||||||||||| ||||||||||||||||||||||||||||| || ||||||||
Sbjct  122   TGCATTTATTATAATTTTTTTTATAGTTATACCAATCATAATCGGAGGATTTGGAAATTG  181

Query  196   ATTAGTTCCTATTATATTAGGTGCCCCAGATATAGCATTTCCACGAATAAATAATATAAG  255
             |||||| ||||| ||||| |||||||||||||||||||||||||||||||||||||||||
Sbjct  182   ATTAGTCCCTATCATATTGGGTGCCCCAGATATAGCATTTCCACGAATAAATAATATAAG  241

Query  256   ATTTTGACTTTTACCCCCTCATTATTTTTATTGATTAACTCTTCATTAATTGAATCCGG  315
```

```
                |||||||||||||||| || ||| |||||||||| ||||||||||||||||||||||| ||
Sbjct  242  ATTTTGACTTTTACCTCCTTCACTATTTTTATTAATTAACTCTTCATTAATTGAATCTGG  301

Query  316  AGCTGGAACAGGATGAACAGTTTATCCCCCTCTATCTTCAAATTTATCACATTATGGTCC  375
            |||||| ||||||||||||||||| || ||||||||||||||||||||||||||||||||
Sbjct  302  AGCTGGTACAGGATGAACAGTTTACCCTCCTCTATCTTCAAATTTATCACATTATGGTCC  361

Query  376  ATCAGTAGATTTAGCTATTTTTCTCTTCACCTTGCTGGTGCTTCTTCAATTTTAGGTGC  435
             |||||||||||||||||||||||||||| ||||||||||||||||||||||||||||||
Sbjct  362  TTCAGTAGATTTAGCTATTTTTCTCTTCATCTTGCTGGTGCTTCTTCAATTTTAGGTGC  421

Query  436  AATTAACTTCATCACAACTATTGTAAATATACGATCAATTGGAATAACAATAGAACGAAT  495
            ||||||||| || |||||||||||||| ||||||||||||||||||||||||||||| ||
Sbjct  422  AATTAACTTTATTACAACTATTGTAAACATACGATCAATTGGAATAACAATAGAACGTAT  481

Query  496  ACCATTATTTGTTTGATCTGTTTTAATTACAGCTATTTTACTACTTCTATCTTTACCTGT  555
            |||||||||||||||||||||||||||||||||||||||| ||||| |||||||||||||
Sbjct  482  ACCATTATTTGTTTGATCTGTTTTAATTACAGCTATTTTATTACTTTTATCTTTACCTGT  541

Query  556  ATTAGCAGGTGCCATTACAATATTATTAACTGATCGAAACTTTAACACATCATTTTTTGA  615
            |||||||||||||||||||||||| |||||||||||||||||||| ||||||||||||||
Sbjct  542  ATTAGCAGGTGCCATTACAATATTGTTAACTGATCGAAACTTTAATACATCATTTTTTGA  601

Query  616  TCCTTCAGGAGGAGGAGATCCCATTTTATATCAACACTTATTT  658
            ||||||||| ||||||||||| |||||||||||||| ||||||
Sbjct  602  TCCTTCAGGGGGAGGAGATCCAATTTTATATCAACATTTATTT  644
```

>Rhipicephalus sanguineus sensu lato 'temperate lineage' isolate S337 cytochrome c oxidase subunit I (COI) gene, partial cds; mitochondrial
Sequence ID: MF426010.1 Length: 824
Range 1: 1 to 648

Score:1037 bits(561), Expect:0.0,
Identities:619/648(96%), Gaps:0/648(0%), Strand: Plus/Plus

```
Query  11   TTAATTTTTGGAGCATGATCAGGTATATTAGGACTTAGTATAAGAATATTAATTCGTATA  70
            ||||||||||||||||||||||||||||||||||||||||||||||||||||||||||||
Sbjct  1    TTAATTTTTGGAGCATGATCAGGTATATTAGGACTTAGTATAAGAATATTAATTCGTATA  60

Query  71   GAATTAGGACAACCTGGAACTTTAATTGGAAATGATCAGATTTATAATGTAATCGTAACA  130
            ||||||||||||||||||||||||||||||||||||||||||||||||||||||||||||
Sbjct  61   GAATTAGGACAACCTGGAACTTTAATTGGAAATGATCAGATTTATAATGTAATCGTAACA  120

Query  131  GCACATGCATTTATTATAATTTTCTTTATAGTTATACCAATCATAATCGGAGGGTTCGGA  190
            ||||||||||||||||||||||| ||||||||||||||||||||||||||||| || |||
Sbjct  121  GCACATGCATTTATTATAATTTTTTTTATAGTTATACCAATCATAATCGGAGGATTTGGA  180

Query  191  AATTGATTAGTTCCTATTATATTAGGTGCCCCAGATATAGCATTTCCACGAATAAATAAT  250
            ||||||||||| ||||| ||||| ||||||||||||||||||||||||||||||||||||
Sbjct  181  AATTGATTAGTCCCTATCATATTGGGTGCCCCAGATATAGCATTTCCACGAATAAATAAT  240
```

```
Query  251  ATAAGATTTTGACTTTTACCCCCCTCATTATTTTTATTGATTAACTCTTCATTAATTGAA  310
            |||||||||||||||||||| || ||| |||||||||| |||||||||||||||||||||
Sbjct  241  ATAAGATTTTGACTTTTACCTCCTTCACTATTTTTATTAATTAACTCTTCATTAATTGAA  300

Query  311  TCCGGAGCTGGAACAGGATGAACAGTTTATCCCCCTCTATCTTCAAATTTATCACATTAT  370
            || |||||||| ||||||||||||||||| || |||||||||||||||||||||||||||
Sbjct  301  TCTGGAGCTGGTACAGGATGAACAGTTTACCCTCCTCTATCTTCAAATTTATCACATTAT  360

Query  371  GGTCCATCAGTAGATTTAGCTATTTTTCTCTTCACCTTGCTGGTGCTTCTTCAATTTTA  430
            || || ||||||||||||||||| |||||||||| |||||||||||||||||||||||||
Sbjct  361  GGCCCTTCAGTAGATTTAGCTATCTTTCTCTTCATCTTGCTGGTGCTTCTTCAATTTTA  420

Query  431  GGTGCAATTAACTTCATCACAACTATTGTAAATATACGATCAATTGGAATAACAATAGAA  490
            |||||||||||||| || |||||||||||||| |||||||||||||||||||||||||||
Sbjct  421  GGTGCAATTAACTTTATTACAACTATTGTAAACATACGATCAATTGGAATAACAATAGAA  480

Query  491  CGAATACCATTATTTGTTTGATCTGTTTTAATTACAGCTATTTTACTACTTCTATCTTTA  550
            || |||||||||||||||||||||||||||||||||||||||||| ||||| ||||||||
Sbjct  481  CGTATACCATTATTTGTTTGATCTGTTTTAATTACAGCTATTTTATTACTTTTATCTTTA  540

Query  551  CCTGTATTAGCAGGTGCCATTACAATATTATTAACTGATCGAAACTTTAACACATCATTT  610
            ||||||||||||||||||||||||||||| |||||||||||||||||||| |||||||||
Sbjct  541  CCTGTATTAGCAGGTGCCATTACAATATTGTTAACTGATCGAAACTTTAATACATCATTT  600

Query  611  TTTGATCCTTCAGGAGGAGGAGATCCCATTTTATATCAACACTTATTT  658
            |||||||||||||| ||||||||||| |||||||||||||| ||||||
Sbjct  601  TTTGATCCTTCAGGGGGAGGAGATCCAATTTTATATCAACATTTATTT  648
```

>Rhipicephalus sanguineus sensu lato 'temperate lineage' isolate S344 cytochrome c oxidase subunit I (COI) gene, partial cds; mitochondrial
Sequence ID: MF426011.1 Length: 809
Range 1: 3 to 644

Score:1031 bits(558), Expect:0.0,
Identities:614/642(96%), Gaps:0/642(0%), Strand: Plus/Plus

```
Query  17   TTTGGAGCATGATCAGGTATATTAGGACTTAGTATAAGAATATTAATTCGTATAGAATTA  76
            ||||||||||||||||||||||||||||||||||||||||||||||||||||||||||||
Sbjct  3    TTTGGAGCATGATCAGGTATATTAGGACTTAGTATAAGAATATTAATTCGTATAGAATTA  62

Query  77   GGACAACCTGGAACTTTAATTGGAAATGATCAGATTTATAATGTAATCGTAACAGCACAT  136
            ||||||||||||||||||||||||||||||||||||||||||||||||||||||||||| 
Sbjct  63   GGACAACCTGGAACTTTAATTGGAAATGATCAGATTTATAATGTAATCGTAACAGCACAC  122

Query  137  GCATTTATTATAATTTTCTTTATAGTTATACCAATCATAATCGGAGGGTTCGGAAATTGA  196
            ||||||||||||||||| ||||||||||||||||||||||||||||| || |||||||||
Sbjct  123  GCATTTATTATAATTTTTTTTATAGTTATACCAATCATAATCGGAGGATTTGGAAATTGA  182

Query  197  TTAGTTCCTATTATATTAGGTGCCCCAGATATAGCATTTCCACGAATAAATAATATAAGA  256
            ||||| ||||||||||||||||||||||||||||||||||||||||||||||||||||||
Sbjct  183  TTAGTCCCTATTATATTAGGTGCCCCAGATATAGCATTTCCACGAATAAATAATATAAGA  242
```

```
Query  257   TTTTGACTTTTACCCCCCTCATTATTTTTATTGATTAACTCTTCATTAATTGAATCCGGA  316
             |||||||||||||| || ||| |||||||||| ||||||||||||||||||||||| |||
Sbjct  243   TTTTGACTTTTACCTCCTTCACTATTTTTATTAATTAACTCTTCATTAATTGAATCTGGA  302

Query  317   GCTGGAACAGGATGAACAGTTTATCCCCCTCTATCTTCAAATTTATCACATTATGGTCCA  376
             ||||| ||||||||||||||||| || ||||||||||||||||||||||||||||| ||
Sbjct  303   GCTGGTACAGGATGAACAGTTTACCCTCCTCTATCTTCAAATTTATCACATTATGGCCCT  362

Query  377   TCAGTAGATTTAGCTATTTTTTCTCTTCACCTTGCTGGTGCTTCTTCAATTTTAGGTGCA  436
             ||||||||||||||||| ||||||||||| ||||||||||||||||||||||||||||||
Sbjct  363   TCAGTAGATTTAGCTATCTTTTCTCTTCATCTTGCTGGTGCTTCTTCAATTTTAGGTGCA  422

Query  437   ATTAACTTCATCACAACTATTGTAAATATACGATCAATTGGAATAACAATAGAACGAATA  496
             |||||||| || |||||||||||||| ||||||||||||||||||||||||||||| |||
Sbjct  423   ATTAACTTTATTACAACTATTGTAAACATACGATCAATTGGAATAACAATAGAACGTATA  482

Query  497   CCATTATTTGTTTGATCTGTTTTAATTACAGCTATTTTACTACTTCTATCTTTACCTGTA  556
             ||||||||||||||||||||||||||||||||||||||| ||||| ||||||||||||||
Sbjct  483   CCATTATTTGTTTGATCTGTTTTAATTACAGCTATTTTATTACTTTTATCTTTACCTGTA  542

Query  557   TTAGCAGGTGCCATTACAATATTATTAACTGATCGAAACTTTAACACATCATTTTTTGAT  616
             ||||||||||||||||||||||| |||||||||||||||||||| |||||||||||||||
Sbjct  543   TTAGCAGGTGCCATTACAATATTGTTAACTGATCGAAACTTTAATACATCATTTTTTGAT  602

Query  617   CCTTCAGGAGGAGGAGATCCCATTTTATATCAACACTTATTT  658
             |||||||| ||||||||||| |||||||||||||| ||||||
Sbjct  603   CCTTCAGGGGGAGGAGATCCAATTTTATATCAACATTTATTT  644
```

>Rhipicephalus sanguineus sensu lato 'temperate lineage' isolate S475 cytochrome c oxidase subunit I (COI) gene, partial cds; mitochondrial
Sequence ID: MF426014.1 Length: 649
Range 1: 28 to 649

Score:1022 bits(553), Expect:0.0,
Identities:599/622(96%), Gaps:0/622(0%), Strand: Plus/Plus

```
Query  1     AACAATATATTTAATTTTTGGAGCATGATCAGGTATATTAGGACTTAGTATAAGAATATT  60
             ||||||||||||||||||||||||||||||||||||||||||||||||||||||||||||
Sbjct  28    AACAATATATTTAATTTTTGGAGCATGATCAGGTATATTAGGACTTAGTATAAGAATATT  87

Query  61    AATTCGTATAGAATTAGGACAACCTGGAACTTTAATTGGAAATGATCAGATTTATAATGT  120
             ||||||||||||||||||||||||||||||||||||||||||||||||||||||||||||
Sbjct  88    AATTCGTATAGAATTAGGACAACCTGGAACTTTAATTGGAAATGATCAGATTTATAATGT  147

Query  121   AATCGTAACAGCACATGCATTTATTATAATTTCTTTATAGTTATACCAATCATAATCGG  180
             |||||||||||||||||||||||||||||||| |||||||||||||||||||||||||||
Sbjct  148   AATCGTAACAGCACATGCATTTATTATAATTTTTTTATAGTTATACCAATCATAATCGG  207

Query  181   AGGGTTCGGAAATTGATTAGTTCCTATTATATTAGGTGCCCCAGATATAGCATTTCCACG  240
             ||| || ||||||||||||| ||||| ||||| |||||||||||||||||||||||||||
```

```
Sbjct  208   AGGATTTGGAAATTGATTAGTCCCTATCATATTGGGTGCCCCAGATATAGCATTTCCACG  267

Query  241   AATAAATAATATAAGATTTTGACTTTTACCCCCCTCATTATTTTTATTGATTAACTCTTC  300
             |||||||||||||||||||||||||||||| || ||| |||||||||| |||||||||||
Sbjct  268   AATAAATAATATAAGATTTTGACTTTTACCTCCTTCACTATTTTTATTAATTAACTCTTC  327

Query  301   ATTAATTGAATCCGGAGCTGGAACAGGATGAACAGTTTATCCCCCTCTATCTTCAAATTT  360
             |||||||||||| |||||||| ||||||||||||||||| || |||||||||||||||||
Sbjct  328   ATTAATTGAATCTGGAGCTGGTACAGGATGAACAGTTTACCCTCCTCTATCTTCAAATTT  387

Query  361   ATCACATTATGGTCCATCAGTAGATTTAGCTATTTTTTCTCTTCACCTTGCTGGTGCTTC  420
             ||||||||||||||| |||||||||||||||||||||||||||| |||||||||||||||
Sbjct  388   ATCACATTATGGTCCTTCAGTAGATTTAGCTATTTTTTCTCTTCATCTTGCTGGTGCTTC  447

Query  421   TTCAATTTTAGGTGCAATTAACTTCATCACAACTATTGTAAATATACGATCAATTGGAAT  480
             |||||||||||||||||||||||| || |||||||||||||| |||||||||||||||||
Sbjct  448   TTCAATTTTAGGTGCAATTAACTTTATTACAACTATTGTAAACATACGATCAATTGGAAT  507

Query  481   AACAATAGAACGAATACCATTATTTGTTTGATCTGTTTTAATTACAGCTATTTTACTACT  540
             |||||||||||| |||||||||||||||||||||||||||||||||||||||||||||||
Sbjct  508   AACAATAGAACGTATACCATTATTTGTTTGATCTGTTTTAATTACAGCTATTTTACTACT  567

Query  541   TCTATCTTTACCTGTATTAGCAGGTGCCATTACAATATTATTAACTGATCGAAACTTTAA  600
             | ||||||||||||||||||||||||||||||||||||| ||||||||||||||||||||
Sbjct  568   TTTATCTTTACCTGTATTAGCAGGTGCCATTACAATATTGTTAACTGATCGAAACTTTAA  627

Query  601   CACATCATTTTTTGATCCTTCA  622
              |||||||||||||||||||||
Sbjct  628   TACATCATTTTTTGATCCTTCA  649
```

>Rhipicephalus sanguineus isolate LIC5424B cytochrome oxidase subunit 1 (COI) gene, partial cds; mitochondrial
Sequence ID: KX383818.1 Length: 768
>Rhipicephalus sanguineus isolate LIC5554A cytochrome oxidase subunit 1 (COI) gene, partial cds; mitochondrial
Sequence ID: KX383819.1 Length: 768
Range 1: 1 to 642

Score:1020 bits(552), Expect:0.0,
Identities:612/642(95%), Gaps:0/642(0%), Strand: Plus/Plus

```
Query  17    TTTGGAGCATGATCAGGTATATTAGGACTTAGTATAAGAATATTAATTCGTATAGAATTA  76
             ||||||||||||||||||||||||||||||||||||||||||||||||||||||||||||
Sbjct  1     TTTGGAGCATGATCAGGTATATTAGGACTTAGTATAAGAATATTAATTCGTATAGAATTA  60

Query  77    GGACAACCTGGAACTTTAATTGGAAATGATCAGATTTATAATGTAATCGTAACAGCACAT  136
             ||||||||||||||||||||||||||||||||||||||||||||||||||||||||||||
Sbjct  61    GGACAACCTGGAACTTTAATTGGAAATGATCAGATTTATAATGTAATCGTAACAGCACAT  120

Query  137   GCATTTATTATAATTTTCTTTATAGTTATACCAATCATAATCGGAGGGTTCGGAAATTGA  196
             ||||||||||||||||| ||||||||||||||||||||||||||||| || |||||||||
```

```
Sbjct  121  GCATTTATTATAATTTTTTTATAGTTATACCAATCATAATCGGAGGATTTGGAAATTGA  180

Query  197  TTAGTTCCTATTATATTAGGTGCCCCAGATATAGCATTTCCACGAATAAATAATATAAGA  256
            ||||| ||||| ||||| ||||||||||||||||||||||||||||||||||||||||||
Sbjct  181  TTAGTCCCTATCATATTGGGTGCCCCAGATATAGCATTTCCACGAATAAATAATATAAGA  240

Query  257  TTTTGACTTTTACCCCCCTCATTATTTTTATTGATTAACTCTTCATTAATTGAATCCGGA  316
            |||||||||||||| || ||| |||||||||| ||||||||||||||||||||||| |||
Sbjct  241  TTTTGACTTTTACCTCCTTCACTATTTTTATTAATTAACTCTTCATTAATTGAATCTGGA  300

Query  317  GCTGGAACAGGATGAACAGTTTATCCCCCTCTATCTTCAAATTTATCACATTATGGTCCA  376
            ||||| ||||||||||||||||| || ||||||||||||||||||||||||||||| ||
Sbjct  301  GCTGGTACAGGATGAACAGTTTACCCTCCTCTATCTTCAAATTTATCACATTATGGCCCT  360

Query  377  TCAGTAGATTTAGCTATTTTTTCTCTTCACCTTGCTGGTGCTTCTTCAATTTTAGGTGCA  436
            ||||||||||||||||| ||||||||||| ||||||||||||||||||||||||||||||
Sbjct  361  TCAGTAGATTTAGCTATCTTTTCTCTTCATCTTGCTGGTGCTTCTTCAATTTTAGGTGCA  420

Query  437  ATTAACTTCATCACAACTATTGTAAATATACGATCAATTGGAATAACAATAGAACGAATA  496
            |||||||| || |||||||||||||| ||||| ||||||||||||||||||||||| |||
Sbjct  421  ATTAACTTTATTACAACTATTGTAAACATACGGTCAATTGGAATAACAATAGAACGTATA  480

Query  497  CCATTATTTGTTTGATCTGTTTTAATTACAGCTATTTTACTACTTCTATCTTTACCTGTA  556
            ||||||||||||||||||||||||||||||||||||||| ||||| ||||||||||||||
Sbjct  481  CCATTATTTGTTTGATCTGTTTTAATTACAGCTATTTTATTACTTTTATCTTTACCTGTA  540

Query  557  TTAGCAGGTGCCATTACAATATTATTAACTGATCGAAACTTTAACACATCATTTTTTGAT  616
            ||||||||||||||||||||||| |||||||||||||||||||| |||||||||||||||
Sbjct  541  TTAGCAGGTGCCATTACAATATTGTTAACTGATCGAAACTTTAATACATCATTTTTTGAT  600

Query  617  CCTTCAGGAGGAGGAGATCCCATTTTATATCAACACTTATTT  658
            |||||||| ||||||||||| |||||||||||||| ||||||
Sbjct  601  CCTTCAGGGGGAGGAGATCCAATTTTATATCAACATTTATTT  642
```

>Rhipicephalus sanguineus sensu lato 'temperate lineage' isolate CR1568
cytochrome c oxidase subunit I (COI) gene, partial cds; mitochondrial
Sequence ID: MF426000.1 Length: 635
Range 1: 14 to 635

Score:1016 bits(550), Expect:0.0,
Identities:598/622(96%), Gaps:0/622(0%), Strand: Plus/Plus

```
Query  1    AACAATATATTTAATTTTTGGAGCATGATCAGGTATATTAGGACTTAGTATAAGAATATT  60
            ||||||||||||||||||||||||||||||||||||||||||||||||||||||||||||
Sbjct  14   AACAATATATTTAATTTTTGGAGCATGATCAGGTATATTAGGACTTAGTATAAGAATATT  73

Query  61   AATTCGTATAGAATTAGGACAACCTGGAACTTTAATTGGAAATGATCAGATTTATAATGT  120
            ||||||||||||||||||||||||||||||||||||||||||||||||||||||||||||
Sbjct  74   AATTCGTATAGAATTAGGACAACCTGGAACTTTAATTGGAAATGATCAGATTTATAATGT  133

Query  121  AATCGTAACAGCACATGCATTTATTATAATTTTCTTTATAGTTATACCAATCATAATCGG  180
```

```
            ||||||||||||||||||||||||||||||||| ||||||||||||||||||||||||||
Sbjct  134  AATCGTAACAGCACATGCATTTATTATAATTTTTTTTATAGTTATACCAATCATAATCGG  193

Query  181  AGGGTTCGGAAATTGATTAGTTCCTATTATATTAGGTGCCCCAGATATAGCATTTCCACG  240
            ||| || |||||||||||||| ||||| ||||| ||||||||||||||||||||||||||
Sbjct  194  AGGATTTGGAAATTGATTAGTCCCTATCATATTGGGTGCCCCAGATATAGCATTTCCACG  253

Query  241  AATAAATAATATAAGATTTTGACTTTTACCCCCCTCATTATTTTTATTGATTAACTCTTC  300
            |||||||||||||||||||||||||||||| || ||| |||||||||| |||||||||||
Sbjct  254  AATAAATAATATAAGATTTTGACTTTTACCTCCTTCACTATTTTTATTAATTAACTCTTC  313

Query  301  ATTAATTGAATCCGGAGCTGGAACAGGATGAACAGTTTATCCCCCTCTATCTTCAAATTT  360
            |||||||||||| |||||||| ||||||||||||||||| || |||||||||||||||||
Sbjct  314  ATTAATTGAATCTGGAGCTGGTACAGGATGAACAGTTTACCCTCCTCTATCTTCAAATTT  373

Query  361  ATCACATTATGGTCCATCAGTAGATTTAGCTATTTTTTCTCTTCACCTTGCTGGTGCTTC  420
            ||||||||||||||| ||||||||||||||||||||||||||||| ||||||||||||||
Sbjct  374  ATCACATTATGGTCCTTCAGTAGATTTAGCTATTTTTTCTCTTCATCTTGCTGGTGCTTC  433

Query  421  TTCAATTTTAGGTGCAATTAACTTCATCACAACTATTGTAAATATACGATCAATTGGAAT  480
            |||||||||||||||||||||||| || ||||||||||||||| ||||||||||||||||
Sbjct  434  TTCAATTTTAGGTGCAATTAACTTTATTACAACTATTGTAAACATACGATCAATTGGAAT  493

Query  481  AACAATAGAACGAATACCATTATTTGTTTGATCTGTTTTAATTACAGCTATTTTACTACT  540
            |||||||||||| |||||||||||||||||||||||||||||||||||||||||| ||||
Sbjct  494  AACAATAGAACGTATACCATTATTTGTTTGATCTGTTTTAATTACAGCTATTTTATTACT  553

Query  541  TCTATCTTTACCTGTATTAGCAGGTGCCATTACAATATTATTAACTGATCGAAACTTTAA  600
            | ||||||||||||||||||||||||||||||||||||| ||||||||||||||||||||
Sbjct  554  TTTATCTTTACCTGTATTAGCAGGTGCCATTACAATATTGTTAACTGATCGAAACTTTAA  613

Query  601  CACATCATTTTTTGATCCTTCA  622
             |||||||||||||||||||||
Sbjct  614  TACATCATTTTTTGATCCTTCA  635
```

>Rhipicephalus sanguineus sensu lato 'temperate lineage' isolate SF3003 cytochrome c oxidase subunit I (COI) gene, partial cds; mitochondrial
Sequence ID: MF425988.1 Length: 654
Range 1: 1 to 640

Score:1016 bits(550), Expect:0.0,
Identities:610/640(95%), Gaps:0/640(0%), Strand: Plus/Plus

```
Query  17   TTTGGAGCATGATCAGGTATATTAGGACTTAGTATAAGAATATTAATTCGTATAGAATTA  76
            ||||||||||||||||||||||||||||||||||||||||||||||||||||||||||||
Sbjct  1    TTTGGAGCATGATCAGGTATATTAGGACTTAGTATAAGAATATTAATTCGTATAGAATTA  60

Query  77   GGACAACCTGGAACTTTAATTGGAAATGATCAGATTTATAATGTAATCGTAACAGCACAT  136
            ||||||||||||||||||||||||||||||||||||||||||||||||||||||||||||
Sbjct  61   GGACAACCTGGAACTTTAATTGGAAATGATCAGATTTATAATGTAATCGTAACAGCACAT  120
```

```
Query  137  GCATTTATTATAATTTTCTTTATAGTTATACCAATCATAATCGGAGGGTTCGGAAATTGA  196
            ||||||||||||||||| ||||||||||||||||||||||||||||| || |||||||||
Sbjct  121  GCATTTATTATAATTTTTTTTATAGTTATACCAATCATAATCGGAGGATTTGGAAATTGA  180

Query  197  TTAGTTCCTATTATATTAGGTGCCCCAGATATAGCATTTCCACGAATAAATAATATAAGA  256
            ||||| ||||| ||||||||||||||||||||||||||||||||||||||||||||||||
Sbjct  181  TTAGTCCCTATCATATTAGGTGCCCCAGATATAGCATTTCCACGAATAAATAATATAAGA  240

Query  257  TTTTGACTTTTACCCCCCTCATTATTTTTATTGATTAACTCTTCATTAATTGAATCCGGA  316
            |||||||||||||| || ||| |||||||||| ||||||||||||||||||||||| |||
Sbjct  241  TTTTGACTTTTACCTCCTTCACTATTTTTATTAATTAACTCTTCATTAATTGAATCTGGA  300

Query  317  GCTGGAACAGGATGAACAGTTTATCCCCCTCTATCTTCAAATTTATCACATTATGGTCCA  376
            ||||| ||||||||||||||||| || ||||||||||||||||||||||||||||| || 
Sbjct  301  GCTGGTACAGGATGAACAGTTTACCCTCCTCTATCTTCAAATTTATCACATTATGGCCCT  360

Query  377  TCAGTAGATTTAGCTATTTTTTCTCTTCACCTTGCTGGTGCTTCTTCAATTTTAGGTGCA  436
            ||||||||||||||||| ||||||||||| ||||||||||||||||||||||||||||||
Sbjct  361  TCAGTAGATTTAGCTATCTTTTCTCTTCATCTTGCTGGTGCTTCTTCAATTTTAGGTGCA  420

Query  437  ATTAACTTCATCACAACTATTGTAAATATACGATCAATTGGAATAACAATAGAACGAATA  496
            |||||||| || |||||||||||||| ||||||||||||||||||||||||||||| |||
Sbjct  421  ATTAACTTTATTACAACTATTGTAAACATACGATCAATTGGAATAACAATAGAACGTATA  480

Query  497  CCATTATTTGTTTGATCTGTTTTAATTACAGCTATTTTACTACTTCTATCTTTACCTGTA  556
            ||||||||||||||||||||||||||||||||||||||| ||||| ||||||||||||||
Sbjct  481  CCATTATTTGTTTGATCTGTTTTAATTACAGCTATTTTATTACTTTTATCTTTACCTGTA  540

Query  557  TTAGCAGGTGCCATTACAATATTATTAACTGATCGAAACTTTAACACATCATTTTTTGAT  616
            ||||||||||||||||||||||| |||||||||||||||||||| |||||||||||||||
Sbjct  541  TTAGCAGGTGCCATTACAATATTGTTAACTGATCGAAACTTTAATACATCATTTTTTGAT  600

Query  617  CCTTCAGGAGGAGGAGATCCCATTTTATATCAACACTTAT  656
            |||||||| |||||| |||| ||||||||||| || ||||
Sbjct  601  CCTTCAGGGGGAGGACATCCAATTTTATATCACCATTTAT  640
```

>Rhipicephalus sanguineus isolate LIC5533B cytochrome oxidase subunit 1 (COI) gene, partial cds; mitochondrial
Sequence ID: KX383811.1 Length: 764
>Rhipicephalus sanguineus isolate LIC5533C cytochrome oxidase subunit 1 (COI) gene, partial cds; mitochondrial
Sequence ID: KX383812.1 Length: 764
Range 1: 1 to 638

Score:1013 bits(548), Expect:0.0,
Identities:608/638(95%), Gaps:0/638(0%), Strand: Plus/Plus

```
Query  21   GAGCATGATCAGGTATATTAGGACTTAGTATAAGAATATTAATTCGTATAGAATTAGGAC  80
            ||||||||||||||||||||||||||||||||||||||||||||||||||||||||||||
Sbjct  1    GAGCATGATCAGGTATATTAGGACTTAGTATAAGAATATTAATTCGTATAGAATTAGGAC  60
```

```
Query  81    AACCTGGAACTTTAATTGGAAATGATCAGATTTATAATGTAATCGTAACAGCACATGCAT  140
             ||||||||||||||||||||||||||||||||||||||||||||||||||||||||||||
Sbjct  61    AACCTGGAACTTTAATTGGAAATGATCAGATTTATAATGTAATCGTAACAGCACATGCAT  120

Query  141   TTATTATAATTTTCTTTATAGTTATACCAATCATAATCGGAGGGTTCGGAAATTGATTAG  200
             ||||||||||||| ||||||||||||||||||||||||||||| || |||||||||||||
Sbjct  121   TTATTATAATTTTTTTTATAGTTATACCAATCATAATCGGAGGATTTGGAAATTGATTAG  180

Query  201   TTCCTATTATATTAGGTGCCCCAGATATAGCATTTCCACGAATAAATAATATAAGATTTT  260
             | ||||| ||||| ||||||||||||||||||||||||||||||||||||||||||||||
Sbjct  181   TCCCTATCATATTGGGTGCCCCAGATATAGCATTTCCACGAATAAATAATATAAGATTTT  240

Query  261   GACTTTTACCCCCCTCATTATTTTTATTGATTAACTCTTCATTAATTGAATCCGGAGCTG  320
             |||||||||| || ||| |||||||||| ||||||||||||||||||||||| |||||||
Sbjct  241   GACTTTTACCTCCTTCACTATTTTTATTAATTAACTCTTCATTAATTGAATCTGGAGCTG  300

Query  321   GAACAGGATGAACAGTTTATCCCCCTCTATCTTCAAATTTATCACATTATGGTCCATCAG  380
             | ||||||||||||||||| || ||||||||||||||||||||||||||||| || ||||
Sbjct  301   GTACAGGATGAACAGTTTACCCTCCTCTATCTTCAAATTTATCACATTATGGCCCTTCAG  360

Query  381   TAGATTTAGCTATTTTTTCTCTTCACCTTGCTGGTGCTTCTTCAATTTTAGGTGCAATTA  440
             ||||||||||||| ||||||||||| ||||||||||||||||||||||||||||||||||
Sbjct  361   TAGATTTAGCTATCTTTTCTCTTCATCTTGCTGGTGCTTCTTCAATTTTAGGTGCAATTA  420

Query  441   ACTTCATCACAACTATTGTAAATATACGATCAATTGGAATAACAATAGAACGAATACCAT  500
             |||| || |||||||||||||| ||||| ||||||||||||||||||||||| |||||||
Sbjct  421   ACTTTATTACAACTATTGTAAACATACGGTCAATTGGAATAACAATAGAACGTATACCAT  480

Query  501   TATTTGTTTGATCTGTTTTAATTACAGCTATTTTACTACTTCTATCTTTACCTGTATTAG  560
             ||||||||||||||||||||||||||||||||||| ||||| ||||||||||||||||||
Sbjct  481   TATTTGTTTGATCTGTTTTAATTACAGCTATTTTATTACTTTTATCTTTACCTGTATTAG  540

Query  561   CAGGTGCCATTACAATATTATTAACTGATCGAAACTTTAACACATCATTTTTTGATCCTT  620
             ||||||||||||||||||| |||||||||||||||||||| |||||||||||||||||||
Sbjct  541   CAGGTGCCATTACAATATTGTTAACTGATCGAAACTTTAATACATCATTTTTTGATCCTT  600

Query  621   CAGGAGGAGGAGATCCCATTTTATATCAACACTTATTT  658
             |||| ||||||||||| |||||||||||||| ||||||
Sbjct  601   CAGGGGGAGGAGATCCAATTTTATATCAACATTTATTT  638
```

>Rhipicephalus sanguineus isolate LIC4736B cytochrome oxidase subunit 1 (COI) gene, partial cds; mitochondrial
Sequence ID: KX383807.1 Length: 763
>Rhipicephalus sanguineus isolate LIC4736C cytochrome oxidase subunit 1 (COI) gene, partial cds; mitochondrial
Sequence ID: KX383808.1 Length: 763
>Rhipicephalus sanguineus isolate LIC4736F cytochrome oxidase subunit 1 (COI) gene, partial cds; mitochondrial
Sequence ID: KX383810.1 Length: 763
>Rhipicephalus sanguineus isolate LIC5535C cytochrome oxidase subunit 1 (COI) gene, partial cds; mitochondrial

Sequence ID: KX383813.1 Length: 763
Range 1: 1 to 637

Score:1011 bits(547), Expect:0.0,
Identities:607/637(95%), Gaps:0/637(0%), Strand: Plus/Plus

```
Query  22   AGCATGATCAGGTATATTAGGACTTAGTATAAGAATATTAATTCGTATAGAATTAGGACA  81
            ||||||||||||||||||||||||||||||||||||||||||||||||||||||||||||
Sbjct  1    AGCATGATCAGGTATATTAGGACTTAGTATAAGAATATTAATTCGTATAGAATTAGGACA  60

Query  82   ACCTGGAACTTTAATTGGAAATGATCAGATTTATAATGTAATCGTAACAGCACATGCATT  141
            ||||||||||||||||||||||||||||||||||||||||||||||||||||||||||||
Sbjct  61   ACCTGGAACTTTAATTGGAAATGATCAGATTTATAATGTAATCGTAACAGCACATGCATT  120

Query  142  TATTATAATTTTCTTTATAGTTATACCAATCATAATCGGAGGGTTCGGAAATTGATTAGT  201
            |||||||||||| ||||||||||||||||||||||||||||| || ||||||||||||||
Sbjct  121  TATTATAATTTTTTTTATAGTTATACCAATCATAATCGGAGGATTTGGAAATTGATTAGT  180

Query  202  TCCTATTATATTAGGTGCCCCAGATATAGCATTTCCACGAATAAATAATATAAGATTTTG  261
             ||||| ||||| |||||||||||||||||||||||||||||||||||||||||||||||
Sbjct  181  CCCTATCATATTGGGTGCCCCAGATATAGCATTTCCACGAATAAATAATATAAGATTTTG  240

Query  262  ACTTTTACCCCCCTCATTATTTTTATTGATTAACTCTTCATTAATTGAATCCGGAGCTGG  321
            ||||||||| || ||| |||||||||| ||||||||||||||||||||||| ||||||||
Sbjct  241  ACTTTTACCTCCTTCACTATTTTTATTAATTAACTCTTCATTAATTGAATCTGGAGCTGG  300

Query  322  AACAGGATGAACAGTTTATCCCCCTCTATCTTCAAATTTATCACATTATGGTCCATCAGT  381
             ||||||||||||||||| || ||||||||||||||||||||||||||||| || |||||
Sbjct  301  TACAGGATGAACAGTTTACCCTCCTCTATCTTCAAATTTATCACATTATGGCCCTTCAGT  360

Query  382  AGATTTAGCTATTTTTTCTCTTCACCTTGCTGGTGCTTCTTCAATTTTAGGTGCAATTAA  441
            |||||||||||| ||||||||||| |||||||||||||||||||||||||||||||||||
Sbjct  361  AGATTTAGCTATCTTTTCTCTTCATCTTGCTGGTGCTTCTTCAATTTTAGGTGCAATTAA  420

Query  442  CTTCATCACAACTATTGTAAATATACGATCAATTGGAATAACAATAGAACGAATACCATT  501
            ||| || |||||||||||||| ||||| ||||||||||||||||||||||| ||||||||
Sbjct  421  CTTTATTACAACTATTGTAAACATACGGTCAATTGGAATAACAATAGAACGTATACCATT  480

Query  502  ATTTGTTTGATCTGTTTTAATTACAGCTATTTTACTACTTCTATCTTTACCTGTATTAGC  561
            |||||||||||||||||||||||||||||||||| ||||| |||||||||||||||||||
Sbjct  481  ATTTGTTTGATCTGTTTTAATTACAGCTATTTTATTACTTTTATCTTTACCTGTATTAGC  540

Query  562  AGGTGCCATTACAATATTATTAACTGATCGAAACTTTAACACATCATTTTTTGATCCTTC  621
            |||||||||||||||||| ||||||||||||||||||||| ||||||||||||||||||||
Sbjct  541  AGGTGCCATTACAATATTGTTAACTGATCGAAACTTTAATACATCATTTTTTGATCCTTC  600

Query  622  AGGAGGAGGAGATCCCATTTTATATCAACACTTATTT  658
            ||| ||||||||||| |||||||||||||| ||||||
Sbjct  601  AGGGGGAGGAGATCCAATTTTATATCAACATTTATTT  637
```

>Rhipicephalus sanguineus voucher Rs7 cytochrome oxidase subunit I (COI) gene,

partial cds; mitochondrial
Sequence ID: KU568516.1 Length: 656
Range 1: 27 to 656

Score:1009 bits(546), Expect:0.0,
Identities:602/630(96%), Gaps:0/630(0%), Strand: Plus/Plus

```
Query  1     AACAATATATTTAATTTTTGGAGCATGATCAGGTATATTAGGACTTAGTATAAGAATATT  60
             ||||||||||||||||||||||||||||||||||||||||||||||||||||||||||||
Sbjct  27    AACAATATATTTAATTTTTGGAGCATGATCAGGTATATTAGGACTTAGTATAAGAATATT  86

Query  61    AATTCGTATAGAATTAGGACAACCTGGAACTTTAATTGGAAATGATCAGATTTATAATGT  120
             ||||||||||||||||||||||||||||||||||||||||||||||||||||||||||||
Sbjct  87    AATTCGTATAGAATTAGGACAACCTGGAACTTTAATTGGAAATGATCAGATTTATAATGT  146

Query  121   AATCGTAACAGCACATGCATTTATTATAATTTTCTTTATAGTTATACCAATCATAATCGG  180
             ||||||||||||||||||||||||||||||||| ||||||||||||||||||||||||||
Sbjct  147   AATCGTAACAGCACATGCATTTATTATAATTTTTTTTATAGTTATACCAATCATAATCGG  206

Query  181   AGGGTTCGGAAATTGATTAGTTCCTATTATATTAGGTGCCCCAGATATAGCATTTCCACG  240
             ||| || |||||||||||| | ||||| ||||||||||||||||||||||||||||||||
Sbjct  207   AGGATTTGGAAATTGATTAATCCCTATCATATTAGGTGCCCCAGATATAGCATTTCCACG  266

Query  241   AATAAATAATATAAGATTTTGACTTTTACCCCCCTCATTATTTTTATTGATTAACTCTTC  300
             |||||||||||||||||||||||||||||| || ||| |||||||||| |||||||||||
Sbjct  267   AATAAATAATATAAGATTTTGACTTTTACCTCCTTCACTATTTTTATTAATTAACTCTTC  326

Query  301   ATTAATTGAATCCGGAGCTGGAACAGGATGAACAGTTTATCCCCCTCTATCTTCAAATTT  360
             |||||||||||| |||||||| ||||||||||||||||| || |||||||||||||||||
Sbjct  327   ATTAATTGAATCTGGAGCTGGTACAGGATGAACAGTTTACCCTCCTCTATCTTCAAATTT  386

Query  361   ATCACATTATGGTCCATCAGTAGATTTAGCTATTTTTTCTCTTCACCTTGCTGGTGCTTC  420
             |||||||||||| || ||||||||||||||||| ||||||||||| ||||||||||||||
Sbjct  387   ATCACATTATGGCCCTTCAGTAGATTTAGCTATCTTTTCTCTTCATCTTGCTGGTGCTTC  446

Query  421   TTCAATTTTAGGTGCAATTAACTTCATCACAACTATTGTAAATATACGATCAATTGGAAT  480
             |||||||||||||||||||||||| || |||||||||||||| |||||||||||||||||
Sbjct  447   TTCAATTTTAGGTGCAATTAACTTTATTACAACTATTGTAAACATACGATCAATTGGAAT  506

Query  481   AACAATAGAACGAATACCATTATTTGTTTGATCTGTTTTAATTACAGCTATTTTACTACT  540
             |||||||||||| |||||||||||||||||||||||||||||||||||||||||| ||||
Sbjct  507   AACAATAGAACGTATACCATTATTTGTTTGATCTGTTTTAATTACAGCTATTTTATTACT  566

Query  541   TCTATCTTTACCTGTATTAGCAGGTGCCATTACAATATTATTAACTGATCGAAACTTTAA  600
               ||||||||||||||||||||||||||||||||||||| ||||||||||||||||||||
Sbjct  567   CTTATCTTTACCTGTATTAGCAGGTGCCATTACAATATTGTTAACTGATCGAAACTTTAA  626

Query  601   CACATCATTTTTTGATCCTTCAGGAGGAGG  630
              |||||||||||||| ||||||||||||||
Sbjct  627   TACATCATTTTTTGACCCTTCAGGAGGAGG  656
```

>Rhipicephalus sanguineus isolate LIC4736D cytochrome oxidase subunit 1 (COI) gene, partial cds; mitochondrial
Sequence ID: KX383809.1 Length: 762
Range 1: 1 to 636

Score:1009 bits(546), Expect:0.0,
Identities:606/636(95%), Gaps:0/636(0%), Strand: Plus/Plus

```
Query  23   GCATGATCAGGTATATTAGGACTTAGTATAAGAATATTAATTCGTATAGAATTAGGACAA  82
            ||||||||||||||||||||||||||||||||||||||||||||||||||||||||||||
Sbjct  1    GCATGATCAGGTATATTAGGACTTAGTATAAGAATATTAATTCGTATAGAATTAGGACAA  60

Query  83   CCTGGAACTTTAATTGGAAATGATCAGATTTATAATGTAATCGTAACAGCACATGCATTT  142
            ||||||||||||||||||||||||||||||||||||||||||||||||||||||||||||
Sbjct  61   CCTGGAACTTTAATTGGAAATGATCAGATTTATAATGTAATCGTAACAGCACATGCATTT  120

Query  143  ATTATAATTTTCTTTATAGTTATACCAATCATAATCGGAGGGTTCGGAAATTGATTAGTT  202
            ||||||||||| ||||||||||||||||||||||||||||| || |||||||||||||| 
Sbjct  121  ATTATAATTTTTTTTATAGTTATACCAATCATAATCGGAGGATTTGGAAATTGATTAGTC  180

Query  203  CCTATTATATTAGGTGCCCCAGATATAGCATTTCCACGAATAAATAATATAAGATTTTGA  262
            ||||| ||||| ||||||||||||||||||||||||||||||||||||||||||||||||
Sbjct  181  CCTATCATATTGGGTGCCCCAGATATAGCATTTCCACGAATAAATAATATAAGATTTTGA  240

Query  263  CTTTTACCCCCCTCATTATTTTTATTGATTAACTCTTCATTAATTGAATCCGGAGCTGGA  322
            |||||||| || ||| |||||||||| ||||||||||||||||||||||| |||||||| 
Sbjct  241  CTTTTACCTCCTTCACTATTTTTATTAATTAACTCTTCATTAATTGAATCTGGAGCTGGT  300

Query  323  ACAGGATGAACAGTTTATCCCCCTCTATCTTCAAATTTATCACATTATGGTCCATCAGTA  382
            ||||||||||||||||| || ||||||||||||||||||||||||||||| || ||||||
Sbjct  301  ACAGGATGAACAGTTTACCCTCCTCTATCTTCAAATTTATCACATTATGGCCCTTCAGTA  360

Query  383  GATTTAGCTATTTTTTCTCTTCACCTTGCTGGTGCTTCTTCAATTTTAGGTGCAATTAAC  442
            ||||||||||| ||||||||||| ||||||||||||||||||||||||||||||||||||
Sbjct  361  GATTTAGCTATCTTTTCTCTTCATCTTGCTGGTGCTTCTTCAATTTTAGGTGCAATTAAC  420

Query  443  TTCATCACAACTATTGTAAATATACGATCAATTGGAATAACAATAGAACGAATACCATTA  502
            || || |||||||||||||| ||||| ||||||||||||||||||||||| |||||||||
Sbjct  421  TTTATTACAACTATTGTAAACATACGGTCAATTGGAATAACAATAGAACGTATACCATTA  480

Query  503  TTTGTTTGATCTGTTTTAATTACAGCTATTTTACTACTTCTATCTTTACCTGTATTAGCA  562
            ||||||||||||||||||||||||||||||||| ||||| ||||||||||||||||||||
Sbjct  481  TTTGTTTGATCTGTTTTAATTACAGCTATTTTATTACTTTTATCTTTACCTGTATTAGCA  540

Query  563  GGTGCCATTACAATATTATTAACTGATCGAAACTTTAACACATCATTTTTTGATCCTTCA  622
            ||||||||||||||||| |||||||||||||||||||| |||||||||||||||||||||
Sbjct  541  GGTGCCATTACAATATTGTTAACTGATCGAAACTTTAATACATCATTTTTTGATCCTTCA  600

Query  623  GGAGGAGGAGATCCCATTTTATATCAACACTTATTT  658
            || ||||||||||| |||||||||||||| ||||||
Sbjct  601  GGGGGAGGAGATCCAATTTTATATCAACATTTATTT  636
```

```
>Rhipicephalus sanguineus isolate LIC4727D cytochrome oxidase subunit 1 (COI)
gene, partial cds; mitochondrial
Sequence ID: KX383806.1 Length: 714
Range 1: 1 to 636

Score:1009 bits(546), Expect:0.0,
Identities:606/636(95%),  Gaps:0/636(0%), Strand: Plus/Plus

Query  23    GCATGATCAGGTATATTAGGACTTAGTATAAGAATATTAATTCGTATAGAATTAGGACAA  82
             ||||||||||||||||||||||||||||||||||||||||||||||||||||||||||||
Sbjct  1     GCATGATCAGGTATATTAGGACTTAGTATAAGAATATTAATTCGTATAGAATTAGGACAA  60

Query  83    CCTGGAACTTTAATTGGAAATGATCAGATTTATAATGTAATCGTAACAGCACATGCATTT  142
             ||||||||||||||||||||||||||||||||||||||||||||||||||||||||||||
Sbjct  61    CCTGGAACTTTAATTGGAAATGATCAGATTTATAATGTAATCGTAACAGCACATGCATTT  120

Query  143   ATTATAATTTTCTTTATAGTTATACCAATCATAATCGGAGGGTTCGGAAATTGATTAGTT  202
             ||||||||||| ||||||||||||||||||||||||||||| || |||||||||||||| 
Sbjct  121   ATTATAATTTTTTTTATAGTTATACCAATCATAATCGGAGGATTTGGAAATTGATTAGTC  180

Query  203   CCTATTATATTAGGTGCCCCAGATATAGCATTTCCACGAATAAATAATATAAGATTTTGA  262
             ||||| ||||| ||||||||||||||||||||||||||||||||||||||||||||||||
Sbjct  181   CCTATCATATTGGGTGCCCCAGATATAGCATTTCCACGAATAAATAATATAAGATTTTGA  240

Query  263   CTTTTACCCCCCTCATTATTTTTATTGATTAACTCTTCATTAATTGAATCCGGAGCTGGA  322
             |||||||| || ||| |||||||||| ||||||||||||||||||||||| |||||||| 
Sbjct  241   CTTTTACCTCCTTCACTATTTTTATTAATTAACTCTTCATTAATTGAATCTGGAGCTGGT  300

Query  323   ACAGGATGAACAGTTTATCCCCCTCTATCTTCAAATTTATCACATTATGGTCCATCAGTA  382
             ||||||||||||||||| || ||||||||||||||||||||||||||||| || ||||||
Sbjct  301   ACAGGATGAACAGTTTACCCTCCTCTATCTTCAAATTTATCACATTATGGCCCTTCAGTA  360

Query  383   GATTTAGCTATTTTTTCTCTTCACCTTGCTGGTGCTTCTTCAATTTTAGGTGCAATTAAC  442
             ||||||||||| ||||||||||| ||||||||||||||||||||||||||||||||||||
Sbjct  361   GATTTAGCTATCTTTTCTCTTCATCTTGCTGGTGCTTCTTCAATTTTAGGTGCAATTAAC  420

Query  443   TTCATCACAACTATTGTAAATATACGATCAATTGGAATAACAATAGAACGAATACCATTA  502
             || || |||||||||||||| ||||| ||||||||||||||||||||||| |||||||||
Sbjct  421   TTTATTACAACTATTGTAAACATACGGTCAATTGGAATAACAATAGAACGTATACCATTA  480

Query  503   TTTGTTTGATCTGTTTTAATTACAGCTATTTTACTACTTCTATCTTTACCTGTATTAGCA  562
             ||||||||||||||||||||||||||||||||| ||||| ||||||||||||||||||||
Sbjct  481   TTTGTTTGATCTGTTTTAATTACAGCTATTTTATTACTTTTATCTTTACCTGTATTAGCA  540

Query  563   GGTGCCATTACAATATTATTAACTGATCGAAACTTTAACACATCATTTTTTGATCCTTCA  622
             ||||||||||||||||| |||||||||||||||||||| |||||||||||||||||||||
Sbjct  541   GGTGCCATTACAATATTGTTAACTGATCGAAACTTTAATACATCATTTTTTGATCCTTCA  600

Query  623   GGAGGAGGAGATCCCATTTTATATCAACACTTATTT  658
             || ||||||||||| |||||||||||||| ||||||
Sbjct  601   GGGGGAGGAGATCCAATTTTATATCAACATTTATTT  636
```

>Rhipicephalus sanguineus isolate LIC4727C cytochrome oxidase subunit 1 (COI) gene, partial cds; mitochondrial
Sequence ID: KX383805.1 Length: 761
Range 1: 1 to 635

Score:1007 bits(545), Expect:0.0,
Identities:605/635(95%), Gaps:0/635(0%), Strand: Plus/Plus

```
Query  24   CATGATCAGGTATATTAGGACTTAGTATAAGAATATTAATTCGTATAGAATTAGGACAAC  83
            ||||||||||||||||||||||||||||||||||||||||||||||||||||||||||||
Sbjct  1    CATGATCAGGTATATTAGGACTTAGTATAAGAATATTAATTCGTATAGAATTAGGACAAC  60

Query  84   CTGGAACTTTAATTGGAAATGATCAGATTTATAATGTAATCGTAACAGCACATGCATTTA  143
            ||||||||||||||||||||||||||||||||||||||||||||||||||||||||||||
Sbjct  61   CTGGAACTTTAATTGGAAATGATCAGATTTATAATGTAATCGTAACAGCACATGCATTTA  120

Query  144  TTATAATTTTCTTTATAGTTATACCAATCATAATCGGAGGGTTCGGAAATTGATTAGTTC  203
            |||||||||| ||||||||||||||||||||||||||||| || |||||||||||||| |
Sbjct  121  TTATAATTTTTTTTATAGTTATACCAATCATAATCGGAGGATTTGGAAATTGATTAGTCC  180

Query  204  CTATTATATTAGGTGCCCCAGATATAGCATTTCCACGAATAAATAATATAAGATTTTGAC  263
            |||| ||||| ||||||||||||||||||||||||||||||||||||||||||||||||||
Sbjct  181  CTATCATATTGGGTGCCCCAGATATAGCATTTCCACGAATAAATAATATAAGATTTTGAC  240

Query  264  TTTTACCCCCCTCATTATTTTTATTGATTAACTCTTCATTAATTGAATCCGGAGCTGGAA  323
            ||||||| || ||| |||||||||| ||||||||||||||||||||||| |||||||| |
Sbjct  241  TTTTACCTCCTTCACTATTTTTATTAATTAACTCTTCATTAATTGAATCTGGAGCTGGTA  300

Query  324  CAGGATGAACAGTTTATCCCCCTCTATCTTCAAATTTATCACATTATGGTCCATCAGTAG  383
            |||||||||||||||| || ||||||||||||||||||||||||||||| || |||||||
Sbjct  301  CAGGATGAACAGTTTACCCTCCTCTATCTTCAAATTTATCACATTATGGCCCTTCAGTAG  360

Query  384  ATTTAGCTATTTTTTCTCTTCACCTTGCTGGTGCTTCTTCAATTTTAGGTGCAATTAACT  443
            |||||||||| ||||||||||| |||||||||||||||||||||||||||||||||||||
Sbjct  361  ATTTAGCTATCTTTTCTCTTCATCTTGCTGGTGCTTCTTCAATTTTAGGTGCAATTAACT  420

Query  444  TCATCACAACTATTGTAAATATACGATCAATTGGAATAACAATAGAACGAATACCATTAT  503
            | || |||||||||||||| ||||| ||||||||||||||||||||||| ||||||||||
Sbjct  421  TTATTACAACTATTGTAAACATACGGTCAATTGGAATAACAATAGAACGTATACCATTAT  480

Query  504  TTGTTTGATCTGTTTTAATTACAGCTATTTTACTACTTCTATCTTTACCTGTATTAGCAG  563
            |||||||||||||||||||||||||||||||| ||||| |||||||||||||||||||||
Sbjct  481  TTGTTTGATCTGTTTTAATTACAGCTATTTTATTACTTTTATCTTTACCTGTATTAGCAG  540

Query  564  GTGCCATTACAATATTATTAACTGATCGAAACTTTAACACATCATTTTTTGATCCTTCAG  623
            |||||||||||||||| |||||||||||||||||||| ||||||||||||||||||||||
Sbjct  541  GTGCCATTACAATATTGTTAACTGATCGAAACTTTAATACATCATTTTTTGATCCTTCAG  600

Query  624  GAGGAGGAGATCCCATTTTATATCAACACTTATTT  658
            | ||||||||||| |||||||||||||| ||||||
Sbjct  601  GGGGAGGAGATCCAATTTTATATCAACATTTATTT  635
```

```
>Rhipicephalus sanguineus isolate Samont2 cytochrome oxidase subunit I (cox1)
gene, partial cds; mitochondrial
Sequence ID: MH630347.1 Length: 644
Range 1: 20 to 641

Score:1005 bits(544), Expect:0.0,
Identities:596/622(96%),  Gaps:0/622(0%), Strand: Plus/Plus

Query  1    AACAATATATTTAATTTTTGGAGCATGATCAGGTATATTAGGACTTAGTATAAGAATATT  60
            ||||||||||||||||||||||||||||||||||||||||||||||||||||||||||||
Sbjct  20   AACAATATATTTAATTTTTGGAGCATGATCAGGTATATTAGGACTTAGTATAAGAATATT  79

Query  61   AATTCGTATAGAATTAGGACAACCTGGAACTTTAATTGGAAATGATCAGATTTATAATGT  120
            |||| |||||||||||||||||||||||||||||||||||||||||||||||||||||||
Sbjct  80   AATTGGTATAGAATTAGGACAACCTGGAACTTTAATTGGAAATGATCAGATTTATAATGT  139

Query  121  AATCGTAACAGCACATGCATTTATTATAATTTTCTTTATAGTTATACCAATCATAATCGG  180
            ||||||||||||||||||||||||||||||||| ||||||||||||||||||||||||||
Sbjct  140  AATCGTAACAGCACATGCATTTATTATAATTTTTTTTATAGTTATACCAATCATAATCGG  199

Query  181  AGGGTTCGGAAATTGATTAGTTCCTATTATATTAGGTGCCCCAGATATAGCATTTCCACG  240
            ||| || |||||||||||||| ||||| ||||| ||||||||||||||||||||||||||
Sbjct  200  AGGATTTGGAAATTGATTAGTCCCTATCATATTGGGTGCCCCAGATATAGCATTTCCACG  259

Query  241  AATAAATAATATAAGATTTTGACTTTTACCCCCCTCATTATTTTTATTGATTAACTCTTC  300
            |||||||||||||||||||||||||||||| || ||| |||||||||| |||||||||||
Sbjct  260  AATAAATAATATAAGATTTTGACTTTTACCTCCTTCACTATTTTTATTAATTAACTCTTC  319

Query  301  ATTAATTGAATCCGGAGCTGGAACAGGATGAACAGTTTATCCCCCTCTATCTTCAAATTT  360
            |||||||||||| |||||||| ||||||||||||||||| || |||||||||||||||||
Sbjct  320  ATTAATTGAATCTGGAGCTGGTACAGGATGAACAGTTTACCCTCCTCTATCTTCAAATTT  379

Query  361  ATCACATTATGGTCCATCAGTAGATTTAGCTATTTTTTCTCTTCACCTTGCTGGTGCTTC  420
            |||||||||||| || ||||||||||||||||||||||||||||| ||||||||||||||
Sbjct  380  ATCACATTATGGCCCTTCAGTAGATTTAGCTATTTTTTCTCTTCATCTTGCTGGTGCTTC  439

Query  421  TTCAATTTTAGGTGCAATTAACTTCATCACAACTATTGTAAATATACGATCAATTGGAAT  480
            |||||||||||||||||||||||| || |||||||||||||| |||||||||||||||||
Sbjct  440  TTCAATTTTAGGTGCAATTAACTTTATTACAACTATTGTAAACATACGATCAATTGGAAT  499

Query  481  AACAATAGAACGAATACCATTATTTGTTTGATCTGTTTTAATTACAGCTATTTTACTACT  540
            |||||||||||| |||||||||||||||||||||||||||||||||||||||||| ||||
Sbjct  500  AACAATAGAACGTATACCATTATTTGTTTGATCTGTTTTAATTACAGCTATTTTATTACT  559

Query  541  TCTATCTTTACCTGTATTAGCAGGTGCCATTACAATATTATTAACTGATCGAAACTTTAA  600
            | ||||||||||||||||||||||||||||||||||||| ||||||||||||||||||||
Sbjct  560  TTTATCTTTACCTGTATTAGCAGGTGCCATTACAATATTGTTAACTGATCGAAACTTTAA  619

Query  601  CACATCATTTTTTGATCCTTCA  622
             |||||||||||||||||||||
Sbjct  620  TACATCATTTTTTGATCCTTCA  641
```

>Rhipicephalus sanguineus isolate Samont1 cytochrome oxidase subunit I (cox1) gene, partial cds; mitochondrial
Sequence ID: MH630346.1 Length: 644
Range 1: 20 to 641

Score:1005 bits(544), Expect:0.0,
Identities:596/622(96%), Gaps:0/622(0%), Strand: Plus/Plus

```
Query  1    AACAATATATTTAATTTTTGGAGCATGATCAGGTATATTAGGACTTAGTATAAGAATATT  60
            ||||||||||||||||||||||||||||||||||||||||||||||||||||||||||||
Sbjct  20   AACAATATATTTAATTTTTGGAGCATGATCAGGTATATTAGGACTTAGTATAAGAATATT  79

Query  61   AATTCGTATAGAATTAGGACAACCTGGAACTTTAATTGGAAATGATCAGATTTATAATGT  120
            |||| |||||||||||||||||||||||||||||||||||||||||||||||||||||||
Sbjct  80   AATTGGTATAGAATTAGGACAACCTGGAACTTTAATTGGAAATGATCAGATTTATAATGT  139

Query  121  AATCGTAACAGCACATGCATTTATTATAATTTTCTTTATAGTTATACCAATCATAATCGG  180
            ||||||||||||||||||||||||||||||||| ||||||||||||||||||||||||||
Sbjct  140  AATCGTAACAGCACATGCATTTATTATAATTTTTTTTATAGTTATACCAATCATAATCGG  199

Query  181  AGGGTTCGGAAATTGATTAGTTCCTATTATATTAGGTGCCCCAGATATAGCATTTCCACG  240
            ||| || |||||||||||||| ||||| ||||| ||||||||||||||||||||||||||
Sbjct  200  AGGATTTGGAAATTGATTAGTCCCTATCATATTGGGTGCCCCAGATATAGCATTTCCACG  259

Query  241  AATAAATAATATAAGATTTTGACTTTTACCCCCCTCATTATTTTTATTGATTAACTCTTC  300
            |||||||||||||||||||||||||||||| || ||| |||||||||| |||||||||||
Sbjct  260  AATAAATAATATAAGATTTTGACTTTTACCTCCTTCACTATTTTTATTAATTAACTCTTC  319

Query  301  ATTAATTGAATCCGGAGCTGGAACAGGATGAACAGTTTATCCCCCTCTATCTTCAAATTT  360
            |||||||||||| |||||||| ||||||||||||||||| || |||||||||||||||||
Sbjct  320  ATTAATTGAATCTGGAGCTGGTACAGGATGAACAGTTTACCCTCCTCTATCTTCAAATTT  379

Query  361  ATCACATTATGGTCCATCAGTAGATTTAGCTATTTTTTCTCTTCACCTTGCTGGTGCTTC  420
            |||||||||||| || ||||||||||||||||||||||||||||| ||||||||||||||
Sbjct  380  ATCACATTATGGCCCTTCAGTAGATTTAGCTATTTTTTCTCTTCATCTTGCTGGTGCTTC  439

Query  421  TTCAATTTTAGGTGCAATTAACTTCATCACAACTATTGTAAATATACGATCAATTGGAAT  480
            |||||||||||||||||||||||| || ||||||||||||||| ||||||||||||||||
Sbjct  440  TTCAATTTTAGGTGCAATTAACTTTATTACAACTATTGTAAACATACGATCAATTGGAAT  499

Query  481  AACAATAGAACGAATACCATTATTTGTTTGATCTGTTTTAATTACAGCTATTTTACTACT  540
            |||||||||||| |||||||||||||||||||||||||||||||||||||||||| ||||
Sbjct  500  AACAATAGAACGTATACCATTATTTGTTTGATCTGTTTTAATTACAGCTATTTTATTACT  559

Query  541  TCTATCTTTACCTGTATTAGCAGGTGCCATTACAATATTATTAACTGATCGAAACTTTAA  600
            | ||||||||||||||||||||||||||||||||||||||| ||||||||||||||||||
Sbjct  560  TTTATCTTTACCTGTATTAGCAGGTGCCATTACAATATTGTTAACTGATCGAAACTTTAA  619

Query  601  CACATCATTTTTTGATCCTTCA  622
             ||||||||||||||||||||||
```

```
Sbjct  620  TACATCATTTTTTGATCCTTCA  641
```

>Rhipicephalus sanguineus clone #7 cytochrome oxidase subunit I (COI) gene, partial cds; mitochondrial
Sequence ID: KX519712.1 Length: 654
Range 1: 3 to 628

Score:1002 bits(542), Expect:0.0,
Identities:598/626(96%), Gaps:0/626(0%), Strand: Plus/Plus

```
Query  33   GTATATTAGGACTTAGTATAAGAATATTAATTCGTATAGAATTAGGACAACCTGGAACTT  92
            ||||||||||||||||||||||||||||||||||||||||||||||||||||||||||||
Sbjct  3    GTATATTAGGACTTAGTATAAGAATATTAATTCGTATAGAATTAGGACAACCTGGAACTT  62

Query  93   TAATTGGAAATGATCAGATTTATAATGTAATCGTAACAGCACATGCATTTATTATAATTT  152
            ||||||||||||||||||||||||||||||||||||||||||||||||||||||||||||
Sbjct  63   TAATTGGAAATGATCAGATTTATAATGTAATCGTAACAGCACATGCATTTATTATAATTT  122

Query  153  TCTTTATAGTTATACCAATCATAATCGGAGGGTTCGGAAATTGATTAGTTCCTATTATAT  212
            | ||||||||||||||||||||||||||||| || |||||||||||||| ||||| ||||
Sbjct  123  TTTTTATAGTTATACCAATCATAATCGGAGGATTTGGAAATTGATTAGTCCCTATCATAT  182

Query  213  TAGGTGCCCCAGATATAGCATTTCCACGAATAAATAATATAAGATTTTGACTTTTACCCC  272
            | |||||||||||||||||||||||||||||||||||||||||||||||||||||||| |
Sbjct  183  TGGGTGCCCCAGATATAGCATTTCCACGAATAAATAATATAAGATTTTGACTTTTACCTC  242

Query  273  CCTCATTATTTTTATTGATTAACTCTTCATTAATTGAATCCGGAGCTGGAACAGGATGAA  332
            | ||| |||||||||| ||||||||||||||||||||||| |||||||| ||||||||||
Sbjct  243  CTTCACTATTTTTATTAATTAACTCTTCATTAATTGAATCTGGAGCTGGTACAGGATGAA  302

Query  333  CAGTTTATCCCCCTCTATCTTCAAATTTATCACATTATGGTCCATCAGTAGATTTAGCTA  392
            ||||||| || ||||||||||||||||||||||||||||| || ||||||||||||||||
Sbjct  303  CAGTTTACCCTCCTCTATCTTCAAATTTATCACATTATGGCCCTTCAGTAGATTTAGCTA  362

Query  393  TTTTTTCTCTTCACCTTGCTGGTGCTTCTTCAATTTTAGGTGCAATTAACTTCATCACAA  452
            ||||||||||||| |||||||||||||||||||||||||||||||||||||||| || ||||
Sbjct  363  TTTTTTCTCTTCATCTTGCTGGTGCTTCTTCAATTTTAGGTGCAATTAACTTTATTACAA  422

Query  453  CTATTGTAAATATACGATCAATTGGAATAACAATAGAACGAATACCATTATTTGTTTGAT  512
            |||||||||| ||||||||||||||||||||||||||||| |||||||||||||||||||
Sbjct  423  CTATTGTAAACATACGATCAATTGGAATAACAATAGAACGTATACCATTATTTGTTTGAT  482

Query  513  CTGTTTTAATTACAGCTATTTTACTACTTCTATCTTTACCTGTATTAGCAGGTGCCATTA  572
            ||||||||||||||||||||||| ||||| ||||||||||||||||||||||||||||||
Sbjct  483  CTGTTTTAATTACAGCTATTTTATTACTTTTATCTTTACCTGTATTAGCAGGTGCCATTA  542

Query  573  CAATATTATTAACTGATCGAAACTTTAACACATCATTTTTTGATCCTTCAGGAGGAGGAG  632
            ||||||| |||||||||||||||||||| ||||||||||||||||||||||| |||||||
Sbjct  543  CAATATTGTTAACTGATCGAAACTTTAATACATCATTTTTTGATCCTTCAGGGGGAGGAG  602

Query  633  ATCCCATTTTATATCAACACTTATTT  658
```

```
                |||| |||||||||||||| ||||||
Sbjct  603  ATCCAATTTTATATCAACATTTATTT  628
```

>Rhipicephalus sanguineus sensu lato 'temperate lineage' isolate CR1575 cytochrome c oxidase subunit I (COI) gene, partial cds; mitochondrial
Sequence ID: MF426002.1 Length: 639
Range 1: 1 to 628

Score:1000 bits(541), Expect:0.0,
Identities:599/628(95%), Gaps:0/628(0%), Strand: Plus/Plus

```
Query  31   AGGTATATTAGGACTTAGTATAAGAATATTAATTCGTATAGAATTAGGACAACCTGGAAC  90
            ||||||||||||||||||||||||||||||||||||||||||||||||||||||||||||
Sbjct  1    AGGTATATTAGGACTTAGTATAAGAATATTAATTCGTATAGAATTAGGACAACCTGGAAC  60

Query  91   TTTAATTGGAAATGATCAGATTTATAATGTAATCGTAACAGCACATGCATTTATTATAAT  150
            ||||||||||||||||||||||||||||||||||||||||||||||||||||||||||||
Sbjct  61   TTTAATTGGAAATGATCAGATTTATAATGTAATCGTAACAGCACATGCATTTATTATAAT  120

Query  151  TTTCTTTATAGTTATACCAATCATAATCGGAGGGTTCGGAAATTGATTAGTTCCTATTAT  210
            ||| ||||||||||||||||||||||||||||| || |||||||||||||| ||||| ||
Sbjct  121  TTTTTTTATAGTTATACCAATCATAATCGGAGGATTTGGAAATTGATTAGTCCCTATCAT  180

Query  211  ATTAGGTGCCCCAGATATAGCATTTCCACGAATAAATAATATAAGATTTTGACTTTTACC  270
            ||| ||||||||||||||||||||||||||||||||||||||||||||||||||||||||
Sbjct  181  ATTGGGTGCCCCAGATATAGCATTTCCACGAATAAATAATATAAGATTTTGACTTTTACC  240

Query  271  CCCCTCATTATTTTTATTGATTAACTCTTCATTAATTGAATCCGGAGCTGGAACAGGATG  330
             || ||| |||||||||| ||||||||||||||||||||||| |||||||| ||||||||
Sbjct  241  TCCTTCACTATTTTTATTAATTAACTCTTCATTAATTGAATCTGGAGCTGGTACAGGATG  300

Query  331  AACAGTTTATCCCCCTCTATCTTCAAATTTATCACATTATGGTCCATCAGTAGATTTAGC  390
            ||||||||| || ||||||||||||||||||||||||||||| || ||||||||||||||
Sbjct  301  AACAGTTTACCCTCCTCTATCTTCAAATTTATCACATTATGGCCCTTCAGTAGATTTAGC  360

Query  391  TATTTTTTCTCTTCACCTTGCTGGTGCTTCTTCAATTTTAGGTGCAATTAACTTCATCAC  450
            ||| ||||||||||| |||||||||||||||||||||||||||||||||||||| || ||
Sbjct  361  TATCTTTTCTCTTCATCTTGCTGGTGCTTCTTCAATTTTAGGTGCAATTAACTTTATTAC  420

Query  451  AACTATTGTAAATATACGATCAATTGGAATAACAATAGAACGAATACCATTATTTGTTTG  510
            |||||||||||| ||||||||||||||||||||||||||||| |||||||||||||||||
Sbjct  421  AACTATTGTAAACATACGATCAATTGGAATAACAATAGAACGTATACCATTATTTGTTTG  480

Query  511  ATCTGTTTTAATTACAGCTATTTTACTACTTCTATCTTTACCTGTATTAGCAGGTGCCAT  570
            ||||||||||||||||||||||||| ||||| ||||||||||||||||||||||||||||
Sbjct  481  ATCTGTTTTAATTACAGCTATTTTATTACTTTTATCTTTACCTGTATTAGCAGGTGCCAT  540

Query  571  TACAATATTATTAACTGATCGAAACTTTAACACATCATTTTTTGATCCTTCAGGAGGAGG  630
            ||||||||| |||||||||||||||||||| ||||||||||||||||||||||| |||||
Sbjct  541  TACAATATTGTTAACTGATCGAAACTTTAATACATCATTTTTTGATCCTTCAGGGGGAGG  600
```

```
Query  631  AGATCCCATTTTATATCAACACTTATTT  658
            |||||| |||||||||||||| ||||||
Sbjct  601  AGATCCAATTTTATATCAACATTTATTT  628
```

>Rhipicephalus sanguineus isolate Rsan-ML4 cytochrome oxidase subunit I (cox1) gene, partial cds; mitochondrial
Sequence ID: MG855658.1 Length: 630
Range 1: 1 to 620

Score:990 bits(536), Expect:0.0,
Identities:592/620(95%), Gaps:0/620(0%), Strand: Plus/Plus

```
Query  39   TAGGACTTAGTATAAGAATATTAATTCGTATAGAATTAGGACAACCTGGAACTTTAATTG  98
            ||||||||||||||||||||||||||||||||||||||||||||||||||||||||||||
Sbjct  1    TAGGACTTAGTATAAGAATATTAATTCGTATAGAATTAGGACAACCTGGAACTTTAATTG  60

Query  99   GAAATGATCAGATTTATAATGTAATCGTAACAGCACATGCATTTATTATAATTTTCTTTA  158
            ||||||||||||||||||||||||||||||||||||||||||||||||||||||| ||||
Sbjct  61   GAAATGATCAGATTTATAATGTAATCGTAACAGCACATGCATTTATTATAATTTTTTTTA  120

Query  159  TAGTTATACCAATCATAATCGGAGGGTTCGGAAATTGATTAGTTCCTATTATATTAGGTG  218
            ||||||||||||||||||||||||| || |||||||||||||| ||||| ||||| ||||
Sbjct  121  TAGTTATACCAATCATAATCGGAGGATTTGGAAATTGATTAGTCCCTATCATATTGGGTG  180

Query  219  CCCCAGATATAGCATTTCCACGAATAAATAATATAAGATTTTGACTTTTACCCCCCTCAT  278
            |||||||||||||||||||||||||||||||||||||||||||||||||||| || |||
Sbjct  181  CCCCAGATATAGCATTTCCACGAATAAATAATATAAGATTTTGACTTTTACCTCCTTCAC  240

Query  279  TATTTTTATTGATTAACTCTTCATTAATTGAATCCGGAGCTGGAACAGGATGAACAGTTT  338
            |||||||||| ||||||||||||||||||||||| |||||||| ||||||||||||||||
Sbjct  241  TATTTTTATTAATTAACTCTTCATTAATTGAATCTGGAGCTGGTACAGGATGAACAGTTT  300

Query  339  ATCCCCCTCTATCTTCAAATTTATCACATTATGGTCCATCAGTAGATTTAGCTATTTTTT  398
            | || ||||||||||||||||||||||||||||| || ||||||||||||||||||||||
Sbjct  301  ACCCTCCTCTATCTTCAAATTTATCACATTATGGCCCTTCAGTAGATTTAGCTATTTTTT  360

Query  399  CTCTTCACCTTGCTGGTGCTTCTTCAATTTTAGGTGCAATTAACTTCATCACAACTATTG  458
            ||||||| |||||||||||||||||||||||||||||||||||||| || ||||||||||
Sbjct  361  CTCTTCATCTTGCTGGTGCTTCTTCAATTTTAGGTGCAATTAACTTTATTACAACTATTG  420

Query  459  TAAATATACGATCAATTGGAATAACAATAGAACGAATACCATTATTTGTTTGATCTGTTT  518
            |||| ||||||||||||||||||||||||||||| |||||||||||||||||||||||||
Sbjct  421  TAAACATACGATCAATTGGAATAACAATAGAACGTATACCATTATTTGTTTGATCTGTTT  480

Query  519  TAATTACAGCTATTTTACTACTTCTATCTTTACCTGTATTAGCAGGTGCCATTACAATAT  578
            ||||||||||||||||| ||||| ||||||||||||||||||||||||||||||||||||
Sbjct  481  TAATTACAGCTATTTTATTACTTTTATCTTTACCTGTATTAGCAGGTGCCATTACAATAT  540

Query  579  TATTAACTGATCGAAACTTTAACACATCATTTTTTGATCCTTCAGGAGGAGGAGATCCCA  638
            | |||||||||||||||||||| ||||||||||||||||||||||| ||||||||||| |
Sbjct  541  TGTTAACTGATCGAAACTTTAATACATCATTTTTTGATCCTTCAGGGGGAGGAGATCCAA  600
```

```
Query  639  TTTTATATCAACACTTATTT  658
            ||||||||||||| ||||||
Sbjct  601  TTTTATATCAACATTTATTT  620
```

>Rhipicephalus sanguineus isolate Rsan-ML3 cytochrome oxidase subunit I (cox1)
gene, partial cds; mitochondrial
Sequence ID: MG855657.1 Length: 630
Range 1: 1 to 620

Score:990 bits(536), Expect:0.0,
Identities:592/620(95%), Gaps:0/620(0%), Strand: Plus/Plus

```
Query  39   TAGGACTTAGTATAAGAATATTAATTCGTATAGAATTAGGACAACCTGGAACTTTAATTG  98
            ||||||||||||||||||||||||||||||||||||||||||||||||||||||||||||
Sbjct  1    TAGGACTTAGTATAAGAATATTAATTCGTATAGAATTAGGACAACCTGGAACTTTAATTG  60

Query  99   GAAATGATCAGATTTATAATGTAATCGTAACAGCACATGCATTTATTATAATTTTCTTTA  158
            ||||||||||||||||||||||||||||||||||||||||||||||||||||||| ||||
Sbjct  61   GAAATGATCAGATTTATAATGTAATCGTAACAGCACATGCATTTATTATAATTTTTTTTA  120

Query  159  TAGTTATACCAATCATAATCGGAGGGTTCGGAAATTGATTAGTTCCTATTATATTAGGTG  218
            ||||||||||||||||||||||||| || |||||||||||||| ||||| ||||| ||||
Sbjct  121  TAGTTATACCAATCATAATCGGAGGATTTGGAAATTGATTAGTCCCTATCATATTGGGTG  180

Query  219  CCCCAGATATAGCATTTCCACGAATAAATAATATAAGATTTTGACTTTTACCCCCCTCAT  278
            |||||||||||||||||||||||||||||||||||||||||||||||||||| || |||
Sbjct  181  CCCCAGATATAGCATTTCCACGAATAAATAATATAAGATTTTGACTTTTACCTCCTTCAC  240

Query  279  TATTTTTATTGATTAACTCTTCATTAATTGAATCCGGAGCTGGAACAGGATGAACAGTTT  338
            |||||||||| ||||||||||||||||||||||| |||||||| ||||||||||||||||
Sbjct  241  TATTTTTATTAATTAACTCTTCATTAATTGAATCTGGAGCTGGTACAGGATGAACAGTTT  300

Query  339  ATCCCCCTCTATCTTCAAATTTATCACATTATGGTCCATCAGTAGATTTAGCTATTTTTT  398
            | || ||||||||||||||||||||||||||||| || ||||||||||||||||||||||
Sbjct  301  ACCCTCCTCTATCTTCAAATTTATCACATTATGGCCCTTCAGTAGATTTAGCTATTTTTT  360

Query  399  CTCTTCACCTTGCTGGTGCTTCTTCAATTTTAGGTGCAATTAACTTCATCACAACTATTG  458
            ||||||| |||||||||||||||||||||||||||||||||||||| || ||||||||||
Sbjct  361  CTCTTCATCTTGCTGGTGCTTCTTCAATTTTAGGTGCAATTAACTTTATTACAACTATTG  420

Query  459  TAAATATACGATCAATTGGAATAACAATAGAACGAATACCATTATTTGTTTGATCTGTTT  518
            |||| ||||||||||||||||||||||||||||| |||||||||||||||||||||||||
Sbjct  421  TAAACATACGATCAATTGGAATAACAATAGAACGTATACCATTATTTGTTTGATCTGTTT  480

Query  519  TAATTACAGCTATTTTACTACTTCTATCTTTACCTGTATTAGCAGGTGCCATTACAATAT  578
            ||||||||||||||||| ||||| ||||||||||||||||||||||||||||||||||||
Sbjct  481  TAATTACAGCTATTTTATTACTTTTATCTTTACCTGTATTAGCAGGTGCCATTACAATAT  540

Query  579  TATTAACTGATCGAAACTTTAACACATCATTTTTTGATCCTTCAGGAGGAGGAGATCCCA  638
            | |||||||||||||||||||| ||||||||||||||||||||||| ||||||||||| |
```

```
Sbjct  541   TGTTAACTGATCGAAACTTTAATACATCATTTTTTGATCCTTCAGGGGGAGGAGATCCAA  600

Query  639   TTTTATATCAACACTTATTT  658
             ||||||||||||| ||||||
Sbjct  601   TTTTATATCAACATTTATTT  620
```

>Rhipicephalus sanguineus isolate Rsan-ML2 cytochrome oxidase subunit I (cox1) gene, partial cds; mitochondrial
Sequence ID: MG855656.1 Length: 630
Range 1: 1 to 620

Score:990 bits(536), Expect:0.0,
Identities:592/620(95%), Gaps:0/620(0%), Strand: Plus/Plus

```
Query  39    TAGGACTTAGTATAAGAATATTAATTCGTATAGAATTAGGACAACCTGGAACTTTAATTG  98
             ||||||||||||||||||||||||||||||||||||||||||||||||||||||||||||
Sbjct  1     TAGGACTTAGTATAAGAATATTAATTCGTATAGAATTAGGACAACCTGGAACTTTAATTG  60

Query  99    GAAATGATCAGATTTATAATGTAATCGTAACAGCACATGCATTTATTATAATTTTCTTTA  158
             ||||||||||||||||||||||||||||||||||||||||||||||||||||||| ||||
Sbjct  61    GAAATGATCAGATTTATAATGTAATCGTAACAGCACATGCATTTATTATAATTTTTTTTA  120

Query  159   TAGTTATACCAATCATAATCGGAGGGTTCGGAAATTGATTAGTTCCTATTATATTAGGTG  218
             ||||||||||||||||||||||||| || |||||||||||||| ||||| ||||| ||||
Sbjct  121   TAGTTATACCAATCATAATCGGAGGATTTGGAAATTGATTAGTCCCTATCATATTGGGTG  180

Query  219   CCCCAGATATAGCATTTCCACGAATAAATAATATAAGATTTTGACTTTTACCCCCCTCAT  278
             |||||||||||||||||||||||||||||||||||||||||||||||||||| || ||| 
Sbjct  181   CCCCAGATATAGCATTTCCACGAATAAATAATATAAGATTTTGACTTTTACCTCCTTCAC  240

Query  279   TATTTTTATTGATTAACTCTTCATTAATTGAATCCGGAGCTGGAACAGGATGAACAGTTT  338
             |||||||||| ||||||||||||||||||||||| |||||||| ||||||||||||||||
Sbjct  241   TATTTTTATTAATTAACTCTTCATTAATTGAATCTGGAGCTGGTACAGGATGAACAGTTT  300

Query  339   ATCCCCCTCTATCTTCAAATTTATCACATTATGGTCCATCAGTAGATTTAGCTATTTTTT  398
             | || ||||||||||||||||||||||||||||| || ||||||||||||||||||||||
Sbjct  301   ACCCTCCTCTATCTTCAAATTTATCACATTATGGCCCTTCAGTAGATTTAGCTATTTTTT  360

Query  399   CTCTTCACCTTGCTGGTGCTTCTTCAATTTTAGGTGCAATTAACTTCATCACAACTATTG  458
             ||||||| |||||||||||||||||||||||||||||||||||||| || ||||||||||
Sbjct  361   CTCTTCATCTTGCTGGTGCTTCTTCAATTTTAGGTGCAATTAACTTTATTACAACTATTG  420

Query  459   TAAATATACGATCAATTGGAATAACAATAGAACGAATACCATTATTTGTTTGATCTGTTT  518
             |||| ||||||||||||||||||||||||||||| |||||||||||||||||||||||||
Sbjct  421   TAAACATACGATCAATTGGAATAACAATAGAACGTATACCATTATTTGTTTGATCTGTTT  480

Query  519   TAATTACAGCTATTTTACTACTTCTATCTTTACCTGTATTAGCAGGTGCCATTACAATAT  578
             ||||||||||||||||| ||||| ||||||||||||||||||||||||||||||||||||
Sbjct  481   TAATTACAGCTATTTTATTACTTTTATCTTTACCTGTATTAGCAGGTGCCATTACAATAT  540

Query  579   TATTAACTGATCGAAACTTTAACACATCATTTTTTGATCCTTCAGGAGGAGGAGATCCCA  638
```

```
            | |||||||||||||||||||| ||||||||||||||||||||||| ||||||||||| |
Sbjct  541  TGTTAACTGATCGAAACTTTAATACATCATTTTTTGATCCTTCAGGGGGAGGAGATCCAA  600

Query  639  TTTTATATCAACACTTATTT  658
            ||||||||||||| ||||||
Sbjct  601  TTTTATATCAACATTTATTT  620
```

>Rhipicephalus sanguineus isolate Kehf-Lagareb-A cytochrome oxidase subunit I (COI) gene, partial cds; mitochondrial
Sequence ID: KX757910.1 Length: 630
Range 1: 1 to 620

Score:990 bits(536), Expect:0.0,
Identities:592/620(95%), Gaps:0/620(0%), Strand: Plus/Plus

```
Query  39   TAGGACTTAGTATAAGAATATTAATTCGTATAGAATTAGGACAACCTGGAACTTTAATTG  98
            ||||||||||||||||||||||||||||||||||||||||||||||||||||||||||||
Sbjct  1    TAGGACTTAGTATAAGAATATTAATTCGTATAGAATTAGGACAACCTGGAACTTTAATTG  60

Query  99   GAAATGATCAGATTTATAATGTAATCGTAACAGCACATGCATTTATTATAATTTTCTTTA  158
            ||||||||||||||||||||||||||||||||||||||||||||||||||||||| ||||
Sbjct  61   GAAATGATCAGATTTATAATGTAATCGTAACAGCACATGCATTTATTATAATTTTTTTTA  120

Query  159  TAGTTATACCAATCATAATCGGAGGGTTCGGAAATTGATTAGTTCCTATTATATTAGGTG  218
            ||||||||||||||||||||||||| || |||||||||||||| ||||| ||||| ||||
Sbjct  121  TAGTTATACCAATCATAATCGGAGGATTTGGAAATTGATTAGTCCCTATCATATTGGGTG  180

Query  219  CCCCAGATATAGCATTTCCACGAATAAATAATATAAGATTTTGACTTTTACCCCCCTCAT  278
            |||||||||||||||||||||||||||||||||||||||||||||||||||| || ||| 
Sbjct  181  CCCCAGATATAGCATTTCCACGAATAAATAATATAAGATTTTGACTTTTACCTCCTTCAC  240

Query  279  TATTTTTATTGATTAACTCTTCATTAATTGAATCCGGAGCTGGAACAGGATGAACAGTTT  338
            |||||||||| ||||||||||||||||||||||| |||||||| ||||||||||||||||
Sbjct  241  TATTTTTATTAATTAACTCTTCATTAATTGAATCTGGAGCTGGTACAGGATGAACAGTTT  300

Query  339  ATCCCCCTCTATCTTCAAATTTATCACATTATGGTCCATCAGTAGATTTAGCTATTTTTT  398
            | || ||||||||||||||||||||||||||||| || ||||||||||||||||||||||
Sbjct  301  ACCCTCCTCTATCTTCAAATTTATCACATTATGGCCCTTCAGTAGATTTAGCTATTTTTT  360

Query  399  CTCTTCACCTTGCTGGTGCTTCTTCAATTTTAGGTGCAATTAACTTCATCACAACTATTG  458
            ||||||| |||||||||||||||||||||||||||||||||||||| || ||||||||||
Sbjct  361  CTCTTCATCTTGCTGGTGCTTCTTCAATTTTAGGTGCAATTAACTTTATTACAACTATTG  420

Query  459  TAAATATACGATCAATTGGAATAACAATAGAACGAATACCATTATTTGTTTGATCTGTTT  518
            |||| ||||||||||||||||||||||||||||| |||||||||||||||||||||||||
Sbjct  421  TAAACATACGATCAATTGGAATAACAATAGAACGTATACCATTATTTGTTTGATCTGTTT  480

Query  519  TAATTACAGCTATTTTACTACTTCTATCTTTACCTGTATTAGCAGGTGCCATTACAATAT  578
            ||||||||||||||||| ||||| ||||||||||||||||||||||||||||||||||||
Sbjct  481  TAATTACAGCTATTTTATTACTTTTATCTTTACCTGTATTAGCAGGTGCCATTACAATAT  540
```

```
Query  579  TATTAACTGATCGAAACTTTAACACATCATTTTTTGATCCTTCAGGAGGAGGAGATCCCA  638
            | |||||||||||||||||||| ||||||||||||||||||||||| ||||||||||| |
Sbjct  541  TGTTAACTGATCGAAACTTTAATACATCATTTTTTGATCCTTCAGGGGGAGGAGATCCAA  600

Query  639  TTTTATATCAACACTTATTT  658
            ||||||||||||| ||||||
Sbjct  601  TTTTATATCAACATTTATTT  620
```

>Rhipicephalus sanguineus isolate Italy-B cytochrome oxidase subunit I (COI) gene, partial cds; mitochondrial
Sequence ID: KX757904.1 Length: 630
Range 1: 1 to 620

Score:990 bits(536), Expect:0.0,
Identities:592/620(95%), Gaps:0/620(0%), Strand: Plus/Plus

```
Query  39   TAGGACTTAGTATAAGAATATTAATTCGTATAGAATTAGGACAACCTGGAACTTTAATTG  98
            ||||||||||||||||||||||||||||||||||||||||||||||||||||||||||||
Sbjct  1    TAGGACTTAGTATAAGAATATTAATTCGTATAGAATTAGGACAACCTGGAACTTTAATTG  60

Query  99   GAAATGATCAGATTTATAATGTAATCGTAACAGCACATGCATTTATTATAATTTTCTTTA  158
            ||||||||||||||||||||||||||||||||||||||||||||||||||||||| ||||
Sbjct  61   GAAATGATCAGATTTATAATGTAATCGTAACAGCACATGCATTTATTATAATTTTTTTTA  120

Query  159  TAGTTATACCAATCATAATCGGAGGGTTCGGAAATTGATTAGTTCCTATTATATTAGGTG  218
            ||||||||||||||||||||||||| || |||||||||||||| ||||| ||||| ||||
Sbjct  121  TAGTTATACCAATCATAATCGGAGGATTTGGAAATTGATTAGTCCCTATCATATTGGGTG  180

Query  219  CCCCAGATATAGCATTTCCACGAATAAATAATATAAGATTTTGACTTTTACCCCCCTCAT  278
            |||||||||||||||||||||||||||||||||||||||||||||||||||| || ||| 
Sbjct  181  CCCCAGATATAGCATTTCCACGAATAAATAATATAAGATTTTGACTTTTACCTCCTTCAC  240

Query  279  TATTTTTATTGATTAACTCTTCATTAATTGAATCCGGAGCTGGAACAGGATGAACAGTTT  338
            |||||||||| |||||||||||||||||||||||| ||||||||| ||||||||||||||
Sbjct  241  TATTTTTATTAATTAACTCTTCATTAATTGAATCTGGAGCTGGTACAGGATGAACAGTTT  300

Query  339  ATCCCCCTCTATCTTCAAATTTATCACATTATGGTCCATCAGTAGATTTAGCTATTTTTT  398
            | || ||||||||||||||||||||||||||||| || ||||||||||||||||||||||
Sbjct  301  ACCCTCCTCTATCTTCAAATTTATCACATTATGGCCCTTCAGTAGATTTAGCTATTTTTT  360

Query  399  CTCTTCACCTTGCTGGTGCTTCTTCAATTTTAGGTGCAATTAACTTCATCACAACTATTG  458
            ||||||| |||||||||||||||||||||||||||||||||||||| || ||||||||||
Sbjct  361  CTCTTCATCTTGCTGGTGCTTCTTCAATTTTAGGTGCAATTAACTTTATTACAACTATTG  420

Query  459  TAAATATACGATCAATTGGAATAACAATAGAACGAATACCATTATTTGTTTGATCTGTTT  518
            |||| ||||||||||||||||||||||||||||| |||||||||||||||||||||||||
Sbjct  421  TAAACATACGATCAATTGGAATAACAATAGAACGTATACCATTATTTGTTTGATCTGTTT  480

Query  519  TAATTACAGCTATTTTACTACTTCTATCTTTACCTGTATTAGCAGGTGCCATTACAATAT  578
            ||||||||||||||||| ||||| ||||||||||||||||||||||||||||||||||||
Sbjct  481  TAATTACAGCTATTTTATTACTTTTATCTTTACCTGTATTAGCAGGTGCCATTACAATAT  540
```

```
Query  579  TATTAACTGATCGAAACTTTAACACATCATTTTTTGATCCTTCAGGAGGAGGAGATCCCA  638
            | |||||||||||||||||||| ||||||||||||||||||||||| ||||||||||| |
Sbjct  541  TGTTAACTGATCGAAACTTTAATACATCATTTTTTGATCCTTCAGGGGGAGGAGATCCAA  600

Query  639  TTTTATATCAACACTTATTT  658
            ||||||||||||| ||||||
Sbjct  601  TTTTATATCAACATTTATTT  620
```

>Rhipicephalus sanguineus isolate Malta-A cytochrome oxidase subunit I (COI) gene, partial cds; mitochondrial
Sequence ID: KX757902.1 Length: 630
Range 1: 1 to 620

Score:990 bits(536), Expect:0.0,
Identities:592/620(95%), Gaps:0/620(0%), Strand: Plus/Plus

```
Query  39   TAGGACTTAGTATAAGAATATTAATTCGTATAGAATTAGGACAACCTGGAACTTTAATTG  98
            ||||||||||||||||||||||||||||||||||||||||||||||||||||||||||||
Sbjct  1    TAGGACTTAGTATAAGAATATTAATTCGTATAGAATTAGGACAACCTGGAACTTTAATTG  60

Query  99   GAAATGATCAGATTTATAATGTAATCGTAACAGCACATGCATTTATTATAATTTTCTTTA  158
            ||||||||||||||||||||||||||||||||||||||||||||||||||||||| ||||
Sbjct  61   GAAATGATCAGATTTATAATGTAATCGTAACAGCACATGCATTTATTATAATTTTTTTTA  120

Query  159  TAGTTATACCAATCATAATCGGAGGGTTCGGAAATTGATTAGTTCCTATTATATTAGGTG  218
            ||||||||||||||||||||||||| || |||||||||||||| ||||| ||||| ||||
Sbjct  121  TAGTTATACCAATCATAATCGGAGGATTTGGAAATTGATTAGTCCCTATCATATTGGGTG  180

Query  219  CCCCAGATATAGCATTTCCACGAATAAATAATATAAGATTTTGACTTTTACCCCCCTCAT  278
            |||||||||||||||||||||||||||||||||||||||||||||||||||| || |||
Sbjct  181  CCCCAGATATAGCATTTCCACGAATAAATAATATAAGATTTTGACTTTTACCTCCTTCAC  240

Query  279  TATTTTTATTGATTAACTCTTCATTAATTGAATCCGGAGCTGGAACAGGATGAACAGTTT  338
            |||||||||| ||||||||||||||||||||||| |||||||| ||||||||||||||||
Sbjct  241  TATTTTTATTAATTAACTCTTCATTAATTGAATCTGGAGCTGGTACAGGATGAACAGTTT  300

Query  339  ATCCCCCTCTATCTTCAAATTTATCACATTATGGTCCATCAGTAGATTTAGCTATTTTTT  398
            | || ||||||||||||||||||||||||||||| || ||||||||||||||||||||||
Sbjct  301  ACCCTCCTCTATCTTCAAATTTATCACATTATGGCCCTTCAGTAGATTTAGCTATTTTTT  360

Query  399  CTCTTCACCTTGCTGGTGCTTCTTCAATTTTAGGTGCAATTAACTTCATCACAACTATTG  458
            ||||||| |||||||||||||||||||||||||||||||||||||| || ||||||||||
Sbjct  361  CTCTTCATCTTGCTGGTGCTTCTTCAATTTTAGGTGCAATTAACTTTATTACAACTATTG  420

Query  459  TAAATATACGATCAATTGGAATAACAATAGAACGAATACCATTATTTGTTTGATCTGTTT  518
            |||| ||||||||||||||||||||||||||||| |||||||||||||||||||||||||
Sbjct  421  TAAACATACGATCAATTGGAATAACAATAGAACGTATACCATTATTTGTTTGATCTGTTT  480

Query  519  TAATTACAGCTATTTTACTACTTCTATCTTTACCTGTATTAGCAGGTGCCATTACAATAT  578
            ||||||||||||||||| ||||| ||||||||||||||||||||||||||||||||||||
```

```
Sbjct  481   TAATTACAGCTATTTTATTACTTTTATCTTTACCTGTATTAGCAGGTGCCATTACAATAT  540

Query  579   TATTAACTGATCGAAACTTTAACACATCATTTTTTGATCCTTCAGGAGGAGGAGATCCCA  638
             | |||||||||||||||||||| ||||||||||||||||||||||| ||||||||||| |
Sbjct  541   TGTTAACTGATCGAAACTTTAATACATCATTTTTTGATCCTTCAGGGGGAGGAGATCCAA  600

Query  639   TTTTATATCAACACTTATTT  658
             ||||||||||||| ||||||
Sbjct  601   TTTTATATCAACATTTATTT  620
```

>Rhipicephalus sanguineus isolate Zagreb-C cytochrome oxidase subunit I (COI) gene, partial cds; mitochondrial
Sequence ID: KX757896.1 Length: 630
Range 1: 1 to 620

Score:990 bits(536), Expect:0.0,
Identities:592/620(95%), Gaps:0/620(0%), Strand: Plus/Plus

```
Query  39    TAGGACTTAGTATAAGAATATTAATTCGTATAGAATTAGGACAACCTGGAACTTTAATTG  98
             ||||||||||||||||||||||||||||||||||||||||||||||||||||||||||||
Sbjct  1     TAGGACTTAGTATAAGAATATTAATTCGTATAGAATTAGGACAACCTGGAACTTTAATTG  60

Query  99    GAAATGATCAGATTTATAATGTAATCGTAACAGCACATGCATTTATTATAATTTTCTTTA  158
             ||||||||||||||||||||||||||||||||||||||||||||||||||||||| ||||
Sbjct  61    GAAATGATCAGATTTATAATGTAATCGTAACAGCACATGCATTTATTATAATTTTTTTTA  120

Query  159   TAGTTATACCAATCATAATCGGAGGGTTCGGAAATTGATTAGTTCCTATTATATTAGGTG  218
             ||||||||||||||||||||||||| || |||||||||||||| ||||| ||||| ||||
Sbjct  121   TAGTTATACCAATCATAATCGGAGGATTTGGAAATTGATTAGTCCCTATCATATTGGGTG  180

Query  219   CCCCAGATATAGCATTTCCACGAATAAATAATATAAGATTTTGACTTTTACCCCCCTCAT  278
             |||||||||||||||||||||||||||||||||||||||||||||||||||| || |||
Sbjct  181   CCCCAGATATAGCATTTCCACGAATAAATAATATAAGATTTTGACTTTTACCTCCTTCAC  240

Query  279   TATTTTTATTGATTAACTCTTCATTAATTGAATCCGGAGCTGGAACAGGATGAACAGTTT  338
             |||||||||| ||||||||||||||||||||||| |||||||| ||||||||||||||||
Sbjct  241   TATTTTTATTAATTAACTCTTCATTAATTGAATCTGGAGCTGGTACAGGATGAACAGTTT  300

Query  339   ATCCCCCTCTATCTTCAAATTTATCACATTATGGTCCATCAGTAGATTTAGCTATTTTTT  398
             | || ||||||||||||||||||||||||||||| || ||||||||||||||||||||||
Sbjct  301   ACCCTCCTCTATCTTCAAATTTATCACATTATGGCCCTTCAGTAGATTTAGCTATTTTTT  360

Query  399   CTCTTCACCTTGCTGGTGCTTCTTCAATTTTAGGTGCAATTAACTTCATCACAACTATTG  458
             ||||||| |||||||||||||||||||||||||||||||||||||| || ||||||||||
Sbjct  361   CTCTTCATCTTGCTGGTGCTTCTTCAATTTTAGGTGCAATTAACTTTATTACAACTATTG  420

Query  459   TAAATATACGATCAATTGGAATAACAATAGAACGAATACCATTATTTGTTTGATCTGTTT  518
             |||| ||||||||||||||||||||||||||||| |||||||||||||||||||||||||
Sbjct  421   TAAACATACGATCAATTGGAATAACAATAGAACGTATACCATTATTTGTTTGATCTGTTT  480

Query  519   TAATTACAGCTATTTTACTACTTCTATCTTTACCTGTATTAGCAGGTGCCATTACAATAT  578
```

```
             ||||||||||||||||| ||||| ||||||||||||||||||||||||||||||||||||
Sbjct  481   TAATTACAGCTATTTTATTACTTTTATCTTTACCTGTATTAGCAGGTGCCATTACAATAT  540

Query  579   TATTAACTGATCGAAACTTTAACACATCATTTTTTGATCCTTCAGGAGGAGGAGATCCCA  638
             | |||||||||||||||||||| ||||||||||||||||||||||| ||||||||||| |
Sbjct  541   TGTTAACTGATCGAAACTTTAATACATCATTTTTTGATCCTTCAGGGGGAGGAGATCCAA  600

Query  639   TTTTATATCAACACTTATTT  658
             ||||||||||||| ||||||
Sbjct  601   TTTTATATCAACATTTATTT  620
```

>Rhipicephalus sanguineus isolate Pula-B cytochrome oxidase subunit I (COI) gene, partial cds; mitochondrial
Sequence ID: KX757889.1 Length: 630
Range 1: 1 to 620

Score:990 bits(536), Expect:0.0,
Identities:592/620(95%), Gaps:0/620(0%), Strand: Plus/Plus

```
Query  39    TAGGACTTAGTATAAGAATATTAATTCGTATAGAATTAGGACAACCTGGAACTTTAATTG  98
             ||||||||||||||||||||||||||||||||||||||||||||||||||||||||||||
Sbjct  1     TAGGACTTAGTATAAGAATATTAATTCGTATAGAATTAGGACAACCTGGAACTTTAATTG  60

Query  99    GAAATGATCAGATTTATAATGTAATCGTAACAGCACATGCATTTATTATAATTTTCTTTA  158
             ||||||||||||||||||||||||||||||||||||||||||||||||||||||| ||||
Sbjct  61    GAAATGATCAGATTTATAATGTAATCGTAACAGCACATGCATTTATTATAATTTTTTTTA  120

Query  159   TAGTTATACCAATCATAATCGGAGGGTTCGGAAATTGATTAGTTCCTATTATATTAGGTG  218
             ||||||||||||||||||||||||| || |||||||||||||| ||||| ||||| ||||
Sbjct  121   TAGTTATACCAATCATAATCGGAGGATTTGGAAATTGATTAGTCCCTATCATATTGGGTG  180

Query  219   CCCCAGATATAGCATTTCCACGAATAAATAATATAAGATTTTGACTTTTACCCCCCTCAT  278
             |||||||||||||||||||||||||||||||||||||||||||||||||||| || ||| 
Sbjct  181   CCCCAGATATAGCATTTCCACGAATAAATAATATAAGATTTTGACTTTTACCTCCTTCAC  240

Query  279   TATTTTTATTGATTAACTCTTCATTAATTGAATCCGGAGCTGGAACAGGATGAACAGTTT  338
             |||||||||| ||||||||||||||||||||||| |||||||| ||||||||||||||||
Sbjct  241   TATTTTTATTAATTAACTCTTCATTAATTGAATCTGGAGCTGGTACAGGATGAACAGTTT  300

Query  339   ATCCCCCTCTATCTTCAAATTTATCACATTATGGTCCATCAGTAGATTTAGCTATTTTTT  398
             | || ||||||||||||||||||||||||||||| || ||||||||||||||||||||||
Sbjct  301   ACCCTCCTCTATCTTCAAATTTATCACATTATGGCCCTTCAGTAGATTTAGCTATTTTTT  360

Query  399   CTCTTCACCTTGCTGGTGCTTCTTCAATTTTAGGTGCAATTAACTTCATCACAACTATTG  458
             ||||||| |||||||||||||||||||||||||||||||||||||| || ||||||||||
Sbjct  361   CTCTTCATCTTGCTGGTGCTTCTTCAATTTTAGGTGCAATTAACTTTATTACAACTATTG  420

Query  459   TAAATATACGATCAATTGGAATAACAATAGAACGAATACCATTATTTGTTTGATCTGTTT  518
             |||| ||||||||||||||||||||||||||||| |||||||||||||||||||||||||
Sbjct  421   TAAACATACGATCAATTGGAATAACAATAGAACGTATACCATTATTTGTTTGATCTGTTT  480
```

```
Query  519   TAATTACAGCTATTTTACTACTTCTATCTTTACCTGTATTAGCAGGTGCCATTACAATAT  578
             ||||||||||||||||| ||||| ||||||||||||||||||||||||||||||||||||
Sbjct  481   TAATTACAGCTATTTTATTACTTTTATCTTTACCTGTATTAGCAGGTGCCATTACAATAT  540

Query  579   TATTAACTGATCGAAACTTTAACACATCATTTTTTGATCCTTCAGGAGGAGGAGATCCCA  638
             | |||||||||||||||||||| ||||||||||||||||||||||| ||||||||||| |
Sbjct  541   TGTTAACTGATCGAAACTTTAATACATCATTTTTTGATCCTTCAGGGGGAGGAGATCCAA  600

Query  639   TTTTATATCAACACTTATTT  658
             ||||||||||||| ||||||
Sbjct  601   TTTTATATCAACATTTATTT  620
```

>Rhipicephalus sanguineus isolate Rovind cytochrome oxidase subunit I (COI) gene, partial cds; mitochondrial
Sequence ID: KX757887.1 Length: 630
Range 1: 1 to 620

Score:990 bits(536), Expect:0.0,
Identities:592/620(95%), Gaps:0/620(0%), Strand: Plus/Plus

```
Query  39    TAGGACTTAGTATAAGAATATTAATTCGTATAGAATTAGGACAACCTGGAACTTTAATTG  98
             ||||||||||||||||||||||||||||||||||||||||||||||||||||||||||||
Sbjct  1     TAGGACTTAGTATAAGAATATTAATTCGTATAGAATTAGGACAACCTGGAACTTTAATTG  60

Query  99    GAAATGATCAGATTTATAATGTAATCGTAACAGCACATGCATTTATTATAATTTTCTTTA  158
             ||||||||||||||||||||||||||||||||||||||||||||||||||||||| ||||
Sbjct  61    GAAATGATCAGATTTATAATGTAATCGTAACAGCACATGCATTTATTATAATTTTTTTTA  120

Query  159   TAGTTATACCAATCATAATCGGAGGGTTCGGAAATTGATTAGTTCCTATTATATTAGGTG  218
             ||||||||||||||||||||||||| || |||||||||||||| ||||| ||||| ||||
Sbjct  121   TAGTTATACCAATCATAATCGGAGGATTTGGAAATTGATTAGTCCCTATCATATTGGGTG  180

Query  219   CCCCAGATATAGCATTTCCACGAATAAATAATATAAGATTTTGACTTTTACCCCCCTCAT  278
             |||||||||||||||||||||||||||||||||||||||||||||||||||| || |||
Sbjct  181   CCCCAGATATAGCATTTCCACGAATAAATAATATAAGATTTTGACTTTTACCTCCTTCAC  240

Query  279   TATTTTTATTGATTAACTCTTCATTAATTGAATCCGGAGCTGGAACAGGATGAACAGTTT  338
             |||||||||| ||||||||||||||||||||||| |||||||| ||||||||||||||||
Sbjct  241   TATTTTTATTAATTAACTCTTCATTAATTGAATCTGGAGCTGGTACAGGATGAACAGTTT  300

Query  339   ATCCCCCTCTATCTTCAAATTTATCACATTATGGTCCATCAGTAGATTTAGCTATTTTTT  398
             | || ||||||||||||||||||||||||||||| || ||||||||||||||||||||||
Sbjct  301   ACCCTCCTCTATCTTCAAATTTATCACATTATGGCCCTTCAGTAGATTTAGCTATTTTTT  360

Query  399   CTCTTCACCTTGCTGGTGCTTCTTCAATTTTAGGTGCAATTAACTTCATCACAACTATTG  458
             ||||||| |||||||||||||||||||||||||||||||||||||| || ||||||||||
Sbjct  361   CTCTTCATCTTGCTGGTGCTTCTTCAATTTTAGGTGCAATTAACTTTATTACAACTATTG  420

Query  459   TAAATATACGATCAATTGGAATAACAATAGAACGAATACCATTATTTGTTTGATCTGTTT  518
             |||| ||||||||||||||||||||||||||||| |||||||||||||||||||||||||
Sbjct  421   TAAACATACGATCAATTGGAATAACAATAGAACGTATACCATTATTTGTTTGATCTGTTT  480
```

```
Query  519   TAATTACAGCTATTTTACTACTTCTATCTTTACCTGTATTAGCAGGTGCCATTACAATAT  578
             ||||||||||||||||| ||||| ||||||||||||||||||||||||||||||||||||
Sbjct  481   TAATTACAGCTATTTTATTACTTTTATCTTTACCTGTATTAGCAGGTGCCATTACAATAT  540

Query  579   TATTAACTGATCGAAACTTTAACACATCATTTTTTGATCCTTCAGGAGGAGGAGATCCCA  638
             | |||||||||||||||||||| ||||||||||||||||||||||| ||||||||||| |
Sbjct  541   TGTTAACTGATCGAAACTTTAATACATCATTTTTTGATCCTTCAGGGGGAGGAGATCCAA  600

Query  639   TTTTATATCAACACTTATTT  658
             ||||||||||||| ||||||
Sbjct  601   TTTTATATCAACATTTATTT  620
```

>Rhipicephalus sanguineus isolate Kajtasovo cytochrome oxidase subunit I (COI) gene, partial cds; mitochondrial
Sequence ID: KX757879.1 Length: 630
Range 1: 1 to 620

Score:990 bits(536), Expect:0.0,
Identities:592/620(95%), Gaps:0/620(0%), Strand: Plus/Plus

```
Query  39    TAGGACTTAGTATAAGAATATTAATTCGTATAGAATTAGGACAACCTGGAACTTTAATTG  98
             ||||||||||||||||||||||||||||||||||||||||||||||||||||||||||||
Sbjct  1     TAGGACTTAGTATAAGAATATTAATTCGTATAGAATTAGGACAACCTGGAACTTTAATTG  60

Query  99    GAAATGATCAGATTTATAATGTAATCGTAACAGCACATGCATTTATTATAATTTTCTTTA  158
             ||||||||||||||||||||||||||||||||||||||||||||||||||||||| ||||
Sbjct  61    GAAATGATCAGATTTATAATGTAATCGTAACAGCACATGCATTTATTATAATTTTTTTTA  120

Query  159   TAGTTATACCAATCATAATCGGAGGGTTCGGAAATTGATTAGTTCCTATTATATTAGGTG  218
             ||||||||||||||||||||||||| || |||||||||||||| ||||| ||||| ||||
Sbjct  121   TAGTTATACCAATCATAATCGGAGGATTTGGAAATTGATTAGTCCCTATCATATTGGGTG  180

Query  219   CCCCAGATATAGCATTTCCACGAATAAATAATATAAGATTTTGACTTTTACCCCCCTCAT  278
             |||||||||||||||||||||||||||||||||||||||||||||||||||| || |||
Sbjct  181   CCCCAGATATAGCATTTCCACGAATAAATAATATAAGATTTTGACTTTTACCTCCTTCAC  240

Query  279   TATTTTTATTGATTAACTCTTCATTAATTGAATCCGGAGCTGGAACAGGATGAACAGTTT  338
             |||||||||| ||||||||||||||||||||||| |||||||| ||||||||||||||||
Sbjct  241   TATTTTTATTAATTAACTCTTCATTAATTGAATCTGGAGCTGGTACAGGATGAACAGTTT  300

Query  339   ATCCCCCTCTATCTTCAAATTTATCACATTATGGTCCATCAGTAGATTTAGCTATTTTTT  398
             | || ||||||||||||||||||||||||||||| || ||||||||||||||||||||||
Sbjct  301   ACCCTCCTCTATCTTCAAATTTATCACATTATGGCCCTTCAGTAGATTTAGCTATTTTTT  360

Query  399   CTCTTCACCTTGCTGGTGCTTCTTCAATTTTAGGTGCAATTAACTTCATCACAACTATTG  458
             ||||||| |||||||||||||||||||||||||||||||||||||| || ||||||||||
Sbjct  361   CTCTTCATCTTGCTGGTGCTTCTTCAATTTTAGGTGCAATTAACTTTATTACAACTATTG  420

Query  459   TAAATATACGATCAATTGGAATAACAATAGAACGAATACCATTATTTGTTTGATCTGTTT  518
             |||| ||||||||||||||||||||||||||||| |||||||||||||||||||||||||
```

```
Sbjct  421   TAAACATACGATCAATTGGAATAACAATAGAACGTATACCATTATTTGTTTGATCTGTTT  480

Query  519   TAATTACAGCTATTTTACTACTTCTATCTTTACCTGTATTAGCAGGTGCCATTACAATAT  578
             ||||||||||||||||| ||||| ||||||||||||||||||||||||||||||||||||
Sbjct  481   TAATTACAGCTATTTTATTACTTTTATCTTTACCTGTATTAGCAGGTGCCATTACAATAT  540

Query  579   TATTAACTGATCGAAACTTTAACACATCATTTTTTGATCCTTCAGGAGGAGGAGATCCCA  638
             | |||||||||||||||||||| ||||||||||||||||||||||| ||||||||||| |
Sbjct  541   TGTTAACTGATCGAAACTTTAATACATCATTTTTTGATCCTTCAGGGGGAGGAGATCCAA  600

Query  639   TTTTATATCAACACTTATTT  658
             ||||||||||||| ||||||
Sbjct  601   TTTTATATCAACATTTATTT  620
```

>Rhipicephalus sanguineus clone #6 cytochrome oxidase subunit I (COI) gene, partial cds; mitochondrial
Sequence ID: KX519711.1 Length: 636
Range 1: 1 to 620

Score:990 bits(536), Expect:0.0,
Identities:592/620(95%), Gaps:0/620(0%), Strand: Plus/Plus

```
Query  39    TAGGACTTAGTATAAGAATATTAATTCGTATAGAATTAGGACAACCTGGAACTTTAATTG  98
             ||||||||||||||||||||||||||||||||||||||||||||||||||||||||||||
Sbjct  1     TAGGACTTAGTATAAGAATATTAATTCGTATAGAATTAGGACAACCTGGAACTTTAATTG  60

Query  99    GAAATGATCAGATTTATAATGTAATCGTAACAGCACATGCATTTATTATAATTTTCTTTA  158
             |||||||||||||||||||||||||||||||||||||||||||||||||||||| |||||
Sbjct  61    GAAATGATCAGATTTATAATGTAATCGTAACAGCACATGCATTTATTATAATTTTTTTTA  120

Query  159   TAGTTATACCAATCATAATCGGAGGGTTCGGAAATTGATTAGTTCCTATTATATTAGGTG  218
             ||||||||||||||||||||||||| || |||||||||||||| ||||| ||||| ||||
Sbjct  121   TAGTTATACCAATCATAATCGGAGGATTTGGAAATTGATTAGTCCCTATCATATTGGGTG  180

Query  219   CCCCAGATATAGCATTTCCACGAATAAATAATATAAGATTTTGACTTTTACCCCCCTCAT  278
             |||||||||||||||||||||||||||||||||||||||||||||||||||| || ||| 
Sbjct  181   CCCCAGATATAGCATTTCCACGAATAAATAATATAAGATTTTGACTTTTACCTCCTTCAC  240

Query  279   TATTTTTATTGATTAACTCTTCATTAATTGAATCCGGAGCTGGAACAGGATGAACAGTTT  338
             |||||||||| ||||||||||||||||||||||| |||||||| ||||||||||||||||
Sbjct  241   TATTTTTATTAATTAACTCTTCATTAATTGAATCTGGAGCTGGTACAGGATGAACAGTTT  300

Query  339   ATCCCCCTCTATCTTCAAATTTATCACATTATGGTCCATCAGTAGATTTAGCTATTTTTT  398
             | || ||||||||||||||||||||||||||||| || ||||||||||||||||||||||
Sbjct  301   ACCCTCCTCTATCTTCAAATTTATCACATTATGGCCCTTCAGTAGATTTAGCTATTTTTT  360

Query  399   CTCTTCACCTTGCTGGTGCTTCTTCAATTTTAGGTGCAATTAACTTCATCACAACTATTG  458
             ||||||| |||||||||||||||||||||||||||||||||||||| || ||||||||||
Sbjct  361   CTCTTCATCTTGCTGGTGCTTCTTCAATTTTAGGTGCAATTAACTTTATTACAACTATTG  420

Query  459   TAAATATACGATCAATTGGAATAACAATAGAACGAATACCATTATTTGTTTGATCTGTTT  518
```

```
            |||| ||||||||||||||||||||||||||||| |||||||||||||||||||||||||
Sbjct  421  TAAACATACGATCAATTGGAATAACAATAGAACGTATACCATTATTTGTTTGATCTGTTT  480

Query  519  TAATTACAGCTATTTTACTACTTCTATCTTTACCTGTATTAGCAGGTGCCATTACAATAT  578
            ||||||||||||||||| ||||| ||||||||||||||||||||||||||||||||||||
Sbjct  481  TAATTACAGCTATTTTATTACTTTTATCTTTACCTGTATTAGCAGGTGCCATTACAATAT  540

Query  579  TATTAACTGATCGAAACTTTAACACATCATTTTTTGATCCTTCAGGAGGAGGAGATCCCA  638
            | |||||||||||||||||||| ||||||||||||||||||||||| ||||||||||| |
Sbjct  541  TGTTAACTGATCGAAACTTTAATACATCATTTTTTGATCCTTCAGGGGGAGGAGATCCAA  600

Query  639  TTTTATATCAACACTTATTT  658
            ||||||||||||| ||||||
Sbjct  601  TTTTATATCAACATTTATTT  620
```

>Rhipicephalus sanguineus isolate SV4 cytochrome c oxidase subunit I (COX1) gene, partial cds; mitochondrial
Sequence ID: MW135448.1 Length: 630
Range 1: 1 to 620

Score:990 bits(536), Expect:0.0,
Identities:592/620(95%), Gaps:0/620(0%), Strand: Plus/Plus

```
Query  39   TAGGACTTAGTATAAGAATATTAATTCGTATAGAATTAGGACAACCTGGAACTTTAATTG  98
            ||||||||||||||||||||||||||||||||||||||||||||||||||||||||||||
Sbjct  1    TAGGACTTAGTATAAGAATATTAATTCGTATAGAATTAGGACAACCTGGAACTTTAATTG  60

Query  99   GAAATGATCAGATTTATAATGTAATCGTAACAGCACATGCATTTATTATAATTTTCTTTA  158
            ||||||||||||||||||||||||||||||||||||||||||||||||||||||| ||||
Sbjct  61   GAAATGATCAGATTTATAATGTAATCGTAACAGCACATGCATTTATTATAATTTTTTTTA  120

Query  159  TAGTTATACCAATCATAATCGGAGGGTTCGGAAATTGATTAGTTCCTATTATATTAGGTG  218
            ||||||||||||||||||||||||| || |||||||||||||| ||||| ||||| ||||
Sbjct  121  TAGTTATACCAATCATAATCGGAGGATTTGGAAATTGATTAGTCCCTATCATATTGGGTG  180

Query  219  CCCCAGATATAGCATTTCCACGAATAAATAATATAAGATTTTGACTTTTACCCCCCTCAT  278
            |||||||||||||||||||||||||||||||||||||||||||||||||||| || |||
Sbjct  181  CCCCAGATATAGCATTTCCACGAATAAATAATATAAGATTTTGACTTTTACCTCCTTCAC  240

Query  279  TATTTTTATTGATTAACTCTTCATTAATTGAATCCGGAGCTGGAACAGGATGAACAGTTT  338
            |||||||||| ||||||||||||||||||||||| |||||||| ||||||||||||||||
Sbjct  241  TATTTTTATTAATTAACTCTTCATTAATTGAATCTGGAGCTGGTACAGGATGAACAGTTT  300

Query  339  ATCCCCCTCTATCTTCAAATTTATCACATTATGGTCCATCAGTAGATTTAGCTATTTTTT  398
            | || ||||||||||||||||||||||||||||| || ||||||||||||||||||||||
Sbjct  301  ACCCTCCTCTATCTTCAAATTTATCACATTATGGCCCTTCAGTAGATTTAGCTATTTTTT  360

Query  399  CTCTTCACCTTGCTGGTGCTTCTTCAATTTAGGTGCAATTAACTTCATCACAACTATTG  458
            ||||||| ||||||||||||||||||||||||||||||||||||| || |||||||||||
Sbjct  361  CTCTTCATCTTGCTGGTGCTTCTTCAATTTAGGTGCAATTAACTTTATTACAACTATTG  420
```

```
Query  459   TAAATATACGATCAATTGGAATAACAATAGAACGAATACCATTATTTGTTTGATCTGTTT  518
             |||| ||||||||||||||||||||||||||||| |||||||||||||||||||||||||
Sbjct  421   TAAACATACGATCAATTGGAATAACAATAGAACGTATACCATTATTTGTTTGATCTGTTT  480

Query  519   TAATTACAGCTATTTTACTACTTCTATCTTTACCTGTATTAGCAGGTGCCATTACAATAT  578
             ||||||||||||||||| ||||| ||||||||||||||||||||||||||||||||||||
Sbjct  481   TAATTACAGCTATTTTATTACTTTTATCTTTACCTGTATTAGCAGGTGCCATTACAATAT  540

Query  579   TATTAACTGATCGAAACTTTAACACATCATTTTTTGATCCTTCAGGAGGAGGAGATCCCA  638
             | |||||||||||||||||||| ||||||||||||||||||||||| ||||||||||| |
Sbjct  541   TGTTAACTGATCGAAACTTTAATACATCATTTTTTGATCCTTCAGGGGGAGGAGATCCAA  600

Query  639   TTTTATATCAACACTTATTT  658
             ||||||||||||| ||||||
Sbjct  601   TTTTATATCAACATTTATTT  620
```

>Rhipicephalus sanguineus isolate Boka-A cytochrome oxidase subunit I (COI) gene, partial cds; mitochondrial
Sequence ID: KX757905.1 Length: 630
Range 1: 1 to 620

Score:985 bits(533), Expect:0.0,
Identities:591/620(95%), Gaps:0/620(0%), Strand: Plus/Plus

```
Query  39    TAGGACTTAGTATAAGAATATTAATTCGTATAGAATTAGGACAACCTGGAACTTTAATTG  98
             ||||||||||||||||||||||||||||||||||||||||||||||||||||||||||||
Sbjct  1     TAGGACTTAGTATAAGAATATTAATTCGTATAGAATTAGGACAACCTGGAACTTTAATTG  60

Query  99    GAAATGATCAGATTTATAATGTAATCGTAACAGCACATGCATTTATTATAATTTTCTTTA  158
             ||||||||||||||||||||||||||||||||||||||||||||||||||||||| ||||
Sbjct  61    GAAATGATCAGATTTATAATGTAATCGTAACAGCACATGCATTTATTATAATTTTTTTTA  120

Query  159   TAGTTATACCAATCATAATCGGAGGGTTCGGAAATTGATTAGTTCCTATTATATTAGGTG  218
             ||||||||||||||||||||||||| || |||||||||||||| ||||| ||||| ||||
Sbjct  121   TAGTTATACCAATCATAATCGGAGGATTTGGAAATTGATTAGTCCCTATCATATTGGGTG  180

Query  219   CCCCAGATATAGCATTTCCACGAATAAATAATATAAGATTTTGACTTTTACCCCCCTCAT  278
             |||||||||||||||||||||||||||||||||||||||||||||||||||| || ||| 
Sbjct  181   CCCCAGATATAGCATTTCCACGAATAAATAATATAAGATTTTGACTTTTACCTCCTTCAC  240

Query  279   TATTTTTATTGATTAACTCTTCATTAATTGAATCCGGAGCTGGAACAGGATGAACAGTTT  338
             |||||||||| ||||| ||||||||||||||||| |||||||| ||||||||||||||||
Sbjct  241   TATTTTTATTAATTAATTCTTCATTAATTGAATCTGGAGCTGGTACAGGATGAACAGTTT  300

Query  339   ATCCCCCTCTATCTTCAAATTTATCACATTATGGTCCATCAGTAGATTTAGCTATTTTTT  398
             | || ||||||||||||||||||||||||||||| || ||||||||||||||||||||||
Sbjct  301   ACCCTCCTCTATCTTCAAATTTATCACATTATGGCCCTTCAGTAGATTTAGCTATTTTTT  360

Query  399   CTCTTCACCTTGCTGGTGCTTCTTCAATTTTAGGTGCAATTAACTTCATCACAACTATTG  458
             ||||||| |||||||||||||||||||||||||||||||||||||| || ||||||||||
Sbjct  361   CTCTTCATCTTGCTGGTGCTTCTTCAATTTTAGGTGCAATTAACTTTATTACAACTATTG  420
```

```
Query  459   TAAATATACGATCAATTGGAATAACAATAGAACGAATACCATTATTTGTTTGATCTGTTT  518
             |||| ||||||||||||||||||||||||||||| |||||||||||||||||||||||||
Sbjct  421   TAAACATACGATCAATTGGAATAACAATAGAACGTATACCATTATTTGTTTGATCTGTTT  480

Query  519   TAATTACAGCTATTTTACTACTTCTATCTTTACCTGTATTAGCAGGTGCCATTACAATAT  578
             ||||||||||||||||| ||||| ||||||||||||||||||||||||||||||||||||
Sbjct  481   TAATTACAGCTATTTTATTACTTTTATCTTTACCTGTATTAGCAGGTGCCATTACAATAT  540

Query  579   TATTAACTGATCGAAACTTTAACACATCATTTTTTGATCCTTCAGGAGGAGGAGATCCCA  638
             | |||||||||||||||||||| ||||||||||||||||||||||| ||||||||||| |
Sbjct  541   TGTTAACTGATCGAAACTTTAATACATCATTTTTTGATCCTTCAGGGGGAGGAGATCCAA  600

Query  639   TTTTATATCAACACTTATTT  658
             ||||||||||||| ||||||
Sbjct  601   TTTTATATCAACATTTATTT  620
```

>Rhipicephalus sanguineus isolate Sibenik cytochrome oxidase subunit I (COI) gene, partial cds; mitochondrial
Sequence ID: KX757895.1 Length: 630
Range 1: 1 to 620

Score:985 bits(533), Expect:0.0,
Identities:591/620(95%), Gaps:0/620(0%), Strand: Plus/Plus

```
Query  39    TAGGACTTAGTATAAGAATATTAATTCGTATAGAATTAGGACAACCTGGAACTTTAATTG  98
             |||||||||||||||||||||||||||||||||| |||||||||||||||||||||||||
Sbjct  1     TAGGACTTAGTATAAGAATATTAATTCGTATAGAGTTAGGACAACCTGGAACTTTAATTG  60

Query  99    GAAATGATCAGATTTATAATGTAATCGTAACAGCACATGCATTTATTATAATTTTCTTTA  158
             ||||||||||||||||||||||||||||||||||||||||||||||||||||||| ||||
Sbjct  61    GAAATGATCAGATTTATAATGTAATCGTAACAGCACATGCATTTATTATAATTTTTTTTA  120

Query  159   TAGTTATACCAATCATAATCGGAGGGTTCGGAAATTGATTAGTTCCTATTATATTAGGTG  218
             ||||||||||||||||||||||||| || |||||||||||||| ||||| ||||| ||||
Sbjct  121   TAGTTATACCAATCATAATCGGAGGATTTGGAAATTGATTAGTCCCTATCATATTGGGTG  180

Query  219   CCCCAGATATAGCATTTCCACGAATAAATAATATAAGATTTTGACTTTTACCCCCCTCAT  278
             |||||||||||||||||||||||||||||||||||||||||||||||||||| || |||
Sbjct  181   CCCCAGATATAGCATTTCCACGAATAAATAATATAAGATTTTGACTTTTACCTCCTTCAC  240

Query  279   TATTTTTATTGATTAACTCTTCATTAATTGAATCCGGAGCTGGAACAGGATGAACAGTTT  338
             |||||||||| ||||||||||||||||||||||| |||||||| ||||||||||||||||
Sbjct  241   TATTTTTATTAATTAACTCTTCATTAATTGAATCTGGAGCTGGTACAGGATGAACAGTTT  300

Query  339   ATCCCCCTCTATCTTCAAATTTATCACATTATGGTCCATCAGTAGATTTAGCTATTTTTT  398
             | || ||||||||||||||||||||||||||||| || ||||||||||||||||||||||
Sbjct  301   ACCCTCCTCTATCTTCAAATTTATCACATTATGGCCCTTCAGTAGATTTAGCTATTTTTT  360

Query  399   CTCTTCACCTTGCTGGTGCTTCTTCAATTTTAGGTGCAATTAACTTCATCACAACTATTG  458
             ||||||| |||||||||||||||||||||||||||||||||||||| || ||||||||||
```

```
Sbjct  361   CTCTTCATCTTGCTGGTGCTTCTTCAATTTTAGGTGCAATTAACTTTATTACAACTATTG  420

Query  459   TAAATATACGATCAATTGGAATAACAATAGAACGAATACCATTATTTGTTTGATCTGTTT  518
             |||| ||||||||||||||||||||||||||||| |||||||||||||||||||||||||
Sbjct  421   TAAACATACGATCAATTGGAATAACAATAGAACGTATACCATTATTTGTTTGATCTGTTT  480

Query  519   TAATTACAGCTATTTTACTACTTCTATCTTTACCTGTATTAGCAGGTGCCATTACAATAT  578
             ||||||||||||||||| ||||| ||||||||||||||||||||||||||||||||||||
Sbjct  481   TAATTACAGCTATTTTATTACTTTTATCTTTACCTGTATTAGCAGGTGCCATTACAATAT  540

Query  579   TATTAACTGATCGAAACTTTAACACATCATTTTTTGATCCTTCAGGAGGAGGAGATCCCA  638
             | |||||||||||||||||||| ||||||||||||||||||||||| ||||||||||| |
Sbjct  541   TGTTAACTGATCGAAACTTTAATACATCATTTTTTGATCCTTCAGGGGGAGGAGATCCAA  600

Query  639   TTTTATATCAACACTTATTT  658
             ||||||||||||| ||||||
Sbjct  601   TTTTATATCAACATTTATTT  620
```

>Rhipicephalus sanguineus isolate Zadar cytochrome oxidase subunit I (COI) gene, partial cds; mitochondrial
Sequence ID: KX757892.1 Length: 630
Range 1: 1 to 620

Score:985 bits(533), Expect:0.0,
Identities:591/620(95%), Gaps:0/620(0%), Strand: Plus/Plus

```
Query  39    TAGGACTTAGTATAAGAATATTAATTCGTATAGAATTAGGACAACCTGGAACTTTAATTG  98
             ||||||||||||||||||||||||||||||||| ||||||||||||||||||||||||||
Sbjct  1     TAGGACTTAGTATAAGAATATTAATTCGTATAGAGTTAGGACAACCTGGAACTTTAATTG  60

Query  99    GAAATGATCAGATTTATAATGTAATCGTAACAGCACATGCATTTATTATAATTTTCTTTA  158
             ||||||||||||||||||||||||||||||||||||||||||||||||||||||| ||||
Sbjct  61    GAAATGATCAGATTTATAATGTAATCGTAACAGCACATGCATTTATTATAATTTTTTTTA  120

Query  159   TAGTTATACCAATCATAATCGGAGGGTTCGGAAATTGATTAGTTCCTATTATATTAGGTG  218
             ||||||||||||||||||||||||| || |||||||||||||| ||||| ||||| ||||
Sbjct  121   TAGTTATACCAATCATAATCGGAGGATTTGGAAATTGATTAGTCCCTATCATATTGGGTG  180

Query  219   CCCCAGATATAGCATTTCCACGAATAAATAATATAAGATTTTGACTTTACCCCCCTCAT  278
             |||||||||||||||||||||||||||||||||||||||||||||||||||| || |||
Sbjct  181   CCCCAGATATAGCATTTCCACGAATAAATAATATAAGATTTTGACTTTACCTCCTTCAC  240

Query  279   TATTTTTATTGATTAACTCTTCATTAATTGAATCCGGAGCTGGAACAGGATGAACAGTTT  338
             |||||||||| ||||||||||||||||||||||| |||||||| ||||||||||||||||
Sbjct  241   TATTTTTATTAATTAACTCTTCATTAATTGAATCTGGAGCTGGTACAGGATGAACAGTTT  300

Query  339   ATCCCCCTCTATCTTCAAATTTATCACATTATGGTCCATCAGTAGATTTAGCTATTTTTT  398
             | || ||||||||||||||||||||||||||||| || ||||||||||||||||||||||
Sbjct  301   ACCCTCCTCTATCTTCAAATTTATCACATTATGGCCCTTCAGTAGATTTAGCTATTTTTT  360

Query  399   CTCTTCACCTTGCTGGTGCTTCTTCAATTTTAGGTGCAATTAACTTCATCACAACTATTG  458
```

```
            ||||||| |||||||||||||||||||||||||||||||||||||| || ||||||||||
Sbjct  361  CTCTTCATCTTGCTGGTGCTTCTTCAATTTTAGGTGCAATTAACTTTATTACAACTATTG  420

Query  459  TAAATATACGATCAATTGGAATAACAATAGAACGAATACCATTATTTGTTTGATCTGTTT  518
            |||| ||||||||||||||||||||||||||||| |||||||||||||||||||||||||
Sbjct  421  TAAACATACGATCAATTGGAATAACAATAGAACGTATACCATTATTTGTTTGATCTGTTT  480

Query  519  TAATTACAGCTATTTTACTACTTCTATCTTTACCTGTATTAGCAGGTGCCATTACAATAT  578
            ||||||||||||||||| ||||| ||||||||||||||||||||||||||||||||||||
Sbjct  481  TAATTACAGCTATTTTATTACTTTTATCTTTACCTGTATTAGCAGGTGCCATTACAATAT  540

Query  579  TATTAACTGATCGAAACTTTAACACATCATTTTTTGATCCTTCAGGAGGAGGAGATCCCA  638
            | |||||||||||||||||||| ||||||||||||||||||||||| ||||||||||| |
Sbjct  541  TGTTAACTGATCGAAACTTTAATACATCATTTTTTGATCCTTCAGGGGGAGGAGATCCAA  600

Query  639  TTTTATATCAACACTTATTT  658
            ||||||||||||| ||||||
Sbjct  601  TTTTATATCAACATTTATTT  620
```

>Rhipicephalus sanguineus isolate Zagreb-A cytochrome oxidase subunit I (COI) gene, partial cds; mitochondrial
Sequence ID: KX757890.1 Length: 630
Range 1: 1 to 620

Score:985 bits(533), Expect:0.0,
Identities:591/620(95%), Gaps:0/620(0%), Strand: Plus/Plus

```
Query  39   TAGGACTTAGTATAAGAATATTAATTCGTATAGAATTAGGACAACCTGGAACTTTAATTG  98
            |||||||||||||||||||||||||||||||||||| |||||||||||||||||||||||
Sbjct  1    TAGGACTTAGTATAAGAATATTAATTCGTATAGAGTTAGGACAACCTGGAACTTTAATTG  60

Query  99   GAAATGATCAGATTTATAATGTAATCGTAACAGCACATGCATTTATTATAATTTTCTTTA  158
            ||||||||||||||||||||||||||||||||||||||||||||||||||||||| ||||
Sbjct  61   GAAATGATCAGATTTATAATGTAATCGTAACAGCACATGCATTTATTATAATTTTTTTTA  120

Query  159  TAGTTATACCAATCATAATCGGAGGGTTCGGAAATTGATTAGTTCCTATTATATTAGGTG  218
            ||||||||||||||||||||||||| || |||||||||||||| ||||| ||||| ||||
Sbjct  121  TAGTTATACCAATCATAATCGGAGGATTTGGAAATTGATTAGTCCCTATCATATTGGGTG  180

Query  219  CCCCAGATATAGCATTTCCACGAATAAATAATATAAGATTTTGACTTTTACCCCCCTCAT  278
            |||||||||||||||||||||||||||||||||||||||||||||||||||| || |||
Sbjct  181  CCCCAGATATAGCATTTCCACGAATAAATAATATAAGATTTTGACTTTTACCTCCTTCAC  240

Query  279  TATTTTTATTGATTAACTCTTCATTAATTGAATCCGGAGCTGGAACAGGATGAACAGTTT  338
            |||||||||| ||||||||||||||||||||||| |||||||| ||||||||||||||||
Sbjct  241  TATTTTTATTAATTAACTCTTCATTAATTGAATCTGGAGCTGGTACAGGATGAACAGTTT  300

Query  339  ATCCCCCTCTATCTTCAAATTTATCACATTATGGTCCATCAGTAGATTTAGCTATTTTTT  398
            | || ||||||||||||||||||||||||||||| || ||||||||||||||||||||||
Sbjct  301  ACCCTCCTCTATCTTCAAATTTATCACATTATGGCCCTTCAGTAGATTTAGCTATTTTTT  360
```

```
Query  399  CTCTTCACCTTGCTGGTGCTTCTTCAATTTTAGGTGCAATTAACTTCATCACAACTATTG  458
            ||||||| |||||||||||||||||||||||||||||||||||||| || ||||||||||
Sbjct  361  CTCTTCATCTTGCTGGTGCTTCTTCAATTTTAGGTGCAATTAACTTTATTACAACTATTG  420

Query  459  TAAATATACGATCAATTGGAATAACAATAGAACGAATACCATTATTTGTTTGATCTGTTT  518
            |||| ||||||||||||||||||||||||||||| |||||||||||||||||||||||||
Sbjct  421  TAAACATACGATCAATTGGAATAACAATAGAACGTATACCATTATTTGTTTGATCTGTTT  480

Query  519  TAATTACAGCTATTTTACTACTTCTATCTTTACCTGTATTAGCAGGTGCCATTACAATAT  578
            ||||||||||||||||| ||||| ||||||||||||||||||||||||||||||||||||
Sbjct  481  TAATTACAGCTATTTTATTACTTTTATCTTTACCTGTATTAGCAGGTGCCATTACAATAT  540

Query  579  TATTAACTGATCGAAACTTTAACACATCATTTTTTGATCCTTCAGGAGGAGGAGATCCCA  638
            | |||||||||||||||||||| ||||||||||||||||||||||| ||||||||||| |
Sbjct  541  TGTTAACTGATCGAAACTTTAATACATCATTTTTTGATCCTTCAGGGGGAGGAGATCCAA  600

Query  639  TTTTATATCAACACTTATTT  658
            ||||||||||||| ||||||
Sbjct  601  TTTTATATCAACATTTATTT  620
```

>Rhipicephalus sanguineus isolate Pula-A cytochrome oxidase subunit I (COI) gene, partial cds; mitochondrial
Sequence ID: KX757888.1 Length: 630
Range 1: 1 to 620

Score:985 bits(533), Expect:0.0,
Identities:591/620(95%), Gaps:0/620(0%), Strand: Plus/Plus

```
Query  39   TAGGACTTAGTATAAGAATATTAATTCGTATAGAATTAGGACAACCTGGAACTTTAATTG  98
            ||||||||||||||||||||||||||||||||||||||||||||||||||||||||||||
Sbjct  1    TAGGACTTAGTATAAGAATATTAATTCGTATAGAATTAGGACAACCTGGAACTTTAATTG  60

Query  99   GAAATGATCAGATTTATAATGTAATCGTAACAGCACATGCATTTATTATAATTTTCTTTA  158
            ||||||||||||||||||||||||||||||||||||||||||||||||||||||| ||||
Sbjct  61   GAAATGATCAGATTTATAATGTAATCGTAACAGCACATGCATTTATTATAATTTTTTTTA  120

Query  159  TAGTTATACCAATCATAATCGGAGGGTTCGGAAATTGATTAGTTCCTATTATATTAGGTG  218
            ||||||||||||||||||||||||| || |||||||||||||| ||||| ||||| ||||
Sbjct  121  TAGTTATACCAATCATAATCGGAGGATTTGGAAATTGATTAGTCCCTATCATATTGGGTG  180

Query  219  CCCCAGATATAGCATTTCCACGAATAAATAATATAAGATTTTGACTTTTACCCCCCTCAT  278
            |||||||||||||||||||||||||||||||||||||||||||||||||||| || ||| 
Sbjct  181  CCCCAGATATAGCATTTCCACGAATAAATAATATAAGATTTTGACTTTTACCTCCTTCAC  240

Query  279  TATTTTTATTGATTAACTCTTCATTAATTGAATCCGGAGCTGGAACAGGATGAACAGTTT  338
            |||||||||| ||||| ||||||||||||||||| |||||||| ||||||||||||||||
Sbjct  241  TATTTTTATTAATTAATTCTTCATTAATTGAATCTGGAGCTGGTACAGGATGAACAGTTT  300

Query  339  ATCCCCCTCTATCTTCAAATTTATCACATTATGGTCCATCAGTAGATTTAGCTATTTTTT  398
            | || ||||||||||||||||||||||||||||| || ||||||||||||||||||||||
Sbjct  301  ACCCTCCTCTATCTTCAAATTTATCACATTATGGCCCTTCAGTAGATTTAGCTATTTTTT  360
```

```
Query  399   CTCTTCACCTTGCTGGTGCTTCTTCAATTTTAGGTGCAATTAACTTCATCACAACTATTG  458
             ||||||| |||||||||||||||||||||||||||||||||||||| || ||||||||||
Sbjct  361   CTCTTCATCTTGCTGGTGCTTCTTCAATTTTAGGTGCAATTAACTTTATTACAACTATTG  420

Query  459   TAAATATACGATCAATTGGAATAACAATAGAACGAATACCATTATTTGTTTGATCTGTTT  518
             |||| ||||||||||||||||||||||||||||| |||||||||||||||||||||||||
Sbjct  421   TAAACATACGATCAATTGGAATAACAATAGAACGTATACCATTATTTGTTTGATCTGTTT  480

Query  519   TAATTACAGCTATTTTACTACTTCTATCTTTACCTGTATTAGCAGGTGCCATTACAATAT  578
             ||||||||||||||||| ||||| ||||||||||||||||||||||||||||||||||||
Sbjct  481   TAATTACAGCTATTTTATTACTTTTATCTTTACCTGTATTAGCAGGTGCCATTACAATAT  540

Query  579   TATTAACTGATCGAAACTTTAACACATCATTTTTTGATCCTTCAGGAGGAGGAGATCCCA  638
             | |||||||||||||||||||| ||||||||||||||||||||||| ||||||||||| |
Sbjct  541   TGTTAACTGATCGAAACTTTAATACATCATTTTTTGATCCTTCAGGGGGAGGAGATCCAA  600

Query  639   TTTTATATCAACACTTATTT  658
             ||||||||||||| ||||||
Sbjct  601   TTTTATATCAACATTTATTT  620
```

>Rhipicephalus sanguineus isolate Petnica cytochrome oxidase subunit I (COI) gene, partial cds; mitochondrial
Sequence ID: KX757883.1 Length: 630
Range 1: 1 to 620

Score:985 bits(533), Expect:0.0,
Identities:591/620(95%), Gaps:0/620(0%), Strand: Plus/Plus

```
Query  39    TAGGACTTAGTATAAGAATATTAATTCGTATAGAATTAGGACAACCTGGAACTTTAATTG  98
             |||||||||||||||||||||||||||||||||| |||||||||||||||||||||||||
Sbjct  1     TAGGACTTAGTATAAGAATATTAATTCGTATAGAGTTAGGACAACCTGGAACTTTAATTG  60

Query  99    GAAATGATCAGATTTATAATGTAATCGTAACAGCACATGCATTTATTATAATTTTCTTTA  158
             ||||||||||||||||||||||||||||||||||||||||||||||||||||||| ||||
Sbjct  61    GAAATGATCAGATTTATAATGTAATCGTAACAGCACATGCATTTATTATAATTTTTTTTA  120

Query  159   TAGTTATACCAATCATAATCGGAGGGTTCGGAAATTGATTAGTTCCTATTATATTAGGTG  218
             ||||||||||||||||||||||||| || |||||||||||||| ||||| ||||| ||||
Sbjct  121   TAGTTATACCAATCATAATCGGAGGATTTGGAAATTGATTAGTCCCTATCATATTGGGTG  180

Query  219   CCCCAGATATAGCATTTCCACGAATAAATAATATAAGATTTTGACTTTTACCCCCCTCAT  278
             |||||||||||||||||||||||||||||||||||||||||||||||||||| || ||| 
Sbjct  181   CCCCAGATATAGCATTTCCACGAATAAATAATATAAGATTTTGACTTTTACCTCCTTCAC  240

Query  279   TATTTTTATTGATTAACTCTTCATTAATTGAATCCGGAGCTGGAACAGGATGAACAGTTT  338
             |||||||||| ||||||||||||||||||||||| |||||||| ||||||||||||||||
Sbjct  241   TATTTTTATTAATTAACTCTTCATTAATTGAATCTGGAGCTGGTACAGGATGAACAGTTT  300

Query  339   ATCCCCCTCTATCTTCAAATTTATCACATTATGGTCCATCAGTAGATTTAGCTATTTTTT  398
             | || ||||||||||||||||||||||||||||| || ||||||||||||||||||||||
```

```
Sbjct  301  ACCCTCCTCTATCTTCAAATTTATCACATTATGGCCCTTCAGTAGATTTAGCTATTTTTT  360

Query  399  CTCTTCACCTTGCTGGTGCTTCTTCAATTTTAGGTGCAATTAACTTCATCACAACTATTG  458
            ||||||| |||||||||||||||||||||||||||||||||||||| || ||||||||||
Sbjct  361  CTCTTCATCTTGCTGGTGCTTCTTCAATTTTAGGTGCAATTAACTTTATTACAACTATTG  420

Query  459  TAAATATACGATCAATTGGAATAACAATAGAACGAATACCATTATTTGTTTGATCTGTTT  518
            |||| ||||||||||||||||||||||||||||| |||||||||||||||||||||||||
Sbjct  421  TAAACATACGATCAATTGGAATAACAATAGAACGTATACCATTATTTGTTTGATCTGTTT  480

Query  519  TAATTACAGCTATTTTACTACTTCTATCTTTACCTGTATTAGCAGGTGCCATTACAATAT  578
            ||||||||||||||||| ||||| ||||||||||||||||||||||||||||||||||||
Sbjct  481  TAATTACAGCTATTTTATTACTTTTATCTTTACCTGTATTAGCAGGTGCCATTACAATAT  540

Query  579  TATTAACTGATCGAAACTTTAACACATCATTTTTTGATCCTTCAGGAGGAGGAGATCCCA  638
            | |||||||||||||||||||| ||||||||||||||||||||||| ||||||||||| |
Sbjct  541  TGTTAACTGATCGAAACTTTAATACATCATTTTTTGATCCTTCAGGGGGAGGAGATCCAA  600

Query  639  TTTTATATCAACACTTATTT  658
            ||||||||||||| ||||||
Sbjct  601  TTTTATATCAACATTTATTT  620
```

>Rhipicephalus sanguineus clone #3 cytochrome oxidase subunit I (COI) gene,
partial cds; mitochondrial
Sequence ID: KX519708.1 Length: 629
Range 1: 4 to 620

Score:985 bits(533), Expect:0.0,
Identities:589/617(95%), Gaps:0/617(0%), Strand: Plus/Plus

```
Query  42   GACTTAGTATAAGAATATTAATTCGTATAGAATTAGGACAACCTGGAACTTTAATTGGAA  101
            ||||||||||||||||||||||||||||||||||||||||||||||||||||||||||||
Sbjct  4    GACTTAGTATAAGAATATTAATTCGTATAGAATTAGGACAACCTGGAACTTTAATTGGAA  63

Query  102  ATGATCAGATTTATAATGTAATCGTAACAGCACATGCATTTATTATAATTTTCTTTATAG  161
            |||||||||||||||||||||||||||||||||||||||||||||||||||| |||||||
Sbjct  64   ATGATCAGATTTATAATGTAATCGTAACAGCACATGCATTTATTATAATTTTTTTTATAG  123

Query  162  TTATACCAATCATAATCGGAGGGTTCGGAAATTGATTAGTTCCTATTATATTAGGTGCCC  221
            |||||||||||||||||||||| || |||||||||||||| ||||| ||||| |||||||
Sbjct  124  TTATACCAATCATAATCGGAGGATTTGGAAATTGATTAGTCCCTATCATATTGGGTGCCC  183

Query  222  CAGATATAGCATTTCCACGAATAAATAATATAAGATTTTGACTTTTACCCCCCTCATTAT  281
            ||||||||||||||||||||||||||||||||||||||||||||||||| || ||| |||
Sbjct  184  CAGATATAGCATTTCCACGAATAAATAATATAAGATTTTGACTTTTACCTCCTTCACTAT  243

Query  282  TTTTATTGATTAACTCTTCATTAATTGAATCCGGAGCTGGAACAGGATGAACAGTTTATC  341
            ||||||| ||||||||||||||||||||||| |||||||| ||||||||||||||||| |
Sbjct  244  TTTTATTAATTAACTCTTCATTAATTGAATCTGGAGCTGGTACAGGATGAACAGTTTACC  303

Query  342  CCCCTCTATCTTCAAATTTATCACATTATGGTCCATCAGTAGATTTAGCTATTTTTTCTC  401
```

```
            | ||||||||||||||||||||||||||||| || |||||||||||||||||||||||||
Sbjct  304  CTCCTCTATCTTCAAATTTATCACATTATGGCCCTTCAGTAGATTTAGCTATTTTTTCTC  363

Query  402  TTCACCTTGCTGGTGCTTCTTCAATTTTAGGTGCAATTAACTTCATCACAACTATTGTAA  461
            |||| |||||||||||||||||||||||||||||||||||||| || |||||||||||||
Sbjct  364  TTCATCTTGCTGGTGCTTCTTCAATTTTAGGTGCAATTAACTTTATTACAACTATTGTAA  423

Query  462  ATATACGATCAATTGGAATAACAATAGAACGAATACCATTATTTGTTTGATCTGTTTTAA  521
            | ||||||||||||||||||||||||||||| ||||||||||||||||||||||||||||
Sbjct  424  ACATACGATCAATTGGAATAACAATAGAACGTATACCATTATTTGTTTGATCTGTTTTAA  483

Query  522  TTACAGCTATTTTACTACTTCTATCTTTACCTGTATTAGCAGGTGCCATTACAATATTAT  581
            |||||||||||||| ||||| ||||||||||||||||||||||||||||||||||||| |
Sbjct  484  TTACAGCTATTTTATTACTTTTATCTTTACCTGTATTAGCAGGTGCCATTACAATATTGT  543

Query  582  TAACTGATCGAAACTTTAACACATCATTTTTTGATCCTTCAGGAGGAGGAGATCCCATTT  641
            ||||||||||||||||||| ||||||||||||||||||||||| ||||||||||| ||||
Sbjct  544  TAACTGATCGAAACTTTAATACATCATTTTTTGATCCTTCAGGGGGAGGAGATCCAATTT  603

Query  642  TATATCAACACTTATTT  658
            |||||||||| ||||||
Sbjct  604  TATATCAACATTTATTT  620
```

>Rhipicephalus sanguineus isolate SV2 cytochrome c oxidase subunit I (COX1) gene, partial cds; mitochondrial
Sequence ID: MW135447.1 Length: 630
Range 1: 1 to 620

Score:985 bits(533), Expect:0.0,
Identities:591/620(95%), Gaps:0/620(0%), Strand: Plus/Plus

```
Query  39   TAGGACTTAGTATAAGAATATTAATTCGTATAGAATTAGGACAACCTGGAACTTTAATTG  98
            ||||||||||||||||||||||||||||||||||||||||||||||||||||||||||||
Sbjct  1    TAGGACTTAGTATAAGAATATTAATTCGTATAGAATTAGGACAACCTGGAACTTTAATTG  60

Query  99   GAAATGATCAGATTTATAATGTAATCGTAACAGCACATGCATTTATTATAATTTTCTTTA  158
            ||||||||||||||||||||||||||||||||||||||||||||||||||||||| ||||
Sbjct  61   GAAATGATCAGATTTATAATGTAATCGTAACAGCACATGCATTTATTATAATTTTTTTTA  120

Query  159  TAGTTATACCAATCATAATCGGAGGGTTCGGAAATTGATTAGTTCCTATTATATTAGGTG  218
            ||||||||||||||||||||||||| || |||||||||||||| ||||| ||||| ||||
Sbjct  121  TAGTTATACCAATCATAATCGGAGGATTTGGAAATTGATTAGTCCCTATCATATTGGGTG  180

Query  219  CCCCAGATATAGCATTTCCACGAATAAATAATATAAGATTTTGACTTTTACCCCCCTCAT  278
            |||||||||||||||||||||||||||||||||||||||||||||||||||| || |||
Sbjct  181  CCCCAGATATAGCATTTCCACGAATAAATAATATAAGATTTTGACTTTTACCTCCTTCAC  240

Query  279  TATTTTTATTGATTAACTCTTCATTAATTGAATCCGGAGCTGGAACAGGATGAACAGTTT  338
            |||||||||| ||||||||||||||||||||||| |||||||| ||||||||||||||||
Sbjct  241  TATTTTTATTAATTAACTCTTCATTAATTGAATCTGGAGCTGGTACAGGATGAACAGTTT  300
```

```
Query  339  ATCCCCCTCTATCTTCAAATTTATCACATTATGGTCCATCAGTAGATTTAGCTATTTTTT  398
            | || ||||||||||||||||||||||||||||| || ||||||||||||||||||||||
Sbjct  301  ACCCTCCTCTATCTTCAAATTTATCACATTATGGCCCTTCAGTAGATTTAGCTATTTTTT  360

Query  399  CTCTTCACCTTGCTGGTGCTTCTTCAATTTTAGGTGCAATTAACTTCATCACAACTATTG  458
            ||||||| |||||||||||||||||||||||||||||||||||||| || ||||||||||
Sbjct  361  CTCTTCATCTTGCTGGTGCTTCTTCAATTTTAGGTGCAATTAACTTTATTACAACTATTG  420

Query  459  TAAATATACGATCAATTGGAATAACAATAGAACGAATACCATTATTTGTTTGATCTGTTT  518
            |||| ||||||||||||||||||||||||||||||| |||||||||||||||||||||||
Sbjct  421  TAAACATACGATCAATTGGAATAACAATAGAACGTATACCATTATTTGTTTGATCTGTTT  480

Query  519  TAATTACAGCTATTTTACTACTTCTATCTTTACCTGTATTAGCAGGTGCCATTACAATAT  578
            ||||||||||||||||| ||||| ||||||||||||||||||||||||||||||||||||
Sbjct  481  TAATTACAGCTATTTTATTACTTTTATCTTTACCTGTATTAGCAGGTGCCATTACAATAT  540

Query  579  TATTAACTGATCGAAACTTTAACACATCATTTTTTGATCCTTCAGGAGGAGGAGATCCCA  638
            | |||||||||||||||||||| |||||||||||||||||||||||| |||||||| || |
Sbjct  541  TGTTAACTGATCGAAACTTTAATACATCATTTTTTGATCCTTCAGGGGGAGGAGACCCAA  600

Query  639  TTTTATATCAACACTTATTT  658
            ||||||||||||| ||||||
Sbjct  601  TTTTATATCAACATTTATTT  620
```

>Rhipicephalus sanguineus clone #2 cytochrome oxidase subunit I (COI) gene, partial cds; mitochondrial
Sequence ID: KX519707.1 Length: 626
Range 1: 1 to 616

Score:983 bits(532), Expect:0.0,
Identities:588/616(95%), Gaps:0/616(0%), Strand: Plus/Plus

```
Query  43   ACTTAGTATAAGAATATTAATTCGTATAGAATTAGGACAACCTGGAACTTTAATTGGAAA  102
            ||||||||||||||||||||||||||||||||||||||||||||||||||||||||||||
Sbjct  1    ACTTAGTATAAGAATATTAATTCGTATAGAATTAGGACAACCTGGAACTTTAATTGGAAA  60

Query  103  TGATCAGATTTATAATGTAATCGTAACAGCACATGCATTTATTATAATTTTCTTTATAGT  162
            ||||||||||||||||||||||||||||||||||||||||||||||||||| ||||||||
Sbjct  61   TGATCAGATTTATAATGTAATCGTAACAGCACATGCATTTATTATAATTTTTTTTATAGT  120

Query  163  TATACCAATCATAATCGGAGGGTTCGGAAATTGATTAGTTCCTATTATATTAGGTGCCCC  222
            |||||||||||||||||||| || ||||||||||||||| ||||| ||||| ||||||||
Sbjct  121  TATACCAATCATAATCGGAGGATTTGGAAATTGATTAGTCCCTATCATATTGGGTGCCCC  180

Query  223  AGATATAGCATTTCCACGAATAAATAATATAAGATTTTGACTTTTACCCCCCTCATTATT  282
            |||||||||||||||||||||||||||||||||||||||||||||||| || ||| ||||
Sbjct  181  AGATATAGCATTTCCACGAATAAATAATATAAGATTTTGACTTTTACCTCCTTCACTATT  240

Query  283  TTTATTGATTAACTCTTCATTAATTGAATCCGGAGCTGGAACAGGATGAACAGTTTATCC  342
            |||||| ||||||||||||||||||||||| |||||||| ||||||||||||||||| ||
Sbjct  241  TTTATTAATTAACTCTTCATTAATTGAATCTGGAGCTGGTACAGGATGAACAGTTTACCC  300
```

```
Query  343   CCCTCTATCTTCAAATTTATCACATTATGGTCCATCAGTAGATTTAGCTATTTTTTCTCT  402
              ||||||||||||||||||||||||||||| || ||||||||||||||||||||||||||
Sbjct  301   TCCTCTATCTTCAAATTTATCACATTATGGCCCTTCAGTAGATTTAGCTATTTTTTCTCT  360

Query  403   TCACCTTGCTGGTGCTTCTTCAATTTTAGGTGCAATTAACTTCATCACAACTATTGTAAA  462
             ||| |||||||||||||||||||||||||||||||||||||| || ||||||||||||||
Sbjct  361   TCATCTTGCTGGTGCTTCTTCAATTTTAGGTGCAATTAACTTTATTACAACTATTGTAAA  420

Query  463   TATACGATCAATTGGAATAACAATAGAACGAATACCATTATTTGTTTGATCTGTTTTAAT  522
              ||||||||||||||||||||||||||||| |||||||||||||||||||||||||||||
Sbjct  421   CATACGATCAATTGGAATAACAATAGAACGTATACCATTATTTGTTTGATCTGTTTTAAT  480

Query  523   TACAGCTATTTTACTACTTCTATCTTTACCTGTATTAGCAGGTGCCATTACAATATTATT  582
             ||||||||||||| ||||| ||||||||||||||||||||||||||||||||||||| ||
Sbjct  481   TACAGCTATTTTATTACTTTTATCTTTACCTGTATTAGCAGGTGCCATTACAATATTGTT  540

Query  583   AACTGATCGAAACTTTAACACATCATTTTTTGATCCTTCAGGAGGAGGAGATCCCATTTT  642
             |||||||||||||||||| ||||||||||||||||||||||| ||||||||||| |||||
Sbjct  541   AACTGATCGAAACTTTAATACATCATTTTTTGATCCTTCAGGGGGAGGAGATCCAATTTT  600

Query  643   ATATCAACACTTATTT  658
             ||||||||| ||||||
Sbjct  601   ATATCAACATTTATTT  616
```

>Rhipicephalus sanguineus isolate Szekszard cytochrome oxidase subunit I (COI) gene, partial cds; mitochondrial
Sequence ID: KX757901.1 Length: 630
Range 1: 1 to 620

Score:979 bits(530), Expect:0.0,
Identities:590/620(95%), Gaps:0/620(0%), Strand: Plus/Plus

```
Query  39    TAGGACTTAGTATAAGAATATTAATTCGTATAGAATTAGGACAACCTGGAACTTTAATTG  98
             ||||||||||||||||||||||||||||||||||||||||||||||||||||||||||||
Sbjct  1     TAGGACTTAGTATAAGAATATTAATTCGTATAGAATTAGGACAACCTGGAACTTTAATTG  60

Query  99    GAAATGATCAGATTTATAATGTAATCGTAACAGCACATGCATTTATTATAATTTTCTTTA  158
             ||||||||||||||||||||||||||||||||||||||||||||||||||||||| ||||
Sbjct  61    GAAATGATCAGATTTATAATGTAATCGTAACAGCACATGCATTTATTATAATTTTTTTTA  120

Query  159   TAGTTATACCAATCATAATCGGAGGGTTCGGAAATTGATTAGTTCCTATTATATTAGGTG  218
             ||||||||||||||||||||||||| || |||||||||||||| ||||| ||||| ||||
Sbjct  121   TAGTTATACCAATCATAATCGGAGGATTTGGAAATTGATTAGTCCCTATCATATTGGGTG  180

Query  219   CCCCAGATATAGCATTTCCACGAATAAATAATATAAGATTTTGACTTTTACCCCCCTCAT  278
             |||||||||||||||||||||||||||||||||||||||||||||||||||| || |||
Sbjct  181   CCCCAGATATAGCATTTCCACGAATAAATAATATAAGATTTTGACTTTTACCTCCTTCAC  240

Query  279   TATTTTTATTGATTAACTCTTCATTAATTGAATCCGGAGCTGGAACAGGATGAACAGTTT  338
             |||||||||| ||||||||||||||||||||||| |||||||| ||||||||||||||||
```

```
Sbjct  241  TATTTTTATTAATTAACTCTTCATTAATTGAATCTGGAGCTGGTACAGGATGAACAGTTT  300

Query  339  ATCCCCCTCTATCTTCAAATTTATCACATTATGGTCCATCAGTAGATTTAGCTATTTTTT  398
            | || ||||||||||||||||||||||||||||| || ||||||||||||||||| ||||
Sbjct  301  ACCCTCCTCTATCTTCAAATTTATCACATTATGGCCCTTCAGTAGATTTAGCTATCTTTT  360

Query  399  CTCTTCACCTTGCTGGTGCTTCTTCAATTTTAGGTGCAATTAACTTCATCACAACTATTG  458
            ||||||| |||||||||||||||||||||||||||||||||||||| || ||||||||||
Sbjct  361  CTCTTCATCTTGCTGGTGCTTCTTCAATTTTAGGTGCAATTAACTTTATTACAACTATTG  420

Query  459  TAAATATACGATCAATTGGAATAACAATAGAACGAATACCATTATTTGTTTGATCTGTTT  518
            |||| ||||| ||||||||||||||||||||||| |||||||||||||||||||||||||
Sbjct  421  TAAACATACGGTCAATTGGAATAACAATAGAACGTATACCATTATTTGTTTGATCTGTTT  480

Query  519  TAATTACAGCTATTTTACTACTTCTATCTTTACCTGTATTAGCAGGTGCCATTACAATAT  578
            ||||||||||||||||| ||||| ||||||||||||||||||||||||||||||||||||
Sbjct  481  TAATTACAGCTATTTTATTACTTTTATCTTTACCTGTATTAGCAGGTGCCATTACAATAT  540

Query  579  TATTAACTGATCGAAACTTTAACACATCATTTTTTGATCCTTCAGGAGGAGGAGATCCCA  638
            | |||||||||||||||||||| ||||||||||||||||||||||| ||||||||||| |
Sbjct  541  TGTTAACTGATCGAAACTTTAATACATCATTTTTTGATCCTTCAGGGGGAGGAGATCCAA  600

Query  639  TTTTATATCAACACTTATTT  658
            ||||||||||||| ||||||
Sbjct  601  TTTTATATCAACATTTATTT  620
```

>Rhipicephalus sanguineus clone #4 cytochrome oxidase subunit I (COI) gene, partial cds; mitochondrial
Sequence ID: KX519709.1 Length: 635
Range 1: 2 to 621

Score:974 bits(527), Expect:0.0,
Identities:589/620(95%), Gaps:0/620(0%), Strand: Plus/Plus

```
Query  39   TAGGACTTAGTATAAGAATATTAATTCGTATAGAATTAGGACAACCTGGAACTTTAATTG  98
            ||||||||||||||||||||||||||||||||||||||||||||||||||||||||||||
Sbjct  2    TAGGACTTAGTATAAGAATATTAATTCGTATAGAATTAGGACAACCTGGAACTTTAATTG  61

Query  99   GAAATGATCAGATTTATAATGTAATCGTAACAGCACATGCATTTATTATAATTTTCTTTA  158
            |||||||||||||||||||||||||||||||||||||||| |||||||||||||| ||||
Sbjct  62   GAAATGATCAGATTTATAATGTAATCGTAACAGCACATGCTTTTATTATAATTTTTTTTA  121

Query  159  TAGTTATACCAATCATAATCGGAGGGTTCGGAAATTGATTAGTTCCTATTATATTAGGTG  218
            ||||||||||||||||||||||||| || |||||||||||||| ||||| ||||| ||||
Sbjct  122  TAGTTATACCAATCATAATCGGAGGATTTGGAAATTGATTAGTCCCTATCATATTGGGTG  181

Query  219  CCCCAGATATAGCATTTCCACGAATAAATAATATAAGATTTTGACTTTTACCCCCCTCAT  278
            |||| ||||||||||||||||||||||||||||||||||||||||||||||| || |||
Sbjct  182  CCCCGGATATAGCATTTCCACGAATAAATAATATAAGATTTTGACTTTTACCTCCTTCAC  241

Query  279  TATTTTTATTGATTAACTCTTCATTAATTGAATCCGGAGCTGGAACAGGATGAACAGTTT  338
```

```
            |||||||||| ||||||||||||||||||||||| |||||||| ||||||||||||||||
Sbjct  242  TATTTTTATTAATTAACTCTTCATTAATTGAATCTGGAGCTGGTACAGGATGAACAGTTT  301

Query  339  ATCCCCCTCTATCTTCAAATTTATCACATTATGGTCCATCAGTAGATTTAGCTATTTTTT  398
            | || ||||||||||||||||||||||||||||| || |||| |||||||||||||||||
Sbjct  302  ACCCTCCTCTATCTTCAAATTTATCACATTATGGCCCTTCAGGAGATTTAGCTATTTTTT  361

Query  399  CTCTTCACCTTGCTGGTGCTTCTTCAATTTTAGGTGCAATTAACTTCATCACAACTATTG  458
            ||||||| |||||||||||||||||||||||||||||||||||||| || ||||||||||
Sbjct  362  CTCTTCATCTTGCTGGTGCTTCTTCAATTTTAGGTGCAATTAACTTTATTACAACTATTG  421

Query  459  TAAATATACGATCAATTGGAATAACAATAGAACGAATACCATTATTTGTTTGATCTGTTT  518
            |||| ||||||||||||||||||||||||||||| |||||||||||||||||||||||||
Sbjct  422  TAAACATACGATCAATTGGAATAACAATAGAACGTATACCATTATTTGTTTGATCTGTTT  481

Query  519  TAATTACAGCTATTTTACTACTTCTATCTTTACCTGTATTAGCAGGTGCCATTACAATAT  578
            ||||||||||||||||| ||||| ||||||||||||||||||||||||||||||||||||
Sbjct  482  TAATTACAGCTATTTTATTACTTTTATCTTTACCTGTATTAGCAGGTGCCATTACAATAT  541

Query  579  TATTAACTGATCGAAACTTTAACACATCATTTTTGATCCTTCAGGAGGAGGAGATCCCA  638
            | ||||||||||||||||||||| |||||||||||||||||||||| ||||||||||| |
Sbjct  542  TGTTAACTGATCGAAACTTTAATACATCATTTTTGATCCTTCAGGGGGAGGAGATCCAA  601

Query  639  TTTTATATCAACACTTATTT  658
            ||||||||||||| ||||||
Sbjct  602  TTTTATATCAACATTTATTT  621
```

>Rhipicephalus sanguineus isolate LIC4723B cytochrome oxidase subunit 1 (COI) gene, partial cds; mitochondrial
Sequence ID: KX714599.1 Length: 723
>Rhipicephalus sanguineus isolate LIC4750B cytochrome oxidase subunit 1 (COI) gene, partial cds; mitochondrial
Sequence ID: KX714600.1 Length: 723
Range 1: 1 to 597

Score:937 bits(507), Expect:0.0,
Identities:567/597(95%), Gaps:0/597(0%), Strand: Plus/Plus

```
Query  62   ATTCGTATAGAATTAGGACAACCTGGAACTTTAATTGGAAATGATCAGATTTATAATGTA  121
            ||||||||||||||||||||||||||||||||||||||||||||||||||||||||||||
Sbjct  1    ATTCGTATAGAATTAGGACAACCTGGAACTTTAATTGGAAATGATCAGATTTATAATGTA  60

Query  122  ATCGTAACAGCACATGCATTTATTATAATTTTCTTTATAGTTATACCAATCATAATCGGA  181
            |||||||||||||||||||||||||||||||| ||||||||||||||||||||||| |||
Sbjct  61   ATCGTAACAGCACATGCATTTATTATAATTTTTTTTATAGTTATACCAATCATAATTGGA  120

Query  182  GGGTTCGGAAATTGATTAGTTCCTATTATATTAGGTGCCCCAGATATAGCATTTCCACGA  241
            || || |||||||||||||| ||||| ||||| |||||||||||||||||||||||||||
Sbjct  121  GGATTTGGAAATTGATTAGTCCCTATCATATTGGGTGCCCCAGATATAGCATTTCCACGA  180

Query  242  ATAAATAATATAAGATTTTGACTTTTACCCCCCTCATTATTTTTATTGATTAACTCTTCA  301
```

```
                |||||||||||||||||||||||||||||| || ||| |||||||||| ||||||||||||
Sbjct  181   ATAAATAATATAAGATTTTGACTTTTACCTCCTTCACTATTTTTATTAATTAACTCTTCA  240

Query  302   TTAATTGAATCCGGAGCTGGAACAGGATGAACAGTTTATCCCCCTCTATCTTCAAATTTA  361
             ||||||||||| |||||||| ||||||||||||||||| || ||||||||||||||||||
Sbjct  241   TTAATTGAATCTGGAGCTGGTACAGGATGAACAGTTTACCCTCCTCTATCTTCAAATTTA  300

Query  362   TCACATTATGGTCCATCAGTAGATTTAGCTATTTTTTCTCTTCACCTTGCTGGTGCTTCT  421
             ||||||||||| || ||||||||||||||||| ||||||||||| |||||||||||||||
Sbjct  301   TCACATTATGGCCCTTCAGTAGATTTAGCTATCTTTTCTCTTCATCTTGCTGGTGCTTCT  360

Query  422   TCAATTTTAGGTGCAATTAACTTCATCACAACTATTGTAAATATACGATCAATTGGAATA  481
             ||||||||||||||||||||||| || |||||||||||||| ||||||||||||||||||
Sbjct  361   TCAATTTTAGGTGCAATTAACTTTATTACAACTATTGTAAACATACGATCAATTGGAATA  420

Query  482   ACAATAGAACGAATACCATTATTTGTTTGATCTGTTTTAATTACAGCTATTTTACTACTT  541
             ||||||||||| |||||||||||||||||||||||||||||||||||||||||| |||||
Sbjct  421   ACAATAGAACGTATACCATTATTTGTTTGATCTGTTTTAATTACAGCTATTTTATTACTT  480

Query  542   CTATCTTTACCTGTATTAGCAGGTGCCATTACAATATTATTAACTGATCGAAACTTTAAC  601
              ||||||||||||||||||||||||||||||||||||| ||||||||||||||||||||
Sbjct  481   TTATCTTTACCTGTATTAGCAGGTGCCATTACAATATTGTTAACTGATCGAAACTTTAAT  540

Query  602   ACATCATTTTTTGATCCTTCAGGAGGAGGAGATCCCATTTTATATCAACACTTATTT  658
             ||||||||||||||||||||||| ||||||||||| |||||||||||||| ||||||
Sbjct  541   ACATCATTTTTTGATCCTTCAGGGGGAGGAGATCCAATTTTATATCAACATTTATTT  597
```

>Rhipicephalus sanguineus clone #8 cytochrome oxidase subunit I (COI) gene,
partial cds; mitochondrial
Sequence ID: KX519713.1 Length: 580
Range 1: 1 to 580

Score:933 bits(505), Expect:0.0,
Identities:555/580(96%), Gaps:0/580(0%), Strand: Plus/Plus

```
Query  43    ACTTAGTATAAGAATATTAATTCGTATAGAATTAGGACAACCTGGAACTTTAATTGGAAA  102
             ||||||||||||||||||||||||||||||||||||||||||||||||||||||||||||
Sbjct  1     ACTTAGTATAAGAATATTAATTCGTATAGAATTAGGACAACCTGGAACTTTAATTGGAAA  60

Query  103   TGATCAGATTTATAATGTAATCGTAACAGCACATGCATTTATTATAATTTCTTTATAGT  162
             ||||||||||||||||||||||||||||||||||||||||||||||||||| ||||||||
Sbjct  61    TGATCAGATTTATAATGTAATCGTAACAGCACATGCATTTATTATAATTTTTTTATAGT  120

Query  163   TATACCAATCATAATCGGAGGGTTCGGAAATTGATTAGTTCCTATTATATTAGGTGCCCC  222
             ||||||||||||||||||||| || |||||||||||||| ||||| ||||| ||||||||
Sbjct  121   TATACCAATCATAATCGGAGGATTTGGAAATTGATTAGTCCCTATCATATTGGGTGCCCC  180

Query  223   AGATATAGCATTTCCACGAATAAATAATATAAGATTTTGACTTTTACCCCCCTCATTATT  282
             |||||||||||||||||||||||||||||||||||||||||||||||| || ||| ||||
Sbjct  181   AGATATAGCATTTCCACGAATAAATAATATAAGATTTTGACTTTTACCTCCTTCACTATT  240
```

```
Query  283   TTTATTGATTAACTCTTCATTAATTGAATCCGGAGCTGGAACAGGATGAACAGTTTATCC  342
             |||||| ||||||||||||||||||||||| |||||||| ||||||||||||||||| ||
Sbjct  241   TTTATTAATTAACTCTTCATTAATTGAATCTGGAGCTGGTACAGGATGAACAGTTTACCC  300

Query  343   CCCTCTATCTTCAAATTTATCACATTATGGTCCATCAGTAGATTTAGCTATTTTTTCTCT  402
              ||||||||||||||||||||||||||||| || ||||||||||||||||||||||||||
Sbjct  301   TCCTCTATCTTCAAATTTATCACATTATGGCCCTTCAGTAGATTTAGCTATTTTTTCTCT  360

Query  403   TCACCTTGCTGGTGCTTCTTCAATTTTAGGTGCAATTAACTTCATCACAACTATTGTAAA  462
             ||| |||||||||||||||||||||||||||||||||||||| || ||||||||||||||
Sbjct  361   TCATCTTGCTGGTGCTTCTTCAATTTTAGGTGCAATTAACTTTATTACAACTATTGTAAA  420

Query  463   TATACGATCAATTGGAATAACAATAGAACGAATACCATTATTTGTTTGATCTGTTTTAAT  522
              ||||||||||||||||||||||||||||| |||||||||||||||||||||||||||||
Sbjct  421   CATACGATCAATTGGAATAACAATAGAACGTATACCATTATTTGTTTGATCTGTTTTAAT  480

Query  523   TACAGCTATTTTACTACTTCTATCTTTACCTGTATTAGCAGGTGCCATTACAATATTATT  582
             ||||||||||||| ||||| ||||||||||||||||||||||||||||||||||||| ||
Sbjct  481   TACAGCTATTTTATTACTTTTATCTTTACCTGTATTAGCAGGTGCCATTACAATATTGTT  540

Query  583   AACTGATCGAAACTTTAACACATCATTTTTTGATCCTTCA  622
             |||||||||||||||||| |||||||||||||||||||||
Sbjct  541   AACTGATCGAAACTTTAATACATCATTTTTTGATCCTTCA  580
```

>Rhipicephalus sanguineus clone #1 cytochrome oxidase subunit I (COI) gene, partial cds; mitochondrial
Sequence ID: KX519706.1 Length: 595
Range 1: 1 to 570

Score:898 bits(486), Expect:0.0,
Identities:542/570(95%), Gaps:0/570(0%), Strand: Plus/Plus

```
Query  89    ACTTTAATTGGAAATGATCAGATTTATAATGTAATCGTAACAGCACATGCATTTATTATA  148
             ||||||||||||||||||||||||||||||||||||||||||||||||||||||||||||
Sbjct  1     ACTTTAATTGGAAATGATCAGATTTATAATGTAATCGTAACAGCACATGCATTTATTATA  60

Query  149   ATTTTCTTTATAGTTATACCAATCATAATCGGAGGGTTCGGAAATTGATTAGTTCCTATT  208
             ||||| ||||||||||||||||||||||||||||| || |||||||||||||| |||||
Sbjct  61    ATTTTTTTTATAGTTATACCAATCATAATCGGAGGATTTGGAAATTGATTAGTCCCTATC  120

Query  209   ATATTAGGTGCCCCAGATATAGCATTTCCACGAATAAATAATATAAGATTTTGACTTTTA  268
             ||||| ||||||||||||||||||||||||||||||||||||||||||||||||||||||
Sbjct  121   ATATTGGGTGCCCCAGATATAGCATTTCCACGAATAAATAATATAAGATTTTGACTTTTA  180

Query  269   CCCCCCTCATTATTTTTATTGATTAACTCTTCATTAATTGAATCCGGAGCTGGAACAGGA  328
             || || ||| |||||||||| ||||||||||||||||||||||| |||||||| ||||||
Sbjct  181   CCTCCTTCACTATTTTTATTAATTAACTCTTCATTAATTGAATCTGGAGCTGGTACAGGA  240

Query  329   TGAACAGTTTATCCCCCTCTATCTTCAAATTTATCACATTATGGTCCATCAGTAGATTTA  388
             ||||||||||| || ||||||||||||||||||||||||||||| || ||||||||||||
Sbjct  241   TGAACAGTTTACCCTCCTCTATCTTCAAATTTATCACATTATGGCCCTTCAGTAGATTTA  300
```

```
Query  389   GCTATTTTTTCTCTTCACCTTGCTGGTGCTTCTTCAATTTTAGGTGCAATTAACTTCATC  448
             ||||||||||||||||| |||||||||||||||||||||||||||||||||||||| ||
Sbjct  301   GCTATTTTTTCTCTTCATCTTGCTGGTGCTTCTTCAATTTTAGGTGCAATTAACTTTATT  360

Query  449   ACAACTATTGTAAATATACGATCAATTGGAATAACAATAGAACGAATACCATTATTTGTT  508
             |||||||||||||| ||||||||||||||||||||||||||||| |||||||||||||||
Sbjct  361   ACAACTATTGTAAACATACGATCAATTGGAATAACAATAGAACGTATACCATTATTTGTT  420

Query  509   TGATCTGTTTAATTACAGCTATTTTACTACTTCTATCTTTACCTGTATTAGCAGGTGCC  568
             |||||||||||||||||||||||||| ||||| |||||||||||||||||||||||||||
Sbjct  421   TGATCTGTTTAATTACAGCTATTTTATTACTTTTATCTTTACCTGTATTAGCAGGTGCC  480

Query  569   ATTACAATATTATTAACTGATCGAAACTTTAACACATCATTTTTTGATCCTTCAGGAGGA  628
             ||||||||||| |||||||||||||||||||| ||||||||||||||||||||||| |||
Sbjct  481   ATTACAATATTGTTAACTGATCGAAACTTTAATACATCATTTTTTGATCCTTCAGGGGGA  540

Query  629   GGAGATCCCATTTTATATCAACACTTATTT  658
             |||||||| |||||||||||||| ||||||
Sbjct  541   GGAGATCCAATTTTATATCAACATTTATTT  570
```

>Rhipicephalus sanguineus isolate Rsang2 cytochrome oxidase subunit 1 gene, partial cds; mitochondrial
Sequence ID: KY678135.1 Length: 790
Range 1: 18 to 675

Score:883 bits(478), Expect:0.0,
Identities:599/659(91%), Gaps:2/659(0%), Strand: Plus/Plus

```
Query  1     AACAATATATTTAATTTTTGGAGCATGATCAGGTATATTAGGACTTAGTATAAGAATATT  60
             |||||||||||||||||||||||||||||| || ||||||||| | || ||||||||| |
Sbjct  18    AACAATATATTTAATTTTTGGAGCATGATCCGGAATATTAGGATTAAGAATAAGAATACT  77

Query  61    AATTCGTATAGAATTAGGACAACCTGGAACTTTAATTGGAAATGATCAGATTTATAATGT  120
             |||||||||||||||||| |||||||||||  ||||||| |||||||| |||||||||||
Sbjct  78    AATTCGTATAGAATTAGGCCAACCTGGAACCCTAATTGGTAATGATCAAATTTATAATGT  137

Query  121   AATCGTAACAGCACATGCATTTATTATAATTTTCTTTATAGTTATACCAATCATAATCGG  180
             ||| || ||||| |||||||||||||||||||| |||||||| |||||||||||||| ||
Sbjct  138   AATTGTTACAGCTCATGCATTTATTATAATTTTTTTTATAGTAATACCAATCATAATTGG  197

Query  181   AGGGTTCGGAAATTGATTAGTTCCTATTATATTAGGTGCCCCAGATATAGCATTTCCACG  240
              || || ||||| |||||||||||||||||| |||| || |||||||||||||| |||||
Sbjct  198   TGGATTTGGAAACTGATTAGTTCCTATTATACTAGGAGCTCCAGATATAGCATTCCCACG  257

Query  241   AATAAATAATATAAGATTTTGACTTTTACCCCCCTCATTATTTTTATTGATTAACTCTTC  300
             ||||||||||||||||||||||||| | || |||||||||||| |||| ||||| |||||
Sbjct  258   AATAAATAATATAAGATTTTGACTTCTTCCTCCCTCATTATTTATATTAATTAATTCTTC  317

Query  301   ATTAATTGAATCCGGAGCTGGAACAGGATGAACAGTTTATCC-CCCTCTATCTTCAAATT  359
             ||||||||| || ||||| || |||||||||||||||||||| |||| |||| |||||||
```

```
Sbjct  318  ATTAATTGAGTCAGGAGCAGGTACAGGATGAACAGTTTATCCTCCCT-TATCCTCAAATT  376

Query  360  TATCACATTATGGTCCATCAGTAGATTTAGCTATTTTTCTCTTCACCTTGCTGGTGCTT  419
            ||||||||||||| |||||||||||||||||||||||||||||||| |||||||||||||
Sbjct  377  TATCACATTATGGGCCATCAGTAGATTTAGCTATTTTTCTCTTCATCTTGCTGGTGCTT  436

Query  420  CTTCAATTTTAGGTGCAATTAACTTCATCACAACTATTGTAAATATACGATCAATTGGAA  479
            |||||||||||||||||||||| || || ||||||||||||||||||||||| |||||||
Sbjct  437  CTTCAATTTTAGGTGCAATTAATTTTATTACAACTATTGTAAATATACGATCTATTGGAA  496

Query  480  TAACAATAGAACGAATACCATTATTTGTTTGATCTGTTTTAATTACAGCTATTTTACTAC  539
            |||||||||||||||||||||||||||| ||||||||||||||||| || |||||| |||
Sbjct  497  TAACAATAGAACGAATACCATTATTTGTATGATCTGTTTTAATTACTGCAATTTTATTAC  556

Query  540  TTCTATCTTTACCTGTATTAGCAGGTGCCATTACAATATTATTAACTGATCGAAACTTTA  599
            |  ||||||||||||| ||||||||||||||||||||| ||||||| |||||||| ||||
Sbjct  557  TATTATCTTTACCTGTTTTAGCAGGTGCCATTACAATACTATTAACCGATCGAAATTTTA  616

Query  600  ACACATCATTTTTTGATCCTTCAGGAGGAGGAGATCCCATTTTATATCAACACTTATTT  658
            |||| ||||||||||| |||||||| ||||| ||||| |||||||||||||| ||||||
Sbjct  617  ACACTTCATTTTTTGACCCTTCAGGGGGAGGGGATCCAATTTTATATCAACATTTATTT  675
```

>Rhipicephalus turanicus isolate COX1-225-Goat cytochrome c oxidase subunit I (COX1) gene, partial cds; mitochondrial
Sequence ID: MT800313.1 Length: 673
Range 1: 8 to 665

Score:883 bits(478), Expect:0.0,
Identities:598/658(91%), Gaps:0/658(0%), Strand: Plus/Plus

```
Query  1    AACAATATATTTAATTTTTGGAGCATGATCAGGTATATTAGGACTTAGTATAAGAATATT  60
            ||||||||||||||||||||| ||||||||||| ||||||||||| || ||||||||| |
Sbjct  8    AACAATATATTTAATTTTTGGGGCATGATCAGGAATATTAGGACTGAGAATAAGAATACT  67

Query  61   AATTCGTATAGAATTAGGACAACCTGGAACTTTAATTGGAAATGATCAGATTTATAATGT  120
            |||||| ||||||||||||||||||||||||||||||||||||||||| |||||||| ||
Sbjct  68   AATTCGCATAGAATTAGGACAACCTGGAACTTTAATTGGAAATGATCAAATTTATAACGT  127

Query  121  AATCGTAACAGCACATGCATTTATTATAATTTTCTTTATAGTTATACCAATCATAATCGG  180
            ||| |||||||| |||||||||||||||||||| ||||| || ||||| || ||||| ||
Sbjct  128  AATTGTAACAGCTCATGCATTTATTATAATTTTTTTTATGGTAATACCTATTATAATTGG  187

Query  181  AGGGTTCGGAAATTGATTAGTTCCTATTATATTAGGTGCCCCAGATATAGCATTTCCACG  240
             || || ||||||||||||||||||||||||||||| || |||||||||||||| |||||
Sbjct  188  TGGATTTGGAAATTGATTAGTTCCTATTATATTAGGGGCTCCAGATATAGCATTCCCACG  247

Query  241  AATAAATAATATAAGATTTTGACTTTTACCCCCCTCATTATTTTTATTGATTAACTCTTC  300
             |||||||| |||||||||||| |  | || || ||||||||| |||| |||||||||||
Sbjct  248  TATAAATAACATAAGATTTTGATTACTTCCTCCTTCATTATTTCTATTAATTAACTCTTC  307

Query  301  ATTAATTGAATCCGGAGCTGGAACAGGATGAACAGTTTATCCCCCTCTATCTTCAAATTT  360
```

```
            |||||||||||| ||||| |||||||||||||| |||||||||||| |||| ||||||||
Sbjct  308  ATTAATTGAATCAGGAGCAGGAACAGGATGAACTGTTTATCCCCCTTTATCCTCAAATTT  367

Query  361  ATCACATTATGGTCCATCAGTAGATTTAGCTATTTTTTCTCTTCACCTTGCTGGTGCTTC  420
            |||||||||||| |||||||||||||||||||||||||||||||| ||||||||||||||
Sbjct  368  ATCACATTATGGACCATCAGTAGATTTAGCTATTTTTTCTCTTCATCTTGCTGGTGCTTC  427

Query  421  TTCAATTTTAGGTGCAATTAACTTCATCACAACTATTGTAAATATACGATCAATTGGAAT  480
            ||||||| ||||||||||||| || || ||||||||||||||||||||||| ||||||||
Sbjct  428  TTCAATTCTAGGTGCAATTAATTTTATTACAACTATTGTAAATATACGATCTATTGGAAT  487

Query  481  AACAATAGAACGAATACCATTATTTGTTTGATCTGTTTTAATTACAGCTATTTTACTACT  540
            ||||||||||||||||||||||||||||||||||||||||||||| || || ||||| ||
Sbjct  488  AACAATAGAACGAATACCATTATTTGTTTGATCTGTTTTAATTACCGCCATCTTACTTCT  547

Query  541  TCTATCTTTACCTGTATTAGCAGGTGCCATTACAATATTATTAACTGATCGAAACTTTAA  600
              ||||||||||||| ||||||||||| |||||||||||||||||||||||||| || ||
Sbjct  548  CTTATCTTTACCTGTTTTAGCAGGTGCTATTACAATATTATTAACTGATCGAAATTTCAA  607

Query  601  CACATCATTTTTTGATCCTTCAGGAGGAGGAGATCCCATTTTATATCAACACTTATTT  658
             || |||||||||||||||||||| ||||| ||||| |||||||| ||||||||||||
Sbjct  608  TACTTCATTTTTTGATCCTTCAGGGGGAGGTGATCCAATTTTATACCAACACTTATTT  665
```

>Rhipicephalus sanguineus sensu lato 'temperate lineage' isolate SC3005 cytochrome c oxidase subunit I (COI) gene, partial cds; mitochondrial
Sequence ID: MF425987.1 Length: 574
Range 1: 1 to 561

Score:881 bits(477), Expect:0.0,
Identities:533/561(95%), Gaps:0/561(0%), Strand: Plus/Plus

```
Query  98   GGAAATGATCAGATTTATAATGTAATCGTAACAGCACATGCATTTATTATAATTTTCTTT  157
            |||||||||||||||||||||||||||||||||||||| ||||||||||||||||| |||
Sbjct  1    GGAAATGATCAGATTTATAATGTAATCGTAACAGCACACGCATTTATTATAATTTTTTTT  60

Query  158  ATAGTTATACCAATCATAATCGGAGGGTTCGGAAATTGATTAGTTCCTATTATATTAGGT  217
            |||||||||||||||||||||||||| || |||||||||||||| |||||||||||||||
Sbjct  61   ATAGTTATACCAATCATAATCGGAGGATTTGGAAATTGATTAGTCCCTATTATATTAGGT  120

Query  218  GCCCCAGATATAGCATTTCCACGAATAAATAATATAAGATTTTGACTTTTACCCCCCTCA  277
            ||||||||||||||||||||||||||||||||||||||||||||||||||||| || |||
Sbjct  121  GCCCCAGATATAGCATTTCCACGAATAAATAATATAAGATTTTGACTTTTACCTCCTTCA  180

Query  278  TTATTTTTATTGATTAACTCTTCATTAATTGAATCCGGAGCTGGAACAGGATGAACAGTT  337
             |||||||||| ||||||||||||||||||||||| |||||||| |||||||||||||||
Sbjct  181  CTATTTTTATTAATTAACTCTTCATTAATTGAATCTGGAGCTGGTACAGGATGAACAGTT  240

Query  338  TATCCCCCTCTATCTTCAAATTTATCACATTATGGTCCATCAGTAGATTTAGCTATTTTT  397
            || || ||||||||||||||||||||||||||||| || ||||||||||||||||| |||
Sbjct  241  TACCCTCCTCTATCTTCAAATTTATCACATTATGGCCCTTCAGTAGATTTAGCTATCTTT  300
```

```
Query  398   TCTCTTCACCTTGCTGGTGCTTCTTCAATTTTAGGTGCAATTAACTTCATCACAACTATT  457
             |||||||| |||||||||||||||||||||||||||||||||||||| || |||||||||
Sbjct  301   TCTCTTCATCTTGCTGGTGCTTCTTCAATTTTAGGTGCAATTAACTTTATTACAACTATT  360

Query  458   GTAAATATACGATCAATTGGAATAACAATAGAACGAATACCATTATTTGTTTGATCTGTT  517
             ||||| ||||||||||||||||||||||||||||| ||||||||||||||||||||||||
Sbjct  361   GTAAACATACGATCAATTGGAATAACAATAGAACGTATACCATTATTTGTTTGATCTGTT  420

Query  518   TTAATTACAGCTATTTTACTACTTCTATCTTTACCTGTATTAGCAGGTGCCATTACAATA  577
             |||||||||||||||||| ||||| |||||||||||||||||||||||||||||||||||
Sbjct  421   TTAATTACAGCTATTTTATTACTTTTATCTTTACCTGTATTAGCAGGTGCCATTACAATA  480

Query  578   TTATTAACTGATCGAAACTTTAACACATCATTTTTTGATCCTTCAGGAGGAGGAGATCCC  637
             || |||||||||||||||||||| ||||||||||||||||||||||| |||||||||||
Sbjct  481   TTGTTAACTGATCGAAACTTTAATACATCATTTTTTGATCCTTCAGGGGGAGGAGATCCA  540

Query  638   ATTTTATATCAACACTTATTT  658
             |||||||||||||| ||||||
Sbjct  541   ATTTTATATCAACATTTATTT  561
```

>Rhipicephalus sanguineus cytochrome oxidase subunit 1 (CO1) gene, partial cds; mitochondrial
Sequence ID: KU214592.1 Length: 829
Range 1: 1 to 648

Score:876 bits(474), Expect:0.0,
Identities:590/648(91%), Gaps:0/648(0%), Strand: Plus/Plus

```
Query  11    TTAATTTTTGGAGCATGATCAGGTATATTAGGACTTAGTATAAGAATATTAATTCGTATA  70
             |||||||||||||||||||| || ||||||||| | || ||||||||| |||||||||||
Sbjct  1     TTAATTTTTGGAGCATGATCCGGAATATTAGGATTAAGAATAAGAATACTAATTCGTATA  60

Query  71    GAATTAGGACAACCTGGAACTTTAATTGGAAATGATCAGATTTATAATGTAATCGTAACA  130
             |||||||| |||||||||||| ||||||| |||||||| |||||||||||||| || |||
Sbjct  61    GAATTAGGCCAACCTGGAACTCTAATTGGTAATGATCAAATTTATAATGTAATTGTTACA  120

Query  131   GCACATGCATTTATTATAATTTTCTTTATAGTTATACCAATCATAATCGGAGGGTTCGGA  190
             || |||||||||||||||||||| |||||||| |||||||||||||| || || || |||
Sbjct  121   GCTCATGCATTTATTATAATTTTTTTTATAGTAATACCAATCATAATTGGTGGATTTGGA  180

Query  191   AATTGATTAGTTCCTATTATATTAGGTGCCCCAGATATAGCATTTCCACGAATAAATAAT  250
             || |||||||||||||||||| |||| || ||||||||||||||||||||||||||||||
Sbjct  181   AACTGATTAGTTCCTATTATACTAGGAGCTCCAGATATAGCATTTCCACGAATAAATAAT  240

Query  251   ATAAGATTTTGACTTTTACCCCCCTCATTATTTTTATTGATTAACTCTTCATTAATTGAA  310
             ||||||||||||||| | || |||||||||||| |||| ||||| ||||||||||||||
Sbjct  241   ATAAGATTTTGACTTCTTCCTCCCTCATTATTTATATTAATTAATTCTTCATTAATTGAG  300

Query  311   TCCGGAGCTGGAACAGGATGAACAGTTTATCCCCCTCTATCTTCAAATTTATCACATTAT  370
             || ||||| || |||||||| ||||||||||| || ||||| ||||||||||||||||||
Sbjct  301   TCAGGAGCAGGTACAGGATGGACAGTTTATCCTCCCCTATCCTCAAATTTATCACATTAT  360
```

```
Query  371  GGTCCATCAGTAGATTTAGCTATTTTTCTCTTCACCTTGCTGGTGCTTCTTCAATTTTA  430
            || ||||||||||||||||||||||||||||||| |||||||||||||||||||||||||
Sbjct  361  GGGCCATCAGTAGATTTAGCTATTTTTCTCTTCATCTTGCTGGTGCTTCTTCAATTTTA  420

Query  431  GGTGCAATTAACTTCATCACAACTATTGTAAATATACGATCAATTGGAATAACAATAGAA  490
            ||||||||||| || || ||||||||||||||||||||||| ||||||||||||||||||
Sbjct  421  GGTGCAATTAATTTTATTACAACTATTGTAAATATACGATCTATTGGAATAACAATAGAA  480

Query  491  CGAATACCATTATTTGTTTGATCTGTTTTAATTACAGCTATTTTACTACTTCTATCTTTA  550
            ||||||||||||||||| ||||||||||||||||| || |||||| ||||  ||||||||
Sbjct  481  CGAATACCATTATTTGTATGATCTGTTTTAATTACTGCAATTTTATTACTATTATCTTTA  540

Query  551  CCTGTATTAGCAGGTGCCATTACAATATTATTAACTGATCGAAACTTTAACACATCATTT  610
            ||||| ||||||||||||||||||||||||||||| |||||||| |||||||| ||||||
Sbjct  541  CCTGTTTTAGCAGGTGCCATTACAATATTATTAACCGATCGAAATTTTAACACTTCATTT  600

Query  611  TTTGATCCTTCAGGAGGAGGAGATCCCATTTTATATCAACACTTATTT  658
            ||||| |||||||| ||||| ||||| |||||||||||||| ||||||
Sbjct  601  TTTGACCCTTCAGGGGGAGGGGATCCAATTTTATATCAACATTTATTT  648
```

>Rhipicephalus turanicus isolate GY34-1 cytochrome c oxidase subunit I (COX1) gene, partial cds; mitochondrial
Sequence ID: MT079206.1 Length: 678
Range 1: 1 to 652

Score:872 bits(472), Expect:0.0,
Identities:592/652(91%), Gaps:0/652(0%), Strand: Plus/Plus

```
Query  7    ATATTTAATTTTTGGAGCATGATCAGGTATATTAGGACTTAGTATAAGAATATTAATTCG  66
            ||||||||||||||||||||||||||||||||||||| | || |||||||||||||||||
Sbjct  1    ATATTTAATTTTTGGAGCATGATCAGGTATATTAGGATTAAGAATAAGAATATTAATTCG  60

Query  67   TATAGAATTAGGACAACCTGGAACTTTAATTGGAAATGATCAGATTTATAATGTAATCGT  126
             ||||||||||||||||||||||||||||||||||||||||| |||||||||||||| ||
Sbjct  61   AATAGAATTAGGACAACCTGGAACTTTAATTGGAAATGATCAAATTTATAATGTAATTGT  120

Query  127  AACAGCACATGCATTTATTATAATTTTCTTTATAGTTATACCAATCATAATCGGAGGGTT  186
            |||||| |||||||||||||||||||| |||||||| |||||||||||||| ||||| ||
Sbjct  121  AACAGCTCATGCATTTATTATAATTTTTTTTATAGTAATACCAATCATAATTGGAGGATT  180

Query  187  CGGAAATTGATTAGTTCCTATTATATTAGGTGCCCCAGATATAGCATTTCCACGAATAAA  246
             ||||| || |||||||| ||||||||||| || ||||| ||||||||||||||||||||
Sbjct  181  TGGAAACTGGTTAGTTCCAATTATATTAGGAGCTCCAGACATAGCATTTCCACGAATAAA  240

Query  247  TAATATAAGATTTTGACTTTTACCCCCCTCATTATTTTTATTGATTAACTCTTCATTAAT  306
            |||||||||||||||| |  | |||||||||||||| ||||| ||||| |||||| | ||
Sbjct  241  TAATATAAGATTTTGATTACTTCCCCCCTCATTATTCTTATTAATTAATTCTTCACTGAT  300

Query  307  TGAATCCGGAGCTGGAACAGGATGAACAGTTTATCCCCCTCTATCTTCAAATTTATCACA  366
            |||||| || || || ||||| ||||| |||||||| ||| |||| ||||||||||| ||
```

```
Sbjct  301  TGAATCTGGGGCAGGGACAGGGTGAACTGTTTATCCTCCTTTATCCTCAAATTTATCCCA  360

Query  367  TTATGGTCCATCAGTAGATTTAGCTATTTTTTCTCTTCACCTTGCTGGTGCTTCTTCAAT  426
            |||||| |||||||||||||||||||| || |||||||| ||||||||||||||||||||
Sbjct  361  TTATGGACCATCAGTAGATTTAGCTATCTTCTCTCTTCATCTTGCTGGTGCTTCTTCAAT  420

Query  427  TTTAGGTGCAATTAACTTCATCACAACTATTGTAAATATACGATCAATTGGAATAACAAT  486
            |||||| |||||||| ||||| |||||||||||||| |||||||| ||||||||||||||
Sbjct  421  TTTAGGCGCAATTAATTTCATTACAACTATTGTAAACATACGATCTATTGGAATAACAAT  480

Query  487  AGAACGAATACCATTATTTGTTTGATCTGTTTTAATTACAGCTATTTTACTACTTCTATC  546
            |||||||||||||||||| ||||||||||||||||| || ||||||||| | ||||||||
Sbjct  481  AGAACGAATACCATTATTCGTTTGATCTGTTTTAATCACCGCTATTTTATTGCTTCTATC  540

Query  547  TTTACCTGTATTAGCAGGTGCCATTACAATATTATTAACTGATCGAAACTTTAACACATC  606
            ||||||||| ||||||||||||||||||||| |||||||||||||||| |||||||| ||
Sbjct  541  TTTACCTGTTTTAGCAGGTGCCATTACAATACTATTAACTGATCGAAATTTTAACACTTC  600

Query  607  ATTTTTTGATCCTTCAGGAGGAGGAGATCCCATTTTATATCAACACTTATTT  658
            ||||||||| |||||||||||||| || || |||||||||||||| ||||||
Sbjct  601  ATTTTTTGACCCTTCAGGAGGAGGTGACCCAATTTTATATCAACATTTATTT  652
```

>Rhipicephalus sanguineus isolate GY34 cytochrome c oxidase subunit I (COX1) gene, partial cds; mitochondrial
Sequence ID: MN862754.1 Length: 678
Range 1: 1 to 652

Score:872 bits(472), Expect:0.0,
Identities:592/652(91%), Gaps:0/652(0%), Strand: Plus/Plus

```
Query  7    ATATTTAATTTTTGGAGCATGATCAGGTATATTAGGACTTAGTATAAGAATATTAATTCG  66
            ||||||||||||||||||||||||||||||||||||| | || |||||||||||||||||
Sbjct  1    ATATTTAATTTTTGGAGCATGATCAGGTATATTAGGATTAAGAATAAGAATATTAATTCG  60

Query  67   TATAGAATTAGGACAACCTGGAACTTTAATTGGAAATGATCAGATTTATAATGTAATCGT  126
             ||||||||||||||||||||||||||||||||||||||||| |||||||||||||| ||
Sbjct  61   AATAGAATTAGGACAACCTGGAACTTTAATTGGAAATGATCAAATTTATAATGTAATTGT  120

Query  127  AACAGCACATGCATTTATTATAATTTTCTTTATAGTTATACCAATCATAATCGGAGGGTT  186
            |||||| |||||||||||||||||||| |||||||| |||||||||||||| ||||| ||
Sbjct  121  AACAGCTCATGCATTTATTATAATTTTTTTTATAGTAATACCAATCATAATTGGAGGATT  180

Query  187  CGGAAATTGATTAGTTCCTATTATATTAGGTGCCCCAGATATAGCATTTCCACGAATAAA  246
             ||||| || |||||||| ||||||||||| || ||||| ||||||||||||||||||||
Sbjct  181  TGGAAACTGGTTAGTTCCAATTATATTAGGAGCTCCAGACATAGCATTTCCACGAATAAA  240

Query  247  TAATATAAGATTTTGACTTTTACCCCCCTCATTATTTTTATTGATTAACTCTTCATTAAT  306
            |||||||||||||||| |  | |||||||||||||| ||||| ||||| |||||| | ||
Sbjct  241  TAATATAAGATTTTGATTACTTCCCCCCTCATTATTCTTATTAATTAATTCTTCACTGAT  300

Query  307  TGAATCCGGAGCTGGAACAGGATGAACAGTTTATCCCCCTCTATCTTCAAATTTATCACA  366
```

```
                ||||||.||.||.||.|||||.|||||.||||||||.|||.||||.|||||||||||.||
Sbjct  301  TGAATCTGGGGCAGGGACAGGGTGAACTGTTTATCCTCCTTTATCCTCAAATTTATCCCA  360

Query  367  TTATGGTCCATCAGTAGATTTAGCTATTTTTTCTCTTCACCTTGCTGGTGCTTCTTCAAT  426
            |||||| |||||||||||||||||||| || |||||||| ||||||||||||||||||||
Sbjct  361  TTATGGACCATCAGTAGATTTAGCTATCTTCTCTCTTCATCTTGCTGGTGCTTCTTCAAT  420

Query  427  TTTAGGTGCAATTAACTTCATCACAACTATTGTAAATATACGATCAATTGGAATAACAAT  486
            |||||| |||||||| ||||| |||||||||||||| |||||||| ||||||||||||||
Sbjct  421  TTTAGGCGCAATTAATTTCATTACAACTATTGTAAACATACGATCTATTGGAATAACAAT  480

Query  487  AGAACGAATACCATTATTTGTTTGATCTGTTTTAATTACAGCTATTTTACTACTTCTATC  546
            |||||||||||||||||| ||||||||||||||||| || ||||||||| | ||||||||
Sbjct  481  AGAACGAATACCATTATTCGTTTGATCTGTTTTAATCACCGCTATTTTATTGCTTCTATC  540

Query  547  TTTACCTGTATTAGCAGGTGCCATTACAATATTATTAACTGATCGAAACTTTAACACATC  606
            ||||||||| ||||||||||||||||||||| |||||||||||||||| |||||||| ||
Sbjct  541  TTTACCTGTTTTAGCAGGTGCCATTACAATACTATTAACTGATCGAAATTTTAACACTTC  600

Query  607  ATTTTTTGATCCTTCAGGAGGAGGAGATCCCATTTTATATCAACACTTATTT  658
            ||||||||| |||||||||||||| || || |||||||||||||| ||||||
Sbjct  601  ATTTTTTGACCCTTCAGGAGGAGGTGACCCAATTTTATATCAACATTTATTT  652
```

>Rhipicephalus sanguineus isolate Gilan-e Gharb cytochrome oxidase subunit 1 gene, complete cds; mitochondrial
Sequence ID: KM494916.1 Length: 1539
Range 1: 45 to 702

Score:872 bits(472), Expect:0.0,
Identities:596/658(91%), Gaps:0/658(0%), Strand: Plus/Plus

```
Query  1    AACAATATATTTAATTTTTGGAGCATGATCAGGTATATTAGGACTTAGTATAAGAATATT  60
            |||||||||||||||||||||||||||||| || ||||||||| | || ||||||||| |
Sbjct  45   AACAATATATTTAATTTTTGGAGCATGATCCGGAATATTAGGATTAAGAATAAGAATACT  104

Query  61   AATTCGTATAGAATTAGGACAACCTGGAACTTTAATTGGAAATGATCAGATTTATAATGT  120
            |||||||||||||||||| |||||||||||| ||||||| |||||||| |||||||||||
Sbjct  105  AATTCGTATAGAATTAGGTCAACCTGGAACTCTAATTGGTAATGATCAAATTTATAATGT  164

Query  121  AATCGTAACAGCACATGCATTTATTATAATTTTCTTTATAGTTATACCAATCATAATCGG  180
            ||| || ||||| |||||||||||||||||||| |||||||| |||||||| ||||| ||
Sbjct  165  AATTGTTACAGCTCATGCATTTATTATAATTTTTTTTATAGTAATACCAATTATAATTGG  224

Query  181  AGGGTTCGGAAATTGATTAGTTCCTATTATATTAGGTGCCCCAGATATAGCATTTCCACG  240
             || || ||||| |||||||| ||||||||| |||| || |||||||||||||| |||||
Sbjct  225  TGGATTTGGAAACTGATTAGTACCTATTATACTAGGAGCTCCAGATATAGCATTCCCACG  284

Query  241  AATAAATAATATAAGATTTTGACTTTTACCCCCCTCATTATTTTTATTGATTAACTCTTC  300
            ||||||||||||||||||||||||| | || |||||||||||| |||| ||||| |||||
Sbjct  285  AATAAATAATATAAGATTTTGACTTCTTCCTCCCTCATTATTTATATTAATTAATTCTTC  344
```

```
Query  301  ATTAATTGAATCCGGAGCTGGAACAGGATGAACAGTTTATCCCCCTCTATCTTCAAATTT  360
            ||||||||| || ||||| || |||||||||||||||||||| || ||||| ||||||||
Sbjct  345  ATTAATTGAGTCAGGAGCAGGTACAGGATGAACAGTTTATCCTCCCCTATCCTCAAATTT  404

Query  361  ATCACATTATGGTCCATCAGTAGATTTAGCTATTTTTCTCTTCACCTTGCTGGTGCTTC  420
            |||||||||||| ||||||||||||||||||||||||||||||| |||||||||||||||
Sbjct  405  ATCACATTATGGGCCATCAGTAGATTTAGCTATTTTTCTCTTCATCTTGCTGGTGCTTC  464

Query  421  TTCAATTTTAGGTGCAATTAACTTCATCACAACTATTGTAAATATACGATCAATTGGAAT  480
            ||||||||||||||||||||| || || ||||||||||| ||||||||||| ||||||||
Sbjct  465  TTCAATTTTAGGTGCAATTAATTTTATTACAACTATTGTGAATATACGATCTATTGGAAT  524

Query  481  AACAATAGAACGAATACCATTATTTGTTTGATCTGTTTTAATTACAGCTATTTTACTACT  540
            ||||||||||||||||||||||||||| ||||||||||||||||| || |||||| ||||
Sbjct  525  AACAATAGAACGAATACCATTATTTGTATGATCTGTTTTAATTACTGCAATTTTATTACT  584

Query  541  TCTATCTTTACCTGTATTAGCAGGTGCCATTACAATATTATTAACTGATCGAAACTTTAA  600
              ||||||||||||| ||||||||||| ||||||||| ||||||| |||||||| |||||
Sbjct  585  ATTATCTTTACCTGTTTTAGCAGGTGCTATTACAATACTATTAACCGATCGAAATTTTAA  644

Query  601  CACATCATTTTTTGATCCTTCAGGAGGAGGAGATCCCATTTTATATCAACACTTATTT  658
            ||| ||||||||||| |||||||||||||| ||||| |||||||||||||| ||||||
Sbjct  645  CACTTCATTTTTTGACCCTTCAGGAGGAGGGGATCCAATTTTATATCAACATTTATTT  702
```

>Rhipicephalus sanguineus isolate Tehran cytochrome oxidase subunit 1 gene, complete cds; mitochondrial
Sequence ID: KM494915.1 Length: 1539
Range 1: 45 to 702

Score:872 bits(472), Expect:0.0,
Identities:596/658(91%), Gaps:0/658(0%), Strand: Plus/Plus

```
Query  1    AACAATATATTTAATTTTTGGAGCATGATCAGGTATATTAGGACTTAGTATAAGAATATT  60
            |||||||||||||||||||||||||||||| || ||||||||| | || ||||||||| |
Sbjct  45   AACAATATATTTAATTTTTGGAGCATGATCCGGAATATTAGGATTAAGAATAAGAATACT  104

Query  61   AATTCGTATAGAATTAGGACAACCTGGAACTTTAATTGGAAATGATCAGATTTATAATGT  120
            |||||||||||||||||| |||||||||||| ||||||| |||||||| |||||||||||
Sbjct  105  AATTCGTATAGAATTAGGTCAACCTGGAACTCTAATTGGTAATGATCAAATTTATAATGT  164

Query  121  AATCGTAACAGCACATGCATTTATTATAATTTTCTTTATAGTTATACCAATCATAATCGG  180
            ||| || ||||| |||||||||||||||||||| |||||||| |||||||| ||||| ||
Sbjct  165  AATTGTTACAGCTCATGCATTTATTATAATTTTTTTTATAGTAATACCAATTATAATTGG  224

Query  181  AGGGTTCGGAAATTGATTAGTTCCTATTATATTAGGTGCCCCAGATATAGCATTTCCACG  240
             || || ||||| |||||||| ||||||||| |||| || |||||||||||||| |||||
Sbjct  225  TGGATTTGGAAACTGATTAGTACCTATTATACTAGGAGCTCCAGATATAGCATTCCCACG  284

Query  241  AATAAATAATATAAGATTTTGACTTTTACCCCCCTCATTATTTTTATTGATTAACTCTTC  300
            ||||||||||||||||||||||||| | || |||||||||||| |||| ||||| |||||
Sbjct  285  AATAAATAATATAAGATTTTGACTTCTTCCTCCCTCATTATTTATATTAATTAATTCTTC  344
```

```
Query  301  ATTAATTGAATCCGGAGCTGGAACAGGATGAACAGTTTATCCCCCTCTATCTTCAAATTT  360
            ||||||||| || ||||| || |||||||||||||||||||| || ||||| ||||||||
Sbjct  345  ATTAATTGAGTCAGGAGCAGGTACAGGATGAACAGTTTATCCTCCCCTATCCTCAAATTT  404

Query  361  ATCACATTATGGTCCATCAGTAGATTTAGCTATTTTTTCTCTTCACCTTGCTGGTGCTTC  420
            |||||||||||| |||||||||||||||||||||||||||||||| ||||||||||||||
Sbjct  405  ATCACATTATGGGCCATCAGTAGATTTAGCTATTTTTTCTCTTCATCTTGCTGGTGCTTC  464

Query  421  TTCAATTTTAGGTGCAATTAACTTCATCACAACTATTGTAAATATACGATCAATTGGAAT  480
            ||||||||||||||||||||| || || ||||||||||| ||||||||||| ||||||||
Sbjct  465  TTCAATTTTAGGTGCAATTAATTTTATTACAACTATTGTGAATATACGATCTATTGGAAT  524

Query  481  AACAATAGAACGAATACCATTATTTGTTTGATCTGTTTTAATTACAGCTATTTTACTACT  540
            |||||||||||||||||||||||||| |||||||||||||||||| || |||||| ||||
Sbjct  525  AACAATAGAACGAATACCATTATTTGTATGATCTGTTTTAATTACTGCAATTTTATTACT  584

Query  541  TCTATCTTTACCTGTATTAGCAGGTGCCATTACAATATTATTAACTGATCGAAACTTTAA  600
              ||||||||||||| ||||||||||| ||||||||| ||||||| |||||||| |||||
Sbjct  585  ATTATCTTTACCTGTTTTAGCAGGTGCTATTACAATACTATTAACCGATCGAAATTTTAA  644

Query  601  CACATCATTTTTTGATCCTTCAGGAGGAGGAGATCCCATTTTATATCAACACTTATTT  658
            ||| ||||||||||| |||||||||||||| ||||| |||||||||||||| ||||||
Sbjct  645  CACTTCATTTTTTGACCCTTCAGGAGGAGGGGATCCAATTTTATATCAACATTTATTT  702
```

>Rhipicephalus turanicus isolate COX1-18-Sheep cytochrome c oxidase subunit I (COX1) gene, partial cds; mitochondrial
Sequence ID: MT800314.1 Length: 673
Range 1: 8 to 664

Score:870 bits(471), Expect:0.0,
Identities:595/657(91%), Gaps:0/657(0%), Strand: Plus/Plus

```
Query  1    AACAATATATTTAATTTTTGGAGCATGATCAGGTATATTAGGACTTAGTATAAGAATATT  60
            ||||||||||||||||||||| ||||||||||| ||||||||||| || ||||||||| |
Sbjct  8    AACAATATATTTAATTTTTGGGGCATGATCAGGAATATTAGGACTGAGAATAAGAATACT  67

Query  61   AATTCGTATAGAATTAGGACAACCTGGAACTTTAATTGGAAATGATCAGATTTATAATGT  120
            |||||| ||||||||||||||||||||||||||||||||||||||||| |||||||| ||
Sbjct  68   AATTCGCATAGAATTAGGACAACCTGGAACTTTAATTGGAAATGATCAAATTTATAACGT  127

Query  121  AATCGTAACAGCACATGCATTTATTATAATTTTCTTTATAGTTATACCAATCATAATCGG  180
            ||| |||||||| |||||||||||||||||||| ||||| || ||||| || ||||| ||
Sbjct  128  AATTGTAACAGCTCATGCATTTATTATAATTTTTTTTATGGTAATACCCATTATAATTGG  187

Query  181  AGGGTTCGGAAATTGATTAGTTCCTATTATATTAGGTGCCCCAGATATAGCATTTCCACG  240
             || || ||||||||||||||||||||||||||||| || |||||||||||||| |||||
Sbjct  188  TGGATTTGGAAATTGATTAGTTCCTATTATATTAGGGGCTCCAGATATAGCATTCCCACG  247

Query  241  AATAAATAATATAAGATTTTGACTTTTACCCCCCTCATTATTTTTATTGATTAACTCTTC  300
             |||||||| |||||||||||| |  | || || ||||||||| |||| |||||||| ||
```

```
Sbjct  248   TATAAATAACATAAGATTTTGATTACTTCCTCCTTCATTATTTCTATTAATTAACTCCTC  307

Query  301   ATTAATTGAATCCGGAGCTGGAACAGGATGAACAGTTTATCCCCCTCTATCTTCAAATTT  360
             |||||||||||| ||||| |||||||||||||| |||||||||||| |||| ||||||||
Sbjct  308   ATTAATTGAATCGGGAGCAGGAACAGGATGAACTGTTTATCCCCCTTTATCCTCAAATTT  367

Query  361   ATCACATTATGGTCCATCAGTAGATTTAGCTATTTTTTCTCTTCACCTTGCTGGTGCTTC  420
             |||||||||||| |||||||||||||||||||||||||||||||| ||||||||||||||
Sbjct  368   ATCACATTATGGACCATCAGTAGATTTAGCTATTTTTTCTCTTCATCTTGCTGGTGCTTC  427

Query  421   TTCAATTTTAGGTGCAATTAACTTCATCACAACTATTGTAAATATACGATCAATTGGAAT  480
             ||||||| ||||||||||||| || || |||||||||||||| |||||||| ||||||||
Sbjct  428   TTCAATTCTAGGTGCAATTAATTTTATTACAACTATTGTAAACATACGATCTATTGGAAT  487

Query  481   AACAATAGAACGAATACCATTATTTGTTTGATCTGTTTTAATTACAGCTATTTTACTACT  540
             ||||||||||||||||||||||||||||||||||||||||||||| || || ||||| ||
Sbjct  488   AACAATAGAACGAATACCATTATTTGTTTGATCTGTTTTAATTACCGCCATCTTACTTCT  547

Query  541   TCTATCTTTACCTGTATTAGCAGGTGCCATTACAATATTATTAACTGATCGAAACTTTAA  600
               ||||||||||||| ||||||||||| |||||||||||||||||||||||||| || ||
Sbjct  548   CTTATCTTTACCTGTTTTAGCAGGTGCTATTACAATATTATTAACTGATCGAAATTTCAA  607

Query  601   CACATCATTTTTTGATCCTTCAGGAGGAGGAGATCCCATTTTATATCAACACTTATT  657
              || |||||||||||||||||||| ||||| ||||| |||||||| |||||||||||
Sbjct  608   TACTTCATTTTTTGATCCTTCAGGGGGAGGTGATCCAATTTTATACCAACACTTATT  664
```

>Rhipicephalus turanicus isolate 31X cytochrome oxidase subunit 1 (COI) gene, partial cds; mitochondrial
Sequence ID: KY606303.1 Length: 873
Range 1: 66 to 723

Score:861 bits(466), Expect:0.0,
Identities:594/658(90%), Gaps:0/658(0%), Strand: Plus/Plus

```
Query  1     AACAATATATTTAATTTTTGGAGCATGATCAGGTATATTAGGACTTAGTATAAGAATATT  60
             |||||||||||||||||||||||||||||| || ||||||||| | || |||||||||||
Sbjct  66    AACAATATATTTAATTTTTGGAGCATGATCCGGGATATTAGGATTAAGAATAAGAATATT  125

Query  61    AATTCGTATAGAATTAGGACAACCTGGAACTTTAATTGGAAATGATCAGATTTATAATGT  120
             |||||| ||||| ||||||||||||||||||||||||||||||||||| |||||||||||
Sbjct  126   AATTCGAATAGAGTTAGGACAACCTGGAACTTTAATTGGAAATGATCAAATTTATAATGT  185

Query  121   AATCGTAACAGCACATGCATTTATTATAATTTTCTTTATAGTTATACCAATCATAATCGG  180
             ||| |||||||| ||||||||||||||||||||| |||||||| |||||||||||||| ||
Sbjct  186   AATTGTAACAGCTCATGCATTTATTATAATTTTTTTTATAGTAATACCAATCATAATTGG  245

Query  181   AGGGTTCGGAAATTGATTAGTTCCTATTATATTAGGTGCCCCAGATATAGCATTTCCACG  240
             ||| || ||||| |||||||| ||||| ||||| || ||||| ||||||||||||||
Sbjct  246   AGGATTTGGAAACTGATTAGTGCCCATTATGTTAGGAGCTCCAGACATAGCATTTCCACG  305

Query  241   AATAAATAATATAAGATTTTGACTTTTACCCCCTCATTATTTTTATTGATTAACTCTTC  300
```

```
                |||||||||||||||||||||| |  | || ||||||||||| ||||| ||||| |||||
Sbjct  306  AATAAATAATATAAGATTTTGATTACTTCCTCCCTCATTATTCTTATTAATTAATTCTTC  365

Query  301  ATTAATTGAATCCGGAGCTGGAACAGGATGAACAGTTTATCCCCCTCTATCTTCAAATTT  360
            | | |||||||| ||||| || ||||||||||| || || || ||| |||||||||||||
Sbjct  366  ACTGATTGAATCTGGAGCAGGGACAGGATGAACTGTCTACCCTCCTTTATCTTCAAATTT  425

Query  361  ATCACATTATGGTCCATCAGTAGATTTAGCTATTTTTTCTCTTCACCTTGCTGGTGCTTC  420
            ||| |||||||| |||||||||||||||||||| || |||||||| || |||||||||||
Sbjct  426  ATCGCATTATGGACCATCAGTAGATTTAGCTATCTTCTCTCTTCATCTCGCTGGTGCTTC  485

Query  421  TTCAATTTTAGGTGCAATTAACTTCATCACAACTATTGTAAATATACGATCAATTGGAAT  480
            ||||||||||||||||||||| ||||| |||||||||||||| |||||||| ||||||||
Sbjct  486  TTCAATTTTAGGTGCAATTAATTTCATTACAACTATTGTAAACATACGATCTATTGGAAT  545

Query  481  AACAATAGAACGAATACCATTATTTGTTTGATCTGTTTTAATTACAGCTATTTTACTACT  540
            |||||||||||||||||||||||| ||||||||||||||||| || ||||||||| | ||
Sbjct  546  AACAATAGAACGAATACCATTATTCGTTTGATCTGTTTTAATCACCGCTATTTTATTGCT  605

Query  541  TCTATCTTTACCTGTATTAGCAGGTGCCATTACAATATTATTAACTGATCGAAACTTTAA  600
            ||||||||||||||| |||||||||||||||||||||||||||||||||||||| || ||
Sbjct  606  TCTATCTTTACCTGTTTTAGCAGGTGCCATTACAATATTATTAACTGATCGAAATTTCAA  665

Query  601  CACATCATTTTTTGATCCTTCAGGAGGAGGAGATCCCATTTTATATCAACACTTATTT  658
            ||| ||||||||||| |||||||||||||| || || |||||||||||||| ||||||
Sbjct  666  CACTTCATTTTTTGACCCTTCAGGAGGAGGTGACCCTATTTTATATCAACATTTATTT  723
```

>Rhipicephalus turanicus isolate 19X cytochrome oxidase subunit 1 (COI) gene, partial cds; mitochondrial
Sequence ID: KY606302.1 Length: 873
Range 1: 66 to 723

Score:861 bits(466), Expect:0.0,
Identities:594/658(90%), Gaps:0/658(0%), Strand: Plus/Plus

```
Query  1    AACAATATATTTAATTTTTGGAGCATGATCAGGTATATTAGGACTTAGTATAAGAATATT  60
            |||||||||||||||||||||||||||||| || ||||||||| | || |||||||||||
Sbjct  66   AACAATATATTTAATTTTTGGAGCATGATCCGGGATATTAGGATTAAGAATAAGAATATT  125

Query  61   AATTCGTATAGAATTAGGACAACCTGGAACTTTAATTGGAAATGATCAGATTTATAATGT  120
            |||||| ||||| ||||||||||||||||||||||||||||||||||| |||||||||||
Sbjct  126  AATTCGAATAGAGTTAGGACAACCTGGAACTTTAATTGGAAATGATCAAATTTATAATGT  185

Query  121  AATCGTAACAGCACATGCATTTATTATAATTTTCTTTATAGTTATACCAATCATAATCGG  180
            ||| |||||||| |||||||||||||||||||| ||||||| ||||||||||||||| ||
Sbjct  186  AATTGTAACAGCTCATGCATTTATTATAATTTTTTTTATAGTAATACCAATCATAATTGG  245

Query  181  AGGGTTCGGAAATTGATTAGTTCCTATTATATTAGGTGCCCCAGATATAGCATTTCCACG  240
            ||| || ||||| |||||||| || ||||| ||||| || ||||| ||||||||||||||
Sbjct  246  AGGATTTGGAAACTGATTAGTGCCCATTATGTTAGGAGCTCCAGACATAGCATTTCCACG  305
```

```
Query  241  AATAAATAATATAAGATTTTGACTTTTACCCCCCTCATTATTTTTATTGATTAACTCTTC  300
            |||||||||||||||||||||| |  | || ||||||||||| ||||| ||||| |||||
Sbjct  306  AATAAATAATATAAGATTTTGATTACTTCCTCCCTCATTATTCTTATTAATTAATTCTTC  365

Query  301  ATTAATTGAATCCGGAGCTGGAACAGGATGAACAGTTTATCCCCCTCTATCTTCAAATTT  360
            | | |||||||| ||||| || ||||||||||| || || || ||| |||||||||||||
Sbjct  366  ACTGATTGAATCTGGAGCAGGGACAGGATGAACTGTCTACCCTCCTTTATCTTCAAATTT  425

Query  361  ATCACATTATGGTCCATCAGTAGATTTAGCTATTTTTTCTCTTCACCTTGCTGGTGCTTC  420
            ||| |||||||| |||||||||||||||||||| || |||||||| || |||||||||||
Sbjct  426  ATCGCATTATGGACCATCAGTAGATTTAGCTATCTTCTCTCTTCATCTCGCTGGTGCTTC  485

Query  421  TTCAATTTTAGGTGCAATTAACTTCATCACAACTATTGTAAATATACGATCAATTGGAAT  480
            ||||||||||||||||||||| ||||| |||||||||||||| |||||||| ||||||||
Sbjct  486  TTCAATTTTAGGTGCAATTAATTTCATTACAACTATTGTAAACATACGATCTATTGGAAT  545

Query  481  AACAATAGAACGAATACCATTATTTGTTTGATCTGTTTTAATTACAGCTATTTTACTACT  540
            |||||||||||||||||||||||| ||||||||||||||||| || ||||||||| | ||
Sbjct  546  AACAATAGAACGAATACCATTATTCGTTTGATCTGTTTTAATCACCGCTATTTTATTGCT  605

Query  541  TCTATCTTTACCTGTATTAGCAGGTGCCATTACAATATTATTAACTGATCGAAACTTTAA  600
            ||||||||||||||| |||||||||||||||||||||||||||||||||||||| || ||
Sbjct  606  TCTATCTTTACCTGTTTTAGCAGGTGCCATTACAATATTATTAACTGATCGAAATTTCAA  665

Query  601  CACATCATTTTTTGATCCTTCAGGAGGAGGAGATCCCATTTTATATCAACACTTATTT  658
            ||| ||||||||||| |||||||||||||| || || |||||||||||||| ||||||
Sbjct  666  CACTTCATTTTTTGACCCTTCAGGAGGAGGTGACCCTATTTTATATCAACATTTATTT  723
```

>Rhipicephalus turanicus isolate 19C cytochrome oxidase subunit 1 (COI) gene, partial cds; mitochondrial
Sequence ID: KY606301.1 Length: 873
Range 1: 66 to 723

Score:861 bits(466), Expect:0.0,
Identities:594/658(90%), Gaps:0/658(0%), Strand: Plus/Plus

```
Query  1    AACAATATATTTAATTTTTGGAGCATGATCAGGTATATTAGGACTTAGTATAAGAATATT  60
            |||||||||||||||||||||||||||||| || ||||||||| | || |||||||||||
Sbjct  66   AACAATATATTTAATTTTTGGAGCATGATCCGGGATATTAGGATTAAGAATAAGAATATT  125

Query  61   AATTCGTATAGAATTAGGACAACCTGGAACTTTAATTGGAAATGATCAGATTTATAATGT  120
            |||||| ||||| ||||||||||||||||||||||||||||||||||| |||||||||||
Sbjct  126  AATTCGAATAGAGTTAGGACAACCTGGAACTTTAATTGGAAATGATCAAATTTATAATGT  185

Query  121  AATCGTAACAGCACATGCATTTATTATAATTTTCTTTATAGTTATACCAATCATAATCGG  180
            ||| |||||||| |||||||||||||||||||| |||||||| |||||||||||||| ||
Sbjct  186  AATTGTAACAGCTCATGCATTTATTATAATTTTTTTTATAGTAATACCAATCATAATTGG  245

Query  181  AGGGTTCGGAAATTGATTAGTTCCTATTATATTAGGTGCCCCAGATATAGCATTTCCACG  240
            ||| || ||||| |||||||| || ||||| ||||| || ||||| ||||||||||||||
Sbjct  246  AGGATTTGGAAACTGATTAGTGCCCATTATGTTAGGAGCTCCAGACATAGCATTTCCACG  305
```

```
Query  241  AATAAATAATATAAGATTTTGACTTTTACCCCCCTCATTATTTTTATTGATTAACTCTTC  300
            |||||||||||||||||||||| |  | || ||||||||||| ||||| ||||| |||||
Sbjct  306  AATAAATAATATAAGATTTTGATTACTTCCTCCCTCATTATTCTTATTAATTAATTCTTC  365

Query  301  ATTAATTGAATCCGGAGCTGGAACAGGATGAACAGTTTATCCCCCTCTATCTTCAAATTT  360
            | | |||||||| ||||| || ||||||||||| || || || ||| |||||||||||||
Sbjct  366  ACTGATTGAATCTGGAGCAGGGACAGGATGAACTGTCTACCCTCCTTTATCTTCAAATTT  425

Query  361  ATCACATTATGGTCCATCAGTAGATTTAGCTATTTTTTCTCTTCACCTTGCTGGTGCTTC  420
            ||| |||||||| |||||||||||||||||||| || |||||||| || |||||||||||
Sbjct  426  ATCGCATTATGGACCATCAGTAGATTTAGCTATCTTCTCTCTTCATCTCGCTGGTGCTTC  485

Query  421  TTCAATTTTAGGTGCAATTAACTTCATCACAACTATTGTAAATATACGATCAATTGGAAT  480
            ||||||||||||||||||||| ||||| |||||||||||||| |||||||| ||||||||
Sbjct  486  TTCAATTTTAGGTGCAATTAATTTCATTACAACTATTGTAAACATACGATCTATTGGAAT  545

Query  481  AACAATAGAACGAATACCATTATTTGTTTGATCTGTTTTAATTACAGCTATTTTACTACT  540
            |||||||||||||||||||||||| ||||||||||||||||| || ||||||||| | ||
Sbjct  546  AACAATAGAACGAATACCATTATTCGTTTGATCTGTTTTAATCACCGCTATTTTATTGCT  605

Query  541  TCTATCTTTACCTGTATTAGCAGGTGCCATTACAATATTATTAACTGATCGAAACTTTAA  600
            ||||||||||||||| |||||||||||||||||||||||||||||||||||||| || ||
Sbjct  606  TCTATCTTTACCTGTTTTAGCAGGTGCCATTACAATATTATTAACTGATCGAAATTTCAA  665

Query  601  CACATCATTTTTTGATCCTTCAGGAGGAGGAGATCCCATTTTATATCAACACTTATTT  658
            ||| ||||||||||| |||||||||||||| || || |||||||||||||| ||||||
Sbjct  666  CACTTCATTTTTTGACCCTTCAGGAGGAGGTGACCCTATTTTATATCAACATTTATTT  723
```

>Rhipicephalus turanicus isolate 12X cytochrome oxidase subunit 1 (COI) gene, partial cds; mitochondrial
Sequence ID: KY606300.1 Length: 873
Range 1: 66 to 723

Score:861 bits(466), Expect:0.0,
Identities:594/658(90%), Gaps:0/658(0%), Strand: Plus/Plus

```
Query  1    AACAATATATTTAATTTTTGGAGCATGATCAGGTATATTAGGACTTAGTATAAGAATATT  60
            |||||||||||||||||||||||||||||| || ||||||||| | || |||||||||||
Sbjct  66   AACAATATATTTAATTTTTGGAGCATGATCCGGGATATTAGGATTAAGAATAAGAATATT  125

Query  61   AATTCGTATAGAATTAGGACAACCTGGAACTTTAATTGGAAATGATCAGATTTATAATGT  120
            |||||| ||||| ||||||||||||||||||||||||||||||||||| |||||||||||
Sbjct  126  AATTCGAATAGAGTTAGGACAACCTGGAACTTTAATTGGAAATGATCAAATTTATAATGT  185

Query  121  AATCGTAACAGCACATGCATTTATTATAATTTTCTTTATAGTTATACCAATCATAATCGG  180
            ||| |||||||| |||||||||||||||||||| |||||||| |||||||||||||| ||
Sbjct  186  AATTGTAACAGCTCATGCATTTATTATAATTTTTTTTATAGTAATACCAATCATAATTGG  245

Query  181  AGGGTTCGGAAATTGATTAGTTCCTATTATATTAGGTGCCCCAGATATAGCATTTCCACG  240
            ||| || ||||| |||||||| || ||||| ||||| || ||||| ||||||||||||||
```

```
Sbjct  246   AGGATTTGGAAACTGATTAGTGCCCATTATGTTAGGAGCTCCAGACATAGCATTTCCACG  305

Query  241   AATAAATAATATAAGATTTTGACTTTTACCCCCCTCATTATTTTTATTGATTAACTCTTC  300
             |||||||||||||||||||||| |  | || ||||||||||| ||||| ||||| |||||
Sbjct  306   AATAAATAATATAAGATTTTGATTACTTCCTCCCTCATTATTCTTATTAATTAATTCTTC  365

Query  301   ATTAATTGAATCCGGAGCTGGAACAGGATGAACAGTTTATCCCCCTCTATCTTCAAATTT  360
             | | |||||||| ||||| || ||||||||||| || || || ||| |||||||||||||
Sbjct  366   ACTGATTGAATCTGGAGCAGGGACAGGATGAACTGTCTACCCTCCTTTATCTTCAAATTT  425

Query  361   ATCACATTATGGTCCATCAGTAGATTTAGCTATTTTTTCTCTTCACCTTGCTGGTGCTTC  420
             ||| |||||||| |||||||||||||||||||| || |||||||| || |||||||||||
Sbjct  426   ATCGCATTATGGACCATCAGTAGATTTAGCTATCTTCTCTCTTCATCTCGCTGGTGCTTC  485

Query  421   TTCAATTTTAGGTGCAATTAACTTCATCACAACTATTGTAAATATACGATCAATTGGAAT  480
             ||||||||||||||||||||| ||||| |||||||||||||| |||||||| ||||||||
Sbjct  486   TTCAATTTTAGGTGCAATTAATTTCATTACAACTATTGTAAACATACGATCTATTGGAAT  545

Query  481   AACAATAGAACGAATACCATTATTTGTTTGATCTGTTTTAATTACAGCTATTTTACTACT  540
             |||||||||||||||||||||||| ||||||||||||||||| || ||||||||| | ||
Sbjct  546   AACAATAGAACGAATACCATTATTCGTTTGATCTGTTTTAATCACCGCTATTTTATTGCT  605

Query  541   TCTATCTTTACCTGTATTAGCAGGTGCCATTACAATATTATTAACTGATCGAAACTTTAA  600
             ||||||||||||||| |||||||||||||||||||||||||||||||||||||| || ||
Sbjct  606   TCTATCTTTACCTGTTTTAGCAGGTGCCATTACAATATTATTAACTGATCGAAATTTCAA  665

Query  601   CACATCATTTTTTGATCCTTCAGGAGGAGGAGATCCCATTTTATATCAACACTTATTT  658
             ||| ||||||||||| |||||||||||||| || || |||||||||||||| ||||||
Sbjct  666   CACTTCATTTTTTGACCCTTCAGGAGGAGGTGACCCTATTTTATATCAACATTTATTT  723
```

>Rhipicephalus turanicus isolate 12C cytochrome oxidase subunit 1 (COI) gene, partial cds; mitochondrial
Sequence ID: KY606299.1 Length: 873
Range 1: 66 to 723

Score:861 bits(466), Expect:0.0,
Identities:594/658(90%), Gaps:0/658(0%), Strand: Plus/Plus

```
Query  1     AACAATATATTTAATTTTTGGAGCATGATCAGGTATATTAGGACTTAGTATAAGAATATT  60
             |||||||||||||||||||||||||||||| || ||||||||| | || |||||||||||
Sbjct  66    AACAATATATTTAATTTTTGGAGCATGATCCGGGATATTAGGATTAAGAATAAGAATATT  125

Query  61    AATTCGTATAGAATTAGGACAACCTGGAACTTTAATTGGAAATGATCAGATTTATAATGT  120
             |||||| ||||| ||||||||||||||||||||||||||||||||||| |||||||||||
Sbjct  126   AATTCGAATAGAGTTAGGACAACCTGGAACTTTAATTGGAAATGATCAAATTTATAATGT  185

Query  121   AATCGTAACAGCACATGCATTTATTATAATTTTCTTTATAGTTATACCAATCATAATCGG  180
             ||| |||||||| |||||||||||||||||||| |||||||| |||||||||||||| ||
Sbjct  186   AATTGTAACAGCTCATGCATTTATTATAATTTTTTTTATAGTAATACCAATCATAATTGG  245

Query  181   AGGGTTCGGAAATTGATTAGTTCCTATTATATTAGGTGCCCCAGATATAGCATTTCCACG  240
```

```
                ||| || ||||| |||||||| || ||||| ||||| || ||||| ||||||||||||||
Sbjct  246   AGGATTTGGAAACTGATTAGTGCCCATTATGTTAGGAGCTCCAGACATAGCATTTCCACG  305

Query  241   AATAAATAATATAAGATTTTGACTTTTACCCCCCTCATTATTTTTATTGATTAACTCTTC  300
             |||||||||||||||||||||| |  | || ||||||||||| ||||| ||||| |||||
Sbjct  306   AATAAATAATATAAGATTTTGATTACTTCCTCCCTCATTATTCTTATTAATTAATTCTTC  365

Query  301   ATTAATTGAATCCGGAGCTGGAACAGGATGAACAGTTTATCCCCCTCTATCTTCAAATTT  360
             | | |||||||| ||||| || ||||||||||| || || || ||| |||||||||||||
Sbjct  366   ACTGATTGAATCTGGAGCAGGGACAGGATGAACTGTCTACCCTCCTTTATCTTCAAATTT  425

Query  361   ATCACATTATGGTCCATCAGTAGATTTAGCTATTTTTTCTCTTCACCTTGCTGGTGCTTC  420
             ||| |||||||| |||||||||||||||||||| || |||||||| || |||||||||||
Sbjct  426   ATCGCATTATGGACCATCAGTAGATTTAGCTATCTTCTCTCTTCATCTCGCTGGTGCTTC  485

Query  421   TTCAATTTTAGGTGCAATTAACTTCATCACAACTATTGTAAATATACGATCAATTGGAAT  480
             ||||||||||||||||||||| ||||| |||||||||||||| |||||||| ||||||||
Sbjct  486   TTCAATTTTAGGTGCAATTAATTTCATTACAACTATTGTAAACATACGATCTATTGGAAT  545

Query  481   AACAATAGAACGAATACCATTATTTGTTTGATCTGTTTTAATTACAGCTATTTTACTACT  540
             |||||||||||||||||||||||| ||||||||||||||||| || ||||||||| | ||
Sbjct  546   AACAATAGAACGAATACCATTATTCGTTTGATCTGTTTTAATCACCGCTATTTTATTGCT  605

Query  541   TCTATCTTTACCTGTATTAGCAGGTGCCATTACAATATTATTAACTGATCGAAACTTTAA  600
             ||||||||||||||| |||||||||||||||||||||||||||||||||||||| || ||
Sbjct  606   TCTATCTTTACCTGTTTTAGCAGGTGCCATTACAATATTATTAACTGATCGAAATTTCAA  665

Query  601   CACATCATTTTTTGATCCTTCAGGAGGAGGAGATCCCATTTTATATCAACACTTATTT  658
             ||| ||||||||||| |||||||||||||| || || |||||||||||||| ||||||
Sbjct  666   CACTTCATTTTTTGACCCTTCAGGAGGAGGTGACCCTATTTTATATCAACATTTATTT  723
```

>Rhipicephalus camicasi isolate SC0126 mitochondrion, complete genome
Sequence ID: NC_061616.1 Length: 14725
>Rhipicephalus camicasi isolate SC0126 mitochondrion, complete genome
Sequence ID: MZ323229.1 Length: 14725
Range 1: 45 to 702

Score:861 bits(466), Expect:0.0,
Identities:595/659(90%), Gaps:2/659(0%), Strand: Plus/Plus

```
Query  1     AACAATATATTTAATTTTTGGAGCATGATCAGGTATATTAGGACTTAGTATAAGAATATT  60
             ||||||||| |||||||||||||||||||| || ||||||||| | || ||||||||| |
Sbjct  45    AACAATATACTTAATTTTTGGAGCATGATCTGGAATATTAGGATTAAGAATAAGAATACT  104

Query  61    AATTCGTATAGAATTAGGACAACCTGGAACTTTAATTGGAAATGATCAGATTTATAATGT  120
             |||||||||||||||||| |||||||||||||||||||||||||| || |||||||||||
Sbjct  105   AATTCGTATAGAATTAGGCCAACCTGGAACTTTAATTGGAAATGACCAAATTTATAATGT  164

Query  121   AATCGTAACAGCACATGCATTTATTATAATTTTCTTTATAGTTATACCAATCATAATCGG  180
             ||| ||||||||||||||||||||||||||||| |||||||| |||||||| || || ||
Sbjct  165   AATTGTAACAGCACATGCATTTATTATAATTTTTTTTATAGTAATACCAATTATGATTGG  224
```

```
Query  181   AGGGTTCGGAAATTGATTAGTTCCTATTATATTAGGTGCCCCAGATATAGCATTTCCACG  240
             ||| ||||||||||||||||| |||||||||||||| || ||||||||||||||||||||
Sbjct  225   AGGATTCGGAAATTGATTAGTCCCTATTATATTAGGGGCTCCAGATATAGCATTTCCACG  284

Query  241   AATAAATAATATAAGATTTTGACTTTTACCCCCCTCATTATTTTTATTGATTAACTCTTC  300
             |||||||||||||||||||||| |  | || || |||||||||||||| ||||| |||||
Sbjct  285   AATAAATAATATAAGATTTTGATTACTTCCTCCTTCATTATTTTTATTAATTAATTCTTC  344

Query  301   ATTAATTGAATCCGGAGCTGGAACAGGATGAACAGTTTATCC-CCCTCTATCTTCAAATT  359
             |||||||||||| ||||| || |||||||||||||||| || |||| |||| |||||||
Sbjct  345   ATTAATTGAATCAGGAGCAGGGACAGGATGAACAGTTTACCCTCCCT-TATCATCAAATT  403

Query  360   TATCACATTATGGTCCATCAGTAGATTTAGCTATTTTTTCTCTTCACCTTGCTGGTGCTT  419
             ||||||| |||||||||||||||||| ||||||||||||||||||| |||||||||||||
Sbjct  404   TATCACACTATGGTCCATCAGTAGATCTAGCTATTTTTTCTCTTCATCTTGCTGGTGCTT  463

Query  420   CTTCAATTTTAGGTGCAATTAACTTCATCACAACTATTGTAAATATACGATCAATTGGAA  479
             ||||||||||||| |||||||| ||||| ||||| |||||||| |||||||| |||||||
Sbjct  464   CTTCAATTTTAGGCGCAATTAATTTCATTACAACCATTGTAAACATACGATCTATTGGAA  523

Query  480   TAACAATAGAACGAATACCATTATTTGTTTGATCTGTTTTAATTACAGCTATTTTACTAC  539
             ||||||||||||||||||| |||||||| ||||||||||||||||| || |||||| || 
Sbjct  524   TAACAATAGAACGAATACCTTTATTTGTATGATCTGTTTTAATTACTGCAATTTTATTAT  583

Query  540   TTCTATCTTTACCTGTATTAGCAGGTGCCATTACAATATTATTAACTGATCGAAACTTTA  599
             |  |||||||||| || ||||| ||||| |||||||||||||||||||||||||| ||||
Sbjct  584   TATTATCTTTACCAGTTTTAGCTGGTGCAATTACAATATTATTAACTGATCGAAATTTTA  643

Query  600   ACACATCATTTTTTGATCCTTCAGGAGGAGGAGATCCCATTTTATATCAACACTTATTT  658
             | || || || |||||||||||||||||||| ||||| |||||||||||||| ||||||
Sbjct  644   ATACCTCGTTCTTTGATCCTTCAGGAGGAGGGGATCCAATTTTATATCAACATTTATTT  702
```

>Rhipicephalus turanicus voucher CROBB808 cytochrome oxidase subunit 1 (COI) gene, partial cds; mitochondrial
Sequence ID: MZ305547.1 Length: 658
Range 1: 1 to 658

Score:861 bits(466), Expect:0.0,
Identities:594/658(90%), Gaps:0/658(0%), Strand: Plus/Plus

```
Query  1     AACAATATATTTAATTTTTGGAGCATGATCAGGTATATTAGGACTTAGTATAAGAATATT  60
             |||||||||||||||||||||||||||||| || ||||||||| | || |||||||||||
Sbjct  1     AACAATATATTTAATTTTTGGAGCATGATCCGGGATATTAGGATTAAGAATAAGAATATT  60

Query  61    AATTCGTATAGAATTAGGACAACCTGGAACTTTAATTGGAAATGATCAGATTTATAATGT  120
             |||||| ||||| |||||||||||||| |||||||||||||||||||| |||||||||||
Sbjct  61    AATTCGAATAGAGTTAGGACAACCTGGGACTTTAATTGGAAATGATCAAATTTATAATGT  120

Query  121   AATCGTAACAGCACATGCATTTATTATAATTTTCTTTATAGTTATACCAATCATAATCGG  180
             ||| |||||||| |||||||||||||||||||| |||||||| |||||||||||||| ||
```

```
Sbjct  121  AATTGTAACAGCTCATGCATTTATTATAATTTTTTTTATAGTAATACCAATCATAATTGG  180

Query  181  AGGGTTCGGAAATTGATTAGTTCCTATTATATTAGGTGCCCCAGATATAGCATTTCCACG  240
            ||| || ||||| |||||||| || ||||| ||||| || ||||| ||||||||||||||
Sbjct  181  AGGATTTGGAAACTGATTAGTGCCCATTATGTTAGGAGCTCCAGACATAGCATTTCCACG  240

Query  241  AATAAATAATATAAGATTTTGACTTTTACCCCCCTCATTATTTTTATTGATTAACTCTTC  300
            |||||||||||||||||||||| |  | || ||||||||||| ||||| ||||| |||||
Sbjct  241  AATAAATAATATAAGATTTTGATTACTTCCTCCCTCATTATTCTTATTAATTAATTCTTC  300

Query  301  ATTAATTGAATCCGGAGCTGGAACAGGATGAACAGTTTATCCCCCTCTATCTTCAAATTT  360
            | | |||||||| ||||| || ||||||||||| || || || ||| |||||||||||||
Sbjct  301  ACTGATTGAATCTGGAGCAGGGACAGGATGAACTGTCTACCCTCCTTTATCTTCAAATTT  360

Query  361  ATCACATTATGGTCCATCAGTAGATTTAGCTATTTTTTCTCTTCACCTTGCTGGTGCTTC  420
            ||| |||||||| |||||||||||||||||||| || |||||||| || |||||||||||
Sbjct  361  ATCGCATTATGGACCATCAGTAGATTTAGCTATCTTCTCTCTTCATCTCGCTGGTGCTTC  420

Query  421  TTCAATTTTAGGTGCAATTAACTTCATCACAACTATTGTAAATATACGATCAATTGGAAT  480
            ||||||||||||||||||||| ||||| ||||||||||||||||||||||| ||||||||
Sbjct  421  TTCAATTTTAGGTGCAATTAATTTCATTACAACTATTGTAAATATACGATCTATTGGAAT  480

Query  481  AACAATAGAACGAATACCATTATTTGTTTGATCTGTTTTAATTACAGCTATTTTACTACT  540
            |||||||||||||||||||||||| ||||||||||||||||| || ||||||||| | ||
Sbjct  481  AACAATAGAACGAATACCATTATTCGTTTGATCTGTTTTAATCACCGCTATTTTATTGCT  540

Query  541  TCTATCTTTACCTGTATTAGCAGGTGCCATTACAATATTATTAACTGATCGAAACTTTAA  600
            ||||||||||||||| |||||||||||||||||||||||||||||||||||||| || ||
Sbjct  541  TCTATCTTTACCTGTTTTAGCAGGTGCCATTACAATATTATTAACTGATCGAAATTTCAA  600

Query  601  CACATCATTTTTTGATCCTTCAGGAGGAGGAGATCCCATTTTATATCAACACTTATTT  658
            ||| ||||||||||| |||||||||||||| || || |||||||||||||| ||||||
Sbjct  601  CACTTCATTTTTTGACCCTTCAGGAGGAGGTGACCCTATTTTATATCAACATTTATTT  658
```

>Rhipicephalus turanicus isolate Xinjiang-WQIA cytochrome oxidase subunit 1 (COI) gene, partial cds; mitochondrial
Sequence ID: MF002581.1 Length: 889
Range 1: 74 to 731

Score:850 bits(460), Expect:0.0,
Identities:592/658(90%), Gaps:0/658(0%), Strand: Plus/Plus

```
Query  1    AACAATATATTTAATTTTTGGAGCATGATCAGGTATATTAGGACTTAGTATAAGAATATT  60
            ||||||||||||||||||||| ||||||||||| ||||||||||| || |||||||||||
Sbjct  74   AACAATATATTTAATTTTTGGGGCATGATCAGGAATATTAGGACTAAGAATAAGAATATT  133

Query  61   AATTCGTATAGAATTAGGACAACCTGGAACTTTAATTGGAAATGATCAGATTTATAATGT  120
            |||||| ||||||||||| ||||||||||||||||||||||||||||| |||||||||||
Sbjct  134  AATTCGAATAGAATTAGGCCAACCTGGAACTTTAATTGGAAATGATCAAATTTATAATGT  193

Query  121  AATCGTAACAGCACATGCATTTATTATAATTTTCTTTATAGTTATACCAATCATAATCGG  180
```

```
            ||| |||||||| |||||||||||||||||||| || ||||| |||||||| ||||| ||
Sbjct  194  AATTGTAACAGCCCATGCATTTATTATAATTTTTTTCATAGTAATACCAATTATAATTGG  253

Query  181  AGGGTTCGGAAATTGATTAGTTCCTATTATATTAGGTGCCCCAGATATAGCATTTCCACG  240
             || || ||||| |||||||| |||||||||||||| || ||||||||||||||||||||
Sbjct  254  TGGATTTGGAAACTGATTAGTACCTATTATATTAGGAGCTCCAGATATAGCATTTCCACG  313

Query  241  AATAAATAATATAAGATTTTGACTTTTACCCCCCTCATTATTTTTATTGATTAACTCTTC  300
            ||||||||| |||||||||||| |  | ||||| ||||||||| |||| ||||| || ||
Sbjct  314  AATAAATAACATAAGATTTTGATTACTTCCCCCTTCATTATTTCTATTAATTAATTCCTC  373

Query  301  ATTAATTGAATCCGGAGCTGGAACAGGATGAACAGTTTATCCCCCTCTATCTTCAAATTT  360
            |||||||||||| ||||| ||||| |||||||| |||||||| ||| |||| ||||||||
Sbjct  374  ATTAATTGAATCAGGAGCAGGAACTGGATGAACTGTTTATCCGCCTTTATCATCAAATTT  433

Query  361  ATCACATTATGGTCCATCAGTAGATTTAGCTATTTTTTCTCTTCACCTTGCTGGTGCTTC  420
            |||||| ||||| ||||||||||||||||||||||| |||||||| ||||||||||||||
Sbjct  434  ATCACACTATGGACCATCAGTAGATTTAGCTATTTTCTCTCTTCATCTTGCTGGTGCTTC  493

Query  421  TTCAATTTTAGGTGCAATTAACTTCATCACAACTATTGTAAATATACGATCAATTGGAAT  480
            |||||||||||||||||| || || || || |||||| ||||||||||||| ||||||||
Sbjct  494  TTCAATTTTAGGTGCAATCAATTTTATTACGACTATTATAAATATACGATCTATTGGAAT  553

Query  481  AACAATAGAACGAATACCATTATTTGTTTGATCTGTTTTAATTACAGCTATTTTACTACT  540
            ||||||||||||||| ||| ||||||||||||||||||||||||| ||||||||  ||||
Sbjct  554  AACAATAGAACGAATGCCACTATTTGTTTGATCTGTTTTAATTACTGCTATTTTGTTACT  613

Query  541  TCTATCTTTACCTGTATTAGCAGGTGCCATTACAATATTATTAACTGATCGAAACTTTAA  600
            | ||||||| ||||| |||||||| ||||||||||||||||||||||||||||| || ||
Sbjct  614  TTTATCTTTGCCTGTTTTAGCAGGAGCCATTACAATATTATTAACTGATCGAAATTTCAA  673

Query  601  CACATCATTTTTTGATCCTTCAGGAGGAGGAGATCCCATTTTATATCAACACTTATTT  658
            ||| ||||||||||||||||||||||| || ||||| |||||||| ||||| ||||||
Sbjct  674  CACTTCATTTTTTGATCCTTCAGGAGGGGGTGATCCAATTTTATACCAACATTTATTT  731
```

>Rhipicephalus turanicus isolate Xinjiang-WS cytochrome oxidase subunit 1 (COI) gene, partial cds; mitochondrial
Sequence ID: MF002580.1 Length: 889
Range 1: 74 to 731

Score:850 bits(460), Expect:0.0,
Identities:592/658(90%), Gaps:0/658(0%), Strand: Plus/Plus

```
Query  1    AACAATATATTTAATTTTTGGAGCATGATCAGGTATATTAGGACTTAGTATAAGAATATT  60
            ||||||||||||||||||||| ||||||||||| ||||||||||| || |||||||||||
Sbjct  74   AACAATATATTTAATTTTTGGGGCATGATCAGGAATATTAGGACTAAGAATAAGAATATT  133

Query  61   AATTCGTATAGAATTAGGACAACCTGGAACTTTAATTGGAAATGATCAGATTTATAATGT  120
            |||||| ||||||||||| ||||||||||||||||||||||||||||| |||||||||||
Sbjct  134  AATTCGAATAGAATTAGGCCAACCTGGAACTTTAATTGGAAATGATCAAATTTATAATGT  193
```

```
Query  121   AATCGTAACAGCACATGCATTTATTATAATTTTCTTTATAGTTATACCAATCATAATCGG  180
             ||| |||||||| |||||||||||||||||||| || ||||| |||||||| ||||| ||
Sbjct  194   AATTGTAACAGCCCATGCATTTATTATAATTTTTTTCATAGTAATACCAATTATAATTGG  253

Query  181   AGGGTTCGGAAATTGATTAGTTCCTATTATATTAGGTGCCCCAGATATAGCATTTCCACG  240
              || || ||||| |||||||| |||||||||||||| || ||||||||||||||||||||
Sbjct  254   TGGATTTGGAAACTGATTAGTACCTATTATATTAGGAGCTCCAGATATAGCATTTCCACG  313

Query  241   AATAAATAATATAAGATTTTGACTTTTACCCCCCTCATTATTTTTATTGATTAACTCTTC  300
             ||||||||| |||||||||||| |  | ||||| ||||||||| |||| ||||| || ||
Sbjct  314   AATAAATAACATAAGATTTTGATTACTTCCCCCTTCATTATTTCTATTAATTAATTCCTC  373

Query  301   ATTAATTGAATCCGGAGCTGGAACAGGATGAACAGTTTATCCCCCTCTATCTTCAAATTT  360
             |||||||||||| ||||| ||||| |||||||| |||||||| ||| |||| ||||||||
Sbjct  374   ATTAATTGAATCAGGAGCAGGAACTGGATGAACTGTTTATCCGCCTTTATCATCAAATTT  433

Query  361   ATCACATTATGGTCCATCAGTAGATTTAGCTATTTTTTCTCTTCACCTTGCTGGTGCTTC  420
             |||||| ||||| ||||||||||||||||||||||| |||||||| ||||||||||||||
Sbjct  434   ATCACACTATGGACCATCAGTAGATTTAGCTATTTTCTCTCTTCATCTTGCTGGTGCTTC  493

Query  421   TTCAATTTTAGGTGCAATTAACTTCATCACAACTATTGTAAATATACGATCAATTGGAAT  480
             |||||||||||||||||| || || || || |||||| ||||||||||||| ||||||||
Sbjct  494   TTCAATTTTAGGTGCAATCAATTTTATTACGACTATTATAAATATACGATCTATTGGAAT  553

Query  481   AACAATAGAACGAATACCATTATTTGTTTGATCTGTTTTAATTACAGCTATTTTACTACT  540
             ||||||||||||||| ||| ||||||||||||||||||||||||| ||||||||  ||||
Sbjct  554   AACAATAGAACGAATGCCACTATTTGTTTGATCTGTTTTAATTACTGCTATTTTGTTACT  613

Query  541   TCTATCTTTACCTGTATTAGCAGGTGCCATTACAATATTATTAACTGATCGAAACTTTAA  600
             | ||||||| ||||| |||||||| ||||||||||||||||||||||||||||| || ||
Sbjct  614   TTTATCTTTGCCTGTTTTAGCAGGAGCCATTACAATATTATTAACTGATCGAAATTTCAA  673

Query  601   CACATCATTTTTTGATCCTTCAGGAGGAGGAGATCCCATTTTATATCAACACTTATTT  658
             ||| ||||||||||||||||||||||| || ||||| |||||||| ||||| ||||||
Sbjct  674   CACTTCATTTTTTGATCCTTCAGGAGGGGGTGATCCAATTTTATACCAACATTTATTT  731
```

>Rhipicephalus turanicus isolate Xinjiang-BC cytochrome oxidase subunit 1 (COI) gene, partial cds; mitochondrial
Sequence ID: MF002579.1 Length: 889
Range 1: 74 to 731

Score:850 bits(460), Expect:0.0,
Identities:592/658(90%), Gaps:0/658(0%), Strand: Plus/Plus

```
Query  1     AACAATATATTTAATTTTTGGAGCATGATCAGGTATATTAGGACTTAGTATAAGAATATT  60
             ||||||||||||||||||||| ||||||||||| ||||||||||| || |||||||||||
Sbjct  74    AACAATATATTTAATTTTTGGGGCATGATCAGGAATATTAGGACTAAGAATAAGAATATT  133

Query  61    AATTCGTATAGAATTAGGACAACCTGGAACTTTAATTGGAAATGATCAGATTTATAATGT  120
             |||||| ||||||||||| ||||||||||||||||||||||||||||| |||||||||||
Sbjct  134   AATTCGAATAGAATTAGGCCAACCTGGAACTTTAATTGGAAATGATCAAATTTATAATGT  193
```

```
Query  121  AATCGTAACAGCACATGCATTTATTATAATTTTCTTTATAGTTATACCAATCATAATCGG  180
            ||| |||||||| |||||||||||||||||||| || ||||| |||||||| ||||| ||
Sbjct  194  AATTGTAACAGCCCATGCATTTATTATAATTTTTTTCATAGTAATACCAATTATAATTGG  253

Query  181  AGGGTTCGGAAATTGATTAGTTCCTATTATATTAGGTGCCCCAGATATAGCATTTCCACG  240
             || || ||||| |||||||| |||||||||||||| || ||||||||||||||||||||
Sbjct  254  TGGATTTGGAAACTGATTAGTACCTATTATATTAGGAGCTCCAGATATAGCATTTCCACG  313

Query  241  AATAAATAATATAAGATTTTGACTTTTACCCCCCTCATTATTTTTATTGATTAACTCTTC  300
            ||||||||| |||||||||||| |  | ||||| ||||||||| |||| ||||| || ||
Sbjct  314  AATAAATAACATAAGATTTTGATTACTTCCCCCTTCATTATTTCTATTAATTAATTCCTC  373

Query  301  ATTAATTGAATCCGGAGCTGGAACAGGATGAACAGTTTATCCCCCTCTATCTTCAAATTT  360
            |||||||||||| ||||| ||||| |||||||| |||||||| ||| |||| ||||||||
Sbjct  374  ATTAATTGAATCAGGAGCAGGAACTGGATGAACTGTTTATCCGCCTTTATCATCAAATTT  433

Query  361  ATCACATTATGGTCCATCAGTAGATTTAGCTATTTTTTCTCTTCACCTTGCTGGTGCTTC  420
            |||||| ||||| ||||||||||||||||||||||| |||||||| ||||||||||||||
Sbjct  434  ATCACACTATGGACCATCAGTAGATTTAGCTATTTTCTCTCTTCATCTTGCTGGTGCTTC  493

Query  421  TTCAATTTTAGGTGCAATTAACTTCATCACAACTATTGTAAATATACGATCAATTGGAAT  480
            |||||||||||||||||| || || || || |||||| ||||||||||||| ||||||||
Sbjct  494  TTCAATTTTAGGTGCAATCAATTTTATTACGACTATTATAAATATACGATCTATTGGAAT  553

Query  481  AACAATAGAACGAATACCATTATTTGTTTGATCTGTTTTAATTACAGCTATTTTACTACT  540
            ||||||||||||||| ||| ||||||||||||||||||||||||| ||||||||  ||||
Sbjct  554  AACAATAGAACGAATGCCACTATTTGTTTGATCTGTTTTAATTACTGCTATTTTGTTACT  613

Query  541  TCTATCTTTACCTGTATTAGCAGGTGCCATTACAATATTATTAACTGATCGAAACTTTAA  600
            | ||||||| ||||| |||||||| ||||||||||||||||||||||||||||| || ||
Sbjct  614  TTTATCTTTGCCTGTTTTAGCAGGAGCCATTACAATATTATTAACTGATCGAAATTTCAA  673

Query  601  CACATCATTTTTTGATCCTTCAGGAGGAGGAGATCCCATTTTATATCAACACTTATTT  658
            ||| ||||||||||||||||||||||| || ||||| |||||||| ||||| ||||||
Sbjct  674  CACTTCATTTTTTGATCCTTCAGGAGGGGGTGATCCAATTTTATACCAACATTTATTT  731
```

>Rhipicephalus turanicus isolate Xinjiang-YC cytochrome oxidase subunit 1 (COI) gene, partial cds; mitochondrial
Sequence ID: MF002578.1 Length: 889
Range 1: 74 to 731

Score:850 bits(460), Expect:0.0,
Identities:592/658(90%), Gaps:0/658(0%), Strand: Plus/Plus

```
Query  1    AACAATATATTTAATTTTTGGAGCATGATCAGGTATATTAGGACTTAGTATAAGAATATT  60
            ||||||||||||||||||||| ||||||||||| ||||||||||| || |||||||||||
Sbjct  74   AACAATATATTTAATTTTTGGGGCATGATCAGGAATATTAGGACTAAGAATAAGAATATT  133

Query  61   AATTCGTATAGAATTAGGACAACCTGGAACTTTAATTGGAAATGATCAGATTTATAATGT  120
            |||||| ||||||||||| ||||||||||||||||||||||||||||| |||||||||||
```

```
Sbjct  134  AATTCGAATAGAATTAGGCCAACCTGGAACTTTAATTGGAAATGATCAAATTTATAATGT  193

Query  121  AATCGTAACAGCACATGCATTTATTATAATTTTCTTTATAGTTATACCAATCATAATCGG  180
            ||| |||||||| |||||||||||||||||||| || ||||| |||||||| ||||| ||
Sbjct  194  AATTGTAACAGCCCATGCATTTATTATAATTTTTTTCATAGTAATACCAATTATAATTGG  253

Query  181  AGGGTTCGGAAATTGATTAGTTCCTATTATATTAGGTGCCCCAGATATAGCATTTCCACG  240
             || || ||||| |||||||| |||||||||||||| || ||||||||||||||||||||
Sbjct  254  TGGATTTGGAAACTGATTAGTACCTATTATATTAGGAGCTCCAGATATAGCATTTCCACG  313

Query  241  AATAAATAATATAAGATTTTGACTTTTACCCCCCTCATTATTTTTATTGATTAACTCTTC  300
            ||||||||| |||||||||||| |  | ||||| ||||||||| |||| ||||| || ||
Sbjct  314  AATAAATAACATAAGATTTTGATTACTTCCCCCTTCATTATTTCTATTAATTAATTCCTC  373

Query  301  ATTAATTGAATCCGGAGCTGGAACAGGATGAACAGTTTATCCCCCTCTATCTTCAAATTT  360
            |||||||||||| ||||| ||||| |||||||| |||||||| ||| |||| ||||||||
Sbjct  374  ATTAATTGAATCAGGAGCAGGAACTGGATGAACTGTTTATCCGCCTTTATCATCAAATTT  433

Query  361  ATCACATTATGGTCCATCAGTAGATTTAGCTATTTTTTCTCTTCACCTTGCTGGTGCTTC  420
            |||||| ||||| ||||||||||||||||||||||| |||||||| ||||||||||||||
Sbjct  434  ATCACACTATGGACCATCAGTAGATTTAGCTATTTTCTCTCTTCATCTTGCTGGTGCTTC  493

Query  421  TTCAATTTTAGGTGCAATTAACTTCATCACAACTATTGTAAATATACGATCAATTGGAAT  480
            |||||||||||||||||| || || || || |||||| ||||||||||||| ||||||||
Sbjct  494  TTCAATTTTAGGTGCAATCAATTTTATTACGACTATTATAAATATACGATCTATTGGAAT  553

Query  481  AACAATAGAACGAATACCATTATTTGTTTGATCTGTTTTAATTACAGCTATTTTACTACT  540
            ||||||||||||||| ||| ||||||||||||||||||||||||| ||||||||  ||||
Sbjct  554  AACAATAGAACGAATGCCACTATTTGTTTGATCTGTTTTAATTACTGCTATTTTGTTACT  613

Query  541  TCTATCTTTACCTGTATTAGCAGGTGCCATTACAATATTATTAACTGATCGAAACTTTAA  600
            | ||||||| ||||| |||||||| ||||||||||||||||||||||||||||| || ||
Sbjct  614  TTTATCTTTGCCTGTTTTAGCAGGAGCCATTACAATATTATTAACTGATCGAAATTTCAA  673

Query  601  CACATCATTTTTTGATCCTTCAGGAGGAGGAGATCCCATTTTATATCAACACTTATTT  658
            ||| ||||||||||||||||||||||| || ||||| |||||||| ||||| ||||||
Sbjct  674  CACTTCATTTTTTGATCCTTCAGGAGGGGGTGATCCAATTTTATACCAACATTTATTT  731
```

>Rhipicephalus turanicus isolate Xinjiang-PS cytochrome oxidase subunit 1 (COI) gene, partial cds; mitochondrial
Sequence ID: MF002577.1 Length: 889
Range 1: 74 to 731

Score:850 bits(460), Expect:0.0,
Identities:592/658(90%), Gaps:0/658(0%), Strand: Plus/Plus

```
Query  1    AACAATATATTTAATTTTTGGAGCATGATCAGGTATATTAGGACTTAGTATAAGAATATT  60
            ||||||||||||||||||||| ||||||||||| ||||||||||| || |||||||||||
Sbjct  74   AACAATATATTTAATTTTTGGGGCATGATCAGGAATATTAGGACTAAGAATAAGAATATT  133

Query  61   AATTCGTATAGAATTAGGACAACCTGGAACTTTAATTGGAAATGATCAGATTTATAATGT  120
```

```
             |||||| ||||||||||| ||||||||||||||||||||||||||||| |||||||||||
Sbjct  134   AATTCGAATAGAATTAGGCCAACCTGGAACTTTAATTGGAAATGATCAAATTTATAATGT  193

Query  121   AATCGTAACAGCACATGCATTTATTATAATTTTCTTTATAGTTATACCAATCATAATCGG  180
             ||| |||||||| |||||||||||||||||||| || ||||| |||||||| ||||| ||
Sbjct  194   AATTGTAACAGCCCATGCATTTATTATAATTTTTTTCATAGTAATACCAATTATAATTGG  253

Query  181   AGGGTTCGGAAATTGATTAGTTCCTATTATATTAGGTGCCCCAGATATAGCATTTCCACG  240
              || || ||||| |||||||| |||||||||||||| || ||||||||||||||||||||
Sbjct  254   TGGATTTGGAAACTGATTAGTACCTATTATATTAGGAGCTCCAGATATAGCATTTCCACG  313

Query  241   AATAAATAATATAAGATTTTGACTTTTACCCCCCTCATTATTTTTATTGATTAACTCTTC  300
             ||||||||| |||||||||||| |  | ||||| ||||||||| |||| ||||| || ||
Sbjct  314   AATAAATAACATAAGATTTTGATTACTTCCCCCTTCATTATTTCTATTAATTAATTCCTC  373

Query  301   ATTAATTGAATCCGGAGCTGGAACAGGATGAACAGTTTATCCCCCTCTATCTTCAAATTT  360
             |||||||||||| ||||| ||||| |||||||| |||||||| ||| |||| ||||||||
Sbjct  374   ATTAATTGAATCAGGAGCAGGAACTGGATGAACTGTTTATCCGCCTTTATCATCAAATTT  433

Query  361   ATCACATTATGGTCCATCAGTAGATTTAGCTATTTTTTCTCTTCACCTTGCTGGTGCTTC  420
             |||||| ||||| ||||||||||||||||||||||| |||||||| ||||||||||||||
Sbjct  434   ATCACACTATGGACCATCAGTAGATTTAGCTATTTTCTCTCTTCATCTTGCTGGTGCTTC  493

Query  421   TTCAATTTTAGGTGCAATTAACTTCATCACAACTATTGTAAATATACGATCAATTGGAAT  480
             |||||||||||||||||| || || || || |||||| ||||||||||||| ||||||||
Sbjct  494   TTCAATTTTAGGTGCAATCAATTTTATTACGACTATTATAAATATACGATCTATTGGAAT  553

Query  481   AACAATAGAACGAATACCATTATTTGTTTGATCTGTTTTAATTACAGCTATTTTACTACT  540
             ||||||||||||||| ||| ||||||||||||||||||||||||| ||||||||  ||||
Sbjct  554   AACAATAGAACGAATGCCACTATTTGTTTGATCTGTTTTAATTACTGCTATTTTGTTACT  613

Query  541   TCTATCTTTACCTGTATTAGCAGGTGCCATTACAATATTATTAACTGATCGAAACTTTAA  600
             | ||||||| ||||| |||||||| ||||||||||||||||||||||||||||| || ||
Sbjct  614   TTTATCTTTGCCTGTTTTAGCAGGAGCCATTACAATATTATTAACTGATCGAAATTTCAA  673

Query  601   CACATCATTTTTTGATCCTTCAGGAGGAGGAGATCCCATTTTATATCAACACTTATTT  658
             ||| ||||||||||||||||||||||| || ||||| |||||||| ||||| ||||||
Sbjct  674   CACTTCATTTTTTGATCCTTCAGGAGGGGTGATCCAATTTTATACCAACATTTATTT  731
```

>Rhipicephalus turanicus isolate SHZ cytochrome oxidase subunit I (CO1) gene, partial cds; mitochondrial
Sequence ID: KY069271.1 Length: 889
Range 1: 74 to 731

Score:850 bits(460), Expect:0.0,
Identities:592/658(90%), Gaps:0/658(0%), Strand: Plus/Plus

```
Query  1     AACAATATATTTAATTTTTGGAGCATGATCAGGTATATTAGGACTTAGTATAAGAATATT  60
             ||||||||||||||||||||| ||||||||||| ||||||||||| || |||||||||||
Sbjct  74    AACAATATATTTAATTTTTGGGGCATGATCAGGAATATTAGGACTAAGAATAAGAATATT  133
```

```
Query  61    AATTCGTATAGAATTAGGACAACCTGGAACTTTAATTGGAAATGATCAGATTTATAATGT  120
             |||||| ||||||||||| ||||||||||||||||||||||||||||| |||||||||||
Sbjct  134   AATTCGAATAGAATTAGGCCAACCTGGAACTTTAATTGGAAATGATCAAATTTATAATGT  193

Query  121   AATCGTAACAGCACATGCATTTATTATAATTTTCTTTATAGTTATACCAATCATAATCGG  180
             ||| |||||||| |||||||||||||||||||| || ||||| |||||||| ||||| ||
Sbjct  194   AATTGTAACAGCCCATGCATTTATTATAATTTTTTTCATAGTAATACCAATTATAATTGG  253

Query  181   AGGGTTCGGAAATTGATTAGTTCCTATTATATTAGGTGCCCCAGATATAGCATTTCCACG  240
              || || ||||| |||||||| |||||||||||||| || ||||||||||||||||||||
Sbjct  254   TGGATTTGGAAACTGATTAGTACCTATTATATTAGGAGCTCCAGATATAGCATTTCCACG  313

Query  241   AATAAATAATATAAGATTTTGACTTTTACCCCCCTCATTATTTTTATTGATTAACTCTTC  300
             ||||||||| |||||||||||| |  | ||||| ||||||||| |||| ||||| || ||
Sbjct  314   AATAAATAACATAAGATTTTGATTACTTCCCCCTTCATTATTTCTATTAATTAATTCCTC  373

Query  301   ATTAATTGAATCCGGAGCTGGAACAGGATGAACAGTTTATCCCCCTCTATCTTCAAATTT  360
             |||||||||||| ||||| ||||| |||||||| |||||||| ||| |||| ||||||||
Sbjct  374   ATTAATTGAATCAGGAGCAGGAACTGGATGAACTGTTTATCCGCCTTTATCATCAAATTT  433

Query  361   ATCACATTATGGTCCATCAGTAGATTTAGCTATTTTTTCTCTTCACCTTGCTGGTGCTTC  420
             |||||| ||||| ||||||||||||||||||||||| |||||||| ||||||||||||||
Sbjct  434   ATCACACTATGGACCATCAGTAGATTTAGCTATTTTCTCTCTTCATCTTGCTGGTGCTTC  493

Query  421   TTCAATTTTAGGTGCAATTAACTTCATCACAACTATTGTAAATATACGATCAATTGGAAT  480
             |||||||||||||||||| || || || || |||||| ||||||||||||| ||||||||
Sbjct  494   TTCAATTTTAGGTGCAATCAATTTTATTACGACTATTATAAATATACGATCTATTGGAAT  553

Query  481   AACAATAGAACGAATACCATTATTTGTTTGATCTGTTTTAATTACAGCTATTTTACTACT  540
             ||||||||||||||| ||| ||||||||||||||||||||||||| ||||||||  ||||
Sbjct  554   AACAATAGAACGAATGCCACTATTTGTTTGATCTGTTTTAATTACTGCTATTTTGTTACT  613

Query  541   TCTATCTTTACCTGTATTAGCAGGTGCCATTACAATATTATTAACTGATCGAAACTTTAA  600
             | ||||||| ||||| |||||||| ||||||||||||||||||||||||||||| || ||
Sbjct  614   TTTATCTTTGCCTGTTTTAGCAGGAGCCATTACAATATTATTAACTGATCGAAATTTCAA  673

Query  601   CACATCATTTTTTGATCCTTCAGGAGGAGGAGATCCCATTTTATATCAACACTTATTT  658
             ||| ||||||||||||||||||||||| || ||||| |||||||| ||||| ||||||
Sbjct  674   CACTTCATTTTTTGATCCTTCAGGAGGGGGTGATCCAATTTTATACCAACATTTATTT  731
```

>Rhipicephalus turanicus isolate FK-1 cytochrome oxidase subunit 1 (COI) gene, partial cds; mitochondrial
Sequence ID: KY606291.1 Length: 889
Range 1: 74 to 731

Score:850 bits(460), Expect:0.0,
Identities:592/658(90%), Gaps:0/658(0%), Strand: Plus/Plus

```
Query  1     AACAATATATTTAATTTTTGGAGCATGATCAGGTATATTAGGACTTAGTATAAGAATATT  60
             ||||||||||||||||||||| ||||||||||| ||||||||||| || |||||||||||
Sbjct  74    AACAATATATTTAATTTTTGGGGCATGATCAGGAATATTAGGACTAAGAATAAGAATATT  133
```

```
Query  61    AATTCGTATAGAATTAGGACAACCTGGAACTTTAATTGGAAATGATCAGATTTATAATGT  120
             |||||| ||||||||||| ||||||||||||||||||||||||||||| |||||||||||
Sbjct  134   AATTCGAATAGAATTAGGCCAACCTGGAACTTTAATTGGAAATGATCAAATTTATAATGT  193

Query  121   AATCGTAACAGCACATGCATTTATTATAATTTTCTTTATAGTTATACCAATCATAATCGG  180
             ||| |||||||| |||||||||||||||||||| || ||||| |||||||| ||||| ||
Sbjct  194   AATTGTAACAGCCCATGCATTTATTATAATTTTTTTCATAGTAATACCAATTATAATTGG  253

Query  181   AGGGTTCGGAAATTGATTAGTTCCTATTATATTAGGTGCCCCAGATATAGCATTTCCACG  240
              || || ||||| |||||||| |||||||||||||| || ||||||||||||||||||||
Sbjct  254   TGGATTTGGAAACTGATTAGTACCTATTATATTAGGAGCTCCAGATATAGCATTTCCACG  313

Query  241   AATAAATAATATAAGATTTTGACTTTTACCCCCCTCATTATTTTTATTGATTAACTCTTC  300
             ||||||||| |||||||||||| |  | ||||| ||||||||| |||| ||||| || ||
Sbjct  314   AATAAATAACATAAGATTTTGATTACTTCCCCCTTCATTATTTCTATTAATTAATTCCTC  373

Query  301   ATTAATTGAATCCGGAGCTGGAACAGGATGAACAGTTTATCCCCCTCTATCTTCAAATTT  360
             |||||||||||| ||||| ||||| |||||||| |||||||| ||| |||| ||||||||
Sbjct  374   ATTAATTGAATCAGGAGCAGGAACTGGATGAACTGTTTATCCGCCTTTATCATCAAATTT  433

Query  361   ATCACATTATGGTCCATCAGTAGATTTAGCTATTTTTTCTCTTCACCTTGCTGGTGCTTC  420
             |||||| ||||| ||||||||||||||||||||||| |||||||| ||||||||||||||
Sbjct  434   ATCACACTATGGACCATCAGTAGATTTAGCTATTTTCTCTCTTCATCTTGCTGGTGCTTC  493

Query  421   TTCAATTTTAGGTGCAATTAACTTCATCACAACTATTGTAAATATACGATCAATTGGAAT  480
             |||||||||||||||||| || || || || |||||| ||||||||||||| ||||||||
Sbjct  494   TTCAATTTTAGGTGCAATCAATTTTATTACGACTATTATAAATATACGATCTATTGGAAT  553

Query  481   AACAATAGAACGAATACCATTATTTGTTTGATCTGTTTTAATTACAGCTATTTTACTACT  540
             ||||||||||||||| ||| ||||||||||||||||||||||||| ||||||||  ||||
Sbjct  554   AACAATAGAACGAATGCCACTATTTGTTTGATCTGTTTTAATTACTGCTATTTTGTTACT  613

Query  541   TCTATCTTTACCTGTATTAGCAGGTGCCATTACAATATTATTAACTGATCGAAACTTTAA  600
             | ||||||| ||||| |||||||| ||||||||||||||||||||||||||||| || ||
Sbjct  614   TTTATCTTTGCCTGTTTTAGCAGGAGCCATTACAATATTATTAACTGATCGAAATTTCAA  673

Query  601   CACATCATTTTTTGATCCTTCAGGAGGAGGAGATCCCATTTTATATCAACACTTATTT  658
             ||| ||||||||||||||||||||||| || ||||| |||||||| ||||| ||||||
Sbjct  674   CACTTCATTTTTTGATCCTTCAGGAGGGGGTGATCCAATTTTATACCAACATTTATTT  731
```

>Rhipicephalus turanicus isolate YN3 cytochrome oxidase subunit 1 (COI) gene, partial cds; mitochondrial
Sequence ID: KY606290.1 Length: 889
Range 1: 74 to 731

Score:850 bits(460), Expect:0.0,
Identities:592/658(90%), Gaps:0/658(0%), Strand: Plus/Plus

```
Query  1     AACAATATATTTAATTTTTGGAGCATGATCAGGTATATTAGGACTTAGTATAAGAATATT  60
             ||||||||||||||||||||| ||||||||||| ||||||||||| || |||||||||||
```

```
Sbjct  74    AACAATATATTTAATTTTTGGGGCATGATCAGGAATATTAGGACTAAGAATAAGAATATT  133

Query  61    AATTCGTATAGAATTAGGACAACCTGGAACTTTAATTGGAAATGATCAGATTTATAATGT  120
             |||||| ||||||||||| ||||||||||||||||||||||||||||| |||||||||||
Sbjct  134   AATTCGAATAGAATTAGGCCAACCTGGAACTTTAATTGGAAATGATCAAATTTATAATGT  193

Query  121   AATCGTAACAGCACATGCATTTATTATAATTTTCTTTATAGTTATACCAATCATAATCGG  180
             ||| |||||||| |||||||||||||||||||| || ||||| |||||||| ||||| ||
Sbjct  194   AATTGTAACAGCCCATGCATTTATTATAATTTTTTTCATAGTAATACCAATTATAATTGG  253

Query  181   AGGGTTCGGAAATTGATTAGTTCCTATTATATTAGGTGCCCCAGATATAGCATTTCCACG  240
              || || ||||| |||||||| |||||||||||||| || ||||||||||||||||||||
Sbjct  254   TGGATTTGGAAACTGATTAGTACCTATTATATTAGGAGCTCCAGATATAGCATTTCCACG  313

Query  241   AATAAATAATATAAGATTTTGACTTTTACCCCCCTCATTATTTTTATTGATTAACTCTTC  300
             ||||||||| |||||||||||| |  | ||||| ||||||||| |||| ||||| || ||
Sbjct  314   AATAAATAACATAAGATTTTGATTACTTCCCCCTTCATTATTTCTATTAATTAATTCCTC  373

Query  301   ATTAATTGAATCCGGAGCTGGAACAGGATGAACAGTTTATCCCCCTCTATCTTCAAATTT  360
             |||||||||||| ||||| ||||| |||||||| |||||||| ||| |||| ||||||||
Sbjct  374   ATTAATTGAATCAGGAGCAGGAACTGGATGAACTGTTTATCCGCCTTTATCATCAAATTT  433

Query  361   ATCACATTATGGTCCATCAGTAGATTTAGCTATTTTTTCTCTTCACCTTGCTGGTGCTTC  420
             |||||| ||||| ||||||||||||||||||||||| |||||||| ||||||||||||||
Sbjct  434   ATCACACTATGGACCATCAGTAGATTTAGCTATTTTCTCTCTTCATCTTGCTGGTGCTTC  493

Query  421   TTCAATTTTAGGTGCAATTAACTTCATCACAACTATTGTAAATATACGATCAATTGGAAT  480
             |||||||||||||||||| || || || || |||||| ||||||||||||| ||||||||
Sbjct  494   TTCAATTTTAGGTGCAATCAATTTTATTACGACTATTATAAATATACGATCTATTGGAAT  553

Query  481   AACAATAGAACGAATACCATTATTTGTTTGATCTGTTTTAATTACAGCTATTTTACTACT  540
             ||||||||||||||| ||| ||||||||||||||||||||||||| ||||||||  ||||
Sbjct  554   AACAATAGAACGAATGCCACTATTTGTTTGATCTGTTTTAATTACTGCTATTTTGTTACT  613

Query  541   TCTATCTTTACCTGTATTAGCAGGTGCCATTACAATATTATTAACTGATCGAAACTTTAA  600
             | ||||||| ||||| |||||||| ||||||||||||||||||||||||||||| || ||
Sbjct  614   TTTATCTTTGCCTGTTTTAGCAGGAGCCATTACAATATTATTAACTGATCGAAATTTCAA  673

Query  601   CACATCATTTTTTGATCCTTCAGGAGGAGGAGATCCCATTTTATATCAACACTTATTT  658
             ||| ||||||||||||||||||||||| || ||||| |||||||| ||||| ||||||
Sbjct  674   CACTTCATTTTTTGATCCTTCAGGAGGGGGTGATCCAATTTTATACCAACATTTATTT  731
```

>Rhipicephalus turanicus isolate YN2 cytochrome oxidase subunit 1 (COI) gene, partial cds; mitochondrial
Sequence ID: KY606289.1 Length: 889
Range 1: 74 to 731

Score:850 bits(460), Expect:0.0,
Identities:592/658(90%), Gaps:0/658(0%), Strand: Plus/Plus

```
Query  1     AACAATATATTTAATTTTTGGAGCATGATCAGGTATATTAGGACTTAGTATAAGAATATT  60
```

```
            |||||||||||||||||||| ||||||||||| ||||||||||| || ||||||||||||
Sbjct  74   AACAATATATTTAATTTTTGGGGCATGATCAGGAATATTAGGACTAAGAATAAGAATATT  133

Query  61   AATTCGTATAGAATTAGGACAACCTGGAACTTTAATTGGAAATGATCAGATTTATAATGT  120
            |||||| ||||||||||| ||||||||||||||||||||||||||||| |||||||||||
Sbjct  134  AATTCGAATAGAATTAGGCCAACCTGGAACTTTAATTGGAAATGATCAAATTTATAATGT  193

Query  121  AATCGTAACAGCACATGCATTTATTATAATTTTCTTTATAGTTATACCAATCATAATCGG  180
            ||| |||||||| |||||||||||||||||||| || ||||| |||||||| ||||| ||
Sbjct  194  AATTGTAACAGCCCATGCATTTATTATAATTTTTTTCATAGTAATACCAATTATAATTGG  253

Query  181  AGGGTTCGGAAATTGATTAGTTCCTATTATATTAGGTGCCCCAGATATAGCATTTCCACG  240
             || || ||||| |||||||| |||||||||||||| || ||||||||||||||||||||
Sbjct  254  TGGATTTGGAAACTGATTAGTACCTATTATATTAGGAGCTCCAGATATAGCATTTCCACG  313

Query  241  AATAAATAATATAAGATTTTGACTTTTACCCCCCTCATTATTTTTATTGATTAACTCTTC  300
            ||||||||| |||||||||||| |  | ||||| ||||||||| |||| ||||| || ||
Sbjct  314  AATAAATAACATAAGATTTTGATTACTTCCCCCTTCATTATTTCTATTAATTAATTCCTC  373

Query  301  ATTAATTGAATCCGGAGCTGGAACAGGATGAACAGTTTATCCCCCTCTATCTTCAAATTT  360
            |||||||||||| ||||| ||||| |||||||| |||||||| ||| |||| ||||||||
Sbjct  374  ATTAATTGAATCAGGAGCAGGAACTGGATGAACTGTTTATCCGCCTTTATCATCAAATTT  433

Query  361  ATCACATTATGGTCCATCAGTAGATTTAGCTATTTTTTCTCTTCACCTTGCTGGTGCTTC  420
            |||||| ||||| ||||||||||||||||||||||| |||||||| ||||||||||||||
Sbjct  434  ATCACACTATGGACCATCAGTAGATTTAGCTATTTTCTCTCTTCATCTTGCTGGTGCTTC  493

Query  421  TTCAATTTTAGGTGCAATTAACTTCATCACAACTATTGTAAATATACGATCAATTGGAAT  480
            |||||||||||||||||| || || || || |||||| ||||||||||||| ||||||||
Sbjct  494  TTCAATTTTAGGTGCAATCAATTTTATTACGACTATTATAAATATACGATCTATTGGAAT  553

Query  481  AACAATAGAACGAATACCATTATTTGTTTGATCTGTTTTAATTACAGCTATTTTACTACT  540
            ||||||||||||||| ||| ||||||||||||||||||||||||| ||||||||  ||||
Sbjct  554  AACAATAGAACGAATGCCACTATTTGTTTGATCTGTTTTAATTACTGCTATTTTGTTACT  613

Query  541  TCTATCTTTACCTGTATTAGCAGGTGCCATTACAATATTATTAACTGATCGAAACTTTAA  600
            | ||||||| ||||| |||||||| ||||||||||||||||||||||||||||| || ||
Sbjct  614  TTTATCTTTGCCTGTTTTAGCAGGAGCCATTACAATATTATTAACTGATCGAAATTTCAA  673

Query  601  CACATCATTTTTTGATCCTTCAGGAGGAGGAGATCCCATTTTATATCAACACTTATTT  658
            ||| ||||||||||||||||||||||| || ||||| |||||||| ||||| ||||||
Sbjct  674  CACTTCATTTTTTGATCCTTCAGGAGGGGGTGATCCAATTTTATACCAACATTTATTT  731
```

Query #4: TKCJJ093-19|TKCJ012018H09|Rhipicephalus sanguineus|COI-5P Query ID: lcl|Query_26766 Length: 658

Sequences producing significant alignments:

```
                                                                  Scientific
 Common                       Max    Total Query   E   Per.   Acc.
```

```
Description                                                         Name
 Name            Taxid      Score  Score cover Value Ident  Len        Accession

Rhipicephalus sanguineus mitochondrial DNA, complete genome
Rhipicephalu... brown dog tick  34632      1216   1216  100%  0.0   100.00 14710
     AF081829.1
Rhipicephalus sanguineus isolate Zagreb-B cytochrome oxidase...
Rhipicephalu... brown dog tick  34632      1140   1140  94%   0.0   99.84  629
     KX757893.1
Rhipicephalus sanguineus isolate Italy-A cytochrome oxidase...
Rhipicephalu... brown dog tick  34632      1129   1129  94%   0.0   99.52  630
     KX757903.1
Rhipicephalus sanguineus isolate Al-Hosema-A cytochrome oxidas...
Rhipicephalu... brown dog tick  34632      1112   1112  94%   0.0   99.03  630
     KX757909.1
Rhipicephalus sanguineus sensu lato 'temperate lineage' isolat...
Rhipicephalu... brown dog tick  34632      1072   1072  100%  0.0   96.05  692
     MF426015.1
Rhipicephalus sanguineus sensu lato 'temperate lineage' isolat...
Rhipicephalu... brown dog tick  34632      1066   1066  100%  0.0   95.90  675
     MF426018.1
Rhipicephalus sanguineus sensu lato 'temperate lineage' isolat...
Rhipicephalu... brown dog tick  34632      1066   1066  100%  0.0   95.90  680
     MF426016.1
Rhipicephalus sanguineus sensu lato 'temperate lineage' isolat...
Rhipicephalu... brown dog tick  34632      1066   1066  100%  0.0   95.90  693
     MF426013.1
Rhipicephalus sanguineus sensu lato 'temperate lineage' isolat...
Rhipicephalu... brown dog tick  34632      1066   1066  100%  0.0   95.90  679
     MF426009.1
Rhipicephalus sanguineus sensu lato 'temperate lineage' isolat...
Rhipicephalu... brown dog tick  34632      1066   1066  100%  0.0   95.90  686
     MF426007.1
Rhipicephalus sanguineus sensu lato 'temperate lineage' isolat...
Rhipicephalu... brown dog tick  34632      1061   1061  100%  0.0   95.74  697
     MF426019.1
Rhipicephalus sanguineus sensu lato 'temperate lineage' isolat...
Rhipicephalu... brown dog tick  34632      1061   1061  100%  0.0   95.74  705
     MF426006.1
Rhipicephalus sanguineus haplotype III cytochrome oxidase...
Rhipicephalu... brown dog tick  34632      1061   1061  100%  0.0   95.74  713
     KU556745.1
Rhipicephalus sanguineus isolate Arizona-1 cytochrome c oxidas...
Rhipicephalu... brown dog tick  34632      1061   1061  100%  0.0   95.74  710
     MN593344.1
Rhipicephalus sanguineus isolate Arizona-2 cytochrome c oxidas...
Rhipicephalu... brown dog tick  34632      1061   1061  100%  0.0   95.74  710
     MN593343.1
Rhipicephalus sanguineus isolate 11N cytochrome oxidase subuni...
Rhipicephalu... brown dog tick  34632      1061   1061  100%  0.0   95.74  703
     MN585197.1
Rhipicephalus sanguineus cytochrome c oxidase I (COI) gene,...
Rhipicephalu... brown dog tick  34632      1061   1061  100%  0.0   95.74  793
     AF132839.1
```

Rhipicephalus sanguineus sensu lato 'temperate lineage' isolat...
Rhipicephalu... brown dog tick 34632 1055 1055 100% 0.0 95.59 680
MF426017.1
Rhipicephalus sanguineus sensu lato 'temperate lineage' isolat...
Rhipicephalu... brown dog tick 34632 1055 1055 100% 0.0 95.59 690
MF426008.1
Rhipicephalus sanguineus sensu lato 'temperate lineage' isolat...
Rhipicephalu... brown dog tick 34632 1055 1055 100% 0.0 95.59 684
MF426001.1
Rhipicephalus sanguineus sensu lato 'temperate lineage' isolat...
Rhipicephalu... brown dog tick 34632 1055 1055 100% 0.0 95.59 689
MF425998.1
Rhipicephalus sanguineus sensu lato 'temperate lineage' isolat...
Rhipicephalu... brown dog tick 34632 1055 1055 100% 0.0 95.59 695
MF425997.1
Rhipicephalus sanguineus haplotype IV cytochrome oxidase subun...
Rhipicephalu... brown dog tick 34632 1055 1113 100% 0.0 95.59 771
KU556746.1
Rhipicephalus sanguineus voucher CROBB813 cytochrome oxidase...
Rhipicephalu... brown dog tick 34632 1055 1055 100% 0.0 95.59 658
MZ305546.1
Rhipicephalus sanguineus voucher CROBB810 cytochrome oxidase...
Rhipicephalu... brown dog tick 34632 1055 1055 100% 0.0 95.59 658
MZ305545.1
Rhipicephalus sanguineus isolate 5MSK cytochrome c oxidase...
Rhipicephalu... brown dog tick 34632 1055 1055 100% 0.0 95.59 676
MW152145.1
Rhipicephalus sanguineus isolate 1MSK cytochrome c oxidase...
Rhipicephalu... brown dog tick 34632 1055 1055 100% 0.0 95.59 675
MW152144.1
Rhipicephalus sanguineus isolate RSN2SK cytochrome c oxidase...
Rhipicephalu... brown dog tick 34632 1055 1055 100% 0.0 95.59 676
MW152143.1
Rhipicephalus sanguineus isolate RSN11SK cytochrome c oxidase...
Rhipicephalu... brown dog tick 34632 1055 1055 100% 0.0 95.59 669
MW152142.1
Rhipicephalus sanguineus sensu lato 'temperate lineage' isolat...
Rhipicephalu... brown dog tick 34632 1050 1050 100% 0.0 95.44 684
MF426012.1
Rhipicephalus sanguineus haplotype I cytochrome oxidase subuni...
Rhipicephalu... brown dog tick 34632 1048 1048 98% 0.0 95.83 659
KU556743.1
Rhipicephalus sanguineus haplotype II cytochrome oxidase subun...
Rhipicephalu... brown dog tick 34632 1046 1046 100% 0.0 95.30 787
KU556744.1
Rhipicephalus sanguineus sensu lato 'temperate lineage' isolat...
Rhipicephalu... brown dog tick 34632 1038 1038 97% 0.0 95.80 812
MF426020.1
Rhipicephalus sanguineus sensu lato 'temperate lineage' isolat...
Rhipicephalu... brown dog tick 34632 1037 1037 98% 0.0 95.52 824
MF426010.1
Rhipicephalus sanguineus sensu lato 'temperate lineage' isolat...
Rhipicephalu... brown dog tick 34632 1031 1031 97% 0.0 95.64 809
MF426011.1

Rhipicephalus sanguineus sensu lato 'temperate lineage' isolat...
Rhipicephalu... brown dog tick 34632 1022 1022 94% 0.0 96.30 649
MF426014.1
Rhipicephalus sanguineus isolate LIC5424B cytochrome oxidase...
Rhipicephalu... brown dog tick 34632 1020 1020 97% 0.0 95.33 768
KX383818.1
Rhipicephalus sanguineus sensu lato 'temperate lineage' isolat...
Rhipicephalu... brown dog tick 34632 1016 1016 94% 0.0 96.14 635
MF426000.1
Rhipicephalus sanguineus sensu lato 'temperate lineage' isolat...
Rhipicephalu... brown dog tick 34632 1016 1016 97% 0.0 95.31 654
MF425988.1
Rhipicephalus sanguineus isolate LIC5533B cytochrome oxidase...
Rhipicephalu... brown dog tick 34632 1013 1013 96% 0.0 95.30 764
KX383811.1
Rhipicephalus sanguineus isolate LIC4736B cytochrome oxidase...
Rhipicephalu... brown dog tick 34632 1011 1011 96% 0.0 95.29 763
KX383807.1
Rhipicephalus sanguineus voucher Rs7 cytochrome oxidase subuni...
Rhipicephalu... brown dog tick 34632 1009 1009 95% 0.0 95.56 656
KU568516.1
Rhipicephalus sanguineus isolate LIC4736D cytochrome oxidase...
Rhipicephalu... brown dog tick 34632 1009 1009 96% 0.0 95.28 762
KX383809.1
Rhipicephalus sanguineus isolate LIC4727D cytochrome oxidase...
Rhipicephalu... brown dog tick 34632 1009 1009 96% 0.0 95.28 714
KX383806.1
Rhipicephalus sanguineus isolate LIC4727C cytochrome oxidase...
Rhipicephalu... brown dog tick 34632 1007 1007 96% 0.0 95.28 761
KX383805.1
Rhipicephalus sanguineus isolate Samont2 cytochrome oxidase...
Rhipicephalu... brown dog tick 34632 1005 1005 94% 0.0 95.82 644
MH630347.1
Rhipicephalus sanguineus isolate Samont1 cytochrome oxidase...
Rhipicephalu... brown dog tick 34632 1005 1005 94% 0.0 95.82 644
MH630346.1
Rhipicephalus sanguineus clone #7 cytochrome oxidase subunit I...
Rhipicephalu... brown dog tick 34632 1002 1002 95% 0.0 95.53 654
KX519712.1
Rhipicephalus sanguineus sensu lato 'temperate lineage' isolat...
Rhipicephalu... brown dog tick 34632 1000 1000 95% 0.0 95.38 639
MF426002.1
Rhipicephalus sanguineus isolate Rsan-ML4 cytochrome oxidase...
Rhipicephalu... brown dog tick 34632 990 990 94% 0.0 95.48 630
MG855658.1
Rhipicephalus sanguineus isolate Rsan-ML3 cytochrome oxidase...
Rhipicephalu... brown dog tick 34632 990 990 94% 0.0 95.48 630
MG855657.1
Rhipicephalus sanguineus isolate Rsan-ML2 cytochrome oxidase...
Rhipicephalu... brown dog tick 34632 990 990 94% 0.0 95.48 630
MG855656.1
Rhipicephalus sanguineus isolate Kehf-Lagareb-A cytochrome...
Rhipicephalu... brown dog tick 34632 990 990 94% 0.0 95.48 630
KX757910.1

Rhipicephalus sanguineus isolate Italy-B cytochrome oxidase...
Rhipicephalu... brown dog tick 34632 990 990 94% 0.0 95.48 630
KX757904.1
Rhipicephalus sanguineus isolate Malta-A cytochrome oxidase...
Rhipicephalu... brown dog tick 34632 990 990 94% 0.0 95.48 630
KX757902.1
Rhipicephalus sanguineus isolate Zagreb-C cytochrome oxidase...
Rhipicephalu... brown dog tick 34632 990 990 94% 0.0 95.48 630
KX757896.1
Rhipicephalus sanguineus isolate Pula-B cytochrome oxidase...
Rhipicephalu... brown dog tick 34632 990 990 94% 0.0 95.48 630
KX757889.1
Rhipicephalus sanguineus isolate Rovind cytochrome oxidase...
Rhipicephalu... brown dog tick 34632 990 990 94% 0.0 95.48 630
KX757887.1
Rhipicephalus sanguineus isolate Kajtasovo cytochrome oxidase...
Rhipicephalu... brown dog tick 34632 990 990 94% 0.0 95.48 630
KX757879.1
Rhipicephalus sanguineus clone #6 cytochrome oxidase subunit I...
Rhipicephalu... brown dog tick 34632 990 990 94% 0.0 95.48 636
KX519711.1
Rhipicephalus sanguineus isolate SV4 cytochrome c oxidase...
Rhipicephalu... brown dog tick 34632 990 990 94% 0.0 95.48 630
MW135448.1
Rhipicephalus sanguineus isolate Boka-A cytochrome oxidase...
Rhipicephalu... brown dog tick 34632 985 985 94% 0.0 95.32 630
KX757905.1
Rhipicephalus sanguineus isolate Sibenik cytochrome oxidase...
Rhipicephalu... brown dog tick 34632 985 985 94% 0.0 95.32 630
KX757895.1
Rhipicephalus sanguineus isolate Zadar cytochrome oxidase...
Rhipicephalu... brown dog tick 34632 985 985 94% 0.0 95.32 630
KX757892.1
Rhipicephalus sanguineus isolate Zagreb-A cytochrome oxidase...
Rhipicephalu... brown dog tick 34632 985 985 94% 0.0 95.32 630
KX757890.1
Rhipicephalus sanguineus isolate Pula-A cytochrome oxidase...
Rhipicephalu... brown dog tick 34632 985 985 94% 0.0 95.32 630
KX757888.1
Rhipicephalus sanguineus isolate Petnica cytochrome oxidase...
Rhipicephalu... brown dog tick 34632 985 985 94% 0.0 95.32 630
KX757883.1
Rhipicephalus sanguineus clone #3 cytochrome oxidase subunit I...
Rhipicephalu... brown dog tick 34632 985 985 93% 0.0 95.46 629
KX519708.1
Rhipicephalus sanguineus isolate SV2 cytochrome c oxidase...
Rhipicephalu... brown dog tick 34632 985 985 94% 0.0 95.32 630
MW135447.1
Rhipicephalus sanguineus clone #2 cytochrome oxidase subunit I...
Rhipicephalu... brown dog tick 34632 983 983 93% 0.0 95.45 626
KX519707.1
Rhipicephalus sanguineus isolate Szekszard cytochrome oxidase...
Rhipicephalu... brown dog tick 34632 979 979 94% 0.0 95.16 630
KX757901.1

Rhipicephalus sanguineus clone #4 cytochrome oxidase subunit I...
Rhipicephalu... brown dog tick 34632 974 974 94% 0.0 95.00 635
KX519709.1
Rhipicephalus sanguineus isolate LIC4723B cytochrome oxidase...
Rhipicephalu... brown dog tick 34632 937 937 90% 0.0 94.97 723
KX714599.1
Rhipicephalus sanguineus clone #8 cytochrome oxidase subunit I...
Rhipicephalu... brown dog tick 34632 933 933 88% 0.0 95.69 580
KX519713.1
Rhipicephalus sanguineus clone #1 cytochrome oxidase subunit I...
Rhipicephalu... brown dog tick 34632 898 898 86% 0.0 95.09 595
KX519706.1
Rhipicephalus sanguineus isolate Rsang2 cytochrome oxidase...
Rhipicephalu... brown dog tick 34632 883 883 100% 0.0 90.90 790
KY678135.1
Rhipicephalus turanicus isolate COX1-225-Goat cytochrome c...
Rhipicephalu... NA 34633 883 883 100% 0.0 90.88 673
MT800313.1
Rhipicephalus sanguineus sensu lato 'temperate lineage' isolat...
Rhipicephalu... brown dog tick 34632 881 881 85% 0.0 95.01 574
MF425987.1
Rhipicephalus sanguineus cytochrome oxidase subunit 1 (CO1)...
Rhipicephalu... brown dog tick 34632 876 876 98% 0.0 91.05 829
KU214592.1
Rhipicephalus turanicus isolate GY34-1 cytochrome c oxidase...
Rhipicephalu... NA 34633 872 872 99% 0.0 90.80 678
MT079206.1
Rhipicephalus sanguineus isolate GY34 cytochrome c oxidase...
Rhipicephalu... brown dog tick 34632 872 872 99% 0.0 90.80 678
MN862754.1
Rhipicephalus sanguineus isolate Gilan-e Gharb cytochrome...
Rhipicephalu... brown dog tick 34632 872 872 100% 0.0 90.58 1539
KM494916.1
Rhipicephalus sanguineus isolate Tehran cytochrome oxidase...
Rhipicephalu... brown dog tick 34632 872 872 100% 0.0 90.58 1539
KM494915.1
Rhipicephalus turanicus isolate COX1-18-Sheep cytochrome c...
Rhipicephalu... NA 34633 870 870 99% 0.0 90.56 673
MT800314.1
Rhipicephalus turanicus isolate 31X cytochrome oxidase subunit...
Rhipicephalu... NA 34633 861 861 100% 0.0 90.27 873
KY606303.1
Rhipicephalus turanicus isolate 19X cytochrome oxidase subunit...
Rhipicephalu... NA 34633 861 861 100% 0.0 90.27 873
KY606302.1
Rhipicephalus turanicus isolate 19C cytochrome oxidase subunit...
Rhipicephalu... NA 34633 861 861 100% 0.0 90.27 873
KY606301.1
Rhipicephalus turanicus isolate 12X cytochrome oxidase subunit...
Rhipicephalu... NA 34633 861 861 100% 0.0 90.27 873
KY606300.1
Rhipicephalus turanicus isolate 12C cytochrome oxidase subunit...
Rhipicephalu... NA 34633 861 861 100% 0.0 90.27 873
KY606299.1

```
Rhipicephalus camicasi isolate SC0126 mitochondrion, complete...
Rhipicephalu... NA              669981     861    861   100%  0.0   90.29  14725
     NC_061616.1
Rhipicephalus turanicus voucher CROBB808 cytochrome oxidase...
Rhipicephalu... NA              34633      861    861   100%  0.0   90.27  658
     MZ305547.1
Rhipicephalus turanicus isolate Xinjiang-WQIA cytochrome oxida...
Rhipicephalu... NA              34633      850    850   100%  0.0   89.97  889
     MF002581.1
Rhipicephalus turanicus isolate Xinjiang-WS cytochrome oxidase...
Rhipicephalu... NA              34633      850    850   100%  0.0   89.97  889
     MF002580.1
Rhipicephalus turanicus isolate Xinjiang-BC cytochrome oxidase...
Rhipicephalu... NA              34633      850    850   100%  0.0   89.97  889
     MF002579.1
Rhipicephalus turanicus isolate Xinjiang-YC cytochrome oxidase...
Rhipicephalu... NA              34633      850    850   100%  0.0   89.97  889
     MF002578.1
Rhipicephalus turanicus isolate Xinjiang-PS cytochrome oxidase...
Rhipicephalu... NA              34633      850    850   100%  0.0   89.97  889
     MF002577.1
Rhipicephalus turanicus isolate SHZ cytochrome oxidase subunit...
Rhipicephalu... NA              34633      850    850   100%  0.0   89.97  889
     KY069271.1
Rhipicephalus turanicus isolate FK-1 cytochrome oxidase subuni...
Rhipicephalu... NA              34633      850    850   100%  0.0   89.97  889
     KY606291.1
Rhipicephalus turanicus isolate YN3 cytochrome oxidase subunit...
Rhipicephalu... NA              34633      850    850   100%  0.0   89.97  889
     KY606290.1
Rhipicephalus turanicus isolate YN2 cytochrome oxidase subunit...
Rhipicephalu... NA              34633      850    850   100%  0.0   89.97  889
     KY606289.1


Alignments:

>Rhipicephalus sanguineus mitochondrial DNA, complete genome
Sequence ID: AF081829.1 Length: 14710
Range 1: 1191 to 1848

Score:1216 bits(658), Expect:0.0,
Identities:658/658(100%),  Gaps:0/658(0%), Strand: Plus/Plus

Query  1     AACAATATATTTAATTTTTGGAGCATGATCAGGTATATTAGGACTTAGTATAAGAATATT  60
             ||||||||||||||||||||||||||||||||||||||||||||||||||||||||||||
Sbjct  1191  AACAATATATTTAATTTTTGGAGCATGATCAGGTATATTAGGACTTAGTATAAGAATATT  1250

Query  61    AATTCGTATAGAATTAGGACAACCTGGAACTTTAATTGGAAATGATCAGATTTATAATGT  120
             ||||||||||||||||||||||||||||||||||||||||||||||||||||||||||||
Sbjct  1251  AATTCGTATAGAATTAGGACAACCTGGAACTTTAATTGGAAATGATCAGATTTATAATGT  1310

Query  121   AATCGTAACAGCACATGCATTTATTATAATTTTCTTTATAGTTATACCAATCATAATCGG  180
             ||||||||||||||||||||||||||||||||||||||||||||||||||||||||||||
```

```
Sbjct  1311  AATCGTAACAGCACATGCATTTATTATAATTTTCTTTATAGTTATACCAATCATAATCGG  1370

Query  181   AGGGTTCGGAAATTGATTAGTTCCTATTATATTAGGTGCCCCAGATATAGCATTTCCACG  240
             ||||||||||||||||||||||||||||||||||||||||||||||||||||||||||||
Sbjct  1371  AGGGTTCGGAAATTGATTAGTTCCTATTATATTAGGTGCCCCAGATATAGCATTTCCACG  1430

Query  241   AATAAATAATATAAGATTTTGACTTTTACCCCCCTCATTATTTTTATTGATTAACTCTTC  300
             ||||||||||||||||||||||||||||||||||||||||||||||||||||||||||||
Sbjct  1431  AATAAATAATATAAGATTTTGACTTTTACCCCCCTCATTATTTTTATTGATTAACTCTTC  1490

Query  301   ATTAATTGAATCCGGAGCTGGAACAGGATGAACAGTTTATCCCCCTCTATCTTCAAATTT  360
             ||||||||||||||||||||||||||||||||||||||||||||||||||||||||||||
Sbjct  1491  ATTAATTGAATCCGGAGCTGGAACAGGATGAACAGTTTATCCCCCTCTATCTTCAAATTT  1550

Query  361   ATCACATTATGGTCCATCAGTAGATTTAGCTATTTTTTCTCTTCACCTTGCTGGTGCTTC  420
             ||||||||||||||||||||||||||||||||||||||||||||||||||||||||||||
Sbjct  1551  ATCACATTATGGTCCATCAGTAGATTTAGCTATTTTTTCTCTTCACCTTGCTGGTGCTTC  1610

Query  421   TTCAATTTTAGGTGCAATTAACTTCATCACAACTATTGTAAATATACGATCAATTGGAAT  480
             ||||||||||||||||||||||||||||||||||||||||||||||||||||||||||||
Sbjct  1611  TTCAATTTTAGGTGCAATTAACTTCATCACAACTATTGTAAATATACGATCAATTGGAAT  1670

Query  481   AACAATAGAACGAATACCATTATTTGTTTGATCTGTTTTAATTACAGCTATTTTACTACT  540
             ||||||||||||||||||||||||||||||||||||||||||||||||||||||||||||
Sbjct  1671  AACAATAGAACGAATACCATTATTTGTTTGATCTGTTTTAATTACAGCTATTTTACTACT  1730

Query  541   TCTATCTTTACCTGTATTAGCAGGTGCCATTACAATATTATTAACTGATCGAAACTTTAA  600
             ||||||||||||||||||||||||||||||||||||||||||||||||||||||||||||
Sbjct  1731  TCTATCTTTACCTGTATTAGCAGGTGCCATTACAATATTATTAACTGATCGAAACTTTAA  1790

Query  601   CACATCATTTTTTGATCCTTCAGGAGGAGGAGATCCCATTTTATATCAACACTTATTT  658
             ||||||||||||||||||||||||||||||||||||||||||||||||||||||||||
Sbjct  1791  CACATCATTTTTTGATCCTTCAGGAGGAGGAGATCCCATTTTATATCAACACTTATTT  1848
```

```
>Rhipicephalus sanguineus isolate Zagreb-B cytochrome oxidase subunit I (COI)
gene, partial cds; mitochondrial
Sequence ID: KX757893.1 Length: 629
Range 1: 1 to 620

Score:1140 bits(617), Expect:0.0,
Identities:619/620(99%),  Gaps:0/620(0%), Strand: Plus/Plus

Query  39   TAGGACTTAGTATAAGAATATTAATTCGTATAGAATTAGGACAACCTGGAACTTTAATTG  98
            ||||||||||||||||||||||||||||||||||||||||||||||||||||||||||||
Sbjct  1    TAGGACTTAGTATAAGAATATTAATTCGTATAGAATTAGGACAACCTGGAACTTTAATTG  60

Query  99   GAAATGATCAGATTTATAATGTAATCGTAACAGCACATGCATTTATTATAATTTTCTTTA  158
            ||||||||||||||||||||||||||||||||||||||||||||||||||||||||||||
Sbjct  61   GAAATGATCAGATTTATAATGTAATCGTAACAGCACATGCATTTATTATAATTTTCTTTA  120

Query  159  TAGTTATACCAATCATAATCGGAGGGTTCGGAAATTGATTAGTTCCTATTATATTAGGTG  218
```

```
            ||||||||||||||||||||||||||||||||||||||||||||||||||||||||||||
Sbjct  121  TAGTTATACCAATCATAATCGGAGGGTTCGGAAATTGATTAGTTCCTATTATATTAGGTG  180

Query  219  CCCCAGATATAGCATTTCCACGAATAAATAATATAAGATTTTGACTTTTACCCCCCTCAT  278
            ||||||||||||||||||||||||||||||||||||||||||||||||||||||||||||
Sbjct  181  CCCCAGATATAGCATTTCCACGAATAAATAATATAAGATTTTGACTTTTACCCCCCTCAT  240

Query  279  TATTTTTATTGATTAACTCTTCATTAATTGAATCCGGAGCTGGAACAGGATGAACAGTTT  338
            ||||||||||||||||||||||||||||||||||||||||||||||||||||||||||||
Sbjct  241  TATTTTTATTGATTAACTCTTCATTAATTGAATCCGGAGCTGGAACAGGATGAACAGTTT  300

Query  339  ATCCCCCTCTATCTTCAAATTTATCACATTATGGTCCATCAGTAGATTTAGCTATTTTTT  398
            ||||||||||||||||||||||||||||||||||||||||||||||||||||||||||||
Sbjct  301  ATCCCCCTCTATCTTCAAATTTATCACATTATGGTCCATCAGTAGATTTAGCTATTTTTT  360

Query  399  CTCTTCACCTTGCTGGTGCTTCTTCAATTTTAGGTGCAATTAACTTCATCACAACTATTG  458
            ||||||||||||||||||||||||||||||||||||||||||||||||||||||||||||
Sbjct  361  CTCTTCACCTTGCTGGTGCTTCTTCAATTTTAGGTGCAATTAACTTCATCACAACTATTG  420

Query  459  TAAATATACGATCAATTGGAATAACAATAGAACGAATACCATTATTTGTTTGATCTGTTT  518
            ||||||||||||||||||||||||||||||||||||||||||||||||||||||||||||
Sbjct  421  TAAATATACGATCAATTGGAATAACAATAGAACGAATACCATTATTTGTTTGATCTGTTT  480

Query  519  TAATTACAGCTATTTTACTACTTCTATCTTTACCTGTATTAGCAGGTGCCATTACAATAT  578
            ||||||||||||||||||||||||||||||||||||||||||||||||||||||||||||
Sbjct  481  TAATTACAGCTATTTTACTACTTCTATCTTTACCTGTATTAGCAGGTGCCATTACAATAT  540

Query  579  TATTAACTGATCGAAACTTTAACACATCATTTTTGATCCTTCAGGAGGAGGAGATCCCA  638
            |||||||||||||||||||||||||||||||||||| |||||||||||||||||||||||
Sbjct  541  TATTAACTGATCGAAACTTTAACACATCATTTTTGACCCTTCAGGAGGAGGAGATCCCA  600

Query  639  TTTTATATCAACACTTATTT  658
            ||||||||||||||||||||
Sbjct  601  TTTTATATCAACACTTATTT  620
```

>Rhipicephalus sanguineus isolate Italy-A cytochrome oxidase subunit I (COI) gene, partial cds; mitochondrial
Sequence ID: KX757903.1 Length: 630
Range 1: 1 to 620

Score:1129 bits(611), Expect:0.0,
Identities:617/620(99%), Gaps:0/620(0%), Strand: Plus/Plus

```
Query  39   TAGGACTTAGTATAAGAATATTAATTCGTATAGAATTAGGACAACCTGGAACTTTAATTG  98
            ||||||||||||||||||||||||||||||||||||||||||||||||||||||||||||
Sbjct  1    TAGGACTTAGTATAAGAATATTAATTCGTATAGAATTAGGACAACCTGGAACTTTAATTG  60

Query  99   GAAATGATCAGATTTATAATGTAATCGTAACAGCACATGCATTTATTATAATTTTCTTTA  158
            ||||||||||||||||||||||||||||||||||||||||||||||||||||||||||||
Sbjct  61   GAAATGATCAGATTTATAATGTAATCGTAACAGCACATGCATTTATTATAATTTTCTTTA  120
```

```
Query  159   TAGTTATACCAATCATAATCGGAGGGTTCGGAAATTGATTAGTTCCTATTATATTAGGTG  218
             ||||||||||||||||||||||||||||||||||||||||||||||||||||||||||||
Sbjct  121   TAGTTATACCAATCATAATCGGAGGGTTCGGAAATTGATTAGTTCCTATTATATTAGGTG  180

Query  219   CCCCAGATATAGCATTTCCACGAATAAATAATATAAGATTTTGACTTTTACCCCCCTCAT  278
             ||||||||||||||||||||||||||||||||||||||||||||||||||||||||||||
Sbjct  181   CCCCAGATATAGCATTTCCACGAATAAATAATATAAGATTTTGACTTTTACCCCCCTCAT  240

Query  279   TATTTTTATTGATTAACTCTTCATTAATTGAATCCGGAGCTGGAACAGGATGAACAGTTT  338
             |||||||||||||||| |||||||||||||||||||||||||||||||||||||||||||
Sbjct  241   TATTTTTATTGATTAATTCTTCATTAATTGAATCCGGAGCTGGAACAGGATGAACAGTTT  300

Query  339   ATCCCCCTCTATCTTCAAATTTATCACATTATGGTCCATCAGTAGATTTAGCTATTTTTT  398
             ||||||||||||||||||||||||||||||||||||||||||||||||||||||||||||
Sbjct  301   ATCCCCCTCTATCTTCAAATTTATCACATTATGGTCCATCAGTAGATTTAGCTATTTTTT  360

Query  399   CTCTTCACCTTGCTGGTGCTTCTTCAATTTTAGGTGCAATTAACTTCATCACAACTATTG  458
             ||||||||||||||||||||||||||||||||||||||||||||||||| ||||||||||
Sbjct  361   CTCTTCACCTTGCTGGTGCTTCTTCAATTTTAGGTGCAATTAACTTCATTACAACTATTG  420

Query  459   TAAATATACGATCAATTGGAATAACAATAGAACGAATACCATTATTTGTTTGATCTGTTT  518
             ||||||||||||||||||||||||||||||||||||||||||||||||||||||||||||
Sbjct  421   TAAATATACGATCAATTGGAATAACAATAGAACGAATACCATTATTTGTTTGATCTGTTT  480

Query  519   TAATTACAGCTATTTTACTACTTCTATCTTTACCTGTATTAGCAGGTGCCATTACAATAT  578
             ||||||||||||||||||||||||||||||||||||||||||||||||||||||||||||
Sbjct  481   TAATTACAGCTATTTTACTACTTCTATCTTTACCTGTATTAGCAGGTGCCATTACAATAT  540

Query  579   TATTAACTGATCGAAACTTTAACACATCATTTTTTGATCCTTCAGGAGGAGGAGATCCCA  638
             ||||||||||||||||||||||||||||||||||||| ||||||||||||||||||||||
Sbjct  541   TATTAACTGATCGAAACTTTAACACATCATTTTTTGACCCTTCAGGAGGAGGAGATCCCA  600

Query  639   TTTTATATCAACACTTATTT  658
             ||||||||||||||||||||
Sbjct  601   TTTTATATCAACACTTATTT  620
```

>Rhipicephalus sanguineus isolate Al-Hosema-A cytochrome oxidase subunit I (COI) gene, partial cds; mitochondrial
Sequence ID: KX757909.1 Length: 630
Range 1: 1 to 620

Score:1112 bits(602), Expect:0.0,
Identities:614/620(99%), Gaps:0/620(0%), Strand: Plus/Plus

```
Query  39    TAGGACTTAGTATAAGAATATTAATTCGTATAGAATTAGGACAACCTGGAACTTTAATTG  98
             ||||||||||||||||||||||||||||||||||||||||||||||||||||||||||||
Sbjct  1     TAGGACTTAGTATAAGAATATTAATTCGTATAGAATTAGGACAACCTGGAACTTTAATTG  60

Query  99    GAAATGATCAGATTTATAATGTAATCGTAACAGCACATGCATTTATTATAATTTTCTTTA  158
             ||||||||||||||||||||||||||||||||||||||||||||||||||||||||||||
Sbjct  61    GAAATGATCAGATTTATAATGTAATCGTAACAGCACATGCATTTATTATAATTTTCTTTA  120
```

```
Query  159   TAGTTATACCAATCATAATCGGAGGGTTCGGAAATTGATTAGTTCCTATTATATTAGGTG  218
             ||||||||||||| ||||||||||||||||||||||||||||||||||||||||||||||
Sbjct  121   TAGTTATACCAATAATAATCGGAGGGTTCGGAAATTGATTAGTTCCTATTATATTAGGTG  180

Query  219   CCCCAGATATAGCATTTCCACGAATAAATAATATAAGATTTTGACTTTTACCCCCCTCAT  278
             ||||||||||||||||||||||||||||||||||||||||||||||||||||||||||||
Sbjct  181   CCCCAGATATAGCATTTCCACGAATAAATAATATAAGATTTTGACTTTTACCCCCCTCAT  240

Query  279   TATTTTTATTGATTAACTCTTCATTAATTGAATCCGGAGCTGGAACAGGATGAACAGTTT  338
             |||||||||||||||||||||||||||||||||| |||||||||||||||||||||||||
Sbjct  241   TATTTTTATTGATTAACTCTTCATTAATTGAATCTGGAGCTGGAACAGGATGAACAGTTT  300

Query  339   ATCCCCCTCTATCTTCAAATTTATCACATTATGGTCCATCAGTAGATTTAGCTATTTTTT  398
             |||||||||||||||||||||||||||||||||| |||||||||||||||||||||||||
Sbjct  301   ATCCCCCTCTATCTTCAAATTTATCACATTATGGCCCATCAGTAGATTTAGCTATTTTTT  360

Query  399   CTCTTCACCTTGCTGGTGCTTCTTCAATTTTAGGTGCAATTAACTTCATCACAACTATTG  458
             ||||||||||||||||||||||||||||||||||||||||||||||||||||||||||||
Sbjct  361   CTCTTCACCTTGCTGGTGCTTCTTCAATTTTAGGTGCAATTAACTTCATCACAACTATTG  420

Query  459   TAAATATACGATCAATTGGAATAACAATAGAACGAATACCATTATTTGTTTGATCTGTTT  518
             ||||||||||||||||||||||||||||||||||||||||||||||||||||||||||||
Sbjct  421   TAAATATACGATCAATTGGAATAACAATAGAACGAATACCATTATTTGTTTGATCTGTTT  480

Query  519   TAATTACAGCTATTTTACTACTTCTATCTTTACCTGTATTAGCAGGTGCCATTACAATAT  578
             ||||||||||||||||||||||||||||||||||||||||||||||||||||||||||||
Sbjct  481   TAATTACAGCTATTTTACTACTTCTATCTTTACCTGTATTAGCAGGTGCCATTACAATAT  540

Query  579   TATTAACTGATCGAAACTTTAACACATCATTTTTTGATCCTTCAGGAGGAGGAGATCCCA  638
             |||| ||||||||||||||||||||||||||||||||| |||||||||||||| |||||||
Sbjct  541   TATTGACTGATCGAAACTTTAACACATCATTTTTTGACCCTTCAGGAGGAGGGGATCCCA  600

Query  639   TTTTATATCAACACTTATTT  658
             ||||||||||||||||||||
Sbjct  601   TTTTATATCAACACTTATTT  620
```

>Rhipicephalus sanguineus sensu lato 'temperate lineage' isolate S48 cytochrome c oxidase subunit I (COI) gene, partial cds; mitochondrial
Sequence ID: MF426015.1 Length: 692
Range 1: 19 to 676

Score:1072 bits(580), Expect:0.0,
Identities:632/658(96%), Gaps:0/658(0%), Strand: Plus/Plus

```
Query  1     AACAATATATTTAATTTTTGGAGCATGATCAGGTATATTAGGACTTAGTATAAGAATATT  60
             ||||||||||||||||||||||||||||||||||||||||||||||||||||||||||||
Sbjct  19    AACAATATATTTAATTTTTGGAGCATGATCAGGTATATTAGGACTTAGTATAAGAATATT  78

Query  61    AATTCGTATAGAATTAGGACAACCTGGAACTTTAATTGGAAATGATCAGATTTATAATGT  120
             ||||||||||||||||||||||||||||||||||||||||||||||||||||||||||||
```

```
Sbjct  79   AATTCGTATAGAATTAGGACAACCTGGAACTTTAATTGGAAATGATCAGATTTATAATGT  138

Query  121  AATCGTAACAGCACATGCATTTATTATAATTTTCTTTATAGTTATACCAATCATAATCGG  180
            ||||||||||||||||||||||||||||||||| ||||||||||||||||||||||||||
Sbjct  139  AATCGTAACAGCACATGCATTTATTATAATTTTTTTTATAGTTATACCAATCATAATCGG  198

Query  181  AGGGTTCGGAAATTGATTAGTTCCTATTATATTAGGTGCCCCAGATATAGCATTTCCACG  240
            ||| || |||||||||||||| ||||| ||||| ||||||||||||||||||||||||||
Sbjct  199  AGGATTTGGAAATTGATTAGTCCCTATCATATTGGGTGCCCCAGATATAGCATTTCCACG  258

Query  241  AATAAATAATATAAGATTTTGACTTTTACCCCCCTCATTATTTTTATTGATTAACTCTTC  300
            |||||||||||||||||||||||||||||| || ||| |||||||||| |||||||||||
Sbjct  259  AATAAATAATATAAGATTTTGACTTTTACCTCCTTCACTATTTTTATTAATTAACTCTTC  318

Query  301  ATTAATTGAATCCGGAGCTGGAACAGGATGAACAGTTTATCCCCCTCTATCTTCAAATTT  360
            |||||||||||| |||||||| ||||||||||||||||| || |||||||||||||||||
Sbjct  319  ATTAATTGAATCTGGAGCTGGTACAGGATGAACAGTTTACCCTCCTCTATCTTCAAATTT  378

Query  361  ATCACATTATGGTCCATCAGTAGATTTAGCTATTTTTTCTCTTCACCTTGCTGGTGCTTC  420
            ||||||||||||||| ||||||||||||||||||||||||||||| ||||||||||||||
Sbjct  379  ATCACATTATGGTCCTTCAGTAGATTTAGCTATTTTTTCTCTTCATCTTGCTGGTGCTTC  438

Query  421  TTCAATTTTAGGTGCAATTAACTTCATCACAACTATTGTAAATATACGATCAATTGGAAT  480
            |||||||||||||||||||||||| || |||||||||||||| |||||||||||||||||
Sbjct  439  TTCAATTTTAGGTGCAATTAACTTTATTACAACTATTGTAAACATACGATCAATTGGAAT  498

Query  481  AACAATAGAACGAATACCATTATTTGTTTGATCTGTTTTAATTACAGCTATTTTACTACT  540
            |||||||||||| |||||||||||||||||||||||||||||||||||||||||||||||
Sbjct  499  AACAATAGAACGTATACCATTATTTGTTTGATCTGTTTTAATTACAGCTATTTTACTACT  558

Query  541  TCTATCTTTACCTGTATTAGCAGGTGCCATTACAATATTATTAACTGATCGAAACTTTAA  600
            | ||||||||||||||||||||||||||||||||||||| ||||||||||||||||||||
Sbjct  559  TTTATCTTTACCTGTATTAGCAGGTGCCATTACAATATTGTTAACTGATCGAAACTTTAA  618

Query  601  CACATCATTTTTTGATCCTTCAGGAGGAGGAGATCCCATTTTATATCAACACTTATTT  658
             ||||||||||||||||||||||| ||||||||||| |||||||||||||| ||||||
Sbjct  619  TACATCATTTTTTGATCCTTCAGGGGGAGGAGATCCAATTTTATATCAACATTTATTT  676
```

>Rhipicephalus sanguineus sensu lato 'temperate lineage' isolate S58 cytochrome c oxidase subunit I (COI) gene, partial cds; mitochondrial
Sequence ID: MF426018.1 Length: 675
Range 1: 11 to 668

Score:1066 bits(577), Expect:0.0,
Identities:631/658(96%), Gaps:0/658(0%), Strand: Plus/Plus

```
Query  1    AACAATATATTTAATTTTTGGAGCATGATCAGGTATATTAGGACTTAGTATAAGAATATT  60
            ||||||||||||||||||||||||||||||||||||||||||||||||||||||||||||
Sbjct  11   AACAATATATTTAATTTTTGGAGCATGATCAGGTATATTAGGACTTAGTATAAGAATATT  70

Query  61   AATTCGTATAGAATTAGGACAACCTGGAACTTTAATTGGAAATGATCAGATTTATAATGT  120
```

```
            ||||||||||||||||||||||||||||||||||||||||||||||||||||||||||||
Sbjct  71   AATTCGTATAGAATTAGGACAACCTGGAACTTTAATTGGAAATGATCAGATTTATAATGT  130

Query  121  AATCGTAACAGCACATGCATTTATTATAATTTTCTTTATAGTTATACCAATCATAATCGG  180
            ||||||||||||||||||||||||||||||||| ||||||||||||||||||||||||||
Sbjct  131  AATCGTAACAGCACATGCATTTATTATAATTTTTTTTATAGTTATACCAATCATAATCGG  190

Query  181  AGGGTTCGGAAATTGATTAGTTCCTATTATATTAGGTGCCCCAGATATAGCATTTCCACG  240
            ||| || |||||||||||||| ||||| ||||| ||||||||||||||||||||||||||
Sbjct  191  AGGATTTGGAAATTGATTAGTCCCTATCATATTGGGTGCCCCAGATATAGCATTTCCACG  250

Query  241  AATAAATAATATAAGATTTTGACTTTTACCCCCCTCATTATTTTTATTGATTAACTCTTC  300
            |||||||||||||||||||||||||||||| || ||| |||||||||| |||||||||||
Sbjct  251  AATAAATAATATAAGATTTTGACTTTTACCTCCTTCACTATTTTTATTAATTAACTCTTC  310

Query  301  ATTAATTGAATCCGGAGCTGGAACAGGATGAACAGTTTATCCCCCTCTATCTTCAAATTT  360
            |||||||||||| |||||||| ||||||||||||||||| || |||||||||||||||||
Sbjct  311  ATTAATTGAATCTGGAGCTGGTACAGGATGAACAGTTTACCCTCCTCTATCTTCAAATTT  370

Query  361  ATCACATTATGGTCCATCAGTAGATTTAGCTATTTTTTCTCTTCACCTTGCTGGTGCTTC  420
            ||||||||||||||| ||||||||||||||||||||||||||||| ||||||||||||||
Sbjct  371  ATCACATTATGGTCCTTCAGTAGATTTAGCTATTTTTTCTCTTCATCTTGCTGGTGCTTC  430

Query  421  TTCAATTTTAGGTGCAATTAACTTCATCACAACTATTGTAAATATACGATCAATTGGAAT  480
            |||||||||||||||||||||||| || |||||||||||||| |||||||||||||||||
Sbjct  431  TTCAATTTTAGGTGCAATTAACTTTATTACAACTATTGTAAACATACGATCAATTGGAAT  490

Query  481  AACAATAGAACGAATACCATTATTTGTTTGATCTGTTTTAATTACAGCTATTTTACTACT  540
            |||||||||||| |||||||||||||||||||||||||||||||||||||||||| ||||
Sbjct  491  AACAATAGAACGTATACCATTATTTGTTTGATCTGTTTTAATTACAGCTATTTTATTACT  550

Query  541  TCTATCTTTACCTGTATTAGCAGGTGCCATTACAATATTATTAACTGATCGAAACTTTAA  600
            | ||||||||||||||||||||||||||||||||||||| ||||||||||||||||||||
Sbjct  551  TTTATCTTTACCTGTATTAGCAGGTGCCATTACAATATTGTTAACTGATCGAAACTTTAA  610

Query  601  CACATCATTTTTTGATCCTTCAGGAGGAGGAGATCCCATTTTATATCAACACTTATTT  658
             ||||||||||||||||||||||| ||||||||||| |||||||||||||| ||||||
Sbjct  611  TACATCATTTTTTGATCCTTCAGGGGGAGGAGATCCAATTTTATATCAACATTTATTT  668
```

>Rhipicephalus sanguineus sensu lato 'temperate lineage' isolate S52 cytochrome c oxidase subunit I (COI) gene, partial cds; mitochondrial
Sequence ID: MF426016.1 Length: 680
Range 1: 12 to 669

Score:1066 bits(577), Expect:0.0,
Identities:631/658(96%), Gaps:0/658(0%), Strand: Plus/Plus

```
Query  1    AACAATATATTTAATTTTTGGAGCATGATCAGGTATATTAGGACTTAGTATAAGAATATT  60
            ||||||||||||||||||||||||||||||||||||||||||||||||||||||||||||
Sbjct  12   AACAATATATTTAATTTTTGGAGCATGATCAGGTATATTAGGACTTAGTATAAGAATATT  71
```

```
Query  61   AATTCGTATAGAATTAGGACAACCTGGAACTTTAATTGGAAATGATCAGATTTATAATGT  120
            ||||||||||||||||||||||||||||||||||||||||||||||||||||||||||||
Sbjct  72   AATTCGTATAGAATTAGGACAACCTGGAACTTTAATTGGAAATGATCAGATTTATAATGT  131

Query  121  AATCGTAACAGCACATGCATTTATTATAATTTTCTTTATAGTTATACCAATCATAATCGG  180
            ||||||||||||||||||||||||||||||||| ||||||||||||||||||||||||||
Sbjct  132  AATCGTAACAGCACATGCATTTATTATAATTTTTTTTATAGTTATACCAATCATAATCGG  191

Query  181  AGGGTTCGGAAATTGATTAGTTCCTATTATATTAGGTGCCCCAGATATAGCATTTCCACG  240
            ||| || |||||||||||||| ||||| ||||| ||||||||||||||||||||||||||
Sbjct  192  AGGATTTGGAAATTGATTAGTCCCTATCATATTGGGTGCCCCAGATATAGCATTTCCACG  251

Query  241  AATAAATAATATAAGATTTTGACTTTTACCCCCCTCATTATTTTTATTGATTAACTCTTC  300
            |||||||||||||||||||||||||||||| || ||| |||||||||| |||||||||||
Sbjct  252  AATAAATAATATAAGATTTTGACTTTTACCTCCTTCACTATTTTTATTAATTAACTCTTC  311

Query  301  ATTAATTGAATCCGGAGCTGGAACAGGATGAACAGTTTATCCCCCTCTATCTTCAAATTT  360
            |||||||||||| |||||||| ||||||||||||||||| || |||||||||||||||||
Sbjct  312  ATTAATTGAATCTGGAGCTGGTACAGGATGAACAGTTTACCCTCCTCTATCTTCAAATTT  371

Query  361  ATCACATTATGGTCCATCAGTAGATTTAGCTATTTTTTCTCTTCACCTTGCTGGTGCTTC  420
            ||||||||||||||| ||||||||||||||||||||||||||||| ||||||||||||||
Sbjct  372  ATCACATTATGGTCCTTCAGTAGATTTAGCTATTTTTTCTCTTCATCTTGCTGGTGCTTC  431

Query  421  TTCAATTTTAGGTGCAATTAACTTCATCACAACTATTGTAAATATACGATCAATTGGAAT  480
            |||||||||||||||||||||||| || |||||||||||||| |||||||||||||||||
Sbjct  432  TTCAATTTTAGGTGCAATTAACTTTATTACAACTATTGTAAACATACGATCAATTGGAAT  491

Query  481  AACAATAGAACGAATACCATTATTTGTTTGATCTGTTTTAATTACAGCTATTTTACTACT  540
            |||||||||||| |||||||||||||||||||||||||||||||||||||||||| ||||
Sbjct  492  AACAATAGAACGTATACCATTATTTGTTTGATCTGTTTTAATTACAGCTATTTTATTACT  551

Query  541  TCTATCTTTACCTGTATTAGCAGGTGCCATTACAATATTATTAACTGATCGAAACTTTAA  600
            | ||||||||||||||||||||||||||||||||||||| ||||||||||||||||||||
Sbjct  552  TTTATCTTTACCTGTATTAGCAGGTGCCATTACAATATTGTTAACTGATCGAAACTTTAA  611

Query  601  CACATCATTTTTTGATCCTTCAGGAGGAGGAGATCCCATTTTATATCAACACTTATTT  658
             ||||||||||||||||||||||| ||||||||||| |||||||||||||| ||||||
Sbjct  612  TACATCATTTTTTGATCCTTCAGGGGGAGGAGATCCAATTTTATATCAACATTTATTT  669
```

>Rhipicephalus sanguineus sensu lato 'temperate lineage' isolate S37 cytochrome c oxidase subunit I (COI) gene, partial cds; mitochondrial
Sequence ID: MF426013.1 Length: 693
Range 1: 20 to 677

Score:1066 bits(577), Expect:0.0,
Identities:631/658(96%), Gaps:0/658(0%), Strand: Plus/Plus

```
Query  1    AACAATATATTTAATTTTTGGAGCATGATCAGGTATATTAGGACTTAGTATAAGAATATT  60
            ||||||||||||||||||||||||||||||||||||||||||||||||||||||||||||
Sbjct  20   AACAATATATTTAATTTTTGGAGCATGATCAGGTATATTAGGACTTAGTATAAGAATATT  79
```

```
Query  61   AATTCGTATAGAATTAGGACAACCTGGAACTTTAATTGGAAATGATCAGATTTATAATGT  120
            ||||||||||||||||||||||||||||||||||||||||||||||||||||||||||||
Sbjct  80   AATTCGTATAGAATTAGGACAACCTGGAACTTTAATTGGAAATGATCAGATTTATAATGT  139

Query  121  AATCGTAACAGCACATGCATTTATTATAATTTCTTTATAGTTATACCAATCATAATCGG  180
            |||||||||||||||||||||||||||||||| |||||||||||||||||||||||||||
Sbjct  140  AATCGTAACAGCACATGCATTTATTATAATTTTTTTATAGTTATACCAATCATAATCGG  199

Query  181  AGGGTTCGGAAATTGATTAGTTCCTATTATATTAGGTGCCCCAGATATAGCATTTCCACG  240
            ||| || |||||||||||||| ||||| ||||| ||||||||||||||||||||||||||
Sbjct  200  AGGATTTGGAAATTGATTAGTCCCTATCATATTGGGTGCCCCAGATATAGCATTTCCACG  259

Query  241  AATAAATAATATAAGATTTTGACTTTTACCCCCCTCATTATTTTTATTGATTAACTCTTC  300
            |||||||||||||||||||||||||||||| || ||| |||||||||| |||||||||||
Sbjct  260  AATAAATAATATAAGATTTTGACTTTTACCTCCTTCACTATTTTTATTAATTAACTCTTC  319

Query  301  ATTAATTGAATCCGGAGCTGGAACAGGATGAACAGTTTATCCCCCTCTATCTTCAAATTT  360
            |||||||||||| |||||||| ||||||||||||||||| || |||||||||||||||||
Sbjct  320  ATTAATTGAATCTGGAGCTGGTACAGGATGAACAGTTTACCCTCCTCTATCTTCAAATTT  379

Query  361  ATCACATTATGGTCCATCAGTAGATTTAGCTATTTTTTCTCTTCACCTTGCTGGTGCTTC  420
            ||||||||||||||| ||||||||||||||||||||||||||||| ||||||||||||||
Sbjct  380  ATCACATTATGGTCCTTCAGTAGATTTAGCTATTTTTTCTCTTCATCTTGCTGGTGCTTC  439

Query  421  TTCAATTTTAGGTGCAATTAACTTCATCACAACTATTGTAAATATACGATCAATTGGAAT  480
            |||||||||||||||||||||||| || |||||||||||||| |||||||||||||||||
Sbjct  440  TTCAATTTTAGGTGCAATTAACTTTATTACAACTATTGTAAACATACGATCAATTGGAAT  499

Query  481  AACAATAGAACGAATACCATTATTTGTTTGATCTGTTTTAATTACAGCTATTTTACTACT  540
            |||||||||||| |||||||||||||||||||||||||||||||||||||||||| ||||
Sbjct  500  AACAATAGAACGTATACCATTATTTGTTTGATCTGTTTTAATTACAGCTATTTTATTACT  559

Query  541  TCTATCTTTACCTGTATTAGCAGGTGCCATTACAATATTATTAACTGATCGAAACTTTAA  600
            | ||||||||||||||||||||||||||||||||||||| ||||||||||||||||||||
Sbjct  560  TTTATCTTTACCTGTATTAGCAGGTGCCATTACAATATTGTTAACTGATCGAAACTTTAA  619

Query  601  CACATCATTTTTTGATCCTTCAGGAGGAGGAGATCCCATTTTATATCAACACTTATTT  658
             ||||||||||||||||||||||| ||||||||||| |||||||||||||| ||||||
Sbjct  620  TACATCATTTTTTGATCCTTCAGGGGGAGGAGATCCAATTTTATATCAACATTTATTT  677
```

>Rhipicephalus sanguineus sensu lato 'temperate lineage' isolate S33 cytochrome c oxidase subunit I (COI) gene, partial cds; mitochondrial
Sequence ID: MF426009.1 Length: 679
Range 1: 11 to 668

Score:1066 bits(577), Expect:0.0,
Identities:631/658(96%), Gaps:0/658(0%), Strand: Plus/Plus

```
Query  1    AACAATATATTTAATTTTTGGAGCATGATCAGGTATATTAGGACTTAGTATAAGAATATT  60
            ||||||||||||||||||||||||||||||||||||||||||||||||||||||||||||
```

```
Sbjct  11   AACAATATATTTAATTTTTGGAGCATGATCAGGTATATTAGGACTTAGTATAAGAATATT  70

Query  61   AATTCGTATAGAATTAGGACAACCTGGAACTTTAATTGGAAATGATCAGATTTATAATGT  120
            ||||||||||||||||||||||||||||||||||||||||||||||||||||||||||||
Sbjct  71   AATTCGTATAGAATTAGGACAACCTGGAACTTTAATTGGAAATGATCAGATTTATAATGT  130

Query  121  AATCGTAACAGCACATGCATTTATTATAATTTTCTTTATAGTTATACCAATCATAATCGG  180
            ||||||||||||||||||||||||||||||||| ||||||||||||||||||||||||||
Sbjct  131  AATCGTAACAGCACATGCATTTATTATAATTTTTTTTATAGTTATACCAATCATAATCGG  190

Query  181  AGGGTTCGGAAATTGATTAGTTCCTATTATATTAGGTGCCCCAGATATAGCATTTCCACG  240
            ||| || |||||||||||||| ||||| ||||| ||||||||||||||||||||||||||
Sbjct  191  AGGATTTGGAAATTGATTAGTCCCTATCATATTGGGTGCCCCAGATATAGCATTTCCACG  250

Query  241  AATAAATAATATAAGATTTTGACTTTTACCCCCCTCATTATTTTTATTGATTAACTCTTC  300
            |||||||||||||||||||||||||||||| || ||| |||||||||| |||||||||||
Sbjct  251  AATAAATAATATAAGATTTTGACTTTTACCTCCTTCACTATTTTTATTAATTAACTCTTC  310

Query  301  ATTAATTGAATCCGGAGCTGGAACAGGATGAACAGTTTATCCCCCTCTATCTTCAAATTT  360
            |||||||||||| |||||||| ||||||||||||||||| || |||||||||||||||||
Sbjct  311  ATTAATTGAATCTGGAGCTGGTACAGGATGAACAGTTTACCCTCCTCTATCTTCAAATTT  370

Query  361  ATCACATTATGGTCCATCAGTAGATTTAGCTATTTTTTCTCTTCACCTTGCTGGTGCTTC  420
            ||||||||||||||| ||||||||||||||||||||||||||||| ||||||||||||||
Sbjct  371  ATCACATTATGGTCCTTCAGTAGATTTAGCTATTTTTTCTCTTCATCTTGCTGGTGCTTC  430

Query  421  TTCAATTTTAGGTGCAATTAACTTCATCACAACTATTGTAAATATACGATCAATTGGAAT  480
            |||||||||||||||||||||||| || |||||||||||||| |||||||||||||||||
Sbjct  431  TTCAATTTTAGGTGCAATTAACTTTATTACAACTATTGTAAACATACGATCAATTGGAAT  490

Query  481  AACAATAGAACGAATACCATTATTTGTTTGATCTGTTTTAATTACAGCTATTTTACTACT  540
            |||||||||||| |||||||||||||||||||||||||||||||||||||||||| ||||
Sbjct  491  AACAATAGAACGTATACCATTATTTGTTTGATCTGTTTTAATTACAGCTATTTTATTACT  550

Query  541  TCTATCTTTACCTGTATTAGCAGGTGCCATTACAATATTATTAACTGATCGAAACTTTAA  600
            | ||||||||||||||||||||||||||||||||||||| ||||||||||||||||||||
Sbjct  551  TTTATCTTTACCTGTATTAGCAGGTGCCATTACAATATTGTTAACTGATCGAAACTTTAA  610

Query  601  CACATCATTTTTTGATCCTTCAGGAGGAGGAGATCCCATTTTATATCAACACTTATTT  658
             ||||||||||||||||||||||| ||||||||||| |||||||||||||| ||||||
Sbjct  611  TACATCATTTTTTGATCCTTCAGGGGGAGGAGATCCAATTTTATATCAACATTTATTT  668
```

>Rhipicephalus sanguineus sensu lato 'temperate lineage' isolate S29 cytochrome c oxidase subunit I (COI) gene, partial cds; mitochondrial
Sequence ID: MF426007.1 Length: 686
Range 1: 14 to 671

Score:1066 bits(577), Expect:0.0,
Identities:631/658(96%), Gaps:0/658(0%), Strand: Plus/Plus

```
Query  1    AACAATATATTTAATTTTTGGAGCATGATCAGGTATATTAGGACTTAGTATAAGAATATT  60
```

```
             ||||||||||||||||||||||||||||||||||||||||||||||||||||||||||||
Sbjct  14    AACAATATATTTAATTTTTGGAGCATGATCAGGTATATTAGGACTTAGTATAAGAATATT  73

Query  61    AATTCGTATAGAATTAGGACAACCTGGAACTTTAATTGGAAATGATCAGATTTATAATGT  120
             ||||||||||||||||||||||||||||||||||||||||||||||||||||||||||||
Sbjct  74    AATTCGTATAGAATTAGGACAACCTGGAACTTTAATTGGAAATGATCAGATTTATAATGT  133

Query  121   AATCGTAACAGCACATGCATTTATTATAATTTTCTTTATAGTTATACCAATCATAATCGG  180
             ||||||||||||||||||||||||||||||||| ||||||||||||||||||||||||||
Sbjct  134   AATCGTAACAGCACATGCATTTATTATAATTTTTTTTATAGTTATACCAATCATAATCGG  193

Query  181   AGGGTTCGGAAATTGATTAGTTCCTATTATATTAGGTGCCCCAGATATAGCATTTCCACG  240
             ||| || |||||||||||||| ||||| ||||| ||||||||||||||||||||||||||
Sbjct  194   AGGATTTGGAAATTGATTAGTCCCTATCATATTGGGTGCCCCAGATATAGCATTTCCACG  253

Query  241   AATAAATAATATAAGATTTTGACTTTTACCCCCCTCATTATTTTTATTGATTAACTCTTC  300
             |||||||||||||||||||||||||||||| || ||| |||||||||| |||||||||||
Sbjct  254   AATAAATAATATAAGATTTTGACTTTTACCTCCTTCACTATTTTTATTAATTAACTCTTC  313

Query  301   ATTAATTGAATCCGGAGCTGGAACAGGATGAACAGTTTATCCCCCTCTATCTTCAAATTT  360
             |||||||||||| |||||||| ||||||||||||||||| || |||||||||||||||||
Sbjct  314   ATTAATTGAATCTGGAGCTGGTACAGGATGAACAGTTTACCCTCCTCTATCTTCAAATTT  373

Query  361   ATCACATTATGGTCCATCAGTAGATTTAGCTATTTTTTCTCTTCACCTTGCTGGTGCTTC  420
             ||||||||||||||| |||||||||||||||||||||||||||| |||||||||||||||
Sbjct  374   ATCACATTATGGTCCTTCAGTAGATTTAGCTATTTTTTCTCTTCATCTTGCTGGTGCTTC  433

Query  421   TTCAATTTTAGGTGCAATTAACTTCATCACAACTATTGTAAATATACGATCAATTGGAAT  480
             |||||||||||||||||||||||| || ||||||||||||||| ||||||||||||||||
Sbjct  434   TTCAATTTTAGGTGCAATTAACTTTATTACAACTATTGTAAACATACGATCAATTGGAAT  493

Query  481   AACAATAGAACGAATACCATTATTTGTTTGATCTGTTTTAATTACAGCTATTTTACTACT  540
             |||||||||||| |||||||||||||||||||||||||||||||||||||||||| ||||
Sbjct  494   AACAATAGAACGTATACCATTATTTGTTTGATCTGTTTTAATTACAGCTATTTTATTACT  553

Query  541   TCTATCTTTACCTGTATTAGCAGGTGCCATTACAATATTATTAACTGATCGAAACTTTAA  600
             | ||||||||||||||||||||||||||||||||||||| ||||||||||||||||||||
Sbjct  554   TTTATCTTTACCTGTATTAGCAGGTGCCATTACAATATTGTTAACTGATCGAAACTTTAA  613

Query  601   CACATCATTTTTTGATCCTTCAGGAGGAGGAGATCCCATTTTATATCAACACTTATTT  658
              ||||||||||||||||||||||| ||||||||||| |||||||||||||| ||||||
Sbjct  614   TACATCATTTTTTGATCCTTCAGGGGGAGGAGATCCAATTTTATATCAACATTTATTT  671
```

>Rhipicephalus sanguineus sensu lato 'temperate lineage' isolate S827 cytochrome c oxidase subunit I (COI) gene, partial cds; mitochondrial
Sequence ID: MF426019.1 Length: 697
Range 1: 19 to 676

Score:1061 bits(574), Expect:0.0,
Identities:630/658(96%), Gaps:0/658(0%), Strand: Plus/Plus

```
Query  1    AACAATATATTTAATTTTTGGAGCATGATCAGGTATATTAGGACTTAGTATAAGAATATT  60
            ||||||||||||||||||||||||||||||||||||||||||||||||||||||||||||
Sbjct  19   AACAATATATTTAATTTTTGGAGCATGATCAGGTATATTAGGACTTAGTATAAGAATATT  78

Query  61   AATTCGTATAGAATTAGGACAACCTGGAACTTTAATTGGAAATGATCAGATTTATAATGT  120
            ||||||||||||||||||||||||||||||||||||||||||||||||||||||||||||
Sbjct  79   AATTCGTATAGAATTAGGACAACCTGGAACTTTAATTGGAAATGATCAGATTTATAATGT  138

Query  121  AATCGTAACAGCACATGCATTTATTATAATTTTCTTTATAGTTATACCAATCATAATCGG  180
            ||||||||||||||||||||||||||||||||| ||||||||||||||||||||||||||
Sbjct  139  AATCGTAACAGCACATGCATTTATTATAATTTTTTTTATAGTTATACCAATCATAATCGG  198

Query  181  AGGGTTCGGAAATTGATTAGTTCCTATTATATTAGGTGCCCCAGATATAGCATTTCCACG  240
            ||| || |||||||||||||| ||||| ||||| ||||||||||||||||||||||||||
Sbjct  199  AGGATTTGGAAATTGATTAGTCCCTATCATATTGGGTGCCCCAGATATAGCATTTCCACG  258

Query  241  AATAAATAATATAAGATTTTGACTTTTACCCCCCTCATTATTTTTATTGATTAACTCTTC  300
            |||||||||||||||||||||||||||||| || ||| |||||||||| |||||||||||
Sbjct  259  AATAAATAATATAAGATTTTGACTTTTACCTCCTTCACTATTTTTATTAATTAACTCTTC  318

Query  301  ATTAATTGAATCCGGAGCTGGAACAGGATGAACAGTTTATCCCCCTCTATCTTCAAATTT  360
            |||||||||||| |||||||| ||||||||||||||||| || |||||||||||||||||
Sbjct  319  ATTAATTGAATCTGGAGCTGGTACAGGATGAACAGTTTACCCTCCTCTATCTTCAAATTT  378

Query  361  ATCACATTATGGTCCATCAGTAGATTTAGCTATTTTTTCTCTTCACCTTGCTGGTGCTTC  420
            |||||||||||| || ||||||||||||||||||||||||||||| ||||||||||||||
Sbjct  379  ATCACATTATGGCCCTTCAGTAGATTTAGCTATTTTTTCTCTTCATCTTGCTGGTGCTTC  438

Query  421  TTCAATTTTAGGTGCAATTAACTTCATCACAACTATTGTAAATATACGATCAATTGGAAT  480
            |||||||||||||||||||||||| || |||||||||||||| |||||||||||||||||
Sbjct  439  TTCAATTTTAGGTGCAATTAACTTTATTACAACTATTGTAAACATACGATCAATTGGAAT  498

Query  481  AACAATAGAACGAATACCATTATTTGTTTGATCTGTTTTAATTACAGCTATTTTACTACT  540
            |||||||||||| |||||||||||||||||||||||||||||||||||||||||| ||||
Sbjct  499  AACAATAGAACGTATACCATTATTTGTTTGATCTGTTTTAATTACAGCTATTTTATTACT  558

Query  541  TCTATCTTTACCTGTATTAGCAGGTGCCATTACAATATTATTAACTGATCGAAACTTTAA  600
            | ||||||||||||||||||||||||||||||||||||| ||||||||||||||||||||
Sbjct  559  TTTATCTTTACCTGTATTAGCAGGTGCCATTACAATATTGTTAACTGATCGAAACTTTAA  618

Query  601  CACATCATTTTTTGATCCTTCAGGAGGAGGAGATCCCATTTTATATCAACACTTATTT  658
             ||||||||||||||||||||||| ||||||||||| |||||||||||||| ||||||
Sbjct  619  TACATCATTTTTTGATCCTTCAGGGGGAGGAGATCCAATTTTATATCAACATTTATTT  676
```

>Rhipicephalus sanguineus sensu lato 'temperate lineage' isolate S1060 cytochrome c oxidase subunit I (COI) gene, partial cds; mitochondrial
Sequence ID: MF426006.1 Length: 705
Range 1: 28 to 685

Score:1061 bits(574), Expect:0.0,
Identities:630/658(96%), Gaps:0/658(0%), Strand: Plus/Plus

```
Query  1    AACAATATATTTAATTTTTGGAGCATGATCAGGTATATTAGGACTTAGTATAAGAATATT  60
            ||||||||||||||||||||||||||||||||||||||||||||||||||||||||||||
Sbjct  28   AACAATATATTTAATTTTTGGAGCATGATCAGGTATATTAGGACTTAGTATAAGAATATT  87

Query  61   AATTCGTATAGAATTAGGACAACCTGGAACTTTAATTGGAAATGATCAGATTTATAATGT  120
            ||||||||||||||||||||||||||||||||||||||||||||||||||||||||||||
Sbjct  88   AATTCGTATAGAATTAGGACAACCTGGAACTTTAATTGGAAATGATCAGATTTATAATGT  147

Query  121  AATCGTAACAGCACATGCATTTATTATAATTTCTTTATAGTTATACCAATCATAATCGG  180
            |||||||||||||||||||||||||||||||| |||||||||||||||||||||||||||
Sbjct  148  AATCGTAACAGCACATGCATTTATTATAATTTTTTTATAGTTATACCAATCATAATCGG  207

Query  181  AGGGTTCGGAAATTGATTAGTTCCTATTATATTAGGTGCCCCAGATATAGCATTTCCACG  240
            ||| || |||||||||||||| ||||| ||||| ||||||||||||||||||||||||||
Sbjct  208  AGGATTTGGAAATTGATTAGTCCCTATCATATTGGGTGCCCCAGATATAGCATTTCCACG  267

Query  241  AATAAATAATATAAGATTTTGACTTTTACCCCCCTCATTATTTTTATTGATTAACTCTTC  300
            |||||||||||||||||||||||||||||| || ||| |||||||||| |||||||||||
Sbjct  268  AATAAATAATATAAGATTTTGACTTTTACCTCCTTCACTATTTTTATTAATTAACTCTTC  327

Query  301  ATTAATTGAATCCGGAGCTGGAACAGGATGAACAGTTTATCCCCCTCTATCTTCAAATTT  360
            |||||||||||| |||||||| ||||||||||||||||| || |||||||||||||||||
Sbjct  328  ATTAATTGAATCTGGAGCTGGTACAGGATGAACAGTTTACCCTCCTCTATCTTCAAATTT  387

Query  361  ATCACATTATGGTCCATCAGTAGATTTAGCTATTTTTTCTCTTCACCTTGCTGGTGCTTC  420
            ||||||||||||||| ||||||||||||||||||||||||||||| ||||||||||||||
Sbjct  388  ATCACATTATGGTCCTTCAGTAGATTTAGCTATTTTTTCTCTTCATCTTGCTGGTGCTTC  447

Query  421  TTCAATTTTAGGTGCAATTAACTTCATCACAACTATTGTAAATATACGATCAATTGGAAT  480
            |||||||||||||||||||||||| || |||||||||||||| ||||||| |||||||||
Sbjct  448  TTCAATTTTAGGTGCAATTAACTTTATTACAACTATTGTAAACATACGATTAATTGGAAT  507

Query  481  AACAATAGAACGAATACCATTATTTGTTTGATCTGTTTTAATTACAGCTATTTTACTACT  540
            |||||||||||| |||||||||||||||||||||||||||||||||||||||||| ||||
Sbjct  508  AACAATAGAACGTATACCATTATTTGTTTGATCTGTTTTAATTACAGCTATTTTATTACT  567

Query  541  TCTATCTTTACCTGTATTAGCAGGTGCCATTACAATATTATTAACTGATCGAAACTTTAA  600
            | ||||||||||||||||||||||||||||||||||||| ||||||||||||||||||||
Sbjct  568  TTTATCTTTACCTGTATTAGCAGGTGCCATTACAATATTGTTAACTGATCGAAACTTTAA  627

Query  601  CACATCATTTTTTGATCCTTCAGGAGGAGGAGATCCCATTTTATATCAACACTTATTT  658
             ||||||||||||||||||||||| ||||||||||| |||||||||||||| ||||||
Sbjct  628  TACATCATTTTTTGATCCTTCAGGGGGAGGAGATCCAATTTTATATCAACATTTATTT  685
```

>Rhipicephalus sanguineus haplotype III cytochrome oxidase subunit I (COI) gene, partial cds; mitochondrial
Sequence ID: KU556745.1 Length: 713
Range 1: 27 to 684

Score:1061 bits(574), Expect:0.0,

Identities:630/658(96%), Gaps:0/658(0%), Strand: Plus/Plus

```
Query  1    AACAATATATTTAATTTTTGGAGCATGATCAGGTATATTAGGACTTAGTATAAGAATATT  60
            ||||||||||||||||||||||||||||||||||||||||||||||||||||||||||||
Sbjct  27   AACAATATATTTAATTTTTGGAGCATGATCAGGTATATTAGGACTTAGTATAAGAATATT  86

Query  61   AATTCGTATAGAATTAGGACAACCTGGAACTTTAATTGGAAATGATCAGATTTATAATGT  120
            ||||||||||||||||||||||||||||||||||||||||||||||||||||||||||||
Sbjct  87   AATTCGTATAGAATTAGGACAACCTGGAACTTTAATTGGAAATGATCAGATTTATAATGT  146

Query  121  AATCGTAACAGCACATGCATTTATTATAATTTTCTTTATAGTTATACCAATCATAATCGG  180
            ||||||||||||||||||||||||||||||||| ||||||||||||||||||||||||||
Sbjct  147  AATCGTAACAGCACATGCATTTATTATAATTTTTTTTATAGTTATACCAATCATAATCGG  206

Query  181  AGGGTTCGGAAATTGATTAGTTCCTATTATATTAGGTGCCCCAGATATAGCATTTCCACG  240
            ||| || |||||||||||||| ||||| ||||| ||||||||||||||||||||||||||
Sbjct  207  AGGATTTGGAAATTGATTAGTCCCTATCATATTGGGTGCCCCAGATATAGCATTTCCACG  266

Query  241  AATAAATAATATAAGATTTTGACTTTTACCCCCCTCATTATTTTTATTGATTAACTCTTC  300
            |||||||||||||||||||||||||||||| || ||| |||||||||| |||||||||||
Sbjct  267  AATAAATAATATAAGATTTTGACTTTTACCTCCTTCACTATTTTTATTAATTAACTCTTC  326

Query  301  ATTAATTGAATCCGGAGCTGGAACAGGATGAACAGTTTATCCCCCTCTATCTTCAAATTT  360
            |||||||||||| |||||||| ||||||||||||||||| || |||||||||||||||||
Sbjct  327  ATTAATTGAATCTGGAGCTGGTACAGGATGAACAGTTTACCCTCCTCTATCTTCAAATTT  386

Query  361  ATCACATTATGGTCCATCAGTAGATTTAGCTATTTTTTCTCTTCACCTTGCTGGTGCTTC  420
            |||||||||||| || ||||||||||||||||||||||||||||| ||||||||||||||
Sbjct  387  ATCACATTATGGCCCTTCAGTAGATTTAGCTATTTTTTCTCTTCATCTTGCTGGTGCTTC  446

Query  421  TTCAATTTTAGGTGCAATTAACTTCATCACAACTATTGTAAATATACGATCAATTGGAAT  480
            |||||||||||||||||||||||| || ||||||||||||||| ||||||||||||||||
Sbjct  447  TTCAATTTTAGGTGCAATTAACTTTATTACAACTATTGTAAACATACGATCAATTGGAAT  506

Query  481  AACAATAGAACGAATACCATTATTTGTTTGATCTGTTTTAATTACAGCTATTTTACTACT  540
            |||||||||||| |||||||||||||||||||||||||||||||||||||||||| ||||
Sbjct  507  AACAATAGAACGTATACCATTATTTGTTTGATCTGTTTTAATTACAGCTATTTTATTACT  566

Query  541  TCTATCTTTACCTGTATTAGCAGGTGCCATTACAATATTATTAACTGATCGAAACTTTAA  600
            | ||||||||||||||||||||||||||||||||||||| ||||||||||||||||||||
Sbjct  567  TTTATCTTTACCTGTATTAGCAGGTGCCATTACAATATTGTTAACTGATCGAAACTTTAA  626

Query  601  CACATCATTTTTTGATCCTTCAGGAGGAGGAGATCCCATTTTATATCAACACTTATTT  658
             ||||||||||||||||||||||| ||||||||||| |||||||||||||| ||||||
Sbjct  627  TACATCATTTTTTGATCCTTCAGGGGGAGGAGATCCAATTTTATATCAACATTTATTT  684
```

>Rhipicephalus sanguineus isolate Arizona-1 cytochrome c oxidase subunit 1 (COI) gene, partial cds; mitochondrial
Sequence ID: MN593344.1 Length: 710
Range 1: 27 to 684

Score:1061 bits(574), Expect:0.0,
Identities:630/658(96%), Gaps:0/658(0%), Strand: Plus/Minus

```
Query  1    AACAATATATTTAATTTTTGGAGCATGATCAGGTATATTAGGACTTAGTATAAGAATATT  60
            ||||||||||||||||||||||||||||||||||||||||||||||||||||||||||||
Sbjct  684  AACAATATATTTAATTTTTGGAGCATGATCAGGTATATTAGGACTTAGTATAAGAATATT  625

Query  61   AATTCGTATAGAATTAGGACAACCTGGAACTTTAATTGGAAATGATCAGATTTATAATGT  120
            ||||||||||||||||||||||||||||||||||||||||||||||||||||||||||||
Sbjct  624  AATTCGTATAGAATTAGGACAACCTGGAACTTTAATTGGAAATGATCAGATTTATAATGT  565

Query  121  AATCGTAACAGCACATGCATTTATTATAATTTTCTTTATAGTTATACCAATCATAATCGG  180
            ||||||||||||||||||||||||||||||||| ||||||||||||||||||||||||||
Sbjct  564  AATCGTAACAGCACATGCATTTATTATAATTTTTTTTATAGTTATACCAATCATAATCGG  505

Query  181  AGGGTTCGGAAATTGATTAGTTCCTATTATATTAGGTGCCCCAGATATAGCATTTCCACG  240
            ||| || |||||||||||||| ||||| ||||| ||||||||||||||||||||||||||
Sbjct  504  AGGATTTGGAAATTGATTAGTCCCTATCATATTGGGTGCCCCAGATATAGCATTTCCACG  445

Query  241  AATAAATAATATAAGATTTTGACTTTTACCCCCCTCATTATTTTTATTGATTAACTCTTC  300
            |||||||||||||||||||||||||||||| || ||| |||||||||| |||||||||||
Sbjct  444  AATAAATAATATAAGATTTTGACTTTTACCTCCTTCACTATTTTTATTAATTAACTCTTC  385

Query  301  ATTAATTGAATCCGGAGCTGGAACAGGATGAACAGTTTATCCCCCTCTATCTTCAAATTT  360
            |||||||||||| |||||||| ||||||||||||||||| || |||||||||||||||||
Sbjct  384  ATTAATTGAATCTGGAGCTGGTACAGGATGAACAGTTTACCCTCCTCTATCTTCAAATTT  325

Query  361  ATCACATTATGGTCCATCAGTAGATTTAGCTATTTTTTCTCTTCACCTTGCTGGTGCTTC  420
            |||||||||||| || ||||||||||||||||||||||||||||| ||||||||||||||
Sbjct  324  ATCACATTATGGCCCTTCAGTAGATTTAGCTATTTTTTCTCTTCATCTTGCTGGTGCTTC  265

Query  421  TTCAATTTTAGGTGCAATTAACTTCATCACAACTATTGTAAATATACGATCAATTGGAAT  480
            |||||||||||||||||||||||| || |||||||||||||| |||||||||||||||||
Sbjct  264  TTCAATTTTAGGTGCAATTAACTTTATTACAACTATTGTAAACATACGATCAATTGGAAT  205

Query  481  AACAATAGAACGAATACCATTATTTGTTTGATCTGTTTTAATTACAGCTATTTTACTACT  540
            |||||||||||| |||||||||||||||||||||||||||||||||||||||||| ||||
Sbjct  204  AACAATAGAACGTATACCATTATTTGTTTGATCTGTTTTAATTACAGCTATTTTATTACT  145

Query  541  TCTATCTTTACCTGTATTAGCAGGTGCCATTACAATATTATTAACTGATCGAAACTTTAA  600
            | |||||||||||||||||||||||||||||||||||||| |||||||||||||||||||
Sbjct  144  TTTATCTTTACCTGTATTAGCAGGTGCCATTACAATATTGTTAACTGATCGAAACTTTAA  85

Query  601  CACATCATTTTTTGATCCTTCAGGAGGAGGAGATCCCATTTTATATCAACACTTATTT  658
             ||||||||||||||||||||||| ||||||||||| |||||||||||||| ||||||
Sbjct  84   TACATCATTTTTTGATCCTTCAGGGGGAGGAGATCCAATTTTATATCAACATTTATTT  27
```

>Rhipicephalus sanguineus isolate Arizona-2 cytochrome c oxidase subunit 1 (COI) gene, partial cds; mitochondrial
Sequence ID: MN593343.1 Length: 710
Range 1: 27 to 684

```
Score:1061 bits(574), Expect:0.0,
Identities:630/658(96%),  Gaps:0/658(0%), Strand: Plus/Minus

Query  1    AACAATATATTTAATTTTTGGAGCATGATCAGGTATATTAGGACTTAGTATAAGAATATT  60
            ||||||||||||||||||||||||||||||||||||||||||||||||||||||||||||
Sbjct  684  AACAATATATTTAATTTTTGGAGCATGATCAGGTATATTAGGACTTAGTATAAGAATATT  625

Query  61   AATTCGTATAGAATTAGGACAACCTGGAACTTTAATTGGAAATGATCAGATTTATAATGT  120
            ||||||||||||||||||||||||||||||||||||||||||||||||||||||||||||
Sbjct  624  AATTCGTATAGAATTAGGACAACCTGGAACTTTAATTGGAAATGATCAGATTTATAATGT  565

Query  121  AATCGTAACAGCACATGCATTTATTATAATTTTCTTTATAGTTATACCAATCATAATCGG  180
            ||||||||||||||||||||||||||||||||| ||||||||||||||||||||||||||
Sbjct  564  AATCGTAACAGCACATGCATTTATTATAATTTTTTTTATAGTTATACCAATCATAATCGG  505

Query  181  AGGGTTCGGAAATTGATTAGTTCCTATTATATTAGGTGCCCCAGATATAGCATTTCCACG  240
            ||| || |||||||||||||| ||||| ||||| ||||||||||||||||||||||||||
Sbjct  504  AGGATTTGGAAATTGATTAGTCCCTATCATATTGGGTGCCCCAGATATAGCATTTCCACG  445

Query  241  AATAAATAATATAAGATTTTGACTTTTACCCCCCTCATTATTTTTATTGATTAACTCTTC  300
            |||||||||||||||||||||||||||||| || ||| |||||||||| |||||||||||
Sbjct  444  AATAAATAATATAAGATTTTGACTTTTACCTCCTTCACTATTTTTATTAATTAACTCTTC  385

Query  301  ATTAATTGAATCCGGAGCTGGAACAGGATGAACAGTTTATCCCCCTCTATCTTCAAATTT  360
            |||||||||||| |||||||| ||||||||||||||||| || |||||||||||||||||
Sbjct  384  ATTAATTGAATCTGGAGCTGGTACAGGATGAACAGTTTACCCTCCTCTATCTTCAAATTT  325

Query  361  ATCACATTATGGTCCATCAGTAGATTTAGCTATTTTTTCTCTTCACCTTGCTGGTGCTTC  420
            |||||||||||| || ||||||||||||||||||||||||||||| ||||||||||||||
Sbjct  324  ATCACATTATGGCCCTTCAGTAGATTTAGCTATTTTTTCTCTTCATCTTGCTGGTGCTTC  265

Query  421  TTCAATTTTAGGTGCAATTAACTTCATCACAACTATTGTAAATATACGATCAATTGGAAT  480
            |||||||||||||||||||||||| || |||||||||||||| |||||||||||||||||
Sbjct  264  TTCAATTTTAGGTGCAATTAACTTTATTACAACTATTGTAAACATACGATCAATTGGAAT  205

Query  481  AACAATAGAACGAATACCATTATTTGTTTGATCTGTTTTAATTACAGCTATTTTACTACT  540
            |||||||||||| |||||||||||||||||||||||||||||||||||||||||| ||||
Sbjct  204  AACAATAGAACGTATACCATTATTTGTTTGATCTGTTTTAATTACAGCTATTTTATTACT  145

Query  541  TCTATCTTTACCTGTATTAGCAGGTGCCATTACAATATTATTAACTGATCGAAACTTTAA  600
            | ||||||||||||||||||||||||||||||||||||| ||||||||||||||||||||
Sbjct  144  TTTATCTTTACCTGTATTAGCAGGTGCCATTACAATATTGTTAACTGATCGAAACTTTAA  85

Query  601  CACATCATTTTTTGATCCTTCAGGAGGAGGAGATCCCATTTTATATCAACACTTATTT  658
             |||||||||||||||||||||||| ||||||||||| |||||||||||||| ||||||
Sbjct  84   TACATCATTTTTTGATCCTTCAGGGGGAGGAGATCCAATTTTATATCAACATTTATTT  27
```

>Rhipicephalus sanguineus isolate 11N cytochrome oxidase subunit I (COX1) gene, partial cds; mitochondrial
Sequence ID: MN585197.1 Length: 703

```
Range 1: 22 to 679

Score:1061 bits(574), Expect:0.0,
Identities:630/658(96%),  Gaps:0/658(0%), Strand: Plus/Minus

Query  1    AACAATATATTTAATTTTTGGAGCATGATCAGGTATATTAGGACTTAGTATAAGAATATT  60
            ||||||||||||||||||||||||||||||||||||||||||||||||||||||||||||
Sbjct  679  AACAATATATTTAATTTTTGGAGCATGATCAGGTATATTAGGACTTAGTATAAGAATATT  620

Query  61   AATTCGTATAGAATTAGGACAACCTGGAACTTTAATTGGAAATGATCAGATTTATAATGT  120
            ||||||||||||||||||||||||||||||||||||||||||||||||||||||||||||
Sbjct  619  AATTCGTATAGAATTAGGACAACCTGGAACTTTAATTGGAAATGATCAGATTTATAATGT  560

Query  121  AATCGTAACAGCACATGCATTTATTATAATTTTCTTTATAGTTATACCAATCATAATCGG  180
            ||||||||||||||||||||||||||||||||| ||||||||||||||||||||||||||
Sbjct  559  AATCGTAACAGCACATGCATTTATTATAATTTTTTTTATAGTTATACCAATCATAATCGG  500

Query  181  AGGGTTCGGAAATTGATTAGTTCCTATTATATTAGGTGCCCCAGATATAGCATTTCCACG  240
            ||| || |||||||||||||| ||||| ||||| ||||||||||||||||||||||||||
Sbjct  499  AGGATTTGGAAATTGATTAGTCCCTATCATATTGGGTGCCCCAGATATAGCATTTCCACG  440

Query  241  AATAAATAATATAAGATTTTGACTTTTACCCCCCTCATTATTTTTATTGATTAACTCTTC  300
            |||||||||||||||||||||||||||||| || ||| |||||||||| |||||||||||
Sbjct  439  AATAAATAATATAAGATTTTGACTTTTACCTCCTTCACTATTTTTATTAATTAACTCTTC  380

Query  301  ATTAATTGAATCCGGAGCTGGAACAGGATGAACAGTTTATCCCCCTCTATCTTCAAATTT  360
            |||||||||||| |||||||| ||||||||||||||||| || |||||||||||||||||
Sbjct  379  ATTAATTGAATCTGGAGCTGGTACAGGATGAACAGTTTACCCTCCTCTATCTTCAAATTT  320

Query  361  ATCACATTATGGTCCATCAGTAGATTTAGCTATTTTTTCTCTTCACCTTGCTGGTGCTTC  420
            |||||||||||| || ||||||||||||||||||||||||||||| ||||||||||||||
Sbjct  319  ATCACATTATGGCCCTTCAGTAGATTTAGCTATTTTTTCTCTTCATCTTGCTGGTGCTTC  260

Query  421  TTCAATTTAGGTGCAATTAACTTCATCACAACTATTGTAAATATACGATCAATTGGAAT  480
            ||||||||||||||||||||||| || |||||||||||||| ||||||||||||||||||
Sbjct  259  TTCAATTTAGGTGCAATTAACTTTATTACAACTATTGTAAACATACGATCAATTGGAAT  200

Query  481  AACAATAGAACGAATACCATTATTTGTTTGATCTGTTTTAATTACAGCTATTTTACTACT  540
            |||||||||||| |||||||||||||||||||||||||||||||||||||||||| ||||
Sbjct  199  AACAATAGAACGTATACCATTATTTGTTTGATCTGTTTTAATTACAGCTATTTTATTACT  140

Query  541  TCTATCTTTACCTGTATTAGCAGGTGCCATTACAATATTATTAACTGATCGAAACTTTAA  600
            | |||||||||||||||||||||||||||||||||||||| |||||||||||||||||||
Sbjct  139  TTTATCTTTACCTGTATTAGCAGGTGCCATTACAATATTGTTAACTGATCGAAACTTTAA  80

Query  601  CACATCATTTTTTGATCCTTCAGGAGGAGGAGATCCCATTTTATATCAACACTTATTT  658
             ||||||||||||||||||||||| ||||||||||| |||||||||||||| ||||||
Sbjct  79   TACATCATTTTTTGATCCTTCAGGGGGAGGAGATCCAATTTTATATCAACATTTATTT  22
```

>Rhipicephalus sanguineus cytochrome c oxidase I (COI) gene, partial cds;
mitochondrial gene for mitochondrial product

Sequence ID: AF132839.1 Length: 793
Range 1: 42 to 699

Score:1061 bits(574), Expect:0.0,
Identities:630/658(96%), Gaps:0/658(0%), Strand: Plus/Plus

```
Query  1    AACAATATATTTAATTTTTGGAGCATGATCAGGTATATTAGGACTTAGTATAAGAATATT  60
            ||||||||||||||||||||||||||||||||||||||||||||||||||||||||||||
Sbjct  42   AACAATATATTTAATTTTTGGAGCATGATCAGGTATATTAGGACTTAGTATAAGAATATT  101

Query  61   AATTCGTATAGAATTAGGACAACCTGGAACTTTAATTGGAAATGATCAGATTTATAATGT  120
            ||||||||||||||||||||||||||||||||||||||||||||||||||||||||||||
Sbjct  102  AATTCGTATAGAATTAGGACAACCTGGAACTTTAATTGGAAATGATCAGATTTATAATGT  161

Query  121  AATCGTAACAGCACATGCATTTATTATAATTTTCTTTATAGTTATACCAATCATAATCGG  180
            ||||||||||||||||||||||||||||||||| ||||||||||||||||||||||||||
Sbjct  162  AATCGTAACAGCACATGCATTTATTATAATTTTTTTTATAGTTATACCAATCATAATCGG  221

Query  181  AGGGTTCGGAAATTGATTAGTTCCTATTATATTAGGTGCCCCAGATATAGCATTTCCACG  240
            ||| || |||||||||||||| ||||| ||||| ||||||||||||||||||||||||||
Sbjct  222  AGGATTTGGAAATTGATTAGTCCCTATCATATTGGGTGCCCCAGATATAGCATTTCCACG  281

Query  241  AATAAATAATATAAGATTTTGACTTTTACCCCCCTCATTATTTTTATTGATTAACTCTTC  300
            |||||||||||||||||||||||||||||| || ||| |||||||||| |||||||||||
Sbjct  282  AATAAATAATATAAGATTTTGACTTTTACCTCCTTCACTATTTTTATTAATTAACTCTTC  341

Query  301  ATTAATTGAATCCGGAGCTGGAACAGGATGAACAGTTTATCCCCCTCTATCTTCAAATTT  360
            |||||||||||| |||||||| ||||||||||||||||| || |||||||||||||||||
Sbjct  342  ATTAATTGAATCTGGAGCTGGTACAGGATGAACAGTTTACCCTCCTCTATCTTCAAATTT  401

Query  361  ATCACATTATGGTCCATCAGTAGATTTAGCTATTTTTTCTCTTCACCTTGCTGGTGCTTC  420
            |||||||||||| || ||||||||||||||||||||||||||||| ||||||||||||||
Sbjct  402  ATCACATTATGGCCCTTCAGTAGATTTAGCTATTTTTTCTCTTCATCTTGCTGGTGCTTC  461

Query  421  TTCAATTTAGGTGCAATTAACTTCATCACAACTATTGTAAATATACGATCAATTGGAAT  480
            |||||||||||||||||||||||| || |||||||||||||| |||||||||||||||||
Sbjct  462  TTCAATTTAGGTGCAATTAACTTTATTACAACTATTGTAAACATACGATCAATTGGAAT  521

Query  481  AACAATAGAACGAATACCATTATTTGTTTGATCTGTTTTAATTACAGCTATTTTACTACT  540
            |||||||||||| |||||||||||||||||||||||||||||||||||||||||| ||||
Sbjct  522  AACAATAGAACGTATACCATTATTTGTTTGATCTGTTTTAATTACAGCTATTTTATTACT  581

Query  541  TCTATCTTTACCTGTATTAGCAGGTGCCATTACAATATTATTAACTGATCGAAACTTTAA  600
            | |||||||||||||||||||||||||||||||||||||| |||||||||||||||||||
Sbjct  582  TTTATCTTTACCTGTATTAGCAGGTGCCATTACAATATTGTTAACTGATCGAAACTTTAA  641

Query  601  CACATCATTTTTTGATCCTTCAGGAGGAGGAGATCCCATTTTATATCAACACTTATTT  658
             ||||||||||||||||||||||| ||||||||||| |||||||||||||| ||||||
Sbjct  642  TACATCATTTTTTGATCCTTCAGGGGGAGGAGATCCAATTTTATATCAACATTTATTT  699
```

>Rhipicephalus sanguineus sensu lato 'temperate lineage' isolate S56 cytochrome

c oxidase subunit I (COI) gene, partial cds; mitochondrial
Sequence ID: MF426017.1 Length: 680
Range 1: 11 to 668

Score:1055 bits(571), Expect:0.0,
Identities:629/658(96%), Gaps:0/658(0%), Strand: Plus/Plus

```
Query  1    AACAATATATTTAATTTTTGGAGCATGATCAGGTATATTAGGACTTAGTATAAGAATATT  60
            ||||||||||||||||||||||||||||||||||||||||||||||||||||||||||||
Sbjct  11   AACAATATATTTAATTTTTGGAGCATGATCAGGTATATTAGGACTTAGTATAAGAATATT  70

Query  61   AATTCGTATAGAATTAGGACAACCTGGAACTTTAATTGGAAATGATCAGATTTATAATGT  120
            ||||||||||||||||||||||||||||||||||||||||||||||||||||||||||||
Sbjct  71   AATTCGTATAGAATTAGGACAACCTGGAACTTTAATTGGAAATGATCAGATTTATAATGT  130

Query  121  AATCGTAACAGCACATGCATTTATTATAATTTTCTTTATAGTTATACCAATCATAATCGG  180
            ||||||||||||||||||||||||||||||||| ||||||||||||||||||||||||||
Sbjct  131  AATCGTAACAGCACATGCATTTATTATAATTTTTTTTATAGTTATACCAATCATAATCGG  190

Query  181  AGGGTTCGGAAATTGATTAGTTCCTATTATATTAGGTGCCCCAGATATAGCATTTCCACG  240
            ||| || |||||||||||||| ||||| ||||| ||||||||||||||||||||||||||
Sbjct  191  AGGATTTGGAAATTGATTAGTCCCTATCATATTGGGTGCCCCAGATATAGCATTTCCACG  250

Query  241  AATAAATAATATAAGATTTTGACTTTTACCCCCCTCATTATTTTTATTGATTAACTCTTC  300
            |||||||||||||||||||||||||||||| || ||| |||||||||| |||||||||||
Sbjct  251  AATAAATAATATAAGATTTTGACTTTTACCTCCTTCACTATTTTTATTAATTAACTCTTC  310

Query  301  ATTAATTGAATCCGGAGCTGGAACAGGATGAACAGTTTATCCCCCTCTATCTTCAAATTT  360
            |||||||||||| |||||||| |||||||||||||||||||| |||||||||||||||||
Sbjct  311  ATTAATTGAATCTGGAGCTGGTACAGGATGAACAGTTTATCCTCCTCTATCTTCAAATTT  370

Query  361  ATCACATTATGGTCCATCAGTAGATTTAGCTATTTTTTCTCTTCACCTTGCTGGTGCTTC  420
            |||||||||||| || ||||||||||||||||| ||||||||||| ||||||||||||||
Sbjct  371  ATCACATTATGGCCCTTCAGTAGATTTAGCTATCTTTTCTCTTCATCTTGCTGGTGCTTC  430

Query  421  TTCAATTTTAGGTGCAATTAACTTCATCACAACTATTGTAAATATACGATCAATTGGAAT  480
            |||||||||||| ||||||||||| || |||||||||||||| |||||||||||||||||
Sbjct  431  TTCAATTTTAGGAGCAATTAACTTTATTACAACTATTGTAAACATACGATCAATTGGAAT  490

Query  481  AACAATAGAACGAATACCATTATTTGTTTGATCTGTTTTAATTACAGCTATTTTACTACT  540
            |||||||||||| |||||||||||||||||||||||||||||||||||||||||| ||||
Sbjct  491  AACAATAGAACGTATACCATTATTTGTTTGATCTGTTTTAATTACAGCTATTTTATTACT  550

Query  541  TCTATCTTTACCTGTATTAGCAGGTGCCATTACAATATTATTAACTGATCGAAACTTTAA  600
            | ||||||||||||||||||||||||||||||||||||| ||||||||||||||||||||
Sbjct  551  TTTATCTTTACCTGTATTAGCAGGTGCCATTACAATATTGTTAACTGATCGAAACTTTAA  610

Query  601  CACATCATTTTTTGATCCTTCAGGAGGAGGAGATCCCATTTTATATCAACACTTATTT  658
             ||||||||||||||||||||||| ||||||||||| |||||||||||||| ||||||
Sbjct  611  TACATCATTTTTTGATCCTTCAGGGGGAGGAGATCCAATTTTATATCAACATTTATTT  668
```

>Rhipicephalus sanguineus sensu lato 'temperate lineage' isolate S32 cytochrome c oxidase subunit I (COI) gene, partial cds; mitochondrial
Sequence ID: MF426008.1 Length: 690
Range 1: 14 to 671

Score:1055 bits(571), Expect:0.0,
Identities:629/658(96%), Gaps:0/658(0%), Strand: Plus/Plus

```
Query  1    AACAATATATTTAATTTTTGGAGCATGATCAGGTATATTAGGACTTAGTATAAGAATATT  60
            ||||||||||||||||||||||||||||||||||||||||||||||||||||||||||||
Sbjct  14   AACAATATATTTAATTTTTGGAGCATGATCAGGTATATTAGGACTTAGTATAAGAATATT  73

Query  61   AATTCGTATAGAATTAGGACAACCTGGAACTTTAATTGGAAATGATCAGATTTATAATGT  120
            ||||||||||||||||||||||||||||||||||||||||||||||||||||||||||||
Sbjct  74   AATTCGTATAGAATTAGGACAACCTGGAACTTTAATTGGAAATGATCAGATTTATAATGT  133

Query  121  AATCGTAACAGCACATGCATTTATTATAATTTTCTTTATAGTTATACCAATCATAATCGG  180
            ||||||||||||||||||||||||||||||||| ||||||||||||||||||||||||||
Sbjct  134  AATCGTAACAGCACATGCATTTATTATAATTTTTTTTATAGTTATACCAATCATAATCGG  193

Query  181  AGGGTTCGGAAATTGATTAGTTCCTATTATATTAGGTGCCCCAGATATAGCATTTCCACG  240
            ||| || |||||||||||||| ||||| ||||| ||||||||||||||||||||||||||
Sbjct  194  AGGATTTGGAAATTGATTAGTCCCTATCATATTGGGTGCCCCAGATATAGCATTTCCACG  253

Query  241  AATAAATAATATAAGATTTTGACTTTTACCCCCCTCATTATTTTTATTGATTAACTCTTC  300
            |||||||||||||||||||||||||||||| || ||| |||||||||| |||||||||||
Sbjct  254  AATAAATAATATAAGATTTTGACTTTTACCTCCTTCACTATTTTTATTAATTAACTCTTC  313

Query  301  ATTAATTGAATCCGGAGCTGGAACAGGATGAACAGTTTATCCCCCTCTATCTTCAAATTT  360
            |||||||||||| |||||||| ||||||||||||||||| || |||||||||||||||||
Sbjct  314  ATTAATTGAATCTGGAGCTGGTACAGGATGAACAGTTTACCCTCCTCTATCTTCAAATTT  373

Query  361  ATCACATTATGGTCCATCAGTAGATTTAGCTATTTTTTCTCTTCACCTTGCTGGTGCTTC  420
            |||||||||||| || ||||||||||||||||| ||||||||||| ||||||||||||||
Sbjct  374  ATCACATTATGGCCCTTCAGTAGATTTAGCTATCTTTTCTCTTCATCTTGCTGGTGCTTC  433

Query  421  TTCAATTTTAGGTGCAATTAACTTCATCACAACTATTGTAAATATACGATCAATTGGAAT  480
            ||||||||||||||||||||||| || ||||||||||||||| |||||||||||||||||
Sbjct  434  TTCAATTTTAGGTGCAATTAACTTTATTACAACTATTGTAAACATACGATCAATTGGAAT  493

Query  481  AACAATAGAACGAATACCATTATTTGTTTGATCTGTTTTAATTACAGCTATTTTACTACT  540
            |||||||||||| |||||||||||||||||||||||||||||||||||||||||| ||||
Sbjct  494  AACAATAGAACGTATACCATTATTTGTTTGATCTGTTTTAATTACAGCTATTTTATTACT  553

Query  541  TCTATCTTTACCTGTATTAGCAGGTGCCATTACAATATTATTAACTGATCGAAACTTTAA  600
            | ||||||||||||||||||||||||||||||||||||| ||||||||||||||||||||
Sbjct  554  TTTATCTTTACCTGTATTAGCAGGTGCCATTACAATATTGTTAACTGATCGAAACTTTAA  613

Query  601  CACATCATTTTTTGATCCTTCAGGAGGAGGAGATCCCATTTTATATCAACACTTATTT  658
             ||||||||||||||||||||||| ||||||||||| |||||||||||||| ||||||
Sbjct  614  TACATCATTTTTTGATCCTTCAGGGGGAGGAGATCCAATTTTATATCAACATTTATTT  671
```

```
>Rhipicephalus sanguineus sensu lato 'temperate lineage' isolate CR1570
cytochrome c oxidase subunit I (COI) gene, partial cds; mitochondrial
Sequence ID: MF426001.1 Length: 684
Range 1: 14 to 671

Score:1055 bits(571), Expect:0.0,
Identities:629/658(96%),  Gaps:0/658(0%), Strand: Plus/Plus

Query  1    AACAATATATTTAATTTTTGGAGCATGATCAGGTATATTAGGACTTAGTATAAGAATATT  60
            ||||||||||||||||||||||||||||||||||||||||||||||||||||||||||||
Sbjct  14   AACAATATATTTAATTTTTGGAGCATGATCAGGTATATTAGGACTTAGTATAAGAATATT  73

Query  61   AATTCGTATAGAATTAGGACAACCTGGAACTTTAATTGGAAATGATCAGATTTATAATGT  120
            ||||||||||||||||||||||||||||||||||||||||||||||||||||||||||||
Sbjct  74   AATTCGTATAGAATTAGGACAACCTGGAACTTTAATTGGAAATGATCAGATTTATAATGT  133

Query  121  AATCGTAACAGCACATGCATTTATTATAATTTCTTTATAGTTATACCAATCATAATCGG  180
            |||||||||||||||||||||||||||||||| |||||||||||||||||||||||||||
Sbjct  134  AATCGTAACAGCACATGCATTTATTATAATTTTTTTATAGTTATACCAATCATAATCGG  193

Query  181  AGGGTTCGGAAATTGATTAGTTCCTATTATATTAGGTGCCCCAGATATAGCATTTCCACG  240
            ||| || |||||||||||||| ||||| ||||| ||||||||||||||||||||||||||
Sbjct  194  AGGATTTGGAAATTGATTAGTCCCTATCATATTGGGTGCCCCAGATATAGCATTTCCACG  253

Query  241  AATAAATAATATAAGATTTTGACTTTTACCCCCCTCATTATTTTTATTGATTAACTCTTC  300
            |||||||||||||||||||||||||||||| || ||| |||||||||| |||||||||||
Sbjct  254  AATAAATAATATAAGATTTTGACTTTTACCTCCTTCACTATTTTTATTAATTAACTCTTC  313

Query  301  ATTAATTGAATCCGGAGCTGGAACAGGATGAACAGTTTATCCCCCTCTATCTTCAAATTT  360
            |||||||||||| |||||||| ||||||||||||||||| || |||||||||||||||||
Sbjct  314  ATTAATTGAATCTGGAGCTGGTACAGGATGAACAGTTTACCCTCCTCTATCTTCAAATTT  373

Query  361  ATCACATTATGGTCCATCAGTAGATTTAGCTATTTTTTCTCTTCACCTTGCTGGTGCTTC  420
            |||||||||||| || ||||||||||||||||| ||||||||||| ||||||||||||||
Sbjct  374  ATCACATTATGGCCCTTCAGTAGATTTAGCTATCTTTTCTCTTCATCTTGCTGGTGCTTC  433

Query  421  TTCAATTTAGGTGCAATTAACTTCATCACAACTATTGTAAATATACGATCAATTGGAAT  480
            ||||||||||||||||||||||| || |||||||||||||| |||||||||||||||||
Sbjct  434  TTCAATTTAGGTGCAATTAACTTTATTACAACTATTGTAAACATACGATCAATTGGAAT  493

Query  481  AACAATAGAACGAATACCATTATTTGTTTGATCTGTTTTAATTACAGCTATTTTACTACT  540
            |||||||||||| |||||||||||||||||||||||||||||||||||||||||| ||||
Sbjct  494  AACAATAGAACGTATACCATTATTTGTTTGATCTGTTTTAATTACAGCTATTTTATTACT  553

Query  541  TCTATCTTTACCTGTATTAGCAGGTGCCATTACAATATTATTAACTGATCGAAACTTTAA  600
            | ||||||||||||||||||||||||||||||||||||| ||||||||||||||||||||
Sbjct  554  TTTATCTTTACCTGTATTAGCAGGTGCCATTACAATATTGTTAACTGATCGAAACTTTAA  613

Query  601  CACATCATTTTTTGATCCTTCAGGAGGAGGAGATCCCATTTTATATCAACACTTATTT  658
             ||||||||||||||||||||||| ||||||||||| |||||||||||||| ||||||
Sbjct  614  TACATCATTTTTTGATCCTTCAGGGGGAGGAGATCCAATTTTATATCAACATTTATTT  671
```

>Rhipicephalus sanguineus sensu lato 'temperate lineage' isolate CR1553 cytochrome c oxidase subunit I (COI) gene, partial cds; mitochondrial
Sequence ID: MF425998.1 Length: 689
Range 1: 20 to 677

Score:1055 bits(571), Expect:0.0,
Identities:629/658(96%), Gaps:0/658(0%), Strand: Plus/Plus

```
Query  1    AACAATATATTTAATTTTTGGAGCATGATCAGGTATATTAGGACTTAGTATAAGAATATT  60
            ||||||||||||||||||||||||||||||||||||||||||||||||||||||||||||
Sbjct  20   AACAATATATTTAATTTTTGGAGCATGATCAGGTATATTAGGACTTAGTATAAGAATATT  79

Query  61   AATTCGTATAGAATTAGGACAACCTGGAACTTTAATTGGAAATGATCAGATTTATAATGT  120
            ||||||||||||||||||||||||||||||||||||||||||||||||||||||||||||
Sbjct  80   AATTCGTATAGAATTAGGACAACCTGGAACTTTAATTGGAAATGATCAGATTTATAATGT  139

Query  121  AATCGTAACAGCACATGCATTTATTATAATTTTCTTTATAGTTATACCAATCATAATCGG  180
            ||||||||||||||||||||||||||||||||| ||||||||||||||||||||||||||
Sbjct  140  AATCGTAACAGCACATGCATTTATTATAATTTTTTTTATAGTTATACCAATCATAATCGG  199

Query  181  AGGGTTCGGAAATTGATTAGTTCCTATTATATTAGGTGCCCCAGATATAGCATTTCCACG  240
            ||| || |||||||||||||| ||||| ||||||||||||||||||||||||||||||||
Sbjct  200  AGGATTTGGAAATTGATTAGTCCCTATCATATTAGGTGCCCCAGATATAGCATTTCCACG  259

Query  241  AATAAATAATATAAGATTTTGACTTTTACCCCCCTCATTATTTTTATTGATTAACTCTTC  300
            |||||||||||||||||||||||||||||| || ||| |||||||||| |||||||||||
Sbjct  260  AATAAATAATATAAGATTTTGACTTTTACCTCCTTCACTATTTTTATTAATTAACTCTTC  319

Query  301  ATTAATTGAATCCGGAGCTGGAACAGGATGAACAGTTTATCCCCCTCTATCTTCAAATTT  360
            |||||||||||| |||||||| ||||||||||||||||| || |||||||||||||||||
Sbjct  320  ATTAATTGAATCTGGAGCTGGTACAGGATGAACAGTTTACCCTCCTCTATCTTCAAATTT  379

Query  361  ATCACATTATGGTCCATCAGTAGATTTAGCTATTTTTTCTCTTCACCTTGCTGGTGCTTC  420
            |||||||||||| || ||||||||||||||||| ||||||||||| ||||||||||||||
Sbjct  380  ATCACATTATGGCCCTTCAGTAGATTTAGCTATCTTTTCTCTTCATCTTGCTGGTGCTTC  439

Query  421  TTCAATTTTAGGTGCAATTAACTTCATCACAACTATTGTAAATATACGATCAATTGGAAT  480
            |||||||||||||||||||||||| || ||||||||||||||| ||||||||||||||||
Sbjct  440  TTCAATTTTAGGTGCAATTAACTTTATTACAACTATTGTAAACATACGATCAATTGGAAT  499

Query  481  AACAATAGAACGAATACCATTATTTGTTTGATCTGTTTTAATTACAGCTATTTTACTACT  540
            |||||||||||| |||||||||||||||||||||||||||||||||||||||||| ||||
Sbjct  500  AACAATAGAACGTATACCATTATTTGTTTGATCTGTTTTAATTACAGCTATTTTATTACT  559

Query  541  TCTATCTTTACCTGTATTAGCAGGTGCCATTACAATATTATTAACTGATCGAAACTTTAA  600
              ||||||||||||||||||||||||||||||||||||| ||||||||||||||||||||
Sbjct  560  CTTATCTTTACCTGTATTAGCAGGTGCCATTACAATATTGTTAACTGATCGAAACTTTAA  619

Query  601  CACATCATTTTTTGATCCTTCAGGAGGAGGAGATCCCATTTTATATCAACACTTATTT  658
             ||||||||||||||||||||||| ||||||||||| |||||||||||||| ||||||
Sbjct  620  TACATCATTTTTTGATCCTTCAGGGGGAGGAGATCCAATTTTATATCAACATTTATTT  677
```

```
>Rhipicephalus sanguineus sensu lato 'temperate lineage' isolate CR1551
cytochrome c oxidase subunit I (COI) gene, partial cds; mitochondrial
Sequence ID: MF425997.1 Length: 695
Range 1: 22 to 679

Score:1055 bits(571), Expect:0.0,
Identities:629/658(96%),  Gaps:0/658(0%), Strand: Plus/Plus

Query  1    AACAATATATTTAATTTTTGGAGCATGATCAGGTATATTAGGACTTAGTATAAGAATATT  60
            ||||||||||||||||||||||||||||||||||||||||||||||||||||||||||||
Sbjct  22   AACAATATATTTAATTTTTGGAGCATGATCAGGTATATTAGGACTTAGTATAAGAATATT  81

Query  61   AATTCGTATAGAATTAGGACAACCTGGAACTTTAATTGGAAATGATCAGATTTATAATGT  120
            ||||||||||||||||||||||||||||||||||||||||||||||||||||||||||||
Sbjct  82   AATTCGTATAGAATTAGGACAACCTGGAACTTTAATTGGAAATGATCAGATTTATAATGT  141

Query  121  AATCGTAACAGCACATGCATTTATTATAATTTTCTTTATAGTTATACCAATCATAATCGG  180
            ||||||||||||||||||||||||||||||||| ||||||||||||||||||||||||||
Sbjct  142  AATCGTAACAGCACATGCATTTATTATAATTTTTTTTATAGTTATACCAATCATAATCGG  201

Query  181  AGGGTTCGGAAATTGATTAGTTCCTATTATATTAGGTGCCCCAGATATAGCATTTCCACG  240
            ||| || |||||||||||||| ||||| ||||| ||||||||||||||||||||||||||
Sbjct  202  AGGATTTGGAAATTGATTAGTCCCTATCATATTGGGTGCCCCAGATATAGCATTTCCACG  261

Query  241  AATAAATAATATAAGATTTTGACTTTTACCCCCCTCATTATTTTTATTGATTAACTCTTC  300
            |||||||||||||||||||||||||||||| || ||| |||||||||| |||||||||||
Sbjct  262  AATAAATAATATAAGATTTTGACTTTTACCTCCTTCACTATTTTTATTAATTAACTCTTC  321

Query  301  ATTAATTGAATCCGGAGCTGGAACAGGATGAACAGTTTATCCCCCTCTATCTTCAAATTT  360
            |||||||||||| |||||||| ||||||||||||||||| || |||||||||||||||||
Sbjct  322  ATTAATTGAATCTGGAGCTGGTACAGGATGAACAGTTTACCCTCCTCTATCTTCAAATTT  381

Query  361  ATCACATTATGGTCCATCAGTAGATTTAGCTATTTTTTCTCTTCACCTTGCTGGTGCTTC  420
            |||||||||||| || ||||||||||||||||| ||||||||||| ||||||||||||||
Sbjct  382  ATCACATTATGGCCCTTCAGTAGATTTAGCTATCTTTTCTCTTCATCTTGCTGGTGCTTC  441

Query  421  TTCAATTTTAGGTGCAATTAACTTCATCACAACTATTGTAAATATACGATCAATTGGAAT  480
            |||||||||||||||||||||||| || ||||||||||||||| ||||||||||||||||
Sbjct  442  TTCAATTTTAGGTGCAATTAACTTTATTACAACTATTGTAAACATACGATCAATTGGAAT  501

Query  481  AACAATAGAACGAATACCATTATTTGTTTGATCTGTTTTAATTACAGCTATTTTACTACT  540
            |||||||||||| |||||||||||||||||||||||||||||||||||||||||| ||||
Sbjct  502  AACAATAGAACGTATACCATTATTTGTTTGATCTGTTTTAATTACAGCTATTTTATTACT  561

Query  541  TCTATCTTTACCTGTATTAGCAGGTGCCATTACAATATTATTAACTGATCGAAACTTTAA  600
            | ||||||||||||||||||||||||||||||||||||| ||||||||||||||||||||
Sbjct  562  TTTATCTTTACCTGTATTAGCAGGTGCCATTACAATATTGTTAACTGATCGAAACTTTAA  621

Query  601  CACATCATTTTTTGATCCTTCAGGAGGAGGAGATCCCATTTTATATCAACACTTATTT  658
             ||||||||||||||||||||||| ||||||||||| |||||||||||||| ||||||
Sbjct  622  TACATCATTTTTTGATCCTTCAGGGGGAGGAGATCCAATTTTATATCAACATTTATTT  679
```

```
>Rhipicephalus sanguineus haplotype IV cytochrome oxidase subunit I (COI) gene,
partial cds; mitochondrial
Sequence ID: KU556746.1 Length: 771
Range 1: 90 to 747

Score:1055 bits(571), Expect:0.0,
Identities:629/658(96%),  Gaps:0/658(0%), Strand: Plus/Plus

Query  1    AACAATATATTTAATTTTTGGAGCATGATCAGGTATATTAGGACTTAGTATAAGAATATT  60
            ||||||||||||||||||||||||||||||||||||||||||||||||||||||||||||
Sbjct  90   AACAATATATTTAATTTTTGGAGCATGATCAGGTATATTAGGACTTAGTATAAGAATATT  149

Query  61   AATTCGTATAGAATTAGGACAACCTGGAACTTTAATTGGAAATGATCAGATTTATAATGT  120
            ||||||||||||||||||||||||||||||||||||||||||||||||||||||||||||
Sbjct  150  AATTCGTATAGAATTAGGACAACCTGGAACTTTAATTGGAAATGATCAGATTTATAATGT  209

Query  121  AATCGTAACAGCACATGCATTTATTATAATTTTCTTTATAGTTATACCAATCATAATCGG  180
            ||||||||||||||||||||||||||||||||| ||||||||||||||||||||||||||
Sbjct  210  AATCGTAACAGCACATGCATTTATTATAATTTTTTTTATAGTTATACCAATCATAATCGG  269

Query  181  AGGGTTCGGAAATTGATTAGTTCCTATTATATTAGGTGCCCCAGATATAGCATTTCCACG  240
            ||| || |||||||||||||| ||||| ||||| ||||||||||||||||||||||||||
Sbjct  270  AGGATTTGGAAATTGATTAGTCCCTATCATATTGGGTGCCCCAGATATAGCATTTCCACG  329

Query  241  AATAAATAATATAAGATTTTGACTTTTACCCCCCTCATTATTTTTATTGATTAACTCTTC  300
            |||||||||||||||||||||||||||||| || ||| |||||||||| |||||||||||
Sbjct  330  AATAAATAATATAAGATTTTGACTTTTACCTCCTTCACTATTTTTATTAATTAACTCTTC  389

Query  301  ATTAATTGAATCCGGAGCTGGAACAGGATGAACAGTTTATCCCCCTCTATCTTCAAATTT  360
            |||||||||||| |||||||| |||||||||||||||||||| |||||||||||||||||
Sbjct  390  ATTAATTGAATCTGGAGCTGGTACAGGATGAACAGTTTATCCTCCTCTATCTTCAAATTT  449

Query  361  ATCACATTATGGTCCATCAGTAGATTTAGCTATTTTTTCTCTTCACCTTGCTGGTGCTTC  420
            |||||||||||| || ||||||||||||||||| ||||||||||| ||||||||||||||
Sbjct  450  ATCACATTATGGCCCTTCAGTAGATTTAGCTATCTTTTCTCTTCATCTTGCTGGTGCTTC  509

Query  421  TTCAATTTTAGGTGCAATTAACTTCATCACAACTATTGTAAATATACGATCAATTGGAAT  480
            |||||||||||| ||||||||||| || |||||||||||||| |||||||||||||||||
Sbjct  510  TTCAATTTTAGGAGCAATTAACTTTATTACAACTATTGTAAACATACGATCAATTGGAAT  569

Query  481  AACAATAGAACGAATACCATTATTTGTTTGATCTGTTTTAATTACAGCTATTTTACTACT  540
            |||||||||||| |||||||||||||||||||||||||||||||||||||||||| ||||
Sbjct  570  AACAATAGAACGTATACCATTATTTGTTTGATCTGTTTTAATTACAGCTATTTTATTACT  629

Query  541  TCTATCTTTACCTGTATTAGCAGGTGCCATTACAATATTATTAACTGATCGAAACTTTAA  600
            | ||||||||||||||||||||||||||||||||||||| ||||||||||||||||||||
Sbjct  630  TTTATCTTTACCTGTATTAGCAGGTGCCATTACAATATTGTTAACTGATCGAAACTTTAA  689

Query  601  CACATCATTTTTTGATCCTTCAGGAGGAGGAGATCCCATTTTATATCAACACTTATTT  658
             ||||||||||||||||||||||| ||||||||||| |||||||||||||| ||||||
```

```
Sbjct  690  TACATCATTTTTTGATCCTTCAGGGGGAGGAGATCCAATTTTATATCAACATTTATTT  747
```

Range 2: 30 to 64

Score:58.4 bits(31), Expect:0.001,
Identities:34/35(97%), Gaps:1/35(2%), Strand: Plus/Plus

```
Query  1   AACAATATATTTAATTTTTGGAGCATGATCA-GGT  34
           ||||||||||||||||||||||||||||||| |||
Sbjct  30  AACAATATATTTAATTTTTGGAGCATGATCATGGT  64
```

>Rhipicephalus sanguineus voucher CROBB813 cytochrome oxidase subunit 1 (COI) gene, partial cds; mitochondrial
Sequence ID: MZ305546.1 Length: 658
Range 1: 1 to 658

Score:1055 bits(571), Expect:0.0,
Identities:629/658(96%), Gaps:0/658(0%), Strand: Plus/Plus

```
Query  1    AACAATATATTTAATTTTTGGAGCATGATCAGGTATATTAGGACTTAGTATAAGAATATT  60
            ||||||||||||||||||||||||||||||||||||||||||||||||||||||||||||
Sbjct  1    AACAATATATTTAATTTTTGGAGCATGATCAGGTATATTAGGACTTAGTATAAGAATATT  60

Query  61   AATTCGTATAGAATTAGGACAACCTGGAACTTTAATTGGAAATGATCAGATTTATAATGT  120
            |||||||||||| |||||||||||||||||||||||||||||||||||||||||||||||
Sbjct  61   AATTCGTATAGAGTTAGGACAACCTGGAACTTTAATTGGAAATGATCAGATTTATAATGT  120

Query  121  AATCGTAACAGCACATGCATTTATTATAATTTTCTTTATAGTTATACCAATCATAATCGG  180
            ||||||||||||||||||||||||||||||||| ||||||||||||||||||||||||||
Sbjct  121  AATCGTAACAGCACATGCATTTATTATAATTTTTTTTATAGTTATACCAATCATAATCGG  180

Query  181  AGGGTTCGGAAATTGATTAGTTCCTATTATATTAGGTGCCCCAGATATAGCATTTCCACG  240
            ||| || |||||||||||||| ||||| ||||| ||||||||||||||||||||||||||
Sbjct  181  AGGATTTGGAAATTGATTAGTCCCTATCATATTGGGTGCCCCAGATATAGCATTTCCACG  240

Query  241  AATAAATAATATAAGATTTTGACTTTTACCCCCCTCATTATTTTTATTGATTAACTCTTC  300
            |||||||||||||||||||||||||||||| || ||| |||||||||| |||||||||||
Sbjct  241  AATAAATAATATAAGATTTTGACTTTTACCTCCTTCACTATTTTTATTAATTAACTCTTC  300

Query  301  ATTAATTGAATCCGGAGCTGGAACAGGATGAACAGTTTATCCCCCTCTATCTTCAAATTT  360
            |||||||||||| |||||||| ||||||||||||||||| || |||||||||||||||||
Sbjct  301  ATTAATTGAATCTGGAGCTGGTACAGGATGAACAGTTTACCCTCCTCTATCTTCAAATTT  360

Query  361  ATCACATTATGGTCCATCAGTAGATTTAGCTATTTTTTCTCTTCACCTTGCTGGTGCTTC  420
            |||||||||||| || ||||||||||||||||||||||||||||| ||||||||||||||
Sbjct  361  ATCACATTATGGCCCTTCAGTAGATTTAGCTATTTTTTCTCTTCATCTTGCTGGTGCTTC  420

Query  421  TTCAATTTTAGGTGCAATTAACTTCATCACAACTATTGTAAATATACGATCAATTGGAAT  480
```

```
             |||||||||||||||||||||||| || ||||||||||||||| ||||||||||||||||
Sbjct  421   TTCAATTTTAGGTGCAATTAACTTTATTACAACTATTGTAAACATACGATCAATTGGAAT  480

Query  481   AACAATAGAACGAATACCATTATTTGTTTGATCTGTTTTAATTACAGCTATTTTACTACT  540
             |||||||||||| |||||||||||||||||||||||||||||||||||||||||| ||||
Sbjct  481   AACAATAGAACGTATACCATTATTTGTTTGATCTGTTTTAATTACAGCTATTTTATTACT  540

Query  541   TCTATCTTTACCTGTATTAGCAGGTGCCATTACAATATTATTAACTGATCGAAACTTTAA  600
             | ||||||||||||||||||||||||||||||||||||| ||||||||||||||||||||
Sbjct  541   TTTATCTTTACCTGTATTAGCAGGTGCCATTACAATATTGTTAACTGATCGAAACTTTAA  600

Query  601   CACATCATTTTTTGATCCTTCAGGAGGAGGAGATCCCATTTTATATCAACACTTATTT  658
              ||||||||||||||||||||||| ||||||||||| |||||||||||||| ||||||
Sbjct  601   TACATCATTTTTTGATCCTTCAGGGGGAGGAGATCCAATTTTATATCAACATTTATTT  658
```

>Rhipicephalus sanguineus voucher CROBB810 cytochrome oxidase subunit 1 (COI) gene, partial cds; mitochondrial
Sequence ID: MZ305545.1 Length: 658
Range 1: 1 to 658

Score:1055 bits(571), Expect:0.0,
Identities:629/658(96%), Gaps:0/658(0%), Strand: Plus/Plus

```
Query  1     AACAATATATTTAATTTTTGGAGCATGATCAGGTATATTAGGACTTAGTATAAGAATATT  60
             ||||||||||||||||||||||||||||||||||||||||||||||||||||||||||||
Sbjct  1     AACAATATATTTAATTTTTGGAGCATGATCAGGTATATTAGGACTTAGTATAAGAATATT  60

Query  61    AATTCGTATAGAATTAGGACAACCTGGAACTTTAATTGGAAATGATCAGATTTATAATGT  120
             ||||||||||||||||||||||||||||||||||||||||||||||||||||||||||||
Sbjct  61    AATTCGTATAGAATTAGGACAACCTGGAACTTTAATTGGAAATGATCAGATTTATAATGT  120

Query  121   AATCGTAACAGCACATGCATTTATTATAATTTCTTTATAGTTATACCAATCATAATCGG  180
             |||||||||||||||||||||||||||||||| |||||||||||||||||||||||||||
Sbjct  121   AATCGTAACAGCACATGCATTTATTATAATTTTTTTATAGTTATACCAATCATAATCGG  180

Query  181   AGGGTTCGGAAATTGATTAGTTCCTATTATATTAGGTGCCCCAGATATAGCATTTCCACG  240
             ||| || |||||||||||||| ||||| ||||| ||||||||||||||||||||||||||
Sbjct  181   AGGATTTGGAAATTGATTAGTCCCTATCATATTGGGTGCCCCAGATATAGCATTTCCACG  240

Query  241   AATAAATAATATAAGATTTTGACTTTTACCCCCCTCATTATTTTTATTGATTAACTCTTC  300
             |||||||||||||||||||||||||||||| || ||| |||||||||| ||||| |||||
Sbjct  241   AATAAATAATATAAGATTTTGACTTTTACCTCCTTCACTATTTTTATTAATTAATTCTTC  300

Query  301   ATTAATTGAATCCGGAGCTGGAACAGGATGAACAGTTTATCCCCCTCTATCTTCAAATTT  360
             |||||||||||| |||||||| ||||||||||||||||| || |||||||||||||||||
Sbjct  301   ATTAATTGAATCTGGAGCTGGTACAGGATGAACAGTTTACCCTCCTCTATCTTCAAATTT  360

Query  361   ATCACATTATGGTCCATCAGTAGATTTAGCTATTTTTTCTCTTCACCTTGCTGGTGCTTC  420
             |||||||||||| || ||||||||||||||||||||||||||||| ||||||||||||||
Sbjct  361   ATCACATTATGGCCCTTCAGTAGATTTAGCTATTTTTTCTCTTCATCTTGCTGGTGCTTC  420
```

```
Query  421   TTCAATTTTAGGTGCAATTAACTTCATCACAACTATTGTAAATATACGATCAATTGGAAT  480
             |||||||||||||||||||||||| || |||||||||||||| |||||||||||||||||
Sbjct  421   TTCAATTTTAGGTGCAATTAACTTTATTACAACTATTGTAAACATACGATCAATTGGAAT  480

Query  481   AACAATAGAACGAATACCATTATTTGTTTGATCTGTTTTAATTACAGCTATTTTACTACT  540
             |||||||||||| |||||||||||||||||||||||||||||||||||||||||| ||||
Sbjct  481   AACAATAGAACGTATACCATTATTTGTTTGATCTGTTTTAATTACAGCTATTTTATTACT  540

Query  541   TCTATCTTTACCTGTATTAGCAGGTGCCATTACAATATTATTAACTGATCGAAACTTTAA  600
             | ||||||||||||||||||||||||||||||||||||| ||||||||||||||||||||
Sbjct  541   TTTATCTTTACCTGTATTAGCAGGTGCCATTACAATATTGTTAACTGATCGAAACTTTAA  600

Query  601   CACATCATTTTTTGATCCTTCAGGAGGAGGAGATCCCATTTTATATCAACACTTATTT  658
              ||||||||||||||||||||||| ||||||||||| |||||||||||||| ||||||
Sbjct  601   TACATCATTTTTTGATCCTTCAGGGGGAGGAGATCCAATTTTATATCAACATTTATTT  658
```

>Rhipicephalus sanguineus isolate 5MSK cytochrome c oxidase subunit I (COX1) gene, partial cds; mitochondrial
Sequence ID: MW152145.1 Length: 676
Range 1: 1 to 658

Score:1055 bits(571), Expect:0.0,
Identities:629/658(96%), Gaps:0/658(0%), Strand: Plus/Plus

```
Query  1     AACAATATATTTAATTTTTGGAGCATGATCAGGTATATTAGGACTTAGTATAAGAATATT  60
             ||||||||||||||||||||||||||||||||||||||||||||||||||||||||||||
Sbjct  1     AACAATATATTTAATTTTTGGAGCATGATCAGGTATATTAGGACTTAGTATAAGAATATT  60

Query  61    AATTCGTATAGAATTAGGACAACCTGGAACTTTAATTGGAAATGATCAGATTTATAATGT  120
             ||||||||||||||||||||||||||||||||||||||||||||||||||||||||||||
Sbjct  61    AATTCGTATAGAATTAGGACAACCTGGAACTTTAATTGGAAATGATCAGATTTATAATGT  120

Query  121   AATCGTAACAGCACATGCATTTATTATAATTTTCTTTATAGTTATACCAATCATAATCGG  180
             ||||||||||||||||||||||||||||||||| ||||||||||||||||||||||||||
Sbjct  121   AATCGTAACAGCACATGCATTTATTATAATTTTTTTTATAGTTATACCAATCATAATCGG  180

Query  181   AGGGTTCGGAAATTGATTAGTTCCTATTATATTAGGTGCCCCAGATATAGCATTTCCACG  240
             ||| || |||||||||||||| ||||| ||||| ||||||||||||||||||||||||||
Sbjct  181   AGGATTTGGAAATTGATTAGTCCCTATCATATTGGGTGCCCCAGATATAGCATTTCCACG  240

Query  241   AATAAATAATATAAGATTTTGACTTTTACCCCCCTCATTATTTTTATTGATTAACTCTTC  300
             |||||||||||||||||||||||||||||| || ||| |||||||||| ||||| |||||
Sbjct  241   AATAAATAATATAAGATTTTGACTTTTACCTCCTTCACTATTTTTATTAATTAATTCTTC  300

Query  301   ATTAATTGAATCCGGAGCTGGAACAGGATGAACAGTTTATCCCCCTCTATCTTCAAATTT  360
             |||||||||||| |||||||| ||||||||||||||||| || |||||||||||||||||
Sbjct  301   ATTAATTGAATCTGGAGCTGGTACAGGATGAACAGTTTACCCTCCTCTATCTTCAAATTT  360

Query  361   ATCACATTATGGTCCATCAGTAGATTTAGCTATTTTTTCTCTTCACCTTGCTGGTGCTTC  420
             |||||||||||| || ||||||||||||||||||||||||||||| ||||||||||||||
Sbjct  361   ATCACATTATGGCCCTTCAGTAGATTTAGCTATTTTTTCTCTTCATCTTGCTGGTGCTTC  420
```

```
Query  421   TTCAATTTTAGGTGCAATTAACTTCATCACAACTATTGTAAATATACGATCAATTGGAAT  480
             |||||||||||||||||||||||| || |||||||||||||| |||||||||||||||||
Sbjct  421   TTCAATTTTAGGTGCAATTAACTTTATTACAACTATTGTAAACATACGATCAATTGGAAT  480

Query  481   AACAATAGAACGAATACCATTATTTGTTTGATCTGTTTTAATTACAGCTATTTTACTACT  540
             |||||||||||| |||||||||||||||||||||||||||||||||||||||||| ||||
Sbjct  481   AACAATAGAACGTATACCATTATTTGTTTGATCTGTTTTAATTACAGCTATTTTATTACT  540

Query  541   TCTATCTTTACCTGTATTAGCAGGTGCCATTACAATATTATTAACTGATCGAAACTTTAA  600
             | ||||||||||||||||||||||||||||||||||||| ||||||||||||||||||||
Sbjct  541   TTTATCTTTACCTGTATTAGCAGGTGCCATTACAATATTGTTAACTGATCGAAACTTTAA  600

Query  601   CACATCATTTTTTGATCCTTCAGGAGGAGGAGATCCCATTTTATATCAACACTTATTT  658
              ||||||||||||||||||||||| ||||||||||| |||||||||||||| ||||||
Sbjct  601   TACATCATTTTTTGATCCTTCAGGGGGAGGAGATCCAATTTTATATCAACATTTATTT  658
```

>Rhipicephalus sanguineus isolate 1MSK cytochrome c oxidase subunit I (COX1) gene, partial cds; mitochondrial
Sequence ID: MW152144.1 Length: 675
Range 1: 1 to 658

Score:1055 bits(571), Expect:0.0,
Identities:629/658(96%), Gaps:0/658(0%), Strand: Plus/Plus

```
Query  1     AACAATATATTTAATTTTTGGAGCATGATCAGGTATATTAGGACTTAGTATAAGAATATT  60
             ||||||||||||||||||||||||||||||||||||||||||||||||||||||||||||
Sbjct  1     AACAATATATTTAATTTTTGGAGCATGATCAGGTATATTAGGACTTAGTATAAGAATATT  60

Query  61    AATTCGTATAGAATTAGGACAACCTGGAACTTTAATTGGAAATGATCAGATTTATAATGT  120
             ||||||||||||||||||||||||||||||||||||||||||||||||||||||||||||
Sbjct  61    AATTCGTATAGAATTAGGACAACCTGGAACTTTAATTGGAAATGATCAGATTTATAATGT  120

Query  121   AATCGTAACAGCACATGCATTTATTATAATTTTCTTTATAGTTATACCAATCATAATCGG  180
             ||||||||||||||||||||||||||||||||| ||||||||||||||||||||||||||
Sbjct  121   AATCGTAACAGCACATGCATTTATTATAATTTTTTTTATAGTTATACCAATCATAATCGG  180

Query  181   AGGGTTCGGAAATTGATTAGTTCCTATTATATTAGGTGCCCCAGATATAGCATTTCCACG  240
             ||| || |||||||||||||| ||||| ||||| ||||||||||||||||||||||||||
Sbjct  181   AGGATTTGGAAATTGATTAGTCCCTATCATATTGGGTGCCCCAGATATAGCATTTCCACG  240

Query  241   AATAAATAATATAAGATTTTGACTTTTACCCCCCTCATTATTTTTATTGATTAACTCTTC  300
             |||||||||||||||||||||||||||||| || ||| |||||||||| ||||| |||||
Sbjct  241   AATAAATAATATAAGATTTTGACTTTTACCTCCTTCACTATTTTTATTAATTAATTCTTC  300

Query  301   ATTAATTGAATCCGGAGCTGGAACAGGATGAACAGTTTATCCCCCTCTATCTTCAAATTT  360
             |||||||||||| |||||||| ||||||||||||||||| || |||||||||||||||||
Sbjct  301   ATTAATTGAATCTGGAGCTGGTACAGGATGAACAGTTTACCCTCCTCTATCTTCAAATTT  360

Query  361   ATCACATTATGGTCCATCAGTAGATTTAGCTATTTTTTCTCTTCACCTTGCTGGTGCTTC  420
             |||||||||||| || ||||||||||||||||||||||||||||| ||||||||||||||
```

```
Sbjct  361  ATCACATTATGGCCCTTCAGTAGATTTAGCTATTTTTTCTCTTCATCTTGCTGGTGCTTC  420

Query  421  TTCAATTTTAGGTGCAATTAACTTCATCACAACTATTGTAAATATACGATCAATTGGAAT  480
            |||||||||||||||||||||||| || |||||||||||||| |||||||||||||||||
Sbjct  421  TTCAATTTTAGGTGCAATTAACTTTATTACAACTATTGTAAACATACGATCAATTGGAAT  480

Query  481  AACAATAGAACGAATACCATTATTTGTTTGATCTGTTTTAATTACAGCTATTTTACTACT  540
            |||||||||||| |||||||||||||||||||||||||||||||||||||||||| ||||
Sbjct  481  AACAATAGAACGTATACCATTATTTGTTTGATCTGTTTTAATTACAGCTATTTTATTACT  540

Query  541  TCTATCTTTACCTGTATTAGCAGGTGCCATTACAATATTATTAACTGATCGAAACTTTAA  600
            | ||||||||||||||||||||||||||||||||||||| ||||||||||||||||||||
Sbjct  541  TTTATCTTTACCTGTATTAGCAGGTGCCATTACAATATTGTTAACTGATCGAAACTTTAA  600

Query  601  CACATCATTTTTTGATCCTTCAGGAGGAGGAGATCCCATTTTATATCAACACTTATTT  658
             ||||||||||||||||||||||| ||||||||||| |||||||||||||| ||||||
Sbjct  601  TACATCATTTTTTGATCCTTCAGGGGGAGGAGATCCAATTTTATATCAACATTTATTT  658
```

>Rhipicephalus sanguineus isolate RSN2SK cytochrome c oxidase subunit I (COX1) gene, partial cds; mitochondrial
Sequence ID: MW152143.1 Length: 676
Range 1: 1 to 658

Score:1055 bits(571), Expect:0.0,
Identities:629/658(96%), Gaps:0/658(0%), Strand: Plus/Plus

```
Query  1    AACAATATATTTAATTTTTGGAGCATGATCAGGTATATTAGGACTTAGTATAAGAATATT  60
            ||||||||||||||||||||||||||||||||||||||||||||||||||||||||||||
Sbjct  1    AACAATATATTTAATTTTTGGAGCATGATCAGGTATATTAGGACTTAGTATAAGAATATT  60

Query  61   AATTCGTATAGAATTAGGACAACCTGGAACTTTAATTGGAAATGATCAGATTTATAATGT  120
            ||||||||||||||||||||||||||||||||||||||||||||||||||||||||||||
Sbjct  61   AATTCGTATAGAATTAGGACAACCTGGAACTTTAATTGGAAATGATCAGATTTATAATGT  120

Query  121  AATCGTAACAGCACATGCATTTATTATAATTTCTTTATAGTTATACCAATCATAATCGG  180
            |||||||||||||||||||||||||||||||| |||||||||||||||||||||||||||
Sbjct  121  AATCGTAACAGCACATGCATTTATTATAATTTTTTTATAGTTATACCAATCATAATCGG  180

Query  181  AGGGTTCGGAAATTGATTAGTTCCTATTATATTAGGTGCCCCAGATATAGCATTTCCACG  240
            ||| || |||||||||||||| ||||| ||||| ||||||||||||||||||||||||||
Sbjct  181  AGGATTTGGAAATTGATTAGTCCCTATCATATTGGGTGCCCCAGATATAGCATTTCCACG  240

Query  241  AATAAATAATATAAGATTTTGACTTTTACCCCCCTCATTATTTTTATTGATTAACTCTTC  300
            |||||||||||||||||||||||||||||| || ||| |||||||||| ||||| |||||
Sbjct  241  AATAAATAATATAAGATTTTGACTTTTACCTCCTTCACTATTTTTATTAATTAATTCTTC  300

Query  301  ATTAATTGAATCCGGAGCTGGAACAGGATGAACAGTTTATCCCCCTCTATCTTCAAATTT  360
            |||||||||||| |||||||| ||||||||||||||||| || |||||||||||||||||
Sbjct  301  ATTAATTGAATCTGGAGCTGGTACAGGATGAACAGTTTACCCTCCTCTATCTTCAAATTT  360

Query  361  ATCACATTATGGTCCATCAGTAGATTTAGCTATTTTTTCTCTTCACCTTGCTGGTGCTTC  420
```

```
                |||||||||||| || ||||||||||||||||||||||||||||| ||||||||||||||
Sbjct  361  ATCACATTATGGCCCTTCAGTAGATTTAGCTATTTTTTCTCTTCATCTTGCTGGTGCTTC  420

Query  421  TTCAATTTTAGGTGCAATTAACTTCATCACAACTATTGTAAATATACGATCAATTGGAAT  480
            |||||||||||||||||||||||| || |||||||||||||| |||||||||||||||||
Sbjct  421  TTCAATTTTAGGTGCAATTAACTTTATTACAACTATTGTAAACATACGATCAATTGGAAT  480

Query  481  AACAATAGAACGAATACCATTATTTGTTTGATCTGTTTTAATTACAGCTATTTTACTACT  540
            |||||||||||| |||||||||||||||||||||||||||||||||||||||||| ||||
Sbjct  481  AACAATAGAACGTATACCATTATTTGTTTGATCTGTTTTAATTACAGCTATTTTATTACT  540

Query  541  TCTATCTTTACCTGTATTAGCAGGTGCCATTACAATATTATTAACTGATCGAAACTTTAA  600
            | ||||||||||||||||||||||||||||||||||||| ||||||||||||||||||||
Sbjct  541  TTTATCTTTACCTGTATTAGCAGGTGCCATTACAATATTGTTAACTGATCGAAACTTTAA  600

Query  601  CACATCATTTTTTGATCCTTCAGGAGGAGGAGATCCCATTTATATCAACACTTATTT  658
             ||||||||||||||||||||||| ||||||||||| ||||||||||||| |||||||
Sbjct  601  TACATCATTTTTTGATCCTTCAGGGGGAGGAGATCCAATTTATATCAACATTTATTT  658
```

>Rhipicephalus sanguineus isolate RSN11SK cytochrome c oxidase subunit I (COX1) gene, partial cds; mitochondrial
Sequence ID: MW152142.1 Length: 669
Range 1: 1 to 658

Score:1055 bits(571), Expect:0.0,
Identities:629/658(96%), Gaps:0/658(0%), Strand: Plus/Plus

```
Query  1    AACAATATATTTAATTTTTGGAGCATGATCAGGTATATTAGGACTTAGTATAAGAATATT  60
            ||||||||||||||||||||||||||||||||||||||||||||||||||||||||||||
Sbjct  1    AACAATATATTTAATTTTTGGAGCATGATCAGGTATATTAGGACTTAGTATAAGAATATT  60

Query  61   AATTCGTATAGAATTAGGACAACCTGGAACTTTAATTGGAAATGATCAGATTTATAATGT  120
            ||||||||||||||||||||||||||||||||||||||||||||||||||||||||||||
Sbjct  61   AATTCGTATAGAATTAGGACAACCTGGAACTTTAATTGGAAATGATCAGATTTATAATGT  120

Query  121  AATCGTAACAGCACATGCATTTATTATAATTTTCTTTATAGTTATACCAATCATAATCGG  180
            ||||||||||||||||||||||||||||||||| ||||||||||||||||||||||||||
Sbjct  121  AATCGTAACAGCACATGCATTTATTATAATTTTTTTTATAGTTATACCAATCATAATCGG  180

Query  181  AGGGTTCGGAAATTGATTAGTTCCTATTATATTAGGTGCCCCAGATATAGCATTTCCACG  240
            ||| || |||||||||||||| ||||| ||||| ||||||||||||||||||||||||||
Sbjct  181  AGGATTTGGAAATTGATTAGTCCCTATCATATTGGGTGCCCCAGATATAGCATTTCCACG  240

Query  241  AATAAATAATATAAGATTTTGACTTTTACCCCCCTCATTATTTTTATTGATTAACTCTTC  300
            |||||||||||||||||||||||||||||| || ||| |||||||||| ||||| |||||
Sbjct  241  AATAAATAATATAAGATTTTGACTTTTACCTCCTTCACTATTTTTATTAATTAATTCTTC  300

Query  301  ATTAATTGAATCCGGAGCTGGAACAGGATGAACAGTTTATCCCCCTCTATCTTCAAATTT  360
            |||||||||||| |||||||| ||||||||||||||||| || |||||||||||||||||
Sbjct  301  ATTAATTGAATCTGGAGCTGGTACAGGATGAACAGTTTACCCTCCTCTATCTTCAAATTT  360
```

```
Query  361  ATCACATTATGGTCCATCAGTAGATTTAGCTATTTTTTCTCTTCACCTTGCTGGTGCTTC  420
            |||||||||||| || ||||||||||||||||||||||||||||| ||||||||||||||
Sbjct  361  ATCACATTATGGCCCTTCAGTAGATTTAGCTATTTTTTCTCTTCATCTTGCTGGTGCTTC  420

Query  421  TTCAATTTTAGGTGCAATTAACTTCATCACAACTATTGTAAATATACGATCAATTGGAAT  480
            |||||||||||||||||||||||| || |||||||||||||| |||||||||||||||||
Sbjct  421  TTCAATTTTAGGTGCAATTAACTTTATTACAACTATTGTAAACATACGATCAATTGGAAT  480

Query  481  AACAATAGAACGAATACCATTATTTGTTTGATCTGTTTTAATTACAGCTATTTTACTACT  540
            |||||||||||| |||||||||||||||||||||||||||||||||||||||||| ||||
Sbjct  481  AACAATAGAACGTATACCATTATTTGTTTGATCTGTTTTAATTACAGCTATTTTATTACT  540

Query  541  TCTATCTTTACCTGTATTAGCAGGTGCCATTACAATATTATTAACTGATCGAAACTTTAA  600
            | ||||||||||||||||||||||||||||||||||||| ||||||||||||||||||||
Sbjct  541  TTTATCTTTACCTGTATTAGCAGGTGCCATTACAATATTGTTAACTGATCGAAACTTTAA  600

Query  601  CACATCATTTTTTGATCCTTCAGGAGGAGGAGATCCCATTTTATATCAACACTTATTT  658
             ||||||||||||||||||||||| ||||||||||| |||||||||||||| ||||||
Sbjct  601  TACATCATTTTTTGATCCTTCAGGGGGAGGAGATCCAATTTTATATCAACATTTATTT  658
```

>Rhipicephalus sanguineus sensu lato 'temperate lineage' isolate S358 cytochrome c oxidase subunit I (COI) gene, partial cds; mitochondrial
Sequence ID: MF426012.1 Length: 684
Range 1: 14 to 671

Score:1050 bits(568), Expect:0.0,
Identities:628/658(95%), Gaps:0/658(0%), Strand: Plus/Plus

```
Query  1    AACAATATATTTAATTTTTGGAGCATGATCAGGTATATTAGGACTTAGTATAAGAATATT  60
            ||||||||||||||||||||||||||||||||||||||||||||||||||||||||||||
Sbjct  14   AACAATATATTTAATTTTTGGAGCATGATCAGGTATATTAGGACTTAGTATAAGAATATT  73

Query  61   AATTCGTATAGAATTAGGACAACCTGGAACTTTAATTGGAAATGATCAGATTTATAATGT  120
            ||||||||||||||||||||||||||||||||||||||||||||||||||||||||||||
Sbjct  74   AATTCGTATAGAATTAGGACAACCTGGAACTTTAATTGGAAATGATCAGATTTATAATGT  133

Query  121  AATCGTAACAGCACATGCATTTATTATAATTTTCTTTATAGTTATACCAATCATAATCGG  180
            ||||||||||||||||||||||||||||||||| ||||||||||||||||||||||||||
Sbjct  134  AATCGTAACAGCACATGCATTTATTATAATTTTTTTTATAGTTATACCAATCATAATCGG  193

Query  181  AGGGTTCGGAAATTGATTAGTTCCTATTATATTAGGTGCCCCAGATATAGCATTTCCACG  240
            ||| || |||||||||||||| ||||| ||||||||||||||||||||||||||||||||
Sbjct  194  AGGATTTGGAAATTGATTAGTCCCTATCATATTAGGTGCCCCAGATATAGCATTTCCACG  253

Query  241  AATAAATAATATAAGATTTTGACTTTTACCCCCCTCATTATTTTTATTGATTAACTCTTC  300
            |||||||||||||||||||||||||||||| || ||| |||||||||| |||||||||||
Sbjct  254  AATAAATAATATAAGATTTTGACTTTTACCTCCTTCACTATTTTTATTAATTAACTCTTC  313

Query  301  ATTAATTGAATCCGGAGCTGGAACAGGATGAACAGTTTATCCCCCTCTATCTTCAAATTT  360
            |||||||||||| |||||||| ||||||||||||||||| || |||||||||||||||||
Sbjct  314  ATTAATTGAATCTGGAGCTGGTACAGGATGAACAGTTTACCCTCCTCTATCTTCAAATTT  373
```

```
Query  361   ATCACATTATGGTCCATCAGTAGATTTAGCTATTTTTCTCTTCACCTTGCTGGTGCTTC  420
             |||||||||||| || ||||||||||||||||| ||||||||||| ||||||||||||||
Sbjct  374   ATCACATTATGGCCCTTCAGTAGATTTAGCTATCTTTTCTCTTCATCTTGCTGGTGCTTC  433

Query  421   TTCAATTTTAGGTGCAATTAACTTCATCACAACTATTGTAAATATACGATCAATTGGAAT  480
             |||||||||||||||||||||||| || ||||||||| |||| |||||||||||||||||
Sbjct  434   TTCAATTTTAGGTGCAATTAACTTTATTACAACTATTATAAACATACGATCAATTGGAAT  493

Query  481   AACAATAGAACGAATACCATTATTTGTTTGATCTGTTTTAATTACAGCTATTTTACTACT  540
             |||||||||||| |||||||||||||||||||||||||||||||||||||||||| ||||
Sbjct  494   AACAATAGAACGTATACCATTATTTGTTTGATCTGTTTTAATTACAGCTATTTTATTACT  553

Query  541   TCTATCTTTACCTGTATTAGCAGGTGCCATTACAATATTATTAACTGATCGAAACTTTAA  600
               ||||||||||||||||||||||||||||||||||||| ||||||||||||||||||||
Sbjct  554   CTTATCTTTACCTGTATTAGCAGGTGCCATTACAATATTGTTAACTGATCGAAACTTTAA  613

Query  601   CACATCATTTTTTGATCCTTCAGGAGGAGGAGATCCCATTTTATATCAACACTTATTT  658
              ||||||||||||||||||||||| ||||||||||| |||||||||||||| ||||||
Sbjct  614   TACATCATTTTTTGATCCTTCAGGGGGAGGAGATCCAATTTTATATCAACATTTATTT  671
```

>Rhipicephalus sanguineus haplotype I cytochrome oxidase subunit I (COI) gene, partial cds; mitochondrial
Sequence ID: KU556743.1 Length: 659
Range 1: 12 to 659

Score:1048 bits(567), Expect:0.0,
Identities:621/648(96%), Gaps:0/648(0%), Strand: Plus/Plus

```
Query  1     AACAATATATTTAATTTTTGGAGCATGATCAGGTATATTAGGACTTAGTATAAGAATATT  60
             ||||||||||||||||||||||||||||||||||||||||||||||||||||||||||||
Sbjct  12    AACAATATATTTAATTTTTGGAGCATGATCAGGTATATTAGGACTTAGTATAAGAATATT  71

Query  61    AATTCGTATAGAATTAGGACAACCTGGAACTTTAATTGGAAATGATCAGATTTATAATGT  120
             ||||||||||||||||||||||||||||||||||||||||||||||||||||||||||||
Sbjct  72    AATTCGTATAGAATTAGGACAACCTGGAACTTTAATTGGAAATGATCAGATTTATAATGT  131

Query  121   AATCGTAACAGCACATGCATTTATTATAATTTTCTTTATAGTTATACCAATCATAATCGG  180
             ||||||||||||||||||||||||||||||||| ||||||||||||||||||||||||||
Sbjct  132   AATCGTAACAGCACATGCATTTATTATAATTTTTTTTATAGTTATACCAATCATAATCGG  191

Query  181   AGGGTTCGGAAATTGATTAGTTCCTATTATATTAGGTGCCCCAGATATAGCATTTCCACG  240
             ||| || |||||||||||| | ||||| ||||| ||||||||||||||||||||||||||
Sbjct  192   AGGATTTGGAAATTGATTAATCCCTATCATATTGGGTGCCCCAGATATAGCATTTCCACG  251

Query  241   AATAAATAATATAAGATTTTGACTTTTACCCCCCTCATTATTTTTATTGATTAACTCTTC  300
             |||||||||||||||||||||||||||||| || ||| |||||||||| |||||||||||
Sbjct  252   AATAAATAATATAAGATTTTGACTTTTACCTCCTTCACTATTTTTATTAATTAACTCTTC  311

Query  301   ATTAATTGAATCCGGAGCTGGAACAGGATGAACAGTTTATCCCCCTCTATCTTCAAATTT  360
             |||||||||||| |||||||| ||||||||||||||||| || |||||||||||||||||
```

```
Sbjct  312  ATTAATTGAATCTGGAGCTGGTACAGGATGAACAGTTTACCCTCCTCTATCTTCAAATTT  371

Query  361  ATCACATTATGGTCCATCAGTAGATTTAGCTATTTTTTCTCTTCACCTTGCTGGTGCTTC  420
            ||||||||||||||| ||||||||||||||||||||||||||||| ||||||||||||||
Sbjct  372  ATCACATTATGGTCCTTCAGTAGATTTAGCTATTTTTTCTCTTCATCTTGCTGGTGCTTC  431

Query  421  TTCAATTTTAGGTGCAATTAACTTCATCACAACTATTGTAAATATACGATCAATTGGAAT  480
            |||||||||||||||||||||||| || |||||||||||||| |||||||||||||||||
Sbjct  432  TTCAATTTTAGGTGCAATTAACTTTATTACAACTATTGTAAACATACGATCAATTGGAAT  491

Query  481  AACAATAGAACGAATACCATTATTTGTTTGATCTGTTTTAATTACAGCTATTTTACTACT  540
            |||||||||||| |||||||||||||||||||||||||||||||||||||||||| ||||
Sbjct  492  AACAATAGAACGTATACCATTATTTGTTTGATCTGTTTTAATTACAGCTATTTTATTACT  551

Query  541  TCTATCTTTACCTGTATTAGCAGGTGCCATTACAATATTATTAACTGATCGAAACTTTAA  600
            | ||||||||||||||||||||||||||||||||||||| ||||||||||||||||||||
Sbjct  552  TTTATCTTTACCTGTATTAGCAGGTGCCATTACAATATTGTTAACTGATCGAAACTTTAA  611

Query  601  CACATCATTTTTTGATCCTTCAGGAGGAGGAGATCCCATTTTATATCA  648
             ||||||||||||||||||||||| ||||||||||| |||||||||||
Sbjct  612  TACATCATTTTTTGATCCTTCAGGGGGAGGAGATCCAATTTTATATCA  659
```

>Rhipicephalus sanguineus haplotype II cytochrome oxidase subunit I (COI) gene, partial cds; mitochondrial
Sequence ID: KU556744.1 Length: 787
Range 1: 29 to 688

Score:1046 bits(566), Expect:0.0,
Identities:629/660(95%), Gaps:2/660(0%), Strand: Plus/Plus

```
Query  1    AACAATATATTTAATTTTTGGAGCATGATCAGGTATATTAGGACTTAGTATAAGAATATT  60
            ||||||||||||||||||||||||||||||||||||||||||||||||||||||||||||
Sbjct  29   AACAATATATTTAATTTTTGGAGCATGATCAGGTATATTAGGACTTAGTATAAGAATATT  88

Query  61   AATTCGTATAGAATTAGGACAACCTGGAACTTTAATTGGAAATGATCAGATTTATAATGT  120
            ||||||||||||||||||||||||||||||||||||||||||||||||||||||||||||
Sbjct  89   AATTCGTATAGAATTAGGACAACCTGGAACTTTAATTGGAAATGATCAGATTTATAATGT  148

Query  121  AATCGTAACAGCACATGCATTTATTATAATTTTCTTTATAGTTATACCAATCATAATCGG  180
            ||||||||||||||||||||||||||||||||| ||||||||||||||||||||||||||
Sbjct  149  AATCGTAACAGCACATGCATTTATTATAATTTTTTTTATAGTTATACCAATCATAATCGG  208

Query  181  AGGGTTCGGAAATTGATTAGTTCCTATTATATTAGGTGCCCCAGATATAGCATTTCCACG  240
            ||| || ||||| |||||||| ||||| ||||| ||||||||||||||||||||||||||
Sbjct  209  AGGATTTGGAAACTGATTAGTCCCTATCATATTGGGTGCCCCAGATATAGCATTTCCACG  268

Query  241  AATAAATAATATAAGATTTTGACTTTTACCCCCCTCATTATTTTTATTGATTAACTCTTC  300
            |||||||||||||||||||||||||||||| || ||| |||||||||| ||||||||||||
Sbjct  269  AATAAATAATATAAGATTTTGACTTTTACCTCCTTCACTATTTTTATTAATTAACTCTTC  328

Query  301  ATTAATTGAATCCGGAGCTGGAACAGGATGAACAGTTTATCCCCCTCTATCTTCAAATTT  360
```

```
            |||||||||||| |||||||| ||||||||||||||||| || |||||||||||||||||
Sbjct  329  ATTAATTGAATCTGGAGCTGGTACAGGATGAACAGTTTACCCTCCTCTATCTTCAAATTT  388

Query  361  ATCACATTATGGTCCATCAGTAGATTTAGCTATTTTTCTCTTCACCTTGCTGGTGCTTC  420
            ||||||||||||||| |||||||||||||||||||||||||||| |||||||||||||||
Sbjct  389  ATCACATTATGGTCCTTCAGTAGATTTAGCTATTTTTCTCTTCATCTTGCTGGTGCTTC  448

Query  421  TTCAATTTTAGGTGCAATTAACTTCATCACAACTATTGTAAATATACGATCAATTGGAAT  480
            |||||||||||||||||||||||| || |||||||||||||| |||||||||||||||||
Sbjct  449  TTCAATTTTAGGTGCAATTAACTTTATTACAACTATTGTAAACATACGATCAATTGGAAT  508

Query  481  AACAATAGAACGAATACCATTATTTGTTTGATCTGTTTTAATTACAGCTATTTTACTACT  540
            |||||||||||| |||||||||||||||||||||||||||||||||||||||||| ||||
Sbjct  509  AACAATAGAACGTATACCATTATTTGTTTGATCTGTTTTAATTACAGCTATTTTATTACT  568

Query  541  TCTATCTTTACCTGTATTAGCAGGTGCCATTACAATATTATTAACTGATCGAAACTTTAA  600
            | ||||||||||||||||||||||||||||||||||||| ||||||||||||||||||||
Sbjct  569  TTTATCTTTACCTGTATTAGCAGGTGCCATTACAATATTGTTAACTGATCGAAACTTTAA  628

Query  601  CACATCATTTTTTGATCCTTCAGGAGG-AGGAGATCCC-ATTTTATATCAACACTTATTT  658
             ||||||||||||||||||||||| || |||||||||  |||||||||||||  ||||||
Sbjct  629  TACATCATTTTTTGATCCTTCAGGGGGGAGGAGATCCAAATTTTATATCAACTTTTATTT  688
```

>Rhipicephalus sanguineus sensu lato 'temperate lineage' isolate S943 cytochrome c oxidase subunit I (COI) gene, partial cds; mitochondrial
Sequence ID: MF426020.1 Length: 812
Range 1: 2 to 644

Score:1038 bits(562), Expect:0.0,
Identities:616/643(96%), Gaps:0/643(0%), Strand: Plus/Plus

```
Query  16   TTTTGGAGCATGATCAGGTATATTAGGACTTAGTATAAGAATATTAATTCGTATAGAATT  75
            ||||||||||||||||||||||||||||||||||||||||||||||||||||||||||||
Sbjct  2    TTTTGGAGCATGATCAGGTATATTAGGACTTAGTATAAGAATATTAATTCGTATAGAATT  61

Query  76   AGGACAACCTGGAACTTTAATTGGAAATGATCAGATTTATAATGTAATCGTAACAGCACA  135
            ||||||||||||||||||||||||||||||||||||||||||||||||||||||||||||
Sbjct  62   AGGACAACCTGGAACTTTAATTGGAAATGATCAGATTTATAATGTAATCGTAACAGCACA  121

Query  136  TGCATTTATTATAATTTTCTTTATAGTTATACCAATCATAATCGGAGGGTTCGGAAATTG  195
            |||||||||||||||||| ||||||||||||||||||||||||||||| || ||||||||
Sbjct  122  TGCATTTATTATAATTTTTTTTATAGTTATACCAATCATAATCGGAGGATTTGGAAATTG  181

Query  196  ATTAGTTCCTATTATATTAGGTGCCCCAGATATAGCATTTCCACGAATAAATAATATAAG  255
            |||||| ||||| ||||| |||||||||||||||||||||||||||||||||||||||||
Sbjct  182  ATTAGTCCCTATCATATTGGGTGCCCCAGATATAGCATTTCCACGAATAAATAATATAAG  241

Query  256  ATTTTGACTTTTACCCCCCTCATTATTTTTATTGATTAACTCTTCATTAATTGAATCCGG  315
            ||||||||||||||| || ||| |||||||||| ||||||||||||||||||||||| ||
Sbjct  242  ATTTTGACTTTTACCTCCTTCACTATTTTTATTAATTAACTCTTCATTAATTGAATCTGG  301
```

```
Query  316  AGCTGGAACAGGATGAACAGTTTATCCCCCTCTATCTTCAAATTTATCACATTATGGTCC  375
            |||||| ||||||||||||||||| || ||||||||||||||||||||||||||||||||
Sbjct  302  AGCTGGTACAGGATGAACAGTTTACCCTCCTCTATCTTCAAATTTATCACATTATGGTCC  361

Query  376  ATCAGTAGATTTAGCTATTTTTTCTCTTCACCTTGCTGGTGCTTCTTCAATTTTAGGTGC  435
             ||||||||||||||||||||||||||||| |||||||||||||||||||||||||||||
Sbjct  362  TTCAGTAGATTTAGCTATTTTTTCTCTTCATCTTGCTGGTGCTTCTTCAATTTTAGGTGC  421

Query  436  AATTAACTTCATCACAACTATTGTAAATATACGATCAATTGGAATAACAATAGAACGAAT  495
            ||||||||| || |||||||||||||| ||||||||||||||||||||||||||||| ||
Sbjct  422  AATTAACTTTATTACAACTATTGTAAACATACGATCAATTGGAATAACAATAGAACGTAT  481

Query  496  ACCATTATTTGTTTGATCTGTTTTAATTACAGCTATTTTACTACTTCTATCTTTACCTGT  555
            |||||||||||||||||||||||||||||||||||||||| ||||| |||||||||||||
Sbjct  482  ACCATTATTTGTTTGATCTGTTTTAATTACAGCTATTTTATTACTTTTATCTTTACCTGT  541

Query  556  ATTAGCAGGTGCCATTACAATATTATTAACTGATCGAAACTTTAACACATCATTTTTTGA  615
            |||||||||||||||||||||||| |||||||||||||||||||| ||||||||||||||
Sbjct  542  ATTAGCAGGTGCCATTACAATATTGTTAACTGATCGAAACTTTAATACATCATTTTTTGA  601

Query  616  TCCTTCAGGAGGAGGAGATCCCATTTTATATCAACACTTATTT  658
            ||||||||| ||||||||||| |||||||||||||| ||||||
Sbjct  602  TCCTTCAGGGGGAGGAGATCCAATTTTATATCAACATTTATTT  644
```

>Rhipicephalus sanguineus sensu lato 'temperate lineage' isolate S337 cytochrome c oxidase subunit I (COI) gene, partial cds; mitochondrial
Sequence ID: MF426010.1 Length: 824
Range 1: 1 to 648

Score:1037 bits(561), Expect:0.0,
Identities:619/648(96%), Gaps:0/648(0%), Strand: Plus/Plus

```
Query  11   TTAATTTTTGGAGCATGATCAGGTATATTAGGACTTAGTATAAGAATATTAATTCGTATA  70
            ||||||||||||||||||||||||||||||||||||||||||||||||||||||||||||
Sbjct  1    TTAATTTTTGGAGCATGATCAGGTATATTAGGACTTAGTATAAGAATATTAATTCGTATA  60

Query  71   GAATTAGGACAACCTGGAACTTTAATTGGAAATGATCAGATTTATAATGTAATCGTAACA  130
            ||||||||||||||||||||||||||||||||||||||||||||||||||||||||||||
Sbjct  61   GAATTAGGACAACCTGGAACTTTAATTGGAAATGATCAGATTTATAATGTAATCGTAACA  120

Query  131  GCACATGCATTTATTATAATTTTCTTTATAGTTATACCAATCATAATCGGAGGGTTCGGA  190
            ||||||||||||||||||||||| ||||||||||||||||||||||||||||| || |||
Sbjct  121  GCACATGCATTTATTATAATTTTTTTTATAGTTATACCAATCATAATCGGAGGATTTGGA  180

Query  191  AATTGATTAGTTCCTATTATATTAGGTGCCCCAGATATAGCATTTCCACGAATAAATAAT  250
            ||||||||||| ||||| ||||| ||||||||||||||||||||||||||||||||||||
Sbjct  181  AATTGATTAGTCCCTATCATATTGGGTGCCCCAGATATAGCATTTCCACGAATAAATAAT  240

Query  251  ATAAGATTTTGACTTTTACCCCCCTCATTATTTTTATTGATTAACTCTTCATTAATTGAA  310
            |||||||||||||||||||| || ||| |||||||||| |||||||||||||||||||||
Sbjct  241  ATAAGATTTTGACTTTTACCTCCTTCACTATTTTTATTAATTAACTCTTCATTAATTGAA  300
```

```
Query  311  TCCGGAGCTGGAACAGGATGAACAGTTTATCCCCCTCTATCTTCAAATTTATCACATTAT  370
            || |||||||| ||||||||||||||||| || |||||||||||||||||||||||||||
Sbjct  301  TCTGGAGCTGGTACAGGATGAACAGTTTACCCTCCTCTATCTTCAAATTTATCACATTAT  360

Query  371  GGTCCATCAGTAGATTTAGCTATTTTTCTCTTCACCTTGCTGGTGCTTCTTCAATTTTA  430
            || || ||||||||||||||||| |||||||||| |||||||||||||||||||||||||
Sbjct  361  GGCCCTTCAGTAGATTTAGCTATCTTTCTCTTCATCTTGCTGGTGCTTCTTCAATTTTA  420

Query  431  GGTGCAATTAACTTCATCACAACTATTGTAAATATACGATCAATTGGAATAACAATAGAA  490
            |||||||||||||| || |||||||||||||| |||||||||||||||||||||||||||
Sbjct  421  GGTGCAATTAACTTTATTACAACTATTGTAAACATACGATCAATTGGAATAACAATAGAA  480

Query  491  CGAATACCATTATTTGTTTGATCTGTTTTAATTACAGCTATTTTACTACTTCTATCTTTA  550
            || |||||||||||||||||||||||||||||||||||||||||| ||||| ||||||||
Sbjct  481  CGTATACCATTATTTGTTTGATCTGTTTTAATTACAGCTATTTTATTACTTTTATCTTTA  540

Query  551  CCTGTATTAGCAGGTGCCATTACAATATTATTAACTGATCGAAACTTTAACACATCATTT  610
            ||||||||||||||||||||||||||||| |||||||||||||||||||| |||||||||
Sbjct  541  CCTGTATTAGCAGGTGCCATTACAATATTGTTAACTGATCGAAACTTTAATACATCATTT  600

Query  611  TTTGATCCTTCAGGAGGAGGAGATCCCATTTTATATCAACACTTATTT  658
            |||||||||||||| ||||||||||| |||||||||||||| ||||||
Sbjct  601  TTTGATCCTTCAGGGGGAGGAGATCCAATTTTATATCAACATTTATTT  648
```

>Rhipicephalus sanguineus sensu lato 'temperate lineage' isolate S344 cytochrome c oxidase subunit I (COI) gene, partial cds; mitochondrial
Sequence ID: MF426011.1 Length: 809
Range 1: 3 to 644

Score:1031 bits(558), Expect:0.0,
Identities:614/642(96%), Gaps:0/642(0%), Strand: Plus/Plus

```
Query  17   TTTGGAGCATGATCAGGTATATTAGGACTTAGTATAAGAATATTAATTCGTATAGAATTA  76
            ||||||||||||||||||||||||||||||||||||||||||||||||||||||||||||
Sbjct  3    TTTGGAGCATGATCAGGTATATTAGGACTTAGTATAAGAATATTAATTCGTATAGAATTA  62

Query  77   GGACAACCTGGAACTTTAATTGGAAATGATCAGATTTATAATGTAATCGTAACAGCACAT  136
            ||||||||||||||||||||||||||||||||||||||||||||||||||||||||||| 
Sbjct  63   GGACAACCTGGAACTTTAATTGGAAATGATCAGATTTATAATGTAATCGTAACAGCACAC  122

Query  137  GCATTTATTATAATTTTCTTTATAGTTATACCAATCATAATCGGAGGGTTCGGAAATTGA  196
            ||||||||||||||||| ||||||||||||||||||||||||||||| || |||||||||
Sbjct  123  GCATTTATTATAATTTTTTTTATAGTTATACCAATCATAATCGGAGGATTTGGAAATTGA  182

Query  197  TTAGTTCCTATTATATTAGGTGCCCCAGATATAGCATTTCCACGAATAAATAATATAAGA  256
            ||||| ||||||||||||||||||||||||||||||||||||||||||||||||||||||
Sbjct  183  TTAGTCCCTATTATATTAGGTGCCCCAGATATAGCATTTCCACGAATAAATAATATAAGA  242

Query  257  TTTTGACTTTACCCCCCTCATTATTTTTATTGATTAACTCTTCATTAATTGAATCCGGA  316
            |||||||||||||| || ||| |||||||||| ||||||||||||||||||||||| |||
```

```
Sbjct  243   TTTTGACTTTTACCTCCTTCACTATTTTTATTAATTAACTCTTCATTAATTGAATCTGGA  302

Query  317   GCTGGAACAGGATGAACAGTTTATCCCCCTCTATCTTCAAATTTATCACATTATGGTCCA  376
             ||||| ||||||||||||||||| || ||||||||||||||||||||||||||||| ||
Sbjct  303   GCTGGTACAGGATGAACAGTTTACCCTCCTCTATCTTCAAATTTATCACATTATGGCCCT  362

Query  377   TCAGTAGATTTAGCTATTTTTTCTCTTCACCTTGCTGGTGCTTCTTCAATTTTAGGTGCA  436
             ||||||||||||||||| ||||||||||| ||||||||||||||||||||||||||||||
Sbjct  363   TCAGTAGATTTAGCTATCTTTTCTCTTCATCTTGCTGGTGCTTCTTCAATTTTAGGTGCA  422

Query  437   ATTAACTTCATCACAACTATTGTAAATATACGATCAATTGGAATAACAATAGAACGAATA  496
             |||||||| || |||||||||||||| ||||||||||||||||||||||||||||| |||
Sbjct  423   ATTAACTTTATTACAACTATTGTAAACATACGATCAATTGGAATAACAATAGAACGTATA  482

Query  497   CCATTATTTGTTTGATCTGTTTTAATTACAGCTATTTTACTACTTCTATCTTTACCTGTA  556
             ||||||||||||||||||||||||||||||||||||||| ||||| ||||||||||||||
Sbjct  483   CCATTATTTGTTTGATCTGTTTTAATTACAGCTATTTTATTACTTTTATCTTTACCTGTA  542

Query  557   TTAGCAGGTGCCATTACAATATTATTAACTGATCGAAACTTTAACACATCATTTTTTGAT  616
             ||||||||||||||||||||||| |||||||||||||||||||| |||||||||||||||
Sbjct  543   TTAGCAGGTGCCATTACAATATTGTTAACTGATCGAAACTTTAATACATCATTTTTTGAT  602

Query  617   CCTTCAGGAGGAGGAGATCCCATTTTATATCAACACTTATTT  658
             |||||||| ||||||||||| |||||||||||||| ||||||
Sbjct  603   CCTTCAGGGGGAGGAGATCCAATTTTATATCAACATTTATTT  644
```

>Rhipicephalus sanguineus sensu lato 'temperate lineage' isolate S475 cytochrome c oxidase subunit I (COI) gene, partial cds; mitochondrial
Sequence ID: MF426014.1 Length: 649
Range 1: 28 to 649

Score:1022 bits(553), Expect:0.0,
Identities:599/622(96%), Gaps:0/622(0%), Strand: Plus/Plus

```
Query  1     AACAATATATTTAATTTTTGGAGCATGATCAGGTATATTAGGACTTAGTATAAGAATATT  60
             ||||||||||||||||||||||||||||||||||||||||||||||||||||||||||||
Sbjct  28    AACAATATATTTAATTTTTGGAGCATGATCAGGTATATTAGGACTTAGTATAAGAATATT  87

Query  61    AATTCGTATAGAATTAGGACAACCTGGAACTTTAATTGGAAATGATCAGATTTATAATGT  120
             ||||||||||||||||||||||||||||||||||||||||||||||||||||||||||||
Sbjct  88    AATTCGTATAGAATTAGGACAACCTGGAACTTTAATTGGAAATGATCAGATTTATAATGT  147

Query  121   AATCGTAACAGCACATGCATTTATTATAATTTCTTTATAGTTATACCAATCATAATCGG  180
             |||||||||||||||||||||||||||||||| |||||||||||||||||||||||||||
Sbjct  148   AATCGTAACAGCACATGCATTTATTATAATTTTTTTATAGTTATACCAATCATAATCGG  207

Query  181   AGGGTTCGGAAATTGATTAGTTCCTATTATATTAGGTGCCCCAGATATAGCATTTCCACG  240
             ||| || |||||||||||||| ||||| ||||| ||||||||||||||||||||||||||
Sbjct  208   AGGATTTGGAAATTGATTAGTCCCTATCATATTGGGTGCCCCAGATATAGCATTTCCACG  267

Query  241   AATAAATAATATAAGATTTTGACTTTTACCCCCTCATTATTTTTATTGATTAACTCTTC  300
```

```
            |||||||||||||||||||||||||||||| || ||| |||||||||| |||||||||||
Sbjct  268  AATAAATAATATAAGATTTTGACTTTTACCTCCTTCACTATTTTTATTAATTAACTCTTC  327

Query  301  ATTAATTGAATCCGGAGCTGGAACAGGATGAACAGTTTATCCCCCTCTATCTTCAAATTT  360
            |||||||||||| |||||||| ||||||||||||||||| || |||||||||||||||||
Sbjct  328  ATTAATTGAATCTGGAGCTGGTACAGGATGAACAGTTTACCCTCCTCTATCTTCAAATTT  387

Query  361  ATCACATTATGGTCCATCAGTAGATTTAGCTATTTTTTCTCTTCACCTTGCTGGTGCTTC  420
            ||||||||||||||| ||||||||||||||||||||||||||||| ||||||||||||||
Sbjct  388  ATCACATTATGGTCCTTCAGTAGATTTAGCTATTTTTTCTCTTCATCTTGCTGGTGCTTC  447

Query  421  TTCAATTTTAGGTGCAATTAACTTCATCACAACTATTGTAAATATACGATCAATTGGAAT  480
            |||||||||||||||||||||||| || |||||||||||||| |||||||||||||||||
Sbjct  448  TTCAATTTTAGGTGCAATTAACTTTATTACAACTATTGTAAACATACGATCAATTGGAAT  507

Query  481  AACAATAGAACGAATACCATTATTTGTTTGATCTGTTTTAATTACAGCTATTTTACTACT  540
            |||||||||||| |||||||||||||||||||||||||||||||||||||||||||||||
Sbjct  508  AACAATAGAACGTATACCATTATTTGTTTGATCTGTTTTAATTACAGCTATTTTACTACT  567

Query  541  TCTATCTTTACCTGTATTAGCAGGTGCCATTACAATATTATTAACTGATCGAAACTTTAA  600
            | ||||||||||||||||||||||||||||||||||||| ||||||||||||||||||||
Sbjct  568  TTTATCTTTACCTGTATTAGCAGGTGCCATTACAATATTGTTAACTGATCGAAACTTTAA  627

Query  601  CACATCATTTTTTGATCCTTCA  622
             |||||||||||||||||||||
Sbjct  628  TACATCATTTTTTGATCCTTCA  649
```

>Rhipicephalus sanguineus isolate LIC5424B cytochrome oxidase subunit 1 (COI) gene, partial cds; mitochondrial
Sequence ID: KX383818.1 Length: 768
>Rhipicephalus sanguineus isolate LIC5554A cytochrome oxidase subunit 1 (COI) gene, partial cds; mitochondrial
Sequence ID: KX383819.1 Length: 768
Range 1: 1 to 642

Score:1020 bits(552), Expect:0.0,
Identities:612/642(95%), Gaps:0/642(0%), Strand: Plus/Plus

```
Query  17   TTTGGAGCATGATCAGGTATATTAGGACTTAGTATAAGAATATTAATTCGTATAGAATTA  76
            ||||||||||||||||||||||||||||||||||||||||||||||||||||||||||||
Sbjct  1    TTTGGAGCATGATCAGGTATATTAGGACTTAGTATAAGAATATTAATTCGTATAGAATTA  60

Query  77   GGACAACCTGGAACTTTAATTGGAAATGATCAGATTTATAATGTAATCGTAACAGCACAT  136
            ||||||||||||||||||||||||||||||||||||||||||||||||||||||||||||
Sbjct  61   GGACAACCTGGAACTTTAATTGGAAATGATCAGATTTATAATGTAATCGTAACAGCACAT  120

Query  137  GCATTTATTATAATTTTCTTTATAGTTATACCAATCATAATCGGAGGGTTCGGAAATTGA  196
            ||||||||||||||||| ||||||||||||||||||||||||||||| || |||||||||
Sbjct  121  GCATTTATTATAATTTTTTTTATAGTTATACCAATCATAATCGGAGGATTTGGAAATTGA  180

Query  197  TTAGTTCCTATTATATTAGGTGCCCCAGATATAGCATTTCCACGAATAAATAATATAAGA  256
```

```
                ||||| ||||| ||||| ||||||||||||||||||||||||||||||||||||||||||
Sbjct  181  TTAGTCCCTATCATATTGGGTGCCCCAGATATAGCATTTCCACGAATAAATAATATAAGA  240

Query  257  TTTTGACTTTTACCCCCCTCATTATTTTTATTGATTAACTCTTCATTAATTGAATCCGGA  316
            |||||||||||||| || ||| |||||||||| ||||||||||||||||||||||| |||
Sbjct  241  TTTTGACTTTTACCTCCTTCACTATTTTTATTAATTAACTCTTCATTAATTGAATCTGGA  300

Query  317  GCTGGAACAGGATGAACAGTTTATCCCCCTCTATCTTCAAATTTATCACATTATGGTCCA  376
            ||||| ||||||||||||||||| || ||||||||||||||||||||||||||||| || 
Sbjct  301  GCTGGTACAGGATGAACAGTTTACCCTCCTCTATCTTCAAATTTATCACATTATGGCCCT  360

Query  377  TCAGTAGATTTAGCTATTTTTTCTCTTCACCTTGCTGGTGCTTCTTCAATTTTAGGTGCA  436
            ||||||||||||||||| ||||||||||| ||||||||||||||||||||||||||||||
Sbjct  361  TCAGTAGATTTAGCTATCTTTTCTCTTCATCTTGCTGGTGCTTCTTCAATTTTAGGTGCA  420

Query  437  ATTAACTTCATCACAACTATTGTAAATATACGATCAATTGGAATAACAATAGAACGAATA  496
            |||||||| || |||||||||||||| ||||| ||||||||||||||||||||||| |||
Sbjct  421  ATTAACTTTATTACAACTATTGTAAACATACGGTCAATTGGAATAACAATAGAACGTATA  480

Query  497  CCATTATTTGTTTGATCTGTTTTAATTACAGCTATTTTACTACTTCTATCTTTACCTGTA  556
            ||||||||||||||||||||||||||||||||||||||| ||||| ||||||||||||||
Sbjct  481  CCATTATTTGTTTGATCTGTTTTAATTACAGCTATTTTATTACTTTTATCTTTACCTGTA  540

Query  557  TTAGCAGGTGCCATTACAATATTATTAACTGATCGAAACTTTAACACATCATTTTTTGAT  616
            ||||||||||||||||||||||| |||||||||||||||||||| |||||||||||||||
Sbjct  541  TTAGCAGGTGCCATTACAATATTGTTAACTGATCGAAACTTTAATACATCATTTTTTGAT  600

Query  617  CCTTCAGGAGGAGGAGATCCCATTTTATATCAACACTTATTT  658
            |||||||| ||||||||||| |||||||||||||| ||||||
Sbjct  601  CCTTCAGGGGGAGGAGATCCAATTTTATATCAACATTTATTT  642
```

>Rhipicephalus sanguineus sensu lato 'temperate lineage' isolate CR1568 cytochrome c oxidase subunit I (COI) gene, partial cds; mitochondrial
Sequence ID: MF426000.1 Length: 635
Range 1: 14 to 635

Score:1016 bits(550), Expect:0.0,
Identities:598/622(96%), Gaps:0/622(0%), Strand: Plus/Plus

```
Query  1    AACAATATATTTAATTTTTGGAGCATGATCAGGTATATTAGGACTTAGTATAAGAATATT  60
            ||||||||||||||||||||||||||||||||||||||||||||||||||||||||||||
Sbjct  14   AACAATATATTTAATTTTTGGAGCATGATCAGGTATATTAGGACTTAGTATAAGAATATT  73

Query  61   AATTCGTATAGAATTAGGACAACCTGGAACTTTAATTGGAAATGATCAGATTTATAATGT  120
            ||||||||||||||||||||||||||||||||||||||||||||||||||||||||||||
Sbjct  74   AATTCGTATAGAATTAGGACAACCTGGAACTTTAATTGGAAATGATCAGATTTATAATGT  133

Query  121  AATCGTAACAGCACATGCATTTATTATAATTTTCTTTATAGTTATACCAATCATAATCGG  180
            ||||||||||||||||||||||||||||||||| ||||||||||||||||||||||||||
Sbjct  134  AATCGTAACAGCACATGCATTTATTATAATTTTTTTTATAGTTATACCAATCATAATCGG  193
```

```
Query  181  AGGGTTCGGAAATTGATTAGTTCCTATTATATTAGGTGCCCCAGATATAGCATTTCCACG  240
            ||| || |||||||||||||| ||||| ||||| ||||||||||||||||||||||||||
Sbjct  194  AGGATTTGGAAATTGATTAGTCCCTATCATATTGGGTGCCCCAGATATAGCATTTCCACG  253

Query  241  AATAAATAATATAAGATTTTGACTTTTACCCCCCTCATTATTTTTATTGATTAACTCTTC  300
            |||||||||||||||||||||||||||||| || ||| |||||||||| |||||||||||
Sbjct  254  AATAAATAATATAAGATTTTGACTTTTACCTCCTTCACTATTTTTATTAATTAACTCTTC  313

Query  301  ATTAATTGAATCCGGAGCTGGAACAGGATGAACAGTTTATCCCCCTCTATCTTCAAATTT  360
            |||||||||||| |||||||| ||||||||||||||||| || |||||||||||||||||
Sbjct  314  ATTAATTGAATCTGGAGCTGGTACAGGATGAACAGTTTACCCTCCTCTATCTTCAAATTT  373

Query  361  ATCACATTATGGTCCATCAGTAGATTTAGCTATTTTTTCTCTTCACCTTGCTGGTGCTTC  420
            ||||||||||||||| ||||||||||||||||||||||||||||||| ||||||||||||
Sbjct  374  ATCACATTATGGTCCTTCAGTAGATTTAGCTATTTTTTCTCTTCATCTTGCTGGTGCTTC  433

Query  421  TTCAATTTTAGGTGCAATTAACTTCATCACAACTATTGTAAATATACGATCAATTGGAAT  480
            |||||||||||||||||||||||| || |||||||||||||| |||||||||||||||||
Sbjct  434  TTCAATTTTAGGTGCAATTAACTTTATTACAACTATTGTAAACATACGATCAATTGGAAT  493

Query  481  AACAATAGAACGAATACCATTATTTGTTTGATCTGTTTTAATTACAGCTATTTTACTACT  540
            |||||||||||| |||||||||||||||||||||||||||||||||||||||||| ||||
Sbjct  494  AACAATAGAACGTATACCATTATTTGTTTGATCTGTTTTAATTACAGCTATTTTATTACT  553

Query  541  TCTATCTTTACCTGTATTAGCAGGTGCCATTACAATATTATTAACTGATCGAAACTTTAA  600
            | ||||||||||||||||||||||||||||||||||||| ||||||||||||||||||||
Sbjct  554  TTTATCTTTACCTGTATTAGCAGGTGCCATTACAATATTGTTAACTGATCGAAACTTTAA  613

Query  601  CACATCATTTTTTGATCCTTCA  622
             |||||||||||||||||||||
Sbjct  614  TACATCATTTTTTGATCCTTCA  635
```

>Rhipicephalus sanguineus sensu lato 'temperate lineage' isolate SF3003 cytochrome c oxidase subunit I (COI) gene, partial cds; mitochondrial
Sequence ID: MF425988.1 Length: 654
Range 1: 1 to 640

Score:1016 bits(550), Expect:0.0,
Identities:610/640(95%), Gaps:0/640(0%), Strand: Plus/Plus

```
Query  17   TTTGGAGCATGATCAGGTATATTAGGACTTAGTATAAGAATATTAATTCGTATAGAATTA  76
            ||||||||||||||||||||||||||||||||||||||||||||||||||||||||||||
Sbjct  1    TTTGGAGCATGATCAGGTATATTAGGACTTAGTATAAGAATATTAATTCGTATAGAATTA  60

Query  77   GGACAACCTGGAACTTTAATTGGAAATGATCAGATTTATAATGTAATCGTAACAGCACAT  136
            ||||||||||||||||||||||||||||||||||||||||||||||||||||||||||||
Sbjct  61   GGACAACCTGGAACTTTAATTGGAAATGATCAGATTTATAATGTAATCGTAACAGCACAT  120

Query  137  GCATTTATTATAATTTTCTTTATAGTTATACCAATCATAATCGGAGGGTTCGGAAATTGA  196
            ||||||||||||||||| ||||||||||||||||||||||||||||| || |||||||||
Sbjct  121  GCATTTATTATAATTTTTTTTATAGTTATACCAATCATAATCGGAGGATTTGGAAATTGA  180
```

```
Query  197  TTAGTTCCTATTATATTAGGTGCCCCAGATATAGCATTTCCACGAATAAATAATATAAGA  256
            ||||| ||||| ||||||||||||||||||||||||||||||||||||||||||||||||
Sbjct  181  TTAGTCCCTATCATATTAGGTGCCCCAGATATAGCATTTCCACGAATAAATAATATAAGA  240

Query  257  TTTTGACTTTTACCCCCCTCATTATTTTTATTGATTAACTCTTCATTAATTGAATCCGGA  316
            |||||||||||||| || ||| |||||||||| ||||||||||||||||||||||| |||
Sbjct  241  TTTTGACTTTTACCTCCTTCACTATTTTTATTAATTAACTCTTCATTAATTGAATCTGGA  300

Query  317  GCTGGAACAGGATGAACAGTTTATCCCCCTCTATCTTCAAATTTATCACATTATGGTCCA  376
            ||||| ||||||||||||||||| || ||||||||||||||||||||||||||||| || 
Sbjct  301  GCTGGTACAGGATGAACAGTTTACCCTCCTCTATCTTCAAATTTATCACATTATGGCCCT  360

Query  377  TCAGTAGATTTAGCTATTTTTTCTCTTCACCTTGCTGGTGCTTCTTCAATTTTAGGTGCA  436
            ||||||||||||||||| ||||||||||| ||||||||||||||||||||||||||||||
Sbjct  361  TCAGTAGATTTAGCTATCTTTTCTCTTCATCTTGCTGGTGCTTCTTCAATTTTAGGTGCA  420

Query  437  ATTAACTTCATCACAACTATTGTAAATATACGATCAATTGGAATAACAATAGAACGAATA  496
            |||||||| || |||||||||||||| ||||||||||||||||||||||||||||| |||
Sbjct  421  ATTAACTTTATTACAACTATTGTAAACATACGATCAATTGGAATAACAATAGAACGTATA  480

Query  497  CCATTATTTGTTTGATCTGTTTTAATTACAGCTATTTTACTACTTCTATCTTTACCTGTA  556
            ||||||||||||||||||||||||||||||||||||||| ||||| ||||||||||||||
Sbjct  481  CCATTATTTGTTTGATCTGTTTTAATTACAGCTATTTTATTACTTTTATCTTTACCTGTA  540

Query  557  TTAGCAGGTGCCATTACAATATTATTAACTGATCGAAACTTTAACACATCATTTTTTGAT  616
            ||||||||||||||||||||||| |||||||||||||||||||| |||||||||||||||
Sbjct  541  TTAGCAGGTGCCATTACAATATTGTTAACTGATCGAAACTTTAATACATCATTTTTTGAT  600

Query  617  CCTTCAGGAGGAGGAGATCCCATTTTATATCAACACTTAT  656
            |||||||| |||||| |||| ||||||||||| || ||||
Sbjct  601  CCTTCAGGGGGAGGACATCCAATTTTATATCACCATTTAT  640
```

>Rhipicephalus sanguineus isolate LIC5533B cytochrome oxidase subunit 1 (COI) gene, partial cds; mitochondrial
Sequence ID: KX383811.1 Length: 764
>Rhipicephalus sanguineus isolate LIC5533C cytochrome oxidase subunit 1 (COI) gene, partial cds; mitochondrial
Sequence ID: KX383812.1 Length: 764
Range 1: 1 to 638

Score:1013 bits(548), Expect:0.0,
Identities:608/638(95%), Gaps:0/638(0%), Strand: Plus/Plus

```
Query  21   GAGCATGATCAGGTATATTAGGACTTAGTATAAGAATATTAATTCGTATAGAATTAGGAC  80
            ||||||||||||||||||||||||||||||||||||||||||||||||||||||||||||
Sbjct  1    GAGCATGATCAGGTATATTAGGACTTAGTATAAGAATATTAATTCGTATAGAATTAGGAC  60

Query  81   AACCTGGAACTTTAATTGGAAATGATCAGATTTATAATGTAATCGTAACAGCACATGCAT  140
            ||||||||||||||||||||||||||||||||||||||||||||||||||||||||||||
Sbjct  61   AACCTGGAACTTTAATTGGAAATGATCAGATTTATAATGTAATCGTAACAGCACATGCAT  120
```

```
Query  141  TTATTATAATTTTCTTTATAGTTATACCAATCATAATCGGAGGGTTCGGAAATTGATTAG  200
            ||||||||||||| ||||||||||||||||||||||||||||| || |||||||||||||
Sbjct  121  TTATTATAATTTTTTTTATAGTTATACCAATCATAATCGGAGGATTTGGAAATTGATTAG  180

Query  201  TTCCTATTATATTAGGTGCCCCAGATATAGCATTTCCACGAATAAATAATATAAGATTTT  260
            | ||||| ||||| ||||||||||||||||||||||||||||||||||||||||||||||
Sbjct  181  TCCCTATCATATTGGGTGCCCCAGATATAGCATTTCCACGAATAAATAATATAAGATTTT  240

Query  261  GACTTTTACCCCCCTCATTATTTTTATTGATTAACTCTTCATTAATTGAATCCGGAGCTG  320
            |||||||||| || ||| |||||||||| ||||||||||||||||||||||| |||||||
Sbjct  241  GACTTTTACCTCCTTCACTATTTTTATTAATTAACTCTTCATTAATTGAATCTGGAGCTG  300

Query  321  GAACAGGATGAACAGTTTATCCCCCTCTATCTTCAAATTTATCACATTATGGTCCATCAG  380
            | ||||||||||||||||| || ||||||||||||||||||||||||||||| || ||||
Sbjct  301  GTACAGGATGAACAGTTTACCCTCCTCTATCTTCAAATTTATCACATTATGGCCCTTCAG  360

Query  381  TAGATTTAGCTATTTTTTCTCTTCACCTTGCTGGTGCTTCTTCAATTTTAGGTGCAATTA  440
            ||||||||||||| ||||||||||| ||||||||||||||||||||||||||||||||||
Sbjct  361  TAGATTTAGCTATCTTTTCTCTTCATCTTGCTGGTGCTTCTTCAATTTTAGGTGCAATTA  420

Query  441  ACTTCATCACAACTATTGTAAATATACGATCAATTGGAATAACAATAGAACGAATACCAT  500
            |||| || ||||||||||||||| ||||| |||||||||||||||||||||| |||||||
Sbjct  421  ACTTTATTACAACTATTGTAAACATACGGTCAATTGGAATAACAATAGAACGTATACCAT  480

Query  501  TATTTGTTTGATCTGTTTTAATTACAGCTATTTTACTACTTCTATCTTTACCTGTATTAG  560
            ||||||||||||||||||||||||||||||||||| ||||| ||||||||||||||||||
Sbjct  481  TATTTGTTTGATCTGTTTTAATTACAGCTATTTTATTACTTTTATCTTTACCTGTATTAG  540

Query  561  CAGGTGCCATTACAATATTATTAACTGATCGAAACTTTAACACATCATTTTTTGATCCTT  620
            ||||||||||||||||||| |||||||||||||||||||| |||||||||||||||||||
Sbjct  541  CAGGTGCCATTACAATATTGTTAACTGATCGAAACTTTAATACATCATTTTTTGATCCTT  600

Query  621  CAGGAGGAGGAGATCCCATTTTATATCAACACTTATTT  658
            |||| |||||||||||| |||||||||||||| ||||||
Sbjct  601  CAGGGGGAGGAGATCCAATTTTATATCAACATTTATTT  638
```

>Rhipicephalus sanguineus isolate LIC4736B cytochrome oxidase subunit 1 (COI) gene, partial cds; mitochondrial
Sequence ID: KX383807.1 Length: 763
>Rhipicephalus sanguineus isolate LIC4736C cytochrome oxidase subunit 1 (COI) gene, partial cds; mitochondrial
Sequence ID: KX383808.1 Length: 763
>Rhipicephalus sanguineus isolate LIC4736F cytochrome oxidase subunit 1 (COI) gene, partial cds; mitochondrial
Sequence ID: KX383810.1 Length: 763
>Rhipicephalus sanguineus isolate LIC5535C cytochrome oxidase subunit 1 (COI) gene, partial cds; mitochondrial
Sequence ID: KX383813.1 Length: 763
Range 1: 1 to 637

Score:1011 bits(547), Expect:0.0,
Identities:607/637(95%), Gaps:0/637(0%), Strand: Plus/Plus

```
Query  22   AGCATGATCAGGTATATTAGGACTTAGTATAAGAATATTAATTCGTATAGAATTAGGACA  81
            ||||||||||||||||||||||||||||||||||||||||||||||||||||||||||||
Sbjct  1    AGCATGATCAGGTATATTAGGACTTAGTATAAGAATATTAATTCGTATAGAATTAGGACA  60

Query  82   ACCTGGAACTTTAATTGGAAATGATCAGATTTATAATGTAATCGTAACAGCACATGCATT  141
            ||||||||||||||||||||||||||||||||||||||||||||||||||||||||||||
Sbjct  61   ACCTGGAACTTTAATTGGAAATGATCAGATTTATAATGTAATCGTAACAGCACATGCATT  120

Query  142  TATTATAATTTTCTTTATAGTTATACCAATCATAATCGGAGGGTTCGGAAATTGATTAGT  201
            |||||||||||| ||||||||||||||||||||||||||||| || ||||||||||||||
Sbjct  121  TATTATAATTTTTTTTATAGTTATACCAATCATAATCGGAGGATTTGGAAATTGATTAGT  180

Query  202  TCCTATTATATTAGGTGCCCCAGATATAGCATTTCCACGAATAAATAATATAAGATTTTG  261
             ||||| ||||| |||||||||||||||||||||||||||||||||||||||||||||||
Sbjct  181  CCCTATCATATTGGGTGCCCCAGATATAGCATTTCCACGAATAAATAATATAAGATTTTG  240

Query  262  ACTTTTACCCCCCTCATTATTTTTATTGATTAACTCTTCATTAATTGAATCCGGAGCTGG  321
            ||||||||| || ||| |||||||||| ||||||||||||||||||||||| ||||||||
Sbjct  241  ACTTTTACCTCCTTCACTATTTTTATTAATTAACTCTTCATTAATTGAATCTGGAGCTGG  300

Query  322  AACAGGATGAACAGTTTATCCCCCTCTATCTTCAAATTTATCACATTATGGTCCATCAGT  381
             ||||||||||||||||| || ||||||||||||||||||||||||||||| || |||||
Sbjct  301  TACAGGATGAACAGTTTACCCTCCTCTATCTTCAAATTTATCACATTATGGCCCTTCAGT  360

Query  382  AGATTTAGCTATTTTTTCTCTTCACCTTGCTGGTGCTTCTTCAATTTTAGGTGCAATTAA  441
            |||||||||||| ||||||||||| |||||||||||||||||||||||||||||||||||
Sbjct  361  AGATTTAGCTATCTTTTCTCTTCATCTTGCTGGTGCTTCTTCAATTTTAGGTGCAATTAA  420

Query  442  CTTCATCACAACTATTGTAAATATACGATCAATTGGAATAACAATAGAACGAATACCATT  501
            ||| || |||||||||||||| ||||| ||||||||||||||||||||||| ||||||||
Sbjct  421  CTTTATTACAACTATTGTAAACATACGGTCAATTGGAATAACAATAGAACGTATACCATT  480

Query  502  ATTTGTTTGATCTGTTTTAATTACAGCTATTTTACTACTTCTATCTTTACCTGTATTAGC  561
            |||||||||||||||||||||||||||||||||| ||||| |||||||||||||||||||
Sbjct  481  ATTTGTTTGATCTGTTTTAATTACAGCTATTTTATTACTTTTATCTTTACCTGTATTAGC  540

Query  562  AGGTGCCATTACAATATTATTAACTGATCGAAACTTTAACACATCATTTTTTGATCCTTC  621
            |||||||||||||||||| |||||||||||||||||||| ||||||||||||||||||||
Sbjct  541  AGGTGCCATTACAATATTGTTAACTGATCGAAACTTTAATACATCATTTTTTGATCCTTC  600

Query  622  AGGAGGAGGAGATCCCATTTTATATCAACACTTATTT  658
            ||| ||||||||||| |||||||||||||| ||||||
Sbjct  601  AGGGGGAGGAGATCCAATTTTATATCAACATTTATTT  637
```

>Rhipicephalus sanguineus voucher Rs7 cytochrome oxidase subunit I (COI) gene, partial cds; mitochondrial
Sequence ID: KU568516.1 Length: 656
Range 1: 27 to 656

```
Score:1009 bits(546), Expect:0.0,
Identities:602/630(96%),  Gaps:0/630(0%), Strand: Plus/Plus

Query  1    AACAATATATTTAATTTTTGGAGCATGATCAGGTATATTAGGACTTAGTATAAGAATATT  60
            ||||||||||||||||||||||||||||||||||||||||||||||||||||||||||||
Sbjct  27   AACAATATATTTAATTTTTGGAGCATGATCAGGTATATTAGGACTTAGTATAAGAATATT  86

Query  61   AATTCGTATAGAATTAGGACAACCTGGAACTTTAATTGGAAATGATCAGATTTATAATGT  120
            ||||||||||||||||||||||||||||||||||||||||||||||||||||||||||||
Sbjct  87   AATTCGTATAGAATTAGGACAACCTGGAACTTTAATTGGAAATGATCAGATTTATAATGT  146

Query  121  AATCGTAACAGCACATGCATTTATTATAATTTTCTTTATAGTTATACCAATCATAATCGG  180
            ||||||||||||||||||||||||||||||||| ||||||||||||||||||||||||||
Sbjct  147  AATCGTAACAGCACATGCATTTATTATAATTTTTTTTATAGTTATACCAATCATAATCGG  206

Query  181  AGGGTTCGGAAATTGATTAGTTCCTATTATATTAGGTGCCCCAGATATAGCATTTCCACG  240
            ||| || |||||||||||| | ||||| ||||||||||||||||||||||||||||||||
Sbjct  207  AGGATTTGGAAATTGATTAATCCCTATCATATTAGGTGCCCCAGATATAGCATTTCCACG  266

Query  241  AATAAATAATATAAGATTTTGACTTTTACCCCCCTCATTATTTTTATTGATTAACTCTTC  300
            |||||||||||||||||||||||||||||| || ||| |||||||||| |||||||||||
Sbjct  267  AATAAATAATATAAGATTTTGACTTTTACCTCCTTCACTATTTTTATTAATTAACTCTTC  326

Query  301  ATTAATTGAATCCGGAGCTGGAACAGGATGAACAGTTTATCCCCCTCTATCTTCAAATTT  360
            |||||||||||| |||||||| ||||||||||||||||| || |||||||||||||||||
Sbjct  327  ATTAATTGAATCTGGAGCTGGTACAGGATGAACAGTTTACCCTCCTCTATCTTCAAATTT  386

Query  361  ATCACATTATGGTCCATCAGTAGATTTAGCTATTTTTCTCTTCACCTTGCTGGTGCTTC  420
            |||||||||||| || ||||||||||||||||| |||||||||| |||||||||||||||
Sbjct  387  ATCACATTATGGCCCTTCAGTAGATTTAGCTATCTTTCTCTTCATCTTGCTGGTGCTTC  446

Query  421  TTCAATTTAGGTGCAATTAACTTCATCACAACTATTGTAAATATACGATCAATTGGAAT  480
            |||||||||||||||||||||||| || |||||||||||||| |||||||||||||||||
Sbjct  447  TTCAATTTAGGTGCAATTAACTTTATTACAACTATTGTAAACATACGATCAATTGGAAT  506

Query  481  AACAATAGAACGAATACCATTATTTGTTTGATCTGTTTTAATTACAGCTATTTTACTACT  540
            |||||||||||| |||||||||||||||||||||||||||||||||||||||||| ||||
Sbjct  507  AACAATAGAACGTATACCATTATTTGTTTGATCTGTTTTAATTACAGCTATTTTATTACT  566

Query  541  TCTATCTTTACCTGTATTAGCAGGTGCCATTACAATATTATTAACTGATCGAAACTTTAA  600
              ||||||||||||||||||||||||||||||||||||| ||||||||||||||||||||
Sbjct  567  CTTATCTTTACCTGTATTAGCAGGTGCCATTACAATATTGTTAACTGATCGAAACTTTAA  626

Query  601  CACATCATTTTTTGATCCTTCAGGAGGAGG  630
             |||||||||||||| ||||||||||||||
Sbjct  627  TACATCATTTTTTGACCCTTCAGGAGGAGG  656
```

>Rhipicephalus sanguineus isolate LIC4736D cytochrome oxidase subunit 1 (COI) gene, partial cds; mitochondrial
Sequence ID: KX383809.1 Length: 762

Range 1: 1 to 636

Score:1009 bits(546), Expect:0.0,
Identities:606/636(95%), Gaps:0/636(0%), Strand: Plus/Plus

```
Query  23   GCATGATCAGGTATATTAGGACTTAGTATAAGAATATTAATTCGTATAGAATTAGGACAA  82
            ||||||||||||||||||||||||||||||||||||||||||||||||||||||||||||
Sbjct  1    GCATGATCAGGTATATTAGGACTTAGTATAAGAATATTAATTCGTATAGAATTAGGACAA  60

Query  83   CCTGGAACTTTAATTGGAAATGATCAGATTTATAATGTAATCGTAACAGCACATGCATTT  142
            ||||||||||||||||||||||||||||||||||||||||||||||||||||||||||||
Sbjct  61   CCTGGAACTTTAATTGGAAATGATCAGATTTATAATGTAATCGTAACAGCACATGCATTT  120

Query  143  ATTATAATTTTCTTTATAGTTATACCAATCATAATCGGAGGGTTCGGAAATTGATTAGTT  202
            ||||||||||| |||||||||||||||||||||||||||| || ||||||||||||||| 
Sbjct  121  ATTATAATTTTTTTTATAGTTATACCAATCATAATCGGAGGATTTGGAAATTGATTAGTC  180

Query  203  CCTATTATATTAGGTGCCCCAGATATAGCATTTCCACGAATAAATAATATAAGATTTTGA  262
            ||||| ||||| ||||||||||||||||||||||||||||||||||||||||||||||||
Sbjct  181  CCTATCATATTGGGTGCCCCAGATATAGCATTTCCACGAATAAATAATATAAGATTTTGA  240

Query  263  CTTTTACCCCCCTCATTATTTTTATTGATTAACTCTTCATTAATTGAATCCGGAGCTGGA  322
            |||||||| || ||| |||||||||| ||||||||||||||||||||||| |||||||| 
Sbjct  241  CTTTTACCTCCTTCACTATTTTTATTAATTAACTCTTCATTAATTGAATCTGGAGCTGGT  300

Query  323  ACAGGATGAACAGTTTATCCCCCTCTATCTTCAAATTTATCACATTATGGTCCATCAGTA  382
            ||||||||||||||||| || ||||||||||||||||||||||||||||| || ||||||
Sbjct  301  ACAGGATGAACAGTTTACCCTCCTCTATCTTCAAATTTATCACATTATGGCCCTTCAGTA  360

Query  383  GATTTAGCTATTTTTTCTCTTCACCTTGCTGGTGCTTCTTCAATTTTAGGTGCAATTAAC  442
            ||||||||||| ||||||||||| ||||||||||||||||||||||||||||||||||||
Sbjct  361  GATTTAGCTATCTTTTCTCTTCATCTTGCTGGTGCTTCTTCAATTTTAGGTGCAATTAAC  420

Query  443  TTCATCACAACTATTGTAAATATACGATCAATTGGAATAACAATAGAACGAATACCATTA  502
            || || |||||||||||||| ||||| ||||||||||||||||||||||| |||||||||
Sbjct  421  TTTATTACAACTATTGTAAACATACGGTCAATTGGAATAACAATAGAACGTATACCATTA  480

Query  503  TTTGTTTGATCTGTTTTAATTACAGCTATTTTACTACTTCTATCTTTACCTGTATTAGCA  562
            ||||||||||||||||||||||||||||||||| ||||| ||||||||||||||||||||
Sbjct  481  TTTGTTTGATCTGTTTTAATTACAGCTATTTTATTACTTTTATCTTTACCTGTATTAGCA  540

Query  563  GGTGCCATTACAATATTATTAACTGATCGAAACTTTAACACATCATTTTTTGATCCTTCA  622
            ||||||||||||||||| |||||||||||||||||||| |||||||||||||||||||||
Sbjct  541  GGTGCCATTACAATATTGTTAACTGATCGAAACTTTAATACATCATTTTTTGATCCTTCA  600

Query  623  GGAGGAGGAGATCCCATTTTATATCAACACTTATTT  658
            || ||||||||||| |||||||||||||| ||||||
Sbjct  601  GGGGGAGGAGATCCAATTTTATATCAACATTTATTT  636
```

>Rhipicephalus sanguineus isolate LIC4727D cytochrome oxidase subunit 1 (COI) gene, partial cds; mitochondrial

Sequence ID: KX383806.1 Length: 714
Range 1: 1 to 636

Score:1009 bits(546), Expect:0.0,
Identities:606/636(95%), Gaps:0/636(0%), Strand: Plus/Plus

```
Query  23   GCATGATCAGGTATATTAGGACTTAGTATAAGAATATTAATTCGTATAGAATTAGGACAA  82
            ||||||||||||||||||||||||||||||||||||||||||||||||||||||||||||
Sbjct  1    GCATGATCAGGTATATTAGGACTTAGTATAAGAATATTAATTCGTATAGAATTAGGACAA  60

Query  83   CCTGGAACTTTAATTGGAAATGATCAGATTTATAATGTAATCGTAACAGCACATGCATTT  142
            ||||||||||||||||||||||||||||||||||||||||||||||||||||||||||||
Sbjct  61   CCTGGAACTTTAATTGGAAATGATCAGATTTATAATGTAATCGTAACAGCACATGCATTT  120

Query  143  ATTATAATTTTCTTTATAGTTATACCAATCATAATCGGAGGGTTCGGAAATTGATTAGTT  202
            ||||||||||| ||||||||||||||||||||||||||||| || |||||||||||||| 
Sbjct  121  ATTATAATTTTTTTTATAGTTATACCAATCATAATCGGAGGATTTGGAAATTGATTAGTC  180

Query  203  CCTATTATATTAGGTGCCCCAGATATAGCATTTCCACGAATAAATAATATAAGATTTTGA  262
            ||||| ||||| ||||||||||||||||||||||||||||||||||||||||||||||||
Sbjct  181  CCTATCATATTGGGTGCCCCAGATATAGCATTTCCACGAATAAATAATATAAGATTTTGA  240

Query  263  CTTTTACCCCCCTCATTATTTTTATTGATTAACTCTTCATTAATTGAATCCGGAGCTGGA  322
            |||||||| || ||| |||||||||| ||||||||||||||||||||||| |||||||| 
Sbjct  241  CTTTTACCTCCTTCACTATTTTTATTAATTAACTCTTCATTAATTGAATCTGGAGCTGGT  300

Query  323  ACAGGATGAACAGTTTATCCCCCTCTATCTTCAAATTTATCACATTATGGTCCATCAGTA  382
            ||||||||||||||||| || ||||||||||||||||||||||||||||| || ||||||
Sbjct  301  ACAGGATGAACAGTTTACCCTCCTCTATCTTCAAATTTATCACATTATGGCCCTTCAGTA  360

Query  383  GATTTAGCTATTTTTTCTCTTCACCTTGCTGGTGCTTCTTCAATTTTAGGTGCAATTAAC  442
            ||||||||||| ||||||||||| ||||||||||||||||||||||||||||||||||||
Sbjct  361  GATTTAGCTATCTTTTCTCTTCATCTTGCTGGTGCTTCTTCAATTTTAGGTGCAATTAAC  420

Query  443  TTCATCACAACTATTGTAAATATACGATCAATTGGAATAACAATAGAACGAATACCATTA  502
            || || |||||||||||||| ||||| ||||||||||||||||||||||| |||||||||
Sbjct  421  TTTATTACAACTATTGTAAACATACGGTCAATTGGAATAACAATAGAACGTATACCATTA  480

Query  503  TTTGTTTGATCTGTTTTAATTACAGCTATTTTACTACTTCTATCTTTACCTGTATTAGCA  562
            ||||||||||||||||||||||||||||||||| ||||| ||||||||||||||||||||
Sbjct  481  TTTGTTTGATCTGTTTTAATTACAGCTATTTTATTACTTTTATCTTTACCTGTATTAGCA  540

Query  563  GGTGCCATTACAATATTATTAACTGATCGAAACTTTAACACATCATTTTTTGATCCTTCA  622
            ||||||||||||||||| |||||||||||||||||||| |||||||||||||||||||||
Sbjct  541  GGTGCCATTACAATATTGTTAACTGATCGAAACTTTAATACATCATTTTTTGATCCTTCA  600

Query  623  GGAGGAGGAGATCCCATTTTATATCAACACTTATTT  658
            || ||||||||||| |||||||||||||| ||||||
Sbjct  601  GGGGGAGGAGATCCAATTTTATATCAACATTTATTT  636
```

>Rhipicephalus sanguineus isolate LIC4727C cytochrome oxidase subunit 1 (COI)

```
gene, partial cds; mitochondrial
Sequence ID: KX383805.1 Length: 761
Range 1: 1 to 635

Score:1007 bits(545), Expect:0.0,
Identities:605/635(95%),  Gaps:0/635(0%), Strand: Plus/Plus

Query  24    CATGATCAGGTATATTAGGACTTAGTATAAGAATATTAATTCGTATAGAATTAGGACAAC  83
             ||||||||||||||||||||||||||||||||||||||||||||||||||||||||||||
Sbjct  1     CATGATCAGGTATATTAGGACTTAGTATAAGAATATTAATTCGTATAGAATTAGGACAAC  60

Query  84    CTGGAACTTTAATTGGAAATGATCAGATTTATAATGTAATCGTAACAGCACATGCATTTA  143
             ||||||||||||||||||||||||||||||||||||||||||||||||||||||||||||
Sbjct  61    CTGGAACTTTAATTGGAAATGATCAGATTTATAATGTAATCGTAACAGCACATGCATTTA  120

Query  144   TTATAATTTTCTTTATAGTTATACCAATCATAATCGGAGGGTTCGGAAATTGATTAGTTC  203
             |||||||||| ||||||||||||||||||||||||||||| || |||||||||||||| |
Sbjct  121   TTATAATTTTTTTTATAGTTATACCAATCATAATCGGAGGATTTGGAAATTGATTAGTCC  180

Query  204   CTATTATATTAGGTGCCCCAGATATAGCATTTCCACGAATAAATAATATAAGATTTTGAC  263
             |||| ||||| |||||||||||||||||||||||||||||||||||||||||||||||||
Sbjct  181   CTATCATATTGGGTGCCCCAGATATAGCATTTCCACGAATAAATAATATAAGATTTTGAC  240

Query  264   TTTTACCCCCCTCATTATTTTTATTGATTAACTCTTCATTAATTGAATCCGGAGCTGGAA  323
             ||||||| || ||| |||||||||| ||||||||||||||||||||||| |||||||| |
Sbjct  241   TTTTACCTCCTTCACTATTTTTATTAATTAACTCTTCATTAATTGAATCTGGAGCTGGTA  300

Query  324   CAGGATGAACAGTTTATCCCCCTCTATCTTCAAATTTATCACATTATGGTCCATCAGTAG  383
             |||||||||||||||| || ||||||||||||||||||||||||||||| || |||||||
Sbjct  301   CAGGATGAACAGTTTACCCTCCTCTATCTTCAAATTTATCACATTATGGCCCTTCAGTAG  360

Query  384   ATTTAGCTATTTTTTCTCTTCACCTTGCTGGTGCTTCTTCAATTTTAGGTGCAATTAACT  443
             |||||||||| ||||||||||| |||||||||||||||||||||||||||||||||||||
Sbjct  361   ATTTAGCTATCTTTTCTCTTCATCTTGCTGGTGCTTCTTCAATTTTAGGTGCAATTAACT  420

Query  444   TCATCACAACTATTGTAAATATACGATCAATTGGAATAACAATAGAACGAATACCATTAT  503
             | || |||||||||||||| ||||| ||||||||||||||||||||||| ||||||||||
Sbjct  421   TTATTACAACTATTGTAAACATACGGTCAATTGGAATAACAATAGAACGTATACCATTAT  480

Query  504   TTGTTTGATCTGTTTTAATTACAGCTATTTTACTACTTCTATCTTTACCTGTATTAGCAG  563
             |||||||||||||||||||||||||||||||| ||||| |||||||||||||||||||||
Sbjct  481   TTGTTTGATCTGTTTTAATTACAGCTATTTTATTACTTTTATCTTTACCTGTATTAGCAG  540

Query  564   GTGCCATTACAATATTATTAACTGATCGAAACTTTAACACATCATTTTTTGATCCTTCAG  623
             |||||||||||||||| |||||||||||||||||||| ||||||||||||||||||||||
Sbjct  541   GTGCCATTACAATATTGTTAACTGATCGAAACTTTAATACATCATTTTTTGATCCTTCAG  600

Query  624   GAGGAGGAGATCCCATTTTATATCAACACTTATTT  658
             | ||||||||||| |||||||||||||| ||||||
Sbjct  601   GGGGAGGAGATCCAATTTTATATCAACATTTATTT  635
```

```
>Rhipicephalus sanguineus isolate Samont2 cytochrome oxidase subunit I (cox1)
gene, partial cds; mitochondrial
Sequence ID: MH630347.1 Length: 644
Range 1: 20 to 641

Score:1005 bits(544), Expect:0.0,
Identities:596/622(96%),  Gaps:0/622(0%), Strand: Plus/Plus

Query  1    AACAATATATTTAATTTTTGGAGCATGATCAGGTATATTAGGACTTAGTATAAGAATATT  60
            ||||||||||||||||||||||||||||||||||||||||||||||||||||||||||||
Sbjct  20   AACAATATATTTAATTTTTGGAGCATGATCAGGTATATTAGGACTTAGTATAAGAATATT  79

Query  61   AATTCGTATAGAATTAGGACAACCTGGAACTTTAATTGGAAATGATCAGATTTATAATGT  120
            |||| |||||||||||||||||||||||||||||||||||||||||||||||||||||||
Sbjct  80   AATTGGTATAGAATTAGGACAACCTGGAACTTTAATTGGAAATGATCAGATTTATAATGT  139

Query  121  AATCGTAACAGCACATGCATTTATTATAATTTTCTTTATAGTTATACCAATCATAATCGG  180
            ||||||||||||||||||||||||||||||||| ||||||||||||||||||||||||||
Sbjct  140  AATCGTAACAGCACATGCATTTATTATAATTTTTTTTATAGTTATACCAATCATAATCGG  199

Query  181  AGGGTTCGGAAATTGATTAGTTCCTATTATATTAGGTGCCCCAGATATAGCATTTCCACG  240
            ||| || |||||||||||||| ||||| ||||| ||||||||||||||||||||||||||
Sbjct  200  AGGATTTGGAAATTGATTAGTCCCTATCATATTGGGTGCCCCAGATATAGCATTTCCACG  259

Query  241  AATAAATAATATAAGATTTTGACTTTTACCCCCCTCATTATTTTTATTGATTAACTCTTC  300
            |||||||||||||||||||||||||||||| || ||| |||||||||| |||||||||||
Sbjct  260  AATAAATAATATAAGATTTTGACTTTTACCTCCTTCACTATTTTTATTAATTAACTCTTC  319

Query  301  ATTAATTGAATCCGGAGCTGGAACAGGATGAACAGTTTATCCCCCTCTATCTTCAAATTT  360
            |||||||||||| |||||||| ||||||||||||||||| || |||||||||||||||||
Sbjct  320  ATTAATTGAATCTGGAGCTGGTACAGGATGAACAGTTTACCCTCCTCTATCTTCAAATTT  379

Query  361  ATCACATTATGGTCCATCAGTAGATTTAGCTATTTTTTCTCTTCACCTTGCTGGTGCTTC  420
            |||||||||||| || ||||||||||||||||||||||||||||| ||||||||||||||
Sbjct  380  ATCACATTATGGCCCTTCAGTAGATTTAGCTATTTTTTCTCTTCATCTTGCTGGTGCTTC  439

Query  421  TTCAATTTTAGGTGCAATTAACTTCATCACAACTATTGTAAATATACGATCAATTGGAAT  480
            |||||||||||||||||||||||| || |||||||||||||| |||||||||||||||||
Sbjct  440  TTCAATTTTAGGTGCAATTAACTTTATTACAACTATTGTAAACATACGATCAATTGGAAT  499

Query  481  AACAATAGAACGAATACCATTATTTGTTTGATCTGTTTTAATTACAGCTATTTTACTACT  540
            |||||||||||| |||||||||||||||||||||||||||||||||||||||||| ||||
Sbjct  500  AACAATAGAACGTATACCATTATTTGTTTGATCTGTTTTAATTACAGCTATTTTATTACT  559

Query  541  TCTATCTTTACCTGTATTAGCAGGTGCCATTACAATATTATTAACTGATCGAAACTTTAA  600
            | ||||||||||||||||||||||||||||||||||||| ||||||||||||||||||||
Sbjct  560  TTTATCTTTACCTGTATTAGCAGGTGCCATTACAATATTGTTAACTGATCGAAACTTTAA  619

Query  601  CACATCATTTTTTGATCCTTCA  622
             |||||||||||||||||||||
Sbjct  620  TACATCATTTTTTGATCCTTCA  641
```

>Rhipicephalus sanguineus isolate Samont1 cytochrome oxidase subunit I (cox1) gene, partial cds; mitochondrial
Sequence ID: MH630346.1 Length: 644
Range 1: 20 to 641

Score:1005 bits(544), Expect:0.0,
Identities:596/622(96%), Gaps:0/622(0%), Strand: Plus/Plus

```
Query  1    AACAATATATTTAATTTTTGGAGCATGATCAGGTATATTAGGACTTAGTATAAGAATATT  60
            ||||||||||||||||||||||||||||||||||||||||||||||||||||||||||||
Sbjct  20   AACAATATATTTAATTTTTGGAGCATGATCAGGTATATTAGGACTTAGTATAAGAATATT  79

Query  61   AATTCGTATAGAATTAGGACAACCTGGAACTTTAATTGGAAATGATCAGATTTATAATGT  120
            |||| |||||||||||||||||||||||||||||||||||||||||||||||||||||||
Sbjct  80   AATTGGTATAGAATTAGGACAACCTGGAACTTTAATTGGAAATGATCAGATTTATAATGT  139

Query  121  AATCGTAACAGCACATGCATTTATTATAATTTCTTTATAGTTATACCAATCATAATCGG  180
            |||||||||||||||||||||||||||||||| |||||||||||||||||||||||||||
Sbjct  140  AATCGTAACAGCACATGCATTTATTATAATTTTTTTATAGTTATACCAATCATAATCGG  199

Query  181  AGGGTTCGGAAATTGATTAGTTCCTATTATATTAGGTGCCCCAGATATAGCATTTCCACG  240
            ||| || |||||||||||||| ||||| ||||| ||||||||||||||||||||||||||
Sbjct  200  AGGATTTGGAAATTGATTAGTCCCTATCATATTGGGTGCCCCAGATATAGCATTTCCACG  259

Query  241  AATAAATAATATAAGATTTTGACTTTTACCCCCCTCATTATTTTTATTGATTAACTCTTC  300
            |||||||||||||||||||||||||||||| || ||| |||||||||| |||||||||||
Sbjct  260  AATAAATAATATAAGATTTTGACTTTTACCTCCTTCACTATTTTTATTAATTAACTCTTC  319

Query  301  ATTAATTGAATCCGGAGCTGGAACAGGATGAACAGTTTATCCCCCTCTATCTTCAAATTT  360
            |||||||||||| |||||||| ||||||||||||||||| || |||||||||||||||||
Sbjct  320  ATTAATTGAATCTGGAGCTGGTACAGGATGAACAGTTTACCCTCCTCTATCTTCAAATTT  379

Query  361  ATCACATTATGGTCCATCAGTAGATTTAGCTATTTTTTCTCTTCACCTTGCTGGTGCTTC  420
            |||||||||||| || ||||||||||||||||||||||||||||| ||||||||||||||
Sbjct  380  ATCACATTATGGCCCTTCAGTAGATTTAGCTATTTTTTCTCTTCATCTTGCTGGTGCTTC  439

Query  421  TTCAATTTAGGTGCAATTAACTTCATCACAACTATTGTAAATATACGATCAATTGGAAT  480
            ||||||||||||||||||||||| || |||||||||||||| ||||||||||||||||||
Sbjct  440  TTCAATTTAGGTGCAATTAACTTTATTACAACTATTGTAAACATACGATCAATTGGAAT  499

Query  481  AACAATAGAACGAATACCATTATTTGTTTGATCTGTTTTAATTACAGCTATTTTACTACT  540
            |||||||||||| |||||||||||||||||||||||||||||||||||||||||| ||||
Sbjct  500  AACAATAGAACGTATACCATTATTTGTTTGATCTGTTTTAATTACAGCTATTTTATTACT  559

Query  541  TCTATCTTTACCTGTATTAGCAGGTGCCATTACAATATTATTAACTGATCGAAACTTTAA  600
            | ||||||||||||||||||||||||||||||||||||| ||||||||||||||||||||
Sbjct  560  TTTATCTTTACCTGTATTAGCAGGTGCCATTACAATATTGTTAACTGATCGAAACTTTAA  619

Query  601  CACATCATTTTTTGATCCTTCA  622
             |||||||||||||||||||||
Sbjct  620  TACATCATTTTTTGATCCTTCA  641
```

```
>Rhipicephalus sanguineus clone #7 cytochrome oxidase subunit I (COI) gene,
partial cds; mitochondrial
Sequence ID: KX519712.1 Length: 654
Range 1: 3 to 628

Score:1002 bits(542), Expect:0.0,
Identities:598/626(96%),  Gaps:0/626(0%), Strand: Plus/Plus

Query  33   GTATATTAGGACTTAGTATAAGAATATTAATTCGTATAGAATTAGGACAACCTGGAACTT  92
            ||||||||||||||||||||||||||||||||||||||||||||||||||||||||||||
Sbjct  3    GTATATTAGGACTTAGTATAAGAATATTAATTCGTATAGAATTAGGACAACCTGGAACTT  62

Query  93   TAATTGGAAATGATCAGATTTATAATGTAATCGTAACAGCACATGCATTTATTATAATTT  152
            ||||||||||||||||||||||||||||||||||||||||||||||||||||||||||||
Sbjct  63   TAATTGGAAATGATCAGATTTATAATGTAATCGTAACAGCACATGCATTTATTATAATTT  122

Query  153  TCTTTATAGTTATACCAATCATAATCGGAGGGTTCGGAAATTGATTAGTTCCTATTATAT  212
            | ||||||||||||||||||||||||||||| || |||||||||||||| ||||| ||||
Sbjct  123  TTTTTATAGTTATACCAATCATAATCGGAGGATTTGGAAATTGATTAGTCCCTATCATAT  182

Query  213  TAGGTGCCCCAGATATAGCATTTCCACGAATAAATAATATAAGATTTTGACTTTTACCCC  272
            | |||||||||||||||||||||||||||||||||||||||||||||||||||||||| |
Sbjct  183  TGGGTGCCCCAGATATAGCATTTCCACGAATAAATAATATAAGATTTTGACTTTTACCTC  242

Query  273  CCTCATTATTTTTATTGATTAACTCTTCATTAATTGAATCCGGAGCTGGAACAGGATGAA  332
            | ||| |||||||||| ||||||||||||||||||||||| |||||||| ||||||||||
Sbjct  243  CTTCACTATTTTTATTAATTAACTCTTCATTAATTGAATCTGGAGCTGGTACAGGATGAA  302

Query  333  CAGTTTATCCCCCTCTATCTTCAAATTTATCACATTATGGTCCATCAGTAGATTTAGCTA  392
            ||||||| || ||||||||||||||||||||||||||||| || ||||||||||||||||
Sbjct  303  CAGTTTACCCTCCTCTATCTTCAAATTTATCACATTATGGCCCTTCAGTAGATTTAGCTA  362

Query  393  TTTTTTCTCTTCACCTTGCTGGTGCTTCTTCAATTTTAGGTGCAATTAACTTCATCACAA  452
            ||||||||||||| |||||||||||||||||||||||||||||||||||||| || ||||
Sbjct  363  TTTTTTCTCTTCATCTTGCTGGTGCTTCTTCAATTTTAGGTGCAATTAACTTTATTACAA  422

Query  453  CTATTGTAAATATACGATCAATTGGAATAACAATAGAACGAATACCATTATTTGTTTGAT  512
            |||||||||| ||||||||||||||||||||||||||||| |||||||||||||||||||
Sbjct  423  CTATTGTAAACATACGATCAATTGGAATAACAATAGAACGTATACCATTATTTGTTTGAT  482

Query  513  CTGTTTTAATTACAGCTATTTTACTACTTCTATCTTTACCTGTATTAGCAGGTGCCATTA  572
            ||||||||||||||||||||||| ||||| ||||||||||||||||||||||||||||||
Sbjct  483  CTGTTTTAATTACAGCTATTTTATTACTTTTATCTTTACCTGTATTAGCAGGTGCCATTA  542

Query  573  CAATATTATTAACTGATCGAAACTTTAACACATCATTTTTTGATCCTTCAGGAGGAGGAG  632
            ||||||| |||||||||||||||||||| ||||||||||||||||||||||| |||||||
Sbjct  543  CAATATTGTTAACTGATCGAAACTTTAATACATCATTTTTTGATCCTTCAGGGGGAGGAG  602

Query  633  ATCCCATTTTATATCAACACTTATTT  658
            |||| |||||||||||||| ||||||
Sbjct  603  ATCCAATTTTATATCAACATTTATTT  628
```

>Rhipicephalus sanguineus sensu lato 'temperate lineage' isolate CR1575 cytochrome c oxidase subunit I (COI) gene, partial cds; mitochondrial
Sequence ID: MF426002.1 Length: 639
Range 1: 1 to 628

Score:1000 bits(541), Expect:0.0,
Identities:599/628(95%), Gaps:0/628(0%), Strand: Plus/Plus

```
Query  31   AGGTATATTAGGACTTAGTATAAGAATATTAATTCGTATAGAATTAGGACAACCTGGAAC  90
            ||||||||||||||||||||||||||||||||||||||||||||||||||||||||||||
Sbjct  1    AGGTATATTAGGACTTAGTATAAGAATATTAATTCGTATAGAATTAGGACAACCTGGAAC  60

Query  91   TTTAATTGGAAATGATCAGATTTATAATGTAATCGTAACAGCACATGCATTTATTATAAT  150
            ||||||||||||||||||||||||||||||||||||||||||||||||||||||||||||
Sbjct  61   TTTAATTGGAAATGATCAGATTTATAATGTAATCGTAACAGCACATGCATTTATTATAAT  120

Query  151  TTTCTTTATAGTTATACCAATCATAATCGGAGGGTTCGGAAATTGATTAGTTCCTATTAT  210
            ||| ||||||||||||||||||||||||||||| || |||||||||||||| ||||| ||
Sbjct  121  TTTTTTTATAGTTATACCAATCATAATCGGAGGATTTGGAAATTGATTAGTCCCTATCAT  180

Query  211  ATTAGGTGCCCCAGATATAGCATTTCCACGAATAAATAATATAAGATTTTGACTTTTACC  270
            ||| ||||||||||||||||||||||||||||||||||||||||||||||||||||||||
Sbjct  181  ATTGGGTGCCCCAGATATAGCATTTCCACGAATAAATAATATAAGATTTTGACTTTTACC  240

Query  271  CCCCTCATTATTTTTATTGATTAACTCTTCATTAATTGAATCCGGAGCTGGAACAGGATG  330
             || ||| |||||||||| ||||||||||||||||||||||| |||||||| ||||||||
Sbjct  241  TCCTTCACTATTTTTATTAATTAACTCTTCATTAATTGAATCTGGAGCTGGTACAGGATG  300

Query  331  AACAGTTTATCCCCCTCTATCTTCAAATTTATCACATTATGGTCCATCAGTAGATTTAGC  390
            ||||||||| || ||||||||||||||||||||||||||||| || ||||||||||||||
Sbjct  301  AACAGTTTACCCTCCTCTATCTTCAAATTTATCACATTATGGCCCTTCAGTAGATTTAGC  360

Query  391  TATTTTTTCTCTTCACCTTGCTGGTGCTTCTTCAATTTTAGGTGCAATTAACTTCATCAC  450
            ||| ||||||||||| |||||||||||||||||||||||||||||||||||||| || ||
Sbjct  361  TATCTTTTCTCTTCATCTTGCTGGTGCTTCTTCAATTTTAGGTGCAATTAACTTTATTAC  420

Query  451  AACTATTGTAAATATACGATCAATTGGAATAACAATAGAACGAATACCATTATTTGTTTG  510
            |||||||||||| ||||||||||||||||||||||||||||| |||||||||||||||||
Sbjct  421  AACTATTGTAAACATACGATCAATTGGAATAACAATAGAACGTATACCATTATTTGTTTG  480

Query  511  ATCTGTTTTAATTACAGCTATTTTACTACTTCTATCTTTACCTGTATTAGCAGGTGCCAT  570
            ||||||||||||||||||||||||| ||||| ||||||||||||||||||||||||||||
Sbjct  481  ATCTGTTTTAATTACAGCTATTTTATTACTTTTATCTTTACCTGTATTAGCAGGTGCCAT  540

Query  571  TACAATATTATTAACTGATCGAAACTTTAACACATCATTTTTTGATCCTTCAGGAGGAGG  630
            ||||||||| |||||||||||||||||||| ||||||||||||||||||||||| |||||
Sbjct  541  TACAATATTGTTAACTGATCGAAACTTTAATACATCATTTTTTGATCCTTCAGGGGGAGG  600

Query  631  AGATCCCATTTTATATCAACACTTATTT  658
            |||||| |||||||||||||| ||||||
Sbjct  601  AGATCCAATTTTATATCAACATTTATTT  628
```

```
>Rhipicephalus sanguineus isolate Rsan-ML4 cytochrome oxidase subunit I (cox1)
gene, partial cds; mitochondrial
Sequence ID: MG855658.1 Length: 630
Range 1: 1 to 620

Score:990 bits(536), Expect:0.0,
Identities:592/620(95%),  Gaps:0/620(0%), Strand: Plus/Plus

Query  39   TAGGACTTAGTATAAGAATATTAATTCGTATAGAATTAGGACAACCTGGAACTTTAATTG  98
            ||||||||||||||||||||||||||||||||||||||||||||||||||||||||||||
Sbjct  1    TAGGACTTAGTATAAGAATATTAATTCGTATAGAATTAGGACAACCTGGAACTTTAATTG  60

Query  99   GAAATGATCAGATTTATAATGTAATCGTAACAGCACATGCATTTATTATAATTTTCTTTA  158
            ||||||||||||||||||||||||||||||||||||||||||||||||||||||| ||||
Sbjct  61   GAAATGATCAGATTTATAATGTAATCGTAACAGCACATGCATTTATTATAATTTTTTTTA  120

Query  159  TAGTTATACCAATCATAATCGGAGGGTTCGGAAATTGATTAGTTCCTATTATATTAGGTG  218
            ||||||||||||||||||||||||| || |||||||||||||| ||||| ||||| ||||
Sbjct  121  TAGTTATACCAATCATAATCGGAGGATTTGGAAATTGATTAGTCCCTATCATATTGGGTG  180

Query  219  CCCCAGATATAGCATTTCCACGAATAAATAATATAAGATTTTGACTTTTACCCCCCTCAT  278
            |||||||||||||||||||||||||||||||||||||||||||||||||||| || ||| 
Sbjct  181  CCCCAGATATAGCATTTCCACGAATAAATAATATAAGATTTTGACTTTTACCTCCTTCAC  240

Query  279  TATTTTTATTGATTAACTCTTCATTAATTGAATCCGGAGCTGGAACAGGATGAACAGTTT  338
            |||||||||| ||||||||||||||||||||||| |||||||| ||||||||||||||||
Sbjct  241  TATTTTTATTAATTAACTCTTCATTAATTGAATCTGGAGCTGGTACAGGATGAACAGTTT  300

Query  339  ATCCCCCTCTATCTTCAAATTTATCACATTATGGTCCATCAGTAGATTTAGCTATTTTTT  398
            | || ||||||||||||||||||||||||||||| || ||||||||||||||||||||||
Sbjct  301  ACCCTCCTCTATCTTCAAATTTATCACATTATGGCCCTTCAGTAGATTTAGCTATTTTTT  360

Query  399  CTCTTCACCTTGCTGGTGCTTCTTCAATTTTAGGTGCAATTAACTTCATCACAACTATTG  458
            ||||||| |||||||||||||||||||||||||||||||||||||| || ||||||||||
Sbjct  361  CTCTTCATCTTGCTGGTGCTTCTTCAATTTTAGGTGCAATTAACTTTATTACAACTATTG  420

Query  459  TAAATATACGATCAATTGGAATAACAATAGAACGAATACCATTATTTGTTTGATCTGTTT  518
            |||| ||||||||||||||||||||||||||||| |||||||||||||||||||||||||
Sbjct  421  TAAACATACGATCAATTGGAATAACAATAGAACGTATACCATTATTTGTTTGATCTGTTT  480

Query  519  TAATTACAGCTATTTTACTACTTCTATCTTTACCTGTATTAGCAGGTGCCATTACAATAT  578
            ||||||||||||||||| ||||| ||||||||||||||||||||||||||||||||||||
Sbjct  481  TAATTACAGCTATTTTATTACTTTTATCTTTACCTGTATTAGCAGGTGCCATTACAATAT  540

Query  579  TATTAACTGATCGAAACTTTAACACATCATTTTTTGATCCTTCAGGAGGAGGAGATCCCA  638
            | |||||||||||||||||||| ||||||||||||||||||||||| ||||||||||| |
Sbjct  541  TGTTAACTGATCGAAACTTTAATACATCATTTTTTGATCCTTCAGGGGGAGGAGATCCAA  600

Query  639  TTTTATATCAACACTTATTT  658
            ||||||||||||| ||||||
```

```
Sbjct  601  TTTTATATCAACATTTATTT  620
```

>Rhipicephalus sanguineus isolate Rsan-ML3 cytochrome oxidase subunit I (cox1) gene, partial cds; mitochondrial
Sequence ID: MG855657.1 Length: 630
Range 1: 1 to 620

Score:990 bits(536), Expect:0.0,
Identities:592/620(95%), Gaps:0/620(0%), Strand: Plus/Plus

```
Query  39   TAGGACTTAGTATAAGAATATTAATTCGTATAGAATTAGGACAACCTGGAACTTTAATTG  98
            ||||||||||||||||||||||||||||||||||||||||||||||||||||||||||||
Sbjct  1    TAGGACTTAGTATAAGAATATTAATTCGTATAGAATTAGGACAACCTGGAACTTTAATTG  60

Query  99   GAAATGATCAGATTTATAATGTAATCGTAACAGCACATGCATTTATTATAATTTTCTTTA  158
            ||||||||||||||||||||||||||||||||||||||||||||||||||||||| ||||
Sbjct  61   GAAATGATCAGATTTATAATGTAATCGTAACAGCACATGCATTTATTATAATTTTTTTTA  120

Query  159  TAGTTATACCAATCATAATCGGAGGGTTCGGAAATTGATTAGTTCCTATTATATTAGGTG  218
            ||||||||||||||||||||||||| || |||||||||||||| ||||| ||||| ||||
Sbjct  121  TAGTTATACCAATCATAATCGGAGGATTTGGAAATTGATTAGTCCCTATCATATTGGGTG  180

Query  219  CCCCAGATATAGCATTTCCACGAATAAATAATATAAGATTTTGACTTTTACCCCCCTCAT  278
            |||||||||||||||||||||||||||||||||||||||||||||||||||| || ||| 
Sbjct  181  CCCCAGATATAGCATTTCCACGAATAAATAATATAAGATTTTGACTTTTACCTCCTTCAC  240

Query  279  TATTTTTATTGATTAACTCTTCATTAATTGAATCCGGAGCTGGAACAGGATGAACAGTTT  338
            |||||||||| ||||||||||||||||||||||| |||||||| ||||||||||||||||
Sbjct  241  TATTTTTATTAATTAACTCTTCATTAATTGAATCTGGAGCTGGTACAGGATGAACAGTTT  300

Query  339  ATCCCCCTCTATCTTCAAATTTATCACATTATGGTCCATCAGTAGATTTAGCTATTTTTT  398
            | || ||||||||||||||||||||||||||||| || ||||||||||||||||||||||
Sbjct  301  ACCCTCCTCTATCTTCAAATTTATCACATTATGGCCCTTCAGTAGATTTAGCTATTTTTT  360

Query  399  CTCTTCACCTTGCTGGTGCTTCTTCAATTTTAGGTGCAATTAACTTCATCACAACTATTG  458
            ||||||| |||||||||||||||||||||||||||||||||||||| || ||||||||||
Sbjct  361  CTCTTCATCTTGCTGGTGCTTCTTCAATTTTAGGTGCAATTAACTTTATTACAACTATTG  420

Query  459  TAAATATACGATCAATTGGAATAACAATAGAACGAATACCATTATTTGTTTGATCTGTTT  518
            |||| ||||||||||||||||||||||||||||| |||||||||||||||||||||||||
Sbjct  421  TAAACATACGATCAATTGGAATAACAATAGAACGTATACCATTATTTGTTTGATCTGTTT  480

Query  519  TAATTACAGCTATTTTACTACTTCTATCTTTACCTGTATTAGCAGGTGCCATTACAATAT  578
            ||||||||||||||||| ||||| ||||||||||||||||||||||||||||||||||||
Sbjct  481  TAATTACAGCTATTTTATTACTTTTATCTTTACCTGTATTAGCAGGTGCCATTACAATAT  540

Query  579  TATTAACTGATCGAAACTTTAACACATCATTTTTTGATCCTTCAGGAGGAGGAGATCCCA  638
            | |||||||||||||||||||| ||||||||||||||||||||||| ||||||||||| |
Sbjct  541  TGTTAACTGATCGAAACTTTAATACATCATTTTTTGATCCTTCAGGGGGAGGAGATCCAA  600

Query  639  TTTTATATCAACACTTATTT  658
```

```
             ||||||||||||| ||||||
Sbjct  601   TTTTATATCAACATTTATTT  620
```

>Rhipicephalus sanguineus isolate Rsan-ML2 cytochrome oxidase subunit I (cox1) gene, partial cds; mitochondrial
Sequence ID: MG855656.1 Length: 630
Range 1: 1 to 620

Score:990 bits(536), Expect:0.0,
Identities:592/620(95%), Gaps:0/620(0%), Strand: Plus/Plus

```
Query  39    TAGGACTTAGTATAAGAATATTAATTCGTATAGAATTAGGACAACCTGGAACTTTAATTG  98
             ||||||||||||||||||||||||||||||||||||||||||||||||||||||||||||
Sbjct  1     TAGGACTTAGTATAAGAATATTAATTCGTATAGAATTAGGACAACCTGGAACTTTAATTG  60

Query  99    GAAATGATCAGATTTATAATGTAATCGTAACAGCACATGCATTTATTATAATTTTCTTTA  158
             ||||||||||||||||||||||||||||||||||||||||||||||||||||||| ||||
Sbjct  61    GAAATGATCAGATTTATAATGTAATCGTAACAGCACATGCATTTATTATAATTTTTTTTA  120

Query  159   TAGTTATACCAATCATAATCGGAGGGTTCGGAAATTGATTAGTTCCTATTATATTAGGTG  218
             ||||||||||||||||||||||||| || |||||||||||||| ||||| ||||| ||||
Sbjct  121   TAGTTATACCAATCATAATCGGAGGATTTGGAAATTGATTAGTCCCTATCATATTGGGTG  180

Query  219   CCCCAGATATAGCATTTCCACGAATAAATAATATAAGATTTTGACTTTTACCCCCCTCAT  278
             |||||||||||||||||||||||||||||||||||||||||||||||||||| || |||
Sbjct  181   CCCCAGATATAGCATTTCCACGAATAAATAATATAAGATTTTGACTTTTACCTCCTTCAC  240

Query  279   TATTTTTATTGATTAACTCTTCATTAATTGAATCCGGAGCTGGAACAGGATGAACAGTTT  338
             |||||||||| ||||||||||||||||||||||| |||||||| ||||||||||||||||
Sbjct  241   TATTTTTATTAATTAACTCTTCATTAATTGAATCTGGAGCTGGTACAGGATGAACAGTTT  300

Query  339   ATCCCCCTCTATCTTCAAATTTATCACATTATGGTCCATCAGTAGATTTAGCTATTTTTT  398
             | || ||||||||||||||||||||||||||||| || ||||||||||||||||||||||
Sbjct  301   ACCCTCCTCTATCTTCAAATTTATCACATTATGGCCCTTCAGTAGATTTAGCTATTTTTT  360

Query  399   CTCTTCACCTTGCTGGTGCTTCTTCAATTTTAGGTGCAATTAACTTCATCACAACTATTG  458
             ||||||| |||||||||||||||||||||||||||||||||||||| || ||||||||||
Sbjct  361   CTCTTCATCTTGCTGGTGCTTCTTCAATTTTAGGTGCAATTAACTTTATTACAACTATTG  420

Query  459   TAAATATACGATCAATTGGAATAACAATAGAACGAATACCATTATTTGTTTGATCTGTTT  518
             |||| ||||||||||||||||||||||||||||| |||||||||||||||||||||||||
Sbjct  421   TAAACATACGATCAATTGGAATAACAATAGAACGTATACCATTATTTGTTTGATCTGTTT  480

Query  519   TAATTACAGCTATTTTACTACTTCTATCTTTACCTGTATTAGCAGGTGCCATTACAATAT  578
             ||||||||||||||||| ||||| ||||||||||||||||||||||||||||||||||||
Sbjct  481   TAATTACAGCTATTTTATTACTTTTATCTTTACCTGTATTAGCAGGTGCCATTACAATAT  540

Query  579   TATTAACTGATCGAAACTTTAACACATCATTTTTTGATCCTTCAGGAGGAGGAGATCCCA  638
             | ||||||||||||||||||||| |||||||||||||||||||||| ||||||||||| |
Sbjct  541   TGTTAACTGATCGAAACTTTAATACATCATTTTTTGATCCTTCAGGGGGAGGAGATCCAA  600
```

```
Query  639  TTTTATATCAACACTTATTT  658
            ||||||||||||| ||||||
Sbjct  601  TTTTATATCAACATTTATTT  620
```

>Rhipicephalus sanguineus isolate Kehf-Lagareb-A cytochrome oxidase subunit I (COI) gene, partial cds; mitochondrial
Sequence ID: KX757910.1 Length: 630
Range 1: 1 to 620

Score:990 bits(536), Expect:0.0,
Identities:592/620(95%), Gaps:0/620(0%), Strand: Plus/Plus

```
Query  39   TAGGACTTAGTATAAGAATATTAATTCGTATAGAATTAGGACAACCTGGAACTTTAATTG  98
            ||||||||||||||||||||||||||||||||||||||||||||||||||||||||||||
Sbjct  1    TAGGACTTAGTATAAGAATATTAATTCGTATAGAATTAGGACAACCTGGAACTTTAATTG  60

Query  99   GAAATGATCAGATTTATAATGTAATCGTAACAGCACATGCATTTATTATAATTTTCTTTA  158
            ||||||||||||||||||||||||||||||||||||||||||||||||||||||| ||||
Sbjct  61   GAAATGATCAGATTTATAATGTAATCGTAACAGCACATGCATTTATTATAATTTTTTTTA  120

Query  159  TAGTTATACCAATCATAATCGGAGGGTTCGGAAATTGATTAGTTCCTATTATATTAGGTG  218
            ||||||||||||||||||||||||| || |||||||||||||| ||||| ||||| ||||
Sbjct  121  TAGTTATACCAATCATAATCGGAGGATTTGGAAATTGATTAGTCCCTATCATATTGGGTG  180

Query  219  CCCCAGATATAGCATTTCCACGAATAAATAATATAAGATTTTGACTTTTACCCCCCTCAT  278
            |||||||||||||||||||||||||||||||||||||||||||||||||||| || |||
Sbjct  181  CCCCAGATATAGCATTTCCACGAATAAATAATATAAGATTTTGACTTTTACCTCCTTCAC  240

Query  279  TATTTTTATTGATTAACTCTTCATTAATTGAATCCGGAGCTGGAACAGGATGAACAGTTT  338
            |||||||||| ||||||||||||||||||||||| |||||||| ||||||||||||||||
Sbjct  241  TATTTTTATTAATTAACTCTTCATTAATTGAATCTGGAGCTGGTACAGGATGAACAGTTT  300

Query  339  ATCCCCCTCTATCTTCAAATTTATCACATTATGGTCCATCAGTAGATTTAGCTATTTTTT  398
            | || ||||||||||||||||||||||||||||| || ||||||||||||||||||||||
Sbjct  301  ACCCTCCTCTATCTTCAAATTTATCACATTATGGCCCTTCAGTAGATTTAGCTATTTTTT  360

Query  399  CTCTTCACCTTGCTGGTGCTTCTTCAATTTTAGGTGCAATTAACTTCATCACAACTATTG  458
            ||||||| |||||||||||||||||||||||||||||||||||||| || ||||||||||
Sbjct  361  CTCTTCATCTTGCTGGTGCTTCTTCAATTTTAGGTGCAATTAACTTTATTACAACTATTG  420

Query  459  TAAATATACGATCAATTGGAATAACAATAGAACGAATACCATTATTTGTTTGATCTGTTT  518
            |||| ||||||||||||||||||||||||||||| |||||||||||||||||||||||||
Sbjct  421  TAAACATACGATCAATTGGAATAACAATAGAACGTATACCATTATTTGTTTGATCTGTTT  480

Query  519  TAATTACAGCTATTTTACTACTTCTATCTTTACCTGTATTAGCAGGTGCCATTACAATAT  578
            ||||||||||||||||| ||||| ||||||||||||||||||||||||||||||||||||
Sbjct  481  TAATTACAGCTATTTTATTACTTTTATCTTTACCTGTATTAGCAGGTGCCATTACAATAT  540

Query  579  TATTAACTGATCGAAACTTTAACACATCATTTTTTGATCCTTCAGGAGGAGGAGATCCCA  638
            | |||||||||||||||||||| ||||||||||||||||||||||| ||||||||||| |
Sbjct  541  TGTTAACTGATCGAAACTTTAATACATCATTTTTTGATCCTTCAGGGGGAGGAGATCCAA  600
```

```
Query  639  TTTTATATCAACACTTATTT  658
            ||||||||||||| ||||||
Sbjct  601  TTTTATATCAACATTTATTT  620
```

>Rhipicephalus sanguineus isolate Italy-B cytochrome oxidase subunit I (COI) gene, partial cds; mitochondrial
Sequence ID: KX757904.1 Length: 630
Range 1: 1 to 620

Score:990 bits(536), Expect:0.0,
Identities:592/620(95%), Gaps:0/620(0%), Strand: Plus/Plus

```
Query  39   TAGGACTTAGTATAAGAATATTAATTCGTATAGAATTAGGACAACCTGGAACTTTAATTG  98
            ||||||||||||||||||||||||||||||||||||||||||||||||||||||||||||
Sbjct  1    TAGGACTTAGTATAAGAATATTAATTCGTATAGAATTAGGACAACCTGGAACTTTAATTG  60

Query  99   GAAATGATCAGATTTATAATGTAATCGTAACAGCACATGCATTTATTATAATTTTCTTTA  158
            ||||||||||||||||||||||||||||||||||||||||||||||||||||||| ||||
Sbjct  61   GAAATGATCAGATTTATAATGTAATCGTAACAGCACATGCATTTATTATAATTTTTTTTA  120

Query  159  TAGTTATACCAATCATAATCGGAGGGTTCGGAAATTGATTAGTTCCTATTATATTAGGTG  218
            ||||||||||||||||||||||||| || |||||||||||||| ||||| ||||| ||||
Sbjct  121  TAGTTATACCAATCATAATCGGAGGATTTGGAAATTGATTAGTCCCTATCATATTGGGTG  180

Query  219  CCCCAGATATAGCATTTCCACGAATAAATAATATAAGATTTTGACTTTTACCCCCCTCAT  278
            |||||||||||||||||||||||||||||||||||||||||||||||||||| || |||
Sbjct  181  CCCCAGATATAGCATTTCCACGAATAAATAATATAAGATTTTGACTTTTACCTCCTTCAC  240

Query  279  TATTTTTATTGATTAACTCTTCATTAATTGAATCCGGAGCTGGAACAGGATGAACAGTTT  338
            |||||||||| ||||||||||||||||||||||| |||||||| ||||||||||||||||
Sbjct  241  TATTTTTATTAATTAACTCTTCATTAATTGAATCTGGAGCTGGTACAGGATGAACAGTTT  300

Query  339  ATCCCCCTCTATCTTCAAATTTATCACATTATGGTCCATCAGTAGATTTAGCTATTTTTT  398
            | || ||||||||||||||||||||||||||||| || ||||||||||||||||||||||
Sbjct  301  ACCCTCCTCTATCTTCAAATTTATCACATTATGGCCCTTCAGTAGATTTAGCTATTTTTT  360

Query  399  CTCTTCACCTTGCTGGTGCTTCTTCAATTTTAGGTGCAATTAACTTCATCACAACTATTG  458
            ||||||| |||||||||||||||||||||||||||||||||||||| || ||||||||||
Sbjct  361  CTCTTCATCTTGCTGGTGCTTCTTCAATTTTAGGTGCAATTAACTTTATTACAACTATTG  420

Query  459  TAAATATACGATCAATTGGAATAACAATAGAACGAATACCATTATTTGTTTGATCTGTTT  518
            |||| ||||||||||||||||||||||||||||| |||||||||||||||||||||||||
Sbjct  421  TAAACATACGATCAATTGGAATAACAATAGAACGTATACCATTATTTGTTTGATCTGTTT  480

Query  519  TAATTACAGCTATTTTACTACTTCTATCTTTACCTGTATTAGCAGGTGCCATTACAATAT  578
            |||||||||||||||| |||||| ||||||||||||||||||||||||||||||||||||
Sbjct  481  TAATTACAGCTATTTTATTACTTTTATCTTTACCTGTATTAGCAGGTGCCATTACAATAT  540

Query  579  TATTAACTGATCGAAACTTTAACACATCATTTTTTGATCCTTCAGGAGGAGGAGATCCCA  638
            | |||||||||||||||||||| ||||||||||||||||||||||| ||||||||||| |
```

```
Sbjct  541   TGTTAACTGATCGAAACTTTAATACATCATTTTTTGATCCTTCAGGGGGAGGAGATCCAA  600

Query  639   TTTTATATCAACACTTATTT  658
             ||||||||||||| ||||||
Sbjct  601   TTTTATATCAACATTTATTT  620
```

>Rhipicephalus sanguineus isolate Malta-A cytochrome oxidase subunit I (COI) gene, partial cds; mitochondrial
Sequence ID: KX757902.1 Length: 630
Range 1: 1 to 620

Score:990 bits(536), Expect:0.0,
Identities:592/620(95%), Gaps:0/620(0%), Strand: Plus/Plus

```
Query  39    TAGGACTTAGTATAAGAATATTAATTCGTATAGAATTAGGACAACCTGGAACTTTAATTG  98
             ||||||||||||||||||||||||||||||||||||||||||||||||||||||||||||
Sbjct  1     TAGGACTTAGTATAAGAATATTAATTCGTATAGAATTAGGACAACCTGGAACTTTAATTG  60

Query  99    GAAATGATCAGATTTATAATGTAATCGTAACAGCACATGCATTTATTATAATTTTCTTTA  158
             ||||||||||||||||||||||||||||||||||||||||||||||||||||||| ||||
Sbjct  61    GAAATGATCAGATTTATAATGTAATCGTAACAGCACATGCATTTATTATAATTTTTTTTA  120

Query  159   TAGTTATACCAATCATAATCGGAGGGTTCGGAAATTGATTAGTTCCTATTATATTAGGTG  218
             ||||||||||||||||||||||||| || |||||||||||||| ||||| ||||| ||||
Sbjct  121   TAGTTATACCAATCATAATCGGAGGATTTGGAAATTGATTAGTCCCTATCATATTGGGTG  180

Query  219   CCCCAGATATAGCATTTCCACGAATAAATAATATAAGATTTTGACTTTTACCCCCCTCAT  278
             |||||||||||||||||||||||||||||||||||||||||||||||||||| || ||| 
Sbjct  181   CCCCAGATATAGCATTTCCACGAATAAATAATATAAGATTTTGACTTTTACCTCCTTCAC  240

Query  279   TATTTTTATTGATTAACTCTTCATTAATTGAATCCGGAGCTGGAACAGGATGAACAGTTT  338
             |||||||||| ||||||||||||||||||||||| |||||||| ||||||||||||||||
Sbjct  241   TATTTTTATTAATTAACTCTTCATTAATTGAATCTGGAGCTGGTACAGGATGAACAGTTT  300

Query  339   ATCCCCCTCTATCTTCAAATTTATCACATTATGGTCCATCAGTAGATTTAGCTATTTTTT  398
             | || ||||||||||||||||||||||||||||| || ||||||||||||||||||||||
Sbjct  301   ACCCTCCTCTATCTTCAAATTTATCACATTATGGCCCTTCAGTAGATTTAGCTATTTTTT  360

Query  399   CTCTTCACCTTGCTGGTGCTTCTTCAATTTTAGGTGCAATTAACTTCATCACAACTATTG  458
             ||||||| ||||||||||||||||||||||||||||||||||||| || |||||||||||
Sbjct  361   CTCTTCATCTTGCTGGTGCTTCTTCAATTTTAGGTGCAATTAACTTTATTACAACTATTG  420

Query  459   TAAATATACGATCAATTGGAATAACAATAGAACGAATACCATTATTTGTTTGATCTGTTT  518
             |||| ||||||||||||||||||||||||||||| |||||||||||||||||||||||||
Sbjct  421   TAAACATACGATCAATTGGAATAACAATAGAACGTATACCATTATTTGTTTGATCTGTTT  480

Query  519   TAATTACAGCTATTTTACTACTTCTATCTTTACCTGTATTAGCAGGTGCCATTACAATAT  578
             ||||||||||||||||| ||||| ||||||||||||||||||||||||||||||||||||
Sbjct  481   TAATTACAGCTATTTTATTACTTTTATCTTTACCTGTATTAGCAGGTGCCATTACAATAT  540

Query  579   TATTAACTGATCGAAACTTTAACACATCATTTTTTGATCCTTCAGGAGGAGGAGATCCCA  638
```

```
                 | |||||||||||||||||||| ||||||||||||||||||||||| ||||||||||| |
Sbjct  541  TGTTAACTGATCGAAACTTTAATACATCATTTTTTGATCCTTCAGGGGGAGGAGATCCAA  600

Query  639  TTTTATATCAACACTTATTT  658
            ||||||||||||| ||||||
Sbjct  601  TTTTATATCAACATTTATTT  620
```

>Rhipicephalus sanguineus isolate Zagreb-C cytochrome oxidase subunit I (COI) gene, partial cds; mitochondrial
Sequence ID: KX757896.1 Length: 630
Range 1: 1 to 620

Score:990 bits(536), Expect:0.0,
Identities:592/620(95%), Gaps:0/620(0%), Strand: Plus/Plus

```
Query  39   TAGGACTTAGTATAAGAATATTAATTCGTATAGAATTAGGACAACCTGGAACTTTAATTG  98
            ||||||||||||||||||||||||||||||||||||||||||||||||||||||||||||
Sbjct  1    TAGGACTTAGTATAAGAATATTAATTCGTATAGAATTAGGACAACCTGGAACTTTAATTG  60

Query  99   GAAATGATCAGATTTATAATGTAATCGTAACAGCACATGCATTTATTATAATTTTCTTTA  158
            ||||||||||||||||||||||||||||||||||||||||||||||||||||||| ||||
Sbjct  61   GAAATGATCAGATTTATAATGTAATCGTAACAGCACATGCATTTATTATAATTTTTTTTA  120

Query  159  TAGTTATACCAATCATAATCGGAGGGTTCGGAAATTGATTAGTTCCTATTATATTAGGTG  218
            ||||||||||||||||||||||||| || |||||||||||||| ||||| ||||| ||||
Sbjct  121  TAGTTATACCAATCATAATCGGAGGATTTGGAAATTGATTAGTCCCTATCATATTGGGTG  180

Query  219  CCCCAGATATAGCATTTCCACGAATAAATAATATAAGATTTTGACTTTTACCCCCCTCAT  278
            |||||||||||||||||||||||||||||||||||||||||||||||||||| || ||| 
Sbjct  181  CCCCAGATATAGCATTTCCACGAATAAATAATATAAGATTTTGACTTTTACCTCCTTCAC  240

Query  279  TATTTTTATTGATTAACTCTTCATTAATTGAATCCGGAGCTGGAACAGGATGAACAGTTT  338
            |||||||||| ||||||||||||||||||||||| |||||||| ||||||||||||||||
Sbjct  241  TATTTTTATTAATTAACTCTTCATTAATTGAATCTGGAGCTGGTACAGGATGAACAGTTT  300

Query  339  ATCCCCCTCTATCTTCAAATTTATCACATTATGGTCCATCAGTAGATTTAGCTATTTTTT  398
            | || ||||||||||||||||||||||||||||| || ||||||||||||||||||||||
Sbjct  301  ACCCTCCTCTATCTTCAAATTTATCACATTATGGCCCTTCAGTAGATTTAGCTATTTTTT  360

Query  399  CTCTTCACCTTGCTGGTGCTTCTTCAATTTAGGTGCAATTAACTTCATCACAACTATTG  458
            ||||||| |||||||||||||||||||||||||||||||||||||| || ||||||||||
Sbjct  361  CTCTTCATCTTGCTGGTGCTTCTTCAATTTAGGTGCAATTAACTTTATTACAACTATTG  420

Query  459  TAAATATACGATCAATTGGAATAACAATAGAACGAATACCATTATTTGTTTGATCTGTTT  518
            |||| ||||||||||||||||||||||||||||| |||||||||||||||||||||||||
Sbjct  421  TAAACATACGATCAATTGGAATAACAATAGAACGTATACCATTATTTGTTTGATCTGTTT  480

Query  519  TAATTACAGCTATTTTACTACTTCTATCTTTACCTGTATTAGCAGGTGCCATTACAATAT  578
            ||||||||||||||||| ||||| ||||||||||||||||||||||||||||||||||||
Sbjct  481  TAATTACAGCTATTTTATTACTTTTATCTTTACCTGTATTAGCAGGTGCCATTACAATAT  540
```

```
Query  579  TATTAACTGATCGAAACTTTAACACATCATTTTTTGATCCTTCAGGAGGAGGAGATCCCA  638
            | |||||||||||||||||||| ||||||||||||||||||||||| ||||||||||| |
Sbjct  541  TGTTAACTGATCGAAACTTTAATACATCATTTTTTGATCCTTCAGGGGGAGGAGATCCAA  600

Query  639  TTTTATATCAACACTTATTT  658
            ||||||||||||| ||||||
Sbjct  601  TTTTATATCAACATTTATTT  620
```

>Rhipicephalus sanguineus isolate Pula-B cytochrome oxidase subunit I (COI) gene, partial cds; mitochondrial
Sequence ID: KX757889.1 Length: 630
Range 1: 1 to 620

Score:990 bits(536), Expect:0.0,
Identities:592/620(95%), Gaps:0/620(0%), Strand: Plus/Plus

```
Query  39   TAGGACTTAGTATAAGAATATTAATTCGTATAGAATTAGGACAACCTGGAACTTTAATTG  98
            ||||||||||||||||||||||||||||||||||||||||||||||||||||||||||||
Sbjct  1    TAGGACTTAGTATAAGAATATTAATTCGTATAGAATTAGGACAACCTGGAACTTTAATTG  60

Query  99   GAAATGATCAGATTTATAATGTAATCGTAACAGCACATGCATTTATTATAATTTTCTTTA  158
            ||||||||||||||||||||||||||||||||||||||||||||||||||||||| ||||
Sbjct  61   GAAATGATCAGATTTATAATGTAATCGTAACAGCACATGCATTTATTATAATTTTTTTTA  120

Query  159  TAGTTATACCAATCATAATCGGAGGGTTCGGAAATTGATTAGTTCCTATTATATTAGGTG  218
            ||||||||||||||||||||||||| || |||||||||||||| ||||| ||||| ||||
Sbjct  121  TAGTTATACCAATCATAATCGGAGGATTTGGAAATTGATTAGTCCCTATCATATTGGGTG  180

Query  219  CCCCAGATATAGCATTTCCACGAATAAATAATATAAGATTTTGACTTTTACCCCCCTCAT  278
            |||||||||||||||||||||||||||||||||||||||||||||||||||| || ||| 
Sbjct  181  CCCCAGATATAGCATTTCCACGAATAAATAATATAAGATTTTGACTTTTACCTCCTTCAC  240

Query  279  TATTTTTATTGATTAACTCTTCATTAATTGAATCCGGAGCTGGAACAGGATGAACAGTTT  338
            |||||||||| ||||||||||||||||||||||| |||||||| ||||||||||||||||
Sbjct  241  TATTTTTATTAATTAACTCTTCATTAATTGAATCTGGAGCTGGTACAGGATGAACAGTTT  300

Query  339  ATCCCCCTCTATCTTCAAATTTATCACATTATGGTCCATCAGTAGATTTAGCTATTTTTT  398
            | || ||||||||||||||||||||||||||||| || ||||||||||||||||||||||
Sbjct  301  ACCCTCCTCTATCTTCAAATTTATCACATTATGGCCCTTCAGTAGATTTAGCTATTTTTT  360

Query  399  CTCTTCACCTTGCTGGTGCTTCTTCAATTTTAGGTGCAATTAACTTCATCACAACTATTG  458
            ||||||| |||||||||||||||||||||||||||||||||||||| || ||||||||||
Sbjct  361  CTCTTCATCTTGCTGGTGCTTCTTCAATTTTAGGTGCAATTAACTTTATTACAACTATTG  420

Query  459  TAAATATACGATCAATTGGAATAACAATAGAACGAATACCATTATTTGTTTGATCTGTTT  518
            |||| ||||||||||||||||||||||||||||| |||||||||||||||||||||||||
Sbjct  421  TAAACATACGATCAATTGGAATAACAATAGAACGTATACCATTATTTGTTTGATCTGTTT  480

Query  519  TAATTACAGCTATTTTACTACTTCTATCTTTACCTGTATTAGCAGGTGCCATTACAATAT  578
            ||||||||||||||||| ||||| ||||||||||||||||||||||||||||||||||||
Sbjct  481  TAATTACAGCTATTTTATTACTTTTATCTTTACCTGTATTAGCAGGTGCCATTACAATAT  540
```

```
Query  579  TATTAACTGATCGAAACTTTAACACATCATTTTTTGATCCTTCAGGAGGAGGAGATCCCA  638
            | |||||||||||||||||||| ||||||||||||||||||||||| ||||||||||| |
Sbjct  541  TGTTAACTGATCGAAACTTTAATACATCATTTTTTGATCCTTCAGGGGGAGGAGATCCAA  600

Query  639  TTTTATATCAACACTTATTT  658
            ||||||||||||| ||||||
Sbjct  601  TTTTATATCAACATTTATTT  620
```

>Rhipicephalus sanguineus isolate Rovind cytochrome oxidase subunit I (COI) gene, partial cds; mitochondrial
Sequence ID: KX757887.1 Length: 630
Range 1: 1 to 620

Score:990 bits(536), Expect:0.0,
Identities:592/620(95%), Gaps:0/620(0%), Strand: Plus/Plus

```
Query  39   TAGGACTTAGTATAAGAATATTAATTCGTATAGAATTAGGACAACCTGGAACTTTAATTG  98
            ||||||||||||||||||||||||||||||||||||||||||||||||||||||||||||
Sbjct  1    TAGGACTTAGTATAAGAATATTAATTCGTATAGAATTAGGACAACCTGGAACTTTAATTG  60

Query  99   GAAATGATCAGATTTATAATGTAATCGTAACAGCACATGCATTTATTATAATTTTCTTTA  158
            ||||||||||||||||||||||||||||||||||||||||||||||||||||||| ||||
Sbjct  61   GAAATGATCAGATTTATAATGTAATCGTAACAGCACATGCATTTATTATAATTTTTTTTA  120

Query  159  TAGTTATACCAATCATAATCGGAGGGTTCGGAAATTGATTAGTTCCTATTATATTAGGTG  218
            ||||||||||||||||||||||||| || |||||||||||||| ||||| ||||| ||||
Sbjct  121  TAGTTATACCAATCATAATCGGAGGATTTGGAAATTGATTAGTCCCTATCATATTGGGTG  180

Query  219  CCCCAGATATAGCATTTCCACGAATAAATAATATAAGATTTTGACTTTTACCCCCCTCAT  278
            |||||||||||||||||||||||||||||||||||||||||||||||||||| || ||| 
Sbjct  181  CCCCAGATATAGCATTTCCACGAATAAATAATATAAGATTTTGACTTTTACCTCCTTCAC  240

Query  279  TATTTTTATTGATTAACTCTTCATTAATTGAATCCGGAGCTGGAACAGGATGAACAGTTT  338
            |||||||||| ||||||||||||||||||||||| |||||||| ||||||||||||||||
Sbjct  241  TATTTTTATTAATTAACTCTTCATTAATTGAATCTGGAGCTGGTACAGGATGAACAGTTT  300

Query  339  ATCCCCCTCTATCTTCAAATTTATCACATTATGGTCCATCAGTAGATTTAGCTATTTTTT  398
            | || ||||||||||||||||||||||||||||| || ||||||||||||||||||||||
Sbjct  301  ACCCTCCTCTATCTTCAAATTTATCACATTATGGCCCTTCAGTAGATTTAGCTATTTTTT  360

Query  399  CTCTTCACCTTGCTGGTGCTTCTTCAATTTTAGGTGCAATTAACTTCATCACAACTATTG  458
            ||||||| |||||||||||||||||||||||||||||||||||||||| || ||||||||||
Sbjct  361  CTCTTCATCTTGCTGGTGCTTCTTCAATTTTAGGTGCAATTAACTTTATTACAACTATTG  420

Query  459  TAAATATACGATCAATTGGAATAACAATAGAACGAATACCATTATTTGTTTGATCTGTTT  518
            |||| ||||||||||||||||||||||||||||| |||||||||||||||||||||||||
Sbjct  421  TAAACATACGATCAATTGGAATAACAATAGAACGTATACCATTATTTGTTTGATCTGTTT  480

Query  519  TAATTACAGCTATTTTACTACTTCTATCTTTACCTGTATTAGCAGGTGCCATTACAATAT  578
            ||||||||||||||||| ||||| ||||||||||||||||||||||||||||||||||||
```

```
Sbjct  481   TAATTACAGCTATTTTATTACTTTTATCTTTACCTGTATTAGCAGGTGCCATTACAATAT  540

Query  579   TATTAACTGATCGAAACTTTAACACATCATTTTTTGATCCTTCAGGAGGAGGAGATCCCA  638
             | |||||||||||||||||||| ||||||||||||||||||||||| ||||||||||| |
Sbjct  541   TGTTAACTGATCGAAACTTTAATACATCATTTTTTGATCCTTCAGGGGGAGGAGATCCAA  600

Query  639   TTTTATATCAACACTTATTT  658
             ||||||||||||| ||||||
Sbjct  601   TTTTATATCAACATTTATTT  620
```

>Rhipicephalus sanguineus isolate Kajtasovo cytochrome oxidase subunit I (COI) gene, partial cds; mitochondrial
Sequence ID: KX757879.1 Length: 630
Range 1: 1 to 620

Score:990 bits(536), Expect:0.0,
Identities:592/620(95%), Gaps:0/620(0%), Strand: Plus/Plus

```
Query  39    TAGGACTTAGTATAAGAATATTAATTCGTATAGAATTAGGACAACCTGGAACTTTAATTG  98
             ||||||||||||||||||||||||||||||||||||||||||||||||||||||||||||
Sbjct  1     TAGGACTTAGTATAAGAATATTAATTCGTATAGAATTAGGACAACCTGGAACTTTAATTG  60

Query  99    GAAATGATCAGATTTATAATGTAATCGTAACAGCACATGCATTTATTATAATTTTCTTTA  158
             ||||||||||||||||||||||||||||||||||||||||||||||||||||||| ||||
Sbjct  61    GAAATGATCAGATTTATAATGTAATCGTAACAGCACATGCATTTATTATAATTTTTTTTA  120

Query  159   TAGTTATACCAATCATAATCGGAGGGTTCGGAAATTGATTAGTTCCTATTATATTAGGTG  218
             ||||||||||||||||||||||||| || |||||||||||||| ||||| ||||| ||||
Sbjct  121   TAGTTATACCAATCATAATCGGAGGATTTGGAAATTGATTAGTCCCTATCATATTGGGTG  180

Query  219   CCCCAGATATAGCATTTCCACGAATAAATAATATAAGATTTTGACTTTTACCCCCCTCAT  278
             |||||||||||||||||||||||||||||||||||||||||||||||||||| || |||
Sbjct  181   CCCCAGATATAGCATTTCCACGAATAAATAATATAAGATTTTGACTTTTACCTCCTTCAC  240

Query  279   TATTTTTATTGATTAACTCTTCATTAATTGAATCCGGAGCTGGAACAGGATGAACAGTTT  338
             |||||||||| ||||||||||||||||||||||| |||||||| ||||||||||||||||
Sbjct  241   TATTTTTATTAATTAACTCTTCATTAATTGAATCTGGAGCTGGTACAGGATGAACAGTTT  300

Query  339   ATCCCCCTCTATCTTCAAATTTATCACATTATGGTCCATCAGTAGATTTAGCTATTTTTT  398
             | || ||||||||||||||||||||||||||||| || ||||||||||||||||||||||
Sbjct  301   ACCCTCCTCTATCTTCAAATTTATCACATTATGGCCCTTCAGTAGATTTAGCTATTTTTT  360

Query  399   CTCTTCACCTTGCTGGTGCTTCTTCAATTTTAGGTGCAATTAACTTCATCACAACTATTG  458
             ||||||| |||||||||||||||||||||||||||||||||||||| || ||||||||||
Sbjct  361   CTCTTCATCTTGCTGGTGCTTCTTCAATTTTAGGTGCAATTAACTTTATTACAACTATTG  420

Query  459   TAAATATACGATCAATTGGAATAACAATAGAACGAATACCATTATTTGTTTGATCTGTTT  518
             |||| ||||||||||||||||||||||||||||| |||||||||||||||||||||||||
Sbjct  421   TAAACATACGATCAATTGGAATAACAATAGAACGTATACCATTATTTGTTTGATCTGTTT  480

Query  519   TAATTACAGCTATTTTACTACTTCTATCTTTACCTGTATTAGCAGGTGCCATTACAATAT  578
```

```
             ||||||||||||||||| ||||| ||||||||||||||||||||||||||||||||||||
Sbjct  481   TAATTACAGCTATTTTATTACTTTTATCTTTACCTGTATTAGCAGGTGCCATTACAATAT  540

Query  579   TATTAACTGATCGAAACTTTAACACATCATTTTTGATCCTTCAGGAGGAGGAGATCCCA  638
             | |||||||||||||||||||| ||||||||||||||||||||||| ||||||||||| |
Sbjct  541   TGTTAACTGATCGAAACTTTAATACATCATTTTTGATCCTTCAGGGGGAGGAGATCCAA  600

Query  639   TTTTATATCAACACTTATTT  658
             ||||||||||||| ||||||
Sbjct  601   TTTTATATCAACATTTATTT  620
```

>Rhipicephalus sanguineus clone #6 cytochrome oxidase subunit I (COI) gene, partial cds; mitochondrial
Sequence ID: KX519711.1 Length: 636
Range 1: 1 to 620

Score:990 bits(536), Expect:0.0,
Identities:592/620(95%), Gaps:0/620(0%), Strand: Plus/Plus

```
Query  39    TAGGACTTAGTATAAGAATATTAATTCGTATAGAATTAGGACAACCTGGAACTTTAATTG  98
             ||||||||||||||||||||||||||||||||||||||||||||||||||||||||||||
Sbjct  1     TAGGACTTAGTATAAGAATATTAATTCGTATAGAATTAGGACAACCTGGAACTTTAATTG  60

Query  99    GAAATGATCAGATTTATAATGTAATCGTAACAGCACATGCATTTATTATAATTTTCTTTA  158
             ||||||||||||||||||||||||||||||||||||||||||||||||||||||| ||||
Sbjct  61    GAAATGATCAGATTTATAATGTAATCGTAACAGCACATGCATTTATTATAATTTTTTTTA  120

Query  159   TAGTTATACCAATCATAATCGGAGGGTTCGGAAATTGATTAGTTCCTATTATATTAGGTG  218
             ||||||||||||||||||||||||| || |||||||||||||| ||||| ||||| ||||
Sbjct  121   TAGTTATACCAATCATAATCGGAGGATTTGGAAATTGATTAGTCCCTATCATATTGGGTG  180

Query  219   CCCCAGATATAGCATTTCCACGAATAAATAATATAAGATTTTGACTTTTACCCCCCTCAT  278
             |||||||||||||||||||||||||||||||||||||||||||||||||||| || |||
Sbjct  181   CCCCAGATATAGCATTTCCACGAATAAATAATATAAGATTTTGACTTTTACCTCCTTCAC  240

Query  279   TATTTTTATTGATTAACTCTTCATTAATTGAATCCGGAGCTGGAACAGGATGAACAGTTT  338
             |||||||||| ||||||||||||||||||||||| |||||||| ||||||||||||||||
Sbjct  241   TATTTTTATTAATTAACTCTTCATTAATTGAATCTGGAGCTGGTACAGGATGAACAGTTT  300

Query  339   ATCCCCCTCTATCTTCAAATTTATCACATTATGGTCCATCAGTAGATTTAGCTATTTTTT  398
             | || ||||||||||||||||||||||||||||| || ||||||||||||||||||||||
Sbjct  301   ACCCTCCTCTATCTTCAAATTTATCACATTATGGCCCTTCAGTAGATTTAGCTATTTTTT  360

Query  399   CTCTTCACCTTGCTGGTGCTTCTTCAATTTTAGGTGCAATTAACTTCATCACAACTATTG  458
             ||||||| |||||||||||||||||||||||||||||||||||||| || ||||||||||
Sbjct  361   CTCTTCATCTTGCTGGTGCTTCTTCAATTTTAGGTGCAATTAACTTTATTACAACTATTG  420

Query  459   TAAATATACGATCAATTGGAATAACAATAGAACGAATACCATTATTTGTTTGATCTGTTT  518
             |||| ||||||||||||||||||||||||||||| |||||||||||||||||||||||||
Sbjct  421   TAAACATACGATCAATTGGAATAACAATAGAACGTATACCATTATTTGTTTGATCTGTTT  480
```

```
Query  519   TAATTACAGCTATTTTACTACTTCTATCTTTACCTGTATTAGCAGGTGCCATTACAATAT  578
             ||||||||||||||||| ||||| ||||||||||||||||||||||||||||||||||||
Sbjct  481   TAATTACAGCTATTTTATTACTTTTATCTTTACCTGTATTAGCAGGTGCCATTACAATAT  540

Query  579   TATTAACTGATCGAAACTTTAACACATCATTTTTTGATCCTTCAGGAGGAGGAGATCCCA  638
             | |||||||||||||||||||| ||||||||||||||||||||||| ||||||||||| |
Sbjct  541   TGTTAACTGATCGAAACTTTAATACATCATTTTTTGATCCTTCAGGGGGAGGAGATCCAA  600

Query  639   TTTTATATCAACACTTATTT  658
             ||||||||||||| ||||||
Sbjct  601   TTTTATATCAACATTTATTT  620
```

>Rhipicephalus sanguineus isolate SV4 cytochrome c oxidase subunit I (COX1) gene, partial cds; mitochondrial
Sequence ID: MW135448.1 Length: 630
Range 1: 1 to 620

Score:990 bits(536), Expect:0.0,
Identities:592/620(95%), Gaps:0/620(0%), Strand: Plus/Plus

```
Query  39    TAGGACTTAGTATAAGAATATTAATTCGTATAGAATTAGGACAACCTGGAACTTTAATTG  98
             ||||||||||||||||||||||||||||||||||||||||||||||||||||||||||||
Sbjct  1     TAGGACTTAGTATAAGAATATTAATTCGTATAGAATTAGGACAACCTGGAACTTTAATTG  60

Query  99    GAAATGATCAGATTTATAATGTAATCGTAACAGCACATGCATTTATTATAATTTTCTTTA  158
             ||||||||||||||||||||||||||||||||||||||||||||||||||||||| ||||
Sbjct  61    GAAATGATCAGATTTATAATGTAATCGTAACAGCACATGCATTTATTATAATTTTTTTTA  120

Query  159   TAGTTATACCAATCATAATCGGAGGGTTCGGAAATTGATTAGTTCCTATTATATTAGGTG  218
             ||||||||||||||||||||||||| || |||||||||||||| ||||| ||||| ||||
Sbjct  121   TAGTTATACCAATCATAATCGGAGGATTTGGAAATTGATTAGTCCCTATCATATTGGGTG  180

Query  219   CCCCAGATATAGCATTTCCACGAATAAATAATATAAGATTTTGACTTTTACCCCCCTCAT  278
             |||||||||||||||||||||||||||||||||||||||||||||||||||| || |||
Sbjct  181   CCCCAGATATAGCATTTCCACGAATAAATAATATAAGATTTTGACTTTTACCTCCTTCAC  240

Query  279   TATTTTTATTGATTAACTCTTCATTAATTGAATCCGGAGCTGGAACAGGATGAACAGTTT  338
             |||||||||| ||||||||||||||||||||||| |||||||| ||||||||||||||||
Sbjct  241   TATTTTTATTAATTAACTCTTCATTAATTGAATCTGGAGCTGGTACAGGATGAACAGTTT  300

Query  339   ATCCCCCTCTATCTTCAAATTTATCACATTATGGTCCATCAGTAGATTTAGCTATTTTTT  398
             | || ||||||||||||||||||||||||||||| || ||||||||||||||||||||||
Sbjct  301   ACCCTCCTCTATCTTCAAATTTATCACATTATGGCCCTTCAGTAGATTTAGCTATTTTTT  360

Query  399   CTCTTCACCTTGCTGGTGCTTCTTCAATTTTAGGTGCAATTAACTTCATCACAACTATTG  458
             ||||||| |||||||||||||||||||||||||||||||||||||| || ||||||||||
Sbjct  361   CTCTTCATCTTGCTGGTGCTTCTTCAATTTTAGGTGCAATTAACTTTATTACAACTATTG  420

Query  459   TAAATATACGATCAATTGGAATAACAATAGAACGAATACCATTATTTGTTTGATCTGTTT  518
             |||| ||||||||||||||||||||||||||||| |||||||||||||||||||||||||
Sbjct  421   TAAACATACGATCAATTGGAATAACAATAGAACGTATACCATTATTTGTTTGATCTGTTT  480
```

```
Query  519   TAATTACAGCTATTTTACTACTTCTATCTTTACCTGTATTAGCAGGTGCCATTACAATAT  578
             ||||||||||||||||| ||||| ||||||||||||||||||||||||||||||||||||
Sbjct  481   TAATTACAGCTATTTTATTACTTTTATCTTTACCTGTATTAGCAGGTGCCATTACAATAT  540

Query  579   TATTAACTGATCGAAACTTTAACACATCATTTTTTGATCCTTCAGGAGGAGGAGATCCCA  638
             | |||||||||||||||||||| ||||||||||||||||||||||| ||||||||||| |
Sbjct  541   TGTTAACTGATCGAAACTTTAATACATCATTTTTTGATCCTTCAGGGGGAGGAGATCCAA  600

Query  639   TTTTATATCAACACTTATTT  658
             ||||||||||||| ||||||
Sbjct  601   TTTTATATCAACATTTATTT  620
```

>Rhipicephalus sanguineus isolate Boka-A cytochrome oxidase subunit I (COI) gene, partial cds; mitochondrial
Sequence ID: KX757905.1 Length: 630
Range 1: 1 to 620

Score:985 bits(533), Expect:0.0,
Identities:591/620(95%), Gaps:0/620(0%), Strand: Plus/Plus

```
Query  39    TAGGACTTAGTATAAGAATATTAATTCGTATAGAATTAGGACAACCTGGAACTTTAATTG  98
             ||||||||||||||||||||||||||||||||||||||||||||||||||||||||||||
Sbjct  1     TAGGACTTAGTATAAGAATATTAATTCGTATAGAATTAGGACAACCTGGAACTTTAATTG  60

Query  99    GAAATGATCAGATTTATAATGTAATCGTAACAGCACATGCATTTATTATAATTTTCTTTA  158
             ||||||||||||||||||||||||||||||||||||||||||||||||||||||| ||||
Sbjct  61    GAAATGATCAGATTTATAATGTAATCGTAACAGCACATGCATTTATTATAATTTTTTTTA  120

Query  159   TAGTTATACCAATCATAATCGGAGGGTTCGGAAATTGATTAGTTCCTATTATATTAGGTG  218
             ||||||||||||||||||||||||| || |||||||||||||| ||||| ||||| ||||
Sbjct  121   TAGTTATACCAATCATAATCGGAGGATTTGGAAATTGATTAGTCCCTATCATATTGGGTG  180

Query  219   CCCCAGATATAGCATTTCCACGAATAAATAATATAAGATTTTGACTTTTACCCCCCTCAT  278
             |||||||||||||||||||||||||||||||||||||||||||||||||||| || |||
Sbjct  181   CCCCAGATATAGCATTTCCACGAATAAATAATATAAGATTTTGACTTTTACCTCCTTCAC  240

Query  279   TATTTTTATTGATTAACTCTTCATTAATTGAATCCGGAGCTGGAACAGGATGAACAGTTT  338
             |||||||||| ||||| ||||||||||||||||| |||||||| ||||||||||||||||
Sbjct  241   TATTTTTATTAATTAATTCTTCATTAATTGAATCTGGAGCTGGTACAGGATGAACAGTTT  300

Query  339   ATCCCCCTCTATCTTCAAATTTATCACATTATGGTCCATCAGTAGATTTAGCTATTTTTT  398
             | || ||||||||||||||||||||||||||||| || ||||||||||||||||||||||
Sbjct  301   ACCCTCCTCTATCTTCAAATTTATCACATTATGGCCCTTCAGTAGATTTAGCTATTTTTT  360

Query  399   CTCTTCACCTTGCTGGTGCTTCTTCAATTTTAGGTGCAATTAACTTCATCACAACTATTG  458
             ||||||| |||||||||||||||||||||||||||||||||||||| || ||||||||||
Sbjct  361   CTCTTCATCTTGCTGGTGCTTCTTCAATTTTAGGTGCAATTAACTTTATTACAACTATTG  420

Query  459   TAAATATACGATCAATTGGAATAACAATAGAACGAATACCATTATTTGTTTGATCTGTTT  518
             |||| ||||||||||||||||||||||||||||| |||||||||||||||||||||||||
```

```
Sbjct  421   TAAACATACGATCAATTGGAATAACAATAGAACGTATACCATTATTTGTTTGATCTGTTT  480

Query  519   TAATTACAGCTATTTTACTACTTCTATCTTTACCTGTATTAGCAGGTGCCATTACAATAT  578
             ||||||||||||||||| ||||| ||||||||||||||||||||||||||||||||||||
Sbjct  481   TAATTACAGCTATTTTATTACTTTTATCTTTACCTGTATTAGCAGGTGCCATTACAATAT  540

Query  579   TATTAACTGATCGAAACTTTAACACATCATTTTTTGATCCTTCAGGAGGAGGAGATCCCA  638
             | |||||||||||||||||||| ||||||||||||||||||||||| ||||||||||| |
Sbjct  541   TGTTAACTGATCGAAACTTTAATACATCATTTTTTGATCCTTCAGGGGGAGGAGATCCAA  600

Query  639   TTTTATATCAACACTTATTT  658
             ||||||||||||| ||||||
Sbjct  601   TTTTATATCAACATTTATTT  620
```

>Rhipicephalus sanguineus isolate Sibenik cytochrome oxidase subunit I (COI) gene, partial cds; mitochondrial
Sequence ID: KX757895.1 Length: 630
Range 1: 1 to 620

Score:985 bits(533), Expect:0.0,
Identities:591/620(95%), Gaps:0/620(0%), Strand: Plus/Plus

```
Query  39    TAGGACTTAGTATAAGAATATTAATTCGTATAGAATTAGGACAACCTGGAACTTTAATTG  98
             |||||||||||||||||||||||||||||||||| |||||||||||||||||||||||||
Sbjct  1     TAGGACTTAGTATAAGAATATTAATTCGTATAGAGTTAGGACAACCTGGAACTTTAATTG  60

Query  99    GAAATGATCAGATTTATAATGTAATCGTAACAGCACATGCATTTATTATAATTTTCTTTA  158
             ||||||||||||||||||||||||||||||||||||||||||||||||||||||| ||||
Sbjct  61    GAAATGATCAGATTTATAATGTAATCGTAACAGCACATGCATTTATTATAATTTTTTTTA  120

Query  159   TAGTTATACCAATCATAATCGGAGGGTTCGGAAATTGATTAGTTCCTATTATATTAGGTG  218
             ||||||||||||||||||||||||| || |||||||||||||| ||||| ||||| ||||
Sbjct  121   TAGTTATACCAATCATAATCGGAGGATTTGGAAATTGATTAGTCCCTATCATATTGGGTG  180

Query  219   CCCCAGATATAGCATTTCCACGAATAAATAATATAAGATTTTGACTTTTACCCCCCTCAT  278
             |||||||||||||||||||||||||||||||||||||||||||||||||||| || ||| 
Sbjct  181   CCCCAGATATAGCATTTCCACGAATAAATAATATAAGATTTTGACTTTTACCTCCTTCAC  240

Query  279   TATTTTTATTGATTAACTCTTCATTAATTGAATCCGGAGCTGGAACAGGATGAACAGTTT  338
             |||||||||| ||||||||||||||||||||||| |||||||| ||||||||||||||||
Sbjct  241   TATTTTTATTAATTAACTCTTCATTAATTGAATCTGGAGCTGGTACAGGATGAACAGTTT  300

Query  339   ATCCCCCTCTATCTTCAAATTTATCACATTATGGTCCATCAGTAGATTTAGCTATTTTTT  398
             | || ||||||||||||||||||||||||||||| || ||||||||||||||||||||||
Sbjct  301   ACCCTCCTCTATCTTCAAATTTATCACATTATGGCCCTTCAGTAGATTTAGCTATTTTTT  360

Query  399   CTCTTCACCTTGCTGGTGCTTCTTCAATTTTAGGTGCAATTAACTTCATCACAACTATTG  458
             ||||||| |||||||||||||||||||||||||||||||||||||| || ||||||||||
Sbjct  361   CTCTTCATCTTGCTGGTGCTTCTTCAATTTTAGGTGCAATTAACTTTATTACAACTATTG  420

Query  459   TAAATATACGATCAATTGGAATAACAATAGAACGAATACCATTATTTGTTTGATCTGTTT  518
```

```
                 |||| ||||||||||||||||||||||||||||| |||||||||||||||||||||||||
Sbjct  421       TAAACATACGATCAATTGGAATAACAATAGAACGTATACCATTATTTGTTTGATCTGTTT  480

Query  519       TAATTACAGCTATTTTACTACTTCTATCTTTACCTGTATTAGCAGGTGCCATTACAATAT  578
                 ||||||||||||||||| ||||| ||||||||||||||||||||||||||||||||||||
Sbjct  481       TAATTACAGCTATTTTATTACTTTTATCTTTACCTGTATTAGCAGGTGCCATTACAATAT  540

Query  579       TATTAACTGATCGAAACTTTAACACATCATTTTTTGATCCTTCAGGAGGAGGAGATCCCA  638
                 | |||||||||||||||||||| ||||||||||||||||||||||| ||||||||||| |
Sbjct  541       TGTTAACTGATCGAAACTTTAATACATCATTTTTTGATCCTTCAGGGGGAGGAGATCCAA  600

Query  639       TTTTATATCAACACTTATTT  658
                 ||||||||||||| ||||||
Sbjct  601       TTTTATATCAACATTTATTT  620
```

>Rhipicephalus sanguineus isolate Zadar cytochrome oxidase subunit I (COI) gene, partial cds; mitochondrial
Sequence ID: KX757892.1 Length: 630
Range 1: 1 to 620

Score:985 bits(533), Expect:0.0,
Identities:591/620(95%), Gaps:0/620(0%), Strand: Plus/Plus

```
Query  39        TAGGACTTAGTATAAGAATATTAATTCGTATAGAATTAGGACAACCTGGAACTTTAATTG  98
                 |||||||||||||||||||||||||||||||||| |||||||||||||||||||||||||
Sbjct  1         TAGGACTTAGTATAAGAATATTAATTCGTATAGAGTTAGGACAACCTGGAACTTTAATTG  60

Query  99        GAAATGATCAGATTTATAATGTAATCGTAACAGCACATGCATTATTATAATTTTCTTTA  158
                 ||||||||||||||||||||||||||||||||||||||||||||||||||||||| ||||
Sbjct  61        GAAATGATCAGATTTATAATGTAATCGTAACAGCACATGCATTATTATAATTTTTTTTA  120

Query  159       TAGTTATACCAATCATAATCGGAGGGTTCGGAAATTGATTAGTTCCTATTATATTAGGTG  218
                 ||||||||||||||||||||||||| || |||||||||||||| ||||| ||||| ||||
Sbjct  121       TAGTTATACCAATCATAATCGGAGGATTTGGAAATTGATTAGTCCCTATCATATTGGGTG  180

Query  219       CCCCAGATATAGCATTTCCACGAATAAATAATATAAGATTTTGACTTTTACCCCCCTCAT  278
                 |||||||||||||||||||||||||||||||||||||||||||||||||||| || |||
Sbjct  181       CCCCAGATATAGCATTTCCACGAATAAATAATATAAGATTTTGACTTTTACCTCCTTCAC  240

Query  279       TATTTTTATTGATTAACTCTTCATTAATTGAATCCGGAGCTGGAACAGGATGAACAGTTT  338
                 |||||||||| ||||||||||||||||||||||| |||||||| ||||||||||||||||
Sbjct  241       TATTTTTATTAATTAACTCTTCATTAATTGAATCTGGAGCTGGTACAGGATGAACAGTTT  300

Query  339       ATCCCCCTCTATCTTCAAATTTATCACATTATGGTCCATCAGTAGATTTAGCTATTTTTT  398
                 | || ||||||||||||||||||||||||||||| || ||||||||||||||||||||||
Sbjct  301       ACCCTCCTCTATCTTCAAATTTATCACATTATGGCCCTTCAGTAGATTTAGCTATTTTTT  360

Query  399       CTCTTCACCTTGCTGGTGCTTCTTCAATTTTAGGTGCAATTAACTTCATCACAACTATTG  458
                 ||||||| |||||||||||||||||||||||||||||||||||||| || ||||||||||
Sbjct  361       CTCTTCATCTTGCTGGTGCTTCTTCAATTTTAGGTGCAATTAACTTTATTACAACTATTG  420
```

```
Query  459   TAAATATACGATCAATTGGAATAACAATAGAACGAATACCATTATTTGTTTGATCTGTTT  518
             |||| ||||||||||||||||||||||||||||| |||||||||||||||||||||||||
Sbjct  421   TAAACATACGATCAATTGGAATAACAATAGAACGTATACCATTATTTGTTTGATCTGTTT  480

Query  519   TAATTACAGCTATTTTACTACTTCTATCTTTACCTGTATTAGCAGGTGCCATTACAATAT  578
             ||||||||||||||||| ||||| ||||||||||||||||||||||||||||||||||||
Sbjct  481   TAATTACAGCTATTTTATTACTTTTATCTTTACCTGTATTAGCAGGTGCCATTACAATAT  540

Query  579   TATTAACTGATCGAAACTTTAACACATCATTTTTTGATCCTTCAGGAGGAGGAGATCCCA  638
             | |||||||||||||||||||| ||||||||||||||||||||||| ||||||||||| |
Sbjct  541   TGTTAACTGATCGAAACTTTAATACATCATTTTTTGATCCTTCAGGGGGAGGAGATCCAA  600

Query  639   TTTTATATCAACACTTATTT  658
             ||||||||||||| ||||||
Sbjct  601   TTTTATATCAACATTTATTT  620
```

>Rhipicephalus sanguineus isolate Zagreb-A cytochrome oxidase subunit I (COI) gene, partial cds; mitochondrial
Sequence ID: KX757890.1 Length: 630
Range 1: 1 to 620

Score:985 bits(533), Expect:0.0,
Identities:591/620(95%), Gaps:0/620(0%), Strand: Plus/Plus

```
Query  39    TAGGACTTAGTATAAGAATATTAATTCGTATAGAATTAGGACAACCTGGAACTTTAATTG  98
             |||||||||||||||||||||||||||||||||| |||||||||||||||||||||||||
Sbjct  1     TAGGACTTAGTATAAGAATATTAATTCGTATAGAGTTAGGACAACCTGGAACTTTAATTG  60

Query  99    GAAATGATCAGATTTATAATGTAATCGTAACAGCACATGCATTTATTATAATTTTCTTTA  158
             ||||||||||||||||||||||||||||||||||||||||||||||||||||||| ||||
Sbjct  61    GAAATGATCAGATTTATAATGTAATCGTAACAGCACATGCATTTATTATAATTTTTTTTA  120

Query  159   TAGTTATACCAATCATAATCGGAGGGTTCGGAAATTGATTAGTTCCTATTATATTAGGTG  218
             ||||||||||||||||||||||||| || |||||||||||||| ||||| ||||| ||||
Sbjct  121   TAGTTATACCAATCATAATCGGAGGATTTGGAAATTGATTAGTCCCTATCATATTGGGTG  180

Query  219   CCCAGATATAGCATTTCCACGAATAAATAATATAAGATTTTGACTTTTACCCCCCTCAT  278
             |||||||||||||||||||||||||||||||||||||||||||||||||||| || |||
Sbjct  181   CCCAGATATAGCATTTCCACGAATAAATAATATAAGATTTTGACTTTTACCTCCTTCAC  240

Query  279   TATTTTTATTGATTAACTCTTCATTAATTGAATCCGGAGCTGGAACAGGATGAACAGTTT  338
             |||||||||| ||||||||||||||||||||||| |||||||| ||||||||||||||||
Sbjct  241   TATTTTTATTAATTAACTCTTCATTAATTGAATCTGGAGCTGGTACAGGATGAACAGTTT  300

Query  339   ATCCCCCTCTATCTTCAAATTTATCACATTATGGTCCATCAGTAGATTTAGCTATTTTTT  398
             | || ||||||||||||||||||||||||||||| || ||||||||||||||||||||||
Sbjct  301   ACCCTCCTCTATCTTCAAATTTATCACATTATGGCCCTTCAGTAGATTTAGCTATTTTTT  360

Query  399   CTCTTCACCTTGCTGGTGCTTCTTCAATTTTAGGTGCAATTAACTTCATCACAACTATTG  458
             ||||||| |||||||||||||||||||||||||||||||||||||| || ||||||||||
Sbjct  361   CTCTTCATCTTGCTGGTGCTTCTTCAATTTTAGGTGCAATTAACTTTATTACAACTATTG  420
```

```
Query  459   TAAATATACGATCAATTGGAATAACAATAGAACGAATACCATTATTTGTTTGATCTGTTT  518
             |||| ||||||||||||||||||||||||||||| |||||||||||||||||||||||||
Sbjct  421   TAAACATACGATCAATTGGAATAACAATAGAACGTATACCATTATTTGTTTGATCTGTTT  480

Query  519   TAATTACAGCTATTTTACTACTTCTATCTTTACCTGTATTAGCAGGTGCCATTACAATAT  578
             ||||||||||||||||| ||||| ||||||||||||||||||||||||||||||||||||
Sbjct  481   TAATTACAGCTATTTTATTACTTTTATCTTTACCTGTATTAGCAGGTGCCATTACAATAT  540

Query  579   TATTAACTGATCGAAACTTTAACACATCATTTTTTGATCCTTCAGGAGGAGGAGATCCCA  638
             | |||||||||||||||||||| ||||||||||||||||||||||| ||||||||||| |
Sbjct  541   TGTTAACTGATCGAAACTTTAATACATCATTTTTTGATCCTTCAGGGGGAGGAGATCCAA  600

Query  639   TTTTATATCAACACTTATTT  658
             ||||||||||||| ||||||
Sbjct  601   TTTTATATCAACATTTATTT  620
```

>Rhipicephalus sanguineus isolate Pula-A cytochrome oxidase subunit I (COI) gene, partial cds; mitochondrial
Sequence ID: KX757888.1 Length: 630
Range 1: 1 to 620

Score:985 bits(533), Expect:0.0,
Identities:591/620(95%), Gaps:0/620(0%), Strand: Plus/Plus

```
Query  39    TAGGACTTAGTATAAGAATATTAATTCGTATAGAATTAGGACAACCTGGAACTTTAATTG  98
             ||||||||||||||||||||||||||||||||||||||||||||||||||||||||||||
Sbjct  1     TAGGACTTAGTATAAGAATATTAATTCGTATAGAATTAGGACAACCTGGAACTTTAATTG  60

Query  99    GAAATGATCAGATTTATAATGTAATCGTAACAGCACATGCATTTATTATAATTTTCTTTA  158
             ||||||||||||||||||||||||||||||||||||||||||||||||||||||| ||||
Sbjct  61    GAAATGATCAGATTTATAATGTAATCGTAACAGCACATGCATTTATTATAATTTTTTTTA  120

Query  159   TAGTTATACCAATCATAATCGGAGGGTTCGGAAATTGATTAGTTCCTATTATATTAGGTG  218
             ||||||||||||||||||||||||| || |||||||||||||| ||||| ||||| ||||
Sbjct  121   TAGTTATACCAATCATAATCGGAGGATTTGGAAATTGATTAGTCCCTATCATATTGGGTG  180

Query  219   CCCCAGATATAGCATTTCCACGAATAAATAATATAAGATTTTGACTTTTACCCCCCTCAT  278
             |||||||||||||||||||||||||||||||||||||||||||||||||||| || |||
Sbjct  181   CCCCAGATATAGCATTTCCACGAATAAATAATATAAGATTTTGACTTTTACCTCCTTCAC  240

Query  279   TATTTTTATTGATTAACTCTTCATTAATTGAATCCGGAGCTGGAACAGGATGAACAGTTT  338
             |||||||||| ||||| ||||||||||||||||| |||||||| ||||||||||||||||
Sbjct  241   TATTTTTATTAATTAATTCTTCATTAATTGAATCTGGAGCTGGTACAGGATGAACAGTTT  300

Query  339   ATCCCCCTCTATCTTCAAATTTATCACATTATGGTCCATCAGTAGATTTAGCTATTTTTT  398
             | || ||||||||||||||||||||||||||||| || ||||||||||||||||||||||
Sbjct  301   ACCCTCCTCTATCTTCAAATTTATCACATTATGGCCCTTCAGTAGATTTAGCTATTTTTT  360

Query  399   CTCTTCACCTTGCTGGTGCTTCTTCAATTTTAGGTGCAATTAACTTCATCACAACTATTG  458
             ||||||| |||||||||||||||||||||||||||||||||||||| || ||||||||||
```

```
Sbjct  361   CTCTTCATCTTGCTGGTGCTTCTTCAATTTTAGGTGCAATTAACTTTATTACAACTATTG  420

Query  459   TAAATATACGATCAATTGGAATAACAATAGAACGAATACCATTATTTGTTTGATCTGTTT  518
             |||| ||||||||||||||||||||||||||||| |||||||||||||||||||||||||
Sbjct  421   TAAACATACGATCAATTGGAATAACAATAGAACGTATACCATTATTTGTTTGATCTGTTT  480

Query  519   TAATTACAGCTATTTTACTACTTCTATCTTTACCTGTATTAGCAGGTGCCATTACAATAT  578
             ||||||||||||||||| ||||| ||||||||||||||||||||||||||||||||||||
Sbjct  481   TAATTACAGCTATTTTATTACTTTTATCTTTACCTGTATTAGCAGGTGCCATTACAATAT  540

Query  579   TATTAACTGATCGAAACTTTAACACATCATTTTTTGATCCTTCAGGAGGAGGAGATCCCA  638
             | |||||||||||||||||||| ||||||||||||||||||||||| ||||||||||| |
Sbjct  541   TGTTAACTGATCGAAACTTTAATACATCATTTTTTGATCCTTCAGGGGGAGGAGATCCAA  600

Query  639   TTTTATATCAACACTTATTT  658
             ||||||||||||| ||||||
Sbjct  601   TTTTATATCAACATTTATTT  620
```

>Rhipicephalus sanguineus isolate Petnica cytochrome oxidase subunit I (COI) gene, partial cds; mitochondrial
Sequence ID: KX757883.1 Length: 630
Range 1: 1 to 620

Score:985 bits(533), Expect:0.0,
Identities:591/620(95%), Gaps:0/620(0%), Strand: Plus/Plus

```
Query  39    TAGGACTTAGTATAAGAATATTAATTCGTATAGAATTAGGACAACCTGGAACTTTAATTG  98
             |||||||||||||||||||||||||||||||||| |||||||||||||||||||||||||
Sbjct  1     TAGGACTTAGTATAAGAATATTAATTCGTATAGAGTTAGGACAACCTGGAACTTTAATTG  60

Query  99    GAAATGATCAGATTTATAATGTAATCGTAACAGCACATGCATTTATTATAATTTTCTTTA  158
             ||||||||||||||||||||||||||||||||||||||||||||||||||||||| ||||
Sbjct  61    GAAATGATCAGATTTATAATGTAATCGTAACAGCACATGCATTTATTATAATTTTTTTTA  120

Query  159   TAGTTATACCAATCATAATCGGAGGGTTCGGAAATTGATTAGTTCCTATTATATTAGGTG  218
             ||||||||||||||||||||||||| || |||||||||||||| ||||| ||||| ||||
Sbjct  121   TAGTTATACCAATCATAATCGGAGGATTTGGAAATTGATTAGTCCCTATCATATTGGGTG  180

Query  219   CCCCAGATATAGCATTTCCACGAATAAATAATATAAGATTTTGACTTTTACCCCCCTCAT  278
             |||||||||||||||||||||||||||||||||||||||||||||||||||| || |||
Sbjct  181   CCCCAGATATAGCATTTCCACGAATAAATAATATAAGATTTTGACTTTTACCTCCTTCAC  240

Query  279   TATTTTTATTGATTAACTCTTCATTAATTGAATCCGGAGCTGGAACAGGATGAACAGTTT  338
             |||||||||| ||||||||||||||||||||||| |||||||| ||||||||||||||||
Sbjct  241   TATTTTTATTAATTAACTCTTCATTAATTGAATCTGGAGCTGGTACAGGATGAACAGTTT  300

Query  339   ATCCCCCTCTATCTTCAAATTTATCACATTATGGTCCATCAGTAGATTTAGCTATTTTTT  398
             | || ||||||||||||||||||||||||||||| || ||||||||||||||||||||||
Sbjct  301   ACCCTCCTCTATCTTCAAATTTATCACATTATGGCCCTTCAGTAGATTTAGCTATTTTTT  360

Query  399   CTCTTCACCTTGCTGGTGCTTCTTCAATTTTAGGTGCAATTAACTTCATCACAACTATTG  458
```

```
                ||||||| |||||||||||||||||||||||||||||||||||||| || ||||||||||
Sbjct  361   CTCTTCATCTTGCTGGTGCTTCTTCAATTTTAGGTGCAATTAACTTTATTACAACTATTG  420

Query  459   TAAATATACGATCAATTGGAATAACAATAGAACGAATACCATTATTTGTTTGATCTGTTT  518
             |||| ||||||||||||||||||||||||||||| |||||||||||||||||||||||||
Sbjct  421   TAAACATACGATCAATTGGAATAACAATAGAACGTATACCATTATTTGTTTGATCTGTTT  480

Query  519   TAATTACAGCTATTTTACTACTTCTATCTTTACCTGTATTAGCAGGTGCCATTACAATAT  578
             ||||||||||||||||| ||||| ||||||||||||||||||||||||||||||||||||
Sbjct  481   TAATTACAGCTATTTTATTACTTTTATCTTTACCTGTATTAGCAGGTGCCATTACAATAT  540

Query  579   TATTAACTGATCGAAACTTTAACACATCATTTTTTGATCCTTCAGGAGGAGGAGATCCCA  638
             | |||||||||||||||||||| |||||||||||||||||||||| |||||||||||| |
Sbjct  541   TGTTAACTGATCGAAACTTTAATACATCATTTTTTGATCCTTCAGGGGGAGGAGATCCAA  600

Query  639   TTTTATATCAACACTTATTT  658
             ||||||||||||| ||||||
Sbjct  601   TTTTATATCAACATTTATTT  620
```

>Rhipicephalus sanguineus clone #3 cytochrome oxidase subunit I (COI) gene, partial cds; mitochondrial
Sequence ID: KX519708.1 Length: 629
Range 1: 4 to 620

Score:985 bits(533), Expect:0.0,
Identities:589/617(95%), Gaps:0/617(0%), Strand: Plus/Plus

```
Query  42    GACTTAGTATAAGAATATTAATTCGTATAGAATTAGGACAACCTGGAACTTTAATTGGAA  101
             ||||||||||||||||||||||||||||||||||||||||||||||||||||||||||||
Sbjct  4     GACTTAGTATAAGAATATTAATTCGTATAGAATTAGGACAACCTGGAACTTTAATTGGAA  63

Query  102   ATGATCAGATTTATAATGTAATCGTAACAGCACATGCATTTATTATAATTTTCTTTATAG  161
             |||||||||||||||||||||||||||||||||||||||||||||||||||| |||||||
Sbjct  64    ATGATCAGATTTATAATGTAATCGTAACAGCACATGCATTTATTATAATTTTTTTTATAG  123

Query  162   TTATACCAATCATAATCGGAGGGTTCGGAAATTGATTAGTTCCTATTATATTAGGTGCCC  221
             |||||||||||||||||||||| || |||||||||||||| ||||| ||||| |||||||
Sbjct  124   TTATACCAATCATAATCGGAGGATTTGGAAATTGATTAGTCCCTATCATATTGGGTGCCC  183

Query  222   CAGATATAGCATTTCCACGAATAAATAATATAAGATTTTGACTTTTACCCCCCTCATTAT  281
             ||||||||||||||||||||||||||||||||||||||||||||||||| || ||| |||
Sbjct  184   CAGATATAGCATTTCCACGAATAAATAATATAAGATTTTGACTTTTACCTCCTTCACTAT  243

Query  282   TTTTATTGATTAACTCTTCATTAATTGAATCCGGAGCTGGAACAGGATGAACAGTTTATC  341
             ||||||| ||||||||||||||||||||||| |||||||| ||||||||||||||||| |
Sbjct  244   TTTTATTAATTAACTCTTCATTAATTGAATCTGGAGCTGGTACAGGATGAACAGTTTACC  303

Query  342   CCCCTCTATCTTCAAATTTATCACATTATGGTCCATCAGTAGATTTAGCTATTTTTTCTC  401
             | ||||||||||||||||||||||||||||| || |||||||||||||||||||||||||
Sbjct  304   CTCCTCTATCTTCAAATTTATCACATTATGGCCCTTCAGTAGATTTAGCTATTTTTTCTC  363
```

```
Query  402  TTCACCTTGCTGGTGCTTCTTCAATTTTAGGTGCAATTAACTTCATCACAACTATTGTAA  461
            |||| |||||||||||||||||||||||||||||||||||||| || |||||||||||||
Sbjct  364  TTCATCTTGCTGGTGCTTCTTCAATTTTAGGTGCAATTAACTTTATTACAACTATTGTAA  423

Query  462  ATATACGATCAATTGGAATAACAATAGAACGAATACCATTATTTGTTTGATCTGTTTTAA  521
            | ||||||||||||||||||||||||||||| ||||||||||||||||||||||||||||
Sbjct  424  ACATACGATCAATTGGAATAACAATAGAACGTATACCATTATTTGTTTGATCTGTTTTAA  483

Query  522  TTACAGCTATTTTACTACTTCTATCTTTACCTGTATTAGCAGGTGCCATTACAATATTAT  581
            |||||||||||||| ||||| ||||||||||||||||||||||||||||||||||||| |
Sbjct  484  TTACAGCTATTTTATTACTTTTATCTTTACCTGTATTAGCAGGTGCCATTACAATATTGT  543

Query  582  TAACTGATCGAAACTTTAACACATCATTTTTTGATCCTTCAGGAGGAGGAGATCCCATTT  641
            ||||||||||||||||||| ||||||||||||||||||||||| |||||||||| ||||
Sbjct  544  TAACTGATCGAAACTTTAATACATCATTTTTTGATCCTTCAGGGGGAGGAGATCCAATTT  603

Query  642  TATATCAACACTTATTT  658
            |||||||||| ||||||
Sbjct  604  TATATCAACATTTATTT  620
```

>Rhipicephalus sanguineus isolate SV2 cytochrome c oxidase subunit I (COX1) gene, partial cds; mitochondrial
Sequence ID: MW135447.1 Length: 630
Range 1: 1 to 620

Score:985 bits(533), Expect:0.0,
Identities:591/620(95%), Gaps:0/620(0%), Strand: Plus/Plus

```
Query  39   TAGGACTTAGTATAAGAATATTAATTCGTATAGAATTAGGACAACCTGGAACTTTAATTG  98
            ||||||||||||||||||||||||||||||||||||||||||||||||||||||||||||
Sbjct  1    TAGGACTTAGTATAAGAATATTAATTCGTATAGAATTAGGACAACCTGGAACTTTAATTG  60

Query  99   GAAATGATCAGATTTATAATGTAATCGTAACAGCACATGCATTTATTATAATTTTCTTTA  158
            ||||||||||||||||||||||||||||||||||||||||||||||||||||||| ||||
Sbjct  61   GAAATGATCAGATTTATAATGTAATCGTAACAGCACATGCATTTATTATAATTTTTTTTA  120

Query  159  TAGTTATACCAATCATAATCGGAGGGTTCGGAAATTGATTAGTTCCTATTATATTAGGTG  218
            ||||||||||||||||||||||||| || |||||||||||||| ||||| ||||| ||||
Sbjct  121  TAGTTATACCAATCATAATCGGAGGATTTGGAAATTGATTAGTCCCTATCATATTGGGTG  180

Query  219  CCCCAGATATAGCATTTCCACGAATAAATAATATAAGATTTTGACTTTTACCCCCCTCAT  278
            |||||||||||||||||||||||||||||||||||||||||||||||||||| || |||
Sbjct  181  CCCCAGATATAGCATTTCCACGAATAAATAATATAAGATTTTGACTTTTACCTCCTTCAC  240

Query  279  TATTTTTATTGATTAACTCTTCATTAATTGAATCCGGAGCTGGAACAGGATGAACAGTTT  338
            |||||||||| ||||||||||||||||||||||| |||||||| ||||||||||||||||
Sbjct  241  TATTTTTATTAATTAACTCTTCATTAATTGAATCTGGAGCTGGTACAGGATGAACAGTTT  300

Query  339  ATCCCCCTCTATCTTCAAATTTATCACATTATGGTCCATCAGTAGATTTAGCTATTTTTT  398
            | || ||||||||||||||||||||||||||||| || ||||||||||||||||||||||
Sbjct  301  ACCCTCCTCTATCTTCAAATTTATCACATTATGGCCCTTCAGTAGATTTAGCTATTTTTT  360
```

```
Query  399   CTCTTCACCTTGCTGGTGCTTCTTCAATTTTAGGTGCAATTAACTTCATCACAACTATTG  458
             ||||||| |||||||||||||||||||||||||||||||||||||| || ||||||||||
Sbjct  361   CTCTTCATCTTGCTGGTGCTTCTTCAATTTTAGGTGCAATTAACTTTATTACAACTATTG  420

Query  459   TAAATATACGATCAATTGGAATAACAATAGAACGAATACCATTATTTGTTTGATCTGTTT  518
             |||| ||||||||||||||||||||||||||||| |||||||||||||||||||||||||
Sbjct  421   TAAACATACGATCAATTGGAATAACAATAGAACGTATACCATTATTTGTTTGATCTGTTT  480

Query  519   TAATTACAGCTATTTTACTACTTCTATCTTTACCTGTATTAGCAGGTGCCATTACAATAT  578
             ||||||||||||||||| ||||| ||||||||||||||||||||||||||||||||||||
Sbjct  481   TAATTACAGCTATTTTATTACTTTTATCTTTACCTGTATTAGCAGGTGCCATTACAATAT  540

Query  579   TATTAACTGATCGAAACTTTAACACATCATTTTTTGATCCTTCAGGAGGAGGAGATCCCA  638
             | |||||||||||||||||||| ||||||||||||||||||||||| |||||||| || |
Sbjct  541   TGTTAACTGATCGAAACTTTAATACATCATTTTTTGATCCTTCAGGGGGAGGAGACCCAA  600

Query  639   TTTTATATCAACACTTATTT  658
             ||||||||||||| ||||||
Sbjct  601   TTTTATATCAACATTTATTT  620
```

>Rhipicephalus sanguineus clone #2 cytochrome oxidase subunit I (COI) gene, partial cds; mitochondrial
Sequence ID: KX519707.1 Length: 626
Range 1: 1 to 616

Score:983 bits(532), Expect:0.0,
Identities:588/616(95%), Gaps:0/616(0%), Strand: Plus/Plus

```
Query  43    ACTTAGTATAAGAATATTAATTCGTATAGAATTAGGACAACCTGGAACTTTAATTGGAAA  102
             ||||||||||||||||||||||||||||||||||||||||||||||||||||||||||||
Sbjct  1     ACTTAGTATAAGAATATTAATTCGTATAGAATTAGGACAACCTGGAACTTTAATTGGAAA  60

Query  103   TGATCAGATTTATAATGTAATCGTAACAGCACATGCATTTATTATAATTTTCTTTATAGT  162
             ||||||||||||||||||||||||||||||||||||||||||||||||||| ||||||||
Sbjct  61    TGATCAGATTTATAATGTAATCGTAACAGCACATGCATTTATTATAATTTTTTTTATAGT  120

Query  163   TATACCAATCATAATCGGAGGGTTCGGAAATTGATTAGTTCCTATTATATTAGGTGCCCC  222
             ||||||||||||||||||||| || |||||||||||||| ||||| ||||| ||||||||
Sbjct  121   TATACCAATCATAATCGGAGGATTTGGAAATTGATTAGTCCCTATCATATTGGGTGCCCC  180

Query  223   AGATATAGCATTTCCACGAATAAATAATATAAGATTTTGACTTTTACCCCCCTCATTATT  282
             |||||||||||||||||||||||||||||||||||||||||||||||| || ||| ||||
Sbjct  181   AGATATAGCATTTCCACGAATAAATAATATAAGATTTTGACTTTTACCTCCTTCACTATT  240

Query  283   TTTATTGATTAACTCTTCATTAATTGAATCCGGAGCTGGAACAGGATGAACAGTTTATCC  342
             |||||| ||||||||||||||||||||||| |||||||| ||||||||||||||||| ||
Sbjct  241   TTTATTAATTAACTCTTCATTAATTGAATCTGGAGCTGGTACAGGATGAACAGTTTACCC  300

Query  343   CCCTCTATCTTCAAATTTATCACATTATGGTCCATCAGTAGATTTAGCTATTTTTTCTCT  402
              ||||||||||||||||||||||||||||| || ||||||||||||||||||||||||||
```

```
Sbjct  301  TCCTCTATCTTCAAATTTATCACATTATGGCCCTTCAGTAGATTTAGCTATTTTTTCTCT  360

Query  403  TCACCTTGCTGGTGCTTCTTCAATTTTAGGTGCAATTAACTTCATCACAACTATTGTAAA  462
            ||| |||||||||||||||||||||||||||||||||||||| || ||||||||||||||
Sbjct  361  TCATCTTGCTGGTGCTTCTTCAATTTTAGGTGCAATTAACTTTATTACAACTATTGTAAA  420

Query  463  TATACGATCAATTGGAATAACAATAGAACGAATACCATTATTTGTTTGATCTGTTTTAAT  522
             ||||||||||||||||||||||||||||| |||||||||||||||||||||||||||||
Sbjct  421  CATACGATCAATTGGAATAACAATAGAACGTATACCATTATTTGTTTGATCTGTTTTAAT  480

Query  523  TACAGCTATTTTACTACTTCTATCTTTACCTGTATTAGCAGGTGCCATTACAATATTATT  582
            ||||||||||||| ||||| ||||||||||||||||||||||||||||||||||||| ||
Sbjct  481  TACAGCTATTTTATTACTTTTATCTTTACCTGTATTAGCAGGTGCCATTACAATATTGTT  540

Query  583  AACTGATCGAAACTTTAACACATCATTTTTTGATCCTTCAGGAGGAGGAGATCCCATTTT  642
            |||||||||||||||||| |||||||||||||||||||||||| |||||||||| |||||
Sbjct  541  AACTGATCGAAACTTTAATACATCATTTTTTGATCCTTCAGGGGGAGGAGATCCAATTTT  600

Query  643  ATATCAACACTTATTT  658
            ||||||||| ||||||
Sbjct  601  ATATCAACATTTATTT  616
```

>Rhipicephalus sanguineus isolate Szekszard cytochrome oxidase subunit I (COI) gene, partial cds; mitochondrial
Sequence ID: KX757901.1 Length: 630
Range 1: 1 to 620

Score:979 bits(530), Expect:0.0,
Identities:590/620(95%), Gaps:0/620(0%), Strand: Plus/Plus

```
Query  39   TAGGACTTAGTATAAGAATATTAATTCGTATAGAATTAGGACAACCTGGAACTTTAATTG  98
            ||||||||||||||||||||||||||||||||||||||||||||||||||||||||||||
Sbjct  1    TAGGACTTAGTATAAGAATATTAATTCGTATAGAATTAGGACAACCTGGAACTTTAATTG  60

Query  99   GAAATGATCAGATTTATAATGTAATCGTAACAGCACATGCATTTATTATAATTTTCTTTA  158
            ||||||||||||||||||||||||||||||||||||||||||||||||||||||| ||||
Sbjct  61   GAAATGATCAGATTTATAATGTAATCGTAACAGCACATGCATTTATTATAATTTTTTTTA  120

Query  159  TAGTTATACCAATCATAATCGGAGGGTTCGGAAATTGATTAGTTCCTATTATATTAGGTG  218
            ||||||||||||||||||||||||| || |||||||||||||| ||||| ||||| ||||
Sbjct  121  TAGTTATACCAATCATAATCGGAGGATTTGGAAATTGATTAGTCCCTATCATATTGGGTG  180

Query  219  CCCCAGATATAGCATTTCCACGAATAAATAATATAAGATTTTGACTTTTACCCCCCTCAT  278
            |||||||||||||||||||||||||||||||||||||||||||||||||||| || ||| 
Sbjct  181  CCCCAGATATAGCATTTCCACGAATAAATAATATAAGATTTTGACTTTTACCTCCTTCAC  240

Query  279  TATTTTTATTGATTAACTCTTCATTAATTGAATCCGGAGCTGGAACAGGATGAACAGTTT  338
            |||||||||| ||||||||||||||||||||||| |||||||| ||||||||||||||||
Sbjct  241  TATTTTTATTAATTAACTCTTCATTAATTGAATCTGGAGCTGGTACAGGATGAACAGTTT  300

Query  339  ATCCCCCTCTATCTTCAAATTTATCACATTATGGTCCATCAGTAGATTTAGCTATTTTTT  398
```

```
                | || ||||||||||||||||||||||||||||| || ||||||||||||||||| ||||
Sbjct  301  ACCCTCCTCTATCTTCAAATTTATCACATTATGGCCCTTCAGTAGATTTAGCTATCTTTT  360

Query  399  CTCTTCACCTTGCTGGTGCTTCTTCAATTTTAGGTGCAATTAACTTCATCACAACTATTG  458
            ||||||| |||||||||||||||||||||||||||||||||||||| || ||||||||||
Sbjct  361  CTCTTCATCTTGCTGGTGCTTCTTCAATTTTAGGTGCAATTAACTTTATTACAACTATTG  420

Query  459  TAAATATACGATCAATTGGAATAACAATAGAACGAATACCATTATTTGTTTGATCTGTTT  518
            |||| ||||| ||||||||||||||||||||||| |||||||||||||||||||||||||
Sbjct  421  TAAACATACGGTCAATTGGAATAACAATAGAACGTATACCATTATTTGTTTGATCTGTTT  480

Query  519  TAATTACAGCTATTTTACTACTTCTATCTTTACCTGTATTAGCAGGTGCCATTACAATAT  578
            ||||||||||||||||| ||||| ||||||||||||||||||||||||||||||||||||
Sbjct  481  TAATTACAGCTATTTTATTACTTTTATCTTTACCTGTATTAGCAGGTGCCATTACAATAT  540

Query  579  TATTAACTGATCGAAACTTTAACACATCATTTTTTGATCCTTCAGGAGGAGGAGATCCCA  638
            | |||||||||||||||||||| ||||||||||||||||||||||| ||||||||||| |
Sbjct  541  TGTTAACTGATCGAAACTTTAATACATCATTTTTTGATCCTTCAGGGGGAGGAGATCCAA  600

Query  639  TTTTATATCAACACTTATTT  658
            ||||||||||||| ||||||
Sbjct  601  TTTTATATCAACATTTATTT  620
```

>Rhipicephalus sanguineus clone #4 cytochrome oxidase subunit I (COI) gene, partial cds; mitochondrial
Sequence ID: KX519709.1 Length: 635
Range 1: 2 to 621

Score:974 bits(527), Expect:0.0,
Identities:589/620(95%), Gaps:0/620(0%), Strand: Plus/Plus

```
Query  39   TAGGACTTAGTATAAGAATATTAATTCGTATAGAATTAGGACAACCTGGAACTTTAATTG  98
            ||||||||||||||||||||||||||||||||||||||||||||||||||||||||||||
Sbjct  2    TAGGACTTAGTATAAGAATATTAATTCGTATAGAATTAGGACAACCTGGAACTTTAATTG  61

Query  99   GAAATGATCAGATTTATAATGTAATCGTAACAGCACATGCATTTATTATAATTTTCTTTA  158
            |||||||||||||||||||||||||||||||||||||||| |||||||||||||| ||||
Sbjct  62   GAAATGATCAGATTTATAATGTAATCGTAACAGCACATGCTTTTATTATAATTTTTTTTA  121

Query  159  TAGTTATACCAATCATAATCGGAGGGTTCGGAAATTGATTAGTTCCTATTATATTAGGTG  218
            ||||||||||||||||||||||||| || |||||||||||||| ||||| ||||| ||||
Sbjct  122  TAGTTATACCAATCATAATCGGAGGATTTGGAAATTGATTAGTCCCTATCATATTGGGTG  181

Query  219  CCCCAGATATAGCATTTCCACGAATAAATAATATAAGATTTTGACTTTTACCCCCCTCAT  278
            |||| ||||||||||||||||||||||||||||||||||||||||||||||| || |||
Sbjct  182  CCCCGGATATAGCATTTCCACGAATAAATAATATAAGATTTTGACTTTTACCTCCTTCAC  241

Query  279  TATTTTTATTGATTAACTCTTCATTAATTGAATCCGGAGCTGGAACAGGATGAACAGTTT  338
            |||||||||| ||||||||||||||||||||||| |||||||| ||||||||||||||||
Sbjct  242  TATTTTTATTAATTAACTCTTCATTAATTGAATCTGGAGCTGGTACAGGATGAACAGTTT  301
```

```
Query  339  ATCCCCCTCTATCTTCAAATTTATCACATTATGGTCCATCAGTAGATTTAGCTATTTTTT  398
            | || ||||||||||||||||||||||||||||| || |||| |||||||||||||||||
Sbjct  302  ACCCTCCTCTATCTTCAAATTTATCACATTATGGCCCTTCAGGAGATTTAGCTATTTTTT  361

Query  399  CTCTTCACCTTGCTGGTGCTTCTTCAATTTTAGGTGCAATTAACTTCATCACAACTATTG  458
            ||||||| |||||||||||||||||||||||||||||||||||||||  || ||||||||||
Sbjct  362  CTCTTCATCTTGCTGGTGCTTCTTCAATTTTAGGTGCAATTAACTTTATTACAACTATTG  421

Query  459  TAAATATACGATCAATTGGAATAACAATAGAACGAATACCATTATTTGTTTGATCTGTTT  518
            |||| ||||||||||||||||||||||||||||| |||||||||||||||||||||||||
Sbjct  422  TAAACATACGATCAATTGGAATAACAATAGAACGTATACCATTATTTGTTTGATCTGTTT  481

Query  519  TAATTACAGCTATTTTACTACTTCTATCTTTACCTGTATTAGCAGGTGCCATTACAATAT  578
            ||||||||||||||||| ||||| ||||||||||||||||||||||||||||||||||||
Sbjct  482  TAATTACAGCTATTTTATTACTTTTATCTTTACCTGTATTAGCAGGTGCCATTACAATAT  541

Query  579  TATTAACTGATCGAAACTTTAACACATCATTTTTTGATCCTTCAGGAGGAGGAGATCCCA  638
            | |||||||||||||||||||| ||||||||||||||||||||||| ||||||||||| |
Sbjct  542  TGTTAACTGATCGAAACTTTAATACATCATTTTTTGATCCTTCAGGGGGAGGAGATCCAA  601

Query  639  TTTTATATCAACACTTATTT  658
            ||||||||||||| ||||||
Sbjct  602  TTTTATATCAACATTTATTT  621
```

>Rhipicephalus sanguineus isolate LIC4723B cytochrome oxidase subunit 1 (COI) gene, partial cds; mitochondrial
Sequence ID: KX714599.1 Length: 723
>Rhipicephalus sanguineus isolate LIC4750B cytochrome oxidase subunit 1 (COI) gene, partial cds; mitochondrial
Sequence ID: KX714600.1 Length: 723
Range 1: 1 to 597

Score:937 bits(507), Expect:0.0,
Identities:567/597(95%), Gaps:0/597(0%), Strand: Plus/Plus

```
Query  62   ATTCGTATAGAATTAGGACAACCTGGAACTTTAATTGGAAATGATCAGATTTATAATGTA  121
            ||||||||||||||||||||||||||||||||||||||||||||||||||||||||||||
Sbjct  1    ATTCGTATAGAATTAGGACAACCTGGAACTTTAATTGGAAATGATCAGATTTATAATGTA  60

Query  122  ATCGTAACAGCACATGCATTTATTATAATTTTCTTTATAGTTATACCAATCATAATCGGA  181
            |||||||||||||||||||||||||||||||| ||||||||||||||||||||||| |||
Sbjct  61   ATCGTAACAGCACATGCATTTATTATAATTTTTTTTATAGTTATACCAATCATAATTGGA  120

Query  182  GGGTTCGGAAATTGATTAGTTCCTATTATATTAGGTGCCCCAGATATAGCATTTCCACGA  241
            || || |||||||||||||| ||||| ||||| |||||||||||||||||||||||||||
Sbjct  121  GGATTTGGAAATTGATTAGTCCCTATCATATTGGGTGCCCCAGATATAGCATTTCCACGA  180

Query  242  ATAAATAATATAAGATTTTGACTTTTACCCCCCTCATTATTTTTATTGATTAACTCTTCA  301
            ||||||||||||||||||||||||||||| || ||| |||||||||| ||||||||||||
Sbjct  181  ATAAATAATATAAGATTTTGACTTTTACCTCCTTCACTATTTTTATTAATTAACTCTTCA  240
```

```
Query  302  TTAATTGAATCCGGAGCTGGAACAGGATGAACAGTTTATCCCCCTCTATCTTCAAATTTA  361
            ||||||||||| |||||||| ||||||||||||||||| || ||||||||||||||||||
Sbjct  241  TTAATTGAATCTGGAGCTGGTACAGGATGAACAGTTTACCCTCCTCTATCTTCAAATTTA  300

Query  362  TCACATTATGGTCCATCAGTAGATTTAGCTATTTTTTCTCTTCACCTTGCTGGTGCTTCT  421
            ||||||||||| || ||||||||||||||||| ||||||||||| |||||||||||||||
Sbjct  301  TCACATTATGGCCCTTCAGTAGATTTAGCTATCTTTTCTCTTCATCTTGCTGGTGCTTCT  360

Query  422  TCAATTTTAGGTGCAATTAACTTCATCACAACTATTGTAAATATACGATCAATTGGAATA  481
            ||||||||||||||||||||||| || |||||||||||||| ||||||||||||||||||
Sbjct  361  TCAATTTTAGGTGCAATTAACTTTATTACAACTATTGTAAACATACGATCAATTGGAATA  420

Query  482  ACAATAGAACGAATACCATTATTTGTTTGATCTGTTTTAATTACAGCTATTTTACTACTT  541
            ||||||||||| |||||||||||||||||||||||||||||||||||||||||| |||||
Sbjct  421  ACAATAGAACGTATACCATTATTTGTTTGATCTGTTTTAATTACAGCTATTTTATTACTT  480

Query  542  CTATCTTTACCTGTATTAGCAGGTGCCATTACAATATTATTAACTGATCGAAACTTTAAC  601
             ||||||||||||||||||||||||||||||||||||| |||||||||||||||||||| 
Sbjct  481  TTATCTTTACCTGTATTAGCAGGTGCCATTACAATATTGTTAACTGATCGAAACTTTAAT  540

Query  602  ACATCATTTTTTGATCCTTCAGGAGGAGGAGATCCCATTTTATATCAACACTTATTT  658
            ||||||||||||||||||||||| ||||||||||| |||||||||||||| ||||||
Sbjct  541  ACATCATTTTTTGATCCTTCAGGGGGAGGAGATCCAATTTTATATCAACATTTATTT  597
```

>Rhipicephalus sanguineus clone #8 cytochrome oxidase subunit I (COI) gene, partial cds; mitochondrial
Sequence ID: KX519713.1 Length: 580
Range 1: 1 to 580

Score:933 bits(505), Expect:0.0,
Identities:555/580(96%), Gaps:0/580(0%), Strand: Plus/Plus

```
Query  43   ACTTAGTATAAGAATATTAATTCGTATAGAATTAGGACAACCTGGAACTTTAATTGGAAA  102
            ||||||||||||||||||||||||||||||||||||||||||||||||||||||||||||
Sbjct  1    ACTTAGTATAAGAATATTAATTCGTATAGAATTAGGACAACCTGGAACTTTAATTGGAAA  60

Query  103  TGATCAGATTTATAATGTAATCGTAACAGCACATGCATTTATTATAATTTTCTTTATAGT  162
            ||||||||||||||||||||||||||||||||||||||||||||||||||| ||||||||
Sbjct  61   TGATCAGATTTATAATGTAATCGTAACAGCACATGCATTTATTATAATTTTTTTTATAGT  120

Query  163  TATACCAATCATAATCGGAGGGTTCGGAAATTGATTAGTTCCTATTATATTAGGTGCCCC  222
            |||||||||||||||||||| || |||||||||||||| ||||| ||||| ||||||||
Sbjct  121  TATACCAATCATAATCGGAGGATTTGGAAATTGATTAGTCCCTATCATATTGGGTGCCCC  180

Query  223  AGATATAGCATTTCCACGAATAAATAATATAAGATTTTGACTTTTACCCCCCTCATTATT  282
            |||||||||||||||||||||||||||||||||||||||||||||||| || ||| ||||
Sbjct  181  AGATATAGCATTTCCACGAATAAATAATATAAGATTTTGACTTTTACCTCCTTCACTATT  240

Query  283  TTTATTGATTAACTCTTCATTAATTGAATCCGGAGCTGGAACAGGATGAACAGTTTATCC  342
            |||||| ||||||||||||||||||||||| |||||||| ||||||||||||||||| ||
Sbjct  241  TTTATTAATTAACTCTTCATTAATTGAATCTGGAGCTGGTACAGGATGAACAGTTTACCC  300
```

```
Query  343  CCCTCTATCTTCAAATTTATCACATTATGGTCCATCAGTAGATTTAGCTATTTTTTCTCT  402
             ||||||||||||||||||||||||||||| || ||||||||||||||||||||||||||
Sbjct  301  TCCTCTATCTTCAAATTTATCACATTATGGCCCTTCAGTAGATTTAGCTATTTTTTCTCT  360

Query  403  TCACCTTGCTGGTGCTTCTTCAATTTTAGGTGCAATTAACTTCATCACAACTATTGTAAA  462
            ||| |||||||||||||||||||||||||||||||||||||| || ||||||||||||||
Sbjct  361  TCATCTTGCTGGTGCTTCTTCAATTTTAGGTGCAATTAACTTTATTACAACTATTGTAAA  420

Query  463  TATACGATCAATTGGAATAACAATAGAACGAATACCATTATTTGTTTGATCTGTTTTAAT  522
             ||||||||||||||||||||||||||||| |||||||||||||||||||||||||||||
Sbjct  421  CATACGATCAATTGGAATAACAATAGAACGTATACCATTATTTGTTTGATCTGTTTTAAT  480

Query  523  TACAGCTATTTTACTACTTCTATCTTTACCTGTATTAGCAGGTGCCATTACAATATTATT  582
            ||||||||||||| ||||| ||||||||||||||||||||||||||||||||||||| ||
Sbjct  481  TACAGCTATTTTATTACTTTTATCTTTACCTGTATTAGCAGGTGCCATTACAATATTGTT  540

Query  583  AACTGATCGAAACTTTAACACATCATTTTTTGATCCTTCA  622
            |||||||||||||||||| |||||||||||||||||||||
Sbjct  541  AACTGATCGAAACTTTAATACATCATTTTTTGATCCTTCA  580
```

>Rhipicephalus sanguineus clone #1 cytochrome oxidase subunit I (COI) gene, partial cds; mitochondrial
Sequence ID: KX519706.1 Length: 595
Range 1: 1 to 570

Score:898 bits(486), Expect:0.0,
Identities:542/570(95%), Gaps:0/570(0%), Strand: Plus/Plus

```
Query  89   ACTTTAATTGGAAATGATCAGATTTATAATGTAATCGTAACAGCACATGCATTTATTATA  148
            ||||||||||||||||||||||||||||||||||||||||||||||||||||||||||||
Sbjct  1    ACTTTAATTGGAAATGATCAGATTTATAATGTAATCGTAACAGCACATGCATTTATTATA  60

Query  149  ATTTTCTTTATAGTTATACCAATCATAATCGGAGGGTTCGGAAATTGATTAGTTCCTATT  208
            ||||| ||||||||||||||||||||||||||||| || |||||||||||||| |||||
Sbjct  61   ATTTTTTTTATAGTTATACCAATCATAATCGGAGGATTTGGAAATTGATTAGTCCCTATC  120

Query  209  ATATTAGGTGCCCCAGATATAGCATTTCCACGAATAAATAATATAAGATTTTGACTTTTA  268
            ||||| ||||||||||||||||||||||||||||||||||||||||||||||||||||||
Sbjct  121  ATATTGGGTGCCCCAGATATAGCATTTCCACGAATAAATAATATAAGATTTTGACTTTTA  180

Query  269  CCCCCCTCATTATTTTTATTGATTAACTCTTCATTAATTGAATCCGGAGCTGGAACAGGA  328
            || || ||| |||||||||| ||||||||||||||||||||||| |||||||| ||||||
Sbjct  181  CCTCCTTCACTATTTTTATTAATTAACTCTTCATTAATTGAATCTGGAGCTGGTACAGGA  240

Query  329  TGAACAGTTTATCCCCCTCTATCTTCAAATTTATCACATTATGGTCCATCAGTAGATTTA  388
            ||||||||||| || ||||||||||||||||||||||||||||| || ||||||||||||
Sbjct  241  TGAACAGTTTACCCTCCTCTATCTTCAAATTTATCACATTATGGCCCTTCAGTAGATTTA  300

Query  389  GCTATTTTTTCTCTTCACCTTGCTGGTGCTTCTTCAATTTTAGGTGCAATTAACTTCATC  448
            ||||||||||||||||| |||||||||||||||||||||||||||||||||||||| ||
```

```
Sbjct  301   GCTATTTTTTCTCTTCATCTTGCTGGTGCTTCTTCAATTTTAGGTGCAATTAACTTTATT  360

Query  449   ACAACTATTGTAAATATACGATCAATTGGAATAACAATAGAACGAATACCATTATTTGTT  508
             |||||||||||||| ||||||||||||||||||||||||||||| |||||||||||||||
Sbjct  361   ACAACTATTGTAAACATACGATCAATTGGAATAACAATAGAACGTATACCATTATTTGTT  420

Query  509   TGATCTGTTTTAATTACAGCTATTTTACTACTTCTATCTTTACCTGTATTAGCAGGTGCC  568
             ||||||||||||||||||||||||||| ||||| ||||||||||||||||||||||||||
Sbjct  421   TGATCTGTTTTAATTACAGCTATTTTATTACTTTTATCTTTACCTGTATTAGCAGGTGCC  480

Query  569   ATTACAATATTATTAACTGATCGAAACTTTAACACATCATTTTTTGATCCTTCAGGAGGA  628
             ||||||||||| |||||||||||||||||||| ||||||||||||||||||||||| |||
Sbjct  481   ATTACAATATTGTTAACTGATCGAAACTTTAATACATCATTTTTTGATCCTTCAGGGGGA  540

Query  629   GGAGATCCCATTTTATATCAACACTTATTT  658
             |||||||| |||||||||||||| ||||||
Sbjct  541   GGAGATCCAATTTTATATCAACATTTATTT  570
```

>Rhipicephalus sanguineus isolate Rsang2 cytochrome oxidase subunit 1 gene,
partial cds; mitochondrial
Sequence ID: KY678135.1 Length: 790
Range 1: 18 to 675

Score:883 bits(478), Expect:0.0,
Identities:599/659(91%), Gaps:2/659(0%), Strand: Plus/Plus

```
Query  1     AACAATATATTTAATTTTTGGAGCATGATCAGGTATATTAGGACTTAGTATAAGAATATT  60
             |||||||||||||||||||||||||||||| || ||||||||| | || ||||||||| |
Sbjct  18    AACAATATATTTAATTTTTGGAGCATGATCCGGAATATTAGGATTAAGAATAAGAATACT  77

Query  61    AATTCGTATAGAATTAGGACAACCTGGAACTTTAATTGGAAATGATCAGATTTATAATGT  120
             |||||||||||||||||| |||||||||||  ||||||| |||||||| |||||||||||
Sbjct  78    AATTCGTATAGAATTAGGCCAACCTGGAACCCTAATTGGTAATGATCAAATTTATAATGT  137

Query  121   AATCGTAACAGCACATGCATTTATTATAATTTTCTTTATAGTTATACCAATCATAATCGG  180
             ||| || ||||| |||||||||||||||||||| |||||||| |||||||||||||| ||
Sbjct  138   AATTGTTACAGCTCATGCATTTATTATAATTTTTTTTATAGTAATACCAATCATAATTGG  197

Query  181   AGGGTTCGGAAATTGATTAGTTCCTATTATATTAGGTGCCCCAGATATAGCATTTCCACG  240
              || || ||||| |||||||||||||||||| |||| || |||||||||||||| |||||
Sbjct  198   TGGATTTGGAAACTGATTAGTTCCTATTATACTAGGAGCTCCAGATATAGCATTCCCACG  257

Query  241   AATAAATAATATAAGATTTTGACTTTTACCCCCCTCATTATTTTTATTGATTAACTCTTC  300
             ||||||||||||||||||||||||| | || |||||||||||| |||| ||||| |||||
Sbjct  258   AATAAATAATATAAGATTTTGACTTCTTCCTCCCTCATTATTTATATTAATTAATTCTTC  317

Query  301   ATTAATTGAATCCGGAGCTGGAACAGGATGAACAGTTTATCC-CCCTCTATCTTCAAATT  359
             ||||||||| || ||||| || |||||||||||||||||||| |||| |||| |||||||
Sbjct  318   ATTAATTGAGTCAGGAGCAGGTACAGGATGAACAGTTTATCCTCCCT-TATCCTCAAATT  376

Query  360   TATCACATTATGGTCCATCAGTAGATTTAGCTATTTTTTCTCTTCACCTTGCTGGTGCTT  419
```

```
             ||||||||||||| |||||||||||||||||||||||||||||||| |||||||||||||
Sbjct  377   TATCACATTATGGGCCATCAGTAGATTTAGCTATTTTTTCTCTTCATCTTGCTGGTGCTT  436

Query  420   CTTCAATTTTAGGTGCAATTAACTTCATCACAACTATTGTAAATATACGATCAATTGGAA  479
             |||||||||||||||||||||| || || ||||||||||||||||||||||| |||||||
Sbjct  437   CTTCAATTTTAGGTGCAATTAATTTTATTACAACTATTGTAAATATACGATCTATTGGAA  496

Query  480   TAACAATAGAACGAATACCATTATTTGTTTGATCTGTTTTAATTACAGCTATTTTACTAC  539
             |||||||||||||||||||||||||||| ||||||||||||||||| || |||||| |||
Sbjct  497   TAACAATAGAACGAATACCATTATTTGTATGATCTGTTTTAATTACTGCAATTTTATTAC  556

Query  540   TTCTATCTTTACCTGTATTAGCAGGTGCCATTACAATATTATTAACTGATCGAAACTTTA  599
             |  ||||||||||||| ||||||||||||||||||||| ||||||| |||||||| ||||
Sbjct  557   TATTATCTTTACCTGTTTTAGCAGGTGCCATTACAATACTATTAACCGATCGAAATTTTA  616

Query  600   ACACATCATTTTTTGATCCTTCAGGAGGAGGAGATCCCATTTTATATCAACACTTATTT  658
             |||| ||||||||||| |||||||| ||||| ||||| |||||||||||||| ||||||
Sbjct  617   ACACTTCATTTTTTGACCCTTCAGGGGGAGGGGATCCAATTTTATATCAACATTTATTT  675
```

>Rhipicephalus turanicus isolate COX1-225-Goat cytochrome c oxidase subunit I (COX1) gene, partial cds; mitochondrial
Sequence ID: MT800313.1 Length: 673
Range 1: 8 to 665

Score:883 bits(478), Expect:0.0,
Identities:598/658(91%), Gaps:0/658(0%), Strand: Plus/Plus

```
Query  1     AACAATATATTTAATTTTTGGAGCATGATCAGGTATATTAGGACTTAGTATAAGAATATT  60
             ||||||||||||||||||||| ||||||||||| ||||||||||| || ||||||||| |
Sbjct  8     AACAATATATTTAATTTTTGGGGCATGATCAGGAATATTAGGACTGAGAATAAGAATACT  67

Query  61    AATTCGTATAGAATTAGGACAACCTGGAACTTTAATTGGAAATGATCAGATTTATAATGT  120
             |||||| ||||||||||||||||||||||||||||||||||||||||| ||||||| ||
Sbjct  68    AATTCGCATAGAATTAGGACAACCTGGAACTTTAATTGGAAATGATCAAATTTATAACGT  127

Query  121   AATCGTAACAGCACATGCATTTATTATAATTTTCTTTATAGTTATACCAATCATAATCGG  180
             ||| |||||||| |||||||||||||||||||| ||||| || ||||| || ||||| ||
Sbjct  128   AATTGTAACAGCTCATGCATTTATTATAATTTTTTTTATGGTAATACCTATTATAATTGG  187

Query  181   AGGGTTCGGAAATTGATTAGTTCCTATTATATTAGGTGCCCCAGATATAGCATTTCCACG  240
              || || ||||||||||||||||||||||||||||| || |||||||||||||| |||||
Sbjct  188   TGGATTTGGAAATTGATTAGTTCCTATTATATTAGGGGCTCCAGATATAGCATTCCCACG  247

Query  241   AATAAATAATATAAGATTTTGACTTTTACCCCCCTCATTATTTTTATTGATTAACTCTTC  300
              |||||||| |||||||||||| |  | || || ||||||||| |||| |||||||||||
Sbjct  248   TATAAATAACATAAGATTTTGATTACTTCCTCCTTCATTATTTCTATTAATTAACTCTTC  307

Query  301   ATTAATTGAATCCGGAGCTGGAACAGGATGAACAGTTTATCCCCCTCTATCTTCAAATTT  360
             |||||||||||| ||||| |||||||||||||| |||||||||||| |||| ||||||||
Sbjct  308   ATTAATTGAATCAGGAGCAGGAACAGGATGAACTGTTTATCCCCCTTTATCCTCAAATTT  367
```

```
Query  361   ATCACATTATGGTCCATCAGTAGATTTAGCTATTTTTTCTCTTCACCTTGCTGGTGCTTC  420
             |||||||||||| |||||||||||||||||||||||||||||||| ||||||||||||||
Sbjct  368   ATCACATTATGGACCATCAGTAGATTTAGCTATTTTTTCTCTTCATCTTGCTGGTGCTTC  427

Query  421   TTCAATTTTAGGTGCAATTAACTTCATCACAACTATTGTAAATATACGATCAATTGGAAT  480
             ||||||| ||||||||||||| || || ||||||||||||||||||||||| ||||||||
Sbjct  428   TTCAATTCTAGGTGCAATTAATTTTATTACAACTATTGTAAATATACGATCTATTGGAAT  487

Query  481   AACAATAGAACGAATACCATTATTTGTTTGATCTGTTTTAATTACAGCTATTTTACTACT  540
             ||||||||||||||||||||||||||||||||||||||||||||| || || ||||| ||
Sbjct  488   AACAATAGAACGAATACCATTATTTGTTTGATCTGTTTTAATTACCGCCATCTTACTTCT  547

Query  541   TCTATCTTTACCTGTATTAGCAGGTGCCATTACAATATTATTAACTGATCGAAACTTTAA  600
               ||||||||||||| ||||||||||| |||||||||||||||||||||||||| || ||
Sbjct  548   CTTATCTTTACCTGTTTTAGCAGGTGCTATTACAATATTATTAACTGATCGAAATTTCAA  607

Query  601   CACATCATTTTTTGATCCTTCAGGAGGAGGAGATCCCATTTTATATCAACACTTATTT  658
              || |||||||||||||||||||| ||||| ||||| |||||||| ||||||||||||
Sbjct  608   TACTTCATTTTTTGATCCTTCAGGGGGAGGTGATCCAATTTTATACCAACACTTATTT  665
```

>Rhipicephalus sanguineus sensu lato 'temperate lineage' isolate SC3005 cytochrome c oxidase subunit I (COI) gene, partial cds; mitochondrial
Sequence ID: MF425987.1 Length: 574
Range 1: 1 to 561

Score:881 bits(477), Expect:0.0,
Identities:533/561(95%), Gaps:0/561(0%), Strand: Plus/Plus

```
Query  98    GGAAATGATCAGATTTATAATGTAATCGTAACAGCACATGCATTTATTATAATTTTCTTT  157
             |||||||||||||||||||||||||||||||||||||| ||||||||||||||||| |||
Sbjct  1     GGAAATGATCAGATTTATAATGTAATCGTAACAGCACACGCATTTATTATAATTTTTTTT  60

Query  158   ATAGTTATACCAATCATAATCGGAGGGTTCGGAAATTGATTAGTTCCTATTATATTAGGT  217
             |||||||||||||||||||||||||| || |||||||||||||| |||||||||||||||
Sbjct  61    ATAGTTATACCAATCATAATCGGAGGATTTGGAAATTGATTAGTCCCTATTATATTAGGT  120

Query  218   GCCCCAGATATAGCATTTCCACGAATAAATAATATAAGATTTTGACTTTTACCCCCCTCA  277
             ||||||||||||||||||||||||||||||||||||||||||||||||||||| || |||
Sbjct  121   GCCCCAGATATAGCATTTCCACGAATAAATAATATAAGATTTTGACTTTTACCTCCTTCA  180

Query  278   TTATTTTTATTGATTAACTCTTCATTAATTGAATCCGGAGCTGGAACAGGATGAACAGTT  337
              |||||||||| ||||||||||||||||||||||| |||||||| |||||||||||||||
Sbjct  181   CTATTTTTATTAATTAACTCTTCATTAATTGAATCTGGAGCTGGTACAGGATGAACAGTT  240

Query  338   TATCCCCCTCTATCTTCAAATTTATCACATTATGGTCCATCAGTAGATTTAGCTATTTTT  397
             || || ||||||||||||||||||||||||||||| || ||||||||||||||||| |||
Sbjct  241   TACCCTCCTCTATCTTCAAATTTATCACATTATGGCCCTTCAGTAGATTTAGCTATCTTT  300

Query  398   TCTCTTCACCTTGCTGGTGCTTCTTCAATTTTAGGTGCAATTAACTTCATCACAACTATT  457
             |||||||| |||||||||||||||||||||||||||||||||||||| || |||||||||
Sbjct  301   TCTCTTCATCTTGCTGGTGCTTCTTCAATTTTAGGTGCAATTAACTTTATTACAACTATT  360
```

```
Query  458   GTAAATATACGATCAATTGGAATAACAATAGAACGAATACCATTATTTGTTTGATCTGTT  517
             ||||| ||||||||||||||||||||||||||||| ||||||||||||||||||||||||
Sbjct  361   GTAAACATACGATCAATTGGAATAACAATAGAACGTATACCATTATTTGTTTGATCTGTT  420

Query  518   TTAATTACAGCTATTTTACTACTTCTATCTTTACCTGTATTAGCAGGTGCCATTACAATA  577
             |||||||||||||||||| ||||| |||||||||||||||||||||||||||||||||||
Sbjct  421   TTAATTACAGCTATTTTATTACTTTTATCTTTACCTGTATTAGCAGGTGCCATTACAATA  480

Query  578   TTATTAACTGATCGAAACTTTAACACATCATTTTTTGATCCTTCAGGAGGAGGAGATCCC  637
             || |||||||||||||||||||| ||||||||||||||||||||||| |||||||||||
Sbjct  481   TTGTTAACTGATCGAAACTTTAATACATCATTTTTTGATCCTTCAGGGGGAGGAGATCCA  540

Query  638   ATTTTATATCAACACTTATTT  658
             |||||||||||||| ||||||
Sbjct  541   ATTTTATATCAACATTTATTT  561
```

>Rhipicephalus sanguineus cytochrome oxidase subunit 1 (CO1) gene, partial cds; mitochondrial
Sequence ID: KU214592.1 Length: 829
Range 1: 1 to 648

Score:876 bits(474), Expect:0.0,
Identities:590/648(91%), Gaps:0/648(0%), Strand: Plus/Plus

```
Query  11    TTAATTTTTGGAGCATGATCAGGTATATTAGGACTTAGTATAAGAATATTAATTCGTATA  70
             |||||||||||||||||||| || ||||||||| | || ||||||||| |||||||||||
Sbjct  1     TTAATTTTTGGAGCATGATCCGGAATATTAGGATTAAGAATAAGAATACTAATTCGTATA  60

Query  71    GAATTAGGACAACCTGGAACTTTAATTGGAAATGATCAGATTTATAATGTAATCGTAACA  130
             |||||||| |||||||||||| ||||||| |||||||| |||||||||||||| || |||
Sbjct  61    GAATTAGGCCAACCTGGAACTCTAATTGGTAATGATCAAATTTATAATGTAATTGTTACA  120

Query  131   GCACATGCATTTATTATAATTTTCTTTATAGTTATACCAATCATAATCGGAGGGTTCGGA  190
             || |||||||||||||||||||| ||||||| ||||||||||||||| || || || |||
Sbjct  121   GCTCATGCATTTATTATAATTTTTTTTATAGTAATACCAATCATAATTGGTGGATTTGGA  180

Query  191   AATTGATTAGTTCCTATTATATTAGGTGCCCCAGATATAGCATTTCCACGAATAAATAAT  250
             || |||||||||||||||||| |||| || ||||||||||||||||||||||||||||||
Sbjct  181   AACTGATTAGTTCCTATTATACTAGGAGCTCCAGATATAGCATTTCCACGAATAAATAAT  240

Query  251   ATAAGATTTTGACTTTTACCCCCCTCATTATTTTTATTGATTAACTCTTCATTAATTGAA  310
             ||||||||||||||| | || |||||||||||| |||| ||||| ||||||||||||||
Sbjct  241   ATAAGATTTTGACTTCTTCCTCCCTCATTATTTATATTAATTAATTCTTCATTAATTGAG  300

Query  311   TCCGGAGCTGGAACAGGATGAACAGTTTATCCCCCTCTATCTTCAAATTTATCACATTAT  370
             || ||||| || |||||||| ||||||||||| ||| |||| ||||||||||||||||||
Sbjct  301   TCAGGAGCAGGTACAGGATGGACAGTTTATCCTCCCCTATCCTCAAATTTATCACATTAT  360

Query  371   GGTCCATCAGTAGATTTAGCTATTTTTTCTCTTCACCTTGCTGGTGCTTCTTCAATTTTA  430
             || ||||||||||||||||||||||||||||||| |||||||||||||||||||||||||
```

```
Sbjct  361   GGGCCATCAGTAGATTTAGCTATTTTTTCTCTTCATCTTGCTGGTGCTTCTTCAATTTTA  420

Query  431   GGTGCAATTAACTTCATCACAACTATTGTAAATATACGATCAATTGGAATAACAATAGAA  490
             ||||||||||| || || ||||||||||||||||||||||| ||||||||||||||||||
Sbjct  421   GGTGCAATTAATTTTATTACAACTATTGTAAATATACGATCTATTGGAATAACAATAGAA  480

Query  491   CGAATACCATTATTTGTTTGATCTGTTTTAATTACAGCTATTTTACTACTTCTATCTTTA  550
             ||||||||||||||||| ||||||||||||||||| || |||||| ||||  ||||||||
Sbjct  481   CGAATACCATTATTTGTATGATCTGTTTTAATTACTGCAATTTTATTACTATTATCTTTA  540

Query  551   CCTGTATTAGCAGGTGCCATTACAATATTATTAACTGATCGAAACTTTAACACATCATTT  610
             ||||| ||||||||||||||||||||||||||||| |||||||| |||||||| ||||||
Sbjct  541   CCTGTTTTAGCAGGTGCCATTACAATATTATTAACCGATCGAAATTTTAACACTTCATTT  600

Query  611   TTTGATCCTTCAGGAGGAGGAGATCCCATTTTATATCAACACTTATTT  658
             ||||| |||||||| ||||| ||||| |||||||||||||| ||||||
Sbjct  601   TTTGACCCTTCAGGGGGAGGGGATCCAATTTTATATCAACATTTATTT  648
```

>Rhipicephalus turanicus isolate GY34-1 cytochrome c oxidase subunit I (COX1) gene, partial cds; mitochondrial
Sequence ID: MT079206.1 Length: 678
Range 1: 1 to 652

Score:872 bits(472), Expect:0.0,
Identities:592/652(91%), Gaps:0/652(0%), Strand: Plus/Plus

```
Query  7     ATATTTAATTTTTGGAGCATGATCAGGTATATTAGGACTTAGTATAAGAATATTAATTCG  66
             ||||||||||||||||||||||||||||||||||||| | || |||||||||||||||||
Sbjct  1     ATATTTAATTTTTGGAGCATGATCAGGTATATTAGGATTAAGAATAAGAATATTAATTCG  60

Query  67    TATAGAATTAGGACAACCTGGAACTTTAATTGGAAATGATCAGATTTATAATGTAATCGT  126
              ||||||||||||||||||||||||||||||||||||||||| |||||||||||||| ||
Sbjct  61    AATAGAATTAGGACAACCTGGAACTTTAATTGGAAATGATCAAATTTATAATGTAATTGT  120

Query  127   AACAGCACATGCATTTATTATAATTTTCTTTATAGTTATACCAATCATAATCGGAGGGTT  186
             |||||| |||||||||||||||||||| |||||||| |||||||||||||| ||||| ||
Sbjct  121   AACAGCTCATGCATTTATTATAATTTTTTTTATAGTAATACCAATCATAATTGGAGGATT  180

Query  187   CGGAAATTGATTAGTTCCTATTATATTAGGTGCCCCAGATATAGCATTTCCACGAATAAA  246
              ||||| || |||||||| ||||||||||| || ||||| ||||||||||||||||||||
Sbjct  181   TGGAAACTGGTTAGTTCCAATTATATTAGGAGCTCCAGACATAGCATTTCCACGAATAAA  240

Query  247   TAATATAAGATTTTGACTTTTACCCCCCTCATTATTTTTATTGATTAACTCTTCATTAAT  306
             |||||||||||||||| |  | |||||||||||||| ||||| ||||| |||||| | ||
Sbjct  241   TAATATAAGATTTTGATTACTTCCCCCCTCATTATTCTTATTAATTAATTCTTCACTGAT  300

Query  307   TGAATCCGGAGCTGGAACAGGATGAACAGTTTATCCCCCTCTATCTTCAAATTTATCACA  366
             |||||| || || || ||||| ||||| |||||||| ||| |||| ||||||||||| ||
Sbjct  301   TGAATCTGGGGCAGGGACAGGGTGAACTGTTTATCCTCCTTTATCCTCAAATTTATCCCA  360

Query  367   TTATGGTCCATCAGTAGATTTAGCTATTTTTTCTCTTCACCTTGCTGGTGCTTCTTCAAT  426
```

```
                ||||||| |||||||||||||||||||| || |||||||| ||||||||||||||||||||
Sbjct  361   TTATGGACCATCAGTAGATTTAGCTATCTTCTCTCTTCATCTTGCTGGTGCTTCTTCAAT  420

Query  427   TTTAGGTGCAATTAACTTCATCACAACTATTGTAAATATACGATCAATTGGAATAACAAT  486
             |||||| |||||||| ||||| |||||||||||||| |||||||| ||||||||||||||
Sbjct  421   TTTAGGCGCAATTAATTTCATTACAACTATTGTAAACATACGATCTATTGGAATAACAAT  480

Query  487   AGAACGAATACCATTATTTGTTTGATCTGTTTTAATTACAGCTATTTTACTACTTCTATC  546
             |||||||||||||||||| ||||||||||||||||| || ||||||||| | ||||||||
Sbjct  481   AGAACGAATACCATTATTCGTTTGATCTGTTTTAATCACCGCTATTTTATTGCTTCTATC  540

Query  547   TTTACCTGTATTAGCAGGTGCCATTACAATATTATTAACTGATCGAAACTTTAACACATC  606
             ||||||||| ||||||||||||||||||||| |||||||||||||||| |||||||| ||
Sbjct  541   TTTACCTGTTTTAGCAGGTGCCATTACAATACTATTAACTGATCGAAATTTTAACACTTC  600

Query  607   ATTTTTTGATCCTTCAGGAGGAGGAGATCCCATTTTATATCAACACTTATTT  658
             ||||||||| |||||||||||||| || || |||||||||||||| ||||||
Sbjct  601   ATTTTTTGACCCTTCAGGAGGAGGTGACCCAATTTTATATCAACATTTATTT  652
```

>Rhipicephalus sanguineus isolate GY34 cytochrome c oxidase subunit I (COX1) gene, partial cds; mitochondrial
Sequence ID: MN862754.1 Length: 678
Range 1: 1 to 652

Score:872 bits(472), Expect:0.0,
Identities:592/652(91%), Gaps:0/652(0%), Strand: Plus/Plus

```
Query  7     ATATTTAATTTTTGGAGCATGATCAGGTATATTAGGACTTAGTATAAGAATATTAATTCG  66
             ||||||||||||||||||||||||||||||||||||| | || |||||||||||||||||
Sbjct  1     ATATTTAATTTTTGGAGCATGATCAGGTATATTAGGATTAAGAATAAGAATATTAATTCG  60

Query  67    TATAGAATTAGGACAACCTGGAACTTTAATTGGAAATGATCAGATTTATAATGTAATCGT  126
              ||||||||||||||||||||||||||||||||||||||||| |||||||||||||| ||
Sbjct  61    AATAGAATTAGGACAACCTGGAACTTTAATTGGAAATGATCAAATTTATAATGTAATTGT  120

Query  127   AACAGCACATGCATTTATTATAATTTTCTTTATAGTTATACCAATCATAATCGGAGGGTT  186
             |||||| |||||||||||||||||||| |||||||| |||||||||||||| ||||| ||
Sbjct  121   AACAGCTCATGCATTTATTATAATTTTTTTTATAGTAATACCAATCATAATTGGAGGATT  180

Query  187   CGGAAATTGATTAGTTCCTATTATATTAGGTGCCCCAGATATAGCATTTCCACGAATAAA  246
              ||||| || |||||||| ||||||||||| || ||||| ||||||||||||||||||||
Sbjct  181   TGGAAACTGGTTAGTTCCAATTATATTAGGAGCTCCAGACATAGCATTTCCACGAATAAA  240

Query  247   TAATATAAGATTTTGACTTTTACCCCCCTCATTATTTTTATTGATTAACTCTTCATTAAT  306
             |||||||||||||||| |  | |||||||||||||| ||||| ||||| |||||| | ||
Sbjct  241   TAATATAAGATTTTGATTACTTCCCCCCTCATTATTCTTATTAATTAATTCTTCACTGAT  300

Query  307   TGAATCCGGAGCTGGAACAGGATGAACAGTTTATCCCCCTCTATCTTCAAATTTATCACA  366
             |||||| || || || ||||| ||||| |||||||| ||| |||| ||||||||||| ||
Sbjct  301   TGAATCTGGGGCAGGGACAGGGTGAACTGTTTATCCTCCTTTATCCTCAAATTTATCCCA  360
```

```
Query  367  TTATGGTCCATCAGTAGATTTAGCTATTTTTTCTCTTCACCTTGCTGGTGCTTCTTCAAT  426
            |||||| |||||||||||||||||||| || |||||||| ||||||||||||||||||||
Sbjct  361  TTATGGACCATCAGTAGATTTAGCTATCTTCTCTCTTCATCTTGCTGGTGCTTCTTCAAT  420

Query  427  TTTAGGTGCAATTAACTTCATCACAACTATTGTAAATATACGATCAATTGGAATAACAAT  486
            |||||| |||||||| ||||| |||||||||||||| |||||||| ||||||||||||||
Sbjct  421  TTTAGGCGCAATTAATTTCATTACAACTATTGTAAACATACGATCTATTGGAATAACAAT  480

Query  487  AGAACGAATACCATTATTTGTTTGATCTGTTTTAATTACAGCTATTTTACTACTTCTATC  546
            |||||||||||||||||| ||||||||||||||||| || ||||||||| | ||||||||
Sbjct  481  AGAACGAATACCATTATTCGTTTGATCTGTTTTAATCACCGCTATTTTATTGCTTCTATC  540

Query  547  TTTACCTGTATTAGCAGGTGCCATTACAATATTATTAACTGATCGAAACTTTAACACATC  606
            ||||||||| ||||||||||||||||||||| |||||||||||||||| |||||||| ||
Sbjct  541  TTTACCTGTTTTAGCAGGTGCCATTACAATACTATTAACTGATCGAAATTTTAACACTTC  600

Query  607  ATTTTTTGATCCTTCAGGAGGAGGAGATCCCATTTTATATCAACACTTATTT  658
            ||||||||| |||||||||||||| || || |||||||||||||| ||||||
Sbjct  601  ATTTTTTGACCCTTCAGGAGGAGGTGACCCAATTTTATATCAACATTTATTT  652
```

>Rhipicephalus sanguineus isolate Gilan-e Gharb cytochrome oxidase subunit 1 gene, complete cds; mitochondrial
Sequence ID: KM494916.1 Length: 1539
Range 1: 45 to 702

Score:872 bits(472), Expect:0.0,
Identities:596/658(91%), Gaps:0/658(0%), Strand: Plus/Plus

```
Query  1    AACAATATATTTAATTTTTGGAGCATGATCAGGTATATTAGGACTTAGTATAAGAATATT  60
            |||||||||||||||||||||||||||||| || ||||||||| | || ||||||||| |
Sbjct  45   AACAATATATTTAATTTTTGGAGCATGATCCGGAATATTAGGATTAAGAATAAGAATACT  104

Query  61   AATTCGTATAGAATTAGGACAACCTGGAACTTTAATTGGAAATGATCAGATTTATAATGT  120
            |||||||||||||||||| |||||||||||| ||||||| |||||||| |||||||||||
Sbjct  105  AATTCGTATAGAATTAGGTCAACCTGGAACTCTAATTGGTAATGATCAAATTTATAATGT  164

Query  121  AATCGTAACAGCACATGCATTTATTATAATTTTCTTTATAGTTATACCAATCATAATCGG  180
            ||| || ||||| |||||||||||||||||||| |||||||| |||||||| ||||| ||
Sbjct  165  AATTGTTACAGCTCATGCATTTATTATAATTTTTTTTATAGTAATACCAATTATAATTGG  224

Query  181  AGGGTTCGGAAATTGATTAGTTCCTATTATATTAGGTGCCCCAGATATAGCATTTCCACG  240
             || || ||||| |||||||| ||||||||| |||| || |||||||||||||| |||||
Sbjct  225  TGGATTTGGAAACTGATTAGTACCTATTATACTAGGAGCTCCAGATATAGCATTCCCACG  284

Query  241  AATAAATAATATAAGATTTTGACTTTTACCCCCCTCATTATTTTTATTGATTAACTCTTC  300
            ||||||||||||||||||||||||| | || |||||||||||| |||| ||||| |||||
Sbjct  285  AATAAATAATATAAGATTTTGACTTCTTCCTCCCTCATTATTTATATTAATTAATTCTTC  344

Query  301  ATTAATTGAATCCGGAGCTGGAACAGGATGAACAGTTTATCCCCCTCTATCTTCAAATTT  360
            ||||||||| || ||||| || |||||||||||||||||||| || ||||| ||||||||
Sbjct  345  ATTAATTGAGTCAGGAGCAGGTACAGGATGAACAGTTTATCCTCCCCTATCCTCAAATTT  404
```

```
Query  361  ATCACATTATGGTCCATCAGTAGATTTAGCTATTTTTTCTCTTCACCTTGCTGGTGCTTC  420
            |||||||||||| |||||||||||||||||||||||||||||||| ||||||||||||||
Sbjct  405  ATCACATTATGGGCCATCAGTAGATTTAGCTATTTTTTCTCTTCATCTTGCTGGTGCTTC  464

Query  421  TTCAATTTTAGGTGCAATTAACTTCATCACAACTATTGTAAATATACGATCAATTGGAAT  480
            ||||||||||||||||||||| || || ||||||||||| ||||||||||| ||||||||
Sbjct  465  TTCAATTTTAGGTGCAATTAATTTTATTACAACTATTGTGAATATACGATCTATTGGAAT  524

Query  481  AACAATAGAACGAATACCATTATTTGTTTGATCTGTTTTAATTACAGCTATTTTACTACT  540
            ||||||||||||||||||||||||||| ||||||||||||||||| || |||||| ||||
Sbjct  525  AACAATAGAACGAATACCATTATTTGTATGATCTGTTTTAATTACTGCAATTTTATTACT  584

Query  541  TCTATCTTTACCTGTATTAGCAGGTGCCATTACAATATTATTAACTGATCGAAACTTTAA  600
              ||||||||||||| ||||||||||| ||||||||| ||||||| |||||||| |||||
Sbjct  585  ATTATCTTTACCTGTTTTAGCAGGTGCTATTACAATACTATTAACCGATCGAAATTTTAA  644

Query  601  CACATCATTTTTTGATCCTTCAGGAGGAGGAGATCCCATTTTATATCAACACTTATTT  658
            ||| ||||||||||| |||||||||||||| ||||| |||||||||||||| ||||||
Sbjct  645  CACTTCATTTTTTGACCCTTCAGGAGGAGGGGATCCAATTTTATATCAACATTTATTT  702
```

>Rhipicephalus sanguineus isolate Tehran cytochrome oxidase subunit 1 gene, complete cds; mitochondrial
Sequence ID: KM494915.1 Length: 1539
Range 1: 45 to 702

Score:872 bits(472), Expect:0.0,
Identities:596/658(91%), Gaps:0/658(0%), Strand: Plus/Plus

```
Query  1    AACAATATATTTAATTTTTGGAGCATGATCAGGTATATTAGGACTTAGTATAAGAATATT  60
            |||||||||||||||||||||||||||||| || ||||||||| | || ||||||||| |
Sbjct  45   AACAATATATTTAATTTTTGGAGCATGATCCGGAATATTAGGATTAAGAATAAGAATACT  104

Query  61   AATTCGTATAGAATTAGGACAACCTGGAACTTTAATTGGAAATGATCAGATTTATAATGT  120
            |||||||||||||||||| |||||||||||| ||||||| |||||||| |||||||||||
Sbjct  105  AATTCGTATAGAATTAGGTCAACCTGGAACTCTAATTGGTAATGATCAAATTTATAATGT  164

Query  121  AATCGTAACAGCACATGCATTTATTATAATTTTCTTTATAGTTATACCAATCATAATCGG  180
            ||| || ||||| |||||||||||||||||||| |||||||| |||||||| ||||| ||
Sbjct  165  AATTGTTACAGCTCATGCATTTATTATAATTTTTTTTATAGTAATACCAATTATAATTGG  224

Query  181  AGGGTTCGGAAATTGATTAGTTCCTATTATATTAGGTGCCCCAGATATAGCATTTCCACG  240
             || || ||||| |||||||| ||||||||| |||| || |||||||||||||| |||||
Sbjct  225  TGGATTTGGAAACTGATTAGTACCTATTATACTAGGAGCTCCAGATATAGCATTCCCACG  284

Query  241  AATAAATAATATAAGATTTTGACTTTTACCCCCCTCATTATTTTTATTGATTAACTCTTC  300
            ||||||||||||||||||||||||| | || |||||||||||| |||| ||||| |||||
Sbjct  285  AATAAATAATATAAGATTTTGACTTCTTCCTCCCTCATTATTTATATTAATTAATTCTTC  344

Query  301  ATTAATTGAATCCGGAGCTGGAACAGGATGAACAGTTTATCCCCCTCTATCTTCAAATTT  360
            ||||||||| || ||||| || |||||||||||||||||||| || ||||| ||||||||
```

```
Sbjct  345  ATTAATTGAGTCAGGAGCAGGTACAGGATGAACAGTTTATCCTCCCCTATCCTCAAATTT  404

Query  361  ATCACATTATGGTCCATCAGTAGATTTAGCTATTTTTTCTCTTCACCTTGCTGGTGCTTC  420
            |||||||||||| |||||||||||||||||||||||||||||||| ||||||||||||||
Sbjct  405  ATCACATTATGGGCCATCAGTAGATTTAGCTATTTTTTCTCTTCATCTTGCTGGTGCTTC  464

Query  421  TTCAATTTTAGGTGCAATTAACTTCATCACAACTATTGTAAATATACGATCAATTGGAAT  480
            ||||||||||||||||||||| || || ||||||||||| ||||||||||| ||||||||
Sbjct  465  TTCAATTTTAGGTGCAATTAATTTTATTACAACTATTGTGAATATACGATCTATTGGAAT  524

Query  481  AACAATAGAACGAATACCATTATTTGTTTGATCTGTTTTAATTACAGCTATTTTACTACT  540
            ||||||||||||||||||||||||||| ||||||||||||||||| || |||||| ||||
Sbjct  525  AACAATAGAACGAATACCATTATTTGTATGATCTGTTTTAATTACTGCAATTTTATTACT  584

Query  541  TCTATCTTTACCTGTATTAGCAGGTGCCATTACAATATTATTAACTGATCGAAACTTTAA  600
              ||||||||||||| ||||||||||| ||||||||| ||||||| |||||||| |||||
Sbjct  585  ATTATCTTTACCTGTTTTAGCAGGTGCTATTACAATACTATTAACCGATCGAAATTTTAA  644

Query  601  CACATCATTTTTTGATCCTTCAGGAGGAGGAGATCCCATTTTATATCAACACTTATTT  658
            ||| ||||||||||| |||||||||||||| ||||| |||||||||||||| ||||||
Sbjct  645  CACTTCATTTTTTGACCCTTCAGGAGGAGGGGATCCAATTTTATATCAACATTTATTT  702
```

>Rhipicephalus turanicus isolate COX1-18-Sheep cytochrome c oxidase subunit I (COX1) gene, partial cds; mitochondrial
Sequence ID: MT800314.1 Length: 673
Range 1: 8 to 664

Score:870 bits(471), Expect:0.0,
Identities:595/657(91%), Gaps:0/657(0%), Strand: Plus/Plus

```
Query  1    AACAATATATTTAATTTTTGGAGCATGATCAGGTATATTAGGACTTAGTATAAGAATATT  60
            ||||||||||||||||||||| ||||||||||| ||||||||||| || ||||||||| |
Sbjct  8    AACAATATATTTAATTTTTGGGGCATGATCAGGAATATTAGGACTGAGAATAAGAATACT  67

Query  61   AATTCGTATAGAATTAGGACAACCTGGAACTTTAATTGGAAATGATCAGATTTATAATGT  120
            |||||| ||||||||||||||||||||||||||||||||||||||||| |||||||| ||
Sbjct  68   AATTCGCATAGAATTAGGACAACCTGGAACTTTAATTGGAAATGATCAAATTTATAACGT  127

Query  121  AATCGTAACAGCACATGCATTTATTATAATTTTCTTTATAGTTATACCAATCATAATCGG  180
            ||| |||||||| |||||||||||||||||||| ||||| || ||||| || ||||| ||
Sbjct  128  AATTGTAACAGCTCATGCATTTATTATAATTTTTTTTATGGTAATACCCATTATAATTGG  187

Query  181  AGGGTTCGGAAATTGATTAGTTCCTATTATATTAGGTGCCCCAGATATAGCATTTCCACG  240
             || || ||||||||||||||||||||||||||||| || |||||||||||||| |||||
Sbjct  188  TGGATTTGGAAATTGATTAGTTCCTATTATATTAGGGGCTCCAGATATAGCATTCCCACG  247

Query  241  AATAAATAATATAAGATTTTGACTTTTACCCCCCTCATTATTTTTATTGATTAACTCTTC  300
             |||||||| |||||||||||| |  | || || ||||||||| |||| |||||||| ||
Sbjct  248  TATAAATAACATAAGATTTTGATTACTTCCTCCTTCATTATTTCTATTAATTAACTCCTC  307

Query  301  ATTAATTGAATCCGGAGCTGGAACAGGATGAACAGTTTATCCCCCTCTATCTTCAAATTT  360
```

```
                ||||||||||||| ||||| |||||||||||||| |||||||||||| |||| ||||||||
Sbjct  308  ATTAATTGAATCGGGAGCAGGAACAGGATGAACTGTTTATCCCCCTTTATCCTCAAATTT  367

Query  361  ATCACATTATGGTCCATCAGTAGATTTAGCTATTTTTTCTCTTCACCTTGCTGGTGCTTC  420
            |||||||||||| ||||||||||||||||||||||||||||||||| ||||||||||||||
Sbjct  368  ATCACATTATGGACCATCAGTAGATTTAGCTATTTTTTCTCTTCATCTTGCTGGTGCTTC  427

Query  421  TTCAATTTTAGGTGCAATTAACTTCATCACAACTATTGTAAATATACGATCAATTGGAAT  480
            ||||||| ||||||||||||| || || ||||||||||||||| ||||||| ||||||||
Sbjct  428  TTCAATTCTAGGTGCAATTAATTTTATTACAACTATTGTAAACATACGATCTATTGGAAT  487

Query  481  AACAATAGAACGAATACCATTATTTGTTTGATCTGTTTTAATTACAGCTATTTTACTACT  540
            ||||||||||||||||||||||||||||||||||||||||||||| || || ||||| ||
Sbjct  488  AACAATAGAACGAATACCATTATTTGTTTGATCTGTTTTAATTACCGCCATCTTACTTCT  547

Query  541  TCTATCTTTACCTGTATTAGCAGGTGCCATTACAATATTATTAACTGATCGAAACTTTAA  600
              ||||||||||||| ||||||||||| |||||||||||||||||||||||||| || ||
Sbjct  548  CTTATCTTTACCTGTTTTAGCAGGTGCTATTACAATATTATTAACTGATCGAAATTTCAA  607

Query  601  CACATCATTTTTTGATCCTTCAGGAGGAGGAGATCCCATTTTATATCAACACTTATT  657
             || |||||||||||||||||||| ||||| ||||| |||||||| |||||||||||
Sbjct  608  TACTTCATTTTTTGATCCTTCAGGGGGAGGTGATCCAATTTTATACCAACACTTATT  664
```

>Rhipicephalus turanicus isolate 31X cytochrome oxidase subunit 1 (COI) gene, partial cds; mitochondrial
Sequence ID: KY606303.1 Length: 873
Range 1: 66 to 723

Score:861 bits(466), Expect:0.0,
Identities:594/658(90%), Gaps:0/658(0%), Strand: Plus/Plus

```
Query  1    AACAATATATTTAATTTTTGGAGCATGATCAGGTATATTAGGACTTAGTATAAGAATATT  60
            |||||||||||||||||||||||||||||| || ||||||||| | || |||||||||||
Sbjct  66   AACAATATATTTAATTTTTGGAGCATGATCCGGGATATTAGGATTAAGAATAAGAATATT  125

Query  61   AATTCGTATAGAATTAGGACAACCTGGAACTTTAATTGGAAATGATCAGATTTATAATGT  120
            |||||| ||||| ||||||||||||||||||||||||||||||||||| |||||||||||
Sbjct  126  AATTCGAATAGAGTTAGGACAACCTGGAACTTTAATTGGAAATGATCAAATTTATAATGT  185

Query  121  AATCGTAACAGCACATGCATTTATTATAATTTTCTTTATAGTTATACCAATCATAATCGG  180
            ||| |||||||| |||||||||||||||||||| |||||||| |||||||||||||| ||
Sbjct  186  AATTGTAACAGCTCATGCATTTATTATAATTTTTTTTATAGTAATACCAATCATAATTGG  245

Query  181  AGGGTTCGGAAATTGATTAGTTCCTATTATATTAGGTGCCCCAGATATAGCATTTCCACG  240
            ||| || ||||| |||||||| || ||||| ||||| || ||||| ||||||||||||||
Sbjct  246  AGGATTTGGAAACTGATTAGTGCCCATTATGTTAGGAGCTCCAGACATAGCATTTCCACG  305

Query  241  AATAAATAATATAAGATTTTGACTTTTACCCCCCTCATTATTTTTATTGATTAACTCTTC  300
            |||||||||||||||||||||| |  | || ||||||||||| ||||| ||||| |||||
Sbjct  306  AATAAATAATATAAGATTTTGATTACTTCCTCCCTCATTATTCTTATTAATTAATTCTTC  365
```

```
Query  301   ATTAATTGAATCCGGAGCTGGAACAGGATGAACAGTTTATCCCCCTCTATCTTCAAATTT  360
             | | |||||||| ||||| || ||||||||||| || || || ||| |||||||||||||
Sbjct  366   ACTGATTGAATCTGGAGCAGGGACAGGATGAACTGTCTACCCTCCTTTATCTTCAAATTT  425

Query  361   ATCACATTATGGTCCATCAGTAGATTTAGCTATTTTTTCTCTTCACCTTGCTGGTGCTTC  420
             ||| |||||||| |||||||||||||||||||| || |||||||| || |||||||||||
Sbjct  426   ATCGCATTATGGACCATCAGTAGATTTAGCTATCTTCTCTCTTCATCTCGCTGGTGCTTC  485

Query  421   TTCAATTTTAGGTGCAATTAACTTCATCACAACTATTGTAAATATACGATCAATTGGAAT  480
             ||||||||||||||||||||| ||||| |||||||||||||| |||||||| ||||||||
Sbjct  486   TTCAATTTTAGGTGCAATTAATTTCATTACAACTATTGTAAACATACGATCTATTGGAAT  545

Query  481   AACAATAGAACGAATACCATTATTTGTTTGATCTGTTTTAATTACAGCTATTTTACTACT  540
             |||||||||||||||||||||||| ||||||||||||||||| || ||||||||| | ||
Sbjct  546   AACAATAGAACGAATACCATTATTCGTTTGATCTGTTTTAATCACCGCTATTTTATTGCT  605

Query  541   TCTATCTTTACCTGTATTAGCAGGTGCCATTACAATATTATTAACTGATCGAAACTTTAA  600
             ||||||||||||||| |||||||||||||||||||||||||||||||||||||| || ||
Sbjct  606   TCTATCTTTACCTGTTTTAGCAGGTGCCATTACAATATTATTAACTGATCGAAATTTCAA  665

Query  601   CACATCATTTTTTGATCCTTCAGGAGGAGGAGATCCCATTTTATATCAACACTTATTT  658
             ||| ||||||||||| |||||||||||||| || || |||||||||||||| ||||||
Sbjct  666   CACTTCATTTTTTGACCCTTCAGGAGGAGGTGACCCTATTTTATATCAACATTTATTT  723
```

>Rhipicephalus turanicus isolate 19X cytochrome oxidase subunit 1 (COI) gene, partial cds; mitochondrial
Sequence ID: KY606302.1 Length: 873
Range 1: 66 to 723

Score:861 bits(466), Expect:0.0,
Identities:594/658(90%), Gaps:0/658(0%), Strand: Plus/Plus

```
Query  1     AACAATATATTTAATTTTTGGAGCATGATCAGGTATATTAGGACTTAGTATAAGAATATT  60
             |||||||||||||||||||||||||||||| || ||||||||| | || |||||||||||
Sbjct  66    AACAATATATTTAATTTTTGGAGCATGATCCGGGATATTAGGATTAAGAATAAGAATATT  125

Query  61    AATTCGTATAGAATTAGGACAACCTGGAACTTTAATTGGAAATGATCAGATTTATAATGT  120
             |||||| ||||| ||||||||||||||||||||||||||||||||||| |||||||||||
Sbjct  126   AATTCGAATAGAGTTAGGACAACCTGGAACTTTAATTGGAAATGATCAAATTTATAATGT  185

Query  121   AATCGTAACAGCACATGCATTTATTATAATTTTCTTTATAGTTATACCAATCATAATCGG  180
             ||| |||||||| |||||||||||||||||||| |||||||| |||||||||||||| ||
Sbjct  186   AATTGTAACAGCTCATGCATTTATTATAATTTTTTTTATAGTAATACCAATCATAATTGG  245

Query  181   AGGGTTCGGAAATTGATTAGTTCCTATTATATTAGGTGCCCCAGATATAGCATTTCCACG  240
             ||| || ||||| |||||||| || ||||| ||||| || ||||| ||||||||||||||
Sbjct  246   AGGATTTGGAAACTGATTAGTGCCCATTATGTTAGGAGCTCCAGACATAGCATTTCCACG  305

Query  241   AATAAATAATATAAGATTTTGACTTTTACCCCCCTCATTATTTTTATTGATTAACTCTTC  300
             |||||||||||||||||||||| |  | || ||||||||||| ||||| ||||| |||||
Sbjct  306   AATAAATAATATAAGATTTTGATTACTTCCTCCCTCATTATTCTTATTAATTAATTCTTC  365
```

```
Query  301   ATTAATTGAATCCGGAGCTGGAACAGGATGAACAGTTTATCCCCCTCTATCTTCAAATTT  360
             | | |||||||| ||||| || ||||||||||| || || || ||| |||||||||||||
Sbjct  366   ACTGATTGAATCTGGAGCAGGGACAGGATGAACTGTCTACCCTCCTTTATCTTCAAATTT  425

Query  361   ATCACATTATGGTCCATCAGTAGATTTAGCTATTTTTTCTCTTCACCTTGCTGGTGCTTC  420
             ||| |||||||| |||||||||||||||||||| || |||||||| || |||||||||||
Sbjct  426   ATCGCATTATGGACCATCAGTAGATTTAGCTATCTTCTCTCTTCATCTCGCTGGTGCTTC  485

Query  421   TTCAATTTTAGGTGCAATTAACTTCATCACAACTATTGTAAATATACGATCAATTGGAAT  480
             ||||||||||||||||||||| ||||| |||||||||||||| |||||||| ||||||||
Sbjct  486   TTCAATTTTAGGTGCAATTAATTTCATTACAACTATTGTAAACATACGATCTATTGGAAT  545

Query  481   AACAATAGAACGAATACCATTATTTGTTTGATCTGTTTTAATTACAGCTATTTTACTACT  540
             |||||||||||||||||||||||| ||||||||||||||||| || ||||||||| | ||
Sbjct  546   AACAATAGAACGAATACCATTATTCGTTTGATCTGTTTTAATCACCGCTATTTTATTGCT  605

Query  541   TCTATCTTTACCTGTATTAGCAGGTGCCATTACAATATTATTAACTGATCGAAACTTTAA  600
             ||||||||||||||| |||||||||||||||||||||||||||||||||||||| || ||
Sbjct  606   TCTATCTTTACCTGTTTTAGCAGGTGCCATTACAATATTATTAACTGATCGAAATTTCAA  665

Query  601   CACATCATTTTTTGATCCTTCAGGAGGAGGAGATCCCATTTTATATCAACACTTATTT  658
             ||| ||||||||||| |||||||||||||| || || |||||||||||||| ||||||
Sbjct  666   CACTTCATTTTTTGACCCTTCAGGAGGAGGTGACCCTATTTTATATCAACATTTATTT  723
```

>Rhipicephalus turanicus isolate 19C cytochrome oxidase subunit 1 (COI) gene, partial cds; mitochondrial
Sequence ID: KY606301.1 Length: 873
Range 1: 66 to 723

Score:861 bits(466), Expect:0.0,
Identities:594/658(90%), Gaps:0/658(0%), Strand: Plus/Plus

```
Query  1     AACAATATATTTAATTTTTGGAGCATGATCAGGTATATTAGGACTTAGTATAAGAATATT  60
             |||||||||||||||||||||||||||||| || ||||||||| | || |||||||||||
Sbjct  66    AACAATATATTTAATTTTTGGAGCATGATCCGGGATATTAGGATTAAGAATAAGAATATT  125

Query  61    AATTCGTATAGAATTAGGACAACCTGGAACTTTAATTGGAAATGATCAGATTTATAATGT  120
             |||||| ||||| ||||||||||||||||||||||||||||||||||| |||||||||||
Sbjct  126   AATTCGAATAGAGTTAGGACAACCTGGAACTTTAATTGGAAATGATCAAATTTATAATGT  185

Query  121   AATCGTAACAGCACATGCATTTATTATAATTTTCTTTATAGTTATACCAATCATAATCGG  180
             ||| |||||||| |||||||||||||||||||| |||||||| |||||||||||||| ||
Sbjct  186   AATTGTAACAGCTCATGCATTTATTATAATTTTTTTTATAGTAATACCAATCATAATTGG  245

Query  181   AGGGTTCGGAAATTGATTAGTTCCTATTATATTAGGTGCCCCAGATATAGCATTTCCACG  240
             ||| || ||||| |||||||| || ||||| ||||| || ||||| ||||||||||||||
Sbjct  246   AGGATTTGGAAACTGATTAGTGCCCATTATGTTAGGAGCTCCAGACATAGCATTTCCACG  305

Query  241   AATAAATAATATAAGATTTTGACTTTTACCCCCCCTCATTATTTTATTGATTAACTCTTC  300
             |||||||||||||||||||||| |  | || ||||||||||| ||||| ||||| |||||
```

```
Sbjct  306  AATAAATAATATAAGATTTTGATTACTTCCTCCCTCATTATTCTTATTAATTAATTCTTC  365

Query  301  ATTAATTGAATCCGGAGCTGGAACAGGATGAACAGTTTATCCCCCTCTATCTTCAAATTT  360
            | | |||||||| ||||| || ||||||||||| || || || ||| |||||||||||||
Sbjct  366  ACTGATTGAATCTGGAGCAGGGACAGGATGAACTGTCTACCCTCCTTTATCTTCAAATTT  425

Query  361  ATCACATTATGGTCCATCAGTAGATTTAGCTATTTTTTCTCTTCACCTTGCTGGTGCTTC  420
            ||| |||||||| |||||||||||||||||||| || |||||||| || |||||||||||
Sbjct  426  ATCGCATTATGGACCATCAGTAGATTTAGCTATCTTCTCTCTTCATCTCGCTGGTGCTTC  485

Query  421  TTCAATTTTAGGTGCAATTAACTTCATCACAACTATTGTAAATATACGATCAATTGGAAT  480
            ||||||||||||||||||||| ||||| |||||||||||||| |||||||| ||||||||
Sbjct  486  TTCAATTTTAGGTGCAATTAATTTCATTACAACTATTGTAAACATACGATCTATTGGAAT  545

Query  481  AACAATAGAACGAATACCATTATTTGTTTGATCTGTTTTAATTACAGCTATTTTACTACT  540
            |||||||||||||||||||||||| ||||||||||||||||| || ||||||||| | ||
Sbjct  546  AACAATAGAACGAATACCATTATTCGTTTGATCTGTTTTAATCACCGCTATTTTATTGCT  605

Query  541  TCTATCTTTACCTGTATTAGCAGGTGCCATTACAATATTATTAACTGATCGAAACTTTAA  600
            ||||||||||||||| |||||||||||||||||||||||||||||||||||||| || ||
Sbjct  606  TCTATCTTTACCTGTTTTAGCAGGTGCCATTACAATATTATTAACTGATCGAAATTTCAA  665

Query  601  CACATCATTTTTTGATCCTTCAGGAGGAGGAGATCCCATTTTATATCAACACTTATTT  658
            ||| ||||||||||| |||||||||||||| || || |||||||||||||| ||||||
Sbjct  666  CACTTCATTTTTTGACCCTTCAGGAGGAGGTGACCCTATTTTATATCAACATTTATTT  723
```

>Rhipicephalus turanicus isolate 12X cytochrome oxidase subunit 1 (COI) gene, partial cds; mitochondrial
Sequence ID: KY606300.1 Length: 873
Range 1: 66 to 723

Score:861 bits(466), Expect:0.0,
Identities:594/658(90%), Gaps:0/658(0%), Strand: Plus/Plus

```
Query  1    AACAATATATTTAATTTTTGGAGCATGATCAGGTATATTAGGACTTAGTATAAGAATATT  60
            |||||||||||||||||||||||||||||| || ||||||||| | || |||||||||||
Sbjct  66   AACAATATATTTAATTTTTGGAGCATGATCCGGGATATTAGGATTAAGAATAAGAATATT  125

Query  61   AATTCGTATAGAATTAGGACAACCTGGAACTTTAATTGGAAATGATCAGATTTATAATGT  120
            |||||| ||||| ||||||||||||||||||||||||||||||||||| |||||||||||
Sbjct  126  AATTCGAATAGAGTTAGGACAACCTGGAACTTTAATTGGAAATGATCAAATTTATAATGT  185

Query  121  AATCGTAACAGCACATGCATTTATTATAATTTTCTTTATAGTTATACCAATCATAATCGG  180
            ||| |||||||| |||||||||||||||||||| |||||||| |||||||||||||| ||
Sbjct  186  AATTGTAACAGCTCATGCATTTATTATAATTTTTTTTATAGTAATACCAATCATAATTGG  245

Query  181  AGGGTTCGGAAATTGATTAGTTCCTATTATATTAGGTGCCCCAGATATAGCATTTCCACG  240
            ||| || ||||| |||||||| || ||||| ||||| || ||||| ||||||||||||||
Sbjct  246  AGGATTTGGAAACTGATTAGTGCCCATTATGTTAGGAGCTCCAGACATAGCATTTCCACG  305

Query  241  AATAAATAATATAAGATTTTGACTTTACCCCCCTCATTATTTTTATTGATTAACTCTTC  300
```

```
                ||||||||||||||||||||||| |  | || ||||||||||| ||||| ||||| |||||
Sbjct  306   AATAAATAATATAAGATTTTGATTACTTCCTCCCTCATTATTCTTATTAATTAATTCTTC  365

Query  301   ATTAATTGAATCCGGAGCTGGAACAGGATGAACAGTTTATCCCCCTCTATCTTCAAATTT  360
             | | |||||||| ||||| || ||||||||||| || || || ||| |||||||||||||
Sbjct  366   ACTGATTGAATCTGGAGCAGGGACAGGATGAACTGTCTACCCTCCTTTATCTTCAAATTT  425

Query  361   ATCACATTATGGTCCATCAGTAGATTTAGCTATTTTTTCTCTTCACCTTGCTGGTGCTTC  420
             ||| |||||||| |||||||||||||||||||| || |||||||| || |||||||||||
Sbjct  426   ATCGCATTATGGACCATCAGTAGATTTAGCTATCTTCTCTCTTCATCTCGCTGGTGCTTC  485

Query  421   TTCAATTTTAGGTGCAATTAACTTCATCACAACTATTGTAAATATACGATCAATTGGAAT  480
             ||||||||||||||||||||| ||||| |||||||||||||| |||||||| ||||||||
Sbjct  486   TTCAATTTTAGGTGCAATTAATTTCATTACAACTATTGTAAACATACGATCTATTGGAAT  545

Query  481   AACAATAGAACGAATACCATTATTTGTTTGATCTGTTTTAATTACAGCTATTTTACTACT  540
             |||||||||||||||||||||||| ||||||||||||||||| || ||||||||| | ||
Sbjct  546   AACAATAGAACGAATACCATTATTCGTTTGATCTGTTTTAATCACCGCTATTTTATTGCT  605

Query  541   TCTATCTTTACCTGTATTAGCAGGTGCCATTACAATATTATTAACTGATCGAAACTTTAA  600
             ||||||||||||||| |||||||||||||||||||||||||||||||||||||| || ||
Sbjct  606   TCTATCTTTACCTGTTTTAGCAGGTGCCATTACAATATTATTAACTGATCGAAATTTCAA  665

Query  601   CACATCATTTTTTGATCCTTCAGGAGGAGGAGATCCCATTTTATATCAACACTTATTT  658
             ||| ||||||||||| |||||||||||||| || || |||||||||||||| ||||||
Sbjct  666   CACTTCATTTTTTGACCCTTCAGGAGGAGGTGACCCTATTTTATATCAACATTTATTT  723
```

>Rhipicephalus turanicus isolate 12C cytochrome oxidase subunit 1 (COI) gene, partial cds; mitochondrial
Sequence ID: KY606299.1 Length: 873
Range 1: 66 to 723

Score:861 bits(466), Expect:0.0,
Identities:594/658(90%), Gaps:0/658(0%), Strand: Plus/Plus

```
Query  1     AACAATATATTTAATTTTTGGAGCATGATCAGGTATATTAGGACTTAGTATAAGAATATT  60
             |||||||||||||||||||||||||||||| || ||||||||| | || ||||||||||||
Sbjct  66    AACAATATATTTAATTTTTGGAGCATGATCCGGGATATTAGGATTAAGAATAAGAATATT  125

Query  61    AATTCGTATAGAATTAGGACAACCTGGAACTTTAATTGGAAATGATCAGATTTATAATGT  120
             |||||| ||||| ||||||||||||||||||||||||||||||||||| |||||||||||
Sbjct  126   AATTCGAATAGAGTTAGGACAACCTGGAACTTTAATTGGAAATGATCAAATTTATAATGT  185

Query  121   AATCGTAACAGCACATGCATTTATTATAATTTTCTTTATAGTTATACCAATCATAATCGG  180
             ||| |||||||| |||||||||||||||||||| |||||||| |||||||||||||| ||
Sbjct  186   AATTGTAACAGCTCATGCATTTATTATAATTTTTTTTATAGTAATACCAATCATAATTGG  245

Query  181   AGGGTTCGGAAATTGATTAGTTCCTATTATATTAGGTGCCCCAGATATAGCATTTCCACG  240
             ||| || ||||| |||||||| || ||||| ||||| || ||||| ||||||||||||||
Sbjct  246   AGGATTTGGAAACTGATTAGTGCCCATTATGTTAGGAGCTCCAGACATAGCATTTCCACG  305
```

```
Query  241  AATAAATAATATAAGATTTTGACTTTTACCCCCCTCATTATTTTTATTGATTAACTCTTC  300
            |||||||||||||||||||||| |  | || ||||||||||| ||||| ||||| |||||
Sbjct  306  AATAAATAATATAAGATTTTGATTACTTCCTCCCTCATTATTCTTATTAATTAATTCTTC  365

Query  301  ATTAATTGAATCCGGAGCTGGAACAGGATGAACAGTTTATCCCCCTCTATCTTCAAATTT  360
            | | |||||||| ||||| || ||||||||||| || || || ||| |||||||||||||
Sbjct  366  ACTGATTGAATCTGGAGCAGGGACAGGATGAACTGTCTACCCTCCTTTATCTTCAAATTT  425

Query  361  ATCACATTATGGTCCATCAGTAGATTTAGCTATTTTTTCTCTTCACCTTGCTGGTGCTTC  420
            ||| |||||||| |||||||||||||||||||| || |||||||| || |||||||||||
Sbjct  426  ATCGCATTATGGACCATCAGTAGATTTAGCTATCTTCTCTCTTCATCTCGCTGGTGCTTC  485

Query  421  TTCAATTTTAGGTGCAATTAACTTCATCACAACTATTGTAAATATACGATCAATTGGAAT  480
            ||||||||||||||||||||| ||||| |||||||||||||| |||||||| ||||||||
Sbjct  486  TTCAATTTTAGGTGCAATTAATTTCATTACAACTATTGTAAACATACGATCTATTGGAAT  545

Query  481  AACAATAGAACGAATACCATTATTTGTTTGATCTGTTTTAATTACAGCTATTTTACTACT  540
            |||||||||||||||||||||||| ||||||||||||||||| || ||||||||| | ||
Sbjct  546  AACAATAGAACGAATACCATTATTCGTTTGATCTGTTTTAATCACCGCTATTTTATTGCT  605

Query  541  TCTATCTTTACCTGTATTAGCAGGTGCCATTACAATATTATTAACTGATCGAAACTTTAA  600
            ||||||||||||||| |||||||||||||||||||||||||||||||||||||| || ||
Sbjct  606  TCTATCTTTACCTGTTTTAGCAGGTGCCATTACAATATTATTAACTGATCGAAATTTCAA  665

Query  601  CACATCATTTTTTGATCCTTCAGGAGGAGGAGATCCCATTTTATATCAACACTTATTT  658
            ||| ||||||||||| |||||||||||||| || || |||||||||||||| ||||||
Sbjct  666  CACTTCATTTTTTGACCCTTCAGGAGGAGGTGACCCTATTTTATATCAACATTTATTT  723
```

>Rhipicephalus camicasi isolate SC0126 mitochondrion, complete genome
Sequence ID: NC_061616.1 Length: 14725
>Rhipicephalus camicasi isolate SC0126 mitochondrion, complete genome
Sequence ID: MZ323229.1 Length: 14725
Range 1: 45 to 702

Score:861 bits(466), Expect:0.0,
Identities:595/659(90%), Gaps:2/659(0%), Strand: Plus/Plus

```
Query  1    AACAATATATTTAATTTTTGGAGCATGATCAGGTATATTAGGACTTAGTATAAGAATATT  60
            ||||||||| |||||||||||||||||||| || ||||||||| | || ||||||||| |
Sbjct  45   AACAATATACTTAATTTTTGGAGCATGATCTGGAATATTAGGATTAAGAATAAGAATACT  104

Query  61   AATTCGTATAGAATTAGGACAACCTGGAACTTTAATTGGAAATGATCAGATTTATAATGT  120
            |||||||||||||||||| |||||||||||||||||||||||||| || |||||||||||
Sbjct  105  AATTCGTATAGAATTAGGCCAACCTGGAACTTTAATTGGAAATGACCAAATTTATAATGT  164

Query  121  AATCGTAACAGCACATGCATTTATTATAATTTTCTTTATAGTTATACCAATCATAATCGG  180
            ||| ||||||||||||||||||||||||||||| |||||||| |||||||| || || ||
Sbjct  165  AATTGTAACAGCACATGCATTTATTATAATTTTTTTTATAGTAATACCAATTATGATTGG  224

Query  181  AGGGTTCGGAAATTGATTAGTTCCTATTATATTAGGTGCCCCAGATATAGCATTTCCACG  240
            ||| ||||||||||||||||| |||||||||||||| || ||||||||||||||||||||
```

```
Sbjct  225  AGGATTCGGAAATTGATTAGTCCCTATTATATTAGGGGCTCCAGATATAGCATTTCCACG  284

Query  241  AATAAATAATATAAGATTTTGACTTTTACCCCCCTCATTATTTTTATTGATTAACTCTTC  300
            |||||||||||||||||||||| |  | || || |||||||||||||| ||||| |||||
Sbjct  285  AATAAATAATATAAGATTTTGATTACTTCCTCCTTCATTATTTTTATTAATTAATTCTTC  344

Query  301  ATTAATTGAATCCGGAGCTGGAACAGGATGAACAGTTTATCC-CCCTCTATCTTCAAATT  359
            |||||||||||| ||||| || ||||||||||||||||| || |||| |||| |||||||
Sbjct  345  ATTAATTGAATCAGGAGCAGGGACAGGATGAACAGTTTACCCTCCCT-TATCATCAAATT  403

Query  360  TATCACATTATGGTCCATCAGTAGATTTAGCTATTTTTCTCTTCACCTTGCTGGTGCTT  419
            ||||||| |||||||||||||||||| |||||||||||||||||| ||||||||||||||
Sbjct  404  TATCACACTATGGTCCATCAGTAGATCTAGCTATTTTTCTCTTCATCTTGCTGGTGCTT  463

Query  420  CTTCAATTTTAGGTGCAATTAACTTCATCACAACTATTGTAAATATACGATCAATTGGAA  479
            ||||||||||||| |||||||| ||||| ||||| |||||||| |||||||| |||||||
Sbjct  464  CTTCAATTTTAGGCGCAATTAATTTCATTACAACCATTGTAAACATACGATCTATTGGAA  523

Query  480  TAACAATAGAACGAATACCATTATTTGTTTGATCTGTTTTAATTACAGCTATTTTACTAC  539
            ||||||||||||||||||| |||||||| ||||||||||||||||| || |||||| || 
Sbjct  524  TAACAATAGAACGAATACCTTTATTTGTATGATCTGTTTTAATTACTGCAATTTTATTAT  583

Query  540  TTCTATCTTTACCTGTATTAGCAGGTGCCATTACAATATTATTAACTGATCGAAACTTTA  599
            |  |||||||||| || ||||| ||||| |||||||||||||||||||||||||| ||||
Sbjct  584  TATTATCTTTACCAGTTTTAGCTGGTGCAATTACAATATTATTAACTGATCGAAATTTTA  643

Query  600  ACACATCATTTTTTGATCCTTCAGGAGGAGGAGATCCCATTTTATATCAACACTTATTT  658
            | || || || ||||||||||||||||||| ||||| |||||||||||||| ||||||
Sbjct  644  ATACCTCGTTCTTTGATCCTTCAGGAGGAGGGGATCCAATTTTATATCAACATTTATTT  702
```

>Rhipicephalus turanicus voucher CROBB808 cytochrome oxidase subunit 1 (COI) gene, partial cds; mitochondrial
Sequence ID: MZ305547.1 Length: 658
Range 1: 1 to 658

Score:861 bits(466), Expect:0.0,
Identities:594/658(90%), Gaps:0/658(0%), Strand: Plus/Plus

```
Query  1    AACAATATATTTAATTTTTGGAGCATGATCAGGTATATTAGGACTTAGTATAAGAATATT  60
            |||||||||||||||||||||||||||||| || ||||||||| | || |||||||||||
Sbjct  1    AACAATATATTTAATTTTTGGAGCATGATCCGGGATATTAGGATTAAGAATAAGAATATT  60

Query  61   AATTCGTATAGAATTAGGACAACCTGGAACTTTAATTGGAAATGATCAGATTTATAATGT  120
            |||||| ||||| |||||||||||||| |||||||||||||||||||| |||||||||||
Sbjct  61   AATTCGAATAGAGTTAGGACAACCTGGGACTTTAATTGGAAATGATCAAATTTATAATGT  120

Query  121  AATCGTAACAGCACATGCATTTATTATAATTTCTTTATAGTTATACCAATCATAATCGG  180
            ||| |||||||| ||||||||||||||||||| |||||||| |||||||||||||| ||
Sbjct  121  AATTGTAACAGCTCATGCATTTATTATAATTTTTTTATAGTAATACCAATCATAATTGG  180

Query  181  AGGGTTCGGAAATTGATTAGTTCCTATTATATTAGGTGCCCCAGATATAGCATTTCCACG  240
```

```
               ||| || ||||| |||||||| || ||||| ||||| || ||||| ||||||||||||||
Sbjct  181   AGGATTTGGAAACTGATTAGTGCCCATTATGTTAGGAGCTCCAGACATAGCATTTCCACG  240

Query  241   AATAAATAATATAAGATTTTGACTTTTACCCCCCTCATTATTTTTATTGATTAACTCTTC  300
             |||||||||||||||||||||| |  | || ||||||||||| ||||| ||||| |||||
Sbjct  241   AATAAATAATATAAGATTTTGATTACTTCCTCCCTCATTATTCTTATTAATTAATTCTTC  300

Query  301   ATTAATTGAATCCGGAGCTGGAACAGGATGAACAGTTTATCCCCCTCTATCTTCAAATTT  360
             | | |||||||| ||||| || ||||||||||| || || || ||| |||||||||||||
Sbjct  301   ACTGATTGAATCTGGAGCAGGGACAGGATGAACTGTCTACCCTCCTTTATCTTCAAATTT  360

Query  361   ATCACATTATGGTCCATCAGTAGATTTAGCTATTTTTTCTCTTCACCTTGCTGGTGCTTC  420
             ||| |||||||| |||||||||||||||||||| || |||||||| || |||||||||||
Sbjct  361   ATCGCATTATGGACCATCAGTAGATTTAGCTATCTTCTCTCTTCATCTCGCTGGTGCTTC  420

Query  421   TTCAATTTTAGGTGCAATTAACTTCATCACAACTATTGTAAATATACGATCAATTGGAAT  480
             ||||||||||||||||||||| ||||| ||||||||||||||||||||||| ||||||||
Sbjct  421   TTCAATTTTAGGTGCAATTAATTTCATTACAACTATTGTAAATATACGATCTATTGGAAT  480

Query  481   AACAATAGAACGAATACCATTATTTGTTTGATCTGTTTTAATTACAGCTATTTTACTACT  540
             |||||||||||||||||||||||| ||||||||||||||||| || ||||||||| | ||
Sbjct  481   AACAATAGAACGAATACCATTATTCGTTTGATCTGTTTTAATCACCGCTATTTTATTGCT  540

Query  541   TCTATCTTTACCTGTATTAGCAGGTGCCATTACAATATTATTAACTGATCGAAACTTTAA  600
             ||||||||||||||| |||||||||||||||||||||||||||||||||||||| || ||
Sbjct  541   TCTATCTTTACCTGTTTTAGCAGGTGCCATTACAATATTATTAACTGATCGAAATTTCAA  600

Query  601   CACATCATTTTTTGATCCTTCAGGAGGAGGAGATCCCATTTTATATCAACACTTATTT  658
             ||| ||||||||||| |||||||||||||| || || |||||||||||||| ||||||
Sbjct  601   CACTTCATTTTTTGACCCTTCAGGAGGAGGTGACCCTATTTTATATCAACATTTATTT  658
```

>Rhipicephalus turanicus isolate Xinjiang-WQIA cytochrome oxidase subunit 1 (COI) gene, partial cds; mitochondrial
Sequence ID: MF002581.1 Length: 889
Range 1: 74 to 731

Score:850 bits(460), Expect:0.0,
Identities:592/658(90%), Gaps:0/658(0%), Strand: Plus/Plus

```
Query  1     AACAATATATTTAATTTTTGGAGCATGATCAGGTATATTAGGACTTAGTATAAGAATATT  60
             ||||||||||||||||||||| ||||||||||| ||||||||||| || |||||||||||
Sbjct  74    AACAATATATTTAATTTTTGGGGCATGATCAGGAATATTAGGACTAAGAATAAGAATATT  133

Query  61    AATTCGTATAGAATTAGGACAACCTGGAACTTTAATTGGAAATGATCAGATTTATAATGT  120
             |||||| |||||||||||| |||||||||||||||||||||||||||| |||||||||||
Sbjct  134   AATTCGAATAGAATTAGGCCAACCTGGAACTTTAATTGGAAATGATCAAATTTATAATGT  193

Query  121   AATCGTAACAGCACATGCATTTATTATAATTTTCTTTATAGTTATACCAATCATAATCGG  180
             ||| |||||||| |||||||||||||||||||| || ||||| |||||||| ||||| ||
Sbjct  194   AATTGTAACAGCCCATGCATTTATTATAATTTTTTTCATAGTAATACCAATTATAATTGG  253
```

```
Query  181  AGGGTTCGGAAATTGATTAGTTCCTATTATATTAGGTGCCCCAGATATAGCATTTCCACG  240
             || || ||||| |||||||| |||||||||||||| || ||||||||||||||||||||
Sbjct  254  TGGATTTGGAAACTGATTAGTACCTATTATATTAGGAGCTCCAGATATAGCATTTCCACG  313

Query  241  AATAAATAATATAAGATTTTGACTTTTACCCCCCTCATTATTTTTATTGATTAACTCTTC  300
            ||||||||| |||||||||||| |  | ||||| ||||||||| |||| ||||| || ||
Sbjct  314  AATAAATAACATAAGATTTTGATTACTTCCCCCTTCATTATTTCTATTAATTAATTCCTC  373

Query  301  ATTAATTGAATCCGGAGCTGGAACAGGATGAACAGTTTATCCCCCTCTATCTTCAAATTT  360
            |||||||||||| ||||| ||||| |||||||| |||||||| ||| |||| ||||||||
Sbjct  374  ATTAATTGAATCAGGAGCAGGAACTGGATGAACTGTTTATCCGCCTTTATCATCAAATTT  433

Query  361  ATCACATTATGGTCCATCAGTAGATTTAGCTATTTTTTCTCTTCACCTTGCTGGTGCTTC  420
            |||||| ||||| ||||||||||||||||||||||| |||||||| ||||||||||||||
Sbjct  434  ATCACACTATGGACCATCAGTAGATTTAGCTATTTTCTCTCTTCATCTTGCTGGTGCTTC  493

Query  421  TTCAATTTTAGGTGCAATTAACTTCATCACAACTATTGTAAATATACGATCAATTGGAAT  480
            |||||||||||||||||| || || || || |||||| ||||||||||||| ||||||||
Sbjct  494  TTCAATTTTAGGTGCAATCAATTTTATTACGACTATTATAAATATACGATCTATTGGAAT  553

Query  481  AACAATAGAACGAATACCATTATTTGTTTGATCTGTTTTAATTACAGCTATTTTACTACT  540
            ||||||||||||||| ||| ||||||||||||||||||||||||| ||||||||  ||||
Sbjct  554  AACAATAGAACGAATGCCACTATTTGTTTGATCTGTTTTAATTACTGCTATTTTGTTACT  613

Query  541  TCTATCTTTACCTGTATTAGCAGGTGCCATTACAATATTATTAACTGATCGAAACTTTAA  600
            | ||||||| ||||| |||||||| ||||||||||||||||||||||||||||| || ||
Sbjct  614  TTTATCTTTGCCTGTTTTAGCAGGAGCCATTACAATATTATTAACTGATCGAAATTTCAA  673

Query  601  CACATCATTTTTTGATCCTTCAGGAGGAGGAGATCCCATTTTATATCAACACTTATTT  658
            ||| ||||||||||||||||||||||| || ||||| |||||||| ||||| ||||||
Sbjct  674  CACTTCATTTTTTGATCCTTCAGGAGGGGGTGATCCAATTTTATACCAACATTTATTT  731
```

>Rhipicephalus turanicus isolate Xinjiang-WS cytochrome oxidase subunit 1 (COI) gene, partial cds; mitochondrial
Sequence ID: MF002580.1 Length: 889
Range 1: 74 to 731

Score:850 bits(460), Expect:0.0,
Identities:592/658(90%), Gaps:0/658(0%), Strand: Plus/Plus

```
Query  1    AACAATATATTTAATTTTTGGAGCATGATCAGGTATATTAGGACTTAGTATAAGAATATT  60
            ||||||||||||||||||||| ||||||||||| ||||||||||| || |||||||||||
Sbjct  74   AACAATATATTTAATTTTTGGGGCATGATCAGGAATATTAGGACTAAGAATAAGAATATT  133

Query  61   AATTCGTATAGAATTAGGACAACCTGGAACTTTAATTGGAAATGATCAGATTTATAATGT  120
            |||||| ||||||||||| ||||||||||||||||||||||||||||| |||||||||||
Sbjct  134  AATTCGAATAGAATTAGGCCAACCTGGAACTTTAATTGGAAATGATCAAATTTATAATGT  193

Query  121  AATCGTAACAGCACATGCATTTATTATAATTTTCTTTATAGTTATACCAATCATAATCGG  180
            ||| |||||||| |||||||||||||||||||| || ||||| |||||||| ||||| ||
Sbjct  194  AATTGTAACAGCCCATGCATTTATTATAATTTTTTTCATAGTAATACCAATTATAATTGG  253
```

```
Query  181  AGGGTTCGGAAATTGATTAGTTCCTATTATATTAGGTGCCCCAGATATAGCATTTCCACG  240
             || || ||||| |||||||| |||||||||||||| || ||||||||||||||||||||
Sbjct  254  TGGATTTGGAAACTGATTAGTACCTATTATATTAGGAGCTCCAGATATAGCATTTCCACG  313

Query  241  AATAAATAATATAAGATTTTGACTTTTACCCCCCTCATTATTTTTATTGATTAACTCTTC  300
            ||||||||| |||||||||||| |  | ||||| ||||||||| |||| ||||| || ||
Sbjct  314  AATAAATAACATAAGATTTTGATTACTTCCCCCTTCATTATTTCTATTAATTAATTCCTC  373

Query  301  ATTAATTGAATCCGGAGCTGGAACAGGATGAACAGTTTATCCCCCTCTATCTTCAAATTT  360
            |||||||||||| ||||| ||||| |||||||| |||||||| ||| |||| ||||||||
Sbjct  374  ATTAATTGAATCAGGAGCAGGAACTGGATGAACTGTTTATCCGCCTTTATCATCAAATTT  433

Query  361  ATCACATTATGGTCCATCAGTAGATTTAGCTATTTTTTCTCTTCACCTTGCTGGTGCTTC  420
            |||||| ||||| ||||||||||||||||||||||| |||||||| ||||||||||||||
Sbjct  434  ATCACACTATGGACCATCAGTAGATTTAGCTATTTTCTCTCTTCATCTTGCTGGTGCTTC  493

Query  421  TTCAATTTTAGGTGCAATTAACTTCATCACAACTATTGTAAATATACGATCAATTGGAAT  480
            |||||||||||||||||| || || || || |||||| ||||||||||||| ||||||||
Sbjct  494  TTCAATTTTAGGTGCAATCAATTTTATTACGACTATTATAAATATACGATCTATTGGAAT  553

Query  481  AACAATAGAACGAATACCATTATTTGTTTGATCTGTTTTAATTACAGCTATTTTACTACT  540
            ||||||||||||||| ||| ||||||||||||||||||||||||| ||||||||  ||||
Sbjct  554  AACAATAGAACGAATGCCACTATTTGTTTGATCTGTTTTAATTACTGCTATTTTGTTACT  613

Query  541  TCTATCTTTACCTGTATTAGCAGGTGCCATTACAATATTATTAACTGATCGAAACTTTAA  600
            | ||||||| ||||| |||||||| ||||||||||||||||||||||||||||| || ||
Sbjct  614  TTTATCTTTGCCTGTTTTAGCAGGAGCCATTACAATATTATTAACTGATCGAAATTTCAA  673

Query  601  CACATCATTTTTTGATCCTTCAGGAGGAGGAGATCCCATTTTATATCAACACTTATTT  658
            ||| ||||||||||||||||||||||| || ||||| |||||||| ||||| ||||||
Sbjct  674  CACTTCATTTTTTGATCCTTCAGGAGGGGGTGATCCAATTTTATACCAACATTTATTT  731
```

>Rhipicephalus turanicus isolate Xinjiang-BC cytochrome oxidase subunit 1 (COI) gene, partial cds; mitochondrial
Sequence ID: MF002579.1 Length: 889
Range 1: 74 to 731

Score:850 bits(460), Expect:0.0,
Identities:592/658(90%), Gaps:0/658(0%), Strand: Plus/Plus

```
Query  1    AACAATATATTTAATTTTTGGAGCATGATCAGGTATATTAGGACTTAGTATAAGAATATT  60
            ||||||||||||||||||||| ||||||||||| ||||||||||| || |||||||||||
Sbjct  74   AACAATATATTTAATTTTTGGGGCATGATCAGGAATATTAGGACTAAGAATAAGAATATT  133

Query  61   AATTCGTATAGAATTAGGACAACCTGGAACTTTAATTGGAAATGATCAGATTTATAATGT  120
            |||||| ||||||||||| ||||||||||||||||||||||||||||| |||||||||||
Sbjct  134  AATTCGAATAGAATTAGGCCAACCTGGAACTTTAATTGGAAATGATCAAATTTATAATGT  193

Query  121  AATCGTAACAGCACATGCATTTATTATAATTTTCTTTATAGTTATACCAATCATAATCGG  180
            ||| |||||||| ||||||||||||||||||| || ||||| |||||||| ||||| ||
```

```
Sbjct  194  AATTGTAACAGCCCATGCATTTATTATAATTTTTTTCATAGTAATACCAATTATAATTGG  253

Query  181  AGGGTTCGGAAATTGATTAGTTCCTATTATATTAGGTGCCCCAGATATAGCATTTCCACG  240
             || || ||||| |||||||| |||||||||||||| || ||||||||||||||||||||
Sbjct  254  TGGATTTGGAAACTGATTAGTACCTATTATATTAGGAGCTCCAGATATAGCATTTCCACG  313

Query  241  AATAAATAATATAAGATTTTGACTTTTACCCCCCTCATTATTTTTATTGATTAACTCTTC  300
            ||||||||| |||||||||||| |  | ||||| ||||||||| |||| ||||| || ||
Sbjct  314  AATAAATAACATAAGATTTTGATTACTTCCCCCTTCATTATTTCTATTAATTAATTCCTC  373

Query  301  ATTAATTGAATCCGGAGCTGGAACAGGATGAACAGTTTATCCCCCTCTATCTTCAAATTT  360
            |||||||||||| ||||| ||||| |||||||| |||||||| ||| |||| ||||||||
Sbjct  374  ATTAATTGAATCAGGAGCAGGAACTGGATGAACTGTTTATCCGCCTTTATCATCAAATTT  433

Query  361  ATCACATTATGGTCCATCAGTAGATTTAGCTATTTTTTCTCTTCACCTTGCTGGTGCTTC  420
            |||||| ||||| ||||||||||||||||||||||| |||||||| ||||||||||||||
Sbjct  434  ATCACACTATGGACCATCAGTAGATTTAGCTATTTTCTCTCTTCATCTTGCTGGTGCTTC  493

Query  421  TTCAATTTTAGGTGCAATTAACTTCATCACAACTATTGTAAATATACGATCAATTGGAAT  480
            |||||||||||||||||| || || || || |||||| ||||||||||||| ||||||||
Sbjct  494  TTCAATTTTAGGTGCAATCAATTTTATTACGACTATTATAAATATACGATCTATTGGAAT  553

Query  481  AACAATAGAACGAATACCATTATTTGTTTGATCTGTTTTAATTACAGCTATTTTACTACT  540
            ||||||||||||||| ||| ||||||||||||||||||||||||| ||||||||  ||||
Sbjct  554  AACAATAGAACGAATGCCACTATTTGTTTGATCTGTTTTAATTACTGCTATTTTGTTACT  613

Query  541  TCTATCTTTACCTGTATTAGCAGGTGCCATTACAATATTATTAACTGATCGAAACTTTAA  600
            | ||||||| ||||| |||||||| ||||||||||||||||||||||||||||| || ||
Sbjct  614  TTTATCTTTGCCTGTTTTAGCAGGAGCCATTACAATATTATTAACTGATCGAAATTTCAA  673

Query  601  CACATCATTTTTTGATCCTTCAGGAGGAGGAGATCCCATTTTATATCAACACTTATTT  658
            ||| ||||||||||||||||||||||| || ||||| |||||||| ||||| ||||||
Sbjct  674  CACTTCATTTTTTGATCCTTCAGGAGGGGGTGATCCAATTTTATACCAACATTTATTT  731
```

>Rhipicephalus turanicus isolate Xinjiang-YC cytochrome oxidase subunit 1 (COI) gene, partial cds; mitochondrial
Sequence ID: MF002578.1 Length: 889
Range 1: 74 to 731

Score:850 bits(460), Expect:0.0,
Identities:592/658(90%), Gaps:0/658(0%), Strand: Plus/Plus

```
Query  1    AACAATATATTTAATTTTTGGAGCATGATCAGGTATATTAGGACTTAGTATAAGAATATT  60
            ||||||||||||||||||||| ||||||||||| ||||||||||| || |||||||||||
Sbjct  74   AACAATATATTTAATTTTTGGGGCATGATCAGGAATATTAGGACTAAGAATAAGAATATT  133

Query  61   AATTCGTATAGAATTAGGACAACCTGGAACTTTAATTGGAAATGATCAGATTTATAATGT  120
            |||||| ||||||||||| ||||||||||||||||||||||||||||| |||||||||||
Sbjct  134  AATTCGAATAGAATTAGGCCAACCTGGAACTTTAATTGGAAATGATCAAATTTATAATGT  193

Query  121  AATCGTAACAGCACATGCATTTATTATAATTTTCTTTATAGTTATACCAATCATAATCGG  180
```

```
            ||| |||||||| |||||||||||||||||||| || ||||| |||||||| ||||| ||
Sbjct  194  AATTGTAACAGCCCATGCATTTATTATAATTTTTTTCATAGTAATACCAATTATAATTGG  253

Query  181  AGGGTTCGGAAATTGATTAGTTCCTATTATATTAGGTGCCCCAGATATAGCATTTCCACG  240
             || || ||||| |||||||| |||||||||||||| || ||||||||||||||||||||
Sbjct  254  TGGATTTGGAAACTGATTAGTACCTATTATATTAGGAGCTCCAGATATAGCATTTCCACG  313

Query  241  AATAAATAATATAAGATTTTGACTTTTACCCCCCTCATTATTTTTATTGATTAACTCTTC  300
            ||||||||| |||||||||||| |  | ||||| ||||||||| |||| ||||| || ||
Sbjct  314  AATAAATAACATAAGATTTTGATTACTTCCCCCTTCATTATTTCTATTAATTAATTCCTC  373

Query  301  ATTAATTGAATCCGGAGCTGGAACAGGATGAACAGTTTATCCCCCTCTATCTTCAAATTT  360
            |||||||||||| ||||| ||||| |||||||| |||||||| ||| |||| ||||||||
Sbjct  374  ATTAATTGAATCAGGAGCAGGAACTGGATGAACTGTTTATCCGCCTTTATCATCAAATTT  433

Query  361  ATCACATTATGGTCCATCAGTAGATTTAGCTATTTTTTCTCTTCACCTTGCTGGTGCTTC  420
            |||||| ||||| ||||||||||||||||||||||| |||||||| ||||||||||||||
Sbjct  434  ATCACACTATGGACCATCAGTAGATTTAGCTATTTTCTCTCTTCATCTTGCTGGTGCTTC  493

Query  421  TTCAATTTTAGGTGCAATTAACTTCATCACAACTATTGTAAATATACGATCAATTGGAAT  480
            |||||||||||||||||| || || || || |||||| ||||||||||||| ||||||||
Sbjct  494  TTCAATTTTAGGTGCAATCAATTTTATTACGACTATTATAAATATACGATCTATTGGAAT  553

Query  481  AACAATAGAACGAATACCATTATTTGTTTGATCTGTTTTAATTACAGCTATTTTACTACT  540
            ||||||||||||||| ||| ||||||||||||||||||||||||| ||||||||  ||||
Sbjct  554  AACAATAGAACGAATGCCACTATTTGTTTGATCTGTTTTAATTACTGCTATTTTGTTACT  613

Query  541  TCTATCTTTACCTGTATTAGCAGGTGCCATTACAATATTATTAACTGATCGAAACTTTAA  600
            | ||||||| ||||| |||||||| ||||||||||||||||||||||||||||| || ||
Sbjct  614  TTTATCTTTGCCTGTTTTAGCAGGAGCCATTACAATATTATTAACTGATCGAAATTTCAA  673

Query  601  CACATCATTTTTTGATCCTTCAGGAGGAGGAGATCCCATTTTATATCAACACTTATTT  658
            ||| ||||||||||||||||||||||| || ||||| |||||||| ||||| ||||||
Sbjct  674  CACTTCATTTTTTGATCCTTCAGGAGGGGGTGATCCAATTTTATACCAACATTTATTT  731
```

>Rhipicephalus turanicus isolate Xinjiang-PS cytochrome oxidase subunit 1 (COI) gene, partial cds; mitochondrial
Sequence ID: MF002577.1 Length: 889
Range 1: 74 to 731

Score:850 bits(460), Expect:0.0,
Identities:592/658(90%), Gaps:0/658(0%), Strand: Plus/Plus

```
Query  1    AACAATATATTTAATTTTTGGAGCATGATCAGGTATATTAGGACTTAGTATAAGAATATT  60
            ||||||||||||||||||||| ||||||||||| ||||||||||| || |||||||||||
Sbjct  74   AACAATATATTTAATTTTTGGGGCATGATCAGGAATATTAGGACTAAGAATAAGAATATT  133

Query  61   AATTCGTATAGAATTAGGACAACCTGGAACTTTAATTGGAAATGATCAGATTTATAATGT  120
            |||||| |||||||||||| |||||||||||||||||||||||||||| |||||||||||
Sbjct  134  AATTCGAATAGAATTAGGCCAACCTGGAACTTTAATTGGAAATGATCAAATTTATAATGT  193
```

```
Query  121  AATCGTAACAGCACATGCATTTATTATAATTTTCTTTATAGTTATACCAATCATAATCGG  180
            ||| |||||||| |||||||||||||||||||| || ||||| |||||||| ||||| ||
Sbjct  194  AATTGTAACAGCCCATGCATTTATTATAATTTTTTTCATAGTAATACCAATTATAATTGG  253

Query  181  AGGGTTCGGAAATTGATTAGTTCCTATTATATTAGGTGCCCCAGATATAGCATTTCCACG  240
             || || ||||| |||||||| |||||||||||||| || ||||||||||||||||||||
Sbjct  254  TGGATTTGGAAACTGATTAGTACCTATTATATTAGGAGCTCCAGATATAGCATTTCCACG  313

Query  241  AATAAATAATATAAGATTTTGACTTTTACCCCCCTCATTATTTTTATTGATTAACTCTTC  300
            ||||||||| |||||||||||| |  | ||||| ||||||||| |||| ||||| || ||
Sbjct  314  AATAAATAACATAAGATTTTGATTACTTCCCCCTTCATTATTTCTATTAATTAATTCCTC  373

Query  301  ATTAATTGAATCCGGAGCTGGAACAGGATGAACAGTTTATCCCCCTCTATCTTCAAATTT  360
            |||||||||||| ||||| ||||| |||||||| |||||||| ||| |||| ||||||||
Sbjct  374  ATTAATTGAATCAGGAGCAGGAACTGGATGAACTGTTTATCCGCCTTTATCATCAAATTT  433

Query  361  ATCACATTATGGTCCATCAGTAGATTTAGCTATTTTTTCTCTTCACCTTGCTGGTGCTTC  420
            |||||| ||||| ||||||||||||||||||||||| |||||||| ||||||||||||||
Sbjct  434  ATCACACTATGGACCATCAGTAGATTTAGCTATTTTCTCTCTTCATCTTGCTGGTGCTTC  493

Query  421  TTCAATTTTAGGTGCAATTAACTTCATCACAACTATTGTAAATATACGATCAATTGGAAT  480
            |||||||||||||||||| || || || || |||||| ||||||||||||| ||||||||
Sbjct  494  TTCAATTTTAGGTGCAATCAATTTTATTACGACTATTATAAATATACGATCTATTGGAAT  553

Query  481  AACAATAGAACGAATACCATTATTTGTTTGATCTGTTTTAATTACAGCTATTTTACTACT  540
            ||||||||||||||| ||| ||||||||||||||||||||||||| ||||||||  ||||
Sbjct  554  AACAATAGAACGAATGCCACTATTTGTTTGATCTGTTTTAATTACTGCTATTTTGTTACT  613

Query  541  TCTATCTTTACCTGTATTAGCAGGTGCCATTACAATATTATTAACTGATCGAAACTTTAA  600
            | ||||||| ||||| |||||||| ||||||||||||||||||||||||||||| || ||
Sbjct  614  TTTATCTTTGCCTGTTTTAGCAGGAGCCATTACAATATTATTAACTGATCGAAATTTCAA  673

Query  601  CACATCATTTTTTGATCCTTCAGGAGGAGGAGATCCCATTTTATATCAACACTTATTT  658
            ||| ||||||||||||||||||||||| || ||||| |||||||| ||||| ||||||
Sbjct  674  CACTTCATTTTTTGATCCTTCAGGAGGGGGTGATCCAATTTTATACCAACATTTATTT  731
```

>Rhipicephalus turanicus isolate SHZ cytochrome oxidase subunit I (CO1) gene, partial cds; mitochondrial
Sequence ID: KY069271.1 Length: 889
Range 1: 74 to 731

Score:850 bits(460), Expect:0.0,
Identities:592/658(90%), Gaps:0/658(0%), Strand: Plus/Plus

```
Query  1    AACAATATATTTAATTTTTGGAGCATGATCAGGTATATTAGGACTTAGTATAAGAATATT  60
            ||||||||||||||||||||| ||||||||||| ||||||||||| || |||||||||||
Sbjct  74   AACAATATATTTAATTTTTGGGGCATGATCAGGAATATTAGGACTAAGAATAAGAATATT  133

Query  61   AATTCGTATAGAATTAGGACAACCTGGAACTTTAATTGGAAATGATCAGATTTATAATGT  120
            |||||| ||||||||||| ||||||||||||||||||||||||||||| |||||||||||
Sbjct  134  AATTCGAATAGAATTAGGCCAACCTGGAACTTTAATTGGAAATGATCAAATTTATAATGT  193
```

```
Query  121  AATCGTAACAGCACATGCATTTATTATAATTTTCTTTATAGTTATACCAATCATAATCGG  180
            ||| |||||||| |||||||||||||||||||| || ||||| |||||||| ||||| ||
Sbjct  194  AATTGTAACAGCCCATGCATTTATTATAATTTTTTTCATAGTAATACCAATTATAATTGG  253

Query  181  AGGGTTCGGAAATTGATTAGTTCCTATTATATTAGGTGCCCCAGATATAGCATTTCCACG  240
             || || ||||| |||||||| |||||||||||||| || ||||||||||||||||||||
Sbjct  254  TGGATTTGGAAACTGATTAGTACCTATTATATTAGGAGCTCCAGATATAGCATTTCCACG  313

Query  241  AATAAATAATATAAGATTTTGACTTTTACCCCCCTCATTATTTTTATTGATTAACTCTTC  300
            ||||||||| |||||||||||| |  | ||||| ||||||||| |||| ||||| || ||
Sbjct  314  AATAAATAACATAAGATTTTGATTACTTCCCCCTTCATTATTTCTATTAATTAATTCCTC  373

Query  301  ATTAATTGAATCCGGAGCTGGAACAGGATGAACAGTTTATCCCCCTCTATCTTCAAATTT  360
            |||||||||||| ||||| ||||| |||||||| |||||||| ||| |||| ||||||||
Sbjct  374  ATTAATTGAATCAGGAGCAGGAACTGGATGAACTGTTTATCCGCCTTTATCATCAAATTT  433

Query  361  ATCACATTATGGTCCATCAGTAGATTTAGCTATTTTTTCTCTTCACCTTGCTGGTGCTTC  420
            |||||| ||||| ||||||||||||||||||||||| |||||||| ||||||||||||||
Sbjct  434  ATCACACTATGGACCATCAGTAGATTTAGCTATTTTCTCTCTTCATCTTGCTGGTGCTTC  493

Query  421  TTCAATTTTAGGTGCAATTAACTTCATCACAACTATTGTAAATATACGATCAATTGGAAT  480
            |||||||||||||||||| || || || || |||||| ||||||||||||| ||||||||
Sbjct  494  TTCAATTTTAGGTGCAATCAATTTTATTACGACTATTATAAATATACGATCTATTGGAAT  553

Query  481  AACAATAGAACGAATACCATTATTTGTTTGATCTGTTTTAATTACAGCTATTTTACTACT  540
            ||||||||||||||| ||| ||||||||||||||||||||||||| ||||||||  ||||
Sbjct  554  AACAATAGAACGAATGCCACTATTTGTTTGATCTGTTTTAATTACTGCTATTTTGTTACT  613

Query  541  TCTATCTTTACCTGTATTAGCAGGTGCCATTACAATATTATTAACTGATCGAAACTTTAA  600
            | ||||||| ||||| |||||||| ||||||||||||||||||||||||||||| || ||
Sbjct  614  TTTATCTTTGCCTGTTTTAGCAGGAGCCATTACAATATTATTAACTGATCGAAATTTCAA  673

Query  601  CACATCATTTTTTGATCCTTCAGGAGGAGGAGATCCCATTTTATATCAACACTTATTT  658
            ||| ||||||||||||||||||||||| || ||||| |||||||| ||||| ||||||
Sbjct  674  CACTTCATTTTTTGATCCTTCAGGAGGGGGTGATCCAATTTTATACCAACATTTATTT  731
```

>Rhipicephalus turanicus isolate FK-1 cytochrome oxidase subunit 1 (COI) gene, partial cds; mitochondrial
Sequence ID: KY606291.1 Length: 889
Range 1: 74 to 731

Score:850 bits(460), Expect:0.0,
Identities:592/658(90%), Gaps:0/658(0%), Strand: Plus/Plus

```
Query  1    AACAATATATTTAATTTTTGGAGCATGATCAGGTATATTAGGACTTAGTATAAGAATATT  60
            ||||||||||||||||||||| ||||||||||| ||||||||||| || |||||||||||
Sbjct  74   AACAATATATTTAATTTTTGGGGCATGATCAGGAATATTAGGACTAAGAATAAGAATATT  133

Query  61   AATTCGTATAGAATTAGGACAACCTGGAACTTTAATTGGAAATGATCAGATTTATAATGT  120
            |||||| ||||||||||| ||||||||||||||||||||||||||||| |||||||||||
```

```
Sbjct  134  AATTCGAATAGAATTAGGCCAACCTGGAACTTTAATTGGAAATGATCAAATTTATAATGT  193

Query  121  AATCGTAACAGCACATGCATTTATTATAATTTTCTTTATAGTTATACCAATCATAATCGG  180
            ||| |||||||| |||||||||||||||||||| || ||||| |||||||| ||||| ||
Sbjct  194  AATTGTAACAGCCCATGCATTTATTATAATTTTTTTCATAGTAATACCAATTATAATTGG  253

Query  181  AGGGTTCGGAAATTGATTAGTTCCTATTATATTAGGTGCCCCAGATATAGCATTTCCACG  240
             || || ||||| |||||||| |||||||||||||| || ||||||||||||||||||||
Sbjct  254  TGGATTTGGAAACTGATTAGTACCTATTATATTAGGAGCTCCAGATATAGCATTTCCACG  313

Query  241  AATAAATAATATAAGATTTTGACTTTTACCCCCCTCATTATTTTTATTGATTAACTCTTC  300
            ||||||||| |||||||||||| |  | ||||| ||||||||| |||| ||||| || ||
Sbjct  314  AATAAATAACATAAGATTTTGATTACTTCCCCCTTCATTATTTCTATTAATTAATTCCTC  373

Query  301  ATTAATTGAATCCGGAGCTGGAACAGGATGAACAGTTTATCCCCCTCTATCTTCAAATTT  360
            |||||||||||| ||||| ||||| |||||||| |||||||| ||| |||| ||||||||
Sbjct  374  ATTAATTGAATCAGGAGCAGGAACTGGATGAACTGTTTATCCGCCTTTATCATCAAATTT  433

Query  361  ATCACATTATGGTCCATCAGTAGATTTAGCTATTTTTTCTCTTCACCTTGCTGGTGCTTC  420
            |||||| ||||| ||||||||||||||||||||||| |||||||| ||||||||||||||
Sbjct  434  ATCACACTATGGACCATCAGTAGATTTAGCTATTTTCTCTCTTCATCTTGCTGGTGCTTC  493

Query  421  TTCAATTTTAGGTGCAATTAACTTCATCACAACTATTGTAAATATACGATCAATTGGAAT  480
            |||||||||||||||||| || || || || |||||| ||||||||||||| ||||||||
Sbjct  494  TTCAATTTTAGGTGCAATCAATTTTATTACGACTATTATAAATATACGATCTATTGGAAT  553

Query  481  AACAATAGAACGAATACCATTATTTGTTTGATCTGTTTTAATTACAGCTATTTTACTACT  540
            ||||||||||||||| ||| ||||||||||||||||||||||||| ||||||||  ||||
Sbjct  554  AACAATAGAACGAATGCCACTATTTGTTTGATCTGTTTTAATTACTGCTATTTTGTTACT  613

Query  541  TCTATCTTTACCTGTATTAGCAGGTGCCATTACAATATTATTAACTGATCGAAACTTTAA  600
            | ||||||| ||||| |||||||| ||||||||||||||||||||||||||||| || ||
Sbjct  614  TTTATCTTTGCCTGTTTTAGCAGGAGCCATTACAATATTATTAACTGATCGAAATTTCAA  673

Query  601  CACATCATTTTTTGATCCTTCAGGAGGAGGAGATCCCATTTTATATCAACACTTATTT  658
            ||| ||||||||||||||||||||||| || ||||| |||||||| ||||| ||||||
Sbjct  674  CACTTCATTTTTTGATCCTTCAGGAGGGGGTGATCCAATTTTATACCAACATTTATTT  731
```

>Rhipicephalus turanicus isolate YN3 cytochrome oxidase subunit 1 (COI) gene, partial cds; mitochondrial
Sequence ID: KY606290.1 Length: 889
Range 1: 74 to 731

Score:850 bits(460), Expect:0.0,
Identities:592/658(90%), Gaps:0/658(0%), Strand: Plus/Plus

```
Query  1    AACAATATATTTAATTTTTGGAGCATGATCAGGTATATTAGGACTTAGTATAAGAATATT  60
            ||||||||||||||||||||| ||||||||||| ||||||||||| || |||||||||||
Sbjct  74   AACAATATATTTAATTTTTGGGGCATGATCAGGAATATTAGGACTAAGAATAAGAATATT  133

Query  61   AATTCGTATAGAATTAGGACAACCTGGAACTTTAATTGGAAATGATCAGATTTATAATGT  120
```

```
            |||||| ||||||||||| ||||||||||||||||||||||||||||| |||||||||||
Sbjct  134  AATTCGAATAGAATTAGGCCAACCTGGAACTTTAATTGGAAATGATCAAATTTATAATGT  193

Query  121  AATCGTAACAGCACATGCATTTATTATAATTTTCTTTATAGTTATACCAATCATAATCGG  180
            ||| |||||||| |||||||||||||||||||| || ||||| |||||||| ||||| ||
Sbjct  194  AATTGTAACAGCCCATGCATTTATTATAATTTTTTTCATAGTAATACCAATTATAATTGG  253

Query  181  AGGGTTCGGAAATTGATTAGTTCCTATTATATTAGGTGCCCCAGATATAGCATTTCCACG  240
             || || ||||| |||||||| |||||||||||||| || ||||||||||||||||||||
Sbjct  254  TGGATTTGGAAACTGATTAGTACCTATTATATTAGGAGCTCCAGATATAGCATTTCCACG  313

Query  241  AATAAATAATATAAGATTTTGACTTTTACCCCCCTCATTATTTTTATTGATTAACTCTTC  300
            ||||||||| |||||||||||| |  | ||||| ||||||||| |||| ||||| || ||
Sbjct  314  AATAAATAACATAAGATTTTGATTACTTCCCCCTTCATTATTTCTATTAATTAATTCCTC  373

Query  301  ATTAATTGAATCCGGAGCTGGAACAGGATGAACAGTTTATCCCCCTCTATCTTCAAATTT  360
            |||||||||||| ||||| ||||| |||||||| |||||||| ||| |||| ||||||||
Sbjct  374  ATTAATTGAATCAGGAGCAGGAACTGGATGAACTGTTTATCCGCCTTTATCATCAAATTT  433

Query  361  ATCACATTATGGTCCATCAGTAGATTTAGCTATTTTTTCTCTTCACCTTGCTGGTGCTTC  420
            |||||| ||||| ||||||||||||||||||||||| |||||||| ||||||||||||||
Sbjct  434  ATCACACTATGGACCATCAGTAGATTTAGCTATTTTCTCTCTTCATCTTGCTGGTGCTTC  493

Query  421  TTCAATTTTAGGTGCAATTAACTTCATCACAACTATTGTAAATATACGATCAATTGGAAT  480
            |||||||||||||||||| || || || || |||||| ||||||||||||| ||||||||
Sbjct  494  TTCAATTTTAGGTGCAATCAATTTTATTACGACTATTATAAATATACGATCTATTGGAAT  553

Query  481  AACAATAGAACGAATACCATTATTTGTTTGATCTGTTTTAATTACAGCTATTTTACTACT  540
            ||||||||||||||| ||| ||||||||||||||||||||||||| ||||||||  ||||
Sbjct  554  AACAATAGAACGAATGCCACTATTTGTTTGATCTGTTTTAATTACTGCTATTTTGTTACT  613

Query  541  TCTATCTTTACCTGTATTAGCAGGTGCCATTACAATATTATTAACTGATCGAAACTTTAA  600
            | ||||||| ||||| |||||||| ||||||||||||||||||||||||||||| || ||
Sbjct  614  TTTATCTTTGCCTGTTTTAGCAGGAGCCATTACAATATTATTAACTGATCGAAATTTCAA  673

Query  601  CACATCATTTTTTGATCCTTCAGGAGGAGGAGATCCCATTTTATATCAACACTTATTT  658
            ||| ||||||||||||||||||||||| || ||||| |||||||| ||||| ||||||
Sbjct  674  CACTTCATTTTTTGATCCTTCAGGAGGGGGTGATCCAATTTTATACCAACATTTATTT  731
```

>Rhipicephalus turanicus isolate YN2 cytochrome oxidase subunit 1 (COI) gene, partial cds; mitochondrial
Sequence ID: KY606289.1 Length: 889
Range 1: 74 to 731

Score:850 bits(460), Expect:0.0,
Identities:592/658(90%), Gaps:0/658(0%), Strand: Plus/Plus

```
Query  1    AACAATATATTTAATTTTTGGAGCATGATCAGGTATATTAGGACTTAGTATAAGAATATT  60
            ||||||||||||||||||||| ||||||||||| ||||||||||| || |||||||||||
Sbjct  74   AACAATATATTTAATTTTTGGGGCATGATCAGGAATATTAGGACTAAGAATAAGAATATT  133
```

```
Query  61   AATTCGTATAGAATTAGGACAACCTGGAACTTTAATTGGAAATGATCAGATTTATAATGT  120
            |||||| ||||||||||| ||||||||||||||||||||||||||||| |||||||||||
Sbjct  134  AATTCGAATAGAATTAGGCCAACCTGGAACTTTAATTGGAAATGATCAAATTTATAATGT  193

Query  121  AATCGTAACAGCACATGCATTTATTATAATTTTCTTTATAGTTATACCAATCATAATCGG  180
            ||| |||||||| |||||||||||||||||||| || ||||| |||||||| ||||| ||
Sbjct  194  AATTGTAACAGCCCATGCATTTATTATAATTTTTTTCATAGTAATACCAATTATAATTGG  253

Query  181  AGGGTTCGGAAATTGATTAGTTCCTATTATATTAGGTGCCCCAGATATAGCATTTCCACG  240
             || || ||||| |||||||| |||||||||||||| || ||||||||||||||||||||
Sbjct  254  TGGATTTGGAAACTGATTAGTACCTATTATATTAGGAGCTCCAGATATAGCATTTCCACG  313

Query  241  AATAAATAATATAAGATTTTGACTTTTACCCCCCTCATTATTTTTATTGATTAACTCTTC  300
            ||||||||| |||||||||||| |  | ||||| ||||||||| |||| ||||| || ||
Sbjct  314  AATAAATAACATAAGATTTTGATTACTTCCCCCTTCATTATTTCTATTAATTAATTCCTC  373

Query  301  ATTAATTGAATCCGGAGCTGGAACAGGATGAACAGTTTATCCCCCTCTATCTTCAAATTT  360
            |||||||||||| ||||| ||||| |||||||| |||||||| ||| |||| ||||||||
Sbjct  374  ATTAATTGAATCAGGAGCAGGAACTGGATGAACTGTTTATCCGCCTTTATCATCAAATTT  433

Query  361  ATCACATTATGGTCCATCAGTAGATTTAGCTATTTTTTCTCTTCACCTTGCTGGTGCTTC  420
            |||||| ||||| ||||||||||||||||||||||| |||||||| ||||||||||||||
Sbjct  434  ATCACACTATGGACCATCAGTAGATTTAGCTATTTTCTCTCTTCATCTTGCTGGTGCTTC  493

Query  421  TTCAATTTTAGGTGCAATTAACTTCATCACAACTATTGTAAATATACGATCAATTGGAAT  480
            |||||||||||||||||| || || || || |||||| ||||||||||||| ||||||||
Sbjct  494  TTCAATTTTAGGTGCAATCAATTTTATTACGACTATTATAAATATACGATCTATTGGAAT  553

Query  481  AACAATAGAACGAATACCATTATTTGTTTGATCTGTTTTAATTACAGCTATTTTACTACT  540
            ||||||||||||||| ||| ||||||||||||||||||||||||| ||||||||  ||||
Sbjct  554  AACAATAGAACGAATGCCACTATTTGTTTGATCTGTTTTAATTACTGCTATTTTGTTACT  613

Query  541  TCTATCTTTACCTGTATTAGCAGGTGCCATTACAATATTATTAACTGATCGAAACTTTAA  600
            | ||||||| ||||| |||||||| ||||||||||||||||||||||||||||| || ||
Sbjct  614  TTTATCTTTGCCTGTTTTAGCAGGAGCCATTACAATATTATTAACTGATCGAAATTTCAA  673

Query  601  CACATCATTTTTTGATCCTTCAGGAGGAGGAGATCCCATTTTATATCAACACTTATTT  658
            ||| ||||||||||||||||||||||| || ||||| |||||||| ||||| ||||||
Sbjct  674  CACTTCATTTTTTGATCCTTCAGGAGGGGGTGATCCAATTTTATACCAACATTTATTT  731
```

Query #5: TKCJJ095-19|TKCJ012018H11|Rhipicephalus sanguineus|COI-5P Query ID: lcl|Query_26767 Length: 658

Sequences producing significant alignments:

```
                                                                    Scientific
 Common                    Max    Total Query   E   Per.   Acc.

Description                                                         Name
 Name            Taxid      Score  Score cover Value Ident  Len         Accession
```

Rhipicephalus sanguineus sensu lato 'temperate lineage' isolat...
Rhipicephalu... brown dog tick 34632 1216 1216 100% 0.0 100.00 697
MF426019.1
Rhipicephalus sanguineus haplotype III cytochrome oxidase...
Rhipicephalu... brown dog tick 34632 1216 1216 100% 0.0 100.00 713
KU556745.1
Rhipicephalus sanguineus isolate Arizona-1 cytochrome c oxidas...
Rhipicephalu... brown dog tick 34632 1216 1216 100% 0.0 100.00 710
MN593344.1
Rhipicephalus sanguineus isolate Arizona-2 cytochrome c oxidas...
Rhipicephalu... brown dog tick 34632 1216 1216 100% 0.0 100.00 710
MN593343.1
Rhipicephalus sanguineus isolate 11N cytochrome oxidase subuni...
Rhipicephalu... brown dog tick 34632 1216 1216 100% 0.0 100.00 703
MN585197.1
Rhipicephalus sanguineus cytochrome c oxidase I (COI) gene,...
Rhipicephalu... brown dog tick 34632 1216 1216 100% 0.0 100.00 793
AF132839.1
Rhipicephalus sanguineus sensu lato 'temperate lineage' isolat...
Rhipicephalu... brown dog tick 34632 1210 1210 100% 0.0 99.85 675
MF426018.1
Rhipicephalus sanguineus sensu lato 'temperate lineage' isolat...
Rhipicephalu... brown dog tick 34632 1210 1210 100% 0.0 99.85 680
MF426016.1
Rhipicephalus sanguineus sensu lato 'temperate lineage' isolat...
Rhipicephalu... brown dog tick 34632 1210 1210 100% 0.0 99.85 693
MF426013.1
Rhipicephalus sanguineus sensu lato 'temperate lineage' isolat...
Rhipicephalu... brown dog tick 34632 1210 1210 100% 0.0 99.85 679
MF426009.1
Rhipicephalus sanguineus sensu lato 'temperate lineage' isolat...
Rhipicephalu... brown dog tick 34632 1210 1210 100% 0.0 99.85 690
MF426008.1
Rhipicephalus sanguineus sensu lato 'temperate lineage' isolat...
Rhipicephalu... brown dog tick 34632 1210 1210 100% 0.0 99.85 686
MF426007.1
Rhipicephalus sanguineus sensu lato 'temperate lineage' isolat...
Rhipicephalu... brown dog tick 34632 1210 1210 100% 0.0 99.85 684
MF426001.1
Rhipicephalus sanguineus sensu lato 'temperate lineage' isolat...
Rhipicephalu... brown dog tick 34632 1210 1210 100% 0.0 99.85 695
MF425997.1
Rhipicephalus sanguineus voucher CROBB813 cytochrome oxidase...
Rhipicephalu... brown dog tick 34632 1210 1210 100% 0.0 99.85 658
MZ305546.1
Rhipicephalus sanguineus voucher CROBB810 cytochrome oxidase...
Rhipicephalu... brown dog tick 34632 1210 1210 100% 0.0 99.85 658
MZ305545.1
Rhipicephalus sanguineus isolate 5MSK cytochrome c oxidase...
Rhipicephalu... brown dog tick 34632 1210 1210 100% 0.0 99.85 676
MW152145.1
Rhipicephalus sanguineus isolate 1MSK cytochrome c oxidase...
Rhipicephalu... brown dog tick 34632 1210 1210 100% 0.0 99.85 675
MW152144.1

Rhipicephalus sanguineus isolate RSN2SK cytochrome c oxidase...
Rhipicephalu... brown dog tick 34632   1210   1210 100% 0.0   99.85 676
     MW152143.1
Rhipicephalus sanguineus isolate RSN11SK cytochrome c oxidase...
Rhipicephalu... brown dog tick 34632   1210   1210 100% 0.0   99.85 669
     MW152142.1
Rhipicephalus sanguineus sensu lato 'temperate lineage' isolat...
Rhipicephalu... brown dog tick 34632   1205   1205 100% 0.0   99.70 692
     MF426015.1
Rhipicephalus sanguineus sensu lato 'temperate lineage' isolat...
Rhipicephalu... brown dog tick 34632   1205   1205 100% 0.0   99.70 705
     MF426006.1
Rhipicephalus sanguineus sensu lato 'temperate lineage' isolat...
Rhipicephalu... brown dog tick 34632   1199   1199 100% 0.0   99.54 680
     MF426017.1
Rhipicephalus sanguineus sensu lato 'temperate lineage' isolat...
Rhipicephalu... brown dog tick 34632   1199   1199 100% 0.0   99.54 689
     MF425998.1
Rhipicephalus sanguineus haplotype IV cytochrome oxidase subun...
Rhipicephalu... brown dog tick 34632   1199   1257 100% 0.0   99.54 771
     KU556746.1
Rhipicephalus sanguineus sensu lato 'temperate lineage' isolat...
Rhipicephalu... brown dog tick 34632   1194   1194 100% 0.0   99.39 684
     MF426012.1
Rhipicephalus sanguineus sensu lato 'temperate lineage' isolat...
Rhipicephalu... brown dog tick 34632   1192   1192 98%   0.0   99.85 824
     MF426010.1
Rhipicephalus sanguineus haplotype II cytochrome oxidase subun...
Rhipicephalu... brown dog tick 34632   1190   1190 100% 0.0   99.24 787
     KU556744.1
Rhipicephalus sanguineus haplotype I cytochrome oxidase subuni...
Rhipicephalu... brown dog tick 34632   1186   1186 98%   0.0   99.69 659
     KU556743.1
Rhipicephalus sanguineus sensu lato 'temperate lineage' isolat...
Rhipicephalu... brown dog tick 34632   1182   1182 97%   0.0   99.84 812
     MF426020.1
Rhipicephalus sanguineus isolate LIC5424B cytochrome oxidase...
Rhipicephalu... brown dog tick 34632   1175   1175 97%   0.0   99.69 768
     KX383818.1
Rhipicephalus sanguineus isolate LIC5533B cytochrome oxidase...
Rhipicephalu... brown dog tick 34632   1168   1168 96%   0.0   99.69 764
     KX383811.1
Rhipicephalus sanguineus isolate LIC4736B cytochrome oxidase...
Rhipicephalu... brown dog tick 34632   1166   1166 96%   0.0   99.69 763
     KX383807.1
Rhipicephalus sanguineus sensu lato 'temperate lineage' isolat...
Rhipicephalu... brown dog tick 34632   1164   1164 97%   0.0   99.38 809
     MF426011.1
Rhipicephalus sanguineus isolate LIC4736D cytochrome oxidase...
Rhipicephalu... brown dog tick 34632   1164   1164 96%   0.0   99.69 762
     KX383809.1
Rhipicephalus sanguineus isolate LIC4727D cytochrome oxidase...
Rhipicephalu... brown dog tick 34632   1164   1164 96%   0.0   99.69 714
     KX383806.1

Rhipicephalus sanguineus isolate LIC4727C cytochrome oxidase...
Rhipicephalu... brown dog tick 34632 1162 1162 96% 0.0 99.69 761
KX383805.1
Rhipicephalus sanguineus sensu lato 'temperate lineage' isolat...
Rhipicephalu... brown dog tick 34632 1160 1160 97% 0.0 99.38 654
MF425988.1
Rhipicephalus sanguineus clone #7 cytochrome oxidase subunit I...
Rhipicephalu... brown dog tick 34632 1157 1157 95% 0.0 100.00 654
KX519712.1
Rhipicephalus sanguineus sensu lato 'temperate lineage' isolat...
Rhipicephalu... brown dog tick 34632 1155 1155 95% 0.0 99.84 639
MF426002.1
Rhipicephalus sanguineus isolate Rsan-ML4 cytochrome oxidase...
Rhipicephalu... brown dog tick 34632 1146 1146 94% 0.0 100.00 630
MG855658.1
Rhipicephalus sanguineus isolate Rsan-ML3 cytochrome oxidase...
Rhipicephalu... brown dog tick 34632 1146 1146 94% 0.0 100.00 630
MG855657.1
Rhipicephalus sanguineus isolate Rsan-ML2 cytochrome oxidase...
Rhipicephalu... brown dog tick 34632 1146 1146 94% 0.0 100.00 630
MG855656.1
Rhipicephalus sanguineus isolate Kehf-Lagareb-A cytochrome...
Rhipicephalu... brown dog tick 34632 1146 1146 94% 0.0 100.00 630
KX757910.1
Rhipicephalus sanguineus isolate Italy-B cytochrome oxidase...
Rhipicephalu... brown dog tick 34632 1146 1146 94% 0.0 100.00 630
KX757904.1
Rhipicephalus sanguineus isolate Malta-A cytochrome oxidase...
Rhipicephalu... brown dog tick 34632 1146 1146 94% 0.0 100.00 630
KX757902.1
Rhipicephalus sanguineus isolate Zagreb-C cytochrome oxidase...
Rhipicephalu... brown dog tick 34632 1146 1146 94% 0.0 100.00 630
KX757896.1
Rhipicephalus sanguineus isolate Pula-B cytochrome oxidase...
Rhipicephalu... brown dog tick 34632 1146 1146 94% 0.0 100.00 630
KX757889.1
Rhipicephalus sanguineus isolate Rovind cytochrome oxidase...
Rhipicephalu... brown dog tick 34632 1146 1146 94% 0.0 100.00 630
KX757887.1
Rhipicephalus sanguineus isolate Kajtasovo cytochrome oxidase...
Rhipicephalu... brown dog tick 34632 1146 1146 94% 0.0 100.00 630
KX757879.1
Rhipicephalus sanguineus clone #6 cytochrome oxidase subunit I...
Rhipicephalu... brown dog tick 34632 1146 1146 94% 0.0 100.00 636
KX519711.1
Rhipicephalus sanguineus isolate SV4 cytochrome c oxidase...
Rhipicephalu... brown dog tick 34632 1146 1146 94% 0.0 100.00 630
MW135448.1
Rhipicephalus sanguineus isolate Samont2 cytochrome oxidase...
Rhipicephalu... brown dog tick 34632 1144 1144 94% 0.0 99.84 644
MH630347.1
Rhipicephalus sanguineus isolate Samont1 cytochrome oxidase...
Rhipicephalu... brown dog tick 34632 1144 1144 94% 0.0 99.84 644
MH630346.1

Rhipicephalus sanguineus sensu lato 'temperate lineage' isolat...
Rhipicephalu... brown dog tick 34632 1144 1144 94% 0.0 99.84 635
MF426000.1
Rhipicephalus sanguineus isolate Boka-A cytochrome oxidase...
Rhipicephalu... brown dog tick 34632 1140 1140 94% 0.0 99.84 630
KX757905.1
Rhipicephalus sanguineus isolate Sibenik cytochrome oxidase...
Rhipicephalu... brown dog tick 34632 1140 1140 94% 0.0 99.84 630
KX757895.1
Rhipicephalus sanguineus isolate Zadar cytochrome oxidase...
Rhipicephalu... brown dog tick 34632 1140 1140 94% 0.0 99.84 630
KX757892.1
Rhipicephalus sanguineus isolate Zagreb-A cytochrome oxidase...
Rhipicephalu... brown dog tick 34632 1140 1140 94% 0.0 99.84 630
KX757890.1
Rhipicephalus sanguineus isolate Pula-A cytochrome oxidase...
Rhipicephalu... brown dog tick 34632 1140 1140 94% 0.0 99.84 630
KX757888.1
Rhipicephalus sanguineus isolate Petnica cytochrome oxidase...
Rhipicephalu... brown dog tick 34632 1140 1140 94% 0.0 99.84 630
KX757883.1
Rhipicephalus sanguineus clone #3 cytochrome oxidase subunit I...
Rhipicephalu... brown dog tick 34632 1140 1140 93% 0.0 100.00 629
KX519708.1
Rhipicephalus sanguineus isolate SV2 cytochrome c oxidase...
Rhipicephalu... brown dog tick 34632 1140 1140 94% 0.0 99.84 630
MW135447.1
Rhipicephalus sanguineus sensu lato 'temperate lineage' isolat...
Rhipicephalu... brown dog tick 34632 1138 1138 94% 0.0 99.68 649
MF426014.1
Rhipicephalus sanguineus clone #2 cytochrome oxidase subunit I...
Rhipicephalu... brown dog tick 34632 1138 1138 93% 0.0 100.00 626
KX519707.1
Rhipicephalus sanguineus isolate Szekszard cytochrome oxidase...
Rhipicephalu... brown dog tick 34632 1134 1134 94% 0.0 99.68 630
KX757901.1
Rhipicephalus sanguineus voucher Rs7 cytochrome oxidase subuni...
Rhipicephalu... brown dog tick 34632 1131 1131 95% 0.0 99.05 656
KU568516.1
Rhipicephalus sanguineus clone #4 cytochrome oxidase subunit I...
Rhipicephalu... brown dog tick 34632 1129 1129 94% 0.0 99.52 635
KX519709.1
Rhipicephalus sanguineus isolate LIC4723B cytochrome oxidase...
Rhipicephalu... brown dog tick 34632 1092 1092 90% 0.0 99.66 723
KX714599.1
Rhipicephalus sanguineus clone #8 cytochrome oxidase subunit I...
Rhipicephalu... brown dog tick 34632 1072 1072 88% 0.0 100.00 580
KX519713.1
Rhipicephalus sanguineus mitochondrial DNA, complete genome
Rhipicephalu... brown dog tick 34632 1061 1061 100% 0.0 95.74 14710
AF081829.1
Rhipicephalus sanguineus clone #1 cytochrome oxidase subunit I...
Rhipicephalu... brown dog tick 34632 1053 1053 86% 0.0 100.00 595
KX519706.1

```
Rhipicephalus sanguineus sensu lato 'temperate lineage' isolat...
Rhipicephalu... brown dog tick  34632      1014   1014  85%   0.0   99.29  574
     MF425987.1
Rhipicephalus sanguineus clone #5 cytochrome oxidase subunit I...
Rhipicephalu... brown dog tick  34632      989    989   83%   0.0   99.27  553
     KX519710.1
Rhipicephalus sanguineus isolate Italy-A cytochrome oxidase...
Rhipicephalu... brown dog tick  34632      985    985   94%   0.0   95.32  630
     KX757903.1
Rhipicephalus sanguineus isolate Zagreb-B cytochrome oxidase...
Rhipicephalu... brown dog tick  34632      985    985   94%   0.0   95.32  629
     KX757893.1
Rhipicephalus sanguineus isolate Al-Hosema-A cytochrome oxidas...
Rhipicephalu... brown dog tick  34632      979    979   94%   0.0   95.16  630
     KX757909.1
Rhipicephalus sanguineus isolate FT157 cytochrome c oxidase...
Rhipicephalu... brown dog tick  34632      963    963   79%   0.0   99.81  535
     MN223690.1
Rhipicephalus sanguineus isolate FT154 cytochrome c oxidase...
Rhipicephalu... brown dog tick  34632      957    957   79%   0.0   99.62  535
     MN223688.1
Rhipicephalus sanguineus isolate CE67 cytochrome c oxidase...
Rhipicephalu... brown dog tick  34632      957    957   79%   0.0   99.62  535
     MN223686.1
Rhipicephalus sanguineus isolate CE61 cytochrome c oxidase...
Rhipicephalu... brown dog tick  34632      952    952   79%   0.0   99.43  535
     MN223689.1
Rhipicephalus sanguineus isolate RI46 cytochrome c oxidase...
Rhipicephalu... brown dog tick  34632      952    952   79%   0.0   99.43  535
     MN223687.1
Rhipicephalus sanguineus isolate RI08 cytochrome c oxidase...
Rhipicephalu... brown dog tick  34632      948    948   79%   0.0   99.43  535
     MN223684.1
Rhipicephalus sanguineus isolate RI54 cytochrome c oxidase...
Rhipicephalu... brown dog tick  34632      946    946   79%   0.0   99.24  535
     MN223685.1
Rhipicephalus sanguineus isolate FT044 cytochrome c oxidase...
Rhipicephalu... brown dog tick  34632      946    946   79%   0.0   99.24  535
     MN223683.1
Rhipicephalus sanguineus isolate Rsang2 cytochrome oxidase...
Rhipicephalu... brown dog tick  34632      900    900   100%  0.0   91.34  790
     KY678135.1
Rhipicephalus sanguineus cytochrome oxidase subunit 1 (CO1)...
Rhipicephalu... brown dog tick  34632      898    898   98%   0.0   91.67  829
     KU214592.1
Rhipicephalus sanguineus isolate Gilan-e Gharb cytochrome...
Rhipicephalu... brown dog tick  34632      889    889   100%  0.0   91.03  1539
     KM494916.1
Rhipicephalus sanguineus isolate Tehran cytochrome oxidase...
Rhipicephalu... brown dog tick  34632      889    889   100%  0.0   91.03  1539
     KM494915.1
Rhipicephalus camicasi isolate SC0126 mitochondrion, complete...
Rhipicephalu... NA              669981     889    889   100%  0.0   91.03  14725
     NC_061616.1
```

```
Rhipicephalus turanicus isolate COX1-225-Goat cytochrome c...
Rhipicephalu... NA             34633      883    883   100%  0.0   90.88  673
     MT800313.1
Rhipicephalus turanicus isolate COX1-18-Sheep cytochrome c...
Rhipicephalu... NA             34633      881    881   99%   0.0   90.87  673
     MT800314.1
Rhipicephalus turanicus isolate 31X cytochrome oxidase subunit...
Rhipicephalu... NA             34633      872    872   100%  0.0   90.58  873
     KY606303.1
Rhipicephalus turanicus isolate 19X cytochrome oxidase subunit...
Rhipicephalu... NA             34633      872    872   100%  0.0   90.58  873
     KY606302.1
Rhipicephalus turanicus isolate 19C cytochrome oxidase subunit...
Rhipicephalu... NA             34633      872    872   100%  0.0   90.58  873
     KY606301.1
Rhipicephalus turanicus isolate 12X cytochrome oxidase subunit...
Rhipicephalu... NA             34633      872    872   100%  0.0   90.58  873
     KY606300.1
Rhipicephalus turanicus isolate 12C cytochrome oxidase subunit...
Rhipicephalu... NA             34633      872    872   100%  0.0   90.58  873
     KY606299.1
Rhipicephalus turanicus isolate COX1-7-Sheep cytochrome c...
Rhipicephalu... NA             34633      865    865   99%   0.0   90.41  673
     MT800312.1
Rhipicephalus turanicus isolate GY34-1 cytochrome c oxidase...
Rhipicephalu... NA             34633      861    861   99%   0.0   90.49  678
     MT079206.1
Rhipicephalus turanicus voucher CROBB808 cytochrome oxidase...
Rhipicephalu... NA             34633      861    861   100%  0.0   90.27  658
     MZ305547.1


Alignments:

>Rhipicephalus sanguineus sensu lato 'temperate lineage' isolate S827 cytochrome
c oxidase subunit I (COI) gene, partial cds; mitochondrial
Sequence ID: MF426019.1 Length: 697
Range 1: 19 to 676

Score:1216 bits(658), Expect:0.0,
Identities:658/658(100%),  Gaps:0/658(0%), Strand: Plus/Plus

Query  1    AACAATATATTTAATTTTTGGAGCATGATCAGGTATATTAGGACTTAGTATAAGAATATT  60
            ||||||||||||||||||||||||||||||||||||||||||||||||||||||||||||
Sbjct  19   AACAATATATTTAATTTTTGGAGCATGATCAGGTATATTAGGACTTAGTATAAGAATATT  78

Query  61   AATTCGTATAGAATTAGGACAACCTGGAACTTTAATTGGAAATGATCAGATTTATAATGT  120
            ||||||||||||||||||||||||||||||||||||||||||||||||||||||||||||
Sbjct  79   AATTCGTATAGAATTAGGACAACCTGGAACTTTAATTGGAAATGATCAGATTTATAATGT  138

Query  121  AATCGTAACAGCACATGCATTTATTATAAttttttttATAGTTATACCAATCATAATCGG  180
            ||||||||||||||||||||||||||||||||||||||||||||||||||||||||||||
Sbjct  139  AATCGTAACAGCACATGCATTTATTATAATTTTTTTTATAGTTATACCAATCATAATCGG  198
```

```
Query  181  AGGATTTGGAAATTGATTAGTCCCTATCATATTGGGTGCCCCAGATATAGCATTTCCACG  240
            ||||||||||||||||||||||||||||||||||||||||||||||||||||||||||||
Sbjct  199  AGGATTTGGAAATTGATTAGTCCCTATCATATTGGGTGCCCCAGATATAGCATTTCCACG  258

Query  241  AATAAATAATATAAGATTTTGACTTTTACCTCCTTCACTATTTTTATTAATTAACTCTTC  300
            ||||||||||||||||||||||||||||||||||||||||||||||||||||||||||||
Sbjct  259  AATAAATAATATAAGATTTTGACTTTTACCTCCTTCACTATTTTTATTAATTAACTCTTC  318

Query  301  ATTAATTGAATCTGGAGCTGGTACAGGATGAACAGTTTACCCTCCTCTATCTTCAAATTT  360
            ||||||||||||||||||||||||||||||||||||||||||||||||||||||||||||
Sbjct  319  ATTAATTGAATCTGGAGCTGGTACAGGATGAACAGTTTACCCTCCTCTATCTTCAAATTT  378

Query  361  ATCACATTATGGCCCTTCAGTAGATTTAGCTATTTTTTCTCTTCATCTTGCTGGTGCTTC  420
            ||||||||||||||||||||||||||||||||||||||||||||||||||||||||||||
Sbjct  379  ATCACATTATGGCCCTTCAGTAGATTTAGCTATTTTTTCTCTTCATCTTGCTGGTGCTTC  438

Query  421  TTCAATTTTAGGTGCAATTAACTTTATTACAACTATTGTAAACATACGATCAATTGGAAT  480
            ||||||||||||||||||||||||||||||||||||||||||||||||||||||||||||
Sbjct  439  TTCAATTTTAGGTGCAATTAACTTTATTACAACTATTGTAAACATACGATCAATTGGAAT  498

Query  481  AACAATAGAACGTATACCATTATTTGTTTGATCTGTTTTAATTACAGCTATTTTATTACT  540
            ||||||||||||||||||||||||||||||||||||||||||||||||||||||||||||
Sbjct  499  AACAATAGAACGTATACCATTATTTGTTTGATCTGTTTTAATTACAGCTATTTTATTACT  558

Query  541  TTTATCTTTACCTGTATTAGCAGGTGCCATTACAATATTGTTAACTGATCGAAACTTTAA  600
            ||||||||||||||||||||||||||||||||||||||||||||||||||||||||||||
Sbjct  559  TTTATCTTTACCTGTATTAGCAGGTGCCATTACAATATTGTTAACTGATCGAAACTTTAA  618

Query  601  TACATCATTTTTTGATCCTTCAGGGGGAGGAGATCCAATTTTATATCAACATTTATTT  658
            ||||||||||||||||||||||||||||||||||||||||||||||||||||||||||
Sbjct  619  TACATCATTTTTTGATCCTTCAGGGGGAGGAGATCCAATTTTATATCAACATTTATTT  676
```

>Rhipicephalus sanguineus haplotype III cytochrome oxidase subunit I (COI) gene, partial cds; mitochondrial
Sequence ID: KU556745.1 Length: 713
Range 1: 27 to 684

Score:1216 bits(658), Expect:0.0,
Identities:658/658(100%), Gaps:0/658(0%), Strand: Plus/Plus

```
Query  1    AACAATATATTTAATTTTTGGAGCATGATCAGGTATATTAGGACTTAGTATAAGAATATT  60
            ||||||||||||||||||||||||||||||||||||||||||||||||||||||||||||
Sbjct  27   AACAATATATTTAATTTTTGGAGCATGATCAGGTATATTAGGACTTAGTATAAGAATATT  86

Query  61   AATTCGTATAGAATTAGGACAACCTGGAACTTTAATTGGAAATGATCAGATTTATAATGT  120
            ||||||||||||||||||||||||||||||||||||||||||||||||||||||||||||
Sbjct  87   AATTCGTATAGAATTAGGACAACCTGGAACTTTAATTGGAAATGATCAGATTTATAATGT  146

Query  121  AATCGTAACAGCACATGCATTTATTATAAttttttttATAGTTATACCAATCATAATCGG  180
            ||||||||||||||||||||||||||||||||||||||||||||||||||||||||||||
Sbjct  147  AATCGTAACAGCACATGCATTTATTATAATTTTTTTTATAGTTATACCAATCATAATCGG  206
```

```
Query  181  AGGATTTGGAAATTGATTAGTCCCTATCATATTGGGTGCCCCAGATATAGCATTTCCACG  240
            ||||||||||||||||||||||||||||||||||||||||||||||||||||||||||||
Sbjct  207  AGGATTTGGAAATTGATTAGTCCCTATCATATTGGGTGCCCCAGATATAGCATTTCCACG  266

Query  241  AATAAATAATATAAGATTTTGACTTTTACCTCCTTCACTATTTTTATTAATTAACTCTTC  300
            ||||||||||||||||||||||||||||||||||||||||||||||||||||||||||||
Sbjct  267  AATAAATAATATAAGATTTTGACTTTTACCTCCTTCACTATTTTTATTAATTAACTCTTC  326

Query  301  ATTAATTGAATCTGGAGCTGGTACAGGATGAACAGTTTACCCTCCTCTATCTTCAAATTT  360
            ||||||||||||||||||||||||||||||||||||||||||||||||||||||||||||
Sbjct  327  ATTAATTGAATCTGGAGCTGGTACAGGATGAACAGTTTACCCTCCTCTATCTTCAAATTT  386

Query  361  ATCACATTATGGCCCTTCAGTAGATTTAGCTATTTTTTCTCTTCATCTTGCTGGTGCTTC  420
            ||||||||||||||||||||||||||||||||||||||||||||||||||||||||||||
Sbjct  387  ATCACATTATGGCCCTTCAGTAGATTTAGCTATTTTTTCTCTTCATCTTGCTGGTGCTTC  446

Query  421  TTCAATTTTAGGTGCAATTAACTTTATTACAACTATTGTAAACATACGATCAATTGGAAT  480
            ||||||||||||||||||||||||||||||||||||||||||||||||||||||||||||
Sbjct  447  TTCAATTTTAGGTGCAATTAACTTTATTACAACTATTGTAAACATACGATCAATTGGAAT  506

Query  481  AACAATAGAACGTATACCATTATTTGTTTGATCTGTTTTAATTACAGCTATTTTATTACT  540
            ||||||||||||||||||||||||||||||||||||||||||||||||||||||||||||
Sbjct  507  AACAATAGAACGTATACCATTATTTGTTTGATCTGTTTTAATTACAGCTATTTTATTACT  566

Query  541  TTTATCTTTACCTGTATTAGCAGGTGCCATTACAATATTGTTAACTGATCGAAACTTTAA  600
            ||||||||||||||||||||||||||||||||||||||||||||||||||||||||||||
Sbjct  567  TTTATCTTTACCTGTATTAGCAGGTGCCATTACAATATTGTTAACTGATCGAAACTTTAA  626

Query  601  TACATCATTTTTTGATCCTTCAGGGGGAGGAGATCCAATTTTATATCAACATTTATTT  658
            ||||||||||||||||||||||||||||||||||||||||||||||||||||||||||
Sbjct  627  TACATCATTTTTTGATCCTTCAGGGGGAGGAGATCCAATTTTATATCAACATTTATTT  684
```

```
>Rhipicephalus sanguineus isolate Arizona-1 cytochrome c oxidase subunit 1 (COI)
gene, partial cds; mitochondrial
Sequence ID: MN593344.1 Length: 710
Range 1: 27 to 684

Score:1216 bits(658), Expect:0.0,
Identities:658/658(100%),  Gaps:0/658(0%), Strand: Plus/Minus

Query  1    AACAATATATTTAATTTTTGGAGCATGATCAGGTATATTAGGACTTAGTATAAGAATATT  60
            ||||||||||||||||||||||||||||||||||||||||||||||||||||||||||||
Sbjct  684  AACAATATATTTAATTTTTGGAGCATGATCAGGTATATTAGGACTTAGTATAAGAATATT  625

Query  61   AATTCGTATAGAATTAGGACAACCTGGAACTTTAATTGGAAATGATCAGATTTATAATGT  120
            ||||||||||||||||||||||||||||||||||||||||||||||||||||||||||||
Sbjct  624  AATTCGTATAGAATTAGGACAACCTGGAACTTTAATTGGAAATGATCAGATTTATAATGT  565

Query  121  AATCGTAACAGCACATGCATTTATTATAAttttttttATAGTTATACCAATCATAATCGG  180
            ||||||||||||||||||||||||||||||||||||||||||||||||||||||||||||
```

```
Sbjct  564  AATCGTAACAGCACATGCATTTATTATAATTTTTTTTATAGTTATACCAATCATAATCGG  505

Query  181  AGGATTTGGAAATTGATTAGTCCCTATCATATTGGGTGCCCCAGATATAGCATTTCCACG  240
            ||||||||||||||||||||||||||||||||||||||||||||||||||||||||||||
Sbjct  504  AGGATTTGGAAATTGATTAGTCCCTATCATATTGGGTGCCCCAGATATAGCATTTCCACG  445

Query  241  AATAAATAATATAAGATTTTGACTTTTACCTCCTTCACTATTTTTATTAATTAACTCTTC  300
            ||||||||||||||||||||||||||||||||||||||||||||||||||||||||||||
Sbjct  444  AATAAATAATATAAGATTTTGACTTTTACCTCCTTCACTATTTTTATTAATTAACTCTTC  385

Query  301  ATTAATTGAATCTGGAGCTGGTACAGGATGAACAGTTTACCCTCCTCTATCTTCAAATTT  360
            ||||||||||||||||||||||||||||||||||||||||||||||||||||||||||||
Sbjct  384  ATTAATTGAATCTGGAGCTGGTACAGGATGAACAGTTTACCCTCCTCTATCTTCAAATTT  325

Query  361  ATCACATTATGGCCCTTCAGTAGATTTAGCTATTTTTTCTCTTCATCTTGCTGGTGCTTC  420
            ||||||||||||||||||||||||||||||||||||||||||||||||||||||||||||
Sbjct  324  ATCACATTATGGCCCTTCAGTAGATTTAGCTATTTTTTCTCTTCATCTTGCTGGTGCTTC  265

Query  421  TTCAATTTTAGGTGCAATTAACTTTATTACAACTATTGTAAACATACGATCAATTGGAAT  480
            ||||||||||||||||||||||||||||||||||||||||||||||||||||||||||||
Sbjct  264  TTCAATTTTAGGTGCAATTAACTTTATTACAACTATTGTAAACATACGATCAATTGGAAT  205

Query  481  AACAATAGAACGTATACCATTATTTGTTTGATCTGTTTTAATTACAGCTATTTTATTACT  540
            ||||||||||||||||||||||||||||||||||||||||||||||||||||||||||||
Sbjct  204  AACAATAGAACGTATACCATTATTTGTTTGATCTGTTTTAATTACAGCTATTTTATTACT  145

Query  541  TTTATCTTTACCTGTATTAGCAGGTGCCATTACAATATTGTTAACTGATCGAAACTTTAA  600
            ||||||||||||||||||||||||||||||||||||||||||||||||||||||||||||
Sbjct  144  TTTATCTTTACCTGTATTAGCAGGTGCCATTACAATATTGTTAACTGATCGAAACTTTAA  85

Query  601  TACATCATTTTTTGATCCTTCAGGGGGAGGAGATCCAATTTTATATCAACATTTATTT  658
            ||||||||||||||||||||||||||||||||||||||||||||||||||||||||||
Sbjct  84   TACATCATTTTTTGATCCTTCAGGGGGAGGAGATCCAATTTTATATCAACATTTATTT  27
```

>Rhipicephalus sanguineus isolate Arizona-2 cytochrome c oxidase subunit 1 (COI) gene, partial cds; mitochondrial
Sequence ID: MN593343.1 Length: 710
Range 1: 27 to 684

Score:1216 bits(658), Expect:0.0,
Identities:658/658(100%), Gaps:0/658(0%), Strand: Plus/Minus

```
Query  1    AACAATATATTTAATTTTTGGAGCATGATCAGGTATATTAGGACTTAGTATAAGAATATT  60
            ||||||||||||||||||||||||||||||||||||||||||||||||||||||||||||
Sbjct  684  AACAATATATTTAATTTTTGGAGCATGATCAGGTATATTAGGACTTAGTATAAGAATATT  625

Query  61   AATTCGTATAGAATTAGGACAACCTGGAACTTTAATTGGAAATGATCAGATTTATAATGT  120
            ||||||||||||||||||||||||||||||||||||||||||||||||||||||||||||
Sbjct  624  AATTCGTATAGAATTAGGACAACCTGGAACTTTAATTGGAAATGATCAGATTTATAATGT  565

Query  121  AATCGTAACAGCACATGCATTTATTATAAttttttttATAGTTATACCAATCATAATCGG  180
```

```
            ||||||||||||||||||||||||||||||||||||||||||||||||||||||||||||
Sbjct  564  AATCGTAACAGCACATGCATTTATTATAATTTTTTTTATAGTTATACCAATCATAATCGG  505

Query  181  AGGATTTGGAAATTGATTAGTCCCTATCATATTGGGTGCCCCAGATATAGCATTTCCACG  240
            ||||||||||||||||||||||||||||||||||||||||||||||||||||||||||||
Sbjct  504  AGGATTTGGAAATTGATTAGTCCCTATCATATTGGGTGCCCCAGATATAGCATTTCCACG  445

Query  241  AATAAATAATATAAGATTTTGACTTTTACCTCCTTCACTATTTTTATTAATTAACTCTTC  300
            ||||||||||||||||||||||||||||||||||||||||||||||||||||||||||||
Sbjct  444  AATAAATAATATAAGATTTTGACTTTTACCTCCTTCACTATTTTTATTAATTAACTCTTC  385

Query  301  ATTAATTGAATCTGGAGCTGGTACAGGATGAACAGTTTACCCTCCTCTATCTTCAAATTT  360
            ||||||||||||||||||||||||||||||||||||||||||||||||||||||||||||
Sbjct  384  ATTAATTGAATCTGGAGCTGGTACAGGATGAACAGTTTACCCTCCTCTATCTTCAAATTT  325

Query  361  ATCACATTATGGCCCTTCAGTAGATTTAGCTATTTTTTCTCTTCATCTTGCTGGTGCTTC  420
            ||||||||||||||||||||||||||||||||||||||||||||||||||||||||||||
Sbjct  324  ATCACATTATGGCCCTTCAGTAGATTTAGCTATTTTTTCTCTTCATCTTGCTGGTGCTTC  265

Query  421  TTCAATTTTAGGTGCAATTAACTTTATTACAACTATTGTAAACATACGATCAATTGGAAT  480
            ||||||||||||||||||||||||||||||||||||||||||||||||||||||||||||
Sbjct  264  TTCAATTTTAGGTGCAATTAACTTTATTACAACTATTGTAAACATACGATCAATTGGAAT  205

Query  481  AACAATAGAACGTATACCATTATTTGTTTGATCTGTTTTAATTACAGCTATTTTATTACT  540
            ||||||||||||||||||||||||||||||||||||||||||||||||||||||||||||
Sbjct  204  AACAATAGAACGTATACCATTATTTGTTTGATCTGTTTTAATTACAGCTATTTTATTACT  145

Query  541  TTTATCTTTACCTGTATTAGCAGGTGCCATTACAATATTGTTAACTGATCGAAACTTTAA  600
            ||||||||||||||||||||||||||||||||||||||||||||||||||||||||||||
Sbjct  144  TTTATCTTTACCTGTATTAGCAGGTGCCATTACAATATTGTTAACTGATCGAAACTTTAA  85

Query  601  TACATCATTTTTTGATCCTTCAGGGGGAGGAGATCCAATTTTATATCAACATTTATTT  658
            ||||||||||||||||||||||||||||||||||||||||||||||||||||||||||
Sbjct  84   TACATCATTTTTTGATCCTTCAGGGGGAGGAGATCCAATTTTATATCAACATTTATTT  27
```

>Rhipicephalus sanguineus isolate 11N cytochrome oxidase subunit I (COX1) gene, partial cds; mitochondrial
Sequence ID: MN585197.1 Length: 703
Range 1: 22 to 679

Score:1216 bits(658), Expect:0.0,
Identities:658/658(100%), Gaps:0/658(0%), Strand: Plus/Minus

```
Query  1    AACAATATATTTAATTTTTGGAGCATGATCAGGTATATTAGGACTTAGTATAAGAATATT  60
            ||||||||||||||||||||||||||||||||||||||||||||||||||||||||||||
Sbjct  679  AACAATATATTTAATTTTTGGAGCATGATCAGGTATATTAGGACTTAGTATAAGAATATT  620

Query  61   AATTCGTATAGAATTAGGACAACCTGGAACTTTAATTGGAAATGATCAGATTTATAATGT  120
            ||||||||||||||||||||||||||||||||||||||||||||||||||||||||||||
Sbjct  619  AATTCGTATAGAATTAGGACAACCTGGAACTTTAATTGGAAATGATCAGATTTATAATGT  560
```

```
Query  121  AATCGTAACAGCACATGCATTTATTATAAttttttttATAGTTATACCAATCATAATCGG  180
            ||||||||||||||||||||||||||||||||||||||||||||||||||||||||||||
Sbjct  559  AATCGTAACAGCACATGCATTTATTATAATTTTTTTTATAGTTATACCAATCATAATCGG  500

Query  181  AGGATTTGGAAATTGATTAGTCCCTATCATATTGGGTGCCCCAGATATAGCATTTCCACG  240
            ||||||||||||||||||||||||||||||||||||||||||||||||||||||||||||
Sbjct  499  AGGATTTGGAAATTGATTAGTCCCTATCATATTGGGTGCCCCAGATATAGCATTTCCACG  440

Query  241  AATAAATAATATAAGATTTTGACTTTTACCTCCTTCACTATTTTTATTAATTAACTCTTC  300
            ||||||||||||||||||||||||||||||||||||||||||||||||||||||||||||
Sbjct  439  AATAAATAATATAAGATTTTGACTTTTACCTCCTTCACTATTTTTATTAATTAACTCTTC  380

Query  301  ATTAATTGAATCTGGAGCTGGTACAGGATGAACAGTTTACCCTCCTCTATCTTCAAATTT  360
            ||||||||||||||||||||||||||||||||||||||||||||||||||||||||||||
Sbjct  379  ATTAATTGAATCTGGAGCTGGTACAGGATGAACAGTTTACCCTCCTCTATCTTCAAATTT  320

Query  361  ATCACATTATGGCCCTTCAGTAGATTTAGCTATTTTTTCTCTTCATCTTGCTGGTGCTTC  420
            ||||||||||||||||||||||||||||||||||||||||||||||||||||||||||||
Sbjct  319  ATCACATTATGGCCCTTCAGTAGATTTAGCTATTTTTTCTCTTCATCTTGCTGGTGCTTC  260

Query  421  TTCAATTTTAGGTGCAATTAACTTTATTACAACTATTGTAAACATACGATCAATTGGAAT  480
            ||||||||||||||||||||||||||||||||||||||||||||||||||||||||||||
Sbjct  259  TTCAATTTTAGGTGCAATTAACTTTATTACAACTATTGTAAACATACGATCAATTGGAAT  200

Query  481  AACAATAGAACGTATACCATTATTTGTTTGATCTGTTTTAATTACAGCTATTTTATTACT  540
            ||||||||||||||||||||||||||||||||||||||||||||||||||||||||||||
Sbjct  199  AACAATAGAACGTATACCATTATTTGTTTGATCTGTTTTAATTACAGCTATTTTATTACT  140

Query  541  TTTATCTTTACCTGTATTAGCAGGTGCCATTACAATATTGTTAACTGATCGAAACTTTAA  600
            ||||||||||||||||||||||||||||||||||||||||||||||||||||||||||||
Sbjct  139  TTTATCTTTACCTGTATTAGCAGGTGCCATTACAATATTGTTAACTGATCGAAACTTTAA  80

Query  601  TACATCATTTTTTGATCCTTCAGGGGGAGGAGATCCAATTTTATATCAACATTTATTT  658
            ||||||||||||||||||||||||||||||||||||||||||||||||||||||||||
Sbjct  79   TACATCATTTTTTGATCCTTCAGGGGGAGGAGATCCAATTTTATATCAACATTTATTT  22
```

>Rhipicephalus sanguineus cytochrome c oxidase I (COI) gene, partial cds;
mitochondrial gene for mitochondrial product
Sequence ID: AF132839.1 Length: 793
Range 1: 42 to 699

Score:1216 bits(658), Expect:0.0,
Identities:658/658(100%), Gaps:0/658(0%), Strand: Plus/Plus

```
Query  1    AACAATATATTTAATTTTTGGAGCATGATCAGGTATATTAGGACTTAGTATAAGAATATT  60
            ||||||||||||||||||||||||||||||||||||||||||||||||||||||||||||
Sbjct  42   AACAATATATTTAATTTTTGGAGCATGATCAGGTATATTAGGACTTAGTATAAGAATATT  101

Query  61   AATTCGTATAGAATTAGGACAACCTGGAACTTTAATTGGAAATGATCAGATTTATAATGT  120
            ||||||||||||||||||||||||||||||||||||||||||||||||||||||||||||
Sbjct  102  AATTCGTATAGAATTAGGACAACCTGGAACTTTAATTGGAAATGATCAGATTTATAATGT  161
```

```
Query  121   AATCGTAACAGCACATGCATTTATTATAAttttttttATAGTTATACCAATCATAATCGG  180
             ||||||||||||||||||||||||||||||||||||||||||||||||||||||||||||
Sbjct  162   AATCGTAACAGCACATGCATTTATTATAATTTTTTTTATAGTTATACCAATCATAATCGG  221

Query  181   AGGATTTGGAAATTGATTAGTCCCTATCATATTGGGTGCCCCAGATATAGCATTTCCACG  240
             ||||||||||||||||||||||||||||||||||||||||||||||||||||||||||||
Sbjct  222   AGGATTTGGAAATTGATTAGTCCCTATCATATTGGGTGCCCCAGATATAGCATTTCCACG  281

Query  241   AATAAATAATATAAGATTTTGACTTTTACCTCCTTCACTATTTTTATTAATTAACTCTTC  300
             ||||||||||||||||||||||||||||||||||||||||||||||||||||||||||||
Sbjct  282   AATAAATAATATAAGATTTTGACTTTTACCTCCTTCACTATTTTTATTAATTAACTCTTC  341

Query  301   ATTAATTGAATCTGGAGCTGGTACAGGATGAACAGTTTACCCTCCTCTATCTTCAAATTT  360
             ||||||||||||||||||||||||||||||||||||||||||||||||||||||||||||
Sbjct  342   ATTAATTGAATCTGGAGCTGGTACAGGATGAACAGTTTACCCTCCTCTATCTTCAAATTT  401

Query  361   ATCACATTATGGCCCTTCAGTAGATTTAGCTATTTTTTCTCTTCATCTTGCTGGTGCTTC  420
             ||||||||||||||||||||||||||||||||||||||||||||||||||||||||||||
Sbjct  402   ATCACATTATGGCCCTTCAGTAGATTTAGCTATTTTTTCTCTTCATCTTGCTGGTGCTTC  461

Query  421   TTCAATTTTAGGTGCAATTAACTTTATTACAACTATTGTAAACATACGATCAATTGGAAT  480
             ||||||||||||||||||||||||||||||||||||||||||||||||||||||||||||
Sbjct  462   TTCAATTTTAGGTGCAATTAACTTTATTACAACTATTGTAAACATACGATCAATTGGAAT  521

Query  481   AACAATAGAACGTATACCATTATTTGTTTGATCTGTTTTAATTACAGCTATTTTATTACT  540
             ||||||||||||||||||||||||||||||||||||||||||||||||||||||||||||
Sbjct  522   AACAATAGAACGTATACCATTATTTGTTTGATCTGTTTTAATTACAGCTATTTTATTACT  581

Query  541   TTTATCTTTACCTGTATTAGCAGGTGCCATTACAATATTGTTAACTGATCGAAACTTTAA  600
             ||||||||||||||||||||||||||||||||||||||||||||||||||||||||||||
Sbjct  582   TTTATCTTTACCTGTATTAGCAGGTGCCATTACAATATTGTTAACTGATCGAAACTTTAA  641

Query  601   TACATCATTTTTTGATCCTTCAGGGGGAGGAGATCCAATTTTATATCAACATTTATTT  658
             ||||||||||||||||||||||||||||||||||||||||||||||||||||||||||
Sbjct  642   TACATCATTTTTTGATCCTTCAGGGGGAGGAGATCCAATTTTATATCAACATTTATTT  699
```

>Rhipicephalus sanguineus sensu lato 'temperate lineage' isolate S58 cytochrome c oxidase subunit I (COI) gene, partial cds; mitochondrial
Sequence ID: MF426018.1 Length: 675
Range 1: 11 to 668

Score:1210 bits(655), Expect:0.0,
Identities:657/658(99%), Gaps:0/658(0%), Strand: Plus/Plus

```
Query  1     AACAATATATTTAATTTTTGGAGCATGATCAGGTATATTAGGACTTAGTATAAGAATATT  60
             ||||||||||||||||||||||||||||||||||||||||||||||||||||||||||||
Sbjct  11    AACAATATATTTAATTTTTGGAGCATGATCAGGTATATTAGGACTTAGTATAAGAATATT  70

Query  61    AATTCGTATAGAATTAGGACAACCTGGAACTTTAATTGGAAATGATCAGATTTATAATGT  120
             ||||||||||||||||||||||||||||||||||||||||||||||||||||||||||||
```

```
Sbjct  71    AATTCGTATAGAATTAGGACAACCTGGAACTTTAATTGGAAATGATCAGATTTATAATGT  130

Query  121   AATCGTAACAGCACATGCATTTATTATAAttttttttATAGTTATACCAATCATAATCGG  180
             ||||||||||||||||||||||||||||||||||||||||||||||||||||||||||||
Sbjct  131   AATCGTAACAGCACATGCATTTATTATAATTTTTTTTATAGTTATACCAATCATAATCGG  190

Query  181   AGGATTTGGAAATTGATTAGTCCCTATCATATTGGGTGCCCCAGATATAGCATTTCCACG  240
             ||||||||||||||||||||||||||||||||||||||||||||||||||||||||||||
Sbjct  191   AGGATTTGGAAATTGATTAGTCCCTATCATATTGGGTGCCCCAGATATAGCATTTCCACG  250

Query  241   AATAAATAATATAAGATTTTGACTTTTACCTCCTTCACTATTTTTATTAATTAACTCTTC  300
             ||||||||||||||||||||||||||||||||||||||||||||||||||||||||||||
Sbjct  251   AATAAATAATATAAGATTTTGACTTTTACCTCCTTCACTATTTTTATTAATTAACTCTTC  310

Query  301   ATTAATTGAATCTGGAGCTGGTACAGGATGAACAGTTTACCCTCCTCTATCTTCAAATTT  360
             ||||||||||||||||||||||||||||||||||||||||||||||||||||||||||||
Sbjct  311   ATTAATTGAATCTGGAGCTGGTACAGGATGAACAGTTTACCCTCCTCTATCTTCAAATTT  370

Query  361   ATCACATTATGGCCCTTCAGTAGATTTAGCTATTTTTTCTCTTCATCTTGCTGGTGCTTC  420
             |||||||||||| |||||||||||||||||||||||||||||||||||||||||||||||
Sbjct  371   ATCACATTATGGTCCTTCAGTAGATTTAGCTATTTTTTCTCTTCATCTTGCTGGTGCTTC  430

Query  421   TTCAATTTTAGGTGCAATTAACTTTATTACAACTATTGTAAACATACGATCAATTGGAAT  480
             ||||||||||||||||||||||||||||||||||||||||||||||||||||||||||||
Sbjct  431   TTCAATTTTAGGTGCAATTAACTTTATTACAACTATTGTAAACATACGATCAATTGGAAT  490

Query  481   AACAATAGAACGTATACCATTATTTGTTTGATCTGTTTTAATTACAGCTATTTTATTACT  540
             ||||||||||||||||||||||||||||||||||||||||||||||||||||||||||||
Sbjct  491   AACAATAGAACGTATACCATTATTTGTTTGATCTGTTTTAATTACAGCTATTTTATTACT  550

Query  541   TTTATCTTTACCTGTATTAGCAGGTGCCATTACAATATTGTTAACTGATCGAAACTTTAA  600
             ||||||||||||||||||||||||||||||||||||||||||||||||||||||||||||
Sbjct  551   TTTATCTTTACCTGTATTAGCAGGTGCCATTACAATATTGTTAACTGATCGAAACTTTAA  610

Query  601   TACATCATTTTTTGATCCTTCAGGGGGAGGAGATCCAATTTTATATCAACATTTATTT  658
             ||||||||||||||||||||||||||||||||||||||||||||||||||||||||||
Sbjct  611   TACATCATTTTTTGATCCTTCAGGGGGAGGAGATCCAATTTTATATCAACATTTATTT  668
```

>Rhipicephalus sanguineus sensu lato 'temperate lineage' isolate S52 cytochrome c oxidase subunit I (COI) gene, partial cds; mitochondrial
Sequence ID: MF426016.1 Length: 680
Range 1: 12 to 669

Score:1210 bits(655), Expect:0.0,
Identities:657/658(99%), Gaps:0/658(0%), Strand: Plus/Plus

```
Query  1     AACAATATATTTAATTTTTGGAGCATGATCAGGTATATTAGGACTTAGTATAAGAATATT  60
             ||||||||||||||||||||||||||||||||||||||||||||||||||||||||||||
Sbjct  12    AACAATATATTTAATTTTTGGAGCATGATCAGGTATATTAGGACTTAGTATAAGAATATT  71

Query  61    AATTCGTATAGAATTAGGACAACCTGGAACTTTAATTGGAAATGATCAGATTTATAATGT  120
```

```
          ||||||||||||||||||||||||||||||||||||||||||||||||||||||||||||
Sbjct  72    AATTCGTATAGAATTAGGACAACCTGGAACTTTAATTGGAAATGATCAGATTTATAATGT  131

Query  121   AATCGTAACAGCACATGCATTTATTATAAtttttttATAGTTATACCAATCATAATCGG  180
             ||||||||||||||||||||||||||||||||||||||||||||||||||||||||||||
Sbjct  132   AATCGTAACAGCACATGCATTTATTATAATTTTTTTATAGTTATACCAATCATAATCGG  191

Query  181   AGGATTTGGAAATTGATTAGTCCCTATCATATTGGGTGCCCCAGATATAGCATTTCCACG  240
             ||||||||||||||||||||||||||||||||||||||||||||||||||||||||||||
Sbjct  192   AGGATTTGGAAATTGATTAGTCCCTATCATATTGGGTGCCCCAGATATAGCATTTCCACG  251

Query  241   AATAAATAATATAAGATTTTGACTTTTACCTCCTTCACTATTTTTATTAATTAACTCTTC  300
             ||||||||||||||||||||||||||||||||||||||||||||||||||||||||||||
Sbjct  252   AATAAATAATATAAGATTTTGACTTTTACCTCCTTCACTATTTTTATTAATTAACTCTTC  311

Query  301   ATTAATTGAATCTGGAGCTGGTACAGGATGAACAGTTTACCCTCCTCTATCTTCAAATTT  360
             ||||||||||||||||||||||||||||||||||||||||||||||||||||||||||||
Sbjct  312   ATTAATTGAATCTGGAGCTGGTACAGGATGAACAGTTTACCCTCCTCTATCTTCAAATTT  371

Query  361   ATCACATTATGGCCCTTCAGTAGATTTAGCTATTTTTTCTCTTCATCTTGCTGGTGCTTC  420
             |||||||||||| |||||||||||||||||||||||||||||||||||||||||||||||
Sbjct  372   ATCACATTATGGTCCTTCAGTAGATTTAGCTATTTTTTCTCTTCATCTTGCTGGTGCTTC  431

Query  421   TTCAATTTTAGGTGCAATTAACTTTATTACAACTATTGTAAACATACGATCAATTGGAAT  480
             ||||||||||||||||||||||||||||||||||||||||||||||||||||||||||||
Sbjct  432   TTCAATTTTAGGTGCAATTAACTTTATTACAACTATTGTAAACATACGATCAATTGGAAT  491

Query  481   AACAATAGAACGTATACCATTATTTGTTTGATCTGTTTTAATTACAGCTATTTTATTACT  540
             ||||||||||||||||||||||||||||||||||||||||||||||||||||||||||||
Sbjct  492   AACAATAGAACGTATACCATTATTTGTTTGATCTGTTTTAATTACAGCTATTTTATTACT  551

Query  541   TTTATCTTTACCTGTATTAGCAGGTGCCATTACAATATTGTTAACTGATCGAAACTTTAA  600
             ||||||||||||||||||||||||||||||||||||||||||||||||||||||||||||
Sbjct  552   TTTATCTTTACCTGTATTAGCAGGTGCCATTACAATATTGTTAACTGATCGAAACTTTAA  611

Query  601   TACATCATTTTTTGATCCTTCAGGGGGAGGAGATCCAATTTTATATCAACATTTATTT  658
             ||||||||||||||||||||||||||||||||||||||||||||||||||||||||||
Sbjct  612   TACATCATTTTTTGATCCTTCAGGGGGAGGAGATCCAATTTTATATCAACATTTATTT  669
```

>Rhipicephalus sanguineus sensu lato 'temperate lineage' isolate S37 cytochrome c oxidase subunit I (COI) gene, partial cds; mitochondrial
Sequence ID: MF426013.1 Length: 693
Range 1: 20 to 677

Score:1210 bits(655), Expect:0.0,
Identities:657/658(99%), Gaps:0/658(0%), Strand: Plus/Plus

```
Query  1     AACAATATATTTAATTTTTGGAGCATGATCAGGTATATTAGGACTTAGTATAAGAATATT  60
             ||||||||||||||||||||||||||||||||||||||||||||||||||||||||||||
Sbjct  20    AACAATATATTTAATTTTTGGAGCATGATCAGGTATATTAGGACTTAGTATAAGAATATT  79
```

```
Query  61    AATTCGTATAGAATTAGGACAACCTGGAACTTTAATTGGAAATGATCAGATTTATAATGT  120
             ||||||||||||||||||||||||||||||||||||||||||||||||||||||||||||
Sbjct  80    AATTCGTATAGAATTAGGACAACCTGGAACTTTAATTGGAAATGATCAGATTTATAATGT  139

Query  121   AATCGTAACAGCACATGCATTTATTATAAttttttttATAGTTATACCAATCATAATCGG  180
             ||||||||||||||||||||||||||||||||||||||||||||||||||||||||||||
Sbjct  140   AATCGTAACAGCACATGCATTTATTATAATTTTTTTTATAGTTATACCAATCATAATCGG  199

Query  181   AGGATTTGGAAATTGATTAGTCCCTATCATATTGGGTGCCCCAGATATAGCATTTCCACG  240
             ||||||||||||||||||||||||||||||||||||||||||||||||||||||||||||
Sbjct  200   AGGATTTGGAAATTGATTAGTCCCTATCATATTGGGTGCCCCAGATATAGCATTTCCACG  259

Query  241   AATAAATAATATAAGATTTTGACTTTTACCTCCTTCACTATTTTTATTAATTAACTCTTC  300
             ||||||||||||||||||||||||||||||||||||||||||||||||||||||||||||
Sbjct  260   AATAAATAATATAAGATTTTGACTTTTACCTCCTTCACTATTTTTATTAATTAACTCTTC  319

Query  301   ATTAATTGAATCTGGAGCTGGTACAGGATGAACAGTTTACCCTCCTCTATCTTCAAATTT  360
             ||||||||||||||||||||||||||||||||||||||||||||||||||||||||||||
Sbjct  320   ATTAATTGAATCTGGAGCTGGTACAGGATGAACAGTTTACCCTCCTCTATCTTCAAATTT  379

Query  361   ATCACATTATGGCCCTTCAGTAGATTTAGCTATTTTTTCTCTTCATCTTGCTGGTGCTTC  420
             |||||||||||| |||||||||||||||||||||||||||||||||||||||||||||||
Sbjct  380   ATCACATTATGGTCCTTCAGTAGATTTAGCTATTTTTTCTCTTCATCTTGCTGGTGCTTC  439

Query  421   TTCAATTTTAGGTGCAATTAACTTTATTACAACTATTGTAAACATACGATCAATTGGAAT  480
             ||||||||||||||||||||||||||||||||||||||||||||||||||||||||||||
Sbjct  440   TTCAATTTTAGGTGCAATTAACTTTATTACAACTATTGTAAACATACGATCAATTGGAAT  499

Query  481   AACAATAGAACGTATACCATTATTTGTTTGATCTGTTTTAATTACAGCTATTTTATTACT  540
             ||||||||||||||||||||||||||||||||||||||||||||||||||||||||||||
Sbjct  500   AACAATAGAACGTATACCATTATTTGTTTGATCTGTTTTAATTACAGCTATTTTATTACT  559

Query  541   TTTATCTTTACCTGTATTAGCAGGTGCCATTACAATATTGTTAACTGATCGAAACTTTAA  600
             ||||||||||||||||||||||||||||||||||||||||||||||||||||||||||||
Sbjct  560   TTTATCTTTACCTGTATTAGCAGGTGCCATTACAATATTGTTAACTGATCGAAACTTTAA  619

Query  601   TACATCATTTTTTGATCCTTCAGGGGGAGGAGATCCAATTTTATATCAACATTTATTT  658
             ||||||||||||||||||||||||||||||||||||||||||||||||||||||||||
Sbjct  620   TACATCATTTTTTGATCCTTCAGGGGGAGGAGATCCAATTTTATATCAACATTTATTT  677
```

>Rhipicephalus sanguineus sensu lato 'temperate lineage' isolate S33 cytochrome c oxidase subunit I (COI) gene, partial cds; mitochondrial
Sequence ID: MF426009.1 Length: 679
Range 1: 11 to 668

Score:1210 bits(655), Expect:0.0,
Identities:657/658(99%), Gaps:0/658(0%), Strand: Plus/Plus

```
Query  1     AACAATATATTTAATTTTTGGAGCATGATCAGGTATATTAGGACTTAGTATAAGAATATT  60
             ||||||||||||||||||||||||||||||||||||||||||||||||||||||||||||
Sbjct  11    AACAATATATTTAATTTTTGGAGCATGATCAGGTATATTAGGACTTAGTATAAGAATATT  70
```

```
Query  61   AATTCGTATAGAATTAGGACAACCTGGAACTTTAATTGGAAATGATCAGATTTATAATGT  120
            ||||||||||||||||||||||||||||||||||||||||||||||||||||||||||||
Sbjct  71   AATTCGTATAGAATTAGGACAACCTGGAACTTTAATTGGAAATGATCAGATTTATAATGT  130

Query  121  AATCGTAACAGCACATGCATTTATTATAAttttttttATAGTTATACCAATCATAATCGG  180
            ||||||||||||||||||||||||||||||||||||||||||||||||||||||||||||
Sbjct  131  AATCGTAACAGCACATGCATTTATTATAATTTTTTTTATAGTTATACCAATCATAATCGG  190

Query  181  AGGATTTGGAAATTGATTAGTCCCTATCATATTGGGTGCCCCAGATATAGCATTTCCACG  240
            ||||||||||||||||||||||||||||||||||||||||||||||||||||||||||||
Sbjct  191  AGGATTTGGAAATTGATTAGTCCCTATCATATTGGGTGCCCCAGATATAGCATTTCCACG  250

Query  241  AATAAATAATATAAGATTTTGACTTTTACCTCCTTCACTATTTTTATTAATTAACTCTTC  300
            ||||||||||||||||||||||||||||||||||||||||||||||||||||||||||||
Sbjct  251  AATAAATAATATAAGATTTTGACTTTTACCTCCTTCACTATTTTTATTAATTAACTCTTC  310

Query  301  ATTAATTGAATCTGGAGCTGGTACAGGATGAACAGTTTACCCTCCTCTATCTTCAAATTT  360
            ||||||||||||||||||||||||||||||||||||||||||||||||||||||||||||
Sbjct  311  ATTAATTGAATCTGGAGCTGGTACAGGATGAACAGTTTACCCTCCTCTATCTTCAAATTT  370

Query  361  ATCACATTATGGCCCTTCAGTAGATTTAGCTATTTTTTCTCTTCATCTTGCTGGTGCTTC  420
            |||||||||||| |||||||||||||||||||||||||||||||||||||||||||||||
Sbjct  371  ATCACATTATGGTCCTTCAGTAGATTTAGCTATTTTTTCTCTTCATCTTGCTGGTGCTTC  430

Query  421  TTCAATTTTAGGTGCAATTAACTTTATTACAACTATTGTAAACATACGATCAATTGGAAT  480
            ||||||||||||||||||||||||||||||||||||||||||||||||||||||||||||
Sbjct  431  TTCAATTTTAGGTGCAATTAACTTTATTACAACTATTGTAAACATACGATCAATTGGAAT  490

Query  481  AACAATAGAACGTATACCATTATTTGTTTGATCTGTTTTAATTACAGCTATTTTATTACT  540
            ||||||||||||||||||||||||||||||||||||||||||||||||||||||||||||
Sbjct  491  AACAATAGAACGTATACCATTATTTGTTTGATCTGTTTTAATTACAGCTATTTTATTACT  550

Query  541  TTTATCTTTACCTGTATTAGCAGGTGCCATTACAATATTGTTAACTGATCGAAACTTTAA  600
            ||||||||||||||||||||||||||||||||||||||||||||||||||||||||||||
Sbjct  551  TTTATCTTTACCTGTATTAGCAGGTGCCATTACAATATTGTTAACTGATCGAAACTTTAA  610

Query  601  TACATCATTTTTTGATCCTTCAGGGGGAGGAGATCCAATTTTATATCAACATTTATTT  658
            ||||||||||||||||||||||||||||||||||||||||||||||||||||||||||
Sbjct  611  TACATCATTTTTTGATCCTTCAGGGGGAGGAGATCCAATTTTATATCAACATTTATTT  668
```

```
>Rhipicephalus sanguineus sensu lato 'temperate lineage' isolate S32 cytochrome
c oxidase subunit I (COI) gene, partial cds; mitochondrial
Sequence ID: MF426008.1 Length: 690
Range 1: 14 to 671

Score:1210 bits(655), Expect:0.0,
Identities:657/658(99%),  Gaps:0/658(0%), Strand: Plus/Plus

Query  1    AACAATATATTTAATTTTTGGAGCATGATCAGGTATATTAGGACTTAGTATAAGAATATT  60
            ||||||||||||||||||||||||||||||||||||||||||||||||||||||||||||
```

```
Sbjct  14   AACAATATATTTAATTTTTGGAGCATGATCAGGTATATTAGGACTTAGTATAAGAATATT  73

Query  61   AATTCGTATAGAATTAGGACAACCTGGAACTTTAATTGGAAATGATCAGATTTATAATGT  120
            ||||||||||||||||||||||||||||||||||||||||||||||||||||||||||||
Sbjct  74   AATTCGTATAGAATTAGGACAACCTGGAACTTTAATTGGAAATGATCAGATTTATAATGT  133

Query  121  AATCGTAACAGCACATGCATTTATTATAAttttttttATAGTTATACCAATCATAATCGG  180
            ||||||||||||||||||||||||||||||||||||||||||||||||||||||||||||
Sbjct  134  AATCGTAACAGCACATGCATTTATTATAATTTTTTTTATAGTTATACCAATCATAATCGG  193

Query  181  AGGATTTGGAAATTGATTAGTCCCTATCATATTGGGTGCCCCAGATATAGCATTTCCACG  240
            ||||||||||||||||||||||||||||||||||||||||||||||||||||||||||||
Sbjct  194  AGGATTTGGAAATTGATTAGTCCCTATCATATTGGGTGCCCCAGATATAGCATTTCCACG  253

Query  241  AATAAATAATATAAGATTTTGACTTTTACCTCCTTCACTATTTTTATTAATTAACTCTTC  300
            ||||||||||||||||||||||||||||||||||||||||||||||||||||||||||||
Sbjct  254  AATAAATAATATAAGATTTTGACTTTTACCTCCTTCACTATTTTTATTAATTAACTCTTC  313

Query  301  ATTAATTGAATCTGGAGCTGGTACAGGATGAACAGTTTACCCTCCTCTATCTTCAAATTT  360
            ||||||||||||||||||||||||||||||||||||||||||||||||||||||||||||
Sbjct  314  ATTAATTGAATCTGGAGCTGGTACAGGATGAACAGTTTACCCTCCTCTATCTTCAAATTT  373

Query  361  ATCACATTATGGCCCTTCAGTAGATTTAGCTATTTTTTCTCTTCATCTTGCTGGTGCTTC  420
            ||||||||||||||||||||||||||||||||| ||||||||||||||||||||||||||
Sbjct  374  ATCACATTATGGCCCTTCAGTAGATTTAGCTATCTTTTCTCTTCATCTTGCTGGTGCTTC  433

Query  421  TTCAATTTTAGGTGCAATTAACTTTATTACAACTATTGTAAACATACGATCAATTGGAAT  480
            ||||||||||||||||||||||||||||||||||||||||||||||||||||||||||||
Sbjct  434  TTCAATTTTAGGTGCAATTAACTTTATTACAACTATTGTAAACATACGATCAATTGGAAT  493

Query  481  AACAATAGAACGTATACCATTATTTGTTTGATCTGTTTTAATTACAGCTATTTTATTACT  540
            ||||||||||||||||||||||||||||||||||||||||||||||||||||||||||||
Sbjct  494  AACAATAGAACGTATACCATTATTTGTTTGATCTGTTTTAATTACAGCTATTTTATTACT  553

Query  541  TTTATCTTTACCTGTATTAGCAGGTGCCATTACAATATTGTTAACTGATCGAAACTTTAA  600
            ||||||||||||||||||||||||||||||||||||||||||||||||||||||||||||
Sbjct  554  TTTATCTTTACCTGTATTAGCAGGTGCCATTACAATATTGTTAACTGATCGAAACTTTAA  613

Query  601  TACATCATTTTTTGATCCTTCAGGGGGAGGAGATCCAATTTTATATCAACATTTATTT  658
            ||||||||||||||||||||||||||||||||||||||||||||||||||||||||||
Sbjct  614  TACATCATTTTTTGATCCTTCAGGGGGAGGAGATCCAATTTTATATCAACATTTATTT  671
```

>Rhipicephalus sanguineus sensu lato 'temperate lineage' isolate S29 cytochrome c oxidase subunit I (COI) gene, partial cds; mitochondrial
Sequence ID: MF426007.1 Length: 686
Range 1: 14 to 671

Score:1210 bits(655), Expect:0.0,
Identities:657/658(99%), Gaps:0/658(0%), Strand: Plus/Plus

```
Query  1    AACAATATATTTAATTTTTGGAGCATGATCAGGTATATTAGGACTTAGTATAAGAATATT  60
```

```
             ||||||||||||||||||||||||||||||||||||||||||||||||||||||||||||
Sbjct  14    AACAATATATTTAATTTTTGGAGCATGATCAGGTATATTAGGACTTAGTATAAGAATATT  73

Query  61    AATTCGTATAGAATTAGGACAACCTGGAACTTTAATTGGAAATGATCAGATTTATAATGT  120
             ||||||||||||||||||||||||||||||||||||||||||||||||||||||||||||
Sbjct  74    AATTCGTATAGAATTAGGACAACCTGGAACTTTAATTGGAAATGATCAGATTTATAATGT  133

Query  121   AATCGTAACAGCACATGCATTTATTATAAtttttttATAGTTATACCAATCATAATCGG  180
             ||||||||||||||||||||||||||||||||||||||||||||||||||||||||||||
Sbjct  134   AATCGTAACAGCACATGCATTTATTATAATTTTTTTATAGTTATACCAATCATAATCGG  193

Query  181   AGGATTTGGAAATTGATTAGTCCCTATCATATTGGGTGCCCCAGATATAGCATTTCCACG  240
             ||||||||||||||||||||||||||||||||||||||||||||||||||||||||||||
Sbjct  194   AGGATTTGGAAATTGATTAGTCCCTATCATATTGGGTGCCCCAGATATAGCATTTCCACG  253

Query  241   AATAAATAATATAAGATTTTGACTTTTACCTCCTTCACTATTTTTATTAATTAACTCTTC  300
             ||||||||||||||||||||||||||||||||||||||||||||||||||||||||||||
Sbjct  254   AATAAATAATATAAGATTTTGACTTTTACCTCCTTCACTATTTTTATTAATTAACTCTTC  313

Query  301   ATTAATTGAATCTGGAGCTGGTACAGGATGAACAGTTTACCCTCCTCTATCTTCAAATTT  360
             ||||||||||||||||||||||||||||||||||||||||||||||||||||||||||||
Sbjct  314   ATTAATTGAATCTGGAGCTGGTACAGGATGAACAGTTTACCCTCCTCTATCTTCAAATTT  373

Query  361   ATCACATTATGGCCCTTCAGTAGATTTAGCTATTTTTTCTCTTCATCTTGCTGGTGCTTC  420
             |||||||||||| |||||||||||||||||||||||||||||||||||||||||||||||
Sbjct  374   ATCACATTATGGTCCTTCAGTAGATTTAGCTATTTTTTCTCTTCATCTTGCTGGTGCTTC  433

Query  421   TTCAATTTTAGGTGCAATTAACTTTATTACAACTATTGTAAACATACGATCAATTGGAAT  480
             ||||||||||||||||||||||||||||||||||||||||||||||||||||||||||||
Sbjct  434   TTCAATTTTAGGTGCAATTAACTTTATTACAACTATTGTAAACATACGATCAATTGGAAT  493

Query  481   AACAATAGAACGTATACCATTATTTGTTTGATCTGTTTTAATTACAGCTATTTTATTACT  540
             ||||||||||||||||||||||||||||||||||||||||||||||||||||||||||||
Sbjct  494   AACAATAGAACGTATACCATTATTTGTTTGATCTGTTTTAATTACAGCTATTTTATTACT  553

Query  541   TTTATCTTTACCTGTATTAGCAGGTGCCATTACAATATTGTTAACTGATCGAAACTTTAA  600
             ||||||||||||||||||||||||||||||||||||||||||||||||||||||||||||
Sbjct  554   TTTATCTTTACCTGTATTAGCAGGTGCCATTACAATATTGTTAACTGATCGAAACTTTAA  613

Query  601   TACATCATTTTTTGATCCTTCAGGGGGAGGAGATCCAATTTTATATCAACATTTATTT  658
             ||||||||||||||||||||||||||||||||||||||||||||||||||||||||||
Sbjct  614   TACATCATTTTTTGATCCTTCAGGGGGAGGAGATCCAATTTTATATCAACATTTATTT  671
```

>Rhipicephalus sanguineus sensu lato 'temperate lineage' isolate CR1570
cytochrome c oxidase subunit I (COI) gene, partial cds; mitochondrial
Sequence ID: MF426001.1 Length: 684
Range 1: 14 to 671

Score:1210 bits(655), Expect:0.0,
Identities:657/658(99%), Gaps:0/658(0%), Strand: Plus/Plus

```
Query  1    AACAATATATTTAATTTTTGGAGCATGATCAGGTATATTAGGACTTAGTATAAGAATATT  60
            ||||||||||||||||||||||||||||||||||||||||||||||||||||||||||||
Sbjct  14   AACAATATATTTAATTTTTGGAGCATGATCAGGTATATTAGGACTTAGTATAAGAATATT  73

Query  61   AATTCGTATAGAATTAGGACAACCTGGAACTTTAATTGGAAATGATCAGATTTATAATGT  120
            ||||||||||||||||||||||||||||||||||||||||||||||||||||||||||||
Sbjct  74   AATTCGTATAGAATTAGGACAACCTGGAACTTTAATTGGAAATGATCAGATTTATAATGT  133

Query  121  AATCGTAACAGCACATGCATTTATTATAAttttttttATAGTTATACCAATCATAATCGG  180
            ||||||||||||||||||||||||||||||||||||||||||||||||||||||||||||
Sbjct  134  AATCGTAACAGCACATGCATTTATTATAATTTTTTTTATAGTTATACCAATCATAATCGG  193

Query  181  AGGATTTGGAAATTGATTAGTCCCTATCATATTGGGTGCCCCAGATATAGCATTTCCACG  240
            ||||||||||||||||||||||||||||||||||||||||||||||||||||||||||||
Sbjct  194  AGGATTTGGAAATTGATTAGTCCCTATCATATTGGGTGCCCCAGATATAGCATTTCCACG  253

Query  241  AATAAATAATATAAGATTTTGACTTTTACCTCCTTCACTATTTTTATTAATTAACTCTTC  300
            ||||||||||||||||||||||||||||||||||||||||||||||||||||||||||||
Sbjct  254  AATAAATAATATAAGATTTTGACTTTTACCTCCTTCACTATTTTTATTAATTAACTCTTC  313

Query  301  ATTAATTGAATCTGGAGCTGGTACAGGATGAACAGTTTACCCTCCTCTATCTTCAAATTT  360
            ||||||||||||||||||||||||||||||||||||||||||||||||||||||||||||
Sbjct  314  ATTAATTGAATCTGGAGCTGGTACAGGATGAACAGTTTACCCTCCTCTATCTTCAAATTT  373

Query  361  ATCACATTATGGCCCTTCAGTAGATTTAGCTATTTTTTCTCTTCATCTTGCTGGTGCTTC  420
            ||||||||||||||||||||||||||||||||| ||||||||||||||||||||||||||
Sbjct  374  ATCACATTATGGCCCTTCAGTAGATTTAGCTATCTTTTCTCTTCATCTTGCTGGTGCTTC  433

Query  421  TTCAATTTTAGGTGCAATTAACTTTATTACAACTATTGTAAACATACGATCAATTGGAAT  480
            ||||||||||||||||||||||||||||||||||||||||||||||||||||||||||||
Sbjct  434  TTCAATTTTAGGTGCAATTAACTTTATTACAACTATTGTAAACATACGATCAATTGGAAT  493

Query  481  AACAATAGAACGTATACCATTATTTGTTTGATCTGTTTTAATTACAGCTATTTTATTACT  540
            ||||||||||||||||||||||||||||||||||||||||||||||||||||||||||||
Sbjct  494  AACAATAGAACGTATACCATTATTTGTTTGATCTGTTTTAATTACAGCTATTTTATTACT  553

Query  541  TTTATCTTTACCTGTATTAGCAGGTGCCATTACAATATTGTTAACTGATCGAAACTTTAA  600
            ||||||||||||||||||||||||||||||||||||||||||||||||||||||||||||
Sbjct  554  TTTATCTTTACCTGTATTAGCAGGTGCCATTACAATATTGTTAACTGATCGAAACTTTAA  613

Query  601  TACATCATTTTTTGATCCTTCAGGGGGAGGAGATCCAATTTTATATCAACATTTATTT  658
            ||||||||||||||||||||||||||||||||||||||||||||||||||||||||||
Sbjct  614  TACATCATTTTTTGATCCTTCAGGGGGAGGAGATCCAATTTTATATCAACATTTATTT  671
```

```
>Rhipicephalus sanguineus sensu lato 'temperate lineage' isolate CR1551
cytochrome c oxidase subunit I (COI) gene, partial cds; mitochondrial
Sequence ID: MF425997.1 Length: 695
Range 1: 22 to 679

Score:1210 bits(655), Expect:0.0,
Identities:657/658(99%),  Gaps:0/658(0%), Strand: Plus/Plus
```

```
Query  1    AACAATATATTTAATTTTTGGAGCATGATCAGGTATATTAGGACTTAGTATAAGAATATT  60
            ||||||||||||||||||||||||||||||||||||||||||||||||||||||||||||
Sbjct  22   AACAATATATTTAATTTTTGGAGCATGATCAGGTATATTAGGACTTAGTATAAGAATATT  81

Query  61   AATTCGTATAGAATTAGGACAACCTGGAACTTTAATTGGAAATGATCAGATTTATAATGT  120
            ||||||||||||||||||||||||||||||||||||||||||||||||||||||||||||
Sbjct  82   AATTCGTATAGAATTAGGACAACCTGGAACTTTAATTGGAAATGATCAGATTTATAATGT  141

Query  121  AATCGTAACAGCACATGCATTTATTATAAttttttttATAGTTATACCAATCATAATCGG  180
            ||||||||||||||||||||||||||||||||||||||||||||||||||||||||||||
Sbjct  142  AATCGTAACAGCACATGCATTTATTATAATTTTTTTTATAGTTATACCAATCATAATCGG  201

Query  181  AGGATTTGGAAATTGATTAGTCCCTATCATATTGGGTGCCCCAGATATAGCATTTCCACG  240
            ||||||||||||||||||||||||||||||||||||||||||||||||||||||||||||
Sbjct  202  AGGATTTGGAAATTGATTAGTCCCTATCATATTGGGTGCCCCAGATATAGCATTTCCACG  261

Query  241  AATAAATAATATAAGATTTTGACTTTTACCTCCTTCACTATTTTTATTAATTAACTCTTC  300
            ||||||||||||||||||||||||||||||||||||||||||||||||||||||||||||
Sbjct  262  AATAAATAATATAAGATTTTGACTTTTACCTCCTTCACTATTTTTATTAATTAACTCTTC  321

Query  301  ATTAATTGAATCTGGAGCTGGTACAGGATGAACAGTTTACCCTCCTCTATCTTCAAATTT  360
            ||||||||||||||||||||||||||||||||||||||||||||||||||||||||||||
Sbjct  322  ATTAATTGAATCTGGAGCTGGTACAGGATGAACAGTTTACCCTCCTCTATCTTCAAATTT  381

Query  361  ATCACATTATGGCCCTTCAGTAGATTTAGCTATTTTTTCTCTTCATCTTGCTGGTGCTTC  420
            ||||||||||||||||||||||||||||||||| ||||||||||||||||||||||||||
Sbjct  382  ATCACATTATGGCCCTTCAGTAGATTTAGCTATCTTTTCTCTTCATCTTGCTGGTGCTTC  441

Query  421  TTCAATTTTAGGTGCAATTAACTTTATTACAACTATTGTAAACATACGATCAATTGGAAT  480
            ||||||||||||||||||||||||||||||||||||||||||||||||||||||||||||
Sbjct  442  TTCAATTTTAGGTGCAATTAACTTTATTACAACTATTGTAAACATACGATCAATTGGAAT  501

Query  481  AACAATAGAACGTATACCATTATTTGTTTGATCTGTTTTAATTACAGCTATTTTATTACT  540
            ||||||||||||||||||||||||||||||||||||||||||||||||||||||||||||
Sbjct  502  AACAATAGAACGTATACCATTATTTGTTTGATCTGTTTTAATTACAGCTATTTTATTACT  561

Query  541  TTTATCTTTACCTGTATTAGCAGGTGCCATTACAATATTGTTAACTGATCGAAACTTTAA  600
            ||||||||||||||||||||||||||||||||||||||||||||||||||||||||||||
Sbjct  562  TTTATCTTTACCTGTATTAGCAGGTGCCATTACAATATTGTTAACTGATCGAAACTTTAA  621

Query  601  TACATCATTTTTTGATCCTTCAGGGGGAGGAGATCCAATTTTATATCAACATTTATTT  658
            ||||||||||||||||||||||||||||||||||||||||||||||||||||||||||
Sbjct  622  TACATCATTTTTTGATCCTTCAGGGGGAGGAGATCCAATTTTATATCAACATTTATTT  679
```

>Rhipicephalus sanguineus voucher CROBB813 cytochrome oxidase subunit 1 (COI) gene, partial cds; mitochondrial
Sequence ID: MZ305546.1 Length: 658
Range 1: 1 to 658

Score:1210 bits(655), Expect:0.0,

```
Identities:657/658(99%),  Gaps:0/658(0%), Strand: Plus/Plus

Query  1    AACAATATATTTAATTTTTGGAGCATGATCAGGTATATTAGGACTTAGTATAAGAATATT  60
            ||||||||||||||||||||||||||||||||||||||||||||||||||||||||||||
Sbjct  1    AACAATATATTTAATTTTTGGAGCATGATCAGGTATATTAGGACTTAGTATAAGAATATT  60

Query  61   AATTCGTATAGAATTAGGACAACCTGGAACTTTAATTGGAAATGATCAGATTTATAATGT  120
            |||||||||||| |||||||||||||||||||||||||||||||||||||||||||||||
Sbjct  61   AATTCGTATAGAGTTAGGACAACCTGGAACTTTAATTGGAAATGATCAGATTTATAATGT  120

Query  121  AATCGTAACAGCACATGCATTTATTATAAttttttttATAGTTATACCAATCATAATCGG  180
            ||||||||||||||||||||||||||||||||||||||||||||||||||||||||||||
Sbjct  121  AATCGTAACAGCACATGCATTTATTATAATTTTTTTTATAGTTATACCAATCATAATCGG  180

Query  181  AGGATTTGGAAATTGATTAGTCCCTATCATATTGGGTGCCCCAGATATAGCATTTCCACG  240
            ||||||||||||||||||||||||||||||||||||||||||||||||||||||||||||
Sbjct  181  AGGATTTGGAAATTGATTAGTCCCTATCATATTGGGTGCCCCAGATATAGCATTTCCACG  240

Query  241  AATAAATAATATAAGATTTTGACTTTTACCTCCTTCACTATTTTTATTAATTAACTCTTC  300
            ||||||||||||||||||||||||||||||||||||||||||||||||||||||||||||
Sbjct  241  AATAAATAATATAAGATTTTGACTTTTACCTCCTTCACTATTTTTATTAATTAACTCTTC  300

Query  301  ATTAATTGAATCTGGAGCTGGTACAGGATGAACAGTTTACCCTCCTCTATCTTCAAATTT  360
            ||||||||||||||||||||||||||||||||||||||||||||||||||||||||||||
Sbjct  301  ATTAATTGAATCTGGAGCTGGTACAGGATGAACAGTTTACCCTCCTCTATCTTCAAATTT  360

Query  361  ATCACATTATGGCCCTTCAGTAGATTTAGCTATTTTTTCTCTTCATCTTGCTGGTGCTTC  420
            ||||||||||||||||||||||||||||||||||||||||||||||||||||||||||||
Sbjct  361  ATCACATTATGGCCCTTCAGTAGATTTAGCTATTTTTTCTCTTCATCTTGCTGGTGCTTC  420

Query  421  TTCAATTTTAGGTGCAATTAACTTTATTACAACTATTGTAAACATACGATCAATTGGAAT  480
            ||||||||||||||||||||||||||||||||||||||||||||||||||||||||||||
Sbjct  421  TTCAATTTTAGGTGCAATTAACTTTATTACAACTATTGTAAACATACGATCAATTGGAAT  480

Query  481  AACAATAGAACGTATACCATTATTTGTTTGATCTGTTTTAATTACAGCTATTTTATTACT  540
            ||||||||||||||||||||||||||||||||||||||||||||||||||||||||||||
Sbjct  481  AACAATAGAACGTATACCATTATTTGTTTGATCTGTTTTAATTACAGCTATTTTATTACT  540

Query  541  TTTATCTTTACCTGTATTAGCAGGTGCCATTACAATATTGTTAACTGATCGAAACTTTAA  600
            ||||||||||||||||||||||||||||||||||||||||||||||||||||||||||||
Sbjct  541  TTTATCTTTACCTGTATTAGCAGGTGCCATTACAATATTGTTAACTGATCGAAACTTTAA  600

Query  601  TACATCATTTTTTGATCCTTCAGGGGGAGGAGATCCAATTTTATATCAACATTTATTT  658
            ||||||||||||||||||||||||||||||||||||||||||||||||||||||||||
Sbjct  601  TACATCATTTTTTGATCCTTCAGGGGGAGGAGATCCAATTTTATATCAACATTTATTT  658
```

>Rhipicephalus sanguineus voucher CROBB810 cytochrome oxidase subunit 1 (COI) gene, partial cds; mitochondrial
Sequence ID: MZ305545.1 Length: 658
Range 1: 1 to 658

```
Score:1210 bits(655), Expect:0.0,
Identities:657/658(99%),  Gaps:0/658(0%), Strand: Plus/Plus

Query  1    AACAATATATTTAATTTTTGGAGCATGATCAGGTATATTAGGACTTAGTATAAGAATATT  60
            ||||||||||||||||||||||||||||||||||||||||||||||||||||||||||||
Sbjct  1    AACAATATATTTAATTTTTGGAGCATGATCAGGTATATTAGGACTTAGTATAAGAATATT  60

Query  61   AATTCGTATAGAATTAGGACAACCTGGAACTTTAATTGGAAATGATCAGATTTATAATGT  120
            ||||||||||||||||||||||||||||||||||||||||||||||||||||||||||||
Sbjct  61   AATTCGTATAGAATTAGGACAACCTGGAACTTTAATTGGAAATGATCAGATTTATAATGT  120

Query  121  AATCGTAACAGCACATGCATTTATTATAAttttttttATAGTTATACCAATCATAATCGG  180
            ||||||||||||||||||||||||||||||||||||||||||||||||||||||||||||
Sbjct  121  AATCGTAACAGCACATGCATTTATTATAATTTTTTTTATAGTTATACCAATCATAATCGG  180

Query  181  AGGATTTGGAAATTGATTAGTCCCTATCATATTGGGTGCCCCAGATATAGCATTTCCACG  240
            ||||||||||||||||||||||||||||||||||||||||||||||||||||||||||||
Sbjct  181  AGGATTTGGAAATTGATTAGTCCCTATCATATTGGGTGCCCCAGATATAGCATTTCCACG  240

Query  241  AATAAATAATATAAGATTTTGACTTTTACCTCCTTCACTATTTTTATTAATTAACTCTTC  300
            |||||||||||||||||||||||||||||||||||||||||||||||||||||| |||||
Sbjct  241  AATAAATAATATAAGATTTTGACTTTTACCTCCTTCACTATTTTTATTAATTAATTCTTC  300

Query  301  ATTAATTGAATCTGGAGCTGGTACAGGATGAACAGTTTACCCTCCTCTATCTTCAAATTT  360
            ||||||||||||||||||||||||||||||||||||||||||||||||||||||||||||
Sbjct  301  ATTAATTGAATCTGGAGCTGGTACAGGATGAACAGTTTACCCTCCTCTATCTTCAAATTT  360

Query  361  ATCACATTATGGCCCTTCAGTAGATTTAGCTATTTTTTCTCTTCATCTTGCTGGTGCTTC  420
            ||||||||||||||||||||||||||||||||||||||||||||||||||||||||||||
Sbjct  361  ATCACATTATGGCCCTTCAGTAGATTTAGCTATTTTTTCTCTTCATCTTGCTGGTGCTTC  420

Query  421  TTCAATTTTAGGTGCAATTAACTTTATTACAACTATTGTAAACATACGATCAATTGGAAT  480
            ||||||||||||||||||||||||||||||||||||||||||||||||||||||||||||
Sbjct  421  TTCAATTTTAGGTGCAATTAACTTTATTACAACTATTGTAAACATACGATCAATTGGAAT  480

Query  481  AACAATAGAACGTATACCATTATTTGTTTGATCTGTTTTAATTACAGCTATTTTATTACT  540
            ||||||||||||||||||||||||||||||||||||||||||||||||||||||||||||
Sbjct  481  AACAATAGAACGTATACCATTATTTGTTTGATCTGTTTTAATTACAGCTATTTTATTACT  540

Query  541  TTTATCTTTACCTGTATTAGCAGGTGCCATTACAATATTGTTAACTGATCGAAACTTTAA  600
            ||||||||||||||||||||||||||||||||||||||||||||||||||||||||||||
Sbjct  541  TTTATCTTTACCTGTATTAGCAGGTGCCATTACAATATTGTTAACTGATCGAAACTTTAA  600

Query  601  TACATCATTTTTTGATCCTTCAGGGGGAGGAGATCCAATTTTATATCAACATTTATTT  658
            ||||||||||||||||||||||||||||||||||||||||||||||||||||||||||
Sbjct  601  TACATCATTTTTTGATCCTTCAGGGGGAGGAGATCCAATTTTATATCAACATTTATTT  658
```

>Rhipicephalus sanguineus isolate 5MSK cytochrome c oxidase subunit I (COX1) gene, partial cds; mitochondrial
Sequence ID: MW152145.1 Length: 676
Range 1: 1 to 658

```
Score:1210 bits(655), Expect:0.0,
Identities:657/658(99%),  Gaps:0/658(0%), Strand: Plus/Plus

Query  1    AACAATATATTTAATTTTTGGAGCATGATCAGGTATATTAGGACTTAGTATAAGAATATT  60
            ||||||||||||||||||||||||||||||||||||||||||||||||||||||||||||
Sbjct  1    AACAATATATTTAATTTTTGGAGCATGATCAGGTATATTAGGACTTAGTATAAGAATATT  60

Query  61   AATTCGTATAGAATTAGGACAACCTGGAACTTTAATTGGAAATGATCAGATTTATAATGT  120
            ||||||||||||||||||||||||||||||||||||||||||||||||||||||||||||
Sbjct  61   AATTCGTATAGAATTAGGACAACCTGGAACTTTAATTGGAAATGATCAGATTTATAATGT  120

Query  121  AATCGTAACAGCACATGCATTTATTATAAttttttttATAGTTATACCAATCATAATCGG  180
            ||||||||||||||||||||||||||||||||||||||||||||||||||||||||||||
Sbjct  121  AATCGTAACAGCACATGCATTTATTATAATTTTTTTTATAGTTATACCAATCATAATCGG  180

Query  181  AGGATTTGGAAATTGATTAGTCCCTATCATATTGGGTGCCCCAGATATAGCATTTCCACG  240
            ||||||||||||||||||||||||||||||||||||||||||||||||||||||||||||
Sbjct  181  AGGATTTGGAAATTGATTAGTCCCTATCATATTGGGTGCCCCAGATATAGCATTTCCACG  240

Query  241  AATAAATAATATAAGATTTTGACTTTTACCTCCTTCACTATTTTTATTAATTAACTCTTC  300
            |||||||||||||||||||||||||||||||||||||||||||||||||||||| |||||
Sbjct  241  AATAAATAATATAAGATTTTGACTTTTACCTCCTTCACTATTTTTATTAATTAATTCTTC  300

Query  301  ATTAATTGAATCTGGAGCTGGTACAGGATGAACAGTTTACCCTCCTCTATCTTCAAATTT  360
            ||||||||||||||||||||||||||||||||||||||||||||||||||||||||||||
Sbjct  301  ATTAATTGAATCTGGAGCTGGTACAGGATGAACAGTTTACCCTCCTCTATCTTCAAATTT  360

Query  361  ATCACATTATGGCCCTTCAGTAGATTTAGCTATTTTTTCTCTTCATCTTGCTGGTGCTTC  420
            ||||||||||||||||||||||||||||||||||||||||||||||||||||||||||||
Sbjct  361  ATCACATTATGGCCCTTCAGTAGATTTAGCTATTTTTTCTCTTCATCTTGCTGGTGCTTC  420

Query  421  TTCAATTTTAGGTGCAATTAACTTTATTACAACTATTGTAAACATACGATCAATTGGAAT  480
            ||||||||||||||||||||||||||||||||||||||||||||||||||||||||||||
Sbjct  421  TTCAATTTTAGGTGCAATTAACTTTATTACAACTATTGTAAACATACGATCAATTGGAAT  480

Query  481  AACAATAGAACGTATACCATTATTTGTTTGATCTGTTTTAATTACAGCTATTTTATTACT  540
            ||||||||||||||||||||||||||||||||||||||||||||||||||||||||||||
Sbjct  481  AACAATAGAACGTATACCATTATTTGTTTGATCTGTTTTAATTACAGCTATTTTATTACT  540

Query  541  TTTATCTTTACCTGTATTAGCAGGTGCCATTACAATATTGTTAACTGATCGAAACTTTAA  600
            ||||||||||||||||||||||||||||||||||||||||||||||||||||||||||||
Sbjct  541  TTTATCTTTACCTGTATTAGCAGGTGCCATTACAATATTGTTAACTGATCGAAACTTTAA  600

Query  601  TACATCATTTTTTGATCCTTCAGGGGGAGGAGATCCAATTTTATATCAACATTTATTT  658
            ||||||||||||||||||||||||||||||||||||||||||||||||||||||||||
Sbjct  601  TACATCATTTTTTGATCCTTCAGGGGGAGGAGATCCAATTTTATATCAACATTTATTT  658
```

>Rhipicephalus sanguineus isolate 1MSK cytochrome c oxidase subunit I (COX1) gene, partial cds; mitochondrial
Sequence ID: MW152144.1 Length: 675

```
Range 1: 1 to 658

Score:1210 bits(655), Expect:0.0,
Identities:657/658(99%),  Gaps:0/658(0%), Strand: Plus/Plus

Query  1    AACAATATATTTAATTTTTGGAGCATGATCAGGTATATTAGGACTTAGTATAAGAATATT  60
            ||||||||||||||||||||||||||||||||||||||||||||||||||||||||||||
Sbjct  1    AACAATATATTTAATTTTTGGAGCATGATCAGGTATATTAGGACTTAGTATAAGAATATT  60

Query  61   AATTCGTATAGAATTAGGACAACCTGGAACTTTAATTGGAAATGATCAGATTTATAATGT  120
            ||||||||||||||||||||||||||||||||||||||||||||||||||||||||||||
Sbjct  61   AATTCGTATAGAATTAGGACAACCTGGAACTTTAATTGGAAATGATCAGATTTATAATGT  120

Query  121  AATCGTAACAGCACATGCATTTATTATAAtttttttATAGTTATACCAATCATAATCGG  180
            ||||||||||||||||||||||||||||||||||||||||||||||||||||||||||||
Sbjct  121  AATCGTAACAGCACATGCATTTATTATAATTTTTTTATAGTTATACCAATCATAATCGG  180

Query  181  AGGATTTGGAAATTGATTAGTCCCTATCATATTGGGTGCCCCAGATATAGCATTTCCACG  240
            ||||||||||||||||||||||||||||||||||||||||||||||||||||||||||||
Sbjct  181  AGGATTTGGAAATTGATTAGTCCCTATCATATTGGGTGCCCCAGATATAGCATTTCCACG  240

Query  241  AATAAATAATATAAGATTTTGACTTTTACCTCCTTCACTATTTTTATTAATTAACTCTTC  300
            |||||||||||||||||||||||||||||||||||||||||||||||||||||| |||||
Sbjct  241  AATAAATAATATAAGATTTTGACTTTTACCTCCTTCACTATTTTTATTAATTAATTCTTC  300

Query  301  ATTAATTGAATCTGGAGCTGGTACAGGATGAACAGTTTACCCTCCTCTATCTTCAAATTT  360
            ||||||||||||||||||||||||||||||||||||||||||||||||||||||||||||
Sbjct  301  ATTAATTGAATCTGGAGCTGGTACAGGATGAACAGTTTACCCTCCTCTATCTTCAAATTT  360

Query  361  ATCACATTATGGCCCTTCAGTAGATTTAGCTATTTTTTCTCTTCATCTTGCTGGTGCTTC  420
            ||||||||||||||||||||||||||||||||||||||||||||||||||||||||||||
Sbjct  361  ATCACATTATGGCCCTTCAGTAGATTTAGCTATTTTTTCTCTTCATCTTGCTGGTGCTTC  420

Query  421  TTCAATTTTAGGTGCAATTAACTTTATTACAACTATTGTAAACATACGATCAATTGGAAT  480
            ||||||||||||||||||||||||||||||||||||||||||||||||||||||||||||
Sbjct  421  TTCAATTTTAGGTGCAATTAACTTTATTACAACTATTGTAAACATACGATCAATTGGAAT  480

Query  481  AACAATAGAACGTATACCATTATTTGTTTGATCTGTTTTAATTACAGCTATTTTATTACT  540
            ||||||||||||||||||||||||||||||||||||||||||||||||||||||||||||
Sbjct  481  AACAATAGAACGTATACCATTATTTGTTTGATCTGTTTTAATTACAGCTATTTTATTACT  540

Query  541  TTTATCTTTACCTGTATTAGCAGGTGCCATTACAATATTGTTAACTGATCGAAACTTTAA  600
            ||||||||||||||||||||||||||||||||||||||||||||||||||||||||||||
Sbjct  541  TTTATCTTTACCTGTATTAGCAGGTGCCATTACAATATTGTTAACTGATCGAAACTTTAA  600

Query  601  TACATCATTTTTTGATCCTTCAGGGGGAGGAGATCCAATTTTATATCAACATTTATTT  658
            ||||||||||||||||||||||||||||||||||||||||||||||||||||||||||
Sbjct  601  TACATCATTTTTTGATCCTTCAGGGGGAGGAGATCCAATTTTATATCAACATTTATTT  658
```

>Rhipicephalus sanguineus isolate RSN2SK cytochrome c oxidase subunit I (COX1) gene, partial cds; mitochondrial

```
Sequence ID: MW152143.1 Length: 676
Range 1: 1 to 658

Score:1210 bits(655), Expect:0.0,
Identities:657/658(99%),  Gaps:0/658(0%), Strand: Plus/Plus

Query  1    AACAATATATTTAATTTTTGGAGCATGATCAGGTATATTAGGACTTAGTATAAGAATATT  60
            ||||||||||||||||||||||||||||||||||||||||||||||||||||||||||||
Sbjct  1    AACAATATATTTAATTTTTGGAGCATGATCAGGTATATTAGGACTTAGTATAAGAATATT  60

Query  61   AATTCGTATAGAATTAGGACAACCTGGAACTTTAATTGGAAATGATCAGATTTATAATGT  120
            ||||||||||||||||||||||||||||||||||||||||||||||||||||||||||||
Sbjct  61   AATTCGTATAGAATTAGGACAACCTGGAACTTTAATTGGAAATGATCAGATTTATAATGT  120

Query  121  AATCGTAACAGCACATGCATTTATTATAAtttttttATAGTTATACCAATCATAATCGG  180
            ||||||||||||||||||||||||||||||||||||||||||||||||||||||||||||
Sbjct  121  AATCGTAACAGCACATGCATTTATTATAATTTTTTTATAGTTATACCAATCATAATCGG  180

Query  181  AGGATTTGGAAATTGATTAGTCCCTATCATATTGGGTGCCCCAGATATAGCATTTCCACG  240
            ||||||||||||||||||||||||||||||||||||||||||||||||||||||||||||
Sbjct  181  AGGATTTGGAAATTGATTAGTCCCTATCATATTGGGTGCCCCAGATATAGCATTTCCACG  240

Query  241  AATAAATAATATAAGATTTTGACTTTTACCTCCTTCACTATTTTTATTAATTAACTCTTC  300
            |||||||||||||||||||||||||||||||||||||||||||||||||||||| |||||
Sbjct  241  AATAAATAATATAAGATTTTGACTTTTACCTCCTTCACTATTTTTATTAATTAATTCTTC  300

Query  301  ATTAATTGAATCTGGAGCTGGTACAGGATGAACAGTTTACCCTCCTCTATCTTCAAATTT  360
            ||||||||||||||||||||||||||||||||||||||||||||||||||||||||||||
Sbjct  301  ATTAATTGAATCTGGAGCTGGTACAGGATGAACAGTTTACCCTCCTCTATCTTCAAATTT  360

Query  361  ATCACATTATGGCCCTTCAGTAGATTTAGCTATTTTTTCTCTTCATCTTGCTGGTGCTTC  420
            ||||||||||||||||||||||||||||||||||||||||||||||||||||||||||||
Sbjct  361  ATCACATTATGGCCCTTCAGTAGATTTAGCTATTTTTTCTCTTCATCTTGCTGGTGCTTC  420

Query  421  TTCAATTTTAGGTGCAATTAACTTTATTACAACTATTGTAAACATACGATCAATTGGAAT  480
            ||||||||||||||||||||||||||||||||||||||||||||||||||||||||||||
Sbjct  421  TTCAATTTTAGGTGCAATTAACTTTATTACAACTATTGTAAACATACGATCAATTGGAAT  480

Query  481  AACAATAGAACGTATACCATTATTTGTTTGATCTGTTTTAATTACAGCTATTTTATTACT  540
            ||||||||||||||||||||||||||||||||||||||||||||||||||||||||||||
Sbjct  481  AACAATAGAACGTATACCATTATTTGTTTGATCTGTTTTAATTACAGCTATTTTATTACT  540

Query  541  TTTATCTTTACCTGTATTAGCAGGTGCCATTACAATATTGTTAACTGATCGAAACTTTAA  600
            ||||||||||||||||||||||||||||||||||||||||||||||||||||||||||||
Sbjct  541  TTTATCTTTACCTGTATTAGCAGGTGCCATTACAATATTGTTAACTGATCGAAACTTTAA  600

Query  601  TACATCATTTTTTGATCCTTCAGGGGGAGGAGATCCAATTTTATATCAACATTTATTT  658
            ||||||||||||||||||||||||||||||||||||||||||||||||||||||||||
Sbjct  601  TACATCATTTTTTGATCCTTCAGGGGGAGGAGATCCAATTTTATATCAACATTTATTT  658
```

>Rhipicephalus sanguineus isolate RSN11SK cytochrome c oxidase subunit I (COX1)

gene, partial cds; mitochondrial
Sequence ID: MW152142.1 Length: 669
Range 1: 1 to 658

Score:1210 bits(655), Expect:0.0,
Identities:657/658(99%), Gaps:0/658(0%), Strand: Plus/Plus

```
Query  1    AACAATATATTTAATTTTTGGAGCATGATCAGGTATATTAGGACTTAGTATAAGAATATT  60
            ||||||||||||||||||||||||||||||||||||||||||||||||||||||||||||
Sbjct  1    AACAATATATTTAATTTTTGGAGCATGATCAGGTATATTAGGACTTAGTATAAGAATATT  60

Query  61   AATTCGTATAGAATTAGGACAACCTGGAACTTTAATTGGAAATGATCAGATTTATAATGT  120
            ||||||||||||||||||||||||||||||||||||||||||||||||||||||||||||
Sbjct  61   AATTCGTATAGAATTAGGACAACCTGGAACTTTAATTGGAAATGATCAGATTTATAATGT  120

Query  121  AATCGTAACAGCACATGCATTTATTATAAtttttttATAGTTATACCAATCATAATCGG  180
            ||||||||||||||||||||||||||||||||||||||||||||||||||||||||||||
Sbjct  121  AATCGTAACAGCACATGCATTTATTATAATTTTTTTATAGTTATACCAATCATAATCGG  180

Query  181  AGGATTTGGAAATTGATTAGTCCCTATCATATTGGGTGCCCCAGATATAGCATTTCCACG  240
            ||||||||||||||||||||||||||||||||||||||||||||||||||||||||||||
Sbjct  181  AGGATTTGGAAATTGATTAGTCCCTATCATATTGGGTGCCCCAGATATAGCATTTCCACG  240

Query  241  AATAAATAATATAAGATTTTGACTTTTACCTCCTTCACTATTTTTATTAATTAACTCTTC  300
            ||||||||||||||||||||||||||||||||||||||||||||||||||||||| |||||
Sbjct  241  AATAAATAATATAAGATTTTGACTTTTACCTCCTTCACTATTTTTATTAATTAATTCTTC  300

Query  301  ATTAATTGAATCTGGAGCTGGTACAGGATGAACAGTTTACCCTCCTCTATCTTCAAATTT  360
            ||||||||||||||||||||||||||||||||||||||||||||||||||||||||||||
Sbjct  301  ATTAATTGAATCTGGAGCTGGTACAGGATGAACAGTTTACCCTCCTCTATCTTCAAATTT  360

Query  361  ATCACATTATGGCCCTTCAGTAGATTTAGCTATTTTTTCTCTTCATCTTGCTGGTGCTTC  420
            ||||||||||||||||||||||||||||||||||||||||||||||||||||||||||||
Sbjct  361  ATCACATTATGGCCCTTCAGTAGATTTAGCTATTTTTTCTCTTCATCTTGCTGGTGCTTC  420

Query  421  TTCAATTTTAGGTGCAATTAACTTTATTACAACTATTGTAAACATACGATCAATTGGAAT  480
            ||||||||||||||||||||||||||||||||||||||||||||||||||||||||||||
Sbjct  421  TTCAATTTTAGGTGCAATTAACTTTATTACAACTATTGTAAACATACGATCAATTGGAAT  480

Query  481  AACAATAGAACGTATACCATTATTTGTTTGATCTGTTTTAATTACAGCTATTTTATTACT  540
            ||||||||||||||||||||||||||||||||||||||||||||||||||||||||||||
Sbjct  481  AACAATAGAACGTATACCATTATTTGTTTGATCTGTTTTAATTACAGCTATTTTATTACT  540

Query  541  TTTATCTTTACCTGTATTAGCAGGTGCCATTACAATATTGTTAACTGATCGAAACTTTAA  600
            ||||||||||||||||||||||||||||||||||||||||||||||||||||||||||||
Sbjct  541  TTTATCTTTACCTGTATTAGCAGGTGCCATTACAATATTGTTAACTGATCGAAACTTTAA  600

Query  601  TACATCATTTTTTGATCCTTCAGGGGGAGGAGATCCAATTTTATATCAACATTTATTT  658
            ||||||||||||||||||||||||||||||||||||||||||||||||||||||||||
Sbjct  601  TACATCATTTTTTGATCCTTCAGGGGGAGGAGATCCAATTTTATATCAACATTTATTT  658
```

```
>Rhipicephalus sanguineus sensu lato 'temperate lineage' isolate S48 cytochrome
c oxidase subunit I (COI) gene, partial cds; mitochondrial
Sequence ID: MF426015.1 Length: 692
Range 1: 19 to 676

Score:1205 bits(652), Expect:0.0,
Identities:656/658(99%),  Gaps:0/658(0%), Strand: Plus/Plus

Query  1    AACAATATATTTAATTTTTGGAGCATGATCAGGTATATTAGGACTTAGTATAAGAATATT  60
            ||||||||||||||||||||||||||||||||||||||||||||||||||||||||||||
Sbjct  19   AACAATATATTTAATTTTTGGAGCATGATCAGGTATATTAGGACTTAGTATAAGAATATT  78

Query  61   AATTCGTATAGAATTAGGACAACCTGGAACTTTAATTGGAAATGATCAGATTTATAATGT  120
            ||||||||||||||||||||||||||||||||||||||||||||||||||||||||||||
Sbjct  79   AATTCGTATAGAATTAGGACAACCTGGAACTTTAATTGGAAATGATCAGATTTATAATGT  138

Query  121  AATCGTAACAGCACATGCATTTATTATAAtttttttATAGTTATACCAATCATAATCGG  180
            ||||||||||||||||||||||||||||||||||||||||||||||||||||||||||||
Sbjct  139  AATCGTAACAGCACATGCATTTATTATAATTTTTTTATAGTTATACCAATCATAATCGG  198

Query  181  AGGATTTGGAAATTGATTAGTCCCTATCATATTGGGTGCCCCAGATATAGCATTTCCACG  240
            ||||||||||||||||||||||||||||||||||||||||||||||||||||||||||||
Sbjct  199  AGGATTTGGAAATTGATTAGTCCCTATCATATTGGGTGCCCCAGATATAGCATTTCCACG  258

Query  241  AATAAATAATATAAGATTTTGACTTTTACCTCCTTCACTATTTTTATTAATTAACTCTTC  300
            ||||||||||||||||||||||||||||||||||||||||||||||||||||||||||||
Sbjct  259  AATAAATAATATAAGATTTTGACTTTTACCTCCTTCACTATTTTTATTAATTAACTCTTC  318

Query  301  ATTAATTGAATCTGGAGCTGGTACAGGATGAACAGTTTACCCTCCTCTATCTTCAAATTT  360
            ||||||||||||||||||||||||||||||||||||||||||||||||||||||||||||
Sbjct  319  ATTAATTGAATCTGGAGCTGGTACAGGATGAACAGTTTACCCTCCTCTATCTTCAAATTT  378

Query  361  ATCACATTATGGCCCTTCAGTAGATTTAGCTATTTTTTCTCTTCATCTTGCTGGTGCTTC  420
            |||||||||||| |||||||||||||||||||||||||||||||||||||||||||||||
Sbjct  379  ATCACATTATGGTCCTTCAGTAGATTTAGCTATTTTTTCTCTTCATCTTGCTGGTGCTTC  438

Query  421  TTCAATTTTAGGTGCAATTAACTTTATTACAACTATTGTAAACATACGATCAATTGGAAT  480
            ||||||||||||||||||||||||||||||||||||||||||||||||||||||||||||
Sbjct  439  TTCAATTTTAGGTGCAATTAACTTTATTACAACTATTGTAAACATACGATCAATTGGAAT  498

Query  481  AACAATAGAACGTATACCATTATTTGTTTGATCTGTTTTAATTACAGCTATTTTATTACT  540
            ||||||||||||||||||||||||||||||||||||||||||||||||||||||| ||||
Sbjct  499  AACAATAGAACGTATACCATTATTTGTTTGATCTGTTTTAATTACAGCTATTTTACTACT  558

Query  541  TTTATCTTTACCTGTATTAGCAGGTGCCATTACAATATTGTTAACTGATCGAAACTTTAA  600
            ||||||||||||||||||||||||||||||||||||||||||||||||||||||||||||
Sbjct  559  TTTATCTTTACCTGTATTAGCAGGTGCCATTACAATATTGTTAACTGATCGAAACTTTAA  618

Query  601  TACATCATTTTTTGATCCTTCAGGGGGAGGAGATCCAATTTTATATCAACATTTATTT  658
            ||||||||||||||||||||||||||||||||||||||||||||||||||||||||||
Sbjct  619  TACATCATTTTTTGATCCTTCAGGGGGAGGAGATCCAATTTTATATCAACATTTATTT  676
```

>Rhipicephalus sanguineus sensu lato 'temperate lineage' isolate S1060 cytochrome c oxidase subunit I (COI) gene, partial cds; mitochondrial
Sequence ID: MF426006.1 Length: 705
Range 1: 28 to 685

Score:1205 bits(652), Expect:0.0,
Identities:656/658(99%), Gaps:0/658(0%), Strand: Plus/Plus

```
Query  1    AACAATATATTTAATTTTTGGAGCATGATCAGGTATATTAGGACTTAGTATAAGAATATT  60
            ||||||||||||||||||||||||||||||||||||||||||||||||||||||||||||
Sbjct  28   AACAATATATTTAATTTTTGGAGCATGATCAGGTATATTAGGACTTAGTATAAGAATATT  87

Query  61   AATTCGTATAGAATTAGGACAACCTGGAACTTTAATTGGAAATGATCAGATTTATAATGT  120
            ||||||||||||||||||||||||||||||||||||||||||||||||||||||||||||
Sbjct  88   AATTCGTATAGAATTAGGACAACCTGGAACTTTAATTGGAAATGATCAGATTTATAATGT  147

Query  121  AATCGTAACAGCACATGCATTTATTATAAtttttttATAGTTATACCAATCATAATCGG  180
            ||||||||||||||||||||||||||||||||||||||||||||||||||||||||||||
Sbjct  148  AATCGTAACAGCACATGCATTTATTATAATTTTTTTATAGTTATACCAATCATAATCGG  207

Query  181  AGGATTTGGAAATTGATTAGTCCCTATCATATTGGGTGCCCCAGATATAGCATTTCCACG  240
            ||||||||||||||||||||||||||||||||||||||||||||||||||||||||||||
Sbjct  208  AGGATTTGGAAATTGATTAGTCCCTATCATATTGGGTGCCCCAGATATAGCATTTCCACG  267

Query  241  AATAAATAATATAAGATTTTGACTTTTACCTCCTTCACTATTTTTATTAATTAACTCTTC  300
            ||||||||||||||||||||||||||||||||||||||||||||||||||||||||||||
Sbjct  268  AATAAATAATATAAGATTTTGACTTTTACCTCCTTCACTATTTTTATTAATTAACTCTTC  327

Query  301  ATTAATTGAATCTGGAGCTGGTACAGGATGAACAGTTTACCCTCCTCTATCTTCAAATTT  360
            ||||||||||||||||||||||||||||||||||||||||||||||||||||||||||||
Sbjct  328  ATTAATTGAATCTGGAGCTGGTACAGGATGAACAGTTTACCCTCCTCTATCTTCAAATTT  387

Query  361  ATCACATTATGGCCCTTCAGTAGATTTAGCTATTTTTTCTCTTCATCTTGCTGGTGCTTC  420
            |||||||||||| |||||||||||||||||||||||||||||||||||||||||||||||
Sbjct  388  ATCACATTATGGTCCTTCAGTAGATTTAGCTATTTTTTCTCTTCATCTTGCTGGTGCTTC  447

Query  421  TTCAATTTTAGGTGCAATTAACTTTATTACAACTATTGTAAACATACGATCAATTGGAAT  480
            ||||||||||||||||||||||||||||||||||||||||||||||||||| ||||||||
Sbjct  448  TTCAATTTTAGGTGCAATTAACTTTATTACAACTATTGTAAACATACGATTAATTGGAAT  507

Query  481  AACAATAGAACGTATACCATTATTTGTTTGATCTGTTTTAATTACAGCTATTTTATTACT  540
            ||||||||||||||||||||||||||||||||||||||||||||||||||||||||||||
Sbjct  508  AACAATAGAACGTATACCATTATTTGTTTGATCTGTTTTAATTACAGCTATTTTATTACT  567

Query  541  TTTATCTTTACCTGTATTAGCAGGTGCCATTACAATATTGTTAACTGATCGAAACTTTAA  600
            ||||||||||||||||||||||||||||||||||||||||||||||||||||||||||||
Sbjct  568  TTTATCTTTACCTGTATTAGCAGGTGCCATTACAATATTGTTAACTGATCGAAACTTTAA  627

Query  601  TACATCATTTTTTGATCCTTCAGGGGGAGGAGATCCAATTTTATATCAACATTTATTT  658
            ||||||||||||||||||||||||||||||||||||||||||||||||||||||||||
Sbjct  628  TACATCATTTTTTGATCCTTCAGGGGGAGGAGATCCAATTTTATATCAACATTTATTT  685
```

>Rhipicephalus sanguineus sensu lato 'temperate lineage' isolate S56 cytochrome c oxidase subunit I (COI) gene, partial cds; mitochondrial
Sequence ID: MF426017.1 Length: 680
Range 1: 11 to 668

Score:1199 bits(649), Expect:0.0,
Identities:655/658(99%), Gaps:0/658(0%), Strand: Plus/Plus

```
Query  1    AACAATATATTTAATTTTTGGAGCATGATCAGGTATATTAGGACTTAGTATAAGAATATT  60
            ||||||||||||||||||||||||||||||||||||||||||||||||||||||||||||
Sbjct  11   AACAATATATTTAATTTTTGGAGCATGATCAGGTATATTAGGACTTAGTATAAGAATATT  70

Query  61   AATTCGTATAGAATTAGGACAACCTGGAACTTTAATTGGAAATGATCAGATTTATAATGT  120
            ||||||||||||||||||||||||||||||||||||||||||||||||||||||||||||
Sbjct  71   AATTCGTATAGAATTAGGACAACCTGGAACTTTAATTGGAAATGATCAGATTTATAATGT  130

Query  121  AATCGTAACAGCACATGCATTTATTATAAttttttttATAGTTATACCAATCATAATCGG  180
            ||||||||||||||||||||||||||||||||||||||||||||||||||||||||||||
Sbjct  131  AATCGTAACAGCACATGCATTTATTATAATTTTTTTTATAGTTATACCAATCATAATCGG  190

Query  181  AGGATTTGGAAATTGATTAGTCCCTATCATATTGGGTGCCCCAGATATAGCATTTCCACG  240
            ||||||||||||||||||||||||||||||||||||||||||||||||||||||||||||
Sbjct  191  AGGATTTGGAAATTGATTAGTCCCTATCATATTGGGTGCCCCAGATATAGCATTTCCACG  250

Query  241  AATAAATAATATAAGATTTTGACTTTTACCTCCTTCACTATTTTTATTAATTAACTCTTC  300
            ||||||||||||||||||||||||||||||||||||||||||||||||||||||||||||
Sbjct  251  AATAAATAATATAAGATTTTGACTTTTACCTCCTTCACTATTTTTATTAATTAACTCTTC  310

Query  301  ATTAATTGAATCTGGAGCTGGTACAGGATGAACAGTTTACCCTCCTCTATCTTCAAATTT  360
            ||||||||||||||||||||||||||||||||||||||| ||||||||||||||||||||
Sbjct  311  ATTAATTGAATCTGGAGCTGGTACAGGATGAACAGTTTATCCTCCTCTATCTTCAAATTT  370

Query  361  ATCACATTATGGCCCTTCAGTAGATTTAGCTATTTTTTCTCTTCATCTTGCTGGTGCTTC  420
            ||||||||||||||||||||||||||||||||| ||||||||||||||||||||||||||
Sbjct  371  ATCACATTATGGCCCTTCAGTAGATTTAGCTATCTTTTCTCTTCATCTTGCTGGTGCTTC  430

Query  421  TTCAATTTTAGGTGCAATTAACTTTATTACAACTATTGTAAACATACGATCAATTGGAAT  480
            |||||||||||| |||||||||||||||||||||||||||||||||||||||||||||||
Sbjct  431  TTCAATTTTAGGAGCAATTAACTTTATTACAACTATTGTAAACATACGATCAATTGGAAT  490

Query  481  AACAATAGAACGTATACCATTATTTGTTTGATCTGTTTTAATTACAGCTATTTTATTACT  540
            ||||||||||||||||||||||||||||||||||||||||||||||||||||||||||||
Sbjct  491  AACAATAGAACGTATACCATTATTTGTTTGATCTGTTTTAATTACAGCTATTTTATTACT  550

Query  541  TTTATCTTTACCTGTATTAGCAGGTGCCATTACAATATTGTTAACTGATCGAAACTTTAA  600
            ||||||||||||||||||||||||||||||||||||||||||||||||||||||||||||
Sbjct  551  TTTATCTTTACCTGTATTAGCAGGTGCCATTACAATATTGTTAACTGATCGAAACTTTAA  610

Query  601  TACATCATTTTTTGATCCTTCAGGGGGAGGAGATCCAATTTTATATCAACATTTATTT  658
            ||||||||||||||||||||||||||||||||||||||||||||||||||||||||||
Sbjct  611  TACATCATTTTTTGATCCTTCAGGGGGAGGAGATCCAATTTTATATCAACATTTATTT  668
```

>Rhipicephalus sanguineus sensu lato 'temperate lineage' isolate CR1553 cytochrome c oxidase subunit I (COI) gene, partial cds; mitochondrial
Sequence ID: MF425998.1 Length: 689
Range 1: 20 to 677

Score:1199 bits(649), Expect:0.0,
Identities:655/658(99%), Gaps:0/658(0%), Strand: Plus/Plus

```
Query  1    AACAATATATTTAATTTTTGGAGCATGATCAGGTATATTAGGACTTAGTATAAGAATATT  60
            ||||||||||||||||||||||||||||||||||||||||||||||||||||||||||||
Sbjct  20   AACAATATATTTAATTTTTGGAGCATGATCAGGTATATTAGGACTTAGTATAAGAATATT  79

Query  61   AATTCGTATAGAATTAGGACAACCTGGAACTTTAATTGGAAATGATCAGATTTATAATGT  120
            ||||||||||||||||||||||||||||||||||||||||||||||||||||||||||||
Sbjct  80   AATTCGTATAGAATTAGGACAACCTGGAACTTTAATTGGAAATGATCAGATTTATAATGT  139

Query  121  AATCGTAACAGCACATGCATTTATTATAAttttttttATAGTTATACCAATCATAATCGG  180
            ||||||||||||||||||||||||||||||||||||||||||||||||||||||||||||
Sbjct  140  AATCGTAACAGCACATGCATTTATTATAATTTTTTTTATAGTTATACCAATCATAATCGG  199

Query  181  AGGATTTGGAAATTGATTAGTCCCTATCATATTGGGTGCCCCAGATATAGCATTTCCACG  240
            ||||||||||||||||||||||||||||||||| ||||||||||||||||||||||||||
Sbjct  200  AGGATTTGGAAATTGATTAGTCCCTATCATATTAGGTGCCCCAGATATAGCATTTCCACG  259

Query  241  AATAAATAATATAAGATTTTGACTTTTACCTCCTTCACTATTTTTATTAATTAACTCTTC  300
            ||||||||||||||||||||||||||||||||||||||||||||||||||||||||||||
Sbjct  260  AATAAATAATATAAGATTTTGACTTTTACCTCCTTCACTATTTTTATTAATTAACTCTTC  319

Query  301  ATTAATTGAATCTGGAGCTGGTACAGGATGAACAGTTTACCCTCCTCTATCTTCAAATTT  360
            ||||||||||||||||||||||||||||||||||||||||||||||||||||||||||||
Sbjct  320  ATTAATTGAATCTGGAGCTGGTACAGGATGAACAGTTTACCCTCCTCTATCTTCAAATTT  379

Query  361  ATCACATTATGGCCCTTCAGTAGATTTAGCTATTTTTTCTCTTCATCTTGCTGGTGCTTC  420
            ||||||||||||||||||||||||||||||||| ||||||||||||||||||||||||||
Sbjct  380  ATCACATTATGGCCCTTCAGTAGATTTAGCTATCTTTTCTCTTCATCTTGCTGGTGCTTC  439

Query  421  TTCAATTTTAGGTGCAATTAACTTTATTACAACTATTGTAAACATACGATCAATTGGAAT  480
            ||||||||||||||||||||||||||||||||||||||||||||||||||||||||||||
Sbjct  440  TTCAATTTTAGGTGCAATTAACTTTATTACAACTATTGTAAACATACGATCAATTGGAAT  499

Query  481  AACAATAGAACGTATACCATTATTTGTTTGATCTGTTTTAATTACAGCTATTTTATTACT  540
            ||||||||||||||||||||||||||||||||||||||||||||||||||||||||||||
Sbjct  500  AACAATAGAACGTATACCATTATTTGTTTGATCTGTTTTAATTACAGCTATTTTATTACT  559

Query  541  TTTATCTTTACCTGTATTAGCAGGTGCCATTACAATATTGTTAACTGATCGAAACTTTAA  600
             |||||||||||||||||||||||||||||||||||||||||||||||||||||||||||
Sbjct  560  CTTATCTTTACCTGTATTAGCAGGTGCCATTACAATATTGTTAACTGATCGAAACTTTAA  619

Query  601  TACATCATTTTTTGATCCTTCAGGGGGAGGAGATCCAATTTTATATCAACATTTATTT  658
            ||||||||||||||||||||||||||||||||||||||||||||||||||||||||||
Sbjct  620  TACATCATTTTTTGATCCTTCAGGGGGAGGAGATCCAATTTTATATCAACATTTATTT  677
```

```
>Rhipicephalus sanguineus haplotype IV cytochrome oxidase subunit I (COI) gene,
partial cds; mitochondrial
Sequence ID: KU556746.1 Length: 771
Range 1: 90 to 747

Score:1199 bits(649), Expect:0.0,
Identities:655/658(99%),  Gaps:0/658(0%), Strand: Plus/Plus

Query  1    AACAATATATTTAATTTTTGGAGCATGATCAGGTATATTAGGACTTAGTATAAGAATATT  60
            ||||||||||||||||||||||||||||||||||||||||||||||||||||||||||||
Sbjct  90   AACAATATATTTAATTTTTGGAGCATGATCAGGTATATTAGGACTTAGTATAAGAATATT  149

Query  61   AATTCGTATAGAATTAGGACAACCTGGAACTTTAATTGGAAATGATCAGATTTATAATGT  120
            ||||||||||||||||||||||||||||||||||||||||||||||||||||||||||||
Sbjct  150  AATTCGTATAGAATTAGGACAACCTGGAACTTTAATTGGAAATGATCAGATTTATAATGT  209

Query  121  AATCGTAACAGCACATGCATTTATTATAAtttttttATAGTTATACCAATCATAATCGG  180
            ||||||||||||||||||||||||||||||||||||||||||||||||||||||||||||
Sbjct  210  AATCGTAACAGCACATGCATTTATTATAATTTTTTTATAGTTATACCAATCATAATCGG  269

Query  181  AGGATTTGGAAATTGATTAGTCCCTATCATATTGGGTGCCCCAGATATAGCATTTCCACG  240
            ||||||||||||||||||||||||||||||||||||||||||||||||||||||||||||
Sbjct  270  AGGATTTGGAAATTGATTAGTCCCTATCATATTGGGTGCCCCAGATATAGCATTTCCACG  329

Query  241  AATAAATAATATAAGATTTTGACTTTTACCTCCTTCACTATTTTTATTAATTAACTCTTC  300
            ||||||||||||||||||||||||||||||||||||||||||||||||||||||||||||
Sbjct  330  AATAAATAATATAAGATTTTGACTTTTACCTCCTTCACTATTTTTATTAATTAACTCTTC  389

Query  301  ATTAATTGAATCTGGAGCTGGTACAGGATGAACAGTTTACCCTCCTCTATCTTCAAATTT  360
            ||||||||||||||||||||||||||||||||||||||| ||||||||||||||||||||
Sbjct  390  ATTAATTGAATCTGGAGCTGGTACAGGATGAACAGTTTATCCTCCTCTATCTTCAAATTT  449

Query  361  ATCACATTATGGCCCTTCAGTAGATTTAGCTATTTTTTCTCTTCATCTTGCTGGTGCTTC  420
            ||||||||||||||||||||||||||||||||| ||||||||||||||||||||||||||
Sbjct  450  ATCACATTATGGCCCTTCAGTAGATTTAGCTATCTTTTCTCTTCATCTTGCTGGTGCTTC  509

Query  421  TTCAATTTAGGTGCAATTAACTTTATTACAACTATTGTAAACATACGATCAATTGGAAT  480
            |||||||||||| |||||||||||||||||||||||||||||||||||||||||||||||
Sbjct  510  TTCAATTTAGGAGCAATTAACTTTATTACAACTATTGTAAACATACGATCAATTGGAAT  569

Query  481  AACAATAGAACGTATACCATTATTTGTTTGATCTGTTTTAATTACAGCTATTTTATTACT  540
            ||||||||||||||||||||||||||||||||||||||||||||||||||||||||||||
Sbjct  570  AACAATAGAACGTATACCATTATTTGTTTGATCTGTTTTAATTACAGCTATTTTATTACT  629

Query  541  TTTATCTTTACCTGTATTAGCAGGTGCCATTACAATATTGTTAACTGATCGAAACTTTAA  600
            ||||||||||||||||||||||||||||||||||||||||||||||||||||||||||||
Sbjct  630  TTTATCTTTACCTGTATTAGCAGGTGCCATTACAATATTGTTAACTGATCGAAACTTTAA  689

Query  601  TACATCATTTTTTGATCCTTCAGGGGGAGGAGATCCAATTTTATATCAACATTTATTT  658
            ||||||||||||||||||||||||||||||||||||||||||||||||||||||||||
```

```
Sbjct  690  TACATCATTTTTTGATCCTTCAGGGGGAGGAGATCCAATTTTATATCAACATTTATTT  747
```

Range 2: 30 to 64

Score:58.4 bits(31), Expect:0.001,
Identities:34/35(97%), Gaps:1/35(2%), Strand: Plus/Plus

```
Query  1   AACAATATATTTAATTTTTGGAGCATGATCA-GGT  34
           ||||||||||||||||||||||||||||||| |||
Sbjct  30  AACAATATATTTAATTTTTGGAGCATGATCATGGT  64
```

>Rhipicephalus sanguineus sensu lato 'temperate lineage' isolate S358 cytochrome c oxidase subunit I (COI) gene, partial cds; mitochondrial
Sequence ID: MF426012.1 Length: 684
Range 1: 14 to 671

Score:1194 bits(646), Expect:0.0,
Identities:654/658(99%), Gaps:0/658(0%), Strand: Plus/Plus

```
Query  1    AACAATATATTTAATTTTTGGAGCATGATCAGGTATATTAGGACTTAGTATAAGAATATT  60
            ||||||||||||||||||||||||||||||||||||||||||||||||||||||||||||
Sbjct  14   AACAATATATTTAATTTTTGGAGCATGATCAGGTATATTAGGACTTAGTATAAGAATATT  73

Query  61   AATTCGTATAGAATTAGGACAACCTGGAACTTTAATTGGAAATGATCAGATTTATAATGT  120
            ||||||||||||||||||||||||||||||||||||||||||||||||||||||||||||
Sbjct  74   AATTCGTATAGAATTAGGACAACCTGGAACTTTAATTGGAAATGATCAGATTTATAATGT  133

Query  121  AATCGTAACAGCACATGCATTTATTATAAtttttttATAGTTATACCAATCATAATCGG  180
            ||||||||||||||||||||||||||||||||||||||||||||||||||||||||||||
Sbjct  134  AATCGTAACAGCACATGCATTTATTATAATTTTTTTATAGTTATACCAATCATAATCGG  193

Query  181  AGGATTTGGAAATTGATTAGTCCCTATCATATTGGGTGCCCCAGATATAGCATTTCCACG  240
            |||||||||||||||||||||||||||||||||| |||||||||||||||||||||||||
Sbjct  194  AGGATTTGGAAATTGATTAGTCCCTATCATATTAGGTGCCCCAGATATAGCATTTCCACG  253

Query  241  AATAAATAATATAAGATTTTGACTTTTACCTCCTTCACTATTTTTATTAATTAACTCTTC  300
            ||||||||||||||||||||||||||||||||||||||||||||||||||||||||||||
Sbjct  254  AATAAATAATATAAGATTTTGACTTTTACCTCCTTCACTATTTTTATTAATTAACTCTTC  313

Query  301  ATTAATTGAATCTGGAGCTGGTACAGGATGAACAGTTTACCCTCCTCTATCTTCAAATTT  360
            ||||||||||||||||||||||||||||||||||||||||||||||||||||||||||||
Sbjct  314  ATTAATTGAATCTGGAGCTGGTACAGGATGAACAGTTTACCCTCCTCTATCTTCAAATTT  373

Query  361  ATCACATTATGGCCCTTCAGTAGATTTAGCTATTTTTTCTCTTCATCTTGCTGGTGCTTC  420
            |||||||||||||||||||||||||||||||||| |||||||||||||||||||||||||
Sbjct  374  ATCACATTATGGCCCTTCAGTAGATTTAGCTATCTTTTCTCTTCATCTTGCTGGTGCTTC  433

Query  421  TTCAATTTTAGGTGCAATTAACTTTATTACAACTATTGTAAACATACGATCAATTGGAAT  480
```

```
            ||||||||||||||||||||||||||||||||||||| ||||||||||||||||||||||
Sbjct  434  TTCAATTTTAGGTGCAATTAACTTTATTACAACTATTATAAACATACGATCAATTGGAAT  493

Query  481  AACAATAGAACGTATACCATTATTTGTTTGATCTGTTTTAATTACAGCTATTTTATTACT  540
            ||||||||||||||||||||||||||||||||||||||||||||||||||||||||||||
Sbjct  494  AACAATAGAACGTATACCATTATTTGTTTGATCTGTTTTAATTACAGCTATTTTATTACT  553

Query  541  TTTATCTTTACCTGTATTAGCAGGTGCCATTACAATATTGTTAACTGATCGAAACTTTAA  600
             |||||||||||||||||||||||||||||||||||||||||||||||||||||||||||
Sbjct  554  CTTATCTTTACCTGTATTAGCAGGTGCCATTACAATATTGTTAACTGATCGAAACTTTAA  613

Query  601  TACATCATTTTTTGATCCTTCAGGGGGAGGAGATCCAATTTTATATCAACATTTATTT  658
            ||||||||||||||||||||||||||||||||||||||||||||||||||||||||||
Sbjct  614  TACATCATTTTTTGATCCTTCAGGGGGAGGAGATCCAATTTTATATCAACATTTATTT  671
```

>Rhipicephalus sanguineus sensu lato 'temperate lineage' isolate S337 cytochrome c oxidase subunit I (COI) gene, partial cds; mitochondrial
Sequence ID: MF426010.1 Length: 824
Range 1: 1 to 648

Score:1192 bits(645), Expect:0.0,
Identities:647/648(99%), Gaps:0/648(0%), Strand: Plus/Plus

```
Query  11   TTAATTTTTGGAGCATGATCAGGTATATTAGGACTTAGTATAAGAATATTAATTCGTATA  70
            ||||||||||||||||||||||||||||||||||||||||||||||||||||||||||||
Sbjct  1    TTAATTTTTGGAGCATGATCAGGTATATTAGGACTTAGTATAAGAATATTAATTCGTATA  60

Query  71   GAATTAGGACAACCTGGAACTTTAATTGGAAATGATCAGATTTATAATGTAATCGTAACA  130
            ||||||||||||||||||||||||||||||||||||||||||||||||||||||||||||
Sbjct  61   GAATTAGGACAACCTGGAACTTTAATTGGAAATGATCAGATTTATAATGTAATCGTAACA  120

Query  131  GCACATGCATTTATTATAAttttttttATAGTTATACCAATCATAATCGGAGGATTTGGA  190
            ||||||||||||||||||||||||||||||||||||||||||||||||||||||||||||
Sbjct  121  GCACATGCATTTATTATAATTTTTTTTATAGTTATACCAATCATAATCGGAGGATTTGGA  180

Query  191  AATTGATTAGTCCCTATCATATTGGGTGCCCCAGATATAGCATTTCCACGAATAAATAAT  250
            ||||||||||||||||||||||||||||||||||||||||||||||||||||||||||||
Sbjct  181  AATTGATTAGTCCCTATCATATTGGGTGCCCCAGATATAGCATTTCCACGAATAAATAAT  240

Query  251  ATAAGATTTTGACTTTTACCTCCTTCACTATTTTTATTAATTAACTCTTCATTAATTGAA  310
            ||||||||||||||||||||||||||||||||||||||||||||||||||||||||||||
Sbjct  241  ATAAGATTTTGACTTTTACCTCCTTCACTATTTTTATTAATTAACTCTTCATTAATTGAA  300

Query  311  TCTGGAGCTGGTACAGGATGAACAGTTTACCCTCCTCTATCTTCAAATTTATCACATTAT  370
            ||||||||||||||||||||||||||||||||||||||||||||||||||||||||||||
Sbjct  301  TCTGGAGCTGGTACAGGATGAACAGTTTACCCTCCTCTATCTTCAAATTTATCACATTAT  360

Query  371  GGCCCTTCAGTAGATTTAGCTATTTTTTCTCTTCATCTTGCTGGTGCTTCTTCAATTTTA  430
            ||||||||||||||||||||||| ||||||||||||||||||||||||||||||||||||
Sbjct  361  GGCCCTTCAGTAGATTTAGCTATCTTTTCTCTTCATCTTGCTGGTGCTTCTTCAATTTTA  420
```

```
Query  431   GGTGCAATTAACTTTATTACAACTATTGTAAACATACGATCAATTGGAATAACAATAGAA  490
             ||||||||||||||||||||||||||||||||||||||||||||||||||||||||||||
Sbjct  421   GGTGCAATTAACTTTATTACAACTATTGTAAACATACGATCAATTGGAATAACAATAGAA  480

Query  491   CGTATACCATTATTTGTTTGATCTGTTTTAATTACAGCTATTTTATTACTTTTATCTTTA  550
             ||||||||||||||||||||||||||||||||||||||||||||||||||||||||||||
Sbjct  481   CGTATACCATTATTTGTTTGATCTGTTTTAATTACAGCTATTTTATTACTTTTATCTTTA  540

Query  551   CCTGTATTAGCAGGTGCCATTACAATATTGTTAACTGATCGAAACTTTAATACATCATTT  610
             ||||||||||||||||||||||||||||||||||||||||||||||||||||||||||||
Sbjct  541   CCTGTATTAGCAGGTGCCATTACAATATTGTTAACTGATCGAAACTTTAATACATCATTT  600

Query  611   TTTGATCCTTCAGGGGGAGGAGATCCAATTTTATATCAACATTTATTT  658
             ||||||||||||||||||||||||||||||||||||||||||||||||
Sbjct  601   TTTGATCCTTCAGGGGGAGGAGATCCAATTTTATATCAACATTTATTT  648
```

>Rhipicephalus sanguineus haplotype II cytochrome oxidase subunit I (COI) gene, partial cds; mitochondrial
Sequence ID: KU556744.1 Length: 787
Range 1: 29 to 688

Score:1190 bits(644), Expect:0.0,
Identities:655/660(99%), Gaps:2/660(0%), Strand: Plus/Plus

```
Query  1     AACAATATATTTAATTTTTGGAGCATGATCAGGTATATTAGGACTTAGTATAAGAATATT  60
             ||||||||||||||||||||||||||||||||||||||||||||||||||||||||||||
Sbjct  29    AACAATATATTTAATTTTTGGAGCATGATCAGGTATATTAGGACTTAGTATAAGAATATT  88

Query  61    AATTCGTATAGAATTAGGACAACCTGGAACTTTAATTGGAAATGATCAGATTTATAATGT  120
             ||||||||||||||||||||||||||||||||||||||||||||||||||||||||||||
Sbjct  89    AATTCGTATAGAATTAGGACAACCTGGAACTTTAATTGGAAATGATCAGATTTATAATGT  148

Query  121   AATCGTAACAGCACATGCATTTATTATAAttttttttATAGTTATACCAATCATAATCGG  180
             ||||||||||||||||||||||||||||||||||||||||||||||||||||||||||||
Sbjct  149   AATCGTAACAGCACATGCATTTATTATAATTTTTTTTATAGTTATACCAATCATAATCGG  208

Query  181   AGGATTTGGAAATTGATTAGTCCCTATCATATTGGGTGCCCCAGATATAGCATTTCCACG  240
             |||||||||||| |||||||||||||||||||||||||||||||||||||||||||||||
Sbjct  209   AGGATTTGGAAACTGATTAGTCCCTATCATATTGGGTGCCCCAGATATAGCATTTCCACG  268

Query  241   AATAAATAATATAAGATTTTGACTTTTACCTCCTTCACTATTTTTATTAATTAACTCTTC  300
             ||||||||||||||||||||||||||||||||||||||||||||||||||||||||||||
Sbjct  269   AATAAATAATATAAGATTTTGACTTTTACCTCCTTCACTATTTTTATTAATTAACTCTTC  328

Query  301   ATTAATTGAATCTGGAGCTGGTACAGGATGAACAGTTTACCCTCCTCTATCTTCAAATTT  360
             ||||||||||||||||||||||||||||||||||||||||||||||||||||||||||||
Sbjct  329   ATTAATTGAATCTGGAGCTGGTACAGGATGAACAGTTTACCCTCCTCTATCTTCAAATTT  388

Query  361   ATCACATTATGGCCCTTCAGTAGATTTAGCTATTTTTTCTCTTCATCTTGCTGGTGCTTC  420
             |||||||||||| |||||||||||||||||||||||||||||||||||||||||||||||
Sbjct  389   ATCACATTATGGTCCTTCAGTAGATTTAGCTATTTTTTCTCTTCATCTTGCTGGTGCTTC  448
```

```
Query  421   TTCAATTTTAGGTGCAATTAACTTTATTACAACTATTGTAAACATACGATCAATTGGAAT  480
             ||||||||||||||||||||||||||||||||||||||||||||||||||||||||||||
Sbjct  449   TTCAATTTTAGGTGCAATTAACTTTATTACAACTATTGTAAACATACGATCAATTGGAAT  508

Query  481   AACAATAGAACGTATACCATTATTTGTTTGATCTGTTTTAATTACAGCTATTTTATTACT  540
             ||||||||||||||||||||||||||||||||||||||||||||||||||||||||||||
Sbjct  509   AACAATAGAACGTATACCATTATTTGTTTGATCTGTTTTAATTACAGCTATTTTATTACT  568

Query  541   TTTATCTTTACCTGTATTAGCAGGTGCCATTACAATATTGTTAACTGATCGAAACTTTAA  600
             ||||||||||||||||||||||||||||||||||||||||||||||||||||||||||||
Sbjct  569   TTTATCTTTACCTGTATTAGCAGGTGCCATTACAATATTGTTAACTGATCGAAACTTTAA  628

Query  601   TACATCATTTTTTGATCCTTCAGGGGG-AGGAGATCC-AATTTTATATCAACATTTATTT  658
             ||||||||||||||||||||||||||| ||||||||| |||||||||||||| |||||||
Sbjct  629   TACATCATTTTTTGATCCTTCAGGGGGGAGGAGATCCAAATTTTATATCAACTTTTATTT  688
```

>Rhipicephalus sanguineus haplotype I cytochrome oxidase subunit I (COI) gene, partial cds; mitochondrial
Sequence ID: KU556743.1 Length: 659
Range 1: 12 to 659

Score:1186 bits(642), Expect:0.0,
Identities:646/648(99%), Gaps:0/648(0%), Strand: Plus/Plus

```
Query  1     AACAATATATTTAATTTTTGGAGCATGATCAGGTATATTAGGACTTAGTATAAGAATATT  60
             ||||||||||||||||||||||||||||||||||||||||||||||||||||||||||||
Sbjct  12    AACAATATATTTAATTTTTGGAGCATGATCAGGTATATTAGGACTTAGTATAAGAATATT  71

Query  61    AATTCGTATAGAATTAGGACAACCTGGAACTTTAATTGGAAATGATCAGATTTATAATGT  120
             ||||||||||||||||||||||||||||||||||||||||||||||||||||||||||||
Sbjct  72    AATTCGTATAGAATTAGGACAACCTGGAACTTTAATTGGAAATGATCAGATTTATAATGT  131

Query  121   AATCGTAACAGCACATGCATTTATTATAAttttttttATAGTTATACCAATCATAATCGG  180
             ||||||||||||||||||||||||||||||||||||||||||||||||||||||||||||
Sbjct  132   AATCGTAACAGCACATGCATTTATTATAATTTTTTTTATAGTTATACCAATCATAATCGG  191

Query  181   AGGATTTGGAAATTGATTAGTCCCTATCATATTGGGTGCCCCAGATATAGCATTTCCACG  240
             ||||||||||||||||||| ||||||||||||||||||||||||||||||||||||||||
Sbjct  192   AGGATTTGGAAATTGATTAATCCCTATCATATTGGGTGCCCCAGATATAGCATTTCCACG  251

Query  241   AATAAATAATATAAGATTTTGACTTTTACCTCCTTCACTATTTTTATTAATTAACTCTTC  300
             ||||||||||||||||||||||||||||||||||||||||||||||||||||||||||||
Sbjct  252   AATAAATAATATAAGATTTTGACTTTTACCTCCTTCACTATTTTTATTAATTAACTCTTC  311

Query  301   ATTAATTGAATCTGGAGCTGGTACAGGATGAACAGTTTACCCTCCTCTATCTTCAAATTT  360
             ||||||||||||||||||||||||||||||||||||||||||||||||||||||||||||
Sbjct  312   ATTAATTGAATCTGGAGCTGGTACAGGATGAACAGTTTACCCTCCTCTATCTTCAAATTT  371

Query  361   ATCACATTATGGCCCTTCAGTAGATTTAGCTATTTTTTCTCTTCATCTTGCTGGTGCTTC  420
             |||||||||||| |||||||||||||||||||||||||||||||||||||||||||||||
```

```
Sbjct  372  ATCACATTATGGTCCTTCAGTAGATTTAGCTATTTTTTCTCTTCATCTTGCTGGTGCTTC  431

Query  421  TTCAATTTTAGGTGCAATTAACTTTATTACAACTATTGTAAACATACGATCAATTGGAAT  480
            ||||||||||||||||||||||||||||||||||||||||||||||||||||||||||||
Sbjct  432  TTCAATTTTAGGTGCAATTAACTTTATTACAACTATTGTAAACATACGATCAATTGGAAT  491

Query  481  AACAATAGAACGTATACCATTATTTGTTTGATCTGTTTTAATTACAGCTATTTTATTACT  540
            ||||||||||||||||||||||||||||||||||||||||||||||||||||||||||||
Sbjct  492  AACAATAGAACGTATACCATTATTTGTTTGATCTGTTTTAATTACAGCTATTTTATTACT  551

Query  541  TTTATCTTTACCTGTATTAGCAGGTGCCATTACAATATTGTTAACTGATCGAAACTTTAA  600
            ||||||||||||||||||||||||||||||||||||||||||||||||||||||||||||
Sbjct  552  TTTATCTTTACCTGTATTAGCAGGTGCCATTACAATATTGTTAACTGATCGAAACTTTAA  611

Query  601  TACATCATTTTTTGATCCTTCAGGGGGAGGAGATCCAATTTTATATCA  648
            ||||||||||||||||||||||||||||||||||||||||||||||||
Sbjct  612  TACATCATTTTTTGATCCTTCAGGGGGAGGAGATCCAATTTTATATCA  659
```

>Rhipicephalus sanguineus sensu lato 'temperate lineage' isolate S943 cytochrome c oxidase subunit I (COI) gene, partial cds; mitochondrial
Sequence ID: MF426020.1 Length: 812
Range 1: 2 to 644

Score:1182 bits(640), Expect:0.0,
Identities:642/643(99%), Gaps:0/643(0%), Strand: Plus/Plus

```
Query  16   TTTTGGAGCATGATCAGGTATATTAGGACTTAGTATAAGAATATTAATTCGTATAGAATT  75
            ||||||||||||||||||||||||||||||||||||||||||||||||||||||||||||
Sbjct  2    TTTTGGAGCATGATCAGGTATATTAGGACTTAGTATAAGAATATTAATTCGTATAGAATT  61

Query  76   AGGACAACCTGGAACTTTAATTGGAAATGATCAGATTTATAATGTAATCGTAACAGCACA  135
            ||||||||||||||||||||||||||||||||||||||||||||||||||||||||||||
Sbjct  62   AGGACAACCTGGAACTTTAATTGGAAATGATCAGATTTATAATGTAATCGTAACAGCACA  121

Query  136  TGCATTTATTATAAttttttttATAGTTATACCAATCATAATCGGAGGATTTGGAAATTG  195
            ||||||||||||||||||||||||||||||||||||||||||||||||||||||||||||
Sbjct  122  TGCATTTATTATAATTTTTTTTATAGTTATACCAATCATAATCGGAGGATTTGGAAATTG  181

Query  196  ATTAGTCCCTATCATATTGGGTGCCCCAGATATAGCATTTCCACGAATAAATAATATAAG  255
            ||||||||||||||||||||||||||||||||||||||||||||||||||||||||||||
Sbjct  182  ATTAGTCCCTATCATATTGGGTGCCCCAGATATAGCATTTCCACGAATAAATAATATAAG  241

Query  256  ATTTTGACTTTTACCTCCTTCACTATTTTTATTAATTAACTCTTCATTAATTGAATCTGG  315
            ||||||||||||||||||||||||||||||||||||||||||||||||||||||||||||
Sbjct  242  ATTTTGACTTTTACCTCCTTCACTATTTTTATTAATTAACTCTTCATTAATTGAATCTGG  301

Query  316  AGCTGGTACAGGATGAACAGTTTACCCTCCTCTATCTTCAAATTTATCACATTATGGCCC  375
            ||||||||||||||||||||||||||||||||||||||||||||||||||||||||| ||
Sbjct  302  AGCTGGTACAGGATGAACAGTTTACCCTCCTCTATCTTCAAATTTATCACATTATGGTCC  361

Query  376  TTCAGTAGATTTAGCTATTTTTTCTCTTCATCTTGCTGGTGCTTCTTCAATTTTAGGTGC  435
```

```
                ||||||||||||||||||||||||||||||||||||||||||||||||||||||||||||
Sbjct  362   TTCAGTAGATTTAGCTATTTTTTCTCTTCATCTTGCTGGTGCTTCTTCAATTTTAGGTGC  421

Query  436   AATTAACTTTATTACAACTATTGTAAACATACGATCAATTGGAATAACAATAGAACGTAT  495
             ||||||||||||||||||||||||||||||||||||||||||||||||||||||||||||
Sbjct  422   AATTAACTTTATTACAACTATTGTAAACATACGATCAATTGGAATAACAATAGAACGTAT  481

Query  496   ACCATTATTTGTTTGATCTGTTTTAATTACAGCTATTTTATTACTTTTATCTTTACCTGT  555
             ||||||||||||||||||||||||||||||||||||||||||||||||||||||||||||
Sbjct  482   ACCATTATTTGTTTGATCTGTTTTAATTACAGCTATTTTATTACTTTTATCTTTACCTGT  541

Query  556   ATTAGCAGGTGCCATTACAATATTGTTAACTGATCGAAACTTTAATACATCATTTTTTGA  615
             ||||||||||||||||||||||||||||||||||||||||||||||||||||||||||||
Sbjct  542   ATTAGCAGGTGCCATTACAATATTGTTAACTGATCGAAACTTTAATACATCATTTTTTGA  601

Query  616   TCCTTCAGGGGGAGGAGATCCAATTTTATATCAACATTTATTT  658
             |||||||||||||||||||||||||||||||||||||||||||
Sbjct  602   TCCTTCAGGGGGAGGAGATCCAATTTTATATCAACATTTATTT  644
```

>Rhipicephalus sanguineus isolate LIC5424B cytochrome oxidase subunit 1 (COI) gene, partial cds; mitochondrial
Sequence ID: KX383818.1 Length: 768
>Rhipicephalus sanguineus isolate LIC5554A cytochrome oxidase subunit 1 (COI) gene, partial cds; mitochondrial
Sequence ID: KX383819.1 Length: 768
Range 1: 1 to 642

Score:1175 bits(636), Expect:0.0,
Identities:640/642(99%), Gaps:0/642(0%), Strand: Plus/Plus

```
Query  17    TTTGGAGCATGATCAGGTATATTAGGACTTAGTATAAGAATATTAATTCGTATAGAATTA  76
             ||||||||||||||||||||||||||||||||||||||||||||||||||||||||||||
Sbjct  1     TTTGGAGCATGATCAGGTATATTAGGACTTAGTATAAGAATATTAATTCGTATAGAATTA  60

Query  77    GGACAACCTGGAACTTTAATTGGAAATGATCAGATTTATAATGTAATCGTAACAGCACAT  136
             ||||||||||||||||||||||||||||||||||||||||||||||||||||||||||||
Sbjct  61    GGACAACCTGGAACTTTAATTGGAAATGATCAGATTTATAATGTAATCGTAACAGCACAT  120

Query  137   GCATTTATTATAAttttttttATAGTTATACCAATCATAATCGGAGGATTTGGAAATTGA  196
             ||||||||||||||||||||||||||||||||||||||||||||||||||||||||||||
Sbjct  121   GCATTTATTATAATTTTTTTTATAGTTATACCAATCATAATCGGAGGATTTGGAAATTGA  180

Query  197   TTAGTCCCTATCATATTGGGTGCCCCAGATATAGCATTTCCACGAATAAATAATATAAGA  256
             ||||||||||||||||||||||||||||||||||||||||||||||||||||||||||||
Sbjct  181   TTAGTCCCTATCATATTGGGTGCCCCAGATATAGCATTTCCACGAATAAATAATATAAGA  240

Query  257   TTTTGACTTTTACCTCCTTCACTATTTTTATTAATTAACTCTTCATTAATTGAATCTGGA  316
             ||||||||||||||||||||||||||||||||||||||||||||||||||||||||||||
Sbjct  241   TTTTGACTTTTACCTCCTTCACTATTTTTATTAATTAACTCTTCATTAATTGAATCTGGA  300

Query  317   GCTGGTACAGGATGAACAGTTTACCCTCCTCTATCTTCAAATTTATCACATTATGGCCCT  376
```

```
            ||||||||||||||||||||||||||||||||||||||||||||||||||||||||||||
Sbjct  301  GCTGGTACAGGATGAACAGTTTACCCTCCTCTATCTTCAAATTTATCACATTATGGCCCT  360

Query  377  TCAGTAGATTTAGCTATTTTTTCTCTTCATCTTGCTGGTGCTTCTTCAATTTTAGGTGCA  436
            ||||||||||||||||| ||||||||||||||||||||||||||||||||||||||||||
Sbjct  361  TCAGTAGATTTAGCTATCTTTTCTCTTCATCTTGCTGGTGCTTCTTCAATTTTAGGTGCA  420

Query  437  ATTAACTTTATTACAACTATTGTAAACATACGATCAATTGGAATAACAATAGAACGTATA  496
            |||||||||||||||||||||||||||||||| |||||||||||||||||||||||||||
Sbjct  421  ATTAACTTTATTACAACTATTGTAAACATACGGTCAATTGGAATAACAATAGAACGTATA  480

Query  497  CCATTATTTGTTTGATCTGTTTTAATTACAGCTATTTTATTACTTTTATCTTTACCTGTA  556
            ||||||||||||||||||||||||||||||||||||||||||||||||||||||||||||
Sbjct  481  CCATTATTTGTTTGATCTGTTTTAATTACAGCTATTTTATTACTTTTATCTTTACCTGTA  540

Query  557  TTAGCAGGTGCCATTACAATATTGTTAACTGATCGAAACTTTAATACATCATTTTTTGAT  616
            ||||||||||||||||||||||||||||||||||||||||||||||||||||||||||||
Sbjct  541  TTAGCAGGTGCCATTACAATATTGTTAACTGATCGAAACTTTAATACATCATTTTTTGAT  600

Query  617  CCTTCAGGGGGAGGAGATCCAATTTTATATCAACATTTATTT  658
            ||||||||||||||||||||||||||||||||||||||||||
Sbjct  601  CCTTCAGGGGGAGGAGATCCAATTTTATATCAACATTTATTT  642
```

>Rhipicephalus sanguineus isolate LIC5533B cytochrome oxidase subunit 1 (COI) gene, partial cds; mitochondrial
Sequence ID: KX383811.1 Length: 764
>Rhipicephalus sanguineus isolate LIC5533C cytochrome oxidase subunit 1 (COI) gene, partial cds; mitochondrial
Sequence ID: KX383812.1 Length: 764
Range 1: 1 to 638

Score:1168 bits(632), Expect:0.0,
Identities:636/638(99%), Gaps:0/638(0%), Strand: Plus/Plus

```
Query  21   GAGCATGATCAGGTATATTAGGACTTAGTATAAGAATATTAATTCGTATAGAATTAGGAC  80
            ||||||||||||||||||||||||||||||||||||||||||||||||||||||||||||
Sbjct  1    GAGCATGATCAGGTATATTAGGACTTAGTATAAGAATATTAATTCGTATAGAATTAGGAC  60

Query  81   AACCTGGAACTTTAATTGGAAATGATCAGATTTATAATGTAATCGTAACAGCACATGCAT  140
            ||||||||||||||||||||||||||||||||||||||||||||||||||||||||||||
Sbjct  61   AACCTGGAACTTTAATTGGAAATGATCAGATTTATAATGTAATCGTAACAGCACATGCAT  120

Query  141  TTATTATAAttttttttATAGTTATACCAATCATAATCGGAGGATTTGGAAATTGATTAG  200
            ||||||||||||||||||||||||||||||||||||||||||||||||||||||||||||
Sbjct  121  TTATTATAATTTTTTTTATAGTTATACCAATCATAATCGGAGGATTTGGAAATTGATTAG  180

Query  201  TCCCTATCATATTGGGTGCCCCAGATATAGCATTTCCACGAATAAATAATATAAGATTTT  260
            ||||||||||||||||||||||||||||||||||||||||||||||||||||||||||||
Sbjct  181  TCCCTATCATATTGGGTGCCCCAGATATAGCATTTCCACGAATAAATAATATAAGATTTT  240

Query  261  GACTTTTACCTCCTTCACTATTTTTATTAATTAACTCTTCATTAATTGAATCTGGAGCTG  320
```

```
            ||||||||||||||||||||||||||||||||||||||||||||||||||||||||||||
Sbjct  241  GACTTTTACCTCCTTCACTATTTTTATTAATTAACTCTTCATTAATTGAATCTGGAGCTG  300

Query  321  GTACAGGATGAACAGTTTACCCTCCTCTATCTTCAAATTTATCACATTATGGCCCTTCAG  380
            ||||||||||||||||||||||||||||||||||||||||||||||||||||||||||||
Sbjct  301  GTACAGGATGAACAGTTTACCCTCCTCTATCTTCAAATTTATCACATTATGGCCCTTCAG  360

Query  381  TAGATTTAGCTATTTTTTCTCTTCATCTTGCTGGTGCTTCTTCAATTTTAGGTGCAATTA  440
            ||||||||||||| ||||||||||||||||||||||||||||||||||||||||||||||
Sbjct  361  TAGATTTAGCTATCTTTTCTCTTCATCTTGCTGGTGCTTCTTCAATTTTAGGTGCAATTA  420

Query  441  ACTTTATTACAACTATTGTAAACATACGATCAATTGGAATAACAATAGAACGTATACCAT  500
            |||||||||||||||||||||||||||| |||||||||||||||||||||||||||||||
Sbjct  421  ACTTTATTACAACTATTGTAAACATACGGTCAATTGGAATAACAATAGAACGTATACCAT  480

Query  501  TATTTGTTTGATCTGTTTTAATTACAGCTATTTTATTACTTTTATCTTTACCTGTATTAG  560
            ||||||||||||||||||||||||||||||||||||||||||||||||||||||||||||
Sbjct  481  TATTTGTTTGATCTGTTTTAATTACAGCTATTTTATTACTTTTATCTTTACCTGTATTAG  540

Query  561  CAGGTGCCATTACAATATTGTTAACTGATCGAAACTTTAATACATCATTTTTTGATCCTT  620
            ||||||||||||||||||||||||||||||||||||||||||||||||||||||||||||
Sbjct  541  CAGGTGCCATTACAATATTGTTAACTGATCGAAACTTTAATACATCATTTTTTGATCCTT  600

Query  621  CAGGGGGAGGAGATCCAATTTTATATCAACATTTATTT  658
            ||||||||||||||||||||||||||||||||||||||
Sbjct  601  CAGGGGGAGGAGATCCAATTTTATATCAACATTTATTT  638
```

>Rhipicephalus sanguineus isolate LIC4736B cytochrome oxidase subunit 1 (COI) gene, partial cds; mitochondrial
Sequence ID: KX383807.1 Length: 763
>Rhipicephalus sanguineus isolate LIC4736C cytochrome oxidase subunit 1 (COI) gene, partial cds; mitochondrial
Sequence ID: KX383808.1 Length: 763
>Rhipicephalus sanguineus isolate LIC4736F cytochrome oxidase subunit 1 (COI) gene, partial cds; mitochondrial
Sequence ID: KX383810.1 Length: 763
>Rhipicephalus sanguineus isolate LIC5535C cytochrome oxidase subunit 1 (COI) gene, partial cds; mitochondrial
Sequence ID: KX383813.1 Length: 763
Range 1: 1 to 637

Score:1166 bits(631), Expect:0.0,
Identities:635/637(99%), Gaps:0/637(0%), Strand: Plus/Plus

```
Query  22   AGCATGATCAGGTATATTAGGACTTAGTATAAGAATATTAATTCGTATAGAATTAGGACA  81
            ||||||||||||||||||||||||||||||||||||||||||||||||||||||||||||
Sbjct  1    AGCATGATCAGGTATATTAGGACTTAGTATAAGAATATTAATTCGTATAGAATTAGGACA  60

Query  82   ACCTGGAACTTTAATTGGAAATGATCAGATTTATAATGTAATCGTAACAGCACATGCATT  141
            ||||||||||||||||||||||||||||||||||||||||||||||||||||||||||||
Sbjct  61   ACCTGGAACTTTAATTGGAAATGATCAGATTTATAATGTAATCGTAACAGCACATGCATT  120
```

```
Query  142   TATTATAAtttttttATAGTTATACCAATCATAATCGGAGGATTTGGAAATTGATTAGT  201
             ||||||||||||||||||||||||||||||||||||||||||||||||||||||||||||
Sbjct  121   TATTATAATTTTTTTTATAGTTATACCAATCATAATCGGAGGATTTGGAAATTGATTAGT  180

Query  202   CCCTATCATATTGGGTGCCCCAGATATAGCATTTCCACGAATAAATAATATAAGATTTTG  261
             ||||||||||||||||||||||||||||||||||||||||||||||||||||||||||||
Sbjct  181   CCCTATCATATTGGGTGCCCCAGATATAGCATTTCCACGAATAAATAATATAAGATTTTG  240

Query  262   ACTTTTACCTCCTTCACTATTTTTATTAATTAACTCTTCATTAATTGAATCTGGAGCTGG  321
             ||||||||||||||||||||||||||||||||||||||||||||||||||||||||||||
Sbjct  241   ACTTTTACCTCCTTCACTATTTTTATTAATTAACTCTTCATTAATTGAATCTGGAGCTGG  300

Query  322   TACAGGATGAACAGTTTACCCTCCTCTATCTTCAAATTTATCACATTATGGCCCTTCAGT  381
             ||||||||||||||||||||||||||||||||||||||||||||||||||||||||||||
Sbjct  301   TACAGGATGAACAGTTTACCCTCCTCTATCTTCAAATTTATCACATTATGGCCCTTCAGT  360

Query  382   AGATTTAGCTATTTTTTCTCTTCATCTTGCTGGTGCTTCTTCAATTTTAGGTGCAATTAA  441
             |||||||||||| |||||||||||||||||||||||||||||||||||||||||||||||
Sbjct  361   AGATTTAGCTATCTTTTCTCTTCATCTTGCTGGTGCTTCTTCAATTTTAGGTGCAATTAA  420

Query  442   CTTTATTACAACTATTGTAAACATACGATCAATTGGAATAACAATAGAACGTATACCATT  501
             |||||||||||||||||||||||||| |||||||||||||||||||||||||||||||||
Sbjct  421   CTTTATTACAACTATTGTAAACATACGGTCAATTGGAATAACAATAGAACGTATACCATT  480

Query  502   ATTTGTTTGATCTGTTTTAATTACAGCTATTTTATTACTTTTATCTTTACCTGTATTAGC  561
             ||||||||||||||||||||||||||||||||||||||||||||||||||||||||||||
Sbjct  481   ATTTGTTTGATCTGTTTTAATTACAGCTATTTTATTACTTTTATCTTTACCTGTATTAGC  540

Query  562   AGGTGCCATTACAATATTGTTAACTGATCGAAACTTTAATACATCATTTTTTGATCCTTC  621
             ||||||||||||||||||||||||||||||||||||||||||||||||||||||||||||
Sbjct  541   AGGTGCCATTACAATATTGTTAACTGATCGAAACTTTAATACATCATTTTTTGATCCTTC  600

Query  622   AGGGGGAGGAGATCCAATTTTATATCAACATTTATTT  658
             |||||||||||||||||||||||||||||||||||||
Sbjct  601   AGGGGGAGGAGATCCAATTTTATATCAACATTTATTT  637
```

>Rhipicephalus sanguineus sensu lato 'temperate lineage' isolate S344 cytochrome c oxidase subunit I (COI) gene, partial cds; mitochondrial
Sequence ID: MF426011.1 Length: 809
Range 1: 3 to 644

Score:1164 bits(630), Expect:0.0,
Identities:638/642(99%), Gaps:0/642(0%), Strand: Plus/Plus

```
Query  17    TTTGGAGCATGATCAGGTATATTAGGACTTAGTATAAGAATATTAATTCGTATAGAATTA  76
             ||||||||||||||||||||||||||||||||||||||||||||||||||||||||||||
Sbjct  3     TTTGGAGCATGATCAGGTATATTAGGACTTAGTATAAGAATATTAATTCGTATAGAATTA  62

Query  77    GGACAACCTGGAACTTTAATTGGAAATGATCAGATTTATAATGTAATCGTAACAGCACAT  136
             |||||||||||||||||||||||||||||||||||||||||||||||||||||||||||
```

```
Sbjct  63    GGACAACCTGGAACTTTAATTGGAAATGATCAGATTTATAATGTAATCGTAACAGCACAC  122

Query  137   GCATTTATTATAAttttttttATAGTTATACCAATCATAATCGGAGGATTTGGAAATTGA  196
             ||||||||||||||||||||||||||||||||||||||||||||||||||||||||||||
Sbjct  123   GCATTTATTATAATTTTTTTTATAGTTATACCAATCATAATCGGAGGATTTGGAAATTGA  182

Query  197   TTAGTCCCTATCATATTGGGTGCCCCAGATATAGCATTTCCACGAATAAATAATATAAGA  256
             ||||||||||| ||||| ||||||||||||||||||||||||||||||||||||||||||
Sbjct  183   TTAGTCCCTATTATATTAGGTGCCCCAGATATAGCATTTCCACGAATAAATAATATAAGA  242

Query  257   TTTTGACTTTTACCTCCTTCACTATTTTTATTAATTAACTCTTCATTAATTGAATCTGGA  316
             ||||||||||||||||||||||||||||||||||||||||||||||||||||||||||||
Sbjct  243   TTTTGACTTTTACCTCCTTCACTATTTTTATTAATTAACTCTTCATTAATTGAATCTGGA  302

Query  317   GCTGGTACAGGATGAACAGTTTACCCTCCTCTATCTTCAAATTTATCACATTATGGCCCT  376
             ||||||||||||||||||||||||||||||||||||||||||||||||||||||||||||
Sbjct  303   GCTGGTACAGGATGAACAGTTTACCCTCCTCTATCTTCAAATTTATCACATTATGGCCCT  362

Query  377   TCAGTAGATTTAGCTATTTTTTCTCTTCATCTTGCTGGTGCTTCTTCAATTTTAGGTGCA  436
             ||||||||||||||||| ||||||||||||||||||||||||||||||||||||||||||
Sbjct  363   TCAGTAGATTTAGCTATCTTTTCTCTTCATCTTGCTGGTGCTTCTTCAATTTTAGGTGCA  422

Query  437   ATTAACTTTATTACAACTATTGTAAACATACGATCAATTGGAATAACAATAGAACGTATA  496
             ||||||||||||||||||||||||||||||||||||||||||||||||||||||||||||
Sbjct  423   ATTAACTTTATTACAACTATTGTAAACATACGATCAATTGGAATAACAATAGAACGTATA  482

Query  497   CCATTATTTGTTTGATCTGTTTTAATTACAGCTATTTTATTACTTTTATCTTTACCTGTA  556
             ||||||||||||||||||||||||||||||||||||||||||||||||||||||||||||
Sbjct  483   CCATTATTTGTTTGATCTGTTTTAATTACAGCTATTTTATTACTTTTATCTTTACCTGTA  542

Query  557   TTAGCAGGTGCCATTACAATATTGTTAACTGATCGAAACTTTAATACATCATTTTTTGAT  616
             ||||||||||||||||||||||||||||||||||||||||||||||||||||||||||||
Sbjct  543   TTAGCAGGTGCCATTACAATATTGTTAACTGATCGAAACTTTAATACATCATTTTTTGAT  602

Query  617   CCTTCAGGGGGAGGAGATCCAATTTTATATCAACATTTATTT  658
             ||||||||||||||||||||||||||||||||||||||||||
Sbjct  603   CCTTCAGGGGGAGGAGATCCAATTTTATATCAACATTTATTT  644
```

>Rhipicephalus sanguineus isolate LIC4736D cytochrome oxidase subunit 1 (COI) gene, partial cds; mitochondrial
Sequence ID: KX383809.1 Length: 762
Range 1: 1 to 636

Score:1164 bits(630), Expect:0.0,
Identities:634/636(99%), Gaps:0/636(0%), Strand: Plus/Plus

```
Query  23    GCATGATCAGGTATATTAGGACTTAGTATAAGAATATTAATTCGTATAGAATTAGGACAA  82
             ||||||||||||||||||||||||||||||||||||||||||||||||||||||||||||
Sbjct  1     GCATGATCAGGTATATTAGGACTTAGTATAAGAATATTAATTCGTATAGAATTAGGACAA  60

Query  83    CCTGGAACTTTAATTGGAAATGATCAGATTTATAATGTAATCGTAACAGCACATGCATTT  142
```

```
                ||||||||||||||||||||||||||||||||||||||||||||||||||||||||||||
Sbjct  61       CCTGGAACTTTAATTGGAAATGATCAGATTTATAATGTAATCGTAACAGCACATGCATTT  120

Query  143      ATTATAAttttttttATAGTTATACCAATCATAATCGGAGGATTTGGAAATTGATTAGTC  202
                ||||||||||||||||||||||||||||||||||||||||||||||||||||||||||||
Sbjct  121      ATTATAATTTTTTTTATAGTTATACCAATCATAATCGGAGGATTTGGAAATTGATTAGTC  180

Query  203      CCTATCATATTGGGTGCCCCAGATATAGCATTTCCACGAATAAATAATATAAGATTTTGA  262
                ||||||||||||||||||||||||||||||||||||||||||||||||||||||||||||
Sbjct  181      CCTATCATATTGGGTGCCCCAGATATAGCATTTCCACGAATAAATAATATAAGATTTTGA  240

Query  263      CTTTTACCTCCTTCACTATTTTTATTAATTAACTCTTCATTAATTGAATCTGGAGCTGGT  322
                ||||||||||||||||||||||||||||||||||||||||||||||||||||||||||||
Sbjct  241      CTTTTACCTCCTTCACTATTTTTATTAATTAACTCTTCATTAATTGAATCTGGAGCTGGT  300

Query  323      ACAGGATGAACAGTTTACCCTCCTCTATCTTCAAATTTATCACATTATGGCCCTTCAGTA  382
                ||||||||||||||||||||||||||||||||||||||||||||||||||||||||||||
Sbjct  301      ACAGGATGAACAGTTTACCCTCCTCTATCTTCAAATTTATCACATTATGGCCCTTCAGTA  360

Query  383      GATTTAGCTATTTTTTCTCTTCATCTTGCTGGTGCTTCTTCAATTTTAGGTGCAATTAAC  442
                ||||||||||| ||||||||||||||||||||||||||||||||||||||||||||||||
Sbjct  361      GATTTAGCTATCTTTTCTCTTCATCTTGCTGGTGCTTCTTCAATTTTAGGTGCAATTAAC  420

Query  443      TTTATTACAACTATTGTAAACATACGATCAATTGGAATAACAATAGAACGTATACCATTA  502
                |||||||||||||||||||||||||| |||||||||||||||||||||||||||||||||
Sbjct  421      TTTATTACAACTATTGTAAACATACGGTCAATTGGAATAACAATAGAACGTATACCATTA  480

Query  503      TTTGTTTGATCTGTTTTAATTACAGCTATTTTATTACTTTTATCTTTACCTGTATTAGCA  562
                ||||||||||||||||||||||||||||||||||||||||||||||||||||||||||||
Sbjct  481      TTTGTTTGATCTGTTTTAATTACAGCTATTTTATTACTTTTATCTTTACCTGTATTAGCA  540

Query  563      GGTGCCATTACAATATTGTTAACTGATCGAAACTTTAATACATCATTTTTTGATCCTTCA  622
                ||||||||||||||||||||||||||||||||||||||||||||||||||||||||||||
Sbjct  541      GGTGCCATTACAATATTGTTAACTGATCGAAACTTTAATACATCATTTTTTGATCCTTCA  600

Query  623      GGGGGAGGAGATCCAATTTTATATCAACATTTATTT  658
                ||||||||||||||||||||||||||||||||||||
Sbjct  601      GGGGGAGGAGATCCAATTTTATATCAACATTTATTT  636
```

>Rhipicephalus sanguineus isolate LIC4727D cytochrome oxidase subunit 1 (COI) gene, partial cds; mitochondrial
Sequence ID: KX383806.1 Length: 714
Range 1: 1 to 636

Score:1164 bits(630), Expect:0.0,
Identities:634/636(99%), Gaps:0/636(0%), Strand: Plus/Plus

```
Query  23       GCATGATCAGGTATATTAGGACTTAGTATAAGAATATTAATTCGTATAGAATTAGGACAA  82
                ||||||||||||||||||||||||||||||||||||||||||||||||||||||||||||
Sbjct  1        GCATGATCAGGTATATTAGGACTTAGTATAAGAATATTAATTCGTATAGAATTAGGACAA  60
```

```
Query  83    CCTGGAACTTTAATTGGAAATGATCAGATTTATAATGTAATCGTAACAGCACATGCATTT  142
             ||||||||||||||||||||||||||||||||||||||||||||||||||||||||||||
Sbjct  61    CCTGGAACTTTAATTGGAAATGATCAGATTTATAATGTAATCGTAACAGCACATGCATTT  120

Query  143   ATTATAAttttttttATAGTTATACCAATCATAATCGGAGGATTTGGAAATTGATTAGTC  202
             ||||||||||||||||||||||||||||||||||||||||||||||||||||||||||||
Sbjct  121   ATTATAATTTTTTTTATAGTTATACCAATCATAATCGGAGGATTTGGAAATTGATTAGTC  180

Query  203   CCTATCATATTGGGTGCCCCAGATATAGCATTTCCACGAATAAATAATATAAGATTTTGA  262
             ||||||||||||||||||||||||||||||||||||||||||||||||||||||||||||
Sbjct  181   CCTATCATATTGGGTGCCCCAGATATAGCATTTCCACGAATAAATAATATAAGATTTTGA  240

Query  263   CTTTTACCTCCTTCACTATTTTTATTAATTAACTCTTCATTAATTGAATCTGGAGCTGGT  322
             ||||||||||||||||||||||||||||||||||||||||||||||||||||||||||||
Sbjct  241   CTTTTACCTCCTTCACTATTTTTATTAATTAACTCTTCATTAATTGAATCTGGAGCTGGT  300

Query  323   ACAGGATGAACAGTTTACCCTCCTCTATCTTCAAATTTATCACATTATGGCCCTTCAGTA  382
             ||||||||||||||||||||||||||||||||||||||||||||||||||||||||||||
Sbjct  301   ACAGGATGAACAGTTTACCCTCCTCTATCTTCAAATTTATCACATTATGGCCCTTCAGTA  360

Query  383   GATTTAGCTATTTTTTCTCTTCATCTTGCTGGTGCTTCTTCAATTTTAGGTGCAATTAAC  442
             ||||||||||| ||||||||||||||||||||||||||||||||||||||||||||||||
Sbjct  361   GATTTAGCTATCTTTTCTCTTCATCTTGCTGGTGCTTCTTCAATTTTAGGTGCAATTAAC  420

Query  443   TTTATTACAACTATTGTAAACATACGATCAATTGGAATAACAATAGAACGTATACCATTA  502
             |||||||||||||||||||||||||| |||||||||||||||||||||||||||||||||
Sbjct  421   TTTATTACAACTATTGTAAACATACGGTCAATTGGAATAACAATAGAACGTATACCATTA  480

Query  503   TTTGTTTGATCTGTTTTAATTACAGCTATTTTATTACTTTTATCTTTACCTGTATTAGCA  562
             ||||||||||||||||||||||||||||||||||||||||||||||||||||||||||||
Sbjct  481   TTTGTTTGATCTGTTTTAATTACAGCTATTTTATTACTTTTATCTTTACCTGTATTAGCA  540

Query  563   GGTGCCATTACAATATTGTTAACTGATCGAAACTTTAATACATCATTTTTTGATCCTTCA  622
             ||||||||||||||||||||||||||||||||||||||||||||||||||||||||||||
Sbjct  541   GGTGCCATTACAATATTGTTAACTGATCGAAACTTTAATACATCATTTTTTGATCCTTCA  600

Query  623   GGGGGAGGAGATCCAATTTTATATCAACATTTATTT  658
             ||||||||||||||||||||||||||||||||||||
Sbjct  601   GGGGGAGGAGATCCAATTTTATATCAACATTTATTT  636
```

>Rhipicephalus sanguineus isolate LIC4727C cytochrome oxidase subunit 1 (COI) gene, partial cds; mitochondrial
Sequence ID: KX383805.1 Length: 761
Range 1: 1 to 635

Score:1162 bits(629), Expect:0.0,
Identities:633/635(99%), Gaps:0/635(0%), Strand: Plus/Plus

```
Query  24    CATGATCAGGTATATTAGGACTTAGTATAAGAATATTAATTCGTATAGAATTAGGACAAC  83
             ||||||||||||||||||||||||||||||||||||||||||||||||||||||||||||
Sbjct  1     CATGATCAGGTATATTAGGACTTAGTATAAGAATATTAATTCGTATAGAATTAGGACAAC  60
```

```
Query  84   CTGGAACTTTAATTGGAAATGATCAGATTTATAATGTAATCGTAACAGCACATGCATTTA  143
            ||||||||||||||||||||||||||||||||||||||||||||||||||||||||||||
Sbjct  61   CTGGAACTTTAATTGGAAATGATCAGATTTATAATGTAATCGTAACAGCACATGCATTTA  120

Query  144  TTATAAtttttttATAGTTATACCAATCATAATCGGAGGATTTGGAAATTGATTAGTCC  203
            ||||||||||||||||||||||||||||||||||||||||||||||||||||||||||||
Sbjct  121  TTATAATTTTTTTATAGTTATACCAATCATAATCGGAGGATTTGGAAATTGATTAGTCC  180

Query  204  CTATCATATTGGGTGCCCCAGATATAGCATTTCCACGAATAAATAATATAAGATTTTGAC  263
            ||||||||||||||||||||||||||||||||||||||||||||||||||||||||||||
Sbjct  181  CTATCATATTGGGTGCCCCAGATATAGCATTTCCACGAATAAATAATATAAGATTTTGAC  240

Query  264  TTTTACCTCCTTCACTATTTTTATTAATTAACTCTTCATTAATTGAATCTGGAGCTGGTA  323
            ||||||||||||||||||||||||||||||||||||||||||||||||||||||||||||
Sbjct  241  TTTTACCTCCTTCACTATTTTTATTAATTAACTCTTCATTAATTGAATCTGGAGCTGGTA  300

Query  324  CAGGATGAACAGTTTACCCTCCTCTATCTTCAAATTTATCACATTATGGCCCTTCAGTAG  383
            ||||||||||||||||||||||||||||||||||||||||||||||||||||||||||||
Sbjct  301  CAGGATGAACAGTTTACCCTCCTCTATCTTCAAATTTATCACATTATGGCCCTTCAGTAG  360

Query  384  ATTTAGCTATTTTTTCTCTTCATCTTGCTGGTGCTTCTTCAATTTTAGGTGCAATTAACT  443
            |||||||||| |||||||||||||||||||||||||||||||||||||||||||||||||
Sbjct  361  ATTTAGCTATCTTTTCTCTTCATCTTGCTGGTGCTTCTTCAATTTTAGGTGCAATTAACT  420

Query  444  TTATTACAACTATTGTAAACATACGATCAATTGGAATAACAATAGAACGTATACCATTAT  503
            ||||||||||||||||||||||||| ||||||||||||||||||||||||||||||||||
Sbjct  421  TTATTACAACTATTGTAAACATACGGTCAATTGGAATAACAATAGAACGTATACCATTAT  480

Query  504  TTGTTTGATCTGTTTTAATTACAGCTATTTTATTACTTTTATCTTTACCTGTATTAGCAG  563
            ||||||||||||||||||||||||||||||||||||||||||||||||||||||||||||
Sbjct  481  TTGTTTGATCTGTTTTAATTACAGCTATTTTATTACTTTTATCTTTACCTGTATTAGCAG  540

Query  564  GTGCCATTACAATATTGTTAACTGATCGAAACTTTAATACATCATTTTTTGATCCTTCAG  623
            ||||||||||||||||||||||||||||||||||||||||||||||||||||||||||||
Sbjct  541  GTGCCATTACAATATTGTTAACTGATCGAAACTTTAATACATCATTTTTTGATCCTTCAG  600

Query  624  GGGGAGGAGATCCAATTTTATATCAACATTTATTT  658
            |||||||||||||||||||||||||||||||||||
Sbjct  601  GGGGAGGAGATCCAATTTTATATCAACATTTATTT  635
```

>Rhipicephalus sanguineus sensu lato 'temperate lineage' isolate SF3003
cytochrome c oxidase subunit I (COI) gene, partial cds; mitochondrial
Sequence ID: MF425988.1 Length: 654
Range 1: 1 to 640

Score:1160 bits(628), Expect:0.0,
Identities:636/640(99%), Gaps:0/640(0%), Strand: Plus/Plus

```
Query  17   TTTGGAGCATGATCAGGTATATTAGGACTTAGTATAAGAATATTAATTCGTATAGAATTA  76
            ||||||||||||||||||||||||||||||||||||||||||||||||||||||||||||
```

```
Sbjct  1    TTTGGAGCATGATCAGGTATATTAGGACTTAGTATAAGAATATTAATTCGTATAGAATTA  60

Query  77   GGACAACCTGGAACTTTAATTGGAAATGATCAGATTTATAATGTAATCGTAACAGCACAT  136
            ||||||||||||||||||||||||||||||||||||||||||||||||||||||||||||
Sbjct  61   GGACAACCTGGAACTTTAATTGGAAATGATCAGATTTATAATGTAATCGTAACAGCACAT  120

Query  137  GCATTTATTATAAttttttttATAGTTATACCAATCATAATCGGAGGATTTGGAAATTGA  196
            ||||||||||||||||||||||||||||||||||||||||||||||||||||||||||||
Sbjct  121  GCATTTATTATAATTTTTTTTATAGTTATACCAATCATAATCGGAGGATTTGGAAATTGA  180

Query  197  TTAGTCCCTATCATATTGGGTGCCCCAGATATAGCATTTCCACGAATAAATAATATAAGA  256
            ||||||||||||||||| ||||||||||||||||||||||||||||||||||||||||||
Sbjct  181  TTAGTCCCTATCATATTAGGTGCCCCAGATATAGCATTTCCACGAATAAATAATATAAGA  240

Query  257  TTTTGACTTTTACCTCCTTCACTATTTTTATTAATTAACTCTTCATTAATTGAATCTGGA  316
            ||||||||||||||||||||||||||||||||||||||||||||||||||||||||||||
Sbjct  241  TTTTGACTTTTACCTCCTTCACTATTTTTATTAATTAACTCTTCATTAATTGAATCTGGA  300

Query  317  GCTGGTACAGGATGAACAGTTTACCCTCCTCTATCTTCAAATTTATCACATTATGGCCCT  376
            ||||||||||||||||||||||||||||||||||||||||||||||||||||||||||||
Sbjct  301  GCTGGTACAGGATGAACAGTTTACCCTCCTCTATCTTCAAATTTATCACATTATGGCCCT  360

Query  377  TCAGTAGATTTAGCTATTTTTTCTCTTCATCTTGCTGGTGCTTCTTCAATTTTAGGTGCA  436
            ||||||||||||||||| ||||||||||||||||||||||||||||||||||||||||||
Sbjct  361  TCAGTAGATTTAGCTATCTTTTCTCTTCATCTTGCTGGTGCTTCTTCAATTTTAGGTGCA  420

Query  437  ATTAACTTTATTACAACTATTGTAAACATACGATCAATTGGAATAACAATAGAACGTATA  496
            ||||||||||||||||||||||||||||||||||||||||||||||||||||||||||||
Sbjct  421  ATTAACTTTATTACAACTATTGTAAACATACGATCAATTGGAATAACAATAGAACGTATA  480

Query  497  CCATTATTTGTTTGATCTGTTTTAATTACAGCTATTTTATTACTTTTATCTTTACCTGTA  556
            ||||||||||||||||||||||||||||||||||||||||||||||||||||||||||||
Sbjct  481  CCATTATTTGTTTGATCTGTTTTAATTACAGCTATTTTATTACTTTTATCTTTACCTGTA  540

Query  557  TTAGCAGGTGCCATTACAATATTGTTAACTGATCGAAACTTTAATACATCATTTTTTGAT  616
            ||||||||||||||||||||||||||||||||||||||||||||||||||||||||||||
Sbjct  541  TTAGCAGGTGCCATTACAATATTGTTAACTGATCGAAACTTTAATACATCATTTTTTGAT  600

Query  617  CCTTCAGGGGGAGGAGATCCAATTTTATATCAACATTTAT  656
            ||||||||||||||| |||||||||||||||| |||||||
Sbjct  601  CCTTCAGGGGGAGGACATCCAATTTTATATCACCATTTAT  640


>Rhipicephalus sanguineus clone #7 cytochrome oxidase subunit I (COI) gene,
partial cds; mitochondrial
Sequence ID: KX519712.1 Length: 654
Range 1: 3 to 628

Score:1157 bits(626), Expect:0.0,
Identities:626/626(100%),  Gaps:0/626(0%), Strand: Plus/Plus

Query  33   GTATATTAGGACTTAGTATAAGAATATTAATTCGTATAGAATTAGGACAACCTGGAACTT  92
```

```
             ||||||||||||||||||||||||||||||||||||||||||||||||||||||||||||
Sbjct  3     GTATATTAGGACTTAGTATAAGAATATTAATTCGTATAGAATTAGGACAACCTGGAACTT  62

Query  93    TAATTGGAAATGATCAGATTTATAATGTAATCGTAACAGCACATGCATTTATTATAAttt  152
             ||||||||||||||||||||||||||||||||||||||||||||||||||||||||||||
Sbjct  63    TAATTGGAAATGATCAGATTTATAATGTAATCGTAACAGCACATGCATTTATTATAATTT  122

Query  153   tttttATAGTTATACCAATCATAATCGGAGGATTTGGAAATTGATTAGTCCCTATCATAT  212
             ||||||||||||||||||||||||||||||||||||||||||||||||||||||||||||
Sbjct  123   TTTTTATAGTTATACCAATCATAATCGGAGGATTTGGAAATTGATTAGTCCCTATCATAT  182

Query  213   TGGGTGCCCCAGATATAGCATTTCCACGAATAAATAATATAAGATTTTGACTTTTACCTC  272
             ||||||||||||||||||||||||||||||||||||||||||||||||||||||||||||
Sbjct  183   TGGGTGCCCCAGATATAGCATTTCCACGAATAAATAATATAAGATTTTGACTTTTACCTC  242

Query  273   CTTCACTATTTTTATTAATTAACTCTTCATTAATTGAATCTGGAGCTGGTACAGGATGAA  332
             ||||||||||||||||||||||||||||||||||||||||||||||||||||||||||||
Sbjct  243   CTTCACTATTTTTATTAATTAACTCTTCATTAATTGAATCTGGAGCTGGTACAGGATGAA  302

Query  333   CAGTTTACCCTCCTCTATCTTCAAATTTATCACATTATGGCCCTTCAGTAGATTTAGCTA  392
             ||||||||||||||||||||||||||||||||||||||||||||||||||||||||||||
Sbjct  303   CAGTTTACCCTCCTCTATCTTCAAATTTATCACATTATGGCCCTTCAGTAGATTTAGCTA  362

Query  393   TTTTTTCTCTTCATCTTGCTGGTGCTTCTTCAATTTTAGGTGCAATTAACTTTATTACAA  452
             ||||||||||||||||||||||||||||||||||||||||||||||||||||||||||||
Sbjct  363   TTTTTTCTCTTCATCTTGCTGGTGCTTCTTCAATTTTAGGTGCAATTAACTTTATTACAA  422

Query  453   CTATTGTAAACATACGATCAATTGGAATAACAATAGAACGTATACCATTATTTGTTTGAT  512
             ||||||||||||||||||||||||||||||||||||||||||||||||||||||||||||
Sbjct  423   CTATTGTAAACATACGATCAATTGGAATAACAATAGAACGTATACCATTATTTGTTTGAT  482

Query  513   CTGTTTTAATTACAGCTATTTTATTACTTTTATCTTTACCTGTATTAGCAGGTGCCATTA  572
             ||||||||||||||||||||||||||||||||||||||||||||||||||||||||||||
Sbjct  483   CTGTTTTAATTACAGCTATTTTATTACTTTTATCTTTACCTGTATTAGCAGGTGCCATTA  542

Query  573   CAATATTGTTAACTGATCGAAACTTTAATACATCATTTTTTGATCCTTCAGGGGGAGGAG  632
             ||||||||||||||||||||||||||||||||||||||||||||||||||||||||||||
Sbjct  543   CAATATTGTTAACTGATCGAAACTTTAATACATCATTTTTTGATCCTTCAGGGGGAGGAG  602

Query  633   ATCCAATTTTATATCAACATTTATTT  658
             ||||||||||||||||||||||||||
Sbjct  603   ATCCAATTTTATATCAACATTTATTT  628
```

>Rhipicephalus sanguineus sensu lato 'temperate lineage' isolate CR1575
cytochrome c oxidase subunit I (COI) gene, partial cds; mitochondrial
Sequence ID: MF426002.1 Length: 639
Range 1: 1 to 628

Score:1155 bits(625), Expect:0.0,
Identities:627/628(99%), Gaps:0/628(0%), Strand: Plus/Plus

```
Query  31   AGGTATATTAGGACTTAGTATAAGAATATTAATTCGTATAGAATTAGGACAACCTGGAAC  90
            ||||||||||||||||||||||||||||||||||||||||||||||||||||||||||||
Sbjct  1    AGGTATATTAGGACTTAGTATAAGAATATTAATTCGTATAGAATTAGGACAACCTGGAAC  60

Query  91   TTTAATTGGAAATGATCAGATTTATAATGTAATCGTAACAGCACATGCATTTATTATAAt  150
            ||||||||||||||||||||||||||||||||||||||||||||||||||||||||||||
Sbjct  61   TTTAATTGGAAATGATCAGATTTATAATGTAATCGTAACAGCACATGCATTTATTATAAT  120

Query  151  tttttttATAGTTATACCAATCATAATCGGAGGATTTGGAAATTGATTAGTCCCTATCAT  210
            ||||||||||||||||||||||||||||||||||||||||||||||||||||||||||||
Sbjct  121  TTTTTTTATAGTTATACCAATCATAATCGGAGGATTTGGAAATTGATTAGTCCCTATCAT  180

Query  211  ATTGGGTGCCCCAGATATAGCATTTCCACGAATAAATAATATAAGATTTTGACTTTTACC  270
            ||||||||||||||||||||||||||||||||||||||||||||||||||||||||||||
Sbjct  181  ATTGGGTGCCCCAGATATAGCATTTCCACGAATAAATAATATAAGATTTTGACTTTTACC  240

Query  271  TCCTTCACTATTTTTATTAATTAACTCTTCATTAATTGAATCTGGAGCTGGTACAGGATG  330
            ||||||||||||||||||||||||||||||||||||||||||||||||||||||||||||
Sbjct  241  TCCTTCACTATTTTTATTAATTAACTCTTCATTAATTGAATCTGGAGCTGGTACAGGATG  300

Query  331  AACAGTTTACCCTCCTCTATCTTCAAATTTATCACATTATGGCCCTTCAGTAGATTTAGC  390
            ||||||||||||||||||||||||||||||||||||||||||||||||||||||||||||
Sbjct  301  AACAGTTTACCCTCCTCTATCTTCAAATTTATCACATTATGGCCCTTCAGTAGATTTAGC  360

Query  391  TATTTTTTCTCTTCATCTTGCTGGTGCTTCTTCAATTTTAGGTGCAATTAACTTTATTAC  450
            ||| ||||||||||||||||||||||||||||||||||||||||||||||||||||||||
Sbjct  361  TATCTTTTCTCTTCATCTTGCTGGTGCTTCTTCAATTTTAGGTGCAATTAACTTTATTAC  420

Query  451  AACTATTGTAAACATACGATCAATTGGAATAACAATAGAACGTATACCATTATTTGTTTG  510
            ||||||||||||||||||||||||||||||||||||||||||||||||||||||||||||
Sbjct  421  AACTATTGTAAACATACGATCAATTGGAATAACAATAGAACGTATACCATTATTTGTTTG  480

Query  511  ATCTGTTTTAATTACAGCTATTTTATTACTTTTATCTTTACCTGTATTAGCAGGTGCCAT  570
            ||||||||||||||||||||||||||||||||||||||||||||||||||||||||||||
Sbjct  481  ATCTGTTTTAATTACAGCTATTTTATTACTTTTATCTTTACCTGTATTAGCAGGTGCCAT  540

Query  571  TACAATATTGTTAACTGATCGAAACTTTAATACATCATTTTTTGATCCTTCAGGGGGAGG  630
            ||||||||||||||||||||||||||||||||||||||||||||||||||||||||||||
Sbjct  541  TACAATATTGTTAACTGATCGAAACTTTAATACATCATTTTTTGATCCTTCAGGGGGAGG  600

Query  631  AGATCCAATTTTATATCAACATTTATTT  658
            ||||||||||||||||||||||||||||
Sbjct  601  AGATCCAATTTTATATCAACATTTATTT  628
```

>Rhipicephalus sanguineus isolate Rsan-ML4 cytochrome oxidase subunit I (cox1) gene, partial cds; mitochondrial
Sequence ID: MG855658.1 Length: 630
Range 1: 1 to 620

Score:1146 bits(620), Expect:0.0,
Identities:620/620(100%), Gaps:0/620(0%), Strand: Plus/Plus

```
Query  39   TAGGACTTAGTATAAGAATATTAATTCGTATAGAATTAGGACAACCTGGAACTTTAATTG  98
            ||||||||||||||||||||||||||||||||||||||||||||||||||||||||||||
Sbjct  1    TAGGACTTAGTATAAGAATATTAATTCGTATAGAATTAGGACAACCTGGAACTTTAATTG  60

Query  99   GAAATGATCAGATTTATAATGTAATCGTAACAGCACATGCATTTATTATAAtttttttA  158
            ||||||||||||||||||||||||||||||||||||||||||||||||||||||||||||
Sbjct  61   GAAATGATCAGATTTATAATGTAATCGTAACAGCACATGCATTTATTATAATTTTTTTA  120

Query  159  TAGTTATACCAATCATAATCGGAGGATTTGGAAATTGATTAGTCCCTATCATATTGGGTG  218
            ||||||||||||||||||||||||||||||||||||||||||||||||||||||||||||
Sbjct  121  TAGTTATACCAATCATAATCGGAGGATTTGGAAATTGATTAGTCCCTATCATATTGGGTG  180

Query  219  CCCCAGATATAGCATTTCCACGAATAAATAATATAAGATTTTGACTTTTACCTCCTTCAC  278
            ||||||||||||||||||||||||||||||||||||||||||||||||||||||||||||
Sbjct  181  CCCCAGATATAGCATTTCCACGAATAAATAATATAAGATTTTGACTTTTACCTCCTTCAC  240

Query  279  TATTTTTATTAATTAACTCTTCATTAATTGAATCTGGAGCTGGTACAGGATGAACAGTTT  338
            ||||||||||||||||||||||||||||||||||||||||||||||||||||||||||||
Sbjct  241  TATTTTTATTAATTAACTCTTCATTAATTGAATCTGGAGCTGGTACAGGATGAACAGTTT  300

Query  339  ACCCTCCTCTATCTTCAAATTTATCACATTATGGCCCTTCAGTAGATTTAGCTATTTTTT  398
            ||||||||||||||||||||||||||||||||||||||||||||||||||||||||||||
Sbjct  301  ACCCTCCTCTATCTTCAAATTTATCACATTATGGCCCTTCAGTAGATTTAGCTATTTTTT  360

Query  399  CTCTTCATCTTGCTGGTGCTTCTTCAATTTTAGGTGCAATTAACTTTATTACAACTATTG  458
            ||||||||||||||||||||||||||||||||||||||||||||||||||||||||||||
Sbjct  361  CTCTTCATCTTGCTGGTGCTTCTTCAATTTTAGGTGCAATTAACTTTATTACAACTATTG  420

Query  459  TAAACATACGATCAATTGGAATAACAATAGAACGTATACCATTATTTGTTTGATCTGTTT  518
            ||||||||||||||||||||||||||||||||||||||||||||||||||||||||||||
Sbjct  421  TAAACATACGATCAATTGGAATAACAATAGAACGTATACCATTATTTGTTTGATCTGTTT  480

Query  519  TAATTACAGCTATTTTATTACTTTTATCTTTACCTGTATTAGCAGGTGCCATTACAATAT  578
            ||||||||||||||||||||||||||||||||||||||||||||||||||||||||||||
Sbjct  481  TAATTACAGCTATTTTATTACTTTTATCTTTACCTGTATTAGCAGGTGCCATTACAATAT  540

Query  579  TGTTAACTGATCGAAACTTTAATACATCATTTTTTGATCCTTCAGGGGGAGGAGATCCAA  638
            ||||||||||||||||||||||||||||||||||||||||||||||||||||||||||||
Sbjct  541  TGTTAACTGATCGAAACTTTAATACATCATTTTTTGATCCTTCAGGGGGAGGAGATCCAA  600

Query  639  TTTTATATCAACATTTATTT  658
            ||||||||||||||||||||
Sbjct  601  TTTTATATCAACATTTATTT  620
```

>Rhipicephalus sanguineus isolate Rsan-ML3 cytochrome oxidase subunit I (cox1) gene, partial cds; mitochondrial
Sequence ID: MG855657.1 Length: 630
Range 1: 1 to 620

Score:1146 bits(620), Expect:0.0,

```
Identities:620/620(100%),  Gaps:0/620(0%), Strand: Plus/Plus

Query  39   TAGGACTTAGTATAAGAATATTAATTCGTATAGAATTAGGACAACCTGGAACTTTAATTG  98
            ||||||||||||||||||||||||||||||||||||||||||||||||||||||||||||
Sbjct  1    TAGGACTTAGTATAAGAATATTAATTCGTATAGAATTAGGACAACCTGGAACTTTAATTG  60

Query  99   GAAATGATCAGATTTATAATGTAATCGTAACAGCACATGCATTTATTATAAtttttttA  158
            ||||||||||||||||||||||||||||||||||||||||||||||||||||||||||||
Sbjct  61   GAAATGATCAGATTTATAATGTAATCGTAACAGCACATGCATTTATTATAATTTTTTTA  120

Query  159  TAGTTATACCAATCATAATCGGAGGATTTGGAAATTGATTAGTCCCTATCATATTGGGTG  218
            ||||||||||||||||||||||||||||||||||||||||||||||||||||||||||||
Sbjct  121  TAGTTATACCAATCATAATCGGAGGATTTGGAAATTGATTAGTCCCTATCATATTGGGTG  180

Query  219  CCCCAGATATAGCATTTCCACGAATAAATAATATAAGATTTTGACTTTTACCTCCTTCAC  278
            ||||||||||||||||||||||||||||||||||||||||||||||||||||||||||||
Sbjct  181  CCCCAGATATAGCATTTCCACGAATAAATAATATAAGATTTTGACTTTTACCTCCTTCAC  240

Query  279  TATTTTTATTAATTAACTCTTCATTAATTGAATCTGGAGCTGGTACAGGATGAACAGTTT  338
            ||||||||||||||||||||||||||||||||||||||||||||||||||||||||||||
Sbjct  241  TATTTTTATTAATTAACTCTTCATTAATTGAATCTGGAGCTGGTACAGGATGAACAGTTT  300

Query  339  ACCCTCCTCTATCTTCAAATTTATCACATTATGGCCCTTCAGTAGATTTAGCTATTTTTT  398
            ||||||||||||||||||||||||||||||||||||||||||||||||||||||||||||
Sbjct  301  ACCCTCCTCTATCTTCAAATTTATCACATTATGGCCCTTCAGTAGATTTAGCTATTTTTT  360

Query  399  CTCTTCATCTTGCTGGTGCTTCTTCAATTTTAGGTGCAATTAACTTTATTACAACTATTG  458
            ||||||||||||||||||||||||||||||||||||||||||||||||||||||||||||
Sbjct  361  CTCTTCATCTTGCTGGTGCTTCTTCAATTTTAGGTGCAATTAACTTTATTACAACTATTG  420

Query  459  TAAACATACGATCAATTGGAATAACAATAGAACGTATACCATTATTTGTTTGATCTGTTT  518
            ||||||||||||||||||||||||||||||||||||||||||||||||||||||||||||
Sbjct  421  TAAACATACGATCAATTGGAATAACAATAGAACGTATACCATTATTTGTTTGATCTGTTT  480

Query  519  TAATTACAGCTATTTTATTACTTTTATCTTTACCTGTATTAGCAGGTGCCATTACAATAT  578
            ||||||||||||||||||||||||||||||||||||||||||||||||||||||||||||
Sbjct  481  TAATTACAGCTATTTTATTACTTTTATCTTTACCTGTATTAGCAGGTGCCATTACAATAT  540

Query  579  TGTTAACTGATCGAAACTTTAATACATCATTTTTTGATCCTTCAGGGGGAGGAGATCCAA  638
            ||||||||||||||||||||||||||||||||||||||||||||||||||||||||||||
Sbjct  541  TGTTAACTGATCGAAACTTTAATACATCATTTTTTGATCCTTCAGGGGGAGGAGATCCAA  600

Query  639  TTTTATATCAACATTTATTT  658
            ||||||||||||||||||||
Sbjct  601  TTTTATATCAACATTTATTT  620
```

>Rhipicephalus sanguineus isolate Rsan-ML2 cytochrome oxidase subunit I (cox1) gene, partial cds; mitochondrial
Sequence ID: MG855656.1 Length: 630
Range 1: 1 to 620

```
Score:1146 bits(620), Expect:0.0,
Identities:620/620(100%),  Gaps:0/620(0%), Strand: Plus/Plus

Query  39   TAGGACTTAGTATAAGAATATTAATTCGTATAGAATTAGGACAACCTGGAACTTTAATTG  98
            ||||||||||||||||||||||||||||||||||||||||||||||||||||||||||||
Sbjct  1    TAGGACTTAGTATAAGAATATTAATTCGTATAGAATTAGGACAACCTGGAACTTTAATTG  60

Query  99   GAAATGATCAGATTTATAATGTAATCGTAACAGCACATGCATTTATTATAAtttttttA  158
            ||||||||||||||||||||||||||||||||||||||||||||||||||||||||||||
Sbjct  61   GAAATGATCAGATTTATAATGTAATCGTAACAGCACATGCATTTATTATAATTTTTTTA  120

Query  159  TAGTTATACCAATCATAATCGGAGGATTTGGAAATTGATTAGTCCCTATCATATTGGGTG  218
            ||||||||||||||||||||||||||||||||||||||||||||||||||||||||||||
Sbjct  121  TAGTTATACCAATCATAATCGGAGGATTTGGAAATTGATTAGTCCCTATCATATTGGGTG  180

Query  219  CCCCAGATATAGCATTTCCACGAATAAATAATATAAGATTTTGACTTTTACCTCCTTCAC  278
            ||||||||||||||||||||||||||||||||||||||||||||||||||||||||||||
Sbjct  181  CCCCAGATATAGCATTTCCACGAATAAATAATATAAGATTTTGACTTTTACCTCCTTCAC  240

Query  279  TATTTTTATTAATTAACTCTTCATTAATTGAATCTGGAGCTGGTACAGGATGAACAGTTT  338
            ||||||||||||||||||||||||||||||||||||||||||||||||||||||||||||
Sbjct  241  TATTTTTATTAATTAACTCTTCATTAATTGAATCTGGAGCTGGTACAGGATGAACAGTTT  300

Query  339  ACCCTCCTCTATCTTCAAATTTATCACATTATGGCCCTTCAGTAGATTTAGCTATTTTTT  398
            ||||||||||||||||||||||||||||||||||||||||||||||||||||||||||||
Sbjct  301  ACCCTCCTCTATCTTCAAATTTATCACATTATGGCCCTTCAGTAGATTTAGCTATTTTTT  360

Query  399  CTCTTCATCTTGCTGGTGCTTCTTCAATTTTAGGTGCAATTAACTTTATTACAACTATTG  458
            ||||||||||||||||||||||||||||||||||||||||||||||||||||||||||||
Sbjct  361  CTCTTCATCTTGCTGGTGCTTCTTCAATTTTAGGTGCAATTAACTTTATTACAACTATTG  420

Query  459  TAAACATACGATCAATTGGAATAACAATAGAACGTATACCATTATTTGTTTGATCTGTTT  518
            ||||||||||||||||||||||||||||||||||||||||||||||||||||||||||||
Sbjct  421  TAAACATACGATCAATTGGAATAACAATAGAACGTATACCATTATTTGTTTGATCTGTTT  480

Query  519  TAATTACAGCTATTTTATTACTTTTATCTTTACCTGTATTAGCAGGTGCCATTACAATAT  578
            ||||||||||||||||||||||||||||||||||||||||||||||||||||||||||||
Sbjct  481  TAATTACAGCTATTTTATTACTTTTATCTTTACCTGTATTAGCAGGTGCCATTACAATAT  540

Query  579  TGTTAACTGATCGAAACTTTAATACATCATTTTTTGATCCTTCAGGGGGAGGAGATCCAA  638
            ||||||||||||||||||||||||||||||||||||||||||||||||||||||||||||
Sbjct  541  TGTTAACTGATCGAAACTTTAATACATCATTTTTTGATCCTTCAGGGGGAGGAGATCCAA  600

Query  639  TTTTATATCAACATTTATTT  658
            ||||||||||||||||||||
Sbjct  601  TTTTATATCAACATTTATTT  620
```

>Rhipicephalus sanguineus isolate Kehf-Lagareb-A cytochrome oxidase subunit I (COI) gene, partial cds; mitochondrial
Sequence ID: KX757910.1 Length: 630
Range 1: 1 to 620

Score:1146 bits(620), Expect:0.0,
Identities:620/620(100%), Gaps:0/620(0%), Strand: Plus/Plus

```
Query  39   TAGGACTTAGTATAAGAATATTAATTCGTATAGAATTAGGACAACCTGGAACTTTAATTG  98
            ||||||||||||||||||||||||||||||||||||||||||||||||||||||||||||
Sbjct  1    TAGGACTTAGTATAAGAATATTAATTCGTATAGAATTAGGACAACCTGGAACTTTAATTG  60

Query  99   GAAATGATCAGATTTATAATGTAATCGTAACAGCACATGCATTTATTATAAtttttttA  158
            ||||||||||||||||||||||||||||||||||||||||||||||||||||||||||||
Sbjct  61   GAAATGATCAGATTTATAATGTAATCGTAACAGCACATGCATTTATTATAATTTTTTTA  120

Query  159  TAGTTATACCAATCATAATCGGAGGATTTGGAAATTGATTAGTCCCTATCATATTGGGTG  218
            ||||||||||||||||||||||||||||||||||||||||||||||||||||||||||||
Sbjct  121  TAGTTATACCAATCATAATCGGAGGATTTGGAAATTGATTAGTCCCTATCATATTGGGTG  180

Query  219  CCCCAGATATAGCATTTCCACGAATAAATAATATAAGATTTTGACTTTTACCTCCTTCAC  278
            ||||||||||||||||||||||||||||||||||||||||||||||||||||||||||||
Sbjct  181  CCCCAGATATAGCATTTCCACGAATAAATAATATAAGATTTTGACTTTTACCTCCTTCAC  240

Query  279  TATTTTTATTAATTAACTCTTCATTAATTGAATCTGGAGCTGGTACAGGATGAACAGTTT  338
            ||||||||||||||||||||||||||||||||||||||||||||||||||||||||||||
Sbjct  241  TATTTTTATTAATTAACTCTTCATTAATTGAATCTGGAGCTGGTACAGGATGAACAGTTT  300

Query  339  ACCCTCCTCTATCTTCAAATTTATCACATTATGGCCCTTCAGTAGATTTAGCTATTTTTT  398
            ||||||||||||||||||||||||||||||||||||||||||||||||||||||||||||
Sbjct  301  ACCCTCCTCTATCTTCAAATTTATCACATTATGGCCCTTCAGTAGATTTAGCTATTTTTT  360

Query  399  CTCTTCATCTTGCTGGTGCTTCTTCAATTTTAGGTGCAATTAACTTTATTACAACTATTG  458
            ||||||||||||||||||||||||||||||||||||||||||||||||||||||||||||
Sbjct  361  CTCTTCATCTTGCTGGTGCTTCTTCAATTTTAGGTGCAATTAACTTTATTACAACTATTG  420

Query  459  TAAACATACGATCAATTGGAATAACAATAGAACGTATACCATTATTTGTTTGATCTGTTT  518
            ||||||||||||||||||||||||||||||||||||||||||||||||||||||||||||
Sbjct  421  TAAACATACGATCAATTGGAATAACAATAGAACGTATACCATTATTTGTTTGATCTGTTT  480

Query  519  TAATTACAGCTATTTTATTACTTTTATCTTTACCTGTATTAGCAGGTGCCATTACAATAT  578
            ||||||||||||||||||||||||||||||||||||||||||||||||||||||||||||
Sbjct  481  TAATTACAGCTATTTTATTACTTTTATCTTTACCTGTATTAGCAGGTGCCATTACAATAT  540

Query  579  TGTTAACTGATCGAAACTTTAATACATCATTTTTTGATCCTTCAGGGGGAGGAGATCCAA  638
            ||||||||||||||||||||||||||||||||||||||||||||||||||||||||||||
Sbjct  541  TGTTAACTGATCGAAACTTTAATACATCATTTTTTGATCCTTCAGGGGGAGGAGATCCAA  600

Query  639  TTTTATATCAACATTTATTT  658
            ||||||||||||||||||||
Sbjct  601  TTTTATATCAACATTTATTT  620
```

>Rhipicephalus sanguineus isolate Italy-B cytochrome oxidase subunit I (COI) gene, partial cds; mitochondrial
Sequence ID: KX757904.1 Length: 630

Range 1: 1 to 620

Score:1146 bits(620), Expect:0.0,
Identities:620/620(100%), Gaps:0/620(0%), Strand: Plus/Plus

```
Query  39   TAGGACTTAGTATAAGAATATTAATTCGTATAGAATTAGGACAACCTGGAACTTTAATTG  98
            ||||||||||||||||||||||||||||||||||||||||||||||||||||||||||||
Sbjct  1    TAGGACTTAGTATAAGAATATTAATTCGTATAGAATTAGGACAACCTGGAACTTTAATTG  60

Query  99   GAAATGATCAGATTTATAATGTAATCGTAACAGCACATGCATTTATTATAAtttttttA  158
            ||||||||||||||||||||||||||||||||||||||||||||||||||||||||||||
Sbjct  61   GAAATGATCAGATTTATAATGTAATCGTAACAGCACATGCATTTATTATAATTTTTTTA  120

Query  159  TAGTTATACCAATCATAATCGGAGGATTTGGAAATTGATTAGTCCCTATCATATTGGGTG  218
            ||||||||||||||||||||||||||||||||||||||||||||||||||||||||||||
Sbjct  121  TAGTTATACCAATCATAATCGGAGGATTTGGAAATTGATTAGTCCCTATCATATTGGGTG  180

Query  219  CCCCAGATATAGCATTTCCACGAATAAATAATATAAGATTTTGACTTTTACCTCCTTCAC  278
            ||||||||||||||||||||||||||||||||||||||||||||||||||||||||||||
Sbjct  181  CCCCAGATATAGCATTTCCACGAATAAATAATATAAGATTTTGACTTTTACCTCCTTCAC  240

Query  279  TATTTTTATTAATTAACTCTTCATTAATTGAATCTGGAGCTGGTACAGGATGAACAGTTT  338
            ||||||||||||||||||||||||||||||||||||||||||||||||||||||||||||
Sbjct  241  TATTTTTATTAATTAACTCTTCATTAATTGAATCTGGAGCTGGTACAGGATGAACAGTTT  300

Query  339  ACCCTCCTCTATCTTCAAATTTATCACATTATGGCCCTTCAGTAGATTTAGCTATTTTTT  398
            ||||||||||||||||||||||||||||||||||||||||||||||||||||||||||||
Sbjct  301  ACCCTCCTCTATCTTCAAATTTATCACATTATGGCCCTTCAGTAGATTTAGCTATTTTTT  360

Query  399  CTCTTCATCTTGCTGGTGCTTCTTCAATTTTAGGTGCAATTAACTTTATTACAACTATTG  458
            ||||||||||||||||||||||||||||||||||||||||||||||||||||||||||||
Sbjct  361  CTCTTCATCTTGCTGGTGCTTCTTCAATTTTAGGTGCAATTAACTTTATTACAACTATTG  420

Query  459  TAAACATACGATCAATTGGAATAACAATAGAACGTATACCATTATTTGTTTGATCTGTTT  518
            ||||||||||||||||||||||||||||||||||||||||||||||||||||||||||||
Sbjct  421  TAAACATACGATCAATTGGAATAACAATAGAACGTATACCATTATTTGTTTGATCTGTTT  480

Query  519  TAATTACAGCTATTTTATTACTTTTATCTTTACCTGTATTAGCAGGTGCCATTACAATAT  578
            ||||||||||||||||||||||||||||||||||||||||||||||||||||||||||||
Sbjct  481  TAATTACAGCTATTTTATTACTTTTATCTTTACCTGTATTAGCAGGTGCCATTACAATAT  540

Query  579  TGTTAACTGATCGAAACTTTAATACATCATTTTTTGATCCTTCAGGGGGAGGAGATCCAA  638
            ||||||||||||||||||||||||||||||||||||||||||||||||||||||||||||
Sbjct  541  TGTTAACTGATCGAAACTTTAATACATCATTTTTTGATCCTTCAGGGGGAGGAGATCCAA  600

Query  639  TTTTATATCAACATTTATTT  658
            ||||||||||||||||||||
Sbjct  601  TTTTATATCAACATTTATTT  620
```

>Rhipicephalus sanguineus isolate Malta-A cytochrome oxidase subunit I (COI) gene, partial cds; mitochondrial

```
Sequence ID: KX757902.1 Length: 630
Range 1: 1 to 620

Score:1146 bits(620), Expect:0.0,
Identities:620/620(100%),  Gaps:0/620(0%), Strand: Plus/Plus

Query  39   TAGGACTTAGTATAAGAATATTAATTCGTATAGAATTAGGACAACCTGGAACTTTAATTG  98
            ||||||||||||||||||||||||||||||||||||||||||||||||||||||||||||
Sbjct  1    TAGGACTTAGTATAAGAATATTAATTCGTATAGAATTAGGACAACCTGGAACTTTAATTG  60

Query  99   GAAATGATCAGATTTATAATGTAATCGTAACAGCACATGCATTTATTATAAtttttttA  158
            ||||||||||||||||||||||||||||||||||||||||||||||||||||||||||||
Sbjct  61   GAAATGATCAGATTTATAATGTAATCGTAACAGCACATGCATTTATTATAATTTTTTTTA  120

Query  159  TAGTTATACCAATCATAATCGGAGGATTTGGAAATTGATTAGTCCCTATCATATTGGGTG  218
            ||||||||||||||||||||||||||||||||||||||||||||||||||||||||||||
Sbjct  121  TAGTTATACCAATCATAATCGGAGGATTTGGAAATTGATTAGTCCCTATCATATTGGGTG  180

Query  219  CCCCAGATATAGCATTTCCACGAATAAATAATATAAGATTTTGACTTTTACCTCCTTCAC  278
            ||||||||||||||||||||||||||||||||||||||||||||||||||||||||||||
Sbjct  181  CCCCAGATATAGCATTTCCACGAATAAATAATATAAGATTTTGACTTTTACCTCCTTCAC  240

Query  279  TATTTTTATTAATTAACTCTTCATTAATTGAATCTGGAGCTGGTACAGGATGAACAGTTT  338
            ||||||||||||||||||||||||||||||||||||||||||||||||||||||||||||
Sbjct  241  TATTTTTATTAATTAACTCTTCATTAATTGAATCTGGAGCTGGTACAGGATGAACAGTTT  300

Query  339  ACCCTCCTCTATCTTCAAATTTATCACATTATGGCCCTTCAGTAGATTTAGCTATTTTTT  398
            ||||||||||||||||||||||||||||||||||||||||||||||||||||||||||||
Sbjct  301  ACCCTCCTCTATCTTCAAATTTATCACATTATGGCCCTTCAGTAGATTTAGCTATTTTTT  360

Query  399  CTCTTCATCTTGCTGGTGCTTCTTCAATTTTAGGTGCAATTAACTTTATTACAACTATTG  458
            ||||||||||||||||||||||||||||||||||||||||||||||||||||||||||||
Sbjct  361  CTCTTCATCTTGCTGGTGCTTCTTCAATTTTAGGTGCAATTAACTTTATTACAACTATTG  420

Query  459  TAAACATACGATCAATTGGAATAACAATAGAACGTATACCATTATTTGTTTGATCTGTTT  518
            ||||||||||||||||||||||||||||||||||||||||||||||||||||||||||||
Sbjct  421  TAAACATACGATCAATTGGAATAACAATAGAACGTATACCATTATTTGTTTGATCTGTTT  480

Query  519  TAATTACAGCTATTTTATTACTTTTATCTTTACCTGTATTAGCAGGTGCCATTACAATAT  578
            ||||||||||||||||||||||||||||||||||||||||||||||||||||||||||||
Sbjct  481  TAATTACAGCTATTTTATTACTTTTATCTTTACCTGTATTAGCAGGTGCCATTACAATAT  540

Query  579  TGTTAACTGATCGAAACTTTAATACATCATTTTTTGATCCTTCAGGGGGAGGAGATCCAA  638
            ||||||||||||||||||||||||||||||||||||||||||||||||||||||||||||
Sbjct  541  TGTTAACTGATCGAAACTTTAATACATCATTTTTTGATCCTTCAGGGGGAGGAGATCCAA  600

Query  639  TTTTATATCAACATTTATTT  658
            ||||||||||||||||||||
Sbjct  601  TTTTATATCAACATTTATTT  620
```

>Rhipicephalus sanguineus isolate Zagreb-C cytochrome oxidase subunit I (COI)

gene, partial cds; mitochondrial
Sequence ID: KX757896.1 Length: 630
Range 1: 1 to 620

Score:1146 bits(620), Expect:0.0,
Identities:620/620(100%), Gaps:0/620(0%), Strand: Plus/Plus

```
Query  39   TAGGACTTAGTATAAGAATATTAATTCGTATAGAATTAGGACAACCTGGAACTTTAATTG  98
            ||||||||||||||||||||||||||||||||||||||||||||||||||||||||||||
Sbjct  1    TAGGACTTAGTATAAGAATATTAATTCGTATAGAATTAGGACAACCTGGAACTTTAATTG  60

Query  99   GAAATGATCAGATTTATAATGTAATCGTAACAGCACATGCATTTATTATAAtttttttA  158
            ||||||||||||||||||||||||||||||||||||||||||||||||||||||||||||
Sbjct  61   GAAATGATCAGATTTATAATGTAATCGTAACAGCACATGCATTTATTATAATTTTTTTA  120

Query  159  TAGTTATACCAATCATAATCGGAGGATTTGGAAATTGATTAGTCCCTATCATATTGGGTG  218
            ||||||||||||||||||||||||||||||||||||||||||||||||||||||||||||
Sbjct  121  TAGTTATACCAATCATAATCGGAGGATTTGGAAATTGATTAGTCCCTATCATATTGGGTG  180

Query  219  CCCCAGATATAGCATTTCCACGAATAAATAATATAAGATTTTGACTTTTACCTCCTTCAC  278
            ||||||||||||||||||||||||||||||||||||||||||||||||||||||||||||
Sbjct  181  CCCCAGATATAGCATTTCCACGAATAAATAATATAAGATTTTGACTTTTACCTCCTTCAC  240

Query  279  TATTTTTATTAATTAACTCTTCATTAATTGAATCTGGAGCTGGTACAGGATGAACAGTTT  338
            ||||||||||||||||||||||||||||||||||||||||||||||||||||||||||||
Sbjct  241  TATTTTTATTAATTAACTCTTCATTAATTGAATCTGGAGCTGGTACAGGATGAACAGTTT  300

Query  339  ACCCTCCTCTATCTTCAAATTTATCACATTATGGCCCTTCAGTAGATTTAGCTATTTTTT  398
            ||||||||||||||||||||||||||||||||||||||||||||||||||||||||||||
Sbjct  301  ACCCTCCTCTATCTTCAAATTTATCACATTATGGCCCTTCAGTAGATTTAGCTATTTTTT  360

Query  399  CTCTTCATCTTGCTGGTGCTTCTTCAATTTTAGGTGCAATTAACTTTATTACAACTATTG  458
            ||||||||||||||||||||||||||||||||||||||||||||||||||||||||||||
Sbjct  361  CTCTTCATCTTGCTGGTGCTTCTTCAATTTTAGGTGCAATTAACTTTATTACAACTATTG  420

Query  459  TAAACATACGATCAATTGGAATAACAATAGAACGTATACCATTATTTGTTTGATCTGTTT  518
            ||||||||||||||||||||||||||||||||||||||||||||||||||||||||||||
Sbjct  421  TAAACATACGATCAATTGGAATAACAATAGAACGTATACCATTATTTGTTTGATCTGTTT  480

Query  519  TAATTACAGCTATTTTATTACTTTTATCTTTACCTGTATTAGCAGGTGCCATTACAATAT  578
            ||||||||||||||||||||||||||||||||||||||||||||||||||||||||||||
Sbjct  481  TAATTACAGCTATTTTATTACTTTTATCTTTACCTGTATTAGCAGGTGCCATTACAATAT  540

Query  579  TGTTAACTGATCGAAACTTTAATACATCATTTTTTGATCCTTCAGGGGGAGGAGATCCAA  638
            ||||||||||||||||||||||||||||||||||||||||||||||||||||||||||||
Sbjct  541  TGTTAACTGATCGAAACTTTAATACATCATTTTTTGATCCTTCAGGGGGAGGAGATCCAA  600

Query  639  TTTTATATCAACATTTATTT  658
            ||||||||||||||||||||
Sbjct  601  TTTTATATCAACATTTATTT  620
```

```
>Rhipicephalus sanguineus isolate Pula-B cytochrome oxidase subunit I (COI)
gene, partial cds; mitochondrial
Sequence ID: KX757889.1 Length: 630
Range 1: 1 to 620

Score:1146 bits(620), Expect:0.0,
Identities:620/620(100%),  Gaps:0/620(0%), Strand: Plus/Plus

Query  39   TAGGACTTAGTATAAGAATATTAATTCGTATAGAATTAGGACAACCTGGAACTTTAATTG  98
            ||||||||||||||||||||||||||||||||||||||||||||||||||||||||||||
Sbjct  1    TAGGACTTAGTATAAGAATATTAATTCGTATAGAATTAGGACAACCTGGAACTTTAATTG  60

Query  99   GAAATGATCAGATTTATAATGTAATCGTAACAGCACATGCATTTATTATAAtttttttA  158
            ||||||||||||||||||||||||||||||||||||||||||||||||||||||||||||
Sbjct  61   GAAATGATCAGATTTATAATGTAATCGTAACAGCACATGCATTTATTATAATTTTTTTA  120

Query  159  TAGTTATACCAATCATAATCGGAGGATTTGGAAATTGATTAGTCCCTATCATATTGGGTG  218
            ||||||||||||||||||||||||||||||||||||||||||||||||||||||||||||
Sbjct  121  TAGTTATACCAATCATAATCGGAGGATTTGGAAATTGATTAGTCCCTATCATATTGGGTG  180

Query  219  CCCCAGATATAGCATTTCCACGAATAAATAATATAAGATTTTGACTTTTACCTCCTTCAC  278
            ||||||||||||||||||||||||||||||||||||||||||||||||||||||||||||
Sbjct  181  CCCCAGATATAGCATTTCCACGAATAAATAATATAAGATTTTGACTTTTACCTCCTTCAC  240

Query  279  TATTTTTATTAATTAACTCTTCATTAATTGAATCTGGAGCTGGTACAGGATGAACAGTTT  338
            ||||||||||||||||||||||||||||||||||||||||||||||||||||||||||||
Sbjct  241  TATTTTTATTAATTAACTCTTCATTAATTGAATCTGGAGCTGGTACAGGATGAACAGTTT  300

Query  339  ACCCTCCTCTATCTTCAAATTTATCACATTATGGCCCTTCAGTAGATTTAGCTATTTTTT  398
            ||||||||||||||||||||||||||||||||||||||||||||||||||||||||||||
Sbjct  301  ACCCTCCTCTATCTTCAAATTTATCACATTATGGCCCTTCAGTAGATTTAGCTATTTTTT  360

Query  399  CTCTTCATCTTGCTGGTGCTTCTTCAATTTTAGGTGCAATTAACTTTATTACAACTATTG  458
            ||||||||||||||||||||||||||||||||||||||||||||||||||||||||||||
Sbjct  361  CTCTTCATCTTGCTGGTGCTTCTTCAATTTTAGGTGCAATTAACTTTATTACAACTATTG  420

Query  459  TAAACATACGATCAATTGGAATAACAATAGAACGTATACCATTATTTGTTTGATCTGTTT  518
            ||||||||||||||||||||||||||||||||||||||||||||||||||||||||||||
Sbjct  421  TAAACATACGATCAATTGGAATAACAATAGAACGTATACCATTATTTGTTTGATCTGTTT  480

Query  519  TAATTACAGCTATTTTATTACTTTTATCTTTACCTGTATTAGCAGGTGCCATTACAATAT  578
            ||||||||||||||||||||||||||||||||||||||||||||||||||||||||||||
Sbjct  481  TAATTACAGCTATTTTATTACTTTTATCTTTACCTGTATTAGCAGGTGCCATTACAATAT  540

Query  579  TGTTAACTGATCGAAACTTTAATACATCATTTTTTGATCCTTCAGGGGGAGGAGATCCAA  638
            ||||||||||||||||||||||||||||||||||||||||||||||||||||||||||||
Sbjct  541  TGTTAACTGATCGAAACTTTAATACATCATTTTTTGATCCTTCAGGGGGAGGAGATCCAA  600

Query  639  TTTTATATCAACATTTATTT  658
            ||||||||||||||||||||
Sbjct  601  TTTTATATCAACATTTATTT  620
```

```
>Rhipicephalus sanguineus isolate Rovind cytochrome oxidase subunit I (COI)
gene, partial cds; mitochondrial
Sequence ID: KX757887.1 Length: 630
Range 1: 1 to 620

Score:1146 bits(620), Expect:0.0,
Identities:620/620(100%),  Gaps:0/620(0%), Strand: Plus/Plus

Query  39   TAGGACTTAGTATAAGAATATTAATTCGTATAGAATTAGGACAACCTGGAACTTTAATTG  98
            ||||||||||||||||||||||||||||||||||||||||||||||||||||||||||||
Sbjct  1    TAGGACTTAGTATAAGAATATTAATTCGTATAGAATTAGGACAACCTGGAACTTTAATTG  60

Query  99   GAAATGATCAGATTTATAATGTAATCGTAACAGCACATGCATTTATTATAAtttttttA  158
            ||||||||||||||||||||||||||||||||||||||||||||||||||||||||||||
Sbjct  61   GAAATGATCAGATTTATAATGTAATCGTAACAGCACATGCATTTATTATAATTTTTTTA  120

Query  159  TAGTTATACCAATCATAATCGGAGGATTTGGAAATTGATTAGTCCCTATCATATTGGGTG  218
            ||||||||||||||||||||||||||||||||||||||||||||||||||||||||||||
Sbjct  121  TAGTTATACCAATCATAATCGGAGGATTTGGAAATTGATTAGTCCCTATCATATTGGGTG  180

Query  219  CCCCAGATATAGCATTTCCACGAATAAATAATATAAGATTTTGACTTTTACCTCCTTCAC  278
            ||||||||||||||||||||||||||||||||||||||||||||||||||||||||||||
Sbjct  181  CCCCAGATATAGCATTTCCACGAATAAATAATATAAGATTTTGACTTTTACCTCCTTCAC  240

Query  279  TATTTTTATTAATTAACTCTTCATTAATTGAATCTGGAGCTGGTACAGGATGAACAGTTT  338
            ||||||||||||||||||||||||||||||||||||||||||||||||||||||||||||
Sbjct  241  TATTTTTATTAATTAACTCTTCATTAATTGAATCTGGAGCTGGTACAGGATGAACAGTTT  300

Query  339  ACCCTCCTCTATCTTCAAATTTATCACATTATGGCCCTTCAGTAGATTTAGCTATTTTTT  398
            ||||||||||||||||||||||||||||||||||||||||||||||||||||||||||||
Sbjct  301  ACCCTCCTCTATCTTCAAATTTATCACATTATGGCCCTTCAGTAGATTTAGCTATTTTTT  360

Query  399  CTCTTCATCTTGCTGGTGCTTCTTCAATTTTAGGTGCAATTAACTTTATTACAACTATTG  458
            ||||||||||||||||||||||||||||||||||||||||||||||||||||||||||||
Sbjct  361  CTCTTCATCTTGCTGGTGCTTCTTCAATTTTAGGTGCAATTAACTTTATTACAACTATTG  420

Query  459  TAAACATACGATCAATTGGAATAACAATAGAACGTATACCATTATTTGTTTGATCTGTTT  518
            ||||||||||||||||||||||||||||||||||||||||||||||||||||||||||||
Sbjct  421  TAAACATACGATCAATTGGAATAACAATAGAACGTATACCATTATTTGTTTGATCTGTTT  480

Query  519  TAATTACAGCTATTTTATTACTTTTATCTTTACCTGTATTAGCAGGTGCCATTACAATAT  578
            ||||||||||||||||||||||||||||||||||||||||||||||||||||||||||||
Sbjct  481  TAATTACAGCTATTTTATTACTTTTATCTTTACCTGTATTAGCAGGTGCCATTACAATAT  540

Query  579  TGTTAACTGATCGAAACTTTAATACATCATTTTTTGATCCTTCAGGGGGAGGAGATCCAA  638
            ||||||||||||||||||||||||||||||||||||||||||||||||||||||||||||
Sbjct  541  TGTTAACTGATCGAAACTTTAATACATCATTTTTTGATCCTTCAGGGGGAGGAGATCCAA  600

Query  639  TTTTATATCAACATTTATTT  658
            ||||||||||||||||||||
Sbjct  601  TTTTATATCAACATTTATTT  620
```

```
>Rhipicephalus sanguineus isolate Kajtasovo cytochrome oxidase subunit I (COI)
gene, partial cds; mitochondrial
Sequence ID: KX757879.1 Length: 630
Range 1: 1 to 620

Score:1146 bits(620), Expect:0.0,
Identities:620/620(100%),  Gaps:0/620(0%), Strand: Plus/Plus

Query  39   TAGGACTTAGTATAAGAATATTAATTCGTATAGAATTAGGACAACCTGGAACTTTAATTG  98
            ||||||||||||||||||||||||||||||||||||||||||||||||||||||||||||
Sbjct  1    TAGGACTTAGTATAAGAATATTAATTCGTATAGAATTAGGACAACCTGGAACTTTAATTG  60

Query  99   GAAATGATCAGATTTATAATGTAATCGTAACAGCACATGCATTTATTATAAtttttttA  158
            ||||||||||||||||||||||||||||||||||||||||||||||||||||||||||||
Sbjct  61   GAAATGATCAGATTTATAATGTAATCGTAACAGCACATGCATTTATTATAATTTTTTTA  120

Query  159  TAGTTATACCAATCATAATCGGAGGATTTGGAAATTGATTAGTCCCTATCATATTGGGTG  218
            ||||||||||||||||||||||||||||||||||||||||||||||||||||||||||||
Sbjct  121  TAGTTATACCAATCATAATCGGAGGATTTGGAAATTGATTAGTCCCTATCATATTGGGTG  180

Query  219  CCCCAGATATAGCATTTCCACGAATAAATAATATAAGATTTTGACTTTTACCTCCTTCAC  278
            ||||||||||||||||||||||||||||||||||||||||||||||||||||||||||||
Sbjct  181  CCCCAGATATAGCATTTCCACGAATAAATAATATAAGATTTTGACTTTTACCTCCTTCAC  240

Query  279  TATTTTTATTAATTAACTCTTCATTAATTGAATCTGGAGCTGGTACAGGATGAACAGTTT  338
            ||||||||||||||||||||||||||||||||||||||||||||||||||||||||||||
Sbjct  241  TATTTTTATTAATTAACTCTTCATTAATTGAATCTGGAGCTGGTACAGGATGAACAGTTT  300

Query  339  ACCCTCCTCTATCTTCAAATTTATCACATTATGGCCCTTCAGTAGATTTAGCTATTTTTT  398
            ||||||||||||||||||||||||||||||||||||||||||||||||||||||||||||
Sbjct  301  ACCCTCCTCTATCTTCAAATTTATCACATTATGGCCCTTCAGTAGATTTAGCTATTTTTT  360

Query  399  CTCTTCATCTTGCTGGTGCTTCTTCAATTTTAGGTGCAATTAACTTTATTACAACTATTG  458
            ||||||||||||||||||||||||||||||||||||||||||||||||||||||||||||
Sbjct  361  CTCTTCATCTTGCTGGTGCTTCTTCAATTTTAGGTGCAATTAACTTTATTACAACTATTG  420

Query  459  TAAACATACGATCAATTGGAATAACAATAGAACGTATACCATTATTTGTTTGATCTGTTT  518
            ||||||||||||||||||||||||||||||||||||||||||||||||||||||||||||
Sbjct  421  TAAACATACGATCAATTGGAATAACAATAGAACGTATACCATTATTTGTTTGATCTGTTT  480

Query  519  TAATTACAGCTATTTTATTACTTTTATCTTTACCTGTATTAGCAGGTGCCATTACAATAT  578
            ||||||||||||||||||||||||||||||||||||||||||||||||||||||||||||
Sbjct  481  TAATTACAGCTATTTTATTACTTTTATCTTTACCTGTATTAGCAGGTGCCATTACAATAT  540

Query  579  TGTTAACTGATCGAAACTTTAATACATCATTTTTTGATCCTTCAGGGGGAGGAGATCCAA  638
            ||||||||||||||||||||||||||||||||||||||||||||||||||||||||||||
Sbjct  541  TGTTAACTGATCGAAACTTTAATACATCATTTTTTGATCCTTCAGGGGGAGGAGATCCAA  600

Query  639  TTTTATATCAACATTTATTT  658
            ||||||||||||||||||||
Sbjct  601  TTTTATATCAACATTTATTT  620
```

```
>Rhipicephalus sanguineus clone #6 cytochrome oxidase subunit I (COI) gene,
partial cds; mitochondrial
Sequence ID: KX519711.1 Length: 636
Range 1: 1 to 620

Score:1146 bits(620), Expect:0.0,
Identities:620/620(100%),  Gaps:0/620(0%), Strand: Plus/Plus

Query  39   TAGGACTTAGTATAAGAATATTAATTCGTATAGAATTAGGACAACCTGGAACTTTAATTG  98
            ||||||||||||||||||||||||||||||||||||||||||||||||||||||||||||
Sbjct  1    TAGGACTTAGTATAAGAATATTAATTCGTATAGAATTAGGACAACCTGGAACTTTAATTG  60

Query  99   GAAATGATCAGATTTATAATGTAATCGTAACAGCACATGCATTTATTATAAtttttttA  158
            ||||||||||||||||||||||||||||||||||||||||||||||||||||||||||||
Sbjct  61   GAAATGATCAGATTTATAATGTAATCGTAACAGCACATGCATTTATTATAATTTTTTTTA  120

Query  159  TAGTTATACCAATCATAATCGGAGGATTTGGAAATTGATTAGTCCCTATCATATTGGGTG  218
            ||||||||||||||||||||||||||||||||||||||||||||||||||||||||||||
Sbjct  121  TAGTTATACCAATCATAATCGGAGGATTTGGAAATTGATTAGTCCCTATCATATTGGGTG  180

Query  219  CCCCAGATATAGCATTTCCACGAATAAATAATATAAGATTTTGACTTTTACCTCCTTCAC  278
            ||||||||||||||||||||||||||||||||||||||||||||||||||||||||||||
Sbjct  181  CCCCAGATATAGCATTTCCACGAATAAATAATATAAGATTTTGACTTTTACCTCCTTCAC  240

Query  279  TATTTTTATTAATTAACTCTTCATTAATTGAATCTGGAGCTGGTACAGGATGAACAGTTT  338
            ||||||||||||||||||||||||||||||||||||||||||||||||||||||||||||
Sbjct  241  TATTTTTATTAATTAACTCTTCATTAATTGAATCTGGAGCTGGTACAGGATGAACAGTTT  300

Query  339  ACCCTCCTCTATCTTCAAATTTATCACATTATGGCCCTTCAGTAGATTTAGCTATTTTTT  398
            ||||||||||||||||||||||||||||||||||||||||||||||||||||||||||||
Sbjct  301  ACCCTCCTCTATCTTCAAATTTATCACATTATGGCCCTTCAGTAGATTTAGCTATTTTTT  360

Query  399  CTCTTCATCTTGCTGGTGCTTCTTCAATTTTAGGTGCAATTAACTTTATTACAACTATTG  458
            ||||||||||||||||||||||||||||||||||||||||||||||||||||||||||||
Sbjct  361  CTCTTCATCTTGCTGGTGCTTCTTCAATTTTAGGTGCAATTAACTTTATTACAACTATTG  420

Query  459  TAAACATACGATCAATTGGAATAACAATAGAACGTATACCATTATTTGTTTGATCTGTTT  518
            ||||||||||||||||||||||||||||||||||||||||||||||||||||||||||||
Sbjct  421  TAAACATACGATCAATTGGAATAACAATAGAACGTATACCATTATTTGTTTGATCTGTTT  480

Query  519  TAATTACAGCTATTTTATTACTTTTATCTTTACCTGTATTAGCAGGTGCCATTACAATAT  578
            ||||||||||||||||||||||||||||||||||||||||||||||||||||||||||||
Sbjct  481  TAATTACAGCTATTTTATTACTTTTATCTTTACCTGTATTAGCAGGTGCCATTACAATAT  540

Query  579  TGTTAACTGATCGAAACTTTAATACATCATTTTTTGATCCTTCAGGGGGAGGAGATCCAA  638
            ||||||||||||||||||||||||||||||||||||||||||||||||||||||||||||
Sbjct  541  TGTTAACTGATCGAAACTTTAATACATCATTTTTTGATCCTTCAGGGGGAGGAGATCCAA  600

Query  639  TTTTATATCAACATTTATTT  658
            ||||||||||||||||||||
Sbjct  601  TTTTATATCAACATTTATTT  620
```

>Rhipicephalus sanguineus isolate SV4 cytochrome c oxidase subunit I (COX1) gene, partial cds; mitochondrial
Sequence ID: MW135448.1 Length: 630
Range 1: 1 to 620

Score:1146 bits(620), Expect:0.0,
Identities:620/620(100%), Gaps:0/620(0%), Strand: Plus/Plus

```
Query  39   TAGGACTTAGTATAAGAATATTAATTCGTATAGAATTAGGACAACCTGGAACTTTAATTG  98
            ||||||||||||||||||||||||||||||||||||||||||||||||||||||||||||
Sbjct  1    TAGGACTTAGTATAAGAATATTAATTCGTATAGAATTAGGACAACCTGGAACTTTAATTG  60

Query  99   GAAATGATCAGATTTATAATGTAATCGTAACAGCACATGCATTTATTATAAtttttttA  158
            ||||||||||||||||||||||||||||||||||||||||||||||||||||||||||||
Sbjct  61   GAAATGATCAGATTTATAATGTAATCGTAACAGCACATGCATTTATTATAATTTTTTTTA  120

Query  159  TAGTTATACCAATCATAATCGGAGGATTTGGAAATTGATTAGTCCCTATCATATTGGGTG  218
            ||||||||||||||||||||||||||||||||||||||||||||||||||||||||||||
Sbjct  121  TAGTTATACCAATCATAATCGGAGGATTTGGAAATTGATTAGTCCCTATCATATTGGGTG  180

Query  219  CCCCAGATATAGCATTTCCACGAATAAATAATATAAGATTTTGACTTTTACCTCCTTCAC  278
            ||||||||||||||||||||||||||||||||||||||||||||||||||||||||||||
Sbjct  181  CCCCAGATATAGCATTTCCACGAATAAATAATATAAGATTTTGACTTTTACCTCCTTCAC  240

Query  279  TATTTTTATTAATTAACTCTTCATTAATTGAATCTGGAGCTGGTACAGGATGAACAGTTT  338
            ||||||||||||||||||||||||||||||||||||||||||||||||||||||||||||
Sbjct  241  TATTTTTATTAATTAACTCTTCATTAATTGAATCTGGAGCTGGTACAGGATGAACAGTTT  300

Query  339  ACCCTCCTCTATCTTCAAATTTATCACATTATGGCCCTTCAGTAGATTTAGCTATTTTTT  398
            ||||||||||||||||||||||||||||||||||||||||||||||||||||||||||||
Sbjct  301  ACCCTCCTCTATCTTCAAATTTATCACATTATGGCCCTTCAGTAGATTTAGCTATTTTTT  360

Query  399  CTCTTCATCTTGCTGGTGCTTCTTCAATTTTAGGTGCAATTAACTTTATTACAACTATTG  458
            ||||||||||||||||||||||||||||||||||||||||||||||||||||||||||||
Sbjct  361  CTCTTCATCTTGCTGGTGCTTCTTCAATTTTAGGTGCAATTAACTTTATTACAACTATTG  420

Query  459  TAAACATACGATCAATTGGAATAACAATAGAACGTATACCATTATTTGTTTGATCTGTTT  518
            ||||||||||||||||||||||||||||||||||||||||||||||||||||||||||||
Sbjct  421  TAAACATACGATCAATTGGAATAACAATAGAACGTATACCATTATTTGTTTGATCTGTTT  480

Query  519  TAATTACAGCTATTTTATTACTTTTATCTTTACCTGTATTAGCAGGTGCCATTACAATAT  578
            ||||||||||||||||||||||||||||||||||||||||||||||||||||||||||||
Sbjct  481  TAATTACAGCTATTTTATTACTTTTATCTTTACCTGTATTAGCAGGTGCCATTACAATAT  540

Query  579  TGTTAACTGATCGAAACTTTAATACATCATTTTTTGATCCTTCAGGGGGAGGAGATCCAA  638
            ||||||||||||||||||||||||||||||||||||||||||||||||||||||||||||
Sbjct  541  TGTTAACTGATCGAAACTTTAATACATCATTTTTTGATCCTTCAGGGGGAGGAGATCCAA  600

Query  639  TTTTATATCAACATTTATTT  658
            ||||||||||||||||||||
```

```
Sbjct  601  TTTTATATCAACATTTATTT  620
```

>Rhipicephalus sanguineus isolate Samont2 cytochrome oxidase subunit I (cox1) gene, partial cds; mitochondrial
Sequence ID: MH630347.1 Length: 644
Range 1: 20 to 641

Score:1144 bits(619), Expect:0.0,
Identities:621/622(99%), Gaps:0/622(0%), Strand: Plus/Plus

```
Query  1    AACAATATATTTAATTTTTGGAGCATGATCAGGTATATTAGGACTTAGTATAAGAATATT  60
            ||||||||||||||||||||||||||||||||||||||||||||||||||||||||||||
Sbjct  20   AACAATATATTTAATTTTTGGAGCATGATCAGGTATATTAGGACTTAGTATAAGAATATT  79

Query  61   AATTCGTATAGAATTAGGACAACCTGGAACTTTAATTGGAAATGATCAGATTTATAATGT  120
            |||| |||||||||||||||||||||||||||||||||||||||||||||||||||||||
Sbjct  80   AATTGGTATAGAATTAGGACAACCTGGAACTTTAATTGGAAATGATCAGATTTATAATGT  139

Query  121  AATCGTAACAGCACATGCATTTATTATAAttttttttATAGTTATACCAATCATAATCGG  180
            ||||||||||||||||||||||||||||||||||||||||||||||||||||||||||||
Sbjct  140  AATCGTAACAGCACATGCATTTATTATAATTTTTTTTATAGTTATACCAATCATAATCGG  199

Query  181  AGGATTTGGAAATTGATTAGTCCCTATCATATTGGGTGCCCCAGATATAGCATTTCCACG  240
            ||||||||||||||||||||||||||||||||||||||||||||||||||||||||||||
Sbjct  200  AGGATTTGGAAATTGATTAGTCCCTATCATATTGGGTGCCCCAGATATAGCATTTCCACG  259

Query  241  AATAAATAATATAAGATTTTGACTTTTACCTCCTTCACTATTTTTATTAATTAACTCTTC  300
            ||||||||||||||||||||||||||||||||||||||||||||||||||||||||||||
Sbjct  260  AATAAATAATATAAGATTTTGACTTTTACCTCCTTCACTATTTTTATTAATTAACTCTTC  319

Query  301  ATTAATTGAATCTGGAGCTGGTACAGGATGAACAGTTTACCCTCCTCTATCTTCAAATTT  360
            ||||||||||||||||||||||||||||||||||||||||||||||||||||||||||||
Sbjct  320  ATTAATTGAATCTGGAGCTGGTACAGGATGAACAGTTTACCCTCCTCTATCTTCAAATTT  379

Query  361  ATCACATTATGGCCCTTCAGTAGATTTAGCTATTTTTTCTCTTCATCTTGCTGGTGCTTC  420
            ||||||||||||||||||||||||||||||||||||||||||||||||||||||||||||
Sbjct  380  ATCACATTATGGCCCTTCAGTAGATTTAGCTATTTTTTCTCTTCATCTTGCTGGTGCTTC  439

Query  421  TTCAATTTTAGGTGCAATTAACTTTATTACAACTATTGTAAACATACGATCAATTGGAAT  480
            ||||||||||||||||||||||||||||||||||||||||||||||||||||||||||||
Sbjct  440  TTCAATTTTAGGTGCAATTAACTTTATTACAACTATTGTAAACATACGATCAATTGGAAT  499

Query  481  AACAATAGAACGTATACCATTATTTGTTTGATCTGTTTTAATTACAGCTATTTTATTACT  540
            ||||||||||||||||||||||||||||||||||||||||||||||||||||||||||||
Sbjct  500  AACAATAGAACGTATACCATTATTTGTTTGATCTGTTTTAATTACAGCTATTTTATTACT  559

Query  541  TTTATCTTTACCTGTATTAGCAGGTGCCATTACAATATTGTTAACTGATCGAAACTTTAA  600
            ||||||||||||||||||||||||||||||||||||||||||||||||||||||||||||
Sbjct  560  TTTATCTTTACCTGTATTAGCAGGTGCCATTACAATATTGTTAACTGATCGAAACTTTAA  619

Query  601  TACATCATTTTTTGATCCTTCA  622
```

```
              |||||||||||||||||||||
Sbjct  620  TACATCATTTTTGATCCTTCA  641
```

>Rhipicephalus sanguineus isolate Samont1 cytochrome oxidase subunit I (cox1) gene, partial cds; mitochondrial
Sequence ID: MH630346.1 Length: 644
Range 1: 20 to 641

Score:1144 bits(619), Expect:0.0,
Identities:621/622(99%), Gaps:0/622(0%), Strand: Plus/Plus

```
Query  1    AACAATATATTTAATTTTTGGAGCATGATCAGGTATATTAGGACTTAGTATAAGAATATT  60
            ||||||||||||||||||||||||||||||||||||||||||||||||||||||||||||
Sbjct  20   AACAATATATTTAATTTTTGGAGCATGATCAGGTATATTAGGACTTAGTATAAGAATATT  79

Query  61   AATTCGTATAGAATTAGGACAACCTGGAACTTTAATTGGAAATGATCAGATTTATAATGT  120
            |||| |||||||||||||||||||||||||||||||||||||||||||||||||||||||
Sbjct  80   AATTGGTATAGAATTAGGACAACCTGGAACTTTAATTGGAAATGATCAGATTTATAATGT  139

Query  121  AATCGTAACAGCACATGCATTTATTATAAtttttttATAGTTATACCAATCATAATCGG  180
            ||||||||||||||||||||||||||||||||||||||||||||||||||||||||||||
Sbjct  140  AATCGTAACAGCACATGCATTTATTATAATTTTTTTATAGTTATACCAATCATAATCGG  199

Query  181  AGGATTTGGAAATTGATTAGTCCCTATCATATTGGGTGCCCCAGATATAGCATTTCCACG  240
            ||||||||||||||||||||||||||||||||||||||||||||||||||||||||||||
Sbjct  200  AGGATTTGGAAATTGATTAGTCCCTATCATATTGGGTGCCCCAGATATAGCATTTCCACG  259

Query  241  AATAAATAATATAAGATTTTGACTTTTACCTCCTTCACTATTTTTATTAATTAACTCTTC  300
            ||||||||||||||||||||||||||||||||||||||||||||||||||||||||||||
Sbjct  260  AATAAATAATATAAGATTTTGACTTTTACCTCCTTCACTATTTTTATTAATTAACTCTTC  319

Query  301  ATTAATTGAATCTGGAGCTGGTACAGGATGAACAGTTTACCCTCCTCTATCTTCAAATTT  360
            ||||||||||||||||||||||||||||||||||||||||||||||||||||||||||||
Sbjct  320  ATTAATTGAATCTGGAGCTGGTACAGGATGAACAGTTTACCCTCCTCTATCTTCAAATTT  379

Query  361  ATCACATTATGGCCCTTCAGTAGATTTAGCTATTTTTTCTCTTCATCTTGCTGGTGCTTC  420
            ||||||||||||||||||||||||||||||||||||||||||||||||||||||||||||
Sbjct  380  ATCACATTATGGCCCTTCAGTAGATTTAGCTATTTTTTCTCTTCATCTTGCTGGTGCTTC  439

Query  421  TTCAATTTTAGGTGCAATTAACTTTATTACAACTATTGTAAACATACGATCAATTGGAAT  480
            ||||||||||||||||||||||||||||||||||||||||||||||||||||||||||||
Sbjct  440  TTCAATTTTAGGTGCAATTAACTTTATTACAACTATTGTAAACATACGATCAATTGGAAT  499

Query  481  AACAATAGAACGTATACCATTATTTGTTTGATCTGTTTTAATTACAGCTATTTTATTACT  540
            ||||||||||||||||||||||||||||||||||||||||||||||||||||||||||||
Sbjct  500  AACAATAGAACGTATACCATTATTTGTTTGATCTGTTTTAATTACAGCTATTTTATTACT  559

Query  541  TTTATCTTTACCTGTATTAGCAGGTGCCATTACAATATTGTTAACTGATCGAAACTTTAA  600
            ||||||||||||||||||||||||||||||||||||||||||||||||||||||||||||
Sbjct  560  TTTATCTTTACCTGTATTAGCAGGTGCCATTACAATATTGTTAACTGATCGAAACTTTAA  619
```

```
Query  601  TACATCATTTTTTGATCCTTCA  622
            ||||||||||||||||||||||
Sbjct  620  TACATCATTTTTTGATCCTTCA  641
```

>Rhipicephalus sanguineus sensu lato 'temperate lineage' isolate CR1568 cytochrome c oxidase subunit I (COI) gene, partial cds; mitochondrial
Sequence ID: MF426000.1 Length: 635
Range 1: 14 to 635

Score:1144 bits(619), Expect:0.0,
Identities:621/622(99%), Gaps:0/622(0%), Strand: Plus/Plus

```
Query  1    AACAATATATTTAATTTTTGGAGCATGATCAGGTATATTAGGACTTAGTATAAGAATATT  60
            ||||||||||||||||||||||||||||||||||||||||||||||||||||||||||||
Sbjct  14   AACAATATATTTAATTTTTGGAGCATGATCAGGTATATTAGGACTTAGTATAAGAATATT  73

Query  61   AATTCGTATAGAATTAGGACAACCTGGAACTTTAATTGGAAATGATCAGATTTATAATGT  120
            ||||||||||||||||||||||||||||||||||||||||||||||||||||||||||||
Sbjct  74   AATTCGTATAGAATTAGGACAACCTGGAACTTTAATTGGAAATGATCAGATTTATAATGT  133

Query  121  AATCGTAACAGCACATGCATTTATTATAAtttttttATAGTTATACCAATCATAATCGG  180
            ||||||||||||||||||||||||||||||||||||||||||||||||||||||||||||
Sbjct  134  AATCGTAACAGCACATGCATTTATTATAATTTTTTTATAGTTATACCAATCATAATCGG  193

Query  181  AGGATTTGGAAATTGATTAGTCCCTATCATATTGGGTGCCCCAGATATAGCATTTCCACG  240
            ||||||||||||||||||||||||||||||||||||||||||||||||||||||||||||
Sbjct  194  AGGATTTGGAAATTGATTAGTCCCTATCATATTGGGTGCCCCAGATATAGCATTTCCACG  253

Query  241  AATAAATAATATAAGATTTTGACTTTTACCTCCTTCACTATTTTTATTAATTAACTCTTC  300
            ||||||||||||||||||||||||||||||||||||||||||||||||||||||||||||
Sbjct  254  AATAAATAATATAAGATTTTGACTTTTACCTCCTTCACTATTTTTATTAATTAACTCTTC  313

Query  301  ATTAATTGAATCTGGAGCTGGTACAGGATGAACAGTTTACCCTCCTCTATCTTCAAATTT  360
            ||||||||||||||||||||||||||||||||||||||||||||||||||||||||||||
Sbjct  314  ATTAATTGAATCTGGAGCTGGTACAGGATGAACAGTTTACCCTCCTCTATCTTCAAATTT  373

Query  361  ATCACATTATGGCCCTTCAGTAGATTTAGCTATTTTTTCTCTTCATCTTGCTGGTGCTTC  420
            |||||||||||| |||||||||||||||||||||||||||||||||||||||||||||||
Sbjct  374  ATCACATTATGGTCCTTCAGTAGATTTAGCTATTTTTTCTCTTCATCTTGCTGGTGCTTC  433

Query  421  TTCAATTTTAGGTGCAATTAACTTTATTACAACTATTGTAAACATACGATCAATTGGAAT  480
            ||||||||||||||||||||||||||||||||||||||||||||||||||||||||||||
Sbjct  434  TTCAATTTTAGGTGCAATTAACTTTATTACAACTATTGTAAACATACGATCAATTGGAAT  493

Query  481  AACAATAGAACGTATACCATTATTTGTTTGATCTGTTTTAATTACAGCTATTTTATTACT  540
            ||||||||||||||||||||||||||||||||||||||||||||||||||||||||||||
Sbjct  494  AACAATAGAACGTATACCATTATTTGTTTGATCTGTTTTAATTACAGCTATTTTATTACT  553

Query  541  TTTATCTTTACCTGTATTAGCAGGTGCCATTACAATATTGTTAACTGATCGAAACTTTAA  600
            ||||||||||||||||||||||||||||||||||||||||||||||||||||||||||||
Sbjct  554  TTTATCTTTACCTGTATTAGCAGGTGCCATTACAATATTGTTAACTGATCGAAACTTTAA  613
```

```
Query  601  TACATCATTTTTTGATCCTTCA  622
            ||||||||||||||||||||||
Sbjct  614  TACATCATTTTTTGATCCTTCA  635
```

>Rhipicephalus sanguineus isolate Boka-A cytochrome oxidase subunit I (COI) gene, partial cds; mitochondrial
Sequence ID: KX757905.1 Length: 630
Range 1: 1 to 620

Score:1140 bits(617), Expect:0.0,
Identities:619/620(99%), Gaps:0/620(0%), Strand: Plus/Plus

```
Query  39   TAGGACTTAGTATAAGAATATTAATTCGTATAGAATTAGGACAACCTGGAACTTTAATTG  98
            ||||||||||||||||||||||||||||||||||||||||||||||||||||||||||||
Sbjct  1    TAGGACTTAGTATAAGAATATTAATTCGTATAGAATTAGGACAACCTGGAACTTTAATTG  60

Query  99   GAAATGATCAGATTTATAATGTAATCGTAACAGCACATGCATTTATTATAAtttttttA  158
            ||||||||||||||||||||||||||||||||||||||||||||||||||||||||||||
Sbjct  61   GAAATGATCAGATTTATAATGTAATCGTAACAGCACATGCATTTATTATAATTTTTTTA  120

Query  159  TAGTTATACCAATCATAATCGGAGGATTTGGAAATTGATTAGTCCCTATCATATTGGGTG  218
            ||||||||||||||||||||||||||||||||||||||||||||||||||||||||||||
Sbjct  121  TAGTTATACCAATCATAATCGGAGGATTTGGAAATTGATTAGTCCCTATCATATTGGGTG  180

Query  219  CCCCAGATATAGCATTTCCACGAATAAATAATATAAGATTTTGACTTTTACCTCCTTCAC  278
            ||||||||||||||||||||||||||||||||||||||||||||||||||||||||||||
Sbjct  181  CCCCAGATATAGCATTTCCACGAATAAATAATATAAGATTTTGACTTTTACCTCCTTCAC  240

Query  279  TATTTTTATTAATTAACTCTTCATTAATTGAATCTGGAGCTGGTACAGGATGAACAGTTT  338
            |||||||||||||||| |||||||||||||||||||||||||||||||||||||||||||
Sbjct  241  TATTTTTATTAATTAATTCTTCATTAATTGAATCTGGAGCTGGTACAGGATGAACAGTTT  300

Query  339  ACCCTCCTCTATCTTCAAATTTATCACATTATGGCCCTTCAGTAGATTTAGCTATTTTTT  398
            ||||||||||||||||||||||||||||||||||||||||||||||||||||||||||||
Sbjct  301  ACCCTCCTCTATCTTCAAATTTATCACATTATGGCCCTTCAGTAGATTTAGCTATTTTTT  360

Query  399  CTCTTCATCTTGCTGGTGCTTCTTCAATTTTAGGTGCAATTAACTTTATTACAACTATTG  458
            ||||||||||||||||||||||||||||||||||||||||||||||||||||||||||||
Sbjct  361  CTCTTCATCTTGCTGGTGCTTCTTCAATTTTAGGTGCAATTAACTTTATTACAACTATTG  420

Query  459  TAAACATACGATCAATTGGAATAACAATAGAACGTATACCATTATTTGTTTGATCTGTTT  518
            ||||||||||||||||||||||||||||||||||||||||||||||||||||||||||||
Sbjct  421  TAAACATACGATCAATTGGAATAACAATAGAACGTATACCATTATTTGTTTGATCTGTTT  480

Query  519  TAATTACAGCTATTTTATTACTTTTATCTTTACCTGTATTAGCAGGTGCCATTACAATAT  578
            ||||||||||||||||||||||||||||||||||||||||||||||||||||||||||||
Sbjct  481  TAATTACAGCTATTTTATTACTTTTATCTTTACCTGTATTAGCAGGTGCCATTACAATAT  540

Query  579  TGTTAACTGATCGAAACTTTAATACATCATTTTTTGATCCTTCAGGGGGAGGAGATCCAA  638
            ||||||||||||||||||||||||||||||||||||||||||||||||||||||||||||
```

```
Sbjct  541   TGTTAACTGATCGAAACTTTAATACATCATTTTTTGATCCTTCAGGGGGAGGAGATCCAA  600

Query  639   TTTTATATCAACATTTATTT  658
             ||||||||||||||||||||
Sbjct  601   TTTTATATCAACATTTATTT  620
```

>Rhipicephalus sanguineus isolate Sibenik cytochrome oxidase subunit I (COI) gene, partial cds; mitochondrial
Sequence ID: KX757895.1 Length: 630
Range 1: 1 to 620

Score:1140 bits(617), Expect:0.0,
Identities:619/620(99%), Gaps:0/620(0%), Strand: Plus/Plus

```
Query  39    TAGGACTTAGTATAAGAATATTAATTCGTATAGAATTAGGACAACCTGGAACTTTAATTG  98
             |||||||||||||||||||||||||||||||||| |||||||||||||||||||||||||
Sbjct  1     TAGGACTTAGTATAAGAATATTAATTCGTATAGAGTTAGGACAACCTGGAACTTTAATTG  60

Query  99    GAAATGATCAGATTTATAATGTAATCGTAACAGCACATGCATTTATTATAAtttttttA  158
             ||||||||||||||||||||||||||||||||||||||||||||||||||||||||||||
Sbjct  61    GAAATGATCAGATTTATAATGTAATCGTAACAGCACATGCATTTATTATAATTTTTTTTA  120

Query  159   TAGTTATACCAATCATAATCGGAGGATTTGGAAATTGATTAGTCCCTATCATATTGGGTG  218
             ||||||||||||||||||||||||||||||||||||||||||||||||||||||||||||
Sbjct  121   TAGTTATACCAATCATAATCGGAGGATTTGGAAATTGATTAGTCCCTATCATATTGGGTG  180

Query  219   CCCCAGATATAGCATTTCCACGAATAAATAATATAAGATTTTGACTTTTACCTCCTTCAC  278
             ||||||||||||||||||||||||||||||||||||||||||||||||||||||||||||
Sbjct  181   CCCCAGATATAGCATTTCCACGAATAAATAATATAAGATTTTGACTTTTACCTCCTTCAC  240

Query  279   TATTTTTATTAATTAACTCTTCATTAATTGAATCTGGAGCTGGTACAGGATGAACAGTTT  338
             ||||||||||||||||||||||||||||||||||||||||||||||||||||||||||||
Sbjct  241   TATTTTTATTAATTAACTCTTCATTAATTGAATCTGGAGCTGGTACAGGATGAACAGTTT  300

Query  339   ACCCTCCTCTATCTTCAAATTTATCACATTATGGCCCTTCAGTAGATTTAGCTATTTTTT  398
             ||||||||||||||||||||||||||||||||||||||||||||||||||||||||||||
Sbjct  301   ACCCTCCTCTATCTTCAAATTTATCACATTATGGCCCTTCAGTAGATTTAGCTATTTTTT  360

Query  399   CTCTTCATCTTGCTGGTGCTTCTTCAATTTTAGGTGCAATTAACTTTATTACAACTATTG  458
             ||||||||||||||||||||||||||||||||||||||||||||||||||||||||||||
Sbjct  361   CTCTTCATCTTGCTGGTGCTTCTTCAATTTTAGGTGCAATTAACTTTATTACAACTATTG  420

Query  459   TAAACATACGATCAATTGGAATAACAATAGAACGTATACCATTATTTGTTTGATCTGTTT  518
             ||||||||||||||||||||||||||||||||||||||||||||||||||||||||||||
Sbjct  421   TAAACATACGATCAATTGGAATAACAATAGAACGTATACCATTATTTGTTTGATCTGTTT  480

Query  519   TAATTACAGCTATTTTATTACTTTTATCTTTACCTGTATTAGCAGGTGCCATTACAATAT  578
             ||||||||||||||||||||||||||||||||||||||||||||||||||||||||||||
Sbjct  481   TAATTACAGCTATTTTATTACTTTTATCTTTACCTGTATTAGCAGGTGCCATTACAATAT  540

Query  579   TGTTAACTGATCGAAACTTTAATACATCATTTTTTGATCCTTCAGGGGGAGGAGATCCAA  638
```

```
               ||||||||||||||||||||||||||||||||||||||||||||||||||||||||||||
Sbjct  541   TGTTAACTGATCGAAACTTTAATACATCATTTTTTGATCCTTCAGGGGGAGGAGATCCAA  600

Query  639   TTTTATATCAACATTTATTT  658
             ||||||||||||||||||||
Sbjct  601   TTTTATATCAACATTTATTT  620
```

```
>Rhipicephalus sanguineus isolate Zadar cytochrome oxidase subunit I (COI) gene,
partial cds; mitochondrial
Sequence ID: KX757892.1 Length: 630
Range 1: 1 to 620

Score:1140 bits(617), Expect:0.0,
Identities:619/620(99%),  Gaps:0/620(0%), Strand: Plus/Plus

Query  39    TAGGACTTAGTATAAGAATATTAATTCGTATAGAATTAGGACAACCTGGAACTTTAATTG  98
             |||||||||||||||||||||||||||||||||| |||||||||||||||||||||||||
Sbjct  1     TAGGACTTAGTATAAGAATATTAATTCGTATAGAGTTAGGACAACCTGGAACTTTAATTG  60

Query  99    GAAATGATCAGATTTATAATGTAATCGTAACAGCACATGCATTTATTATAAtttttttA  158
             ||||||||||||||||||||||||||||||||||||||||||||||||||||||||||||
Sbjct  61    GAAATGATCAGATTTATAATGTAATCGTAACAGCACATGCATTTATTATAATTTTTTTTA  120

Query  159   TAGTTATACCAATCATAATCGGAGGATTTGGAAATTGATTAGTCCCTATCATATTGGGTG  218
             ||||||||||||||||||||||||||||||||||||||||||||||||||||||||||||
Sbjct  121   TAGTTATACCAATCATAATCGGAGGATTTGGAAATTGATTAGTCCCTATCATATTGGGTG  180

Query  219   CCCCAGATATAGCATTTCCACGAATAAATAATATAAGATTTTGACTTTTACCTCCTTCAC  278
             ||||||||||||||||||||||||||||||||||||||||||||||||||||||||||||
Sbjct  181   CCCCAGATATAGCATTTCCACGAATAAATAATATAAGATTTTGACTTTTACCTCCTTCAC  240

Query  279   TATTTTTATTAATTAACTCTTCATTAATTGAATCTGGAGCTGGTACAGGATGAACAGTTT  338
             ||||||||||||||||||||||||||||||||||||||||||||||||||||||||||||
Sbjct  241   TATTTTTATTAATTAACTCTTCATTAATTGAATCTGGAGCTGGTACAGGATGAACAGTTT  300

Query  339   ACCCTCCTCTATCTTCAAATTTATCACATTATGGCCCTTCAGTAGATTTAGCTATTTTTT  398
             ||||||||||||||||||||||||||||||||||||||||||||||||||||||||||||
Sbjct  301   ACCCTCCTCTATCTTCAAATTTATCACATTATGGCCCTTCAGTAGATTTAGCTATTTTTT  360

Query  399   CTCTTCATCTTGCTGGTGCTTCTTCAATTTTAGGTGCAATTAACTTTATTACAACTATTG  458
             ||||||||||||||||||||||||||||||||||||||||||||||||||||||||||||
Sbjct  361   CTCTTCATCTTGCTGGTGCTTCTTCAATTTTAGGTGCAATTAACTTTATTACAACTATTG  420

Query  459   TAAACATACGATCAATTGGAATAACAATAGAACGTATACCATTATTTGTTTGATCTGTTT  518
             ||||||||||||||||||||||||||||||||||||||||||||||||||||||||||||
Sbjct  421   TAAACATACGATCAATTGGAATAACAATAGAACGTATACCATTATTTGTTTGATCTGTTT  480

Query  519   TAATTACAGCTATTTTATTACTTTTATCTTTACCTGTATTAGCAGGTGCCATTACAATAT  578
             ||||||||||||||||||||||||||||||||||||||||||||||||||||||||||||
Sbjct  481   TAATTACAGCTATTTTATTACTTTTATCTTTACCTGTATTAGCAGGTGCCATTACAATAT  540
```

```
Query  579   TGTTAACTGATCGAAACTTTAATACATCATTTTTTGATCCTTCAGGGGGAGGAGATCCAA  638
             ||||||||||||||||||||||||||||||||||||||||||||||||||||||||||||
Sbjct  541   TGTTAACTGATCGAAACTTTAATACATCATTTTTTGATCCTTCAGGGGGAGGAGATCCAA  600

Query  639   TTTTATATCAACATTTATTT  658
             ||||||||||||||||||||
Sbjct  601   TTTTATATCAACATTTATTT  620
```

>Rhipicephalus sanguineus isolate Zagreb-A cytochrome oxidase subunit I (COI) gene, partial cds; mitochondrial
Sequence ID: KX757890.1 Length: 630
Range 1: 1 to 620

Score:1140 bits(617), Expect:0.0,
Identities:619/620(99%), Gaps:0/620(0%), Strand: Plus/Plus

```
Query  39    TAGGACTTAGTATAAGAATATTAATTCGTATAGAATTAGGACAACCTGGAACTTTAATTG  98
             |||||||||||||||||||||||||||||||||| |||||||||||||||||||||||||
Sbjct  1     TAGGACTTAGTATAAGAATATTAATTCGTATAGAGTTAGGACAACCTGGAACTTTAATTG  60

Query  99    GAAATGATCAGATTTATAATGTAATCGTAACAGCACATGCATTTATTATAAtttttttA  158
             ||||||||||||||||||||||||||||||||||||||||||||||||||||||||||||
Sbjct  61    GAAATGATCAGATTTATAATGTAATCGTAACAGCACATGCATTTATTATAATTTTTTTA  120

Query  159   TAGTTATACCAATCATAATCGGAGGATTTGGAAATTGATTAGTCCCTATCATATTGGGTG  218
             ||||||||||||||||||||||||||||||||||||||||||||||||||||||||||||
Sbjct  121   TAGTTATACCAATCATAATCGGAGGATTTGGAAATTGATTAGTCCCTATCATATTGGGTG  180

Query  219   CCCCAGATATAGCATTTCCACGAATAAATAATATAAGATTTTGACTTTTACCTCCTTCAC  278
             ||||||||||||||||||||||||||||||||||||||||||||||||||||||||||||
Sbjct  181   CCCCAGATATAGCATTTCCACGAATAAATAATATAAGATTTTGACTTTTACCTCCTTCAC  240

Query  279   TATTTTTATTAATTAACTCTTCATTAATTGAATCTGGAGCTGGTACAGGATGAACAGTTT  338
             ||||||||||||||||||||||||||||||||||||||||||||||||||||||||||||
Sbjct  241   TATTTTTATTAATTAACTCTTCATTAATTGAATCTGGAGCTGGTACAGGATGAACAGTTT  300

Query  339   ACCCTCCTCTATCTTCAAATTTATCACATTATGGCCCTTCAGTAGATTTAGCTATTTTTT  398
             ||||||||||||||||||||||||||||||||||||||||||||||||||||||||||||
Sbjct  301   ACCCTCCTCTATCTTCAAATTTATCACATTATGGCCCTTCAGTAGATTTAGCTATTTTTT  360

Query  399   CTCTTCATCTTGCTGGTGCTTCTTCAATTTTAGGTGCAATTAACTTTATTACAACTATTG  458
             ||||||||||||||||||||||||||||||||||||||||||||||||||||||||||||
Sbjct  361   CTCTTCATCTTGCTGGTGCTTCTTCAATTTTAGGTGCAATTAACTTTATTACAACTATTG  420

Query  459   TAAACATACGATCAATTGGAATAACAATAGAACGTATACCATTATTTGTTTGATCTGTTT  518
             ||||||||||||||||||||||||||||||||||||||||||||||||||||||||||||
Sbjct  421   TAAACATACGATCAATTGGAATAACAATAGAACGTATACCATTATTTGTTTGATCTGTTT  480

Query  519   TAATTACAGCTATTTTATTACTTTTATCTTTACCTGTATTAGCAGGTGCCATTACAATAT  578
             ||||||||||||||||||||||||||||||||||||||||||||||||||||||||||||
Sbjct  481   TAATTACAGCTATTTTATTACTTTTATCTTTACCTGTATTAGCAGGTGCCATTACAATAT  540
```

```
Query  579   TGTTAACTGATCGAAACTTTAATACATCATTTTTTGATCCTTCAGGGGGAGGAGATCCAA  638
             ||||||||||||||||||||||||||||||||||||||||||||||||||||||||||||
Sbjct  541   TGTTAACTGATCGAAACTTTAATACATCATTTTTTGATCCTTCAGGGGGAGGAGATCCAA  600

Query  639   TTTTATATCAACATTTATTT  658
             ||||||||||||||||||||
Sbjct  601   TTTTATATCAACATTTATTT  620
```

>Rhipicephalus sanguineus isolate Pula-A cytochrome oxidase subunit I (COI) gene, partial cds; mitochondrial
Sequence ID: KX757888.1 Length: 630
Range 1: 1 to 620

Score:1140 bits(617), Expect:0.0,
Identities:619/620(99%), Gaps:0/620(0%), Strand: Plus/Plus

```
Query  39    TAGGACTTAGTATAAGAATATTAATTCGTATAGAATTAGGACAACCTGGAACTTTAATTG  98
             ||||||||||||||||||||||||||||||||||||||||||||||||||||||||||||
Sbjct  1     TAGGACTTAGTATAAGAATATTAATTCGTATAGAATTAGGACAACCTGGAACTTTAATTG  60

Query  99    GAAATGATCAGATTTATAATGTAATCGTAACAGCACATGCATTTATTATAAtttttttA  158
             ||||||||||||||||||||||||||||||||||||||||||||||||||||||||||||
Sbjct  61    GAAATGATCAGATTTATAATGTAATCGTAACAGCACATGCATTTATTATAATTTTTTTA  120

Query  159   TAGTTATACCAATCATAATCGGAGGATTTGGAAATTGATTAGTCCCTATCATATTGGGTG  218
             ||||||||||||||||||||||||||||||||||||||||||||||||||||||||||||
Sbjct  121   TAGTTATACCAATCATAATCGGAGGATTTGGAAATTGATTAGTCCCTATCATATTGGGTG  180

Query  219   CCCCAGATATAGCATTTCCACGAATAAATAATATAAGATTTTGACTTTTACCTCCTTCAC  278
             ||||||||||||||||||||||||||||||||||||||||||||||||||||||||||||
Sbjct  181   CCCCAGATATAGCATTTCCACGAATAAATAATATAAGATTTTGACTTTTACCTCCTTCAC  240

Query  279   TATTTTTATTAATTAACTCTTCATTAATTGAATCTGGAGCTGGTACAGGATGAACAGTTT  338
             |||||||||||||||| |||||||||||||||||||||||||||||||||||||||||||
Sbjct  241   TATTTTTATTAATTAATTCTTCATTAATTGAATCTGGAGCTGGTACAGGATGAACAGTTT  300

Query  339   ACCCTCCTCTATCTTCAAATTTATCACATTATGGCCCTTCAGTAGATTTAGCTATTTTTT  398
             ||||||||||||||||||||||||||||||||||||||||||||||||||||||||||||
Sbjct  301   ACCCTCCTCTATCTTCAAATTTATCACATTATGGCCCTTCAGTAGATTTAGCTATTTTTT  360

Query  399   CTCTTCATCTTGCTGGTGCTTCTTCAATTTTAGGTGCAATTAACTTTATTACAACTATTG  458
             ||||||||||||||||||||||||||||||||||||||||||||||||||||||||||||
Sbjct  361   CTCTTCATCTTGCTGGTGCTTCTTCAATTTTAGGTGCAATTAACTTTATTACAACTATTG  420

Query  459   TAAACATACGATCAATTGGAATAACAATAGAACGTATACCATTATTTGTTTGATCTGTTT  518
             ||||||||||||||||||||||||||||||||||||||||||||||||||||||||||||
Sbjct  421   TAAACATACGATCAATTGGAATAACAATAGAACGTATACCATTATTTGTTTGATCTGTTT  480

Query  519   TAATTACAGCTATTTTATTACTTTTATCTTTACCTGTATTAGCAGGTGCCATTACAATAT  578
             ||||||||||||||||||||||||||||||||||||||||||||||||||||||||||||
```

```
Sbjct  481   TAATTACAGCTATTTTATTACTTTTATCTTTACCTGTATTAGCAGGTGCCATTACAATAT  540

Query  579   TGTTAACTGATCGAAACTTTAATACATCATTTTTTGATCCTTCAGGGGGAGGAGATCCAA  638
             ||||||||||||||||||||||||||||||||||||||||||||||||||||||||||||
Sbjct  541   TGTTAACTGATCGAAACTTTAATACATCATTTTTTGATCCTTCAGGGGGAGGAGATCCAA  600

Query  639   TTTTATATCAACATTTATTT  658
             ||||||||||||||||||||
Sbjct  601   TTTTATATCAACATTTATTT  620
```

>Rhipicephalus sanguineus isolate Petnica cytochrome oxidase subunit I (COI) gene, partial cds; mitochondrial
Sequence ID: KX757883.1 Length: 630
Range 1: 1 to 620

Score:1140 bits(617), Expect:0.0,
Identities:619/620(99%), Gaps:0/620(0%), Strand: Plus/Plus

```
Query  39    TAGGACTTAGTATAAGAATATTAATTCGTATAGAATTAGGACAACCTGGAACTTTAATTG  98
             |||||||||||||||||||||||||||||||||| |||||||||||||||||||||||||
Sbjct  1     TAGGACTTAGTATAAGAATATTAATTCGTATAGAGTTAGGACAACCTGGAACTTTAATTG  60

Query  99    GAAATGATCAGATTTATAATGTAATCGTAACAGCACATGCATTTATTATAAtttttttA  158
             ||||||||||||||||||||||||||||||||||||||||||||||||||||||||||||
Sbjct  61    GAAATGATCAGATTTATAATGTAATCGTAACAGCACATGCATTTATTATAATTTTTTTA  120

Query  159   TAGTTATACCAATCATAATCGGAGGATTTGGAAATTGATTAGTCCCTATCATATTGGGTG  218
             ||||||||||||||||||||||||||||||||||||||||||||||||||||||||||||
Sbjct  121   TAGTTATACCAATCATAATCGGAGGATTTGGAAATTGATTAGTCCCTATCATATTGGGTG  180

Query  219   CCCCAGATATAGCATTTCCACGAATAAATAATATAAGATTTTGACTTTTACCTCCTTCAC  278
             ||||||||||||||||||||||||||||||||||||||||||||||||||||||||||||
Sbjct  181   CCCCAGATATAGCATTTCCACGAATAAATAATATAAGATTTTGACTTTTACCTCCTTCAC  240

Query  279   TATTTTTATTAATTAACTCTTCATTAATTGAATCTGGAGCTGGTACAGGATGAACAGTTT  338
             ||||||||||||||||||||||||||||||||||||||||||||||||||||||||||||
Sbjct  241   TATTTTTATTAATTAACTCTTCATTAATTGAATCTGGAGCTGGTACAGGATGAACAGTTT  300

Query  339   ACCCTCCTCTATCTTCAAATTTATCACATTATGGCCCTTCAGTAGATTTAGCTATTTTTT  398
             ||||||||||||||||||||||||||||||||||||||||||||||||||||||||||||
Sbjct  301   ACCCTCCTCTATCTTCAAATTTATCACATTATGGCCCTTCAGTAGATTTAGCTATTTTTT  360

Query  399   CTCTTCATCTTGCTGGTGCTTCTTCAATTTTAGGTGCAATTAACTTTATTACAACTATTG  458
             ||||||||||||||||||||||||||||||||||||||||||||||||||||||||||||
Sbjct  361   CTCTTCATCTTGCTGGTGCTTCTTCAATTTTAGGTGCAATTAACTTTATTACAACTATTG  420

Query  459   TAAACATACGATCAATTGGAATAACAATAGAACGTATACCATTATTTGTTTGATCTGTTT  518
             ||||||||||||||||||||||||||||||||||||||||||||||||||||||||||||
Sbjct  421   TAAACATACGATCAATTGGAATAACAATAGAACGTATACCATTATTTGTTTGATCTGTTT  480

Query  519   TAATTACAGCTATTTTATTACTTTTATCTTTACCTGTATTAGCAGGTGCCATTACAATAT  578
```

```
              ||||||||||||||||||||||||||||||||||||||||||||||||||||||||||||
Sbjct  481   TAATTACAGCTATTTTATTACTTTTATCTTTACCTGTATTAGCAGGTGCCATTACAATAT  540

Query  579   TGTTAACTGATCGAAACTTTAATACATCATTTTTTGATCCTTCAGGGGGAGGAGATCCAA  638
              ||||||||||||||||||||||||||||||||||||||||||||||||||||||||||||
Sbjct  541   TGTTAACTGATCGAAACTTTAATACATCATTTTTTGATCCTTCAGGGGGAGGAGATCCAA  600

Query  639   TTTTATATCAACATTTATTT  658
              ||||||||||||||||||||
Sbjct  601   TTTTATATCAACATTTATTT  620
```

>Rhipicephalus sanguineus clone #3 cytochrome oxidase subunit I (COI) gene, partial cds; mitochondrial
Sequence ID: KX519708.1 Length: 629
Range 1: 4 to 620

Score:1140 bits(617), Expect:0.0,
Identities:617/617(100%), Gaps:0/617(0%), Strand: Plus/Plus

```
Query  42    GACTTAGTATAAGAATATTAATTCGTATAGAATTAGGACAACCTGGAACTTTAATTGGAA  101
              ||||||||||||||||||||||||||||||||||||||||||||||||||||||||||||
Sbjct  4     GACTTAGTATAAGAATATTAATTCGTATAGAATTAGGACAACCTGGAACTTTAATTGGAA  63

Query  102   ATGATCAGATTTATAATGTAATCGTAACAGCACATGCATTTATTATAAttttttttATAG  161
              ||||||||||||||||||||||||||||||||||||||||||||||||||||||||||||
Sbjct  64    ATGATCAGATTTATAATGTAATCGTAACAGCACATGCATTTATTATAATTTTTTTTATAG  123

Query  162   TTATACCAATCATAATCGGAGGATTTGGAAATTGATTAGTCCCTATCATATTGGGTGCCC  221
              ||||||||||||||||||||||||||||||||||||||||||||||||||||||||||||
Sbjct  124   TTATACCAATCATAATCGGAGGATTTGGAAATTGATTAGTCCCTATCATATTGGGTGCCC  183

Query  222   CAGATATAGCATTTCCACGAATAAATAATATAAGATTTTGACTTTTACCTCCTTCACTAT  281
              ||||||||||||||||||||||||||||||||||||||||||||||||||||||||||||
Sbjct  184   CAGATATAGCATTTCCACGAATAAATAATATAAGATTTTGACTTTTACCTCCTTCACTAT  243

Query  282   TTTTATTAATTAACTCTTCATTAATTGAATCTGGAGCTGGTACAGGATGAACAGTTTACC  341
              ||||||||||||||||||||||||||||||||||||||||||||||||||||||||||||
Sbjct  244   TTTTATTAATTAACTCTTCATTAATTGAATCTGGAGCTGGTACAGGATGAACAGTTTACC  303

Query  342   CTCCTCTATCTTCAAATTTATCACATTATGGCCCTTCAGTAGATTTAGCTATTTTTTCTC  401
              ||||||||||||||||||||||||||||||||||||||||||||||||||||||||||||
Sbjct  304   CTCCTCTATCTTCAAATTTATCACATTATGGCCCTTCAGTAGATTTAGCTATTTTTTCTC  363

Query  402   TTCATCTTGCTGGTGCTTCTTCAATTTTAGGTGCAATTAACTTTATTACAACTATTGTAA  461
              ||||||||||||||||||||||||||||||||||||||||||||||||||||||||||||
Sbjct  364   TTCATCTTGCTGGTGCTTCTTCAATTTTAGGTGCAATTAACTTTATTACAACTATTGTAA  423

Query  462   ACATACGATCAATTGGAATAACAATAGAACGTATACCATTATTTGTTTGATCTGTTTTAA  521
              ||||||||||||||||||||||||||||||||||||||||||||||||||||||||||||
Sbjct  424   ACATACGATCAATTGGAATAACAATAGAACGTATACCATTATTTGTTTGATCTGTTTTAA  483
```

```
Query  522  TTACAGCTATTTTATTACTTTTATCTTTACCTGTATTAGCAGGTGCCATTACAATATTGT  581
            ||||||||||||||||||||||||||||||||||||||||||||||||||||||||||||
Sbjct  484  TTACAGCTATTTTATTACTTTTATCTTTACCTGTATTAGCAGGTGCCATTACAATATTGT  543

Query  582  TAACTGATCGAAACTTTAATACATCATTTTTTGATCCTTCAGGGGGAGGAGATCCAATTT  641
            ||||||||||||||||||||||||||||||||||||||||||||||||||||||||||||
Sbjct  544  TAACTGATCGAAACTTTAATACATCATTTTTTGATCCTTCAGGGGGAGGAGATCCAATTT  603

Query  642  TATATCAACATTTATTT  658
            |||||||||||||||||
Sbjct  604  TATATCAACATTTATTT  620
```

>Rhipicephalus sanguineus isolate SV2 cytochrome c oxidase subunit I (COX1) gene, partial cds; mitochondrial
Sequence ID: MW135447.1 Length: 630
Range 1: 1 to 620

Score:1140 bits(617), Expect:0.0,
Identities:619/620(99%), Gaps:0/620(0%), Strand: Plus/Plus

```
Query  39   TAGGACTTAGTATAAGAATATTAATTCGTATAGAATTAGGACAACCTGGAACTTTAATTG  98
            ||||||||||||||||||||||||||||||||||||||||||||||||||||||||||||
Sbjct  1    TAGGACTTAGTATAAGAATATTAATTCGTATAGAATTAGGACAACCTGGAACTTTAATTG  60

Query  99   GAAATGATCAGATTTATAATGTAATCGTAACAGCACATGCATTTATTATAAtttttttA  158
            ||||||||||||||||||||||||||||||||||||||||||||||||||||||||||||
Sbjct  61   GAAATGATCAGATTTATAATGTAATCGTAACAGCACATGCATTTATTATAATTTTTTTA  120

Query  159  TAGTTATACCAATCATAATCGGAGGATTTGGAAATTGATTAGTCCCTATCATATTGGGTG  218
            ||||||||||||||||||||||||||||||||||||||||||||||||||||||||||||
Sbjct  121  TAGTTATACCAATCATAATCGGAGGATTTGGAAATTGATTAGTCCCTATCATATTGGGTG  180

Query  219  CCCCAGATATAGCATTTCCACGAATAAATAATATAAGATTTTGACTTTTACCTCCTTCAC  278
            ||||||||||||||||||||||||||||||||||||||||||||||||||||||||||||
Sbjct  181  CCCCAGATATAGCATTTCCACGAATAAATAATATAAGATTTTGACTTTTACCTCCTTCAC  240

Query  279  TATTTTTATTAATTAACTCTTCATTAATTGAATCTGGAGCTGGTACAGGATGAACAGTTT  338
            ||||||||||||||||||||||||||||||||||||||||||||||||||||||||||||
Sbjct  241  TATTTTTATTAATTAACTCTTCATTAATTGAATCTGGAGCTGGTACAGGATGAACAGTTT  300

Query  339  ACCCTCCTCTATCTTCAAATTTATCACATTATGGCCCTTCAGTAGATTTAGCTATTTTTT  398
            ||||||||||||||||||||||||||||||||||||||||||||||||||||||||||||
Sbjct  301  ACCCTCCTCTATCTTCAAATTTATCACATTATGGCCCTTCAGTAGATTTAGCTATTTTTT  360

Query  399  CTCTTCATCTTGCTGGTGCTTCTTCAATTTTAGGTGCAATTAACTTTATTACAACTATTG  458
            ||||||||||||||||||||||||||||||||||||||||||||||||||||||||||||
Sbjct  361  CTCTTCATCTTGCTGGTGCTTCTTCAATTTTAGGTGCAATTAACTTTATTACAACTATTG  420

Query  459  TAAACATACGATCAATTGGAATAACAATAGAACGTATACCATTATTTGTTTGATCTGTTT  518
            ||||||||||||||||||||||||||||||||||||||||||||||||||||||||||||
Sbjct  421  TAAACATACGATCAATTGGAATAACAATAGAACGTATACCATTATTTGTTTGATCTGTTT  480
```

```
Query  519   TAATTACAGCTATTTTATTACTTTTATCTTTACCTGTATTAGCAGGTGCCATTACAATAT  578
             ||||||||||||||||||||||||||||||||||||||||||||||||||||||||||||
Sbjct  481   TAATTACAGCTATTTTATTACTTTTATCTTTACCTGTATTAGCAGGTGCCATTACAATAT  540

Query  579   TGTTAACTGATCGAAACTTTAATACATCATTTTTTGATCCTTCAGGGGGAGGAGATCCAA  638
             ||||||||||||||||||||||||||||||||||||||||||||||||||||||| ||||
Sbjct  541   TGTTAACTGATCGAAACTTTAATACATCATTTTTTGATCCTTCAGGGGGAGGAGACCCAA  600

Query  639   TTTTATATCAACATTTATTT  658
             ||||||||||||||||||||
Sbjct  601   TTTTATATCAACATTTATTT  620
```

>Rhipicephalus sanguineus sensu lato 'temperate lineage' isolate S475 cytochrome c oxidase subunit I (COI) gene, partial cds; mitochondrial
Sequence ID: MF426014.1 Length: 649
Range 1: 28 to 649

Score:1138 bits(616), Expect:0.0,
Identities:620/622(99%), Gaps:0/622(0%), Strand: Plus/Plus

```
Query  1     AACAATATATTTAATTTTTGGAGCATGATCAGGTATATTAGGACTTAGTATAAGAATATT  60
             ||||||||||||||||||||||||||||||||||||||||||||||||||||||||||||
Sbjct  28    AACAATATATTTAATTTTTGGAGCATGATCAGGTATATTAGGACTTAGTATAAGAATATT  87

Query  61    AATTCGTATAGAATTAGGACAACCTGGAACTTTAATTGGAAATGATCAGATTTATAATGT  120
             ||||||||||||||||||||||||||||||||||||||||||||||||||||||||||||
Sbjct  88    AATTCGTATAGAATTAGGACAACCTGGAACTTTAATTGGAAATGATCAGATTTATAATGT  147

Query  121   AATCGTAACAGCACATGCATTTATTATAAttttttttATAGTTATACCAATCATAATCGG  180
             ||||||||||||||||||||||||||||||||||||||||||||||||||||||||||||
Sbjct  148   AATCGTAACAGCACATGCATTTATTATAATTTTTTTTATAGTTATACCAATCATAATCGG  207

Query  181   AGGATTTGGAAATTGATTAGTCCCTATCATATTGGGTGCCCCAGATATAGCATTTCCACG  240
             ||||||||||||||||||||||||||||||||||||||||||||||||||||||||||||
Sbjct  208   AGGATTTGGAAATTGATTAGTCCCTATCATATTGGGTGCCCCAGATATAGCATTTCCACG  267

Query  241   AATAAATAATATAAGATTTTGACTTTTACCTCCTTCACTATTTTTATTAATTAACTCTTC  300
             ||||||||||||||||||||||||||||||||||||||||||||||||||||||||||||
Sbjct  268   AATAAATAATATAAGATTTTGACTTTTACCTCCTTCACTATTTTTATTAATTAACTCTTC  327

Query  301   ATTAATTGAATCTGGAGCTGGTACAGGATGAACAGTTTACCCTCCTCTATCTTCAAATTT  360
             ||||||||||||||||||||||||||||||||||||||||||||||||||||||||||||
Sbjct  328   ATTAATTGAATCTGGAGCTGGTACAGGATGAACAGTTTACCCTCCTCTATCTTCAAATTT  387

Query  361   ATCACATTATGGCCCTTCAGTAGATTTAGCTATTTTTTCTCTTCATCTTGCTGGTGCTTC  420
             |||||||||||| |||||||||||||||||||||||||||||||||||||||||||||||
Sbjct  388   ATCACATTATGGTCCTTCAGTAGATTTAGCTATTTTTTCTCTTCATCTTGCTGGTGCTTC  447

Query  421   TTCAATTTTAGGTGCAATTAACTTTATTACAACTATTGTAAACATACGATCAATTGGAAT  480
             ||||||||||||||||||||||||||||||||||||||||||||||||||||||||||||
```

```
Sbjct  448  TTCAATTTTAGGTGCAATTAACTTTATTACAACTATTGTAAACATACGATCAATTGGAAT  507

Query  481  AACAATAGAACGTATACCATTATTTGTTTGATCTGTTTTAATTACAGCTATTTTATTACT  540
            |||||||||||||||||||||||||||||||||||||||||||||||||||||| ||||
Sbjct  508  AACAATAGAACGTATACCATTATTTGTTTGATCTGTTTTAATTACAGCTATTTTACTACT  567

Query  541  TTTATCTTTACCTGTATTAGCAGGTGCCATTACAATATTGTTAACTGATCGAAACTTTAA  600
            ||||||||||||||||||||||||||||||||||||||||||||||||||||||||||||
Sbjct  568  TTTATCTTTACCTGTATTAGCAGGTGCCATTACAATATTGTTAACTGATCGAAACTTTAA  627

Query  601  TACATCATTTTTTGATCCTTCA  622
            ||||||||||||||||||||||
Sbjct  628  TACATCATTTTTTGATCCTTCA  649
```

>Rhipicephalus sanguineus clone #2 cytochrome oxidase subunit I (COI) gene, partial cds; mitochondrial
Sequence ID: KX519707.1 Length: 626
Range 1: 1 to 616

Score:1138 bits(616), Expect:0.0,
Identities:616/616(100%), Gaps:0/616(0%), Strand: Plus/Plus

```
Query  43   ACTTAGTATAAGAATATTAATTCGTATAGAATTAGGACAACCTGGAACTTTAATTGGAAA  102
            ||||||||||||||||||||||||||||||||||||||||||||||||||||||||||||
Sbjct  1    ACTTAGTATAAGAATATTAATTCGTATAGAATTAGGACAACCTGGAACTTTAATTGGAAA  60

Query  103  TGATCAGATTTATAATGTAATCGTAACAGCACATGCATTTATTATAAttttttttATAGT  162
            ||||||||||||||||||||||||||||||||||||||||||||||||||||||||||||
Sbjct  61   TGATCAGATTTATAATGTAATCGTAACAGCACATGCATTTATTATAATTTTTTTTATAGT  120

Query  163  TATACCAATCATAATCGGAGGATTTGGAAATTGATTAGTCCCTATCATATTGGGTGCCCC  222
            ||||||||||||||||||||||||||||||||||||||||||||||||||||||||||||
Sbjct  121  TATACCAATCATAATCGGAGGATTTGGAAATTGATTAGTCCCTATCATATTGGGTGCCCC  180

Query  223  AGATATAGCATTTCCACGAATAAATAATATAAGATTTTGACTTTTACCTCCTTCACTATT  282
            ||||||||||||||||||||||||||||||||||||||||||||||||||||||||||||
Sbjct  181  AGATATAGCATTTCCACGAATAAATAATATAAGATTTTGACTTTTACCTCCTTCACTATT  240

Query  283  TTTATTAATTAACTCTTCATTAATTGAATCTGGAGCTGGTACAGGATGAACAGTTTACCC  342
            ||||||||||||||||||||||||||||||||||||||||||||||||||||||||||||
Sbjct  241  TTTATTAATTAACTCTTCATTAATTGAATCTGGAGCTGGTACAGGATGAACAGTTTACCC  300

Query  343  TCCTCTATCTTCAAATTTATCACATTATGGCCCTTCAGTAGATTTAGCTATTTTTTCTCT  402
            ||||||||||||||||||||||||||||||||||||||||||||||||||||||||||||
Sbjct  301  TCCTCTATCTTCAAATTTATCACATTATGGCCCTTCAGTAGATTTAGCTATTTTTTCTCT  360

Query  403  TCATCTTGCTGGTGCTTCTTCAATTTTAGGTGCAATTAACTTTATTACAACTATTGTAAA  462
            ||||||||||||||||||||||||||||||||||||||||||||||||||||||||||||
Sbjct  361  TCATCTTGCTGGTGCTTCTTCAATTTTAGGTGCAATTAACTTTATTACAACTATTGTAAA  420

Query  463  CATACGATCAATTGGAATAACAATAGAACGTATACCATTATTTGTTTGATCTGTTTTAAT  522
```

```
           ||||||||||||||||||||||||||||||||||||||||||||||||||||||||||||
Sbjct  421  CATACGATCAATTGGAATAACAATAGAACGTATACCATTATTTGTTTGATCTGTTTTAAT  480

Query  523  TACAGCTATTTTATTACTTTTATCTTTACCTGTATTAGCAGGTGCCATTACAATATTGTT  582
            ||||||||||||||||||||||||||||||||||||||||||||||||||||||||||||
Sbjct  481  TACAGCTATTTTATTACTTTTATCTTTACCTGTATTAGCAGGTGCCATTACAATATTGTT  540

Query  583  AACTGATCGAAACTTTAATACATCATTTTTTGATCCTTCAGGGGGAGGAGATCCAATTTT  642
            ||||||||||||||||||||||||||||||||||||||||||||||||||||||||||||
Sbjct  541  AACTGATCGAAACTTTAATACATCATTTTTTGATCCTTCAGGGGGAGGAGATCCAATTTT  600

Query  643  ATATCAACATTTATTT  658
            ||||||||||||||||
Sbjct  601  ATATCAACATTTATTT  616
```

>Rhipicephalus sanguineus isolate Szekszard cytochrome oxidase subunit I (COI) gene, partial cds; mitochondrial
Sequence ID: KX757901.1 Length: 630
Range 1: 1 to 620

Score:1134 bits(614), Expect:0.0,
Identities:618/620(99%), Gaps:0/620(0%), Strand: Plus/Plus

```
Query  39   TAGGACTTAGTATAAGAATATTAATTCGTATAGAATTAGGACAACCTGGAACTTTAATTG  98
            ||||||||||||||||||||||||||||||||||||||||||||||||||||||||||||
Sbjct  1    TAGGACTTAGTATAAGAATATTAATTCGTATAGAATTAGGACAACCTGGAACTTTAATTG  60

Query  99   GAAATGATCAGATTTATAATGTAATCGTAACAGCACATGCATTTATTATAAtttttttA  158
            ||||||||||||||||||||||||||||||||||||||||||||||||||||||||||||
Sbjct  61   GAAATGATCAGATTTATAATGTAATCGTAACAGCACATGCATTTATTATAATTTTTTTA  120

Query  159  TAGTTATACCAATCATAATCGGAGGATTTGGAAATTGATTAGTCCCTATCATATTGGGTG  218
            ||||||||||||||||||||||||||||||||||||||||||||||||||||||||||||
Sbjct  121  TAGTTATACCAATCATAATCGGAGGATTTGGAAATTGATTAGTCCCTATCATATTGGGTG  180

Query  219  CCCCAGATATAGCATTTCCACGAATAAATAATATAAGATTTTGACTTTTACCTCCTTCAC  278
            ||||||||||||||||||||||||||||||||||||||||||||||||||||||||||||
Sbjct  181  CCCCAGATATAGCATTTCCACGAATAAATAATATAAGATTTTGACTTTTACCTCCTTCAC  240

Query  279  TATTTTTATTAATTAACTCTTCATTAATTGAATCTGGAGCTGGTACAGGATGAACAGTTT  338
            ||||||||||||||||||||||||||||||||||||||||||||||||||||||||||||
Sbjct  241  TATTTTTATTAATTAACTCTTCATTAATTGAATCTGGAGCTGGTACAGGATGAACAGTTT  300

Query  339  ACCCTCCTCTATCTTCAAATTTATCACATTATGGCCCTTCAGTAGATTTAGCTATTTTTT  398
            ||||||||||||||||||||||||||||||||||||||||||||||||||||||| ||||
Sbjct  301  ACCCTCCTCTATCTTCAAATTTATCACATTATGGCCCTTCAGTAGATTTAGCTATCTTTT  360

Query  399  CTCTTCATCTTGCTGGTGCTTCTTCAATTTTAGGTGCAATTAACTTTATTACAACTATTG  458
            ||||||||||||||||||||||||||||||||||||||||||||||||||||||||||||
Sbjct  361  CTCTTCATCTTGCTGGTGCTTCTTCAATTTTAGGTGCAATTAACTTTATTACAACTATTG  420
```

```
Query  459   TAAACATACGATCAATTGGAATAACAATAGAACGTATACCATTATTTGTTTGATCTGTTT  518
             |||||||||| |||||||||||||||||||||||||||||||||||||||||||||||||
Sbjct  421   TAAACATACGGTCAATTGGAATAACAATAGAACGTATACCATTATTTGTTTGATCTGTTT  480

Query  519   TAATTACAGCTATTTTATTACTTTTATCTTTACCTGTATTAGCAGGTGCCATTACAATAT  578
             ||||||||||||||||||||||||||||||||||||||||||||||||||||||||||||
Sbjct  481   TAATTACAGCTATTTTATTACTTTTATCTTTACCTGTATTAGCAGGTGCCATTACAATAT  540

Query  579   TGTTAACTGATCGAAACTTTAATACATCATTTTTTGATCCTTCAGGGGGAGGAGATCCAA  638
             ||||||||||||||||||||||||||||||||||||||||||||||||||||||||||||
Sbjct  541   TGTTAACTGATCGAAACTTTAATACATCATTTTTTGATCCTTCAGGGGGAGGAGATCCAA  600

Query  639   TTTTATATCAACATTTATTT  658
             ||||||||||||||||||||
Sbjct  601   TTTTATATCAACATTTATTT  620
```

>Rhipicephalus sanguineus voucher Rs7 cytochrome oxidase subunit I (COI) gene, partial cds; mitochondrial
Sequence ID: KU568516.1 Length: 656
Range 1: 27 to 656

Score:1131 bits(612), Expect:0.0,
Identities:624/630(99%), Gaps:0/630(0%), Strand: Plus/Plus

```
Query  1     AACAATATATTTAATTTTTGGAGCATGATCAGGTATATTAGGACTTAGTATAAGAATATT  60
             ||||||||||||||||||||||||||||||||||||||||||||||||||||||||||||
Sbjct  27    AACAATATATTTAATTTTTGGAGCATGATCAGGTATATTAGGACTTAGTATAAGAATATT  86

Query  61    AATTCGTATAGAATTAGGACAACCTGGAACTTTAATTGGAAATGATCAGATTTATAATGT  120
             ||||||||||||||||||||||||||||||||||||||||||||||||||||||||||||
Sbjct  87    AATTCGTATAGAATTAGGACAACCTGGAACTTTAATTGGAAATGATCAGATTTATAATGT  146

Query  121   AATCGTAACAGCACATGCATTTATTATAAtttttttATAGTTATACCAATCATAATCGG  180
             ||||||||||||||||||||||||||||||||||||||||||||||||||||||||||||
Sbjct  147   AATCGTAACAGCACATGCATTTATTATAATTTTTTTATAGTTATACCAATCATAATCGG  206

Query  181   AGGATTTGGAAATTGATTAGTCCCTATCATATTGGGTGCCCCAGATATAGCATTTCCACG  240
             ||||||||||||||||||| ||||||||||||| ||||||||||||||||||||||||||
Sbjct  207   AGGATTTGGAAATTGATTAATCCCTATCATATTAGGTGCCCCAGATATAGCATTTCCACG  266

Query  241   AATAAATAATATAAGATTTTGACTTTTACCTCCTTCACTATTTTTATTAATTAACTCTTC  300
             ||||||||||||||||||||||||||||||||||||||||||||||||||||||||||||
Sbjct  267   AATAAATAATATAAGATTTTGACTTTTACCTCCTTCACTATTTTTATTAATTAACTCTTC  326

Query  301   ATTAATTGAATCTGGAGCTGGTACAGGATGAACAGTTTACCCTCCTCTATCTTCAAATTT  360
             ||||||||||||||||||||||||||||||||||||||||||||||||||||||||||||
Sbjct  327   ATTAATTGAATCTGGAGCTGGTACAGGATGAACAGTTTACCCTCCTCTATCTTCAAATTT  386

Query  361   ATCACATTATGGCCCTTCAGTAGATTTAGCTATTTTTTCTCTTCATCTTGCTGGTGCTTC  420
             ||||||||||||||||||||||||||||||||| ||||||||||||||||||||||||||
Sbjct  387   ATCACATTATGGCCCTTCAGTAGATTTAGCTATCTTTTCTCTTCATCTTGCTGGTGCTTC  446
```

```
Query  421   TTCAATTTTAGGTGCAATTAACTTTATTACAACTATTGTAAACATACGATCAATTGGAAT  480
             ||||||||||||||||||||||||||||||||||||||||||||||||||||||||||||
Sbjct  447   TTCAATTTTAGGTGCAATTAACTTTATTACAACTATTGTAAACATACGATCAATTGGAAT  506

Query  481   AACAATAGAACGTATACCATTATTTGTTTGATCTGTTTTAATTACAGCTATTTTATTACT  540
             ||||||||||||||||||||||||||||||||||||||||||||||||||||||||||||
Sbjct  507   AACAATAGAACGTATACCATTATTTGTTTGATCTGTTTTAATTACAGCTATTTTATTACT  566

Query  541   TTTATCTTTACCTGTATTAGCAGGTGCCATTACAATATTGTTAACTGATCGAAACTTTAA  600
              |||||||||||||||||||||||||||||||||||||||||||||||||||||||||||
Sbjct  567   CTTATCTTTACCTGTATTAGCAGGTGCCATTACAATATTGTTAACTGATCGAAACTTTAA  626

Query  601   TACATCATTTTTTGATCCTTCAGGGGGAGG  630
             ||||||||||||||| |||||||| |||||
Sbjct  627   TACATCATTTTTTGACCCTTCAGGAGGAGG  656
```

>Rhipicephalus sanguineus clone #4 cytochrome oxidase subunit I (COI) gene, partial cds; mitochondrial
Sequence ID: KX519709.1 Length: 635
Range 1: 2 to 621

Score:1129 bits(611), Expect:0.0,
Identities:617/620(99%), Gaps:0/620(0%), Strand: Plus/Plus

```
Query  39    TAGGACTTAGTATAAGAATATTAATTCGTATAGAATTAGGACAACCTGGAACTTTAATTG  98
             ||||||||||||||||||||||||||||||||||||||||||||||||||||||||||||
Sbjct  2     TAGGACTTAGTATAAGAATATTAATTCGTATAGAATTAGGACAACCTGGAACTTTAATTG  61

Query  99    GAAATGATCAGATTTATAATGTAATCGTAACAGCACATGCATTTATTATAAtttttttA  158
             |||||||||||||||||||||||||||||||||||||||| |||||||||||||||||||
Sbjct  62    GAAATGATCAGATTTATAATGTAATCGTAACAGCACATGCTTTTATTATAATTTTTTTTA  121

Query  159   TAGTTATACCAATCATAATCGGAGGATTTGGAAATTGATTAGTCCCTATCATATTGGGTG  218
             ||||||||||||||||||||||||||||||||||||||||||||||||||||||||||||
Sbjct  122   TAGTTATACCAATCATAATCGGAGGATTTGGAAATTGATTAGTCCCTATCATATTGGGTG  181

Query  219   CCCCAGATATAGCATTTCCACGAATAAATAATATAAGATTTTGACTTTTACCTCCTTCAC  278
             |||| |||||||||||||||||||||||||||||||||||||||||||||||||||||||
Sbjct  182   CCCCGGATATAGCATTTCCACGAATAAATAATATAAGATTTTGACTTTTACCTCCTTCAC  241

Query  279   TATTTTTATTAATTAACTCTTCATTAATTGAATCTGGAGCTGGTACAGGATGAACAGTTT  338
             ||||||||||||||||||||||||||||||||||||||||||||||||||||||||||||
Sbjct  242   TATTTTTATTAATTAACTCTTCATTAATTGAATCTGGAGCTGGTACAGGATGAACAGTTT  301

Query  339   ACCCTCCTCTATCTTCAAATTTATCACATTATGGCCCTTCAGTAGATTTAGCTATTTTTT  398
             |||||||||||||||||||||||||||||||||||||||||| |||||||||||||||||
Sbjct  302   ACCCTCCTCTATCTTCAAATTTATCACATTATGGCCCTTCAGGAGATTTAGCTATTTTTT  361

Query  399   CTCTTCATCTTGCTGGTGCTTCTTCAATTTTAGGTGCAATTAACTTTATTACAACTATTG  458
             ||||||||||||||||||||||||||||||||||||||||||||||||||||||||||||
```

```
Sbjct  362   CTCTTCATCTTGCTGGTGCTTCTTCAATTTTAGGTGCAATTAACTTTATTACAACTATTG  421

Query  459   TAAACATACGATCAATTGGAATAACAATAGAACGTATACCATTATTTGTTTGATCTGTTT  518
             ||||||||||||||||||||||||||||||||||||||||||||||||||||||||||||
Sbjct  422   TAAACATACGATCAATTGGAATAACAATAGAACGTATACCATTATTTGTTTGATCTGTTT  481

Query  519   TAATTACAGCTATTTTATTACTTTTATCTTTACCTGTATTAGCAGGTGCCATTACAATAT  578
             ||||||||||||||||||||||||||||||||||||||||||||||||||||||||||||
Sbjct  482   TAATTACAGCTATTTTATTACTTTTATCTTTACCTGTATTAGCAGGTGCCATTACAATAT  541

Query  579   TGTTAACTGATCGAAACTTTAATACATCATTTTTTGATCCTTCAGGGGGAGGAGATCCAA  638
             ||||||||||||||||||||||||||||||||||||||||||||||||||||||||||||
Sbjct  542   TGTTAACTGATCGAAACTTTAATACATCATTTTTTGATCCTTCAGGGGGAGGAGATCCAA  601

Query  639   TTTTATATCAACATTTATTT  658
             ||||||||||||||||||||
Sbjct  602   TTTTATATCAACATTTATTT  621
```

>Rhipicephalus sanguineus isolate LIC4723B cytochrome oxidase subunit 1 (COI) gene, partial cds; mitochondrial
Sequence ID: KX714599.1 Length: 723
>Rhipicephalus sanguineus isolate LIC4750B cytochrome oxidase subunit 1 (COI) gene, partial cds; mitochondrial
Sequence ID: KX714600.1 Length: 723
Range 1: 1 to 597

Score:1092 bits(591), Expect:0.0,
Identities:595/597(99%), Gaps:0/597(0%), Strand: Plus/Plus

```
Query  62    ATTCGTATAGAATTAGGACAACCTGGAACTTTAATTGGAAATGATCAGATTTATAATGTA  121
             ||||||||||||||||||||||||||||||||||||||||||||||||||||||||||||
Sbjct  1     ATTCGTATAGAATTAGGACAACCTGGAACTTTAATTGGAAATGATCAGATTTATAATGTA  60

Query  122   ATCGTAACAGCACATGCATTTATTATAAttttttttATAGTTATACCAATCATAATCGGA  181
             |||||||||||||||||||||||||||||||||||||||||||||||||||||||| |||
Sbjct  61    ATCGTAACAGCACATGCATTTATTATAATTTTTTTTATAGTTATACCAATCATAATTGGA  120

Query  182   GGATTTGGAAATTGATTAGTCCCTATCATATTGGGTGCCCCAGATATAGCATTTCCACGA  241
             ||||||||||||||||||||||||||||||||||||||||||||||||||||||||||||
Sbjct  121   GGATTTGGAAATTGATTAGTCCCTATCATATTGGGTGCCCCAGATATAGCATTTCCACGA  180

Query  242   ATAAATAATATAAGATTTTGACTTTTACCTCCTTCACTATTTTTATTAATTAACTCTTCA  301
             ||||||||||||||||||||||||||||||||||||||||||||||||||||||||||||
Sbjct  181   ATAAATAATATAAGATTTTGACTTTTACCTCCTTCACTATTTTTATTAATTAACTCTTCA  240

Query  302   TTAATTGAATCTGGAGCTGGTACAGGATGAACAGTTTACCCTCCTCTATCTTCAAATTTA  361
             ||||||||||||||||||||||||||||||||||||||||||||||||||||||||||||
Sbjct  241   TTAATTGAATCTGGAGCTGGTACAGGATGAACAGTTTACCCTCCTCTATCTTCAAATTTA  300

Query  362   TCACATTATGGCCCTTCAGTAGATTTAGCTATTTTTTCTCTTCATCTTGCTGGTGCTTCT  421
             |||||||||||||||||||||||||||||||| |||||||||||||||||||||||||||
```

```
Sbjct  301   TCACATTATGGCCCTTCAGTAGATTTAGCTATCTTTTCTCTTCATCTTGCTGGTGCTTCT  360

Query  422   TCAATTTTAGGTGCAATTAACTTTATTACAACTATTGTAAACATACGATCAATTGGAATA  481
             ||||||||||||||||||||||||||||||||||||||||||||||||||||||||||||
Sbjct  361   TCAATTTTAGGTGCAATTAACTTTATTACAACTATTGTAAACATACGATCAATTGGAATA  420

Query  482   ACAATAGAACGTATACCATTATTTGTTTGATCTGTTTTAATTACAGCTATTTTATTACTT  541
             ||||||||||||||||||||||||||||||||||||||||||||||||||||||||||||
Sbjct  421   ACAATAGAACGTATACCATTATTTGTTTGATCTGTTTTAATTACAGCTATTTTATTACTT  480

Query  542   TTATCTTTACCTGTATTAGCAGGTGCCATTACAATATTGTTAACTGATCGAAACTTTAAT  601
             ||||||||||||||||||||||||||||||||||||||||||||||||||||||||||||
Sbjct  481   TTATCTTTACCTGTATTAGCAGGTGCCATTACAATATTGTTAACTGATCGAAACTTTAAT  540

Query  602   ACATCATTTTTTGATCCTTCAGGGGGAGGAGATCCAATTTTATATCAACATTTATTT  658
             |||||||||||||||||||||||||||||||||||||||||||||||||||||||||
Sbjct  541   ACATCATTTTTTGATCCTTCAGGGGGAGGAGATCCAATTTTATATCAACATTTATTT  597
```

>Rhipicephalus sanguineus clone #8 cytochrome oxidase subunit I (COI) gene, partial cds; mitochondrial
Sequence ID: KX519713.1 Length: 580
Range 1: 1 to 580

Score:1072 bits(580), Expect:0.0,
Identities:580/580(100%), Gaps:0/580(0%), Strand: Plus/Plus

```
Query  43    ACTTAGTATAAGAATATTAATTCGTATAGAATTAGGACAACCTGGAACTTTAATTGGAAA  102
             ||||||||||||||||||||||||||||||||||||||||||||||||||||||||||||
Sbjct  1     ACTTAGTATAAGAATATTAATTCGTATAGAATTAGGACAACCTGGAACTTTAATTGGAAA  60

Query  103   TGATCAGATTTATAATGTAATCGTAACAGCACATGCATTTATTATAAtttttttATAGT  162
             ||||||||||||||||||||||||||||||||||||||||||||||||||||||||||||
Sbjct  61    TGATCAGATTTATAATGTAATCGTAACAGCACATGCATTTATTATAATTTTTTTATAGT  120

Query  163   TATACCAATCATAATCGGAGGATTTGGAAATTGATTAGTCCCTATCATATTGGGTGCCCC  222
             ||||||||||||||||||||||||||||||||||||||||||||||||||||||||||||
Sbjct  121   TATACCAATCATAATCGGAGGATTTGGAAATTGATTAGTCCCTATCATATTGGGTGCCCC  180

Query  223   AGATATAGCATTTCCACGAATAAATAATATAAGATTTTGACTTTTACCTCCTTCACTATT  282
             ||||||||||||||||||||||||||||||||||||||||||||||||||||||||||||
Sbjct  181   AGATATAGCATTTCCACGAATAAATAATATAAGATTTTGACTTTTACCTCCTTCACTATT  240

Query  283   TTTATTAATTAACTCTTCATTAATTGAATCTGGAGCTGGTACAGGATGAACAGTTTACCC  342
             ||||||||||||||||||||||||||||||||||||||||||||||||||||||||||||
Sbjct  241   TTTATTAATTAACTCTTCATTAATTGAATCTGGAGCTGGTACAGGATGAACAGTTTACCC  300

Query  343   TCCTCTATCTTCAAATTTATCACATTATGGCCCTTCAGTAGATTTAGCTATTTTTTCTCT  402
             ||||||||||||||||||||||||||||||||||||||||||||||||||||||||||||
Sbjct  301   TCCTCTATCTTCAAATTTATCACATTATGGCCCTTCAGTAGATTTAGCTATTTTTTCTCT  360

Query  403   TCATCTTGCTGGTGCTTCTTCAATTTTAGGTGCAATTAACTTTATTACAACTATTGTAAA  462
```

```
                ||||||||||||||||||||||||||||||||||||||||||||||||||||||||||||
Sbjct  361   TCATCTTGCTGGTGCTTCTTCAATTTTAGGTGCAATTAACTTTATTACAACTATTGTAAA  420

Query  463   CATACGATCAATTGGAATAACAATAGAACGTATACCATTATTTGTTTGATCTGTTTTAAT  522
             ||||||||||||||||||||||||||||||||||||||||||||||||||||||||||||
Sbjct  421   CATACGATCAATTGGAATAACAATAGAACGTATACCATTATTTGTTTGATCTGTTTTAAT  480

Query  523   TACAGCTATTTTATTACTTTTATCTTTACCTGTATTAGCAGGTGCCATTACAATATTGTT  582
             ||||||||||||||||||||||||||||||||||||||||||||||||||||||||||||
Sbjct  481   TACAGCTATTTTATTACTTTTATCTTTACCTGTATTAGCAGGTGCCATTACAATATTGTT  540

Query  583   AACTGATCGAAACTTTAATACATCATTTTTTGATCCTTCA  622
             ||||||||||||||||||||||||||||||||||||||||
Sbjct  541   AACTGATCGAAACTTTAATACATCATTTTTTGATCCTTCA  580
```

>Rhipicephalus sanguineus mitochondrial DNA, complete genome
Sequence ID: AF081829.1 Length: 14710
Range 1: 1191 to 1848

Score:1061 bits(574), Expect:0.0,
Identities:630/658(96%), Gaps:0/658(0%), Strand: Plus/Plus

```
Query  1      AACAATATATTTAATTTTTGGAGCATGATCAGGTATATTAGGACTTAGTATAAGAATATT  60
              ||||||||||||||||||||||||||||||||||||||||||||||||||||||||||||
Sbjct  1191   AACAATATATTTAATTTTTGGAGCATGATCAGGTATATTAGGACTTAGTATAAGAATATT  1250

Query  61     AATTCGTATAGAATTAGGACAACCTGGAACTTTAATTGGAAATGATCAGATTTATAATGT  120
              ||||||||||||||||||||||||||||||||||||||||||||||||||||||||||||
Sbjct  1251   AATTCGTATAGAATTAGGACAACCTGGAACTTTAATTGGAAATGATCAGATTTATAATGT  1310

Query  121    AATCGTAACAGCACATGCATTTATTATAAttttttttATAGTTATACCAATCATAATCGG  180
              ||||||||||||||||||||||||||||||||| ||||||||||||||||||||||||||
Sbjct  1311   AATCGTAACAGCACATGCATTTATTATAATTTTCTTTATAGTTATACCAATCATAATCGG  1370

Query  181    AGGATTTGGAAATTGATTAGTCCCTATCATATTGGGTGCCCCAGATATAGCATTTCCACG  240
              ||| || |||||||||||||| ||||| ||||| ||||||||||||||||||||||||||
Sbjct  1371   AGGGTTCGGAAATTGATTAGTTCCTATTATATTAGGTGCCCCAGATATAGCATTTCCACG  1430

Query  241    AATAAATAATATAAGATTTTGACTTTTACCTCCTTCACTATTTTTATTAATTAACTCTTC  300
              |||||||||||||||||||||||||||||| || ||| |||||||||| |||||||||||
Sbjct  1431   AATAAATAATATAAGATTTTGACTTTTACCCCCCTCATTATTTTTATTGATTAACTCTTC  1490

Query  301    ATTAATTGAATCTGGAGCTGGTACAGGATGAACAGTTTACCCTCCTCTATCTTCAAATTT  360
              |||||||||||| |||||||| ||||||||||||||||| || |||||||||||||||||
Sbjct  1491   ATTAATTGAATCCGGAGCTGGAACAGGATGAACAGTTTATCCCCCTCTATCTTCAAATTT  1550

Query  361    ATCACATTATGGCCCTTCAGTAGATTTAGCTATTTTTTCTCTTCATCTTGCTGGTGCTTC  420
              |||||||||||| || ||||||||||||||||||||||||||||| ||||||||||||||
Sbjct  1551   ATCACATTATGGTCCATCAGTAGATTTAGCTATTTTTTCTCTTCACCTTGCTGGTGCTTC  1610

Query  421    TTCAATTTTAGGTGCAATTAACTTTATTACAACTATTGTAAACATACGATCAATTGGAAT  480
```

```
                ||||||||||||||||||||||||| || |||||||||||||| |||||||||||||||||
Sbjct  1611  TTCAATTTTAGGTGCAATTAACTTCATCACAACTATTGTAAATATACGATCAATTGGAAT  1670

Query  481   AACAATAGAACGTATACCATTATTTGTTTGATCTGTTTTAATTACAGCTATTTTATTACT  540
             |||||||||||| |||||||||||||||||||||||||||||||||||||||||| ||||
Sbjct  1671  AACAATAGAACGAATACCATTATTTGTTTGATCTGTTTTAATTACAGCTATTTTACTACT  1730

Query  541   TTTATCTTTACCTGTATTAGCAGGTGCCATTACAATATTGTTAACTGATCGAAACTTTAA  600
             | ||||||||||||||||||||||||||||||||||||| ||||||||||||||||||||
Sbjct  1731  TCTATCTTTACCTGTATTAGCAGGTGCCATTACAATATTATTAACTGATCGAAACTTTAA  1790

Query  601   TACATCATTTTTTGATCCTTCAGGGGGAGGAGATCCAATTTTATATCAACATTTATTT  658
              ||||||||||||||||||||||| ||||||||||| |||||||||||||| ||||||
Sbjct  1791  CACATCATTTTTTGATCCTTCAGGAGGAGGAGATCCCATTTTATATCAACACTTATTT  1848
```

>Rhipicephalus sanguineus clone #1 cytochrome oxidase subunit I (COI) gene, partial cds; mitochondrial
Sequence ID: KX519706.1 Length: 595
Range 1: 1 to 570

Score:1053 bits(570), Expect:0.0,
Identities:570/570(100%), Gaps:0/570(0%), Strand: Plus/Plus

```
Query  89   ACTTTAATTGGAAATGATCAGATTTATAATGTAATCGTAACAGCACATGCATTTATTATA  148
            ||||||||||||||||||||||||||||||||||||||||||||||||||||||||||||
Sbjct  1    ACTTTAATTGGAAATGATCAGATTTATAATGTAATCGTAACAGCACATGCATTTATTATA  60

Query  149  AttttttttATAGTTATACCAATCATAATCGGAGGATTTGGAAATTGATTAGTCCCTATC  208
            ||||||||||||||||||||||||||||||||||||||||||||||||||||||||||||
Sbjct  61   ATTTTTTTTATAGTTATACCAATCATAATCGGAGGATTTGGAAATTGATTAGTCCCTATC  120

Query  209  ATATTGGGTGCCCCAGATATAGCATTTCCACGAATAAATAATATAAGATTTTGACTTTTA  268
            ||||||||||||||||||||||||||||||||||||||||||||||||||||||||||||
Sbjct  121  ATATTGGGTGCCCCAGATATAGCATTTCCACGAATAAATAATATAAGATTTTGACTTTTA  180

Query  269  CCTCCTTCACTATTTTTATTAATTAACTCTTCATTAATTGAATCTGGAGCTGGTACAGGA  328
            ||||||||||||||||||||||||||||||||||||||||||||||||||||||||||||
Sbjct  181  CCTCCTTCACTATTTTTATTAATTAACTCTTCATTAATTGAATCTGGAGCTGGTACAGGA  240

Query  329  TGAACAGTTTACCCTCCTCTATCTTCAAATTTATCACATTATGGCCCTTCAGTAGATTTA  388
            ||||||||||||||||||||||||||||||||||||||||||||||||||||||||||||
Sbjct  241  TGAACAGTTTACCCTCCTCTATCTTCAAATTTATCACATTATGGCCCTTCAGTAGATTTA  300

Query  389  GCTATTTTTTCTCTTCATCTTGCTGGTGCTTCTTCAATTTTAGGTGCAATTAACTTTATT  448
            ||||||||||||||||||||||||||||||||||||||||||||||||||||||||||||
Sbjct  301  GCTATTTTTTCTCTTCATCTTGCTGGTGCTTCTTCAATTTTAGGTGCAATTAACTTTATT  360

Query  449  ACAACTATTGTAAACATACGATCAATTGGAATAACAATAGAACGTATACCATTATTTGTT  508
            ||||||||||||||||||||||||||||||||||||||||||||||||||||||||||||
Sbjct  361  ACAACTATTGTAAACATACGATCAATTGGAATAACAATAGAACGTATACCATTATTTGTT  420
```

```
Query  509   TGATCTGTTTTAATTACAGCTATTTTATTACTTTTATCTTTACCTGTATTAGCAGGTGCC  568
             ||||||||||||||||||||||||||||||||||||||||||||||||||||||||||||
Sbjct  421   TGATCTGTTTTAATTACAGCTATTTTATTACTTTTATCTTTACCTGTATTAGCAGGTGCC  480

Query  569   ATTACAATATTGTTAACTGATCGAAACTTTAATACATCATTTTTTGATCCTTCAGGGGGA  628
             ||||||||||||||||||||||||||||||||||||||||||||||||||||||||||||
Sbjct  481   ATTACAATATTGTTAACTGATCGAAACTTTAATACATCATTTTTTGATCCTTCAGGGGGA  540

Query  629   GGAGATCCAATTTTATATCAACATTTATTT  658
             ||||||||||||||||||||||||||||||
Sbjct  541   GGAGATCCAATTTTATATCAACATTTATTT  570
```

>Rhipicephalus sanguineus sensu lato 'temperate lineage' isolate SC3005
cytochrome c oxidase subunit I (COI) gene, partial cds; mitochondrial
Sequence ID: MF425987.1 Length: 574
Range 1: 1 to 561

Score:1014 bits(549), Expect:0.0,
Identities:557/561(99%), Gaps:0/561(0%), Strand: Plus/Plus

```
Query  98    GGAAATGATCAGATTTATAATGTAATCGTAACAGCACATGCATTTATTATAAtttttttt  157
             |||||||||||||||||||||||||||||||||||||| |||||||||||||||||||||
Sbjct  1     GGAAATGATCAGATTTATAATGTAATCGTAACAGCACACGCATTTATTATAATTTTTTTT  60

Query  158   ATAGTTATACCAATCATAATCGGAGGATTTGGAAATTGATTAGTCCCTATCATATTGGGT  217
             |||||||||||||||||||||||||||||||||||||||||||||||||| ||||| |||
Sbjct  61    ATAGTTATACCAATCATAATCGGAGGATTTGGAAATTGATTAGTCCCTATTATATTAGGT  120

Query  218   GCCCCAGATATAGCATTTCCACGAATAAATAATATAAGATTTTGACTTTTACCTCCTTCA  277
             ||||||||||||||||||||||||||||||||||||||||||||||||||||||||||||
Sbjct  121   GCCCCAGATATAGCATTTCCACGAATAAATAATATAAGATTTTGACTTTTACCTCCTTCA  180

Query  278   CTATTTTTATTAATTAACTCTTCATTAATTGAATCTGGAGCTGGTACAGGATGAACAGTT  337
             ||||||||||||||||||||||||||||||||||||||||||||||||||||||||||||
Sbjct  181   CTATTTTTATTAATTAACTCTTCATTAATTGAATCTGGAGCTGGTACAGGATGAACAGTT  240

Query  338   TACCCTCCTCTATCTTCAAATTTATCACATTATGGCCCTTCAGTAGATTTAGCTATTTTT  397
             |||||||||||||||||||||||||||||||||||||||||||||||||||||||| |||
Sbjct  241   TACCCTCCTCTATCTTCAAATTTATCACATTATGGCCCTTCAGTAGATTTAGCTATCTTT  300

Query  398   TCTCTTCATCTTGCTGGTGCTTCTTCAATTTTAGGTGCAATTAACTTTATTACAACTATT  457
             ||||||||||||||||||||||||||||||||||||||||||||||||||||||||||||
Sbjct  301   TCTCTTCATCTTGCTGGTGCTTCTTCAATTTTAGGTGCAATTAACTTTATTACAACTATT  360

Query  458   GTAAACATACGATCAATTGGAATAACAATAGAACGTATACCATTATTTGTTTGATCTGTT  517
             ||||||||||||||||||||||||||||||||||||||||||||||||||||||||||||
Sbjct  361   GTAAACATACGATCAATTGGAATAACAATAGAACGTATACCATTATTTGTTTGATCTGTT  420

Query  518   TTAATTACAGCTATTTTATTACTTTTATCTTTACCTGTATTAGCAGGTGCCATTACAATA  577
             ||||||||||||||||||||||||||||||||||||||||||||||||||||||||||||
Sbjct  421   TTAATTACAGCTATTTTATTACTTTTATCTTTACCTGTATTAGCAGGTGCCATTACAATA  480
```

```
Query  578   TTGTTAACTGATCGAAACTTTAATACATCATTTTTTGATCCTTCAGGGGGAGGAGATCCA  637
             ||||||||||||||||||||||||||||||||||||||||||||||||||||||||||||
Sbjct  481   TTGTTAACTGATCGAAACTTTAATACATCATTTTTTGATCCTTCAGGGGGAGGAGATCCA  540

Query  638   ATTTTATATCAACATTTATTT  658
             |||||||||||||||||||||
Sbjct  541   ATTTTATATCAACATTTATTT  561
```

>Rhipicephalus sanguineus clone #5 cytochrome oxidase subunit I (COI) gene, partial cds; mitochondrial
Sequence ID: KX519710.1 Length: 553
Range 1: 1 to 547

Score:989 bits(535), Expect:0.0,
Identities:543/547(99%), Gaps:0/547(0%), Strand: Plus/Plus

```
Query  77    GGACAACCTGGAACTTTAATTGGAAATGATCAGATTTATAATGTAATCGTAACAGCACAT  136
             |||||||||||||||||||||||||||||||||||||||||||| |||||||||||||||
Sbjct  1     GGACAACCTGGAACTTTAATTGGAAATGATCAGATTTATAATGGAATCGTAACAGCACAT  60

Query  137   GCATTTATTATAAtttttttATAGTTATACCAATCATAATCGGAGGATTTGGAAATTGA  196
             || |||||||||||||||||||||||||||||||||||||||||||||||||||||||||
Sbjct  61    GCTTTTATTATAATTTTTTTATAGTTATACCAATCATAATCGGAGGATTTGGAAATTGA  120

Query  197   TTAGTCCCTATCATATTGGGTGCCCCAGATATAGCATTTCCACGAATAAATAATATAAGA  256
             ||||||||||||||||||||||||||||||||||||||||||||||||||||||||||||
Sbjct  121   TTAGTCCCTATCATATTGGGTGCCCCAGATATAGCATTTCCACGAATAAATAATATAAGA  180

Query  257   TTTTGACTTTTACCTCCTTCACTATTTTTATTAATTAACTCTTCATTAATTGAATCTGGA  316
             ||||||||||||||||||||||||||||||||||||||||||||||||||||||||||||
Sbjct  181   TTTTGACTTTTACCTCCTTCACTATTTTTATTAATTAACTCTTCATTAATTGAATCTGGA  240

Query  317   GCTGGTACAGGATGAACAGTTTACCCTCCTCTATCTTCAAATTTATCACATTATGGCCCT  376
             ||||||||||||||||||||||||||||||||||||||||||||||||||||||||||||
Sbjct  241   GCTGGTACAGGATGAACAGTTTACCCTCCTCTATCTTCAAATTTATCACATTATGGCCCT  300

Query  377   TCAGTAGATTTAGCTATTTTTTCTCTTCATCTTGCTGGTGCTTCTTCAATTTTAGGTGCA  436
             |||| |||||||||||||||||||||||||||||||||||||||||||||||||||||||
Sbjct  301   TCAGGAGATTTAGCTATTTTTTCTCTTCATCTTGCTGGTGCTTCTTCAATTTTAGGTGCA  360

Query  437   ATTAACTTTATTACAACTATTGTAAACATACGATCAATTGGAATAACAATAGAACGTATA  496
             ||||||||||||||||||||||||||||||||||||||||||||||||||||||||||||
Sbjct  361   ATTAACTTTATTACAACTATTGTAAACATACGATCAATTGGAATAACAATAGAACGTATA  420

Query  497   CCATTATTTGTTTGATCTGTTTTAATTACAGCTATTTTATTACTTTTATCTTTACCTGTA  556
             ||||||||||||||||||||||||||||||||||||||||||||||||||||||||||||
Sbjct  421   CCATTATTTGTTTGATCTGTTTTAATTACAGCTATTTTATTACTTTTATCTTTACCTGTA  480

Query  557   TTAGCAGGTGCCATTACAATATTGTTAACTGATCGAAACTTTAATACATCATTTTTTGAT  616
             |||||||||||||||||||||||||||||||||||| |||||||||||||||||||||||
```

```
Sbjct  481   TTAGCAGGTGCCATTACAATATTGTTAACTGATCGATACTTTAATACATCATTTTTTGAT  540

Query  617   CCTTCAG  623
             |||||||
Sbjct  541   CCTTCAG  547
```

>Rhipicephalus sanguineus isolate Italy-A cytochrome oxidase subunit I (COI) gene, partial cds; mitochondrial
Sequence ID: KX757903.1 Length: 630
Range 1: 1 to 620

Score:985 bits(533), Expect:0.0,
Identities:591/620(95%), Gaps:0/620(0%), Strand: Plus/Plus

```
Query  39    TAGGACTTAGTATAAGAATATTAATTCGTATAGAATTAGGACAACCTGGAACTTTAATTG  98
             ||||||||||||||||||||||||||||||||||||||||||||||||||||||||||||
Sbjct  1     TAGGACTTAGTATAAGAATATTAATTCGTATAGAATTAGGACAACCTGGAACTTTAATTG  60

Query  99    GAAATGATCAGATTTATAATGTAATCGTAACAGCACATGCATTTATTATAAttttttttA  158
             ||||||||||||||||||||||||||||||||||||||||||||||||||||||| ||||
Sbjct  61    GAAATGATCAGATTTATAATGTAATCGTAACAGCACATGCATTTATTATAATTTTCTTTA  120

Query  159   TAGTTATACCAATCATAATCGGAGGATTTGGAAATTGATTAGTCCCTATCATATTGGGTG  218
             ||||||||||||||||||||||||| || |||||||||||||| ||||| ||||| ||||
Sbjct  121   TAGTTATACCAATCATAATCGGAGGGTTCGGAAATTGATTAGTTCCTATTATATTAGGTG  180

Query  219   CCCCAGATATAGCATTTCCACGAATAAATAATATAAGATTTTGACTTTTACCTCCTTCAC  278
             |||||||||||||||||||||||||||||||||||||||||||||||||||| || |||
Sbjct  181   CCCCAGATATAGCATTTCCACGAATAAATAATATAAGATTTTGACTTTTACCCCCCTCAT  240

Query  279   TATTTTTATTAATTAACTCTTCATTAATTGAATCTGGAGCTGGTACAGGATGAACAGTTT  338
             |||||||||| ||||| ||||||||||||||||| |||||||| ||||||||||||||||
Sbjct  241   TATTTTTATTGATTAATTCTTCATTAATTGAATCCGGAGCTGGAACAGGATGAACAGTTT  300

Query  339   ACCCTCCTCTATCTTCAAATTTATCACATTATGGCCCTTCAGTAGATTTAGCTATTTTTT  398
             | || ||||||||||||||||||||||||||||| || ||||||||||||||||||||||
Sbjct  301   ATCCCCCTCTATCTTCAAATTTATCACATTATGGTCCATCAGTAGATTTAGCTATTTTTT  360

Query  399   CTCTTCATCTTGCTGGTGCTTCTTCAATTTAGGTGCAATTAACTTTATTACAACTATTG  458
             ||||||| ||||||||||||||||||||||||||||||||||||| ||||||||||||||
Sbjct  361   CTCTTCACCTTGCTGGTGCTTCTTCAATTTAGGTGCAATTAACTTCATTACAACTATTG  420

Query  459   TAAACATACGATCAATTGGAATAACAATAGAACGTATACCATTATTTGTTTGATCTGTTT  518
             |||| ||||||||||||||||||||||||||||||| |||||||||||||||||||||||
Sbjct  421   TAAATATACGATCAATTGGAATAACAATAGAACGAATACCATTATTTGTTTGATCTGTTT  480

Query  519   TAATTACAGCTATTTTATTACTTTTATCTTTACCTGTATTAGCAGGTGCCATTACAATAT  578
             ||||||||||||||||| ||||| ||||||||||||||||||||||||||||||||||||
Sbjct  481   TAATTACAGCTATTTTACTACTTCTATCTTTACCTGTATTAGCAGGTGCCATTACAATAT  540

Query  579   TGTTAACTGATCGAAACTTTAATACATCATTTTTTGATCCTTCAGGGGGAGGAGATCCAA  638
```

```
          | |||||||||||||||||||| |||||||||||||| |||||||| ||||||||||| |
Sbjct  541   TATTAACTGATCGAAACTTTAACACATCATTTTTTGACCCTTCAGGAGGAGGAGATCCCA  600

Query  639   TTTTATATCAACATTTATTT  658
             ||||||||||||| ||||||
Sbjct  601   TTTTATATCAACACTTATTT  620
```

>Rhipicephalus sanguineus isolate Zagreb-B cytochrome oxidase subunit I (COI) gene, partial cds; mitochondrial
Sequence ID: KX757893.1 Length: 629
Range 1: 1 to 620

Score:985 bits(533), Expect:0.0,
Identities:591/620(95%), Gaps:0/620(0%), Strand: Plus/Plus

```
Query  39    TAGGACTTAGTATAAGAATATTAATTCGTATAGAATTAGGACAACCTGGAACTTTAATTG  98
             ||||||||||||||||||||||||||||||||||||||||||||||||||||||||||||
Sbjct  1     TAGGACTTAGTATAAGAATATTAATTCGTATAGAATTAGGACAACCTGGAACTTTAATTG  60

Query  99    GAAATGATCAGATTTATAATGTAATCGTAACAGCACATGCATTTATTATAAttttttttA  158
             ||||||||||||||||||||||||||||||||||||||||||||||||||||||| ||||
Sbjct  61    GAAATGATCAGATTTATAATGTAATCGTAACAGCACATGCATTTATTATAATTTTCTTTA  120

Query  159   TAGTTATACCAATCATAATCGGAGGATTTGGAAATTGATTAGTCCCTATCATATTGGGTG  218
             ||||||||||||||||||||||||| || |||||||||||||| ||||| ||||| ||||
Sbjct  121   TAGTTATACCAATCATAATCGGAGGGTTCGGAAATTGATTAGTTCCTATTATATTAGGTG  180

Query  219   CCCCAGATATAGCATTTCCACGAATAAATAATATAAGATTTTGACTTTTACCTCCTTCAC  278
             |||||||||||||||||||||||||||||||||||||||||||||||||||| || ||| 
Sbjct  181   CCCCAGATATAGCATTTCCACGAATAAATAATATAAGATTTTGACTTTTACCCCCCTCAT  240

Query  279   TATTTTTATTAATTAACTCTTCATTAATTGAATCTGGAGCTGGTACAGGATGAACAGTTT  338
             |||||||||| ||||||||||||||||||||||| |||||||| ||||||||||||||||
Sbjct  241   TATTTTTATTGATTAACTCTTCATTAATTGAATCCGGAGCTGGAACAGGATGAACAGTTT  300

Query  339   ACCCTCCTCTATCTTCAAATTTATCACATTATGGCCCTTCAGTAGATTTAGCTATTTTTT  398
             | || ||||||||||||||||||||||||||||| || ||||||||||||||||||||||
Sbjct  301   ATCCCCCTCTATCTTCAAATTTATCACATTATGGTCCATCAGTAGATTTAGCTATTTTTT  360

Query  399   CTCTTCATCTTGCTGGTGCTTCTTCAATTTTAGGTGCAATTAACTTTATTACAACTATTG  458
             ||||||| |||||||||||||||||||||||||||||||||||||| || ||||||||||
Sbjct  361   CTCTTCACCTTGCTGGTGCTTCTTCAATTTTAGGTGCAATTAACTTCATCACAACTATTG  420

Query  459   TAAACATACGATCAATTGGAATAACAATAGAACGTATACCATTATTTGTTTGATCTGTTT  518
             |||| ||||||||||||||||||||||||||||| |||||||||||||||||||||||||
Sbjct  421   TAAATATACGATCAATTGGAATAACAATAGAACGAATACCATTATTTGTTTGATCTGTTT  480

Query  519   TAATTACAGCTATTTTATTACTTTTATCTTTACCTGTATTAGCAGGTGCCATTACAATAT  578
             ||||||||||||||||| ||||| ||||||||||||||||||||||||||||||||||||
Sbjct  481   TAATTACAGCTATTTTACTACTTCTATCTTTACCTGTATTAGCAGGTGCCATTACAATAT  540
```

```
Query  579   TGTTAACTGATCGAAACTTTAATACATCATTTTTTGATCCTTCAGGGGGAGGAGATCCAA  638
             | |||||||||||||||||||| |||||||||||||| |||||||| ||||||||||| |
Sbjct  541   TATTAACTGATCGAAACTTTAACACATCATTTTTTGACCCTTCAGGAGGAGGAGATCCCA  600

Query  639   TTTTATATCAACATTTATTT  658
             ||||||||||||| ||||||
Sbjct  601   TTTTATATCAACACTTATTT  620
```

>Rhipicephalus sanguineus isolate Al-Hosema-A cytochrome oxidase subunit I (COI) gene, partial cds; mitochondrial
Sequence ID: KX757909.1 Length: 630
Range 1: 1 to 620

Score:979 bits(530), Expect:0.0,
Identities:590/620(95%), Gaps:0/620(0%), Strand: Plus/Plus

```
Query  39    TAGGACTTAGTATAAGAATATTAATTCGTATAGAATTAGGACAACCTGGAACTTTAATTG  98
             ||||||||||||||||||||||||||||||||||||||||||||||||||||||||||||
Sbjct  1     TAGGACTTAGTATAAGAATATTAATTCGTATAGAATTAGGACAACCTGGAACTTTAATTG  60

Query  99    GAAATGATCAGATTTATAATGTAATCGTAACAGCACATGCATTTATTATAAtttttttA  158
             ||||||||||||||||||||||||||||||||||||||||||||||||||||||| ||||
Sbjct  61    GAAATGATCAGATTTATAATGTAATCGTAACAGCACATGCATTTATTATAATTTTCTTA  120

Query  159   TAGTTATACCAATCATAATCGGAGGATTTGGAAATTGATTAGTCCCTATCATATTGGGTG  218
             ||||||||||||| ||||||||||| || |||||||||||||| ||||| ||||| ||||
Sbjct  121   TAGTTATACCAATAATAATCGGAGGGTTCGGAAATTGATTAGTTCCTATTATATTAGGTG  180

Query  219   CCCCAGATATAGCATTTCCACGAATAAATAATATAAGATTTTGACTTTTACCTCCTTCAC  278
             |||||||||||||||||||||||||||||||||||||||||||||||||||| || |||
Sbjct  181   CCCCAGATATAGCATTTCCACGAATAAATAATATAAGATTTTGACTTTTACCCCCCTCAT  240

Query  279   TATTTTTATTAATTAACTCTTCATTAATTGAATCTGGAGCTGGTACAGGATGAACAGTTT  338
             |||||||||| |||||||||||||||||||||||||||||||| ||||||||||||||||
Sbjct  241   TATTTTTATTGATTAACTCTTCATTAATTGAATCTGGAGCTGGAACAGGATGAACAGTTT  300

Query  339   ACCCTCCTCTATCTTCAAATTTATCACATTATGGCCCTTCAGTAGATTTAGCTATTTTTT  398
             | || |||||||||||||||||||||||||||||||| ||||||||||||||||||||||
Sbjct  301   ATCCCCCTCTATCTTCAAATTTATCACATTATGGCCCATCAGTAGATTTAGCTATTTTTT  360

Query  399   CTCTTCATCTTGCTGGTGCTTCTTCAATTTTAGGTGCAATTAACTTTATTACAACTATTG  458
             ||||||| |||||||||||||||||||||||||||||||||||||| || ||||||||||
Sbjct  361   CTCTTCACCTTGCTGGTGCTTCTTCAATTTTAGGTGCAATTAACTTCATCACAACTATTG  420

Query  459   TAAACATACGATCAATTGGAATAACAATAGAACGTATACCATTATTTGTTTGATCTGTTT  518
             |||| ||||||||||||||||||||||||||||| |||||||||||||||||||||||||
Sbjct  421   TAAATATACGATCAATTGGAATAACAATAGAACGAATACCATTATTTGTTTGATCTGTTT  480

Query  519   TAATTACAGCTATTTTATTACTTTTATCTTTACCTGTATTAGCAGGTGCCATTACAATAT  578
             ||||||||||||||||| ||||| ||||||||||||||||||||||||||||||||||||
Sbjct  481   TAATTACAGCTATTTTACTACTTCTATCTTTACCTGTATTAGCAGGTGCCATTACAATAT  540
```

```
Query  579  TGTTAACTGATCGAAACTTTAATACATCATTTTTTGATCCTTCAGGGGGAGGAGATCCAA  638
            | || ||||||||||||||||| |||||||||||||| |||||||| ||||| ||||| |
Sbjct  541  TATTGACTGATCGAAACTTTAACACATCATTTTTTGACCCTTCAGGAGGAGGGGATCCCA  600

Query  639  TTTTATATCAACATTTATTT  658
            ||||||||||||| ||||||
Sbjct  601  TTTTATATCAACACTTATTT  620
```

```
>Rhipicephalus sanguineus isolate FT157 cytochrome c oxidase subunit I (COI)
gene, partial cds; mitochondrial
Sequence ID: MN223690.1 Length: 535
Range 1: 1 to 524

Score:963 bits(521), Expect:0.0,
Identities:523/524(99%),  Gaps:0/524(0%), Strand: Plus/Plus

Query  135  ATGCATTTATTATAAttttttttATAGTTATACCAATCATAATCGGAGGATTTGGAAATT  194
            ||||||||||||||||||||||||||||||||||||||||||||||||||||||||||||
Sbjct  1    ATGCATTTATTATAATTTTTTTTATAGTTATACCAATCATAATCGGAGGATTTGGAAATT  60

Query  195  GATTAGTCCCTATCATATTGGGTGCCCCAGATATAGCATTTCCACGAATAAATAATATAA  254
            ||||||||||||||||||||||||||||||||||||||||||||||||||||||||||||
Sbjct  61   GATTAGTCCCTATCATATTGGGTGCCCCAGATATAGCATTTCCACGAATAAATAATATAA  120

Query  255  GATTTTGACTTTTACCTCCTTCACTATTTTTATTAATTAACTCTTCATTAATTGAATCTG  314
            ||||||||||||||||||||||||||||||||||||||||||||||||||||||||||||
Sbjct  121  GATTTTGACTTTTACCTCCTTCACTATTTTTATTAATTAACTCTTCATTAATTGAATCTG  180

Query  315  GAGCTGGTACAGGATGAACAGTTTACCCTCCTCTATCTTCAAATTTATCACATTATGGCC  374
            |||||||||||||||||||||||||||||||| |||||||||||||||||||||||||||
Sbjct  181  GAGCTGGTACAGGATGAACAGTTTACCCTCCTTTATCTTCAAATTTATCACATTATGGCC  240

Query  375  CTTCAGTAGATTTAGCTATTTTTTCTCTTCATCTTGCTGGTGCTTCTTCAATTTTAGGTG  434
            ||||||||||||||||||||||||||||||||||||||||||||||||||||||||||||
Sbjct  241  CTTCAGTAGATTTAGCTATTTTTTCTCTTCATCTTGCTGGTGCTTCTTCAATTTTAGGTG  300

Query  435  CAATTAACTTTATTACAACTATTGTAAACATACGATCAATTGGAATAACAATAGAACGTA  494
            ||||||||||||||||||||||||||||||||||||||||||||||||||||||||||||
Sbjct  301  CAATTAACTTTATTACAACTATTGTAAACATACGATCAATTGGAATAACAATAGAACGTA  360

Query  495  TACCATTATTTGTTTGATCTGTTTTAATTACAGCTATTTTATTACTTTTATCTTTACCTG  554
            ||||||||||||||||||||||||||||||||||||||||||||||||||||||||||||
Sbjct  361  TACCATTATTTGTTTGATCTGTTTTAATTACAGCTATTTTATTACTTTTATCTTTACCTG  420

Query  555  TATTAGCAGGTGCCATTACAATATTGTTAACTGATCGAAACTTTAATACATCATTTTTTG  614
            ||||||||||||||||||||||||||||||||||||||||||||||||||||||||||||
Sbjct  421  TATTAGCAGGTGCCATTACAATATTGTTAACTGATCGAAACTTTAATACATCATTTTTTG  480

Query  615  ATCCTTCAGGGGGAGGAGATCCAATTTTATATCAACATTTATTT  658
            ||||||||||||||||||||||||||||||||||||||||||||
```

```
Sbjct  481  ATCCTTCAGGGGGAGGAGATCCAATTTTATATCAACATTTATTT  524
```

>Rhipicephalus sanguineus isolate FT154 cytochrome c oxidase subunit I (COI) gene, partial cds; mitochondrial
Sequence ID: MN223688.1 Length: 535
Range 1: 1 to 524

Score:957 bits(518), Expect:0.0,
Identities:522/524(99%), Gaps:0/524(0%), Strand: Plus/Plus

```
Query  135  ATGCATTTATTATAAtttttttATAGTTATACCAATCATAATCGGAGGATTTGGAAATT  194
            ||||||||||||||||||||||||||||||||||||||||||||||||||||||||||||
Sbjct  1    ATGCATTTATTATAATTTTTTTATAGTTATACCAATCATAATCGGAGGATTTGGAAATT  60

Query  195  GATTAGTCCCTATCATATTGGGTGCCCCAGATATAGCATTTCCACGAATAAATAATATAA  254
            ||||||||||||||||||||||||||||||||||||||||||||||||||||||||||||
Sbjct  61   GATTAGTCCCTATCATATTGGGTGCCCCAGATATAGCATTTCCACGAATAAATAATATAA  120

Query  255  GATTTTGACTTTTACCTCCTTCACTATTTTTATTAATTAACTCTTCATTAATTGAATCTG  314
            ||||||||||||||||||||||||||||||||||||||||||||||| ||||||||||||
Sbjct  121  GATTTTGACTTTTACCTCCTTCACTATTTTTATTAATTAACTCTTCACTAATTGAATCTG  180

Query  315  GAGCTGGTACAGGATGAACAGTTTACCCTCCTCTATCTTCAAATTTATCACATTATGGCC  374
            ||||||||||||||||||||||||||||||||||||||||||| ||||||||||||||||
Sbjct  181  GAGCTGGTACAGGATGAACAGTTTACCCTCCTCTATCTTCAAACTTATCACATTATGGCC  240

Query  375  CTTCAGTAGATTTAGCTATTTTTTCTCTTCATCTTGCTGGTGCTTCTTCAATTTTAGGTG  434
            ||||||||||||||||||||||||||||||||||||||||||||||||||||||||||||
Sbjct  241  CTTCAGTAGATTTAGCTATTTTTTCTCTTCATCTTGCTGGTGCTTCTTCAATTTTAGGTG  300

Query  435  CAATTAACTTTATTACAACTATTGTAAACATACGATCAATTGGAATAACAATAGAACGTA  494
            ||||||||||||||||||||||||||||||||||||||||||||||||||||||||||||
Sbjct  301  CAATTAACTTTATTACAACTATTGTAAACATACGATCAATTGGAATAACAATAGAACGTA  360

Query  495  TACCATTATTTGTTTGATCTGTTTTAATTACAGCTATTTTATTACTTTTATCTTTACCTG  554
            ||||||||||||||||||||||||||||||||||||||||||||||||||||||||||||
Sbjct  361  TACCATTATTTGTTTGATCTGTTTTAATTACAGCTATTTTATTACTTTTATCTTTACCTG  420

Query  555  TATTAGCAGGTGCCATTACAATATTGTTAACTGATCGAAACTTTAATACATCATTTTTTG  614
            ||||||||||||||||||||||||||||||||||||||||||||||||||||||||||||
Sbjct  421  TATTAGCAGGTGCCATTACAATATTGTTAACTGATCGAAACTTTAATACATCATTTTTTG  480

Query  615  ATCCTTCAGGGGGAGGAGATCCAATTTTATATCAACATTTATTT  658
            ||||||||||||||||||||||||||||||||||||||||||||
Sbjct  481  ATCCTTCAGGGGGAGGAGATCCAATTTTATATCAACATTTATTT  524
```

>Rhipicephalus sanguineus isolate CE67 cytochrome c oxidase subunit I (COI) gene, partial cds; mitochondrial

```
Sequence ID: MN223686.1 Length: 535
Range 1: 1 to 524

Score:957 bits(518), Expect:0.0,
Identities:522/524(99%),  Gaps:0/524(0%), Strand: Plus/Plus

Query  135   ATGCATTTATTATAAtttttttATAGTTATACCAATCATAATCGGAGGATTTGGAAATT  194
             |||||||||||||||||||||||||||||||||||||||||||||||||||||||||||
Sbjct  1     ATGCATTTATTATAATTTTTTTATAGTTATACCAATCATAATCGGAGGATTTGGAAATT  60

Query  195   GATTAGTCCCTATCATATTGGGTGCCCCAGATATAGCATTTCCACGAATAAATAATATAA  254
             ||||||||||||||||||||||||||||||||||||||||||||||||||||||||||||
Sbjct  61    GATTAGTCCCTATCATATTGGGTGCCCCAGATATAGCATTTCCACGAATAAATAATATAA  120

Query  255   GATTTTGACTTTTACCTCCTTCACTATTTTTATTAATTAACTCTTCATTAATTGAATCTG  314
             ||||||||||||||||||||||||||||||||||||||||||||||||||||||||||||
Sbjct  121   GATTTTGACTTTTACCTCCTTCACTATTTTTATTAATTAACTCTTCATTAATTGAATCTG  180

Query  315   GAGCTGGTACAGGATGAACAGTTTACCCTCCTCTATCTTCAAATTTATCACATTATGGCC  374
             ||||||||||||||||||||||||||||||||||||||||||||||||||||||||||||
Sbjct  181   GAGCTGGTACAGGATGAACAGTTTACCCTCCTCTATCTTCAAATTTATCACATTATGGCC  240

Query  375   CTTCAGTAGATTTAGCTATTTTTTCTCTTCATCTTGCTGGTGCTTCTTCAATTTTAGGTG  434
             ||||||||||||||||||| ||||||||||||||||||||||||||||||||||||||||
Sbjct  241   CTTCAGTAGATTTAGCTATCTTTTCTCTTCATCTTGCTGGTGCTTCTTCAATTTTAGGTG  300

Query  435   CAATTAACTTTATTACAACTATTGTAAACATACGATCAATTGGAATAACAATAGAACGTA  494
             ||||||||||||||||||||||||||||||||||||||||||||||||||||||||||| 
Sbjct  301   CAATTAACTTTATTACAACTATTGTAAACATACGATCAATTGGAATAACAATAGAACGTG  360

Query  495   TACCATTATTTGTTTGATCTGTTTTAATTACAGCTATTTTATTACTTTTATCTTTACCTG  554
             ||||||||||||||||||||||||||||||||||||||||||||||||||||||||||||
Sbjct  361   TACCATTATTTGTTTGATCTGTTTTAATTACAGCTATTTTATTACTTTTATCTTTACCTG  420

Query  555   TATTAGCAGGTGCCATTACAATATTGTTAACTGATCGAAACTTTAATACATCATTTTTTG  614
             ||||||||||||||||||||||||||||||||||||||||||||||||||||||||||||
Sbjct  421   TATTAGCAGGTGCCATTACAATATTGTTAACTGATCGAAACTTTAATACATCATTTTTTG  480

Query  615   ATCCTTCAGGGGGAGGAGATCCAATTTTATATCAACATTTATTT  658
             ||||||||||||||||||||||||||||||||||||||||||||
Sbjct  481   ATCCTTCAGGGGGAGGAGATCCAATTTTATATCAACATTTATTT  524
```

```
>Rhipicephalus sanguineus isolate CE61 cytochrome c oxidase subunit I (COI)
gene, partial cds; mitochondrial
Sequence ID: MN223689.1 Length: 535
Range 1: 1 to 524

Score:952 bits(515), Expect:0.0,
Identities:521/524(99%),  Gaps:0/524(0%), Strand: Plus/Plus

Query  135   ATGCATTTATTATAAtttttttATAGTTATACCAATCATAATCGGAGGATTTGGAAATT  194
```

```
                |||||||||||||||||||||||||||||||||||||||||||||||||||||||||||||
Sbjct  1        ATGCATTTATTATAATTTTTTTTATAGTTATACCAATCATAATCGGAGGATTTGGAAATT  60

Query  195  GATTAGTCCCTATCATATTGGGTGCCCCAGATATAGCATTTCCACGAATAAATAATATAA  254
            ||||||||||||||||||||||||||||||||||||||||||||||||||||||||||||
Sbjct  61   GATTAGTCCCTATCATATTGGGTGCCCCAGATATAGCATTTCCACGAATAAATAATATAA  120

Query  255  GATTTTGACTTTTACCTCCTTCACTATTTTTATTAATTAACTCTTCATTAATTGAATCTG  314
            ||||||||||||||||||||||||||||||||||||||||||||||||||||||||||||
Sbjct  121  GATTTTGACTTTTACCTCCTTCACTATTTTTATTAATTAACTCTTCATTAATTGAATCTG  180

Query  315  GAGCTGGTACAGGATGAACAGTTTACCCTCCTCTATCTTCAAATTTATCACATTATGGCC  374
            |||||||||||||||||||||||||||||||||||||||||||||||||||||||||| |
Sbjct  181  GAGCTGGTACAGGATGAACAGTTTACCCTCCTCTATCTTCAAATTTATCACATTATGGTC  240

Query  375  CTTCAGTAGATTTAGCTATTTTTTCTCTTCATCTTGCTGGTGCTTCTTCAATTTTAGGTG  434
            |||||||||||||| |||||||||||||||||||||||||||||||||||||||||||||
Sbjct  241  CTTCAGTAGATTTAACTATTTTTTCTCTTCATCTTGCTGGTGCTTCTTCAATTTTAGGTG  300

Query  435  CAATTAACTTTATTACAACTATTGTAAACATACGATCAATTGGAATAACAATAGAACGTA  494
            ||||||||||||||||||||||||||||||||||||||||||||||||||||||||||||
Sbjct  301  CAATTAACTTTATTACAACTATTGTAAACATACGATCAATTGGAATAACAATAGAACGTA  360

Query  495  TACCATTATTTGTTTGATCTGTTTTAATTACAGCTATTTTATTACTTTTATCTTTACCTG  554
            ||||||||||||||||||||||||||||||||||||||||| ||||||||||||||||||
Sbjct  361  TACCATTATTTGTTTGATCTGTTTTAATTACAGCTATTTTACTACTTTTATCTTTACCTG  420

Query  555  TATTAGCAGGTGCCATTACAATATTGTTAACTGATCGAAACTTTAATACATCATTTTTTG  614
            ||||||||||||||||||||||||||||||||||||||||||||||||||||||||||||
Sbjct  421  TATTAGCAGGTGCCATTACAATATTGTTAACTGATCGAAACTTTAATACATCATTTTTTG  480

Query  615  ATCCTTCAGGGGGAGGAGATCCAATTTTATATCAACATTTATTT  658
            ||||||||||||||||||||||||||||||||||||||||||||
Sbjct  481  ATCCTTCAGGGGGAGGAGATCCAATTTTATATCAACATTTATTT  524
```

>Rhipicephalus sanguineus isolate RI46 cytochrome c oxidase subunit I (COI) gene, partial cds; mitochondrial
Sequence ID: MN223687.1 Length: 535
Range 1: 1 to 524

Score:952 bits(515), Expect:0.0,
Identities:521/524(99%), Gaps:0/524(0%), Strand: Plus/Plus

```
Query  135  ATGCATTTATTATAAttttttttATAGTTATACCAATCATAATCGGAGGATTTGGAAATT  194
            ||||||||||||||||||||||||||||||||||||||||||||||||||||||||||||
Sbjct  1    ATGCATTTATTATAATTTTTTTTATAGTTATACCAATCATAATCGGAGGATTTGGAAATT  60

Query  195  GATTAGTCCCTATCATATTGGGTGCCCCAGATATAGCATTTCCACGAATAAATAATATAA  254
            ||||||||||||| ||||||||||||||||||||||||||||||||||||||||||||||
Sbjct  61   GATTAGTCCCTATTATATTGGGTGCCCCAGATATAGCATTTCCACGAATAAATAATATAA  120
```

```
Query  255  GATTTTGACTTTTACCTCCTTCACTATTTTTATTAATTAACTCTTCATTAATTGAATCTG  314
            ||||||||||||||||||||||||||||||||||||||||||||||||||||||||||||
Sbjct  121  GATTTTGACTTTTACCTCCTTCACTATTTTTATTAATTAACTCTTCATTAATTGAATCTG  180

Query  315  GAGCTGGTACAGGATGAACAGTTTACCCTCCTCTATCTTCAAATTTATCACATTATGGCC  374
            |||||||||||||||||||||||||||||||||||||||||||||||||||| |||||||
Sbjct  181  GAGCTGGTACAGGATGAACAGTTTACCCTCCTCTATCTTCAAATTTATCACACTATGGCC  240

Query  375  CTTCAGTAGATTTAGCTATTTTTTCTCTTCATCTTGCTGGTGCTTCTTCAATTTTAGGTG  434
            ||||||||||||||||||||||||||||||||||||||||||||||||||||||||||||
Sbjct  241  CTTCAGTAGATTTAGCTATTTTTTCTCTTCATCTTGCTGGTGCTTCTTCAATTTTAGGTG  300

Query  435  CAATTAACTTTATTACAACTATTGTAAACATACGATCAATTGGAATAACAATAGAACGTA  494
            ||||||||||||||||||||||||||||||||||||||||||||||||||||||||||||
Sbjct  301  CAATTAACTTTATTACAACTATTGTAAACATACGATCAATTGGAATAACAATAGAACGTA  360

Query  495  TACCATTATTTGTTTGATCTGTTTTAATTACAGCTATTTTATTACTTTTATCTTTACCTG  554
            ||||| ||||||||||||||||||||||||||||||||||||||||||||||||||||||
Sbjct  361  TACCACTATTTGTTTGATCTGTTTTAATTACAGCTATTTTATTACTTTTATCTTTACCTG  420

Query  555  TATTAGCAGGTGCCATTACAATATTGTTAACTGATCGAAACTTTAATACATCATTTTTTG  614
            ||||||||||||||||||||||||||||||||||||||||||||||||||||||||||||
Sbjct  421  TATTAGCAGGTGCCATTACAATATTGTTAACTGATCGAAACTTTAATACATCATTTTTTG  480

Query  615  ATCCTTCAGGGGGAGGAGATCCAATTTTATATCAACATTTATTT  658
            ||||||||||||||||||||||||||||||||||||||||||||
Sbjct  481  ATCCTTCAGGGGGAGGAGATCCAATTTTATATCAACATTTATTT  524
```

>Rhipicephalus sanguineus isolate RI08 cytochrome c oxidase subunit I (COI) gene, partial cds; mitochondrial
Sequence ID: MN223684.1 Length: 535
Range 1: 3 to 524

Score:948 bits(513), Expect:0.0,
Identities:519/522(99%), Gaps:0/522(0%), Strand: Plus/Plus

```
Query  137  GCATTTATTATAAttttttttATAGTTATACCAATCATAATCGGAGGATTTGGAAATTGA  196
            ||||||||||||||||||||||||||||||||||||||||||||||||||||||||||||
Sbjct  3    GCATTTATTATAATTTTTTTTATAGTTATACCAATCATAATCGGAGGATTTGGAAATTGA  62

Query  197  TTAGTCCCTATCATATTGGGTGCCCCAGATATAGCATTTCCACGAATAAATAATATAAGA  256
            ||||||||||| ||||| ||||||||||||||||||||||||||||||||||||||||||
Sbjct  63   TTAGTCCCTATTATATTAGGTGCCCCAGATATAGCATTTCCACGAATAAATAATATAAGA  122

Query  257  TTTTGACTTTTACCTCCTTCACTATTTTTATTAATTAACTCTTCATTAATTGAATCTGGA  316
            ||||||||||||||||||||||||||||||||||||||||||||||||||||||||||||
Sbjct  123  TTTTGACTTTTACCTCCTTCACTATTTTTATTAATTAACTCTTCATTAATTGAATCTGGA  182

Query  317  GCTGGTACAGGATGAACAGTTTACCCTCCTCTATCTTCAAATTTATCACATTATGGCCCT  376
            ||||||||||||||||||||||||||||||||||||||||||||||||||||||||||||
Sbjct  183  GCTGGTACAGGATGAACAGTTTACCCTCCTCTATCTTCAAATTTATCACATTATGGCCCT  242
```

```
Query  377   TCAGTAGATTTAGCTATTTTTTCTCTTCATCTTGCTGGTGCTTCTTCAATTTTAGGTGCA  436
             ||||||||||||||||| ||||||||||||||||||||||||||||||||||||||||||
Sbjct  243   TCAGTAGATTTAGCTATCTTTTCTCTTCATCTTGCTGGTGCTTCTTCAATTTTAGGTGCA  302

Query  437   ATTAACTTTATTACAACTATTGTAAACATACGATCAATTGGAATAACAATAGAACGTATA  496
             ||||||||||||||||||||||||||||||||||||||||||||||||||||||||||||
Sbjct  303   ATTAACTTTATTACAACTATTGTAAACATACGATCAATTGGAATAACAATAGAACGTATA  362

Query  497   CCATTATTTGTTTGATCTGTTTTAATTACAGCTATTTTATTACTTTTATCTTTACCTGTA  556
             ||||||||||||||||||||||||||||||||||||||||||||||||||||||||||||
Sbjct  363   CCATTATTTGTTTGATCTGTTTTAATTACAGCTATTTTATTACTTTTATCTTTACCTGTA  422

Query  557   TTAGCAGGTGCCATTACAATATTGTTAACTGATCGAAACTTTAATACATCATTTTTTGAT  616
             ||||||||||||||||||||||||||||||||||||||||||||||||||||||||||||
Sbjct  423   TTAGCAGGTGCCATTACAATATTGTTAACTGATCGAAACTTTAATACATCATTTTTTGAT  482

Query  617   CCTTCAGGGGGAGGAGATCCAATTTTATATCAACATTTATTT  658
             ||||||||||||||||||||||||||||||||||||||||||
Sbjct  483   CCTTCAGGGGGAGGAGATCCAATTTTATATCAACATTTATTT  524
```

>Rhipicephalus sanguineus isolate RI54 cytochrome c oxidase subunit I (COI) gene, partial cds; mitochondrial
Sequence ID: MN223685.1 Length: 535
Range 1: 1 to 524

Score:946 bits(512), Expect:0.0,
Identities:520/524(99%), Gaps:0/524(0%), Strand: Plus/Plus

```
Query  135   ATGCATTTATTATAAttttttttATAGTTATACCAATCATAATCGGAGGATTTGGAAATT  194
             |||||||||||||||||||||||||||||||||| |||||||||||||||||||||||||
Sbjct  1     ATGCATTTATTATAATTTTTTTTATAGTTATACCGATCATAATCGGAGGATTTGGAAATT  60

Query  195   GATTAGTCCCTATCATATTGGGTGCCCCAGATATAGCATTTCCACGAATAAATAATATAA  254
             ||||||||||||||||||| ||||||||||||||||||||||||||||||||||||||||
Sbjct  61    GATTAGTCCCTATCATATTAGGTGCCCCAGATATAGCATTTCCACGAATAAATAATATAA  120

Query  255   GATTTTGACTTTTACCTCCTTCACTATTTTTATTAATTAACTCTTCATTAATTGAATCTG  314
             ||||||||||||||||||||||||||||||||||||||||||||||||||||||||||||
Sbjct  121   GATTTTGACTTTTACCTCCTTCACTATTTTTATTAATTAACTCTTCATTAATTGAATCTG  180

Query  315   GAGCTGGTACAGGATGAACAGTTTACCCTCCTCTATCTTCAAATTTATCACATTATGGCC  374
             ||||||||||||||||||||||||||||||||||||||||||||||||||||||||||||
Sbjct  181   GAGCTGGTACAGGATGAACAGTTTACCCTCCTCTATCTTCAAATTTATCACATTATGGCC  240

Query  375   CTTCAGTAGATTTAGCTATTTTTTCTCTTCATCTTGCTGGTGCTTCTTCAATTTTAGGTG  434
             |||||||||||||||||| |||||||||||||||||||||||||||||||||||||||||
Sbjct  241   CTTCAGTAGATTTAGCTATCTTTTCTCTTCATCTTGCTGGTGCTTCTTCAATTTTAGGTG  300

Query  435   CAATTAACTTTATTACAACTATTGTAAACATACGATCAATTGGAATAACAATAGAACGTA  494
             ||||||||||||||||||||||||||||||||||||||||||||||||||||||||||||
```

```
Sbjct  301   CAATTAACTTTATTACAACTATTGTAAACATACGATCAATTGGAATAACAATAGAACGTA  360

Query  495   TACCATTATTTGTTTGATCTGTTTTAATTACAGCTATTTTATTACTTTTATCTTTACCTG  554
             |||||||||||||||||||||||||||||||||||||||||||||| |||||||||||||
Sbjct  361   TACCATTATTTGTTTGATCTGTTTTAATTACAGCTATTTTATTACTCTTATCTTTACCTG  420

Query  555   TATTAGCAGGTGCCATTACAATATTGTTAACTGATCGAAACTTTAATACATCATTTTTTG  614
             ||||||||||||||||||||||||||||||||||||||||||||||||||||||||||||
Sbjct  421   TATTAGCAGGTGCCATTACAATATTGTTAACTGATCGAAACTTTAATACATCATTTTTTG  480

Query  615   ATCCTTCAGGGGGAGGAGATCCAATTTTATATCAACATTTATTT  658
             ||||||||||||||||||||||||||||||||||||||||||||
Sbjct  481   ATCCTTCAGGGGGAGGAGATCCAATTTTATATCAACATTTATTT  524
```

>Rhipicephalus sanguineus isolate FT044 cytochrome c oxidase subunit I (COI) gene, partial cds; mitochondrial
Sequence ID: MN223683.1 Length: 535
Range 1: 1 to 524

Score:946 bits(512), Expect:0.0,
Identities:520/524(99%), Gaps:0/524(0%), Strand: Plus/Plus

```
Query  135   ATGCATTTATTATAAtttttttATAGTTATACCAATCATAATCGGAGGATTTGGAAATT  194
             ||||||||||||||||||||||||||||||||||||||||||||||||||||||||||||
Sbjct  1     ATGCATTTATTATAATTTTTTTTATAGTTATACCAATCATAATCGGAGGATTTGGAAATT  60

Query  195   GATTAGTCCCTATCATATTGGGTGCCCCAGATATAGCATTTCCACGAATAAATAATATAA  254
             ||||||| ||||||||||| ||||||||||||||||||||||||||||||||||||||||
Sbjct  61    GATTAGTTCCTATCATATTAGGTGCCCCAGATATAGCATTTCCACGAATAAATAATATAA  120

Query  255   GATTTTGACTTTTACCTCCTTCACTATTTTTATTAATTAACTCTTCATTAATTGAATCTG  314
             ||||||||||||||||||||||||||||||||||||||||||||||||||||||||||||
Sbjct  121   GATTTTGACTTTTACCTCCTTCACTATTTTTATTAATTAACTCTTCATTAATTGAATCTG  180

Query  315   GAGCTGGTACAGGATGAACAGTTTACCCTCCTCTATCTTCAAATTTATCACATTATGGCC  374
             ||||||||||||||||||||||||||||||||||||||||||||||||||||||||||||
Sbjct  181   GAGCTGGTACAGGATGAACAGTTTACCCTCCTCTATCTTCAAATTTATCACATTATGGCC  240

Query  375   CTTCAGTAGATTTAGCTATTTTTTCTCTTCATCTTGCTGGTGCTTCTTCAATTTTAGGTG  434
             ||||||||||||||||||| ||||||||||||||||||||||||||||||||||||||||
Sbjct  241   CTTCAGTAGATTTAGCTATCTTTTCTCTTCATCTTGCTGGTGCTTCTTCAATTTTAGGTG  300

Query  435   CAATTAACTTTATTACAACTATTGTAAACATACGATCAATTGGAATAACAATAGAACGTA  494
             ||||||||||||||||||||||||||||||||||||||||||||||||||||||||||||
Sbjct  301   CAATTAACTTTATTACAACTATTGTAAACATACGATCAATTGGAATAACAATAGAACGTA  360

Query  495   TACCATTATTTGTTTGATCTGTTTTAATTACAGCTATTTTATTACTTTTATCTTTACCTG  554
             |||||||||||||||||||||||||||||||||||||||||||||| |||||||||||||
Sbjct  361   TACCATTATTTGTTTGATCTGTTTTAATTACAGCTATTTTATTACTGTTATCTTTACCTG  420

Query  555   TATTAGCAGGTGCCATTACAATATTGTTAACTGATCGAAACTTTAATACATCATTTTTTG  614
```

```
            ||||||||||||||||||||||||||||||||||||||||||||||||||||||||||||
Sbjct  421  TATTAGCAGGTGCCATTACAATATTGTTAACTGATCGAAACTTTAATACATCATTTTTG  480

Query  615  ATCCTTCAGGGGGAGGAGATCCAATTTTATATCAACATTTATTT  658
            ||||||||||||||||||||||||||||||||||||||||||||
Sbjct  481  ATCCTTCAGGGGGAGGAGATCCAATTTTATATCAACATTTATTT  524
```

>Rhipicephalus sanguineus isolate Rsang2 cytochrome oxidase subunit 1 gene, partial cds; mitochondrial
Sequence ID: KY678135.1 Length: 790
Range 1: 18 to 675

Score:900 bits(487), Expect:0.0,
Identities:601/658(91%), Gaps:0/658(0%), Strand: Plus/Plus

```
Query  1    AACAATATATTTAATTTTTGGAGCATGATCAGGTATATTAGGACTTAGTATAAGAATATT  60
            |||||||||||||||||||||||||||||| || ||||||||| | || ||||||||| |
Sbjct  18   AACAATATATTTAATTTTTGGAGCATGATCCGGAATATTAGGATTAAGAATAAGAATACT  77

Query  61   AATTCGTATAGAATTAGGACAACCTGGAACTTTAATTGGAAATGATCAGATTTATAATGT  120
            |||||||||||||||||| |||||||||||  ||||||| |||||||| |||||||||||
Sbjct  78   AATTCGTATAGAATTAGGCCAACCTGGAACCCTAATTGGTAATGATCAAATTTATAATGT  137

Query  121  AATCGTAACAGCACATGCATTTATTATAAttttttttATAGTTATACCAATCATAATCGG  180
            ||| || ||||| ||||||||||||||||||||||||||||| |||||||||||||| ||
Sbjct  138  AATTGTTACAGCTCATGCATTTATTATAATTTTTTTTATAGTAATACCAATCATAATTGG  197

Query  181  AGGATTTGGAAATTGATTAGTCCCTATCATATTGGGTGCCCCAGATATAGCATTTCCACG  240
             ||||||||||| |||||||| ||||| ||| | || || |||||||||||||| |||||
Sbjct  198  TGGATTTGGAAACTGATTAGTTCCTATTATACTAGGAGCTCCAGATATAGCATTCCCACG  257

Query  241  AATAAATAATATAAGATTTTGACTTTTACCTCCTTCACTATTTTTATTAATTAACTCTTC  300
            ||||||||||||||||||||||||| | ||||| ||| ||||| |||||||||| |||||
Sbjct  258  AATAAATAATATAAGATTTTGACTTCTTCCTCCCTCATTATTTATATTAATTAATTCTTC  317

Query  301  ATTAATTGAATCTGGAGCTGGTACAGGATGAACAGTTTACCCTCCTCTATCTTCAAATTT  360
            ||||||||| || ||||| |||||||||||||||||||| |||||  |||| ||||||||
Sbjct  318  ATTAATTGAGTCAGGAGCAGGTACAGGATGAACAGTTTATCCTCCCTTATCCTCAAATTT  377

Query  361  ATCACATTATGGCCCTTCAGTAGATTTAGCTATTTTTTCTCTTCATCTTGCTGGTGCTTC  420
            |||||||||||| || ||||||||||||||||||||||||||||||||||||||||||||
Sbjct  378  ATCACATTATGGGCCATCAGTAGATTTAGCTATTTTTTCTCTTCATCTTGCTGGTGCTTC  437

Query  421  TTCAATTTTAGGTGCAATTAACTTTATTACAACTATTGTAAACATACGATCAATTGGAAT  480
            ||||||||||||||||||||| |||||||||||||||||||| |||||||| ||||||||
Sbjct  438  TTCAATTTTAGGTGCAATTAATTTATTACAACTATTGTAAATATACGATCTATTGGAAT  497

Query  481  AACAATAGAACGTATACCATTATTTGTTTGATCTGTTTTAATTACAGCTATTTTATTACT  540
            |||||||||||| |||||||||||||| ||||||||||||||||| || |||||||||||
Sbjct  498  AACAATAGAACGAATACCATTATTTGTATGATCTGTTTTAATTACTGCAATTTTATTACT  557
```

```
Query  541  TTTATCTTTACCTGTATTAGCAGGTGCCATTACAATATTGTTAACTGATCGAAACTTTAA  600
             |||||||||||||| ||||||||||||||||||||| | ||||| |||||||| |||||
Sbjct  558  ATTATCTTTACCTGTTTTAGCAGGTGCCATTACAATACTATTAACCGATCGAAATTTTAA  617

Query  601  TACATCATTTTTTGATCCTTCAGGGGGAGGAGATCCAATTTTATATCAACATTTATTT  658
             || ||||||||||| |||||||||||||| |||||||||||||||||||||||||||
Sbjct  618  CACTTCATTTTTTGACCCTTCAGGGGGAGGGGATCCAATTTTATATCAACATTTATTT  675
```

>Rhipicephalus sanguineus cytochrome oxidase subunit 1 (CO1) gene, partial cds; mitochondrial
Sequence ID: KU214592.1 Length: 829
Range 1: 1 to 648

Score:898 bits(486), Expect:0.0,
Identities:594/648(92%), Gaps:0/648(0%), Strand: Plus/Plus

```
Query  11   TTAATTTTTGGAGCATGATCAGGTATATTAGGACTTAGTATAAGAATATTAATTCGTATA  70
            |||||||||||||||||||| || ||||||||| | || ||||||||| |||||||||||
Sbjct  1    TTAATTTTTGGAGCATGATCCGGAATATTAGGATTAAGAATAAGAATACTAATTCGTATA  60

Query  71   GAATTAGGACAACCTGGAACTTTAATTGGAAATGATCAGATTTATAATGTAATCGTAACA  130
            |||||||| |||||||||||| ||||||| |||||||| |||||||||||||| || |||
Sbjct  61   GAATTAGGCCAACCTGGAACTCTAATTGGTAATGATCAAATTTATAATGTAATTGTTACA  120

Query  131  GCACATGCATTTATTATAAttttttttATAGTTATACCAATCATAATCGGAGGATTTGGA  190
            || ||||||||||||||||||||||||||||| |||||||||||||| || |||||||||
Sbjct  121  GCTCATGCATTTATTATAATTTTTTTTATAGTAATACCAATCATAATTGGTGGATTTGGA  180

Query  191  AATTGATTAGTCCCTATCATATTGGGTGCCCCAGATATAGCATTTCCACGAATAAATAAT  250
            || |||||||| ||||| ||| | || || ||||||||||||||||||||||||||||||
Sbjct  181  AACTGATTAGTTCCTATTATACTAGGAGCTCCAGATATAGCATTTCCACGAATAAATAAT  240

Query  251  ATAAGATTTTGACTTTTACCTCCTTCACTATTTTTATTAATTAACTCTTCATTAATTGAA  310
            ||||||||||||||| | ||||| ||| ||||| |||||||||| ||||||||||||||
Sbjct  241  ATAAGATTTTGACTTCTTCCTCCCTCATTATTTATATTAATTAATTCTTCATTAATTGAG  300

Query  311  TCTGGAGCTGGTACAGGATGAACAGTTTACCCTCCTCTATCTTCAAATTTATCACATTAT  370
            || ||||| ||||||||||| |||||||| ||||| ||||| ||||||||||||||||||
Sbjct  301  TCAGGAGCAGGTACAGGATGGACAGTTTATCCTCCCCTATCCTCAAATTTATCACATTAT  360

Query  371  GGCCCTTCAGTAGATTTAGCTATTTTTCTCTTCATCTTGCTGGTGCTTCTTCAATTTTA  430
            || || ||||||||||||||||||||||||||||||||||||||||||||||||||||||
Sbjct  361  GGGCCATCAGTAGATTTAGCTATTTTTCTCTTCATCTTGCTGGTGCTTCTTCAATTTTA  420

Query  431  GGTGCAATTAACTTTATTACAACTATTGTAAACATACGATCAATTGGAATAACAATAGAA  490
            ||||||||||| |||||||||||||||||||| |||||||| ||||||||||||||||||
Sbjct  421  GGTGCAATTAATTTTATTACAACTATTGTAAATATACGATCTATTGGAATAACAATAGAA  480

Query  491  CGTATACCATTATTTGTTTGATCTGTTTTAATTACAGCTATTTTATTACTTTTATCTTTA  550
            || |||||||||||||| ||||||||||||||||| || ||||||||||| |||||||||
Sbjct  481  CGAATACCATTATTTGTATGATCTGTTTTAATTACTGCAATTTTATTACTATTATCTTTA  540
```

```
Query  551  CCTGTATTAGCAGGTGCCATTACAATATTGTTAACTGATCGAAACTTTAATACATCATTT  610
            ||||| ||||||||||||||||||||||| ||||| |||||||| ||||| || ||||||
Sbjct  541  CCTGTTTTAGCAGGTGCCATTACAATATTATTAACCGATCGAAATTTTAACACTTCATTT  600

Query  611  TTTGATCCTTCAGGGGGAGGAGATCCAATTTTATATCAACATTTATTT  658
            ||||| |||||||||||||| |||||||||||||||||||||||||||
Sbjct  601  TTTGACCCTTCAGGGGGAGGGGATCCAATTTTATATCAACATTTATTT  648
```

>Rhipicephalus sanguineus isolate Gilan-e Gharb cytochrome oxidase subunit 1 gene, complete cds; mitochondrial
Sequence ID: KM494916.1 Length: 1539
Range 1: 45 to 702

Score:889 bits(481), Expect:0.0,
Identities:599/658(91%), Gaps:0/658(0%), Strand: Plus/Plus

```
Query  1    AACAATATATTTAATTTTTGGAGCATGATCAGGTATATTAGGACTTAGTATAAGAATATT  60
            |||||||||||||||||||||||||||||| || ||||||||| | || ||||||||| |
Sbjct  45   AACAATATATTTAATTTTTGGAGCATGATCCGGAATATTAGGATTAAGAATAAGAATACT  104

Query  61   AATTCGTATAGAATTAGGACAACCTGGAACTTTAATTGGAAATGATCAGATTTATAATGT  120
            |||||||||||||||||| |||||||||||| ||||||| |||||||| |||||||||||
Sbjct  105  AATTCGTATAGAATTAGGTCAACCTGGAACTCTAATTGGTAATGATCAAATTTATAATGT  164

Query  121  AATCGTAACAGCACATGCATTTATTATAAttttttttATAGTTATACCAATCATAATCGG  180
            ||| || ||||| ||||||||||||||||||||||||||||| |||||||| ||||| ||
Sbjct  165  AATTGTTACAGCTCATGCATTTATTATAATTTTTTTTATAGTAATACCAATTATAATTGG  224

Query  181  AGGATTTGGAAATTGATTAGTCCCTATCATATTGGGTGCCCCAGATATAGCATTTCCACG  240
             ||||||||||| |||||||| ||||| ||| | || || |||||||||||||| |||||
Sbjct  225  TGGATTTGGAAACTGATTAGTACCTATTATACTAGGAGCTCCAGATATAGCATTCCCACG  284

Query  241  AATAAATAATATAAGATTTTGACTTTTACCTCCTTCACTATTTTTATTAATTAACTCTTC  300
            ||||||||||||||||||||||||| | ||||| ||| ||||| |||||||||| |||||
Sbjct  285  AATAAATAATATAAGATTTTGACTTCTTCCTCCCTCATTATTTATATTAATTAATTCTTC  344

Query  301  ATTAATTGAATCTGGAGCTGGTACAGGATGAACAGTTTACCCTCCTCTATCTTCAAATTT  360
            ||||||||| || ||||| |||||||||||||||||||| ||||| ||||| ||||||||
Sbjct  345  ATTAATTGAGTCAGGAGCAGGTACAGGATGAACAGTTTATCCTCCCCTATCCTCAAATTT  404

Query  361  ATCACATTATGGCCCTTCAGTAGATTTAGCTATTTTTTCTCTTCATCTTGCTGGTGCTTC  420
            |||||||||||| || ||||||||||||||||||||||||||||||||||||||||||||
Sbjct  405  ATCACATTATGGGCCATCAGTAGATTTAGCTATTTTTTCTCTTCATCTTGCTGGTGCTTC  464

Query  421  TTCAATTTTAGGTGCAATTAACTTTATTACAACTATTGTAAACATACGATCAATTGGAAT  480
            ||||||||||||||||||||| ||||||||||||||||| || |||||||| ||||||||
Sbjct  465  TTCAATTTTAGGTGCAATTAATTTTATTACAACTATTGTGAATATACGATCTATTGGAAT  524

Query  481  AACAATAGAACGTATACCATTATTTGTTTGATCTGTTTTAATTACAGCTATTTTATTACT  540
            |||||||||||| |||||||||||||| ||||||||||||||||| || |||||||||||
```

```
Sbjct  525  AACAATAGAACGAATACCATTATTTGTATGATCTGTTTTAATTACTGCAATTTTATTACT  584

Query  541  TTTATCTTTACCTGTATTAGCAGGTGCCATTACAATATTGTTAACTGATCGAAACTTTAA  600
             |||||||||||||| ||||||||||| ||||||||| | ||||| |||||||| |||||
Sbjct  585  ATTATCTTTACCTGTTTTAGCAGGTGCTATTACAATACTATTAACCGATCGAAATTTTAA  644

Query  601  TACATCATTTTTTGATCCTTCAGGGGGAGGAGATCCAATTTTATATCAACATTTATTT  658
             || ||||||||||| |||||||| ||||| |||||||||||||||||||||||||||
Sbjct  645  CACTTCATTTTTTGACCCTTCAGGAGGAGGGGATCCAATTTTATATCAACATTTATTT  702
```

>Rhipicephalus sanguineus isolate Tehran cytochrome oxidase subunit 1 gene, complete cds; mitochondrial
Sequence ID: KM494915.1 Length: 1539
Range 1: 45 to 702

Score:889 bits(481), Expect:0.0,
Identities:599/658(91%), Gaps:0/658(0%), Strand: Plus/Plus

```
Query  1    AACAATATATTTAATTTTTGGAGCATGATCAGGTATATTAGGACTTAGTATAAGAATATT  60
            |||||||||||||||||||||||||||||| || ||||||||| | || ||||||||| |
Sbjct  45   AACAATATATTTAATTTTTGGAGCATGATCCGGAATATTAGGATTAAGAATAAGAATACT  104

Query  61   AATTCGTATAGAATTAGGACAACCTGGAACTTTAATTGGAAATGATCAGATTTATAATGT  120
            |||||||||||||||||| |||||||||||| ||||||| |||||||| |||||||||||
Sbjct  105  AATTCGTATAGAATTAGGTCAACCTGGAACTCTAATTGGTAATGATCAAATTTATAATGT  164

Query  121  AATCGTAACAGCACATGCATTTATTATAAttttttttATAGTTATACCAATCATAATCGG  180
            ||| || ||||| ||||||||||||||||||||||||||||| |||||||| ||||| ||
Sbjct  165  AATTGTTACAGCTCATGCATTTATTATAATTTTTTTTATAGTAATACCAATTATAATTGG  224

Query  181  AGGATTTGGAAATTGATTAGTCCCTATCATATTGGGTGCCCCAGATATAGCATTTCCACG  240
             ||||||||||| |||||||| ||||| ||| | || || |||||||||||||| |||||
Sbjct  225  TGGATTTGGAAACTGATTAGTACCTATTATACTAGGAGCTCCAGATATAGCATTCCCACG  284

Query  241  AATAAATAATATAAGATTTTGACTTTTACCTCCTTCACTATTTTTATTAATTAACTCTTC  300
            ||||||||||||||||||||||||| | ||||| ||| ||||| |||||||||| |||||
Sbjct  285  AATAAATAATATAAGATTTTGACTTCTTCCTCCCTCATTATTTATATTAATTAATTCTTC  344

Query  301  ATTAATTGAATCTGGAGCTGGTACAGGATGAACAGTTTACCCTCCTCTATCTTCAAATTT  360
            ||||||||| || ||||| |||||||||||||||||||| ||||| ||||| ||||||||
Sbjct  345  ATTAATTGAGTCAGGAGCAGGTACAGGATGAACAGTTTATCCTCCCCTATCCTCAAATTT  404

Query  361  ATCACATTATGGCCCTTCAGTAGATTTAGCTATTTTTTCTCTTCATCTTGCTGGTGCTTC  420
            |||||||||||| || ||||||||||||||||||||||||||||||||||||||||||||
Sbjct  405  ATCACATTATGGGCCATCAGTAGATTTAGCTATTTTTTCTCTTCATCTTGCTGGTGCTTC  464

Query  421  TTCAATTTTAGGTGCAATTAACTTTATTACAACTATTGTAAACATACGATCAATTGGAAT  480
            ||||||||||||||||||||| ||||||||||||||||| || |||||||| ||||||||
Sbjct  465  TTCAATTTTAGGTGCAATTAATTTTATTACAACTATTGTGAATATACGATCTATTGGAAT  524

Query  481  AACAATAGAACGTATACCATTATTTGTTTGATCTGTTTTAATTACAGCTATTTTATTACT  540
```

```
            |||||||||||| |||||||||||||| ||||||||||||||||| || |||||||||||
Sbjct  525  AACAATAGAACGAATACCATTATTTGTATGATCTGTTTTAATTACTGCAATTTTATTACT  584

Query  541  TTTATCTTTACCTGTATTAGCAGGTGCCATTACAATATTGTTAACTGATCGAAACTTTAA  600
             |||||||||||||| ||||||||||| ||||||||| | ||||| |||||||| |||||
Sbjct  585  ATTATCTTTACCTGTTTTAGCAGGTGCTATTACAATACTATTAACCGATCGAAATTTTAA  644

Query  601  TACATCATTTTTTGATCCTTCAGGGGGAGGAGATCCAATTTTATATCAACATTTATTT  658
             || ||||||||||| |||||||| ||||| |||||||||||||||||||||||||||
Sbjct  645  CACTTCATTTTTTGACCCTTCAGGAGGAGGGGATCCAATTTTATATCAACATTTATTT  702
```

>Rhipicephalus camicasi isolate SC0126 mitochondrion, complete genome
Sequence ID: NC_061616.1 Length: 14725
>Rhipicephalus camicasi isolate SC0126 mitochondrion, complete genome
Sequence ID: MZ323229.1 Length: 14725
Range 1: 45 to 702

Score:889 bits(481), Expect:0.0,
Identities:599/658(91%), Gaps:0/658(0%), Strand: Plus/Plus

```
Query  1    AACAATATATTTAATTTTTGGAGCATGATCAGGTATATTAGGACTTAGTATAAGAATATT  60
            ||||||||| |||||||||||||||||||| || ||||||||| | || ||||||||| |
Sbjct  45   AACAATATACTTAATTTTTGGAGCATGATCTGGAATATTAGGATTAAGAATAAGAATACT  104

Query  61   AATTCGTATAGAATTAGGACAACCTGGAACTTTAATTGGAAATGATCAGATTTATAATGT  120
            |||||||||||||||||| |||||||||||||||||||||||||| || |||||||||||
Sbjct  105  AATTCGTATAGAATTAGGCCAACCTGGAACTTTAATTGGAAATGACCAAATTTATAATGT  164

Query  121  AATCGTAACAGCACATGCATTTATTATAAttttttttATAGTTATACCAATCATAATCGG  180
            ||| |||||||||||||||||||||||||||||||||||||| |||||||| || || ||
Sbjct  165  AATTGTAACAGCACATGCATTTATTATAATTTTTTTTATAGTAATACCAATTATGATTGG  224

Query  181  AGGATTTGGAAATTGATTAGTCCCTATCATATTGGGTGCCCCAGATATAGCATTTCCACG  240
            |||||| |||||||||||||||||||| ||||| || || ||||||||||||||||||||
Sbjct  225  AGGATTCGGAAATTGATTAGTCCCTATTATATTAGGGGCTCCAGATATAGCATTTCCACG  284

Query  241  AATAAATAATATAAGATTTTGACTTTTACCTCCTTCACTATTTTTATTAATTAACTCTTC  300
            |||||||||||||||||||||| |  | ||||||||| |||||||||||||||| |||||
Sbjct  285  AATAAATAATATAAGATTTTGATTACTTCCTCCTTCATTATTTTTATTAATTAATTCTTC  344

Query  301  ATTAATTGAATCTGGAGCTGGTACAGGATGAACAGTTTACCCTCCTCTATCTTCAAATTT  360
            |||||||||||| ||||| || |||||||||||||||||||||||  |||| ||||||||
Sbjct  345  ATTAATTGAATCAGGAGCAGGGACAGGATGAACAGTTTACCCTCCCTTATCATCAAATTT  404

Query  361  ATCACATTATGGCCCTTCAGTAGATTTAGCTATTTTTTCTCTTCATCTTGCTGGTGCTTC  420
            |||||| ||||| || ||||||||| ||||||||||||||||||||||||||||||||||
Sbjct  405  ATCACACTATGGTCCATCAGTAGATCTAGCTATTTTTTCTCTTCATCTTGCTGGTGCTTC  464

Query  421  TTCAATTTTAGGTGCAATTAACTTTATTACAACTATTGTAAACATACGATCAATTGGAAT  480
            |||||||||||| |||||||| || |||||||| ||||||||||||||||| ||||||||
Sbjct  465  TTCAATTTTAGGCGCAATTAATTTCATTACAACCATTGTAAACATACGATCTATTGGAAT  524
```

```
Query  481   AACAATAGAACGTATACCATTATTTGTTTGATCTGTTTTAATTACAGCTATTTTATTACT  540
             |||||||||||| ||||| |||||||| ||||||||||||||||| || ||||||||| |
Sbjct  525   AACAATAGAACGAATACCTTTATTTGTATGATCTGTTTTAATTACTGCAATTTTATTATT  584

Query  541   TTTATCTTTACCTGTATTAGCAGGTGCCATTACAATATTGTTAACTGATCGAAACTTTAA  600
              ||||||||||| || ||||| ||||| ||||||||||| |||||||||||||| |||||
Sbjct  585   ATTATCTTTACCAGTTTTAGCTGGTGCAATTACAATATTATTAACTGATCGAAATTTTAA  644

Query  601   TACATCATTTTTTGATCCTTCAGGGGGAGGAGATCCAATTTTATATCAACATTTATTT  658
             ||| || || |||||||||||||| ||||| |||||||||||||||||||||||||||
Sbjct  645   TACCTCGTTCTTTGATCCTTCAGGAGGAGGGGATCCAATTTTATATCAACATTTATTT  702
```

>Rhipicephalus turanicus isolate COX1-225-Goat cytochrome c oxidase subunit I (COX1) gene, partial cds; mitochondrial
Sequence ID: MT800313.1 Length: 673
Range 1: 8 to 665

Score:883 bits(478), Expect:0.0,
Identities:598/658(91%), Gaps:0/658(0%), Strand: Plus/Plus

```
Query  1     AACAATATATTTAATTTTTGGAGCATGATCAGGTATATTAGGACTTAGTATAAGAATATT  60
             ||||||||||||||||||||| ||||||||||| ||||||||||| || ||||||||| |
Sbjct  8     AACAATATATTTAATTTTTGGGGCATGATCAGGAATATTAGGACTGAGAATAAGAATACT  67

Query  61    AATTCGTATAGAATTAGGACAACCTGGAACTTTAATTGGAAATGATCAGATTTATAATGT  120
             |||||| ||||||||||||||||||||||||||||||||||||||||| |||||||| ||
Sbjct  68    AATTCGCATAGAATTAGGACAACCTGGAACTTTAATTGGAAATGATCAAATTTATAACGT  127

Query  121   AATCGTAACAGCACATGCATTTATTATAAttttttttATAGTTATACCAATCATAATCGG  180
             ||| |||||||| |||||||||||||||||||||||||| || ||||| || ||||| ||
Sbjct  128   AATTGTAACAGCTCATGCATTTATTATAATTTTTTTTATGGTAATACCTATTATAATTGG  187

Query  181   AGGATTTGGAAATTGATTAGTCCCTATCATATTGGGTGCCCCAGATATAGCATTTCCACG  240
              |||||||||||||||||||| ||||| ||||| || || |||||||||||||| |||||
Sbjct  188   TGGATTTGGAAATTGATTAGTTCCTATTATATTAGGGGCTCCAGATATAGCATTCCCACG  247

Query  241   AATAAATAATATAAGATTTTGACTTTTACCTCCTTCACTATTTTTATTAATTAACTCTTC  300
              |||||||| |||||||||||| |  | ||||||||| ||||| ||||||||||||||||
Sbjct  248   TATAAATAACATAAGATTTTGATTACTTCCTCCTTCATTATTTCTATTAATTAACTCTTC  307

Query  301   ATTAATTGAATCTGGAGCTGGTACAGGATGAACAGTTTACCCTCCTCTATCTTCAAATTT  360
             |||||||||||| ||||| || ||||||||||| ||||| || ||| |||| ||||||||
Sbjct  308   ATTAATTGAATCAGGAGCAGGAACAGGATGAACTGTTTATCCCCCTTTATCCTCAAATTT  367

Query  361   ATCACATTATGGCCCTTCAGTAGATTTAGCTATTTTTTCTCTTCATCTTGCTGGTGCTTC  420
             |||||||||||| || ||||||||||||||||||||||||||||||||||||||||||||
Sbjct  368   ATCACATTATGGACCATCAGTAGATTTAGCTATTTTTTCTCTTCATCTTGCTGGTGCTTC  427

Query  421   TTCAATTTTAGGTGCAATTAACTTTATTACAACTATTGTAAACATACGATCAATTGGAAT  480
             ||||||| ||||||||||||| |||||||||||||||||||| |||||||| ||||||||
```

```
Sbjct  428   TTCAATTCTAGGTGCAATTAATTTTATTACAACTATTGTAAATATACGATCTATTGGAAT  487

Query  481   AACAATAGAACGTATACCATTATTTGTTTGATCTGTTTTAATTACAGCTATTTTATTACT  540
             |||||||||||| |||||||||||||||||||||||||||||||| || || ||| | ||
Sbjct  488   AACAATAGAACGAATACCATTATTTGTTTGATCTGTTTTAATTACCGCCATCTTACTTCT  547

Query  541   TTTATCTTTACCTGTATTAGCAGGTGCCATTACAATATTGTTAACTGATCGAAACTTTAA  600
              |||||||||||||| ||||||||||| ||||||||||| |||||||||||||| || ||
Sbjct  548   CTTATCTTTACCTGTTTTAGCAGGTGCTATTACAATATTATTAACTGATCGAAATTTCAA  607

Query  601   TACATCATTTTTTGATCCTTCAGGGGGAGGAGATCCAATTTTATATCAACATTTATTT  658
             ||| |||||||||||||||||||||||||| |||||||||||||| ||||| ||||||
Sbjct  608   TACTTCATTTTTTGATCCTTCAGGGGGAGGTGATCCAATTTTATACCAACACTTATTT  665
```

>Rhipicephalus turanicus isolate COX1-18-Sheep cytochrome c oxidase subunit I (COX1) gene, partial cds; mitochondrial
Sequence ID: MT800314.1 Length: 673
Range 1: 8 to 664

Score:881 bits(477), Expect:0.0,
Identities:597/657(91%), Gaps:0/657(0%), Strand: Plus/Plus

```
Query  1     AACAATATATTTAATTTTTGGAGCATGATCAGGTATATTAGGACTTAGTATAAGAATATT  60
             ||||||||||||||||||||| ||||||||||| ||||||||||| || ||||||||| |
Sbjct  8     AACAATATATTTAATTTTTGGGGCATGATCAGGAATATTAGGACTGAGAATAAGAATACT  67

Query  61    AATTCGTATAGAATTAGGACAACCTGGAACTTTAATTGGAAATGATCAGATTTATAATGT  120
             |||||| ||||||||||||||||||||||||||||||||||||||||| |||||||| ||
Sbjct  68    AATTCGCATAGAATTAGGACAACCTGGAACTTTAATTGGAAATGATCAAATTTATAACGT  127

Query  121   AATCGTAACAGCACATGCATTTATTATAAttttttttATAGTTATACCAATCATAATCGG  180
             ||| |||||||| |||||||||||||||||||||||||| || ||||| || ||||| ||
Sbjct  128   AATTGTAACAGCTCATGCATTTATTATAATTTTTTTTATGGTAATACCCATTATAATTGG  187

Query  181   AGGATTTGGAAATTGATTAGTCCCTATCATATTGGGTGCCCCAGATATAGCATTTCCACG  240
              |||||||||||||||||||| ||||| ||||| || || |||||||||||||| |||||
Sbjct  188   TGGATTTGGAAATTGATTAGTTCCTATTATATTAGGGGCTCCAGATATAGCATTCCCACG  247

Query  241   AATAAATAATATAAGATTTTGACTTTTACCTCCTTCACTATTTTTATTAATTAACTCTTC  300
              |||||||| |||||||||||| |  | ||||||||| ||||| ||||||||||||| ||
Sbjct  248   TATAAATAACATAAGATTTTGATTACTTCCTCCTTCATTATTTCTATTAATTAACTCCTC  307

Query  301   ATTAATTGAATCTGGAGCTGGTACAGGATGAACAGTTTACCCTCCTCTATCTTCAAATTT  360
             |||||||||||| ||||| || ||||||||||| ||||| || ||| |||| ||||||||
Sbjct  308   ATTAATTGAATCGGGAGCAGGAACAGGATGAACTGTTTATCCCCCTTTATCCTCAAATTT  367

Query  361   ATCACATTATGGCCCTTCAGTAGATTTAGCTATTTTTTCTCTTCATCTTGCTGGTGCTTC  420
             |||||||||||| || ||||||||||||||||||||||||||||||||||||||||||||
Sbjct  368   ATCACATTATGGACCATCAGTAGATTTAGCTATTTTTTCTCTTCATCTTGCTGGTGCTTC  427

Query  421   TTCAATTTTAGGTGCAATTAACTTTATTACAACTATTGTAAACATACGATCAATTGGAAT  480
```

```
                ||||||| ||||||||||||| ||||||||||||||||||||||||||||| ||||||||
Sbjct  428   TTCAATTCTAGGTGCAATTAATTTTATTACAACTATTGTAAACATACGATCTATTGGAAT  487

Query  481   AACAATAGAACGTATACCATTATTTGTTTGATCTGTTTTAATTACAGCTATTTTATTACT  540
             |||||||||||| |||||||||||||||||||||||||||||||| || || ||| | ||
Sbjct  488   AACAATAGAACGAATACCATTATTTGTTTGATCTGTTTTAATTACCGCCATCTTACTTCT  547

Query  541   TTTATCTTTACCTGTATTAGCAGGTGCCATTACAATATTGTTAACTGATCGAAACTTTAA  600
              |||||||||||||| ||||||||||| ||||||||||| |||||||||||||| || ||
Sbjct  548   CTTATCTTTACCTGTTTTAGCAGGTGCTATTACAATATTATTAACTGATCGAAATTTCAA  607

Query  601   TACATCATTTTTTGATCCTTCAGGGGGAGGAGATCCAATTTTATATCAACATTTATT  657
             ||| |||||||||||||||||||||||||| |||||||||||||| ||||| |||||
Sbjct  608   TACTTCATTTTTTGATCCTTCAGGGGGAGGTGATCCAATTTTATACCAACACTTATT  664
```

>Rhipicephalus turanicus isolate 31X cytochrome oxidase subunit 1 (COI) gene, partial cds; mitochondrial
Sequence ID: KY606303.1 Length: 873
Range 1: 66 to 723

Score:872 bits(472), Expect:0.0,
Identities:596/658(91%), Gaps:0/658(0%), Strand: Plus/Plus

```
Query  1     AACAATATATTTAATTTTTGGAGCATGATCAGGTATATTAGGACTTAGTATAAGAATATT  60
             |||||||||||||||||||||||||||||| || ||||||||| | || |||||||||||
Sbjct  66    AACAATATATTTAATTTTTGGAGCATGATCCGGGATATTAGGATTAAGAATAAGAATATT  125

Query  61    AATTCGTATAGAATTAGGACAACCTGGAACTTTAATTGGAAATGATCAGATTTATAATGT  120
             |||||| ||||| ||||||||||||||||||||||||||||||||||| |||||||||||
Sbjct  126   AATTCGAATAGAGTTAGGACAACCTGGAACTTTAATTGGAAATGATCAAATTTATAATGT  185

Query  121   AATCGTAACAGCACATGCATTTATTATAAttttttttATAGTTATACCAATCATAATCGG  180
             ||| |||||||| ||||||||||||||||||||||||||||| |||||||||||||| ||
Sbjct  186   AATTGTAACAGCTCATGCATTTATTATAATTTTTTTTATAGTAATACCAATCATAATTGG  245

Query  181   AGGATTTGGAAATTGATTAGTCCCTATCATATTGGGTGCCCCAGATATAGCATTTCCACG  240
             |||||||||||| |||||||| || || || || || || ||||| ||||||||||||||
Sbjct  246   AGGATTTGGAAACTGATTAGTGCCCATTATGTTAGGAGCTCCAGACATAGCATTTCCACG  305

Query  241   AATAAATAATATAAGATTTTGACTTTTACCTCCTTCACTATTTTTATTAATTAACTCTTC  300
             |||||||||||||||||||||| |  | ||||| ||| |||| ||||||||||| |||||
Sbjct  306   AATAAATAATATAAGATTTTGATTACTTCCTCCCTCATTATTCTTATTAATTAATTCTTC  365

Query  301   ATTAATTGAATCTGGAGCTGGTACAGGATGAACAGTTTACCCTCCTCTATCTTCAAATTT  360
             | | |||||||||||||| || ||||||||||| || ||||||||| |||||||||||||
Sbjct  366   ACTGATTGAATCTGGAGCAGGGACAGGATGAACTGTCTACCCTCCTTTATCTTCAAATTT  425

Query  361   ATCACATTATGGCCCTTCAGTAGATTTAGCTATTTTTTCTCTTCATCTTGCTGGTGCTTC  420
             ||| |||||||| || ||||||||||||||||| || ||||||||||| |||||||||||
Sbjct  426   ATCGCATTATGGACCATCAGTAGATTTAGCTATCTTCTCTCTTCATCTCGCTGGTGCTTC  485
```

```
Query  421  TTCAATTTTAGGTGCAATTAACTTTATTACAACTATTGTAAACATACGATCAATTGGAAT  480
            ||||||||||||||||||||| || |||||||||||||||||||||||||| ||||||||
Sbjct  486  TTCAATTTTAGGTGCAATTAATTTCATTACAACTATTGTAAACATACGATCTATTGGAAT  545

Query  481  AACAATAGAACGTATACCATTATTTGTTTGATCTGTTTTAATTACAGCTATTTTATTACT  540
            |||||||||||| ||||||||||| ||||||||||||||||| || ||||||||||| ||
Sbjct  546  AACAATAGAACGAATACCATTATTCGTTTGATCTGTTTTAATCACCGCTATTTTATTGCT  605

Query  541  TTTATCTTTACCTGTATTAGCAGGTGCCATTACAATATTGTTAACTGATCGAAACTTTAA  600
            | ||||||||||||| ||||||||||||||||||||||| |||||||||||||| || ||
Sbjct  606  TCTATCTTTACCTGTTTTAGCAGGTGCCATTACAATATTATTAACTGATCGAAATTTCAA  665

Query  601  TACATCATTTTTTGATCCTTCAGGGGGAGGAGATCCAATTTTATATCAACATTTATTT  658
             || ||||||||||| |||||||| ||||| || || |||||||||||||||||||||
Sbjct  666  CACTTCATTTTTTGACCCTTCAGGAGGAGGTGACCCTATTTTATATCAACATTTATTT  723
```

>Rhipicephalus turanicus isolate 19X cytochrome oxidase subunit 1 (COI) gene, partial cds; mitochondrial
Sequence ID: KY606302.1 Length: 873
Range 1: 66 to 723

Score:872 bits(472), Expect:0.0,
Identities:596/658(91%), Gaps:0/658(0%), Strand: Plus/Plus

```
Query  1    AACAATATATTTAATTTTTGGAGCATGATCAGGTATATTAGGACTTAGTATAAGAATATT  60
            |||||||||||||||||||||||||||||| || ||||||||| | || |||||||||||
Sbjct  66   AACAATATATTTAATTTTTGGAGCATGATCCGGGATATTAGGATTAAGAATAAGAATATT  125

Query  61   AATTCGTATAGAATTAGGACAACCTGGAACTTTAATTGGAAATGATCAGATTTATAATGT  120
            |||||| ||||| ||||||||||||||||||||||||||||||||||| |||||||||||
Sbjct  126  AATTCGAATAGAGTTAGGACAACCTGGAACTTTAATTGGAAATGATCAAATTTATAATGT  185

Query  121  AATCGTAACAGCACATGCATTTATTATAAttttttttATAGTTATACCAATCATAATCGG  180
            ||| |||||||| ||||||||||||||||||||||||||||| |||||||||||||| ||
Sbjct  186  AATTGTAACAGCTCATGCATTTATTATAATTTTTTTTATAGTAATACCAATCATAATTGG  245

Query  181  AGGATTTGGAAATTGATTAGTCCCTATCATATTGGGTGCCCCAGATATAGCATTTCCACG  240
            |||||||||||| |||||||| || || || || || || ||||| ||||||||||||||
Sbjct  246  AGGATTTGGAAACTGATTAGTGCCCATTATGTTAGGAGCTCCAGACATAGCATTTCCACG  305

Query  241  AATAAATAATATAAGATTTTGACTTTTACCTCCTTCACTATTTTTATTAATTAACTCTTC  300
            |||||||||||||||||||||| |  | ||||| ||| |||| ||||||||||| |||||
Sbjct  306  AATAAATAATATAAGATTTTGATTACTTCCTCCCTCATTATTCTTATTAATTAATTCTTC  365

Query  301  ATTAATTGAATCTGGAGCTGGTACAGGATGAACAGTTTACCCTCCTCTATCTTCAAATTT  360
            | | |||||||||||||| || ||||||||||| || ||||||||| |||||||||||||
Sbjct  366  ACTGATTGAATCTGGAGCAGGGACAGGATGAACTGTCTACCCTCCTTTATCTTCAAATTT  425

Query  361  ATCACATTATGGCCCTTCAGTAGATTTAGCTATTTTTTCTCTTCATCTTGCTGGTGCTTC  420
            ||| |||||||| || ||||||||||||||||| || ||||||||||| |||||||||||
Sbjct  426  ATCGCATTATGGACCATCAGTAGATTTAGCTATCTTCTCTCTTCATCTCGCTGGTGCTTC  485
```

```
Query  421  TTCAATTTTAGGTGCAATTAACTTTATTACAACTATTGTAAACATACGATCAATTGGAAT  480
            ||||||||||||||||||||| || |||||||||||||||||||||||||| ||||||||
Sbjct  486  TTCAATTTTAGGTGCAATTAATTTCATTACAACTATTGTAAACATACGATCTATTGGAAT  545

Query  481  AACAATAGAACGTATACCATTATTTGTTTGATCTGTTTTAATTACAGCTATTTTATTACT  540
            |||||||||||| ||||||||||| ||||||||||||||||| || ||||||||||| ||
Sbjct  546  AACAATAGAACGAATACCATTATTCGTTTGATCTGTTTTAATCACCGCTATTTTATTGCT  605

Query  541  TTTATCTTTACCTGTATTAGCAGGTGCCATTACAATATTGTTAACTGATCGAAACTTTAA  600
            | ||||||||||||| ||||||||||||||||||||||| |||||||||||||| || ||
Sbjct  606  TCTATCTTTACCTGTTTTAGCAGGTGCCATTACAATATTATTAACTGATCGAAATTTCAA  665

Query  601  TACATCATTTTTTGATCCTTCAGGGGGAGGAGATCCAATTTATATCAACATTTATTT  658
             || ||||||||||| |||||||| ||||| || || |||||||||||||||||||||
Sbjct  666  CACTTCATTTTTTGACCCTTCAGGAGGAGGTGACCCTATTTATATCAACATTTATTT  723
```

>Rhipicephalus turanicus isolate 19C cytochrome oxidase subunit 1 (COI) gene, partial cds; mitochondrial
Sequence ID: KY606301.1 Length: 873
Range 1: 66 to 723

Score:872 bits(472), Expect:0.0,
Identities:596/658(91%), Gaps:0/658(0%), Strand: Plus/Plus

```
Query  1    AACAATATATTTAATTTTTGGAGCATGATCAGGTATATTAGGACTTAGTATAAGAATATT  60
            |||||||||||||||||||||||||||||| || ||||||||| | || |||||||||||
Sbjct  66   AACAATATATTTAATTTTTGGAGCATGATCCGGGATATTAGGATTAAGAATAAGAATATT  125

Query  61   AATTCGTATAGAATTAGGACAACCTGGAACTTTAATTGGAAATGATCAGATTTATAATGT  120
            |||||| ||||| ||||||||||||||||||||||||||||||||||| |||||||||||
Sbjct  126  AATTCGAATAGAGTTAGGACAACCTGGAACTTTAATTGGAAATGATCAAATTTATAATGT  185

Query  121  AATCGTAACAGCACATGCATTTATTATAAttttttttATAGTTATACCAATCATAATCGG  180
            ||| |||||||| |||||||||||||||||||||||||||||| |||||||||||||| ||
Sbjct  186  AATTGTAACAGCTCATGCATTTATTATAATTTTTTTTATAGTAATACCAATCATAATTGG  245

Query  181  AGGATTTGGAAATTGATTAGTCCCTATCATATTGGGTGCCCCAGATATAGCATTTCCACG  240
            |||||||||||| |||||||| || || || || || || ||||| ||||||||||||||
Sbjct  246  AGGATTTGGAAACTGATTAGTGCCCATTATGTTAGGAGCTCCAGACATAGCATTTCCACG  305

Query  241  AATAAATAATATAAGATTTTGACTTTTACCTCCTTCACTATTTTTATTAATTAACTCTTC  300
            |||||||||||||||||||||| |  | ||||| ||| |||| ||||||||||| |||||
Sbjct  306  AATAAATAATATAAGATTTTGATTACTTCCTCCCTCATTATTCTTATTAATTAATTCTTC  365

Query  301  ATTAATTGAATCTGGAGCTGGTACAGGATGAACAGTTTACCCTCCTCTATCTTCAAATTT  360
            | | |||||||||||||| || ||||||||||| || ||||||||| |||||||||||||
Sbjct  366  ACTGATTGAATCTGGAGCAGGGACAGGATGAACTGTCTACCCTCCTTTATCTTCAAATTT  425

Query  361  ATCACATTATGGCCCTTCAGTAGATTTAGCTATTTTTTCTCTTCATCTTGCTGGTGCTTC  420
            ||| |||||||| || |||||||||||||||| || ||||||||||| ||||||||||||
```

```
Sbjct  426    ATCGCATTATGGACCATCAGTAGATTTAGCTATCTTCTCTCTTCATCTCGCTGGTGCTTC  485

Query  421    TTCAATTTTAGGTGCAATTAACTTTATTACAACTATTGTAAACATACGATCAATTGGAAT  480
              ||||||||||||||||||||| || |||||||||||||||||||||||||| ||||||||
Sbjct  486    TTCAATTTTAGGTGCAATTAATTTCATTACAACTATTGTAAACATACGATCTATTGGAAT  545

Query  481    AACAATAGAACGTATACCATTATTTGTTTGATCTGTTTTAATTACAGCTATTTTATTACT  540
              |||||||||||| ||||||||||| ||||||||||||||||| || ||||||||||| ||
Sbjct  546    AACAATAGAACGAATACCATTATTCGTTTGATCTGTTTTAATCACCGCTATTTTATTGCT  605

Query  541    TTTATCTTTACCTGTATTAGCAGGTGCCATTACAATATTGTTAACTGATCGAAACTTTAA  600
              | ||||||||||||| ||||||||||||||||||||||| |||||||||||||| || ||
Sbjct  606    TCTATCTTTACCTGTTTTAGCAGGTGCCATTACAATATTATTAACTGATCGAAATTTCAA  665

Query  601    TACATCATTTTTTGATCCTTCAGGGGGAGGAGATCCAATTTTATATCAACATTTATTT  658
               || ||||||||||| |||||||| ||||| || || |||||||||||||||||||||
Sbjct  666    CACTTCATTTTTTGACCCTTCAGGAGGAGGTGACCCTATTTTATATCAACATTTATTT  723
```

>Rhipicephalus turanicus isolate 12X cytochrome oxidase subunit 1 (COI) gene, partial cds; mitochondrial
Sequence ID: KY606300.1 Length: 873
Range 1: 66 to 723

Score:872 bits(472), Expect:0.0,
Identities:596/658(91%), Gaps:0/658(0%), Strand: Plus/Plus

```
Query  1      AACAATATATTTAATTTTTGGAGCATGATCAGGTATATTAGGACTTAGTATAAGAATATT  60
              |||||||||||||||||||||||||||||| || ||||||||| | || |||||||||||
Sbjct  66     AACAATATATTTAATTTTTGGAGCATGATCCGGGATATTAGGATTAAGAATAAGAATATT  125

Query  61     AATTCGTATAGAATTAGGACAACCTGGAACTTTAATTGGAAATGATCAGATTTATAATGT  120
              |||||| ||||| ||||||||||||||||||||||||||||||||||| |||||||||||
Sbjct  126    AATTCGAATAGAGTTAGGACAACCTGGAACTTTAATTGGAAATGATCAAATTTATAATGT  185

Query  121    AATCGTAACAGCACATGCATTTATTATAAttttttttATAGTTATACCAATCATAATCGG  180
              ||| |||||||| ||||||||||||||||||||||||||||| |||||||||||||| ||
Sbjct  186    AATTGTAACAGCTCATGCATTTATTATAATTTTTTTTATAGTAATACCAATCATAATTGG  245

Query  181    AGGATTTGGAAATTGATTAGTCCCTATCATATTGGGTGCCCCAGATATAGCATTTCCACG  240
              |||||||||||| |||||||| || || || || || || ||||| ||||||||||||||
Sbjct  246    AGGATTTGGAAACTGATTAGTGCCCATTATGTTAGGAGCTCCAGACATAGCATTTCCACG  305

Query  241    AATAAATAATATAAGATTTTGACTTTTACCTCCTTCACTATTTTTATTAATTAACTCTTC  300
              |||||||||||||||||||||| |  | ||||| ||| |||| ||||||||||| |||||
Sbjct  306    AATAAATAATATAAGATTTTGATTACTTCCTCCCTCATTATTCTTATTAATTAATTCTTC  365

Query  301    ATTAATTGAATCTGGAGCTGGTACAGGATGAACAGTTTACCCTCCTCTATCTTCAAATTT  360
              | | |||||||||||||| || ||||||||||| || ||||||||| |||||||||||||
Sbjct  366    ACTGATTGAATCTGGAGCAGGGACAGGATGAACTGTCTACCCTCCTTTATCTTCAAATTT  425

Query  361    ATCACATTATGGCCCTTCAGTAGATTTAGCTATTTTTTCTCTTCATCTTGCTGGTGCTTC  420
```

```
                 ||| |||||||| || ||||||||||||||||| || ||||||||||| |||||||||||
Sbjct  426  ATCGCATTATGGACCATCAGTAGATTTAGCTATCTTCTCTCTTCATCTCGCTGGTGCTTC  485

Query  421  TTCAATTTTAGGTGCAATTAACTTTATTACAACTATTGTAAACATACGATCAATTGGAAT  480
            ||||||||||||||||||||| || |||||||||||||||||||||||||| ||||||||
Sbjct  486  TTCAATTTTAGGTGCAATTAATTTCATTACAACTATTGTAAACATACGATCTATTGGAAT  545

Query  481  AACAATAGAACGTATACCATTATTTGTTTGATCTGTTTTAATTACAGCTATTTTATTACT  540
            |||||||||||| ||||||||||| ||||||||||||||||| || ||||||||||| ||
Sbjct  546  AACAATAGAACGAATACCATTATTCGTTTGATCTGTTTTAATCACCGCTATTTTATTGCT  605

Query  541  TTTATCTTTACCTGTATTAGCAGGTGCCATTACAATATTGTTAACTGATCGAAACTTTAA  600
            | ||||||||||||| ||||||||||||||||||||||| |||||||||||||| || ||
Sbjct  606  TCTATCTTTACCTGTTTTAGCAGGTGCCATTACAATATTATTAACTGATCGAAATTTCAA  665

Query  601  TACATCATTTTTTGATCCTTCAGGGGGAGGAGATCCAATTTTATATCAACATTTATTT  658
             || ||||||||||| |||||||| ||||| || || |||||||||||||||||||||
Sbjct  666  CACTTCATTTTTTGACCCTTCAGGAGGAGGTGACCCTATTTTATATCAACATTTATTT  723
```

>Rhipicephalus turanicus isolate 12C cytochrome oxidase subunit 1 (COI) gene, partial cds; mitochondrial
Sequence ID: KY606299.1 Length: 873
Range 1: 66 to 723

Score:872 bits(472), Expect:0.0,
Identities:596/658(91%), Gaps:0/658(0%), Strand: Plus/Plus

```
Query  1    AACAATATATTTAATTTTTGGAGCATGATCAGGTATATTAGGACTTAGTATAAGAATATT  60
            |||||||||||||||||||||||||||||| || ||||||||| | || |||||||||||
Sbjct  66   AACAATATATTTAATTTTTGGAGCATGATCCGGGATATTAGGATTAAGAATAAGAATATT  125

Query  61   AATTCGTATAGAATTAGGACAACCTGGAACTTTAATTGGAAATGATCAGATTTATAATGT  120
            |||||| ||||| ||||||||||||||||||||||||||||||||||| |||||||||||
Sbjct  126  AATTCGAATAGAGTTAGGACAACCTGGAACTTTAATTGGAAATGATCAAATTTATAATGT  185

Query  121  AATCGTAACAGCACATGCATTTATTATAAttttttttATAGTTATACCAATCATAATCGG  180
            ||| |||||||| ||||||||||||||||||||||||||||| |||||||||||||| ||
Sbjct  186  AATTGTAACAGCTCATGCATTTATTATAATTTTTTTTATAGTAATACCAATCATAATTGG  245

Query  181  AGGATTTGGAAATTGATTAGTCCCTATCATATTGGGTGCCCCAGATATAGCATTTCCACG  240
            |||||||||||| |||||||| || || || || || || ||||| ||||||||||||||
Sbjct  246  AGGATTTGGAAACTGATTAGTGCCCATTATGTTAGGAGCTCCAGACATAGCATTTCCACG  305

Query  241  AATAAATAATATAAGATTTTGACTTTTACCTCCTTCACTATTTTTATTAATTAACTCTTC  300
            |||||||||||||||||||||| |  | ||||| ||| |||| ||||||||||| |||||
Sbjct  306  AATAAATAATATAAGATTTTGATTACTTCCTCCCTCATTATTCTTATTAATTAATTCTTC  365

Query  301  ATTAATTGAATCTGGAGCTGGTACAGGATGAACAGTTTACCCTCCTCTATCTTCAAATTT  360
            | | |||||||||||||| || ||||||||||| || ||||||||| |||||||||||||
Sbjct  366  ACTGATTGAATCTGGAGCAGGGACAGGATGAACTGTCTACCCTCCTTTATCTTCAAATTT  425
```

```
Query  361  ATCACATTATGGCCCTTCAGTAGATTTAGCTATTTTTTCTCTTCATCTTGCTGGTGCTTC  420
            ||| |||||||| || ||||||||||||||||| || ||||||||||| |||||||||||
Sbjct  426  ATCGCATTATGGACCATCAGTAGATTTAGCTATCTTCTCTCTTCATCTCGCTGGTGCTTC  485

Query  421  TTCAATTTTAGGTGCAATTAACTTTATTACAACTATTGTAAACATACGATCAATTGGAAT  480
            ||||||||||||||||||||| || |||||||||||||||||||||||||| ||||||||
Sbjct  486  TTCAATTTTAGGTGCAATTAATTTCATTACAACTATTGTAAACATACGATCTATTGGAAT  545

Query  481  AACAATAGAACGTATACCATTATTTGTTTGATCTGTTTTAATTACAGCTATTTTATTACT  540
            |||||||||||| ||||||||||| ||||||||||||||||| || ||||||||||| ||
Sbjct  546  AACAATAGAACGAATACCATTATTCGTTTGATCTGTTTTAATCACCGCTATTTTATTGCT  605

Query  541  TTTATCTTTACCTGTATTAGCAGGTGCCATTACAATATTGTTAACTGATCGAAACTTTAA  600
            | ||||||||||||| ||||||||||||||||||||||| |||||||||||||| || ||
Sbjct  606  TCTATCTTTACCTGTTTTAGCAGGTGCCATTACAATATTATTAACTGATCGAAATTTCAA  665

Query  601  TACATCATTTTTTGATCCTTCAGGGGGAGGAGATCCAATTTTATATCAACATTTATTT  658
             || ||||||||||| |||||||| ||||| || || |||||||||||||||||||||
Sbjct  666  CACTTCATTTTTTGACCCTTCAGGAGGAGGTGACCCTATTTTATATCAACATTTATTT  723
```

>Rhipicephalus turanicus isolate COX1-7-Sheep cytochrome c oxidase subunit I (COX1) gene, partial cds; mitochondrial
Sequence ID: MT800312.1 Length: 673
Range 1: 8 to 664

Score:865 bits(468), Expect:0.0,
Identities:594/657(90%), Gaps:0/657(0%), Strand: Plus/Plus

```
Query  1    AACAATATATTTAATTTTTGGAGCATGATCAGGTATATTAGGACTTAGTATAAGAATATT  60
            ||||||||||||||||||||| ||||||||||| ||||||||||| || ||||||||| |
Sbjct  8    AACAATATATTTAATTTTTGGGGCATGATCAGGAATATTAGGACTGAGAATAAGAATACT  67

Query  61   AATTCGTATAGAATTAGGACAACCTGGAACTTTAATTGGAAATGATCAGATTTATAATGT  120
            |||||| |||||||||||||||||||||||||||||||||||||| || |||||||| ||
Sbjct  68   AATTCGCATAGAATTAGGACAACCTGGAACTTTAATTGGAAATGACCAAATTTATAACGT  127

Query  121  AATCGTAACAGCACATGCATTTATTATAAttttttttATAGTTATACCAATCATAATCGG  180
            ||| |||||||| |||||||||||||||||||||||||| || ||||| || ||||| ||
Sbjct  128  AATTGTAACAGCTCATGCATTTATTATAATTTTTTTTATGGTAATACCTATTATAATTGG  187

Query  181  AGGATTTGGAAATTGATTAGTCCCTATCATATTGGGTGCCCCAGATATAGCATTTCCACG  240
             |||||||||||||||||||| ||||||||||| || || ||||||||||||||||||||
Sbjct  188  TGGATTTGGAAATTGATTAGTTCCTATCATATTAGGGGCTCCAGATATAGCATTTCCACG  247

Query  241  AATAAATAATATAAGATTTTGACTTTTACCTCCTTCACTATTTTTATTAATTAACTCTTC  300
             |||||||| |||||||||||| |  | ||||||||| ||||| ||||||||||||| ||
Sbjct  248  TATAAATAACATAAGATTTTGATTACTTCCTCCTTCATTATTTCTATTAATTAACTCCTC  307

Query  301  ATTAATTGAATCTGGAGCTGGTACAGGATGAACAGTTTACCCTCCTCTATCTTCAAATTT  360
            |||||||||||| ||||| || || |||||||| ||||| || ||| |||| ||||||||
Sbjct  308  ATTAATTGAATCGGGAGCAGGAACGGGATGAACTGTTTATCCCCCTTTATCCTCAAATTT  367
```

```
Query  361  ATCACATTATGGCCCTTCAGTAGATTTAGCTATTTTTCTCTTCATCTTGCTGGTGCTTC  420
            |||||||||||| || ||||||||||||||||||||||||||||||||||||||||||||
Sbjct  368  ATCACATTATGGACCATCAGTAGATTTAGCTATTTTTCTCTTCATCTTGCTGGTGCTTC  427

Query  421  TTCAATTTTAGGTGCAATTAACTTTATTACAACTATTGTAAACATACGATCAATTGGAAT  480
            ||||||| ||||||||||||| ||||||||||||||| ||||||||||||| ||||||||
Sbjct  428  TTCAATTCTAGGTGCAATTAATTTTATTACAACTATTCTAAACATACGATCTATTGGAAT  487

Query  481  AACAATAGAACGTATACCATTATTTGTTTGATCTGTTTTAATTACAGCTATTTTATTACT  540
            |||||||||||| |||||||||||||| |||||||||||||||||  | || ||| | ||
Sbjct  488  AACAATAGAACGAATACCATTATTTGTCTGATCTGTTTTAATTACCACCATCTTACTTCT  547

Query  541  TTTATCTTTACCTGTATTAGCAGGTGCCATTACAATATTGTTAACTGATCGAAACTTTAA  600
             |||||||||||||| ||||||||||| ||||||||||| |||||||||||||| || ||
Sbjct  548  CTTATCTTTACCTGTTTTAGCAGGTGCTATTACAATATTATTAACTGATCGAAATTTCAA  607

Query  601  TACATCATTTTTTGATCCTTCAGGGGGAGGAGATCCAATTTTATATCAACATTTATT  657
            ||| |||||||||||||||||||||||||| |||||||||||||| ||||| |||||
Sbjct  608  TACCTCATTTTTTGATCCTTCAGGGGGAGGTGATCCAATTTTATACCAACACTTATT  664
```

>Rhipicephalus turanicus isolate GY34-1 cytochrome c oxidase subunit I (COX1) gene, partial cds; mitochondrial
Sequence ID: MT079206.1 Length: 678
Range 1: 1 to 652

Score:861 bits(466), Expect:0.0,
Identities:590/652(90%), Gaps:0/652(0%), Strand: Plus/Plus

```
Query  7    ATATTTAATTTTTGGAGCATGATCAGGTATATTAGGACTTAGTATAAGAATATTAATTCG  66
            ||||||||||||||||||||||||||||||||||||| | || |||||||||||||||||
Sbjct  1    ATATTTAATTTTTGGAGCATGATCAGGTATATTAGGATTAAGAATAAGAATATTAATTCG  60

Query  67   TATAGAATTAGGACAACCTGGAACTTTAATTGGAAATGATCAGATTTATAATGTAATCGT  126
             ||||||||||||||||||||||||||||||||||||||||| |||||||||||||| ||
Sbjct  61   AATAGAATTAGGACAACCTGGAACTTTAATTGGAAATGATCAAATTTATAATGTAATTGT  120

Query  127  AACAGCACATGCATTTATTATAAttttttttATAGTTATACCAATCATAATCGGAGGATT  186
            |||||| ||||||||||||||||||||||||||||| |||||||||||||| ||||||||
Sbjct  121  AACAGCTCATGCATTTATTATAATTTTTTTTATAGTAATACCAATCATAATTGGAGGATT  180

Query  187  TGGAAATTGATTAGTCCCTATCATATTGGGTGCCCCAGATATAGCATTTCCACGAATAAA  246
            |||||| || ||||| || || ||||| || || ||||| ||||||||||||||||||||
Sbjct  181  TGGAAACTGGTTAGTTCCAATTATATTAGGAGCTCCAGACATAGCATTTCCACGAATAAA  240

Query  247  TAATATAAGATTTTGACTTTTACCTCCTTCACTATTTTTATTAATTAACTCTTCATTAAT  306
            |||||||||||||||| |  | || || ||| |||| ||||||||||| |||||| | ||
Sbjct  241  TAATATAAGATTTTGATTACTTCCCCCCTCATTATTCTTATTAATTAATTCTTCACTGAT  300

Query  307  TGAATCTGGAGCTGGTACAGGATGAACAGTTTACCCTCCTCTATCTTCAAATTTATCACA  366
            ||||||||| || || ||||| ||||| ||||| |||||| |||| ||||||||||| ||
```

```
Sbjct  301   TGAATCTGGGGCAGGGACAGGGTGAACTGTTTATCCTCCTTTATCCTCAAATTTATCCCA  360

Query  367   TTATGGCCCTTCAGTAGATTTAGCTATTTTTTCTCTTCATCTTGCTGGTGCTTCTTCAAT  426
             |||||| || ||||||||||||||||| || |||||||||||||||||||||||||||||
Sbjct  361   TTATGGACCATCAGTAGATTTAGCTATCTTCTCTCTTCATCTTGCTGGTGCTTCTTCAAT  420

Query  427   TTTAGGTGCAATTAACTTTATTACAACTATTGTAAACATACGATCAATTGGAATAACAAT  486
             |||||| |||||||| || |||||||||||||||||||||||||| ||||||||||||||
Sbjct  421   TTTAGGCGCAATTAATTTCATTACAACTATTGTAAACATACGATCTATTGGAATAACAAT  480

Query  487   AGAACGTATACCATTATTTGTTTGATCTGTTTTAATTACAGCTATTTTATTACTTTTATC  546
             |||||| ||||||||||| ||||||||||||||||| || ||||||||||| ||| ||||
Sbjct  481   AGAACGAATACCATTATTCGTTTGATCTGTTTTAATCACCGCTATTTTATTGCTTCTATC  540

Query  547   TTTACCTGTATTAGCAGGTGCCATTACAATATTGTTAACTGATCGAAACTTTAATACATC  606
             ||||||||| ||||||||||||||||||||| | |||||||||||||| ||||| || ||
Sbjct  541   TTTACCTGTTTTAGCAGGTGCCATTACAATACTATTAACTGATCGAAATTTTAACACTTC  600

Query  607   ATTTTTTGATCCTTCAGGGGGAGGAGATCCAATTTTATATCAACATTTATTT  658
             ||||||||| |||||||| ||||| || ||||||||||||||||||||||||
Sbjct  601   ATTTTTTGACCCTTCAGGAGGAGGTGACCCAATTTTATATCAACATTTATTT  652


>Rhipicephalus turanicus voucher CROBB808 cytochrome oxidase subunit 1 (COI)
gene, partial cds; mitochondrial
Sequence ID: MZ305547.1 Length: 658
Range 1: 1 to 658

Score:861 bits(466), Expect:0.0,
Identities:594/658(90%),  Gaps:0/658(0%), Strand: Plus/Plus

Query  1     AACAATATATTTAATTTTTGGAGCATGATCAGGTATATTAGGACTTAGTATAAGAATATT  60
             |||||||||||||||||||||||||||||| || ||||||||| | || |||||||||||
Sbjct  1     AACAATATATTTAATTTTTGGAGCATGATCCGGGATATTAGGATTAAGAATAAGAATATT  60

Query  61    AATTCGTATAGAATTAGGACAACCTGGAACTTTAATTGGAAATGATCAGATTTATAATGT  120
             |||||| ||||| |||||||||||||| ||||||||||||||||||| ||||||||||||
Sbjct  61    AATTCGAATAGAGTTAGGACAACCTGGGACTTTAATTGGAAATGATCAAATTTATAATGT  120

Query  121   AATCGTAACAGCACATGCATTTATTATAAttttttttATAGTTATACCAATCATAATCGG  180
             ||| |||||||| ||||||||||||||||||||||||||||| |||||||||||||| ||
Sbjct  121   AATTGTAACAGCTCATGCATTTATTATAATTTTTTTTATAGTAATACCAATCATAATTGG  180

Query  181   AGGATTTGGAAATTGATTAGTCCCTATCATATTGGGTGCCCCAGATATAGCATTTCCACG  240
             |||||||||||| |||||||| || || || || || || ||||| ||||||||||||||
Sbjct  181   AGGATTTGGAAACTGATTAGTGCCCATTATGTTAGGAGCTCCAGACATAGCATTTCCACG  240

Query  241   AATAAATAATATAAGATTTTGACTTTTACCTCCTTCACTATTTTTATTAATTAACTCTTC  300
             |||||||||||||||||||||| |  | ||||| ||| |||| ||||||||||| |||||
Sbjct  241   AATAAATAATATAAGATTTTGATTACTTCCTCCCTCATTATTCTTATTAATTAATTCTTC  300

Query  301   ATTAATTGAATCTGGAGCTGGTACAGGATGAACAGTTTACCCTCCTCTATCTTCAAATTT  360
```

```
               | | |||||||||||||| || ||||||||||| || ||||||||| |||||||||||||
Sbjct  301  ACTGATTGAATCTGGAGCAGGGACAGGATGAACTGTCTACCCTCCTTTATCTTCAAATTT  360

Query  361  ATCACATTATGGCCCTTCAGTAGATTTAGCTATTTTTTCTCTTCATCTTGCTGGTGCTTC  420
            ||| |||||||| || ||||||||||||||||| || ||||||||||| |||||||||||
Sbjct  361  ATCGCATTATGGACCATCAGTAGATTTAGCTATCTTCTCTCTTCATCTCGCTGGTGCTTC  420

Query  421  TTCAATTTTAGGTGCAATTAACTTTATTACAACTATTGTAAACATACGATCAATTGGAAT  480
            ||||||||||||||||||||| || ||||||||||||||||| |||||||| ||||||||
Sbjct  421  TTCAATTTTAGGTGCAATTAATTTCATTACAACTATTGTAAATATACGATCTATTGGAAT  480

Query  481  AACAATAGAACGTATACCATTATTTGTTTGATCTGTTTTAATTACAGCTATTTTATTACT  540
            |||||||||||| ||||||||||| ||||||||||||||||| || ||||||||||| ||
Sbjct  481  AACAATAGAACGAATACCATTATTCGTTTGATCTGTTTTAATCACCGCTATTTTATTGCT  540

Query  541  TTTATCTTTACCTGTATTAGCAGGTGCCATTACAATATTGTTAACTGATCGAAACTTTAA  600
            | ||||||||||||| ||||||||||||||||||||||| |||||||||||||| || ||
Sbjct  541  TCTATCTTTACCTGTTTTAGCAGGTGCCATTACAATATTATTAACTGATCGAAATTTCAA  600

Query  601  TACATCATTTTTTGATCCTTCAGGGGGAGGAGATCCAATTTTATATCAACATTTATTT  658
             || ||||||||||| |||||||| ||||| || || |||||||||||||||||||||
Sbjct  601  CACTTCATTTTTTGACCCTTCAGGAGGAGGTGACCCTATTTTATATCAACATTTATTT  658
```

```
Query #6: TKCJJ012-19|TKCJ012018A12|Rhipicephalus sanguineus|COI-5P Query ID:
lcl|Query_26768 Length: 658

Sequences producing significant alignments:
                                                                  Scientific
 Common                     Max    Total Query   E   Per.   Acc.

Description                                                       Name
 Name            Taxid      Score  Score cover Value Ident  Len        Accession

Rhipicephalus sanguineus sensu lato 'tropical lineage' isolate...
Rhipicephalu... NA              2138177    1216   1216  100%  0.0   100.00 688
     MF425995.1
Rhipicephalus sanguineus sensu lato 'tropical lineage' isolate...
Rhipicephalu... NA              2138177    1216   1216  100%  0.0   100.00 703
     MF425994.1
Rhipicephalus sanguineus sensu lato 'tropical lineage' isolate...
Rhipicephalu... NA              2138177    1216   1216  100%  0.0   100.00 702
     MF425993.1
Rhipicephalus sanguineus sensu lato 'tropical lineage' isolate...
Rhipicephalu... NA              2138177    1216   1216  100%  0.0   100.00 693
     MF425992.1
Rhipicephalus sanguineus haplotype RsanColSinSP cytochrome c...
Rhipicephalu... brown dog tick  34632      1216   1216  100%  0.0   100.00 690
     KT906183.1
Rhipicephalus sanguineus haplotype RsanColSanM cytochrome c...
Rhipicephalu... brown dog tick  34632      1216   1216  100%  0.0   100.00 690
```

KT906182.1
Rhipicephalus sanguineus isolate Q3 mitochondrion, complete...
Rhipicephalu... brown dog tick 34632 1216 1216 100% 0.0 100.00 14711
OM368327.1
Rhipicephalus sanguineus isolate C19 mitochondrion, complete...
Rhipicephalu... brown dog tick 34632 1216 1216 100% 0.0 100.00 14714
OM368323.1
Rhipicephalus sanguineus isolate A44 mitochondrion, complete...
Rhipicephalu... brown dog tick 34632 1216 1216 100% 0.0 100.00 14713
OM368322.1
Rhipicephalus sanguineus isolate Guangxi cytochrome oxidase...
Rhipicephalu... brown dog tick 34632 1216 1216 100% 0.0 100.00 707
JQ737084.1
Rhipicephalus sanguineus cytochrome oxidase subunit 1 (COI)...
Rhipicephalu... brown dog tick 34632 1216 1216 100% 0.0 100.00 658
KF437543.1
Rhipicephalus linnaei isolate LAO32-2 clone JS5930...
Rhipicephalu... NA 2138177 1216 1216 100% 0.0 100.00 14715
MW429383.1
Rhipicephalus linnaei mitochondrion, complete sequence
Rhipicephalu... NA 2138177 1216 1216 100% 0.0 100.00 14711
NC_060409.1
Ixodidae sp. sc_01082 cytochrome oxidase subunit 1 (COI) gene,... Ixodidae sp.... NA 1901042 1210 1210 100% 0.0 99.85 658
KX053537.1
Rhipicephalus sanguineus haplotype RsanColCorL cytochrome c...
Rhipicephalu... brown dog tick 34632 1210 1210 100% 0.0 99.85 690
KT906185.1
Rhipicephalus sanguineus haplotype RsanColTolC cytochrome c...
Rhipicephalu... brown dog tick 34632 1210 1210 100% 0.0 99.85 690
KT906184.1
Rhipicephalus sanguineus voucher CAS-T065 cytochrome oxidase...
Rhipicephalu... brown dog tick 34632 1210 1210 100% 0.0 99.85 658
KF200113.1
Rhipicephalus linnaei isolate SC931-1 clone JS5927...
Rhipicephalu... NA 2138177 1210 1210 100% 0.0 99.85 14717
MW429382.1
Rhipicephalus sanguineus voucher DO-311 cytochrome oxidase...
Rhipicephalu... brown dog tick 34632 1205 1205 99% 0.0 99.85 658
KX360367.1
Rhipicephalus sanguineus haplotype RsanColSamCM cytochrome c...
Rhipicephalu... brown dog tick 34632 1205 1205 100% 0.0 99.70 690
KT906186.1
Rhipicephalus sanguineus voucher GRP5-154 cytochrome oxidase...
Rhipicephalu... brown dog tick 34632 1205 1205 100% 0.0 99.70 658
KF200112.1
Rhipicephalus sanguineus mitochondrion, complete genome
Rhipicephalu... brown dog tick 34632 1205 1205 100% 0.0 99.70 14714
JX416325.1
Rhipicephalus sanguineus isolate T95D15 cytochrome c oxidase...
Rhipicephalu... brown dog tick 34632 1192 1192 98% 0.0 100.00 661
MZ401443.1
Rhipicephalus sanguineus isolate T81D7 cytochrome c oxidase...
Rhipicephalu... brown dog tick 34632 1192 1192 98% 0.0 100.00 661

```
     MZ401441.1
Rhipicephalus sanguineus isolate T79D2 cytochrome c oxidase...
Rhipicephalu... brown dog tick  34632      1192   1192  98%   0.0   99.85  664
     MZ401440.1
Rhipicephalus sanguineus isolate WZ004 cytochrome oxidase...
Rhipicephalu... brown dog tick  34632      1190   1190  97%   0.0   100.00 664
     MG969507.1
Rhipicephalus sanguineus voucher GRP5-150 cytochrome oxidase...
Rhipicephalu... brown dog tick  34632      1188   1188  98%   0.0   99.69  649
     KF200084.1
Rhipicephalus sanguineus isolate LIC6084B cytochrome oxidase...
Rhipicephalu... brown dog tick  34632      1186   1186  97%   0.0   100.00 768
     KX383817.1
Rhipicephalus sanguineus isolate T91D13 cytochrome c oxidase...
Rhipicephalu... brown dog tick  34632      1186   1186  97%   0.0   100.00 657
     MZ401442.1
Rhipicephalus sanguineus isolate T63D1 cytochrome c oxidase...
Rhipicephalu... brown dog tick  34632      1186   1186  97%   0.0   100.00 661
     MZ401438.1
Rhipicephalus sanguineus isolate WZ003 cytochrome oxidase...
Rhipicephalu... brown dog tick  34632      1184   1184  97%   0.0   99.84  664
     MG969506.1
Rhipicephalus sanguineus isolate WZ002 cytochrome oxidase...
Rhipicephalu... brown dog tick  34632      1184   1184  97%   0.0   99.84  664
     MG969505.1
Rhipicephalus sanguineus isolate WZ001 cytochrome oxidase...
Rhipicephalu... brown dog tick  34632      1184   1184  97%   0.0   99.84  664
     MG969504.1
Rhipicephalus sanguineus voucher CAS-T066 cytochrome oxidase...
Rhipicephalu... brown dog tick  34632      1179   1179  97%   0.0   99.69  644
     KF200096.1
Rhipicephalus linnaei voucher P1/22_18-4 cytochrome c oxidase...
Rhipicephalu... NA              2138177    1177   1177  100%  0.0   98.94  658
     OM984983.1
Rhipicephalus sanguineus isolate GHM1 cytochrome oxidase subun...
Rhipicephalu... brown dog tick  34632      1177   1177  96%   0.0   100.00 802
     JX416302.1
Rhipicephalus sanguineus isolate CHM1 cytochrome oxidase subun...
Rhipicephalu... brown dog tick  34632      1177   1177  96%   0.0   100.00 802
     JX416298.1
Rhipicephalus sanguineus voucher AMMS-RS-5 cytochrome c oxidas...
Rhipicephalu... brown dog tick  34632      1177   1177  97%   0.0   99.69  670
     HM193873.1
Rhipicephalus sanguineus isolate LIC6767D cytochrome oxidase...
Rhipicephalu... brown dog tick  34632      1175   1175  97%   0.0   99.69  768
     KX383820.1
Rhipicephalus sanguineus isolate LIC5766G cytochrome oxidase...
Rhipicephalu... brown dog tick  34632      1171   1171  97%   0.0   99.53  724
     KX383816.1
Rhipicephalus linnaei voucher P1/22_18-5 cytochrome c oxidase...
Rhipicephalu... NA              2138177    1171   1171  100%  0.0   98.78  658
     OM984984.1
Rhipicephalus linnaei voucher P1/22_8-1 cytochrome c oxidase...
Rhipicephalu... NA              2138177    1171   1171  100%  0.0   98.78  658
```

OM984974.1
Rhipicephalus linnaei voucher P1/22_6-2 cytochrome c oxidase...
Rhipicephalu... NA 2138177 1171 1171 100% 0.0 98.78 658
OM984973.1
Rhipicephalus sanguineus isolate SZM2 cytochrome oxidase subun...
Rhipicephalu... brown dog tick 34632 1171 1171 96% 0.0 99.84 802
JX416308.1
Rhipicephalus sanguineus isolate GTF3 cytochrome oxidase subun...
Rhipicephalu... brown dog tick 34632 1171 1171 96% 0.0 99.84 802
JX416304.1
Rhipicephalus sanguineus isolate GBM1 cytochrome oxidase subun...
Rhipicephalu... brown dog tick 34632 1171 1171 96% 0.0 99.84 802
JX416300.1
Rhipicephalus sanguineus isolate GBF1 cytochrome oxidase subun...
Rhipicephalu... brown dog tick 34632 1171 1171 96% 0.0 99.84 802
JX416299.1
Rhipicephalus sanguineus isolate CHF1 cytochrome oxidase subun...
Rhipicephalu... brown dog tick 34632 1171 1171 96% 0.0 99.84 802
JX416297.1
Rhipicephalus sanguineus isolate DGM1 cytochrome oxidase subun...
Rhipicephalu... brown dog tick 34632 1171 1171 96% 0.0 99.84 802
JX416296.1
Rhipicephalus sanguineus isolate FSF1 cytochrome oxidase subun...
Rhipicephalu... brown dog tick 34632 1171 1171 96% 0.0 99.84 802
JX416293.1
Rhipicephalus sanguineus isolate T78D2 cytochrome c oxidase...
Rhipicephalu... brown dog tick 34632 1171 1171 96% 0.0 100.00 650
MZ401439.1
Rhipicephalus sanguineus isolate LIC5762 cytochrome oxidase...
Rhipicephalu... brown dog tick 34632 1166 1166 96% 0.0 99.69 763
KX383802.1
Rhipicephalus linnaei voucher P1/22_16-1 cytochrome c oxidase...
Rhipicephalu... NA 2138177 1166 1166 100% 0.0 98.63 658
OM984979.1
Rhipicephalus linnaei voucher P1/22_1-1 cytochrome c oxidase...
Rhipicephalu... NA 2138177 1166 1166 100% 0.0 98.63 658
OM984968.1
Rhipicephalus sanguineus isolate DGF1 cytochrome oxidase subun...
Rhipicephalu... brown dog tick 34632 1166 1166 96% 0.0 99.69 802
JX416295.1
Rhipicephalus sanguineus isolate FSM1 cytochrome oxidase subun...
Rhipicephalu... brown dog tick 34632 1166 1166 96% 0.0 99.69 802
JX416294.1
Rhipicephalus sanguineus isolate sanguineus0926 cytochrome...
Rhipicephalu... brown dog tick 34632 1162 1162 99% 0.0 98.77 658
MH513252.1
Rhipicephalus linnaei voucher P1/22_19-1 cytochrome c oxidase...
Rhipicephalu... NA 2138177 1160 1160 100% 0.0 98.48 658
OM984985.1
Rhipicephalus linnaei voucher P1/22_14-1 cytochrome c oxidase...
Rhipicephalu... NA 2138177 1160 1160 100% 0.0 98.48 658
OM984978.1
Rhipicephalus linnaei voucher P1/22_13-1 cytochrome c oxidase...
Rhipicephalu... NA 2138177 1160 1160 100% 0.0 98.48 658

```
     OM984977.1
Rhipicephalus linnaei voucher P1/22_10-1 cytochrome c oxidase...
Rhipicephalu... NA              2138177    1160   1160  100%  0.0   98.48  658
     OM984975.1
Rhipicephalus linnaei voucher P1/22_5-1 cytochrome c oxidase...
Rhipicephalu... NA              2138177    1160   1160  100%  0.0   98.48  658
     OM984971.1
Rhipicephalus linnaei voucher P1/22_4-1 cytochrome c oxidase...
Rhipicephalu... NA              2138177    1160   1160  100%  0.0   98.48  658
     OM984970.1
Rhipicephalus linnaei voucher P1/22_3-1 cytochrome c oxidase...
Rhipicephalu... NA              2138177    1160   1160  100%  0.0   98.48  658
     OM984969.1
Rhipicephalus sanguineus isolate GHF1 cytochrome oxidase subun...
Rhipicephalu... brown dog tick  34632      1160   1160  96%   0.0   99.53  802
     JX416301.1
Rhipicephalus sanguineus isolate Tropical lineage RJ cytochrom...
Rhipicephalu... brown dog tick  34632      1155   1155  95%   0.0   99.68  642
     MT010523.1
Rhipicephalus linnaei voucher P1/22_21-1 cytochrome c oxidase...
Rhipicephalu... NA              2138177    1155   1155  100%  0.0   98.33  658
     OM984988.1
Rhipicephalus linnaei voucher P1/22_12-1 cytochrome c oxidase...
Rhipicephalu... NA              2138177    1155   1155  100%  0.0   98.33  658
     OM984976.1
Rhipicephalus sanguineus isolate GTF2 cytochrome oxidase subun...
Rhipicephalu... brown dog tick  34632      1155   1155  96%   0.0   99.37  802
     JX416303.1
Rhipicephalus sanguineus sensu lato 'tropical lineage' isolate...
Rhipicephalu... NA              2138177    1153   1153  98%   0.0   98.77  826
     MF426003.1
Rhipicephalus sanguineus voucher AMMS-RS-4 cytochrome c oxidas...
Rhipicephalu... brown dog tick  34632      1146   1146  96%   0.0   99.21  665
     HM193874.1
Rhipicephalus sanguineus isolate Abidjan cytochrome oxidase...
Rhipicephalu... brown dog tick  34632      1140   1140  94%   0.0   99.84  630
     KX757914.1
Rhipicephalus sanguineus isolate LIC5960 cytochrome oxidase...
Rhipicephalu... brown dog tick  34632      1136   1136  97%   0.0   98.60  768
     KX383800.1
Rhipicephalus sanguineus isolate LIC5682B cytochrome oxidase...
Rhipicephalu... brown dog tick  34632      1131   1131  93%   0.0   99.68  744
     KX383796.1
Rhipicephalus sanguineus isolate LIC5958B cytochrome oxidase...
Rhipicephalu... brown dog tick  34632      1127   1127  96%   0.0   98.59  698
     KX383798.1
Rhipicephalus sanguineus isolate LIC5584D cytochrome oxidase...
Rhipicephalu... brown dog tick  34632      1125   1125  97%   0.0   98.29  768
     KX383814.1
Rhipicephalus sanguineus isolate LIC5755 cytochrome oxidase...
Rhipicephalu... brown dog tick  34632      1122   1122  96%   0.0   98.43  763
     KX383801.1
Rhipicephalus sanguineus isolate 400 cytochrome c oxidase...
Rhipicephalu... brown dog tick  34632      1120   1120  97%   0.0   98.28  657
```

MW558150.1
Rhipicephalus linnaei isolate C2-T1 cytochrome c oxidase subun...
Rhipicephalu... NA 2138177 1116 1116 91% 0.0 100.00 604
MZ726445.1
Rhipicephalus linnaei isolate C1-T1 cytochrome c oxidase subun...
Rhipicephalu... NA 2138177 1116 1116 91% 0.0 100.00 604
MZ726444.1
Rhipicephalus linnaei isolate B15-T1 cytochrome c oxidase...
Rhipicephalu... NA 2138177 1116 1116 91% 0.0 100.00 604
MZ726443.1
Rhipicephalus linnaei isolate B12-T1 cytochrome c oxidase...
Rhipicephalu... NA 2138177 1116 1116 91% 0.0 100.00 604
MZ726442.1
Rhipicephalus linnaei isolate B8-T1 cytochrome c oxidase subun...
Rhipicephalu... NA 2138177 1116 1116 91% 0.0 100.00 604
MZ726441.1
Rhipicephalus linnaei isolate B7-T3 cytochrome c oxidase subun...
Rhipicephalu... NA 2138177 1116 1116 91% 0.0 100.00 604
MZ726440.1
Rhipicephalus linnaei isolate B7-T2 cytochrome c oxidase subun...
Rhipicephalu... NA 2138177 1116 1116 91% 0.0 100.00 604
MZ726439.1
Rhipicephalus linnaei isolate B7-T1 cytochrome c oxidase subun...
Rhipicephalu... NA 2138177 1116 1116 91% 0.0 100.00 604
MZ726438.1
Rhipicephalus linnaei isolate B6-T1 cytochrome c oxidase subun...
Rhipicephalu... NA 2138177 1116 1116 91% 0.0 100.00 604
MZ726437.1
Rhipicephalus linnaei isolate B4-T1 cytochrome c oxidase subun...
Rhipicephalu... NA 2138177 1116 1116 91% 0.0 100.00 604
MZ726436.1
Rhipicephalus linnaei isolate B3-T1 cytochrome c oxidase subun...
Rhipicephalu... NA 2138177 1116 1116 91% 0.0 100.00 604
MZ726435.1
Rhipicephalus linnaei isolate B2-T1 cytochrome c oxidase subun...
Rhipicephalu... NA 2138177 1116 1116 91% 0.0 100.00 604
MZ726434.1
Rhipicephalus linnaei isolate A7-T1 cytochrome c oxidase subun...
Rhipicephalu... NA 2138177 1116 1116 91% 0.0 100.00 604
MZ726433.1
Rhipicephalus linnaei isolate A6-T1 cytochrome c oxidase subun...
Rhipicephalu... NA 2138177 1116 1116 91% 0.0 100.00 604
MZ726432.1
Rhipicephalus linnaei isolate A5-T1 cytochrome c oxidase subun...
Rhipicephalu... NA 2138177 1116 1116 91% 0.0 100.00 604
MZ726431.1
Rhipicephalus linnaei isolate A2-T1 cytochrome c oxidase subun...
Rhipicephalu... NA 2138177 1116 1116 91% 0.0 100.00 604
MZ726430.1
Rhipicephalus linnaei isolate B9-T1 cytochrome c oxidase subun...
Rhipicephalu... NA 2138177 1110 1110 91% 0.0 99.83 604
MZ726449.1
Rhipicephalus linnaei isolate B18-T1 cytochrome c oxidase...
Rhipicephalu... NA 2138177 1110 1110 91% 0.0 99.83 604

```
     MZ726429.1
Rhipicephalus linnaei isolate B17-T1 cytochrome c oxidase...
Rhipicephalu... NA              2138177    1110   1110  91%   0.0   99.83  604
     MZ726428.1
Rhipicephalus linnaei isolate B16-T1 cytochrome c oxidase...
Rhipicephalu... NA              2138177    1110   1110  91%   0.0   99.83  604
     MZ726427.1
Rhipicephalus linnaei isolate B1-T1 cytochrome c oxidase subun...
Rhipicephalu... NA              2138177    1110   1110  91%   0.0   99.83  604
     MZ726426.1
Rhipicephalus sanguineus voucher DO-306 cytochrome oxidase...
Rhipicephalu... brown dog tick  34632      1092   1092  91%   0.0   99.33  638
     KX360338.1


Alignments:

>Rhipicephalus sanguineus sensu lato 'tropical lineage' isolate A3021 cytochrome
c oxidase subunit I (COI) gene, partial cds; mitochondrial
Sequence ID: MF425995.1 Length: 688
Range 1: 18 to 675

Score:1216 bits(658), Expect:0.0,
Identities:658/658(100%),  Gaps:0/658(0%), Strand: Plus/Plus

Query  1    GACAATATACTTAATTTTTGGAGCATGATCCGGAATACTAGGATTAAGAATAAGAATATT  60
            ||||||||||||||||||||||||||||||||||||||||||||||||||||||||||||
Sbjct  18   GACAATATACTTAATTTTTGGAGCATGATCCGGAATACTAGGATTAAGAATAAGAATATT  77

Query  61   AATCCGCATAGAATTAGGTCAACCAGGAACTTTAATTGGAAACGATCAAATTTATAATGT  120
            ||||||||||||||||||||||||||||||||||||||||||||||||||||||||||||
Sbjct  78   AATCCGCATAGAATTAGGTCAACCAGGAACTTTAATTGGAAACGATCAAATTTATAATGT  137

Query  121  AATTGTAACAGCACATGCATTTATTATAAttttttttATAGTTATACCAATTATAATCGG  180
            ||||||||||||||||||||||||||||||||||||||||||||||||||||||||||||
Sbjct  138  AATTGTAACAGCACATGCATTTATTATAATTTTTTTTATAGTTATACCAATTATAATCGG  197

Query  181  AGGATTTGGAAATTGATTAGTTCCTATTATACTAGGAGCTCCAGATATAGCATTTCCACG  240
            ||||||||||||||||||||||||||||||||||||||||||||||||||||||||||||
Sbjct  198  AGGATTTGGAAATTGATTAGTTCCTATTATACTAGGAGCTCCAGATATAGCATTTCCACG  257

Query  241  AATAAATAATATAAGATTTTGGTTACTTCCTCCTTCTCTATTTCTACTAATTAATTCTTC  300
            ||||||||||||||||||||||||||||||||||||||||||||||||||||||||||||
Sbjct  258  AATAAATAATATAAGATTTTGGTTACTTCCTCCTTCTCTATTTCTACTAATTAATTCTTC  317

Query  301  ATTAATCGAATCAGGAGCAGGTACAGGGTGAACAGTTTAcccccccTTATCATCAAATTT  360
            ||||||||||||||||||||||||||||||||||||||||||||||||||||||||||||
Sbjct  318  ATTAATCGAATCAGGAGCAGGTACAGGGTGAACAGTTTACCCCCCCTTATCATCAAATTT  377

Query  361  ATCACACTACGGACCATCAGTAGATTTAGCTATTTTTTCTCTGCATCTTGCAGGTGCTTC  420
            ||||||||||||||||||||||||||||||||||||||||||||||||||||||||||||
Sbjct  378  ATCACACTACGGACCATCAGTAGATTTAGCTATTTTTTCTCTGCATCTTGCAGGTGCTTC  437

Query  421  TTCAATTTTAGGTGCAATCAATTTCATTACAACTATTGTAAATATACGATCCATTGGAAT  480
```

```
            ||||||||||||||||||||||||||||||||||||||||||||||||||||||||||||
Sbjct  438  TTCAATTTTAGGTGCAATCAATTTCATTACAACTATTGTAAATATACGATCCATTGGAAT  497

Query  481  AACAATAGAACGAATACCATTATTTGTATGATCTGTTTTAATTACTGCAATTTTATTATT  540
            ||||||||||||||||||||||||||||||||||||||||||||||||||||||||||||
Sbjct  498  AACAATAGAACGAATACCATTATTTGTATGATCTGTTTTAATTACTGCAATTTTATTATT  557

Query  541  ATTATCTTTACCAGTCTTAGCTGGCGCAATTACAATATTATTAACTGATCGAAACTTTAA  600
            ||||||||||||||||||||||||||||||||||||||||||||||||||||||||||||
Sbjct  558  ATTATCTTTACCAGTCTTAGCTGGCGCAATTACAATATTATTAACTGATCGAAACTTTAA  617

Query  601  TACATCATTCTTTGACCCTTCAGGAGGAGGTGACCCAATTCTATATCAACATTTATTT  658
            ||||||||||||||||||||||||||||||||||||||||||||||||||||||||||
Sbjct  618  TACATCATTCTTTGACCCTTCAGGAGGAGGTGACCCAATTCTATATCAACATTTATTT  675
```

>Rhipicephalus sanguineus sensu lato 'tropical lineage' isolate A3015 cytochrome c oxidase subunit I (COI) gene, partial cds; mitochondrial
Sequence ID: MF425994.1 Length: 703
>Rhipicephalus sanguineus sensu lato 'tropical lineage' isolate A3022 cytochrome c oxidase subunit I (COI) gene, partial cds; mitochondrial
Sequence ID: MF425996.1 Length: 703
Range 1: 20 to 677

Score:1216 bits(658), Expect:0.0,
Identities:658/658(100%), Gaps:0/658(0%), Strand: Plus/Plus

```
Query  1    GACAATATACTTAATTTTTGGAGCATGATCCGGAATACTAGGATTAAGAATAAGAATATT  60
            ||||||||||||||||||||||||||||||||||||||||||||||||||||||||||||
Sbjct  20   GACAATATACTTAATTTTTGGAGCATGATCCGGAATACTAGGATTAAGAATAAGAATATT  79

Query  61   AATCCGCATAGAATTAGGTCAACCAGGAACTTTAATTGGAAACGATCAAATTTATAATGT  120
            ||||||||||||||||||||||||||||||||||||||||||||||||||||||||||||
Sbjct  80   AATCCGCATAGAATTAGGTCAACCAGGAACTTTAATTGGAAACGATCAAATTTATAATGT  139

Query  121  AATTGTAACAGCACATGCATTTATTATAAttttttttATAGTTATACCAATTATAATCGG  180
            ||||||||||||||||||||||||||||||||||||||||||||||||||||||||||||
Sbjct  140  AATTGTAACAGCACATGCATTTATTATAATTTTTTTTATAGTTATACCAATTATAATCGG  199

Query  181  AGGATTTGGAAATTGATTAGTTCCTATTATACTAGGAGCTCCAGATATAGCATTTCCACG  240
            ||||||||||||||||||||||||||||||||||||||||||||||||||||||||||||
Sbjct  200  AGGATTTGGAAATTGATTAGTTCCTATTATACTAGGAGCTCCAGATATAGCATTTCCACG  259

Query  241  AATAAATAATATAAGATTTTGGTTACTTCCTCCTTCTCTATTTCTACTAATTAATTCTTC  300
            ||||||||||||||||||||||||||||||||||||||||||||||||||||||||||||
Sbjct  260  AATAAATAATATAAGATTTTGGTTACTTCCTCCTTCTCTATTTCTACTAATTAATTCTTC  319

Query  301  ATTAATCGAATCAGGAGCAGGTACAGGGTGAACAGTTTAcccccccTTATCATCAAATTT  360
            ||||||||||||||||||||||||||||||||||||||||||||||||||||||||||||
Sbjct  320  ATTAATCGAATCAGGAGCAGGTACAGGGTGAACAGTTTACCCCCCCTTATCATCAAATTT  379

Query  361  ATCACACTACGGACCATCAGTAGATTTAGCTATTTTTTCTCTGCATCTTGCAGGTGCTTC  420
```

```
          ||||||||||||||||||||||||||||||||||||||||||||||||||||||||||||
Sbjct  380  ATCACACTACGGACCATCAGTAGATTTAGCTATTTTTTCTCTGCATCTTGCAGGTGCTTC  439

Query  421  TTCAATTTTAGGTGCAATCAATTTCATTACAACTATTGTAAATATACGATCCATTGGAAT  480
            ||||||||||||||||||||||||||||||||||||||||||||||||||||||||||||
Sbjct  440  TTCAATTTTAGGTGCAATCAATTTCATTACAACTATTGTAAATATACGATCCATTGGAAT  499

Query  481  AACAATAGAACGAATACCATTATTTGTATGATCTGTTTTAATTACTGCAATTTTATTATT  540
            ||||||||||||||||||||||||||||||||||||||||||||||||||||||||||||
Sbjct  500  AACAATAGAACGAATACCATTATTTGTATGATCTGTTTTAATTACTGCAATTTTATTATT  559

Query  541  ATTATCTTTACCAGTCTTAGCTGGCGCAATTACAATATTATTAACTGATCGAAACTTTAA  600
            ||||||||||||||||||||||||||||||||||||||||||||||||||||||||||||
Sbjct  560  ATTATCTTTACCAGTCTTAGCTGGCGCAATTACAATATTATTAACTGATCGAAACTTTAA  619

Query  601  TACATCATTCTTTGACCCTTCAGGAGGAGGTGACCCAATTCTATATCAACATTTATTT  658
            ||||||||||||||||||||||||||||||||||||||||||||||||||||||||||
Sbjct  620  TACATCATTCTTTGACCCTTCAGGAGGAGGTGACCCAATTCTATATCAACATTTATTT  677
```

>Rhipicephalus sanguineus sensu lato 'tropical lineage' isolate A3014 cytochrome c oxidase subunit I (COI) gene, partial cds; mitochondrial
Sequence ID: MF425993.1 Length: 702
Range 1: 20 to 677

Score:1216 bits(658), Expect:0.0,
Identities:658/658(100%), Gaps:0/658(0%), Strand: Plus/Plus

```
Query  1    GACAATATACTTAATTTTTGGAGCATGATCCGGAATACTAGGATTAAGAATAAGAATATT  60
            ||||||||||||||||||||||||||||||||||||||||||||||||||||||||||||
Sbjct  20   GACAATATACTTAATTTTTGGAGCATGATCCGGAATACTAGGATTAAGAATAAGAATATT  79

Query  61   AATCCGCATAGAATTAGGTCAACCAGGAACTTTAATTGGAAACGATCAAATTTATAATGT  120
            ||||||||||||||||||||||||||||||||||||||||||||||||||||||||||||
Sbjct  80   AATCCGCATAGAATTAGGTCAACCAGGAACTTTAATTGGAAACGATCAAATTTATAATGT  139

Query  121  AATTGTAACAGCACATGCATTTATTATAAttttttttATAGTTATACCAATTATAATCGG  180
            ||||||||||||||||||||||||||||||||||||||||||||||||||||||||||||
Sbjct  140  AATTGTAACAGCACATGCATTTATTATAATTTTTTTTATAGTTATACCAATTATAATCGG  199

Query  181  AGGATTTGGAAATTGATTAGTTCCTATTATACTAGGAGCTCCAGATATAGCATTTCCACG  240
            ||||||||||||||||||||||||||||||||||||||||||||||||||||||||||||
Sbjct  200  AGGATTTGGAAATTGATTAGTTCCTATTATACTAGGAGCTCCAGATATAGCATTTCCACG  259

Query  241  AATAAATAATATAAGATTTTGGTTACTTCCTCCTTCTCTATTTCTACTAATTAATTCTTC  300
            ||||||||||||||||||||||||||||||||||||||||||||||||||||||||||||
Sbjct  260  AATAAATAATATAAGATTTTGGTTACTTCCTCCTTCTCTATTTCTACTAATTAATTCTTC  319

Query  301  ATTAATCGAATCAGGAGCAGGTACAGGGTGAACAGTTTAcccccccTTATCATCAAATTT  360
            ||||||||||||||||||||||||||||||||||||||||||||||||||||||||||||
Sbjct  320  ATTAATCGAATCAGGAGCAGGTACAGGGTGAACAGTTTACCCCCCCTTATCATCAAATTT  379
```

```
Query  361   ATCACACTACGGACCATCAGTAGATTTAGCTATTTTTTCTCTGCATCTTGCAGGTGCTTC  420
             ||||||||||||||||||||||||||||||||||||||||||||||||||||||||||||
Sbjct  380   ATCACACTACGGACCATCAGTAGATTTAGCTATTTTTTCTCTGCATCTTGCAGGTGCTTC  439

Query  421   TTCAATTTTAGGTGCAATCAATTTCATTACAACTATTGTAAATATACGATCCATTGGAAT  480
             ||||||||||||||||||||||||||||||||||||||||||||||||||||||||||||
Sbjct  440   TTCAATTTTAGGTGCAATCAATTTCATTACAACTATTGTAAATATACGATCCATTGGAAT  499

Query  481   AACAATAGAACGAATACCATTATTTGTATGATCTGTTTTAATTACTGCAATTTTATTATT  540
             ||||||||||||||||||||||||||||||||||||||||||||||||||||||||||||
Sbjct  500   AACAATAGAACGAATACCATTATTTGTATGATCTGTTTTAATTACTGCAATTTTATTATT  559

Query  541   ATTATCTTTACCAGTCTTAGCTGGCGCAATTACAATATTATTAACTGATCGAAACTTTAA  600
             ||||||||||||||||||||||||||||||||||||||||||||||||||||||||||||
Sbjct  560   ATTATCTTTACCAGTCTTAGCTGGCGCAATTACAATATTATTAACTGATCGAAACTTTAA  619

Query  601   TACATCATTCTTTGACCCTTCAGGAGGAGGTGACCCAATTCTATATCAACATTTATTT  658
             ||||||||||||||||||||||||||||||||||||||||||||||||||||||||||
Sbjct  620   TACATCATTCTTTGACCCTTCAGGAGGAGGTGACCCAATTCTATATCAACATTTATTT  677
```

>Rhipicephalus sanguineus sensu lato 'tropical lineage' isolate A3013 cytochrome c oxidase subunit I (COI) gene, partial cds; mitochondrial
Sequence ID: MF425992.1 Length: 693
Range 1: 20 to 677

Score:1216 bits(658), Expect:0.0,
Identities:658/658(100%), Gaps:0/658(0%), Strand: Plus/Plus

```
Query  1     GACAATATACTTAATTTTTGGAGCATGATCCGGAATACTAGGATTAAGAATAAGAATATT  60
             ||||||||||||||||||||||||||||||||||||||||||||||||||||||||||||
Sbjct  20    GACAATATACTTAATTTTTGGAGCATGATCCGGAATACTAGGATTAAGAATAAGAATATT  79

Query  61    AATCCGCATAGAATTAGGTCAACCAGGAACTTTAATTGGAAACGATCAAATTTATAATGT  120
             ||||||||||||||||||||||||||||||||||||||||||||||||||||||||||||
Sbjct  80    AATCCGCATAGAATTAGGTCAACCAGGAACTTTAATTGGAAACGATCAAATTTATAATGT  139

Query  121   AATTGTAACAGCACATGCATTTATTATAAttttttttATAGTTATACCAATTATAATCGG  180
             ||||||||||||||||||||||||||||||||||||||||||||||||||||||||||||
Sbjct  140   AATTGTAACAGCACATGCATTTATTATAATTTTTTTTATAGTTATACCAATTATAATCGG  199

Query  181   AGGATTTGGAAATTGATTAGTTCCTATTATACTAGGAGCTCCAGATATAGCATTTCCACG  240
             ||||||||||||||||||||||||||||||||||||||||||||||||||||||||||||
Sbjct  200   AGGATTTGGAAATTGATTAGTTCCTATTATACTAGGAGCTCCAGATATAGCATTTCCACG  259

Query  241   AATAAATAATATAAGATTTTGGTTACTTCCTCCTTCTCTATTTCTACTAATTAATTCTTC  300
             ||||||||||||||||||||||||||||||||||||||||||||||||||||||||||||
Sbjct  260   AATAAATAATATAAGATTTTGGTTACTTCCTCCTTCTCTATTTCTACTAATTAATTCTTC  319

Query  301   ATTAATCGAATCAGGAGCAGGTACAGGGTGAACAGTTTAcccccccTTATCATCAAATTT  360
             ||||||||||||||||||||||||||||||||||||||||||||||||||||||||||||
Sbjct  320   ATTAATCGAATCAGGAGCAGGTACAGGGTGAACAGTTTACCCCCCCTTATCATCAAATTT  379
```

```
Query  361   ATCACACTACGGACCATCAGTAGATTTAGCTATTTTTTCTCTGCATCTTGCAGGTGCTTC  420
             ||||||||||||||||||||||||||||||||||||||||||||||||||||||||||||
Sbjct  380   ATCACACTACGGACCATCAGTAGATTTAGCTATTTTTTCTCTGCATCTTGCAGGTGCTTC  439

Query  421   TTCAATTTTAGGTGCAATCAATTTCATTACAACTATTGTAAATATACGATCCATTGGAAT  480
             ||||||||||||||||||||||||||||||||||||||||||||||||||||||||||||
Sbjct  440   TTCAATTTTAGGTGCAATCAATTTCATTACAACTATTGTAAATATACGATCCATTGGAAT  499

Query  481   AACAATAGAACGAATACCATTATTTGTATGATCTGTTTTAATTACTGCAATTTTATTATT  540
             ||||||||||||||||||||||||||||||||||||||||||||||||||||||||||||
Sbjct  500   AACAATAGAACGAATACCATTATTTGTATGATCTGTTTTAATTACTGCAATTTTATTATT  559

Query  541   ATTATCTTTACCAGTCTTAGCTGGCGCAATTACAATATTATTAACTGATCGAAACTTTAA  600
             ||||||||||||||||||||||||||||||||||||||||||||||||||||||||||||
Sbjct  560   ATTATCTTTACCAGTCTTAGCTGGCGCAATTACAATATTATTAACTGATCGAAACTTTAA  619

Query  601   TACATCATTCTTTGACCCTTCAGGAGGAGGTGACCCAATTCTATATCAACATTTATTT  658
             ||||||||||||||||||||||||||||||||||||||||||||||||||||||||||
Sbjct  620   TACATCATTCTTTGACCCTTCAGGAGGAGGTGACCCAATTCTATATCAACATTTATTT  677
```

```
>Rhipicephalus sanguineus haplotype RsanColSinSP cytochrome c oxidase subunit I
(COX1) gene, partial cds; mitochondrial
Sequence ID: KT906183.1 Length: 690
Range 1: 18 to 675

Score:1216 bits(658), Expect:0.0,
Identities:658/658(100%),  Gaps:0/658(0%), Strand: Plus/Plus

Query  1     GACAATATACTTAATTTTTGGAGCATGATCCGGAATACTAGGATTAAGAATAAGAATATT  60
             ||||||||||||||||||||||||||||||||||||||||||||||||||||||||||||
Sbjct  18    GACAATATACTTAATTTTTGGAGCATGATCCGGAATACTAGGATTAAGAATAAGAATATT  77

Query  61    AATCCGCATAGAATTAGGTCAACCAGGAACTTTAATTGGAAACGATCAAATTTATAATGT  120
             ||||||||||||||||||||||||||||||||||||||||||||||||||||||||||||
Sbjct  78    AATCCGCATAGAATTAGGTCAACCAGGAACTTTAATTGGAAACGATCAAATTTATAATGT  137

Query  121   AATTGTAACAGCACATGCATTTATTATAAttttttttATAGTTATACCAATTATAATCGG  180
             ||||||||||||||||||||||||||||||||||||||||||||||||||||||||||||
Sbjct  138   AATTGTAACAGCACATGCATTTATTATAATTTTTTTTATAGTTATACCAATTATAATCGG  197

Query  181   AGGATTTGGAAATTGATTAGTTCCTATTATACTAGGAGCTCCAGATATAGCATTTCCACG  240
             ||||||||||||||||||||||||||||||||||||||||||||||||||||||||||||
Sbjct  198   AGGATTTGGAAATTGATTAGTTCCTATTATACTAGGAGCTCCAGATATAGCATTTCCACG  257

Query  241   AATAAATAATATAAGATTTTGGTTACTTCCTCCTTCTCTATTTCTACTAATTAATTCTTC  300
             ||||||||||||||||||||||||||||||||||||||||||||||||||||||||||||
Sbjct  258   AATAAATAATATAAGATTTTGGTTACTTCCTCCTTCTCTATTTCTACTAATTAATTCTTC  317

Query  301   ATTAATCGAATCAGGAGCAGGTACAGGGTGAACAGTTTAcccccccTTATCATCAAATTT  360
             ||||||||||||||||||||||||||||||||||||||||||||||||||||||||||||
```

```
Sbjct  318   ATTAATCGAATCAGGAGCAGGTACAGGGTGAACAGTTTACCCCCCCTTATCATCAAATTT  377

Query  361   ATCACACTACGGACCATCAGTAGATTTAGCTATTTTTTCTCTGCATCTTGCAGGTGCTTC  420
             ||||||||||||||||||||||||||||||||||||||||||||||||||||||||||||
Sbjct  378   ATCACACTACGGACCATCAGTAGATTTAGCTATTTTTTCTCTGCATCTTGCAGGTGCTTC  437

Query  421   TTCAATTTTAGGTGCAATCAATTTCATTACAACTATTGTAAATATACGATCCATTGGAAT  480
             ||||||||||||||||||||||||||||||||||||||||||||||||||||||||||||
Sbjct  438   TTCAATTTTAGGTGCAATCAATTTCATTACAACTATTGTAAATATACGATCCATTGGAAT  497

Query  481   AACAATAGAACGAATACCATTATTTGTATGATCTGTTTTAATTACTGCAATTTTATTATT  540
             ||||||||||||||||||||||||||||||||||||||||||||||||||||||||||||
Sbjct  498   AACAATAGAACGAATACCATTATTTGTATGATCTGTTTTAATTACTGCAATTTTATTATT  557

Query  541   ATTATCTTTACCAGTCTTAGCTGGCGCAATTACAATATTATTAACTGATCGAAACTTTAA  600
             ||||||||||||||||||||||||||||||||||||||||||||||||||||||||||||
Sbjct  558   ATTATCTTTACCAGTCTTAGCTGGCGCAATTACAATATTATTAACTGATCGAAACTTTAA  617

Query  601   TACATCATTCTTTGACCCTTCAGGAGGAGGTGACCCAATTCTATATCAACATTTATTT  658
             ||||||||||||||||||||||||||||||||||||||||||||||||||||||||||
Sbjct  618   TACATCATTCTTTGACCCTTCAGGAGGAGGTGACCCAATTCTATATCAACATTTATTT  675
```

>Rhipicephalus sanguineus haplotype RsanColSanM cytochrome c oxidase subunit I (COX1) gene, partial cds; mitochondrial
Sequence ID: KT906182.1 Length: 690
Range 1: 18 to 675

Score:1216 bits(658), Expect:0.0,
Identities:658/658(100%), Gaps:0/658(0%), Strand: Plus/Plus

```
Query  1     GACAATATACTTAATTTTTGGAGCATGATCCGGAATACTAGGATTAAGAATAAGAATATT  60
             ||||||||||||||||||||||||||||||||||||||||||||||||||||||||||||
Sbjct  18    GACAATATACTTAATTTTTGGAGCATGATCCGGAATACTAGGATTAAGAATAAGAATATT  77

Query  61    AATCCGCATAGAATTAGGTCAACCAGGAACTTTAATTGGAAACGATCAAATTTATAATGT  120
             ||||||||||||||||||||||||||||||||||||||||||||||||||||||||||||
Sbjct  78    AATCCGCATAGAATTAGGTCAACCAGGAACTTTAATTGGAAACGATCAAATTTATAATGT  137

Query  121   AATTGTAACAGCACATGCATTTATTATAAttttttttATAGTTATACCAATTATAATCGG  180
             ||||||||||||||||||||||||||||||||||||||||||||||||||||||||||||
Sbjct  138   AATTGTAACAGCACATGCATTTATTATAATTTTTTTTATAGTTATACCAATTATAATCGG  197

Query  181   AGGATTTGGAAATTGATTAGTTCCTATTATACTAGGAGCTCCAGATATAGCATTTCCACG  240
             ||||||||||||||||||||||||||||||||||||||||||||||||||||||||||||
Sbjct  198   AGGATTTGGAAATTGATTAGTTCCTATTATACTAGGAGCTCCAGATATAGCATTTCCACG  257

Query  241   AATAAATAATATAAGATTTTGGTTACTTCCTCCTTCTCTATTTCTACTAATTAATTCTTC  300
             ||||||||||||||||||||||||||||||||||||||||||||||||||||||||||||
Sbjct  258   AATAAATAATATAAGATTTTGGTTACTTCCTCCTTCTCTATTTCTACTAATTAATTCTTC  317

Query  301   ATTAATCGAATCAGGAGCAGGTACAGGGTGAACAGTTTAcccccccTTATCATCAAATTT  360
```

```
            ||||||||||||||||||||||||||||||||||||||||||||||||||||||||||||
Sbjct  318  ATTAATCGAATCAGGAGCAGGTACAGGGTGAACAGTTTACCCCCCCTTATCATCAAATTT  377

Query  361  ATCACACTACGGACCATCAGTAGATTTAGCTATTTTTTCTCTGCATCTTGCAGGTGCTTC  420
            ||||||||||||||||||||||||||||||||||||||||||||||||||||||||||||
Sbjct  378  ATCACACTACGGACCATCAGTAGATTTAGCTATTTTTTCTCTGCATCTTGCAGGTGCTTC  437

Query  421  TTCAATTTTAGGTGCAATCAATTTCATTACAACTATTGTAAATATACGATCCATTGGAAT  480
            ||||||||||||||||||||||||||||||||||||||||||||||||||||||||||||
Sbjct  438  TTCAATTTTAGGTGCAATCAATTTCATTACAACTATTGTAAATATACGATCCATTGGAAT  497

Query  481  AACAATAGAACGAATACCATTATTTGTATGATCTGTTTTAATTACTGCAATTTTATTATT  540
            ||||||||||||||||||||||||||||||||||||||||||||||||||||||||||||
Sbjct  498  AACAATAGAACGAATACCATTATTTGTATGATCTGTTTTAATTACTGCAATTTTATTATT  557

Query  541  ATTATCTTTACCAGTCTTAGCTGGCGCAATTACAATATTATTAACTGATCGAAACTTTAA  600
            ||||||||||||||||||||||||||||||||||||||||||||||||||||||||||||
Sbjct  558  ATTATCTTTACCAGTCTTAGCTGGCGCAATTACAATATTATTAACTGATCGAAACTTTAA  617

Query  601  TACATCATTCTTTGACCCTTCAGGAGGAGGTGACCCAATTCTATATCAACATTTATTT  658
            ||||||||||||||||||||||||||||||||||||||||||||||||||||||||||
Sbjct  618  TACATCATTCTTTGACCCTTCAGGAGGAGGTGACCCAATTCTATATCAACATTTATTT  675
```

```
&gt;Rhipicephalus sanguineus isolate Q3 mitochondrion, complete genome
Sequence ID: OM368327.1 Length: 14711
Range 1: 1185 to 1842

Score:1216 bits(658), Expect:0.0,
Identities:658/658(100%),  Gaps:0/658(0%), Strand: Plus/Plus

Query  1     GACAATATACTTAATTTTTGGAGCATGATCCGGAATACTAGGATTAAGAATAAGAATATT  60
             ||||||||||||||||||||||||||||||||||||||||||||||||||||||||||||
Sbjct  1185  GACAATATACTTAATTTTTGGAGCATGATCCGGAATACTAGGATTAAGAATAAGAATATT  1244

Query  61    AATCCGCATAGAATTAGGTCAACCAGGAACTTTAATTGGAAACGATCAAATTTATAATGT  120
             ||||||||||||||||||||||||||||||||||||||||||||||||||||||||||||
Sbjct  1245  AATCCGCATAGAATTAGGTCAACCAGGAACTTTAATTGGAAACGATCAAATTTATAATGT  1304

Query  121   AATTGTAACAGCACATGCATTTATTATAAttttttttATAGTTATACCAATTATAATCGG  180
             ||||||||||||||||||||||||||||||||||||||||||||||||||||||||||||
Sbjct  1305  AATTGTAACAGCACATGCATTTATTATAATTTTTTTTATAGTTATACCAATTATAATCGG  1364

Query  181   AGGATTTGGAAATTGATTAGTTCCTATTATACTAGGAGCTCCAGATATAGCATTTCCACG  240
             ||||||||||||||||||||||||||||||||||||||||||||||||||||||||||||
Sbjct  1365  AGGATTTGGAAATTGATTAGTTCCTATTATACTAGGAGCTCCAGATATAGCATTTCCACG  1424

Query  241   AATAAATAATATAAGATTTTGGTTACTTCCTCCTTCTCTATTTCTACTAATTAATTCTTC  300
             ||||||||||||||||||||||||||||||||||||||||||||||||||||||||||||
Sbjct  1425  AATAAATAATATAAGATTTTGGTTACTTCCTCCTTCTCTATTTCTACTAATTAATTCTTC  1484

Query  301   ATTAATCGAATCAGGAGCAGGTACAGGGTGAACAGTTTAcccccccTTATCATCAAATTT  360
```

```
               ||||||||||||||||||||||||||||||||||||||||||||||||||||||||||||
Sbjct  1485    ATTAATCGAATCAGGAGCAGGTACAGGGTGAACAGTTTACCCCCCCTTATCATCAAATTT  1544

Query  361     ATCACACTACGGACCATCAGTAGATTTAGCTATTTTTTCTCTGCATCTTGCAGGTGCTTC  420
               ||||||||||||||||||||||||||||||||||||||||||||||||||||||||||||
Sbjct  1545    ATCACACTACGGACCATCAGTAGATTTAGCTATTTTTTCTCTGCATCTTGCAGGTGCTTC  1604

Query  421     TTCAATTTTAGGTGCAATCAATTTCATTACAACTATTGTAAATATACGATCCATTGGAAT  480
               ||||||||||||||||||||||||||||||||||||||||||||||||||||||||||||
Sbjct  1605    TTCAATTTTAGGTGCAATCAATTTCATTACAACTATTGTAAATATACGATCCATTGGAAT  1664

Query  481     AACAATAGAACGAATACCATTATTTGTATGATCTGTTTTAATTACTGCAATTTTATTATT  540
               ||||||||||||||||||||||||||||||||||||||||||||||||||||||||||||
Sbjct  1665    AACAATAGAACGAATACCATTATTTGTATGATCTGTTTTAATTACTGCAATTTTATTATT  1724

Query  541     ATTATCTTTACCAGTCTTAGCTGGCGCAATTACAATATTATTAACTGATCGAAACTTTAA  600
               ||||||||||||||||||||||||||||||||||||||||||||||||||||||||||||
Sbjct  1725    ATTATCTTTACCAGTCTTAGCTGGCGCAATTACAATATTATTAACTGATCGAAACTTTAA  1784

Query  601     TACATCATTCTTTGACCCTTCAGGAGGAGGTGACCCAATTCTATATCAACATTTATTT  658
               ||||||||||||||||||||||||||||||||||||||||||||||||||||||||||
Sbjct  1785    TACATCATTCTTTGACCCTTCAGGAGGAGGTGACCCAATTCTATATCAACATTTATTT  1842
```

>Rhipicephalus sanguineus isolate C19 mitochondrion, complete genome
Sequence ID: OM368323.1 Length: 14714
Range 1: 1185 to 1842

Score:1216 bits(658), Expect:0.0,
Identities:658/658(100%), Gaps:0/658(0%), Strand: Plus/Plus

```
Query  1       GACAATATACTTAATTTTTGGAGCATGATCCGGAATACTAGGATTAAGAATAAGAATATT  60
               ||||||||||||||||||||||||||||||||||||||||||||||||||||||||||||
Sbjct  1185    GACAATATACTTAATTTTTGGAGCATGATCCGGAATACTAGGATTAAGAATAAGAATATT  1244

Query  61      AATCCGCATAGAATTAGGTCAACCAGGAACTTTAATTGGAAACGATCAAATTTATAATGT  120
               ||||||||||||||||||||||||||||||||||||||||||||||||||||||||||||
Sbjct  1245    AATCCGCATAGAATTAGGTCAACCAGGAACTTTAATTGGAAACGATCAAATTTATAATGT  1304

Query  121     AATTGTAACAGCACATGCATTTATTATAAttttttttATAGTTATACCAATTATAATCGG  180
               ||||||||||||||||||||||||||||||||||||||||||||||||||||||||||||
Sbjct  1305    AATTGTAACAGCACATGCATTTATTATAATTTTTTTTATAGTTATACCAATTATAATCGG  1364

Query  181     AGGATTTGGAAATTGATTAGTTCCTATTATACTAGGAGCTCCAGATATAGCATTTCCACG  240
               ||||||||||||||||||||||||||||||||||||||||||||||||||||||||||||
Sbjct  1365    AGGATTTGGAAATTGATTAGTTCCTATTATACTAGGAGCTCCAGATATAGCATTTCCACG  1424

Query  241     AATAAATAATATAAGATTTTGGTTACTTCCTCCTTCTCTATTTCTACTAATTAATTCTTC  300
               ||||||||||||||||||||||||||||||||||||||||||||||||||||||||||||
Sbjct  1425    AATAAATAATATAAGATTTTGGTTACTTCCTCCTTCTCTATTTCTACTAATTAATTCTTC  1484

Query  301     ATTAATCGAATCAGGAGCAGGTACAGGGTGAACAGTTTAcccccccTTATCATCAAATTT  360
```

```
                ||||||||||||||||||||||||||||||||||||||||||||||||||||||||||||
Sbjct  1485   ATTAATCGAATCAGGAGCAGGTACAGGGTGAACAGTTTACCCCCCCTTATCATCAAATTT  1544

Query  361    ATCACACTACGGACCATCAGTAGATTTAGCTATTTTTTCTCTGCATCTTGCAGGTGCTTC  420
              ||||||||||||||||||||||||||||||||||||||||||||||||||||||||||||
Sbjct  1545   ATCACACTACGGACCATCAGTAGATTTAGCTATTTTTTCTCTGCATCTTGCAGGTGCTTC  1604

Query  421    TTCAATTTTAGGTGCAATCAATTTCATTACAACTATTGTAAATATACGATCCATTGGAAT  480
              ||||||||||||||||||||||||||||||||||||||||||||||||||||||||||||
Sbjct  1605   TTCAATTTTAGGTGCAATCAATTTCATTACAACTATTGTAAATATACGATCCATTGGAAT  1664

Query  481    AACAATAGAACGAATACCATTATTTGTATGATCTGTTTTAATTACTGCAATTTTATTATT  540
              ||||||||||||||||||||||||||||||||||||||||||||||||||||||||||||
Sbjct  1665   AACAATAGAACGAATACCATTATTTGTATGATCTGTTTTAATTACTGCAATTTTATTATT  1724

Query  541    ATTATCTTTACCAGTCTTAGCTGGCGCAATTACAATATTATTAACTGATCGAAACTTTAA  600
              ||||||||||||||||||||||||||||||||||||||||||||||||||||||||||||
Sbjct  1725   ATTATCTTTACCAGTCTTAGCTGGCGCAATTACAATATTATTAACTGATCGAAACTTTAA  1784

Query  601    TACATCATTCTTTGACCCTTCAGGAGGAGGTGACCCAATTCTATATCAACATTTATTT  658
              ||||||||||||||||||||||||||||||||||||||||||||||||||||||||||
Sbjct  1785   TACATCATTCTTTGACCCTTCAGGAGGAGGTGACCCAATTCTATATCAACATTTATTT  1842



>Rhipicephalus sanguineus isolate A44 mitochondrion, complete genome
Sequence ID: OM368322.1 Length: 14713
Range 1: 1185 to 1842

Score:1216 bits(658), Expect:0.0,
Identities:658/658(100%),  Gaps:0/658(0%), Strand: Plus/Plus

Query  1      GACAATATACTTAATTTTTGGAGCATGATCCGGAATACTAGGATTAAGAATAAGAATATT  60
              ||||||||||||||||||||||||||||||||||||||||||||||||||||||||||||
Sbjct  1185   GACAATATACTTAATTTTTGGAGCATGATCCGGAATACTAGGATTAAGAATAAGAATATT  1244

Query  61     AATCCGCATAGAATTAGGTCAACCAGGAACTTTAATTGGAAACGATCAAATTTATAATGT  120
              ||||||||||||||||||||||||||||||||||||||||||||||||||||||||||||
Sbjct  1245   AATCCGCATAGAATTAGGTCAACCAGGAACTTTAATTGGAAACGATCAAATTTATAATGT  1304

Query  121    AATTGTAACAGCACATGCATTTATTATAAtttttttATAGTTATACCAATTATAATCGG  180
              ||||||||||||||||||||||||||||||||||||||||||||||||||||||||||||
Sbjct  1305   AATTGTAACAGCACATGCATTTATTATAATTTTTTTATAGTTATACCAATTATAATCGG  1364

Query  181    AGGATTTGGAAATTGATTAGTTCCTATTATACTAGGAGCTCCAGATATAGCATTTCCACG  240
              ||||||||||||||||||||||||||||||||||||||||||||||||||||||||||||
Sbjct  1365   AGGATTTGGAAATTGATTAGTTCCTATTATACTAGGAGCTCCAGATATAGCATTTCCACG  1424

Query  241    AATAAATAATATAAGATTTTGGTTACTTCCTCCTTCTCTATTTCTACTAATTAATTCTTC  300
              ||||||||||||||||||||||||||||||||||||||||||||||||||||||||||||
Sbjct  1425   AATAAATAATATAAGATTTTGGTTACTTCCTCCTTCTCTATTTCTACTAATTAATTCTTC  1484

Query  301    ATTAATCGAATCAGGAGCAGGTACAGGGTGAACAGTTTAcccccccTTATCATCAAATTT  360
```

```
             ||||||||||||||||||||||||||||||||||||||||||||||||||||||||||||
Sbjct  1485  ATTAATCGAATCAGGAGCAGGTACAGGGTGAACAGTTTACCCCCCCTTATCATCAAATTT  1544

Query  361   ATCACACTACGGACCATCAGTAGATTTAGCTATTTTTTCTCTGCATCTTGCAGGTGCTTC  420
             ||||||||||||||||||||||||||||||||||||||||||||||||||||||||||||
Sbjct  1545  ATCACACTACGGACCATCAGTAGATTTAGCTATTTTTTCTCTGCATCTTGCAGGTGCTTC  1604

Query  421   TTCAATTTTAGGTGCAATCAATTTCATTACAACTATTGTAAATATACGATCCATTGGAAT  480
             ||||||||||||||||||||||||||||||||||||||||||||||||||||||||||||
Sbjct  1605  TTCAATTTTAGGTGCAATCAATTTCATTACAACTATTGTAAATATACGATCCATTGGAAT  1664

Query  481   AACAATAGAACGAATACCATTATTTGTATGATCTGTTTTAATTACTGCAATTTTATTATT  540
             ||||||||||||||||||||||||||||||||||||||||||||||||||||||||||||
Sbjct  1665  AACAATAGAACGAATACCATTATTTGTATGATCTGTTTTAATTACTGCAATTTTATTATT  1724

Query  541   ATTATCTTTACCAGTCTTAGCTGGCGCAATTACAATATTATTAACTGATCGAAACTTTAA  600
             ||||||||||||||||||||||||||||||||||||||||||||||||||||||||||||
Sbjct  1725  ATTATCTTTACCAGTCTTAGCTGGCGCAATTACAATATTATTAACTGATCGAAACTTTAA  1784

Query  601   TACATCATTCTTTGACCCTTCAGGAGGAGGTGACCCAATTCTATATCAACATTTATTT  658
             ||||||||||||||||||||||||||||||||||||||||||||||||||||||||||
Sbjct  1785  TACATCATTCTTTGACCCTTCAGGAGGAGGTGACCCAATTCTATATCAACATTTATTT  1842
```

>Rhipicephalus sanguineus isolate Guangxi cytochrome oxidase subunit I (COI) gene, partial cds; mitochondrial
Sequence ID: JQ737084.1 Length: 707
Range 1: 24 to 681

Score:1216 bits(658), Expect:0.0,
Identities:658/658(100%), Gaps:0/658(0%), Strand: Plus/Plus

```
Query  1    GACAATATACTTAATTTTTGGAGCATGATCCGGAATACTAGGATTAAGAATAAGAATATT  60
            ||||||||||||||||||||||||||||||||||||||||||||||||||||||||||||
Sbjct  24   GACAATATACTTAATTTTTGGAGCATGATCCGGAATACTAGGATTAAGAATAAGAATATT  83

Query  61   AATCCGCATAGAATTAGGTCAACCAGGAACTTTAATTGGAAACGATCAAATTTATAATGT  120
            ||||||||||||||||||||||||||||||||||||||||||||||||||||||||||||
Sbjct  84   AATCCGCATAGAATTAGGTCAACCAGGAACTTTAATTGGAAACGATCAAATTTATAATGT  143

Query  121  AATTGTAACAGCACATGCATTTATTATAAttttttttATAGTTATACCAATTATAATCGG  180
            ||||||||||||||||||||||||||||||||||||||||||||||||||||||||||||
Sbjct  144  AATTGTAACAGCACATGCATTTATTATAATTTTTTTTATAGTTATACCAATTATAATCGG  203

Query  181  AGGATTTGGAAATTGATTAGTTCCTATTATACTAGGAGCTCCAGATATAGCATTTCCACG  240
            ||||||||||||||||||||||||||||||||||||||||||||||||||||||||||||
Sbjct  204  AGGATTTGGAAATTGATTAGTTCCTATTATACTAGGAGCTCCAGATATAGCATTTCCACG  263

Query  241  AATAAATAATATAAGATTTTGGTTACTTCCTCCTTCTCTATTTCTACTAATTAATTCTTC  300
            ||||||||||||||||||||||||||||||||||||||||||||||||||||||||||||
Sbjct  264  AATAAATAATATAAGATTTTGGTTACTTCCTCCTTCTCTATTTCTACTAATTAATTCTTC  323
```

```
Query  301   ATTAATCGAATCAGGAGCAGGTACAGGGTGAACAGTTTAcccccccTTATCATCAAATTT  360
             ||||||||||||||||||||||||||||||||||||||||||||||||||||||||||||
Sbjct  324   ATTAATCGAATCAGGAGCAGGTACAGGGTGAACAGTTTACCCCCCCTTATCATCAAATTT  383

Query  361   ATCACACTACGGACCATCAGTAGATTTAGCTATTTTTTCTCTGCATCTTGCAGGTGCTTC  420
             ||||||||||||||||||||||||||||||||||||||||||||||||||||||||||||
Sbjct  384   ATCACACTACGGACCATCAGTAGATTTAGCTATTTTTTCTCTGCATCTTGCAGGTGCTTC  443

Query  421   TTCAATTTTAGGTGCAATCAATTTCATTACAACTATTGTAAATATACGATCCATTGGAAT  480
             ||||||||||||||||||||||||||||||||||||||||||||||||||||||||||||
Sbjct  444   TTCAATTTTAGGTGCAATCAATTTCATTACAACTATTGTAAATATACGATCCATTGGAAT  503

Query  481   AACAATAGAACGAATACCATTATTTGTATGATCTGTTTTAATTACTGCAATTTTATTATT  540
             ||||||||||||||||||||||||||||||||||||||||||||||||||||||||||||
Sbjct  504   AACAATAGAACGAATACCATTATTTGTATGATCTGTTTTAATTACTGCAATTTTATTATT  563

Query  541   ATTATCTTTACCAGTCTTAGCTGGCGCAATTACAATATTATTAACTGATCGAAACTTTAA  600
             ||||||||||||||||||||||||||||||||||||||||||||||||||||||||||||
Sbjct  564   ATTATCTTTACCAGTCTTAGCTGGCGCAATTACAATATTATTAACTGATCGAAACTTTAA  623

Query  601   TACATCATTCTTTGACCCTTCAGGAGGAGGTGACCCAATTCTATATCAACATTTATTT  658
             ||||||||||||||||||||||||||||||||||||||||||||||||||||||||||
Sbjct  624   TACATCATTCTTTGACCCTTCAGGAGGAGGTGACCCAATTCTATATCAACATTTATTT  681
```

>Rhipicephalus sanguineus cytochrome oxidase subunit 1 (COI) gene, partial cds; mitochondrial
Sequence ID: KF437543.1 Length: 658
>Ixodidae sp. sc_00964 cytochrome oxidase subunit 1 (COI) gene, partial cds; mitochondrial
Sequence ID: KX053539.1 Length: 658
Range 1: 1 to 658

Score:1216 bits(658), Expect:0.0,
Identities:658/658(100%), Gaps:0/658(0%), Strand: Plus/Plus

```
Query  1     GACAATATACTTAATTTTTGGAGCATGATCCGGAATACTAGGATTAAGAATAAGAATATT  60
             ||||||||||||||||||||||||||||||||||||||||||||||||||||||||||||
Sbjct  1     GACAATATACTTAATTTTTGGAGCATGATCCGGAATACTAGGATTAAGAATAAGAATATT  60

Query  61    AATCCGCATAGAATTAGGTCAACCAGGAACTTTAATTGGAAACGATCAAATTTATAATGT  120
             ||||||||||||||||||||||||||||||||||||||||||||||||||||||||||||
Sbjct  61    AATCCGCATAGAATTAGGTCAACCAGGAACTTTAATTGGAAACGATCAAATTTATAATGT  120

Query  121   AATTGTAACAGCACATGCATTTATTATAAttttttttATAGTTATACCAATTATAATCGG  180
             ||||||||||||||||||||||||||||||||||||||||||||||||||||||||||||
Sbjct  121   AATTGTAACAGCACATGCATTTATTATAATTTTTTTTATAGTTATACCAATTATAATCGG  180

Query  181   AGGATTTGGAAATTGATTAGTTCCTATTATACTAGGAGCTCCAGATATAGCATTTCCACG  240
             ||||||||||||||||||||||||||||||||||||||||||||||||||||||||||||
Sbjct  181   AGGATTTGGAAATTGATTAGTTCCTATTATACTAGGAGCTCCAGATATAGCATTTCCACG  240
```

```
Query  241  AATAAATAATATAAGATTTTGGTTACTTCCTCCTTCTCTATTTCTACTAATTAATTCTTC  300
            ||||||||||||||||||||||||||||||||||||||||||||||||||||||||||||
Sbjct  241  AATAAATAATATAAGATTTTGGTTACTTCCTCCTTCTCTATTTCTACTAATTAATTCTTC  300

Query  301  ATTAATCGAATCAGGAGCAGGTACAGGGTGAACAGTTTAcccccccTTATCATCAAATTT  360
            ||||||||||||||||||||||||||||||||||||||||||||||||||||||||||||
Sbjct  301  ATTAATCGAATCAGGAGCAGGTACAGGGTGAACAGTTTACCCCCCCTTATCATCAAATTT  360

Query  361  ATCACACTACGGACCATCAGTAGATTTAGCTATTTTTTCTCTGCATCTTGCAGGTGCTTC  420
            ||||||||||||||||||||||||||||||||||||||||||||||||||||||||||||
Sbjct  361  ATCACACTACGGACCATCAGTAGATTTAGCTATTTTTTCTCTGCATCTTGCAGGTGCTTC  420

Query  421  TTCAATTTTAGGTGCAATCAATTTCATTACAACTATTGTAAATATACGATCCATTGGAAT  480
            ||||||||||||||||||||||||||||||||||||||||||||||||||||||||||||
Sbjct  421  TTCAATTTTAGGTGCAATCAATTTCATTACAACTATTGTAAATATACGATCCATTGGAAT  480

Query  481  AACAATAGAACGAATACCATTATTTGTATGATCTGTTTTAATTACTGCAATTTTATTATT  540
            ||||||||||||||||||||||||||||||||||||||||||||||||||||||||||||
Sbjct  481  AACAATAGAACGAATACCATTATTTGTATGATCTGTTTTAATTACTGCAATTTTATTATT  540

Query  541  ATTATCTTTACCAGTCTTAGCTGGCGCAATTACAATATTATTAACTGATCGAAACTTTAA  600
            ||||||||||||||||||||||||||||||||||||||||||||||||||||||||||||
Sbjct  541  ATTATCTTTACCAGTCTTAGCTGGCGCAATTACAATATTATTAACTGATCGAAACTTTAA  600

Query  601  TACATCATTCTTTGACCCTTCAGGAGGAGGTGACCCAATTCTATATCAACATTTATTT  658
            ||||||||||||||||||||||||||||||||||||||||||||||||||||||||||
Sbjct  601  TACATCATTCTTTGACCCTTCAGGAGGAGGTGACCCAATTCTATATCAACATTTATTT  658
```

>Rhipicephalus linnaei isolate LAO32-2 clone JS5930 mitochondrion, complete genome
Sequence ID: MW429383.1 Length: 14715
Range 1: 45 to 702

Score:1216 bits(658), Expect:0.0,
Identities:658/658(100%), Gaps:0/658(0%), Strand: Plus/Plus

```
Query  1    GACAATATACTTAATTTTTGGAGCATGATCCGGAATACTAGGATTAAGAATAAGAATATT  60
            ||||||||||||||||||||||||||||||||||||||||||||||||||||||||||||
Sbjct  45   GACAATATACTTAATTTTTGGAGCATGATCCGGAATACTAGGATTAAGAATAAGAATATT  104

Query  61   AATCCGCATAGAATTAGGTCAACCAGGAACTTTAATTGGAAACGATCAAATTTATAATGT  120
            ||||||||||||||||||||||||||||||||||||||||||||||||||||||||||||
Sbjct  105  AATCCGCATAGAATTAGGTCAACCAGGAACTTTAATTGGAAACGATCAAATTTATAATGT  164

Query  121  AATTGTAACAGCACATGCATTTATTATAAttttttttATAGTTATACCAATTATAATCGG  180
            ||||||||||||||||||||||||||||||||||||||||||||||||||||||||||||
Sbjct  165  AATTGTAACAGCACATGCATTTATTATAATTTTTTTTATAGTTATACCAATTATAATCGG  224

Query  181  AGGATTTGGAAATTGATTAGTTCCTATTATACTAGGAGCTCCAGATATAGCATTTCCACG  240
            ||||||||||||||||||||||||||||||||||||||||||||||||||||||||||||
Sbjct  225  AGGATTTGGAAATTGATTAGTTCCTATTATACTAGGAGCTCCAGATATAGCATTTCCACG  284
```

```
Query  241   AATAAATAATATAAGATTTTGGTTACTTCCTCCTTCTCTATTTCTACTAATTAATTCTTC  300
             ||||||||||||||||||||||||||||||||||||||||||||||||||||||||||||
Sbjct  285   AATAAATAATATAAGATTTTGGTTACTTCCTCCTTCTCTATTTCTACTAATTAATTCTTC  344

Query  301   ATTAATCGAATCAGGAGCAGGTACAGGGTGAACAGTTTAcccccccTTATCATCAAATTT  360
             ||||||||||||||||||||||||||||||||||||||||||||||||||||||||||||
Sbjct  345   ATTAATCGAATCAGGAGCAGGTACAGGGTGAACAGTTTACCCCCCCTTATCATCAAATTT  404

Query  361   ATCACACTACGGACCATCAGTAGATTTAGCTATTTTTTCTCTGCATCTTGCAGGTGCTTC  420
             ||||||||||||||||||||||||||||||||||||||||||||||||||||||||||||
Sbjct  405   ATCACACTACGGACCATCAGTAGATTTAGCTATTTTTTCTCTGCATCTTGCAGGTGCTTC  464

Query  421   TTCAATTTTAGGTGCAATCAATTTCATTACAACTATTGTAAATATACGATCCATTGGAAT  480
             ||||||||||||||||||||||||||||||||||||||||||||||||||||||||||||
Sbjct  465   TTCAATTTTAGGTGCAATCAATTTCATTACAACTATTGTAAATATACGATCCATTGGAAT  524

Query  481   AACAATAGAACGAATACCATTATTTGTATGATCTGTTTTAATTACTGCAATTTTATTATT  540
             ||||||||||||||||||||||||||||||||||||||||||||||||||||||||||||
Sbjct  525   AACAATAGAACGAATACCATTATTTGTATGATCTGTTTTAATTACTGCAATTTTATTATT  584

Query  541   ATTATCTTTACCAGTCTTAGCTGGCGCAATTACAATATTATTAACTGATCGAAACTTTAA  600
             ||||||||||||||||||||||||||||||||||||||||||||||||||||||||||||
Sbjct  585   ATTATCTTTACCAGTCTTAGCTGGCGCAATTACAATATTATTAACTGATCGAAACTTTAA  644

Query  601   TACATCATTCTTTGACCCTTCAGGAGGAGGTGACCCAATTCTATATCAACATTTATTT  658
             ||||||||||||||||||||||||||||||||||||||||||||||||||||||||||
Sbjct  645   TACATCATTCTTTGACCCTTCAGGAGGAGGTGACCCAATTCTATATCAACATTTATTT  702
```

>Rhipicephalus linnaei mitochondrion, complete sequence
Sequence ID: NC_060409.1 Length: 14711
>Rhipicephalus linnaei strain Coominya isolate AUS3 clone JS5929 mitochondrion, complete genome
Sequence ID: MW429381.1 Length: 14711
Range 1: 45 to 702

Score:1216 bits(658), Expect:0.0,
Identities:658/658(100%), Gaps:0/658(0%), Strand: Plus/Plus

```
Query  1     GACAATATACTTAATTTTTGGAGCATGATCCGGAATACTAGGATTAAGAATAAGAATATT  60
             ||||||||||||||||||||||||||||||||||||||||||||||||||||||||||||
Sbjct  45    GACAATATACTTAATTTTTGGAGCATGATCCGGAATACTAGGATTAAGAATAAGAATATT  104

Query  61    AATCCGCATAGAATTAGGTCAACCAGGAACTTTAATTGGAAACGATCAAATTTATAATGT  120
             ||||||||||||||||||||||||||||||||||||||||||||||||||||||||||||
Sbjct  105   AATCCGCATAGAATTAGGTCAACCAGGAACTTTAATTGGAAACGATCAAATTTATAATGT  164

Query  121   AATTGTAACAGCACATGCATTTATTATAAttttttttATAGTTATACCAATTATAATCGG  180
             ||||||||||||||||||||||||||||||||||||||||||||||||||||||||||||
Sbjct  165   AATTGTAACAGCACATGCATTTATTATAATTTTTTTTATAGTTATACCAATTATAATCGG  224
```

```
Query  181  AGGATTTGGAAATTGATTAGTTCCTATTATACTAGGAGCTCCAGATATAGCATTTCCACG  240
            ||||||||||||||||||||||||||||||||||||||||||||||||||||||||||||
Sbjct  225  AGGATTTGGAAATTGATTAGTTCCTATTATACTAGGAGCTCCAGATATAGCATTTCCACG  284

Query  241  AATAAATAATATAAGATTTTGGTTACTTCCTCCTTCTCTATTTCTACTAATTAATTCTTC  300
            ||||||||||||||||||||||||||||||||||||||||||||||||||||||||||||
Sbjct  285  AATAAATAATATAAGATTTTGGTTACTTCCTCCTTCTCTATTTCTACTAATTAATTCTTC  344

Query  301  ATTAATCGAATCAGGAGCAGGTACAGGGTGAACAGTTTAcccccccTTATCATCAAATTT  360
            ||||||||||||||||||||||||||||||||||||||||||||||||||||||||||||
Sbjct  345  ATTAATCGAATCAGGAGCAGGTACAGGGTGAACAGTTTACCCCCCCTTATCATCAAATTT  404

Query  361  ATCACACTACGGACCATCAGTAGATTTAGCTATTTTTTCTCTGCATCTTGCAGGTGCTTC  420
            ||||||||||||||||||||||||||||||||||||||||||||||||||||||||||||
Sbjct  405  ATCACACTACGGACCATCAGTAGATTTAGCTATTTTTTCTCTGCATCTTGCAGGTGCTTC  464

Query  421  TTCAATTTTAGGTGCAATCAATTTCATTACAACTATTGTAAATATACGATCCATTGGAAT  480
            ||||||||||||||||||||||||||||||||||||||||||||||||||||||||||||
Sbjct  465  TTCAATTTTAGGTGCAATCAATTTCATTACAACTATTGTAAATATACGATCCATTGGAAT  524

Query  481  AACAATAGAACGAATACCATTATTTGTATGATCTGTTTTAATTACTGCAATTTTATTATT  540
            ||||||||||||||||||||||||||||||||||||||||||||||||||||||||||||
Sbjct  525  AACAATAGAACGAATACCATTATTTGTATGATCTGTTTTAATTACTGCAATTTTATTATT  584

Query  541  ATTATCTTTACCAGTCTTAGCTGGCGCAATTACAATATTATTAACTGATCGAAACTTTAA  600
            ||||||||||||||||||||||||||||||||||||||||||||||||||||||||||||
Sbjct  585  ATTATCTTTACCAGTCTTAGCTGGCGCAATTACAATATTATTAACTGATCGAAACTTTAA  644

Query  601  TACATCATTCTTTGACCCTTCAGGAGGAGGTGACCCAATTCTATATCAACATTTATTT  658
            ||||||||||||||||||||||||||||||||||||||||||||||||||||||||||
Sbjct  645  TACATCATTCTTTGACCCTTCAGGAGGAGGTGACCCAATTCTATATCAACATTTATTT  702
```

>Ixodidae sp. sc_01082 cytochrome oxidase subunit 1 (COI) gene, partial cds; mitochondrial
Sequence ID: KX053537.1 Length: 658
Range 1: 1 to 658

Score:1210 bits(655), Expect:0.0,
Identities:657/658(99%), Gaps:0/658(0%), Strand: Plus/Plus

```
Query  1    GACAATATACTTAATTTTTGGAGCATGATCCGGAATACTAGGATTAAGAATAAGAATATT  60
            ||||||||||||||||||||||||||||||||||||||||||||||||||||||||||||
Sbjct  1    GACAATATACTTAATTTTTGGAGCATGATCCGGAATACTAGGATTAAGAATAAGAATATT  60

Query  61   AATCCGCATAGAATTAGGTCAACCAGGAACTTTAATTGGAAACGATCAAATTTATAATGT  120
            ||||||||||||||||||||||||||||||||||||||||||||||||||||||||||||
Sbjct  61   AATCCGCATAGAATTAGGTCAACCAGGAACTTTAATTGGAAACGATCAAATTTATAATGT  120

Query  121  AATTGTAACAGCACATGCATTTATTATAAttttttttATAGTTATACCAATTATAATCGG  180
            ||||||||||||||||||||||||||||||||||||||||||||||||||||||||||||
Sbjct  121  AATTGTAACAGCACATGCATTTATTATAATTTTTTTTATAGTTATACCAATTATAATCGG  180
```

```
Query  181  AGGATTTGGAAATTGATTAGTTCCTATTATACTAGGAGCTCCAGATATAGCATTTCCACG  240
            ||||||||||||||||||||||||||||||||||||||||||||||||||||||||||||
Sbjct  181  AGGATTTGGAAATTGATTAGTTCCTATTATACTAGGAGCTCCAGATATAGCATTTCCACG  240

Query  241  AATAAATAATATAAGATTTTGGTTACTTCCTCCTTCTCTATTTCTACTAATTAATTCTTC  300
            ||||||||||||||||||||||||||||||||||||||||||||||||||||||||||||
Sbjct  241  AATAAATAATATAAGATTTTGGTTACTTCCTCCTTCTCTATTTCTACTAATTAATTCTTC  300

Query  301  ATTAATCGAATCAGGAGCAGGTACAGGGTGAACAGTTTAcccccccTTATCATCAAATTT  360
            ||||||||||||||||||||||||||||||||||||||||||||||||||||||||||||
Sbjct  301  ATTAATCGAATCAGGAGCAGGTACAGGGTGAACAGTTTACCCCCCCTTATCATCAAATTT  360

Query  361  ATCACACTACGGACCATCAGTAGATTTAGCTATTTTTTCTCTGCATCTTGCAGGTGCTTC  420
            |||||||||||||||||||||||| |||||||||||||||||||||||||||||||||||
Sbjct  361  ATCACACTACGGACCATCAGTAGATCTAGCTATTTTTTCTCTGCATCTTGCAGGTGCTTC  420

Query  421  TTCAATTTTAGGTGCAATCAATTTCATTACAACTATTGTAAATATACGATCCATTGGAAT  480
            ||||||||||||||||||||||||||||||||||||||||||||||||||||||||||||
Sbjct  421  TTCAATTTTAGGTGCAATCAATTTCATTACAACTATTGTAAATATACGATCCATTGGAAT  480

Query  481  AACAATAGAACGAATACCATTATTTGTATGATCTGTTTTAATTACTGCAATTTTATTATT  540
            ||||||||||||||||||||||||||||||||||||||||||||||||||||||||||||
Sbjct  481  AACAATAGAACGAATACCATTATTTGTATGATCTGTTTTAATTACTGCAATTTTATTATT  540

Query  541  ATTATCTTTACCAGTCTTAGCTGGCGCAATTACAATATTATTAACTGATCGAAACTTTAA  600
            ||||||||||||||||||||||||||||||||||||||||||||||||||||||||||||
Sbjct  541  ATTATCTTTACCAGTCTTAGCTGGCGCAATTACAATATTATTAACTGATCGAAACTTTAA  600

Query  601  TACATCATTCTTTGACCCTTCAGGAGGAGGTGACCCAATTCTATATCAACATTTATTT  658
            ||||||||||||||||||||||||||||||||||||||||||||||||||||||||||
Sbjct  601  TACATCATTCTTTGACCCTTCAGGAGGAGGTGACCCAATTCTATATCAACATTTATTT  658
```

>Rhipicephalus sanguineus haplotype RsanColCorL cytochrome c oxidase subunit I (COX1) gene, partial cds; mitochondrial
Sequence ID: KT906185.1 Length: 690
Range 1: 18 to 675

Score:1210 bits(655), Expect:0.0,
Identities:657/658(99%), Gaps:0/658(0%), Strand: Plus/Plus

```
Query  1    GACAATATACTTAATTTTTGGAGCATGATCCGGAATACTAGGATTAAGAATAAGAATATT  60
            ||||||||||||||||||||||||||||||||||||||||||||||||||||||||||||
Sbjct  18   GACAATATACTTAATTTTTGGAGCATGATCCGGAATACTAGGATTAAGAATAAGAATATT  77

Query  61   AATCCGCATAGAATTAGGTCAACCAGGAACTTTAATTGGAAACGATCAAATTTATAATGT  120
            ||||||||||||||||||||||||||||||||||||||||||||||||||||||||||||
Sbjct  78   AATCCGCATAGAATTAGGTCAACCAGGAACTTTAATTGGAAACGATCAAATTTATAATGT  137

Query  121  AATTGTAACAGCACATGCATTTATTATAAttttttttATAGTTATACCAATTATAATCGG  180
            ||||||||||||||||||||||||||||||||||||||||||||||||||||||||||||
```

```
Sbjct  138   AATTGTAACAGCACATGCATTTATTATAATTTTTTTTATAGTTATACCAATTATAATCGG  197

Query  181   AGGATTTGGAAATTGATTAGTTCCTATTATACTAGGAGCTCCAGATATAGCATTTCCACG  240
             ||||||||||||||||||||||||||||||||| ||||||||||||||||||||||||||
Sbjct  198   AGGATTTGGAAATTGATTAGTTCCTATTATACTGGGAGCTCCAGATATAGCATTTCCACG  257

Query  241   AATAAATAATATAAGATTTTGGTTACTTCCTCCTTCTCTATTTCTACTAATTAATTCTTC  300
             ||||||||||||||||||||||||||||||||||||||||||||||||||||||||||||
Sbjct  258   AATAAATAATATAAGATTTTGGTTACTTCCTCCTTCTCTATTTCTACTAATTAATTCTTC  317

Query  301   ATTAATCGAATCAGGAGCAGGTACAGGGTGAACAGTTTAcccccccTTATCATCAAATTT  360
             ||||||||||||||||||||||||||||||||||||||||||||||||||||||||||||
Sbjct  318   ATTAATCGAATCAGGAGCAGGTACAGGGTGAACAGTTTACCCCCCCTTATCATCAAATTT  377

Query  361   ATCACACTACGGACCATCAGTAGATTTAGCTATTTTTTCTCTGCATCTTGCAGGTGCTTC  420
             ||||||||||||||||||||||||||||||||||||||||||||||||||||||||||||
Sbjct  378   ATCACACTACGGACCATCAGTAGATTTAGCTATTTTTTCTCTGCATCTTGCAGGTGCTTC  437

Query  421   TTCAATTTTAGGTGCAATCAATTTCATTACAACTATTGTAAATATACGATCCATTGGAAT  480
             ||||||||||||||||||||||||||||||||||||||||||||||||||||||||||||
Sbjct  438   TTCAATTTTAGGTGCAATCAATTTCATTACAACTATTGTAAATATACGATCCATTGGAAT  497

Query  481   AACAATAGAACGAATACCATTATTTGTATGATCTGTTTTAATTACTGCAATTTTATTATT  540
             ||||||||||||||||||||||||||||||||||||||||||||||||||||||||||||
Sbjct  498   AACAATAGAACGAATACCATTATTTGTATGATCTGTTTTAATTACTGCAATTTTATTATT  557

Query  541   ATTATCTTTACCAGTCTTAGCTGGCGCAATTACAATATTATTAACTGATCGAAACTTTAA  600
             ||||||||||||||||||||||||||||||||||||||||||||||||||||||||||||
Sbjct  558   ATTATCTTTACCAGTCTTAGCTGGCGCAATTACAATATTATTAACTGATCGAAACTTTAA  617

Query  601   TACATCATTCTTTGACCCTTCAGGAGGAGGTGACCCAATTCTATATCAACATTTATTT  658
             ||||||||||||||||||||||||||||||||||||||||||||||||||||||||||
Sbjct  618   TACATCATTCTTTGACCCTTCAGGAGGAGGTGACCCAATTCTATATCAACATTTATTT  675
```

>Rhipicephalus sanguineus haplotype RsanColTolC cytochrome c oxidase subunit I (COX1) gene, partial cds; mitochondrial
Sequence ID: KT906184.1 Length: 690
Range 1: 18 to 675

Score:1210 bits(655), Expect:0.0,
Identities:657/658(99%), Gaps:0/658(0%), Strand: Plus/Plus

```
Query  1     GACAATATACTTAATTTTTGGAGCATGATCCGGAATACTAGGATTAAGAATAAGAATATT  60
             ||||||||||||||||||||||||||||||||| ||||||||||||||||||||||||||
Sbjct  18    GACAATATACTTAATTTTTGGAGCATGATCCGGGATACTAGGATTAAGAATAAGAATATT  77

Query  61    AATCCGCATAGAATTAGGTCAACCAGGAACTTTAATTGGAAACGATCAAATTTATAATGT  120
             ||||||||||||||||||||||||||||||||||||||||||||||||||||||||||||
Sbjct  78    AATCCGCATAGAATTAGGTCAACCAGGAACTTTAATTGGAAACGATCAAATTTATAATGT  137

Query  121   AATTGTAACAGCACATGCATTTATTATAAttttttttATAGTTATACCAATTATAATCGG  180
```

```
               ||||||||||||||||||||||||||||||||||||||||||||||||||||||||||||
Sbjct  138  AATTGTAACAGCACATGCATTTATTATAATTTTTTTTATAGTTATACCAATTATAATCGG  197

Query  181  AGGATTTGGAAATTGATTAGTTCCTATTATACTAGGAGCTCCAGATATAGCATTTCCACG  240
            ||||||||||||||||||||||||||||||||||||||||||||||||||||||||||||
Sbjct  198  AGGATTTGGAAATTGATTAGTTCCTATTATACTAGGAGCTCCAGATATAGCATTTCCACG  257

Query  241  AATAAATAATATAAGATTTTGGTTACTTCCTCCTTCTCTATTTCTACTAATTAATTCTTC  300
            ||||||||||||||||||||||||||||||||||||||||||||||||||||||||||||
Sbjct  258  AATAAATAATATAAGATTTTGGTTACTTCCTCCTTCTCTATTTCTACTAATTAATTCTTC  317

Query  301  ATTAATCGAATCAGGAGCAGGTACAGGGTGAACAGTTTAcccccccTTATCATCAAATTT  360
            ||||||||||||||||||||||||||||||||||||||||||||||||||||||||||||
Sbjct  318  ATTAATCGAATCAGGAGCAGGTACAGGGTGAACAGTTTACCCCCCCTTATCATCAAATTT  377

Query  361  ATCACACTACGGACCATCAGTAGATTTAGCTATTTTTTCTCTGCATCTTGCAGGTGCTTC  420
            ||||||||||||||||||||||||||||||||||||||||||||||||||||||||||||
Sbjct  378  ATCACACTACGGACCATCAGTAGATTTAGCTATTTTTTCTCTGCATCTTGCAGGTGCTTC  437

Query  421  TTCAATTTTAGGTGCAATCAATTTCATTACAACTATTGTAAATATACGATCCATTGGAAT  480
            ||||||||||||||||||||||||||||||||||||||||||||||||||||||||||||
Sbjct  438  TTCAATTTTAGGTGCAATCAATTTCATTACAACTATTGTAAATATACGATCCATTGGAAT  497

Query  481  AACAATAGAACGAATACCATTATTTGTATGATCTGTTTTAATTACTGCAATTTTATTATT  540
            ||||||||||||||||||||||||||||||||||||||||||||||||||||||||||||
Sbjct  498  AACAATAGAACGAATACCATTATTTGTATGATCTGTTTTAATTACTGCAATTTTATTATT  557

Query  541  ATTATCTTTACCAGTCTTAGCTGGCGCAATTACAATATTATTAACTGATCGAAACTTTAA  600
            ||||||||||||||||||||||||||||||||||||||||||||||||||||||||||||
Sbjct  558  ATTATCTTTACCAGTCTTAGCTGGCGCAATTACAATATTATTAACTGATCGAAACTTTAA  617

Query  601  TACATCATTCTTTGACCCTTCAGGAGGAGGTGACCCAATTCTATATCAACATTTATTT  658
            ||||||||||||||||||||||||||||||||||||||||||||||||||||||||||
Sbjct  618  TACATCATTCTTTGACCCTTCAGGAGGAGGTGACCCAATTCTATATCAACATTTATTT  675
```

>Rhipicephalus sanguineus voucher CAS-T065 cytochrome oxidase subunit 1 (COI) gene, partial cds; mitochondrial
Sequence ID: KF200113.1 Length: 658
Range 1: 1 to 658

Score:1210 bits(655), Expect:0.0,
Identities:657/658(99%), Gaps:0/658(0%), Strand: Plus/Plus

```
Query  1    GACAATATACTTAATTTTTGGAGCATGATCCGGAATACTAGGATTAAGAATAAGAATATT  60
            ||||||||||||||||||||||||||||||||||||||||||||||||||||||||||||
Sbjct  1    GACAATATACTTAATTTTTGGAGCATGATCCGGAATACTAGGATTAAGAATAAGAATATT  60

Query  61   AATCCGCATAGAATTAGGTCAACCAGGAACTTTAATTGGAAACGATCAAATTTATAATGT  120
            ||||||||||||||||||||||||||||||||||||||||||||||||||||||||||||
Sbjct  61   AATCCGCATAGAATTAGGTCAACCAGGAACTTTAATTGGAAACGATCAAATTTATAATGT  120
```

```
Query  121  AATTGTAACAGCACATGCATTTATTATAAttttttttATAGTTATACCAATTATAATCGG  180
            ||||||||||||||||||||||||||||||||||||||||||||||||||||||||||||
Sbjct  121  AATTGTAACAGCACATGCATTTATTATAATTTTTTTTATAGTTATACCAATTATAATCGG  180

Query  181  AGGATTTGGAAATTGATTAGTTCCTATTATACTAGGAGCTCCAGATATAGCATTTCCACG  240
            ||||||||||||||||||||||||||||||||||||||||||||||||||||||||||||
Sbjct  181  AGGATTTGGAAATTGATTAGTTCCTATTATACTAGGAGCTCCAGATATAGCATTTCCACG  240

Query  241  AATAAATAATATAAGATTTTGGTTACTTCCTCCTTCTCTATTTCTACTAATTAATTCTTC  300
            ||||||||||||||||||||||||||||||||||||||||||||||||||||||||||||
Sbjct  241  AATAAATAATATAAGATTTTGGTTACTTCCTCCTTCTCTATTTCTACTAATTAATTCTTC  300

Query  301  ATTAATCGAATCAGGAGCAGGTACAGGGTGAACAGTTTAcccccccTTATCATCAAATTT  360
            ||||||||||||||||||||| ||||||||||||||||||||||||||||||||||||||
Sbjct  301  ATTAATCGAATCAGGAGCAGGAACAGGGTGAACAGTTTACCCCCCCTTATCATCAAATTT  360

Query  361  ATCACACTACGGACCATCAGTAGATTTAGCTATTTTTTCTCTGCATCTTGCAGGTGCTTC  420
            ||||||||||||||||||||||||||||||||||||||||||||||||||||||||||||
Sbjct  361  ATCACACTACGGACCATCAGTAGATTTAGCTATTTTTTCTCTGCATCTTGCAGGTGCTTC  420

Query  421  TTCAATTTTAGGTGCAATCAATTTCATTACAACTATTGTAAATATACGATCCATTGGAAT  480
            ||||||||||||||||||||||||||||||||||||||||||||||||||||||||||||
Sbjct  421  TTCAATTTTAGGTGCAATCAATTTCATTACAACTATTGTAAATATACGATCCATTGGAAT  480

Query  481  AACAATAGAACGAATACCATTATTTGTATGATCTGTTTTAATTACTGCAATTTTATTATT  540
            ||||||||||||||||||||||||||||||||||||||||||||||||||||||||||||
Sbjct  481  AACAATAGAACGAATACCATTATTTGTATGATCTGTTTTAATTACTGCAATTTTATTATT  540

Query  541  ATTATCTTTACCAGTCTTAGCTGGCGCAATTACAATATTATTAACTGATCGAAACTTTAA  600
            ||||||||||||||||||||||||||||||||||||||||||||||||||||||||||||
Sbjct  541  ATTATCTTTACCAGTCTTAGCTGGCGCAATTACAATATTATTAACTGATCGAAACTTTAA  600

Query  601  TACATCATTCTTTGACCCTTCAGGAGGAGGTGACCCAATTCTATATCAACATTTATTT  658
            ||||||||||||||||||||||||||||||||||||||||||||||||||||||||||
Sbjct  601  TACATCATTCTTTGACCCTTCAGGAGGAGGTGACCCAATTCTATATCAACATTTATTT  658
```

```
>Rhipicephalus linnaei isolate SC931-1 clone JS5927 mitochondrion, complete
genome
Sequence ID: MW429382.1 Length: 14717
Range 1: 45 to 702

Score:1210 bits(655), Expect:0.0,
Identities:657/658(99%),  Gaps:0/658(0%), Strand: Plus/Plus

Query  1    GACAATATACTTAATTTTTGGAGCATGATCCGGAATACTAGGATTAAGAATAAGAATATT  60
            ||||||||||||||||||||||||||||||||||||||||||||||||||||||||||||
Sbjct  45   GACAATATACTTAATTTTTGGAGCATGATCCGGAATACTAGGATTAAGAATAAGAATATT  104

Query  61   AATCCGCATAGAATTAGGTCAACCAGGAACTTTAATTGGAAACGATCAAATTTATAATGT  120
            ||||||||||||||||||||||||||||||||||||||||||||||||||||||||||||
Sbjct  105  AATCCGCATAGAATTAGGTCAACCAGGAACTTTAATTGGAAACGATCAAATTTATAATGT  164
```

```
Query  121  AATTGTAACAGCACATGCATTTATTATAAttttttttATAGTTATACCAATTATAATCGG  180
            ||||||||||||||||||||||||||||||||||||||||||||||||||||||||||||
Sbjct  165  AATTGTAACAGCACATGCATTTATTATAATTTTTTTTATAGTTATACCAATTATAATCGG  224

Query  181  AGGATTTGGAAATTGATTAGTTCCTATTATACTAGGAGCTCCAGATATAGCATTTCCACG  240
            ||||||||||||||||||||||||||||||||||||||||||||||||||||||||||||
Sbjct  225  AGGATTTGGAAATTGATTAGTTCCTATTATACTAGGAGCTCCAGATATAGCATTTCCACG  284

Query  241  AATAAATAATATAAGATTTTGGTTACTTCCTCCTTCTCTATTTCTACTAATTAATTCTTC  300
            ||||||||||||||||||||||||||||||||||||||||||||||||||||||||||||
Sbjct  285  AATAAATAATATAAGATTTTGGTTACTTCCTCCTTCTCTATTTCTACTAATTAATTCTTC  344

Query  301  ATTAATCGAATCAGGAGCAGGTACAGGGTGAACAGTTTAcccccccTTATCATCAAATTT  360
            ||||||||||||||||||||||||||||||||||||||||||||||||||||||||||||
Sbjct  345  ATTAATCGAATCAGGAGCAGGTACAGGGTGAACAGTTTACCCCCCCTTATCATCAAATTT  404

Query  361  ATCACACTACGGACCATCAGTAGATTTAGCTATTTTTCTCTGCATCTTGCAGGTGCTTC  420
            ||||||||||||||||||||||||||||||||||||||||| ||||||||||||||||||
Sbjct  405  ATCACACTACGGACCATCAGTAGATTTAGCTATTTTTCTCTACATCTTGCAGGTGCTTC  464

Query  421  TTCAATTTTAGGTGCAATCAATTTCATTACAACTATTGTAAATATACGATCCATTGGAAT  480
            ||||||||||||||||||||||||||||||||||||||||||||||||||||||||||||
Sbjct  465  TTCAATTTTAGGTGCAATCAATTTCATTACAACTATTGTAAATATACGATCCATTGGAAT  524

Query  481  AACAATAGAACGAATACCATTATTTGTATGATCTGTTTTAATTACTGCAATTTTATTATT  540
            ||||||||||||||||||||||||||||||||||||||||||||||||||||||||||||
Sbjct  525  AACAATAGAACGAATACCATTATTTGTATGATCTGTTTTAATTACTGCAATTTTATTATT  584

Query  541  ATTATCTTTACCAGTCTTAGCTGGCGCAATTACAATATTATTAACTGATCGAAACTTTAA  600
            ||||||||||||||||||||||||||||||||||||||||||||||||||||||||||||
Sbjct  585  ATTATCTTTACCAGTCTTAGCTGGCGCAATTACAATATTATTAACTGATCGAAACTTTAA  644

Query  601  TACATCATTCTTTGACCCTTCAGGAGGAGGTGACCCAATTCTATATCAACATTTATTT  658
            ||||||||||||||||||||||||||||||||||||||||||||||||||||||||||
Sbjct  645  TACATCATTCTTTGACCCTTCAGGAGGAGGTGACCCAATTCTATATCAACATTTATTT  702
```

```
>Rhipicephalus sanguineus voucher DO-311 cytochrome oxidase subunit 1 (COI)
gene, partial cds; mitochondrial
Sequence ID: KX360367.1 Length: 658
Range 1: 4 to 658

Score:1205 bits(652), Expect:0.0,
Identities:654/655(99%),  Gaps:0/655(0%), Strand: Plus/Plus

Query  4    AATATACTTAATTTTTGGAGCATGATCCGGAATACTAGGATTAAGAATAAGAATATTAAT  63
            ||||||||||||||||||||||||||||||||||||||||||||||||||||||||||||
Sbjct  4    AATATACTTAATTTTTGGAGCATGATCCGGAATACTAGGATTAAGAATAAGAATATTAAT  63

Query  64   CCGCATAGAATTAGGTCAACCAGGAACTTTAATTGGAAACGATCAAATTTATAATGTAAT  123
            ||||||||||||||||||||||||||||||||||||||||||||||||||||||||||||
```

```
Sbjct  64   CCGCATAGAATTAGGTCAACCAGGAACTTTAATTGGAAACGATCAAATTTATAATGTAAT  123

Query  124  TGTAACAGCACATGCATTTATTATAAttttttttATAGTTATACCAATTATAATCGGAGG  183
            ||||||||||||||||||||||||||||||||||||||||||||||||||||||||||||
Sbjct  124  TGTAACAGCACATGCATTTATTATAATTTTTTTTATAGTTATACCAATTATAATCGGAGG  183

Query  184  ATTTGGAAATTGATTAGTTCCTATTATACTAGGAGCTCCAGATATAGCATTTCCACGAAT  243
            ||||||||||||||||||||||||||||||||||||||||||||||||||||||||||||
Sbjct  184  ATTTGGAAATTGATTAGTTCCTATTATACTAGGAGCTCCAGATATAGCATTTCCACGAAT  243

Query  244  AAATAATATAAGATTTTGGTTACTTCCTCCTTCTCTATTTCTACTAATTAATTCTTCATT  303
            ||||||||||||||||||||||||||||||||||||||||||||||||||||||||||||
Sbjct  244  AAATAATATAAGATTTTGGTTACTTCCTCCTTCTCTATTTCTACTAATTAATTCTTCATT  303

Query  304  AATCGAATCAGGAGCAGGTACAGGGTGAACAGTTTAcccccccTTATCATCAAATTTATC  363
            ||||||||||||||||||||||||||||||||||||||||||||||||||||||||||||
Sbjct  304  AATCGAATCAGGAGCAGGTACAGGGTGAACAGTTTACCCCCCCTTATCATCAAATTTATC  363

Query  364  ACACTACGGACCATCAGTAGATTTAGCTATTTTTTCTCTGCATCTTGCAGGTGCTTCTTC  423
            ||||||||||||||||||||||||||||||||||||||| ||||||||||||||||||||
Sbjct  364  ACACTACGGACCATCAGTAGATTTAGCTATTTTTTCTCTACATCTTGCAGGTGCTTCTTC  423

Query  424  AATTTTAGGTGCAATCAATTTCATTACAACTATTGTAAATATACGATCCATTGGAATAAC  483
            ||||||||||||||||||||||||||||||||||||||||||||||||||||||||||||
Sbjct  424  AATTTTAGGTGCAATCAATTTCATTACAACTATTGTAAATATACGATCCATTGGAATAAC  483

Query  484  AATAGAACGAATACCATTATTTGTATGATCTGTTTTAATTACTGCAATTTTATTATTATT  543
            ||||||||||||||||||||||||||||||||||||||||||||||||||||||||||||
Sbjct  484  AATAGAACGAATACCATTATTTGTATGATCTGTTTTAATTACTGCAATTTTATTATTATT  543

Query  544  ATCTTTACCAGTCTTAGCTGGCGCAATTACAATATTATTAACTGATCGAAACTTTAATAC  603
            ||||||||||||||||||||||||||||||||||||||||||||||||||||||||||||
Sbjct  544  ATCTTTACCAGTCTTAGCTGGCGCAATTACAATATTATTAACTGATCGAAACTTTAATAC  603

Query  604  ATCATTCTTTGACCCTTCAGGAGGAGGTGACCCAATTCTATATCAACATTTATTT  658
            |||||||||||||||||||||||||||||||||||||||||||||||||||||||
Sbjct  604  ATCATTCTTTGACCCTTCAGGAGGAGGTGACCCAATTCTATATCAACATTTATTT  658
```

>Rhipicephalus sanguineus haplotype RsanColSamCM cytochrome c oxidase subunit I (COX1) gene, partial cds; mitochondrial
Sequence ID: KT906186.1 Length: 690
Range 1: 18 to 675

Score:1205 bits(652), Expect:0.0,
Identities:656/658(99%), Gaps:0/658(0%), Strand: Plus/Plus

```
Query  1    GACAATATACTTAATTTTTGGAGCATGATCCGGAATACTAGGATTAAGAATAAGAATATT  60
            ||||||||||||||||||||||||||||||||||||| ||||||||||||||||||||||
Sbjct  18   GACAATATACTTAATTTTTGGAGCATGATCCGGAATATTAGGATTAAGAATAAGAATATT  77

Query  61   AATCCGCATAGAATTAGGTCAACCAGGAACTTTAATTGGAAACGATCAAATTTATAATGT  120
```

```
                ||||||||||||||||||||||||||||||||||||||||||||||||||||||||||||
Sbjct  78       AATCCGCATAGAATTAGGTCAACCAGGAACTTTAATTGGAAACGATCAAATTTATAATGT  137

Query  121      AATTGTAACAGCACATGCATTTATTATAAtttttttATAGTTATACCAATTATAATCGG  180
                ||||||||||||||||||||||||||||||||||||||||||||||||||||||||||||
Sbjct  138      AATTGTAACAGCACATGCATTTATTATAATTTTTTTATAGTTATACCAATTATAATCGG  197

Query  181      AGGATTTGGAAATTGATTAGTTCCTATTATACTAGGAGCTCCAGATATAGCATTTCCACG  240
                |||||||||||||||||||||||||||||||||||| |||||||||||||||||||||||
Sbjct  198      AGGATTTGGAAATTGATTAGTTCCTATTATACTAGGGGCTCCAGATATAGCATTTCCACG  257

Query  241      AATAAATAATATAAGATTTTGGTTACTTCCTCCTTCTCTATTTCTACTAATTAATTCTTC  300
                ||||||||||||||||||||||||||||||||||||||||||||||||||||||||||||
Sbjct  258      AATAAATAATATAAGATTTTGGTTACTTCCTCCTTCTCTATTTCTACTAATTAATTCTTC  317

Query  301      ATTAATCGAATCAGGAGCAGGTACAGGGTGAACAGTTTAcccccccTTATCATCAAATTT  360
                ||||||||||||||||||||||||||||||||||||||||||||||||||||||||||||
Sbjct  318      ATTAATCGAATCAGGAGCAGGTACAGGGTGAACAGTTTACCCCCCCTTATCATCAAATTT  377

Query  361      ATCACACTACGGACCATCAGTAGATTTAGCTATTTTTTCTCTGCATCTTGCAGGTGCTTC  420
                ||||||||||||||||||||||||||||||||||||||||||||||||||||||||||||
Sbjct  378      ATCACACTACGGACCATCAGTAGATTTAGCTATTTTTTCTCTGCATCTTGCAGGTGCTTC  437

Query  421      TTCAATTTTAGGTGCAATCAATTTCATTACAACTATTGTAAATATACGATCCATTGGAAT  480
                ||||||||||||||||||||||||||||||||||||||||||||||||||||||||||||
Sbjct  438      TTCAATTTTAGGTGCAATCAATTTCATTACAACTATTGTAAATATACGATCCATTGGAAT  497

Query  481      AACAATAGAACGAATACCATTATTTGTATGATCTGTTTTAATTACTGCAATTTTATTATT  540
                ||||||||||||||||||||||||||||||||||||||||||||||||||||||||||||
Sbjct  498      AACAATAGAACGAATACCATTATTTGTATGATCTGTTTTAATTACTGCAATTTTATTATT  557

Query  541      ATTATCTTTACCAGTCTTAGCTGGCGCAATTACAATATTATTAACTGATCGAAACTTTAA  600
                ||||||||||||||||||||||||||||||||||||||||||||||||||||||||||||
Sbjct  558      ATTATCTTTACCAGTCTTAGCTGGCGCAATTACAATATTATTAACTGATCGAAACTTTAA  617

Query  601      TACATCATTCTTTGACCCTTCAGGAGGAGGTGACCCAATTCTATATCAACATTTATTT  658
                ||||||||||||||||||||||||||||||||||||||||||||||||||||||||||
Sbjct  618      TACATCATTCTTTGACCCTTCAGGAGGAGGTGACCCAATTCTATATCAACATTTATTT  675
```

>Rhipicephalus sanguineus voucher GRP5-154 cytochrome oxidase subunit 1 (COI) gene, partial cds; mitochondrial
Sequence ID: KF200112.1 Length: 658
>Rhipicephalus sanguineus voucher DO-309 cytochrome oxidase subunit 1 (COI) gene, partial cds; mitochondrial
Sequence ID: KX360403.1 Length: 658
Range 1: 1 to 658

Score:1205 bits(652), Expect:0.0,
Identities:656/658(99%), Gaps:0/658(0%), Strand: Plus/Plus

```
Query  1        GACAATATACTTAATTTTTGGAGCATGATCCGGAATACTAGGATTAAGAATAAGAATATT  60
```

```
              ||||||||||||||||||||||||||||||||||||| ||||||||||||||||||||||
Sbjct  1      GACAATATACTTAATTTTTGGAGCATGATCCGGAATATTAGGATTAAGAATAAGAATATT  60

Query  61     AATCCGCATAGAATTAGGTCAACCAGGAACTTTAATTGGAAACGATCAAATTTATAATGT  120
              ||||||||||||||||||||||||||||||||||||||||||||||||||||||||||||
Sbjct  61     AATCCGCATAGAATTAGGTCAACCAGGAACTTTAATTGGAAACGATCAAATTTATAATGT  120

Query  121    AATTGTAACAGCACATGCATTTATTATAAtttttttATAGTTATACCAATTATAATCGG  180
              ||||||||||||||||||||||||||||||||||||||||||||||||||||||||||||
Sbjct  121    AATTGTAACAGCACATGCATTTATTATAATTTTTTTATAGTTATACCAATTATAATCGG  180

Query  181    AGGATTTGGAAATTGATTAGTTCCTATTATACTAGGAGCTCCAGATATAGCATTTCCACG  240
              |||||||||||||||||||||||||||||||||||| |||||||||||||||||||||||
Sbjct  181    AGGATTTGGAAATTGATTAGTTCCTATTATACTAGGGGCTCCAGATATAGCATTTCCACG  240

Query  241    AATAAATAATATAAGATTTTGGTTACTTCCTCCTTCTCTATTTCTACTAATTAATTCTTC  300
              ||||||||||||||||||||||||||||||||||||||||||||||||||||||||||||
Sbjct  241    AATAAATAATATAAGATTTTGGTTACTTCCTCCTTCTCTATTTCTACTAATTAATTCTTC  300

Query  301    ATTAATCGAATCAGGAGCAGGTACAGGGTGAACAGTTTAcccccccTTATCATCAAATTT  360
              ||||||||||||||||||||||||||||||||||||||||||||||||||||||||||||
Sbjct  301    ATTAATCGAATCAGGAGCAGGTACAGGGTGAACAGTTTACCCCCCCTTATCATCAAATTT  360

Query  361    ATCACACTACGGACCATCAGTAGATTTAGCTATTTTTTCTCTGCATCTTGCAGGTGCTTC  420
              ||||||||||||||||||||||||||||||||||||||||||||||||||||||||||||
Sbjct  361    ATCACACTACGGACCATCAGTAGATTTAGCTATTTTTTCTCTGCATCTTGCAGGTGCTTC  420

Query  421    TTCAATTTTAGGTGCAATCAATTTCATTACAACTATTGTAAATATACGATCCATTGGAAT  480
              ||||||||||||||||||||||||||||||||||||||||||||||||||||||||||||
Sbjct  421    TTCAATTTTAGGTGCAATCAATTTCATTACAACTATTGTAAATATACGATCCATTGGAAT  480

Query  481    AACAATAGAACGAATACCATTATTTGTATGATCTGTTTTAATTACTGCAATTTTATTATT  540
              ||||||||||||||||||||||||||||||||||||||||||||||||||||||||||||
Sbjct  481    AACAATAGAACGAATACCATTATTTGTATGATCTGTTTTAATTACTGCAATTTTATTATT  540

Query  541    ATTATCTTTACCAGTCTTAGCTGGCGCAATTACAATATTATTAACTGATCGAAACTTTAA  600
              ||||||||||||||||||||||||||||||||||||||||||||||||||||||||||||
Sbjct  541    ATTATCTTTACCAGTCTTAGCTGGCGCAATTACAATATTATTAACTGATCGAAACTTTAA  600

Query  601    TACATCATTCTTTGACCCTTCAGGAGGAGGTGACCCAATTCTATATCAACATTTATTT  658
              ||||||||||||||||||||||||||||||||||||||||||||||||||||||||||
Sbjct  601    TACATCATTCTTTGACCCTTCAGGAGGAGGTGACCCAATTCTATATCAACATTTATTT  658
```

```
>Rhipicephalus sanguineus mitochondrion, complete genome
Sequence ID: JX416325.1 Length: 14714
Range 1: 1185 to 1842

Score:1205 bits(652), Expect:0.0,
Identities:656/658(99%),  Gaps:0/658(0%), Strand: Plus/Plus

Query  1      GACAATATACTTAATTTTTGGAGCATGATCCGGAATACTAGGATTAAGAATAAGAATATT  60
```

```
                ||||||||||||||||||||||||||||||||||||||||||||||||||||||||||||
Sbjct  1185  GACAATATACTTAATTTTTGGAGCATGATCCGGAATACTAGGATTAAGAATAAGAATATT  1244

Query  61    AATCCGCATAGAATTAGGTCAACCAGGAACTTTAATTGGAAACGATCAAATTTATAATGT  120
             ||||||||||||||||||||||||||||||||||||||||||||||||||||||||||||
Sbjct  1245  AATCCGCATAGAATTAGGTCAACCAGGAACTTTAATTGGAAACGATCAAATTTATAATGT  1304

Query  121   AATTGTAACAGCACATGCATTTATTATAAtttttttATAGTTATACCAATTATAATCGG  180
             ||||||||||||||||||||||||||||||||||||||||||||||||||||||||||||
Sbjct  1305  AATTGTAACAGCACATGCATTTATTATAATTTTTTTATAGTTATACCAATTATAATCGG  1364

Query  181   AGGATTTGGAAATTGATTAGTTCCTATTATACTAGGAGCTCCAGATATAGCATTTCCACG  240
             |||||||||||||||||||||||||||||||||||||||||||||| |||||||||||||
Sbjct  1365  AGGATTTGGAAATTGATTAGTTCCTATTATACTAGGAGCTCCAGATGTAGCATTTCCACG  1424

Query  241   AATAAATAATATAAGATTTTGGTTACTTCCTCCTTCTCTATTTCTACTAATTAATTCTTC  300
             ||||||||||||||||||||||||||||||||||||||||||||||||||||||||||||
Sbjct  1425  AATAAATAATATAAGATTTTGGTTACTTCCTCCTTCTCTATTTCTACTAATTAATTCTTC  1484

Query  301   ATTAATCGAATCAGGAGCAGGTACAGGGTGAACAGTTTAcccccccTTATCATCAAATTT  360
             ||||||||||||||||||||||||||||||||||||||||||||||||||||||||||||
Sbjct  1485  ATTAATCGAATCAGGAGCAGGTACAGGGTGAACAGTTTACCCCCCCTTATCATCAAATTT  1544

Query  361   ATCACACTACGGACCATCAGTAGATTTAGCTATTTTTTCTCTGCATCTTGCAGGTGCTTC  420
             ||||||||||||||||||||||||||||||||||||||||||||||||||||||||||||
Sbjct  1545  ATCACACTACGGACCATCAGTAGATTTAGCTATTTTTTCTCTGCATCTTGCAGGTGCTTC  1604

Query  421   TTCAATTTTAGGTGCAATCAATTTCATTACAACTATTGTAAATATACGATCCATTGGAAT  480
             ||||||||||||||||||||||||||||||||||||||||||||||||||||||||||||
Sbjct  1605  TTCAATTTTAGGTGCAATCAATTTCATTACAACTATTGTAAATATACGATCCATTGGAAT  1664

Query  481   AACAATAGAACGAATACCATTATTTGTATGATCTGTTTTAATTACTGCAATTTTATTATT  540
             ||||||||||||||||||||||||||||||||||||||||||||||||||||||||||||
Sbjct  1665  AACAATAGAACGAATACCATTATTTGTATGATCTGTTTTAATTACTGCAATTTTATTATT  1724

Query  541   ATTATCTTTACCAGTCTTAGCTGGCGCAATTACAATATTATTAACTGATCGAAACTTTAA  600
             ||||||||||||||||||||||||||||||||||||||||||||||||||||||||||||
Sbjct  1725  ATTATCTTTACCAGTCTTAGCTGGCGCAATTACAATATTATTAACTGATCGAAACTTTAA  1784

Query  601   TACATCATTCTTTGACCCTTCAGGAGGAGGTGACCCAATTCTATATCAACATTTATTT  658
             |||||||||||||||||||||||||||||||||||||||||||||||||| |||||||
Sbjct  1785  TACATCATTCTTTGACCCTTCAGGAGGAGGTGACCCAATTCTATATCAACGTTTATTT  1842
```

>Rhipicephalus sanguineus isolate T95D15 cytochrome c oxidase subunit I (COX1) gene, partial cds; mitochondrial
Sequence ID: MZ401443.1 Length: 661
Range 1: 1 to 645

Score:1192 bits(645), Expect:0.0,
Identities:645/645(100%), Gaps:0/645(0%), Strand: Plus/Plus

```
Query  14   ATTTTTGGAGCATGATCCGGAATACTAGGATTAAGAATAAGAATATTAATCCGCATAGAA  73
            ||||||||||||||||||||||||||||||||||||||||||||||||||||||||||||
Sbjct  1    ATTTTTGGAGCATGATCCGGAATACTAGGATTAAGAATAAGAATATTAATCCGCATAGAA  60

Query  74   TTAGGTCAACCAGGAACTTTAATTGGAAACGATCAAATTTATAATGTAATTGTAACAGCA  133
            ||||||||||||||||||||||||||||||||||||||||||||||||||||||||||||
Sbjct  61   TTAGGTCAACCAGGAACTTTAATTGGAAACGATCAAATTTATAATGTAATTGTAACAGCA  120

Query  134  CATGCATTTATTATAAttttttttATAGTTATACCAATTATAATCGGAGGATTTGGAAAT  193
            ||||||||||||||||||||||||||||||||||||||||||||||||||||||||||||
Sbjct  121  CATGCATTTATTATAATTTTTTTTATAGTTATACCAATTATAATCGGAGGATTTGGAAAT  180

Query  194  TGATTAGTTCCTATTATACTAGGAGCTCCAGATATAGCATTTCCACGAATAAATAATATA  253
            ||||||||||||||||||||||||||||||||||||||||||||||||||||||||||||
Sbjct  181  TGATTAGTTCCTATTATACTAGGAGCTCCAGATATAGCATTTCCACGAATAAATAATATA  240

Query  254  AGATTTTGGTTACTTCCTCCTTCTCTATTTCTACTAATTAATTCTTCATTAATCGAATCA  313
            ||||||||||||||||||||||||||||||||||||||||||||||||||||||||||||
Sbjct  241  AGATTTTGGTTACTTCCTCCTTCTCTATTTCTACTAATTAATTCTTCATTAATCGAATCA  300

Query  314  GGAGCAGGTACAGGGTGAACAGTTTAcccccccTTATCATCAAATTTATCACACTACGGA  373
            ||||||||||||||||||||||||||||||||||||||||||||||||||||||||||||
Sbjct  301  GGAGCAGGTACAGGGTGAACAGTTTACCCCCCCTTATCATCAAATTTATCACACTACGGA  360

Query  374  CCATCAGTAGATTTAGCTATTTTTTCTCTGCATCTTGCAGGTGCTTCTTCAATTTTAGGT  433
            ||||||||||||||||||||||||||||||||||||||||||||||||||||||||||||
Sbjct  361  CCATCAGTAGATTTAGCTATTTTTTCTCTGCATCTTGCAGGTGCTTCTTCAATTTTAGGT  420

Query  434  GCAATCAATTTCATTACAACTATTGTAAATATACGATCCATTGGAATAACAATAGAACGA  493
            ||||||||||||||||||||||||||||||||||||||||||||||||||||||||||||
Sbjct  421  GCAATCAATTTCATTACAACTATTGTAAATATACGATCCATTGGAATAACAATAGAACGA  480

Query  494  ATACCATTATTTGTATGATCTGTTTTAATTACTGCAATTTTATTATTATTATCTTTACCA  553
            ||||||||||||||||||||||||||||||||||||||||||||||||||||||||||||
Sbjct  481  ATACCATTATTTGTATGATCTGTTTTAATTACTGCAATTTTATTATTATTATCTTTACCA  540

Query  554  GTCTTAGCTGGCGCAATTACAATATTATTAACTGATCGAAACTTTAATACATCATTCTTT  613
            ||||||||||||||||||||||||||||||||||||||||||||||||||||||||||||
Sbjct  541  GTCTTAGCTGGCGCAATTACAATATTATTAACTGATCGAAACTTTAATACATCATTCTTT  600

Query  614  GACCCTTCAGGAGGAGGTGACCCAATTCTATATCAACATTTATTT  658
            |||||||||||||||||||||||||||||||||||||||||||||
Sbjct  601  GACCCTTCAGGAGGAGGTGACCCAATTCTATATCAACATTTATTT  645
```

>Rhipicephalus sanguineus isolate T81D7 cytochrome c oxidase subunit I (COX1) gene, partial cds; mitochondrial
Sequence ID: MZ401441.1 Length: 661
Range 1: 1 to 645

Score:1192 bits(645), Expect:0.0,
Identities:645/645(100%), Gaps:0/645(0%), Strand: Plus/Plus

```
Query  14   ATTTTTGGAGCATGATCCGGAATACTAGGATTAAGAATAAGAATATTAATCCGCATAGAA  73
            ||||||||||||||||||||||||||||||||||||||||||||||||||||||||||||
Sbjct  1    ATTTTTGGAGCATGATCCGGAATACTAGGATTAAGAATAAGAATATTAATCCGCATAGAA  60

Query  74   TTAGGTCAACCAGGAACTTTAATTGGAAACGATCAAATTTATAATGTAATTGTAACAGCA  133
            ||||||||||||||||||||||||||||||||||||||||||||||||||||||||||||
Sbjct  61   TTAGGTCAACCAGGAACTTTAATTGGAAACGATCAAATTTATAATGTAATTGTAACAGCA  120

Query  134  CATGCATTTATTATAAttttttttATAGTTATACCAATTATAATCGGAGGATTTGGAAAT  193
            ||||||||||||||||||||||||||||||||||||||||||||||||||||||||||||
Sbjct  121  CATGCATTTATTATAATTTTTTTTATAGTTATACCAATTATAATCGGAGGATTTGGAAAT  180

Query  194  TGATTAGTTCCTATTATACTAGGAGCTCCAGATATAGCATTTCCACGAATAAATAATATA  253
            ||||||||||||||||||||||||||||||||||||||||||||||||||||||||||||
Sbjct  181  TGATTAGTTCCTATTATACTAGGAGCTCCAGATATAGCATTTCCACGAATAAATAATATA  240

Query  254  AGATTTTGGTTACTTCCTCCTTCTCTATTTCTACTAATTAATTCTTCATTAATCGAATCA  313
            ||||||||||||||||||||||||||||||||||||||||||||||||||||||||||||
Sbjct  241  AGATTTTGGTTACTTCCTCCTTCTCTATTTCTACTAATTAATTCTTCATTAATCGAATCA  300

Query  314  GGAGCAGGTACAGGGTGAACAGTTTAcccccccTTATCATCAAATTTATCACACTACGGA  373
            ||||||||||||||||||||||||||||||||||||||||||||||||||||||||||||
Sbjct  301  GGAGCAGGTACAGGGTGAACAGTTTACCCCCCCTTATCATCAAATTTATCACACTACGGA  360

Query  374  CCATCAGTAGATTTAGCTATTTTTTCTCTGCATCTTGCAGGTGCTTCTTCAATTTTAGGT  433
            ||||||||||||||||||||||||||||||||||||||||||||||||||||||||||||
Sbjct  361  CCATCAGTAGATTTAGCTATTTTTTCTCTGCATCTTGCAGGTGCTTCTTCAATTTTAGGT  420

Query  434  GCAATCAATTTCATTACAACTATTGTAAATATACGATCCATTGGAATAACAATAGAACGA  493
            ||||||||||||||||||||||||||||||||||||||||||||||||||||||||||||
Sbjct  421  GCAATCAATTTCATTACAACTATTGTAAATATACGATCCATTGGAATAACAATAGAACGA  480

Query  494  ATACCATTATTTGTATGATCTGTTTTAATTACTGCAATTTTATTATTATTATCTTTACCA  553
            ||||||||||||||||||||||||||||||||||||||||||||||||||||||||||||
Sbjct  481  ATACCATTATTTGTATGATCTGTTTTAATTACTGCAATTTTATTATTATTATCTTTACCA  540

Query  554  GTCTTAGCTGGCGCAATTACAATATTATTAACTGATCGAAACTTTAATACATCATTCTTT  613
            ||||||||||||||||||||||||||||||||||||||||||||||||||||||||||||
Sbjct  541  GTCTTAGCTGGCGCAATTACAATATTATTAACTGATCGAAACTTTAATACATCATTCTTT  600

Query  614  GACCCTTCAGGAGGAGGTGACCCAATTCTATATCAACATTTATTT  658
            |||||||||||||||||||||||||||||||||||||||||||||
Sbjct  601  GACCCTTCAGGAGGAGGTGACCCAATTCTATATCAACATTTATTT  645
```

>Rhipicephalus sanguineus isolate T79D2 cytochrome c oxidase subunit I (COX1) gene, partial cds; mitochondrial
Sequence ID: MZ401440.1 Length: 664
Range 1: 1 to 648

Score:1192 bits(645), Expect:0.0,

```
Identities:647/648(99%),  Gaps:0/648(0%), Strand: Plus/Plus

Query  11   TTAATTTTTGGAGCATGATCCGGAATACTAGGATTAAGAATAAGAATATTAATCCGCATA  70
            ||||||||||||||||||||||||||||||||||||||||||||||||||||||||||||
Sbjct  1    TTAATTTTTGGAGCATGATCCGGAATACTAGGATTAAGAATAAGAATATTAATCCGCATA  60

Query  71   GAATTAGGTCAACCAGGAACTTTAATTGGAAACGATCAAATTTATAATGTAATTGTAACA  130
            ||||||||||||||||||||||||||||||||||||||||||||||||||||||||||||
Sbjct  61   GAATTAGGTCAACCAGGAACTTTAATTGGAAACGATCAAATTTATAATGTAATTGTAACA  120

Query  131  GCACATGCATTTATTATAAttttttttATAGTTATACCAATTATAATCGGAGGATTTGGA  190
            ||||||||||||||||||||||||||||||||||||||||||||||||||||||||||||
Sbjct  121  GCACATGCATTTATTATAATTTTTTTTATAGTTATACCAATTATAATCGGAGGATTTGGA  180

Query  191  AATTGATTAGTTCCTATTATACTAGGAGCTCCAGATATAGCATTTCCACGAATAAATAAT  250
            ||||||||||||||||||||||||||||||||||||||||||||||||||||||||||||
Sbjct  181  AATTGATTAGTTCCTATTATACTAGGAGCTCCAGATATAGCATTTCCACGAATAAATAAT  240

Query  251  ATAAGATTTTGGTTACTTCCTCCTTCTCTATTTCTACTAATTAATTCTTCATTAATCGAA  310
            ||||||||||||||||||||||||||||||||||||||||||||||||||||||||||||
Sbjct  241  ATAAGATTTTGGTTACTTCCTCCTTCTCTATTTCTACTAATTAATTCTTCATTAATCGAA  300

Query  311  TCAGGAGCAGGTACAGGGTGAACAGTTTAcccccccTTATCATCAAATTTATCACACTAC  370
            ||||||||||| ||||||||||||||||||||||||||||||||||||||||||||||||
Sbjct  301  TCAGGAGCAGGAACAGGGTGAACAGTTTACCCCCCCTTATCATCAAATTTATCACACTAC  360

Query  371  GGACCATCAGTAGATTTAGCTATTTTTTCTCTGCATCTTGCAGGTGCTTCTTCAATTTTA  430
            ||||||||||||||||||||||||||||||||||||||||||||||||||||||||||||
Sbjct  361  GGACCATCAGTAGATTTAGCTATTTTTTCTCTGCATCTTGCAGGTGCTTCTTCAATTTTA  420

Query  431  GGTGCAATCAATTTCATTACAACTATTGTAAATATACGATCCATTGGAATAACAATAGAA  490
            ||||||||||||||||||||||||||||||||||||||||||||||||||||||||||||
Sbjct  421  GGTGCAATCAATTTCATTACAACTATTGTAAATATACGATCCATTGGAATAACAATAGAA  480

Query  491  CGAATACCATTATTTGTATGATCTGTTTTAATTACTGCAATTTTATTATTATTATCTTTA  550
            ||||||||||||||||||||||||||||||||||||||||||||||||||||||||||||
Sbjct  481  CGAATACCATTATTTGTATGATCTGTTTTAATTACTGCAATTTTATTATTATTATCTTTA  540

Query  551  CCAGTCTTAGCTGGCGCAATTACAATATTATTAACTGATCGAAACTTTAATACATCATTC  610
            ||||||||||||||||||||||||||||||||||||||||||||||||||||||||||||
Sbjct  541  CCAGTCTTAGCTGGCGCAATTACAATATTATTAACTGATCGAAACTTTAATACATCATTC  600

Query  611  TTTGACCCTTCAGGAGGAGGTGACCCAATTCTATATCAACATTTATTT  658
            ||||||||||||||||||||||||||||||||||||||||||||||||
Sbjct  601  TTTGACCCTTCAGGAGGAGGTGACCCAATTCTATATCAACATTTATTT  648
```

>Rhipicephalus sanguineus isolate WZ004 cytochrome oxidase subunit I gene, partial cds; mitochondrial
Sequence ID: MG969507.1 Length: 664
Range 1: 1 to 644

```
Score:1190 bits(644), Expect:0.0,
Identities:644/644(100%),  Gaps:0/644(0%), Strand: Plus/Plus

Query  15   TTTTTGGAGCATGATCCGGAATACTAGGATTAAGAATAAGAATATTAATCCGCATAGAAT  74
            ||||||||||||||||||||||||||||||||||||||||||||||||||||||||||||
Sbjct  1    TTTTTGGAGCATGATCCGGAATACTAGGATTAAGAATAAGAATATTAATCCGCATAGAAT  60

Query  75   TAGGTCAACCAGGAACTTTAATTGGAAACGATCAAATTTATAATGTAATTGTAACAGCAC  134
            ||||||||||||||||||||||||||||||||||||||||||||||||||||||||||||
Sbjct  61   TAGGTCAACCAGGAACTTTAATTGGAAACGATCAAATTTATAATGTAATTGTAACAGCAC  120

Query  135  ATGCATTTATTATAAtttttttATAGTTATACCAATTATAATCGGAGGATTTGGAAATT  194
            ||||||||||||||||||||||||||||||||||||||||||||||||||||||||||||
Sbjct  121  ATGCATTTATTATAATTTTTTTTATAGTTATACCAATTATAATCGGAGGATTTGGAAATT  180

Query  195  GATTAGTTCCTATTATACTAGGAGCTCCAGATATAGCATTTCCACGAATAAATAATATAA  254
            ||||||||||||||||||||||||||||||||||||||||||||||||||||||||||||
Sbjct  181  GATTAGTTCCTATTATACTAGGAGCTCCAGATATAGCATTTCCACGAATAAATAATATAA  240

Query  255  GATTTTGGTTACTTCCTCCTTCTCTATTTCTACTAATTAATTCTTCATTAATCGAATCAG  314
            ||||||||||||||||||||||||||||||||||||||||||||||||||||||||||||
Sbjct  241  GATTTTGGTTACTTCCTCCTTCTCTATTTCTACTAATTAATTCTTCATTAATCGAATCAG  300

Query  315  GAGCAGGTACAGGGTGAACAGTTTAcccccccTTATCATCAAATTTATCACACTACGGAC  374
            ||||||||||||||||||||||||||||||||||||||||||||||||||||||||||||
Sbjct  301  GAGCAGGTACAGGGTGAACAGTTTACCCCCCCTTATCATCAAATTTATCACACTACGGAC  360

Query  375  CATCAGTAGATTTAGCTATTTTTTCTCTGCATCTTGCAGGTGCTTCTTCAATTTTAGGTG  434
            ||||||||||||||||||||||||||||||||||||||||||||||||||||||||||||
Sbjct  361  CATCAGTAGATTTAGCTATTTTTTCTCTGCATCTTGCAGGTGCTTCTTCAATTTTAGGTG  420

Query  435  CAATCAATTTCATTACAACTATTGTAAATATACGATCCATTGGAATAACAATAGAACGAA  494
            ||||||||||||||||||||||||||||||||||||||||||||||||||||||||||||
Sbjct  421  CAATCAATTTCATTACAACTATTGTAAATATACGATCCATTGGAATAACAATAGAACGAA  480

Query  495  TACCATTATTTGTATGATCTGTTTTAATTACTGCAATTTTATTATTATTATCTTTACCAG  554
            ||||||||||||||||||||||||||||||||||||||||||||||||||||||||||||
Sbjct  481  TACCATTATTTGTATGATCTGTTTTAATTACTGCAATTTTATTATTATTATCTTTACCAG  540

Query  555  TCTTAGCTGGCGCAATTACAATATTATTAACTGATCGAAACTTTAATACATCATTCTTTG  614
            ||||||||||||||||||||||||||||||||||||||||||||||||||||||||||||
Sbjct  541  TCTTAGCTGGCGCAATTACAATATTATTAACTGATCGAAACTTTAATACATCATTCTTTG  600

Query  615  ACCCTTCAGGAGGAGGTGACCCAATTCTATATCAACATTTATTT  658
            ||||||||||||||||||||||||||||||||||||||||||||
Sbjct  601  ACCCTTCAGGAGGAGGTGACCCAATTCTATATCAACATTTATTT  644
```

>Rhipicephalus sanguineus voucher GRP5-150 cytochrome oxidase subunit 1 (COI) gene, partial cds; mitochondrial
Sequence ID: KF200084.1 Length: 649
Range 1: 1 to 649

```
Score:1188 bits(643), Expect:0.0,
Identities:647/649(99%),  Gaps:0/649(0%), Strand: Plus/Plus

Query  10   CTTAATTTTTGGAGCATGATCCGGAATACTAGGATTAAGAATAAGAATATTAATCCGCAT  69
            ||||||||||||||||||||||||||||| ||||||||||||||||||||||||||||||
Sbjct  1    CTTAATTTTTGGAGCATGATCCGGAATATTAGGATTAAGAATAAGAATATTAATCCGCAT  60

Query  70   AGAATTAGGTCAACCAGGAACTTTAATTGGAAACGATCAAATTTATAATGTAATTGTAAC  129
            ||||||||||||||||||||||||||||||||||||||||||||||||||||||||||||
Sbjct  61   AGAATTAGGTCAACCAGGAACTTTAATTGGAAACGATCAAATTTATAATGTAATTGTAAC  120

Query  130  AGCACATGCATTTATTATAAttttttttATAGTTATACCAATTATAATCGGAGGATTTGG  189
            ||||||||||||||||||||||||||||||||||||||||||||||||||||||||||||
Sbjct  121  AGCACATGCATTTATTATAATTTTTTTTATAGTTATACCAATTATAATCGGAGGATTTGG  180

Query  190  AAATTGATTAGTTCCTATTATACTAGGAGCTCCAGATATAGCATTTCCACGAATAAATAA  249
            |||||||||||||||||||||||||||| |||||||||||||||||||||||||||||||
Sbjct  181  AAATTGATTAGTTCCTATTATACTAGGGGCTCCAGATATAGCATTTCCACGAATAAATAA  240

Query  250  TATAAGATTTTGGTTACTTCCTCCTTCTCTATTTCTACTAATTAATTCTTCATTAATCGA  309
            ||||||||||||||||||||||||||||||||||||||||||||||||||||||||||||
Sbjct  241  TATAAGATTTTGGTTACTTCCTCCTTCTCTATTTCTACTAATTAATTCTTCATTAATCGA  300

Query  310  ATCAGGAGCAGGTACAGGGTGAACAGTTTAccccccccTTATCATCAAATTTATCACACTA  369
            ||||||||||||||||||||||||||||||||||||||||||||||||||||||||||||
Sbjct  301  ATCAGGAGCAGGTACAGGGTGAACAGTTTACCCCCCCTTATCATCAAATTTATCACACTA  360

Query  370  CGGACCATCAGTAGATTTAGCTATTTTTTCTCTGCATCTTGCAGGTGCTTCTTCAATTTT  429
            ||||||||||||||||||||||||||||||||||||||||||||||||||||||||||||
Sbjct  361  CGGACCATCAGTAGATTTAGCTATTTTTTCTCTGCATCTTGCAGGTGCTTCTTCAATTTT  420

Query  430  AGGTGCAATCAATTTCATTACAACTATTGTAAATATACGATCCATTGGAATAACAATAGA  489
            ||||||||||||||||||||||||||||||||||||||||||||||||||||||||||||
Sbjct  421  AGGTGCAATCAATTTCATTACAACTATTGTAAATATACGATCCATTGGAATAACAATAGA  480

Query  490  ACGAATACCATTATTTGTATGATCTGTTTTAATTACTGCAATTTTATTATTATTATCTTT  549
            ||||||||||||||||||||||||||||||||||||||||||||||||||||||||||||
Sbjct  481  ACGAATACCATTATTTGTATGATCTGTTTTAATTACTGCAATTTTATTATTATTATCTTT  540

Query  550  ACCAGTCTTAGCTGGCGCAATTACAATATTATTAACTGATCGAAACTTTAATACATCATT  609
            ||||||||||||||||||||||||||||||||||||||||||||||||||||||||||||
Sbjct  541  ACCAGTCTTAGCTGGCGCAATTACAATATTATTAACTGATCGAAACTTTAATACATCATT  600

Query  610  CTTTGACCCTTCAGGAGGAGGTGACCCAATTCTATATCAACATTTATTT  658
            |||||||||||||||||||||||||||||||||||||||||||||||||
Sbjct  601  CTTTGACCCTTCAGGAGGAGGTGACCCAATTCTATATCAACATTTATTT  649
```

>Rhipicephalus sanguineus isolate LIC6084B cytochrome oxidase subunit 1 (COI) gene, partial cds; mitochondrial
Sequence ID: KX383817.1 Length: 768

```
Range 1: 1 to 642

Score:1186 bits(642), Expect:0.0,
Identities:642/642(100%),  Gaps:0/642(0%), Strand: Plus/Plus

Query  17   TTTGGAGCATGATCCGGAATACTAGGATTAAGAATAAGAATATTAATCCGCATAGAATTA  76
            ||||||||||||||||||||||||||||||||||||||||||||||||||||||||||||
Sbjct  1    TTTGGAGCATGATCCGGAATACTAGGATTAAGAATAAGAATATTAATCCGCATAGAATTA  60

Query  77   GGTCAACCAGGAACTTTAATTGGAAACGATCAAATTTATAATGTAATTGTAACAGCACAT  136
            ||||||||||||||||||||||||||||||||||||||||||||||||||||||||||||
Sbjct  61   GGTCAACCAGGAACTTTAATTGGAAACGATCAAATTTATAATGTAATTGTAACAGCACAT  120

Query  137  GCATTTATTATAAtttttttATAGTTATACCAATTATAATCGGAGGATTTGGAAATTGA  196
            ||||||||||||||||||||||||||||||||||||||||||||||||||||||||||||
Sbjct  121  GCATTTATTATAATTTTTTTATAGTTATACCAATTATAATCGGAGGATTTGGAAATTGA  180

Query  197  TTAGTTCCTATTATACTAGGAGCTCCAGATATAGCATTTCCACGAATAAATAATATAAGA  256
            ||||||||||||||||||||||||||||||||||||||||||||||||||||||||||||
Sbjct  181  TTAGTTCCTATTATACTAGGAGCTCCAGATATAGCATTTCCACGAATAAATAATATAAGA  240

Query  257  TTTTGGTTACTTCCTCCTTCTCTATTTCTACTAATTAATTCTTCATTAATCGAATCAGGA  316
            ||||||||||||||||||||||||||||||||||||||||||||||||||||||||||||
Sbjct  241  TTTTGGTTACTTCCTCCTTCTCTATTTCTACTAATTAATTCTTCATTAATCGAATCAGGA  300

Query  317  GCAGGTACAGGGTGAACAGTTTAcccccccTTATCATCAAATTTATCACACTACGGACCA  376
            ||||||||||||||||||||||||||||||||||||||||||||||||||||||||||||
Sbjct  301  GCAGGTACAGGGTGAACAGTTTACCCCCCCTTATCATCAAATTTATCACACTACGGACCA  360

Query  377  TCAGTAGATTTAGCTATTTTTTCTCTGCATCTTGCAGGTGCTTCTTCAATTTTAGGTGCA  436
            ||||||||||||||||||||||||||||||||||||||||||||||||||||||||||||
Sbjct  361  TCAGTAGATTTAGCTATTTTTTCTCTGCATCTTGCAGGTGCTTCTTCAATTTTAGGTGCA  420

Query  437  ATCAATTTCATTACAACTATTGTAAATATACGATCCATTGGAATAACAATAGAACGAATA  496
            ||||||||||||||||||||||||||||||||||||||||||||||||||||||||||||
Sbjct  421  ATCAATTTCATTACAACTATTGTAAATATACGATCCATTGGAATAACAATAGAACGAATA  480

Query  497  CCATTATTTGTATGATCTGTTTTAATTACTGCAATTTTATTATTATTATCTTTACCAGTC  556
            ||||||||||||||||||||||||||||||||||||||||||||||||||||||||||||
Sbjct  481  CCATTATTTGTATGATCTGTTTTAATTACTGCAATTTTATTATTATTATCTTTACCAGTC  540

Query  557  TTAGCTGGCGCAATTACAATATTATTAACTGATCGAAACTTTAATACATCATTCTTTGAC  616
            ||||||||||||||||||||||||||||||||||||||||||||||||||||||||||||
Sbjct  541  TTAGCTGGCGCAATTACAATATTATTAACTGATCGAAACTTTAATACATCATTCTTTGAC  600

Query  617  CCTTCAGGAGGAGGTGACCCAATTCTATATCAACATTTATTT  658
            ||||||||||||||||||||||||||||||||||||||||||
Sbjct  601  CCTTCAGGAGGAGGTGACCCAATTCTATATCAACATTTATTT  642
```

>Rhipicephalus sanguineus isolate T91D13 cytochrome c oxidase subunit I (COX1) gene, partial cds; mitochondrial

```
Sequence ID: MZ401442.1 Length: 657
Range 1: 1 to 642

Score:1186 bits(642), Expect:0.0,
Identities:642/642(100%),  Gaps:0/642(0%), Strand: Plus/Plus

Query  17   TTTGGAGCATGATCCGGAATACTAGGATTAAGAATAAGAATATTAATCCGCATAGAATTA  76
            ||||||||||||||||||||||||||||||||||||||||||||||||||||||||||||
Sbjct  1    TTTGGAGCATGATCCGGAATACTAGGATTAAGAATAAGAATATTAATCCGCATAGAATTA  60

Query  77   GGTCAACCAGGAACTTTAATTGGAAACGATCAAATTTATAATGTAATTGTAACAGCACAT  136
            ||||||||||||||||||||||||||||||||||||||||||||||||||||||||||||
Sbjct  61   GGTCAACCAGGAACTTTAATTGGAAACGATCAAATTTATAATGTAATTGTAACAGCACAT  120

Query  137  GCATTTATTATAAttttttttATAGTTATACCAATTATAATCGGAGGATTTGGAAATTGA  196
            ||||||||||||||||||||||||||||||||||||||||||||||||||||||||||||
Sbjct  121  GCATTTATTATAATTTTTTTTATAGTTATACCAATTATAATCGGAGGATTTGGAAATTGA  180

Query  197  TTAGTTCCTATTATACTAGGAGCTCCAGATATAGCATTTCCACGAATAAATAATATAAGA  256
            ||||||||||||||||||||||||||||||||||||||||||||||||||||||||||||
Sbjct  181  TTAGTTCCTATTATACTAGGAGCTCCAGATATAGCATTTCCACGAATAAATAATATAAGA  240

Query  257  TTTTGGTTACTTCCTCCTTCTCTATTTCTACTAATTAATTCTTCATTAATCGAATCAGGA  316
            ||||||||||||||||||||||||||||||||||||||||||||||||||||||||||||
Sbjct  241  TTTTGGTTACTTCCTCCTTCTCTATTTCTACTAATTAATTCTTCATTAATCGAATCAGGA  300

Query  317  GCAGGTACAGGGTGAACAGTTTAcccccccTTATCATCAAATTTATCACACTACGGACCA  376
            ||||||||||||||||||||||||||||||||||||||||||||||||||||||||||||
Sbjct  301  GCAGGTACAGGGTGAACAGTTTACCCCCCCTTATCATCAAATTTATCACACTACGGACCA  360

Query  377  TCAGTAGATTTAGCTATTTTTTCTCTGCATCTTGCAGGTGCTTCTTCAATTTTAGGTGCA  436
            ||||||||||||||||||||||||||||||||||||||||||||||||||||||||||||
Sbjct  361  TCAGTAGATTTAGCTATTTTTTCTCTGCATCTTGCAGGTGCTTCTTCAATTTTAGGTGCA  420

Query  437  ATCAATTTCATTACAACTATTGTAAATATACGATCCATTGGAATAACAATAGAACGAATA  496
            ||||||||||||||||||||||||||||||||||||||||||||||||||||||||||||
Sbjct  421  ATCAATTTCATTACAACTATTGTAAATATACGATCCATTGGAATAACAATAGAACGAATA  480

Query  497  CCATTATTTGTATGATCTGTTTTAATTACTGCAATTTTATTATTATTATCTTTACCAGTC  556
            ||||||||||||||||||||||||||||||||||||||||||||||||||||||||||||
Sbjct  481  CCATTATTTGTATGATCTGTTTTAATTACTGCAATTTTATTATTATTATCTTTACCAGTC  540

Query  557  TTAGCTGGCGCAATTACAATATTATTAACTGATCGAAACTTTAATACATCATTCTTTGAC  616
            ||||||||||||||||||||||||||||||||||||||||||||||||||||||||||||
Sbjct  541  TTAGCTGGCGCAATTACAATATTATTAACTGATCGAAACTTTAATACATCATTCTTTGAC  600

Query  617  CCTTCAGGAGGAGGTGACCCAATTCTATATCAACATTTATTT  658
            ||||||||||||||||||||||||||||||||||||||||||
Sbjct  601  CCTTCAGGAGGAGGTGACCCAATTCTATATCAACATTTATTT  642
```

>Rhipicephalus sanguineus isolate T63D1 cytochrome c oxidase subunit I (COX1)

gene, partial cds; mitochondrial
Sequence ID: MZ401438.1 Length: 661
Range 1: 1 to 642

Score:1186 bits(642), Expect:0.0,
Identities:642/642(100%), Gaps:0/642(0%), Strand: Plus/Plus

```
Query  17   TTTGGAGCATGATCCGGAATACTAGGATTAAGAATAAGAATATTAATCCGCATAGAATTA  76
            ||||||||||||||||||||||||||||||||||||||||||||||||||||||||||||
Sbjct  1    TTTGGAGCATGATCCGGAATACTAGGATTAAGAATAAGAATATTAATCCGCATAGAATTA  60

Query  77   GGTCAACCAGGAACTTTAATTGGAAACGATCAAATTTATAATGTAATTGTAACAGCACAT  136
            ||||||||||||||||||||||||||||||||||||||||||||||||||||||||||||
Sbjct  61   GGTCAACCAGGAACTTTAATTGGAAACGATCAAATTTATAATGTAATTGTAACAGCACAT  120

Query  137  GCATTTATTATAAttttttttATAGTTATACCAATTATAATCGGAGGATTTGGAAATTGA  196
            ||||||||||||||||||||||||||||||||||||||||||||||||||||||||||||
Sbjct  121  GCATTTATTATAATTTTTTTTATAGTTATACCAATTATAATCGGAGGATTTGGAAATTGA  180

Query  197  TTAGTTCCTATTATACTAGGAGCTCCAGATATAGCATTTCCACGAATAAATAATATAAGA  256
            ||||||||||||||||||||||||||||||||||||||||||||||||||||||||||||
Sbjct  181  TTAGTTCCTATTATACTAGGAGCTCCAGATATAGCATTTCCACGAATAAATAATATAAGA  240

Query  257  TTTTGGTTACTTCCTCCTTCTCTATTTCTACTAATTAATTCTTCATTAATCGAATCAGGA  316
            ||||||||||||||||||||||||||||||||||||||||||||||||||||||||||||
Sbjct  241  TTTTGGTTACTTCCTCCTTCTCTATTTCTACTAATTAATTCTTCATTAATCGAATCAGGA  300

Query  317  GCAGGTACAGGGTGAACAGTTTAcccccccTTATCATCAAATTTATCACACTACGGACCA  376
            ||||||||||||||||||||||||||||||||||||||||||||||||||||||||||||
Sbjct  301  GCAGGTACAGGGTGAACAGTTTACCCCCCCTTATCATCAAATTTATCACACTACGGACCA  360

Query  377  TCAGTAGATTTAGCTATTTTTTCTCTGCATCTTGCAGGTGCTTCTTCAATTTTAGGTGCA  436
            ||||||||||||||||||||||||||||||||||||||||||||||||||||||||||||
Sbjct  361  TCAGTAGATTTAGCTATTTTTTCTCTGCATCTTGCAGGTGCTTCTTCAATTTTAGGTGCA  420

Query  437  ATCAATTTCATTACAACTATTGTAAATATACGATCCATTGGAATAACAATAGAACGAATA  496
            ||||||||||||||||||||||||||||||||||||||||||||||||||||||||||||
Sbjct  421  ATCAATTTCATTACAACTATTGTAAATATACGATCCATTGGAATAACAATAGAACGAATA  480

Query  497  CCATTATTTGTATGATCTGTTTTAATTACTGCAATTTTATTATTATTATCTTTACCAGTC  556
            ||||||||||||||||||||||||||||||||||||||||||||||||||||||||||||
Sbjct  481  CCATTATTTGTATGATCTGTTTTAATTACTGCAATTTTATTATTATTATCTTTACCAGTC  540

Query  557  TTAGCTGGCGCAATTACAATATTATTAACTGATCGAAACTTTAATACATCATTCTTTGAC  616
            ||||||||||||||||||||||||||||||||||||||||||||||||||||||||||||
Sbjct  541  TTAGCTGGCGCAATTACAATATTATTAACTGATCGAAACTTTAATACATCATTCTTTGAC  600

Query  617  CCTTCAGGAGGAGGTGACCCAATTCTATATCAACATTTATTT  658
            ||||||||||||||||||||||||||||||||||||||||||
Sbjct  601  CCTTCAGGAGGAGGTGACCCAATTCTATATCAACATTTATTT  642
```

>Rhipicephalus sanguineus isolate WZ003 cytochrome oxidase subunit I gene, partial cds; mitochondrial
Sequence ID: MG969506.1 Length: 664
Range 1: 1 to 644

Score:1184 bits(641), Expect:0.0,
Identities:643/644(99%), Gaps:0/644(0%), Strand: Plus/Plus

```
Query  15   TTTTTGGAGCATGATCCGGAATACTAGGATTAAGAATAAGAATATTAATCCGCATAGAAT  74
            || |||||||||||||||||||||||||||||||||||||||||||||||||||||||||
Sbjct  1    TTGTTGGAGCATGATCCGGAATACTAGGATTAAGAATAAGAATATTAATCCGCATAGAAT  60

Query  75   TAGGTCAACCAGGAACTTTAATTGGAAACGATCAAATTTATAATGTAATTGTAACAGCAC  134
            ||||||||||||||||||||||||||||||||||||||||||||||||||||||||||||
Sbjct  61   TAGGTCAACCAGGAACTTTAATTGGAAACGATCAAATTTATAATGTAATTGTAACAGCAC  120

Query  135  ATGCATTTATTATAAtttttttATAGTTATACCAATTATAATCGGAGGATTTGGAAATT  194
            ||||||||||||||||||||||||||||||||||||||||||||||||||||||||||||
Sbjct  121  ATGCATTTATTATAATTTTTTTATAGTTATACCAATTATAATCGGAGGATTTGGAAATT  180

Query  195  GATTAGTTCCTATTATACTAGGAGCTCCAGATATAGCATTTCCACGAATAAATAATATAA  254
            ||||||||||||||||||||||||||||||||||||||||||||||||||||||||||||
Sbjct  181  GATTAGTTCCTATTATACTAGGAGCTCCAGATATAGCATTTCCACGAATAAATAATATAA  240

Query  255  GATTTTGGTTACTTCCTCCTTCTCTATTTCTACTAATTAATTCTTCATTAATCGAATCAG  314
            ||||||||||||||||||||||||||||||||||||||||||||||||||||||||||||
Sbjct  241  GATTTTGGTTACTTCCTCCTTCTCTATTTCTACTAATTAATTCTTCATTAATCGAATCAG  300

Query  315  GAGCAGGTACAGGGTGAACAGTTTAcccccccTTATCATCAAATTTATCACACTACGGAC  374
            ||||||||||||||||||||||||||||||||||||||||||||||||||||||||||||
Sbjct  301  GAGCAGGTACAGGGTGAACAGTTTACCCCCCCTTATCATCAAATTTATCACACTACGGAC  360

Query  375  CATCAGTAGATTTAGCTATTTTTTCTCTGCATCTTGCAGGTGCTTCTTCAATTTTAGGTG  434
            ||||||||||||||||||||||||||||||||||||||||||||||||||||||||||||
Sbjct  361  CATCAGTAGATTTAGCTATTTTTTCTCTGCATCTTGCAGGTGCTTCTTCAATTTTAGGTG  420

Query  435  CAATCAATTTCATTACAACTATTGTAAATATACGATCCATTGGAATAACAATAGAACGAA  494
            ||||||||||||||||||||||||||||||||||||||||||||||||||||||||||||
Sbjct  421  CAATCAATTTCATTACAACTATTGTAAATATACGATCCATTGGAATAACAATAGAACGAA  480

Query  495  TACCATTATTTGTATGATCTGTTTTAATTACTGCAATTTTATTATTATTATCTTTACCAG  554
            ||||||||||||||||||||||||||||||||||||||||||||||||||||||||||||
Sbjct  481  TACCATTATTTGTATGATCTGTTTTAATTACTGCAATTTTATTATTATTATCTTTACCAG  540

Query  555  TCTTAGCTGGCGCAATTACAATATTATTAACTGATCGAAACTTTAATACATCATTCTTTG  614
            ||||||||||||||||||||||||||||||||||||||||||||||||||||||||||||
Sbjct  541  TCTTAGCTGGCGCAATTACAATATTATTAACTGATCGAAACTTTAATACATCATTCTTTG  600

Query  615  ACCCTTCAGGAGGAGGTGACCCAATTCTATATCAACATTTATTT  658
            ||||||||||||||||||||||||||||||||||||||||||||
Sbjct  601  ACCCTTCAGGAGGAGGTGACCCAATTCTATATCAACATTTATTT  644
```

```
>Rhipicephalus sanguineus isolate WZ002 cytochrome oxidase subunit I gene,
partial cds; mitochondrial
Sequence ID: MG969505.1 Length: 664
Range 1: 1 to 644

Score:1184 bits(641), Expect:0.0,
Identities:643/644(99%),  Gaps:0/644(0%), Strand: Plus/Plus

Query  15   TTTTTGGAGCATGATCCGGAATACTAGGATTAAGAATAAGAATATTAATCCGCATAGAAT  74
            || |||||||||||||||||||||||||||||||||||||||||||||||||||||||||
Sbjct  1    TTATTGGAGCATGATCCGGAATACTAGGATTAAGAATAAGAATATTAATCCGCATAGAAT  60

Query  75   TAGGTCAACCAGGAACTTTAATTGGAAACGATCAAATTTATAATGTAATTGTAACAGCAC  134
            ||||||||||||||||||||||||||||||||||||||||||||||||||||||||||||
Sbjct  61   TAGGTCAACCAGGAACTTTAATTGGAAACGATCAAATTTATAATGTAATTGTAACAGCAC  120

Query  135  ATGCATTTATTATAAtttttttATAGTTATACCAATTATAATCGGAGGATTTGGAAATT  194
            ||||||||||||||||||||||||||||||||||||||||||||||||||||||||||||
Sbjct  121  ATGCATTTATTATAATTTTTTTATAGTTATACCAATTATAATCGGAGGATTTGGAAATT  180

Query  195  GATTAGTTCCTATTATACTAGGAGCTCCAGATATAGCATTTCCACGAATAAATAATATAA  254
            ||||||||||||||||||||||||||||||||||||||||||||||||||||||||||||
Sbjct  181  GATTAGTTCCTATTATACTAGGAGCTCCAGATATAGCATTTCCACGAATAAATAATATAA  240

Query  255  GATTTTGGTTACTTCCTCCTTCTCTATTTCTACTAATTAATTCTTCATTAATCGAATCAG  314
            ||||||||||||||||||||||||||||||||||||||||||||||||||||||||||||
Sbjct  241  GATTTTGGTTACTTCCTCCTTCTCTATTTCTACTAATTAATTCTTCATTAATCGAATCAG  300

Query  315  GAGCAGGTACAGGGTGAACAGTTTAcccccccTTATCATCAAATTTATCACACTACGGAC  374
            ||||||||||||||||||||||||||||||||||||||||||||||||||||||||||||
Sbjct  301  GAGCAGGTACAGGGTGAACAGTTTACCCCCCCTTATCATCAAATTTATCACACTACGGAC  360

Query  375  CATCAGTAGATTTAGCTATTTTTTCTCTGCATCTTGCAGGTGCTTCTTCAATTTTAGGTG  434
            ||||||||||||||||||||||||||||||||||||||||||||||||||||||||||||
Sbjct  361  CATCAGTAGATTTAGCTATTTTTTCTCTGCATCTTGCAGGTGCTTCTTCAATTTTAGGTG  420

Query  435  CAATCAATTTCATTACAACTATTGTAAATATACGATCCATTGGAATAACAATAGAACGAA  494
            ||||||||||||||||||||||||||||||||||||||||||||||||||||||||||||
Sbjct  421  CAATCAATTTCATTACAACTATTGTAAATATACGATCCATTGGAATAACAATAGAACGAA  480

Query  495  TACCATTATTTGTATGATCTGTTTTAATTACTGCAATTTTATTATTATTATCTTTACCAG  554
            ||||||||||||||||||||||||||||||||||||||||||||||||||||||||||||
Sbjct  481  TACCATTATTTGTATGATCTGTTTTAATTACTGCAATTTTATTATTATTATCTTTACCAG  540

Query  555  TCTTAGCTGGCGCAATTACAATATTATTAACTGATCGAAACTTTAATACATCATTCTTTG  614
            ||||||||||||||||||||||||||||||||||||||||||||||||||||||||||||
Sbjct  541  TCTTAGCTGGCGCAATTACAATATTATTAACTGATCGAAACTTTAATACATCATTCTTTG  600

Query  615  ACCCTTCAGGAGGAGGTGACCCAATTCTATATCAACATTTATTT  658
            ||||||||||||||||||||||||||||||||||||||||||||
Sbjct  601  ACCCTTCAGGAGGAGGTGACCCAATTCTATATCAACATTTATTT  644
```

```
>Rhipicephalus sanguineus isolate WZ001 cytochrome oxidase subunit I gene,
partial cds; mitochondrial
Sequence ID: MG969504.1 Length: 664
Range 1: 1 to 644

Score:1184 bits(641), Expect:0.0,
Identities:643/644(99%),  Gaps:0/644(0%), Strand: Plus/Plus

Query  15   TTTTTGGAGCATGATCCGGAATACTAGGATTAAGAATAAGAATATTAATCCGCATAGAAT  74
            || |||||||||||||||||||||||||||||||||||||||||||||||||||||||||
Sbjct  1    TTGTTGGAGCATGATCCGGAATACTAGGATTAAGAATAAGAATATTAATCCGCATAGAAT  60

Query  75   TAGGTCAACCAGGAACTTTAATTGGAAACGATCAAATTTATAATGTAATTGTAACAGCAC  134
            ||||||||||||||||||||||||||||||||||||||||||||||||||||||||||||
Sbjct  61   TAGGTCAACCAGGAACTTTAATTGGAAACGATCAAATTTATAATGTAATTGTAACAGCAC  120

Query  135  ATGCATTTATTATAAttttttttATAGTTATACCAATTATAATCGGAGGATTTGGAAATT  194
            ||||||||||||||||||||||||||||||||||||||||||||||||||||||||||||
Sbjct  121  ATGCATTTATTATAATTTTTTTTATAGTTATACCAATTATAATCGGAGGATTTGGAAATT  180

Query  195  GATTAGTTCCTATTATACTAGGAGCTCCAGATATAGCATTTCCACGAATAAATAATATAA  254
            ||||||||||||||||||||||||||||||||||||||||||||||||||||||||||||
Sbjct  181  GATTAGTTCCTATTATACTAGGAGCTCCAGATATAGCATTTCCACGAATAAATAATATAA  240

Query  255  GATTTTGGTTACTTCCTCCTTCTCTATTTCTACTAATTAATTCTTCATTAATCGAATCAG  314
            ||||||||||||||||||||||||||||||||||||||||||||||||||||||||||||
Sbjct  241  GATTTTGGTTACTTCCTCCTTCTCTATTTCTACTAATTAATTCTTCATTAATCGAATCAG  300

Query  315  GAGCAGGTACAGGGTGAACAGTTTAcccccccTTATCATCAAATTTATCACACTACGGAC  374
            ||||||||||||||||||||||||||||||||||||||||||||||||||||||||||||
Sbjct  301  GAGCAGGTACAGGGTGAACAGTTTACCCCCCCTTATCATCAAATTTATCACACTACGGAC  360

Query  375  CATCAGTAGATTTAGCTATTTTTTCTCTGCATCTTGCAGGTGCTTCTTCAATTTTAGGTG  434
            ||||||||||||||||||||||||||||||||||||||||||||||||||||||||||||
Sbjct  361  CATCAGTAGATTTAGCTATTTTTTCTCTGCATCTTGCAGGTGCTTCTTCAATTTTAGGTG  420

Query  435  CAATCAATTTCATTACAACTATTGTAAATATACGATCCATTGGAATAACAATAGAACGAA  494
            ||||||||||||||||||||||||||||||||||||||||||||||||||||||||||||
Sbjct  421  CAATCAATTTCATTACAACTATTGTAAATATACGATCCATTGGAATAACAATAGAACGAA  480

Query  495  TACCATTATTTGTATGATCTGTTTTAATTACTGCAATTTTATTATTATTATCTTTACCAG  554
            ||||||||||||||||||||||||||||||||||||||||||||||||||||||||||||
Sbjct  481  TACCATTATTTGTATGATCTGTTTTAATTACTGCAATTTTATTATTATTATCTTTACCAG  540

Query  555  TCTTAGCTGGCGCAATTACAATATTATTAACTGATCGAAACTTTAATACATCATTCTTTG  614
            ||||||||||||||||||||||||||||||||||||||||||||||||||||||||||||
Sbjct  541  TCTTAGCTGGCGCAATTACAATATTATTAACTGATCGAAACTTTAATACATCATTCTTTG  600

Query  615  ACCCTTCAGGAGGAGGTGACCCAATTCTATATCAACATTTATTT  658
            ||||||||||||||||||||||||||||||||||||||||||||
Sbjct  601  ACCCTTCAGGAGGAGGTGACCCAATTCTATATCAACATTTATTT  644
```

>Rhipicephalus sanguineus voucher CAS-T066 cytochrome oxidase subunit 1 (COI) gene, partial cds; mitochondrial
Sequence ID: KF200096.1 Length: 644
Range 1: 1 to 644

Score:1179 bits(638), Expect:0.0,
Identities:642/644(99%), Gaps:0/644(0%), Strand: Plus/Plus

```
Query  6    TATACTTAATTTTTGGAGCATGATCCGGAATACTAGGATTAAGAATAAGAATATTAATCC  65
            |||||||||||||||||||||||||||||||| |||||||||||||||||||||||||||
Sbjct  1    TATACTTAATTTTTGGAGCATGATCCGGAATATTAGGATTAAGAATAAGAATATTAATCC  60

Query  66   GCATAGAATTAGGTCAACCAGGAACTTTAATTGGAAACGATCAAATTTATAATGTAATTG  125
            ||||||||||||||||||||||||||||||||||||||||||||||||||||||||||||
Sbjct  61   GCATAGAATTAGGTCAACCAGGAACTTTAATTGGAAACGATCAAATTTATAATGTAATTG  120

Query  126  TAACAGCACATGCATTTATTATAAtttttttATAGTTATACCAATTATAATCGGAGGAT  185
            ||||||||||||||||||||||||||||||||||||||||||||||||||||||||||||
Sbjct  121  TAACAGCACATGCATTTATTATAATTTTTTTATAGTTATACCAATTATAATCGGAGGAT  180

Query  186  TTGGAAATTGATTAGTTCCTATTATACTAGGAGCTCCAGATATAGCATTTCCACGAATAA  245
            ||||||||||||||||||||||||||||||| ||||||||||||||||||||||||||||
Sbjct  181  TTGGAAATTGATTAGTTCCTATTATACTAGGGGCTCCAGATATAGCATTTCCACGAATAA  240

Query  246  ATAATATAAGATTTTGGTTACTTCCTCCTTCTCTATTTCTACTAATTAATTCTTCATTAA  305
            ||||||||||||||||||||||||||||||||||||||||||||||||||||||||||||
Sbjct  241  ATAATATAAGATTTTGGTTACTTCCTCCTTCTCTATTTCTACTAATTAATTCTTCATTAA  300

Query  306  TCGAATCAGGAGCAGGTACAGGGTGAACAGTTTAcccccccTTATCATCAAATTTATCAC  365
            ||||||||||||||||||||||||||||||||||||||||||||||||||||||||||||
Sbjct  301  TCGAATCAGGAGCAGGTACAGGGTGAACAGTTTACCCCCCCTTATCATCAAATTTATCAC  360

Query  366  ACTACGGACCATCAGTAGATTTAGCTATTTTTTCTCTGCATCTTGCAGGTGCTTCTTCAA  425
            ||||||||||||||||||||||||||||||||||||||||||||||||||||||||||||
Sbjct  361  ACTACGGACCATCAGTAGATTTAGCTATTTTTTCTCTGCATCTTGCAGGTGCTTCTTCAA  420

Query  426  TTTTAGGTGCAATCAATTTCATTACAACTATTGTAAATATACGATCCATTGGAATAACAA  485
            ||||||||||||||||||||||||||||||||||||||||||||||||||||||||||||
Sbjct  421  TTTTAGGTGCAATCAATTTCATTACAACTATTGTAAATATACGATCCATTGGAATAACAA  480

Query  486  TAGAACGAATACCATTATTTGTATGATCTGTTTTAATTACTGCAATTTTATTATTATTAT  545
            ||||||||||||||||||||||||||||||||||||||||||||||||||||||||||||
Sbjct  481  TAGAACGAATACCATTATTTGTATGATCTGTTTTAATTACTGCAATTTTATTATTATTAT  540

Query  546  CTTTACCAGTCTTAGCTGGCGCAATTACAATATTATTAACTGATCGAAACTTTAATACAT  605
            ||||||||||||||||||||||||||||||||||||||||||||||||||||||||||||
Sbjct  541  CTTTACCAGTCTTAGCTGGCGCAATTACAATATTATTAACTGATCGAAACTTTAATACAT  600

Query  606  CATTCTTTGACCCTTCAGGAGGAGGTGACCCAATTCTATATCAA  649
            ||||||||||||||||||||||||||||||||||||||||||||
Sbjct  601  CATTCTTTGACCCTTCAGGAGGAGGTGACCCAATTCTATATCAA  644
```

>Rhipicephalus linnaei voucher P1/22_18-4 cytochrome c oxidase subunit I (COX1) gene, partial cds; mitochondrial
Sequence ID: OM984983.1 Length: 658
Range 1: 1 to 658

Score:1177 bits(637), Expect:0.0,
Identities:651/658(99%), Gaps:0/658(0%), Strand: Plus/Plus

```
Query  1    GACAATATACTTAATTTTTGGAGCATGATCCGGAATACTAGGATTAAGAATAAGAATATT  60
            ||||||||||||||||||||||||||||||||||||| ||||||||||||||||||||||
Sbjct  1    GACAATATACTTAATTTTTGGAGCATGATCCGGAATATTAGGATTAAGAATAAGAATATT  60

Query  61   AATCCGCATAGAATTAGGTCAACCAGGAACTTTAATTGGAAACGATCAAATTTATAATGT  120
            ||||||||||||||||||||||||||||||||||||||||||||||||||||||||||||
Sbjct  61   AATCCGCATAGAATTAGGTCAACCAGGAACTTTAATTGGAAACGATCAAATTTATAATGT  120

Query  121  AATTGTAACAGCACATGCATTTATTATAAttttttttATAGTTATACCAATTATAATCGG  180
            ||||||||||||||||||||||||||||||||||||||||||||||||||||||||||||
Sbjct  121  AATTGTAACAGCACATGCATTTATTATAATTTTTTTTATAGTTATACCAATTATAATCGG  180

Query  181  AGGATTTGGAAATTGATTAGTTCCTATTATACTAGGAGCTCCAGATATAGCATTTCCACG  240
            ||||||||||||||||||||||||||||||||||||||||||||||||||||||||||||
Sbjct  181  AGGATTTGGAAATTGATTAGTTCCTATTATACTAGGAGCTCCAGATATAGCATTTCCACG  240

Query  241  AATAAATAATATAAGATTTTGGTTACTTCCTCCTTCTCTATTTCTACTAATTAATTCTTC  300
            ||||||||||||||||||||||||||||||||||||||||||||||||||||||||||||
Sbjct  241  AATAAATAATATAAGATTTTGGTTACTTCCTCCTTCTCTATTTCTACTAATTAATTCTTC  300

Query  301  ATTAATCGAATCAGGAGCAGGTACAGGGTGAACAGTTTAcccccccTTATCATCAAATTT  360
            ||||||||||||||||||||||||||||||||||||||||||||||||||||||||||||
Sbjct  301  ATTAATCGAATCAGGAGCAGGTACAGGGTGAACAGTTTACCCCCCCTTATCATCAAATTT  360

Query  361  ATCACACTACGGACCATCAGTAGATTTAGCTATTTTTTCTCTGCATCTTGCAGGTGCTTC  420
            |||||||||||| |||||||||||||||||||| ||||||||||||||||| ||||||||
Sbjct  361  ATCACACTACGGGCCATCAGTAGATTTAGCTATCTTTTCTCTGCATCTTGCTGGTGCTTC  420

Query  421  TTCAATTTTAGGTGCAATCAATTTCATTACAACTATTGTAAATATACGATCCATTGGAAT  480
            |||||||||||||||||| |||||||||||||||||||||||||||||||||||||||||
Sbjct  421  TTCAATTTTAGGTGCAATTAATTTCATTACAACTATTGTAAATATACGATCCATTGGAAT  480

Query  481  AACAATAGAACGAATACCATTATTTGTATGATCTGTTTTAATTACTGCAATTTTATTATT  540
            ||||||||||||||||||||||||||||||||||||||||||||||||||||||||||||
Sbjct  481  AACAATAGAACGAATACCATTATTTGTATGATCTGTTTTAATTACTGCAATTTTATTATT  540

Query  541  ATTATCTTTACCAGTCTTAGCTGGCGCAATTACAATATTATTAACTGATCGAAACTTTAA  600
            |||||||||||||||||||||||| |||||||||||||||||||||||||||||||||||
Sbjct  541  ATTATCTTTACCAGTCTTAGCTGGTGCAATTACAATATTATTAACTGATCGAAACTTTAA  600

Query  601  TACATCATTCTTTGACCCTTCAGGAGGAGGTGACCCAATTCTATATCAACATTTATTT  658
            |||||||||||||||||||||||||||||||||||||||||| |||||||||||||||
```

```
Sbjct  601   TACATCATTCTTTGACCCTTCAGGAGGAGGTGACCCAATTCTGTATCAACATTTATTT  658
```

>Rhipicephalus sanguineus isolate GHM1 cytochrome oxidase subunit I (cox1) gene, partial cds; mitochondrial
Sequence ID: JX416302.1 Length: 802
>Rhipicephalus sanguineus isolate GPF1 cytochrome oxidase subunit I (cox1) gene, partial cds; mitochondrial
Sequence ID: JX416305.1 Length: 802
>Rhipicephalus sanguineus isolate GPF2 cytochrome oxidase subunit I (cox1) gene, partial cds; mitochondrial
Sequence ID: JX416306.1 Length: 802
>Rhipicephalus sanguineus isolate SZM1 cytochrome oxidase subunit I (cox1) gene, partial cds; mitochondrial
Sequence ID: JX416307.1 Length: 802
Range 1: 1 to 637

Score:1177 bits(637), Expect:0.0,
Identities:637/637(100%), Gaps:0/637(0%), Strand: Plus/Plus

```
Query  22    AGCATGATCCGGAATACTAGGATTAAGAATAAGAATATTAATCCGCATAGAATTAGGTCA  81
             ||||||||||||||||||||||||||||||||||||||||||||||||||||||||||||
Sbjct  1     AGCATGATCCGGAATACTAGGATTAAGAATAAGAATATTAATCCGCATAGAATTAGGTCA  60

Query  82    ACCAGGAACTTTAATTGGAAACGATCAAATTTATAATGTAATTGTAACAGCACATGCATT  141
             ||||||||||||||||||||||||||||||||||||||||||||||||||||||||||||
Sbjct  61    ACCAGGAACTTTAATTGGAAACGATCAAATTTATAATGTAATTGTAACAGCACATGCATT  120

Query  142   TATTATAAttttttttATAGTTATACCAATTATAATCGGAGGATTTGGAAATTGATTAGT  201
             ||||||||||||||||||||||||||||||||||||||||||||||||||||||||||||
Sbjct  121   TATTATAATTTTTTTTATAGTTATACCAATTATAATCGGAGGATTTGGAAATTGATTAGT  180

Query  202   TCCTATTATACTAGGAGCTCCAGATATAGCATTTCCACGAATAAATAATATAAGATTTTG  261
             ||||||||||||||||||||||||||||||||||||||||||||||||||||||||||||
Sbjct  181   TCCTATTATACTAGGAGCTCCAGATATAGCATTTCCACGAATAAATAATATAAGATTTTG  240

Query  262   GTTACTTCCTCCTTCTCTATTTCTACTAATTAATTCTTCATTAATCGAATCAGGAGCAGG  321
             ||||||||||||||||||||||||||||||||||||||||||||||||||||||||||||
Sbjct  241   GTTACTTCCTCCTTCTCTATTTCTACTAATTAATTCTTCATTAATCGAATCAGGAGCAGG  300

Query  322   TACAGGGTGAACAGTTTAcccccccTTATCATCAAATTTATCACACTACGGACCATCAGT  381
             ||||||||||||||||||||||||||||||||||||||||||||||||||||||||||||
Sbjct  301   TACAGGGTGAACAGTTTACCCCCCCTTATCATCAAATTTATCACACTACGGACCATCAGT  360

Query  382   AGATTTAGCTATTTTTTCTCTGCATCTTGCAGGTGCTTCTTCAATTTTAGGTGCAATCAA  441
             ||||||||||||||||||||||||||||||||||||||||||||||||||||||||||||
Sbjct  361   AGATTTAGCTATTTTTTCTCTGCATCTTGCAGGTGCTTCTTCAATTTTAGGTGCAATCAA  420

Query  442   TTTCATTACAACTATTGTAAATATACGATCCATTGGAATAACAATAGAACGAATACCATT  501
             ||||||||||||||||||||||||||||||||||||||||||||||||||||||||||||
Sbjct  421   TTTCATTACAACTATTGTAAATATACGATCCATTGGAATAACAATAGAACGAATACCATT  480
```

```
Query  502   ATTTGTATGATCTGTTTTAATTACTGCAATTTTATTATTATTATCTTTACCAGTCTTAGC  561
             ||||||||||||||||||||||||||||||||||||||||||||||||||||||||||||
Sbjct  481   ATTTGTATGATCTGTTTTAATTACTGCAATTTTATTATTATTATCTTTACCAGTCTTAGC  540

Query  562   TGGCGCAATTACAATATTATTAACTGATCGAAACTTTAATACATCATTCTTTGACCCTTC  621
             ||||||||||||||||||||||||||||||||||||||||||||||||||||||||||||
Sbjct  541   TGGCGCAATTACAATATTATTAACTGATCGAAACTTTAATACATCATTCTTTGACCCTTC  600

Query  622   AGGAGGAGGTGACCCAATTCTATATCAACATTTATTT  658
             |||||||||||||||||||||||||||||||||||||
Sbjct  601   AGGAGGAGGTGACCCAATTCTATATCAACATTTATTT  637
```

>Rhipicephalus sanguineus isolate CHM1 cytochrome oxidase subunit I (cox1) gene, partial cds; mitochondrial
Sequence ID: JX416298.1 Length: 802
Range 1: 1 to 637

Score:1177 bits(637), Expect:0.0,
Identities:637/637(100%), Gaps:0/637(0%), Strand: Plus/Plus

```
Query  22    AGCATGATCCGGAATACTAGGATTAAGAATAAGAATATTAATCCGCATAGAATTAGGTCA  81
             ||||||||||||||||||||||||||||||||||||||||||||||||||||||||||||
Sbjct  1     AGCATGATCCGGAATACTAGGATTAAGAATAAGAATATTAATCCGCATAGAATTAGGTCA  60

Query  82    ACCAGGAACTTTAATTGGAAACGATCAAATTTATAATGTAATTGTAACAGCACATGCATT  141
             ||||||||||||||||||||||||||||||||||||||||||||||||||||||||||||
Sbjct  61    ACCAGGAACTTTAATTGGAAACGATCAAATTTATAATGTAATTGTAACAGCACATGCATT  120

Query  142   TATTATAAttttttttATAGTTATACCAATTATAATCGGAGGATTTGGAAATTGATTAGT  201
             ||||||||||||||||||||||||||||||||||||||||||||||||||||||||||||
Sbjct  121   TATTATAATTTTTTTTATAGTTATACCAATTATAATCGGAGGATTTGGAAATTGATTAGT  180

Query  202   TCCTATTATACTAGGAGCTCCAGATATAGCATTTCCACGAATAAATAATATAAGATTTTG  261
             ||||||||||||||||||||||||||||||||||||||||||||||||||||||||||||
Sbjct  181   TCCTATTATACTAGGAGCTCCAGATATAGCATTTCCACGAATAAATAATATAAGATTTTG  240

Query  262   GTTACTTCCTCCTTCTCTATTTCTACTAATTAATTCTTCATTAATCGAATCAGGAGCAGG  321
             ||||||||||||||||||||||||||||||||||||||||||||||||||||||||||||
Sbjct  241   GTTACTTCCTCCTTCTCTATTTCTACTAATTAATTCTTCATTAATCGAATCAGGAGCAGG  300

Query  322   TACAGGGTGAACAGTTTAcccccccTTATCATCAAATTTATCACACTACGGACCATCAGT  381
             ||||||||||||||||||||||||||||||||||||||||||||||||||||||||||||
Sbjct  301   TACAGGGTGAACAGTTTACCCCCCCTTATCATCAAATTTATCACACTACGGACCATCAGT  360

Query  382   AGATTTAGCTATTTTTTCTCTGCATCTTGCAGGTGCTTCTTCAATTTTAGGTGCAATCAA  441
             ||||||||||||||||||||||||||||||||||||||||||||||||||||||||||||
Sbjct  361   AGATTTAGCTATTTTTTCTCTGCATCTTGCAGGTGCTTCTTCAATTTTAGGTGCAATCAA  420

Query  442   TTTCATTACAACTATTGTAAATATACGATCCATTGGAATAACAATAGAACGAATACCATT  501
             ||||||||||||||||||||||||||||||||||||||||||||||||||||||||||||
Sbjct  421   TTTCATTACAACTATTGTAAATATACGATCCATTGGAATAACAATAGAACGAATACCATT  480
```

```
Query  502  ATTTGTATGATCTGTTTTAATTACTGCAATTTTATTATTATTATCTTTACCAGTCTTAGC  561
            ||||||||||||||||||||||||||||||||||||||||||||||||||||||||||||
Sbjct  481  ATTTGTATGATCTGTTTTAATTACTGCAATTTTATTATTATTATCTTTACCAGTCTTAGC  540

Query  562  TGGCGCAATTACAATATTATTAACTGATCGAAACTTTAATACATCATTCTTTGACCCTTC  621
            ||||||||||||||||||||||||||||||||||||||||||||||||||||||||||||
Sbjct  541  TGGCGCAATTACAATATTATTAACTGATCGAAACTTTAATACATCATTCTTTGACCCTTC  600

Query  622  AGGAGGAGGTGACCCAATTCTATATCAACATTTATTT  658
            |||||||||||||||||||||||||||||||||||||
Sbjct  601  AGGAGGAGGTGACCCAATTCTATATCAACATTTATTT  637
```

```
>Rhipicephalus sanguineus voucher AMMS-RS-5 cytochrome c oxidase subunit I-like
(COI) gene, partial sequence; mitochondrial
Sequence ID: HM193873.1 Length: 670
Range 1: 1 to 642

Score:1177 bits(637), Expect:0.0,
Identities:642/644(99%),  Gaps:2/644(0%), Strand: Plus/Plus

Query  15   TTTTTGGAGCATGATCCGGAATACTAGGATTAAGAATAAGAATATTAATCCGCATAGAAT  74
            |||||||| |||||||||| |||||||||||||||||||||||||||||||||||||||||
Sbjct  1    TTTTTGGA-CATGATCCGG-ATACTAGGATTAAGAATAAGAATATTAATCCGCATAGAAT  58

Query  75   TAGGTCAACCAGGAACTTTAATTGGAAACGATCAAATTTATAATGTAATTGTAACAGCAC  134
            ||||||||||||||||||||||||||||||||||||||||||||||||||||||||||||
Sbjct  59   TAGGTCAACCAGGAACTTTAATTGGAAACGATCAAATTTATAATGTAATTGTAACAGCAC  118

Query  135  ATGCATTTATTATAAttttttttATAGTTATACCAATTATAATCGGAGGATTTGGAAATT  194
            ||||||||||||||||||||||||||||||||||||||||||||||||||||||||||||
Sbjct  119  ATGCATTTATTATAATTTTTTTTATAGTTATACCAATTATAATCGGAGGATTTGGAAATT  178

Query  195  GATTAGTTCCTATTATACTAGGAGCTCCAGATATAGCATTTCCACGAATAAATAATATAA  254
            ||||||||||||||||||||||||||||||||||||||||||||||||||||||||||||
Sbjct  179  GATTAGTTCCTATTATACTAGGAGCTCCAGATATAGCATTTCCACGAATAAATAATATAA  238

Query  255  GATTTTGGTTACTTCCTCCTTCTCTATTTCTACTAATTAATTCTTCATTAATCGAATCAG  314
            ||||||||||||||||||||||||||||||||||||||||||||||||||||||||||||
Sbjct  239  GATTTTGGTTACTTCCTCCTTCTCTATTTCTACTAATTAATTCTTCATTAATCGAATCAG  298

Query  315  GAGCAGGTACAGGGTGAACAGTTTAcccccccTTATCATCAAATTTATCACACTACGGAC  374
            ||||||||||||||||||||||||||||||||||||||||||||||||||||||||||||
Sbjct  299  GAGCAGGTACAGGGTGAACAGTTTACCCCCCCTTATCATCAAATTTATCACACTACGGAC  358

Query  375  CATCAGTAGATTTAGCTATTTTTTCTCTGCATCTTGCAGGTGCTTCTTCAATTTTAGGTG  434
            ||||||||||||||||||||||||||||||||||||||||||||||||||||||||||||
Sbjct  359  CATCAGTAGATTTAGCTATTTTTTCTCTGCATCTTGCAGGTGCTTCTTCAATTTTAGGTG  418

Query  435  CAATCAATTTCATTACAACTATTGTAAATATACGATCCATTGGAATAACAATAGAACGAA  494
            ||||||||||||||||||||||||||||||||||||||||||||||||||||||||||||
```

```
Sbjct  419   CAATCAATTTCATTACAACTATTGTAAATATACGATCCATTGGAATAACAATAGAACGAA  478

Query  495   TACCATTATTTGTATGATCTGTTTTAATTACTGCAATTTTATTATTATTATCTTTACCAG  554
             ||||||||||||||||||||||||||||||||||||||||||||||||||||||||||||
Sbjct  479   TACCATTATTTGTATGATCTGTTTTAATTACTGCAATTTTATTATTATTATCTTTACCAG  538

Query  555   TCTTAGCTGGCGCAATTACAATATTATTAACTGATCGAAACTTTAATACATCATTCTTTG  614
             ||||||||||||||||||||||||||||||||||||||||||||||||||||||||||||
Sbjct  539   TCTTAGCTGGCGCAATTACAATATTATTAACTGATCGAAACTTTAATACATCATTCTTTG  598

Query  615   ACCCTTCAGGAGGAGGTGACCCAATTCTATATCAACATTTATTT  658
             ||||||||||||||||||||||||||||||||||||||||||||
Sbjct  599   ACCCTTCAGGAGGAGGTGACCCAATTCTATATCAACATTTATTT  642
```

>Rhipicephalus sanguineus isolate LIC6767D cytochrome oxidase subunit 1 (COI) gene, partial cds; mitochondrial
Sequence ID: KX383820.1 Length: 768
Range 1: 1 to 642

Score:1175 bits(636), Expect:0.0,
Identities:640/642(99%), Gaps:0/642(0%), Strand: Plus/Plus

```
Query  17    TTTGGAGCATGATCCGGAATACTAGGATTAAGAATAAGAATATTAATCCGCATAGAATTA  76
             ||||||||||||||||||||||||||||||||||||||||||||||||||||||||||||
Sbjct  1     TTTGGAGCATGATCCGGAATACTAGGATTAAGAATAAGAATATTAATCCGCATAGAATTA  60

Query  77    GGTCAACCAGGAACTTTAATTGGAAACGATCAAATTTATAATGTAATTGTAACAGCACAT  136
             ||||||||||||||||||||||||||||||||||||||||||||||||||||||||||||
Sbjct  61    GGTCAACCAGGAACTTTAATTGGAAACGATCAAATTTATAATGTAATTGTAACAGCACAT  120

Query  137   GCATTTATTATAAttttttttATAGTTATACCAATTATAATCGGAGGATTTGGAAATTGA  196
             ||||||||||||||||||||||||||||||||||||||||||||||||||||||||||||
Sbjct  121   GCATTTATTATAATTTTTTTTATAGTTATACCAATTATAATCGGAGGATTTGGAAATTGA  180

Query  197   TTAGTTCCTATTATACTAGGAGCTCCAGATATAGCATTTCCACGAATAAATAATATAAGA  256
             ||||||||||||||||||||||||||||||||||||||||||||||||||||||||||||
Sbjct  181   TTAGTTCCTATTATACTAGGAGCTCCAGATATAGCATTTCCACGAATAAATAATATAAGA  240

Query  257   TTTTGGTTACTTCCTCCTTCTCTATTTCTACTAATTAATTCTTCATTAATCGAATCAGGA  316
             ||||||||||||||||||||||||||||||||||||||||||||||||||||||||||||
Sbjct  241   TTTTGGTTACTTCCTCCTTCTCTATTTCTACTAATTAATTCTTCATTAATCGAATCAGGA  300

Query  317   GCAGGTACAGGGTGAACAGTTTAcccccccTTATCATCAAATTTATCACACTACGGACCA  376
             ||||||||||||||||||||||||||||||||||||||||||||||||||||||||||||
Sbjct  301   GCAGGTACAGGGTGAACAGTTTACCCCCCCTTATCATCAAATTTATCACACTACGGACCA  360

Query  377   TCAGTAGATTTAGCTATTTTTTCTCTGCATCTTGCAGGTGCTTCTTCAATTTTAGGTGCA  436
             |||||||||||||||||||||||||| |||||||||||||||||||||||||||||||||
Sbjct  361   TCAGTAGATTTAGCTATTTTTTCTCTACATCTTGCAGGTGCTTCTTCAATTTTAGGTGCA  420

Query  437   ATCAATTTCATTACAACTATTGTAAATATACGATCCATTGGAATAACAATAGAACGAATA  496
```

```
                ||||||||||||||||||||||||||||||||||||||||||||||||||||||||||||
Sbjct  421  ATCAATTTCATTACAACTATTGTAAATATACGATCCATTGGAATAACAATAGAACGAATA  480

Query  497  CCATTATTTGTATGATCTGTTTTAATTACTGCAATTTTATTATTATTATCTTTACCAGTC  556
            ||||||||||||||||||||||||||||||||||||||||||||||||||||||||||||
Sbjct  481  CCATTATTTGTATGATCTGTTTTAATTACTGCAATTTTATTATTATTATCTTTACCAGTC  540

Query  557  TTAGCTGGCGCAATTACAATATTATTAACTGATCGAAACTTTAATACATCATTCTTTGAC  616
            ||||||||||||||||||||||||||||||||||||||||||||||||||||||||||||
Sbjct  541  TTAGCTGGCGCAATTACAATATTATTAACTGATCGAAACTTTAATACATCATTCTTTGAC  600

Query  617  CCTTCAGGAGGAGGTGACCCAATTCTATATCAACATTTATTT  658
            ||||||||||| ||||||||||||||||||||||||||||||
Sbjct  601  CCTTCAGGAGGGGGTGACCCAATTCTATATCAACATTTATTT  642
```

>Rhipicephalus sanguineus isolate LIC5766G cytochrome oxidase subunit 1 (COI) gene, partial cds; mitochondrial
Sequence ID: KX383816.1 Length: 724
Range 1: 1 to 642

Score:1171 bits(634), Expect:0.0,
Identities:639/642(99%), Gaps:0/642(0%), Strand: Plus/Plus

```
Query  17   TTTGGAGCATGATCCGGAATACTAGGATTAAGAATAAGAATATTAATCCGCATAGAATTA  76
            ||||||||||||||||||||||||||||||||||||||||||||||||||||||||||||
Sbjct  1    TTTGGAGCATGATCCGGAATACTAGGATTAAGAATAAGAATATTAATCCGCATAGAATTA  60

Query  77   GGTCAACCAGGAACTTTAATTGGAAACGATCAAATTTATAATGTAATTGTAACAGCACAT  136
            |||||||||||||||||||||||||||||| |||||||||||||||||||||||||||||
Sbjct  61   GGTCAACCAGGAACTTTAATTGGAAACGATSAAATTTATAATGTAATTGTAACAGCACAT  120

Query  137  GCATTTATTATAAttttttttATAGTTATACCAATTATAATCGGAGGATTTGGAAATTGA  196
            ||||||||||||||||||||||||||||||||||||||||||||||||||||||||||||
Sbjct  121  GCATTTATTATAATTTTTTTTATAGTTATACCAATTATAATCGGAGGATTTGGAAATTGA  180

Query  197  TTAGTTCCTATTATACTAGGAGCTCCAGATATAGCATTTCCACGAATAAATAATATAAGA  256
            ||||||||||||||||||||||||||||||||||||||||||||||||||||||||||||
Sbjct  181  TTAGTTCCTATTATACTAGGAGCTCCAGATATAGCATTTCCACGAATAAATAATATAAGA  240

Query  257  TTTTGGTTACTTCCTCCTTCTCTATTTCTACTAATTAATTCTTCATTAATCGAATCAGGA  316
            ||||||||||||||||||||||||||||||||||||||||||||||||||||||||||||
Sbjct  241  TTTTGGTTACTTCCTCCTTCTCTATTTCTACTAATTAATTCTTCATTAATCGAATCAGGA  300

Query  317  GCAGGTACAGGGTGAACAGTTTAcccccccTTATCATCAAATTTATCACACTACGGACCA  376
            ||||||||||||||||||||||||||||||||||||||||||||||||||||||||||||
Sbjct  301  GCAGGTACAGGGTGAACAGTTTACCCCCCCTTATCATCAAATTTATCACACTACGGACCA  360

Query  377  TCAGTAGATTTAGCTATTTTTTCTCTGCATCTTGCAGGTGCTTCTTCAATTTTAGGTGCA  436
            |||||||||||||||||||||||||| |||||||||||||||||||||||||||||||||
Sbjct  361  TCAGTAGATTTAGCTATTTTTTCTCTACATCTTGCAGGTGCTTCTTCAATTTTAGGTGCA  420
```

```
Query  437   ATCAATTTCATTACAACTATTGTAAATATACGATCCATTGGAATAACAATAGAACGAATA  496
             ||||||||||||||||||||||||||||||||||||||||||||||||||||||||||||
Sbjct  421   ATCAATTTCATTACAACTATTGTAAATATACGATCCATTGGAATAACAATAGAACGAATA  480

Query  497   CCATTATTTGTATGATCTGTTTTAATTACTGCAATTTTATTATTATTATCTTTACCAGTC  556
             ||||||||||||||||||||||||||||||||||||||||||||||||||||||||||||
Sbjct  481   CCATTATTTGTATGATCTGTTTTAATTACTGCAATTTTATTATTATTATCTTTACCAGTC  540

Query  557   TTAGCTGGCGCAATTACAATATTATTAACTGATCGAAACTTTAATACATCATTCTTTGAC  616
             ||||||||||||||||||||||||||||||||||||||||||||||||||||||||||||
Sbjct  541   TTAGCTGGCGCAATTACAATATTATTAACTGATCGAAACTTTAATACATCATTCTTTGAC  600

Query  617   CCTTCAGGAGGAGGTGACCCAATTCTATATCAACATTTATTT  658
             ||||||||||| ||||||||||||||||||||||||||||||
Sbjct  601   CCTTCAGGAGGGGGTGACCCAATTCTATATCAACATTTATTT  642
```

>Rhipicephalus linnaei voucher P1/22_18-5 cytochrome c oxidase subunit I (COX1) gene, partial cds; mitochondrial
Sequence ID: OM984984.1 Length: 658
Range 1: 1 to 658

Score:1171 bits(634), Expect:0.0,
Identities:650/658(99%), Gaps:0/658(0%), Strand: Plus/Plus

```
Query  1     GACAATATACTTAATTTTTGGAGCATGATCCGGAATACTAGGATTAAGAATAAGAATATT  60
             ||||||||||||||||||||||||||||||||||||| ||||||||||||||||||||||
Sbjct  1     GACAATATACTTAATTTTTGGAGCATGATCCGGAATATTAGGATTAAGAATAAGAATATT  60

Query  61    AATCCGCATAGAATTAGGTCAACCAGGAACTTTAATTGGAAACGATCAAATTTATAATGT  120
             ||||||||||||||||||||||||||||||||||||||||||||||||||||||||||||
Sbjct  61    AATCCGCATAGAATTAGGTCAACCAGGAACTTTAATTGGAAACGATCAAATTTATAATGT  120

Query  121   AATTGTAACAGCACATGCATTTATTATAAttttttttATAGTTATACCAATTATAATCGG  180
             ||||||||||||||||||||||||||||||||||||||||||||||||||||||||||||
Sbjct  121   AATTGTAACAGCACATGCATTTATTATAATTTTTTTTATAGTTATACCAATTATAATCGG  180

Query  181   AGGATTTGGAAATTGATTAGTTCCTATTATACTAGGAGCTCCAGATATAGCATTTCCACG  240
             ||||||||||||||||||||||||||||||||||||||||||||||||||||||||||||
Sbjct  181   AGGATTTGGAAATTGATTAGTTCCTATTATACTAGGAGCTCCAGATATAGCATTTCCACG  240

Query  241   AATAAATAATATAAGATTTTGGTTACTTCCTCCTTCTCTATTTCTACTAATTAATTCTTC  300
             ||||||||||||||||||||||||||||||||||||||||||||||||||||||||||||
Sbjct  241   AATAAATAATATAAGATTTTGGTTACTTCCTCCTTCTCTATTTCTACTAATTAATTCTTC  300

Query  301   ATTAATCGAATCAGGAGCAGGTACAGGGTGAACAGTTTAcccccccTTATCATCAAATTT  360
             ||||||||||||||||||||||||||||||||||||||||||||||||||||||||||||
Sbjct  301   ATTAATCGAATCAGGAGCAGGTACAGGGTGAACAGTTTACCCCCCCTTATCATCAAATTT  360

Query  361   ATCACACTACGGACCATCAGTAGATTTAGCTATTTTTTCTCTGCATCTTGCAGGTGCTTC  420
             |||||||||||| |||||||||||||||||||| ||||||||||||||||| ||||||||
Sbjct  361   ATCACACTACGGGCCATCAGTAGATTTAGCTATCTTTTCTCTGCATCTTGCTGGTGCTTC  420
```

```
Query  421   TTCAATTTTAGGTGCAATCAATTTCATTACAACTATTGTAAATATACGATCCATTGGAAT  480
             |||||||||||||||||| |||||||||||||||||||||||||||||||||||||||||
Sbjct  421   TTCAATTTTAGGTGCAATTAATTTCATTACAACTATTGTAAATATACGATCCATTGGAAT  480

Query  481   AACAATAGAACGAATACCATTATTTGTATGATCTGTTTTAATTACTGCAATTTTATTATT  540
             ||||||||||||||||||||||||||||||||||||||||||||||||||||||||||||
Sbjct  481   AACAATAGAACGAATACCATTATTTGTATGATCTGTTTTAATTACTGCAATTTTATTATT  540

Query  541   ATTATCTTTACCAGTCTTAGCTGGCGCAATTACAATATTATTAACTGATCGAAACTTTAA  600
             |||||||||||||||||||||||| |||||||||||||||||||||||||||||||||||
Sbjct  541   ATTATCTTTACCAGTCTTAGCTGGTGCAATTACAATATTATTAACTGATCGAAACTTTAA  600

Query  601   TACATCATTCTTTGACCCTTCAGGAGGAGGTGACCCAATTCTATATCAACATTTATTT  658
             ||||||||||||||||||||||||||| |||||||||||||| |||||||||||||||
Sbjct  601   TACATCATTCTTTGACCCTTCAGGAGGGGGTGACCCAATTCTGTATCAACATTTATTT  658
```

>Rhipicephalus linnaei voucher P1/22_8-1 cytochrome c oxidase subunit I (COX1) gene, partial cds; mitochondrial
Sequence ID: OM984974.1 Length: 658
Range 1: 1 to 658

Score:1171 bits(634), Expect:0.0,
Identities:650/658(99%), Gaps:0/658(0%), Strand: Plus/Plus

```
Query  1     GACAATATACTTAATTTTTGGAGCATGATCCGGAATACTAGGATTAAGAATAAGAATATT  60
             ||||||||||||||||||||||||||||||||||||| ||||||||||||||||||||||
Sbjct  1     GACAATATACTTAATTTTTGGAGCATGATCCGGAATATTAGGATTAAGAATAAGAATATT  60

Query  61    AATCCGCATAGAATTAGGTCAACCAGGAACTTTAATTGGAAACGATCAAATTTATAATGT  120
             ||||||||||||||||||||||||||||||||||||||||||||||||||||||||||||
Sbjct  61    AATCCGCATAGAATTAGGTCAACCAGGAACTTTAATTGGAAACGATCAAATTTATAATGT  120

Query  121   AATTGTAACAGCACATGCATTTATTATAAttttttttATAGTTATACCAATTATAATCGG  180
             ||||||||||||||||||||||||||||||||||||||||||||||||||||||||||||
Sbjct  121   AATTGTAACAGCACATGCATTTATTATAATTTTTTTTATAGTTATACCAATTATAATCGG  180

Query  181   AGGATTTGGAAATTGATTAGTTCCTATTATACTAGGAGCTCCAGATATAGCATTTCCACG  240
             ||||||||||||||||||||||||||||||||||||||||||||||||||||||||||||
Sbjct  181   AGGATTTGGAAATTGATTAGTTCCTATTATACTAGGAGCTCCAGATATAGCATTTCCACG  240

Query  241   AATAAATAATATAAGATTTTGGTTACTTCCTCCTTCTCTATTTCTACTAATTAATTCTTC  300
             ||||||||||||||||||||||||||||||||||||||||||||||||||||||||||||
Sbjct  241   AATAAATAATATAAGATTTTGGTTACTTCCTCCTTCTCTATTTCTACTAATTAATTCTTC  300

Query  301   ATTAATCGAATCAGGAGCAGGTACAGGGTGAACAGTTTAcccccccTTATCATCAAATTT  360
             ||||||||||||||||||||||||||||||||||||||||||||||||||||||||||||
Sbjct  301   ATTAATCGAATCAGGAGCAGGTACAGGGTGAACAGTTTACCCCCCCTTATCATCAAATTT  360

Query  361   ATCACACTACGGACCATCAGTAGATTTAGCTATTTTTCTCTGCATCTTGCAGGTGCTTC  420
             |||||||||||| |||||||||||||||||||| ||||||||||||||||| ||||||||
```

```
Sbjct  361   ATCACACTACGGGCCATCAGTAGATTTAGCTATCTTTTCTCTGCATCTTGCTGGTGCTTC  420

Query  421   TTCAATTTTAGGTGCAATCAATTTCATTACAACTATTGTAAATATACGATCCATTGGAAT  480
             |||||||||||||||||| |||||||||||||||||||||||||||||||||||||||||
Sbjct  421   TTCAATTTTAGGTGCAATTAATTTCATTACAACTATTGTAAATATACGATCCATTGGAAT  480

Query  481   AACAATAGAACGAATACCATTATTTGTATGATCTGTTTTAATTACTGCAATTTTATTATT  540
             ||||||||||||||||||||||||||||||||||||||||||||||||||||||||||||
Sbjct  481   AACAATAGAACGAATACCATTATTTGTATGATCTGTTTTAATTACTGCAATTTTATTATT  540

Query  541   ATTATCTTTACCAGTCTTAGCTGGCGCAATTACAATATTATTAACTGATCGAAACTTTAA  600
             |||||||||||||||||||||||| |||||||||||||||||||||||||||||||||||
Sbjct  541   ATTATCTTTACCAGTCTTAGCTGGTGCAATTACAATATTATTAACTGATCGAAACTTTAA  600

Query  601   TACATCATTCTTTGACCCTTCAGGAGGAGGTGACCCAATTCTATATCAACATTTATTT  658
             ||||||||||||||||||||||||||| |||||||||||||| |||||||||||||||
Sbjct  601   TACATCATTCTTTGACCCTTCAGGAGGGGGTGACCCAATTCTGTATCAACATTTATTT  658
```

>Rhipicephalus linnaei voucher P1/22_6-2 cytochrome c oxidase subunit I (COX1) gene, partial cds; mitochondrial
Sequence ID: OM984973.1 Length: 658
Range 1: 1 to 658

Score:1171 bits(634), Expect:0.0,
Identities:650/658(99%), Gaps:0/658(0%), Strand: Plus/Plus

```
Query  1     GACAATATACTTAATTTTTGGAGCATGATCCGGAATACTAGGATTAAGAATAAGAATATT  60
             ||||||||||||||||||||||||||||||||||||| ||||||||||||||||||||||
Sbjct  1     GACAATATACTTAATTTTTGGAGCATGATCCGGAATATTAGGATTAAGAATAAGAATATT  60

Query  61    AATCCGCATAGAATTAGGTCAACCAGGAACTTTAATTGGAAACGATCAAATTTATAATGT  120
             ||||||||||||||||||||||||||||||||||||||||||||||||||||||||||||
Sbjct  61    AATCCGCATAGAATTAGGTCAACCAGGAACTTTAATTGGAAACGATCAAATTTATAATGT  120

Query  121   AATTGTAACAGCACATGCATTTATTATAAttttttttATAGTTATACCAATTATAATCGG  180
             ||||||||||||||||||||||||||||||||||||||||||||||||||||||||||||
Sbjct  121   AATTGTAACAGCACATGCATTTATTATAATTTTTTTTATAGTTATACCAATTATAATCGG  180

Query  181   AGGATTTGGAAATTGATTAGTTCCTATTATACTAGGAGCTCCAGATATAGCATTTCCACG  240
             ||||||||||||||||||||||||||||||||||||||||||||||||||||||||||||
Sbjct  181   AGGATTTGGAAATTGATTAGTTCCTATTATACTAGGAGCTCCAGATATAGCATTTCCACG  240

Query  241   AATAAATAATATAAGATTTTGGTTACTTCCTCCTTCTCTATTTCTACTAATTAATTCTTC  300
             ||||||||||||||||||||||||||||||||||||||||||||||||||||||||||||
Sbjct  241   AATAAATAATATAAGATTTTGGTTACTTCCTCCTTCTCTATTTCTACTAATTAATTCTTC  300

Query  301   ATTAATCGAATCAGGAGCAGGTACAGGGTGAACAGTTTAcccccccTTATCATCAAATTT  360
             ||||||||||||||||||||||||||||||||||||||||||||||||||||||||||||
Sbjct  301   ATTAATCGAATCAGGAGCAGGTACAGGGTGAACAGTTTACCCCCCCTTATCATCAAATTT  360

Query  361   ATCACACTACGGACCATCAGTAGATTTAGCTATTTTTTCTCTGCATCTTGCAGGTGCTTC  420
```

```
                ||||||||||||| |||||||||||||||||||| ||||||||||||||||| ||||||||
Sbjct  361   ATCACACTACGGGCCATCAGTAGATTTAGCTATCTTTTCTCTGCATCTTGCTGGTGCTTC  420

Query  421   TTCAATTTTAGGTGCAATCAATTTCATTACAACTATTGTAAATATACGATCCATTGGAAT  480
             |||||||||||||||||| |||||||||||||||||||||||||||||||||||||||||
Sbjct  421   TTCAATTTTAGGTGCAATTAATTTCATTACAACTATTGTAAATATACGATCCATTGGAAT  480

Query  481   AACAATAGAACGAATACCATTATTTGTATGATCTGTTTTAATTACTGCAATTTTATTATT  540
             ||||||||||||||||||||||||||||||||||||||||||||||||||||||||||||
Sbjct  481   AACAATAGAACGAATACCATTATTTGTATGATCTGTTTTAATTACTGCAATTTTATTATT  540

Query  541   ATTATCTTTACCAGTCTTAGCTGGCGCAATTACAATATTATTAACTGATCGAAACTTTAA  600
             |||||||||||||||||||||||| |||||||||||||||||||||||||||||||||||
Sbjct  541   ATTATCTTTACCAGTCTTAGCTGGTGCAATTACAATATTATTAACTGATCGAAACTTTAA  600

Query  601   TACATCATTCTTTGACCCTTCAGGAGGAGGTGACCCAATTCTATATCAACATTTATTT  658
             ||||||||||||||||||||||||||| |||||||||||||| |||||||||||||||
Sbjct  601   TACATCATTCTTTGACCCTTCAGGAGGGGGTGACCCAATTCTGTATCAACATTTATTT  658
```

>Rhipicephalus sanguineus isolate SZM2 cytochrome oxidase subunit I (cox1) gene, partial cds; mitochondrial
Sequence ID: JX416308.1 Length: 802
Range 1: 1 to 637

Score:1171 bits(634), Expect:0.0,
Identities:636/637(99%), Gaps:0/637(0%), Strand: Plus/Plus

```
Query  22    AGCATGATCCGGAATACTAGGATTAAGAATAAGAATATTAATCCGCATAGAATTAGGTCA  81
             ||||||||||||||||||||||||||||||||||||||||||||||||||||||||||||
Sbjct  1     AGCATGATCCGGAATACTAGGATTAAGAATAAGAATATTAATCCGCATAGAATTAGGTCA  60

Query  82    ACCAGGAACTTTAATTGGAAACGATCAAATTTATAATGTAATTGTAACAGCACATGCATT  141
             ||||||||||||||||||||||||||||||||||||||||||||||||||||||||||||
Sbjct  61    ACCAGGAACTTTAATTGGAAACGATCAAATTTATAATGTAATTGTAACAGCACATGCATT  120

Query  142   TATTATAAtttttttATAGTTATACCAATTATAATCGGAGGATTTGGAAATTGATTAGT  201
             ||||||||||||||||||||||||||||||||||||||||||||||||||||||||||||
Sbjct  121   TATTATAATTTTTTTTATAGTTATACCAATTATAATCGGAGGATTTGGAAATTGATTAGT  180

Query  202   TCCTATTATACTAGGAGCTCCAGATATAGCATTTCCACGAATAAATAATATAAGATTTTG  261
             ||||||||||||||||||||||||||||||||||||||||||||||||||||||||||||
Sbjct  181   TCCTATTATACTAGGAGCTCCAGATATAGCATTTCCACGAATAAATAATATAAGATTTTG  240

Query  262   GTTACTTCCTCCTTCTCTATTTCTACTAATTAATTCTTCATTAATCGAATCAGGAGCAGG  321
             ||||||||||||||||||||||||||||||||||||||||||||||||||||||||||||
Sbjct  241   GTTACTTCCTCCTTCTCTATTTCTACTAATTAATTCTTCATTAATCGAATCAGGAGCAGG  300

Query  322   TACAGGGTGAACAGTTTAcccccccTTATCATCAAATTTATCACACTACGGACCATCAGT  381
             ||||||||||||||||||||||||||||||||||||||||||||||||||||||||||||
Sbjct  301   TACAGGGTGAACAGTTTACCCCCCCTTATCATCAAATTTATCACACTACGGACCATCAGT  360
```

```
Query  382   AGATTTAGCTATTTTTTCTCTGCATCTTGCAGGTGCTTCTTCAATTTTAGGTGCAATCAA  441
             ||||||||||||||||||||||||||||||||||||||||||||||||||||||||||||
Sbjct  361   AGATTTAGCTATTTTTTCTCTGCATCTTGCAGGTGCTTCTTCAATTTTAGGTGCAATCAA  420

Query  442   TTTCATTACAACTATTGTAAATATACGATCCATTGGAATAACAATAGAACGAATACCATT  501
             ||||||||||||||||||||||||||||||||||||||||||||||||||||||||||||
Sbjct  421   TTTCATTACAACTATTGTAAATATACGATCCATTGGAATAACAATAGAACGAATACCATT  480

Query  502   ATTTGTATGATCTGTTTTAATTACTGCAATTTTATTATTATTATCTTTACCAGTCTTAGC  561
             ||||||||||||||||||||||||||||||||||||||||||||||||||||||||||||
Sbjct  481   ATTTGTATGATCTGTTTTAATTACTGCAATTTTATTATTATTATCTTTACCAGTCTTAGC  540

Query  562   TGGCGCAATTACAATATTATTAACTGATCGAAACTTTAATACATCATTCTTTGACCCTTC  621
             ||||||||||||||||||||||||||||||||||||||||||| ||||||||||||||||
Sbjct  541   TGGCGCAATTACAATATTATTAACTGATCGAAACTTTAATACAACATTCTTTGACCCTTC  600

Query  622   AGGAGGAGGTGACCCAATTCTATATCAACATTTATTT  658
             |||||||||||||||||||||||||||||||||||||
Sbjct  601   AGGAGGAGGTGACCCAATTCTATATCAACATTTATTT  637
```

>Rhipicephalus sanguineus isolate GTF3 cytochrome oxidase subunit I (cox1) gene, partial cds; mitochondrial
Sequence ID: JX416304.1 Length: 802
Range 1: 1 to 637

Score:1171 bits(634), Expect:0.0,
Identities:636/637(99%), Gaps:0/637(0%), Strand: Plus/Plus

```
Query  22    AGCATGATCCGGAATACTAGGATTAAGAATAAGAATATTAATCCGCATAGAATTAGGTCA  81
             ||||||||||||||||||||||||||||||||||||||||||||||||||||||||||||
Sbjct  1     AGCATGATCCGGAATACTAGGATTAAGAATAAGAATATTAATCCGCATAGAATTAGGTCA  60

Query  82    ACCAGGAACTTTAATTGGAAACGATCAAATTTATAATGTAATTGTAACAGCACATGCATT  141
             ||||||||||||||||||||||||||||||||||||||||||||||||||| ||||||||
Sbjct  61    ACCAGGAACTTTAATTGGAAACGATCAAATTTATAATGTAATTGTAACAGCTCATGCATT  120

Query  142   TATTATAAttttttttATAGTTATACCAATTATAATCGGAGGATTTGGAAATTGATTAGT  201
             ||||||||||||||||||||||||||||||||||||||||||||||||||||||||||||
Sbjct  121   TATTATAATTTTTTTTATAGTTATACCAATTATAATCGGAGGATTTGGAAATTGATTAGT  180

Query  202   TCCTATTATACTAGGAGCTCCAGATATAGCATTTCCACGAATAAATAATATAAGATTTTG  261
             ||||||||||||||||||||||||||||||||||||||||||||||||||||||||||||
Sbjct  181   TCCTATTATACTAGGAGCTCCAGATATAGCATTTCCACGAATAAATAATATAAGATTTTG  240

Query  262   GTTACTTCCTCCTTCTCTATTTCTACTAATTAATTCTTCATTAATCGAATCAGGAGCAGG  321
             ||||||||||||||||||||||||||||||||||||||||||||||||||||||||||||
Sbjct  241   GTTACTTCCTCCTTCTCTATTTCTACTAATTAATTCTTCATTAATCGAATCAGGAGCAGG  300

Query  322   TACAGGGTGAACAGTTTAcccccccTTATCATCAAATTTATCACACTACGGACCATCAGT  381
             ||||||||||||||||||||||||||||||||||||||||||||||||||||||||||||
Sbjct  301   TACAGGGTGAACAGTTTACCCCCCCTTATCATCAAATTTATCACACTACGGACCATCAGT  360
```

```
Query  382   AGATTTAGCTATTTTTTCTCTGCATCTTGCAGGTGCTTCTTCAATTTTAGGTGCAATCAA  441
             ||||||||||||||||||||||||||||||||||||||||||||||||||||||||||||
Sbjct  361   AGATTTAGCTATTTTTTCTCTGCATCTTGCAGGTGCTTCTTCAATTTTAGGTGCAATCAA  420

Query  442   TTTCATTACAACTATTGTAAATATACGATCCATTGGAATAACAATAGAACGAATACCATT  501
             ||||||||||||||||||||||||||||||||||||||||||||||||||||||||||||
Sbjct  421   TTTCATTACAACTATTGTAAATATACGATCCATTGGAATAACAATAGAACGAATACCATT  480

Query  502   ATTTGTATGATCTGTTTTAATTACTGCAATTTTATTATTATTATCTTTACCAGTCTTAGC  561
             ||||||||||||||||||||||||||||||||||||||||||||||||||||||||||||
Sbjct  481   ATTTGTATGATCTGTTTTAATTACTGCAATTTTATTATTATTATCTTTACCAGTCTTAGC  540

Query  562   TGGCGCAATTACAATATTATTAACTGATCGAAACTTTAATACATCATTCTTTGACCCTTC  621
             ||||||||||||||||||||||||||||||||||||||||||||||||||||||||||||
Sbjct  541   TGGCGCAATTACAATATTATTAACTGATCGAAACTTTAATACATCATTCTTTGACCCTTC  600

Query  622   AGGAGGAGGTGACCCAATTCTATATCAACATTTATTT  658
             |||||||||||||||||||||||||||||||||||||
Sbjct  601   AGGAGGAGGTGACCCAATTCTATATCAACATTTATTT  637
```

>Rhipicephalus sanguineus isolate GBM1 cytochrome oxidase subunit I (cox1) gene, partial cds; mitochondrial
Sequence ID: JX416300.1 Length: 802
Range 1: 1 to 637

Score:1171 bits(634), Expect:0.0,
Identities:636/637(99%), Gaps:0/637(0%), Strand: Plus/Plus

```
Query  22    AGCATGATCCGGAATACTAGGATTAAGAATAAGAATATTAATCCGCATAGAATTAGGTCA  81
             ||||||||||||||||||||||||||||||||||||||||||||||||||||||||||||
Sbjct  1     AGCATGATCCGGAATACTAGGATTAAGAATAAGAATATTAATCCGCATAGAATTAGGTCA  60

Query  82    ACCAGGAACTTTAATTGGAAACGATCAAATTTATAATGTAATTGTAACAGCACATGCATT  141
             ||||||||||||||||||||||||||||||||||||||||||||||||||||||||||||
Sbjct  61    ACCAGGAACTTTAATTGGAAACGATCAAATTTATAATGTAATTGTAACAGCACATGCATT  120

Query  142   TATTATAAttttttttATAGTTATACCAATTATAATCGGAGGATTTGGAAATTGATTAGT  201
             |||||||||||||||||||||||||||||||||||||||||||||||| |||||||||||
Sbjct  121   TATTATAATTTTTTTTATAGTTATACCAATTATAATCGGAGGATTTGGGAATTGATTAGT  180

Query  202   TCCTATTATACTAGGAGCTCCAGATATAGCATTTCCACGAATAAATAATATAAGATTTTG  261
             ||||||||||||||||||||||||||||||||||||||||||||||||||||||||||||
Sbjct  181   TCCTATTATACTAGGAGCTCCAGATATAGCATTTCCACGAATAAATAATATAAGATTTTG  240

Query  262   GTTACTTCCTCCTTCTCTATTTCTACTAATTAATTCTTCATTAATCGAATCAGGAGCAGG  321
             ||||||||||||||||||||||||||||||||||||||||||||||||||||||||||||
Sbjct  241   GTTACTTCCTCCTTCTCTATTTCTACTAATTAATTCTTCATTAATCGAATCAGGAGCAGG  300

Query  322   TACAGGGTGAACAGTTTAcccccccTTATCATCAAATTTATCACACTACGGACCATCAGT  381
             ||||||||||||||||||||||||||||||||||||||||||||||||||||||||||||
```

```
Sbjct  301   TACAGGGTGAACAGTTTACCCCCCCTTATCATCAAATTTATCACACTACGGACCATCAGT  360

Query  382   AGATTTAGCTATTTTTTCTCTGCATCTTGCAGGTGCTTCTTCAATTTTAGGTGCAATCAA  441
             ||||||||||||||||||||||||||||||||||||||||||||||||||||||||||||
Sbjct  361   AGATTTAGCTATTTTTTCTCTGCATCTTGCAGGTGCTTCTTCAATTTTAGGTGCAATCAA  420

Query  442   TTTCATTACAACTATTGTAAATATACGATCCATTGGAATAACAATAGAACGAATACCATT  501
             ||||||||||||||||||||||||||||||||||||||||||||||||||||||||||||
Sbjct  421   TTTCATTACAACTATTGTAAATATACGATCCATTGGAATAACAATAGAACGAATACCATT  480

Query  502   ATTTGTATGATCTGTTTTAATTACTGCAATTTTATTATTATTATCTTTACCAGTCTTAGC  561
             ||||||||||||||||||||||||||||||||||||||||||||||||||||||||||||
Sbjct  481   ATTTGTATGATCTGTTTTAATTACTGCAATTTTATTATTATTATCTTTACCAGTCTTAGC  540

Query  562   TGGCGCAATTACAATATTATTAACTGATCGAAACTTTAATACATCATTCTTTGACCCTTC  621
             ||||||||||||||||||||||||||||||||||||||||||||||||||||||||||||
Sbjct  541   TGGCGCAATTACAATATTATTAACTGATCGAAACTTTAATACATCATTCTTTGACCCTTC  600

Query  622   AGGAGGAGGTGACCCAATTCTATATCAACATTTATTT  658
             |||||||||||||||||||||||||||||||||||||
Sbjct  601   AGGAGGAGGTGACCCAATTCTATATCAACATTTATTT  637
```

>Rhipicephalus sanguineus isolate GBF1 cytochrome oxidase subunit I (cox1) gene, partial cds; mitochondrial
Sequence ID: JX416299.1 Length: 802
Range 1: 1 to 637

Score:1171 bits(634), Expect:0.0,
Identities:636/637(99%), Gaps:0/637(0%), Strand: Plus/Plus

```
Query  22    AGCATGATCCGGAATACTAGGATTAAGAATAAGAATATTAATCCGCATAGAATTAGGTCA  81
             |||||||||||| |||||||||||||||||||||||||||||||||||||||||||||||
Sbjct  1     AGCATGATCCGGGATACTAGGATTAAGAATAAGAATATTAATCCGCATAGAATTAGGTCA  60

Query  82    ACCAGGAACTTTAATTGGAAACGATCAAATTTATAATGTAATTGTAACAGCACATGCATT  141
             ||||||||||||||||||||||||||||||||||||||||||||||||||||||||||||
Sbjct  61    ACCAGGAACTTTAATTGGAAACGATCAAATTTATAATGTAATTGTAACAGCACATGCATT  120

Query  142   TATTATAAttttttttATAGTTATACCAATTATAATCGGAGGATTTGGAAATTGATTAGT  201
             ||||||||||||||||||||||||||||||||||||||||||||||||||||||||||||
Sbjct  121   TATTATAATTTTTTTTATAGTTATACCAATTATAATCGGAGGATTTGGAAATTGATTAGT  180

Query  202   TCCTATTATACTAGGAGCTCCAGATATAGCATTTCCACGAATAAATAATATAAGATTTTG  261
             ||||||||||||||||||||||||||||||||||||||||||||||||||||||||||||
Sbjct  181   TCCTATTATACTAGGAGCTCCAGATATAGCATTTCCACGAATAAATAATATAAGATTTTG  240

Query  262   GTTACTTCCTCCTTCTCTATTTCTACTAATTAATTCTTCATTAATCGAATCAGGAGCAGG  321
             ||||||||||||||||||||||||||||||||||||||||||||||||||||||||||||
Sbjct  241   GTTACTTCCTCCTTCTCTATTTCTACTAATTAATTCTTCATTAATCGAATCAGGAGCAGG  300

Query  322   TACAGGGTGAACAGTTTAcccccccTTATCATCAAATTTATCACACTACGGACCATCAGT  381
```

```
            ||||||||||||||||||||||||||||||||||||||||||||||||||||||||||||
Sbjct  301  TACAGGGTGAACAGTTTACCCCCCCTTATCATCAAATTTATCACACTACGGACCATCAGT  360

Query  382  AGATTTAGCTATTTTTTCTCTGCATCTTGCAGGTGCTTCTTCAATTTTAGGTGCAATCAA  441
            ||||||||||||||||||||||||||||||||||||||||||||||||||||||||||||
Sbjct  361  AGATTTAGCTATTTTTTCTCTGCATCTTGCAGGTGCTTCTTCAATTTTAGGTGCAATCAA  420

Query  442  TTTCATTACAACTATTGTAAATATACGATCCATTGGAATAACAATAGAACGAATACCATT  501
            ||||||||||||||||||||||||||||||||||||||||||||||||||||||||||||
Sbjct  421  TTTCATTACAACTATTGTAAATATACGATCCATTGGAATAACAATAGAACGAATACCATT  480

Query  502  ATTTGTATGATCTGTTTTAATTACTGCAATTTTATTATTATTATCTTTACCAGTCTTAGC  561
            ||||||||||||||||||||||||||||||||||||||||||||||||||||||||||||
Sbjct  481  ATTTGTATGATCTGTTTTAATTACTGCAATTTTATTATTATTATCTTTACCAGTCTTAGC  540

Query  562  TGGCGCAATTACAATATTATTAACTGATCGAAACTTTAATACATCATTCTTTGACCCTTC  621
            ||||||||||||||||||||||||||||||||||||||||||||||||||||||||||||
Sbjct  541  TGGCGCAATTACAATATTATTAACTGATCGAAACTTTAATACATCATTCTTTGACCCTTC  600

Query  622  AGGAGGAGGTGACCCAATTCTATATCAACATTTATTT  658
            |||||||||||||||||||||||||||||||||||||
Sbjct  601  AGGAGGAGGTGACCCAATTCTATATCAACATTTATTT  637
```

>Rhipicephalus sanguineus isolate CHF1 cytochrome oxidase subunit I (cox1) gene, partial cds; mitochondrial
Sequence ID: JX416297.1 Length: 802
Range 1: 1 to 637

Score:1171 bits(634), Expect:0.0,
Identities:636/637(99%), Gaps:0/637(0%), Strand: Plus/Plus

```
Query  22   AGCATGATCCGGAATACTAGGATTAAGAATAAGAATATTAATCCGCATAGAATTAGGTCA  81
            ||||||||||||||||||||||||||||||||||||||||||||||||||||||||||||
Sbjct  1    AGCATGATCCGGAATACTAGGATTAAGAATAAGAATATTAATCCGCATAGAATTAGGTCA  60

Query  82   ACCAGGAACTTTAATTGGAAACGATCAAATTTATAATGTAATTGTAACAGCACATGCATT  141
            ||||||||||||||||||||||||||||||||||||||||||||||||||||||||||||
Sbjct  61   ACCAGGAACTTTAATTGGAAACGATCAAATTTATAATGTAATTGTAACAGCACATGCATT  120

Query  142  TATTATAAttttttttATAGTTATACCAATTATAATCGGAGGATTTGGAAATTGATTAGT  201
            ||||||||||||||||||||||||||||||||||||||||||||||||||||||||||||
Sbjct  121  TATTATAATTTTTTTTATAGTTATACCAATTATAATCGGAGGATTTGGAAATTGATTAGT  180

Query  202  TCCTATTATACTAGGAGCTCCAGATATAGCATTTCCACGAATAAATAATATAAGATTTTG  261
            ||||||||||||||||||||||||||||||||||||||||||||||||||||||||||||
Sbjct  181  TCCTATTATACTAGGAGCTCCAGATATAGCATTTCCACGAATAAATAATATAAGATTTTG  240

Query  262  GTTACTTCCTCCTTCTCTATTTCTACTAATTAATTCTTCATTAATCGAATCAGGAGCAGG  321
            ||||||||||||||||||||||||||||||||||||||||||||||||||||||||||||
Sbjct  241  GTTACTTCCTCCTTCTCTATTTCTACTAATTAATTCTTCATTAATCGAATCAGGAGCAGG  300
```

```
Query  322   TACAGGGTGAACAGTTTAcccccccTTATCATCAAATTTATCACACTACGGACCATCAGT  381
             ||||||||||||||||||||||||||||||||||||||||||||||||||||||||||||
Sbjct  301   TACAGGGTGAACAGTTTACCCCCCCTTATCATCAAATTTATCACACTACGGACCATCAGT  360

Query  382   AGATTTAGCTATTTTTTCTCTGCATCTTGCAGGTGCTTCTTCAATTTTAGGTGCAATCAA  441
             ||||||||||||||||||||||||||||||||||||||||||||||||||||||||||||
Sbjct  361   AGATTTAGCTATTTTTTCTCTGCATCTTGCAGGTGCTTCTTCAATTTTAGGTGCAATCAA  420

Query  442   TTTCATTACAACTATTGTAAATATACGATCCATTGGAATAACAATAGAACGAATACCATT  501
             ||||||||||||||||||||||||||||||||||||||||||||||||||||||||||||
Sbjct  421   TTTCATTACAACTATTGTAAATATACGATCCATTGGAATAACAATAGAACGAATACCATT  480

Query  502   ATTTGTATGATCTGTTTTAATTACTGCAATTTTATTATTATTATCTTTACCAGTCTTAGC  561
             ||||||||||||||||||||||||||||||||||||||||| ||||||||||||||||||
Sbjct  481   ATTTGTATGATCTGTTTTAATTACTGCAATTTTATTATTACTATCTTTACCAGTCTTAGC  540

Query  562   TGGCGCAATTACAATATTATTAACTGATCGAAACTTTAATACATCATTCTTTGACCCTTC  621
             ||||||||||||||||||||||||||||||||||||||||||||||||||||||||||||
Sbjct  541   TGGCGCAATTACAATATTATTAACTGATCGAAACTTTAATACATCATTCTTTGACCCTTC  600

Query  622   AGGAGGAGGTGACCCAATTCTATATCAACATTTATTT  658
             |||||||||||||||||||||||||||||||||||||
Sbjct  601   AGGAGGAGGTGACCCAATTCTATATCAACATTTATTT  637
```

>Rhipicephalus sanguineus isolate DGM1 cytochrome oxidase subunit I (cox1) gene, partial cds; mitochondrial
Sequence ID: JX416296.1 Length: 802
Range 1: 1 to 637

Score:1171 bits(634), Expect:0.0,
Identities:636/637(99%), Gaps:0/637(0%), Strand: Plus/Plus

```
Query  22    AGCATGATCCGGAATACTAGGATTAAGAATAAGAATATTAATCCGCATAGAATTAGGTCA  81
             ||||||||||||||||||||||||||||||||||||||||||||||||||||||||||||
Sbjct  1     AGCATGATCCGGAATACTAGGATTAAGAATAAGAATATTAATCCGCATAGAATTAGGTCA  60

Query  82    ACCAGGAACTTTAATTGGAAACGATCAAATTTATAATGTAATTGTAACAGCACATGCATT  141
             ||||||||||||||||||||||||||||||||||||||||||||||||||||||||||||
Sbjct  61    ACCAGGAACTTTAATTGGAAACGATCAAATTTATAATGTAATTGTAACAGCACATGCATT  120

Query  142   TATTATAAttttttttATAGTTATACCAATTATAATCGGAGGATTTGGAAATTGATTAGT  201
             |||||| |||||||||||||||||||||||||||||||||||||||||||||||||||||
Sbjct  121   TATTATGATTTTTTTTATAGTTATACCAATTATAATCGGAGGATTTGGAAATTGATTAGT  180

Query  202   TCCTATTATACTAGGAGCTCCAGATATAGCATTTCCACGAATAAATAATATAAGATTTTG  261
             ||||||||||||||||||||||||||||||||||||||||||||||||||||||||||||
Sbjct  181   TCCTATTATACTAGGAGCTCCAGATATAGCATTTCCACGAATAAATAATATAAGATTTTG  240

Query  262   GTTACTTCCTCCTTCTCTATTTCTACTAATTAATTCTTCATTAATCGAATCAGGAGCAGG  321
             ||||||||||||||||||||||||||||||||||||||||||||||||||||||||||||
Sbjct  241   GTTACTTCCTCCTTCTCTATTTCTACTAATTAATTCTTCATTAATCGAATCAGGAGCAGG  300
```

```
Query  322   TACAGGGTGAACAGTTTAcccccccTTATCATCAAATTTATCACACTACGGACCATCAGT  381
             ||||||||||||||||||||||||||||||||||||||||||||||||||||||||||||
Sbjct  301   TACAGGGTGAACAGTTTACCCCCCCTTATCATCAAATTTATCACACTACGGACCATCAGT  360

Query  382   AGATTTAGCTATTTTTTCTCTGCATCTTGCAGGTGCTTCTTCAATTTTAGGTGCAATCAA  441
             ||||||||||||||||||||||||||||||||||||||||||||||||||||||||||||
Sbjct  361   AGATTTAGCTATTTTTTCTCTGCATCTTGCAGGTGCTTCTTCAATTTTAGGTGCAATCAA  420

Query  442   TTTCATTACAACTATTGTAAATATACGATCCATTGGAATAACAATAGAACGAATACCATT  501
             ||||||||||||||||||||||||||||||||||||||||||||||||||||||||||||
Sbjct  421   TTTCATTACAACTATTGTAAATATACGATCCATTGGAATAACAATAGAACGAATACCATT  480

Query  502   ATTTGTATGATCTGTTTTAATTACTGCAATTTTATTATTATTATCTTTACCAGTCTTAGC  561
             ||||||||||||||||||||||||||||||||||||||||||||||||||||||||||||
Sbjct  481   ATTTGTATGATCTGTTTTAATTACTGCAATTTTATTATTATTATCTTTACCAGTCTTAGC  540

Query  562   TGGCGCAATTACAATATTATTAACTGATCGAAACTTTAATACATCATTCTTTGACCCTTC  621
             ||||||||||||||||||||||||||||||||||||||||||||||||||||||||||||
Sbjct  541   TGGCGCAATTACAATATTATTAACTGATCGAAACTTTAATACATCATTCTTTGACCCTTC  600

Query  622   AGGAGGAGGTGACCCAATTCTATATCAACATTTATTT  658
             |||||||||||||||||||||||||||||||||||||
Sbjct  601   AGGAGGAGGTGACCCAATTCTATATCAACATTTATTT  637
```

>Rhipicephalus sanguineus isolate FSF1 cytochrome oxidase subunit I (cox1) gene, partial cds; mitochondrial
Sequence ID: JX416293.1 Length: 802
Range 1: 1 to 637

Score:1171 bits(634), Expect:0.0,
Identities:636/637(99%), Gaps:0/637(0%), Strand: Plus/Plus

```
Query  22    AGCATGATCCGGAATACTAGGATTAAGAATAAGAATATTAATCCGCATAGAATTAGGTCA  81
             ||||||||||||||||||||||||| ||||||||||||||||||||||||||||||||||
Sbjct  1     AGCATGATCCGGAATACTAGGATTACGAATAAGAATATTAATCCGCATAGAATTAGGTCA  60

Query  82    ACCAGGAACTTTAATTGGAAACGATCAAATTTATAATGTAATTGTAACAGCACATGCATT  141
             ||||||||||||||||||||||||||||||||||||||||||||||||||||||||||||
Sbjct  61    ACCAGGAACTTTAATTGGAAACGATCAAATTTATAATGTAATTGTAACAGCACATGCATT  120

Query  142   TATTATAAtttttttATAGTTATACCAATTATAATCGGAGGATTTGGAAATTGATTAGT  201
             ||||||||||||||||||||||||||||||||||||||||||||||||||||||||||||
Sbjct  121   TATTATAATTTTTTTTATAGTTATACCAATTATAATCGGAGGATTTGGAAATTGATTAGT  180

Query  202   TCCTATTATACTAGGAGCTCCAGATATAGCATTTCCACGAATAAATAATATAAGATTTTG  261
             ||||||||||||||||||||||||||||||||||||||||||||||||||||||||||||
Sbjct  181   TCCTATTATACTAGGAGCTCCAGATATAGCATTTCCACGAATAAATAATATAAGATTTTG  240

Query  262   GTTACTTCCTCCTTCTCTATTTCTACTAATTAATTCTTCATTAATCGAATCAGGAGCAGG  321
             ||||||||||||||||||||||||||||||||||||||||||||||||||||||||||||
```

```
Sbjct  241   GTTACTTCCTCCTTCTCTATTTCTACTAATTAATTCTTCATTAATCGAATCAGGAGCAGG  300

Query  322   TACAGGGTGAACAGTTTAcccccccTTATCATCAAATTTATCACACTACGGACCATCAGT  381
             ||||||||||||||||||||||||||||||||||||||||||||||||||||||||||||
Sbjct  301   TACAGGGTGAACAGTTTACCCCCCCTTATCATCAAATTTATCACACTACGGACCATCAGT  360

Query  382   AGATTTAGCTATTTTTTCTCTGCATCTTGCAGGTGCTTCTTCAATTTTAGGTGCAATCAA  441
             ||||||||||||||||||||||||||||||||||||||||||||||||||||||||||||
Sbjct  361   AGATTTAGCTATTTTTTCTCTGCATCTTGCAGGTGCTTCTTCAATTTTAGGTGCAATCAA  420

Query  442   TTTCATTACAACTATTGTAAATATACGATCCATTGGAATAACAATAGAACGAATACCATT  501
             ||||||||||||||||||||||||||||||||||||||||||||||||||||||||||||
Sbjct  421   TTTCATTACAACTATTGTAAATATACGATCCATTGGAATAACAATAGAACGAATACCATT  480

Query  502   ATTTGTATGATCTGTTTTAATTACTGCAATTTTATTATTATTATCTTTACCAGTCTTAGC  561
             ||||||||||||||||||||||||||||||||||||||||||||||||||||||||||||
Sbjct  481   ATTTGTATGATCTGTTTTAATTACTGCAATTTTATTATTATTATCTTTACCAGTCTTAGC  540

Query  562   TGGCGCAATTACAATATTATTAACTGATCGAAACTTTAATACATCATTCTTTGACCCTTC  621
             ||||||||||||||||||||||||||||||||||||||||||||||||||||||||||||
Sbjct  541   TGGCGCAATTACAATATTATTAACTGATCGAAACTTTAATACATCATTCTTTGACCCTTC  600

Query  622   AGGAGGAGGTGACCCAATTCTATATCAACATTTATTT  658
             |||||||||||||||||||||||||||||||||||||
Sbjct  601   AGGAGGAGGTGACCCAATTCTATATCAACATTTATTT  637
```

>Rhipicephalus sanguineus isolate T78D2 cytochrome c oxidase subunit I (COX1) gene, partial cds; mitochondrial
Sequence ID: MZ401439.1 Length: 650
Range 1: 1 to 634

Score:1171 bits(634), Expect:0.0,
Identities:634/634(100%), Gaps:0/634(0%), Strand: Plus/Plus

```
Query  25    ATGATCCGGAATACTAGGATTAAGAATAAGAATATTAATCCGCATAGAATTAGGTCAACC  84
             ||||||||||||||||||||||||||||||||||||||||||||||||||||||||||||
Sbjct  1     ATGATCCGGAATACTAGGATTAAGAATAAGAATATTAATCCGCATAGAATTAGGTCAACC  60

Query  85    AGGAACTTTAATTGGAAACGATCAAATTTATAATGTAATTGTAACAGCACATGCATTTAT  144
             ||||||||||||||||||||||||||||||||||||||||||||||||||||||||||||
Sbjct  61    AGGAACTTTAATTGGAAACGATCAAATTTATAATGTAATTGTAACAGCACATGCATTTAT  120

Query  145   TATAAtttttttATAGTTATACCAATTATAATCGGAGGATTTGGAAATTGATTAGTTCC  204
             ||||||||||||||||||||||||||||||||||||||||||||||||||||||||||||
Sbjct  121   TATAATTTTTTTATAGTTATACCAATTATAATCGGAGGATTTGGAAATTGATTAGTTCC  180

Query  205   TATTATACTAGGAGCTCCAGATATAGCATTTCCACGAATAAATAATATAAGATTTTGGTT  264
             ||||||||||||||||||||||||||||||||||||||||||||||||||||||||||||
Sbjct  181   TATTATACTAGGAGCTCCAGATATAGCATTTCCACGAATAAATAATATAAGATTTTGGTT  240

Query  265   ACTTCCTCCTTCTCTATTTCTACTAATTAATTCTTCATTAATCGAATCAGGAGCAGGTAC  324
```

```
                ||||||||||||||||||||||||||||||||||||||||||||||||||||||||||||
Sbjct  241   ACTTCCTCCTTCTCTATTTCTACTAATTAATTCTTCATTAATCGAATCAGGAGCAGGTAC  300

Query  325   AGGGTGAACAGTTTAcccccccTTATCATCAAATTTATCACACTACGGACCATCAGTAGA  384
             ||||||||||||||||||||||||||||||||||||||||||||||||||||||||||||
Sbjct  301   AGGGTGAACAGTTTACCCCCCCTTATCATCAAATTTATCACACTACGGACCATCAGTAGA  360

Query  385   TTTAGCTATTTTTTCTCTGCATCTTGCAGGTGCTTCTTCAATTTTAGGTGCAATCAATTT  444
             ||||||||||||||||||||||||||||||||||||||||||||||||||||||||||||
Sbjct  361   TTTAGCTATTTTTTCTCTGCATCTTGCAGGTGCTTCTTCAATTTTAGGTGCAATCAATTT  420

Query  445   CATTACAACTATTGTAAATATACGATCCATTGGAATAACAATAGAACGAATACCATTATT  504
             ||||||||||||||||||||||||||||||||||||||||||||||||||||||||||||
Sbjct  421   CATTACAACTATTGTAAATATACGATCCATTGGAATAACAATAGAACGAATACCATTATT  480

Query  505   TGTATGATCTGTTTTAATTACTGCAATTTTATTATTATTATCTTTACCAGTCTTAGCTGG  564
             ||||||||||||||||||||||||||||||||||||||||||||||||||||||||||||
Sbjct  481   TGTATGATCTGTTTTAATTACTGCAATTTTATTATTATTATCTTTACCAGTCTTAGCTGG  540

Query  565   CGCAATTACAATATTATTAACTGATCGAAACTTTAATACATCATTCTTTGACCCTTCAGG  624
             ||||||||||||||||||||||||||||||||||||||||||||||||||||||||||||
Sbjct  541   CGCAATTACAATATTATTAACTGATCGAAACTTTAATACATCATTCTTTGACCCTTCAGG  600

Query  625   AGGAGGTGACCCAATTCTATATCAACATTTATTT  658
             ||||||||||||||||||||||||||||||||||
Sbjct  601   AGGAGGTGACCCAATTCTATATCAACATTTATTT  634
```

>Rhipicephalus sanguineus isolate LIC5762 cytochrome oxidase subunit 1 (COI) gene, partial cds; mitochondrial
Sequence ID: KX383802.1 Length: 763
>Rhipicephalus sanguineus isolate LIC5767C cytochrome oxidase subunit 1 (COI) gene, partial cds; mitochondrial
Sequence ID: KX383803.1 Length: 763
>Rhipicephalus sanguineus isolate LIC5767D cytochrome oxidase subunit 1 (COI) gene, partial cds; mitochondrial
Sequence ID: KX383804.1 Length: 763
Range 1: 1 to 637

Score:1166 bits(631), Expect:0.0,
Identities:635/637(99%), Gaps:0/637(0%), Strand: Plus/Plus

```
Query  22    AGCATGATCCGGAATACTAGGATTAAGAATAAGAATATTAATCCGCATAGAATTAGGTCA  81
             ||||||||||||||||||||||||||||||||||||||||||||||||||||||||||||
Sbjct  1     AGCATGATCCGGAATACTAGGATTAAGAATAAGAATATTAATCCGCATAGAATTAGGTCA  60

Query  82    ACCAGGAACTTTAATTGGAAACGATCAAATTTATAATGTAATTGTAACAGCACATGCATT  141
             ||||||||||||||||||||||||||||||||||||||||||||||||||||||||||||
Sbjct  61    ACCAGGAACTTTAATTGGAAACGATCAAATTTATAATGTAATTGTAACAGCACATGCATT  120

Query  142   TATTATAAtttttttATAGTTATACCAATTATAATCGGAGGATTTGGAAATTGATTAGT  201
             ||||||||||||||||||||||||||||||||||||||||||||||||||||||||||||
```

```
Sbjct  121  TATTATAATTTTTTTTATAGTTATACCAATTATAATCGGAGGATTTGGAAATTGATTAGT  180

Query  202  TCCTATTATACTAGGAGCTCCAGATATAGCATTTCCACGAATAAATAATATAAGATTTTG  261
            ||||||||||||||||||||||||||||||||||||||||||||||||||||||||||||
Sbjct  181  TCCTATTATACTAGGAGCTCCAGATATAGCATTTCCACGAATAAATAATATAAGATTTTG  240

Query  262  GTTACTTCCTCCTTCTCTATTTCTACTAATTAATTCTTCATTAATCGAATCAGGAGCAGG  321
            ||||||||||||||||||||||||||||||||||||||||||||||||||||||||||||
Sbjct  241  GTTACTTCCTCCTTCTCTATTTCTACTAATTAATTCTTCATTAATCGAATCAGGAGCAGG  300

Query  322  TACAGGGTGAACAGTTTAcccccccTTATCATCAAATTTATCACACTACGGACCATCAGT  381
            ||||||||||||||||||||||||||||||||||||||||||||||||||||||||||||
Sbjct  301  TACAGGGTGAACAGTTTACCCCCCCTTATCATCAAATTTATCACACTACGGACCATCAGT  360

Query  382  AGATTTAGCTATTTTTTCTCTGCATCTTGCAGGTGCTTCTTCAATTTTAGGTGCAATCAA  441
            ||||||||||||||||||||| ||||||||||||||||||||||||||||||||||||||
Sbjct  361  AGATTTAGCTATTTTTTCTCTACATCTTGCAGGTGCTTCTTCAATTTTAGGTGCAATCAA  420

Query  442  TTTCATTACAACTATTGTAAATATACGATCCATTGGAATAACAATAGAACGAATACCATT  501
            ||||||||||||||||||||||||||||||||||||||||||||||||||||||||||||
Sbjct  421  TTTCATTACAACTATTGTAAATATACGATCCATTGGAATAACAATAGAACGAATACCATT  480

Query  502  ATTTGTATGATCTGTTTTAATTACTGCAATTTTATTATTATTATCTTTACCAGTCTTAGC  561
            ||||||||||||||||||||||||||||||||||||||||||||||||||||||||||||
Sbjct  481  ATTTGTATGATCTGTTTTAATTACTGCAATTTTATTATTATTATCTTTACCAGTCTTAGC  540

Query  562  TGGCGCAATTACAATATTATTAACTGATCGAAACTTTAATACATCATTCTTTGACCCTTC  621
            ||||||||||||||||||||||||||||||||||||||||||||||||||||||||||||
Sbjct  541  TGGCGCAATTACAATATTATTAACTGATCGAAACTTTAATACATCATTCTTTGACCCTTC  600

Query  622  AGGAGGAGGTGACCCAATTCTATATCAACATTTATTT  658
            |||||| ||||||||||||||||||||||||||||||
Sbjct  601  AGGAGGGGGTGACCCAATTCTATATCAACATTTATTT  637
```

```
>Rhipicephalus linnaei voucher P1/22_16-1 cytochrome c oxidase subunit I (COX1)
gene, partial cds; mitochondrial
Sequence ID: OM984979.1 Length: 658
Range 1: 1 to 658

Score:1166 bits(631), Expect:0.0,
Identities:649/658(99%),  Gaps:0/658(0%), Strand: Plus/Plus

Query  1    GACAATATACTTAATTTTTGGAGCATGATCCGGAATACTAGGATTAAGAATAAGAATATT  60
            ||||||||||||||||||||||||||||||||||||| ||||||||||||||||||||||
Sbjct  1    GACAATATACTTAATTTTTGGAGCATGATCCGGAATATTAGGATTAAGAATAAGAATATT  60

Query  61   AATCCGCATAGAATTAGGTCAACCAGGAACTTTAATTGGAAACGATCAAATTTATAATGT  120
            ||||||||||||||||||||||||||||||||||||||||||||||||||||||||||||
Sbjct  61   AATCCGCATAGAATTAGGTCAACCAGGAACTTTAATTGGAAACGATCAAATTTATAATGT  120

Query  121  AATTGTAACAGCACATGCATTTATTATAAttttttttATAGTTATACCAATTATAATCGG  180
```

```
            ||||||||||||||||||||||||||||||||||||||||||||||||||||||||||||
Sbjct  121  AATTGTAACAGCACATGCATTTATTATAATTTTTTTATAGTTATACCAATTATAATCGG  180

Query  181  AGGATTTGGAAATTGATTAGTTCCTATTATACTAGGAGCTCCAGATATAGCATTTCCACG  240
            |||||||||||||||||||||||||||||| |||||||||||||||||||||||||||||
Sbjct  181  AGGATTTGGAAATTGATTAGTTCCTATTATCCTAGGAGCTCCAGATATAGCATTTCCACG  240

Query  241  AATAAATAATATAAGATTTTGGTTACTTCCTCCTTCTCTATTTCTACTAATTAATTCTTC  300
            ||||||||||||||||||||||||||||||||||||||||||||||||||||||||||||
Sbjct  241  AATAAATAATATAAGATTTTGGTTACTTCCTCCTTCTCTATTTCTACTAATTAATTCTTC  300

Query  301  ATTAATCGAATCAGGAGCAGGTACAGGGTGAACAGTTTAcccccccTTATCATCAAATTT  360
            ||||||||||||||||||||||||||||||||||||||||||||||||||||||||||||
Sbjct  301  ATTAATCGAATCAGGAGCAGGTACAGGGTGAACAGTTTACCCCCCCTTATCATCAAATTT  360

Query  361  ATCACACTACGGACCATCAGTAGATTTAGCTATTTTTTCTCTGCATCTTGCAGGTGCTTC  420
            |||||||||||| |||||||||||||||||||| ||||||||||||||||| ||||||||
Sbjct  361  ATCACACTACGGGCCATCAGTAGATTTAGCTATCTTTTCTCTGCATCTTGCCGGTGCTTC  420

Query  421  TTCAATTTTAGGTGCAATCAATTTCATTACAACTATTGTAAATATACGATCCATTGGAAT  480
            |||||||||||||||||| |||||||||||||||||||||||||||||||||||||| ||
Sbjct  421  TTCAATTTTAGGTGCAATTAATTTCATTACAACTATTGTAAATATACGATCCATTGGGAT  480

Query  481  AACAATAGAACGAATACCATTATTTGTATGATCTGTTTTAATTACTGCAATTTTATTATT  540
            ||||||||||||||||||||||||||||||||||||||||||||||||||||||||||||
Sbjct  481  AACAATAGAACGAATACCATTATTTGTATGATCTGTTTTAATTACTGCAATTTTATTATT  540

Query  541  ATTATCTTTACCAGTCTTAGCTGGCGCAATTACAATATTATTAACTGATCGAAACTTTAA  600
            ||||||||||||||||||||||||||||||||||||||||||||||||||||||||||||
Sbjct  541  ATTATCTTTACCAGTCTTAGCTGGCGCAATTACAATATTATTAACTGATCGAAACTTTAA  600

Query  601  TACATCATTCTTTGACCCTTCAGGAGGAGGTGACCCAATTCTATATCAACATTTATTT  658
            ||||||||||||||||||||||||||| |||||||||||||| |||||||||||||||
Sbjct  601  TACATCATTCTTTGACCCTTCAGGAGGGGGTGACCCAATTCTGTATCAACATTTATTT  658
```

>Rhipicephalus linnaei voucher P1/22_1-1 cytochrome c oxidase subunit I (COX1) gene, partial cds; mitochondrial
Sequence ID: OM984968.1 Length: 658
Range 1: 1 to 658

Score:1166 bits(631), Expect:0.0,
Identities:649/658(99%), Gaps:0/658(0%), Strand: Plus/Plus

```
Query  1    GACAATATACTTAATTTTTGGAGCATGATCCGGAATACTAGGATTAAGAATAAGAATATT  60
            ||||||||||||||||||||||||||||||||||||| ||||||||||||||||||||||
Sbjct  1    GACAATATACTTAATTTTTGGAGCATGATCCGGAATATTAGGATTAAGAATAAGAATATT  60

Query  61   AATCCGCATAGAATTAGGTCAACCAGGAACTTTAATTGGAAACGATCAAATTTATAATGT  120
            ||||||||||||||||||||||||||||||||||||||||||||||||||||||||||||
Sbjct  61   AATCCGCATAGAATTAGGTCAACCAGGAACTTTAATTGGAAACGATCAAATTTATAATGT  120
```

```
Query  121  AATTGTAACAGCACATGCATTTATTATAAttttttttATAGTTATACCAATTATAATCGG  180
            ||||||||||||||||||||||||||||||||||||||||||||||||||||||||||||
Sbjct  121  AATTGTAACAGCACATGCATTTATTATAATTTTTTTTATAGTTATACCAATTATAATCGG  180

Query  181  AGGATTTGGAAATTGATTAGTTCCTATTATACTAGGAGCTCCAGATATAGCATTTCCACG  240
            |||||||||||||||||||||||||||||| |||||||||||||||||||||||||||||
Sbjct  181  AGGATTTGGAAATTGATTAGTTCCTATTATGCTAGGAGCTCCAGATATAGCATTTCCACG  240

Query  241  AATAAATAATATAAGATTTTGGTTACTTCCTCCTTCTCTATTTCTACTAATTAATTCTTC  300
            ||||||||||||||||||||||||||||||||||||||||||||||||||||||||||||
Sbjct  241  AATAAATAATATAAGATTTTGGTTACTTCCTCCTTCTCTATTTCTACTAATTAATTCTTC  300

Query  301  ATTAATCGAATCAGGAGCAGGTACAGGGTGAACAGTTTAccccccTTATCATCAAATTT  360
            |||||||||||||||||||||||||||||||||||||||||| |||||||||||||||||
Sbjct  301  ATTAATCGAATCAGGAGCAGGTACAGGGTGAACAGTTTACCCTCCCTTATCATCAAATTT  360

Query  361  ATCACACTACGGACCATCAGTAGATTTAGCTATTTTTCTCTGCATCTTGCAGGTGCTTC  420
            |||||||||||| ||||||||||||||||||||||| |||||||||||||| ||||||||
Sbjct  361  ATCACACTACGGGCCATCAGTAGATTTAGCTATTTTCTCTCTGCATCTTGCCGGTGCTTC  420

Query  421  TTCAATTTTAGGTGCAATCAATTTCATTACAACTATTGTAAATATACGATCCATTGGAAT  480
            ||||||||||||||||||| ||||||||||||||||||||||||||||||||||||||||
Sbjct  421  TTCAATTTTAGGTGCAATTAATTTCATTACAACTATTGTAAATATACGATCCATTGGAAT  480

Query  481  AACAATAGAACGAATACCATTATTTGTATGATCTGTTTTAATTACTGCAATTTTATTATT  540
            ||||||||||||||||||||||||||||||||||||||||||||||||||||||||||||
Sbjct  481  AACAATAGAACGAATACCATTATTTGTATGATCTGTTTTAATTACTGCAATTTTATTATT  540

Query  541  ATTATCTTTACCAGTCTTAGCTGGCGCAATTACAATATTATTAACTGATCGAAACTTTAA  600
            |||||||||||||||||||||||| |||||||||||||||||||||||||||||||||||
Sbjct  541  ATTATCTTTACCAGTCTTAGCTGGTGCAATTACAATATTATTAACTGATCGAAACTTTAA  600

Query  601  TACATCATTCTTTGACCCTTCAGGAGGAGGTGACCCAATTCTATATCAACATTTATTT  658
            |||||||||||||||||||||||||||||||||||||||||| |||||||||||||||
Sbjct  601  TACATCATTCTTTGACCCTTCAGGAGGAGGTGACCCAATTCTGTATCAACATTTATTT  658
```

>Rhipicephalus sanguineus isolate DGF1 cytochrome oxidase subunit I (cox1) gene, partial cds; mitochondrial
Sequence ID: JX416295.1 Length: 802
Range 1: 1 to 637

Score:1166 bits(631), Expect:0.0,
Identities:635/637(99%), Gaps:0/637(0%), Strand: Plus/Plus

```
Query  22   AGCATGATCCGGAATACTAGGATTAAGAATAAGAATATTAATCCGCATAGAATTAGGTCA  81
            ||||||||||||||||||||||||||||||||||||||||||||||||||||||||||||
Sbjct  1    AGCATGATCCGGAATACTAGGATTAAGAATAAGAATATTAATCCGCATAGAATTAGGTCA  60

Query  82   ACCAGGAACTTTAATTGGAAACGATCAAATTTATAATGTAATTGTAACAGCACATGCATT  141
            ||||||||||||||||||||||||||||||||||||||||||||||||||||||||||||
Sbjct  61   ACCAGGAACTTTAATTGGAAACGATCAAATTTATAATGTAATTGTAACAGCACATGCATT  120
```

```
Query  142   TATTATAAtttttttATAGTTATACCAATTATAATCGGAGGATTTGGAAATTGATTAGT  201
             |||||||||||||||||||||||||||||||||||||||||||||||||||||||||||
Sbjct  121   TATTATAATTTTTTTATAGTTATACCAATTATAATCGGAGGATTTGGAAATTGATTAGT  180

Query  202   TCCTATTATACTAGGAGCTCCAGATATAGCATTTCCACGAATAAATAATATAAGATTTTG  261
             ||||||||||||||||||||||||||||||||||||||||||||||||||||||||||||
Sbjct  181   TCCTATTATACTAGGAGCTCCAGATATAGCATTTCCACGAATAAATAATATAAGATTTTG  240

Query  262   GTTACTTCCTCCTTCTCTATTTCTACTAATTAATTCTTCATTAATCGAATCAGGAGCAGG  321
             ||||||||||||||||||||||||||||||||||||||||||||||||||||||||||||
Sbjct  241   GTTACTTCCTCCTTCTCTATTTCTACTAATTAATTCTTCATTAATCGAATCAGGAGCAGG  300

Query  322   TACAGGGTGAACAGTTTAcccccccTTATCATCAAATTTATCACACTACGGACCATCAGT  381
             |||||||||||| |||||||||||||||||||||||||||||||||||||||||||||||
Sbjct  301   TACAGGGTGAACGGTTTACCCCCCCTTATCATCAAATTTATCACACTACGGACCATCAGT  360

Query  382   AGATTTAGCTATTTTTTCTCTGCATCTTGCAGGTGCTTCTTCAATTTTAGGTGCAATCAA  441
             ||||||||||||||||||||||||||||||||||||||||||||||||||||||||||||
Sbjct  361   AGATTTAGCTATTTTTTCTCTGCATCTTGCAGGTGCTTCTTCAATTTTAGGTGCAATCAA  420

Query  442   TTTCATTACAACTATTGTAAATATACGATCCATTGGAATAACAATAGAACGAATACCATT  501
             ||||||||||||||||||||||||||||||||||||||||||||||||||||||||||||
Sbjct  421   TTTCATTACAACTATTGTAAATATACGATCCATTGGAATAACAATAGAACGAATACCATT  480

Query  502   ATTTGTATGATCTGTTTTAATTACTGCAATTTTATTATTATTATCTTTACCAGTCTTAGC  561
             ||||||||||||||||||||||||||||||||||||||||||||||||||||||||||||
Sbjct  481   ATTTGTATGATCTGTTTTAATTACTGCAATTTTATTATTATTATCTTTACCAGTCTTAGC  540

Query  562   TGGCGCAATTACAATATTATTAACTGATCGAAACTTTAATACATCATTCTTTGACCCTTC  621
             ||||||||||||||||||||||||||||||||||||||||||||||||||||||||||||
Sbjct  541   TGGCGCAATTACAATATTATTAACTGATCGAAACTTTAATACATCATTCTTTGACCCTTC  600

Query  622   AGGAGGAGGTGACCCAATTCTATATCAACATTTATTT  658
             |||||||||||||||||||||||||| ||||||||||
Sbjct  601   AGGAGGAGGTGACCCAATTCTATATCGACATTTATTT  637
```

>Rhipicephalus sanguineus isolate FSM1 cytochrome oxidase subunit I (cox1) gene, partial cds; mitochondrial
Sequence ID: JX416294.1 Length: 802
Range 1: 1 to 637

Score:1166 bits(631), Expect:0.0,
Identities:635/637(99%), Gaps:0/637(0%), Strand: Plus/Plus

```
Query  22    AGCATGATCCGGAATACTAGGATTAAGAATAAGAATATTAATCCGCATAGAATTAGGTCA  81
             |||||||||||||||||||||||||||||||||||||||||||||||||| |||||||||
Sbjct  1     AGCATGATCCGGAATACTAGGATTAAGAATAAGAATATTAATCCGCATAGTATTAGGTCA  60

Query  82    ACCAGGAACTTTAATTGGAAACGATCAAATTTATAATGTAATTGTAACAGCACATGCATT  141
             ||||||||||||||||||||||||||||||||||||||||||||||||||||||||||||
```

```
Sbjct  61   ACCAGGAACTTTAATTGGAAACGATCAAATTTATAATGTAATTGTAACAGCACATGCATT  120

Query  142  TATTATAAtttttttATAGTTATACCAATTATAATCGGAGGATTTGGAAATTGATTAGT  201
            |||||||||||||||||||||||||| |||||||||||||||||||||||||||||||||
Sbjct  121  TATTATAATTTTTTTATAGTTATACCTATTATAATCGGAGGATTTGGAAATTGATTAGT  180

Query  202  TCCTATTATACTAGGAGCTCCAGATATAGCATTTCCACGAATAAATAATATAAGATTTTG  261
            ||||||||||||||||||||||||||||||||||||||||||||||||||||||||||||
Sbjct  181  TCCTATTATACTAGGAGCTCCAGATATAGCATTTCCACGAATAAATAATATAAGATTTTG  240

Query  262  GTTACTTCCTCCTTCTCTATTTCTACTAATTAATTCTTCATTAATCGAATCAGGAGCAGG  321
            ||||||||||||||||||||||||||||||||||||||||||||||||||||||||||||
Sbjct  241  GTTACTTCCTCCTTCTCTATTTCTACTAATTAATTCTTCATTAATCGAATCAGGAGCAGG  300

Query  322  TACAGGGTGAACAGTTTAcccccccTTATCATCAAATTTATCACACTACGGACCATCAGT  381
            ||||||||||||||||||||||||||||||||||||||||||||||||||||||||||||
Sbjct  301  TACAGGGTGAACAGTTTACCCCCCCTTATCATCAAATTTATCACACTACGGACCATCAGT  360

Query  382  AGATTTAGCTATTTTTTCTCTGCATCTTGCAGGTGCTTCTTCAATTTTAGGTGCAATCAA  441
            ||||||||||||||||||||||||||||||||||||||||||||||||||||||||||||
Sbjct  361  AGATTTAGCTATTTTTTCTCTGCATCTTGCAGGTGCTTCTTCAATTTTAGGTGCAATCAA  420

Query  442  TTTCATTACAACTATTGTAAATATACGATCCATTGGAATAACAATAGAACGAATACCATT  501
            ||||||||||||||||||||||||||||||||||||||||||||||||||||||||||||
Sbjct  421  TTTCATTACAACTATTGTAAATATACGATCCATTGGAATAACAATAGAACGAATACCATT  480

Query  502  ATTTGTATGATCTGTTTTAATTACTGCAATTTTATTATTATTATCTTTACCAGTCTTAGC  561
            ||||||||||||||||||||||||||||||||||||||||||||||||||||||||||||
Sbjct  481  ATTTGTATGATCTGTTTTAATTACTGCAATTTTATTATTATTATCTTTACCAGTCTTAGC  540

Query  562  TGGCGCAATTACAATATTATTAACTGATCGAAACTTTAATACATCATTCTTTGACCCTTC  621
            ||||||||||||||||||||||||||||||||||||||||||||||||||||||||||||
Sbjct  541  TGGCGCAATTACAATATTATTAACTGATCGAAACTTTAATACATCATTCTTTGACCCTTC  600

Query  622  AGGAGGAGGTGACCCAATTCTATATCAACATTTATTT  658
            |||||||||||||||||||||||||||||||||||||
Sbjct  601  AGGAGGAGGTGACCCAATTCTATATCAACATTTATTT  637
```

>Rhipicephalus sanguineus isolate sanguineus0926 cytochrome oxidase subunit 1 (COX1) gene, partial cds; mitochondrial
Sequence ID: MH513252.1 Length: 658
Range 1: 6 to 658

Score:1162 bits(629), Expect:0.0,
Identities:645/653(99%), Gaps:0/653(0%), Strand: Plus/Plus

```
Query  1    GACAATATACTTAATTTTTGGAGCATGATCCGGAATACTAGGATTAAGAATAAGAATATT  60
            ||||||||||||||||||||||||||||||||||||||||||||||||||||||||||||
Sbjct  6    GACAATATACTTAATTTTTGGAGCATGATCCGGAATACTAGGATTAAGAATAAGAATATT  65

Query  61   AATCCGCATAGAATTAGGTCAACCAGGAACTTTAATTGGAAACGATCAAATTTATAATGT  120
```

```
             ||| ||||||||||||||||||||||||||||||||||||||||||||||||||||||||
Sbjct  66    AATTCGCATAGAATTAGGTCAACCAGGAACTTTAATTGGAAACGATCAAATTTATAATGT  125

Query  121   AATTGTAACAGCACATGCATTTATTATAAttttttttATAGTTATACCAATTATAATCGG  180
             ||||||||||||||||||||||||||||||||||||||||||||||||||||||||||||
Sbjct  126   AATTGTAACAGCACATGCATTTATTATAATTTTTTTTATAGTTATACCAATTATAATCGG  185

Query  181   AGGATTTGGAAATTGATTAGTTCCTATTATACTAGGAGCTCCAGATATAGCATTTCCACG  240
             ||||||||||||||||||||||||||||||||||||||||||||||||||||||||||||
Sbjct  186   AGGATTTGGAAATTGATTAGTTCCTATTATACTAGGAGCTCCAGATATAGCATTTCCACG  245

Query  241   AATAAATAATATAAGATTTTGGTTACTTCCTCCTTCTCTATTTCTACTAATTAATTCTTC  300
             |||||| |||||||||||||||||||||||||||||||||||||||||||||||||||||
Sbjct  246   AATAAACAATATAAGATTTTGGTTACTTCCTCCTTCTCTATTTCTACTAATTAATTCTTC  305

Query  301   ATTAATCGAATCAGGAGCAGGTACAGGGTGAACAGTTTAcccccccTTATCATCAAATTT  360
             |||||||||||||||||| |||||||||||||||||||||||||||||||||||||||||
Sbjct  306   ATTAATCGAATCAGGAGCGGGTACAGGGTGAACAGTTTACCCCCCCTTATCATCAAATTT  365

Query  361   ATCACACTACGGACCATCAGTAGATTTAGCTATTTTTTCTCTGCATCTTGCAGGTGCTTC  420
             ||||||||||||||||||||||||||||||||| ||||||||||||||||||||||||||
Sbjct  366   ATCACACTACGGACCATCAGTAGATTTAGCTATCTTTTCTCTGCATCTTGCAGGTGCTTC  425

Query  421   TTCAATTTTAGGTGCAATCAATTTCATTACAACTATTGTAAATATACGATCCATTGGAAT  480
             |||||||||||| |||||||||||||||||||||||||||||||||||||||||||||||
Sbjct  426   TTCAATTTTAGGCGCAATCAATTTCATTACAACTATTGTAAATATACGATCCATTGGAAT  485

Query  481   AACAATAGAACGAATACCATTATTTGTATGATCTGTTTTAATTACTGCAATTTTATTATT  540
             ||||||||||||||||||||||||||||||||||||||||||||||||||||||||||||
Sbjct  486   AACAATAGAACGAATACCATTATTTGTATGATCTGTTTTAATTACTGCAATTTTATTATT  545

Query  541   ATTATCTTTACCAGTCTTAGCTGGCGCAATTACAATATTATTAACTGATCGAAACTTTAA  600
             |||||||||||||||||||||||| |||||||||||||| ||||||||||||||||||||
Sbjct  546   ATTATCTTTACCAGTCTTAGCTGGTGCAATTACAATATTGTTAACTGATCGAAACTTTAA  605

Query  601   TACATCATTCTTTGACCCTTCAGGAGGAGGTGACCCAATTCTATATCAACATT  653
             |||||||||||||||||||||||||||||||||||||||||| ||||||||||
Sbjct  606   TACATCATTCTTTGACCCTTCAGGAGGAGGTGACCCAATTCTGTATCAACATT  658
```

>Rhipicephalus linnaei voucher P1/22_19-1 cytochrome c oxidase subunit I (COX1) gene, partial cds; mitochondrial
Sequence ID: OM984985.1 Length: 658
Range 1: 1 to 658

Score:1160 bits(628), Expect:0.0,
Identities:648/658(98%), Gaps:0/658(0%), Strand: Plus/Plus

```
Query  1     GACAATATACTTAATTTTTGGAGCATGATCCGGAATACTAGGATTAAGAATAAGAATATT  60
             ||||||||||||||||||||||||||||||||||||| ||||||||||||||||||||||
Sbjct  1     GACAATATACTTAATTTTTGGAGCATGATCCGGAATATTAGGATTAAGAATAAGAATATT  60
```

```
Query  61   AATCCGCATAGAATTAGGTCAACCAGGAACTTTAATTGGAAACGATCAAATTTATAATGT  120
            ||||||||||||||||||||||||||||||||||||||||||||||||||||||||||||
Sbjct  61   AATCCGCATAGAATTAGGTCAACCAGGAACTTTAATTGGAAACGATCAAATTTATAATGT  120

Query  121  AATTGTAACAGCACATGCATTTATTATAAtttttttATAGTTATACCAATTATAATCGG  180
            ||||||||||||||||||||||||||||||||||||||||||||||||||||||||||||
Sbjct  121  AATTGTAACAGCACATGCATTTATTATAATTTTTTTATAGTTATACCAATTATAATCGG  180

Query  181  AGGATTTGGAAATTGATTAGTTCCTATTATACTAGGAGCTCCAGATATAGCATTTCCACG  240
            |||||||||||||||||||||||||||||| |||||||||||||||||||||||||||||
Sbjct  181  AGGATTTGGAAATTGATTAGTTCCTATTATGCTAGGAGCTCCAGATATAGCATTTCCACG  240

Query  241  AATAAATAATATAAGATTTTGGTTACTTCCTCCTTCTCTATTTCTACTAATTAATTCTTC  300
            ||||||||||||||||||||||||||||||||||||||||||||||||||||||||||||
Sbjct  241  AATAAATAATATAAGATTTTGGTTACTTCCTCCTTCTCTATTTCTACTAATTAATTCTTC  300

Query  301  ATTAATCGAATCAGGAGCAGGTACAGGGTGAACAGTTTAcccccccTTATCATCAAATTT  360
            ||||||||||||||||||||||||||||||||||||||||||| ||||||||||||||||
Sbjct  301  ATTAATCGAATCAGGAGCAGGTACAGGGTGAACAGTTTACCCTCCCTTATCATCAAATTT  360

Query  361  ATCACACTACGGACCATCAGTAGATTTAGCTATTTTTTCTCTGCATCTTGCAGGTGCTTC  420
            |||||||||||| |||||||||||||||||||| || |||||||||||||| ||||||||
Sbjct  361  ATCACACTACGGGCCATCAGTAGATTTAGCTATCTTCTCTCTGCATCTTGCCGGTGCTTC  420

Query  421  TTCAATTTTAGGTGCAATCAATTTCATTACAACTATTGTAAATATACGATCCATTGGAAT  480
            |||||||||||||||||| |||||||||||||||||||||||||||||||||||||||||
Sbjct  421  TTCAATTTTAGGTGCAATTAATTTCATTACAACTATTGTAAATATACGATCCATTGGAAT  480

Query  481  AACAATAGAACGAATACCATTATTTGTATGATCTGTTTTAATTACTGCAATTTTATTATT  540
            ||||||||||||||||||||||||||||||||||||||||||||||||||||||||||||
Sbjct  481  AACAATAGAACGAATACCATTATTTGTATGATCTGTTTTAATTACTGCAATTTTATTATT  540

Query  541  ATTATCTTTACCAGTCTTAGCTGGCGCAATTACAATATTATTAACTGATCGAAACTTTAA  600
            |||||||||||||||||||||||| |||||||||||||||||||||||||||||||||||
Sbjct  541  ATTATCTTTACCAGTCTTAGCTGGTGCAATTACAATATTATTAACTGATCGAAACTTTAA  600

Query  601  TACATCATTCTTTGACCCTTCAGGAGGAGGTGACCCAATTCTATATCAACATTTATTT  658
            |||||||||||||||||||||||||||||||||||||||||| |||||||||||||||
Sbjct  601  TACATCATTCTTTGACCCTTCAGGAGGAGGTGACCCAATTCTGTATCAACATTTATTT  658
```

>Rhipicephalus linnaei voucher P1/22_14-1 cytochrome c oxidase subunit I (COX1) gene, partial cds; mitochondrial
Sequence ID: OM984978.1 Length: 658
Range 1: 1 to 658

Score:1160 bits(628), Expect:0.0,
Identities:648/658(98%), Gaps:0/658(0%), Strand: Plus/Plus

```
Query  1    GACAATATACTTAATTTTTGGAGCATGATCCGGAATACTAGGATTAAGAATAAGAATATT  60
            ||||||||||||||||||||||||||||||||||||| ||||||||||||||||||||||
Sbjct  1    GACAATATACTTAATTTTTGGAGCATGATCCGGAATATTAGGATTAAGAATAAGAATATT  60
```

```
Query  61   AATCCGCATAGAATTAGGTCAACCAGGAACTTTAATTGGAAACGATCAAATTTATAATGT  120
            ||||||||||||||||||||||||||||||||||||||||||||||||||||||||||||
Sbjct  61   AATCCGCATAGAATTAGGTCAACCAGGAACTTTAATTGGAAACGATCAAATTTATAATGT  120

Query  121  AATTGTAACAGCACATGCATTTATTATAAttttttttATAGTTATACCAATTATAATCGG  180
            ||||||||||||||||||||||||||||||||||||||||||||||||||||||||||||
Sbjct  121  AATTGTAACAGCACATGCATTTATTATAATTTTTTTTATAGTTATACCAATTATAATCGG  180

Query  181  AGGATTTGGAAATTGATTAGTTCCTATTATACTAGGAGCTCCAGATATAGCATTTCCACG  240
            |||||||||||||||||||||||||||||| |||||||||||||||||||||||||||||
Sbjct  181  AGGATTTGGAAATTGATTAGTTCCTATTATGCTAGGAGCTCCAGATATAGCATTTCCACG  240

Query  241  AATAAATAATATAAGATTTTGGTTACTTCCTCCTTCTCTATTTCTACTAATTAATTCTTC  300
            ||||||||||||||||||||||||||||||||||||||||||||||||||||||||||||
Sbjct  241  AATAAATAATATAAGATTTTGGTTACTTCCTCCTTCTCTATTTCTACTAATTAATTCTTC  300

Query  301  ATTAATCGAATCAGGAGCAGGTACAGGGTGAACAGTTTAcccccccTTATCATCAAATTT  360
            |||||||||||||||||||||||||||||||||||||||||| |||||||||||||||||
Sbjct  301  ATTAATCGAATCAGGAGCAGGTACAGGGTGAACAGTTTACCCTCCCTTATCATCAAATTT  360

Query  361  ATCACACTACGGACCATCAGTAGATTTAGCTATTTTTTCTCTGCATCTTGCAGGTGCTTC  420
            |||||||||||| |||||||||||||||||||| || |||||||||||||| ||||||||
Sbjct  361  ATCACACTACGGGCCATCAGTAGATTTAGCTATCTTCTCTCTGCATCTTGCCGGTGCTTC  420

Query  421  TTCAATTTTAGGTGCAATCAATTTCATTACAACTATTGTAAATATACGATCCATTGGAAT  480
            |||||||||||||||||| |||||||||||||||||||||||||||||||||||||||||
Sbjct  421  TTCAATTTTAGGTGCAATTAATTTCATTACAACTATTGTAAATATACGATCCATTGGAAT  480

Query  481  AACAATAGAACGAATACCATTATTTGTATGATCTGTTTTAATTACTGCAATTTTATTATT  540
            ||||||||||||||||||||||||||||||||||||||||||||||||||||||||||||
Sbjct  481  AACAATAGAACGAATACCATTATTTGTATGATCTGTTTTAATTACTGCAATTTTATTATT  540

Query  541  ATTATCTTTACCAGTCTTAGCTGGCGCAATTACAATATTATTAACTGATCGAAACTTTAA  600
            |||||||||||||||||||||||| |||||||||||||||||||||||||||||||||||
Sbjct  541  ATTATCTTTACCAGTCTTAGCTGGTGCAATTACAATATTATTAACTGATCGAAACTTTAA  600

Query  601  TACATCATTCTTTGACCCTTCAGGAGGAGGTGACCCAATTCTATATCAACATTTATTT  658
            |||||||||||||||||||||||||||||||||||||||||| |||||||||||||||
Sbjct  601  TACATCATTCTTTGACCCTTCAGGAGGAGGTGACCCAATTCTGTATCAACATTTATTT  658
```

>Rhipicephalus linnaei voucher P1/22_13-1 cytochrome c oxidase subunit I (COX1) gene, partial cds; mitochondrial
Sequence ID: OM984977.1 Length: 658
Range 1: 1 to 658

Score:1160 bits(628), Expect:0.0,
Identities:648/658(98%), Gaps:0/658(0%), Strand: Plus/Plus

```
Query  1    GACAATATACTTAATTTTTGGAGCATGATCCGGAATACTAGGATTAAGAATAAGAATATT  60
            ||||||||||||||||||||||||||||||||||||| ||||||||||||||||||||||
```

```
Sbjct  1    GACAATATACTTAATTTTTGGAGCATGATCCGGAATATTAGGATTAAGAATAAGAATATT  60

Query  61   AATCCGCATAGAATTAGGTCAACCAGGAACTTTAATTGGAAACGATCAAATTTATAATGT  120
            ||||||||||||||||||||||||||||||||||||||||||||||||||||||||||||
Sbjct  61   AATCCGCATAGAATTAGGTCAACCAGGAACTTTAATTGGAAACGATCAAATTTATAATGT  120

Query  121  AATTGTAACAGCACATGCATTTATTATAAttttttttATAGTTATACCAATTATAATCGG  180
            ||||||||||||||||||||||||||||||||||||||||||||||||||||||||||||
Sbjct  121  AATTGTAACAGCACATGCATTTATTATAATTTTTTTTATAGTTATACCAATTATAATCGG  180

Query  181  AGGATTTGGAAATTGATTAGTTCCTATTATACTAGGAGCTCCAGATATAGCATTTCCACG  240
            |||||||||||||||||||||||||||||| |||||||||||||||||||||||||||||
Sbjct  181  AGGATTTGGAAATTGATTAGTTCCTATTATGCTAGGAGCTCCAGATATAGCATTTCCACG  240

Query  241  AATAAATAATATAAGATTTTGGTTACTTCCTCCTTCTCTATTTCTACTAATTAATTCTTC  300
            ||||||||||||||||||||||||||||||||||||||||||||||||||||||||||||
Sbjct  241  AATAAATAATATAAGATTTTGGTTACTTCCTCCTTCTCTATTTCTACTAATTAATTCTTC  300

Query  301  ATTAATCGAATCAGGAGCAGGTACAGGGTGAACAGTTTAccccccTTATCATCAAATTT  360
            |||||||||||||||||||||||||||||||||||||||||| |||||||||||||||||
Sbjct  301  ATTAATCGAATCAGGAGCAGGTACAGGGTGAACAGTTTACCCTCCCTTATCATCAAATTT  360

Query  361  ATCACACTACGGACCATCAGTAGATTTAGCTATTTTTTCTCTGCATCTTGCAGGTGCTTC  420
            |||||||||||| |||||||||||||||||||| || |||||||||||||| ||||||||
Sbjct  361  ATCACACTACGGGCCATCAGTAGATTTAGCTATCTTCTCTCTGCATCTTGCCGGTGCTTC  420

Query  421  TTCAATTTTAGGTGCAATCAATTTCATTACAACTATTGTAAATATACGATCCATTGGAAT  480
            |||||||||||||||||| |||||||||||||||||||||||||||||||||||||||||
Sbjct  421  TTCAATTTTAGGTGCAATTAATTTCATTACAACTATTGTAAATATACGATCCATTGGAAT  480

Query  481  AACAATAGAACGAATACCATTATTTGTATGATCTGTTTTAATTACTGCAATTTTATTATT  540
            ||||||||||||||||||||||||||||||||||||||||||||||||||||||||||||
Sbjct  481  AACAATAGAACGAATACCATTATTTGTATGATCTGTTTTAATTACTGCAATTTTATTATT  540

Query  541  ATTATCTTTACCAGTCTTAGCTGGCGCAATTACAATATTATTAACTGATCGAAACTTTAA  600
            |||||||||||||||||||||||| |||||||||||||||||||||||||||||||||||
Sbjct  541  ATTATCTTTACCAGTCTTAGCTGGTGCAATTACAATATTATTAACTGATCGAAACTTTAA  600

Query  601  TACATCATTCTTTGACCCTTCAGGAGGAGGTGACCCAATTCTATATCAACATTTATTT  658
            |||||||||||||||||||||||||||||||||||||||||| |||||||||||||||
Sbjct  601  TACATCATTCTTTGACCCTTCAGGAGGAGGTGACCCAATTCTGTATCAACATTTATTT  658
```

>Rhipicephalus linnaei voucher P1/22_10-1 cytochrome c oxidase subunit I (COX1) gene, partial cds; mitochondrial
Sequence ID: OM984975.1 Length: 658
Range 1: 1 to 658

Score:1160 bits(628), Expect:0.0,
Identities:648/658(98%), Gaps:0/658(0%), Strand: Plus/Plus

```
Query  1    GACAATATACTTAATTTTTGGAGCATGATCCGGAATACTAGGATTAAGAATAAGAATATT  60
```

```
            ||||||||||||||||||||||||||||||||||||||| |||||||||||||||||||||
Sbjct  1    GACAATATACTTAATTTTTGGAGCATGATCCGGAATATTAGGATTAAGAATAAGAATATT  60

Query  61   AATCCGCATAGAATTAGGTCAACCAGGAACTTTAATTGGAAACGATCAAATTTATAATGT  120
            ||||||||||||||||||||||||||||||||||||||||||||||||||||||||||||
Sbjct  61   AATCCGCATAGAATTAGGTCAACCAGGAACTTTAATTGGAAACGATCAAATTTATAATGT  120

Query  121  AATTGTAACAGCACATGCATTTATTATAAttttttttATAGTTATACCAATTATAATCGG  180
            ||||||||||||||||||||||||||||||||||||||||||||||||||||||||||||
Sbjct  121  AATTGTAACAGCACATGCATTTATTATAATTTTTTTTATAGTTATACCAATTATAATCGG  180

Query  181  AGGATTTGGAAATTGATTAGTTCCTATTATACTAGGAGCTCCAGATATAGCATTTCCACG  240
            |||||||||||||||||||||||||||||| |||||||||||||||||||||||||||||
Sbjct  181  AGGATTTGGAAATTGATTAGTTCCTATTATGCTAGGAGCTCCAGATATAGCATTTCCACG  240

Query  241  AATAAATAATATAAGATTTTGGTTACTTCCTCCTTCTCTATTTCTACTAATTAATTCTTC  300
            ||||||||||||||||||||||||||||||||||||||||||||||||||||||||||||
Sbjct  241  AATAAATAATATAAGATTTTGGTTACTTCCTCCTTCTCTATTTCTACTAATTAATTCTTC  300

Query  301  ATTAATCGAATCAGGAGCAGGTACAGGGTGAACAGTTTAcccccccTTATCATCAAATTT  360
            |||||||||||||||||||||||||||||||||||||||||| |||||||||||||||||
Sbjct  301  ATTAATCGAATCAGGAGCAGGTACAGGGTGAACAGTTTACCCTCCCTTATCATCAAATTT  360

Query  361  ATCACACTACGGACCATCAGTAGATTTAGCTATTTTTTCTCTGCATCTTGCAGGTGCTTC  420
            |||||||||||| |||||||||||||||||||| || |||||||||||||| ||||||||
Sbjct  361  ATCACACTACGGGCCATCAGTAGATTTAGCTATCTTCTCTCTGCATCTTGCCGGTGCTTC  420

Query  421  TTCAATTTTAGGTGCAATCAATTTCATTACAACTATTGTAAATATACGATCCATTGGAAT  480
            |||||||||||||||||| |||||||||||||||||||||||||||||||||||||||||
Sbjct  421  TTCAATTTTAGGTGCAATTAATTTCATTACAACTATTGTAAATATACGATCCATTGGAAT  480

Query  481  AACAATAGAACGAATACCATTATTTGTATGATCTGTTTTAATTACTGCAATTTTATTATT  540
            ||||||||||||||||||||||||||||||||||||||||||||||||||||||||||||
Sbjct  481  AACAATAGAACGAATACCATTATTTGTATGATCTGTTTTAATTACTGCAATTTTATTATT  540

Query  541  ATTATCTTTACCAGTCTTAGCTGGCGCAATTACAATATTATTAACTGATCGAAACTTTAA  600
            |||||||||||||||||||||||| |||||||||||||||||||||||||||||||||||
Sbjct  541  ATTATCTTTACCAGTCTTAGCTGGTGCAATTACAATATTATTAACTGATCGAAACTTTAA  600

Query  601  TACATCATTCTTTGACCCTTCAGGAGGAGGTGACCCAATTCTATATCAACATTTATTT  658
            |||||||||||||||||||||||||||||||||||||||||| |||||||||||||||
Sbjct  601  TACATCATTCTTTGACCCTTCAGGAGGAGGTGACCCAATTCTGTATCAACATTTATTT  658
```

>Rhipicephalus linnaei voucher P1/22_5-1 cytochrome c oxidase subunit I (COX1) gene, partial cds; mitochondrial
Sequence ID: OM984971.1 Length: 658
Range 1: 1 to 658

Score:1160 bits(628), Expect:0.0,
Identities:648/658(98%), Gaps:0/658(0%), Strand: Plus/Plus

```
Query  1    GACAATATACTTAATTTTTGGAGCATGATCCGGAATACTAGGATTAAGAATAAGAATATT  60
            ||||||||||||||||||||||||||||||||||||| ||||||||||||||||||||||
Sbjct  1    GACAATATACTTAATTTTTGGAGCATGATCCGGAATATTAGGATTAAGAATAAGAATATT  60

Query  61   AATCCGCATAGAATTAGGTCAACCAGGAACTTTAATTGGAAACGATCAAATTTATAATGT  120
            ||||||||||||||||||||||||||||||||||||||||||||||||||||||||||||
Sbjct  61   AATCCGCATAGAATTAGGTCAACCAGGAACTTTAATTGGAAACGATCAAATTTATAATGT  120

Query  121  AATTGTAACAGCACATGCATTTATTATAAttttttttATAGTTATACCAATTATAATCGG  180
            ||||||||||||||||||||||||||||||||||||||||||||||||||||||||||||
Sbjct  121  AATTGTAACAGCACATGCATTTATTATAATTTTTTTTATAGTTATACCAATTATAATCGG  180

Query  181  AGGATTTGGAAATTGATTAGTTCCTATTATACTAGGAGCTCCAGATATAGCATTTCCACG  240
            |||||||||||||||||||||||||||||| |||||||||||||||||||||||||||||
Sbjct  181  AGGATTTGGAAATTGATTAGTTCCTATTATGCTAGGAGCTCCAGATATAGCATTTCCACG  240

Query  241  AATAAATAATATAAGATTTTGGTTACTTCCTCCTTCTCTATTTCTACTAATTAATTCTTC  300
            ||||||||||||||||||||||||||||||||||||||||||||||||||||||||||||
Sbjct  241  AATAAATAATATAAGATTTTGGTTACTTCCTCCTTCTCTATTTCTACTAATTAATTCTTC  300

Query  301  ATTAATCGAATCAGGAGCAGGTACAGGGTGAACAGTTTAcccccccTTATCATCAAATTT  360
            ||||||||||||||||||||||||||||||||||||||||||||||||||||||||||||
Sbjct  301  ATTAATCGAATCAGGAGCAGGTACAGGGTGAACAGTTTACCCCCCCTTATCATCAAATTT  360

Query  361  ATCACACTACGGACCATCAGTAGATTTAGCTATTTTTTCTCTGCATCTTGCAGGTGCTTC  420
            |||||||||||| |||||||||||||||||||| ||||||||||||||||| ||||||||
Sbjct  361  ATCACACTACGGGCCATCAGTAGATTTAGCTATCTTTTCTCTGCATCTTGCCGGTGCTTC  420

Query  421  TTCAATTTTAGGTGCAATCAATTTCATTACAACTATTGTAAATATACGATCCATTGGAAT  480
            |||||||||||||||||| ||||||||||||||||| |||||||||||||||||||| ||
Sbjct  421  TTCAATTTTAGGTGCAATTAATTTCATTACAACTATGGTAAATATACGATCCATTGGGAT  480

Query  481  AACAATAGAACGAATACCATTATTTGTATGATCTGTTTTAATTACTGCAATTTTATTATT  540
            ||||||||||||||||||||||||||||||||||||||||||||||||||||||||||||
Sbjct  481  AACAATAGAACGAATACCATTATTTGTATGATCTGTTTTAATTACTGCAATTTTATTATT  540

Query  541  ATTATCTTTACCAGTCTTAGCTGGCGCAATTACAATATTATTAACTGATCGAAACTTTAA  600
            ||||||||||||||||||||||||||||||||||||||||||||||||||||||||||||
Sbjct  541  ATTATCTTTACCAGTCTTAGCTGGCGCAATTACAATATTATTAACTGATCGAAACTTTAA  600

Query  601  TACATCATTCTTTGACCCTTCAGGAGGAGGTGACCCAATTCTATATCAACATTTATTT  658
            ||||||||||||||||||||||||||| |||||||||||||| |||||||||||||||
Sbjct  601  TACATCATTCTTTGACCCTTCAGGAGGGGGTGACCCAATTCTGTATCAACATTTATTT  658
```

>Rhipicephalus linnaei voucher P1/22_4-1 cytochrome c oxidase subunit I (COX1) gene, partial cds; mitochondrial
Sequence ID: OM984970.1 Length: 658
Range 1: 1 to 658

Score:1160 bits(628), Expect:0.0,
Identities:648/658(98%), Gaps:0/658(0%), Strand: Plus/Plus

```
Query  1    GACAATATACTTAATTTTTGGAGCATGATCCGGAATACTAGGATTAAGAATAAGAATATT  60
            |||||||||||||||||||||||||||||||||||||| |||||||||||||||||||||
Sbjct  1    GACAATATACTTAATTTTTGGAGCATGATCCGGAATATTAGGATTAAGAATAAGAATATT  60

Query  61   AATCCGCATAGAATTAGGTCAACCAGGAACTTTAATTGGAAACGATCAAATTTATAATGT  120
            ||||||||||||||||||||||||||||||||||||||||||||||||||||||||||||
Sbjct  61   AATCCGCATAGAATTAGGTCAACCAGGAACTTTAATTGGAAACGATCAAATTTATAATGT  120

Query  121  AATTGTAACAGCACATGCATTTATTATAAtttttttATAGTTATACCAATTATAATCGG  180
            ||||||||||||||||||||||||||||||||||||||||||||||||||||||||||||
Sbjct  121  AATTGTAACAGCACATGCATTTATTATAATTTTTTTATAGTTATACCAATTATAATCGG  180

Query  181  AGGATTTGGAAATTGATTAGTTCCTATTATACTAGGAGCTCCAGATATAGCATTTCCACG  240
            |||||||||||||||||||||||||||||| |||||||||||||||||||||||||||||
Sbjct  181  AGGATTTGGAAATTGATTAGTTCCTATTATGCTAGGAGCTCCAGATATAGCATTTCCACG  240

Query  241  AATAAATAATATAAGATTTTGGTTACTTCCTCCTTCTCTATTTCTACTAATTAATTCTTC  300
            ||||||||||||||||||||||||||||||||||||||||||||||||||||||||||||
Sbjct  241  AATAAATAATATAAGATTTTGGTTACTTCCTCCTTCTCTATTTCTACTAATTAATTCTTC  300

Query  301  ATTAATCGAATCAGGAGCAGGTACAGGGTGAACAGTTTAcccccccTTATCATCAAATTT  360
            |||||||||||||||||||||||||||||||||||||||||| |||||||||||||||||
Sbjct  301  ATTAATCGAATCAGGAGCAGGTACAGGGTGAACAGTTTACCCTCCCTTATCATCAAATTT  360

Query  361  ATCACACTACGGACCATCAGTAGATTTAGCTATTTTTTCTCTGCATCTTGCAGGTGCTTC  420
            |||||||||||| |||||||||||||||||||| || |||||||||||||| ||||||||
Sbjct  361  ATCACACTACGGGCCATCAGTAGATTTAGCTATCTTCTCTCTGCATCTTGCCGGTGCTTC  420

Query  421  TTCAATTTTAGGTGCAATCAATTTCATTACAACTATTGTAAATATACGATCCATTGGAAT  480
            |||||||||||||||||| |||||||||||||||||||||||||||||||||||||||||
Sbjct  421  TTCAATTTTAGGTGCAATTAATTTCATTACAACTATTGTAAATATACGATCCATTGGAAT  480

Query  481  AACAATAGAACGAATACCATTATTTGTATGATCTGTTTTAATTACTGCAATTTTATTATT  540
            ||||||||||||||||||||||||||||||||||||||||||||||||||||||||||||
Sbjct  481  AACAATAGAACGAATACCATTATTTGTATGATCTGTTTTAATTACTGCAATTTTATTATT  540

Query  541  ATTATCTTTACCAGTCTTAGCTGGCGCAATTACAATATTATTAACTGATCGAAACTTTAA  600
            |||||||||||||||||||||||| |||||||||||||||||||||||||||||||||||
Sbjct  541  ATTATCTTTACCAGTCTTAGCTGGTGCAATTACAATATTATTAACTGATCGAAACTTTAA  600

Query  601  TACATCATTCTTTGACCCTTCAGGAGGAGGTGACCCAATTCTATATCAACATTTATTT  658
            |||||||||||||||||||||||||||||||||||||||||| |||||||||||||||
Sbjct  601  TACATCATTCTTTGACCCTTCAGGAGGAGGTGACCCAATTCTGTATCAACATTTATTT  658
```

>Rhipicephalus linnaei voucher P1/22_3-1 cytochrome c oxidase subunit I (COX1) gene, partial cds; mitochondrial
Sequence ID: OM984969.1 Length: 658
Range 1: 1 to 658

Score:1160 bits(628), Expect:0.0,

```
Identities:648/658(98%),  Gaps:0/658(0%), Strand: Plus/Plus

Query  1    GACAATATACTTAATTTTTGGAGCATGATCCGGAATACTAGGATTAAGAATAAGAATATT  60
            ||||||||||||||||||||||||||||||||||||| ||||||||||||||||||||||
Sbjct  1    GACAATATACTTAATTTTTGGAGCATGATCCGGAATATTAGGATTAAGAATAAGAATATT  60

Query  61   AATCCGCATAGAATTAGGTCAACCAGGAACTTTAATTGGAAACGATCAAATTTATAATGT  120
            ||||||||||||||||||||||||||||||||||||||||||||||||||||||||||||
Sbjct  61   AATCCGCATAGAATTAGGTCAACCAGGAACTTTAATTGGAAACGATCAAATTTATAATGT  120

Query  121  AATTGTAACAGCACATGCATTTATTATAAtttttttATAGTTATACCAATTATAATCGG  180
            ||||||||||||||||||||||||||||||||||||||||||||||||||||||||||||
Sbjct  121  AATTGTAACAGCACATGCATTTATTATAATTTTTTTATAGTTATACCAATTATAATCGG  180

Query  181  AGGATTTGGAAATTGATTAGTTCCTATTATACTAGGAGCTCCAGATATAGCATTTCCACG  240
            |||||||||||||||||||||||||||||| |||||||||||||||||||||||||||||
Sbjct  181  AGGATTTGGAAATTGATTAGTTCCTATTATGCTAGGAGCTCCAGATATAGCATTTCCACG  240

Query  241  AATAAATAATATAAGATTTTGGTTACTTCCTCCTTCTCTATTTCTACTAATTAATTCTTC  300
            ||||||||||||||||||||||||||||||||||||||||||||||||||||||||||||
Sbjct  241  AATAAATAATATAAGATTTTGGTTACTTCCTCCTTCTCTATTTCTACTAATTAATTCTTC  300

Query  301  ATTAATCGAATCAGGAGCAGGTACAGGGTGAACAGTTTAcccccccTTATCATCAAATTT  360
            |||||||||||||||||||||||||||||||||||||||||| |||||||||||||||||
Sbjct  301  ATTAATCGAATCAGGAGCAGGTACAGGGTGAACAGTTTACCCTCCCTTATCATCAAATTT  360

Query  361  ATCACACTACGGACCATCAGTAGATTTAGCTATTTTTTCTCTGCATCTTGCAGGTGCTTC  420
            |||||||||||| |||||||||||||||||||| || |||||||||||||| ||||||||
Sbjct  361  ATCACACTACGGGCCATCAGTAGATTTAGCTATCTTCTCTCTGCATCTTGCCGGTGCTTC  420

Query  421  TTCAATTTTAGGTGCAATCAATTTCATTACAACTATTGTAAATATACGATCCATTGGAAT  480
            |||||||||||||||||| |||||||||||||||||||||||||||||||||||||||||
Sbjct  421  TTCAATTTTAGGTGCAATTAATTTCATTACAACTATTGTAAATATACGATCCATTGGAAT  480

Query  481  AACAATAGAACGAATACCATTATTTGTATGATCTGTTTTAATTACTGCAATTTTATTATT  540
            ||||||||||||||||||||||||||||||||||||||||||||||||||||||||||||
Sbjct  481  AACAATAGAACGAATACCATTATTTGTATGATCTGTTTTAATTACTGCAATTTTATTATT  540

Query  541  ATTATCTTTACCAGTCTTAGCTGGCGCAATTACAATATTATTAACTGATCGAAACTTTAA  600
            |||||||||||||||||||||||| |||||||||||||||||||||||||||||||||||
Sbjct  541  ATTATCTTTACCAGTCTTAGCTGGTGCAATTACAATATTATTAACTGATCGAAACTTTAA  600

Query  601  TACATCATTCTTTGACCCTTCAGGAGGAGGTGACCCAATTCTATATCAACATTTATTT  658
            |||||||||||||||||||||||||||||||||||||||||| |||||||||||||||
Sbjct  601  TACATCATTCTTTGACCCTTCAGGAGGAGGTGACCCAATTCTGTATCAACATTTATTT  658
```

>Rhipicephalus sanguineus isolate GHF1 cytochrome oxidase subunit I (cox1) gene, partial cds; mitochondrial
Sequence ID: JX416301.1 Length: 802
Range 1: 1 to 637

```
Score:1160 bits(628), Expect:0.0,
Identities:634/637(99%),  Gaps:0/637(0%), Strand: Plus/Plus

Query  22   AGCATGATCCGGAATACTAGGATTAAGAATAAGAATATTAATCCGCATAGAATTAGGTCA  81
            ||||||||||||||||||||||||||||||||||||||||||||||||||||||||||||
Sbjct  1    AGCATGATCCGGAATACTAGGATTAAGAATAAGAATATTAATCCGCATAGAATTAGGTCA  60

Query  82   ACCAGGAACTTTAATTGGAAACGATCAAATTTATAATGTAATTGTAACAGCACATGCATT  141
            ||||||||||||||||||||||||||||||||||||||||||||||||||||||||||||
Sbjct  61   ACCAGGAACTTTAATTGGAAACGATCAAATTTATAATGTAATTGTAACAGCACATGCATT  120

Query  142  TATTATAAtttttttATAGTTATACCAATTATAATCGGAGGATTTGGAAATTGATTAGT  201
            ||||||||||||||||||||||||||||||||||||||||||||||||||||||||||||
Sbjct  121  TATTATAATTTTTTTATAGTTATACCAATTATAATCGGAGGATTTGGAAATTGATTAGT  180

Query  202  TCCTATTATACTAGGAGCTCCAGATATAGCATTTCCACGAATAAATAATATAAGATTTTG  261
            ||||||||||||||||||||||||||||||||||||||||||||||||||||||||||||
Sbjct  181  TCCTATTATACTAGGAGCTCCAGATATAGCATTTCCACGAATAAATAATATAAGATTTTG  240

Query  262  GTTACTTCCTCCTTCTCTATTTCTACTAATTAATTCTTCATTAATCGAATCAGGAGCAGG  321
            |||||||||||||||||| |||||||||||||||||||||||||||||||||||||||||
Sbjct  241  GTTACTTCCTCCTTCTCTAATTCTACTAATTAATTCTTCATTAATCGAATCAGGAGCAGG  300

Query  322  TACAGGGTGAACAGTTTAcccccccTTATCATCAAATTTATCACACTACGGACCATCAGT  381
            ||||||||||||||||||||||||||||||||||||||||||||||||||||||||||||
Sbjct  301  TACAGGGTGAACAGTTTACCCCCCCTTATCATCAAATTTATCACACTACGGACCATCAGT  360

Query  382  AGATTTAGCTATTTTTTCTCTGCATCTTGCAGGTGCTTCTTCAATTTTAGGTGCAATCAA  441
            ||||||||||||||||||||||||||||||||||||||||||||||||||||||||||||
Sbjct  361  AGATTTAGCTATTTTTTCTCTGCATCTTGCAGGTGCTTCTTCAATTTTAGGTGCAATCAA  420

Query  442  TTTCATTACAACTATTGTAAATATACGATCCATTGGAATAACAATAGAACGAATACCATT  501
            ||||||||||||||||||||||||||||||||||||||||||||||||||||||||||||
Sbjct  421  TTTCATTACAACTATTGTAAATATACGATCCATTGGAATAACAATAGAACGAATACCATT  480

Query  502  ATTTGTATGATCTGTTTTAATTACTGCAATTTTATTATTATTATCTTTACCAGTCTTAGC  561
            ||||||||||||||||||||||||||||||||||||||||||||||||||||||||||||
Sbjct  481  ATTTGTATGATCTGTTTTAATTACTGCAATTTTATTATTATTATCTTTACCAGTCTTAGC  540

Query  562  TGGCGCAATTACAATATTATTAACTGATCGAAACTTTAATACATCATTCTTTGACCCTTC  621
            |||||| ||||||||||||||||||||||||||||||||||||| |||||||||||||||
Sbjct  541  TGGCGCTATTACAATATTATTAACTGATCGAAACTTTAATACATTATTCTTTGACCCTTC  600

Query  622  AGGAGGAGGTGACCCAATTCTATATCAACATTTATTT  658
            |||||||||||||||||||||||||||||||||||||
Sbjct  601  AGGAGGAGGTGACCCAATTCTATATCAACATTTATTT  637
```

```
>Rhipicephalus sanguineus isolate Tropical lineage RJ cytochrome c oxidase
subunit I (COX1) gene, partial cds; mitochondrial
Sequence ID: MT010523.1 Length: 642
Range 1: 1 to 631
```

```
Score:1155 bits(625), Expect:0.0,
Identities:629/631(99%),  Gaps:0/631(0%), Strand: Plus/Plus

Query  28   ATCCGGAATACTAGGATTAAGAATAAGAATATTAATCCGCATAGAATTAGGTCAACCAGG  87
            ||||||||||||||||||||||||||||||||||||||||||||||||||||||||||||
Sbjct  1    ATCCGGAATACTAGGATTAAGAATAAGAATATTAATCCGCATAGAATTAGGTCAACCAGG  60

Query  88   AACTTTAATTGGAAACGATCAAATTTATAATGTAATTGTAACAGCACATGCATTTATTAT  147
            ||||||||||||||||||||||||||||||||||||||||||||||||||||||||||||
Sbjct  61   AACTTTAATTGGAAACGATCAAATTTATAATGTAATTGTAACAGCACATGCATTTATTAT  120

Query  148  AAttttttttATAGTTATACCAATTATAATCGGAGGATTTGGAAATTGATTAGTTCCTAT  207
            ||||||||||||||||||||||||||||||||||||||||||||||||||||||||||||
Sbjct  121  AATTTTTTTTATAGTTATACCAATTATAATCGGAGGATTTGGAAATTGATTAGTTCCTAT  180

Query  208  TATACTAGGAGCTCCAGATATAGCATTTCCACGAATAAATAATATAAGATTTTGGTTACT  267
            ||||||||||||||||||||||||||||||||||||||||||||||||||||||||||||
Sbjct  181  TATACTAGGAGCTCCAGATATAGCATTTCCACGAATAAATAATATAAGATTTTGGTTACT  240

Query  268  TCCTCCTTCTCTATTTCTACTAATTAATTCTTCATTAATCGAATCAGGAGCAGGTACAGG  327
            ||||||||||||||||||||||||||||||||||||||||||||||||||||||||||||
Sbjct  241  TCCTCCTTCTCTATTTCTACTAATTAATTCTTCATTAATCGAATCAGGAGCAGGTACAGG  300

Query  328  GTGAACAGTTTAcccccccTTATCATCAAATTTATCACACTACGGACCATCAGTAGATTT  387
            ||||||||||||||||||||||||||||||||||||||||||||||||||||||||||||
Sbjct  301  GTGAACAGTTTACCCCCCCTTATCATCAAATTTATCACACTACGGACCATCAGTAGATTT  360

Query  388  AGCTATTTTTCTCTGCATCTTGCAGGTGCTTCTTCAATTTTAGGTGCAATCAATTTCAT  447
            ||||||||||||||| ||||||||||||||||||||||||||||||||||||||||||||
Sbjct  361  AGCTATTTTTCTCTACATCTTGCAGGTGCTTCTTCAATTTTAGGTGCAATCAATTTCAT  420

Query  448  TACAACTATTGTAAATATACGATCCATTGGAATAACAATAGAACGAATACCATTATTTGT  507
            ||||||||||||||||||||||||||||||||||||||||||||||||||||||||||||
Sbjct  421  TACAACTATTGTAAATATACGATCCATTGGAATAACAATAGAACGAATACCATTATTTGT  480

Query  508  ATGATCTGTTTTAATTACTGCAATTTTATTATTATTATCTTTACCAGTCTTAGCTGGCGC  567
            ||||||||||||||||||||||||||||||||||||||||||||||||||||||||||||
Sbjct  481  ATGATCTGTTTTAATTACTGCAATTTTATTATTATTATCTTTACCAGTCTTAGCTGGCGC  540

Query  568  AATTACAATATTATTAACTGATCGAAACTTTAATACATCATTCTTTGACCCTTCAGGAGG  627
            ||||||||||||||||||||||||||||||||||||||||||||||||||||||||||||
Sbjct  541  AATTACAATATTATTAACTGATCGAAACTTTAATACATCATTCTTTGACCCTTCAGGAGG  600

Query  628  AGGTGACCCAATTCTATATCAACATTTATTT  658
             ||||||||||||||||||||||||||||||
Sbjct  601  GGGTGACCCAATTCTATATCAACATTTATTT  631
```

>Rhipicephalus linnaei voucher P1/22_21-1 cytochrome c oxidase subunit I (COX1) gene, partial cds; mitochondrial
Sequence ID: OM984988.1 Length: 658

```
Range 1: 1 to 658

Score:1155 bits(625), Expect:0.0,
Identities:647/658(98%),  Gaps:0/658(0%), Strand: Plus/Plus

Query  1    GACAATATACTTAATTTTTGGAGCATGATCCGGAATACTAGGATTAAGAATAAGAATATT  60
            ||||||||||||||||||||||||||||||||||||| ||||||||||||||||||||||
Sbjct  1    GACAATATACTTAATTTTTGGAGCATGATCCGGAATATTAGGATTAAGAATAAGAATATT  60

Query  61   AATCCGCATAGAATTAGGTCAACCAGGAACTTTAATTGGAAACGATCAAATTTATAATGT  120
            ||||||||||||||||||||||||||||||||||||||||||||||||||||||||||||
Sbjct  61   AATCCGCATAGAATTAGGTCAACCAGGAACTTTAATTGGAAACGATCAAATTTATAATGT  120

Query  121  AATTGTAACAGCACATGCATTTATTATAAtttttttATAGTTATACCAATTATAATCGG  180
            ||||||||||||||||||||||||||||||||||||||||||||||||||||||||||||
Sbjct  121  AATTGTAACAGCACATGCATTTATTATAATTTTTTTATAGTTATACCAATTATAATCGG  180

Query  181  AGGATTTGGAAATTGATTAGTTCCTATTATACTAGGAGCTCCAGATATAGCATTTCCACG  240
            |||||||||||||||||||||||||||||| |||||||||||||||||||||||||||||
Sbjct  181  AGGATTTGGAAATTGATTAGTTCCTATTATGCTAGGAGCTCCAGATATAGCATTTCCACG  240

Query  241  AATAAATAATATAAGATTTTGGTTACTTCCTCCTTCTCTATTTCTACTAATTAATTCTTC  300
            ||||||||||||||||||||||||||||||||||||||||||||||||||||||||||||
Sbjct  241  AATAAATAATATAAGATTTTGGTTACTTCCTCCTTCTCTATTTCTACTAATTAATTCTTC  300

Query  301  ATTAATCGAATCAGGAGCAGGTACAGGGTGAACAGTTTAcccccccTTATCATCAAATTT  360
            |||||||||||||||||||||||||||||||||||||||||| |||||||||||||||||
Sbjct  301  ATTAATCGAATCAGGAGCAGGTACAGGGTGAACAGTTTACCCTCCCTTATCATCAAATTT  360

Query  361  ATCACACTACGGACCATCAGTAGATTTAGCTATTTTTTCTCTGCATCTTGCAGGTGCTTC  420
            |||||||||||| |||||||||||||||||||| || |||||||||||||| ||||||||
Sbjct  361  ATCACACTACGGGCCATCAGTAGATTTAGCTATCTTCTCTCTGCATCTTGCCGGTGCTTC  420

Query  421  TTCAATTTAGGTGCAATCAATTTCATTACAACTATTGTAAATATACGATCCATTGGAAT  480
            ||||||||||||||||| ||||||||||||||||||||||||||||||||||||||||||
Sbjct  421  TTCAATTTAGGTGCAATTAATTTCATTACAACTATTGTAAATATACGATCCATTGGAAT  480

Query  481  AACAATAGAACGAATACCATTATTTGTATGATCTGTTTTAATTACTGCAATTTTATTATT  540
            ||||||||||||||||||||||||||||||||||||||||||||||||||||||||||||
Sbjct  481  AACAATAGAACGAATACCATTATTTGTATGATCTGTTTTAATTACTGCAATTTTATTATT  540

Query  541  ATTATCTTTACCAGTCTTAGCTGGCGCAATTACAATATTATTAACTGATCGAAACTTTAA  600
            |||||||||||||||||||||||| |||||||||||||||||||||||||||||||||||
Sbjct  541  ATTATCTTTACCAGTCTTAGCTGGTGCAATTACAATATTATTAACTGATCGAAACTTTAA  600

Query  601  TACATCATTCTTTGACCCTTCAGGAGGAGGTGACCCAATTCTATATCAACATTTATTT  658
            ||||||||| |||||||||||||||||||||||||||||||| |||||||||||||||
Sbjct  601  TACATCATTTTTTGACCCTTCAGGAGGAGGTGACCCAATTCTGTATCAACATTTATTT  658
```

>Rhipicephalus linnaei voucher P1/22_12-1 cytochrome c oxidase subunit I (COX1) gene, partial cds; mitochondrial

Sequence ID: OM984976.1 Length: 658
Range 1: 1 to 658

Score:1155 bits(625), Expect:0.0,
Identities:647/658(98%), Gaps:0/658(0%), Strand: Plus/Plus

```
Query  1    GACAATATACTTAATTTTTGGAGCATGATCCGGAATACTAGGATTAAGAATAAGAATATT  60
            ||||||||||||||||||||||||||||||||||||| ||||||||||||||||||||||
Sbjct  1    GACAATATACTTAATTTTTGGAGCATGATCCGGAATATTAGGATTAAGAATAAGAATATT  60

Query  61   AATCCGCATAGAATTAGGTCAACCAGGAACTTTAATTGGAAACGATCAAATTTATAATGT  120
            ||||||||||||||||||||||||||||||||||||||||||||||||||||||||||||
Sbjct  61   AATCCGCATAGAATTAGGTCAACCAGGAACTTTAATTGGAAACGATCAAATTTATAATGT  120

Query  121  AATTGTAACAGCACATGCATTTATTATAAttttttttATAGTTATACCAATTATAATCGG  180
            ||||||||||||||||||||||||||||||||||||||||||||||||||||||||||||
Sbjct  121  AATTGTAACAGCACATGCATTTATTATAATTTTTTTTATAGTTATACCAATTATAATCGG  180

Query  181  AGGATTTGGAAATTGATTAGTTCCTATTATACTAGGAGCTCCAGATATAGCATTTCCACG  240
            |||||||||||||||||||||||||||||||| |||||||||||||||||||||||||||
Sbjct  181  AGGATTTGGAAATTGATTAGTTCCTATTATGCTAGGAGCTCCAGATATAGCATTTCCACG  240

Query  241  AATAAATAATATAAGATTTTGGTTACTTCCTCCTTCTCTATTTCTACTAATTAATTCTTC  300
            ||||||||||||||||||||||||||||||||||||||||||||||||||||||||||||
Sbjct  241  AATAAATAATATAAGATTTTGGTTACTTCCTCCTTCTCTATTTCTACTAATTAATTCTTC  300

Query  301  ATTAATCGAATCAGGAGCAGGTACAGGGTGAACAGTTTAcccccccTTATCATCAAATTT  360
            |||||||||||||||||||||||||||||||||||||||||||| |||||||||||||||
Sbjct  301  ATTAATCGAATCAGGAGCAGGTACAGGGTGAACAGTTTACCCTCCCTTATCATCAAATTT  360

Query  361  ATCACACTACGGACCATCAGTAGATTTAGCTATTTTTTCTCTGCATCTTGCAGGTGCTTC  420
            |||||||||||| ||||||||||||||||||| || |||||||||||||| ||||||||
Sbjct  361  ATCACACTACGGGCCATCAGTAGATTTAGCTATCTTCTCTCTGCATCTTGCCGGTGCTTC  420

Query  421  TTCAATTTTAGGTGCAATCAATTTCATTACAACTATTGTAAATATACGATCCATTGGAAT  480
            |||||||||||||||||| |||||||||||||||||||||||||||||||||||||||||
Sbjct  421  TTCAATTTTAGGTGCAATTAATTTCATTACAACTATTGTAAATATACGATCCATTGGAAT  480

Query  481  AACAATAGAACGAATACCATTATTTGTATGATCTGTTTTAATTACTGCAATTTTATTATT  540
            ||||||||||||||||||||||||||||||||||||||||||||||||||||||||||||
Sbjct  481  AACAATAGAACGAATACCATTATTTGTATGATCTGTTTTAATTACTGCAATTTTATTATT  540

Query  541  ATTATCTTTACCAGTCTTAGCTGGCGCAATTACAATATTATTAACTGATCGAAACTTTAA  600
            ||||||||||||||||||||||||| ||||||||||||||||||||||||||||||||||
Sbjct  541  ATTATCTTTACCAGTCTTAGCTGGTGCAATTACAATATTATTAACTGATCGAAACTTTAA  600

Query  601  TACATCATTCTTTGACCCTTCAGGAGGAGGTGACCCAATTCTATATCAACATTTATTT  658
            ||||||||| |||||||||||||||||||||||||||||||| |||||||||||||||
Sbjct  601  TACATCATTTTTTGACCCTTCAGGAGGAGGTGACCCAATTCTGTATCAACATTTATTT  658
```

>Rhipicephalus sanguineus isolate GTF2 cytochrome oxidase subunit I (cox1) gene,

```
partial cds; mitochondrial
Sequence ID: JX416303.1 Length: 802
Range 1: 1 to 637

Score:1155 bits(625), Expect:0.0,
Identities:633/637(99%),  Gaps:0/637(0%), Strand: Plus/Plus

Query  22   AGCATGATCCGGAATACTAGGATTAAGAATAAGAATATTAATCCGCATAGAATTAGGTCA  81
            ||||||||||||||||||||||||||||||||||||||||||||||||||||||||||||
Sbjct  1    AGCATGATCCGGAATACTAGGATTAAGAATAAGAATATTAATCCGCATAGAATTAGGTCA  60

Query  82   ACCAGGAACTTTAATTGGAAACGATCAAATTTATAATGTAATTGTAACAGCACATGCATT  141
            ||||||||||||||||||||||||||||||||||||||||||||||||||||||||||||
Sbjct  61   ACCAGGAACTTTAATTGGAAACGATCAAATTTATAATGTAATTGTAACAGCACATGCATT  120

Query  142  TATTATAAttttttttATAGTTATACCAATTATAATCGGAGGATTTGGAAATTGATTAGT  201
            ||||||||||||||||||||||||||||||||||||||||||||||||| ||||||||||
Sbjct  121  TATTATAATTTTTTTTATAGTTATACCAATTATAATCGGAGGATTTGGAGATTGATTAGT  180

Query  202  TCCTATTATACTAGGAGCTCCAGATATAGCATTTCCACGAATAAATAATATAAGATTTTG  261
            ||||||||||||||||||||||||||||||||||||||||||||||||||||||||||||
Sbjct  181  TCCTATTATACTAGGAGCTCCAGATATAGCATTTCCACGAATAAATAATATAAGATTTTG  240

Query  262  GTTACTTCCTCCTTCTCTATTTCTACTAATTAATTCTTCATTAATCGAATCAGGAGCAGG  321
            ||||||||||||||||||||||||||||||||||||||||||||||||||||||||||||
Sbjct  241  GTTACTTCCTCCTTCTCTATTTCTACTAATTAATTCTTCATTAATCGAATCAGGAGCAGG  300

Query  322  TACAGGGTGAACAGTTTAcccccccTTATCATCAAATTTATCACACTACGGACCATCAGT  381
            ||||||||||||||||||||||||||||||||||||||||||||||||||||||||||||
Sbjct  301  TACAGGGTGAACAGTTTACCCCCCCTTATCATCAAATTTATCACACTACGGACCATCAGT  360

Query  382  AGATTTAGCTATTTTTTCTCTGCATCTTGCAGGTGCTTCTTCAATTTTAGGTGCAATCAA  441
            ||||||||||||||||||||||||||||||||||||||||||||| ||||||||||||||
Sbjct  361  AGATTTAGCTATTTTTTCTCTGCATCTTGCAGGTGCTTCTTCAATCTTAGGTGCAATCAA  420

Query  442  TTTCATTACAACTATTGTAAATATACGATCCATTGGAATAACAATAGAACGAATACCATT  501
            ||||||||||||||||||||||| ||||||||||||||||||||||||||||||||||||
Sbjct  421  TTTCATTACAACTATTGTAAATACACGATCCATTGGAATAACAATAGAACGAATACCATT  480

Query  502  ATTTGTATGATCTGTTTTAATTACTGCAATTTTATTATTATTATCTTTACCAGTCTTAGC  561
            |||||||||||||||||||||||||||||||||||||||||||| |||||||||||||||
Sbjct  481  ATTTGTATGATCTGTTTTAATTACTGCAATTTTATTATTATTATTTTTACCAGTCTTAGC  540

Query  562  TGGCGCAATTACAATATTATTAACTGATCGAAACTTTAATACATCATTCTTTGACCCTTC  621
            ||||||||||||||||||||||||||||||||||||||||||||||||||||||||||||
Sbjct  541  TGGCGCAATTACAATATTATTAACTGATCGAAACTTTAATACATCATTCTTTGACCCTTC  600

Query  622  AGGAGGAGGTGACCCAATTCTATATCAACATTTATTT  658
            |||||||||||||||||||||||||||||||||||||
Sbjct  601  AGGAGGAGGTGACCCAATTCTATATCAACATTTATTT  637
```

```
>Rhipicephalus sanguineus sensu lato 'tropical lineage' isolate CV3042
cytochrome c oxidase subunit I (COI) gene, partial cds; mitochondrial
Sequence ID: MF426003.1 Length: 826
Range 1: 3 to 650

Score:1153 bits(624), Expect:0.0,
Identities:640/648(99%),  Gaps:0/648(0%), Strand: Plus/Plus

Query  11   TTAATTTTTGGAGCATGATCCGGAATACTAGGATTAAGAATAAGAATATTAATCCGCATA  70
            ||||||||||||||||||||||||||||||||||||||||||||||||||||| ||||||
Sbjct  3    TTAATTTTTGGAGCATGATCCGGAATACTAGGATTAAGAATAAGAATATTAATTCGCATA  62

Query  71   GAATTAGGTCAACCAGGAACTTTAATTGGAAACGATCAAATTTATAATGTAATTGTAACA  130
            ||||||||||||||||||||||||||||||||||||||||||||||||||||||||||||
Sbjct  63   GAATTAGGTCAACCAGGAACTTTAATTGGAAACGATCAAATTTATAATGTAATTGTAACA  122

Query  131  GCACATGCATTTATTATAAttttttttATAGTTATACCAATTATAATCGGAGGATTTGGA  190
            ||||||||||||||||||||||||||||||||||||||||||||||||||||||||||||
Sbjct  123  GCACATGCATTTATTATAATTTTTTTTATAGTTATACCAATTATAATCGGAGGATTTGGA  182

Query  191  AATTGATTAGTTCCTATTATACTAGGAGCTCCAGATATAGCATTTCCACGAATAAATAAT  250
            |||||||||||||||||||||||||||||||||||||||||||||||||||||||| |||
Sbjct  183  AATTGATTAGTTCCTATTATACTAGGAGCTCCAGATATAGCATTTCCACGAATAAACAAT  242

Query  251  ATAAGATTTTGGTTACTTCCTCCTTCTCTATTTCTACTAATTAATTCTTCATTAATCGAA  310
            ||||||||||||||||||||||||||||||||||||||||||||||||||||||||||||
Sbjct  243  ATAAGATTTTGGTTACTTCCTCCTTCTCTATTTCTACTAATTAATTCTTCATTAATCGAA  302

Query  311  TCAGGAGCAGGTACAGGGTGAACAGTTTAcccccccTTATCATCAAATTTATCACACTAC  370
            |||||||| |||||||||||||||||||||||||||||||||||||||||||||||||||
Sbjct  303  TCAGGAGCGGGTACAGGGTGAACAGTTTACCCCCCCTTATCATCAAATTTATCACACTAC  362

Query  371  GGACCATCAGTAGATTTAGCTATTTTTCTCTGCATCTTGCAGGTGCTTCTTCAATTTTA  430
            ||||||||||||||||||||||| ||||||||||||||||||||||||||||||||||||
Sbjct  363  GGACCATCAGTAGATTTAGCTATCTTTTCTCTGCATCTTGCAGGTGCTTCTTCAATTTTA  422

Query  431  GGTGCAATCAATTTCATTACAACTATTGTAAATATACGATCCATTGGAATAACAATAGAA  490
            || |||||||||||||||||||||||||||||||||||||||||||||||||||||||||
Sbjct  423  GGCGCAATCAATTTCATTACAACTATTGTAAATATACGATCCATTGGAATAACAATAGAA  482

Query  491  CGAATACCATTATTTGTATGATCTGTTTTAATTACTGCAATTTTATTATTATTATCTTTA  550
            ||||||||||||||||||||||||||||||||||||||||||||||||||||||||||||
Sbjct  483  CGAATACCATTATTTGTATGATCTGTTTTAATTACTGCAATTTTATTATTATTATCTTTA  542

Query  551  CCAGTCTTAGCTGGCGCAATTACAATATTATTAACTGATCGAAACTTTAATACATCATTC  610
            |||||||||||||| |||||||||||||| ||||||||||||||||||||||||||||||
Sbjct  543  CCAGTCTTAGCTGGTGCAATTACAATATTGTTAACTGATCGAAACTTTAATACATCATTC  602

Query  611  TTTGACCCTTCAGGAGGAGGTGACCCAATTCTATATCAACATTTATTT  658
            |||||||||||||||||||||||||||||||| |||||||||||||||
Sbjct  603  TTTGACCCTTCAGGAGGAGGTGACCCAATTCTGTATCAACATTTATTT  650
```

```
>Rhipicephalus sanguineus voucher AMMS-RS-4 cytochrome c oxidase subunit I-like
(COI) gene, partial sequence; mitochondrial
Sequence ID: HM193874.1 Length: 665
Range 1: 31 to 665

Score:1146 bits(620), Expect:0.0,
Identities:631/636(99%),  Gaps:1/636(0%), Strand: Plus/Plus

Query  1    GACAATATACTTAATTTTTGGAGCATGATCCGGAATACTAGGATTAAGAATAAGAATATT  60
            ||||||||||||||||||||||||||||||||||||||||||||||||||||||||||||
Sbjct  31   GACAATATACTTAATTTTTGGAGCATGATCCGGAATACTAGGATTAAGAATAAGAATATT  90

Query  61   AATCCGCATAGAATTAGGTCAACCAGGAACTTTAATTGGAAACGATCAAATTTATAATGT  120
            ||||||||||||||||||||||||||||||||||||||||||||||||||||||||||||
Sbjct  91   AATCCGCATAGAATTAGGTCAACCAGGAACTTTAATTGGAAACGATCAAATTTATAATGT  150

Query  121  AATTGTAACAGCACATGCATTTATTATAAtttttttATAGTTATACCAATTATAATCGG  180
            ||||||||||||||||||||||||||||||||||||||||||||||||||||||||||||
Sbjct  151  AATTGTAACAGCACATGCATTTATTATAATTTTTTTATAGTTATACCAATTATAATCGG  210

Query  181  AGGATTTGGAAATTGATTAGTTCCTATTATACTAGGAGCTCCAGATATAGCATTTCCACG  240
            ||||||||||||||||||||||||||||||||||||||||||||||||||||||||||||
Sbjct  211  AGGATTTGGAAATTGATTAGTTCCTATTATACTAGGAGCTCCAGATATAGCATTTCCACG  270

Query  241  AATAAATAATATAAGATTTTGGTTACTTCCTCCTTCTCTATTTCTACTAATTAATTCTTC  300
            ||||||||||||||||||||||||||||||||||||||||||||||||||||||||||||
Sbjct  271  AATAAATAATATAAGATTTTGGTTACTTCCTCCTTCTCTATTTCTACTAATTAATTCTTC  330

Query  301  ATTAATCGAATCAGGAGCAGGTACAGGGTGAACAGTTTAcccccccTTATCATCAAATTT  360
            ||||||||||||||||||||||||||||||||||||||||||||||||||||||||||||
Sbjct  331  ATTAATCGAATCAGGAGCAGGTACAGGGTGAACAGTTTACCCCCCCTTATCATCAAATTT  390

Query  361  ATCACACTACGGACCATCAGTAGATTTAGCTATTTTTTCTCTGCATCTTGCAGGTGCTTC  420
            ||||||||||||||||||||||||||||||||||||||||||||||||||||||||||||
Sbjct  391  ATCACACTACGGACCATCAGTAGATTTAGCTATTTTTTCTCTGCATCTTGCAGGTGCTTC  450

Query  421  TTCAATTTTAGGTGCAATCAATTTCATTACAACTATTGTAAATATACGATCCATTGGAAT  480
            ||||||||||||||||||||||||||||||||||||||||||||||||||||||||||||
Sbjct  451  TTCAATTTTAGGTGCAATCAATTTCATTACAACTATTGTAAATATACGATCCATTGGAAT  510

Query  481  AACAATAGAACGAATACCATTATTTGTATGATCTGTTTTAATTACTGCAATTTTATTATT  540
            ||||||||||||||| ||||||||||||||||||||||||||||||||||||||||||||
Sbjct  511  AACAATAGAACGAATCCCATTATTTGTATGATCTGTTTTAATTACTGCAATTTTATTATT  570

Query  541  ATTATCTTTACCAGTCTTAGCTGGCGCAATTACAATATTATTAACTGATCGAAACTTTAA  600
            |||||||||||||||||||||||||||||||||||| |||||||||||||||||||||||
Sbjct  571  ATTATCTTTACCAGTCTTAGCTGGCGCAATTACAATAATATTAACTGATCGAAACTTTAA  630

Query  601  TACATCATTCTTTGACCCTTCAGGAGGAGGTGACCC  636
            ||||||||||||||||||  |||||||||| |||||
Sbjct  631  TACATCATTCTTTGACCCAGCAGGAGGAGG-GACCC  665
```

>Rhipicephalus sanguineus isolate Abidjan cytochrome oxidase subunit I (COI) gene, partial cds; mitochondrial
Sequence ID: KX757914.1 Length: 630
Range 1: 1 to 620

Score:1140 bits(617), Expect:0.0,
Identities:619/620(99%), Gaps:0/620(0%), Strand: Plus/Plus

```
Query  39   TAGGATTAAGAATAAGAATATTAATCCGCATAGAATTAGGTCAACCAGGAACTTTAATTG  98
            ||||||||||||||||||||||||||||||||||||||||||||||||||||||||||||
Sbjct  1    TAGGATTAAGAATAAGAATATTAATCCGCATAGAATTAGGTCAACCAGGAACTTTAATTG  60

Query  99   GAAACGATCAAATTTATAATGTAATTGTAACAGCACATGCATTTATTATAAttttttttA  158
            ||||||||||||||||||||||||||||||||||||||||||||||||||||||||||||
Sbjct  61   GAAACGATCAAATTTATAATGTAATTGTAACAGCACATGCATTTATTATAATTTTTTTTA  120

Query  159  TAGTTATACCAATTATAATCGGAGGATTTGGAAATTGATTAGTTCCTATTATACTAGGAG  218
            |||||||||||||||||||||||||||||||||||||||||||||||||||||||||| |
Sbjct  121  TAGTTATACCAATTATAATCGGAGGATTTGGAAATTGATTAGTTCCTATTATACTAGGGG  180

Query  219  CTCCAGATATAGCATTTCCACGAATAAATAATATAAGATTTTGGTTACTTCCTCCTTCTC  278
            ||||||||||||||||||||||||||||||||||||||||||||||||||||||||||||
Sbjct  181  CTCCAGATATAGCATTTCCACGAATAAATAATATAAGATTTTGGTTACTTCCTCCTTCTC  240

Query  279  TATTTCTACTAATTAATTCTTCATTAATCGAATCAGGAGCAGGTACAGGGTGAACAGTTT  338
            ||||||||||||||||||||||||||||||||||||||||||||||||||||||||||||
Sbjct  241  TATTTCTACTAATTAATTCTTCATTAATCGAATCAGGAGCAGGTACAGGGTGAACAGTTT  300

Query  339  AcccccccTTATCATCAAATTTATCACACTACGGACCATCAGTAGATTTAGCTATTTTTT  398
            ||||||||||||||||||||||||||||||||||||||||||||||||||||||||||||
Sbjct  301  ACCCCCCCTTATCATCAAATTTATCACACTACGGACCATCAGTAGATTTAGCTATTTTTT  360

Query  399  CTCTGCATCTTGCAGGTGCTTCTTCAATTTTAGGTGCAATCAATTTCATTACAACTATTG  458
            ||||||||||||||||||||||||||||||||||||||||||||||||||||||||||||
Sbjct  361  CTCTGCATCTTGCAGGTGCTTCTTCAATTTTAGGTGCAATCAATTTCATTACAACTATTG  420

Query  459  TAAATATACGATCCATTGGAATAACAATAGAACGAATACCATTATTTGTATGATCTGTTT  518
            ||||||||||||||||||||||||||||||||||||||||||||||||||||||||||||
Sbjct  421  TAAATATACGATCCATTGGAATAACAATAGAACGAATACCATTATTTGTATGATCTGTTT  480

Query  519  TAATTACTGCAATTTTATTATTATTATCTTTACCAGTCTTAGCTGGCGCAATTACAATAT  578
            ||||||||||||||||||||||||||||||||||||||||||||||||||||||||||||
Sbjct  481  TAATTACTGCAATTTTATTATTATTATCTTTACCAGTCTTAGCTGGCGCAATTACAATAT  540

Query  579  TATTAACTGATCGAAACTTTAATACATCATTCTTTGACCCTTCAGGAGGAGGTGACCCAA  638
            ||||||||||||||||||||||||||||||||||||||||||||||||||||||||||||
Sbjct  541  TATTAACTGATCGAAACTTTAATACATCATTCTTTGACCCTTCAGGAGGAGGTGACCCAA  600

Query  639  TTCTATATCAACATTTATTT  658
            ||||||||||||||||||||
Sbjct  601  TTCTATATCAACATTTATTT  620
```

>Rhipicephalus sanguineus isolate LIC5960 cytochrome oxidase subunit 1 (COI) gene, partial cds; mitochondrial
Sequence ID: KX383800.1 Length: 768
>Rhipicephalus sanguineus isolate LIC5766D cytochrome oxidase subunit 1 (COI) gene, partial cds; mitochondrial
Sequence ID: KX383815.1 Length: 768
Range 1: 1 to 642

Score:1136 bits(615), Expect:0.0,
Identities:633/642(99%), Gaps:0/642(0%), Strand: Plus/Plus

```
Query  17   TTTGGAGCATGATCCGGAATACTAGGATTAAGAATAAGAATATTAATCCGCATAGAATTA  76
            ||||||||||||||||||||||||||||||||||||||||||||||| ||||||||||||
Sbjct  1    TTTGGAGCATGATCCGGAATACTAGGATTAAGAATAAGAATATTAATTCGCATAGAATTA  60

Query  77   GGTCAACCAGGAACTTTAATTGGAAACGATCAAATTTATAATGTAATTGTAACAGCACAT  136
            |||||||||||||||||||||||||||||||||||||||||||||||||| |||||||||
Sbjct  61   GGTCAACCAGGAACTTTAATTGGAAACGATCAAATTTATAATGTAATTGTGACAGCACAT  120

Query  137  GCATTTATTATAAttttttttATAGTTATACCAATTATAATCGGAGGATTTGGAAATTGA  196
            |||||||||||||| |||||||||||||||||||||||||||||||||||||||||||||
Sbjct  121  GCATTTATTATAATCTTTTTTATAGTTATACCAATTATAATCGGAGGATTTGGAAATTGA  180

Query  197  TTAGTTCCTATTATACTAGGAGCTCCAGATATAGCATTTCCACGAATAAATAATATAAGA  256
            |||||||||||||||||||||||||||||||||||||||||||||||||| |||||||||
Sbjct  181  TTAGTTCCTATTATACTAGGAGCTCCAGATATAGCATTTCCACGAATAAACAATATAAGA  240

Query  257  TTTTGGTTACTTCCTCCTTCTCTATTTCTACTAATTAATTCTTCATTAATCGAATCAGGA  316
            ||||||||||||||||||||||||||||||||||||||||||||||||||||||||||||
Sbjct  241  TTTTGGTTACTTCCTCCTTCTCTATTTCTACTAATTAATTCTTCATTAATCGAATCAGGA  300

Query  317  GCAGGTACAGGGTGAACAGTTTAcccccccTTATCATCAAATTTATCACACTACGGACCA  376
            ||||||||||||||||||||||||||||||||||||||||||||||||||||||||||||
Sbjct  301  GCAGGTACAGGGTGAACAGTTTACCCCCCCTTATCATCAAATTTATCACACTACGGACCA  360

Query  377  TCAGTAGATTTAGCTATTTTTTCTCTGCATCTTGCAGGTGCTTCTTCAATTTTAGGTGCA  436
            ||||||||||||||||| ||||||||||||||||||||||||||||||||||||||| |||
Sbjct  361  TCAGTAGATTTAGCTATCTTTTCTCTGCATCTTGCAGGTGCTTCTTCAATTTTAGGCGCA  420

Query  437  ATCAATTTCATTACAACTATTGTAAATATACGATCCATTGGAATAACAATAGAACGAATA  496
            ||||||||||||||||||||||||||||||||||||||||||||||||||||||||||||
Sbjct  421  ATCAATTTCATTACAACTATTGTAAATATACGATCCATTGGAATAACAATAGAACGAATA  480

Query  497  CCATTATTTGTATGATCTGTTTTAATTACTGCAATTTTATTATTATTATCTTTACCAGTC  556
            |||||||||||||||||||||||||||||||||||||||||||| |||||||||||||||
Sbjct  481  CCATTATTTGTATGATCTGTTTTAATTACTGCAATTTTATTATTGTTATCTTTACCAGTC  540

Query  557  TTAGCTGGCGCAATTACAATATTATTAACTGATCGAAACTTTAATACATCATTCTTTGAC  616
            |||||||| |||||||||||||||||||||||||||||||||||||||||||||||||||
Sbjct  541  TTAGCTGGTGCAATTACAATATTATTAACTGATCGAAACTTTAATACATCATTCTTTGAC  600
```

```
Query  617  CCTTCAGGAGGAGGTGACCCAATTCTATATCAACATTTATTT  658
            |||||||||||||||||||||||||| |||||||||||||||
Sbjct  601  CCTTCAGGAGGAGGTGACCCAATTCTGTATCAACATTTATTT  642
```

>Rhipicephalus sanguineus isolate LIC5682B cytochrome oxidase subunit 1 (COI) gene, partial cds; mitochondrial
Sequence ID: KX383796.1 Length: 744
Range 1: 1 to 618

Score:1131 bits(612), Expect:0.0,
Identities:616/618(99%), Gaps:0/618(0%), Strand: Plus/Plus

```
Query  41   GGATTAAGAATAAGAATATTAATCCGCATAGAATTAGGTCAACCAGGAACTTTAATTGGA  100
            ||||||||||||||||||||||||||||||||||||||||||||||||||||||||||||
Sbjct  1    GGATTAAGAATAAGAATATTAATCCGCATAGAATTAGGTCAACCAGGAACTTTAATTGGA  60

Query  101  AACGATCAAATTTATAATGTAATTGTAACAGCACATGCATTTATTATAAttttttttATA  160
            ||||||||||||||||||||||||||||||||||||||||||||||||||||||||||||
Sbjct  61   AACGATCAAATTTATAATGTAATTGTAACAGCACATGCATTTATTATAATTTTTTTTATA  120

Query  161  GTTATACCAATTATAATCGGAGGATTTGGAAATTGATTAGTTCCTATTATACTAGGAGCT  220
            ||||||||||||||||||||||||||||||||||||||||||||||||||||||||||||
Sbjct  121  GTTATACCAATTATAATCGGAGGATTTGGAAATTGATTAGTTCCTATTATACTAGGAGCT  180

Query  221  CCAGATATAGCATTTCCACGAATAAATAATATAAGATTTTGGTTACTTCCTCCTTCTCTA  280
            ||||||||||||||||||||||||||||||||||||||||||||||||||||||||||||
Sbjct  181  CCAGATATAGCATTTCCACGAATAAATAATATAAGATTTTGGTTACTTCCTCCTTCTCTA  240

Query  281  TTTCTACTAATTAATTCTTCATTAATCGAATCAGGAGCAGGTACAGGGTGAACAGTTTAc  340
            ||||||||||||||||||||||||||||||||||||||||||||||||||||||||||||
Sbjct  241  TTTCTACTAATTAATTCTTCATTAATCGAATCAGGAGCAGGTACAGGGTGAACAGTTTAC  300

Query  341  ccccccTTATCATCAAATTTATCACACTACGGACCATCAGTAGATTTAGCTATTTTTTCT  400
            ||||||||||||||||||||||||||||||||||||||||||||||||||||||||||||
Sbjct  301  CCCCCCTTATCATCAAATTTATCACACTACGGACCATCAGTAGATTTAGCTATTTTTTCT  360

Query  401  CTGCATCTTGCAGGTGCTTCTTCAATTTTAGGTGCAATCAATTTCATTACAACTATTGTA  460
            || |||||||||||||||||||||||||||||||||||||||||||||||||||||||||
Sbjct  361  CTACATCTTGCAGGTGCTTCTTCAATTTTAGGTGCAATCAATTTCATTACAACTATTGTA  420

Query  461  AATATACGATCCATTGGAATAACAATAGAACGAATACCATTATTTGTATGATCTGTTTTA  520
            ||||||||||||||||||||||||||||||||||||||||||||||||||||||||||||
Sbjct  421  AATATACGATCCATTGGAATAACAATAGAACGAATACCATTATTTGTATGATCTGTTTTA  480

Query  521  ATTACTGCAATTTTATTATTATTATCTTTACCAGTCTTAGCTGGCGCAATTACAATATTA  580
            ||||||||||||||||||||||||||||||||||||||||||||||||||||||||||||
Sbjct  481  ATTACTGCAATTTTATTATTATTATCTTTACCAGTCTTAGCTGGCGCAATTACAATATTA  540

Query  581  TTAACTGATCGAAACTTTAATACATCATTCTTTGACCCTTCAGGAGGAGGTGACCCAATT  640
            ||||||||||||||||||||||||||||||||||||||||||||||| ||||||||||||
Sbjct  541  TTAACTGATCGAAACTTTAATACATCATTCTTTGACCCTTCAGGAGGGGGTGACCCAATT  600
```

```
Query  641  CTATATCAACATTTATTT  658
            ||||||||||||||||||
Sbjct  601  CTATATCAACATTTATTT  618
```

>Rhipicephalus sanguineus isolate LIC5958B cytochrome oxidase subunit 1 (COI) gene, partial cds; mitochondrial
Sequence ID: KX383798.1 Length: 698
Range 1: 1 to 637

Score:1127 bits(610), Expect:0.0,
Identities:628/637(99%), Gaps:0/637(0%), Strand: Plus/Plus

```
Query  22   AGCATGATCCGGAATACTAGGATTAAGAATAAGAATATTAATCCGCATAGAATTAGGTCA  81
            |||||||||||||||||||||||||||||||||||||||||| |||||||||||||||||
Sbjct  1    AGCATGATCCGGAATACTAGGATTAAGAATAAGAATATTAATTCGCATAGAATTAGGTCA  60

Query  82   ACCAGGAACTTTAATTGGAAACGATCAAATTTATAATGTAATTGTAACAGCACATGCATT  141
            ||||||||||||||||||||||||||||||||||||||||||||| ||||||||||||||
Sbjct  61   ACCAGGAACTTTAATTGGAAACGATCAAATTTATAATGTAATTGTGACAGCACATGCATT  120

Query  142  TATTATAAttttttttATAGTTATACCAATTATAATCGGAGGATTTGGAAATTGATTAGT  201
            ||||||||| ||||||||||||||||||||||||||||||||||||||||||||||||||
Sbjct  121  TATTATAATCTTTTTTATAGTTATACCAATTATAATCGGAGGATTTGGAAATTGATTAGT  180

Query  202  TCCTATTATACTAGGAGCTCCAGATATAGCATTTCCACGAATAAATAATATAAGATTTTG  261
            ||||||||||||||||||||||||||||||||||||||||||||| ||||||||||||||
Sbjct  181  TCCTATTATACTAGGAGCTCCAGATATAGCATTTCCACGAATAAACAATATAAGATTTTG  240

Query  262  GTTACTTCCTCCTTCTCTATTTCTACTAATTAATTCTTCATTAATCGAATCAGGAGCAGG  321
            ||||||||||||||||||||||||||||||||||||||||||||||||||||||||||||
Sbjct  241  GTTACTTCCTCCTTCTCTATTTCTACTAATTAATTCTTCATTAATCGAATCAGGAGCAGG  300

Query  322  TACAGGGTGAACAGTTTAccccccccTTATCATCAAATTTATCACACTACGGACCATCAGT  381
            ||||||||||||||||||||||||||||||||||||||||||||||||||||||||||||
Sbjct  301  TACAGGGTGAACAGTTTACCCCCCCTTATCATCAAATTTATCACACTACGGACCATCAGT  360

Query  382  AGATTTAGCTATTTTTTCTCTGCATCTTGCAGGTGCTTCTTCAATTTTAGGTGCAATCAA  441
            |||||||||||| ||||||||||||||||||||||||||||||||||||||| |||||||
Sbjct  361  AGATTTAGCTATCTTTTCTCTGCATCTTGCAGGTGCTTCTTCAATTTTAGGCGCAATCAA  420

Query  442  TTTCATTACAACTATTGTAAATATACGATCCATTGGAATAACAATAGAACGAATACCATT  501
            ||||||||||||||||||||||||||||||||||||||||||||||||||||||||||||
Sbjct  421  TTTCATTACAACTATTGTAAATATACGATCCATTGGAATAACAATAGAACGAATACCATT  480

Query  502  ATTTGTATGATCTGTTTTAATTACTGCAATTTTATTATTATTATCTTTACCAGTCTTAGC  561
            ||||||||||||||||||||||||||||||||||||||| ||||||||||||||||||||
Sbjct  481  ATTTGTATGATCTGTTTTAATTACTGCAATTTTATTATTGTTATCTTTACCAGTCTTAGC  540

Query  562  TGGCGCAATTACAATATTATTAACTGATCGAAACTTTAATACATCATTCTTTGACCCTTC  621
            ||| ||||||||||||||||||||||||||||||||||||||||||||||||||||||||
```

```
Sbjct  541   TGGTGCAATTACAATATTATTAACTGATCGAAACTTTAATACATCATTCTTTGACCCTTC  600

Query  622   AGGAGGAGGTGACCCAATTCTATATCAACATTTATTT  658
             ||||||||||||||||||||| |||||||||||||||
Sbjct  601   AGGAGGAGGTGACCCAATTCTGTATCAACATTTATTT  637
```

>Rhipicephalus sanguineus isolate LIC5584D cytochrome oxidase subunit 1 (COI) gene, partial cds; mitochondrial
Sequence ID: KX383814.1 Length: 768
Range 1: 1 to 642

Score:1125 bits(609), Expect:0.0,
Identities:631/642(98%), Gaps:0/642(0%), Strand: Plus/Plus

```
Query  17    TTTGGAGCATGATCCGGAATACTAGGATTAAGAATAAGAATATTAATCCGCATAGAATTA  76
             ||||||||||||||||||||||||||||||||||||||||||||||| ||||||||||||
Sbjct  1     TTTGGAGCATGATCCGGAATACTAGGATTAAGAATAAGAATATTAATTCGCATAGAATTA  60

Query  77    GGTCAACCAGGAACTTTAATTGGAAACGATCAAATTTATAATGTAATTGTAACAGCACAT  136
             |||||||||||||||||||||||||||||||||||||||||||||||||| |||||||||
Sbjct  61    GGTCAACCAGGAACTTTAATTGGAAACGATCAAATTTATAATGTAATTGTGACAGCACAT  120

Query  137   GCATTTATTATAAttttttttATAGTTATACCAATTATAATCGGAGGATTTGGAAATTGA  196
             |||||||||||||| |||||||||||||||||||||||||||||||||||||||||||||
Sbjct  121   GCATTTATTATAATCTTTTTTATAGTTATACCAATTATAATCGGAGGATTTGGAAATTGA  180

Query  197   TTAGTTCCTATTATACTAGGAGCTCCAGATATAGCATTTCCACGAATAAATAATATAAGA  256
             |||||||||||||||||||||||||||||||||||||||||||||||||| |||||||||
Sbjct  181   TTAGTTCCTATTATACTAGGAGCTCCAGATATAGCATTTCCACGAATAAACAATATAAGA  240

Query  257   TTTTGGTTACTTCCTCCTTCTCTATTTCTACTAATTAATTCTTCATTAATCGAATCAGGA  316
             ||||||||||||||||||||||||||||||||||||||||||||||||||||||||||||
Sbjct  241   TTTTGGTTACTTCCTCCTTCTCTATTTCTACTAATTAATTCTTCATTAATCGAATCAGGA  300

Query  317   GCAGGTACAGGGTGAACAGTTTAcccccccTTATCATCAAATTTATCACACTACGGACCA  376
             ||||||||||||||||||||||||||||||||||||||||||||||||||||||||| ||
Sbjct  301   GCAGGTACAGGGTGAACAGTTTACCCCCCCTTATCATCAAATTTATCACACTACGGATCA  360

Query  377   TCAGTAGATTTAGCTATTTTTTCTCTGCATCTTGCAGGTGCTTCTTCAATTTTAGGTGCA  436
             ||||||||||||||||| ||||||||||||||||||||||||||||||||||||||| |||
Sbjct  361   TCAGTAGATTTAGCTATCTTTTCTCTGCATCTTGCAGGTGCTTCTTCAATTTTAGGCGCA  420

Query  437   ATCAATTTCATTACAACTATTGTAAATATACGATCCATTGGAATAACAATAGAACGAATA  496
             ||||||||||||||||||||||||||||||||||||||||||||||||||||||||||||
Sbjct  421   ATCAATTTCATTACAACTATTGTAAATATACGATCCATTGGAATAACAATAGAACGAATA  480

Query  497   CCATTATTTGTATGATCTGTTTTAATTACTGCAATTTTATTATTATTATCTTTACCAGTC  556
             |||||||||||||||||||||||||||||||||||||||||||| ||||||||||||||
Sbjct  481   CCATTATTTGTATGATCTGTTTTAATTACTGCAATTTTATTATTGTTATCTTTACCAGTT  540

Query  557   TTAGCTGGCGCAATTACAATATTATTAACTGATCGAAACTTTAATACATCATTCTTTGAC  616
```

```
           |||||||| |||||||||||||||||||||||||||||||||||||||||||||||||||
Sbjct  541  TTAGCTGGTGCAATTACAATATTATTAACTGATCGAAACTTTAATACATCATTCTTTGAC  600

Query  617  CCTTCAGGAGGAGGTGACCCAATTCTATATCAACATTTATTT  658
            ||||||||||||||||||||||||| ||||||||||||||||
Sbjct  601  CCTTCAGGAGGAGGTGACCCAATTCTGTATCAACATTTATTT  642
```

>Rhipicephalus sanguineus isolate LIC5755 cytochrome oxidase subunit 1 (COI) gene, partial cds; mitochondrial
Sequence ID: KX383801.1 Length: 763
Range 1: 1 to 637

Score:1122 bits(607), Expect:0.0,
Identities:627/637(98%), Gaps:0/637(0%), Strand: Plus/Plus

```
Query  22   AGCATGATCCGGAATACTAGGATTAAGAATAAGAATATTAATCCGCATAGAATTAGGTCA  81
            ||||||||||||||||||||||||||||||||||||||||||| |||||||||||||||||
Sbjct  1    AGCATGATCCGGAATACTAGGATTAAGAATAAGAATATTAATTCGCATAGAATTAGGTCA  60

Query  82   ACCAGGAACTTTAATTGGAAACGATCAAATTTATAATGTAATTGTAACAGCACATGCATT  141
            |||||||||||||||||||||||||||||||||||||||||||||| ||||||||||||||
Sbjct  61   ACCAGGAACTTTAATTGGAAACGATCAAATTTATAATGTAATTGTGACAGCACATGCATT  120

Query  142  TATTATAAtttttttATAGTTATACCAATTATAATCGGAGGATTTGGAAATTGATTAGT  201
            ||||||||| ||||||||||||||||||||||||||||||||||||||||||||||||||
Sbjct  121  TATTATAATCTTTTTTATAGTTATACCAATTATAATCGGAGGATTTGGAAATTGATTAGT  180

Query  202  TCCTATTATACTAGGAGCTCCAGATATAGCATTTCCACGAATAAATAATATAAGATTTTG  261
            |||||||||||||||||||||||||||||||||||||||||||||| ||||||||||||||
Sbjct  181  TCCTATTATACTAGGAGCTCCAGATATAGCATTTCCACGAATAAACAATATAAGATTTTG  240

Query  262  GTTACTTCCTCCTTCTCTATTTCTACTAATTAATTCTTCATTAATCGAATCAGGAGCAGG  321
            ||||||||||||||||||||||||||||||||||||||||||||||||||||||||||||
Sbjct  241  GTTACTTCCTCCTTCTCTATTTCTACTAATTAATTCTTCATTAATCGAATCAGGAGCAGG  300

Query  322  TACAGGGTGAACAGTTTAcccccccTTATCATCAAATTTATCACACTACGGACCATCAGT  381
            |||||||||||||||||||||||||||||||||||||||||||||||||||| |||||||
Sbjct  301  TACAGGGTGAACAGTTTACCCCCCCTTATCATCAAATTTATCACACTACGGATCATCAGT  360

Query  382  AGATTTAGCTATTTTTTCTCTGCATCTTGCAGGTGCTTCTTCAATTTTAGGTGCAATCAA  441
            ||||||||||||| ||||||||||||||||||||||||||||||||||||| ||||||||
Sbjct  361  AGATTTAGCTATCTTTTCTCTGCATCTTGCAGGTGCTTCTTCAATTTTAGGCGCAATCAA  420

Query  442  TTTCATTACAACTATTGTAAATATACGATCCATTGGAATAACAATAGAACGAATACCATT  501
            ||||||||||||||||||||||||||||||||||||||||||||||||||||||||||||
Sbjct  421  TTTCATTACAACTATTGTAAATATACGATCCATTGGAATAACAATAGAACGAATACCATT  480

Query  502  ATTTGTATGATCTGTTTTAATTACTGCAATTTTATTATTATTATCTTTACCAGTCTTAGC  561
            ||||||||||||||||||||||||||||||||||||||| ||||||||||||||||||||
Sbjct  481  ATTTGTATGATCTGTTTTAATTACTGCAATTTTATTATTGTTATCTTTACCAGTCTTAGC  540
```

```
Query  562  TGGCGCAATTACAATATTATTAACTGATCGAAACTTTAATACATCATTCTTTGACCCTTC  621
            ||| ||||||||||||||||||||||||||||||||||||||||||||||||||||||||
Sbjct  541  TGGTGCAATTACAATATTATTAACTGATCGAAACTTTAATACATCATTCTTTGACCCTTC  600

Query  622  AGGAGGAGGTGACCCAATTCTATATCAACATTTATTT  658
            ||||||||||||||||||||| |||||||||||||||
Sbjct  601  AGGAGGAGGTGACCCAATTCTGTATCAACATTTATTT  637
```

>Rhipicephalus sanguineus isolate 400 cytochrome c oxidase subunit I (COX1) gene, partial cds; mitochondrial
Sequence ID: MW558150.1 Length: 657
Range 1: 1 to 639

Score:1120 bits(606), Expect:0.0,
Identities:628/639(98%), Gaps:0/639(0%), Strand: Plus/Plus

```
Query  20   GGAGCATGATCCGGAATACTAGGATTAAGAATAAGAATATTAATCCGCATAGAATTAGGT  79
            |||||| ||||||||||| |||||||||||||||||||||||||||||||||||||||||
Sbjct  1    GGAGCAGGATCCGGAATATTAGGATTAAGAATAAGAATATTAATCCGCATAGAATTAGGT  60

Query  80   CAACCAGGAACTTTAATTGGAAACGATCAAATTTATAATGTAATTGTAACAGCACATGCA  139
            ||||||||||||||||||||||||||||||||||||||||||||||||||||||||||||
Sbjct  61   CAACCAGGAACTTTAATTGGAAACGATCAAATTTATAATGTAATTGTAACAGCACATGCA  120

Query  140  TTTATTATAAttttttttATAGTTATACCAATTATAATCGGAGGATTTGGAAATTGATTA  199
            |||||||||||||||||||||||||||||||||||||||||||||||||||||| |||||
Sbjct  121  TTTATTATAATTTTTTTTATAGTTATACCAATTATAATCGGAGGATTTGGAAATGGATTA  180

Query  200  GTTCCTATTATACTAGGAGCTCCAGATATAGCATTTCCACGAATAAATAATATAAGATTT  259
            ||||||||||| ||||||||||||||||||||||||||||||||||||||||||||||||
Sbjct  181  GTTCCTATTATGCTAGGAGCTCCAGATATAGCATTTCCACGAATAAATAATATAAGATTT  240

Query  260  TGGTTACTTCCTCCTTCTCTATTTCTACTAATTAATTCTTCATTAATCGAATCAGGAGCA  319
            ||||||||||||||||||||||||||||||||||||||||||||||||||||||||||||
Sbjct  241  TGGTTACTTCCTCCTTCTCTATTTCTACTAATTAATTCTTCATTAATCGAATCAGGAGCA  300

Query  320  GGTACAGGGTGAACAGTTAcccccccTTATCATCAAATTTATCACACTACGGACCATCA  379
            ||||||||| ||||||||||||||||||||||||||||||||||||||||||| ||||||
Sbjct  301  GGTACAGGGGGAACAGTTACCCCCCCTTATCATCAAATTTATCACACTACGGGCCATCA  360

Query  380  GTAGATTTAGCTATTTTTTCTCTGCATCTTGCAGGTGCTTCTTCAATTTTAGGTGCAATC  439
            |||||||||||||| ||||||||||||||||| |||||||||||||||||||||||||||
Sbjct  361  GTAGATTTAGCTATCTTTTCTCTGCATCTTGCCGGTGCTTCTTCAATTTTAGGTGCAATC  420

Query  440  AATTTCATTACAACTATTGTAAATATACGATCCATTGGAATAACAATAGAACGAATACCA  499
            ||||||||||||||||||||||||||||||||||||||||||||||||||||||||||||
Sbjct  421  AATTTCATTACAACTATTGTAAATATACGATCCATTGGAATAACAATAGAACGAATACCA  480

Query  500  TTATTTGTATGATCTGTTTTAATTACTGCAATTTTATTATTATTATCTTTACCAGTCTTA  559
            ||||||||| ||||||||||||||||||||||||||||||||||||||||||||||||||
Sbjct  481  TTATTTGTAGGATCTGTTTTAATTACTGCAATTTTATTATTATTATCTTTACCAGTCTTA  540
```

```
Query  560   GCTGGCGCAATTACAATATTATTAACTGATCGAAACTTTAATACATCATTCTTTGACCCT  619
             ||||| ||||||||||||||||||||||||||||||||||||||||||||||||||||||
Sbjct  541   GCTGGTGCAATTACAATATTATTAACTGATCGAAACTTTAATACATCATTCTTTGACCCT  600

Query  620   TCAGGAGGAGGTGACCCAATTCTATATCAACATTTATTT  658
             |||||||| ||||||||||||||||||||||||||||||
Sbjct  601   TCAGGAGGGGGTGACCCAATTCTATATCAACATTTATTT  639
```

>Rhipicephalus linnaei isolate C2-T1 cytochrome c oxidase subunit I (COX1) gene, partial cds; mitochondrial
Sequence ID: MZ726445.1 Length: 604
Range 1: 1 to 604

Score:1116 bits(604), Expect:0.0,
Identities:604/604(100%), Gaps:0/604(0%), Strand: Plus/Plus

```
Query  1     GACAATATACTTAATTTTTGGAGCATGATCCGGAATACTAGGATTAAGAATAAGAATATT  60
             ||||||||||||||||||||||||||||||||||||||||||||||||||||||||||||
Sbjct  1     GACAATATACTTAATTTTTGGAGCATGATCCGGAATACTAGGATTAAGAATAAGAATATT  60

Query  61    AATCCGCATAGAATTAGGTCAACCAGGAACTTTAATTGGAAACGATCAAATTTATAATGT  120
             ||||||||||||||||||||||||||||||||||||||||||||||||||||||||||||
Sbjct  61    AATCCGCATAGAATTAGGTCAACCAGGAACTTTAATTGGAAACGATCAAATTTATAATGT  120

Query  121   AATTGTAACAGCACATGCATTTATTATAAtttttttATAGTTATACCAATTATAATCGG  180
             ||||||||||||||||||||||||||||||||||||||||||||||||||||||||||||
Sbjct  121   AATTGTAACAGCACATGCATTTATTATAATTTTTTTATAGTTATACCAATTATAATCGG  180

Query  181   AGGATTTGGAAATTGATTAGTTCCTATTATACTAGGAGCTCCAGATATAGCATTTCCACG  240
             ||||||||||||||||||||||||||||||||||||||||||||||||||||||||||||
Sbjct  181   AGGATTTGGAAATTGATTAGTTCCTATTATACTAGGAGCTCCAGATATAGCATTTCCACG  240

Query  241   AATAAATAATATAAGATTTTGGTTACTTCCTCCTTCTCTATTTCTACTAATTAATTCTTC  300
             ||||||||||||||||||||||||||||||||||||||||||||||||||||||||||||
Sbjct  241   AATAAATAATATAAGATTTTGGTTACTTCCTCCTTCTCTATTTCTACTAATTAATTCTTC  300

Query  301   ATTAATCGAATCAGGAGCAGGTACAGGGTGAACAGTTTAcccccccTTATCATCAAATTT  360
             ||||||||||||||||||||||||||||||||||||||||||||||||||||||||||||
Sbjct  301   ATTAATCGAATCAGGAGCAGGTACAGGGTGAACAGTTTACCCCCCCTTATCATCAAATTT  360

Query  361   ATCACACTACGGACCATCAGTAGATTTAGCTATTTTTTCTCTGCATCTTGCAGGTGCTTC  420
             ||||||||||||||||||||||||||||||||||||||||||||||||||||||||||||
Sbjct  361   ATCACACTACGGACCATCAGTAGATTTAGCTATTTTTTCTCTGCATCTTGCAGGTGCTTC  420

Query  421   TTCAATTTTAGGTGCAATCAATTTCATTACAACTATTGTAAATATACGATCCATTGGAAT  480
             ||||||||||||||||||||||||||||||||||||||||||||||||||||||||||||
Sbjct  421   TTCAATTTTAGGTGCAATCAATTTCATTACAACTATTGTAAATATACGATCCATTGGAAT  480

Query  481   AACAATAGAACGAATACCATTATTTGTATGATCTGTTTTAATTACTGCAATTTTATTATT  540
             ||||||||||||||||||||||||||||||||||||||||||||||||||||||||||||
```

```
Sbjct  481  AACAATAGAACGAATACCATTATTTGTATGATCTGTTTTAATTACTGCAATTTTATTATT  540

Query  541  ATTATCTTTACCAGTCTTAGCTGGCGCAATTACAATATTATTAACTGATCGAAACTTTAA  600
            ||||||||||||||||||||||||||||||||||||||||||||||||||||||||||||
Sbjct  541  ATTATCTTTACCAGTCTTAGCTGGCGCAATTACAATATTATTAACTGATCGAAACTTTAA  600

Query  601  TACA  604
            ||||
Sbjct  601  TACA  604
```

>Rhipicephalus linnaei isolate C1-T1 cytochrome c oxidase subunit I (COX1) gene, partial cds; mitochondrial
Sequence ID: MZ726444.1 Length: 604
Range 1: 1 to 604

Score:1116 bits(604), Expect:0.0,
Identities:604/604(100%), Gaps:0/604(0%), Strand: Plus/Plus

```
Query  1    GACAATATACTTAATTTTTGGAGCATGATCCGGAATACTAGGATTAAGAATAAGAATATT  60
            ||||||||||||||||||||||||||||||||||||||||||||||||||||||||||||
Sbjct  1    GACAATATACTTAATTTTTGGAGCATGATCCGGAATACTAGGATTAAGAATAAGAATATT  60

Query  61   AATCCGCATAGAATTAGGTCAACCAGGAACTTTAATTGGAAACGATCAAATTTATAATGT  120
            ||||||||||||||||||||||||||||||||||||||||||||||||||||||||||||
Sbjct  61   AATCCGCATAGAATTAGGTCAACCAGGAACTTTAATTGGAAACGATCAAATTTATAATGT  120

Query  121  AATTGTAACAGCACATGCATTTATTATAAtttttttATAGTTATACCAATTATAATCGG  180
            ||||||||||||||||||||||||||||||||||||||||||||||||||||||||||||
Sbjct  121  AATTGTAACAGCACATGCATTTATTATAATTTTTTTATAGTTATACCAATTATAATCGG  180

Query  181  AGGATTTGGAAATTGATTAGTTCCTATTATACTAGGAGCTCCAGATATAGCATTTCCACG  240
            ||||||||||||||||||||||||||||||||||||||||||||||||||||||||||||
Sbjct  181  AGGATTTGGAAATTGATTAGTTCCTATTATACTAGGAGCTCCAGATATAGCATTTCCACG  240

Query  241  AATAAATAATATAAGATTTTGGTTACTTCCTCCTTCTCTATTTCTACTAATTAATTCTTC  300
            ||||||||||||||||||||||||||||||||||||||||||||||||||||||||||||
Sbjct  241  AATAAATAATATAAGATTTTGGTTACTTCCTCCTTCTCTATTTCTACTAATTAATTCTTC  300

Query  301  ATTAATCGAATCAGGAGCAGGTACAGGGTGAACAGTTTAcccccccTTATCATCAAATTT  360
            ||||||||||||||||||||||||||||||||||||||||||||||||||||||||||||
Sbjct  301  ATTAATCGAATCAGGAGCAGGTACAGGGTGAACAGTTTACCCCCCCTTATCATCAAATTT  360

Query  361  ATCACACTACGGACCATCAGTAGATTTAGCTATTTTTTCTCTGCATCTTGCAGGTGCTTC  420
            ||||||||||||||||||||||||||||||||||||||||||||||||||||||||||||
Sbjct  361  ATCACACTACGGACCATCAGTAGATTTAGCTATTTTTTCTCTGCATCTTGCAGGTGCTTC  420

Query  421  TTCAATTTTAGGTGCAATCAATTTCATTACAACTATTGTAAATATACGATCCATTGGAAT  480
            ||||||||||||||||||||||||||||||||||||||||||||||||||||||||||||
Sbjct  421  TTCAATTTTAGGTGCAATCAATTTCATTACAACTATTGTAAATATACGATCCATTGGAAT  480

Query  481  AACAATAGAACGAATACCATTATTTGTATGATCTGTTTTAATTACTGCAATTTTATTATT  540
```

```
          ||||||||||||||||||||||||||||||||||||||||||||||||||||||||||||
Sbjct  481  AACAATAGAACGAATACCATTATTTGTATGATCTGTTTTAATTACTGCAATTTTATTATT  540

Query  541  ATTATCTTTACCAGTCTTAGCTGGCGCAATTACAATATTATTAACTGATCGAAACTTTAA  600
            ||||||||||||||||||||||||||||||||||||||||||||||||||||||||||||
Sbjct  541  ATTATCTTTACCAGTCTTAGCTGGCGCAATTACAATATTATTAACTGATCGAAACTTTAA  600

Query  601  TACA  604
            ||||
Sbjct  601  TACA  604
```

>Rhipicephalus linnaei isolate B15-T1 cytochrome c oxidase subunit I (COX1) gene, partial cds; mitochondrial
Sequence ID: MZ726443.1 Length: 604
Range 1: 1 to 604

Score:1116 bits(604), Expect:0.0,
Identities:604/604(100%), Gaps:0/604(0%), Strand: Plus/Plus

```
Query  1    GACAATATACTTAATTTTTGGAGCATGATCCGGAATACTAGGATTAAGAATAAGAATATT  60
            ||||||||||||||||||||||||||||||||||||||||||||||||||||||||||||
Sbjct  1    GACAATATACTTAATTTTTGGAGCATGATCCGGAATACTAGGATTAAGAATAAGAATATT  60

Query  61   AATCCGCATAGAATTAGGTCAACCAGGAACTTTAATTGGAAACGATCAAATTTATAATGT  120
            ||||||||||||||||||||||||||||||||||||||||||||||||||||||||||||
Sbjct  61   AATCCGCATAGAATTAGGTCAACCAGGAACTTTAATTGGAAACGATCAAATTTATAATGT  120

Query  121  AATTGTAACAGCACATGCATTTATTATAAttttttttATAGTTATACCAATTATAATCGG  180
            ||||||||||||||||||||||||||||||||||||||||||||||||||||||||||||
Sbjct  121  AATTGTAACAGCACATGCATTTATTATAATTTTTTTTATAGTTATACCAATTATAATCGG  180

Query  181  AGGATTTGGAAATTGATTAGTTCCTATTATACTAGGAGCTCCAGATATAGCATTTCCACG  240
            ||||||||||||||||||||||||||||||||||||||||||||||||||||||||||||
Sbjct  181  AGGATTTGGAAATTGATTAGTTCCTATTATACTAGGAGCTCCAGATATAGCATTTCCACG  240

Query  241  AATAAATAATATAAGATTTTGGTTACTTCCTCCTTCTCTATTTCTACTAATTAATTCTTC  300
            ||||||||||||||||||||||||||||||||||||||||||||||||||||||||||||
Sbjct  241  AATAAATAATATAAGATTTTGGTTACTTCCTCCTTCTCTATTTCTACTAATTAATTCTTC  300

Query  301  ATTAATCGAATCAGGAGCAGGTACAGGGTGAACAGTTTAcccccccTTATCATCAAATTT  360
            ||||||||||||||||||||||||||||||||||||||||||||||||||||||||||||
Sbjct  301  ATTAATCGAATCAGGAGCAGGTACAGGGTGAACAGTTTACCCCCCCTTATCATCAAATTT  360

Query  361  ATCACACTACGGACCATCAGTAGATTTAGCTATTTTTTCTCTGCATCTTGCAGGTGCTTC  420
            ||||||||||||||||||||||||||||||||||||||||||||||||||||||||||||
Sbjct  361  ATCACACTACGGACCATCAGTAGATTTAGCTATTTTTTCTCTGCATCTTGCAGGTGCTTC  420

Query  421  TTCAATTTTAGGTGCAATCAATTTCATTACAACTATTGTAAATATACGATCCATTGGAAT  480
            ||||||||||||||||||||||||||||||||||||||||||||||||||||||||||||
Sbjct  421  TTCAATTTTAGGTGCAATCAATTTCATTACAACTATTGTAAATATACGATCCATTGGAAT  480
```

```
Query  481   AACAATAGAACGAATACCATTATTTGTATGATCTGTTTTAATTACTGCAATTTTATTATT  540
             ||||||||||||||||||||||||||||||||||||||||||||||||||||||||||||
Sbjct  481   AACAATAGAACGAATACCATTATTTGTATGATCTGTTTTAATTACTGCAATTTTATTATT  540

Query  541   ATTATCTTTACCAGTCTTAGCTGGCGCAATTACAATATTATTAACTGATCGAAACTTTAA  600
             ||||||||||||||||||||||||||||||||||||||||||||||||||||||||||||
Sbjct  541   ATTATCTTTACCAGTCTTAGCTGGCGCAATTACAATATTATTAACTGATCGAAACTTTAA  600

Query  601   TACA  604
             ||||
Sbjct  601   TACA  604
```

>Rhipicephalus linnaei isolate B12-T1 cytochrome c oxidase subunit I (COX1) gene, partial cds; mitochondrial
Sequence ID: MZ726442.1 Length: 604
Range 1: 1 to 604

Score:1116 bits(604), Expect:0.0,
Identities:604/604(100%), Gaps:0/604(0%), Strand: Plus/Plus

```
Query  1     GACAATATACTTAATTTTTGGAGCATGATCCGGAATACTAGGATTAAGAATAAGAATATT  60
             ||||||||||||||||||||||||||||||||||||||||||||||||||||||||||||
Sbjct  1     GACAATATACTTAATTTTTGGAGCATGATCCGGAATACTAGGATTAAGAATAAGAATATT  60

Query  61    AATCCGCATAGAATTAGGTCAACCAGGAACTTTAATTGGAAACGATCAAATTTATAATGT  120
             ||||||||||||||||||||||||||||||||||||||||||||||||||||||||||||
Sbjct  61    AATCCGCATAGAATTAGGTCAACCAGGAACTTTAATTGGAAACGATCAAATTTATAATGT  120

Query  121   AATTGTAACAGCACATGCATTTATTATAAttttttttATAGTTATACCAATTATAATCGG  180
             ||||||||||||||||||||||||||||||||||||||||||||||||||||||||||||
Sbjct  121   AATTGTAACAGCACATGCATTTATTATAATTTTTTTTATAGTTATACCAATTATAATCGG  180

Query  181   AGGATTTGGAAATTGATTAGTTCCTATTATACTAGGAGCTCCAGATATAGCATTTCCACG  240
             ||||||||||||||||||||||||||||||||||||||||||||||||||||||||||||
Sbjct  181   AGGATTTGGAAATTGATTAGTTCCTATTATACTAGGAGCTCCAGATATAGCATTTCCACG  240

Query  241   AATAAATAATATAAGATTTTGGTTACTTCCTCCTTCTCTATTTCTACTAATTAATTCTTC  300
             ||||||||||||||||||||||||||||||||||||||||||||||||||||||||||||
Sbjct  241   AATAAATAATATAAGATTTTGGTTACTTCCTCCTTCTCTATTTCTACTAATTAATTCTTC  300

Query  301   ATTAATCGAATCAGGAGCAGGTACAGGGTGAACAGTTTAcccccccTTATCATCAAATTT  360
             ||||||||||||||||||||||||||||||||||||||||||||||||||||||||||||
Sbjct  301   ATTAATCGAATCAGGAGCAGGTACAGGGTGAACAGTTTACCCCCCCTTATCATCAAATTT  360

Query  361   ATCACACTACGGACCATCAGTAGATTTAGCTATTTTTTCTCTGCATCTTGCAGGTGCTTC  420
             ||||||||||||||||||||||||||||||||||||||||||||||||||||||||||||
Sbjct  361   ATCACACTACGGACCATCAGTAGATTTAGCTATTTTTTCTCTGCATCTTGCAGGTGCTTC  420

Query  421   TTCAATTTTAGGTGCAATCAATTTCATTACAACTATTGTAAATATACGATCCATTGGAAT  480
             ||||||||||||||||||||||||||||||||||||||||||||||||||||||||||||
Sbjct  421   TTCAATTTTAGGTGCAATCAATTTCATTACAACTATTGTAAATATACGATCCATTGGAAT  480
```

```
Query  481   AACAATAGAACGAATACCATTATTTGTATGATCTGTTTTAATTACTGCAATTTTATTATT  540
             ||||||||||||||||||||||||||||||||||||||||||||||||||||||||||||
Sbjct  481   AACAATAGAACGAATACCATTATTTGTATGATCTGTTTTAATTACTGCAATTTTATTATT  540

Query  541   ATTATCTTTACCAGTCTTAGCTGGCGCAATTACAATATTATTAACTGATCGAAACTTTAA  600
             ||||||||||||||||||||||||||||||||||||||||||||||||||||||||||||
Sbjct  541   ATTATCTTTACCAGTCTTAGCTGGCGCAATTACAATATTATTAACTGATCGAAACTTTAA  600

Query  601   TACA  604
             ||||
Sbjct  601   TACA  604
```

>Rhipicephalus linnaei isolate B8-T1 cytochrome c oxidase subunit I (COX1) gene, partial cds; mitochondrial
Sequence ID: MZ726441.1 Length: 604
Range 1: 1 to 604

Score:1116 bits(604), Expect:0.0,
Identities:604/604(100%), Gaps:0/604(0%), Strand: Plus/Plus

```
Query  1     GACAATATACTTAATTTTTGGAGCATGATCCGGAATACTAGGATTAAGAATAAGAATATT  60
             ||||||||||||||||||||||||||||||||||||||||||||||||||||||||||||
Sbjct  1     GACAATATACTTAATTTTTGGAGCATGATCCGGAATACTAGGATTAAGAATAAGAATATT  60

Query  61    AATCCGCATAGAATTAGGTCAACCAGGAACTTTAATTGGAAACGATCAAATTTATAATGT  120
             ||||||||||||||||||||||||||||||||||||||||||||||||||||||||||||
Sbjct  61    AATCCGCATAGAATTAGGTCAACCAGGAACTTTAATTGGAAACGATCAAATTTATAATGT  120

Query  121   AATTGTAACAGCACATGCATTTATTATAAttttttttATAGTTATACCAATTATAATCGG  180
             ||||||||||||||||||||||||||||||||||||||||||||||||||||||||||||
Sbjct  121   AATTGTAACAGCACATGCATTTATTATAATTTTTTTTATAGTTATACCAATTATAATCGG  180

Query  181   AGGATTTGGAAATTGATTAGTTCCTATTATACTAGGAGCTCCAGATATAGCATTTCCACG  240
             ||||||||||||||||||||||||||||||||||||||||||||||||||||||||||||
Sbjct  181   AGGATTTGGAAATTGATTAGTTCCTATTATACTAGGAGCTCCAGATATAGCATTTCCACG  240

Query  241   AATAAATAATATAAGATTTTGGTTACTTCCTCCTTCTCTATTTCTACTAATTAATTCTTC  300
             ||||||||||||||||||||||||||||||||||||||||||||||||||||||||||||
Sbjct  241   AATAAATAATATAAGATTTTGGTTACTTCCTCCTTCTCTATTTCTACTAATTAATTCTTC  300

Query  301   ATTAATCGAATCAGGAGCAGGTACAGGGTGAACAGTTTAcccccccTTATCATCAAATTT  360
             ||||||||||||||||||||||||||||||||||||||||||||||||||||||||||||
Sbjct  301   ATTAATCGAATCAGGAGCAGGTACAGGGTGAACAGTTTACCCCCCCTTATCATCAAATTT  360

Query  361   ATCACACTACGGACCATCAGTAGATTTAGCTATTTTTTCTCTGCATCTTGCAGGTGCTTC  420
             ||||||||||||||||||||||||||||||||||||||||||||||||||||||||||||
Sbjct  361   ATCACACTACGGACCATCAGTAGATTTAGCTATTTTTTCTCTGCATCTTGCAGGTGCTTC  420

Query  421   TTCAATTTTAGGTGCAATCAATTTCATTACAACTATTGTAAATATACGATCCATTGGAAT  480
             ||||||||||||||||||||||||||||||||||||||||||||||||||||||||||||
```

```
Sbjct  421  TTCAATTTTAGGTGCAATCAATTTCATTACAACTATTGTAAATATACGATCCATTGGAAT  480

Query  481  AACAATAGAACGAATACCATTATTTGTATGATCTGTTTTAATTACTGCAATTTTATTATT  540
            ||||||||||||||||||||||||||||||||||||||||||||||||||||||||||||
Sbjct  481  AACAATAGAACGAATACCATTATTTGTATGATCTGTTTTAATTACTGCAATTTTATTATT  540

Query  541  ATTATCTTTACCAGTCTTAGCTGGCGCAATTACAATATTATTAACTGATCGAAACTTTAA  600
            ||||||||||||||||||||||||||||||||||||||||||||||||||||||||||||
Sbjct  541  ATTATCTTTACCAGTCTTAGCTGGCGCAATTACAATATTATTAACTGATCGAAACTTTAA  600

Query  601  TACA  604
            ||||
Sbjct  601  TACA  604
```

>Rhipicephalus linnaei isolate B7-T3 cytochrome c oxidase subunit I (COX1) gene, partial cds; mitochondrial
Sequence ID: MZ726440.1 Length: 604
Range 1: 1 to 604

Score:1116 bits(604), Expect:0.0,
Identities:604/604(100%), Gaps:0/604(0%), Strand: Plus/Plus

```
Query  1    GACAATATACTTAATTTTTGGAGCATGATCCGGAATACTAGGATTAAGAATAAGAATATT  60
            ||||||||||||||||||||||||||||||||||||||||||||||||||||||||||||
Sbjct  1    GACAATATACTTAATTTTTGGAGCATGATCCGGAATACTAGGATTAAGAATAAGAATATT  60

Query  61   AATCCGCATAGAATTAGGTCAACCAGGAACTTTAATTGGAAACGATCAAATTTATAATGT  120
            ||||||||||||||||||||||||||||||||||||||||||||||||||||||||||||
Sbjct  61   AATCCGCATAGAATTAGGTCAACCAGGAACTTTAATTGGAAACGATCAAATTTATAATGT  120

Query  121  AATTGTAACAGCACATGCATTTATTATAAttttttttATAGTTATACCAATTATAATCGG  180
            ||||||||||||||||||||||||||||||||||||||||||||||||||||||||||||
Sbjct  121  AATTGTAACAGCACATGCATTTATTATAATTTTTTTTATAGTTATACCAATTATAATCGG  180

Query  181  AGGATTTGGAAATTGATTAGTTCCTATTATACTAGGAGCTCCAGATATAGCATTTCCACG  240
            ||||||||||||||||||||||||||||||||||||||||||||||||||||||||||||
Sbjct  181  AGGATTTGGAAATTGATTAGTTCCTATTATACTAGGAGCTCCAGATATAGCATTTCCACG  240

Query  241  AATAAATAATATAAGATTTTGGTTACTTCCTCCTTCTCTATTTCTACTAATTAATTCTTC  300
            ||||||||||||||||||||||||||||||||||||||||||||||||||||||||||||
Sbjct  241  AATAAATAATATAAGATTTTGGTTACTTCCTCCTTCTCTATTTCTACTAATTAATTCTTC  300

Query  301  ATTAATCGAATCAGGAGCAGGTACAGGGTGAACAGTTTAcccccccTTATCATCAAATTT  360
            ||||||||||||||||||||||||||||||||||||||||||||||||||||||||||||
Sbjct  301  ATTAATCGAATCAGGAGCAGGTACAGGGTGAACAGTTTACCCCCCCTTATCATCAAATTT  360

Query  361  ATCACACTACGGACCATCAGTAGATTTAGCTATTTTTTCTCTGCATCTTGCAGGTGCTTC  420
            ||||||||||||||||||||||||||||||||||||||||||||||||||||||||||||
Sbjct  361  ATCACACTACGGACCATCAGTAGATTTAGCTATTTTTTCTCTGCATCTTGCAGGTGCTTC  420

Query  421  TTCAATTTTAGGTGCAATCAATTTCATTACAACTATTGTAAATATACGATCCATTGGAAT  480
```

```
                ||||||||||||||||||||||||||||||||||||||||||||||||||||||||||||
Sbjct  421   TTCAATTTTAGGTGCAATCAATTTCATTACAACTATTGTAAATATACGATCCATTGGAAT  480

Query  481   AACAATAGAACGAATACCATTATTTGTATGATCTGTTTTAATTACTGCAATTTTATTATT  540
             ||||||||||||||||||||||||||||||||||||||||||||||||||||||||||||
Sbjct  481   AACAATAGAACGAATACCATTATTTGTATGATCTGTTTTAATTACTGCAATTTTATTATT  540

Query  541   ATTATCTTTACCAGTCTTAGCTGGCGCAATTACAATATTATTAACTGATCGAAACTTTAA  600
             ||||||||||||||||||||||||||||||||||||||||||||||||||||||||||||
Sbjct  541   ATTATCTTTACCAGTCTTAGCTGGCGCAATTACAATATTATTAACTGATCGAAACTTTAA  600

Query  601   TACA  604
             ||||
Sbjct  601   TACA  604
```

>Rhipicephalus linnaei isolate B7-T2 cytochrome c oxidase subunit I (COX1) gene, partial cds; mitochondrial
Sequence ID: MZ726439.1 Length: 604
Range 1: 1 to 604

Score:1116 bits(604), Expect:0.0,
Identities:604/604(100%), Gaps:0/604(0%), Strand: Plus/Plus

```
Query  1     GACAATATACTTAATTTTTGGAGCATGATCCGGAATACTAGGATTAAGAATAAGAATATT  60
             ||||||||||||||||||||||||||||||||||||||||||||||||||||||||||||
Sbjct  1     GACAATATACTTAATTTTTGGAGCATGATCCGGAATACTAGGATTAAGAATAAGAATATT  60

Query  61    AATCCGCATAGAATTAGGTCAACCAGGAACTTTAATTGGAAACGATCAAATTTATAATGT  120
             ||||||||||||||||||||||||||||||||||||||||||||||||||||||||||||
Sbjct  61    AATCCGCATAGAATTAGGTCAACCAGGAACTTTAATTGGAAACGATCAAATTTATAATGT  120

Query  121   AATTGTAACAGCACATGCATTTATTATAAtttttttATAGTTATACCAATTATAATCGG  180
             ||||||||||||||||||||||||||||||||||||||||||||||||||||||||||||
Sbjct  121   AATTGTAACAGCACATGCATTTATTATAATTTTTTTATAGTTATACCAATTATAATCGG  180

Query  181   AGGATTTGGAAATTGATTAGTTCCTATTATACTAGGAGCTCCAGATATAGCATTTCCACG  240
             ||||||||||||||||||||||||||||||||||||||||||||||||||||||||||||
Sbjct  181   AGGATTTGGAAATTGATTAGTTCCTATTATACTAGGAGCTCCAGATATAGCATTTCCACG  240

Query  241   AATAAATAATATAAGATTTTGGTTACTTCCTCCTTCTCTATTTCTACTAATTAATTCTTC  300
             ||||||||||||||||||||||||||||||||||||||||||||||||||||||||||||
Sbjct  241   AATAAATAATATAAGATTTTGGTTACTTCCTCCTTCTCTATTTCTACTAATTAATTCTTC  300

Query  301   ATTAATCGAATCAGGAGCAGGTACAGGGTGAACAGTTTAcccccccTTATCATCAAATTT  360
             ||||||||||||||||||||||||||||||||||||||||||||||||||||||||||||
Sbjct  301   ATTAATCGAATCAGGAGCAGGTACAGGGTGAACAGTTTACCCCCCCTTATCATCAAATTT  360

Query  361   ATCACACTACGGACCATCAGTAGATTTAGCTATTTTTTCTCTGCATCTTGCAGGTGCTTC  420
             ||||||||||||||||||||||||||||||||||||||||||||||||||||||||||||
Sbjct  361   ATCACACTACGGACCATCAGTAGATTTAGCTATTTTTTCTCTGCATCTTGCAGGTGCTTC  420
```

```
Query  421   TTCAATTTTAGGTGCAATCAATTTCATTACAACTATTGTAAATATACGATCCATTGGAAT  480
             ||||||||||||||||||||||||||||||||||||||||||||||||||||||||||||
Sbjct  421   TTCAATTTTAGGTGCAATCAATTTCATTACAACTATTGTAAATATACGATCCATTGGAAT  480

Query  481   AACAATAGAACGAATACCATTATTTGTATGATCTGTTTTAATTACTGCAATTTTATTATT  540
             ||||||||||||||||||||||||||||||||||||||||||||||||||||||||||||
Sbjct  481   AACAATAGAACGAATACCATTATTTGTATGATCTGTTTTAATTACTGCAATTTTATTATT  540

Query  541   ATTATCTTTACCAGTCTTAGCTGGCGCAATTACAATATTATTAACTGATCGAAACTTTAA  600
             ||||||||||||||||||||||||||||||||||||||||||||||||||||||||||||
Sbjct  541   ATTATCTTTACCAGTCTTAGCTGGCGCAATTACAATATTATTAACTGATCGAAACTTTAA  600

Query  601   TACA  604
             ||||
Sbjct  601   TACA  604
```

>Rhipicephalus linnaei isolate B7-T1 cytochrome c oxidase subunit I (COX1) gene, partial cds; mitochondrial
Sequence ID: MZ726438.1 Length: 604
Range 1: 1 to 604

Score:1116 bits(604), Expect:0.0,
Identities:604/604(100%), Gaps:0/604(0%), Strand: Plus/Plus

```
Query  1     GACAATATACTTAATTTTTGGAGCATGATCCGGAATACTAGGATTAAGAATAAGAATATT  60
             ||||||||||||||||||||||||||||||||||||||||||||||||||||||||||||
Sbjct  1     GACAATATACTTAATTTTTGGAGCATGATCCGGAATACTAGGATTAAGAATAAGAATATT  60

Query  61    AATCCGCATAGAATTAGGTCAACCAGGAACTTTAATTGGAAACGATCAAATTTATAATGT  120
             ||||||||||||||||||||||||||||||||||||||||||||||||||||||||||||
Sbjct  61    AATCCGCATAGAATTAGGTCAACCAGGAACTTTAATTGGAAACGATCAAATTTATAATGT  120

Query  121   AATTGTAACAGCACATGCATTTATTATAAtttttttATAGTTATACCAATTATAATCGG  180
             ||||||||||||||||||||||||||||||||||||||||||||||||||||||||||||
Sbjct  121   AATTGTAACAGCACATGCATTTATTATAATTTTTTTATAGTTATACCAATTATAATCGG  180

Query  181   AGGATTTGGAAATTGATTAGTTCCTATTATACTAGGAGCTCCAGATATAGCATTTCCACG  240
             ||||||||||||||||||||||||||||||||||||||||||||||||||||||||||||
Sbjct  181   AGGATTTGGAAATTGATTAGTTCCTATTATACTAGGAGCTCCAGATATAGCATTTCCACG  240

Query  241   AATAAATAATATAAGATTTTGGTTACTTCCTCCTTCTCTATTTCTACTAATTAATTCTTC  300
             ||||||||||||||||||||||||||||||||||||||||||||||||||||||||||||
Sbjct  241   AATAAATAATATAAGATTTTGGTTACTTCCTCCTTCTCTATTTCTACTAATTAATTCTTC  300

Query  301   ATTAATCGAATCAGGAGCAGGTACAGGGTGAACAGTTTAcccccccTTATCATCAAATTT  360
             ||||||||||||||||||||||||||||||||||||||||||||||||||||||||||||
Sbjct  301   ATTAATCGAATCAGGAGCAGGTACAGGGTGAACAGTTTACCCCCCCTTATCATCAAATTT  360

Query  361   ATCACACTACGGACCATCAGTAGATTTAGCTATTTTTTCTCTGCATCTTGCAGGTGCTTC  420
             ||||||||||||||||||||||||||||||||||||||||||||||||||||||||||||
Sbjct  361   ATCACACTACGGACCATCAGTAGATTTAGCTATTTTTTCTCTGCATCTTGCAGGTGCTTC  420
```

```
Query  421   TTCAATTTTAGGTGCAATCAATTTCATTACAACTATTGTAAATATACGATCCATTGGAAT  480
             ||||||||||||||||||||||||||||||||||||||||||||||||||||||||||||
Sbjct  421   TTCAATTTTAGGTGCAATCAATTTCATTACAACTATTGTAAATATACGATCCATTGGAAT  480

Query  481   AACAATAGAACGAATACCATTATTTGTATGATCTGTTTTAATTACTGCAATTTTATTATT  540
             ||||||||||||||||||||||||||||||||||||||||||||||||||||||||||||
Sbjct  481   AACAATAGAACGAATACCATTATTTGTATGATCTGTTTTAATTACTGCAATTTTATTATT  540

Query  541   ATTATCTTTACCAGTCTTAGCTGGCGCAATTACAATATTATTAACTGATCGAAACTTTAA  600
             ||||||||||||||||||||||||||||||||||||||||||||||||||||||||||||
Sbjct  541   ATTATCTTTACCAGTCTTAGCTGGCGCAATTACAATATTATTAACTGATCGAAACTTTAA  600

Query  601   TACA  604
             ||||
Sbjct  601   TACA  604
```

>Rhipicephalus linnaei isolate B6-T1 cytochrome c oxidase subunit I (COX1) gene, partial cds; mitochondrial
Sequence ID: MZ726437.1 Length: 604
Range 1: 1 to 604

Score:1116 bits(604), Expect:0.0,
Identities:604/604(100%), Gaps:0/604(0%), Strand: Plus/Plus

```
Query  1     GACAATATACTTAATTTTTGGAGCATGATCCGGAATACTAGGATTAAGAATAAGAATATT  60
             ||||||||||||||||||||||||||||||||||||||||||||||||||||||||||||
Sbjct  1     GACAATATACTTAATTTTTGGAGCATGATCCGGAATACTAGGATTAAGAATAAGAATATT  60

Query  61    AATCCGCATAGAATTAGGTCAACCAGGAACTTTAATTGGAAACGATCAAATTTATAATGT  120
             ||||||||||||||||||||||||||||||||||||||||||||||||||||||||||||
Sbjct  61    AATCCGCATAGAATTAGGTCAACCAGGAACTTTAATTGGAAACGATCAAATTTATAATGT  120

Query  121   AATTGTAACAGCACATGCATTTATTATAAttttttttATAGTTATACCAATTATAATCGG  180
             ||||||||||||||||||||||||||||||||||||||||||||||||||||||||||||
Sbjct  121   AATTGTAACAGCACATGCATTTATTATAATTTTTTTTATAGTTATACCAATTATAATCGG  180

Query  181   AGGATTTGGAAATTGATTAGTTCCTATTATACTAGGAGCTCCAGATATAGCATTTCCACG  240
             ||||||||||||||||||||||||||||||||||||||||||||||||||||||||||||
Sbjct  181   AGGATTTGGAAATTGATTAGTTCCTATTATACTAGGAGCTCCAGATATAGCATTTCCACG  240

Query  241   AATAAATAATATAAGATTTTGGTTACTTCCTCCTTCTCTATTTCTACTAATTAATTCTTC  300
             ||||||||||||||||||||||||||||||||||||||||||||||||||||||||||||
Sbjct  241   AATAAATAATATAAGATTTTGGTTACTTCCTCCTTCTCTATTTCTACTAATTAATTCTTC  300

Query  301   ATTAATCGAATCAGGAGCAGGTACAGGGTGAACAGTTTAcccccccTTATCATCAAATTT  360
             ||||||||||||||||||||||||||||||||||||||||||||||||||||||||||||
Sbjct  301   ATTAATCGAATCAGGAGCAGGTACAGGGTGAACAGTTTACCCCCCCTTATCATCAAATTT  360

Query  361   ATCACACTACGGACCATCAGTAGATTTAGCTATTTTTTCTCTGCATCTTGCAGGTGCTTC  420
             ||||||||||||||||||||||||||||||||||||||||||||||||||||||||||||
```

```
Sbjct  361   ATCACACTACGGACCATCAGTAGATTTAGCTATTTTTTCTCTGCATCTTGCAGGTGCTTC  420

Query  421   TTCAATTTTAGGTGCAATCAATTTCATTACAACTATTGTAAATATACGATCCATTGGAAT  480
             ||||||||||||||||||||||||||||||||||||||||||||||||||||||||||||
Sbjct  421   TTCAATTTTAGGTGCAATCAATTTCATTACAACTATTGTAAATATACGATCCATTGGAAT  480

Query  481   AACAATAGAACGAATACCATTATTTGTATGATCTGTTTTAATTACTGCAATTTTATTATT  540
             ||||||||||||||||||||||||||||||||||||||||||||||||||||||||||||
Sbjct  481   AACAATAGAACGAATACCATTATTTGTATGATCTGTTTTAATTACTGCAATTTTATTATT  540

Query  541   ATTATCTTTACCAGTCTTAGCTGGCGCAATTACAATATTATTAACTGATCGAAACTTTAA  600
             ||||||||||||||||||||||||||||||||||||||||||||||||||||||||||||
Sbjct  541   ATTATCTTTACCAGTCTTAGCTGGCGCAATTACAATATTATTAACTGATCGAAACTTTAA  600

Query  601   TACA  604
             ||||
Sbjct  601   TACA  604
```

>Rhipicephalus linnaei isolate B4-T1 cytochrome c oxidase subunit I (COX1) gene, partial cds; mitochondrial
Sequence ID: MZ726436.1 Length: 604
Range 1: 1 to 604

Score:1116 bits(604), Expect:0.0,
Identities:604/604(100%), Gaps:0/604(0%), Strand: Plus/Plus

```
Query  1     GACAATATACTTAATTTTTGGAGCATGATCCGGAATACTAGGATTAAGAATAAGAATATT  60
             ||||||||||||||||||||||||||||||||||||||||||||||||||||||||||||
Sbjct  1     GACAATATACTTAATTTTTGGAGCATGATCCGGAATACTAGGATTAAGAATAAGAATATT  60

Query  61    AATCCGCATAGAATTAGGTCAACCAGGAACTTTAATTGGAAACGATCAAATTTATAATGT  120
             ||||||||||||||||||||||||||||||||||||||||||||||||||||||||||||
Sbjct  61    AATCCGCATAGAATTAGGTCAACCAGGAACTTTAATTGGAAACGATCAAATTTATAATGT  120

Query  121   AATTGTAACAGCACATGCATTTATTATAAttttttttATAGTTATACCAATTATAATCGG  180
             ||||||||||||||||||||||||||||||||||||||||||||||||||||||||||||
Sbjct  121   AATTGTAACAGCACATGCATTTATTATAATTTTTTTTATAGTTATACCAATTATAATCGG  180

Query  181   AGGATTTGGAAATTGATTAGTTCCTATTATACTAGGAGCTCCAGATATAGCATTTCCACG  240
             ||||||||||||||||||||||||||||||||||||||||||||||||||||||||||||
Sbjct  181   AGGATTTGGAAATTGATTAGTTCCTATTATACTAGGAGCTCCAGATATAGCATTTCCACG  240

Query  241   AATAAATAATATAAGATTTTGGTTACTTCCTCCTTCTCTATTTCTACTAATTAATTCTTC  300
             ||||||||||||||||||||||||||||||||||||||||||||||||||||||||||||
Sbjct  241   AATAAATAATATAAGATTTTGGTTACTTCCTCCTTCTCTATTTCTACTAATTAATTCTTC  300

Query  301   ATTAATCGAATCAGGAGCAGGTACAGGGTGAACAGTTTAcccccccTTATCATCAAATTT  360
             ||||||||||||||||||||||||||||||||||||||||||||||||||||||||||||
Sbjct  301   ATTAATCGAATCAGGAGCAGGTACAGGGTGAACAGTTTACCCCCCCTTATCATCAAATTT  360

Query  361   ATCACACTACGGACCATCAGTAGATTTAGCTATTTTTTCTCTGCATCTTGCAGGTGCTTC  420
```

```
            ||||||||||||||||||||||||||||||||||||||||||||||||||||||||||||
Sbjct  361  ATCACACTACGGACCATCAGTAGATTTAGCTATTTTTTCTCTGCATCTTGCAGGTGCTTC  420

Query  421  TTCAATTTTAGGTGCAATCAATTTCATTACAACTATTGTAAATATACGATCCATTGGAAT  480
            ||||||||||||||||||||||||||||||||||||||||||||||||||||||||||||
Sbjct  421  TTCAATTTTAGGTGCAATCAATTTCATTACAACTATTGTAAATATACGATCCATTGGAAT  480

Query  481  AACAATAGAACGAATACCATTATTTGTATGATCTGTTTTAATTACTGCAATTTTATTATT  540
            ||||||||||||||||||||||||||||||||||||||||||||||||||||||||||||
Sbjct  481  AACAATAGAACGAATACCATTATTTGTATGATCTGTTTTAATTACTGCAATTTTATTATT  540

Query  541  ATTATCTTTACCAGTCTTAGCTGGCGCAATTACAATATTATTAACTGATCGAAACTTTAA  600
            ||||||||||||||||||||||||||||||||||||||||||||||||||||||||||||
Sbjct  541  ATTATCTTTACCAGTCTTAGCTGGCGCAATTACAATATTATTAACTGATCGAAACTTTAA  600

Query  601  TACA  604
            ||||
Sbjct  601  TACA  604
```

>Rhipicephalus linnaei isolate B3-T1 cytochrome c oxidase subunit I (COX1) gene, partial cds; mitochondrial
Sequence ID: MZ726435.1 Length: 604
Range 1: 1 to 604

Score:1116 bits(604), Expect:0.0,
Identities:604/604(100%), Gaps:0/604(0%), Strand: Plus/Plus

```
Query  1    GACAATATACTTAATTTTTGGAGCATGATCCGGAATACTAGGATTAAGAATAAGAATATT  60
            ||||||||||||||||||||||||||||||||||||||||||||||||||||||||||||
Sbjct  1    GACAATATACTTAATTTTTGGAGCATGATCCGGAATACTAGGATTAAGAATAAGAATATT  60

Query  61   AATCCGCATAGAATTAGGTCAACCAGGAACTTTAATTGGAAACGATCAAATTTATAATGT  120
            ||||||||||||||||||||||||||||||||||||||||||||||||||||||||||||
Sbjct  61   AATCCGCATAGAATTAGGTCAACCAGGAACTTTAATTGGAAACGATCAAATTTATAATGT  120

Query  121  AATTGTAACAGCACATGCATTTATTATAAttttttttATAGTTATACCAATTATAATCGG  180
            ||||||||||||||||||||||||||||||||||||||||||||||||||||||||||||
Sbjct  121  AATTGTAACAGCACATGCATTTATTATAATTTTTTTTATAGTTATACCAATTATAATCGG  180

Query  181  AGGATTTGGAAATTGATTAGTTCCTATTATACTAGGAGCTCCAGATATAGCATTTCCACG  240
            ||||||||||||||||||||||||||||||||||||||||||||||||||||||||||||
Sbjct  181  AGGATTTGGAAATTGATTAGTTCCTATTATACTAGGAGCTCCAGATATAGCATTTCCACG  240

Query  241  AATAAATAATATAAGATTTTGGTTACTTCCTCCTTCTCTATTTCTACTAATTAATTCTTC  300
            ||||||||||||||||||||||||||||||||||||||||||||||||||||||||||||
Sbjct  241  AATAAATAATATAAGATTTTGGTTACTTCCTCCTTCTCTATTTCTACTAATTAATTCTTC  300

Query  301  ATTAATCGAATCAGGAGCAGGTACAGGGTGAACAGTTTAcccccccTTATCATCAAATTT  360
            ||||||||||||||||||||||||||||||||||||||||||||||||||||||||||||
Sbjct  301  ATTAATCGAATCAGGAGCAGGTACAGGGTGAACAGTTTACCCCCCCTTATCATCAAATTT  360
```

```
Query  361   ATCACACTACGGACCATCAGTAGATTTAGCTATTTTTTCTCTGCATCTTGCAGGTGCTTC  420
             ||||||||||||||||||||||||||||||||||||||||||||||||||||||||||||
Sbjct  361   ATCACACTACGGACCATCAGTAGATTTAGCTATTTTTTCTCTGCATCTTGCAGGTGCTTC  420

Query  421   TTCAATTTTAGGTGCAATCAATTTCATTACAACTATTGTAAATATACGATCCATTGGAAT  480
             ||||||||||||||||||||||||||||||||||||||||||||||||||||||||||||
Sbjct  421   TTCAATTTTAGGTGCAATCAATTTCATTACAACTATTGTAAATATACGATCCATTGGAAT  480

Query  481   AACAATAGAACGAATACCATTATTTGTATGATCTGTTTTAATTACTGCAATTTTATTATT  540
             ||||||||||||||||||||||||||||||||||||||||||||||||||||||||||||
Sbjct  481   AACAATAGAACGAATACCATTATTTGTATGATCTGTTTTAATTACTGCAATTTTATTATT  540

Query  541   ATTATCTTTACCAGTCTTAGCTGGCGCAATTACAATATTATTAACTGATCGAAACTTTAA  600
             ||||||||||||||||||||||||||||||||||||||||||||||||||||||||||||
Sbjct  541   ATTATCTTTACCAGTCTTAGCTGGCGCAATTACAATATTATTAACTGATCGAAACTTTAA  600

Query  601   TACA  604
             ||||
Sbjct  601   TACA  604
```

>Rhipicephalus linnaei isolate B2-T1 cytochrome c oxidase subunit I (COX1) gene, partial cds; mitochondrial
Sequence ID: MZ726434.1 Length: 604
Range 1: 1 to 604

Score:1116 bits(604), Expect:0.0,
Identities:604/604(100%), Gaps:0/604(0%), Strand: Plus/Plus

```
Query  1     GACAATATACTTAATTTTTGGAGCATGATCCGGAATACTAGGATTAAGAATAAGAATATT  60
             ||||||||||||||||||||||||||||||||||||||||||||||||||||||||||||
Sbjct  1     GACAATATACTTAATTTTTGGAGCATGATCCGGAATACTAGGATTAAGAATAAGAATATT  60

Query  61    AATCCGCATAGAATTAGGTCAACCAGGAACTTTAATTGGAAACGATCAAATTTATAATGT  120
             ||||||||||||||||||||||||||||||||||||||||||||||||||||||||||||
Sbjct  61    AATCCGCATAGAATTAGGTCAACCAGGAACTTTAATTGGAAACGATCAAATTTATAATGT  120

Query  121   AATTGTAACAGCACATGCATTTATTATAAttttttttATAGTTATACCAATTATAATCGG  180
             ||||||||||||||||||||||||||||||||||||||||||||||||||||||||||||
Sbjct  121   AATTGTAACAGCACATGCATTTATTATAATTTTTTTTATAGTTATACCAATTATAATCGG  180

Query  181   AGGATTTGGAAATTGATTAGTTCCTATTATACTAGGAGCTCCAGATATAGCATTTCCACG  240
             ||||||||||||||||||||||||||||||||||||||||||||||||||||||||||||
Sbjct  181   AGGATTTGGAAATTGATTAGTTCCTATTATACTAGGAGCTCCAGATATAGCATTTCCACG  240

Query  241   AATAAATAATATAAGATTTTGGTTACTTCCTCCTTCTCTATTTCTACTAATTAATTCTTC  300
             ||||||||||||||||||||||||||||||||||||||||||||||||||||||||||||
Sbjct  241   AATAAATAATATAAGATTTTGGTTACTTCCTCCTTCTCTATTTCTACTAATTAATTCTTC  300

Query  301   ATTAATCGAATCAGGAGCAGGTACAGGGTGAACAGTTTAcccccccTTATCATCAAATTT  360
             ||||||||||||||||||||||||||||||||||||||||||||||||||||||||||||
Sbjct  301   ATTAATCGAATCAGGAGCAGGTACAGGGTGAACAGTTTACCCCCCCTTATCATCAAATTT  360
```

```
Query  361  ATCACACTACGGACCATCAGTAGATTTAGCTATTTTTTCTCTGCATCTTGCAGGTGCTTC  420
            ||||||||||||||||||||||||||||||||||||||||||||||||||||||||||||
Sbjct  361  ATCACACTACGGACCATCAGTAGATTTAGCTATTTTTTCTCTGCATCTTGCAGGTGCTTC  420

Query  421  TTCAATTTTAGGTGCAATCAATTTCATTACAACTATTGTAAATATACGATCCATTGGAAT  480
            ||||||||||||||||||||||||||||||||||||||||||||||||||||||||||||
Sbjct  421  TTCAATTTTAGGTGCAATCAATTTCATTACAACTATTGTAAATATACGATCCATTGGAAT  480

Query  481  AACAATAGAACGAATACCATTATTTGTATGATCTGTTTTAATTACTGCAATTTTATTATT  540
            ||||||||||||||||||||||||||||||||||||||||||||||||||||||||||||
Sbjct  481  AACAATAGAACGAATACCATTATTTGTATGATCTGTTTTAATTACTGCAATTTTATTATT  540

Query  541  ATTATCTTTACCAGTCTTAGCTGGCGCAATTACAATATTATTAACTGATCGAAACTTTAA  600
            ||||||||||||||||||||||||||||||||||||||||||||||||||||||||||||
Sbjct  541  ATTATCTTTACCAGTCTTAGCTGGCGCAATTACAATATTATTAACTGATCGAAACTTTAA  600

Query  601  TACA  604
            ||||
Sbjct  601  TACA  604
```

>Rhipicephalus linnaei isolate A7-T1 cytochrome c oxidase subunit I (COX1) gene, partial cds; mitochondrial
Sequence ID: MZ726433.1 Length: 604
Range 1: 1 to 604

Score:1116 bits(604), Expect:0.0,
Identities:604/604(100%), Gaps:0/604(0%), Strand: Plus/Plus

```
Query  1    GACAATATACTTAATTTTTGGAGCATGATCCGGAATACTAGGATTAAGAATAAGAATATT  60
            ||||||||||||||||||||||||||||||||||||||||||||||||||||||||||||
Sbjct  1    GACAATATACTTAATTTTTGGAGCATGATCCGGAATACTAGGATTAAGAATAAGAATATT  60

Query  61   AATCCGCATAGAATTAGGTCAACCAGGAACTTTAATTGGAAACGATCAAATTTATAATGT  120
            ||||||||||||||||||||||||||||||||||||||||||||||||||||||||||||
Sbjct  61   AATCCGCATAGAATTAGGTCAACCAGGAACTTTAATTGGAAACGATCAAATTTATAATGT  120

Query  121  AATTGTAACAGCACATGCATTTATTATAAttttttttATAGTTATACCAATTATAATCGG  180
            ||||||||||||||||||||||||||||||||||||||||||||||||||||||||||||
Sbjct  121  AATTGTAACAGCACATGCATTTATTATAATTTTTTTTATAGTTATACCAATTATAATCGG  180

Query  181  AGGATTTGGAAATTGATTAGTTCCTATTATACTAGGAGCTCCAGATATAGCATTTCCACG  240
            ||||||||||||||||||||||||||||||||||||||||||||||||||||||||||||
Sbjct  181  AGGATTTGGAAATTGATTAGTTCCTATTATACTAGGAGCTCCAGATATAGCATTTCCACG  240

Query  241  AATAAATAATATAAGATTTTGGTTACTTCCTCCTTCTCTATTTCTACTAATTAATTCTTC  300
            ||||||||||||||||||||||||||||||||||||||||||||||||||||||||||||
Sbjct  241  AATAAATAATATAAGATTTTGGTTACTTCCTCCTTCTCTATTTCTACTAATTAATTCTTC  300

Query  301  ATTAATCGAATCAGGAGCAGGTACAGGGTGAACAGTTTAcccccccTTATCATCAAATTT  360
            ||||||||||||||||||||||||||||||||||||||||||||||||||||||||||||
```

```
Sbjct  301   ATTAATCGAATCAGGAGCAGGTACAGGGTGAACAGTTTACCCCCCCTTATCATCAAATTT  360

Query  361   ATCACACTACGGACCATCAGTAGATTTAGCTATTTTTTCTCTGCATCTTGCAGGTGCTTC  420
             ||||||||||||||||||||||||||||||||||||||||||||||||||||||||||||
Sbjct  361   ATCACACTACGGACCATCAGTAGATTTAGCTATTTTTTCTCTGCATCTTGCAGGTGCTTC  420

Query  421   TTCAATTTTAGGTGCAATCAATTTCATTACAACTATTGTAAATATACGATCCATTGGAAT  480
             ||||||||||||||||||||||||||||||||||||||||||||||||||||||||||||
Sbjct  421   TTCAATTTTAGGTGCAATCAATTTCATTACAACTATTGTAAATATACGATCCATTGGAAT  480

Query  481   AACAATAGAACGAATACCATTATTTGTATGATCTGTTTTAATTACTGCAATTTTATTATT  540
             ||||||||||||||||||||||||||||||||||||||||||||||||||||||||||||
Sbjct  481   AACAATAGAACGAATACCATTATTTGTATGATCTGTTTTAATTACTGCAATTTTATTATT  540

Query  541   ATTATCTTTACCAGTCTTAGCTGGCGCAATTACAATATTATTAACTGATCGAAACTTTAA  600
             ||||||||||||||||||||||||||||||||||||||||||||||||||||||||||||
Sbjct  541   ATTATCTTTACCAGTCTTAGCTGGCGCAATTACAATATTATTAACTGATCGAAACTTTAA  600

Query  601   TACA  604
             ||||
Sbjct  601   TACA  604
```

>Rhipicephalus linnaei isolate A6-T1 cytochrome c oxidase subunit I (COX1) gene, partial cds; mitochondrial
Sequence ID: MZ726432.1 Length: 604
Range 1: 1 to 604

Score:1116 bits(604), Expect:0.0,
Identities:604/604(100%), Gaps:0/604(0%), Strand: Plus/Plus

```
Query  1     GACAATATACTTAATTTTTGGAGCATGATCCGGAATACTAGGATTAAGAATAAGAATATT  60
             ||||||||||||||||||||||||||||||||||||||||||||||||||||||||||||
Sbjct  1     GACAATATACTTAATTTTTGGAGCATGATCCGGAATACTAGGATTAAGAATAAGAATATT  60

Query  61    AATCCGCATAGAATTAGGTCAACCAGGAACTTTAATTGGAAACGATCAAATTTATAATGT  120
             ||||||||||||||||||||||||||||||||||||||||||||||||||||||||||||
Sbjct  61    AATCCGCATAGAATTAGGTCAACCAGGAACTTTAATTGGAAACGATCAAATTTATAATGT  120

Query  121   AATTGTAACAGCACATGCATTTATTATAAtttttttATAGTTATACCAATTATAATCGG  180
             ||||||||||||||||||||||||||||||||||||||||||||||||||||||||||||
Sbjct  121   AATTGTAACAGCACATGCATTTATTATAATTTTTTTATAGTTATACCAATTATAATCGG  180

Query  181   AGGATTTGGAAATTGATTAGTTCCTATTATACTAGGAGCTCCAGATATAGCATTTCCACG  240
             ||||||||||||||||||||||||||||||||||||||||||||||||||||||||||||
Sbjct  181   AGGATTTGGAAATTGATTAGTTCCTATTATACTAGGAGCTCCAGATATAGCATTTCCACG  240

Query  241   AATAAATAATATAAGATTTTGGTTACTTCCTCCTTCTCTATTTCTACTAATTAATTCTTC  300
             ||||||||||||||||||||||||||||||||||||||||||||||||||||||||||||
Sbjct  241   AATAAATAATATAAGATTTTGGTTACTTCCTCCTTCTCTATTTCTACTAATTAATTCTTC  300

Query  301   ATTAATCGAATCAGGAGCAGGTACAGGGTGAACAGTTTAcccccccTTATCATCAAATTT  360
```

```
           ||||||||||||||||||||||||||||||||||||||||||||||||||||||||||||
Sbjct  301  ATTAATCGAATCAGGAGCAGGTACAGGGTGAACAGTTTACCCCCCCTTATCATCAAATTT  360

Query  361  ATCACACTACGGACCATCAGTAGATTTAGCTATTTTTTCTCTGCATCTTGCAGGTGCTTC  420
            ||||||||||||||||||||||||||||||||||||||||||||||||||||||||||||
Sbjct  361  ATCACACTACGGACCATCAGTAGATTTAGCTATTTTTTCTCTGCATCTTGCAGGTGCTTC  420

Query  421  TTCAATTTTAGGTGCAATCAATTTCATTACAACTATTGTAAATATACGATCCATTGGAAT  480
            ||||||||||||||||||||||||||||||||||||||||||||||||||||||||||||
Sbjct  421  TTCAATTTTAGGTGCAATCAATTTCATTACAACTATTGTAAATATACGATCCATTGGAAT  480

Query  481  AACAATAGAACGAATACCATTATTTGTATGATCTGTTTTAATTACTGCAATTTTATTATT  540
            ||||||||||||||||||||||||||||||||||||||||||||||||||||||||||||
Sbjct  481  AACAATAGAACGAATACCATTATTTGTATGATCTGTTTTAATTACTGCAATTTTATTATT  540

Query  541  ATTATCTTTACCAGTCTTAGCTGGCGCAATTACAATATTATTAACTGATCGAAACTTTAA  600
            ||||||||||||||||||||||||||||||||||||||||||||||||||||||||||||
Sbjct  541  ATTATCTTTACCAGTCTTAGCTGGCGCAATTACAATATTATTAACTGATCGAAACTTTAA  600

Query  601  TACA  604
            ||||
Sbjct  601  TACA  604
```

>Rhipicephalus linnaei isolate A5-T1 cytochrome c oxidase subunit I (COX1) gene, partial cds; mitochondrial
Sequence ID: MZ726431.1 Length: 604
Range 1: 1 to 604

Score:1116 bits(604), Expect:0.0,
Identities:604/604(100%), Gaps:0/604(0%), Strand: Plus/Plus

```
Query  1    GACAATATACTTAATTTTTGGAGCATGATCCGGAATACTAGGATTAAGAATAAGAATATT  60
            ||||||||||||||||||||||||||||||||||||||||||||||||||||||||||||
Sbjct  1    GACAATATACTTAATTTTTGGAGCATGATCCGGAATACTAGGATTAAGAATAAGAATATT  60

Query  61   AATCCGCATAGAATTAGGTCAACCAGGAACTTTAATTGGAAACGATCAAATTTATAATGT  120
            ||||||||||||||||||||||||||||||||||||||||||||||||||||||||||||
Sbjct  61   AATCCGCATAGAATTAGGTCAACCAGGAACTTTAATTGGAAACGATCAAATTTATAATGT  120

Query  121  AATTGTAACAGCACATGCATTTATTATAAttttttttATAGTTATACCAATTATAATCGG  180
            ||||||||||||||||||||||||||||||||||||||||||||||||||||||||||||
Sbjct  121  AATTGTAACAGCACATGCATTTATTATAATTTTTTTTATAGTTATACCAATTATAATCGG  180

Query  181  AGGATTTGGAAATTGATTAGTTCCTATTATACTAGGAGCTCCAGATATAGCATTTCCACG  240
            ||||||||||||||||||||||||||||||||||||||||||||||||||||||||||||
Sbjct  181  AGGATTTGGAAATTGATTAGTTCCTATTATACTAGGAGCTCCAGATATAGCATTTCCACG  240

Query  241  AATAAATAATATAAGATTTTGGTTACTTCCTCCTTCTCTATTTCTACTAATTAATTCTTC  300
            ||||||||||||||||||||||||||||||||||||||||||||||||||||||||||||
Sbjct  241  AATAAATAATATAAGATTTTGGTTACTTCCTCCTTCTCTATTTCTACTAATTAATTCTTC  300
```

```
Query  301   ATTAATCGAATCAGGAGCAGGTACAGGGTGAACAGTTTAccccccTTATCATCAAATTT  360
             ||||||||||||||||||||||||||||||||||||||||||||||||||||||||||||
Sbjct  301   ATTAATCGAATCAGGAGCAGGTACAGGGTGAACAGTTTACCCCCCTTATCATCAAATTT  360

Query  361   ATCACACTACGGACCATCAGTAGATTTAGCTATTTTTTCTCTGCATCTTGCAGGTGCTTC  420
             ||||||||||||||||||||||||||||||||||||||||||||||||||||||||||||
Sbjct  361   ATCACACTACGGACCATCAGTAGATTTAGCTATTTTTTCTCTGCATCTTGCAGGTGCTTC  420

Query  421   TTCAATTTTAGGTGCAATCAATTTCATTACAACTATTGTAAATATACGATCCATTGGAAT  480
             ||||||||||||||||||||||||||||||||||||||||||||||||||||||||||||
Sbjct  421   TTCAATTTTAGGTGCAATCAATTTCATTACAACTATTGTAAATATACGATCCATTGGAAT  480

Query  481   AACAATAGAACGAATACCATTATTTGTATGATCTGTTTTAATTACTGCAATTTTATTATT  540
             ||||||||||||||||||||||||||||||||||||||||||||||||||||||||||||
Sbjct  481   AACAATAGAACGAATACCATTATTTGTATGATCTGTTTTAATTACTGCAATTTTATTATT  540

Query  541   ATTATCTTTACCAGTCTTAGCTGGCGCAATTACAATATTATTAACTGATCGAAACTTTAA  600
             ||||||||||||||||||||||||||||||||||||||||||||||||||||||||||||
Sbjct  541   ATTATCTTTACCAGTCTTAGCTGGCGCAATTACAATATTATTAACTGATCGAAACTTTAA  600

Query  601   TACA  604
             ||||
Sbjct  601   TACA  604
```

>Rhipicephalus linnaei isolate A2-T1 cytochrome c oxidase subunit I (COX1) gene, partial cds; mitochondrial
Sequence ID: MZ726430.1 Length: 604
Range 1: 1 to 604

Score:1116 bits(604), Expect:0.0,
Identities:604/604(100%), Gaps:0/604(0%), Strand: Plus/Plus

```
Query  1     GACAATATACTTAATTTTTGGAGCATGATCCGGAATACTAGGATTAAGAATAAGAATATT  60
             ||||||||||||||||||||||||||||||||||||||||||||||||||||||||||||
Sbjct  1     GACAATATACTTAATTTTTGGAGCATGATCCGGAATACTAGGATTAAGAATAAGAATATT  60

Query  61    AATCCGCATAGAATTAGGTCAACCAGGAACTTTAATTGGAAACGATCAAATTTATAATGT  120
             ||||||||||||||||||||||||||||||||||||||||||||||||||||||||||||
Sbjct  61    AATCCGCATAGAATTAGGTCAACCAGGAACTTTAATTGGAAACGATCAAATTTATAATGT  120

Query  121   AATTGTAACAGCACATGCATTTATTATAAttttttttATAGTTATACCAATTATAATCGG  180
             ||||||||||||||||||||||||||||||||||||||||||||||||||||||||||||
Sbjct  121   AATTGTAACAGCACATGCATTTATTATAATTTTTTTTATAGTTATACCAATTATAATCGG  180

Query  181   AGGATTTGGAAATTGATTAGTTCCTATTATACTAGGAGCTCCAGATATAGCATTTCCACG  240
             ||||||||||||||||||||||||||||||||||||||||||||||||||||||||||||
Sbjct  181   AGGATTTGGAAATTGATTAGTTCCTATTATACTAGGAGCTCCAGATATAGCATTTCCACG  240

Query  241   AATAAATAATATAAGATTTTGGTTACTTCCTCCTTCTCTATTTCTACTAATTAATTCTTC  300
             ||||||||||||||||||||||||||||||||||||||||||||||||||||||||||||
Sbjct  241   AATAAATAATATAAGATTTTGGTTACTTCCTCCTTCTCTATTTCTACTAATTAATTCTTC  300
```

```
Query  301  ATTAATCGAATCAGGAGCAGGTACAGGGTGAACAGTTTAcccccccTTATCATCAAATTT  360
            ||||||||||||||||||||||||||||||||||||||||||||||||||||||||||||
Sbjct  301  ATTAATCGAATCAGGAGCAGGTACAGGGTGAACAGTTTACCCCCCCTTATCATCAAATTT  360

Query  361  ATCACACTACGGACCATCAGTAGATTTAGCTATTTTTTCTCTGCATCTTGCAGGTGCTTC  420
            ||||||||||||||||||||||||||||||||||||||||||||||||||||||||||||
Sbjct  361  ATCACACTACGGACCATCAGTAGATTTAGCTATTTTTTCTCTGCATCTTGCAGGTGCTTC  420

Query  421  TTCAATTTTAGGTGCAATCAATTTCATTACAACTATTGTAAATATACGATCCATTGGAAT  480
            ||||||||||||||||||||||||||||||||||||||||||||||||||||||||||||
Sbjct  421  TTCAATTTTAGGTGCAATCAATTTCATTACAACTATTGTAAATATACGATCCATTGGAAT  480

Query  481  AACAATAGAACGAATACCATTATTTGTATGATCTGTTTTAATTACTGCAATTTTATTATT  540
            ||||||||||||||||||||||||||||||||||||||||||||||||||||||||||||
Sbjct  481  AACAATAGAACGAATACCATTATTTGTATGATCTGTTTTAATTACTGCAATTTTATTATT  540

Query  541  ATTATCTTTACCAGTCTTAGCTGGCGCAATTACAATATTATTAACTGATCGAAACTTTAA  600
            ||||||||||||||||||||||||||||||||||||||||||||||||||||||||||||
Sbjct  541  ATTATCTTTACCAGTCTTAGCTGGCGCAATTACAATATTATTAACTGATCGAAACTTTAA  600

Query  601  TACA  604
            ||||
Sbjct  601  TACA  604
```

```
>Rhipicephalus linnaei isolate B9-T1 cytochrome c oxidase subunit I (COX1) gene,
partial cds; mitochondrial
Sequence ID: MZ726449.1 Length: 604
Range 1: 1 to 604

Score:1110 bits(601), Expect:0.0,
Identities:603/604(99%),  Gaps:0/604(0%), Strand: Plus/Plus

Query  1    GACAATATACTTAATTTTTGGAGCATGATCCGGAATACTAGGATTAAGAATAAGAATATT  60
            ||||||||||||||||||||||||||||||||||||||||||||||||||||||||||||
Sbjct  1    GACAATATACTTAATTTTTGGAGCATGATCCGGAATACTAGGATTAAGAATAAGAATATT  60

Query  61   AATCCGCATAGAATTAGGTCAACCAGGAACTTTAATTGGAAACGATCAAATTTATAATGT  120
            ||||||||||||||||||||||||||||||||||||||||||||||||||||||||||||
Sbjct  61   AATCCGCATAGAATTAGGTCAACCAGGAACTTTAATTGGAAACGATCAAATTTATAATGT  120

Query  121  AATTGTAACAGCACATGCATTTATTATAAttttttttATAGTTATACCAATTATAATCGG  180
            ||||||||||||||||||||||||||||||||||||||||||||||||||||||||||||
Sbjct  121  AATTGTAACAGCACATGCATTTATTATAATTTTTTTTATAGTTATACCAATTATAATCGG  180

Query  181  AGGATTTGGAAATTGATTAGTTCCTATTATACTAGGAGCTCCAGATATAGCATTTCCACG  240
            ||||||||||||||||||||||||||||||||||||||||||||||||||||||||||||
Sbjct  181  AGGATTTGGAAATTGATTAGTTCCTATTATACTAGGAGCTCCAGATATAGCATTTCCACG  240

Query  241  AATAAATAATATAAGATTTTGGTTACTTCCTCCTTCTCTATTTCTACTAATTAATTCTTC  300
            ||||||||||||||||||||||||||||||||||||||||||||||||||||||||||||
```

```
Sbjct  241  AATAAATAATATAAGATTTTGGTTACTTCCTCCTTCTCTATTTCTACTAATTAATTCTTC  300

Query  301  ATTAATCGAATCAGGAGCAGGTACAGGGTGAACAGTTTAcccccccTTATCATCAAATTT  360
            ||||||||||||||||||||||| ||||||||||||||||||||||||||||||||||||
Sbjct  301  ATTAATCGAATCAGGAGCAGGTACGGGGTGAACAGTTTACCCCCCCTTATCATCAAATTT  360

Query  361  ATCACACTACGGACCATCAGTAGATTTAGCTATTTTTTCTCTGCATCTTGCAGGTGCTTC  420
            ||||||||||||||||||||||||||||||||||||||||||||||||||||||||||||
Sbjct  361  ATCACACTACGGACCATCAGTAGATTTAGCTATTTTTTCTCTGCATCTTGCAGGTGCTTC  420

Query  421  TTCAATTTTAGGTGCAATCAATTTCATTACAACTATTGTAAATATACGATCCATTGGAAT  480
            ||||||||||||||||||||||||||||||||||||||||||||||||||||||||||||
Sbjct  421  TTCAATTTTAGGTGCAATCAATTTCATTACAACTATTGTAAATATACGATCCATTGGAAT  480

Query  481  AACAATAGAACGAATACCATTATTTGTATGATCTGTTTTAATTACTGCAATTTTATTATT  540
            ||||||||||||||||||||||||||||||||||||||||||||||||||||||||||||
Sbjct  481  AACAATAGAACGAATACCATTATTTGTATGATCTGTTTTAATTACTGCAATTTTATTATT  540

Query  541  ATTATCTTTACCAGTCTTAGCTGGCGCAATTACAATATTATTAACTGATCGAAACTTTAA  600
            ||||||||||||||||||||||||||||||||||||||||||||||||||||||||||||
Sbjct  541  ATTATCTTTACCAGTCTTAGCTGGCGCAATTACAATATTATTAACTGATCGAAACTTTAA  600

Query  601  TACA  604
            ||||
Sbjct  601  TACA  604
```

>Rhipicephalus linnaei isolate B18-T1 cytochrome c oxidase subunit I (COX1) gene, partial cds; mitochondrial
Sequence ID: MZ726429.1 Length: 604
Range 1: 1 to 604

Score:1110 bits(601), Expect:0.0,
Identities:603/604(99%), Gaps:0/604(0%), Strand: Plus/Plus

```
Query  1    GACAATATACTTAATTTTTGGAGCATGATCCGGAATACTAGGATTAAGAATAAGAATATT  60
            ||||||||||||||||||||||||||||||||||||||||||||||||||||||||||||
Sbjct  1    GACAATATACTTAATTTTTGGAGCATGATCCGGAATACTAGGATTAAGAATAAGAATATT  60

Query  61   AATCCGCATAGAATTAGGTCAACCAGGAACTTTAATTGGAAACGATCAAATTTATAATGT  120
            ||||||||||||||||||||||||||||||||||||||||||||||||||||||||||||
Sbjct  61   AATCCGCATAGAATTAGGTCAACCAGGAACTTTAATTGGAAACGATCAAATTTATAATGT  120

Query  121  AATTGTAACAGCACATGCATTTATTATAAtttttttATAGTTATACCAATTATAATCGG  180
            ||||||||||||||||||||||||||||||||||||||||||||||||||||||||||||
Sbjct  121  AATTGTAACAGCACATGCATTTATTATAATTTTTTTATAGTTATACCAATTATAATCGG  180

Query  181  AGGATTTGGAAATTGATTAGTTCCTATTATACTAGGAGCTCCAGATATAGCATTTCCACG  240
            ||||||||||||||||||||||||||||||||||||||||||||||||||||||||||||
Sbjct  181  AGGATTTGGAAATTGATTAGTTCCTATTATACTAGGAGCTCCAGATATAGCATTTCCACG  240

Query  241  AATAAATAATATAAGATTTTGGTTACTTCCTCCTTCTCTATTTCTACTAATTAATTCTTC  300
```

```
                ||||||||||||||||||||||||||||||||||||||||||||||||||||||||||||
Sbjct  241  AATAAATAATATAAGATTTTGGTTACTTCCTCCTTCTCTATTTCTACTAATTAATTCTTC  300

Query  301  ATTAATCGAATCAGGAGCAGGTACAGGGTGAACAGTTTAcccccccTTATCATCAAATTT  360
            ||||||||||||||||||||||||||||||||||||||||||||||||||||||||||||
Sbjct  301  ATTAATCGAATCAGGAGCAGGTACAGGGTGAACAGTTTACCCCCCCTTATCATCAAATTT  360

Query  361  ATCACACTACGGACCATCAGTAGATTTAGCTATTTTTTCTCTGCATCTTGCAGGTGCTTC  420
            |||||||||||||||||||||||||||||||||||||||||||| ||||||||||||||||
Sbjct  361  ATCACACTACGGACCATCAGTAGATTTAGCTATTTTTTCTCTACATCTTGCAGGTGCTTC  420

Query  421  TTCAATTTTAGGTGCAATCAATTTCATTACAACTATTGTAAATATACGATCCATTGGAAT  480
            ||||||||||||||||||||||||||||||||||||||||||||||||||||||||||||
Sbjct  421  TTCAATTTTAGGTGCAATCAATTTCATTACAACTATTGTAAATATACGATCCATTGGAAT  480

Query  481  AACAATAGAACGAATACCATTATTTGTATGATCTGTTTTAATTACTGCAATTTTATTATT  540
            ||||||||||||||||||||||||||||||||||||||||||||||||||||||||||||
Sbjct  481  AACAATAGAACGAATACCATTATTTGTATGATCTGTTTTAATTACTGCAATTTTATTATT  540

Query  541  ATTATCTTTACCAGTCTTAGCTGGCGCAATTACAATATTATTAACTGATCGAAACTTTAA  600
            ||||||||||||||||||||||||||||||||||||||||||||||||||||||||||||
Sbjct  541  ATTATCTTTACCAGTCTTAGCTGGCGCAATTACAATATTATTAACTGATCGAAACTTTAA  600

Query  601  TACA  604
            ||||
Sbjct  601  TACA  604
```

>Rhipicephalus linnaei isolate B17-T1 cytochrome c oxidase subunit I (COX1) gene, partial cds; mitochondrial
Sequence ID: MZ726428.1 Length: 604
Range 1: 1 to 604

Score:1110 bits(601), Expect:0.0,
Identities:603/604(99%), Gaps:0/604(0%), Strand: Plus/Plus

```
Query  1    GACAATATACTTAATTTTTGGAGCATGATCCGGAATACTAGGATTAAGAATAAGAATATT  60
            ||||||||||||||||||||||||||||||||||||||||||||||||||||||||||||
Sbjct  1    GACAATATACTTAATTTTTGGAGCATGATCCGGAATACTAGGATTAAGAATAAGAATATT  60

Query  61   AATCCGCATAGAATTAGGTCAACCAGGAACTTTAATTGGAAACGATCAAATTTATAATGT  120
            ||||||||||||||||||||||||||||||||||||||||||||||||||||||||||||
Sbjct  61   AATCCGCATAGAATTAGGTCAACCAGGAACTTTAATTGGAAACGATCAAATTTATAATGT  120

Query  121  AATTGTAACAGCACATGCATTTATTATAAttttttttATAGTTATACCAATTATAATCGG  180
            ||||||||||||||||||||||||||||||||||||||||||||||||||||||||||||
Sbjct  121  AATTGTAACAGCACATGCATTTATTATAATTTTTTTTATAGTTATACCAATTATAATCGG  180

Query  181  AGGATTTGGAAATTGATTAGTTCCTATTATACTAGGAGCTCCAGATATAGCATTTCCACG  240
            ||||||||||||||||||||||||||||||||||||||||||||||||||||||||||||
Sbjct  181  AGGATTTGGAAATTGATTAGTTCCTATTATACTAGGAGCTCCAGATATAGCATTTCCACG  240
```

```
Query  241  AATAAATAATATAAGATTTTGGTTACTTCCTCCTTCTCTATTTCTACTAATTAATTCTTC  300
            ||||||||||||||||||||||||||||||||||||||||||||||||||||||||||||
Sbjct  241  AATAAATAATATAAGATTTTGGTTACTTCCTCCTTCTCTATTTCTACTAATTAATTCTTC  300

Query  301  ATTAATCGAATCAGGAGCAGGTACAGGGTGAACAGTTTAcccccccTTATCATCAAATTT  360
            ||||||||||||||||||||||||||||||||||||||||||||||||||||||||||||
Sbjct  301  ATTAATCGAATCAGGAGCAGGTACAGGGTGAACAGTTTACCCCCCCTTATCATCAAATTT  360

Query  361  ATCACACTACGGACCATCAGTAGATTTAGCTATTTTTCTCTGCATCTTGCAGGTGCTTC  420
            ||||||||||||||||||||||||||||||||||||||||| ||||||||||||||||||
Sbjct  361  ATCACACTACGGACCATCAGTAGATTTAGCTATTTTTCTCTACATCTTGCAGGTGCTTC  420

Query  421  TTCAATTTTAGGTGCAATCAATTTCATTACAACTATTGTAAATATACGATCCATTGGAAT  480
            ||||||||||||||||||||||||||||||||||||||||||||||||||||||||||||
Sbjct  421  TTCAATTTTAGGTGCAATCAATTTCATTACAACTATTGTAAATATACGATCCATTGGAAT  480

Query  481  AACAATAGAACGAATACCATTATTTGTATGATCTGTTTTAATTACTGCAATTTTATTATT  540
            ||||||||||||||||||||||||||||||||||||||||||||||||||||||||||||
Sbjct  481  AACAATAGAACGAATACCATTATTTGTATGATCTGTTTTAATTACTGCAATTTTATTATT  540

Query  541  ATTATCTTTACCAGTCTTAGCTGGCGCAATTACAATATTATTAACTGATCGAAACTTTAA  600
            ||||||||||||||||||||||||||||||||||||||||||||||||||||||||||||
Sbjct  541  ATTATCTTTACCAGTCTTAGCTGGCGCAATTACAATATTATTAACTGATCGAAACTTTAA  600

Query  601  TACA  604
            ||||
Sbjct  601  TACA  604
```

>Rhipicephalus linnaei isolate B16-T1 cytochrome c oxidase subunit I (COX1) gene, partial cds; mitochondrial
Sequence ID: MZ726427.1 Length: 604
Range 1: 1 to 604

Score:1110 bits(601), Expect:0.0,
Identities:603/604(99%), Gaps:0/604(0%), Strand: Plus/Plus

```
Query  1    GACAATATACTTAATTTTTGGAGCATGATCCGGAATACTAGGATTAAGAATAAGAATATT  60
            ||||||||||||||||||||||||||||||||||||||||||||||||||||||||||||
Sbjct  1    GACAATATACTTAATTTTTGGAGCATGATCCGGAATACTAGGATTAAGAATAAGAATATT  60

Query  61   AATCCGCATAGAATTAGGTCAACCAGGAACTTTAATTGGAAACGATCAAATTTATAATGT  120
            ||||||||||||||||||||||||||||||||||||||||||||||||||||||||||||
Sbjct  61   AATCCGCATAGAATTAGGTCAACCAGGAACTTTAATTGGAAACGATCAAATTTATAATGT  120

Query  121  AATTGTAACAGCACATGCATTTATTATAAttttttttATAGTTATACCAATTATAATCGG  180
            ||||||||||||||||||||||||||||||||||||||||||||||||||||||||||||
Sbjct  121  AATTGTAACAGCACATGCATTTATTATAATTTTTTTTATAGTTATACCAATTATAATCGG  180

Query  181  AGGATTTGGAAATTGATTAGTTCCTATTATACTAGGAGCTCCAGATATAGCATTTCCACG  240
            ||||||||||||||||||||||||||||||||||||||||||||||||||||||||||||
Sbjct  181  AGGATTTGGAAATTGATTAGTTCCTATTATACTAGGAGCTCCAGATATAGCATTTCCACG  240
```

```
Query  241   AATAAATAATATAAGATTTTGGTTACTTCCTCCTTCTCTATTTCTACTAATTAATTCTTC  300
             ||||||||||||||||||||||||||||||||||||||||||||||||||||||||||||
Sbjct  241   AATAAATAATATAAGATTTTGGTTACTTCCTCCTTCTCTATTTCTACTAATTAATTCTTC  300

Query  301   ATTAATCGAATCAGGAGCAGGTACAGGGTGAACAGTTTAcccccccTTATCATCAAATTT  360
             ||||||||||||||||||||||||||||||||||||||||||||||||||||||||||||
Sbjct  301   ATTAATCGAATCAGGAGCAGGTACAGGGTGAACAGTTTACCCCCCCTTATCATCAAATTT  360

Query  361   ATCACACTACGGACCATCAGTAGATTTAGCTATTTTTTCTCTGCATCTTGCAGGTGCTTC  420
             ||||||||||||||||||||||||||||||||||||||||||| ||||||||||||||||
Sbjct  361   ATCACACTACGGACCATCAGTAGATTTAGCTATTTTTTCTCTACATCTTGCAGGTGCTTC  420

Query  421   TTCAATTTTAGGTGCAATCAATTTCATTACAACTATTGTAAATATACGATCCATTGGAAT  480
             ||||||||||||||||||||||||||||||||||||||||||||||||||||||||||||
Sbjct  421   TTCAATTTTAGGTGCAATCAATTTCATTACAACTATTGTAAATATACGATCCATTGGAAT  480

Query  481   AACAATAGAACGAATACCATTATTTGTATGATCTGTTTTAATTACTGCAATTTTATTATT  540
             ||||||||||||||||||||||||||||||||||||||||||||||||||||||||||||
Sbjct  481   AACAATAGAACGAATACCATTATTTGTATGATCTGTTTTAATTACTGCAATTTTATTATT  540

Query  541   ATTATCTTTACCAGTCTTAGCTGGCGCAATTACAATATTATTAACTGATCGAAACTTTAA  600
             ||||||||||||||||||||||||||||||||||||||||||||||||||||||||||||
Sbjct  541   ATTATCTTTACCAGTCTTAGCTGGCGCAATTACAATATTATTAACTGATCGAAACTTTAA  600

Query  601   TACA  604
             ||||
Sbjct  601   TACA  604
```

>Rhipicephalus linnaei isolate B1-T1 cytochrome c oxidase subunit I (COX1) gene, partial cds; mitochondrial
Sequence ID: MZ726426.1 Length: 604
Range 1: 1 to 604

Score:1110 bits(601), Expect:0.0,
Identities:603/604(99%), Gaps:0/604(0%), Strand: Plus/Plus

```
Query  1     GACAATATACTTAATTTTTGGAGCATGATCCGGAATACTAGGATTAAGAATAAGAATATT  60
             ||||||||||||||||||||||||||||||||||||||||||||||||||||||||||||
Sbjct  1     GACAATATACTTAATTTTTGGAGCATGATCCGGAATACTAGGATTAAGAATAAGAATATT  60

Query  61    AATCCGCATAGAATTAGGTCAACCAGGAACTTTAATTGGAAACGATCAAATTTATAATGT  120
             ||||||||||||||||||||||||||||||||||||||||||||||||||||||||||||
Sbjct  61    AATCCGCATAGAATTAGGTCAACCAGGAACTTTAATTGGAAACGATCAAATTTATAATGT  120

Query  121   AATTGTAACAGCACATGCATTTATTATAAttttttttATAGTTATACCAATTATAATCGG  180
             ||||||||||||||||||||||||||||||||||||||||||||||||||||||||||||
Sbjct  121   AATTGTAACAGCACATGCATTTATTATAATTTTTTTTATAGTTATACCAATTATAATCGG  180

Query  181   AGGATTTGGAAATTGATTAGTTCCTATTATACTAGGAGCTCCAGATATAGCATTTCCACG  240
             ||||||||||||||||||||||||||||||||||||||||||||||||||||||||||||
```

```
Sbjct  181  AGGATTTGGAAATTGATTAGTTCCTATTATACTAGGAGCTCCAGATATAGCATTTCCACG  240

Query  241  AATAAATAATATAAGATTTTGGTTACTTCCTCCTTCTCTATTTCTACTAATTAATTCTTC  300
            ||||||||||||||||||||||||||||||||||||||||||||||||||||||||||||
Sbjct  241  AATAAATAATATAAGATTTTGGTTACTTCCTCCTTCTCTATTTCTACTAATTAATTCTTC  300

Query  301  ATTAATCGAATCAGGAGCAGGTACAGGGTGAACAGTTTAcccccccTTATCATCAAATTT  360
            ||||||||||||||||||||||||||||||||||||||||||||||||||||||||||||
Sbjct  301  ATTAATCGAATCAGGAGCAGGTACAGGGTGAACAGTTTACCCCCCCTTATCATCAAATTT  360

Query  361  ATCACACTACGGACCATCAGTAGATTTAGCTATTTTTTCTCTGCATCTTGCAGGTGCTTC  420
            |||||||||||||||||||||||||||||||||||||||||| |||||||||||||||||
Sbjct  361  ATCACACTACGGACCATCAGTAGATTTAGCTATTTTTTCTCTACATCTTGCAGGTGCTTC  420

Query  421  TTCAATTTTAGGTGCAATCAATTTCATTACAACTATTGTAAATATACGATCCATTGGAAT  480
            ||||||||||||||||||||||||||||||||||||||||||||||||||||||||||||
Sbjct  421  TTCAATTTTAGGTGCAATCAATTTCATTACAACTATTGTAAATATACGATCCATTGGAAT  480

Query  481  AACAATAGAACGAATACCATTATTTGTATGATCTGTTTTAATTACTGCAATTTTATTATT  540
            ||||||||||||||||||||||||||||||||||||||||||||||||||||||||||||
Sbjct  481  AACAATAGAACGAATACCATTATTTGTATGATCTGTTTTAATTACTGCAATTTTATTATT  540

Query  541  ATTATCTTTACCAGTCTTAGCTGGCGCAATTACAATATTATTAACTGATCGAAACTTTAA  600
            ||||||||||||||||||||||||||||||||||||||||||||||||||||||||||||
Sbjct  541  ATTATCTTTACCAGTCTTAGCTGGCGCAATTACAATATTATTAACTGATCGAAACTTTAA  600

Query  601  TACA  604
            ||||
Sbjct  601  TACA  604
```

>Rhipicephalus sanguineus voucher DO-306 cytochrome oxidase subunit 1 (COI) gene, partial cds; mitochondrial
Sequence ID: KX360338.1 Length: 638
Range 1: 1 to 601

Score:1092 bits(591), Expect:0.0,
Identities:597/601(99%), Gaps:0/601(0%), Strand: Plus/Plus

```
Query  20   GGAGCATGATCCGGAATACTAGGATTAAGAATAAGAATATTAATCCGCATAGAATTAGGT  79
            |||||||||||||||||| |||||||||||||||||||||||||||||||||||||||||
Sbjct  1    GGAGCATGATCCGGAATATTAGGATTAAGAATAAGAATATTAATCCGCATAGAATTAGGT  60

Query  80   CAACCAGGAACTTTAATTGGAAACGATCAAATTTATAATGTAATTGTAACAGCACATGCA  139
            ||||||||||||||||||||||||||||||||||||||||||||||||||||||||||||
Sbjct  61   CAACCAGGAACTTTAATTGGAAACGATCAAATTTATAATGTAATTGTAACAGCACATGCA  120

Query  140  TTTATTATAAttttttttATAGTTATACCAATTATAATCGGAGGATTTGGAAATTGATTA  199
            ||||||||||||||||||||||||||||||||||||||||||||||||||||||||||||
Sbjct  121  TTTATTATAATTTTTTTTATAGTTATACCAATTATAATCGGAGGATTTGGAAATTGATTA  180

Query  200  GTTCCTATTATACTAGGAGCTCCAGATATAGCATTTCCACGAATAAATAATATAAGATTT  259
```

```
                ||||||||||||||||| ||||||||||||||||||||||||||||||||||||||||||
Sbjct  181  GTTCCTATTATACTAGGGGCTCCAGATATAGCATTTCCACGAATAAATAATATAAGATTT  240

Query  260  TGGTTACTTCCTCCTTCTCTATTTCTACTAATTAATTCTTCATTAATCGAATCAGGAGCA  319
            ||||||||||||||||||||||||||||||||||||||||||||||||||||||||||||
Sbjct  241  TGGTTACTTCCTCCTTCTCTATTTCTACTAATTAATTCTTCATTAATCGAATCAGGAGCA  300

Query  320  GGTACAGGGTGAACAGTTTAcccccccTTATCATCAAATTTATCACACTACGGACCATCA  379
            || |||||||||||||||||||||||||||||||||||||||||||||||||||||||||
Sbjct  301  GGNACAGGGTGAACAGTTTACCCCCCCTTATCATCAAATTTATCACACTACGGACCATCA  360

Query  380  GTAGATTTAGCTATTTTTTCTCTGCATCTTGCAGGTGCTTCTTCAATTTTAGGTGCAATC  439
            ||||||||||||||||||||||||||||||||||||||||||||||||||||||||||||
Sbjct  361  GTAGATTTAGCTATTTTTTCTCTGCATCTTGCAGGTGCTTCTTCAATTTTAGGTGCAATC  420

Query  440  AATTTCATTACAACTATTGTAAATATACGATCCATTGGAATAACAATAGAACGAATACCA  499
            ||||||||||||||||||||||||||||||||||||||||||||||||||||||||||||
Sbjct  421  AATTTCATTACAACTATTGTAAATATACGATCCATTGGAATAACAATAGAACGAATACCA  480

Query  500  TTATTTGTATGATCTGTTTTAATTACTGCAATTTTATTATTATTATCTTTACCAGTCTTA  559
            |||||||||||||||||||||||||| |||||||||||||||||||||||||||||||||
Sbjct  481  TTATTTGTATGATCTGTTTTAATTACNGCAATTTTATTATTATTATCTTTACCAGTCTTA  540

Query  560  GCTGGCGCAATTACAATATTATTAACTGATCGAAACTTTAATACATCATTCTTTGACCCT  619
            ||||||||||||||||||||||||||||||||||||||||||||||||||||||||||||
Sbjct  541  GCTGGCGCAATTACAATATTATTAACTGATCGAAACTTTAATACATCATTCTTTGACCCT  600

Query  620  T  620
            |
Sbjct  601  T  601
```

Query #7: TKCJJ021-19|TKCJ012018B09|Rhipicephalus sanguineus|COI-5P Query ID: lcl|Query_26769 Length: 658

Sequences producing significant alignments:

```
                                                                  Scientific
 Common                     Max    Total Query   E   Per.   Acc.

Description                                                       Name
 Name            Taxid      Score  Score cover Value Ident  Len        Accession

Rhipicephalus sanguineus mitochondrial DNA, complete genome
Rhipicephalu... brown dog tick  34632      1216   1216  100%  0.0   100.00 14710
     AF081829.1
Rhipicephalus sanguineus isolate Zagreb-B cytochrome oxidase...
Rhipicephalu... brown dog tick  34632      1140   1140  94%   0.0   99.84  629
     KX757893.1
Rhipicephalus sanguineus isolate Italy-A cytochrome oxidase...
Rhipicephalu... brown dog tick  34632      1129   1129  94%   0.0   99.52  630
     KX757903.1
```

Rhipicephalus sanguineus isolate Al-Hosema-A cytochrome oxidas...
Rhipicephalu... brown dog tick 34632 1112 1112 94% 0.0 99.03 630
KX757909.1
Rhipicephalus sanguineus sensu lato 'temperate lineage' isolat...
Rhipicephalu... brown dog tick 34632 1072 1072 100% 0.0 96.05 692
MF426015.1
Rhipicephalus sanguineus sensu lato 'temperate lineage' isolat...
Rhipicephalu... brown dog tick 34632 1066 1066 100% 0.0 95.90 675
MF426018.1
Rhipicephalus sanguineus sensu lato 'temperate lineage' isolat...
Rhipicephalu... brown dog tick 34632 1066 1066 100% 0.0 95.90 680
MF426016.1
Rhipicephalus sanguineus sensu lato 'temperate lineage' isolat...
Rhipicephalu... brown dog tick 34632 1066 1066 100% 0.0 95.90 693
MF426013.1
Rhipicephalus sanguineus sensu lato 'temperate lineage' isolat...
Rhipicephalu... brown dog tick 34632 1066 1066 100% 0.0 95.90 679
MF426009.1
Rhipicephalus sanguineus sensu lato 'temperate lineage' isolat...
Rhipicephalu... brown dog tick 34632 1066 1066 100% 0.0 95.90 686
MF426007.1
Rhipicephalus sanguineus sensu lato 'temperate lineage' isolat...
Rhipicephalu... brown dog tick 34632 1061 1061 100% 0.0 95.74 697
MF426019.1
Rhipicephalus sanguineus sensu lato 'temperate lineage' isolat...
Rhipicephalu... brown dog tick 34632 1061 1061 100% 0.0 95.74 705
MF426006.1
Rhipicephalus sanguineus haplotype III cytochrome oxidase...
Rhipicephalu... brown dog tick 34632 1061 1061 100% 0.0 95.74 713
KU556745.1
Rhipicephalus sanguineus isolate Arizona-1 cytochrome c oxidas...
Rhipicephalu... brown dog tick 34632 1061 1061 100% 0.0 95.74 710
MN593344.1
Rhipicephalus sanguineus isolate Arizona-2 cytochrome c oxidas...
Rhipicephalu... brown dog tick 34632 1061 1061 100% 0.0 95.74 710
MN593343.1
Rhipicephalus sanguineus isolate 11N cytochrome oxidase subuni...
Rhipicephalu... brown dog tick 34632 1061 1061 100% 0.0 95.74 703
MN585197.1
Rhipicephalus sanguineus cytochrome c oxidase I (COI) gene,...
Rhipicephalu... brown dog tick 34632 1061 1061 100% 0.0 95.74 793
AF132839.1
Rhipicephalus sanguineus sensu lato 'temperate lineage' isolat...
Rhipicephalu... brown dog tick 34632 1055 1055 100% 0.0 95.59 680
MF426017.1
Rhipicephalus sanguineus sensu lato 'temperate lineage' isolat...
Rhipicephalu... brown dog tick 34632 1055 1055 100% 0.0 95.59 690
MF426008.1
Rhipicephalus sanguineus sensu lato 'temperate lineage' isolat...
Rhipicephalu... brown dog tick 34632 1055 1055 100% 0.0 95.59 684
MF426001.1
Rhipicephalus sanguineus sensu lato 'temperate lineage' isolat...
Rhipicephalu... brown dog tick 34632 1055 1055 100% 0.0 95.59 689
MF425998.1

Rhipicephalus sanguineus sensu lato 'temperate lineage' isolat...
Rhipicephalu... brown dog tick 34632 1055 1055 100% 0.0 95.59 695
MF425997.1
Rhipicephalus sanguineus haplotype IV cytochrome oxidase subun...
Rhipicephalu... brown dog tick 34632 1055 1113 100% 0.0 95.59 771
KU556746.1
Rhipicephalus sanguineus voucher CROBB813 cytochrome oxidase...
Rhipicephalu... brown dog tick 34632 1055 1055 100% 0.0 95.59 658
MZ305546.1
Rhipicephalus sanguineus voucher CROBB810 cytochrome oxidase...
Rhipicephalu... brown dog tick 34632 1055 1055 100% 0.0 95.59 658
MZ305545.1
Rhipicephalus sanguineus isolate 5MSK cytochrome c oxidase...
Rhipicephalu... brown dog tick 34632 1055 1055 100% 0.0 95.59 676
MW152145.1
Rhipicephalus sanguineus isolate 1MSK cytochrome c oxidase...
Rhipicephalu... brown dog tick 34632 1055 1055 100% 0.0 95.59 675
MW152144.1
Rhipicephalus sanguineus isolate RSN2SK cytochrome c oxidase...
Rhipicephalu... brown dog tick 34632 1055 1055 100% 0.0 95.59 676
MW152143.1
Rhipicephalus sanguineus isolate RSN11SK cytochrome c oxidase...
Rhipicephalu... brown dog tick 34632 1055 1055 100% 0.0 95.59 669
MW152142.1
Rhipicephalus sanguineus sensu lato 'temperate lineage' isolat...
Rhipicephalu... brown dog tick 34632 1050 1050 100% 0.0 95.44 684
MF426012.1
Rhipicephalus sanguineus haplotype I cytochrome oxidase subuni...
Rhipicephalu... brown dog tick 34632 1048 1048 98% 0.0 95.83 659
KU556743.1
Rhipicephalus sanguineus haplotype II cytochrome oxidase subun...
Rhipicephalu... brown dog tick 34632 1046 1046 100% 0.0 95.30 787
KU556744.1
Rhipicephalus sanguineus sensu lato 'temperate lineage' isolat...
Rhipicephalu... brown dog tick 34632 1038 1038 97% 0.0 95.80 812
MF426020.1
Rhipicephalus sanguineus sensu lato 'temperate lineage' isolat...
Rhipicephalu... brown dog tick 34632 1037 1037 98% 0.0 95.52 824
MF426010.1
Rhipicephalus sanguineus sensu lato 'temperate lineage' isolat...
Rhipicephalu... brown dog tick 34632 1031 1031 97% 0.0 95.64 809
MF426011.1
Rhipicephalus sanguineus sensu lato 'temperate lineage' isolat...
Rhipicephalu... brown dog tick 34632 1022 1022 94% 0.0 96.30 649
MF426014.1
Rhipicephalus sanguineus isolate LIC5424B cytochrome oxidase...
Rhipicephalu... brown dog tick 34632 1020 1020 97% 0.0 95.33 768
KX383818.1
Rhipicephalus sanguineus sensu lato 'temperate lineage' isolat...
Rhipicephalu... brown dog tick 34632 1016 1016 94% 0.0 96.14 635
MF426000.1
Rhipicephalus sanguineus sensu lato 'temperate lineage' isolat...
Rhipicephalu... brown dog tick 34632 1016 1016 97% 0.0 95.31 654
MF425988.1

Rhipicephalus sanguineus isolate LIC5533B cytochrome oxidase...
Rhipicephalu... brown dog tick 34632 1013 1013 96% 0.0 95.30 764
KX383811.1
Rhipicephalus sanguineus isolate LIC4736B cytochrome oxidase...
Rhipicephalu... brown dog tick 34632 1011 1011 96% 0.0 95.29 763
KX383807.1
Rhipicephalus sanguineus voucher Rs7 cytochrome oxidase subuni...
Rhipicephalu... brown dog tick 34632 1009 1009 95% 0.0 95.56 656
KU568516.1
Rhipicephalus sanguineus isolate LIC4736D cytochrome oxidase...
Rhipicephalu... brown dog tick 34632 1009 1009 96% 0.0 95.28 762
KX383809.1
Rhipicephalus sanguineus isolate LIC4727D cytochrome oxidase...
Rhipicephalu... brown dog tick 34632 1009 1009 96% 0.0 95.28 714
KX383806.1
Rhipicephalus sanguineus isolate LIC4727C cytochrome oxidase...
Rhipicephalu... brown dog tick 34632 1007 1007 96% 0.0 95.28 761
KX383805.1
Rhipicephalus sanguineus isolate Samont2 cytochrome oxidase...
Rhipicephalu... brown dog tick 34632 1005 1005 94% 0.0 95.82 644
MH630347.1
Rhipicephalus sanguineus isolate Samont1 cytochrome oxidase...
Rhipicephalu... brown dog tick 34632 1005 1005 94% 0.0 95.82 644
MH630346.1
Rhipicephalus sanguineus clone #7 cytochrome oxidase subunit I...
Rhipicephalu... brown dog tick 34632 1002 1002 95% 0.0 95.53 654
KX519712.1
Rhipicephalus sanguineus sensu lato 'temperate lineage' isolat...
Rhipicephalu... brown dog tick 34632 1000 1000 95% 0.0 95.38 639
MF426002.1
Rhipicephalus sanguineus isolate Rsan-ML4 cytochrome oxidase...
Rhipicephalu... brown dog tick 34632 990 990 94% 0.0 95.48 630
MG855658.1
Rhipicephalus sanguineus isolate Rsan-ML3 cytochrome oxidase...
Rhipicephalu... brown dog tick 34632 990 990 94% 0.0 95.48 630
MG855657.1
Rhipicephalus sanguineus isolate Rsan-ML2 cytochrome oxidase...
Rhipicephalu... brown dog tick 34632 990 990 94% 0.0 95.48 630
MG855656.1
Rhipicephalus sanguineus isolate Kehf-Lagareb-A cytochrome...
Rhipicephalu... brown dog tick 34632 990 990 94% 0.0 95.48 630
KX757910.1
Rhipicephalus sanguineus isolate Italy-B cytochrome oxidase...
Rhipicephalu... brown dog tick 34632 990 990 94% 0.0 95.48 630
KX757904.1
Rhipicephalus sanguineus isolate Malta-A cytochrome oxidase...
Rhipicephalu... brown dog tick 34632 990 990 94% 0.0 95.48 630
KX757902.1
Rhipicephalus sanguineus isolate Zagreb-C cytochrome oxidase...
Rhipicephalu... brown dog tick 34632 990 990 94% 0.0 95.48 630
KX757896.1
Rhipicephalus sanguineus isolate Pula-B cytochrome oxidase...
Rhipicephalu... brown dog tick 34632 990 990 94% 0.0 95.48 630
KX757889.1

Rhipicephalus sanguineus isolate Rovind cytochrome oxidase...
Rhipicephalu... brown dog tick 34632 990 990 94% 0.0 95.48 630
KX757887.1
Rhipicephalus sanguineus isolate Kajtasovo cytochrome oxidase...
Rhipicephalu... brown dog tick 34632 990 990 94% 0.0 95.48 630
KX757879.1
Rhipicephalus sanguineus clone #6 cytochrome oxidase subunit I...
Rhipicephalu... brown dog tick 34632 990 990 94% 0.0 95.48 636
KX519711.1
Rhipicephalus sanguineus isolate SV4 cytochrome c oxidase...
Rhipicephalu... brown dog tick 34632 990 990 94% 0.0 95.48 630
MW135448.1
Rhipicephalus sanguineus isolate Boka-A cytochrome oxidase...
Rhipicephalu... brown dog tick 34632 985 985 94% 0.0 95.32 630
KX757905.1
Rhipicephalus sanguineus isolate Sibenik cytochrome oxidase...
Rhipicephalu... brown dog tick 34632 985 985 94% 0.0 95.32 630
KX757895.1
Rhipicephalus sanguineus isolate Zadar cytochrome oxidase...
Rhipicephalu... brown dog tick 34632 985 985 94% 0.0 95.32 630
KX757892.1
Rhipicephalus sanguineus isolate Zagreb-A cytochrome oxidase...
Rhipicephalu... brown dog tick 34632 985 985 94% 0.0 95.32 630
KX757890.1
Rhipicephalus sanguineus isolate Pula-A cytochrome oxidase...
Rhipicephalu... brown dog tick 34632 985 985 94% 0.0 95.32 630
KX757888.1
Rhipicephalus sanguineus isolate Petnica cytochrome oxidase...
Rhipicephalu... brown dog tick 34632 985 985 94% 0.0 95.32 630
KX757883.1
Rhipicephalus sanguineus clone #3 cytochrome oxidase subunit I...
Rhipicephalu... brown dog tick 34632 985 985 93% 0.0 95.46 629
KX519708.1
Rhipicephalus sanguineus isolate SV2 cytochrome c oxidase...
Rhipicephalu... brown dog tick 34632 985 985 94% 0.0 95.32 630
MW135447.1
Rhipicephalus sanguineus clone #2 cytochrome oxidase subunit I...
Rhipicephalu... brown dog tick 34632 983 983 93% 0.0 95.45 626
KX519707.1
Rhipicephalus sanguineus isolate Szekszard cytochrome oxidase...
Rhipicephalu... brown dog tick 34632 979 979 94% 0.0 95.16 630
KX757901.1
Rhipicephalus sanguineus clone #4 cytochrome oxidase subunit I...
Rhipicephalu... brown dog tick 34632 974 974 94% 0.0 95.00 635
KX519709.1
Rhipicephalus sanguineus isolate LIC4723B cytochrome oxidase...
Rhipicephalu... brown dog tick 34632 937 937 90% 0.0 94.97 723
KX714599.1
Rhipicephalus sanguineus clone #8 cytochrome oxidase subunit I...
Rhipicephalu... brown dog tick 34632 933 933 88% 0.0 95.69 580
KX519713.1
Rhipicephalus sanguineus clone #1 cytochrome oxidase subunit I...
Rhipicephalu... brown dog tick 34632 898 898 86% 0.0 95.09 595
KX519706.1

Rhipicephalus sanguineus isolate Rsang2 cytochrome oxidase...
Rhipicephalu... brown dog tick 34632 883 883 100% 0.0 90.90 790
KY678135.1
Rhipicephalus turanicus isolate COX1-225-Goat cytochrome c...
Rhipicephalu... NA 34633 883 883 100% 0.0 90.88 673
MT800313.1
Rhipicephalus sanguineus sensu lato 'temperate lineage' isolat...
Rhipicephalu... brown dog tick 34632 881 881 85% 0.0 95.01 574
MF425987.1
Rhipicephalus sanguineus cytochrome oxidase subunit 1 (CO1)...
Rhipicephalu... brown dog tick 34632 876 876 98% 0.0 91.05 829
KU214592.1
Rhipicephalus turanicus isolate GY34-1 cytochrome c oxidase...
Rhipicephalu... NA 34633 872 872 99% 0.0 90.80 678
MT079206.1
Rhipicephalus sanguineus isolate GY34 cytochrome c oxidase...
Rhipicephalu... brown dog tick 34632 872 872 99% 0.0 90.80 678
MN862754.1
Rhipicephalus sanguineus isolate Gilan-e Gharb cytochrome...
Rhipicephalu... brown dog tick 34632 872 872 100% 0.0 90.58 1539
KM494916.1
Rhipicephalus sanguineus isolate Tehran cytochrome oxidase...
Rhipicephalu... brown dog tick 34632 872 872 100% 0.0 90.58 1539
KM494915.1
Rhipicephalus turanicus isolate COX1-18-Sheep cytochrome c...
Rhipicephalu... NA 34633 870 870 99% 0.0 90.56 673
MT800314.1
Rhipicephalus turanicus isolate 31X cytochrome oxidase subunit...
Rhipicephalu... NA 34633 861 861 100% 0.0 90.27 873
KY606303.1
Rhipicephalus turanicus isolate 19X cytochrome oxidase subunit...
Rhipicephalu... NA 34633 861 861 100% 0.0 90.27 873
KY606302.1
Rhipicephalus turanicus isolate 19C cytochrome oxidase subunit...
Rhipicephalu... NA 34633 861 861 100% 0.0 90.27 873
KY606301.1
Rhipicephalus turanicus isolate 12X cytochrome oxidase subunit...
Rhipicephalu... NA 34633 861 861 100% 0.0 90.27 873
KY606300.1
Rhipicephalus turanicus isolate 12C cytochrome oxidase subunit...
Rhipicephalu... NA 34633 861 861 100% 0.0 90.27 873
KY606299.1
Rhipicephalus camicasi isolate SC0126 mitochondrion, complete...
Rhipicephalu... NA 669981 861 861 100% 0.0 90.29 14725
NC_061616.1
Rhipicephalus turanicus voucher CROBB808 cytochrome oxidase...
Rhipicephalu... NA 34633 861 861 100% 0.0 90.27 658
MZ305547.1
Rhipicephalus turanicus isolate Xinjiang-WQIA cytochrome oxida...
Rhipicephalu... NA 34633 850 850 100% 0.0 89.97 889
MF002581.1
Rhipicephalus turanicus isolate Xinjiang-WS cytochrome oxidase...
Rhipicephalu... NA 34633 850 850 100% 0.0 89.97 889
MF002580.1

```
Rhipicephalus turanicus isolate Xinjiang-BC cytochrome oxidase...
Rhipicephalu... NA              34633      850    850   100%  0.0   89.97  889
     MF002579.1
Rhipicephalus turanicus isolate Xinjiang-YC cytochrome oxidase...
Rhipicephalu... NA              34633      850    850   100%  0.0   89.97  889
     MF002578.1
Rhipicephalus turanicus isolate Xinjiang-PS cytochrome oxidase...
Rhipicephalu... NA              34633      850    850   100%  0.0   89.97  889
     MF002577.1
Rhipicephalus turanicus isolate SHZ cytochrome oxidase subunit...
Rhipicephalu... NA              34633      850    850   100%  0.0   89.97  889
     KY069271.1
Rhipicephalus turanicus isolate FK-1 cytochrome oxidase subuni...
Rhipicephalu... NA              34633      850    850   100%  0.0   89.97  889
     KY606291.1
Rhipicephalus turanicus isolate YN3 cytochrome oxidase subunit...
Rhipicephalu... NA              34633      850    850   100%  0.0   89.97  889
     KY606290.1
Rhipicephalus turanicus isolate YN2 cytochrome oxidase subunit...
Rhipicephalu... NA              34633      850    850   100%  0.0   89.97  889
     KY606289.1


Alignments:

>Rhipicephalus sanguineus mitochondrial DNA, complete genome
Sequence ID: AF081829.1 Length: 14710
Range 1: 1191 to 1848

Score:1216 bits(658), Expect:0.0,
Identities:658/658(100%),  Gaps:0/658(0%), Strand: Plus/Plus

Query  1     AACAATATATTTAATTTTTGGAGCATGATCAGGTATATTAGGACTTAGTATAAGAATATT  60
             ||||||||||||||||||||||||||||||||||||||||||||||||||||||||||||
Sbjct  1191  AACAATATATTTAATTTTTGGAGCATGATCAGGTATATTAGGACTTAGTATAAGAATATT  1250

Query  61    AATTCGTATAGAATTAGGACAACCTGGAACTTTAATTGGAAATGATCAGATTTATAATGT  120
             ||||||||||||||||||||||||||||||||||||||||||||||||||||||||||||
Sbjct  1251  AATTCGTATAGAATTAGGACAACCTGGAACTTTAATTGGAAATGATCAGATTTATAATGT  1310

Query  121   AATCGTAACAGCACATGCATTTATTATAATTTTCTTTATAGTTATACCAATCATAATCGG  180
             ||||||||||||||||||||||||||||||||||||||||||||||||||||||||||||
Sbjct  1311  AATCGTAACAGCACATGCATTTATTATAATTTTCTTTATAGTTATACCAATCATAATCGG  1370

Query  181   AGGGTTCGGAAATTGATTAGTTCCTATTATATTAGGTGCCCCAGATATAGCATTTCCACG  240
             ||||||||||||||||||||||||||||||||||||||||||||||||||||||||||||
Sbjct  1371  AGGGTTCGGAAATTGATTAGTTCCTATTATATTAGGTGCCCCAGATATAGCATTTCCACG  1430

Query  241   AATAAATAATATAAGATTTTGACTTTTACCCCCCTCATTATTTTTATTGATTAACTCTTC  300
             ||||||||||||||||||||||||||||||||||||||||||||||||||||||||||||
Sbjct  1431  AATAAATAATATAAGATTTTGACTTTTACCCCCCTCATTATTTTTATTGATTAACTCTTC  1490

Query  301   ATTAATTGAATCCGGAGCTGGAACAGGATGAACAGTTTATCCCCCTCTATCTTCAAATTT  360
             ||||||||||||||||||||||||||||||||||||||||||||||||||||||||||||
```

```
Sbjct  1491  ATTAATTGAATCCGGAGCTGGAACAGGATGAACAGTTTATCCCCCTCTATCTTCAAATTT  1550

Query  361   ATCACATTATGGTCCATCAGTAGATTTAGCTATTTTTTCTCTTCACCTTGCTGGTGCTTC  420
             ||||||||||||||||||||||||||||||||||||||||||||||||||||||||||||
Sbjct  1551  ATCACATTATGGTCCATCAGTAGATTTAGCTATTTTTTCTCTTCACCTTGCTGGTGCTTC  1610

Query  421   TTCAATTTTAGGTGCAATTAACTTCATCACAACTATTGTAAATATACGATCAATTGGAAT  480
             ||||||||||||||||||||||||||||||||||||||||||||||||||||||||||||
Sbjct  1611  TTCAATTTTAGGTGCAATTAACTTCATCACAACTATTGTAAATATACGATCAATTGGAAT  1670

Query  481   AACAATAGAACGAATACCATTATTTGTTTGATCTGTTTTAATTACAGCTATTTTACTACT  540
             ||||||||||||||||||||||||||||||||||||||||||||||||||||||||||||
Sbjct  1671  AACAATAGAACGAATACCATTATTTGTTTGATCTGTTTTAATTACAGCTATTTTACTACT  1730

Query  541   TCTATCTTTACCTGTATTAGCAGGTGCCATTACAATATTATTAACTGATCGAAACTTTAA  600
             ||||||||||||||||||||||||||||||||||||||||||||||||||||||||||||
Sbjct  1731  TCTATCTTTACCTGTATTAGCAGGTGCCATTACAATATTATTAACTGATCGAAACTTTAA  1790

Query  601   CACATCATTTTTTGATCCTTCAGGAGGAGGAGATCCCATTTTATATCAACACTTATTT  658
             ||||||||||||||||||||||||||||||||||||||||||||||||||||||||||
Sbjct  1791  CACATCATTTTTTGATCCTTCAGGAGGAGGAGATCCCATTTTATATCAACACTTATTT  1848
```

>Rhipicephalus sanguineus isolate Zagreb-B cytochrome oxidase subunit I (COI) gene, partial cds; mitochondrial
Sequence ID: KX757893.1 Length: 629
Range 1: 1 to 620

Score:1140 bits(617), Expect:0.0,
Identities:619/620(99%), Gaps:0/620(0%), Strand: Plus/Plus

```
Query  39   TAGGACTTAGTATAAGAATATTAATTCGTATAGAATTAGGACAACCTGGAACTTTAATTG  98
            ||||||||||||||||||||||||||||||||||||||||||||||||||||||||||||
Sbjct  1    TAGGACTTAGTATAAGAATATTAATTCGTATAGAATTAGGACAACCTGGAACTTTAATTG  60

Query  99   GAAATGATCAGATTTATAATGTAATCGTAACAGCACATGCATTTATTATAATTTTCTTTA  158
            ||||||||||||||||||||||||||||||||||||||||||||||||||||||||||||
Sbjct  61   GAAATGATCAGATTTATAATGTAATCGTAACAGCACATGCATTTATTATAATTTTCTTTA  120

Query  159  TAGTTATACCAATCATAATCGGAGGGTTCGGAAATTGATTAGTTCCTATTATATTAGGTG  218
            ||||||||||||||||||||||||||||||||||||||||||||||||||||||||||||
Sbjct  121  TAGTTATACCAATCATAATCGGAGGGTTCGGAAATTGATTAGTTCCTATTATATTAGGTG  180

Query  219  CCCCAGATATAGCATTTCCACGAATAAATAATATAAGATTTTGACTTTTACCCCCCTCAT  278
            ||||||||||||||||||||||||||||||||||||||||||||||||||||||||||||
Sbjct  181  CCCCAGATATAGCATTTCCACGAATAAATAATATAAGATTTTGACTTTTACCCCCCTCAT  240

Query  279  TATTTTTATTGATTAACTCTTCATTAATTGAATCCGGAGCTGGAACAGGATGAACAGTTT  338
            ||||||||||||||||||||||||||||||||||||||||||||||||||||||||||||
Sbjct  241  TATTTTTATTGATTAACTCTTCATTAATTGAATCCGGAGCTGGAACAGGATGAACAGTTT  300

Query  339  ATCCCCCTCTATCTTCAAATTTATCACATTATGGTCCATCAGTAGATTTAGCTATTTTTT  398
```

```
            ||||||||||||||||||||||||||||||||||||||||||||||||||||||||||||
Sbjct  301  ATCCCCCTCTATCTTCAAATTTATCACATTATGGTCCATCAGTAGATTTAGCTATTTTTT  360

Query  399  CTCTTCACCTTGCTGGTGCTTCTTCAATTTTAGGTGCAATTAACTTCATCACAACTATTG  458
            ||||||||||||||||||||||||||||||||||||||||||||||||||||||||||||
Sbjct  361  CTCTTCACCTTGCTGGTGCTTCTTCAATTTTAGGTGCAATTAACTTCATCACAACTATTG  420

Query  459  TAAATATACGATCAATTGGAATAACAATAGAACGAATACCATTATTTGTTTGATCTGTTT  518
            ||||||||||||||||||||||||||||||||||||||||||||||||||||||||||||
Sbjct  421  TAAATATACGATCAATTGGAATAACAATAGAACGAATACCATTATTTGTTTGATCTGTTT  480

Query  519  TAATTACAGCTATTTTACTACTTCTATCTTTACCTGTATTAGCAGGTGCCATTACAATAT  578
            ||||||||||||||||||||||||||||||||||||||||||||||||||||||||||||
Sbjct  481  TAATTACAGCTATTTTACTACTTCTATCTTTACCTGTATTAGCAGGTGCCATTACAATAT  540

Query  579  TATTAACTGATCGAAACTTTAACACATCATTTTTGATCCTTCAGGAGGAGGAGATCCCA  638
            |||||||||||||||||||||||||||||||||||| |||||||||||||||||||||||
Sbjct  541  TATTAACTGATCGAAACTTTAACACATCATTTTTGACCCTTCAGGAGGAGGAGATCCCA  600

Query  639  TTTTATATCAACACTTATTT  658
            ||||||||||||||||||||
Sbjct  601  TTTTATATCAACACTTATTT  620
```

>Rhipicephalus sanguineus isolate Italy-A cytochrome oxidase subunit I (COI) gene, partial cds; mitochondrial
Sequence ID: KX757903.1 Length: 630
Range 1: 1 to 620

Score:1129 bits(611), Expect:0.0,
Identities:617/620(99%), Gaps:0/620(0%), Strand: Plus/Plus

```
Query  39   TAGGACTTAGTATAAGAATATTAATTCGTATAGAATTAGGACAACCTGGAACTTTAATTG  98
            ||||||||||||||||||||||||||||||||||||||||||||||||||||||||||||
Sbjct  1    TAGGACTTAGTATAAGAATATTAATTCGTATAGAATTAGGACAACCTGGAACTTTAATTG  60

Query  99   GAAATGATCAGATTTATAATGTAATCGTAACAGCACATGCATTTATTATAATTTTCTTTA  158
            ||||||||||||||||||||||||||||||||||||||||||||||||||||||||||||
Sbjct  61   GAAATGATCAGATTTATAATGTAATCGTAACAGCACATGCATTTATTATAATTTTCTTTA  120

Query  159  TAGTTATACCAATCATAATCGGAGGGTTCGGAAATTGATTAGTTCCTATTATATTAGGTG  218
            ||||||||||||||||||||||||||||||||||||||||||||||||||||||||||||
Sbjct  121  TAGTTATACCAATCATAATCGGAGGGTTCGGAAATTGATTAGTTCCTATTATATTAGGTG  180

Query  219  CCCCAGATATAGCATTTCCACGAATAAATAATATAAGATTTTGACTTTTACCCCCCTCAT  278
            ||||||||||||||||||||||||||||||||||||||||||||||||||||||||||||
Sbjct  181  CCCCAGATATAGCATTTCCACGAATAAATAATATAAGATTTTGACTTTTACCCCCCTCAT  240

Query  279  TATTTTTATTGATTAACTCTTCATTAATTGAATCCGGAGCTGGAACAGGATGAACAGTTT  338
            |||||||||||||||| |||||||||||||||||||||||||||||||||||||||||||
Sbjct  241  TATTTTTATTGATTAATTCTTCATTAATTGAATCCGGAGCTGGAACAGGATGAACAGTTT  300
```

```
Query  339  ATCCCCCTCTATCTTCAAATTTATCACATTATGGTCCATCAGTAGATTTAGCTATTTTTT  398
            ||||||||||||||||||||||||||||||||||||||||||||||||||||||||||||
Sbjct  301  ATCCCCCTCTATCTTCAAATTTATCACATTATGGTCCATCAGTAGATTTAGCTATTTTTT  360

Query  399  CTCTTCACCTTGCTGGTGCTTCTTCAATTTTAGGTGCAATTAACTTCATCACAACTATTG  458
            ||||||||||||||||||||||||||||||||||||||||||||||||| ||||||||||
Sbjct  361  CTCTTCACCTTGCTGGTGCTTCTTCAATTTTAGGTGCAATTAACTTCATTACAACTATTG  420

Query  459  TAAATATACGATCAATTGGAATAACAATAGAACGAATACCATTATTTGTTTGATCTGTTT  518
            ||||||||||||||||||||||||||||||||||||||||||||||||||||||||||||
Sbjct  421  TAAATATACGATCAATTGGAATAACAATAGAACGAATACCATTATTTGTTTGATCTGTTT  480

Query  519  TAATTACAGCTATTTTACTACTTCTATCTTTACCTGTATTAGCAGGTGCCATTACAATAT  578
            ||||||||||||||||||||||||||||||||||||||||||||||||||||||||||||
Sbjct  481  TAATTACAGCTATTTTACTACTTCTATCTTTACCTGTATTAGCAGGTGCCATTACAATAT  540

Query  579  TATTAACTGATCGAAACTTTAACACATCATTTTTGATCCTTCAGGAGGAGGAGATCCCA  638
            ||||||||||||||||||||||||||||||||||||| ||||||||||||||||||||||
Sbjct  541  TATTAACTGATCGAAACTTTAACACATCATTTTTGACCCTTCAGGAGGAGGAGATCCCA  600

Query  639  TTTTATATCAACACTTATTT  658
            ||||||||||||||||||||
Sbjct  601  TTTTATATCAACACTTATTT  620
```

>Rhipicephalus sanguineus isolate Al-Hosema-A cytochrome oxidase subunit I (COI) gene, partial cds; mitochondrial
Sequence ID: KX757909.1 Length: 630
Range 1: 1 to 620

Score:1112 bits(602), Expect:0.0,
Identities:614/620(99%), Gaps:0/620(0%), Strand: Plus/Plus

```
Query  39   TAGGACTTAGTATAAGAATATTAATTCGTATAGAATTAGGACAACCTGGAACTTTAATTG  98
            ||||||||||||||||||||||||||||||||||||||||||||||||||||||||||||
Sbjct  1    TAGGACTTAGTATAAGAATATTAATTCGTATAGAATTAGGACAACCTGGAACTTTAATTG  60

Query  99   GAAATGATCAGATTTATAATGTAATCGTAACAGCACATGCATTTATTATAATTTTCTTTA  158
            ||||||||||||||||||||||||||||||||||||||||||||||||||||||||||||
Sbjct  61   GAAATGATCAGATTTATAATGTAATCGTAACAGCACATGCATTTATTATAATTTTCTTTA  120

Query  159  TAGTTATACCAATCATAATCGGAGGGTTCGGAAATTGATTAGTTCCTATTATATTAGGTG  218
            ||||||||||||| ||||||||||||||||||||||||||||||||||||||||||||||
Sbjct  121  TAGTTATACCAATAATAATCGGAGGGTTCGGAAATTGATTAGTTCCTATTATATTAGGTG  180

Query  219  CCCCAGATATAGCATTTCCACGAATAAATAATATAAGATTTTGACTTTTACCCCCCTCAT  278
            ||||||||||||||||||||||||||||||||||||||||||||||||||||||||||||
Sbjct  181  CCCCAGATATAGCATTTCCACGAATAAATAATATAAGATTTTGACTTTTACCCCCCTCAT  240

Query  279  TATTTTTATTGATTAACTCTTCATTAATTGAATCCGGAGCTGGAACAGGATGAACAGTTT  338
            |||||||||||||||||||||||||||||||||| |||||||||||||||||||||||||
Sbjct  241  TATTTTTATTGATTAACTCTTCATTAATTGAATCTGGAGCTGGAACAGGATGAACAGTTT  300
```

```
Query  339   ATCCCCCTCTATCTTCAAATTTATCACATTATGGTCCATCAGTAGATTTAGCTATTTTTT  398
             |||||||||||||||||||||||||||||||||| |||||||||||||||||||||||||
Sbjct  301   ATCCCCCTCTATCTTCAAATTTATCACATTATGGCCCATCAGTAGATTTAGCTATTTTTT  360

Query  399   CTCTTCACCTTGCTGGTGCTTCTTCAATTTTAGGTGCAATTAACTTCATCACAACTATTG  458
             ||||||||||||||||||||||||||||||||||||||||||||||||||||||||||||
Sbjct  361   CTCTTCACCTTGCTGGTGCTTCTTCAATTTTAGGTGCAATTAACTTCATCACAACTATTG  420

Query  459   TAAATATACGATCAATTGGAATAACAATAGAACGAATACCATTATTTGTTTGATCTGTTT  518
             ||||||||||||||||||||||||||||||||||||||||||||||||||||||||||||
Sbjct  421   TAAATATACGATCAATTGGAATAACAATAGAACGAATACCATTATTTGTTTGATCTGTTT  480

Query  519   TAATTACAGCTATTTTACTACTTCTATCTTTACCTGTATTAGCAGGTGCCATTACAATAT  578
             ||||||||||||||||||||||||||||||||||||||||||||||||||||||||||||
Sbjct  481   TAATTACAGCTATTTTACTACTTCTATCTTTACCTGTATTAGCAGGTGCCATTACAATAT  540

Query  579   TATTAACTGATCGAAACTTTAACACATCATTTTTTGATCCTTCAGGAGGAGGAGATCCCA  638
             |||| |||||||||||||||||||||||||||||||| |||||||||||||| |||||||
Sbjct  541   TATTGACTGATCGAAACTTTAACACATCATTTTTTGACCCTTCAGGAGGAGGGGATCCCA  600

Query  639   TTTTATATCAACACTTATTT  658
             ||||||||||||||||||||
Sbjct  601   TTTTATATCAACACTTATTT  620
```

>Rhipicephalus sanguineus sensu lato 'temperate lineage' isolate S48 cytochrome c oxidase subunit I (COI) gene, partial cds; mitochondrial
Sequence ID: MF426015.1 Length: 692
Range 1: 19 to 676

Score:1072 bits(580), Expect:0.0,
Identities:632/658(96%), Gaps:0/658(0%), Strand: Plus/Plus

```
Query  1     AACAATATATTTAATTTTTGGAGCATGATCAGGTATATTAGGACTTAGTATAAGAATATT  60
             ||||||||||||||||||||||||||||||||||||||||||||||||||||||||||||
Sbjct  19    AACAATATATTTAATTTTTGGAGCATGATCAGGTATATTAGGACTTAGTATAAGAATATT  78

Query  61    AATTCGTATAGAATTAGGACAACCTGGAACTTTAATTGGAAATGATCAGATTTATAATGT  120
             ||||||||||||||||||||||||||||||||||||||||||||||||||||||||||||
Sbjct  79    AATTCGTATAGAATTAGGACAACCTGGAACTTTAATTGGAAATGATCAGATTTATAATGT  138

Query  121   AATCGTAACAGCACATGCATTTATTATAATTTTCTTTATAGTTATACCAATCATAATCGG  180
             ||||||||||||||||||||||||||||||||| ||||||||||||||||||||||||||
Sbjct  139   AATCGTAACAGCACATGCATTTATTATAATTTTTTTTATAGTTATACCAATCATAATCGG  198

Query  181   AGGGTTCGGAAATTGATTAGTTCCTATTATATTAGGTGCCCCAGATATAGCATTTCCACG  240
             ||| || |||||||||||||| ||||| ||||| ||||||||||||||||||||||||||
Sbjct  199   AGGATTTGGAAATTGATTAGTCCCTATCATATTGGGTGCCCCAGATATAGCATTTCCACG  258

Query  241   AATAAATAATATAAGATTTTGACTTTTACCCCCCTCATTATTTTTATTGATTAACTCTTC  300
             |||||||||||||||||||||||||||||| || ||| |||||||||| |||||||||||
```

```
Sbjct  259   AATAAATAATATAAGATTTTGACTTTTACCTCCTTCACTATTTTTATTAATTAACTCTTC  318

Query  301   ATTAATTGAATCCGGAGCTGGAACAGGATGAACAGTTTATCCCCCTCTATCTTCAAATTT  360
             |||||||||||| |||||||| ||||||||||||||||| || |||||||||||||||||
Sbjct  319   ATTAATTGAATCTGGAGCTGGTACAGGATGAACAGTTTACCCTCCTCTATCTTCAAATTT  378

Query  361   ATCACATTATGGTCCATCAGTAGATTTAGCTATTTTTTCTCTTCACCTTGCTGGTGCTTC  420
             ||||||||||||||| ||||||||||||||||||||||||||||| ||||||||||||||
Sbjct  379   ATCACATTATGGTCCTTCAGTAGATTTAGCTATTTTTTCTCTTCATCTTGCTGGTGCTTC  438

Query  421   TTCAATTTTAGGTGCAATTAACTTCATCACAACTATTGTAAATATACGATCAATTGGAAT  480
             |||||||||||||||||||||||| || |||||||||||||| |||||||||||||||||
Sbjct  439   TTCAATTTTAGGTGCAATTAACTTTATTACAACTATTGTAAACATACGATCAATTGGAAT  498

Query  481   AACAATAGAACGAATACCATTATTTGTTTGATCTGTTTTAATTACAGCTATTTTACTACT  540
             |||||||||||| |||||||||||||||||||||||||||||||||||||||||||||||
Sbjct  499   AACAATAGAACGTATACCATTATTTGTTTGATCTGTTTTAATTACAGCTATTTTACTACT  558

Query  541   TCTATCTTTACCTGTATTAGCAGGTGCCATTACAATATTATTAACTGATCGAAACTTTAA  600
             | ||||||||||||||||||||||||||||||||||||| ||||||||||||||||||||
Sbjct  559   TTTATCTTTACCTGTATTAGCAGGTGCCATTACAATATTGTTAACTGATCGAAACTTTAA  618

Query  601   CACATCATTTTTTGATCCTTCAGGAGGAGGAGATCCCATTTTATATCAACACTTATTT  658
              ||||||||||||||||||||||| ||||||||||| |||||||||||||| ||||||
Sbjct  619   TACATCATTTTTTGATCCTTCAGGGGGAGGAGATCCAATTTTATATCAACATTTATTT  676
```

>Rhipicephalus sanguineus sensu lato 'temperate lineage' isolate S58 cytochrome c oxidase subunit I (COI) gene, partial cds; mitochondrial
Sequence ID: MF426018.1 Length: 675
Range 1: 11 to 668

Score:1066 bits(577), Expect:0.0,
Identities:631/658(96%), Gaps:0/658(0%), Strand: Plus/Plus

```
Query  1     AACAATATATTTAATTTTTGGAGCATGATCAGGTATATTAGGACTTAGTATAAGAATATT  60
             ||||||||||||||||||||||||||||||||||||||||||||||||||||||||||||
Sbjct  11    AACAATATATTTAATTTTTGGAGCATGATCAGGTATATTAGGACTTAGTATAAGAATATT  70

Query  61    AATTCGTATAGAATTAGGACAACCTGGAACTTTAATTGGAAATGATCAGATTTATAATGT  120
             ||||||||||||||||||||||||||||||||||||||||||||||||||||||||||||
Sbjct  71    AATTCGTATAGAATTAGGACAACCTGGAACTTTAATTGGAAATGATCAGATTTATAATGT  130

Query  121   AATCGTAACAGCACATGCATTTATTATAATTTTCTTTATAGTTATACCAATCATAATCGG  180
             ||||||||||||||||||||||||||||||||| ||||||||||||||||||||||||||
Sbjct  131   AATCGTAACAGCACATGCATTTATTATAATTTTTTTTATAGTTATACCAATCATAATCGG  190

Query  181   AGGGTTCGGAAATTGATTAGTTCCTATTATATTAGGTGCCCCAGATATAGCATTTCCACG  240
             ||| || |||||||||||||| ||||| ||||| ||||||||||||||||||||||||||
Sbjct  191   AGGATTTGGAAATTGATTAGTCCCTATCATATTGGGTGCCCCAGATATAGCATTTCCACG  250

Query  241   AATAAATAATATAAGATTTTGACTTTTACCCCCTCATTATTTTTATTGATTAACTCTTC  300
```

```
                ||||||||||||||||||||||||||||||| || ||| |||||||||| |||||||||||
Sbjct  251   AATAAATAATATAAGATTTTGACTTTTACCTCCTTCACTATTTTTATTAATTAACTCTTC  310

Query  301   ATTAATTGAATCCGGAGCTGGAACAGGATGAACAGTTTATCCCCCTCTATCTTCAAATTT  360
             |||||||||||| |||||||| ||||||||||||||||| || |||||||||||||||||
Sbjct  311   ATTAATTGAATCTGGAGCTGGTACAGGATGAACAGTTTACCCTCCTCTATCTTCAAATTT  370

Query  361   ATCACATTATGGTCCATCAGTAGATTTAGCTATTTTTTCTCTTCACCTTGCTGGTGCTTC  420
             ||||||||||||||| ||||||||||||||||||||||||||||| ||||||||||||||
Sbjct  371   ATCACATTATGGTCCTTCAGTAGATTTAGCTATTTTTTCTCTTCATCTTGCTGGTGCTTC  430

Query  421   TTCAATTTTAGGTGCAATTAACTTCATCACAACTATTGTAAATATACGATCAATTGGAAT  480
             |||||||||||||||||||||||| || |||||||||||||| |||||||||||||||||
Sbjct  431   TTCAATTTTAGGTGCAATTAACTTTATTACAACTATTGTAAACATACGATCAATTGGAAT  490

Query  481   AACAATAGAACGAATACCATTATTTGTTTGATCTGTTTTAATTACAGCTATTTTACTACT  540
             |||||||||||| |||||||||||||||||||||||||||||||||||||||||| ||||
Sbjct  491   AACAATAGAACGTATACCATTATTTGTTTGATCTGTTTTAATTACAGCTATTTTATTACT  550

Query  541   TCTATCTTTACCTGTATTAGCAGGTGCCATTACAATATTATTAACTGATCGAAACTTTAA  600
             | ||||||||||||||||||||||||||||||||||||| ||||||||||||||||||||
Sbjct  551   TTTATCTTTACCTGTATTAGCAGGTGCCATTACAATATTGTTAACTGATCGAAACTTTAA  610

Query  601   CACATCATTTTTTGATCCTTCAGGAGGAGGAGATCCCATTTTATATCAACACTTATTT  658
              ||||||||||||||||||||||| ||||||||||| |||||||||||||| ||||||
Sbjct  611   TACATCATTTTTTGATCCTTCAGGGGGAGGAGATCCAATTTTATATCAACATTTATTT  668
```

>Rhipicephalus sanguineus sensu lato 'temperate lineage' isolate S52 cytochrome c oxidase subunit I (COI) gene, partial cds; mitochondrial
Sequence ID: MF426016.1 Length: 680
Range 1: 12 to 669

Score:1066 bits(577), Expect:0.0,
Identities:631/658(96%), Gaps:0/658(0%), Strand: Plus/Plus

```
Query  1     AACAATATATTTAATTTTTGGAGCATGATCAGGTATATTAGGACTTAGTATAAGAATATT  60
             ||||||||||||||||||||||||||||||||||||||||||||||||||||||||||||
Sbjct  12    AACAATATATTTAATTTTTGGAGCATGATCAGGTATATTAGGACTTAGTATAAGAATATT  71

Query  61    AATTCGTATAGAATTAGGACAACCTGGAACTTTAATTGGAAATGATCAGATTTATAATGT  120
             ||||||||||||||||||||||||||||||||||||||||||||||||||||||||||||
Sbjct  72    AATTCGTATAGAATTAGGACAACCTGGAACTTTAATTGGAAATGATCAGATTTATAATGT  131

Query  121   AATCGTAACAGCACATGCATTTATTATAATTTCTTTATAGTTATACCAATCATAATCGG  180
             |||||||||||||||||||||||||||||||| |||||||||||||||||||||||||||
Sbjct  132   AATCGTAACAGCACATGCATTTATTATAATTTTTTTATAGTTATACCAATCATAATCGG  191

Query  181   AGGGTTCGGAAATTGATTAGTTCCTATTATATTAGGTGCCCCAGATATAGCATTTCCACG  240
             ||| || |||||||||||||| ||||| ||||| ||||||||||||||||||||||||||
Sbjct  192   AGGATTTGGAAATTGATTAGTCCCTATCATATTGGGTGCCCCAGATATAGCATTTCCACG  251
```

```
Query  241  AATAAATAATATAAGATTTTGACTTTTACCCCCCTCATTATTTTTATTGATTAACTCTTC  300
            |||||||||||||||||||||||||||||| || ||| |||||||||| |||||||||||
Sbjct  252  AATAAATAATATAAGATTTTGACTTTTACCTCCTTCACTATTTTTATTAATTAACTCTTC  311

Query  301  ATTAATTGAATCCGGAGCTGGAACAGGATGAACAGTTTATCCCCCTCTATCTTCAAATTT  360
            |||||||||||| |||||||| ||||||||||||||||| || |||||||||||||||||
Sbjct  312  ATTAATTGAATCTGGAGCTGGTACAGGATGAACAGTTTACCCTCCTCTATCTTCAAATTT  371

Query  361  ATCACATTATGGTCCATCAGTAGATTTAGCTATTTTTTCTCTTCACCTTGCTGGTGCTTC  420
            ||||||||||||||| ||||||||||||||||||||||||||||| ||||||||||||||
Sbjct  372  ATCACATTATGGTCCTTCAGTAGATTTAGCTATTTTTTCTCTTCATCTTGCTGGTGCTTC  431

Query  421  TTCAATTTTAGGTGCAATTAACTTCATCACAACTATTGTAAATATACGATCAATTGGAAT  480
            |||||||||||||||||||||||| || |||||||||||||| |||||||||||||||||
Sbjct  432  TTCAATTTTAGGTGCAATTAACTTTATTACAACTATTGTAAACATACGATCAATTGGAAT  491

Query  481  AACAATAGAACGAATACCATTATTTGTTTGATCTGTTTTAATTACAGCTATTTTACTACT  540
            |||||||||||| |||||||||||||||||||||||||||||||||||||||||| ||||
Sbjct  492  AACAATAGAACGTATACCATTATTTGTTTGATCTGTTTTAATTACAGCTATTTTATTACT  551

Query  541  TCTATCTTTACCTGTATTAGCAGGTGCCATTACAATATTATTAACTGATCGAAACTTTAA  600
            | ||||||||||||||||||||||||||||||||||||| ||||||||||||||||||||
Sbjct  552  TTTATCTTTACCTGTATTAGCAGGTGCCATTACAATATTGTTAACTGATCGAAACTTTAA  611

Query  601  CACATCATTTTTTGATCCTTCAGGAGGAGGAGATCCCATTTTATATCAACACTTATTT  658
             ||||||||||||||||||||||| ||||||||||| |||||||||||||| ||||||
Sbjct  612  TACATCATTTTTTGATCCTTCAGGGGGAGGAGATCCAATTTTATATCAACATTTATTT  669
```

>Rhipicephalus sanguineus sensu lato 'temperate lineage' isolate S37 cytochrome c oxidase subunit I (COI) gene, partial cds; mitochondrial
Sequence ID: MF426013.1 Length: 693
Range 1: 20 to 677

Score:1066 bits(577), Expect:0.0,
Identities:631/658(96%), Gaps:0/658(0%), Strand: Plus/Plus

```
Query  1    AACAATATATTTAATTTTTGGAGCATGATCAGGTATATTAGGACTTAGTATAAGAATATT  60
            ||||||||||||||||||||||||||||||||||||||||||||||||||||||||||||
Sbjct  20   AACAATATATTTAATTTTTGGAGCATGATCAGGTATATTAGGACTTAGTATAAGAATATT  79

Query  61   AATTCGTATAGAATTAGGACAACCTGGAACTTTAATTGGAAATGATCAGATTTATAATGT  120
            ||||||||||||||||||||||||||||||||||||||||||||||||||||||||||||
Sbjct  80   AATTCGTATAGAATTAGGACAACCTGGAACTTTAATTGGAAATGATCAGATTTATAATGT  139

Query  121  AATCGTAACAGCACATGCATTTATTATAATTTTCTTTATAGTTATACCAATCATAATCGG  180
            ||||||||||||||||||||||||||||||||| ||||||||||||||||||||||||||
Sbjct  140  AATCGTAACAGCACATGCATTTATTATAATTTTTTTTATAGTTATACCAATCATAATCGG  199

Query  181  AGGGTTCGGAAATTGATTAGTTCCTATTATATTAGGTGCCCCAGATATAGCATTTCCACG  240
            ||| || |||||||||||||| ||||| ||||| ||||||||||||||||||||||||||
Sbjct  200  AGGATTTGGAAATTGATTAGTCCCTATCATATTGGGTGCCCCAGATATAGCATTTCCACG  259
```

```
Query  241  AATAAATAATATAAGATTTTGACTTTTACCCCCCTCATTATTTTTATTGATTAACTCTTC  300
            |||||||||||||||||||||||||||||| || ||| |||||||||| |||||||||||
Sbjct  260  AATAAATAATATAAGATTTTGACTTTTACCTCCTTCACTATTTTTATTAATTAACTCTTC  319

Query  301  ATTAATTGAATCCGGAGCTGGAACAGGATGAACAGTTTATCCCCCTCTATCTTCAAATTT  360
            |||||||||||| |||||||| ||||||||||||||||| || |||||||||||||||||
Sbjct  320  ATTAATTGAATCTGGAGCTGGTACAGGATGAACAGTTTACCCTCCTCTATCTTCAAATTT  379

Query  361  ATCACATTATGGTCCATCAGTAGATTTAGCTATTTTTTCTCTTCACCTTGCTGGTGCTTC  420
            ||||||||||||||| |||||||||||||||||||||||||||||| ||||||||||||||
Sbjct  380  ATCACATTATGGTCCTTCAGTAGATTTAGCTATTTTTTCTCTTCATCTTGCTGGTGCTTC  439

Query  421  TTCAATTTTAGGTGCAATTAACTTCATCACAACTATTGTAAATATACGATCAATTGGAAT  480
            |||||||||||||||||||||||| || ||||||||||||||| ||||||||||||||||
Sbjct  440  TTCAATTTTAGGTGCAATTAACTTTATTACAACTATTGTAAACATACGATCAATTGGAAT  499

Query  481  AACAATAGAACGAATACCATTATTTGTTTGATCTGTTTTAATTACAGCTATTTTACTACT  540
            |||||||||||| |||||||||||||||||||||||||||||||||||||||||| ||||
Sbjct  500  AACAATAGAACGTATACCATTATTTGTTTGATCTGTTTTAATTACAGCTATTTTATTACT  559

Query  541  TCTATCTTTACCTGTATTAGCAGGTGCCATTACAATATTATTAACTGATCGAAACTTTAA  600
            | ||||||||||||||||||||||||||||||||||||| ||||||||||||||||||||
Sbjct  560  TTTATCTTTACCTGTATTAGCAGGTGCCATTACAATATTGTTAACTGATCGAAACTTTAA  619

Query  601  CACATCATTTTTTGATCCTTCAGGAGGAGGAGATCCCATTTTATATCAACACTTATTT  658
             ||||||||||||||||||||||| ||||||||||| |||||||||||||| ||||||
Sbjct  620  TACATCATTTTTTGATCCTTCAGGGGGAGGAGATCCAATTTTATATCAACATTTATTT  677
```

>Rhipicephalus sanguineus sensu lato 'temperate lineage' isolate S33 cytochrome c oxidase subunit I (COI) gene, partial cds; mitochondrial
Sequence ID: MF426009.1 Length: 679
Range 1: 11 to 668

Score:1066 bits(577), Expect:0.0,
Identities:631/658(96%), Gaps:0/658(0%), Strand: Plus/Plus

```
Query  1    AACAATATATTTAATTTTTGGAGCATGATCAGGTATATTAGGACTTAGTATAAGAATATT  60
            ||||||||||||||||||||||||||||||||||||||||||||||||||||||||||||
Sbjct  11   AACAATATATTTAATTTTTGGAGCATGATCAGGTATATTAGGACTTAGTATAAGAATATT  70

Query  61   AATTCGTATAGAATTAGGACAACCTGGAACTTTAATTGGAAATGATCAGATTTATAATGT  120
            ||||||||||||||||||||||||||||||||||||||||||||||||||||||||||||
Sbjct  71   AATTCGTATAGAATTAGGACAACCTGGAACTTTAATTGGAAATGATCAGATTTATAATGT  130

Query  121  AATCGTAACAGCACATGCATTTATTATAATTTCTTTATAGTTATACCAATCATAATCGG  180
            |||||||||||||||||||||||||||||||| |||||||||||||||||||||||||||
Sbjct  131  AATCGTAACAGCACATGCATTTATTATAATTTTTTTATAGTTATACCAATCATAATCGG  190

Query  181  AGGGTTCGGAAATTGATTAGTTCCTATTATATTAGGTGCCCCAGATATAGCATTTCCACG  240
            ||| || |||||||||||||| ||||| ||||| ||||||||||||||||||||||||||
```

```
Sbjct  191  AGGATTTGGAAATTGATTAGTCCCTATCATATTGGGTGCCCCAGATATAGCATTTCCACG  250

Query  241  AATAAATAATATAAGATTTTGACTTTTACCCCCCTCATTATTTTTATTGATTAACTCTTC  300
            |||||||||||||||||||||||||||||| || ||| |||||||||| |||||||||||
Sbjct  251  AATAAATAATATAAGATTTTGACTTTTACCTCCTTCACTATTTTTATTAATTAACTCTTC  310

Query  301  ATTAATTGAATCCGGAGCTGGAACAGGATGAACAGTTTATCCCCCTCTATCTTCAAATTT  360
            |||||||||||| |||||||| ||||||||||||||||| || |||||||||||||||||
Sbjct  311  ATTAATTGAATCTGGAGCTGGTACAGGATGAACAGTTTACCCTCCTCTATCTTCAAATTT  370

Query  361  ATCACATTATGGTCCATCAGTAGATTTAGCTATTTTTTCTCTTCACCTTGCTGGTGCTTC  420
            ||||||||||||||| |||||||||||||||||||||||||||||| |||||||||||||
Sbjct  371  ATCACATTATGGTCCTTCAGTAGATTTAGCTATTTTTTCTCTTCATCTTGCTGGTGCTTC  430

Query  421  TTCAATTTAGGTGCAATTAACTTCATCACAACTATTGTAAATATACGATCAATTGGAAT  480
            ||||||||||||||||||||||| || |||||||||||||| ||||||||||||||||||
Sbjct  431  TTCAATTTAGGTGCAATTAACTTTATTACAACTATTGTAAACATACGATCAATTGGAAT  490

Query  481  AACAATAGAACGAATACCATTATTTGTTTGATCTGTTTTAATTACAGCTATTTTACTACT  540
            |||||||||||| |||||||||||||||||||||||||||||||||||||||||| ||||
Sbjct  491  AACAATAGAACGTATACCATTATTTGTTTGATCTGTTTTAATTACAGCTATTTTATTACT  550

Query  541  TCTATCTTTACCTGTATTAGCAGGTGCCATTACAATATTATTAACTGATCGAAACTTTAA  600
            | ||||||||||||||||||||||||||||||||||||| ||||||||||||||||||||
Sbjct  551  TTTATCTTTACCTGTATTAGCAGGTGCCATTACAATATTGTTAACTGATCGAAACTTTAA  610

Query  601  CACATCATTTTTTGATCCTTCAGGAGGAGGAGATCCCATTTTATATCAACACTTATTT  658
             ||||||||||||||||||||||| ||||||||||| |||||||||||||| ||||||
Sbjct  611  TACATCATTTTTTGATCCTTCAGGGGGAGGAGATCCAATTTTATATCAACATTTATTT  668
```

>Rhipicephalus sanguineus sensu lato 'temperate lineage' isolate S29 cytochrome c oxidase subunit I (COI) gene, partial cds; mitochondrial
Sequence ID: MF426007.1 Length: 686
Range 1: 14 to 671

Score:1066 bits(577), Expect:0.0,
Identities:631/658(96%), Gaps:0/658(0%), Strand: Plus/Plus

```
Query  1    AACAATATATTTAATTTTTGGAGCATGATCAGGTATATTAGGACTTAGTATAAGAATATT  60
            ||||||||||||||||||||||||||||||||||||||||||||||||||||||||||||
Sbjct  14   AACAATATATTTAATTTTTGGAGCATGATCAGGTATATTAGGACTTAGTATAAGAATATT  73

Query  61   AATTCGTATAGAATTAGGACAACCTGGAACTTTAATTGGAAATGATCAGATTTATAATGT  120
            ||||||||||||||||||||||||||||||||||||||||||||||||||||||||||||
Sbjct  74   AATTCGTATAGAATTAGGACAACCTGGAACTTTAATTGGAAATGATCAGATTTATAATGT  133

Query  121  AATCGTAACAGCACATGCATTTATTATAATTTCTTTATAGTTATACCAATCATAATCGG  180
            |||||||||||||||||||||||||||||||| |||||||||||||||||||||||||||
Sbjct  134  AATCGTAACAGCACATGCATTTATTATAATTTTTTTATAGTTATACCAATCATAATCGG  193

Query  181  AGGGTTCGGAAATTGATTAGTTCCTATTATATTAGGTGCCCCAGATATAGCATTTCCACG  240
```

```
            ||| || |||||||||||||| ||||| ||||| ||||||||||||||||||||||||||
Sbjct  194  AGGATTTGGAAATTGATTAGTCCCTATCATATTGGGTGCCCCAGATATAGCATTTCCACG  253

Query  241  AATAAATAATATAAGATTTTGACTTTTACCCCCTCATTATTTTTATTGATTAACTCTTC  300
            |||||||||||||||||||||||||||||| || ||| |||||||||| |||||||||||
Sbjct  254  AATAAATAATATAAGATTTTGACTTTTACCTCCTTCACTATTTTTATTAATTAACTCTTC  313

Query  301  ATTAATTGAATCCGGAGCTGGAACAGGATGAACAGTTTATCCCCCTCTATCTTCAAATTT  360
            |||||||||||| |||||||| ||||||||||||||||| || |||||||||||||||||
Sbjct  314  ATTAATTGAATCTGGAGCTGGTACAGGATGAACAGTTTACCCTCCTCTATCTTCAAATTT  373

Query  361  ATCACATTATGGTCCATCAGTAGATTTAGCTATTTTTTCTCTTCACCTTGCTGGTGCTTC  420
            ||||||||||||||| ||||||||||||||||||||||||||||| ||||||||||||||
Sbjct  374  ATCACATTATGGTCCTTCAGTAGATTTAGCTATTTTTTCTCTTCATCTTGCTGGTGCTTC  433

Query  421  TTCAATTTTAGGTGCAATTAACTTCATCACAACTATTGTAAATATACGATCAATTGGAAT  480
            |||||||||||||||||||||||| || |||||||||||||| |||||||||||||||||
Sbjct  434  TTCAATTTTAGGTGCAATTAACTTTATTACAACTATTGTAAACATACGATCAATTGGAAT  493

Query  481  AACAATAGAACGAATACCATTATTTGTTTGATCTGTTTTAATTACAGCTATTTTACTACT  540
            |||||||||||| |||||||||||||||||||||||||||||||||||||||||| ||||
Sbjct  494  AACAATAGAACGTATACCATTATTTGTTTGATCTGTTTTAATTACAGCTATTTTATTACT  553

Query  541  TCTATCTTTACCTGTATTAGCAGGTGCCATTACAATATTATTAACTGATCGAAACTTTAA  600
            | ||||||||||||||||||||||||||||||||||||| ||||||||||||||||||||
Sbjct  554  TTTATCTTTACCTGTATTAGCAGGTGCCATTACAATATTGTTAACTGATCGAAACTTTAA  613

Query  601  CACATCATTTTTTGATCCTTCAGGAGGAGGAGATCCCATTTTATATCAACACTTATTT  658
             ||||||||||||||||||||||| ||||||||||| |||||||||||||| ||||||
Sbjct  614  TACATCATTTTTTGATCCTTCAGGGGGAGGAGATCCAATTTTATATCAACATTTATTT  671
```

>Rhipicephalus sanguineus sensu lato 'temperate lineage' isolate S827 cytochrome c oxidase subunit I (COI) gene, partial cds; mitochondrial
Sequence ID: MF426019.1 Length: 697
Range 1: 19 to 676

Score:1061 bits(574), Expect:0.0,
Identities:630/658(96%), Gaps:0/658(0%), Strand: Plus/Plus

```
Query  1    AACAATATATTTAATTTTTGGAGCATGATCAGGTATATTAGGACTTAGTATAAGAATATT  60
            ||||||||||||||||||||||||||||||||||||||||||||||||||||||||||||
Sbjct  19   AACAATATATTTAATTTTTGGAGCATGATCAGGTATATTAGGACTTAGTATAAGAATATT  78

Query  61   AATTCGTATAGAATTAGGACAACCTGGAACTTTAATTGGAAATGATCAGATTTATAATGT  120
            ||||||||||||||||||||||||||||||||||||||||||||||||||||||||||||
Sbjct  79   AATTCGTATAGAATTAGGACAACCTGGAACTTTAATTGGAAATGATCAGATTTATAATGT  138

Query  121  AATCGTAACAGCACATGCATTTATTATAATTTTCTTTATAGTTATACCAATCATAATCGG  180
            ||||||||||||||||||||||||||||||||| ||||||||||||||||||||||||||
Sbjct  139  AATCGTAACAGCACATGCATTTATTATAATTTTTTTTATAGTTATACCAATCATAATCGG  198
```

```
Query  181  AGGGTTCGGAAATTGATTAGTTCCTATTATATTAGGTGCCCCAGATATAGCATTTCCACG  240
            ||| || |||||||||||||| ||||| ||||| ||||||||||||||||||||||||||
Sbjct  199  AGGATTTGGAAATTGATTAGTCCCTATCATATTGGGTGCCCCAGATATAGCATTTCCACG  258

Query  241  AATAAATAATATAAGATTTTGACTTTTACCCCCCTCATTATTTTTATTGATTAACTCTTC  300
            |||||||||||||||||||||||||||||| || ||| |||||||||| |||||||||||
Sbjct  259  AATAAATAATATAAGATTTTGACTTTTACCTCCTTCACTATTTTTATTAATTAACTCTTC  318

Query  301  ATTAATTGAATCCGGAGCTGGAACAGGATGAACAGTTTATCCCCCTCTATCTTCAAATTT  360
            |||||||||||| |||||||| ||||||||||||||||| || |||||||||||||||||
Sbjct  319  ATTAATTGAATCTGGAGCTGGTACAGGATGAACAGTTTACCCTCCTCTATCTTCAAATTT  378

Query  361  ATCACATTATGGTCCATCAGTAGATTTAGCTATTTTTTCTCTTCACCTTGCTGGTGCTTC  420
            |||||||||||| || ||||||||||||||||||||||||||||| ||||||||||||||
Sbjct  379  ATCACATTATGGCCCTTCAGTAGATTTAGCTATTTTTTCTCTTCATCTTGCTGGTGCTTC  438

Query  421  TTCAATTTTAGGTGCAATTAACTTCATCACAACTATTGTAAATATACGATCAATTGGAAT  480
            |||||||||||||||||||||||| || ||||||||||||||| ||||||||||||||||
Sbjct  439  TTCAATTTTAGGTGCAATTAACTTTATTACAACTATTGTAAACATACGATCAATTGGAAT  498

Query  481  AACAATAGAACGAATACCATTATTTGTTTGATCTGTTTTAATTACAGCTATTTTACTACT  540
            |||||||||||| |||||||||||||||||||||||||||||||||||||||||| ||||
Sbjct  499  AACAATAGAACGTATACCATTATTTGTTTGATCTGTTTTAATTACAGCTATTTTATTACT  558

Query  541  TCTATCTTTACCTGTATTAGCAGGTGCCATTACAATATTATTAACTGATCGAAACTTTAA  600
            | ||||||||||||||||||||||||||||||||||||| ||||||||||||||||||||
Sbjct  559  TTTATCTTTACCTGTATTAGCAGGTGCCATTACAATATTGTTAACTGATCGAAACTTTAA  618

Query  601  CACATCATTTTTTGATCCTTCAGGAGGAGGAGATCCCATTTTATATCAACACTTATTT  658
             ||||||||||||||||||||||| ||||||||||| |||||||||||||| ||||||
Sbjct  619  TACATCATTTTTTGATCCTTCAGGGGGAGGAGATCCAATTTTATATCAACATTTATTT  676
```

>Rhipicephalus sanguineus sensu lato 'temperate lineage' isolate S1060 cytochrome c oxidase subunit I (COI) gene, partial cds; mitochondrial
Sequence ID: MF426006.1 Length: 705
Range 1: 28 to 685

Score:1061 bits(574), Expect:0.0,
Identities:630/658(96%), Gaps:0/658(0%), Strand: Plus/Plus

```
Query  1    AACAATATATTTAATTTTTGGAGCATGATCAGGTATATTAGGACTTAGTATAAGAATATT  60
            ||||||||||||||||||||||||||||||||||||||||||||||||||||||||||||
Sbjct  28   AACAATATATTTAATTTTTGGAGCATGATCAGGTATATTAGGACTTAGTATAAGAATATT  87

Query  61   AATTCGTATAGAATTAGGACAACCTGGAACTTTAATTGGAAATGATCAGATTTATAATGT  120
            ||||||||||||||||||||||||||||||||||||||||||||||||||||||||||||
Sbjct  88   AATTCGTATAGAATTAGGACAACCTGGAACTTTAATTGGAAATGATCAGATTTATAATGT  147

Query  121  AATCGTAACAGCACATGCATTTATTATAATTTTCTTTATAGTTATACCAATCATAATCGG  180
            ||||||||||||||||||||||||||||||||| ||||||||||||||||||||||||||
Sbjct  148  AATCGTAACAGCACATGCATTTATTATAATTTTTTTTATAGTTATACCAATCATAATCGG  207
```

```
Query  181  AGGGTTCGGAAATTGATTAGTTCCTATTATATTAGGTGCCCCAGATATAGCATTTCCACG  240
            ||| || |||||||||||||| ||||| ||||| ||||||||||||||||||||||||||
Sbjct  208  AGGATTTGGAAATTGATTAGTCCCTATCATATTGGGTGCCCCAGATATAGCATTTCCACG  267

Query  241  AATAAATAATATAAGATTTTGACTTTTACCCCCCTCATTATTTTTATTGATTAACTCTTC  300
            |||||||||||||||||||||||||||||| || ||| |||||||||| |||||||||||
Sbjct  268  AATAAATAATATAAGATTTTGACTTTTACCTCCTTCACTATTTTTATTAATTAACTCTTC  327

Query  301  ATTAATTGAATCCGGAGCTGGAACAGGATGAACAGTTTATCCCCCTCTATCTTCAAATTT  360
            |||||||||||| |||||||| ||||||||||||||||| || |||||||||||||||||
Sbjct  328  ATTAATTGAATCTGGAGCTGGTACAGGATGAACAGTTTACCCTCCTCTATCTTCAAATTT  387

Query  361  ATCACATTATGGTCCATCAGTAGATTTAGCTATTTTTTCTCTTCACCTTGCTGGTGCTTC  420
            ||||||||||||||| ||||||||||||||||||||||||||||| ||||||||||||||
Sbjct  388  ATCACATTATGGTCCTTCAGTAGATTTAGCTATTTTTTCTCTTCATCTTGCTGGTGCTTC  447

Query  421  TTCAATTTTAGGTGCAATTAACTTCATCACAACTATTGTAAATATACGATCAATTGGAAT  480
            |||||||||||||||||||||||| || |||||||||||||| ||||||| |||||||||
Sbjct  448  TTCAATTTTAGGTGCAATTAACTTTATTACAACTATTGTAAACATACGATTAATTGGAAT  507

Query  481  AACAATAGAACGAATACCATTATTTGTTTGATCTGTTTTAATTACAGCTATTTTACTACT  540
            |||||||||||| |||||||||||||||||||||||||||||||||||||||||| ||||
Sbjct  508  AACAATAGAACGTATACCATTATTTGTTTGATCTGTTTTAATTACAGCTATTTTATTACT  567

Query  541  TCTATCTTTACCTGTATTAGCAGGTGCCATTACAATATTATTAACTGATCGAAACTTTAA  600
            | ||||||||||||||||||||||||||||||||||||| ||||||||||||||||||||
Sbjct  568  TTTATCTTTACCTGTATTAGCAGGTGCCATTACAATATTGTTAACTGATCGAAACTTTAA  627

Query  601  CACATCATTTTTTGATCCTTCAGGAGGAGGAGATCCCATTTTATATCAACACTTATTT  658
             ||||||||||||||||||||||| ||||||||||| |||||||||||||| ||||||
Sbjct  628  TACATCATTTTTTGATCCTTCAGGGGGAGGAGATCCAATTTTATATCAACATTTATTT  685
```

```
>Rhipicephalus sanguineus haplotype III cytochrome oxidase subunit I (COI) gene,
partial cds; mitochondrial
Sequence ID: KU556745.1 Length: 713
Range 1: 27 to 684

Score:1061 bits(574), Expect:0.0,
Identities:630/658(96%),  Gaps:0/658(0%), Strand: Plus/Plus

Query  1    AACAATATATTTAATTTTTGGAGCATGATCAGGTATATTAGGACTTAGTATAAGAATATT  60
            ||||||||||||||||||||||||||||||||||||||||||||||||||||||||||||
Sbjct  27   AACAATATATTTAATTTTTGGAGCATGATCAGGTATATTAGGACTTAGTATAAGAATATT  86

Query  61   AATTCGTATAGAATTAGGACAACCTGGAACTTTAATTGGAAATGATCAGATTTATAATGT  120
            ||||||||||||||||||||||||||||||||||||||||||||||||||||||||||||
Sbjct  87   AATTCGTATAGAATTAGGACAACCTGGAACTTTAATTGGAAATGATCAGATTTATAATGT  146

Query  121  AATCGTAACAGCACATGCATTTATTATAATTTTCTTTATAGTTATACCAATCATAATCGG  180
            ||||||||||||||||||||||||||||||||| ||||||||||||||||||||||||||
```

```
Sbjct  147   AATCGTAACAGCACATGCATTTATTATAATTTTTTTATAGTTATACCAATCATAATCGG  206

Query  181   AGGGTTCGGAAATTGATTAGTTCCTATTATATTAGGTGCCCCAGATATAGCATTTCCACG  240
             ||| || |||||||||||||| ||||| ||||| ||||||||||||||||||||||||||
Sbjct  207   AGGATTTGGAAATTGATTAGTCCCTATCATATTGGGTGCCCCAGATATAGCATTTCCACG  266

Query  241   AATAAATAATATAAGATTTTGACTTTTACCCCCCTCATTATTTTTATTGATTAACTCTTC  300
             |||||||||||||||||||||||||||||| || ||| |||||||||| |||||||||||
Sbjct  267   AATAAATAATATAAGATTTTGACTTTTACCTCCTTCACTATTTTTATTAATTAACTCTTC  326

Query  301   ATTAATTGAATCCGGAGCTGGAACAGGATGAACAGTTTATCCCCCTCTATCTTCAAATTT  360
             |||||||||||| |||||||| ||||||||||||||||| || |||||||||||||||||
Sbjct  327   ATTAATTGAATCTGGAGCTGGTACAGGATGAACAGTTTACCCTCCTCTATCTTCAAATTT  386

Query  361   ATCACATTATGGTCCATCAGTAGATTTAGCTATTTTTTCTCTTCACCTTGCTGGTGCTTC  420
             |||||||||||| || ||||||||||||||||||||||||||||| ||||||||||||||
Sbjct  387   ATCACATTATGGCCCTTCAGTAGATTTAGCTATTTTTTCTCTTCATCTTGCTGGTGCTTC  446

Query  421   TTCAATTTTAGGTGCAATTAACTTCATCACAACTATTGTAAATATACGATCAATTGGAAT  480
             |||||||||||||||||||||||| || |||||||||||||| |||||||||||||||||
Sbjct  447   TTCAATTTTAGGTGCAATTAACTTTATTACAACTATTGTAAACATACGATCAATTGGAAT  506

Query  481   AACAATAGAACGAATACCATTATTTGTTTGATCTGTTTTAATTACAGCTATTTTACTACT  540
             |||||||||||| |||||||||||||||||||||||||||||||||||||||||| ||||
Sbjct  507   AACAATAGAACGTATACCATTATTTGTTTGATCTGTTTTAATTACAGCTATTTTATTACT  566

Query  541   TCTATCTTTACCTGTATTAGCAGGTGCCATTACAATATTATTAACTGATCGAAACTTTAA  600
             | ||||||||||||||||||||||||||||||||||||| ||||||||||||||||||||
Sbjct  567   TTTATCTTTACCTGTATTAGCAGGTGCCATTACAATATTGTTAACTGATCGAAACTTTAA  626

Query  601   CACATCATTTTTTGATCCTTCAGGAGGAGGAGATCCCATTTTATATCAACACTTATTT  658
              ||||||||||||||||||||||| ||||||||||| |||||||||||||| ||||||
Sbjct  627   TACATCATTTTTTGATCCTTCAGGGGGAGGAGATCCAATTTTATATCAACATTTATTT  684
```

>Rhipicephalus sanguineus isolate Arizona-1 cytochrome c oxidase subunit 1 (COI) gene, partial cds; mitochondrial
Sequence ID: MN593344.1 Length: 710
Range 1: 27 to 684

Score:1061 bits(574), Expect:0.0,
Identities:630/658(96%), Gaps:0/658(0%), Strand: Plus/Minus

```
Query  1     AACAATATATTTAATTTTTGGAGCATGATCAGGTATATTAGGACTTAGTATAAGAATATT  60
             ||||||||||||||||||||||||||||||||||||||||||||||||||||||||||||
Sbjct  684   AACAATATATTTAATTTTTGGAGCATGATCAGGTATATTAGGACTTAGTATAAGAATATT  625

Query  61    AATTCGTATAGAATTAGGACAACCTGGAACTTTAATTGGAAATGATCAGATTTATAATGT  120
             ||||||||||||||||||||||||||||||||||||||||||||||||||||||||||||
Sbjct  624   AATTCGTATAGAATTAGGACAACCTGGAACTTTAATTGGAAATGATCAGATTTATAATGT  565

Query  121   AATCGTAACAGCACATGCATTTATTATAATTTTCTTTATAGTTATACCAATCATAATCGG  180
```

```
            ||||||||||||||||||||||||||||||||| ||||||||||||||||||||||||||
Sbjct  564  AATCGTAACAGCACATGCATTTATTATAATTTTTTTATAGTTATACCAATCATAATCGG  505

Query  181  AGGGTTCGGAAATTGATTAGTTCCTATTATATTAGGTGCCCCAGATATAGCATTTCCACG  240
            ||| || |||||||||||||| ||||| ||||| ||||||||||||||||||||||||||
Sbjct  504  AGGATTTGGAAATTGATTAGTCCCTATCATATTGGGTGCCCCAGATATAGCATTTCCACG  445

Query  241  AATAAATAATATAAGATTTTGACTTTTACCCCCCTCATTATTTTTATTGATTAACTCTTC  300
            |||||||||||||||||||||||||||||| || ||| |||||||||| |||||||||||
Sbjct  444  AATAAATAATATAAGATTTTGACTTTTACCTCCTTCACTATTTTTATTAATTAACTCTTC  385

Query  301  ATTAATTGAATCCGGAGCTGGAACAGGATGAACAGTTTATCCCCCTCTATCTTCAAATTT  360
            |||||||||||| |||||||| ||||||||||||||||| || |||||||||||||||||
Sbjct  384  ATTAATTGAATCTGGAGCTGGTACAGGATGAACAGTTTACCCTCCTCTATCTTCAAATTT  325

Query  361  ATCACATTATGGTCCATCAGTAGATTTAGCTATTTTTTCTCTTCACCTTGCTGGTGCTTC  420
            |||||||||||| || ||||||||||||||||||||||||||||| ||||||||||||||
Sbjct  324  ATCACATTATGGCCCTTCAGTAGATTTAGCTATTTTTTCTCTTCATCTTGCTGGTGCTTC  265

Query  421  TTCAATTTTAGGTGCAATTAACTTCATCACAACTATTGTAAATATACGATCAATTGGAAT  480
            |||||||||||||||||||||||| || ||||||||||||||| ||||||||||||||||
Sbjct  264  TTCAATTTTAGGTGCAATTAACTTTATTACAACTATTGTAAACATACGATCAATTGGAAT  205

Query  481  AACAATAGAACGAATACCATTATTTGTTTGATCTGTTTTAATTACAGCTATTTTACTACT  540
            |||||||||||| |||||||||||||||||||||||||||||||||||||||||| ||||
Sbjct  204  AACAATAGAACGTATACCATTATTTGTTTGATCTGTTTTAATTACAGCTATTTTATTACT  145

Query  541  TCTATCTTTACCTGTATTAGCAGGTGCCATTACAATATTATTAACTGATCGAAACTTTAA  600
            | ||||||||||||||||||||||||||||||||||||| ||||||||||||||||||||
Sbjct  144  TTTATCTTTACCTGTATTAGCAGGTGCCATTACAATATTGTTAACTGATCGAAACTTTAA  85

Query  601  CACATCATTTTTTGATCCTTCAGGAGGAGGAGATCCCATTTTATATCAACACTTATTT  658
             ||||||||||||||||||||||| ||||||||||| |||||||||||||| ||||||
Sbjct  84   TACATCATTTTTTGATCCTTCAGGGGGAGGAGATCCAATTTTATATCAACATTTATTT  27
```

>Rhipicephalus sanguineus isolate Arizona-2 cytochrome c oxidase subunit 1 (COI) gene, partial cds; mitochondrial
Sequence ID: MN593343.1 Length: 710
Range 1: 27 to 684

Score:1061 bits(574), Expect:0.0,
Identities:630/658(96%), Gaps:0/658(0%), Strand: Plus/Minus

```
Query  1    AACAATATATTTAATTTTTGGAGCATGATCAGGTATATTAGGACTTAGTATAAGAATATT  60
            ||||||||||||||||||||||||||||||||||||||||||||||||||||||||||||
Sbjct  684  AACAATATATTTAATTTTTGGAGCATGATCAGGTATATTAGGACTTAGTATAAGAATATT  625

Query  61   AATTCGTATAGAATTAGGACAACCTGGAACTTTAATTGGAAATGATCAGATTTATAATGT  120
            ||||||||||||||||||||||||||||||||||||||||||||||||||||||||||||
Sbjct  624  AATTCGTATAGAATTAGGACAACCTGGAACTTTAATTGGAAATGATCAGATTTATAATGT  565
```

```
Query  121   AATCGTAACAGCACATGCATTTATTATAATTTTCTTTATAGTTATACCAATCATAATCGG  180
             ||||||||||||||||||||||||||||||||| ||||||||||||||||||||||||||
Sbjct  564   AATCGTAACAGCACATGCATTTATTATAATTTTTTTTATAGTTATACCAATCATAATCGG  505

Query  181   AGGGTTCGGAAATTGATTAGTTCCTATTATATTAGGTGCCCCAGATATAGCATTTCCACG  240
             ||| || |||||||||||||| ||||| ||||| ||||||||||||||||||||||||||
Sbjct  504   AGGATTTGGAAATTGATTAGTCCCTATCATATTGGGTGCCCCAGATATAGCATTTCCACG  445

Query  241   AATAAATAATATAAGATTTTGACTTTTACCCCCCTCATTATTTTTATTGATTAACTCTTC  300
             |||||||||||||||||||||||||||||| || ||| |||||||||| |||||||||||
Sbjct  444   AATAAATAATATAAGATTTTGACTTTTACCTCCTTCACTATTTTTATTAATTAACTCTTC  385

Query  301   ATTAATTGAATCCGGAGCTGGAACAGGATGAACAGTTTATCCCCCTCTATCTTCAAATTT  360
             |||||||||||| |||||||| ||||||||||||||||| || |||||||||||||||||
Sbjct  384   ATTAATTGAATCTGGAGCTGGTACAGGATGAACAGTTTACCCTCCTCTATCTTCAAATTT  325

Query  361   ATCACATTATGGTCCATCAGTAGATTTAGCTATTTTTTCTCTTCACCTTGCTGGTGCTTC  420
             |||||||||||| || ||||||||||||||||||||||||||||| ||||||||||||||
Sbjct  324   ATCACATTATGGCCCTTCAGTAGATTTAGCTATTTTTTCTCTTCATCTTGCTGGTGCTTC  265

Query  421   TTCAATTTTAGGTGCAATTAACTTCATCACAACTATTGTAAATATACGATCAATTGGAAT  480
             |||||||||||||||||||||||| || |||||||||||||| |||||||||||||||||
Sbjct  264   TTCAATTTTAGGTGCAATTAACTTTATTACAACTATTGTAAACATACGATCAATTGGAAT  205

Query  481   AACAATAGAACGAATACCATTATTTGTTTGATCTGTTTTAATTACAGCTATTTTACTACT  540
             |||||||||||| |||||||||||||||||||||||||||||||||||||||||| ||||
Sbjct  204   AACAATAGAACGTATACCATTATTTGTTTGATCTGTTTTAATTACAGCTATTTTATTACT  145

Query  541   TCTATCTTTACCTGTATTAGCAGGTGCCATTACAATATTATTAACTGATCGAAACTTTAA  600
             | ||||||||||||||||||||||||||||||||||||| ||||||||||||||||||||
Sbjct  144   TTTATCTTTACCTGTATTAGCAGGTGCCATTACAATATTGTTAACTGATCGAAACTTTAA  85

Query  601   CACATCATTTTTTGATCCTTCAGGAGGAGGAGATCCCATTTTATATCAACACTTATTT  658
              ||||||||||||||||||||||| ||||||||||| |||||||||||||| ||||||
Sbjct  84    TACATCATTTTTTGATCCTTCAGGGGGAGGAGATCCAATTTTATATCAACATTTATTT  27
```

>Rhipicephalus sanguineus isolate 11N cytochrome oxidase subunit I (COX1) gene, partial cds; mitochondrial
Sequence ID: MN585197.1 Length: 703
Range 1: 22 to 679

Score:1061 bits(574), Expect:0.0,
Identities:630/658(96%), Gaps:0/658(0%), Strand: Plus/Minus

```
Query  1     AACAATATATTTAATTTTTGGAGCATGATCAGGTATATTAGGACTTAGTATAAGAATATT  60
             ||||||||||||||||||||||||||||||||||||||||||||||||||||||||||||
Sbjct  679   AACAATATATTTAATTTTTGGAGCATGATCAGGTATATTAGGACTTAGTATAAGAATATT  620

Query  61    AATTCGTATAGAATTAGGACAACCTGGAACTTTAATTGGAAATGATCAGATTTATAATGT  120
             ||||||||||||||||||||||||||||||||||||||||||||||||||||||||||||
Sbjct  619   AATTCGTATAGAATTAGGACAACCTGGAACTTTAATTGGAAATGATCAGATTTATAATGT  560
```

```
Query  121   AATCGTAACAGCACATGCATTTATTATAATTTTCTTTATAGTTATACCAATCATAATCGG  180
             ||||||||||||||||||||||||||||||||| ||||||||||||||||||||||||||
Sbjct  559   AATCGTAACAGCACATGCATTTATTATAATTTTTTTTATAGTTATACCAATCATAATCGG  500

Query  181   AGGGTTCGGAAATTGATTAGTTCCTATTATATTAGGTGCCCCAGATATAGCATTTCCACG  240
             ||| || |||||||||||||| ||||| ||||| ||||||||||||||||||||||||||
Sbjct  499   AGGATTTGGAAATTGATTAGTCCCTATCATATTGGGTGCCCCAGATATAGCATTTCCACG  440

Query  241   AATAAATAATATAAGATTTTGACTTTTACCCCCCTCATTATTTTTATTGATTAACTCTTC  300
             |||||||||||||||||||||||||||||| || ||| |||||||||| |||||||||||
Sbjct  439   AATAAATAATATAAGATTTTGACTTTTACCTCCTTCACTATTTTTATTAATTAACTCTTC  380

Query  301   ATTAATTGAATCCGGAGCTGGAACAGGATGAACAGTTTATCCCCCTCTATCTTCAAATTT  360
             |||||||||||| |||||||| ||||||||||||||||| || |||||||||||||||||
Sbjct  379   ATTAATTGAATCTGGAGCTGGTACAGGATGAACAGTTTACCCTCCTCTATCTTCAAATTT  320

Query  361   ATCACATTATGGTCCATCAGTAGATTTAGCTATTTTTTCTCTTCACCTTGCTGGTGCTTC  420
             |||||||||||| || ||||||||||||||||||||||||||||| ||||||||||||||
Sbjct  319   ATCACATTATGGCCCTTCAGTAGATTTAGCTATTTTTTCTCTTCATCTTGCTGGTGCTTC  260

Query  421   TTCAATTTTAGGTGCAATTAACTTCATCACAACTATTGTAAATATACGATCAATTGGAAT  480
             |||||||||||||||||||||||| || |||||||||||||| |||||||||||||||||
Sbjct  259   TTCAATTTTAGGTGCAATTAACTTTATTACAACTATTGTAAACATACGATCAATTGGAAT  200

Query  481   AACAATAGAACGAATACCATTATTTGTTTGATCTGTTTTAATTACAGCTATTTTACTACT  540
             |||||||||||| |||||||||||||||||||||||||||||||||||||||||| ||||
Sbjct  199   AACAATAGAACGTATACCATTATTTGTTTGATCTGTTTTAATTACAGCTATTTTATTACT  140

Query  541   TCTATCTTTACCTGTATTAGCAGGTGCCATTACAATATTATTAACTGATCGAAACTTTAA  600
             | ||||||||||||||||||||||||||||||||||||| ||||||||||||||||||||
Sbjct  139   TTTATCTTTACCTGTATTAGCAGGTGCCATTACAATATTGTTAACTGATCGAAACTTTAA  80

Query  601   CACATCATTTTTTGATCCTTCAGGAGGAGGAGATCCCATTTTATATCAACACTTATTT  658
              ||||||||||||||||||||||| ||||||||||| |||||||||||||| ||||||
Sbjct  79    TACATCATTTTTTGATCCTTCAGGGGGAGGAGATCCAATTTTATATCAACATTTATTT  22
```

>Rhipicephalus sanguineus cytochrome c oxidase I (COI) gene, partial cds;
mitochondrial gene for mitochondrial product
Sequence ID: AF132839.1 Length: 793
Range 1: 42 to 699

Score:1061 bits(574), Expect:0.0,
Identities:630/658(96%), Gaps:0/658(0%), Strand: Plus/Plus

```
Query  1     AACAATATATTTAATTTTTGGAGCATGATCAGGTATATTAGGACTTAGTATAAGAATATT  60
             ||||||||||||||||||||||||||||||||||||||||||||||||||||||||||||
Sbjct  42    AACAATATATTTAATTTTTGGAGCATGATCAGGTATATTAGGACTTAGTATAAGAATATT  101

Query  61    AATTCGTATAGAATTAGGACAACCTGGAACTTTAATTGGAAATGATCAGATTTATAATGT  120
             ||||||||||||||||||||||||||||||||||||||||||||||||||||||||||||
```

```
Sbjct  102  AATTCGTATAGAATTAGGACAACCTGGAACTTTAATTGGAAATGATCAGATTTATAATGT  161

Query  121  AATCGTAACAGCACATGCATTTATTATAATTTTCTTTATAGTTATACCAATCATAATCGG  180
            ||||||||||||||||||||||||||||||||| ||||||||||||||||||||||||||
Sbjct  162  AATCGTAACAGCACATGCATTTATTATAATTTTTTTTATAGTTATACCAATCATAATCGG  221

Query  181  AGGGTTCGGAAATTGATTAGTTCCTATTATATTAGGTGCCCCAGATATAGCATTTCCACG  240
            ||| || |||||||||||||| ||||| ||||| ||||||||||||||||||||||||||
Sbjct  222  AGGATTTGGAAATTGATTAGTCCCTATCATATTGGGTGCCCCAGATATAGCATTTCCACG  281

Query  241  AATAAATAATATAAGATTTTGACTTTTACCCCCCTCATTATTTTTATTGATTAACTCTTC  300
            |||||||||||||||||||||||||||||| || ||| |||||||||| |||||||||||
Sbjct  282  AATAAATAATATAAGATTTTGACTTTTACCTCCTTCACTATTTTTATTAATTAACTCTTC  341

Query  301  ATTAATTGAATCCGGAGCTGGAACAGGATGAACAGTTTATCCCCCTCTATCTTCAAATTT  360
            |||||||||||| |||||||| ||||||||||||||||| || |||||||||||||||||
Sbjct  342  ATTAATTGAATCTGGAGCTGGTACAGGATGAACAGTTTACCCTCCTCTATCTTCAAATTT  401

Query  361  ATCACATTATGGTCCATCAGTAGATTTAGCTATTTTTTCTCTTCACCTTGCTGGTGCTTC  420
            |||||||||||| || |||||||||||||||||||||||||||||| |||||||||||||
Sbjct  402  ATCACATTATGGCCCTTCAGTAGATTTAGCTATTTTTTCTCTTCATCTTGCTGGTGCTTC  461

Query  421  TTCAATTTTAGGTGCAATTAACTTCATCACAACTATTGTAAATATACGATCAATTGGAAT  480
            |||||||||||||||||||||||| || |||||||||||||| |||||||||||||||||
Sbjct  462  TTCAATTTTAGGTGCAATTAACTTTATTACAACTATTGTAAACATACGATCAATTGGAAT  521

Query  481  AACAATAGAACGAATACCATTATTTGTTTGATCTGTTTTAATTACAGCTATTTTACTACT  540
            |||||||||||| ||||||||||||||||||||||||||||||||||||||||||| ||||
Sbjct  522  AACAATAGAACGTATACCATTATTTGTTTGATCTGTTTTAATTACAGCTATTTTATTACT  581

Query  541  TCTATCTTTACCTGTATTAGCAGGTGCCATTACAATATTATTAACTGATCGAAACTTTAA  600
            | ||||||||||||||||||||||||||||||||||||| ||||||||||||||||||||
Sbjct  582  TTTATCTTTACCTGTATTAGCAGGTGCCATTACAATATTGTTAACTGATCGAAACTTTAA  641

Query  601  CACATCATTTTTTGATCCTTCAGGAGGAGGAGATCCCATTTTATATCAACACTTATTT  658
             ||||||||||||||||||||||| ||||||||||| |||||||||||||| ||||||
Sbjct  642  TACATCATTTTTTGATCCTTCAGGGGGAGGAGATCCAATTTTATATCAACATTTATTT  699
```

```
>Rhipicephalus sanguineus sensu lato 'temperate lineage' isolate S56 cytochrome
c oxidase subunit I (COI) gene, partial cds; mitochondrial
Sequence ID: MF426017.1 Length: 680
Range 1: 11 to 668

Score:1055 bits(571), Expect:0.0,
Identities:629/658(96%),  Gaps:0/658(0%), Strand: Plus/Plus

Query  1    AACAATATATTTAATTTTTGGAGCATGATCAGGTATATTAGGACTTAGTATAAGAATATT  60
            ||||||||||||||||||||||||||||||||||||||||||||||||||||||||||||
Sbjct  11   AACAATATATTTAATTTTTGGAGCATGATCAGGTATATTAGGACTTAGTATAAGAATATT  70

Query  61   AATTCGTATAGAATTAGGACAACCTGGAACTTTAATTGGAAATGATCAGATTTATAATGT  120
```

```
            ||||||||||||||||||||||||||||||||||||||||||||||||||||||||||||
Sbjct  71   AATTCGTATAGAATTAGGACAACCTGGAACTTTAATTGGAAATGATCAGATTTATAATGT  130

Query  121  AATCGTAACAGCACATGCATTTATTATAATTTTCTTTATAGTTATACCAATCATAATCGG  180
            ||||||||||||||||||||||||||||||||| ||||||||||||||||||||||||||
Sbjct  131  AATCGTAACAGCACATGCATTTATTATAATTTTTTTTATAGTTATACCAATCATAATCGG  190

Query  181  AGGGTTCGGAAATTGATTAGTTCCTATTATATTAGGTGCCCCAGATATAGCATTTCCACG  240
            ||| || |||||||||||||| ||||| ||||| ||||||||||||||||||||||||||
Sbjct  191  AGGATTTGGAAATTGATTAGTCCCTATCATATTGGGTGCCCCAGATATAGCATTTCCACG  250

Query  241  AATAAATAATATAAGATTTTGACTTTTACCCCCCTCATTATTTTTATTGATTAACTCTTC  300
            |||||||||||||||||||||||||||||| || ||| |||||||||| |||||||||||
Sbjct  251  AATAAATAATATAAGATTTTGACTTTTACCTCCTTCACTATTTTTATTAATTAACTCTTC  310

Query  301  ATTAATTGAATCCGGAGCTGGAACAGGATGAACAGTTTATCCCCCTCTATCTTCAAATTT  360
            |||||||||||| |||||||| |||||||||||||||||||| |||||||||||||||||
Sbjct  311  ATTAATTGAATCTGGAGCTGGTACAGGATGAACAGTTTATCCTCCTCTATCTTCAAATTT  370

Query  361  ATCACATTATGGTCCATCAGTAGATTTAGCTATTTTTTCTCTTCACCTTGCTGGTGCTTC  420
            |||||||||||| || ||||||||||||||||| ||||||||||| ||||||||||||||
Sbjct  371  ATCACATTATGGCCCTTCAGTAGATTTAGCTATCTTTTCTCTTCATCTTGCTGGTGCTTC  430

Query  421  TTCAATTTTAGGTGCAATTAACTTCATCACAACTATTGTAAATATACGATCAATTGGAAT  480
            |||||||||||| ||||||||||| || |||||||||||||| |||||||||||||||||
Sbjct  431  TTCAATTTTAGGAGCAATTAACTTTATTACAACTATTGTAAACATACGATCAATTGGAAT  490

Query  481  AACAATAGAACGAATACCATTATTTGTTTGATCTGTTTTAATTACAGCTATTTTACTACT  540
            |||||||||||| ||||||||||||||||||||||||||||||||||||||||||| ||||
Sbjct  491  AACAATAGAACGTATACCATTATTTGTTTGATCTGTTTTAATTACAGCTATTTTATTACT  550

Query  541  TCTATCTTTACCTGTATTAGCAGGTGCCATTACAATATTATTAACTGATCGAAACTTTAA  600
            | ||||||||||||||||||||||||||||||||||||| ||||||||||||||||||||
Sbjct  551  TTTATCTTTACCTGTATTAGCAGGTGCCATTACAATATTGTTAACTGATCGAAACTTTAA  610

Query  601  CACATCATTTTTTGATCCTTCAGGAGGAGGAGATCCCATTTTATATCAACACTTATTT  658
             ||||||||||||||||||||||| ||||||||||| |||||||||||||| ||||||
Sbjct  611  TACATCATTTTTTGATCCTTCAGGGGGAGGAGATCCAATTTTATATCAACATTTATTT  668
```

>Rhipicephalus sanguineus sensu lato 'temperate lineage' isolate S32 cytochrome c oxidase subunit I (COI) gene, partial cds; mitochondrial
Sequence ID: MF426008.1 Length: 690
Range 1: 14 to 671

Score:1055 bits(571), Expect:0.0,
Identities:629/658(96%), Gaps:0/658(0%), Strand: Plus/Plus

```
Query  1    AACAATATATTTAATTTTTGGAGCATGATCAGGTATATTAGGACTTAGTATAAGAATATT  60
            ||||||||||||||||||||||||||||||||||||||||||||||||||||||||||||
Sbjct  14   AACAATATATTTAATTTTTGGAGCATGATCAGGTATATTAGGACTTAGTATAAGAATATT  73
```

```
Query  61    AATTCGTATAGAATTAGGACAACCTGGAACTTTAATTGGAAATGATCAGATTTATAATGT  120
             ||||||||||||||||||||||||||||||||||||||||||||||||||||||||||||
Sbjct  74    AATTCGTATAGAATTAGGACAACCTGGAACTTTAATTGGAAATGATCAGATTTATAATGT  133

Query  121   AATCGTAACAGCACATGCATTTATTATAATTTTCTTTATAGTTATACCAATCATAATCGG  180
             ||||||||||||||||||||||||||||||||| ||||||||||||||||||||||||||
Sbjct  134   AATCGTAACAGCACATGCATTTATTATAATTTTTTTTATAGTTATACCAATCATAATCGG  193

Query  181   AGGGTTCGGAAATTGATTAGTTCCTATTATATTAGGTGCCCCAGATATAGCATTTCCACG  240
             ||| || |||||||||||||| ||||| ||||| ||||||||||||||||||||||||||
Sbjct  194   AGGATTTGGAAATTGATTAGTCCCTATCATATTGGGTGCCCCAGATATAGCATTTCCACG  253

Query  241   AATAAATAATATAAGATTTTGACTTTTACCCCCCTCATTATTTTTATTGATTAACTCTTC  300
             |||||||||||||||||||||||||||||| || ||| |||||||||| |||||||||||
Sbjct  254   AATAAATAATATAAGATTTTGACTTTTACCTCCTTCACTATTTTTATTAATTAACTCTTC  313

Query  301   ATTAATTGAATCCGGAGCTGGAACAGGATGAACAGTTTATCCCCCTCTATCTTCAAATTT  360
             |||||||||||| |||||||| ||||||||||||||||| || |||||||||||||||||
Sbjct  314   ATTAATTGAATCTGGAGCTGGTACAGGATGAACAGTTTACCCTCCTCTATCTTCAAATTT  373

Query  361   ATCACATTATGGTCCATCAGTAGATTTAGCTATTTTTTCTCTTCACCTTGCTGGTGCTTC  420
             |||||||||||| || ||||||||||||||||| ||||||||||| ||||||||||||||
Sbjct  374   ATCACATTATGGCCCTTCAGTAGATTTAGCTATCTTTTCTCTTCATCTTGCTGGTGCTTC  433

Query  421   TTCAATTTTAGGTGCAATTAACTTCATCACAACTATTGTAAATATACGATCAATTGGAAT  480
             |||||||||||||||||||||||| || |||||||||||||| |||||||||||||||||
Sbjct  434   TTCAATTTTAGGTGCAATTAACTTTATTACAACTATTGTAAACATACGATCAATTGGAAT  493

Query  481   AACAATAGAACGAATACCATTATTTGTTTGATCTGTTTTAATTACAGCTATTTTACTACT  540
             |||||||||||| |||||||||||||||||||||||||||||||||||||||||| ||||
Sbjct  494   AACAATAGAACGTATACCATTATTTGTTTGATCTGTTTTAATTACAGCTATTTTATTACT  553

Query  541   TCTATCTTTACCTGTATTAGCAGGTGCCATTACAATATTATTAACTGATCGAAACTTTAA  600
             | ||||||||||||||||||||||||||||||||||||| ||||||||||||||||||||
Sbjct  554   TTTATCTTTACCTGTATTAGCAGGTGCCATTACAATATTGTTAACTGATCGAAACTTTAA  613

Query  601   CACATCATTTTTTGATCCTTCAGGAGGAGGAGATCCCATTTTATATCAACACTTATTT  658
              ||||||||||||||||||||||| ||||||||||| |||||||||||||| ||||||
Sbjct  614   TACATCATTTTTTGATCCTTCAGGGGGAGGAGATCCAATTTTATATCAACATTTATTT  671
```

>Rhipicephalus sanguineus sensu lato 'temperate lineage' isolate CR1570 cytochrome c oxidase subunit I (COI) gene, partial cds; mitochondrial
Sequence ID: MF426001.1 Length: 684
Range 1: 14 to 671

Score:1055 bits(571), Expect:0.0,
Identities:629/658(96%), Gaps:0/658(0%), Strand: Plus/Plus

```
Query  1     AACAATATATTTAATTTTTGGAGCATGATCAGGTATATTAGGACTTAGTATAAGAATATT  60
             ||||||||||||||||||||||||||||||||||||||||||||||||||||||||||||
Sbjct  14    AACAATATATTTAATTTTTGGAGCATGATCAGGTATATTAGGACTTAGTATAAGAATATT  73
```

```
Query  61    AATTCGTATAGAATTAGGACAACCTGGAACTTTAATTGGAAATGATCAGATTTATAATGT  120
             ||||||||||||||||||||||||||||||||||||||||||||||||||||||||||||
Sbjct  74    AATTCGTATAGAATTAGGACAACCTGGAACTTTAATTGGAAATGATCAGATTTATAATGT  133

Query  121   AATCGTAACAGCACATGCATTTATTATAATTTTCTTTATAGTTATACCAATCATAATCGG  180
             ||||||||||||||||||||||||||||||||| ||||||||||||||||||||||||||
Sbjct  134   AATCGTAACAGCACATGCATTTATTATAATTTTTTTTATAGTTATACCAATCATAATCGG  193

Query  181   AGGGTTCGGAAATTGATTAGTTCCTATTATATTAGGTGCCCCAGATATAGCATTTCCACG  240
             ||| || |||||||||||||| ||||| ||||| ||||||||||||||||||||||||||
Sbjct  194   AGGATTTGGAAATTGATTAGTCCCTATCATATTGGGTGCCCCAGATATAGCATTTCCACG  253

Query  241   AATAAATAATATAAGATTTTGACTTTTACCCCCCTCATTATTTTTATTGATTAACTCTTC  300
             |||||||||||||||||||||||||||||| || ||| |||||||||| |||||||||||
Sbjct  254   AATAAATAATATAAGATTTTGACTTTTACCTCCTTCACTATTTTTATTAATTAACTCTTC  313

Query  301   ATTAATTGAATCCGGAGCTGGAACAGGATGAACAGTTTATCCCCCTCTATCTTCAAATTT  360
             |||||||||||| |||||||| ||||||||||||||||| || |||||||||||||||||
Sbjct  314   ATTAATTGAATCTGGAGCTGGTACAGGATGAACAGTTTACCCTCCTCTATCTTCAAATTT  373

Query  361   ATCACATTATGGTCCATCAGTAGATTTAGCTATTTTTTCTCTTCACCTTGCTGGTGCTTC  420
             |||||||||||| || ||||||||||||||||| ||||||||||| ||||||||||||||
Sbjct  374   ATCACATTATGGCCCTTCAGTAGATTTAGCTATCTTTTCTCTTCATCTTGCTGGTGCTTC  433

Query  421   TTCAATTTTAGGTGCAATTAACTTCATCACAACTATTGTAAATATACGATCAATTGGAAT  480
             |||||||||||||||||||||||| || |||||||||||||| |||||||||||||||||
Sbjct  434   TTCAATTTTAGGTGCAATTAACTTTATTACAACTATTGTAAACATACGATCAATTGGAAT  493

Query  481   AACAATAGAACGAATACCATTATTTGTTTGATCTGTTTTAATTACAGCTATTTTACTACT  540
             |||||||||||| |||||||||||||||||||||||||||||||||||||||||| ||||
Sbjct  494   AACAATAGAACGTATACCATTATTTGTTTGATCTGTTTTAATTACAGCTATTTTATTACT  553

Query  541   TCTATCTTTACCTGTATTAGCAGGTGCCATTACAATATTATTAACTGATCGAAACTTTAA  600
             | ||||||||||||||||||||||||||||||||||||| ||||||||||||||||||||
Sbjct  554   TTTATCTTTACCTGTATTAGCAGGTGCCATTACAATATTGTTAACTGATCGAAACTTTAA  613

Query  601   CACATCATTTTTTGATCCTTCAGGAGGAGGAGATCCCATTTTATATCAACACTTATTT  658
              ||||||||||||||||||||||| ||||||||||| |||||||||||||| ||||||
Sbjct  614   TACATCATTTTTTGATCCTTCAGGGGGAGGAGATCCAATTTTATATCAACATTTATTT  671
```

>Rhipicephalus sanguineus sensu lato 'temperate lineage' isolate CR1553
cytochrome c oxidase subunit I (COI) gene, partial cds; mitochondrial
Sequence ID: MF425998.1 Length: 689
Range 1: 20 to 677

Score:1055 bits(571), Expect:0.0,
Identities:629/658(96%), Gaps:0/658(0%), Strand: Plus/Plus

```
Query  1     AACAATATATTTAATTTTTGGAGCATGATCAGGTATATTAGGACTTAGTATAAGAATATT  60
             ||||||||||||||||||||||||||||||||||||||||||||||||||||||||||||
```

```
Sbjct  20   AACAATATATTTAATTTTTGGAGCATGATCAGGTATATTAGGACTTAGTATAAGAATATT  79

Query  61   AATTCGTATAGAATTAGGACAACCTGGAACTTTAATTGGAAATGATCAGATTTATAATGT  120
            ||||||||||||||||||||||||||||||||||||||||||||||||||||||||||||
Sbjct  80   AATTCGTATAGAATTAGGACAACCTGGAACTTTAATTGGAAATGATCAGATTTATAATGT  139

Query  121  AATCGTAACAGCACATGCATTTATTATAATTTTCTTTATAGTTATACCAATCATAATCGG  180
            ||||||||||||||||||||||||||||||||| ||||||||||||||||||||||||||
Sbjct  140  AATCGTAACAGCACATGCATTTATTATAATTTTTTTTATAGTTATACCAATCATAATCGG  199

Query  181  AGGGTTCGGAAATTGATTAGTTCCTATTATATTAGGTGCCCCAGATATAGCATTTCCACG  240
            ||| || |||||||||||||| ||||| ||||||||||||||||||||||||||||||||
Sbjct  200  AGGATTTGGAAATTGATTAGTCCCTATCATATTAGGTGCCCCAGATATAGCATTTCCACG  259

Query  241  AATAAATAATATAAGATTTTGACTTTTACCCCCCTCATTATTTTTATTGATTAACTCTTC  300
            |||||||||||||||||||||||||||||| || ||| |||||||||| |||||||||||
Sbjct  260  AATAAATAATATAAGATTTTGACTTTTACCTCCTTCACTATTTTTATTAATTAACTCTTC  319

Query  301  ATTAATTGAATCCGGAGCTGGAACAGGATGAACAGTTTATCCCCCTCTATCTTCAAATTT  360
            |||||||||||| |||||||| ||||||||||||||||| || |||||||||||||||||
Sbjct  320  ATTAATTGAATCTGGAGCTGGTACAGGATGAACAGTTTACCCTCCTCTATCTTCAAATTT  379

Query  361  ATCACATTATGGTCCATCAGTAGATTTAGCTATTTTTTCTCTTCACCTTGCTGGTGCTTC  420
            |||||||||||| || ||||||||||||||||| ||||||||||| ||||||||||||||
Sbjct  380  ATCACATTATGGCCCTTCAGTAGATTTAGCTATCTTTTCTCTTCATCTTGCTGGTGCTTC  439

Query  421  TTCAATTTTAGGTGCAATTAACTTCATCACAACTATTGTAAATATACGATCAATTGGAAT  480
            |||||||||||||||||||||||| || |||||||||||||| |||||||||||||||||
Sbjct  440  TTCAATTTTAGGTGCAATTAACTTTATTACAACTATTGTAAACATACGATCAATTGGAAT  499

Query  481  AACAATAGAACGAATACCATTATTTGTTTGATCTGTTTTAATTACAGCTATTTTACTACT  540
            |||||||||||| |||||||||||||||||||||||||||||||||||||||||| ||||
Sbjct  500  AACAATAGAACGTATACCATTATTTGTTTGATCTGTTTTAATTACAGCTATTTTATTACT  559

Query  541  TCTATCTTTACCTGTATTAGCAGGTGCCATTACAATATTATTAACTGATCGAAACTTTAA  600
              ||||||||||||||||||||||||||||||||||||| ||||||||||||||||||||
Sbjct  560  CTTATCTTTACCTGTATTAGCAGGTGCCATTACAATATTGTTAACTGATCGAAACTTTAA  619

Query  601  CACATCATTTTTTGATCCTTCAGGAGGAGGAGATCCCATTTTATATCAACACTTATTT  658
             ||||||||||||||||||||||| ||||||||||| |||||||||||||| ||||||
Sbjct  620  TACATCATTTTTTGATCCTTCAGGGGGAGGAGATCCAATTTTATATCAACATTTATTT  677
```

>Rhipicephalus sanguineus sensu lato 'temperate lineage' isolate CR1551
cytochrome c oxidase subunit I (COI) gene, partial cds; mitochondrial
Sequence ID: MF425997.1 Length: 695
Range 1: 22 to 679

Score:1055 bits(571), Expect:0.0,
Identities:629/658(96%), Gaps:0/658(0%), Strand: Plus/Plus

```
Query  1    AACAATATATTTAATTTTTGGAGCATGATCAGGTATATTAGGACTTAGTATAAGAATATT  60
```

```
            ||||||||||||||||||||||||||||||||||||||||||||||||||||||||||||
Sbjct  22   AACAATATATTTAATTTTTGGAGCATGATCAGGTATATTAGGACTTAGTATAAGAATATT  81

Query  61   AATTCGTATAGAATTAGGACAACCTGGAACTTTAATTGGAAATGATCAGATTTATAATGT  120
            ||||||||||||||||||||||||||||||||||||||||||||||||||||||||||||
Sbjct  82   AATTCGTATAGAATTAGGACAACCTGGAACTTTAATTGGAAATGATCAGATTTATAATGT  141

Query  121  AATCGTAACAGCACATGCATTTATTATAATTTTCTTTATAGTTATACCAATCATAATCGG  180
            ||||||||||||||||||||||||||||||||| ||||||||||||||||||||||||||
Sbjct  142  AATCGTAACAGCACATGCATTTATTATAATTTTTTTTATAGTTATACCAATCATAATCGG  201

Query  181  AGGGTTCGGAAATTGATTAGTTCCTATTATATTAGGTGCCCCAGATATAGCATTTCCACG  240
            ||| || |||||||||||||| ||||| ||||| ||||||||||||||||||||||||||
Sbjct  202  AGGATTTGGAAATTGATTAGTCCCTATCATATTGGGTGCCCCAGATATAGCATTTCCACG  261

Query  241  AATAAATAATATAAGATTTTGACTTTTACCCCCCTCATTATTTTTATTGATTAACTCTTC  300
            |||||||||||||||||||||||||||||| || ||| |||||||||| |||||||||||
Sbjct  262  AATAAATAATATAAGATTTTGACTTTTACCTCCTTCACTATTTTTATTAATTAACTCTTC  321

Query  301  ATTAATTGAATCCGGAGCTGGAACAGGATGAACAGTTTATCCCCCTCTATCTTCAAATTT  360
            |||||||||||| |||||||| ||||||||||||||||| || |||||||||||||||||
Sbjct  322  ATTAATTGAATCTGGAGCTGGTACAGGATGAACAGTTTACCCTCCTCTATCTTCAAATTT  381

Query  361  ATCACATTATGGTCCATCAGTAGATTTAGCTATTTTTTCTCTTCACCTTGCTGGTGCTTC  420
            |||||||||||| || ||||||||||||||||| ||||||||||| ||||||||||||||
Sbjct  382  ATCACATTATGGCCCTTCAGTAGATTTAGCTATCTTTTCTCTTCATCTTGCTGGTGCTTC  441

Query  421  TTCAATTTTAGGTGCAATTAACTTCATCACAACTATTGTAAATATACGATCAATTGGAAT  480
            |||||||||||||||||||||||| || |||||||||||||| |||||||||||||||||
Sbjct  442  TTCAATTTTAGGTGCAATTAACTTTATTACAACTATTGTAAACATACGATCAATTGGAAT  501

Query  481  AACAATAGAACGAATACCATTATTTGTTTGATCTGTTTTAATTACAGCTATTTTACTACT  540
            |||||||||||| |||||||||||||||||||||||||||||||||||||||||| ||||
Sbjct  502  AACAATAGAACGTATACCATTATTTGTTTGATCTGTTTTAATTACAGCTATTTTATTACT  561

Query  541  TCTATCTTTACCTGTATTAGCAGGTGCCATTACAATATTATTAACTGATCGAAACTTTAA  600
            | ||||||||||||||||||||||||||||||||||||| ||||||||||||||||||||
Sbjct  562  TTTATCTTTACCTGTATTAGCAGGTGCCATTACAATATTGTTAACTGATCGAAACTTTAA  621

Query  601  CACATCATTTTTTGATCCTTCAGGAGGAGGAGATCCCATTTTATATCAACACTTATTT  658
             ||||||||||||||||||||||| ||||||||||| |||||||||||||| ||||||
Sbjct  622  TACATCATTTTTTGATCCTTCAGGGGGAGGAGATCCAATTTTATATCAACATTTATTT  679
```

>Rhipicephalus sanguineus haplotype IV cytochrome oxidase subunit I (COI) gene, partial cds; mitochondrial
Sequence ID: KU556746.1 Length: 771
Range 1: 90 to 747

Score:1055 bits(571), Expect:0.0,
Identities:629/658(96%), Gaps:0/658(0%), Strand: Plus/Plus

```
Query  1    AACAATATATTTAATTTTTGGAGCATGATCAGGTATATTAGGACTTAGTATAAGAATATT  60
            ||||||||||||||||||||||||||||||||||||||||||||||||||||||||||||
Sbjct  90   AACAATATATTTAATTTTTGGAGCATGATCAGGTATATTAGGACTTAGTATAAGAATATT  149

Query  61   AATTCGTATAGAATTAGGACAACCTGGAACTTTAATTGGAAATGATCAGATTTATAATGT  120
            ||||||||||||||||||||||||||||||||||||||||||||||||||||||||||||
Sbjct  150  AATTCGTATAGAATTAGGACAACCTGGAACTTTAATTGGAAATGATCAGATTTATAATGT  209

Query  121  AATCGTAACAGCACATGCATTTATTATAATTTTCTTTATAGTTATACCAATCATAATCGG  180
            ||||||||||||||||||||||||||||||||| ||||||||||||||||||||||||||
Sbjct  210  AATCGTAACAGCACATGCATTTATTATAATTTTTTTTATAGTTATACCAATCATAATCGG  269

Query  181  AGGGTTCGGAAATTGATTAGTTCCTATTATATTAGGTGCCCCAGATATAGCATTTCCACG  240
            ||| || |||||||||||||| ||||| ||||| ||||||||||||||||||||||||||
Sbjct  270  AGGATTTGGAAATTGATTAGTCCCTATCATATTGGGTGCCCCAGATATAGCATTTCCACG  329

Query  241  AATAAATAATATAAGATTTTGACTTTTACCCCCCTCATTATTTTTATTGATTAACTCTTC  300
            |||||||||||||||||||||||||||||| || ||| |||||||||| |||||||||||
Sbjct  330  AATAAATAATATAAGATTTTGACTTTTACCTCCTTCACTATTTTTATTAATTAACTCTTC  389

Query  301  ATTAATTGAATCCGGAGCTGGAACAGGATGAACAGTTTATCCCCCTCTATCTTCAAATTT  360
            |||||||||||| |||||||| |||||||||||||||||||| |||||||||||||||||
Sbjct  390  ATTAATTGAATCTGGAGCTGGTACAGGATGAACAGTTTATCCTCCTCTATCTTCAAATTT  449

Query  361  ATCACATTATGGTCCATCAGTAGATTTAGCTATTTTTTCTCTTCACCTTGCTGGTGCTTC  420
            |||||||||||| || ||||||||||||||||| ||||||||||| ||||||||||||||
Sbjct  450  ATCACATTATGGCCCTTCAGTAGATTTAGCTATCTTTTCTCTTCATCTTGCTGGTGCTTC  509

Query  421  TTCAATTTTAGGTGCAATTAACTTCATCACAACTATTGTAAATATACGATCAATTGGAAT  480
            |||||||||||| ||||||||||| || |||||||||||||| |||||||||||||||||
Sbjct  510  TTCAATTTTAGGAGCAATTAACTTTATTACAACTATTGTAAACATACGATCAATTGGAAT  569

Query  481  AACAATAGAACGAATACCATTATTTGTTTGATCTGTTTTAATTACAGCTATTTTACTACT  540
            |||||||||||| |||||||||||||||||||||||||||||||||||||||||| ||||
Sbjct  570  AACAATAGAACGTATACCATTATTTGTTTGATCTGTTTTAATTACAGCTATTTTATTACT  629

Query  541  TCTATCTTTACCTGTATTAGCAGGTGCCATTACAATATTATTAACTGATCGAAACTTTAA  600
            | ||||||||||||||||||||||||||||||||||||| ||||||||||||||||||||
Sbjct  630  TTTATCTTTACCTGTATTAGCAGGTGCCATTACAATATTGTTAACTGATCGAAACTTTAA  689

Query  601  CACATCATTTTTTGATCCTTCAGGAGGAGGAGATCCCATTTTATATCAACACTTATTT  658
             ||||||||||||||||||||||| ||||||||||| |||||||||||||| ||||||
Sbjct  690  TACATCATTTTTTGATCCTTCAGGGGGAGGAGATCCAATTTTATATCAACATTTATTT  747
```

Range 2: 30 to 64

Score:58.4 bits(31), Expect:0.001,
Identities:34/35(97%), Gaps:1/35(2%), Strand: Plus/Plus

```
Query  1   AACAATATATTTAATTTTTGGAGCATGATCA-GGT  34
           ||||||||||||||||||||||||||||||| |||
```

```
Sbjct  30  AACAATATATTTAATTTTTGGAGCATGATCATGGT  64
```

>Rhipicephalus sanguineus voucher CROBB813 cytochrome oxidase subunit 1 (COI) gene, partial cds; mitochondrial
Sequence ID: MZ305546.1 Length: 658
Range 1: 1 to 658

Score:1055 bits(571), Expect:0.0,
Identities:629/658(96%), Gaps:0/658(0%), Strand: Plus/Plus

```
Query  1    AACAATATATTTAATTTTTGGAGCATGATCAGGTATATTAGGACTTAGTATAAGAATATT  60
            ||||||||||||||||||||||||||||||||||||||||||||||||||||||||||||
Sbjct  1    AACAATATATTTAATTTTTGGAGCATGATCAGGTATATTAGGACTTAGTATAAGAATATT  60

Query  61   AATTCGTATAGAATTAGGACAACCTGGAACTTTAATTGGAAATGATCAGATTTATAATGT  120
            |||||||||||| |||||||||||||||||||||||||||||||||||||||||||||||
Sbjct  61   AATTCGTATAGAGTTAGGACAACCTGGAACTTTAATTGGAAATGATCAGATTTATAATGT  120

Query  121  AATCGTAACAGCACATGCATTTATTATAATTTCTTTATAGTTATACCAATCATAATCGG  180
            |||||||||||||||||||||||||||||||| |||||||||||||||||||||||||||
Sbjct  121  AATCGTAACAGCACATGCATTTATTATAATTTTTTTATAGTTATACCAATCATAATCGG  180

Query  181  AGGGTTCGGAAATTGATTAGTTCCTATTATATTAGGTGCCCCAGATATAGCATTTCCACG  240
            ||| || |||||||||||||| ||||| ||||| ||||||||||||||||||||||||||
Sbjct  181  AGGATTTGGAAATTGATTAGTCCCTATCATATTGGGTGCCCCAGATATAGCATTTCCACG  240

Query  241  AATAAATAATATAAGATTTTGACTTTTACCCCCCTCATTATTTTTATTGATTAACTCTTC  300
            |||||||||||||||||||||||||||||| || ||| |||||||||| |||||||||||
Sbjct  241  AATAAATAATATAAGATTTTGACTTTTACCTCCTTCACTATTTTTATTAATTAACTCTTC  300

Query  301  ATTAATTGAATCCGGAGCTGGAACAGGATGAACAGTTTATCCCCCTCTATCTTCAAATTT  360
            |||||||||||| |||||||| ||||||||||||||||| || |||||||||||||||||
Sbjct  301  ATTAATTGAATCTGGAGCTGGTACAGGATGAACAGTTTACCCTCCTCTATCTTCAAATTT  360

Query  361  ATCACATTATGGTCCATCAGTAGATTTAGCTATTTTTTCTCTTCACCTTGCTGGTGCTTC  420
            |||||||||||| || ||||||||||||||||||||||||||||| ||||||||||||||
Sbjct  361  ATCACATTATGGCCCTTCAGTAGATTTAGCTATTTTTTCTCTTCATCTTGCTGGTGCTTC  420

Query  421  TTCAATTTTAGGTGCAATTAACTTCATCACAACTATTGTAAATATACGATCAATTGGAAT  480
            |||||||||||||||||||||||| || |||||||||||||| |||||||||||||||||
Sbjct  421  TTCAATTTTAGGTGCAATTAACTTTATTACAACTATTGTAAACATACGATCAATTGGAAT  480

Query  481  AACAATAGAACGAATACCATTATTTGTTTGATCTGTTTTAATTACAGCTATTTTACTACT  540
            |||||||||||| |||||||||||||||||||||||||||||||||||||||||| ||||
Sbjct  481  AACAATAGAACGTATACCATTATTTGTTTGATCTGTTTTAATTACAGCTATTTTATTACT  540

Query  541  TCTATCTTTACCTGTATTAGCAGGTGCCATTACAATATTATTAACTGATCGAAACTTTAA  600
            | ||||||||||||||||||||||||||||||||||||| ||||||||||||||||||||
Sbjct  541  TTTATCTTTACCTGTATTAGCAGGTGCCATTACAATATTGTTAACTGATCGAAACTTTAA  600

Query  601  CACATCATTTTTTGATCCTTCAGGAGGAGGAGATCCCATTTTATATCAACACTTATTT  658
```

```
                |||||||||||||||||||||||| ||||||||||| |||||||||||||| ||||||
Sbjct  601  TACATCATTTTTTGATCCTTCAGGGGGAGGAGATCCAATTTTATATCAACATTTATTT  658
```

>Rhipicephalus sanguineus voucher CROBB810 cytochrome oxidase subunit 1 (COI) gene, partial cds; mitochondrial
Sequence ID: MZ305545.1 Length: 658
Range 1: 1 to 658

Score:1055 bits(571), Expect:0.0,
Identities:629/658(96%), Gaps:0/658(0%), Strand: Plus/Plus

```
Query  1    AACAATATATTTAATTTTTGGAGCATGATCAGGTATATTAGGACTTAGTATAAGAATATT  60
            ||||||||||||||||||||||||||||||||||||||||||||||||||||||||||||
Sbjct  1    AACAATATATTTAATTTTTGGAGCATGATCAGGTATATTAGGACTTAGTATAAGAATATT  60

Query  61   AATTCGTATAGAATTAGGACAACCTGGAACTTTAATTGGAAATGATCAGATTTATAATGT  120
            ||||||||||||||||||||||||||||||||||||||||||||||||||||||||||||
Sbjct  61   AATTCGTATAGAATTAGGACAACCTGGAACTTTAATTGGAAATGATCAGATTTATAATGT  120

Query  121  AATCGTAACAGCACATGCATTTATTATAATTTTCTTTATAGTTATACCAATCATAATCGG  180
            ||||||||||||||||||||||||||||||||| ||||||||||||||||||||||||||
Sbjct  121  AATCGTAACAGCACATGCATTTATTATAATTTTTTTTATAGTTATACCAATCATAATCGG  180

Query  181  AGGGTTCGGAAATTGATTAGTTCCTATTATATTAGGTGCCCCAGATATAGCATTTCCACG  240
            ||| || |||||||||||||| ||||| ||||| ||||||||||||||||||||||||||
Sbjct  181  AGGATTTGGAAATTGATTAGTCCCTATCATATTGGGTGCCCCAGATATAGCATTTCCACG  240

Query  241  AATAAATAATATAAGATTTTGACTTTTACCCCCCTCATTATTTTTATTGATTAACTCTTC  300
            |||||||||||||||||||||||||||||| || ||| |||||||||| ||||| |||||
Sbjct  241  AATAAATAATATAAGATTTTGACTTTTACCTCCTTCACTATTTTTATTAATTAATTCTTC  300

Query  301  ATTAATTGAATCCGGAGCTGGAACAGGATGAACAGTTTATCCCCCTCTATCTTCAAATTT  360
            |||||||||||| |||||||| ||||||||||||||||| || |||||||||||||||||
Sbjct  301  ATTAATTGAATCTGGAGCTGGTACAGGATGAACAGTTTACCCTCCTCTATCTTCAAATTT  360

Query  361  ATCACATTATGGTCCATCAGTAGATTTAGCTATTTTTTCTCTTCACCTTGCTGGTGCTTC  420
            |||||||||||| || ||||||||||||||||||||||||||||| ||||||||||||||
Sbjct  361  ATCACATTATGGCCCTTCAGTAGATTTAGCTATTTTTTCTCTTCATCTTGCTGGTGCTTC  420

Query  421  TTCAATTTTAGGTGCAATTAACTTCATCACAACTATTGTAAATATACGATCAATTGGAAT  480
            |||||||||||||||||||||||| || |||||||||||||| |||||||||||||||||
Sbjct  421  TTCAATTTTAGGTGCAATTAACTTTATTACAACTATTGTAAACATACGATCAATTGGAAT  480

Query  481  AACAATAGAACGAATACCATTATTTGTTTGATCTGTTTTAATTACAGCTATTTTACTACT  540
            |||||||||||| ||||||||||||||||||||||||||||||||||||||||||| ||||
Sbjct  481  AACAATAGAACGTATACCATTATTTGTTTGATCTGTTTTAATTACAGCTATTTTATTACT  540

Query  541  TCTATCTTTACCTGTATTAGCAGGTGCCATTACAATATTATTAACTGATCGAAACTTTAA  600
            | ||||||||||||||||||||||||||||||||||||| ||||||||||||||||||||
Sbjct  541  TTTATCTTTACCTGTATTAGCAGGTGCCATTACAATATTGTTAACTGATCGAAACTTTAA  600
```

```
Query  601  CACATCATTTTTTGATCCTTCAGGAGGAGGAGATCCCATTTTATATCAACACTTATTT  658
             ||||||||||||||||||||||| ||||||||||| |||||||||||||| ||||||
Sbjct  601  TACATCATTTTTTGATCCTTCAGGGGGAGGAGATCCAATTTTATATCAACATTTATTT  658
```

>Rhipicephalus sanguineus isolate 5MSK cytochrome c oxidase subunit I (COX1) gene, partial cds; mitochondrial
Sequence ID: MW152145.1 Length: 676
Range 1: 1 to 658

Score:1055 bits(571), Expect:0.0,
Identities:629/658(96%), Gaps:0/658(0%), Strand: Plus/Plus

```
Query  1    AACAATATATTTAATTTTTGGAGCATGATCAGGTATATTAGGACTTAGTATAAGAATATT  60
            ||||||||||||||||||||||||||||||||||||||||||||||||||||||||||||
Sbjct  1    AACAATATATTTAATTTTTGGAGCATGATCAGGTATATTAGGACTTAGTATAAGAATATT  60

Query  61   AATTCGTATAGAATTAGGACAACCTGGAACTTTAATTGGAAATGATCAGATTTATAATGT  120
            ||||||||||||||||||||||||||||||||||||||||||||||||||||||||||||
Sbjct  61   AATTCGTATAGAATTAGGACAACCTGGAACTTTAATTGGAAATGATCAGATTTATAATGT  120

Query  121  AATCGTAACAGCACATGCATTTATTATAATTTTCTTTATAGTTATACCAATCATAATCGG  180
            ||||||||||||||||||||||||||||||||| ||||||||||||||||||||||||||
Sbjct  121  AATCGTAACAGCACATGCATTTATTATAATTTTTTTTATAGTTATACCAATCATAATCGG  180

Query  181  AGGGTTCGGAAATTGATTAGTTCCTATTATATTAGGTGCCCCAGATATAGCATTTCCACG  240
            ||| || |||||||||||||| ||||| ||||| ||||||||||||||||||||||||||
Sbjct  181  AGGATTTGGAAATTGATTAGTCCCTATCATATTGGGTGCCCCAGATATAGCATTTCCACG  240

Query  241  AATAAATAATATAAGATTTTGACTTTTACCCCCCTCATTATTTTTATTGATTAACTCTTC  300
            |||||||||||||||||||||||||||||| || ||| |||||||||| ||||| |||||
Sbjct  241  AATAAATAATATAAGATTTTGACTTTTACCTCCTTCACTATTTTTATTAATTAATTCTTC  300

Query  301  ATTAATTGAATCCGGAGCTGGAACAGGATGAACAGTTTATCCCCCTCTATCTTCAAATTT  360
            |||||||||||| |||||||| ||||||||||||||||| || |||||||||||||||||
Sbjct  301  ATTAATTGAATCTGGAGCTGGTACAGGATGAACAGTTTACCCTCCTCTATCTTCAAATTT  360

Query  361  ATCACATTATGGTCCATCAGTAGATTTAGCTATTTTTTCTCTTCACCTTGCTGGTGCTTC  420
            |||||||||||| || ||||||||||||||||||||||||||||| ||||||||||||||
Sbjct  361  ATCACATTATGGCCCTTCAGTAGATTTAGCTATTTTTTCTCTTCATCTTGCTGGTGCTTC  420

Query  421  TTCAATTTTAGGTGCAATTAACTTCATCACAACTATTGTAAATATACGATCAATTGGAAT  480
            |||||||||||||||||||||||| || |||||||||||||| |||||||||||||||||
Sbjct  421  TTCAATTTTAGGTGCAATTAACTTTATTACAACTATTGTAAACATACGATCAATTGGAAT  480

Query  481  AACAATAGAACGAATACCATTATTTGTTTGATCTGTTTTAATTACAGCTATTTTACTACT  540
            |||||||||||| |||||||||||||||||||||||||||||||||||||||||| ||||
Sbjct  481  AACAATAGAACGTATACCATTATTTGTTTGATCTGTTTTAATTACAGCTATTTTATTACT  540

Query  541  TCTATCTTTACCTGTATTAGCAGGTGCCATTACAATATTATTAACTGATCGAAACTTTAA  600
            | ||||||||||||||||||||||||||||||||||||| ||||||||||||||||||||
Sbjct  541  TTTATCTTTACCTGTATTAGCAGGTGCCATTACAATATTGTTAACTGATCGAAACTTTAA  600
```

```
Query  601  CACATCATTTTTTGATCCTTCAGGAGGAGGAGATCCCATTTTATATCAACACTTATTT  658
             |||||||||||||||||||||| ||||||||||| |||||||||||||| ||||||
Sbjct  601  TACATCATTTTTTGATCCTTCAGGGGGAGGAGATCCAATTTTATATCAACATTTATTT  658
```

>Rhipicephalus sanguineus isolate 1MSK cytochrome c oxidase subunit I (COX1) gene, partial cds; mitochondrial
Sequence ID: MW152144.1 Length: 675
Range 1: 1 to 658

Score:1055 bits(571), Expect:0.0,
Identities:629/658(96%), Gaps:0/658(0%), Strand: Plus/Plus

```
Query  1    AACAATATATTTAATTTTTGGAGCATGATCAGGTATATTAGGACTTAGTATAAGAATATT  60
            ||||||||||||||||||||||||||||||||||||||||||||||||||||||||||||
Sbjct  1    AACAATATATTTAATTTTTGGAGCATGATCAGGTATATTAGGACTTAGTATAAGAATATT  60

Query  61   AATTCGTATAGAATTAGGACAACCTGGAACTTTAATTGGAAATGATCAGATTTATAATGT  120
            ||||||||||||||||||||||||||||||||||||||||||||||||||||||||||||
Sbjct  61   AATTCGTATAGAATTAGGACAACCTGGAACTTTAATTGGAAATGATCAGATTTATAATGT  120

Query  121  AATCGTAACAGCACATGCATTTATTATAATTTTCTTTATAGTTATACCAATCATAATCGG  180
            |||||||||||||||||||||||||||||||||| |||||||||||||||||||||||||
Sbjct  121  AATCGTAACAGCACATGCATTTATTATAATTTTTTTTATAGTTATACCAATCATAATCGG  180

Query  181  AGGGTTCGGAAATTGATTAGTTCCTATTATATTAGGTGCCCCAGATATAGCATTTCCACG  240
            ||| || |||||||||||||| ||||| ||||| ||||||||||||||||||||||||||
Sbjct  181  AGGATTTGGAAATTGATTAGTCCCTATCATATTGGGTGCCCCAGATATAGCATTTCCACG  240

Query  241  AATAAATAATATAAGATTTTGACTTTTACCCCCCTCATTATTTTTATTGATTAACTCTTC  300
            |||||||||||||||||||||||||||||| || ||| |||||||||| ||||| |||||
Sbjct  241  AATAAATAATATAAGATTTTGACTTTTACCTCCTTCACTATTTTTATTAATTAATTCTTC  300

Query  301  ATTAATTGAATCCGGAGCTGGAACAGGATGAACAGTTTATCCCCCTCTATCTTCAAATTT  360
            |||||||||||| |||||||| |||||||||||||||| || ||||||||||||||||||
Sbjct  301  ATTAATTGAATCTGGAGCTGGTACAGGATGAACAGTTTACCCTCCTCTATCTTCAAATTT  360

Query  361  ATCACATTATGGTCCATCAGTAGATTTAGCTATTTTTTCTCTTCACCTTGCTGGTGCTTC  420
            |||||||||||| || |||||||||||||||||||||||||||||| ||||||||||||||
Sbjct  361  ATCACATTATGGCCCTTCAGTAGATTTAGCTATTTTTTCTCTTCATCTTGCTGGTGCTTC  420

Query  421  TTCAATTTTAGGTGCAATTAACTTCATCACAACTATTGTAAATATACGATCAATTGGAAT  480
            ||||||||||||||||||||||| || |||||||||||||| ||||||||||||||||||
Sbjct  421  TTCAATTTTAGGTGCAATTAACTTTATTACAACTATTGTAAACATACGATCAATTGGAAT  480

Query  481  AACAATAGAACGAATACCATTATTTGTTTGATCTGTTTTAATTACAGCTATTTTACTACT  540
            |||||||||||| |||||||||||||||||||||||||||||||||||||||||| ||||
Sbjct  481  AACAATAGAACGTATACCATTATTTGTTTGATCTGTTTTAATTACAGCTATTTTATTACT  540

Query  541  TCTATCTTTACCTGTATTAGCAGGTGCCATTACAATATTATTAACTGATCGAAACTTTAA  600
            | |||||||||||||||||||||||||||||||||||| |||||||||||||||||||||
```

```
Sbjct  541   TTTATCTTTACCTGTATTAGCAGGTGCCATTACAATATTGTTAACTGATCGAAACTTTAA  600

Query  601   CACATCATTTTTTGATCCTTCAGGAGGAGGAGATCCCATTTTATATCAACACTTATTT  658
              ||||||||||||||||||||||| ||||||||||| |||||||||||||| ||||||
Sbjct  601   TACATCATTTTTTGATCCTTCAGGGGGAGGAGATCCAATTTTATATCAACATTTATTT  658
```

>Rhipicephalus sanguineus isolate RSN2SK cytochrome c oxidase subunit I (COX1) gene, partial cds; mitochondrial
Sequence ID: MW152143.1 Length: 676
Range 1: 1 to 658

Score:1055 bits(571), Expect:0.0,
Identities:629/658(96%), Gaps:0/658(0%), Strand: Plus/Plus

```
Query  1     AACAATATATTTAATTTTTGGAGCATGATCAGGTATATTAGGACTTAGTATAAGAATATT  60
             ||||||||||||||||||||||||||||||||||||||||||||||||||||||||||||
Sbjct  1     AACAATATATTTAATTTTTGGAGCATGATCAGGTATATTAGGACTTAGTATAAGAATATT  60

Query  61    AATTCGTATAGAATTAGGACAACCTGGAACTTTAATTGGAAATGATCAGATTTATAATGT  120
             ||||||||||||||||||||||||||||||||||||||||||||||||||||||||||||
Sbjct  61    AATTCGTATAGAATTAGGACAACCTGGAACTTTAATTGGAAATGATCAGATTTATAATGT  120

Query  121   AATCGTAACAGCACATGCATTTATTATAATTTTCTTTATAGTTATACCAATCATAATCGG  180
             ||||||||||||||||||||||||||||||||| ||||||||||||||||||||||||||
Sbjct  121   AATCGTAACAGCACATGCATTTATTATAATTTTTTTTATAGTTATACCAATCATAATCGG  180

Query  181   AGGGTTCGGAAATTGATTAGTTCCTATTATATTAGGTGCCCCAGATATAGCATTTCCACG  240
             ||| || |||||||||||||| ||||| ||||| ||||||||||||||||||||||||||
Sbjct  181   AGGATTTGGAAATTGATTAGTCCCTATCATATTGGGTGCCCCAGATATAGCATTTCCACG  240

Query  241   AATAAATAATATAAGATTTTGACTTTTACCCCCCTCATTATTTTTATTGATTAACTCTTC  300
             |||||||||||||||||||||||||||||| || ||| |||||||||| ||||| |||||
Sbjct  241   AATAAATAATATAAGATTTTGACTTTTACCTCCTTCACTATTTTTATTAATTAATTCTTC  300

Query  301   ATTAATTGAATCCGGAGCTGGAACAGGATGAACAGTTTATCCCCCTCTATCTTCAAATTT  360
             |||||||||||| |||||||| ||||||||||||||||| || |||||||||||||||||
Sbjct  301   ATTAATTGAATCTGGAGCTGGTACAGGATGAACAGTTTACCCTCCTCTATCTTCAAATTT  360

Query  361   ATCACATTATGGTCCATCAGTAGATTTAGCTATTTTTTCTCTTCACCTTGCTGGTGCTTC  420
             |||||||||||| || ||||||||||||||||||||||||||||| ||||||||||||||
Sbjct  361   ATCACATTATGGCCCTTCAGTAGATTTAGCTATTTTTTCTCTTCATCTTGCTGGTGCTTC  420

Query  421   TTCAATTTTAGGTGCAATTAACTTCATCACAACTATTGTAAATATACGATCAATTGGAAT  480
             |||||||||||||||||||||||| || |||||||||||||| |||||||||||||||||
Sbjct  421   TTCAATTTTAGGTGCAATTAACTTTATTACAACTATTGTAAACATACGATCAATTGGAAT  480

Query  481   AACAATAGAACGAATACCATTATTTGTTTGATCTGTTTTAATTACAGCTATTTTACTACT  540
             |||||||||||| |||||||||||||||||||||||||||||||||||||||||| ||||
Sbjct  481   AACAATAGAACGTATACCATTATTTGTTTGATCTGTTTTAATTACAGCTATTTTATTACT  540

Query  541   TCTATCTTTACCTGTATTAGCAGGTGCCATTACAATATTATTAACTGATCGAAACTTTAA  600
```

```
                | ||||||||||||||||||||||||||||||||||||| ||||||||||||||||||||
Sbjct  541  TTTATCTTTACCTGTATTAGCAGGTGCCATTACAATATTGTTAACTGATCGAAACTTTAA  600

Query  601  CACATCATTTTTTGATCCTTCAGGAGGAGGAGATCCCATTTTATATCAACACTTATTT  658
             ||||||||||||||||||||||| ||||||||||| |||||||||||||| ||||||
Sbjct  601  TACATCATTTTTTGATCCTTCAGGGGGAGGAGATCCAATTTTATATCAACATTTATTT  658
```

>Rhipicephalus sanguineus isolate RSN11SK cytochrome c oxidase subunit I (COX1) gene, partial cds; mitochondrial
Sequence ID: MW152142.1 Length: 669
Range 1: 1 to 658

Score:1055 bits(571), Expect:0.0,
Identities:629/658(96%), Gaps:0/658(0%), Strand: Plus/Plus

```
Query  1    AACAATATATTTAATTTTTGGAGCATGATCAGGTATATTAGGACTTAGTATAAGAATATT  60
            ||||||||||||||||||||||||||||||||||||||||||||||||||||||||||||
Sbjct  1    AACAATATATTTAATTTTTGGAGCATGATCAGGTATATTAGGACTTAGTATAAGAATATT  60

Query  61   AATTCGTATAGAATTAGGACAACCTGGAACTTTAATTGGAAATGATCAGATTTATAATGT  120
            ||||||||||||||||||||||||||||||||||||||||||||||||||||||||||||
Sbjct  61   AATTCGTATAGAATTAGGACAACCTGGAACTTTAATTGGAAATGATCAGATTTATAATGT  120

Query  121  AATCGTAACAGCACATGCATTTATTATAATTTCTTTATAGTTATACCAATCATAATCGG  180
            |||||||||||||||||||||||||||||||| |||||||||||||||||||||||||||
Sbjct  121  AATCGTAACAGCACATGCATTTATTATAATTTTTTTATAGTTATACCAATCATAATCGG  180

Query  181  AGGGTTCGGAAATTGATTAGTTCCTATTATATTAGGTGCCCCAGATATAGCATTTCCACG  240
            ||| || |||||||||||||| ||||| ||||| ||||||||||||||||||||||||||
Sbjct  181  AGGATTTGGAAATTGATTAGTCCCTATCATATTGGGTGCCCCAGATATAGCATTTCCACG  240

Query  241  AATAAATAATATAAGATTTTGACTTTTACCCCCCTCATTATTTTTATTGATTAACTCTTC  300
            |||||||||||||||||||||||||||||| || ||| |||||||||| ||||| |||||
Sbjct  241  AATAAATAATATAAGATTTTGACTTTTACCTCCTTCACTATTTTTATTAATTAATTCTTC  300

Query  301  ATTAATTGAATCCGGAGCTGGAACAGGATGAACAGTTTATCCCCCTCTATCTTCAAATTT  360
            |||||||||||| |||||||| ||||||||||||||||| || |||||||||||||||||
Sbjct  301  ATTAATTGAATCTGGAGCTGGTACAGGATGAACAGTTTACCCTCCTCTATCTTCAAATTT  360

Query  361  ATCACATTATGGTCCATCAGTAGATTTAGCTATTTTTTCTCTTCACCTTGCTGGTGCTTC  420
            |||||||||||| || ||||||||||||||||||||||||||||| ||||||||||||||
Sbjct  361  ATCACATTATGGCCCTTCAGTAGATTTAGCTATTTTTTCTCTTCATCTTGCTGGTGCTTC  420

Query  421  TTCAATTTTAGGTGCAATTAACTTCATCACAACTATTGTAAATATACGATCAATTGGAAT  480
            |||||||||||||||||||||||| || |||||||||||||| |||||||||||||||||
Sbjct  421  TTCAATTTTAGGTGCAATTAACTTTATTACAACTATTGTAAACATACGATCAATTGGAAT  480

Query  481  AACAATAGAACGAATACCATTATTTGTTTGATCTGTTTTAATTACAGCTATTTTACTACT  540
            |||||||||||| ||||||||||||||||||||||||||||||||||||||||||| ||||
Sbjct  481  AACAATAGAACGTATACCATTATTTGTTTGATCTGTTTTAATTACAGCTATTTTATTACT  540
```

```
Query  541  TCTATCTTTACCTGTATTAGCAGGTGCCATTACAATATTATTAACTGATCGAAACTTTAA  600
            | ||||||||||||||||||||||||||||||||||||| ||||||||||||||||||||
Sbjct  541  TTTATCTTTACCTGTATTAGCAGGTGCCATTACAATATTGTTAACTGATCGAAACTTTAA  600

Query  601  CACATCATTTTTTGATCCTTCAGGAGGAGGAGATCCCATTTTATATCAACACTTATTT  658
             ||||||||||||||||||||||| ||||||||||| |||||||||||||| ||||||
Sbjct  601  TACATCATTTTTTGATCCTTCAGGGGGAGGAGATCCAATTTTATATCAACATTTATTT  658
```

>Rhipicephalus sanguineus sensu lato 'temperate lineage' isolate S358 cytochrome c oxidase subunit I (COI) gene, partial cds; mitochondrial
Sequence ID: MF426012.1 Length: 684
Range 1: 14 to 671

Score:1050 bits(568), Expect:0.0,
Identities:628/658(95%), Gaps:0/658(0%), Strand: Plus/Plus

```
Query  1    AACAATATATTTAATTTTTGGAGCATGATCAGGTATATTAGGACTTAGTATAAGAATATT  60
            ||||||||||||||||||||||||||||||||||||||||||||||||||||||||||||
Sbjct  14   AACAATATATTTAATTTTTGGAGCATGATCAGGTATATTAGGACTTAGTATAAGAATATT  73

Query  61   AATTCGTATAGAATTAGGACAACCTGGAACTTTAATTGGAAATGATCAGATTTATAATGT  120
            ||||||||||||||||||||||||||||||||||||||||||||||||||||||||||||
Sbjct  74   AATTCGTATAGAATTAGGACAACCTGGAACTTTAATTGGAAATGATCAGATTTATAATGT  133

Query  121  AATCGTAACAGCACATGCATTTATTATAATTTTCTTTATAGTTATACCAATCATAATCGG  180
            ||||||||||||||||||||||||||||||||| ||||||||||||||||||||||||||
Sbjct  134  AATCGTAACAGCACATGCATTTATTATAATTTTTTTTATAGTTATACCAATCATAATCGG  193

Query  181  AGGGTTCGGAAATTGATTAGTTCCTATTATATTAGGTGCCCCAGATATAGCATTTCCACG  240
            ||| || |||||||||||||| ||||| ||||||||||||||||||||||||||||||||
Sbjct  194  AGGATTTGGAAATTGATTAGTCCCTATCATATTAGGTGCCCCAGATATAGCATTTCCACG  253

Query  241  AATAAATAATATAAGATTTTGACTTTTACCCCCCTCATTATTTTTATTGATTAACTCTTC  300
            |||||||||||||||||||||||||||||| || ||| |||||||||| |||||||||||
Sbjct  254  AATAAATAATATAAGATTTTGACTTTTACCTCCTTCACTATTTTTATTAATTAACTCTTC  313

Query  301  ATTAATTGAATCCGGAGCTGGAACAGGATGAACAGTTTATCCCCCTCTATCTTCAAATTT  360
            |||||||||||| |||||||| ||||||||||||||||| || |||||||||||||||||
Sbjct  314  ATTAATTGAATCTGGAGCTGGTACAGGATGAACAGTTTACCCTCCTCTATCTTCAAATTT  373

Query  361  ATCACATTATGGTCCATCAGTAGATTTAGCTATTTTTTCTCTTCACCTTGCTGGTGCTTC  420
            |||||||||||| || ||||||||||||||||| ||||||||||| ||||||||||||||
Sbjct  374  ATCACATTATGGCCCTTCAGTAGATTTAGCTATCTTTTCTCTTCATCTTGCTGGTGCTTC  433

Query  421  TTCAATTTTAGGTGCAATTAACTTCATCACAACTATTGTAAATATACGATCAATTGGAAT  480
            |||||||||||||||||||||||| || ||||||||| |||| |||||||||||||||||
Sbjct  434  TTCAATTTTAGGTGCAATTAACTTTATTACAACTATTATAAACATACGATCAATTGGAAT  493

Query  481  AACAATAGAACGAATACCATTATTTGTTTGATCTGTTTTAATTACAGCTATTTTACTACT  540
            |||||||||||| |||||||||||||||||||||||||||||||||||||||||| ||||
Sbjct  494  AACAATAGAACGTATACCATTATTTGTTTGATCTGTTTTAATTACAGCTATTTTATTACT  553
```

```
Query  541  TCTATCTTTACCTGTATTAGCAGGTGCCATTACAATATTATTAACTGATCGAAACTTTAA  600
              ||||||||||||||||||||||||||||||||||||| ||||||||||||||||||||
Sbjct  554  CTTATCTTTACCTGTATTAGCAGGTGCCATTACAATATTGTTAACTGATCGAAACTTTAA  613

Query  601  CACATCATTTTTTGATCCTTCAGGAGGAGGAGATCCCATTTTATATCAACACTTATTT  658
             ||||||||||||||||||||||| ||||||||||| |||||||||||||| ||||||
Sbjct  614  TACATCATTTTTTGATCCTTCAGGGGGAGGAGATCCAATTTTATATCAACATTTATTT  671
```

>Rhipicephalus sanguineus haplotype I cytochrome oxidase subunit I (COI) gene, partial cds; mitochondrial
Sequence ID: KU556743.1 Length: 659
Range 1: 12 to 659

Score:1048 bits(567), Expect:0.0,
Identities:621/648(96%), Gaps:0/648(0%), Strand: Plus/Plus

```
Query  1    AACAATATATTTAATTTTTGGAGCATGATCAGGTATATTAGGACTTAGTATAAGAATATT  60
            ||||||||||||||||||||||||||||||||||||||||||||||||||||||||||||
Sbjct  12   AACAATATATTTAATTTTTGGAGCATGATCAGGTATATTAGGACTTAGTATAAGAATATT  71

Query  61   AATTCGTATAGAATTAGGACAACCTGGAACTTTAATTGGAAATGATCAGATTTATAATGT  120
            ||||||||||||||||||||||||||||||||||||||||||||||||||||||||||||
Sbjct  72   AATTCGTATAGAATTAGGACAACCTGGAACTTTAATTGGAAATGATCAGATTTATAATGT  131

Query  121  AATCGTAACAGCACATGCATTTATTATAATTTTCTTTATAGTTATACCAATCATAATCGG  180
            ||||||||||||||||||||||||||||||||| ||||||||||||||||||||||||||
Sbjct  132  AATCGTAACAGCACATGCATTTATTATAATTTTTTTTATAGTTATACCAATCATAATCGG  191

Query  181  AGGGTTCGGAAATTGATTAGTTCCTATTATATTAGGTGCCCCAGATATAGCATTTCCACG  240
            ||| || |||||||||||| | ||||| ||||| ||||||||||||||||||||||||||
Sbjct  192  AGGATTTGGAAATTGATTAATCCCTATCATATTGGGTGCCCCAGATATAGCATTTCCACG  251

Query  241  AATAAATAATATAAGATTTTGACTTTTACCCCCCTCATTATTTTTATTGATTAACTCTTC  300
            |||||||||||||||||||||||||||||| || ||| |||||||||| |||||||||||
Sbjct  252  AATAAATAATATAAGATTTTGACTTTTACCTCCTTCACTATTTTTATTAATTAACTCTTC  311

Query  301  ATTAATTGAATCCGGAGCTGGAACAGGATGAACAGTTTATCCCCCTCTATCTTCAAATTT  360
            |||||||||||| |||||||| ||||||||||||||||| || |||||||||||||||||
Sbjct  312  ATTAATTGAATCTGGAGCTGGTACAGGATGAACAGTTTACCCTCCTCTATCTTCAAATTT  371

Query  361  ATCACATTATGGTCCATCAGTAGATTTAGCTATTTTTTCTCTTCACCTTGCTGGTGCTTC  420
            ||||||||||||||| ||||||||||||||||||||||||||||| ||||||||||||||
Sbjct  372  ATCACATTATGGTCCTTCAGTAGATTTAGCTATTTTTTCTCTTCATCTTGCTGGTGCTTC  431

Query  421  TTCAATTTTAGGTGCAATTAACTTCATCACAACTATTGTAAATATACGATCAATTGGAAT  480
            |||||||||||||||||||||||| || ||||||||||||||| ||||||||||||||||
Sbjct  432  TTCAATTTTAGGTGCAATTAACTTTATTACAACTATTGTAAACATACGATCAATTGGAAT  491

Query  481  AACAATAGAACGAATACCATTATTTGTTTGATCTGTTTTAATTACAGCTATTTTACTACT  540
            |||||||||||| ||||||||||||||||||||||||||||||||||||||||||| ||||
```

```
Sbjct  492   AACAATAGAACGTATACCATTATTTGTTTGATCTGTTTTAATTACAGCTATTTTATTACT  551

Query  541   TCTATCTTTACCTGTATTAGCAGGTGCCATTACAATATTATTAACTGATCGAAACTTTAA  600
             | ||||||||||||||||||||||||||||||||||||| ||||||||||||||||||||
Sbjct  552   TTTATCTTTACCTGTATTAGCAGGTGCCATTACAATATTGTTAACTGATCGAAACTTTAA  611

Query  601   CACATCATTTTTTGATCCTTCAGGAGGAGGAGATCCCATTTTATATCA  648
              ||||||||||||||||||||||| ||||||||||| |||||||||||
Sbjct  612   TACATCATTTTTTGATCCTTCAGGGGGAGGAGATCCAATTTTATATCA  659
```

>Rhipicephalus sanguineus haplotype II cytochrome oxidase subunit I (COI) gene, partial cds; mitochondrial
Sequence ID: KU556744.1 Length: 787
Range 1: 29 to 688

Score:1046 bits(566), Expect:0.0,
Identities:629/660(95%), Gaps:2/660(0%), Strand: Plus/Plus

```
Query  1     AACAATATATTTAATTTTTGGAGCATGATCAGGTATATTAGGACTTAGTATAAGAATATT  60
             ||||||||||||||||||||||||||||||||||||||||||||||||||||||||||||
Sbjct  29    AACAATATATTTAATTTTTGGAGCATGATCAGGTATATTAGGACTTAGTATAAGAATATT  88

Query  61    AATTCGTATAGAATTAGGACAACCTGGAACTTTAATTGGAAATGATCAGATTTATAATGT  120
             ||||||||||||||||||||||||||||||||||||||||||||||||||||||||||||
Sbjct  89    AATTCGTATAGAATTAGGACAACCTGGAACTTTAATTGGAAATGATCAGATTTATAATGT  148

Query  121   AATCGTAACAGCACATGCATTTATTATAATTTTCTTTATAGTTATACCAATCATAATCGG  180
             ||||||||||||||||||||||||||||||||| ||||||||||||||||||||||||||
Sbjct  149   AATCGTAACAGCACATGCATTTATTATAATTTTTTTTATAGTTATACCAATCATAATCGG  208

Query  181   AGGGTTCGGAAATTGATTAGTTCCTATTATATTAGGTGCCCCAGATATAGCATTTCCACG  240
             ||| || ||||| |||||||| ||||| ||||| ||||||||||||||||||||||||||
Sbjct  209   AGGATTTGGAAACTGATTAGTCCCTATCATATTGGGTGCCCCAGATATAGCATTTCCACG  268

Query  241   AATAAATAATATAAGATTTTGACTTTTACCCCCCTCATTATTTTTATTGATTAACTCTTC  300
             |||||||||||||||||||||||||||||| || ||| |||||||||| |||||||||||
Sbjct  269   AATAAATAATATAAGATTTTGACTTTTACCTCCTTCACTATTTTTATTAATTAACTCTTC  328

Query  301   ATTAATTGAATCCGGAGCTGGAACAGGATGAACAGTTTATCCCCCTCTATCTTCAAATTT  360
             |||||||||||| |||||||| ||||||||||||||||| || |||||||||||||||||
Sbjct  329   ATTAATTGAATCTGGAGCTGGTACAGGATGAACAGTTTACCCTCCTCTATCTTCAAATTT  388

Query  361   ATCACATTATGGTCCATCAGTAGATTTAGCTATTTTTTCTCTTCACCTTGCTGGTGCTTC  420
             ||||||||||||||| ||||||||||||||||||||||||||||| ||||||||||||||
Sbjct  389   ATCACATTATGGTCCTTCAGTAGATTTAGCTATTTTTTCTCTTCATCTTGCTGGTGCTTC  448

Query  421   TTCAATTTTAGGTGCAATTAACTTCATCACAACTATTGTAAATATACGATCAATTGGAAT  480
             |||||||||||||||||||||||| || |||||||||||||| |||||||||||||||||
Sbjct  449   TTCAATTTTAGGTGCAATTAACTTTATTACAACTATTGTAAACATACGATCAATTGGAAT  508

Query  481   AACAATAGAACGAATACCATTATTTGTTTGATCTGTTTTAATTACAGCTATTTTACTACT  540
```

```
            |||||||||||| |||||||||||||||||||||||||||||||||||||||||| ||||
Sbjct  509  AACAATAGAACGTATACCATTATTTGTTTGATCTGTTTTAATTACAGCTATTTTATTACT  568

Query  541  TCTATCTTTACCTGTATTAGCAGGTGCCATTACAATATTATTAACTGATCGAAACTTTAA  600
            | ||||||||||||||||||||||||||||||||||||| ||||||||||||||||||||
Sbjct  569  TTTATCTTTACCTGTATTAGCAGGTGCCATTACAATATTGTTAACTGATCGAAACTTTAA  628

Query  601  CACATCATTTTTTGATCCTTCAGGAGG-AGGAGATCCC-ATTTTATATCAACACTTATTT  658
             ||||||||||||||||||||||| || |||||||||  |||||||||||||  ||||||
Sbjct  629  TACATCATTTTTTGATCCTTCAGGGGGGAGGAGATCCAAATTTTATATCAACTTTTATTT  688
```

>Rhipicephalus sanguineus sensu lato 'temperate lineage' isolate S943 cytochrome c oxidase subunit I (COI) gene, partial cds; mitochondrial
Sequence ID: MF426020.1 Length: 812
Range 1: 2 to 644

Score:1038 bits(562), Expect:0.0,
Identities:616/643(96%), Gaps:0/643(0%), Strand: Plus/Plus

```
Query  16   TTTTGGAGCATGATCAGGTATATTAGGACTTAGTATAAGAATATTAATTCGTATAGAATT  75
            ||||||||||||||||||||||||||||||||||||||||||||||||||||||||||||
Sbjct  2    TTTTGGAGCATGATCAGGTATATTAGGACTTAGTATAAGAATATTAATTCGTATAGAATT  61

Query  76   AGGACAACCTGGAACTTTAATTGGAAATGATCAGATTTATAATGTAATCGTAACAGCACA  135
            ||||||||||||||||||||||||||||||||||||||||||||||||||||||||||||
Sbjct  62   AGGACAACCTGGAACTTTAATTGGAAATGATCAGATTTATAATGTAATCGTAACAGCACA  121

Query  136  TGCATTTATTATAATTTTCTTTATAGTTATACCAATCATAATCGGAGGGTTCGGAAATTG  195
            |||||||||||||||||| ||||||||||||||||||||||||||||| || ||||||||
Sbjct  122  TGCATTTATTATAATTTTTTTTATAGTTATACCAATCATAATCGGAGGATTTGGAAATTG  181

Query  196  ATTAGTTCCTATTATATTAGGTGCCCCAGATATAGCATTTCCACGAATAAATAATATAAG  255
            |||||| ||||| ||||| |||||||||||||||||||||||||||||||||||||||||
Sbjct  182  ATTAGTCCCTATCATATTGGGTGCCCCAGATATAGCATTTCCACGAATAAATAATATAAG  241

Query  256  ATTTTGACTTTTACCCCCCTCATTATTTTTATTGATTAACTCTTCATTAATTGAATCCGG  315
            ||||||||||||||| || ||| |||||||||| ||||||||||||||||||||||| ||
Sbjct  242  ATTTTGACTTTTACCTCCTTCACTATTTTTATTAATTAACTCTTCATTAATTGAATCTGG  301

Query  316  AGCTGGAACAGGATGAACAGTTTATCCCCCTCTATCTTCAAATTTATCACATTATGGTCC  375
            |||||| ||||||||||||||||| || ||||||||||||||||||||||||||||||||
Sbjct  302  AGCTGGTACAGGATGAACAGTTTACCCTCCTCTATCTTCAAATTTATCACATTATGGTCC  361

Query  376  ATCAGTAGATTTAGCTATTTTTCTCTTCACCTTGCTGGTGCTTCTTCAATTTTAGGTGC  435
             |||||||||||||||||||||||||||| ||||||||||||||||||||||||||||||
Sbjct  362  TTCAGTAGATTTAGCTATTTTTCTCTTCATCTTGCTGGTGCTTCTTCAATTTTAGGTGC  421

Query  436  AATTAACTTCATCACAACTATTGTAAATATACGATCAATTGGAATAACAATAGAACGAAT  495
            ||||||||| || |||||||||||||| ||||||||||||||||||||||||||||| ||
Sbjct  422  AATTAACTTTATTACAACTATTGTAAACATACGATCAATTGGAATAACAATAGAACGTAT  481
```

```
Query  496  ACCATTATTTGTTTGATCTGTTTTAATTACAGCTATTTTACTACTTCTATCTTTACCTGT  555
            |||||||||||||||||||||||||||||||||||||||| ||||| |||||||||||||
Sbjct  482  ACCATTATTTGTTTGATCTGTTTTAATTACAGCTATTTTATTACTTTTATCTTTACCTGT  541

Query  556  ATTAGCAGGTGCCATTACAATATTATTAACTGATCGAAACTTTAACACATCATTTTTTGA  615
            |||||||||||||||||||||||| |||||||||||||||||||| ||||||||||||||
Sbjct  542  ATTAGCAGGTGCCATTACAATATTGTTAACTGATCGAAACTTTAATACATCATTTTTTGA  601

Query  616  TCCTTCAGGAGGAGGAGATCCCATTTTATATCAACACTTATTT  658
            ||||||||| ||||||||||| |||||||||||||| ||||||
Sbjct  602  TCCTTCAGGGGGAGGAGATCCAATTTTATATCAACATTTATTT  644
```

>Rhipicephalus sanguineus sensu lato 'temperate lineage' isolate S337 cytochrome c oxidase subunit I (COI) gene, partial cds; mitochondrial
Sequence ID: MF426010.1 Length: 824
Range 1: 1 to 648

Score:1037 bits(561), Expect:0.0,
Identities:619/648(96%), Gaps:0/648(0%), Strand: Plus/Plus

```
Query  11   TTAATTTTTGGAGCATGATCAGGTATATTAGGACTTAGTATAAGAATATTAATTCGTATA  70
            ||||||||||||||||||||||||||||||||||||||||||||||||||||||||||||
Sbjct  1    TTAATTTTTGGAGCATGATCAGGTATATTAGGACTTAGTATAAGAATATTAATTCGTATA  60

Query  71   GAATTAGGACAACCTGGAACTTTAATTGGAAATGATCAGATTTATAATGTAATCGTAACA  130
            ||||||||||||||||||||||||||||||||||||||||||||||||||||||||||||
Sbjct  61   GAATTAGGACAACCTGGAACTTTAATTGGAAATGATCAGATTTATAATGTAATCGTAACA  120

Query  131  GCACATGCATTTATTATAATTTTCTTTATAGTTATACCAATCATAATCGGAGGGTTCGGA  190
            ||||||||||||||||||||||| ||||||||||||||||||||||||||||| || |||
Sbjct  121  GCACATGCATTTATTATAATTTTTTTTATAGTTATACCAATCATAATCGGAGGATTTGGA  180

Query  191  AATTGATTAGTTCCTATTATATTAGGTGCCCCAGATATAGCATTTCCACGAATAAATAAT  250
            ||||||||||| ||||| ||||| ||||||||||||||||||||||||||||||||||||
Sbjct  181  AATTGATTAGTCCCTATCATATTGGGTGCCCCAGATATAGCATTTCCACGAATAAATAAT  240

Query  251  ATAAGATTTTGACTTTTACCCCCCTCATTATTTTTATTGATTAACTCTTCATTAATTGAA  310
            |||||||||||||||||||| || ||| |||||||||| |||||||||||||||||||||
Sbjct  241  ATAAGATTTTGACTTTTACCTCCTTCACTATTTTTATTAATTAACTCTTCATTAATTGAA  300

Query  311  TCCGGAGCTGGAACAGGATGAACAGTTTATCCCCCTCTATCTTCAAATTTATCACATTAT  370
            || |||||||| ||||||||||||||||| || |||||||||||||||||||||||||||
Sbjct  301  TCTGGAGCTGGTACAGGATGAACAGTTTACCCTCCTCTATCTTCAAATTTATCACATTAT  360

Query  371  GGTCCATCAGTAGATTTAGCTATTTTTCTCTTCACCTTGCTGGTGCTTCTTCAATTTTA  430
            || || ||||||||||||||||| |||||||||| |||||||||||||||||||||||||
Sbjct  361  GGCCCTTCAGTAGATTTAGCTATCTTTCTCTTCATCTTGCTGGTGCTTCTTCAATTTTA  420

Query  431  GGTGCAATTAACTTCATCACAACTATTGTAAATATACGATCAATTGGAATAACAATAGAA  490
            |||||||||||||| || |||||||||||||| |||||||||||||||||||||||||||
Sbjct  421  GGTGCAATTAACTTTATTACAACTATTGTAAACATACGATCAATTGGAATAACAATAGAA  480
```

```
Query  491   CGAATACCATTATTTGTTTGATCTGTTTTAATTACAGCTATTTTACTACTTCTATCTTA  550
             || |||||||||||||||||||||||||||||||||||||||||| ||||| ||||||||
Sbjct  481   CGTATACCATTATTTGTTTGATCTGTTTTAATTACAGCTATTTTATTACTTTTATCTTA  540

Query  551   CCTGTATTAGCAGGTGCCATTACAATATTATTAACTGATCGAAACTTTAACACATCATTT  610
             ||||||||||||||||||||||||||||| |||||||||||||||||||| |||||||||
Sbjct  541   CCTGTATTAGCAGGTGCCATTACAATATTGTTAACTGATCGAAACTTTAATACATCATTT  600

Query  611   TTTGATCCTTCAGGAGGAGGAGATCCCATTTTATATCAACACTTATTT  658
             |||||||||||||| ||||||||||| |||||||||||||| ||||||
Sbjct  601   TTTGATCCTTCAGGGGGAGGAGATCCAATTTTATATCAACATTTATTT  648
```

>Rhipicephalus sanguineus sensu lato 'temperate lineage' isolate S344 cytochrome c oxidase subunit I (COI) gene, partial cds; mitochondrial
Sequence ID: MF426011.1 Length: 809
Range 1: 3 to 644

Score:1031 bits(558), Expect:0.0,
Identities:614/642(96%), Gaps:0/642(0%), Strand: Plus/Plus

```
Query  17    TTTGGAGCATGATCAGGTATATTAGGACTTAGTATAAGAATATTAATTCGTATAGAATTA  76
             ||||||||||||||||||||||||||||||||||||||||||||||||||||||||||||
Sbjct  3     TTTGGAGCATGATCAGGTATATTAGGACTTAGTATAAGAATATTAATTCGTATAGAATTA  62

Query  77    GGACAACCTGGAACTTTAATTGGAAATGATCAGATTTATAATGTAATCGTAACAGCACAT  136
             ||||||||||||||||||||||||||||||||||||||||||||||||||||||||||| 
Sbjct  63    GGACAACCTGGAACTTTAATTGGAAATGATCAGATTTATAATGTAATCGTAACAGCACAC  122

Query  137   GCATTTATTATAATTTTCTTTATAGTTATACCAATCATAATCGGAGGGTTCGGAAATTGA  196
             ||||||||||||||||| ||||||||||||||||||||||||||||| || |||||||||
Sbjct  123   GCATTTATTATAATTTTTTTTATAGTTATACCAATCATAATCGGAGGATTTGGAAATTGA  182

Query  197   TTAGTTCCTATTATATTAGGTGCCCCAGATATAGCATTTCCACGAATAAATAATATAAGA  256
             ||||| ||||||||||||||||||||||||||||||||||||||||||||||||||||||
Sbjct  183   TTAGTCCCTATTATATTAGGTGCCCCAGATATAGCATTTCCACGAATAAATAATATAAGA  242

Query  257   TTTTGACTTTTACCCCCCTCATTATTTTTATTGATTAACTCTTCATTAATTGAATCCGGA  316
             |||||||||||||| || ||| |||||||||| ||||||||||||||||||||||| |||
Sbjct  243   TTTTGACTTTTACCTCCTTCACTATTTTTATTAATTAACTCTTCATTAATTGAATCTGGA  302

Query  317   GCTGGAACAGGATGAACAGTTTATCCCCCTCTATCTTCAAATTTATCACATTATGGTCCA  376
             ||||| ||||||||||||||||| || ||||||||||||||||||||||||||||| || 
Sbjct  303   GCTGGTACAGGATGAACAGTTTACCCTCCTCTATCTTCAAATTTATCACATTATGGCCCT  362

Query  377   TCAGTAGATTTAGCTATTTTTTCTCTTCACCTTGCTGGTGCTTCTTCAATTTTAGGTGCA  436
             ||||||||||||||||| ||||||||||| ||||||||||||||||||||||||||||||
Sbjct  363   TCAGTAGATTTAGCTATCTTTTCTCTTCATCTTGCTGGTGCTTCTTCAATTTTAGGTGCA  422

Query  437   ATTAACTTCATCACAACTATTGTAAATATACGATCAATTGGAATAACAATAGAACGAATA  496
             |||||||| || |||||||||||||| ||||||||||||||||||||||||||||| |||
```

```
Sbjct  423  ATTAACTTTATTACAACTATTGTAAACATACGATCAATTGGAATAACAATAGAACGTATA  482

Query  497  CCATTATTTGTTTGATCTGTTTTAATTACAGCTATTTTACTACTTCTATCTTTACCTGTA  556
            ||||||||||||||||||||||||||||||||||||||| ||||| ||||||||||||||
Sbjct  483  CCATTATTTGTTTGATCTGTTTTAATTACAGCTATTTTATTACTTTTATCTTTACCTGTA  542

Query  557  TTAGCAGGTGCCATTACAATATTATTAACTGATCGAAACTTTAACACATCATTTTTTGAT  616
            ||||||||||||||||||||||| |||||||||||||||||||| |||||||||||||||
Sbjct  543  TTAGCAGGTGCCATTACAATATTGTTAACTGATCGAAACTTTAATACATCATTTTTTGAT  602

Query  617  CCTTCAGGAGGAGGAGATCCCATTTTATATCAACACTTATTT  658
            |||||||| ||||||||||| |||||||||||||| ||||||
Sbjct  603  CCTTCAGGGGGAGGAGATCCAATTTTATATCAACATTTATTT  644
```

>Rhipicephalus sanguineus sensu lato 'temperate lineage' isolate S475 cytochrome c oxidase subunit I (COI) gene, partial cds; mitochondrial
Sequence ID: MF426014.1 Length: 649
Range 1: 28 to 649

Score:1022 bits(553), Expect:0.0,
Identities:599/622(96%), Gaps:0/622(0%), Strand: Plus/Plus

```
Query  1    AACAATATATTTAATTTTTGGAGCATGATCAGGTATATTAGGACTTAGTATAAGAATATT  60
            ||||||||||||||||||||||||||||||||||||||||||||||||||||||||||||
Sbjct  28   AACAATATATTTAATTTTTGGAGCATGATCAGGTATATTAGGACTTAGTATAAGAATATT  87

Query  61   AATTCGTATAGAATTAGGACAACCTGGAACTTTAATTGGAAATGATCAGATTTATAATGT  120
            ||||||||||||||||||||||||||||||||||||||||||||||||||||||||||||
Sbjct  88   AATTCGTATAGAATTAGGACAACCTGGAACTTTAATTGGAAATGATCAGATTTATAATGT  147

Query  121  AATCGTAACAGCACATGCATTTATTATAATTTTCTTTATAGTTATACCAATCATAATCGG  180
            ||||||||||||||||||||||||||||||||| ||||||||||||||||||||||||||
Sbjct  148  AATCGTAACAGCACATGCATTTATTATAATTTTTTTTATAGTTATACCAATCATAATCGG  207

Query  181  AGGGTTCGGAAATTGATTAGTTCCTATTATATTAGGTGCCCCAGATATAGCATTTCCACG  240
            ||| || |||||||||||||| ||||| ||||| ||||||||||||||||||||||||||
Sbjct  208  AGGATTTGGAAATTGATTAGTCCCTATCATATTGGGTGCCCCAGATATAGCATTTCCACG  267

Query  241  AATAAATAATATAAGATTTTGACTTTTACCCCCCTCATTATTTTTATTGATTAACTCTTC  300
            |||||||||||||||||||||||||||||| || ||| |||||||||| |||||||||||
Sbjct  268  AATAAATAATATAAGATTTTGACTTTTACCTCCTTCACTATTTTTATTAATTAACTCTTC  327

Query  301  ATTAATTGAATCCGGAGCTGGAACAGGATGAACAGTTTATCCCCCTCTATCTTCAAATTT  360
            |||||||||||| |||||||| ||||||||||||||||| || |||||||||||||||||
Sbjct  328  ATTAATTGAATCTGGAGCTGGTACAGGATGAACAGTTTACCCTCCTCTATCTTCAAATTT  387

Query  361  ATCACATTATGGTCCATCAGTAGATTTAGCTATTTTTTCTCTTCACCTTGCTGGTGCTTC  420
            ||||||||||||||| ||||||||||||||||||||||||||||| ||||||||||||||
Sbjct  388  ATCACATTATGGTCCTTCAGTAGATTTAGCTATTTTTTCTCTTCATCTTGCTGGTGCTTC  447

Query  421  TTCAATTTTAGGTGCAATTAACTTCATCACAACTATTGTAAATATACGATCAATTGGAAT  480
```

```
                ||||||||||||||||||||||| || |||||||||||||| |||||||||||||||||
Sbjct  448  TTCAATTTTAGGTGCAATTAACTTTATTACAACTATTGTAAACATACGATCAATTGGAAT  507

Query  481  AACAATAGAACGAATACCATTATTTGTTTGATCTGTTTTAATTACAGCTATTTTACTACT  540
            |||||||||||| |||||||||||||||||||||||||||||||||||||||||||||||
Sbjct  508  AACAATAGAACGTATACCATTATTTGTTTGATCTGTTTTAATTACAGCTATTTTACTACT  567

Query  541  TCTATCTTTACCTGTATTAGCAGGTGCCATTACAATATTATTAACTGATCGAAACTTTAA  600
            | ||||||||||||||||||||||||||||||||||||| ||||||||||||||||||||
Sbjct  568  TTTATCTTTACCTGTATTAGCAGGTGCCATTACAATATTGTTAACTGATCGAAACTTTAA  627

Query  601  CACATCATTTTTTGATCCTTCA  622
             |||||||||||||||||||||
Sbjct  628  TACATCATTTTTTGATCCTTCA  649
```

>Rhipicephalus sanguineus isolate LIC5424B cytochrome oxidase subunit 1 (COI) gene, partial cds; mitochondrial
Sequence ID: KX383818.1 Length: 768
>Rhipicephalus sanguineus isolate LIC5554A cytochrome oxidase subunit 1 (COI) gene, partial cds; mitochondrial
Sequence ID: KX383819.1 Length: 768
Range 1: 1 to 642

Score:1020 bits(552), Expect:0.0,
Identities:612/642(95%), Gaps:0/642(0%), Strand: Plus/Plus

```
Query  17   TTTGGAGCATGATCAGGTATATTAGGACTTAGTATAAGAATATTAATTCGTATAGAATTA  76
            ||||||||||||||||||||||||||||||||||||||||||||||||||||||||||||
Sbjct  1    TTTGGAGCATGATCAGGTATATTAGGACTTAGTATAAGAATATTAATTCGTATAGAATTA  60

Query  77   GGACAACCTGGAACTTTAATTGGAAATGATCAGATTTATAATGTAATCGTAACAGCACAT  136
            ||||||||||||||||||||||||||||||||||||||||||||||||||||||||||||
Sbjct  61   GGACAACCTGGAACTTTAATTGGAAATGATCAGATTTATAATGTAATCGTAACAGCACAT  120

Query  137  GCATTTATTATAATTTTCTTTATAGTTATACCAATCATAATCGGAGGGTTCGGAAATTGA  196
            ||||||||||||||||| ||||||||||||||||||||||||||||| || |||||||||
Sbjct  121  GCATTTATTATAATTTTTTTTATAGTTATACCAATCATAATCGGAGGATTTGGAAATTGA  180

Query  197  TTAGTTCCTATTATATTAGGTGCCCCAGATATAGCATTTCCACGAATAAATAATATAAGA  256
            ||||| ||||| ||||| ||||||||||||||||||||||||||||||||||||||||||
Sbjct  181  TTAGTCCCTATCATATTGGGTGCCCCAGATATAGCATTTCCACGAATAAATAATATAAGA  240

Query  257  TTTTGACTTTTACCCCCCTCATTATTTTTATTGATTAACTCTTCATTAATTGAATCCGGA  316
            |||||||||||||| || ||| |||||||||| ||||||||||||||||||||||| |||
Sbjct  241  TTTTGACTTTTACCTCCTTCACTATTTTTATTAATTAACTCTTCATTAATTGAATCTGGA  300

Query  317  GCTGGAACAGGATGAACAGTTTATCCCCCTCTATCTTCAAATTTATCACATTATGGTCCA  376
            ||||| |||||||||||||||| || |||||||||||||||||||||||||||||| || 
Sbjct  301  GCTGGTACAGGATGAACAGTTTACCCTCCTCTATCTTCAAATTTATCACATTATGGCCCT  360

Query  377  TCAGTAGATTTAGCTATTTTTTCTCTTCACCTTGCTGGTGCTTCTTCAATTTTAGGTGCA  436
```

```
                 ||||||||||||||||| ||||||||||| ||||||||||||||||||||||||||||||
Sbjct  361   TCAGTAGATTTAGCTATCTTTTCTCTTCATCTTGCTGGTGCTTCTTCAATTTTAGGTGCA  420

Query  437   ATTAACTTCATCACAACTATTGTAAATATACGATCAATTGGAATAACAATAGAACGAATA  496
             |||||||| || |||||||||||||| ||||| ||||||||||||||||||||||| |||
Sbjct  421   ATTAACTTTATTACAACTATTGTAAACATACGGTCAATTGGAATAACAATAGAACGTATA  480

Query  497   CCATTATTTGTTTGATCTGTTTTAATTACAGCTATTTTACTACTTCTATCTTTACCTGTA  556
             ||||||||||||||||||||||||||||||||||||||| ||||| ||||||||||||||
Sbjct  481   CCATTATTTGTTTGATCTGTTTTAATTACAGCTATTTTATTACTTTTATCTTTACCTGTA  540

Query  557   TTAGCAGGTGCCATTACAATATTATTAACTGATCGAAACTTTAACACATCATTTTTTGAT  616
             ||||||||||||||||||||||| |||||||||||||||||||| |||||||||||||||
Sbjct  541   TTAGCAGGTGCCATTACAATATTGTTAACTGATCGAAACTTTAATACATCATTTTTTGAT  600

Query  617   CCTTCAGGAGGAGGAGATCCCATTTTATATCAACACTTATTT  658
             |||||||| ||||||||||| |||||||||||||| ||||||
Sbjct  601   CCTTCAGGGGGAGGAGATCCAATTTTATATCAACATTTATTT  642
```

>Rhipicephalus sanguineus sensu lato 'temperate lineage' isolate CR1568 cytochrome c oxidase subunit I (COI) gene, partial cds; mitochondrial
Sequence ID: MF426000.1 Length: 635
Range 1: 14 to 635

Score:1016 bits(550), Expect:0.0,
Identities:598/622(96%), Gaps:0/622(0%), Strand: Plus/Plus

```
Query  1     AACAATATATTTAATTTTTGGAGCATGATCAGGTATATTAGGACTTAGTATAAGAATATT  60
             ||||||||||||||||||||||||||||||||||||||||||||||||||||||||||||
Sbjct  14    AACAATATATTTAATTTTTGGAGCATGATCAGGTATATTAGGACTTAGTATAAGAATATT  73

Query  61    AATTCGTATAGAATTAGGACAACCTGGAACTTTAATTGGAAATGATCAGATTTATAATGT  120
             ||||||||||||||||||||||||||||||||||||||||||||||||||||||||||||
Sbjct  74    AATTCGTATAGAATTAGGACAACCTGGAACTTTAATTGGAAATGATCAGATTTATAATGT  133

Query  121   AATCGTAACAGCACATGCATTTATTATAATTTCTTTATAGTTATACCAATCATAATCGG  180
             |||||||||||||||||||||||||||||||| |||||||||||||||||||||||||||
Sbjct  134   AATCGTAACAGCACATGCATTTATTATAATTTTTTTATAGTTATACCAATCATAATCGG  193

Query  181   AGGGTTCGGAAATTGATTAGTTCCTATTATATTAGGTGCCCCAGATATAGCATTTCCACG  240
             ||| || |||||||||||||| ||||| ||||| ||||||||||||||||||||||||||
Sbjct  194   AGGATTTGGAAATTGATTAGTCCCTATCATATTGGGTGCCCCAGATATAGCATTTCCACG  253

Query  241   AATAAATAATATAAGATTTTGACTTTTACCCCCCTCATTATTTTTATTGATTAACTCTTC  300
             |||||||||||||||||||||||||||||| || ||| ||||||||||| ||||||||||
Sbjct  254   AATAAATAATATAAGATTTTGACTTTTACCTCCTTCACTATTTTTATTAATTAACTCTTC  313

Query  301   ATTAATTGAATCCGGAGCTGGAACAGGATGAACAGTTTATCCCCCTCTATCTTCAAATTT  360
             |||||||||||| |||||||| ||||||||||||||||| || |||||||||||||||||
Sbjct  314   ATTAATTGAATCTGGAGCTGGTACAGGATGAACAGTTTACCCTCCTCTATCTTCAAATTT  373
```

```
Query  361  ATCACATTATGGTCCATCAGTAGATTTAGCTATTTTTTCTCTTCACCTTGCTGGTGCTTC  420
            ||||||||||||||| ||||||||||||||||||||||||||||| ||||||||||||||
Sbjct  374  ATCACATTATGGTCCTTCAGTAGATTTAGCTATTTTTTCTCTTCATCTTGCTGGTGCTTC  433

Query  421  TTCAATTTTAGGTGCAATTAACTTCATCACAACTATTGTAAATATACGATCAATTGGAAT  480
            |||||||||||||||||||||||| || |||||||||||||| |||||||||||||||||
Sbjct  434  TTCAATTTTAGGTGCAATTAACTTTATTACAACTATTGTAAACATACGATCAATTGGAAT  493

Query  481  AACAATAGAACGAATACCATTATTTGTTTGATCTGTTTTAATTACAGCTATTTTACTACT  540
            |||||||||||| |||||||||||||||||||||||||||||||||||||||||| ||||
Sbjct  494  AACAATAGAACGTATACCATTATTTGTTTGATCTGTTTTAATTACAGCTATTTTATTACT  553

Query  541  TCTATCTTTACCTGTATTAGCAGGTGCCATTACAATATTATTAACTGATCGAAACTTTAA  600
            | ||||||||||||||||||||||||||||||||||||| ||||||||||||||||||||
Sbjct  554  TTTATCTTTACCTGTATTAGCAGGTGCCATTACAATATTGTTAACTGATCGAAACTTTAA  613

Query  601  CACATCATTTTTTGATCCTTCA  622
             |||||||||||||||||||||
Sbjct  614  TACATCATTTTTTGATCCTTCA  635
```

>Rhipicephalus sanguineus sensu lato 'temperate lineage' isolate SF3003 cytochrome c oxidase subunit I (COI) gene, partial cds; mitochondrial
Sequence ID: MF425988.1 Length: 654
Range 1: 1 to 640

Score:1016 bits(550), Expect:0.0,
Identities:610/640(95%), Gaps:0/640(0%), Strand: Plus/Plus

```
Query  17   TTTGGAGCATGATCAGGTATATTAGGACTTAGTATAAGAATATTAATTCGTATAGAATTA  76
            ||||||||||||||||||||||||||||||||||||||||||||||||||||||||||||
Sbjct  1    TTTGGAGCATGATCAGGTATATTAGGACTTAGTATAAGAATATTAATTCGTATAGAATTA  60

Query  77   GGACAACCTGGAACTTTAATTGGAAATGATCAGATTTATAATGTAATCGTAACAGCACAT  136
            ||||||||||||||||||||||||||||||||||||||||||||||||||||||||||||
Sbjct  61   GGACAACCTGGAACTTTAATTGGAAATGATCAGATTTATAATGTAATCGTAACAGCACAT  120

Query  137  GCATTTATTATAATTTTCTTTATAGTTATACCAATCATAATCGGAGGGTTCGGAAATTGA  196
            ||||||||||||||||| ||||||||||||||||||||||||||||| || |||||||||
Sbjct  121  GCATTTATTATAATTTTTTTTATAGTTATACCAATCATAATCGGAGGATTTGGAAATTGA  180

Query  197  TTAGTTCCTATTATATTAGGTGCCCCAGATATAGCATTTCCACGAATAAATAATATAAGA  256
            ||||| ||||| ||||||||||||||||||||||||||||||||||||||||||||||||
Sbjct  181  TTAGTCCCTATCATATTAGGTGCCCCAGATATAGCATTTCCACGAATAAATAATATAAGA  240

Query  257  TTTTGACTTTTACCCCCCTCATTATTTTTATTGATTAACTCTTCATTAATTGAATCCGGA  316
            |||||||||||||| || ||| |||||||||| ||||||||||||||||||||||| |||
Sbjct  241  TTTTGACTTTTACCTCCTTCACTATTTTTATTAATTAACTCTTCATTAATTGAATCTGGA  300

Query  317  GCTGGAACAGGATGAACAGTTTATCCCCCTCTATCTTCAAATTTATCACATTATGGTCCA  376
            ||||| ||||||||||||||||| || ||||||||||||||||||||||||||||| || 
Sbjct  301  GCTGGTACAGGATGAACAGTTTACCCTCCTCTATCTTCAAATTTATCACATTATGGCCCT  360
```

```
Query  377  TCAGTAGATTTAGCTATTTTTTCTCTTCACCTTGCTGGTGCTTCTTCAATTTTAGGTGCA  436
            ||||||||||||||||| ||||||||||| ||||||||||||||||||||||||||||||
Sbjct  361  TCAGTAGATTTAGCTATCTTTTCTCTTCATCTTGCTGGTGCTTCTTCAATTTTAGGTGCA  420

Query  437  ATTAACTTCATCACAACTATTGTAAATATACGATCAATTGGAATAACAATAGAACGAATA  496
            |||||||| || |||||||||||||| ||||||||||||||||||||||||||||| |||
Sbjct  421  ATTAACTTTATTACAACTATTGTAAACATACGATCAATTGGAATAACAATAGAACGTATA  480

Query  497  CCATTATTTGTTTGATCTGTTTTAATTACAGCTATTTTACTACTTCTATCTTTACCTGTA  556
            ||||||||||||||||||||||||||||||||||||||| ||||| ||||||||||||||
Sbjct  481  CCATTATTTGTTTGATCTGTTTTAATTACAGCTATTTTATTACTTTTATCTTTACCTGTA  540

Query  557  TTAGCAGGTGCCATTACAATATTATTAACTGATCGAAACTTTAACACATCATTTTTGAT  616
            ||||||||||||||||||||||| |||||||||||||||||||| |||||||||||||||
Sbjct  541  TTAGCAGGTGCCATTACAATATTGTTAACTGATCGAAACTTTAATACATCATTTTTGAT  600

Query  617  CCTTCAGGAGGAGGAGATCCCATTTTATATCAACACTTAT  656
            |||||||| |||||| |||| ||||||||||| || ||||
Sbjct  601  CCTTCAGGGGGAGGACATCCAATTTTATATCACCATTTAT  640
```

>Rhipicephalus sanguineus isolate LIC5533B cytochrome oxidase subunit 1 (COI) gene, partial cds; mitochondrial
Sequence ID: KX383811.1 Length: 764
>Rhipicephalus sanguineus isolate LIC5533C cytochrome oxidase subunit 1 (COI) gene, partial cds; mitochondrial
Sequence ID: KX383812.1 Length: 764
Range 1: 1 to 638

Score:1013 bits(548), Expect:0.0,
Identities:608/638(95%), Gaps:0/638(0%), Strand: Plus/Plus

```
Query  21   GAGCATGATCAGGTATATTAGGACTTAGTATAAGAATATTAATTCGTATAGAATTAGGAC  80
            ||||||||||||||||||||||||||||||||||||||||||||||||||||||||||||
Sbjct  1    GAGCATGATCAGGTATATTAGGACTTAGTATAAGAATATTAATTCGTATAGAATTAGGAC  60

Query  81   AACCTGGAACTTTAATTGGAAATGATCAGATTTATAATGTAATCGTAACAGCACATGCAT  140
            ||||||||||||||||||||||||||||||||||||||||||||||||||||||||||||
Sbjct  61   AACCTGGAACTTTAATTGGAAATGATCAGATTTATAATGTAATCGTAACAGCACATGCAT  120

Query  141  TTATTATAATTTTCTTTATAGTTATACCAATCATAATCGGAGGGTTCGGAAATTGATTAG  200
            ||||||||||||| ||||||||||||||||||||||||||||| || |||||||||||||
Sbjct  121  TTATTATAATTTTTTTTATAGTTATACCAATCATAATCGGAGGATTTGGAAATTGATTAG  180

Query  201  TTCCTATTATATTAGGTGCCCCAGATATAGCATTTCCACGAATAAATAATATAAGATTTT  260
            | ||||| ||||| ||||||||||||||||||||||||||||||||||||||||||||||
Sbjct  181  TCCCTATCATATTGGGTGCCCCAGATATAGCATTTCCACGAATAAATAATATAAGATTTT  240

Query  261  GACTTTTACCCCCCTCATTATTTTTATTGATTAACTCTTCATTAATTGAATCCGGAGCTG  320
            |||||||||| || ||| |||||||||| ||||||||||||||||||||||| |||||||
Sbjct  241  GACTTTTACCTCCTTCACTATTTTTATTAATTAACTCTTCATTAATTGAATCTGGAGCTG  300
```

```
Query  321   GAACAGGATGAACAGTTTATCCCCCTCTATCTTCAAATTTATCACATTATGGTCCATCAG  380
             | ||||||||||||||||| || ||||||||||||||||||||||||||||| || ||||
Sbjct  301   GTACAGGATGAACAGTTTACCCTCCTCTATCTTCAAATTTATCACATTATGGCCCTTCAG  360

Query  381   TAGATTTAGCTATTTTTTCTCTTCACCTTGCTGGTGCTTCTTCAATTTTAGGTGCAATTA  440
             ||||||||||||| ||||||||||| ||||||||||||||||||||||||||||||||||
Sbjct  361   TAGATTTAGCTATCTTTTCTCTTCATCTTGCTGGTGCTTCTTCAATTTTAGGTGCAATTA  420

Query  441   ACTTCATCACAACTATTGTAAATATACGATCAATTGGAATAACAATAGAACGAATACCAT  500
             |||| || ||||||||||||||| |||| ||||||||||||||||||||||| |||||||
Sbjct  421   ACTTTATTACAACTATTGTAAACATACGGTCAATTGGAATAACAATAGAACGTATACCAT  480

Query  501   TATTTGTTTGATCTGTTTTAATTACAGCTATTTTACTACTTCTATCTTTACCTGTATTAG  560
             ||||||||||||||||||||||||||||||||||| ||||| ||||||||||||||||||
Sbjct  481   TATTTGTTTGATCTGTTTTAATTACAGCTATTTTATTACTTTTATCTTTACCTGTATTAG  540

Query  561   CAGGTGCCATTACAATATTATTAACTGATCGAAACTTTAACACATCATTTTTTGATCCTT  620
             ||||||||||||||||||| |||||||||||||||||||| |||||||||||||||||||
Sbjct  541   CAGGTGCCATTACAATATTGTTAACTGATCGAAACTTTAATACATCATTTTTTGATCCTT  600

Query  621   CAGGAGGAGGAGATCCCATTTTATATCAACACTTATTT  658
             |||| ||||||||||| |||||||||||||| ||||||
Sbjct  601   CAGGGGGAGGAGATCCAATTTTATATCAACATTTATTT  638
```

>Rhipicephalus sanguineus isolate LIC4736B cytochrome oxidase subunit 1 (COI) gene, partial cds; mitochondrial
Sequence ID: KX383807.1 Length: 763
>Rhipicephalus sanguineus isolate LIC4736C cytochrome oxidase subunit 1 (COI) gene, partial cds; mitochondrial
Sequence ID: KX383808.1 Length: 763
>Rhipicephalus sanguineus isolate LIC4736F cytochrome oxidase subunit 1 (COI) gene, partial cds; mitochondrial
Sequence ID: KX383810.1 Length: 763
>Rhipicephalus sanguineus isolate LIC5535C cytochrome oxidase subunit 1 (COI) gene, partial cds; mitochondrial
Sequence ID: KX383813.1 Length: 763
Range 1: 1 to 637

Score:1011 bits(547), Expect:0.0,
Identities:607/637(95%), Gaps:0/637(0%), Strand: Plus/Plus

```
Query  22    AGCATGATCAGGTATATTAGGACTTAGTATAAGAATATTAATTCGTATAGAATTAGGACA  81
             ||||||||||||||||||||||||||||||||||||||||||||||||||||||||||||
Sbjct  1     AGCATGATCAGGTATATTAGGACTTAGTATAAGAATATTAATTCGTATAGAATTAGGACA  60

Query  82    ACCTGGAACTTTAATTGGAAATGATCAGATTTATAATGTAATCGTAACAGCACATGCATT  141
             ||||||||||||||||||||||||||||||||||||||||||||||||||||||||||||
Sbjct  61    ACCTGGAACTTTAATTGGAAATGATCAGATTTATAATGTAATCGTAACAGCACATGCATT  120

Query  142   TATTATAATTTTCTTTATAGTTATACCAATCATAATCGGAGGGTTCGGAAATTGATTAGT  201
```

```
             |||||||||||| ||||||||||||||||||||||||||||| || ||||||||||||||
Sbjct  121   TATTATAATTTTTTTTATAGTTATACCAATCATAATCGGAGGATTTGGAAATTGATTAGT  180

Query  202   TCCTATTATATTAGGTGCCCCAGATATAGCATTTCCACGAATAAATAATATAAGATTTTG  261
              ||||| ||||| |||||||||||||||||||||||||||||||||||||||||||||||
Sbjct  181   CCCTATCATATTGGGTGCCCCAGATATAGCATTTCCACGAATAAATAATATAAGATTTTG  240

Query  262   ACTTTTACCCCCCTCATTATTTTTATTGATTAACTCTTCATTAATTGAATCCGGAGCTGG  321
             ||||||||| || ||| |||||||||| ||||||||||||||||||||||| ||||||||
Sbjct  241   ACTTTTACCTCCTTCACTATTTTTATTAATTAACTCTTCATTAATTGAATCTGGAGCTGG  300

Query  322   AACAGGATGAACAGTTTATCCCCCTCTATCTTCAAATTTATCACATTATGGTCCATCAGT  381
              ||||||||||||||||| || ||||||||||||||||||||||||||||| || |||||
Sbjct  301   TACAGGATGAACAGTTTACCCTCCTCTATCTTCAAATTTATCACATTATGGCCCTTCAGT  360

Query  382   AGATTTAGCTATTTTTTCTCTTCACCTTGCTGGTGCTTCTTCAATTTTAGGTGCAATTAA  441
             |||||||||||| ||||||||||| |||||||||||||||||||||||||||||||||||
Sbjct  361   AGATTTAGCTATCTTTTCTCTTCATCTTGCTGGTGCTTCTTCAATTTTAGGTGCAATTAA  420

Query  442   CTTCATCACAACTATTGTAAATATACGATCAATTGGAATAACAATAGAACGAATACCATT  501
             ||| || |||||||||||||| ||||| ||||||||||||||||||||||| ||||||||
Sbjct  421   CTTTATTACAACTATTGTAAACATACGGTCAATTGGAATAACAATAGAACGTATACCATT  480

Query  502   ATTTGTTTGATCTGTTTTAATTACAGCTATTTTACTACTTCTATCTTTACCTGTATTAGC  561
             |||||||||||||||||||||||||||||||||| ||||| |||||||||||||||||||
Sbjct  481   ATTTGTTTGATCTGTTTTAATTACAGCTATTTTATTACTTTTATCTTTACCTGTATTAGC  540

Query  562   AGGTGCCATTACAATATTATTAACTGATCGAAACTTTAACACATCATTTTTTGATCCTTC  621
             |||||||||||||||||| |||||||||||||||||||| ||||||||||||||||||||
Sbjct  541   AGGTGCCATTACAATATTGTTAACTGATCGAAACTTTAATACATCATTTTTTGATCCTTC  600

Query  622   AGGAGGAGGAGATCCCATTTTATATCAACACTTATTT  658
             ||| ||||||||||| |||||||||||||| ||||||
Sbjct  601   AGGGGGAGGAGATCCAATTTTATATCAACATTTATTT  637
```

>Rhipicephalus sanguineus voucher Rs7 cytochrome oxidase subunit I (COI) gene, partial cds; mitochondrial
Sequence ID: KU568516.1 Length: 656
Range 1: 27 to 656

Score:1009 bits(546), Expect:0.0,
Identities:602/630(96%), Gaps:0/630(0%), Strand: Plus/Plus

```
Query  1     AACAATATATTTAATTTTTGGAGCATGATCAGGTATATTAGGACTTAGTATAAGAATATT  60
             ||||||||||||||||||||||||||||||||||||||||||||||||||||||||||||
Sbjct  27    AACAATATATTTAATTTTTGGAGCATGATCAGGTATATTAGGACTTAGTATAAGAATATT  86

Query  61    AATTCGTATAGAATTAGGACAACCTGGAACTTTAATTGGAAATGATCAGATTTATAATGT  120
             ||||||||||||||||||||||||||||||||||||||||||||||||||||||||||||
Sbjct  87    AATTCGTATAGAATTAGGACAACCTGGAACTTTAATTGGAAATGATCAGATTTATAATGT  146
```

```
Query  121  AATCGTAACAGCACATGCATTTATTATAATTTTCTTTATAGTTATACCAATCATAATCGG  180
            ||||||||||||||||||||||||||||||||| ||||||||||||||||||||||||||
Sbjct  147  AATCGTAACAGCACATGCATTTATTATAATTTTTTTTATAGTTATACCAATCATAATCGG  206

Query  181  AGGGTTCGGAAATTGATTAGTTCCTATTATATTAGGTGCCCCAGATATAGCATTTCCACG  240
            ||| || |||||||||||| | ||||| ||||||||||||||||||||||||||||||||
Sbjct  207  AGGATTTGGAAATTGATTAATCCCTATCATATTAGGTGCCCCAGATATAGCATTTCCACG  266

Query  241  AATAAATAATATAAGATTTTGACTTTTACCCCCCTCATTATTTTTATTGATTAACTCTTC  300
            |||||||||||||||||||||||||||||| || ||| |||||||||| |||||||||||
Sbjct  267  AATAAATAATATAAGATTTTGACTTTTACCTCCTTCACTATTTTTATTAATTAACTCTTC  326

Query  301  ATTAATTGAATCCGGAGCTGGAACAGGATGAACAGTTTATCCCCCTCTATCTTCAAATTT  360
            |||||||||||| |||||||| ||||||||||||||||| || |||||||||||||||||
Sbjct  327  ATTAATTGAATCTGGAGCTGGTACAGGATGAACAGTTTACCCTCCTCTATCTTCAAATTT  386

Query  361  ATCACATTATGGTCCATCAGTAGATTTAGCTATTTTTTCTCTTCACCTTGCTGGTGCTTC  420
            |||||||||||| || ||||||||||||||||| ||||||||||| ||||||||||||||
Sbjct  387  ATCACATTATGGCCCTTCAGTAGATTTAGCTATCTTTTCTCTTCATCTTGCTGGTGCTTC  446

Query  421  TTCAATTTTAGGTGCAATTAACTTCATCACAACTATTGTAAATATACGATCAATTGGAAT  480
            |||||||||||||||||||||||| || |||||||||||||| |||||||||||||||||
Sbjct  447  TTCAATTTTAGGTGCAATTAACTTTATTACAACTATTGTAAACATACGATCAATTGGAAT  506

Query  481  AACAATAGAACGAATACCATTATTTGTTTGATCTGTTTTAATTACAGCTATTTTACTACT  540
            |||||||||||| |||||||||||||||||||||||||||||||||||||||||| ||||
Sbjct  507  AACAATAGAACGTATACCATTATTTGTTTGATCTGTTTTAATTACAGCTATTTTATTACT  566

Query  541  TCTATCTTTACCTGTATTAGCAGGTGCCATTACAATATTATTAACTGATCGAAACTTTAA  600
              ||||||||||||||||||||||||||||||||||||| ||||||||||||||||||||
Sbjct  567  CTTATCTTTACCTGTATTAGCAGGTGCCATTACAATATTGTTAACTGATCGAAACTTTAA  626

Query  601  CACATCATTTTTTGATCCTTCAGGAGGAGG  630
             |||||||||||||| ||||||||||||||
Sbjct  627  TACATCATTTTTTGACCCTTCAGGAGGAGG  656
```

>Rhipicephalus sanguineus isolate LIC4736D cytochrome oxidase subunit 1 (COI) gene, partial cds; mitochondrial
Sequence ID: KX383809.1 Length: 762
Range 1: 1 to 636

Score:1009 bits(546), Expect:0.0,
Identities:606/636(95%), Gaps:0/636(0%), Strand: Plus/Plus

```
Query  23   GCATGATCAGGTATATTAGGACTTAGTATAAGAATATTAATTCGTATAGAATTAGGACAA  82
            ||||||||||||||||||||||||||||||||||||||||||||||||||||||||||||
Sbjct  1    GCATGATCAGGTATATTAGGACTTAGTATAAGAATATTAATTCGTATAGAATTAGGACAA  60

Query  83   CCTGGAACTTTAATTGGAAATGATCAGATTTATAATGTAATCGTAACAGCACATGCATTT  142
            ||||||||||||||||||||||||||||||||||||||||||||||||||||||||||||
Sbjct  61   CCTGGAACTTTAATTGGAAATGATCAGATTTATAATGTAATCGTAACAGCACATGCATTT  120
```

```
Query  143  ATTATAATTTTCTTTATAGTTATACCAATCATAATCGGAGGGTTCGGAAATTGATTAGTT  202
            ||||||||||| ||||||||||||||||||||||||||||| || ||||||||||||||
Sbjct  121  ATTATAATTTTTTTTATAGTTATACCAATCATAATCGGAGGATTTGGAAATTGATTAGTC  180

Query  203  CCTATTATATTAGGTGCCCCAGATATAGCATTTCCACGAATAAATAATATAAGATTTTGA  262
            ||||| ||||| ||||||||||||||||||||||||||||||||||||||||||||||||
Sbjct  181  CCTATCATATTGGGTGCCCCAGATATAGCATTTCCACGAATAAATAATATAAGATTTTGA  240

Query  263  CTTTTACCCCCCTCATTATTTTTATTGATTAACTCTTCATTAATTGAATCCGGAGCTGGA  322
            |||||||| || ||| |||||||||| ||||||||||||||||||||||| ||||||||
Sbjct  241  CTTTTACCTCCTTCACTATTTTTATTAATTAACTCTTCATTAATTGAATCTGGAGCTGGT  300

Query  323  ACAGGATGAACAGTTTATCCCCCTCTATCTTCAAATTTATCACATTATGGTCCATCAGTA  382
            ||||||||||||||||| || ||||||||||||||||||||||||||||| || ||||||
Sbjct  301  ACAGGATGAACAGTTTACCCTCCTCTATCTTCAAATTTATCACATTATGGCCCTTCAGTA  360

Query  383  GATTTAGCTATTTTTTCTCTTCACCTTGCTGGTGCTTCTTCAATTTTAGGTGCAATTAAC  442
            ||||||||||| ||||||||||| ||||||||||||||||||||||||||||||||||||
Sbjct  361  GATTTAGCTATCTTTTCTCTTCATCTTGCTGGTGCTTCTTCAATTTTAGGTGCAATTAAC  420

Query  443  TTCATCACAACTATTGTAAATATACGATCAATTGGAATAACAATAGAACGAATACCATTA  502
            || || |||||||||||||| ||||| ||||||||||||||||||||||| |||||||||
Sbjct  421  TTTATTACAACTATTGTAAACATACGGTCAATTGGAATAACAATAGAACGTATACCATTA  480

Query  503  TTTGTTTGATCTGTTTTAATTACAGCTATTTTACTACTTCTATCTTTACCTGTATTAGCA  562
            ||||||||||||||||||||||||||||||||| ||||| ||||||||||||||||||||
Sbjct  481  TTTGTTTGATCTGTTTTAATTACAGCTATTTTATTACTTTTATCTTTACCTGTATTAGCA  540

Query  563  GGTGCCATTACAATATTATTAACTGATCGAAACTTTAACACATCATTTTTTGATCCTTCA  622
            ||||||||||||||||| |||||||||||||||||||| |||||||||||||||||||||
Sbjct  541  GGTGCCATTACAATATTGTTAACTGATCGAAACTTTAATACATCATTTTTTGATCCTTCA  600

Query  623  GGAGGAGGAGATCCCATTTTATATCAACACTTATTT  658
            || ||||||||||| |||||||||||||| ||||||
Sbjct  601  GGGGGAGGAGATCCAATTTTATATCAACATTTATTT  636
```

>Rhipicephalus sanguineus isolate LIC4727D cytochrome oxidase subunit 1 (COI) gene, partial cds; mitochondrial
Sequence ID: KX383806.1 Length: 714
Range 1: 1 to 636

Score:1009 bits(546), Expect:0.0,
Identities:606/636(95%), Gaps:0/636(0%), Strand: Plus/Plus

```
Query  23   GCATGATCAGGTATATTAGGACTTAGTATAAGAATATTAATTCGTATAGAATTAGGACAA  82
            ||||||||||||||||||||||||||||||||||||||||||||||||||||||||||||
Sbjct  1    GCATGATCAGGTATATTAGGACTTAGTATAAGAATATTAATTCGTATAGAATTAGGACAA  60

Query  83   CCTGGAACTTTAATTGGAAATGATCAGATTTATAATGTAATCGTAACAGCACATGCATTT  142
            ||||||||||||||||||||||||||||||||||||||||||||||||||||||||||||
```

```
Sbjct  61    CCTGGAACTTTAATTGGAAATGATCAGATTTATAATGTAATCGTAACAGCACATGCATTT  120

Query  143   ATTATAATTTTCTTTATAGTTATACCAATCATAATCGGAGGGTTCGGAAATTGATTAGTT  202
             ||||||||||| ||||||||||||||||||||||||||||| || ||||||||||||||
Sbjct  121   ATTATAATTTTTTTTATAGTTATACCAATCATAATCGGAGGATTTGGAAATTGATTAGTC  180

Query  203   CCTATTATATTAGGTGCCCCAGATATAGCATTTCCACGAATAAATAATATAAGATTTTGA  262
             ||||| ||||| ||||||||||||||||||||||||||||||||||||||||||||||||
Sbjct  181   CCTATCATATTGGGTGCCCCAGATATAGCATTTCCACGAATAAATAATATAAGATTTTGA  240

Query  263   CTTTTACCCCCCTCATTATTTTTATTGATTAACTCTTCATTAATTGAATCCGGAGCTGGA  322
             |||||||| || ||| |||||||||| ||||||||||||||||||||||| ||||||||
Sbjct  241   CTTTTACCTCCTTCACTATTTTTATTAATTAACTCTTCATTAATTGAATCTGGAGCTGGT  300

Query  323   ACAGGATGAACAGTTTATCCCCCTCTATCTTCAAATTTATCACATTATGGTCCATCAGTA  382
             ||||||||||||||||| || ||||||||||||||||||||||||||||| || ||||||
Sbjct  301   ACAGGATGAACAGTTTACCCTCCTCTATCTTCAAATTTATCACATTATGGCCCTTCAGTA  360

Query  383   GATTTAGCTATTTTTTCTCTTCACCTTGCTGGTGCTTCTTCAATTTTAGGTGCAATTAAC  442
             ||||||||||| ||||||||||| ||||||||||||||||||||||||||||||||||||
Sbjct  361   GATTTAGCTATCTTTTCTCTTCATCTTGCTGGTGCTTCTTCAATTTTAGGTGCAATTAAC  420

Query  443   TTCATCACAACTATTGTAAATATACGATCAATTGGAATAACAATAGAACGAATACCATTA  502
             || || |||||||||||||| ||||| ||||||||||||||||||||||| |||||||||
Sbjct  421   TTTATTACAACTATTGTAAACATACGGTCAATTGGAATAACAATAGAACGTATACCATTA  480

Query  503   TTTGTTTGATCTGTTTTAATTACAGCTATTTTACTACTTCTATCTTTACCTGTATTAGCA  562
             ||||||||||||||||||||||||||||||||| ||||| ||||||||||||||||||||
Sbjct  481   TTTGTTTGATCTGTTTTAATTACAGCTATTTTATTACTTTTATCTTTACCTGTATTAGCA  540

Query  563   GGTGCCATTACAATATTATTAACTGATCGAAACTTTAACACATCATTTTTTGATCCTTCA  622
             ||||||||||||||||| |||||||||||||||||||| |||||||||||||||||||||
Sbjct  541   GGTGCCATTACAATATTGTTAACTGATCGAAACTTTAATACATCATTTTTTGATCCTTCA  600

Query  623   GGAGGAGGAGATCCCATTTTATATCAACACTTATTT  658
             || ||||||||||| |||||||||||||| ||||||
Sbjct  601   GGGGGAGGAGATCCAATTTTATATCAACATTTATTT  636
```

>Rhipicephalus sanguineus isolate LIC4727C cytochrome oxidase subunit 1 (COI) gene, partial cds; mitochondrial
Sequence ID: KX383805.1 Length: 761
Range 1: 1 to 635

Score:1007 bits(545), Expect:0.0,
Identities:605/635(95%), Gaps:0/635(0%), Strand: Plus/Plus

```
Query  24    CATGATCAGGTATATTAGGACTTAGTATAAGAATATTAATTCGTATAGAATTAGGACAAC  83
             ||||||||||||||||||||||||||||||||||||||||||||||||||||||||||||
Sbjct  1     CATGATCAGGTATATTAGGACTTAGTATAAGAATATTAATTCGTATAGAATTAGGACAAC  60

Query  84    CTGGAACTTTAATTGGAAATGATCAGATTTATAATGTAATCGTAACAGCACATGCATTTA  143
```

```
                 ||||||||||||||||||||||||||||||||||||||||||||||||||||||||||||
Sbjct  61    CTGGAACTTTAATTGGAAATGATCAGATTTATAATGTAATCGTAACAGCACATGCATTTA  120

Query  144   TTATAATTTTCTTTATAGTTATACCAATCATAATCGGAGGGTTCGGAAATTGATTAGTTC  203
             |||||||||| ||||||||||||||||||||||||||||| || |||||||||||||| |
Sbjct  121   TTATAATTTTTTTTATAGTTATACCAATCATAATCGGAGGATTTGGAAATTGATTAGTCC  180

Query  204   CTATTATATTAGGTGCCCCAGATATAGCATTTCCACGAATAAATAATATAAGATTTTGAC  263
             |||| ||||| |||||||||||||||||||||||||||||||||||||||||||||||||
Sbjct  181   CTATCATATTGGGTGCCCCAGATATAGCATTTCCACGAATAAATAATATAAGATTTTGAC  240

Query  264   TTTTACCCCCCTCATTATTTTTATTGATTAACTCTTCATTAATTGAATCCGGAGCTGGAA  323
             ||||||| || ||| |||||||||| ||||||||||||||||||||||| |||||||| |
Sbjct  241   TTTTACCTCCTTCACTATTTTTATTAATTAACTCTTCATTAATTGAATCTGGAGCTGGTA  300

Query  324   CAGGATGAACAGTTTATCCCCCTCTATCTTCAAATTTATCACATTATGGTCCATCAGTAG  383
             |||||||||||||||| || ||||||||||||||||||||||||||||| || |||||||
Sbjct  301   CAGGATGAACAGTTTACCCTCCTCTATCTTCAAATTTATCACATTATGGCCCTTCAGTAG  360

Query  384   ATTTAGCTATTTTTTCTCTTCACCTTGCTGGTGCTTCTTCAATTTTAGGTGCAATTAACT  443
             |||||||||| ||||||||||| |||||||||||||||||||||||||||||||||||||
Sbjct  361   ATTTAGCTATCTTTTCTCTTCATCTTGCTGGTGCTTCTTCAATTTTAGGTGCAATTAACT  420

Query  444   TCATCACAACTATTGTAAATATACGATCAATTGGAATAACAATAGAACGAATACCATTAT  503
             | || |||||||||||||| ||||| ||||||||||||||||||||||| ||||||||||
Sbjct  421   TTATTACAACTATTGTAAACATACGGTCAATTGGAATAACAATAGAACGTATACCATTAT  480

Query  504   TTGTTTGATCTGTTTTAATTACAGCTATTTTACTACTTCTATCTTTACCTGTATTAGCAG  563
             |||||||||||||||||||||||||||||||| ||||| |||||||||||||||||||||
Sbjct  481   TTGTTTGATCTGTTTTAATTACAGCTATTTTATTACTTTTATCTTTACCTGTATTAGCAG  540

Query  564   GTGCCATTACAATATTATTAACTGATCGAAACTTTAACACATCATTTTTTGATCCTTCAG  623
             |||||||||||||||| |||||||||||||||||||| ||||||||||||||||||||||
Sbjct  541   GTGCCATTACAATATTGTTAACTGATCGAAACTTTAATACATCATTTTTTGATCCTTCAG  600

Query  624   GAGGAGGAGATCCCATTTTATATCAACACTTATTT  658
             | ||||||||||| |||||||||||||| ||||||
Sbjct  601   GGGGAGGAGATCCAATTTTATATCAACATTTATTT  635
```

>Rhipicephalus sanguineus isolate Samont2 cytochrome oxidase subunit I (cox1) gene, partial cds; mitochondrial
Sequence ID: MH630347.1 Length: 644
Range 1: 20 to 641

Score:1005 bits(544), Expect:0.0,
Identities:596/622(96%), Gaps:0/622(0%), Strand: Plus/Plus

```
Query  1     AACAATATATTTAATTTTTGGAGCATGATCAGGTATATTAGGACTTAGTATAAGAATATT  60
             ||||||||||||||||||||||||||||||||||||||||||||||||||||||||||||
Sbjct  20    AACAATATATTTAATTTTTGGAGCATGATCAGGTATATTAGGACTTAGTATAAGAATATT  79
```

```
Query  61   AATTCGTATAGAATTAGGACAACCTGGAACTTTAATTGGAAATGATCAGATTTATAATGT  120
            |||| |||||||||||||||||||||||||||||||||||||||||||||||||||||||
Sbjct  80   AATTGGTATAGAATTAGGACAACCTGGAACTTTAATTGGAAATGATCAGATTTATAATGT  139

Query  121  AATCGTAACAGCACATGCATTTATTATAATTTTCTTTATAGTTATACCAATCATAATCGG  180
            ||||||||||||||||||||||||||||||||| ||||||||||||||||||||||||||
Sbjct  140  AATCGTAACAGCACATGCATTTATTATAATTTTTTTTATAGTTATACCAATCATAATCGG  199

Query  181  AGGGTTCGGAAATTGATTAGTTCCTATTATATTAGGTGCCCCAGATATAGCATTTCCACG  240
            ||| || |||||||||||||| ||||| ||||| ||||||||||||||||||||||||||
Sbjct  200  AGGATTTGGAAATTGATTAGTCCCTATCATATTGGGTGCCCCAGATATAGCATTTCCACG  259

Query  241  AATAAATAATATAAGATTTTGACTTTTACCCCCCTCATTATTTTTATTGATTAACTCTTC  300
            |||||||||||||||||||||||||||||| || ||| |||||||||| |||||||||||
Sbjct  260  AATAAATAATATAAGATTTTGACTTTTACCTCCTTCACTATTTTTATTAATTAACTCTTC  319

Query  301  ATTAATTGAATCCGGAGCTGGAACAGGATGAACAGTTTATCCCCCTCTATCTTCAAATTT  360
            |||||||||||| |||||||| ||||||||||||||||| || |||||||||||||||||
Sbjct  320  ATTAATTGAATCTGGAGCTGGTACAGGATGAACAGTTTACCCTCCTCTATCTTCAAATTT  379

Query  361  ATCACATTATGGTCCATCAGTAGATTTAGCTATTTTTTCTCTTCACCTTGCTGGTGCTTC  420
            |||||||||||| || ||||||||||||||||||||||||||||| ||||||||||||||
Sbjct  380  ATCACATTATGGCCCTTCAGTAGATTTAGCTATTTTTTCTCTTCATCTTGCTGGTGCTTC  439

Query  421  TTCAATTTTAGGTGCAATTAACTTCATCACAACTATTGTAAATATACGATCAATTGGAAT  480
            ||||||||||||||||||||||||| || |||||||||||||| ||||||||||||||||
Sbjct  440  TTCAATTTTAGGTGCAATTAACTTTATTACAACTATTGTAAACATACGATCAATTGGAAT  499

Query  481  AACAATAGAACGAATACCATTATTTGTTTGATCTGTTTTAATTACAGCTATTTTACTACT  540
            |||||||||||| |||||||||||||||||||||||||||||||||||||||||| ||||
Sbjct  500  AACAATAGAACGTATACCATTATTTGTTTGATCTGTTTTAATTACAGCTATTTTATTACT  559

Query  541  TCTATCTTTACCTGTATTAGCAGGTGCCATTACAATATTATTAACTGATCGAAACTTTAA  600
            | |||||||||||||||||||||||||||||||||||||| |||||||||||||||||||
Sbjct  560  TTTATCTTTACCTGTATTAGCAGGTGCCATTACAATATTGTTAACTGATCGAAACTTTAA  619

Query  601  CACATCATTTTTTGATCCTTCA  622
             |||||||||||||||||||||
Sbjct  620  TACATCATTTTTTGATCCTTCA  641
```

>Rhipicephalus sanguineus isolate Samont1 cytochrome oxidase subunit I (cox1) gene, partial cds; mitochondrial
Sequence ID: MH630346.1 Length: 644
Range 1: 20 to 641

Score:1005 bits(544), Expect:0.0,
Identities:596/622(96%), Gaps:0/622(0%), Strand: Plus/Plus

```
Query  1    AACAATATATTTAATTTTTGGAGCATGATCAGGTATATTAGGACTTAGTATAAGAATATT  60
            ||||||||||||||||||||||||||||||||||||||||||||||||||||||||||||
Sbjct  20   AACAATATATTTAATTTTTGGAGCATGATCAGGTATATTAGGACTTAGTATAAGAATATT  79
```

```
Query  61    AATTCGTATAGAATTAGGACAACCTGGAACTTTAATTGGAAATGATCAGATTTATAATGT  120
             |||| |||||||||||||||||||||||||||||||||||||||||||||||||||||||
Sbjct  80    AATTGGTATAGAATTAGGACAACCTGGAACTTTAATTGGAAATGATCAGATTTATAATGT  139

Query  121   AATCGTAACAGCACATGCATTTATTATAATTTTCTTTATAGTTATACCAATCATAATCGG  180
             ||||||||||||||||||||||||||||||||| ||||||||||||||||||||||||||
Sbjct  140   AATCGTAACAGCACATGCATTTATTATAATTTTTTTTATAGTTATACCAATCATAATCGG  199

Query  181   AGGGTTCGGAAATTGATTAGTTCCTATTATATTAGGTGCCCCAGATATAGCATTTCCACG  240
             ||| || |||||||||||||| ||||| ||||| ||||||||||||||||||||||||||
Sbjct  200   AGGATTTGGAAATTGATTAGTCCCTATCATATTGGGTGCCCCAGATATAGCATTTCCACG  259

Query  241   AATAAATAATATAAGATTTTGACTTTTACCCCCCTCATTATTTTTATTGATTAACTCTTC  300
             |||||||||||||||||||||||||||||| || ||| |||||||||| |||||||||||
Sbjct  260   AATAAATAATATAAGATTTTGACTTTTACCTCCTTCACTATTTTTATTAATTAACTCTTC  319

Query  301   ATTAATTGAATCCGGAGCTGGAACAGGATGAACAGTTTATCCCCCTCTATCTTCAAATTT  360
             |||||||||||| |||||||| ||||||||||||||||| || |||||||||||||||||
Sbjct  320   ATTAATTGAATCTGGAGCTGGTACAGGATGAACAGTTTACCCTCCTCTATCTTCAAATTT  379

Query  361   ATCACATTATGGTCCATCAGTAGATTTAGCTATTTTTTCTCTTCACCTTGCTGGTGCTTC  420
             |||||||||||| || ||||||||||||||||||||||||||||| ||||||||||||||
Sbjct  380   ATCACATTATGGCCCTTCAGTAGATTTAGCTATTTTTTCTCTTCATCTTGCTGGTGCTTC  439

Query  421   TTCAATTTTAGGTGCAATTAACTTCATCACAACTATTGTAAATATACGATCAATTGGAAT  480
             |||||||||||||||||||||||| || ||||||||||||||| ||||||||||||||||
Sbjct  440   TTCAATTTTAGGTGCAATTAACTTTATTACAACTATTGTAAACATACGATCAATTGGAAT  499

Query  481   AACAATAGAACGAATACCATTATTTGTTTGATCTGTTTTAATTACAGCTATTTTACTACT  540
             |||||||||||| |||||||||||||||||||||||||||||||||||||||||| ||||
Sbjct  500   AACAATAGAACGTATACCATTATTTGTTTGATCTGTTTTAATTACAGCTATTTTATTACT  559

Query  541   TCTATCTTTACCTGTATTAGCAGGTGCCATTACAATATTATTAACTGATCGAAACTTTAA  600
             | ||||||||||||||||||||||||||||||||||||| ||||||||||||||||||||
Sbjct  560   TTTATCTTTACCTGTATTAGCAGGTGCCATTACAATATTGTTAACTGATCGAAACTTTAA  619

Query  601   CACATCATTTTTTGATCCTTCA  622
              |||||||||||||||||||||
Sbjct  620   TACATCATTTTTTGATCCTTCA  641
```

>Rhipicephalus sanguineus clone #7 cytochrome oxidase subunit I (COI) gene, partial cds; mitochondrial
Sequence ID: KX519712.1 Length: 654
Range 1: 3 to 628

Score:1002 bits(542), Expect:0.0,
Identities:598/626(96%), Gaps:0/626(0%), Strand: Plus/Plus

```
Query  33    GTATATTAGGACTTAGTATAAGAATATTAATTCGTATAGAATTAGGACAACCTGGAACTT  92
             ||||||||||||||||||||||||||||||||||||||||||||||||||||||||||||
```

```
Sbjct  3    GTATATTAGGACTTAGTATAAGAATATTAATTCGTATAGAATTAGGACAACCTGGAACTT  62

Query  93   TAATTGGAAATGATCAGATTTATAATGTAATCGTAACAGCACATGCATTTATTATAATTT  152
            ||||||||||||||||||||||||||||||||||||||||||||||||||||||||||||
Sbjct  63   TAATTGGAAATGATCAGATTTATAATGTAATCGTAACAGCACATGCATTTATTATAATTT  122

Query  153  TCTTTATAGTTATACCAATCATAATCGGAGGGTTCGGAAATTGATTAGTTCCTATTATAT  212
            | ||||||||||||||||||||||||||||| || |||||||||||||| ||||| ||||
Sbjct  123  TTTTTATAGTTATACCAATCATAATCGGAGGATTTGGAAATTGATTAGTCCCTATCATAT  182

Query  213  TAGGTGCCCCAGATATAGCATTTCCACGAATAAATAATATAAGATTTTGACTTTTACCCC  272
            | |||||||||||||||||||||||||||||||||||||||||||||||||||||||| |
Sbjct  183  TGGGTGCCCCAGATATAGCATTTCCACGAATAAATAATATAAGATTTTGACTTTTACCTC  242

Query  273  CCTCATTATTTTTATTGATTAACTCTTCATTAATTGAATCCGGAGCTGGAACAGGATGAA  332
            | ||| |||||||||| ||||||||||||||||||||||| |||||||| ||||||||||
Sbjct  243  CTTCACTATTTTTATTAATTAACTCTTCATTAATTGAATCTGGAGCTGGTACAGGATGAA  302

Query  333  CAGTTTATCCCCCTCTATCTTCAAATTTATCACATTATGGTCCATCAGTAGATTTAGCTA  392
            ||||||| || ||||||||||||||||||||||||||||| || ||||||||||||||||
Sbjct  303  CAGTTTACCCTCCTCTATCTTCAAATTTATCACATTATGGCCCTTCAGTAGATTTAGCTA  362

Query  393  TTTTTTCTCTTCACCTTGCTGGTGCTTCTTCAATTTTAGGTGCAATTAACTTCATCACAA  452
            ||||||||||||| |||||||||||||||||||||||||||||||||||||| || ||||
Sbjct  363  TTTTTTCTCTTCATCTTGCTGGTGCTTCTTCAATTTTAGGTGCAATTAACTTTATTACAA  422

Query  453  CTATTGTAAATATACGATCAATTGGAATAACAATAGAACGAATACCATTATTTGTTTGAT  512
            |||||||||| ||||||||||||||||||||||||||||| |||||||||||||||||||
Sbjct  423  CTATTGTAAACATACGATCAATTGGAATAACAATAGAACGTATACCATTATTTGTTTGAT  482

Query  513  CTGTTTTAATTACAGCTATTTTACTACTTCTATCTTTACCTGTATTAGCAGGTGCCATTA  572
            ||||||||||||||||||||||| ||||| ||||||||||||||||||||||||||||||
Sbjct  483  CTGTTTTAATTACAGCTATTTTATTACTTTTATCTTTACCTGTATTAGCAGGTGCCATTA  542

Query  573  CAATATTATTAACTGATCGAAACTTTAACACATCATTTTTGATCCTTCAGGAGGAGGAG  632
            ||||||| |||||||||||||||||||| ||||||||||||||||||||||| ||||||||
Sbjct  543  CAATATTGTTAACTGATCGAAACTTTAATACATCATTTTTGATCCTTCAGGGGGAGGAG  602

Query  633  ATCCCATTTTATATCAACACTTATTT  658
            |||| |||||||||||||| ||||||
Sbjct  603  ATCCAATTTTATATCAACATTTATTT  628
```

>Rhipicephalus sanguineus sensu lato 'temperate lineage' isolate CR1575 cytochrome c oxidase subunit I (COI) gene, partial cds; mitochondrial
Sequence ID: MF426002.1 Length: 639
Range 1: 1 to 628

Score:1000 bits(541), Expect:0.0,
Identities:599/628(95%), Gaps:0/628(0%), Strand: Plus/Plus

```
Query  31   AGGTATATTAGGACTTAGTATAAGAATATTAATTCGTATAGAATTAGGACAACCTGGAAC  90
```

```
             ||||||||||||||||||||||||||||||||||||||||||||||||||||||||||||
Sbjct  1     AGGTATATTAGGACTTAGTATAAGAATATTAATTCGTATAGAATTAGGACAACCTGGAAC  60

Query  91    TTTAATTGGAAATGATCAGATTTATAATGTAATCGTAACAGCACATGCATTTATTATAAT  150
             ||||||||||||||||||||||||||||||||||||||||||||||||||||||||||||
Sbjct  61    TTTAATTGGAAATGATCAGATTTATAATGTAATCGTAACAGCACATGCATTTATTATAAT  120

Query  151   TTTCTTTATAGTTATACCAATCATAATCGGAGGGTTCGGAAATTGATTAGTTCCTATTAT  210
             ||| ||||||||||||||||||||||||||||| || |||||||||||||| ||||| ||
Sbjct  121   TTTTTTTATAGTTATACCAATCATAATCGGAGGATTTGGAAATTGATTAGTCCCTATCAT  180

Query  211   ATTAGGTGCCCCAGATATAGCATTTCCACGAATAAATAATATAAGATTTTGACTTTTACC  270
             ||| ||||||||||||||||||||||||||||||||||||||||||||||||||||||||
Sbjct  181   ATTGGGTGCCCCAGATATAGCATTTCCACGAATAAATAATATAAGATTTTGACTTTTACC  240

Query  271   CCCCTCATTATTTTTATTGATTAACTCTTCATTAATTGAATCCGGAGCTGGAACAGGATG  330
              || ||| |||||||||| ||||||||||||||||||||||| |||||||| ||||||||
Sbjct  241   TCCTTCACTATTTTTATTAATTAACTCTTCATTAATTGAATCTGGAGCTGGTACAGGATG  300

Query  331   AACAGTTTATCCCCCTCTATCTTCAAATTTATCACATTATGGTCCATCAGTAGATTTAGC  390
             ||||||||| || ||||||||||||||||||||||||||||| || ||||||||||||||
Sbjct  301   AACAGTTTACCCTCCTCTATCTTCAAATTTATCACATTATGGCCCTTCAGTAGATTTAGC  360

Query  391   TATTTTTTCTCTTCACCTTGCTGGTGCTTCTTCAATTTTAGGTGCAATTAACTTCATCAC  450
             ||| ||||||||||| |||||||||||||||||||||||||||||||||||||| || ||
Sbjct  361   TATCTTTTCTCTTCATCTTGCTGGTGCTTCTTCAATTTTAGGTGCAATTAACTTTATTAC  420

Query  451   AACTATTGTAAATATACGATCAATTGGAATAACAATAGAACGAATACCATTATTTGTTTG  510
             |||||||||||| ||||||||||||||||||||||||||||| |||||||||||||||||
Sbjct  421   AACTATTGTAAACATACGATCAATTGGAATAACAATAGAACGTATACCATTATTTGTTTG  480

Query  511   ATCTGTTTTAATTACAGCTATTTTACTACTTCTATCTTTACCTGTATTAGCAGGTGCCAT  570
             ||||||||||||||||||||||||| ||||| ||||||||||||||||||||||||||||
Sbjct  481   ATCTGTTTTAATTACAGCTATTTTATTACTTTTATCTTTACCTGTATTAGCAGGTGCCAT  540

Query  571   TACAATATTATTAACTGATCGAAACTTTAACACATCATTTTTTGATCCTTCAGGAGGAGG  630
             ||||||||| |||||||||||||||||||| ||||||||||||||||||||||| |||||
Sbjct  541   TACAATATTGTTAACTGATCGAAACTTTAATACATCATTTTTTGATCCTTCAGGGGGAGG  600

Query  631   AGATCCCATTTTATATCAACACTTATTT  658
             |||||| |||||||||||||| ||||||
Sbjct  601   AGATCCAATTTTATATCAACATTTATTT  628
```

>Rhipicephalus sanguineus isolate Rsan-ML4 cytochrome oxidase subunit I (cox1) gene, partial cds; mitochondrial
Sequence ID: MG855658.1 Length: 630
Range 1: 1 to 620

Score:990 bits(536), Expect:0.0,
Identities:592/620(95%), Gaps:0/620(0%), Strand: Plus/Plus

```
Query  39   TAGGACTTAGTATAAGAATATTAATTCGTATAGAATTAGGACAACCTGGAACTTTAATTG  98
            ||||||||||||||||||||||||||||||||||||||||||||||||||||||||||||
Sbjct  1    TAGGACTTAGTATAAGAATATTAATTCGTATAGAATTAGGACAACCTGGAACTTTAATTG  60

Query  99   GAAATGATCAGATTTATAATGTAATCGTAACAGCACATGCATTTATTATAATTTTCTTTA  158
            ||||||||||||||||||||||||||||||||||||||||||||||||||||||| ||||
Sbjct  61   GAAATGATCAGATTTATAATGTAATCGTAACAGCACATGCATTTATTATAATTTTTTTTA  120

Query  159  TAGTTATACCAATCATAATCGGAGGGTTCGGAAATTGATTAGTTCCTATTATATTAGGTG  218
            ||||||||||||||||||||||||| || |||||||||||||| ||||| ||||| ||||
Sbjct  121  TAGTTATACCAATCATAATCGGAGGATTTGGAAATTGATTAGTCCCTATCATATTGGGTG  180

Query  219  CCCCAGATATAGCATTTCCACGAATAAATAATATAAGATTTTGACTTTTACCCCCCTCAT  278
            |||||||||||||||||||||||||||||||||||||||||||||||||||| || ||| 
Sbjct  181  CCCCAGATATAGCATTTCCACGAATAAATAATATAAGATTTTGACTTTTACCTCCTTCAC  240

Query  279  TATTTTTATTGATTAACTCTTCATTAATTGAATCCGGAGCTGGAACAGGATGAACAGTTT  338
            |||||||||| ||||||||||||||||||||||| |||||||| ||||||||||||||||
Sbjct  241  TATTTTTATTAATTAACTCTTCATTAATTGAATCTGGAGCTGGTACAGGATGAACAGTTT  300

Query  339  ATCCCCCTCTATCTTCAAATTTATCACATTATGGTCCATCAGTAGATTTAGCTATTTTTT  398
            | || ||||||||||||||||||||||||||||| || ||||||||||||||||||||||
Sbjct  301  ACCCTCCTCTATCTTCAAATTTATCACATTATGGCCCTTCAGTAGATTTAGCTATTTTTT  360

Query  399  CTCTTCACCTTGCTGGTGCTTCTTCAATTTTAGGTGCAATTAACTTCATCACAACTATTG  458
            ||||||| |||||||||||||||||||||||||||||||||||||| || ||||||||||
Sbjct  361  CTCTTCATCTTGCTGGTGCTTCTTCAATTTTAGGTGCAATTAACTTTATTACAACTATTG  420

Query  459  TAAATATACGATCAATTGGAATAACAATAGAACGAATACCATTATTTGTTTGATCTGTTT  518
            |||| ||||||||||||||||||||||||||||| |||||||||||||||||||||||||
Sbjct  421  TAAACATACGATCAATTGGAATAACAATAGAACGTATACCATTATTTGTTTGATCTGTTT  480

Query  519  TAATTACAGCTATTTTACTACTTCTATCTTTACCTGTATTAGCAGGTGCCATTACAATAT  578
            ||||||||||||||||| ||||| ||||||||||||||||||||||||||||||||||||
Sbjct  481  TAATTACAGCTATTTTATTACTTTTATCTTTACCTGTATTAGCAGGTGCCATTACAATAT  540

Query  579  TATTAACTGATCGAAACTTTAACACATCATTTTTTGATCCTTCAGGAGGAGGAGATCCCA  638
            | |||||||||||||||||||| ||||||||||||||||||||||| ||||||||||| |
Sbjct  541  TGTTAACTGATCGAAACTTTAATACATCATTTTTTGATCCTTCAGGGGGAGGAGATCCAA  600

Query  639  TTTTATATCAACACTTATTT  658
            ||||||||||||| ||||||
Sbjct  601  TTTTATATCAACATTTATTT  620
```

>Rhipicephalus sanguineus isolate Rsan-ML3 cytochrome oxidase subunit I (cox1) gene, partial cds; mitochondrial
Sequence ID: MG855657.1 Length: 630
Range 1: 1 to 620

Score:990 bits(536), Expect:0.0,
Identities:592/620(95%), Gaps:0/620(0%), Strand: Plus/Plus

```
Query  39   TAGGACTTAGTATAAGAATATTAATTCGTATAGAATTAGGACAACCTGGAACTTTAATTG  98
            ||||||||||||||||||||||||||||||||||||||||||||||||||||||||||||
Sbjct  1    TAGGACTTAGTATAAGAATATTAATTCGTATAGAATTAGGACAACCTGGAACTTTAATTG  60

Query  99   GAAATGATCAGATTTATAATGTAATCGTAACAGCACATGCATTTATTATAATTTTCTTTA  158
            ||||||||||||||||||||||||||||||||||||||||||||||||||||||| ||||
Sbjct  61   GAAATGATCAGATTTATAATGTAATCGTAACAGCACATGCATTTATTATAATTTTTTTTA  120

Query  159  TAGTTATACCAATCATAATCGGAGGGTTCGGAAATTGATTAGTTCCTATTATATTAGGTG  218
            ||||||||||||||||||||||||| || |||||||||||||| ||||| ||||| ||||
Sbjct  121  TAGTTATACCAATCATAATCGGAGGATTTGGAAATTGATTAGTCCCTATCATATTGGGTG  180

Query  219  CCCCAGATATAGCATTTCCACGAATAAATAATATAAGATTTTGACTTTTACCCCCCTCAT  278
            |||||||||||||||||||||||||||||||||||||||||||||||||||| || |||
Sbjct  181  CCCCAGATATAGCATTTCCACGAATAAATAATATAAGATTTTGACTTTTACCTCCTTCAC  240

Query  279  TATTTTTATTGATTAACTCTTCATTAATTGAATCCGGAGCTGGAACAGGATGAACAGTTT  338
            |||||||||| ||||||||||||||||||||||| |||||||| ||||||||||||||||
Sbjct  241  TATTTTTATTAATTAACTCTTCATTAATTGAATCTGGAGCTGGTACAGGATGAACAGTTT  300

Query  339  ATCCCCCTCTATCTTCAAATTTATCACATTATGGTCCATCAGTAGATTTAGCTATTTTTT  398
            | || ||||||||||||||||||||||||||||| || ||||||||||||||||||||||
Sbjct  301  ACCCTCCTCTATCTTCAAATTTATCACATTATGGCCCTTCAGTAGATTTAGCTATTTTTT  360

Query  399  CTCTTCACCTTGCTGGTGCTTCTTCAATTTTAGGTGCAATTAACTTCATCACAACTATTG  458
            ||||||| |||||||||||||||||||||||||||||||||||||| || ||||||||||
Sbjct  361  CTCTTCATCTTGCTGGTGCTTCTTCAATTTTAGGTGCAATTAACTTTATTACAACTATTG  420

Query  459  TAAATATACGATCAATTGGAATAACAATAGAACGAATACCATTATTTGTTTGATCTGTTT  518
            |||| ||||||||||||||||||||||||||||| |||||||||||||||||||||||||
Sbjct  421  TAAACATACGATCAATTGGAATAACAATAGAACGTATACCATTATTTGTTTGATCTGTTT  480

Query  519  TAATTACAGCTATTTTACTACTTCTATCTTTACCTGTATTAGCAGGTGCCATTACAATAT  578
            ||||||||||||||||| ||||| ||||||||||||||||||||||||||||||||||||
Sbjct  481  TAATTACAGCTATTTTATTACTTTTATCTTTACCTGTATTAGCAGGTGCCATTACAATAT  540

Query  579  TATTAACTGATCGAAACTTTAACACATCATTTTTTGATCCTTCAGGAGGAGGAGATCCCA  638
            | |||||||||||||||||||| ||||||||||||||||||||||| ||||||||||| |
Sbjct  541  TGTTAACTGATCGAAACTTTAATACATCATTTTTTGATCCTTCAGGGGGAGGAGATCCAA  600

Query  639  TTTTATATCAACACTTATTT  658
            ||||||||||||| ||||||
Sbjct  601  TTTTATATCAACATTTATTT  620
```

>Rhipicephalus sanguineus isolate Rsan-ML2 cytochrome oxidase subunit I (cox1) gene, partial cds; mitochondrial
Sequence ID: MG855656.1 Length: 630
Range 1: 1 to 620

Score:990 bits(536), Expect:0.0,

```
Identities:592/620(95%),  Gaps:0/620(0%), Strand: Plus/Plus

Query  39   TAGGACTTAGTATAAGAATATTAATTCGTATAGAATTAGGACAACCTGGAACTTTAATTG  98
            ||||||||||||||||||||||||||||||||||||||||||||||||||||||||||||
Sbjct  1    TAGGACTTAGTATAAGAATATTAATTCGTATAGAATTAGGACAACCTGGAACTTTAATTG  60

Query  99   GAAATGATCAGATTTATAATGTAATCGTAACAGCACATGCATTTATTATAATTTTCTTTA  158
            ||||||||||||||||||||||||||||||||||||||||||||||||||||||| ||||
Sbjct  61   GAAATGATCAGATTTATAATGTAATCGTAACAGCACATGCATTTATTATAATTTTTTTTA  120

Query  159  TAGTTATACCAATCATAATCGGAGGGTTCGGAAATTGATTAGTTCCTATTATATTAGGTG  218
            ||||||||||||||||||||||||| || |||||||||||||| ||||| ||||| ||||
Sbjct  121  TAGTTATACCAATCATAATCGGAGGATTTGGAAATTGATTAGTCCCTATCATATTGGGTG  180

Query  219  CCCCAGATATAGCATTTCCACGAATAAATAATATAAGATTTTGACTTTTACCCCCCTCAT  278
            |||||||||||||||||||||||||||||||||||||||||||||||||||| || ||| 
Sbjct  181  CCCCAGATATAGCATTTCCACGAATAAATAATATAAGATTTTGACTTTTACCTCCTTCAC  240

Query  279  TATTTTTATTGATTAACTCTTCATTAATTGAATCCGGAGCTGGAACAGGATGAACAGTTT  338
            |||||||||| ||||||||||||||||||||||| |||||||| ||||||||||||||||
Sbjct  241  TATTTTTATTAATTAACTCTTCATTAATTGAATCTGGAGCTGGTACAGGATGAACAGTTT  300

Query  339  ATCCCCCTCTATCTTCAAATTTATCACATTATGGTCCATCAGTAGATTTAGCTATTTTTT  398
            | || ||||||||||||||||||||||||||||| || ||||||||||||||||||||||
Sbjct  301  ACCCTCCTCTATCTTCAAATTTATCACATTATGGCCCTTCAGTAGATTTAGCTATTTTTT  360

Query  399  CTCTTCACCTTGCTGGTGCTTCTTCAATTTTAGGTGCAATTAACTTCATCACAACTATTG  458
            ||||||| |||||||||||||||||||||||||||||||||||||| || ||||||||||
Sbjct  361  CTCTTCATCTTGCTGGTGCTTCTTCAATTTTAGGTGCAATTAACTTTATTACAACTATTG  420

Query  459  TAAATATACGATCAATTGGAATAACAATAGAACGAATACCATTATTTGTTTGATCTGTTT  518
            |||| ||||||||||||||||||||||||||||| |||||||||||||||||||||||||
Sbjct  421  TAAACATACGATCAATTGGAATAACAATAGAACGTATACCATTATTTGTTTGATCTGTTT  480

Query  519  TAATTACAGCTATTTTACTACTTCTATCTTTACCTGTATTAGCAGGTGCCATTACAATAT  578
            ||||||||||||||||| ||||| ||||||||||||||||||||||||||||||||||||
Sbjct  481  TAATTACAGCTATTTTATTACTTTTATCTTTACCTGTATTAGCAGGTGCCATTACAATAT  540

Query  579  TATTAACTGATCGAAACTTTAACACATCATTTTTTGATCCTTCAGGAGGAGGAGATCCCA  638
            | |||||||||||||||||||| ||||||||||||||||||||||| ||||||||||| |
Sbjct  541  TGTTAACTGATCGAAACTTTAATACATCATTTTTTGATCCTTCAGGGGGAGGAGATCCAA  600

Query  639  TTTTATATCAACACTTATTT  658
            ||||||||||||| ||||||
Sbjct  601  TTTTATATCAACATTTATTT  620
```

>Rhipicephalus sanguineus isolate Kehf-Lagareb-A cytochrome oxidase subunit I (COI) gene, partial cds; mitochondrial
Sequence ID: KX757910.1 Length: 630
Range 1: 1 to 620

```
Score:990 bits(536), Expect:0.0,
Identities:592/620(95%),  Gaps:0/620(0%), Strand: Plus/Plus

Query  39   TAGGACTTAGTATAAGAATATTAATTCGTATAGAATTAGGACAACCTGGAACTTTAATTG  98
            ||||||||||||||||||||||||||||||||||||||||||||||||||||||||||||
Sbjct  1    TAGGACTTAGTATAAGAATATTAATTCGTATAGAATTAGGACAACCTGGAACTTTAATTG  60

Query  99   GAAATGATCAGATTTATAATGTAATCGTAACAGCACATGCATTTATTATAATTTTCTTTA  158
            ||||||||||||||||||||||||||||||||||||||||||||||||||||||| ||||
Sbjct  61   GAAATGATCAGATTTATAATGTAATCGTAACAGCACATGCATTTATTATAATTTTTTTTA  120

Query  159  TAGTTATACCAATCATAATCGGAGGGTTCGGAAATTGATTAGTTCCTATTATATTAGGTG  218
            ||||||||||||||||||||||||| || |||||||||||||| ||||| ||||| ||||
Sbjct  121  TAGTTATACCAATCATAATCGGAGGATTTGGAAATTGATTAGTCCCTATCATATTGGGTG  180

Query  219  CCCCAGATATAGCATTTCCACGAATAAATAATATAAGATTTTGACTTTTACCCCCCTCAT  278
            |||||||||||||||||||||||||||||||||||||||||||||||||||| || ||| 
Sbjct  181  CCCCAGATATAGCATTTCCACGAATAAATAATATAAGATTTTGACTTTTACCTCCTTCAC  240

Query  279  TATTTTTATTGATTAACTCTTCATTAATTGAATCCGGAGCTGGAACAGGATGAACAGTTT  338
            |||||||||| ||||||||||||||||||||||| |||||||| ||||||||||||||||
Sbjct  241  TATTTTTATTAATTAACTCTTCATTAATTGAATCTGGAGCTGGTACAGGATGAACAGTTT  300

Query  339  ATCCCCCTCTATCTTCAAATTTATCACATTATGGTCCATCAGTAGATTTAGCTATTTTTT  398
            | || ||||||||||||||||||||||||||||| || ||||||||||||||||||||||
Sbjct  301  ACCCTCCTCTATCTTCAAATTTATCACATTATGGCCCTTCAGTAGATTTAGCTATTTTTT  360

Query  399  CTCTTCACCTTGCTGGTGCTTCTTCAATTTTAGGTGCAATTAACTTCATCACAACTATTG  458
            ||||||| |||||||||||||||||||||||||||||||||||||| || ||||||||||
Sbjct  361  CTCTTCATCTTGCTGGTGCTTCTTCAATTTTAGGTGCAATTAACTTTATTACAACTATTG  420

Query  459  TAAATATACGATCAATTGGAATAACAATAGAACGAATACCATTATTTGTTTGATCTGTTT  518
            |||| ||||||||||||||||||||||||||||| |||||||||||||||||||||||||
Sbjct  421  TAAACATACGATCAATTGGAATAACAATAGAACGTATACCATTATTTGTTTGATCTGTTT  480

Query  519  TAATTACAGCTATTTTACTACTTCTATCTTTACCTGTATTAGCAGGTGCCATTACAATAT  578
            ||||||||||||||||| ||||| ||||||||||||||||||||||||||||||||||||
Sbjct  481  TAATTACAGCTATTTTATTACTTTTATCTTTACCTGTATTAGCAGGTGCCATTACAATAT  540

Query  579  TATTAACTGATCGAAACTTTAACACATCATTTTTTGATCCTTCAGGAGGAGGAGATCCCA  638
            | |||||||||||||||||||| ||||||||||||||||||||||| ||||||||||| |
Sbjct  541  TGTTAACTGATCGAAACTTTAATACATCATTTTTTGATCCTTCAGGGGGAGGAGATCCAA  600

Query  639  TTTTATATCAACACTTATTT  658
            ||||||||||||| ||||||
Sbjct  601  TTTTATATCAACATTTATTT  620
```

>Rhipicephalus sanguineus isolate Italy-B cytochrome oxidase subunit I (COI) gene, partial cds; mitochondrial

Sequence ID: KX757904.1 Length: 630

Range 1: 1 to 620

```
Score:990 bits(536), Expect:0.0,
Identities:592/620(95%),  Gaps:0/620(0%), Strand: Plus/Plus

Query  39   TAGGACTTAGTATAAGAATATTAATTCGTATAGAATTAGGACAACCTGGAACTTTAATTG  98
            ||||||||||||||||||||||||||||||||||||||||||||||||||||||||||||
Sbjct  1    TAGGACTTAGTATAAGAATATTAATTCGTATAGAATTAGGACAACCTGGAACTTTAATTG  60

Query  99   GAAATGATCAGATTTATAATGTAATCGTAACAGCACATGCATTTATTATAATTTTCTTTA  158
            ||||||||||||||||||||||||||||||||||||||||||||||||||||||| ||||
Sbjct  61   GAAATGATCAGATTTATAATGTAATCGTAACAGCACATGCATTTATTATAATTTTTTTTA  120

Query  159  TAGTTATACCAATCATAATCGGAGGGTTCGGAAATTGATTAGTTCCTATTATATTAGGTG  218
            ||||||||||||||||||||||||| || |||||||||||||| ||||| ||||| ||||
Sbjct  121  TAGTTATACCAATCATAATCGGAGGATTTGGAAATTGATTAGTCCCTATCATATTGGGTG  180

Query  219  CCCCAGATATAGCATTTCCACGAATAAATAATATAAGATTTTGACTTTTACCCCCCTCAT  278
            |||||||||||||||||||||||||||||||||||||||||||||||||||| || |||
Sbjct  181  CCCCAGATATAGCATTTCCACGAATAAATAATATAAGATTTTGACTTTTACCTCCTTCAC  240

Query  279  TATTTTTATTGATTAACTCTTCATTAATTGAATCCGGAGCTGGAACAGGATGAACAGTTT  338
            |||||||||| ||||||||||||||||||||||| |||||||| ||||||||||||||||
Sbjct  241  TATTTTTATTAATTAACTCTTCATTAATTGAATCTGGAGCTGGTACAGGATGAACAGTTT  300

Query  339  ATCCCCCTCTATCTTCAAATTTATCACATTATGGTCCATCAGTAGATTTAGCTATTTTTT  398
            | || ||||||||||||||||||||||||||||| || ||||||||||||||||||||||
Sbjct  301  ACCCTCCTCTATCTTCAAATTTATCACATTATGGCCCTTCAGTAGATTTAGCTATTTTTT  360

Query  399  CTCTTCACCTTGCTGGTGCTTCTTCAATTTTAGGTGCAATTAACTTCATCACAACTATTG  458
            ||||||| |||||||||||||||||||||||||||||||||||||| || ||||||||||
Sbjct  361  CTCTTCATCTTGCTGGTGCTTCTTCAATTTTAGGTGCAATTAACTTTATTACAACTATTG  420

Query  459  TAAATATACGATCAATTGGAATAACAATAGAACGAATACCATTATTTGTTTGATCTGTTT  518
            |||| ||||||||||||||||||||||||||||| |||||||||||||||||||||||||
Sbjct  421  TAAACATACGATCAATTGGAATAACAATAGAACGTATACCATTATTTGTTTGATCTGTTT  480

Query  519  TAATTACAGCTATTTTACTACTTCTATCTTTACCTGTATTAGCAGGTGCCATTACAATAT  578
            ||||||||||||||||| ||||| ||||||||||||||||||||||||||||||||||||
Sbjct  481  TAATTACAGCTATTTTATTACTTTTATCTTTACCTGTATTAGCAGGTGCCATTACAATAT  540

Query  579  TATTAACTGATCGAAACTTTAACACATCATTTTTTGATCCTTCAGGAGGAGGAGATCCCA  638
            | |||||||||||||||||||| ||||||||||||||||||||||| ||||||||||| |
Sbjct  541  TGTTAACTGATCGAAACTTTAATACATCATTTTTTGATCCTTCAGGGGGAGGAGATCCAA  600

Query  639  TTTTATATCAACACTTATTT  658
            ||||||||||||| ||||||
Sbjct  601  TTTTATATCAACATTTATTT  620
```

>Rhipicephalus sanguineus isolate Malta-A cytochrome oxidase subunit I (COI) gene, partial cds; mitochondrial
Sequence ID: KX757902.1 Length: 630

```
Range 1: 1 to 620

Score:990 bits(536), Expect:0.0,
Identities:592/620(95%),  Gaps:0/620(0%), Strand: Plus/Plus

Query  39   TAGGACTTAGTATAAGAATATTAATTCGTATAGAATTAGGACAACCTGGAACTTTAATTG  98
            ||||||||||||||||||||||||||||||||||||||||||||||||||||||||||||
Sbjct  1    TAGGACTTAGTATAAGAATATTAATTCGTATAGAATTAGGACAACCTGGAACTTTAATTG  60

Query  99   GAAATGATCAGATTTATAATGTAATCGTAACAGCACATGCATTTATTATAATTTTCTTTA  158
            ||||||||||||||||||||||||||||||||||||||||||||||||||||||| ||||
Sbjct  61   GAAATGATCAGATTTATAATGTAATCGTAACAGCACATGCATTTATTATAATTTTTTTTA  120

Query  159  TAGTTATACCAATCATAATCGGAGGGTTCGGAAATTGATTAGTTCCTATTATATTAGGTG  218
            ||||||||||||||||||||||||| || |||||||||||||| ||||| ||||| ||||
Sbjct  121  TAGTTATACCAATCATAATCGGAGGATTTGGAAATTGATTAGTCCCTATCATATTGGGTG  180

Query  219  CCCCAGATATAGCATTTCCACGAATAAATAATATAAGATTTGACTTTTACCCCCCTCAT  278
            |||||||||||||||||||||||||||||||||||||||||||||||||||| || |||
Sbjct  181  CCCCAGATATAGCATTTCCACGAATAAATAATATAAGATTTGACTTTTACCTCCTTCAC  240

Query  279  TATTTTTATTGATTAACTCTTCATTAATTGAATCCGGAGCTGGAACAGGATGAACAGTTT  338
            |||||||||| ||||||||||||||||||||||| |||||||| ||||||||||||||||
Sbjct  241  TATTTTTATTAATTAACTCTTCATTAATTGAATCTGGAGCTGGTACAGGATGAACAGTTT  300

Query  339  ATCCCCCTCTATCTTCAAATTTATCACATTATGGTCCATCAGTAGATTTAGCTATTTTTT  398
            | || ||||||||||||||||||||||||||||| || ||||||||||||||||||||||
Sbjct  301  ACCCTCCTCTATCTTCAAATTTATCACATTATGGCCCTTCAGTAGATTTAGCTATTTTTT  360

Query  399  CTCTTCACCTTGCTGGTGCTTCTTCAATTTAGGTGCAATTAACTTCATCACAACTATTG  458
            ||||||| ||||||||||||||||||||||||||||||||||||| || |||||||||||
Sbjct  361  CTCTTCATCTTGCTGGTGCTTCTTCAATTTAGGTGCAATTAACTTTATTACAACTATTG  420

Query  459  TAAATATACGATCAATTGGAATAACAATAGAACGAATACCATTATTTGTTTGATCTGTTT  518
            |||| ||||||||||||||||||||||||||||||| |||||||||||||||||||||||
Sbjct  421  TAAACATACGATCAATTGGAATAACAATAGAACGTATACCATTATTTGTTTGATCTGTTT  480

Query  519  TAATTACAGCTATTTTACTACTTCTATCTTTACCTGTATTAGCAGGTGCCATTACAATAT  578
            ||||||||||||||||| ||||| ||||||||||||||||||||||||||||||||||||
Sbjct  481  TAATTACAGCTATTTTATTACTTTTATCTTTACCTGTATTAGCAGGTGCCATTACAATAT  540

Query  579  TATTAACTGATCGAAACTTTAACACATCATTTTTTGATCCTTCAGGAGGAGGAGATCCCA  638
            | ||||||||||||||||||||| |||||||||||||||||||||| ||||||||||| |
Sbjct  541  TGTTAACTGATCGAAACTTTAATACATCATTTTTTGATCCTTCAGGGGGAGGAGATCCAA  600

Query  639  TTTTATATCAACACTTATTT  658
            ||||||||||||| ||||||
Sbjct  601  TTTTATATCAACATTTATTT  620
```

>Rhipicephalus sanguineus isolate Zagreb-C cytochrome oxidase subunit I (COI) gene, partial cds; mitochondrial

Sequence ID: KX757896.1 Length: 630
Range 1: 1 to 620

Score:990 bits(536), Expect:0.0,
Identities:592/620(95%), Gaps:0/620(0%), Strand: Plus/Plus

```
Query  39   TAGGACTTAGTATAAGAATATTAATTCGTATAGAATTAGGACAACCTGGAACTTTAATTG  98
            ||||||||||||||||||||||||||||||||||||||||||||||||||||||||||||
Sbjct  1    TAGGACTTAGTATAAGAATATTAATTCGTATAGAATTAGGACAACCTGGAACTTTAATTG  60

Query  99   GAAATGATCAGATTTATAATGTAATCGTAACAGCACATGCATTTATTATAATTTTCTTTA  158
            ||||||||||||||||||||||||||||||||||||||||||||||||||||||| ||||
Sbjct  61   GAAATGATCAGATTTATAATGTAATCGTAACAGCACATGCATTTATTATAATTTTTTTTA  120

Query  159  TAGTTATACCAATCATAATCGGAGGGTTCGGAAATTGATTAGTTCCTATTATATTAGGTG  218
            ||||||||||||||||||||||||| || |||||||||||||| ||||| ||||| ||||
Sbjct  121  TAGTTATACCAATCATAATCGGAGGATTTGGAAATTGATTAGTCCCTATCATATTGGGTG  180

Query  219  CCCCAGATATAGCATTTCCACGAATAAATAATATAAGATTTTGACTTTTACCCCCCTCAT  278
            |||||||||||||||||||||||||||||||||||||||||||||||||||| || ||| 
Sbjct  181  CCCCAGATATAGCATTTCCACGAATAAATAATATAAGATTTTGACTTTTACCTCCTTCAC  240

Query  279  TATTTTTATTGATTAACTCTTCATTAATTGAATCCGGAGCTGGAACAGGATGAACAGTTT  338
            |||||||||| ||||||||||||||||||||||| |||||||| ||||||||||||||||
Sbjct  241  TATTTTTATTAATTAACTCTTCATTAATTGAATCTGGAGCTGGTACAGGATGAACAGTTT  300

Query  339  ATCCCCCTCTATCTTCAAATTTATCACATTATGGTCCATCAGTAGATTTAGCTATTTTTT  398
            | || ||||||||||||||||||||||||||||| || ||||||||||||||||||||||
Sbjct  301  ACCCTCCTCTATCTTCAAATTTATCACATTATGGCCCTTCAGTAGATTTAGCTATTTTTT  360

Query  399  CTCTTCACCTTGCTGGTGCTTCTTCAATTTAGGTGCAATTAACTTCATCACAACTATTG  458
            ||||||| ||||||||||||||||||||||||||||||||||||| || ||||||||||
Sbjct  361  CTCTTCATCTTGCTGGTGCTTCTTCAATTTAGGTGCAATTAACTTTATTACAACTATTG  420

Query  459  TAAATATACGATCAATTGGAATAACAATAGAACGAATACCATTATTTGTTTGATCTGTTT  518
            |||| ||||||||||||||||||||||||||||| |||||||||||||||||||||||||
Sbjct  421  TAAACATACGATCAATTGGAATAACAATAGAACGTATACCATTATTTGTTTGATCTGTTT  480

Query  519  TAATTACAGCTATTTTACTACTTCTATCTTTACCTGTATTAGCAGGTGCCATTACAATAT  578
            ||||||||||||||||| ||||| ||||||||||||||||||||||||||||||||||||
Sbjct  481  TAATTACAGCTATTTTATTACTTTTATCTTTACCTGTATTAGCAGGTGCCATTACAATAT  540

Query  579  TATTAACTGATCGAAACTTTAACACATCATTTTTTGATCCTTCAGGAGGAGGAGATCCCA  638
            | |||||||||||||||||||| |||||||||||||||||||||| |||||||||||| |
Sbjct  541  TGTTAACTGATCGAAACTTTAATACATCATTTTTTGATCCTTCAGGGGGAGGAGATCCAA  600

Query  639  TTTTATATCAACACTTATTT  658
            ||||||||||||| ||||||
Sbjct  601  TTTTATATCAACATTTATTT  620
```

>Rhipicephalus sanguineus isolate Pula-B cytochrome oxidase subunit I (COI)

gene, partial cds; mitochondrial
Sequence ID: KX757889.1 Length: 630
Range 1: 1 to 620

Score:990 bits(536), Expect:0.0,
Identities:592/620(95%), Gaps:0/620(0%), Strand: Plus/Plus

```
Query  39   TAGGACTTAGTATAAGAATATTAATTCGTATAGAATTAGGACAACCTGGAACTTTAATTG  98
            ||||||||||||||||||||||||||||||||||||||||||||||||||||||||||||
Sbjct  1    TAGGACTTAGTATAAGAATATTAATTCGTATAGAATTAGGACAACCTGGAACTTTAATTG  60

Query  99   GAAATGATCAGATTTATAATGTAATCGTAACAGCACATGCATTTATTATAATTTTCTTTA  158
            ||||||||||||||||||||||||||||||||||||||||||||||||||||||| ||||
Sbjct  61   GAAATGATCAGATTTATAATGTAATCGTAACAGCACATGCATTTATTATAATTTTTTTTA  120

Query  159  TAGTTATACCAATCATAATCGGAGGGTTCGGAAATTGATTAGTTCCTATTATATTAGGTG  218
            ||||||||||||||||||||||||| || |||||||||||||| ||||| ||||| ||||
Sbjct  121  TAGTTATACCAATCATAATCGGAGGATTTGGAAATTGATTAGTCCCTATCATATTGGGTG  180

Query  219  CCCCAGATATAGCATTTCCACGAATAAATAATATAAGATTTTGACTTTTACCCCCCTCAT  278
            |||||||||||||||||||||||||||||||||||||||||||||||||||| || ||| 
Sbjct  181  CCCCAGATATAGCATTTCCACGAATAAATAATATAAGATTTTGACTTTTACCTCCTTCAC  240

Query  279  TATTTTTATTGATTAACTCTTCATTAATTGAATCCGGAGCTGGAACAGGATGAACAGTTT  338
            |||||||||| ||||||||||||||||||||||| |||||||| ||||||||||||||||
Sbjct  241  TATTTTTATTAATTAACTCTTCATTAATTGAATCTGGAGCTGGTACAGGATGAACAGTTT  300

Query  339  ATCCCCCTCTATCTTCAAATTTATCACATTATGGTCCATCAGTAGATTTAGCTATTTTTT  398
            | || ||||||||||||||||||||||||||||| || ||||||||||||||||||||||
Sbjct  301  ACCCTCCTCTATCTTCAAATTTATCACATTATGGCCCTTCAGTAGATTTAGCTATTTTTT  360

Query  399  CTCTTCACCTTGCTGGTGCTTCTTCAATTTTAGGTGCAATTAACTTCATCACAACTATTG  458
            ||||||| |||||||||||||||||||||||||||||||||||||| || ||||||||||
Sbjct  361  CTCTTCATCTTGCTGGTGCTTCTTCAATTTTAGGTGCAATTAACTTTATTACAACTATTG  420

Query  459  TAAATATACGATCAATTGGAATAACAATAGAACGAATACCATTATTTGTTTGATCTGTTT  518
            |||| ||||||||||||||||||||||||||||| |||||||||||||||||||||||||
Sbjct  421  TAAACATACGATCAATTGGAATAACAATAGAACGTATACCATTATTTGTTTGATCTGTTT  480

Query  519  TAATTACAGCTATTTTACTACTTCTATCTTTACCTGTATTAGCAGGTGCCATTACAATAT  578
            ||||||||||||||||| ||||| ||||||||||||||||||||||||||||||||||||
Sbjct  481  TAATTACAGCTATTTTATTACTTTTATCTTTACCTGTATTAGCAGGTGCCATTACAATAT  540

Query  579  TATTAACTGATCGAAACTTTAACACATCATTTTTTGATCCTTCAGGAGGAGGAGATCCCA  638
            | |||||||||||||||||||| ||||||||||||||||||||||| ||||||||||| |
Sbjct  541  TGTTAACTGATCGAAACTTTAATACATCATTTTTTGATCCTTCAGGGGGAGGAGATCCAA  600

Query  639  TTTTATATCAACACTTATTT  658
            ||||||||||||| ||||||
Sbjct  601  TTTTATATCAACATTTATTT  620
```

```
>Rhipicephalus sanguineus isolate Rovind cytochrome oxidase subunit I (COI)
gene, partial cds; mitochondrial
Sequence ID: KX757887.1 Length: 630
Range 1: 1 to 620

Score:990 bits(536), Expect:0.0,
Identities:592/620(95%),  Gaps:0/620(0%), Strand: Plus/Plus

Query  39    TAGGACTTAGTATAAGAATATTAATTCGTATAGAATTAGGACAACCTGGAACTTTAATTG  98
             ||||||||||||||||||||||||||||||||||||||||||||||||||||||||||||
Sbjct  1     TAGGACTTAGTATAAGAATATTAATTCGTATAGAATTAGGACAACCTGGAACTTTAATTG  60

Query  99    GAAATGATCAGATTTATAATGTAATCGTAACAGCACATGCATTTATTATAATTTTCTTTA  158
             ||||||||||||||||||||||||||||||||||||||||||||||||||||||| ||||
Sbjct  61    GAAATGATCAGATTTATAATGTAATCGTAACAGCACATGCATTTATTATAATTTTTTTTA  120

Query  159   TAGTTATACCAATCATAATCGGAGGGTTCGGAAATTGATTAGTTCCTATTATATTAGGTG  218
             ||||||||||||||||||||||||| || |||||||||||||| ||||| ||||| ||||
Sbjct  121   TAGTTATACCAATCATAATCGGAGGATTTGGAAATTGATTAGTCCCTATCATATTGGGTG  180

Query  219   CCCCAGATATAGCATTTCCACGAATAAATAATATAAGATTTTGACTTTTACCCCCCTCAT  278
             |||||||||||||||||||||||||||||||||||||||||||||||||||| || |||
Sbjct  181   CCCCAGATATAGCATTTCCACGAATAAATAATATAAGATTTTGACTTTTACCTCCTTCAC  240

Query  279   TATTTTTATTGATTAACTCTTCATTAATTGAATCCGGAGCTGGAACAGGATGAACAGTTT  338
             |||||||||| ||||||||||||||||||||||| |||||||| ||||||||||||||||
Sbjct  241   TATTTTTATTAATTAACTCTTCATTAATTGAATCTGGAGCTGGTACAGGATGAACAGTTT  300

Query  339   ATCCCCCTCTATCTTCAAATTTATCACATTATGGTCCATCAGTAGATTTAGCTATTTTTT  398
             | || ||||||||||||||||||||||||||||| || ||||||||||||||||||||||
Sbjct  301   ACCCTCCTCTATCTTCAAATTTATCACATTATGGCCCTTCAGTAGATTTAGCTATTTTTT  360

Query  399   CTCTTCACCTTGCTGGTGCTTCTTCAATTTTAGGTGCAATTAACTTCATCACAACTATTG  458
             ||||||| |||||||||||||||||||||||||||||||||||||| || ||||||||||
Sbjct  361   CTCTTCATCTTGCTGGTGCTTCTTCAATTTTAGGTGCAATTAACTTTATTACAACTATTG  420

Query  459   TAAATATACGATCAATTGGAATAACAATAGAACGAATACCATTATTTGTTTGATCTGTTT  518
             |||| ||||||||||||||||||||||||||||| |||||||||||||||||||||||||
Sbjct  421   TAAACATACGATCAATTGGAATAACAATAGAACGTATACCATTATTTGTTTGATCTGTTT  480

Query  519   TAATTACAGCTATTTTACTACTTCTATCTTTACCTGTATTAGCAGGTGCCATTACAATAT  578
             ||||||||||||||||| ||||| ||||||||||||||||||||||||||||||||||||
Sbjct  481   TAATTACAGCTATTTTATTACTTTTATCTTTACCTGTATTAGCAGGTGCCATTACAATAT  540

Query  579   TATTAACTGATCGAAACTTTAACACATCATTTTTTGATCCTTCAGGAGGAGGAGATCCCA  638
             | |||||||||||||||||||| |||||||||||||||||||||| |||||||||||| |
Sbjct  541   TGTTAACTGATCGAAACTTTAATACATCATTTTTTGATCCTTCAGGGGGAGGAGATCCAA  600

Query  639   TTTTATATCAACACTTATTT  658
             ||||||||||||| ||||||
Sbjct  601   TTTTATATCAACATTTATTT  620
```

```
>Rhipicephalus sanguineus isolate Kajtasovo cytochrome oxidase subunit I (COI)
gene, partial cds; mitochondrial
Sequence ID: KX757879.1 Length: 630
Range 1: 1 to 620

Score:990 bits(536), Expect:0.0,
Identities:592/620(95%),  Gaps:0/620(0%), Strand: Plus/Plus

Query  39    TAGGACTTAGTATAAGAATATTAATTCGTATAGAATTAGGACAACCTGGAACTTTAATTG  98
             ||||||||||||||||||||||||||||||||||||||||||||||||||||||||||||
Sbjct  1     TAGGACTTAGTATAAGAATATTAATTCGTATAGAATTAGGACAACCTGGAACTTTAATTG  60

Query  99    GAAATGATCAGATTTATAATGTAATCGTAACAGCACATGCATTTATTATAATTTTCTTTA  158
             ||||||||||||||||||||||||||||||||||||||||||||||||||||||| ||||
Sbjct  61    GAAATGATCAGATTTATAATGTAATCGTAACAGCACATGCATTTATTATAATTTTTTTTA  120

Query  159   TAGTTATACCAATCATAATCGGAGGGTTCGGAAATTGATTAGTTCCTATTATATTAGGTG  218
             ||||||||||||||||||||||||| || |||||||||||||| ||||| ||||| ||||
Sbjct  121   TAGTTATACCAATCATAATCGGAGGATTTGGAAATTGATTAGTCCCTATCATATTGGGTG  180

Query  219   CCCCAGATATAGCATTTCCACGAATAAATAATATAAGATTTTGACTTTTACCCCCCTCAT  278
             |||||||||||||||||||||||||||||||||||||||||||||||||||| || ||| 
Sbjct  181   CCCCAGATATAGCATTTCCACGAATAAATAATATAAGATTTTGACTTTTACCTCCTTCAC  240

Query  279   TATTTTTATTGATTAACTCTTCATTAATTGAATCCGGAGCTGGAACAGGATGAACAGTTT  338
             |||||||||| ||||||||||||||||||||||| |||||||| ||||||||||||||||
Sbjct  241   TATTTTTATTAATTAACTCTTCATTAATTGAATCTGGAGCTGGTACAGGATGAACAGTTT  300

Query  339   ATCCCCCTCTATCTTCAAATTTATCACATTATGGTCCATCAGTAGATTTAGCTATTTTTT  398
             | || ||||||||||||||||||||||||||||| || ||||||||||||||||||||||
Sbjct  301   ACCCTCCTCTATCTTCAAATTTATCACATTATGGCCCTTCAGTAGATTTAGCTATTTTTT  360

Query  399   CTCTTCACCTTGCTGGTGCTTCTTCAATTTTAGGTGCAATTAACTTCATCACAACTATTG  458
             ||||||| |||||||||||||||||||||||||||||||||||||| || ||||||||||
Sbjct  361   CTCTTCATCTTGCTGGTGCTTCTTCAATTTTAGGTGCAATTAACTTTATTACAACTATTG  420

Query  459   TAAATATACGATCAATTGGAATAACAATAGAACGAATACCATTATTTGTTTGATCTGTTT  518
             |||| ||||||||||||||||||||||||||||| |||||||||||||||||||||||||
Sbjct  421   TAAACATACGATCAATTGGAATAACAATAGAACGTATACCATTATTTGTTTGATCTGTTT  480

Query  519   TAATTACAGCTATTTTACTACTTCTATCTTTACCTGTATTAGCAGGTGCCATTACAATAT  578
             ||||||||||||||||| ||||| ||||||||||||||||||||||||||||||||||||
Sbjct  481   TAATTACAGCTATTTTATTACTTTTATCTTTACCTGTATTAGCAGGTGCCATTACAATAT  540

Query  579   TATTAACTGATCGAAACTTTAACACATCATTTTTTGATCCTTCAGGAGGAGGAGATCCCA  638
             | |||||||||||||||||||| ||||||||||||||||||||||| ||||||||||| |
Sbjct  541   TGTTAACTGATCGAAACTTTAATACATCATTTTTTGATCCTTCAGGGGGAGGAGATCCAA  600

Query  639   TTTTATATCAACACTTATTT  658
             ||||||||||||| ||||||
Sbjct  601   TTTTATATCAACATTTATTT  620
```

```
>Rhipicephalus sanguineus clone #6 cytochrome oxidase subunit I (COI) gene,
partial cds; mitochondrial
Sequence ID: KX519711.1 Length: 636
Range 1: 1 to 620

Score:990 bits(536), Expect:0.0,
Identities:592/620(95%),  Gaps:0/620(0%), Strand: Plus/Plus

Query  39    TAGGACTTAGTATAAGAATATTAATTCGTATAGAATTAGGACAACCTGGAACTTTAATTG  98
             ||||||||||||||||||||||||||||||||||||||||||||||||||||||||||||
Sbjct  1     TAGGACTTAGTATAAGAATATTAATTCGTATAGAATTAGGACAACCTGGAACTTTAATTG  60

Query  99    GAAATGATCAGATTTATAATGTAATCGTAACAGCACATGCATTTATTATAATTTTCTTTA  158
             ||||||||||||||||||||||||||||||||||||||||||||||||||||||| ||||
Sbjct  61    GAAATGATCAGATTTATAATGTAATCGTAACAGCACATGCATTTATTATAATTTTTTTTA  120

Query  159   TAGTTATACCAATCATAATCGGAGGGTTCGGAAATTGATTAGTTCCTATTATATTAGGTG  218
             ||||||||||||||||||||||||| || |||||||||||||| ||||| ||||| ||||
Sbjct  121   TAGTTATACCAATCATAATCGGAGGATTTGGAAATTGATTAGTCCCTATCATATTGGGTG  180

Query  219   CCCCAGATATAGCATTTCCACGAATAAATAATATAAGATTTTGACTTTTACCCCCCTCAT  278
             |||||||||||||||||||||||||||||||||||||||||||||||||||| || |||
Sbjct  181   CCCCAGATATAGCATTTCCACGAATAAATAATATAAGATTTTGACTTTTACCTCCTTCAC  240

Query  279   TATTTTTATTGATTAACTCTTCATTAATTGAATCCGGAGCTGGAACAGGATGAACAGTTT  338
             |||||||||| ||||||||||||||||||||||| |||||||| ||||||||||||||||
Sbjct  241   TATTTTTATTAATTAACTCTTCATTAATTGAATCTGGAGCTGGTACAGGATGAACAGTTT  300

Query  339   ATCCCCCTCTATCTTCAAATTTATCACATTATGGTCCATCAGTAGATTTAGCTATTTTTT  398
             | || ||||||||||||||||||||||||||||| || ||||||||||||||||||||||
Sbjct  301   ACCCTCCTCTATCTTCAAATTTATCACATTATGGCCCTTCAGTAGATTTAGCTATTTTTT  360

Query  399   CTCTTCACCTTGCTGGTGCTTCTTCAATTTTAGGTGCAATTAACTTCATCACAACTATTG  458
             ||||||| |||||||||||||||||||||||||||||||||||||| || ||||||||||
Sbjct  361   CTCTTCATCTTGCTGGTGCTTCTTCAATTTTAGGTGCAATTAACTTTATTACAACTATTG  420

Query  459   TAAATATACGATCAATTGGAATAACAATAGAACGAATACCATTATTTGTTTGATCTGTTT  518
             |||| ||||||||||||||||||||||||||||| |||||||||||||||||||||||||
Sbjct  421   TAAACATACGATCAATTGGAATAACAATAGAACGTATACCATTATTTGTTTGATCTGTTT  480

Query  519   TAATTACAGCTATTTTACTACTTCTATCTTTACCTGTATTAGCAGGTGCCATTACAATAT  578
             ||||||||||||||||| ||||| ||||||||||||||||||||||||||||||||||||
Sbjct  481   TAATTACAGCTATTTTATTACTTTTATCTTTACCTGTATTAGCAGGTGCCATTACAATAT  540

Query  579   TATTAACTGATCGAAACTTTAACACATCATTTTTTGATCCTTCAGGAGGAGGAGATCCCA  638
             | |||||||||||||||||||| ||||||||||||||||||||||| ||||||||||| |
Sbjct  541   TGTTAACTGATCGAAACTTTAATACATCATTTTTTGATCCTTCAGGGGGAGGAGATCCAA  600

Query  639   TTTTATATCAACACTTATTT  658
             ||||||||||||| ||||||
Sbjct  601   TTTTATATCAACATTTATTT  620
```

>Rhipicephalus sanguineus isolate SV4 cytochrome c oxidase subunit I (COX1) gene, partial cds; mitochondrial
Sequence ID: MW135448.1 Length: 630
Range 1: 1 to 620

Score:990 bits(536), Expect:0.0,
Identities:592/620(95%), Gaps:0/620(0%), Strand: Plus/Plus

```
Query  39    TAGGACTTAGTATAAGAATATTAATTCGTATAGAATTAGGACAACCTGGAACTTTAATTG  98
             ||||||||||||||||||||||||||||||||||||||||||||||||||||||||||||
Sbjct  1     TAGGACTTAGTATAAGAATATTAATTCGTATAGAATTAGGACAACCTGGAACTTTAATTG  60

Query  99    GAAATGATCAGATTTATAATGTAATCGTAACAGCACATGCATTTATTATAATTTTCTTTA  158
             ||||||||||||||||||||||||||||||||||||||||||||||||||||||| ||||
Sbjct  61    GAAATGATCAGATTTATAATGTAATCGTAACAGCACATGCATTTATTATAATTTTTTTTA  120

Query  159   TAGTTATACCAATCATAATCGGAGGGTTCGGAAATTGATTAGTTCCTATTATATTAGGTG  218
             ||||||||||||||||||||||||| || |||||||||||||| ||||| ||||| ||||
Sbjct  121   TAGTTATACCAATCATAATCGGAGGATTTGGAAATTGATTAGTCCCTATCATATTGGGTG  180

Query  219   CCCAGATATAGCATTTCCACGAATAAATAATATAAGATTTTGACTTTTACCCCCCTCAT  278
             |||||||||||||||||||||||||||||||||||||||||||||||||||| || |||
Sbjct  181   CCCAGATATAGCATTTCCACGAATAAATAATATAAGATTTTGACTTTTACCTCCTTCAC  240

Query  279   TATTTTTATTGATTAACTCTTCATTAATTGAATCCGGAGCTGGAACAGGATGAACAGTTT  338
             |||||||||| ||||||||||||||||||||||| |||||||| ||||||||||||||||
Sbjct  241   TATTTTTATTAATTAACTCTTCATTAATTGAATCTGGAGCTGGTACAGGATGAACAGTTT  300

Query  339   ATCCCCCTCTATCTTCAAATTTATCACATTATGGTCCATCAGTAGATTTAGCTATTTTTT  398
             | || ||||||||||||||||||||||||||||| || ||||||||||||||||||||||
Sbjct  301   ACCCTCCTCTATCTTCAAATTTATCACATTATGGCCCTTCAGTAGATTTAGCTATTTTTT  360

Query  399   CTCTTCACCTTGCTGGTGCTTCTTCAATTTTAGGTGCAATTAACTTCATCACAACTATTG  458
             ||||||| |||||||||||||||||||||||||||||||||||||| || ||||||||||
Sbjct  361   CTCTTCATCTTGCTGGTGCTTCTTCAATTTTAGGTGCAATTAACTTTATTACAACTATTG  420

Query  459   TAAATATACGATCAATTGGAATAACAATAGAACGAATACCATTATTTGTTTGATCTGTTT  518
             |||| ||||||||||||||||||||||||||||| |||||||||||||||||||||||||
Sbjct  421   TAAACATACGATCAATTGGAATAACAATAGAACGTATACCATTATTTGTTTGATCTGTTT  480

Query  519   TAATTACAGCTATTTTACTACTTCTATCTTTACCTGTATTAGCAGGTGCCATTACAATAT  578
             ||||||||||||||||| ||||| ||||||||||||||||||||||||||||||||||||
Sbjct  481   TAATTACAGCTATTTTATTACTTTTATCTTTACCTGTATTAGCAGGTGCCATTACAATAT  540

Query  579   TATTAACTGATCGAAACTTTAACACATCATTTTTTGATCCTTCAGGAGGAGGAGATCCCA  638
             | |||||||||||||||||||| ||||||||||||||||||||||| ||||||||||| |
Sbjct  541   TGTTAACTGATCGAAACTTTAATACATCATTTTTTGATCCTTCAGGGGGAGGAGATCCAA  600

Query  639   TTTTATATCAACACTTATTT  658
             ||||||||||||| ||||||
Sbjct  601   TTTTATATCAACATTTATTT  620
```

```
>Rhipicephalus sanguineus isolate Boka-A cytochrome oxidase subunit I (COI)
gene, partial cds; mitochondrial
Sequence ID: KX757905.1 Length: 630
Range 1: 1 to 620

Score:985 bits(533), Expect:0.0,
Identities:591/620(95%),  Gaps:0/620(0%), Strand: Plus/Plus

Query  39   TAGGACTTAGTATAAGAATATTAATTCGTATAGAATTAGGACAACCTGGAACTTTAATTG  98
            ||||||||||||||||||||||||||||||||||||||||||||||||||||||||||||
Sbjct  1    TAGGACTTAGTATAAGAATATTAATTCGTATAGAATTAGGACAACCTGGAACTTTAATTG  60

Query  99   GAAATGATCAGATTTATAATGTAATCGTAACAGCACATGCATTTATTATAATTTTCTTTA  158
            ||||||||||||||||||||||||||||||||||||||||||||||||||||||| ||||
Sbjct  61   GAAATGATCAGATTTATAATGTAATCGTAACAGCACATGCATTTATTATAATTTTTTTTA  120

Query  159  TAGTTATACCAATCATAATCGGAGGGTTCGGAAATTGATTAGTTCCTATTATATTAGGTG  218
            ||||||||||||||||||||||||| || |||||||||||||| ||||| ||||| ||||
Sbjct  121  TAGTTATACCAATCATAATCGGAGGATTTGGAAATTGATTAGTCCCTATCATATTGGGTG  180

Query  219  CCCCAGATATAGCATTTCCACGAATAAATAATATAAGATTTTGACTTTTACCCCCCTCAT  278
            |||||||||||||||||||||||||||||||||||||||||||||||||||| || ||| 
Sbjct  181  CCCCAGATATAGCATTTCCACGAATAAATAATATAAGATTTTGACTTTTACCTCCTTCAC  240

Query  279  TATTTTTATTGATTAACTCTTCATTAATTGAATCCGGAGCTGGAACAGGATGAACAGTTT  338
            |||||||||| ||||| ||||||||||||||||| |||||||| ||||||||||||||||
Sbjct  241  TATTTTTATTAATTAATTCTTCATTAATTGAATCTGGAGCTGGTACAGGATGAACAGTTT  300

Query  339  ATCCCCCTCTATCTTCAAATTTATCACATTATGGTCCATCAGTAGATTTAGCTATTTTTT  398
            | || ||||||||||||||||||||||||||||| || ||||||||||||||||||||||
Sbjct  301  ACCCTCCTCTATCTTCAAATTTATCACATTATGGCCCTTCAGTAGATTTAGCTATTTTTT  360

Query  399  CTCTTCACCTTGCTGGTGCTTCTTCAATTTTAGGTGCAATTAACTTCATCACAACTATTG  458
            ||||||| |||||||||||||||||||||||||||||||||||||| || ||||||||||
Sbjct  361  CTCTTCATCTTGCTGGTGCTTCTTCAATTTTAGGTGCAATTAACTTTATTACAACTATTG  420

Query  459  TAAATATACGATCAATTGGAATAACAATAGAACGAATACCATTATTTGTTTGATCTGTTT  518
            |||| |||||||||||||||||||||||||||||| ||||||||||||||||||||||||
Sbjct  421  TAAACATACGATCAATTGGAATAACAATAGAACGTATACCATTATTTGTTTGATCTGTTT  480

Query  519  TAATTACAGCTATTTTACTACTTCTATCTTTACCTGTATTAGCAGGTGCCATTACAATAT  578
            ||||||||||||||||| ||||| ||||||||||||||||||||||||||||||||||||
Sbjct  481  TAATTACAGCTATTTTATTACTTTTATCTTTACCTGTATTAGCAGGTGCCATTACAATAT  540

Query  579  TATTAACTGATCGAAACTTTAACACATCATTTTTTGATCCTTCAGGAGGAGGAGATCCCA  638
            | ||||||||||||||||||||| |||||||||||||||||||||| ||||||||||| |
Sbjct  541  TGTTAACTGATCGAAACTTTAATACATCATTTTTTGATCCTTCAGGGGGAGGAGATCCAA  600

Query  639  TTTTATATCAACACTTATTT  658
            ||||||||||||| ||||||
```

```
Sbjct  601  TTTTATATCAACATTTATTT  620
```

>Rhipicephalus sanguineus isolate Sibenik cytochrome oxidase subunit I (COI) gene, partial cds; mitochondrial
Sequence ID: KX757895.1 Length: 630
Range 1: 1 to 620

Score:985 bits(533), Expect:0.0,
Identities:591/620(95%), Gaps:0/620(0%), Strand: Plus/Plus

```
Query  39   TAGGACTTAGTATAAGAATATTAATTCGTATAGAATTAGGACAACCTGGAACTTTAATTG  98
            |||||||||||||||||||||||||||||||||| |||||||||||||||||||||||||
Sbjct  1    TAGGACTTAGTATAAGAATATTAATTCGTATAGAGTTAGGACAACCTGGAACTTTAATTG  60

Query  99   GAAATGATCAGATTTATAATGTAATCGTAACAGCACATGCATTTATTATAATTTTCTTTA  158
            ||||||||||||||||||||||||||||||||||||||||||||||||||||||| ||||
Sbjct  61   GAAATGATCAGATTTATAATGTAATCGTAACAGCACATGCATTTATTATAATTTTTTTTA  120

Query  159  TAGTTATACCAATCATAATCGGAGGGTTCGGAAATTGATTAGTTCCTATTATATTAGGTG  218
            ||||||||||||||||||||||||| || |||||||||||||| ||||| ||||| ||||
Sbjct  121  TAGTTATACCAATCATAATCGGAGGATTTGGAAATTGATTAGTCCCTATCATATTGGGTG  180

Query  219  CCCCAGATATAGCATTTCCACGAATAAATAATATAAGATTTTGACTTTTACCCCCCTCAT  278
            |||||||||||||||||||||||||||||||||||||||||||||||||||| || |||
Sbjct  181  CCCCAGATATAGCATTTCCACGAATAAATAATATAAGATTTTGACTTTTACCTCCTTCAC  240

Query  279  TATTTTTATTGATTAACTCTTCATTAATTGAATCCGGAGCTGGAACAGGATGAACAGTTT  338
            |||||||||| ||||||||||||||||||||||| |||||||| ||||||||||||||||
Sbjct  241  TATTTTTATTAATTAACTCTTCATTAATTGAATCTGGAGCTGGTACAGGATGAACAGTTT  300

Query  339  ATCCCCCTCTATCTTCAAATTTATCACATTATGGTCCATCAGTAGATTTAGCTATTTTTT  398
            | || ||||||||||||||||||||||||||||| || ||||||||||||||||||||||
Sbjct  301  ACCCTCCTCTATCTTCAAATTTATCACATTATGGCCCTTCAGTAGATTTAGCTATTTTTT  360

Query  399  CTCTTCACCTTGCTGGTGCTTCTTCAATTTTAGGTGCAATTAACTTCATCACAACTATTG  458
            ||||||| |||||||||||||||||||||||||||||||||||||| || ||||||||||
Sbjct  361  CTCTTCATCTTGCTGGTGCTTCTTCAATTTTAGGTGCAATTAACTTTATTACAACTATTG  420

Query  459  TAAATATACGATCAATTGGAATAACAATAGAACGAATACCATTATTTGTTTGATCTGTTT  518
            |||| ||||||||||||||||||||||||||||| |||||||||||||||||||||||||
Sbjct  421  TAAACATACGATCAATTGGAATAACAATAGAACGTATACCATTATTTGTTTGATCTGTTT  480

Query  519  TAATTACAGCTATTTTACTACTTCTATCTTTACCTGTATTAGCAGGTGCCATTACAATAT  578
            ||||||||||||||||| ||||| ||||||||||||||||||||||||||||||||||||
Sbjct  481  TAATTACAGCTATTTTATTACTTTTATCTTTACCTGTATTAGCAGGTGCCATTACAATAT  540

Query  579  TATTAACTGATCGAAACTTTAACACATCATTTTTTGATCCTTCAGGAGGAGGAGATCCCA  638
            | |||||||||||||||||||| ||||||||||||||||||||||| ||||||||||| |
Sbjct  541  TGTTAACTGATCGAAACTTTAATACATCATTTTTTGATCCTTCAGGGGGAGGAGATCCAA  600

Query  639  TTTTATATCAACACTTATTT  658
```

```
             ||||||||||||| ||||||
Sbjct  601   TTTTATATCAACATTTATTT  620
```

>Rhipicephalus sanguineus isolate Zadar cytochrome oxidase subunit I (COI) gene, partial cds; mitochondrial
Sequence ID: KX757892.1 Length: 630
Range 1: 1 to 620

Score:985 bits(533), Expect:0.0,
Identities:591/620(95%), Gaps:0/620(0%), Strand: Plus/Plus

```
Query  39    TAGGACTTAGTATAAGAATATTAATTCGTATAGAATTAGGACAACCTGGAACTTTAATTG  98
             |||||||||||||||||||||||||||||||||| |||||||||||||||||||||||||
Sbjct  1     TAGGACTTAGTATAAGAATATTAATTCGTATAGAGTTAGGACAACCTGGAACTTTAATTG  60

Query  99    GAAATGATCAGATTTATAATGTAATCGTAACAGCACATGCATTTATTATAATTTTCTTTA  158
             ||||||||||||||||||||||||||||||||||||||||||||||||||||||| ||||
Sbjct  61    GAAATGATCAGATTTATAATGTAATCGTAACAGCACATGCATTTATTATAATTTTTTTTA  120

Query  159   TAGTTATACCAATCATAATCGGAGGGTTCGGAAATTGATTAGTTCCTATTATATTAGGTG  218
             ||||||||||||||||||||||||| || |||||||||||||| ||||| ||||| ||||
Sbjct  121   TAGTTATACCAATCATAATCGGAGGATTTGGAAATTGATTAGTCCCTATCATATTGGGTG  180

Query  219   CCCCAGATATAGCATTTCCACGAATAAATAATATAAGATTTTGACTTTTACCCCCCTCAT  278
             |||||||||||||||||||||||||||||||||||||||||||||||||||| || ||| 
Sbjct  181   CCCCAGATATAGCATTTCCACGAATAAATAATATAAGATTTTGACTTTTACCTCCTTCAC  240

Query  279   TATTTTTATTGATTAACTCTTCATTAATTGAATCCGGAGCTGGAACAGGATGAACAGTTT  338
             |||||||||| ||||||||||||||||||||||| |||||||| ||||||||||||||||
Sbjct  241   TATTTTTATTAATTAACTCTTCATTAATTGAATCTGGAGCTGGTACAGGATGAACAGTTT  300

Query  339   ATCCCCCTCTATCTTCAAATTTATCACATTATGGTCCATCAGTAGATTTAGCTATTTTTT  398
             | || ||||||||||||||||||||||||||||| || ||||||||||||||||||||||
Sbjct  301   ACCCTCCTCTATCTTCAAATTTATCACATTATGGCCCTTCAGTAGATTTAGCTATTTTTT  360

Query  399   CTCTTCACCTTGCTGGTGCTTCTTCAATTTTAGGTGCAATTAACTTCATCACAACTATTG  458
             ||||||| |||||||||||||||||||||||||||||||||||||| || ||||||||||
Sbjct  361   CTCTTCATCTTGCTGGTGCTTCTTCAATTTTAGGTGCAATTAACTTTATTACAACTATTG  420

Query  459   TAAATATACGATCAATTGGAATAACAATAGAACGAATACCATTATTTGTTTGATCTGTTT  518
             |||| ||||||||||||||||||||||||||||| |||||||||||||||||||||||||
Sbjct  421   TAAACATACGATCAATTGGAATAACAATAGAACGTATACCATTATTTGTTTGATCTGTTT  480

Query  519   TAATTACAGCTATTTTACTACTTCTATCTTTACCTGTATTAGCAGGTGCCATTACAATAT  578
             ||||||||||||||||| ||||| ||||||||||||||||||||||||||||||||||||
Sbjct  481   TAATTACAGCTATTTTATTACTTTTATCTTTACCTGTATTAGCAGGTGCCATTACAATAT  540

Query  579   TATTAACTGATCGAAACTTTAACACATCATTTTTTGATCCTTCAGGAGGAGGAGATCCCA  638
             | ||||||||||||||||||||| |||||||||||||||||||||| ||||||||||| |
Sbjct  541   TGTTAACTGATCGAAACTTTAATACATCATTTTTTGATCCTTCAGGGGGAGGAGATCCAA  600
```

```
Query  639  TTTTATATCAACACTTATTT  658
            ||||||||||||| ||||||
Sbjct  601  TTTTATATCAACATTTATTT  620
```

>Rhipicephalus sanguineus isolate Zagreb-A cytochrome oxidase subunit I (COI) gene, partial cds; mitochondrial
Sequence ID: KX757890.1 Length: 630
Range 1: 1 to 620

Score:985 bits(533), Expect:0.0,
Identities:591/620(95%), Gaps:0/620(0%), Strand: Plus/Plus

```
Query  39   TAGGACTTAGTATAAGAATATTAATTCGTATAGAATTAGGACAACCTGGAACTTTAATTG  98
            |||||||||||||||||||||||||||||||||| |||||||||||||||||||||||||
Sbjct  1    TAGGACTTAGTATAAGAATATTAATTCGTATAGAGTTAGGACAACCTGGAACTTTAATTG  60

Query  99   GAAATGATCAGATTTATAATGTAATCGTAACAGCACATGCATTTATTATAATTTTCTTTA  158
            ||||||||||||||||||||||||||||||||||||||||||||||||||||||| ||||
Sbjct  61   GAAATGATCAGATTTATAATGTAATCGTAACAGCACATGCATTTATTATAATTTTTTTTA  120

Query  159  TAGTTATACCAATCATAATCGGAGGGTTCGGAAATTGATTAGTTCCTATTATATTAGGTG  218
            ||||||||||||||||||||||||| || |||||||||||||| ||||| ||||| ||||
Sbjct  121  TAGTTATACCAATCATAATCGGAGGATTTGGAAATTGATTAGTCCCTATCATATTGGGTG  180

Query  219  CCCCAGATATAGCATTTCCACGAATAAATAATATAAGATTTTGACTTTTACCCCCCTCAT  278
            |||||||||||||||||||||||||||||||||||||||||||||||||||| || |||
Sbjct  181  CCCCAGATATAGCATTTCCACGAATAAATAATATAAGATTTTGACTTTTACCTCCTTCAC  240

Query  279  TATTTTTATTGATTAACTCTTCATTAATTGAATCCGGAGCTGGAACAGGATGAACAGTTT  338
            |||||||||| |||||||||||||||||||||||| |||||||| |||||||||||||||
Sbjct  241  TATTTTTATTAATTAACTCTTCATTAATTGAATCTGGAGCTGGTACAGGATGAACAGTTT  300

Query  339  ATCCCCCTCTATCTTCAAATTTATCACATTATGGTCCATCAGTAGATTTAGCTATTTTTT  398
            | || ||||||||||||||||||||||||||||| || ||||||||||||||||||||||
Sbjct  301  ACCCTCCTCTATCTTCAAATTTATCACATTATGGCCCTTCAGTAGATTTAGCTATTTTTT  360

Query  399  CTCTTCACCTTGCTGGTGCTTCTTCAATTTTAGGTGCAATTAACTTCATCACAACTATTG  458
            ||||||| |||||||||||||||||||||||||||||||||||||| || ||||||||||
Sbjct  361  CTCTTCATCTTGCTGGTGCTTCTTCAATTTTAGGTGCAATTAACTTTATTACAACTATTG  420

Query  459  TAAATATACGATCAATTGGAATAACAATAGAACGAATACCATTATTTGTTTGATCTGTTT  518
            |||| |||||||||||||||||||||||||||||| ||||||||||||||||||||||||
Sbjct  421  TAAACATACGATCAATTGGAATAACAATAGAACGTATACCATTATTTGTTTGATCTGTTT  480

Query  519  TAATTACAGCTATTTTACTACTTCTATCTTTACCTGTATTAGCAGGTGCCATTACAATAT  578
            ||||||||||||||||| ||||| ||||||||||||||||||||||||||||||||||||
Sbjct  481  TAATTACAGCTATTTTATTACTTTTATCTTTACCTGTATTAGCAGGTGCCATTACAATAT  540

Query  579  TATTAACTGATCGAAACTTTAACACATCATTTTTTGATCCTTCAGGAGGAGGAGATCCCA  638
            | ||||||||||||||||||||| |||||||||||||||||||||| ||||||||||| |
Sbjct  541  TGTTAACTGATCGAAACTTTAATACATCATTTTTTGATCCTTCAGGGGGAGGAGATCCAA  600
```

```
Query  639   TTTTATATCAACACTTATTT  658
             ||||||||||||| ||||||
Sbjct  601   TTTTATATCAACATTTATTT  620
```

>Rhipicephalus sanguineus isolate Pula-A cytochrome oxidase subunit I (COI) gene, partial cds; mitochondrial
Sequence ID: KX757888.1 Length: 630
Range 1: 1 to 620

Score:985 bits(533), Expect:0.0,
Identities:591/620(95%), Gaps:0/620(0%), Strand: Plus/Plus

```
Query  39    TAGGACTTAGTATAAGAATATTAATTCGTATAGAATTAGGACAACCTGGAACTTTAATTG  98
             ||||||||||||||||||||||||||||||||||||||||||||||||||||||||||||
Sbjct  1     TAGGACTTAGTATAAGAATATTAATTCGTATAGAATTAGGACAACCTGGAACTTTAATTG  60

Query  99    GAAATGATCAGATTTATAATGTAATCGTAACAGCACATGCATTTATTATAATTTTCTTTA  158
             ||||||||||||||||||||||||||||||||||||||||||||||||||||||| ||||
Sbjct  61    GAAATGATCAGATTTATAATGTAATCGTAACAGCACATGCATTTATTATAATTTTTTTTA  120

Query  159   TAGTTATACCAATCATAATCGGAGGGTTCGGAAATTGATTAGTTCCTATTATATTAGGTG  218
             ||||||||||||||||||||||||| || |||||||||||||| ||||| ||||| ||||
Sbjct  121   TAGTTATACCAATCATAATCGGAGGATTTGGAAATTGATTAGTCCCTATCATATTGGGTG  180

Query  219   CCCCAGATATAGCATTTCCACGAATAAATAATATAAGATTTTGACTTTTACCCCCCTCAT  278
             |||||||||||||||||||||||||||||||||||||||||||||||||||| || ||| 
Sbjct  181   CCCCAGATATAGCATTTCCACGAATAAATAATATAAGATTTTGACTTTTACCTCCTTCAC  240

Query  279   TATTTTTATTGATTAACTCTTCATTAATTGAATCCGGAGCTGGAACAGGATGAACAGTTT  338
             |||||||||| ||||| ||||||||||||||||| |||||||| ||||||||||||||||
Sbjct  241   TATTTTTATTAATTAATTCTTCATTAATTGAATCTGGAGCTGGTACAGGATGAACAGTTT  300

Query  339   ATCCCCCTCTATCTTCAAATTTATCACATTATGGTCCATCAGTAGATTTAGCTATTTTTT  398
             | || ||||||||||||||||||||||||||||| || ||||||||||||||||||||||
Sbjct  301   ACCCTCCTCTATCTTCAAATTTATCACATTATGGCCCTTCAGTAGATTTAGCTATTTTTT  360

Query  399   CTCTTCACCTTGCTGGTGCTTCTTCAATTTTAGGTGCAATTAACTTCATCACAACTATTG  458
             ||||||| |||||||||||||||||||||||||||||||||||||| || ||||||||||
Sbjct  361   CTCTTCATCTTGCTGGTGCTTCTTCAATTTTAGGTGCAATTAACTTTATTACAACTATTG  420

Query  459   TAAATATACGATCAATTGGAATAACAATAGAACGAATACCATTATTTGTTTGATCTGTTT  518
             |||| ||||||||||||||||||||||||||||| |||||||||||||||||||||||||
Sbjct  421   TAAACATACGATCAATTGGAATAACAATAGAACGTATACCATTATTTGTTTGATCTGTTT  480

Query  519   TAATTACAGCTATTTTACTACTTCTATCTTTACCTGTATTAGCAGGTGCCATTACAATAT  578
             ||||||||||||||||| ||||| ||||||||||||||||||||||||||||||||||||
Sbjct  481   TAATTACAGCTATTTTATTACTTTTATCTTTACCTGTATTAGCAGGTGCCATTACAATAT  540

Query  579   TATTAACTGATCGAAACTTTAACACATCATTTTTTGATCCTTCAGGAGGAGGAGATCCCA  638
             | |||||||||||||||||||| ||||||||||||||||||||||| ||||||||||| |
```

```
Sbjct  541   TGTTAACTGATCGAAACTTTAATACATCATTTTTTGATCCTTCAGGGGGAGGAGATCCAA  600

Query  639   TTTTATATCAACACTTATTT  658
             ||||||||||||| ||||||
Sbjct  601   TTTTATATCAACATTTATTT  620
```

>Rhipicephalus sanguineus isolate Petnica cytochrome oxidase subunit I (COI) gene, partial cds; mitochondrial
Sequence ID: KX757883.1 Length: 630
Range 1: 1 to 620

Score:985 bits(533), Expect:0.0,
Identities:591/620(95%), Gaps:0/620(0%), Strand: Plus/Plus

```
Query  39    TAGGACTTAGTATAAGAATATTAATTCGTATAGAATTAGGACAACCTGGAACTTTAATTG  98
             |||||||||||||||||||||||||||||||||| |||||||||||||||||||||||||
Sbjct  1     TAGGACTTAGTATAAGAATATTAATTCGTATAGAGTTAGGACAACCTGGAACTTTAATTG  60

Query  99    GAAATGATCAGATTTATAATGTAATCGTAACAGCACATGCATTATTATAATTTTCTTTA  158
             ||||||||||||||||||||||||||||||||||||||||||||||||||||||| ||||
Sbjct  61    GAAATGATCAGATTTATAATGTAATCGTAACAGCACATGCATTATTATAATTTTTTTTA  120

Query  159   TAGTTATACCAATCATAATCGGAGGGTTCGGAAATTGATTAGTTCCTATTATATTAGGTG  218
             ||||||||||||||||||||||||| || |||||||||||||| ||||| ||||| ||||
Sbjct  121   TAGTTATACCAATCATAATCGGAGGATTTGGAAATTGATTAGTCCCTATCATATTGGGTG  180

Query  219   CCCCAGATATAGCATTTCCACGAATAAATAATATAAGATTTTGACTTTTACCCCCCTCAT  278
             |||||||||||||||||||||||||||||||||||||||||||||||||||| || |||
Sbjct  181   CCCCAGATATAGCATTTCCACGAATAAATAATATAAGATTTTGACTTTTACCTCCTTCAC  240

Query  279   TATTTTTATTGATTAACTCTTCATTAATTGAATCCGGAGCTGGAACAGGATGAACAGTTT  338
             |||||||||| ||||||||||||||||||||||| |||||||| ||||||||||||||||
Sbjct  241   TATTTTTATTAATTAACTCTTCATTAATTGAATCTGGAGCTGGTACAGGATGAACAGTTT  300

Query  339   ATCCCCCTCTATCTTCAAATTTATCACATTATGGTCCATCAGTAGATTTAGCTATTTTTT  398
             | || ||||||||||||||||||||||||||||| || ||||||||||||||||||||||
Sbjct  301   ACCCTCCTCTATCTTCAAATTTATCACATTATGGCCCTTCAGTAGATTTAGCTATTTTTT  360

Query  399   CTCTTCACCTTGCTGGTGCTTCTTCAATTTTAGGTGCAATTAACTTCATCACAACTATTG  458
             ||||||| |||||||||||||||||||||||||||||||||||||| || ||||||||||
Sbjct  361   CTCTTCATCTTGCTGGTGCTTCTTCAATTTTAGGTGCAATTAACTTTATTACAACTATTG  420

Query  459   TAAATATACGATCAATTGGAATAACAATAGAACGAATACCATTATTTGTTTGATCTGTTT  518
             |||| ||||||||||||||||||||||||||||| |||||||||||||||||||||||||
Sbjct  421   TAAACATACGATCAATTGGAATAACAATAGAACGTATACCATTATTTGTTTGATCTGTTT  480

Query  519   TAATTACAGCTATTTTACTACTTCTATCTTTACCTGTATTAGCAGGTGCCATTACAATAT  578
             ||||||||||||||||| ||||| ||||||||||||||||||||||||||||||||||||
Sbjct  481   TAATTACAGCTATTTTATTACTTTTATCTTTACCTGTATTAGCAGGTGCCATTACAATAT  540

Query  579   TATTAACTGATCGAAACTTTAACACATCATTTTTTGATCCTTCAGGAGGAGGAGATCCCA  638
```

```
            | |||||||||||||||||||| ||||||||||||||||||||||| ||||||||||| |
Sbjct  541  TGTTAACTGATCGAAACTTTAATACATCATTTTTTGATCCTTCAGGGGGAGGAGATCCAA  600

Query  639  TTTTATATCAACACTTATTT  658
            ||||||||||||| ||||||
Sbjct  601  TTTTATATCAACATTTATTT  620
```

>Rhipicephalus sanguineus clone #3 cytochrome oxidase subunit I (COI) gene, partial cds; mitochondrial
Sequence ID: KX519708.1 Length: 629
Range 1: 4 to 620

Score:985 bits(533), Expect:0.0,
Identities:589/617(95%), Gaps:0/617(0%), Strand: Plus/Plus

```
Query  42   GACTTAGTATAAGAATATTAATTCGTATAGAATTAGGACAACCTGGAACTTTAATTGGAA  101
            ||||||||||||||||||||||||||||||||||||||||||||||||||||||||||||
Sbjct  4    GACTTAGTATAAGAATATTAATTCGTATAGAATTAGGACAACCTGGAACTTTAATTGGAA  63

Query  102  ATGATCAGATTTATAATGTAATCGTAACAGCACATGCATTTATTATAATTTTCTTTATAG  161
            |||||||||||||||||||||||||||||||||||||||||||||||||||| |||||||
Sbjct  64   ATGATCAGATTTATAATGTAATCGTAACAGCACATGCATTTATTATAATTTTTTTTATAG  123

Query  162  TTATACCAATCATAATCGGAGGGTTCGGAAATTGATTAGTTCCTATTATATTAGGTGCCC  221
            |||||||||||||||||||||| || |||||||||||||| ||||| ||||| |||||||
Sbjct  124  TTATACCAATCATAATCGGAGGATTTGGAAATTGATTAGTCCCTATCATATTGGGTGCCC  183

Query  222  CAGATATAGCATTTCCACGAATAAATAATATAAGATTTTGACTTTTACCCCCCTCATTAT  281
            ||||||||||||||||||||||||||||||||||||||||||||||||| || ||| |||
Sbjct  184  CAGATATAGCATTTCCACGAATAAATAATATAAGATTTTGACTTTTACCTCCTTCACTAT  243

Query  282  TTTTATTGATTAACTCTTCATTAATTGAATCCGGAGCTGGAACAGGATGAACAGTTTATC  341
            ||||||| ||||||||||||||||||||||| |||||||| ||||||||||||||||| |
Sbjct  244  TTTTATTAATTAACTCTTCATTAATTGAATCTGGAGCTGGTACAGGATGAACAGTTTACC  303

Query  342  CCCCTCTATCTTCAAATTTATCACATTATGGTCCATCAGTAGATTTAGCTATTTTTTCTC  401
            | ||||||||||||||||||||||||||||| || |||||||||||||||||||||||||
Sbjct  304  CTCCTCTATCTTCAAATTTATCACATTATGGCCCTTCAGTAGATTTAGCTATTTTTTCTC  363

Query  402  TTCACCTTGCTGGTGCTTCTTCAATTTTAGGTGCAATTAACTTCATCACAACTATTGTAA  461
            |||| |||||||||||||||||||||||||||||||||||||| || |||||||||||||
Sbjct  364  TTCATCTTGCTGGTGCTTCTTCAATTTTAGGTGCAATTAACTTTATTACAACTATTGTAA  423

Query  462  ATATACGATCAATTGGAATAACAATAGAACGAATACCATTATTTGTTTGATCTGTTTTAA  521
            | ||||||||||||||||||||||||||||| ||||||||||||||||||||||||||||
Sbjct  424  ACATACGATCAATTGGAATAACAATAGAACGTATACCATTATTTGTTTGATCTGTTTTAA  483

Query  522  TTACAGCTATTTTACTACTTCTATCTTTACCTGTATTAGCAGGTGCCATTACAATATTAT  581
            |||||||||||||| ||||| ||||||||||||||||||||||||||||||||||||| |
Sbjct  484  TTACAGCTATTTTATTACTTTTATCTTTACCTGTATTAGCAGGTGCCATTACAATATTGT  543
```

```
Query  582  TAACTGATCGAAACTTTAACACATCATTTTTTGATCCTTCAGGAGGAGGAGATCCCATTT  641
            ||||||||||||||||||| ||||||||||||||||||||||| ||||||||||| ||||
Sbjct  544  TAACTGATCGAAACTTTAATACATCATTTTTTGATCCTTCAGGGGGAGGAGATCCAATTT  603

Query  642  TATATCAACACTTATTT  658
            |||||||||| ||||||
Sbjct  604  TATATCAACATTTATTT  620
```

>Rhipicephalus sanguineus isolate SV2 cytochrome c oxidase subunit I (COX1) gene, partial cds; mitochondrial
Sequence ID: MW135447.1 Length: 630
Range 1: 1 to 620

Score:985 bits(533), Expect:0.0,
Identities:591/620(95%), Gaps:0/620(0%), Strand: Plus/Plus

```
Query  39   TAGGACTTAGTATAAGAATATTAATTCGTATAGAATTAGGACAACCTGGAACTTTAATTG  98
            ||||||||||||||||||||||||||||||||||||||||||||||||||||||||||||
Sbjct  1    TAGGACTTAGTATAAGAATATTAATTCGTATAGAATTAGGACAACCTGGAACTTTAATTG  60

Query  99   GAAATGATCAGATTTATAATGTAATCGTAACAGCACATGCATTTATTATAATTTTCTTTA  158
            ||||||||||||||||||||||||||||||||||||||||||||||||||||||| ||||
Sbjct  61   GAAATGATCAGATTTATAATGTAATCGTAACAGCACATGCATTTATTATAATTTTTTTTA  120

Query  159  TAGTTATACCAATCATAATCGGAGGGTTCGGAAATTGATTAGTTCCTATTATATTAGGTG  218
            ||||||||||||||||||||||||| || |||||||||||||| ||||| ||||| ||||
Sbjct  121  TAGTTATACCAATCATAATCGGAGGATTTGGAAATTGATTAGTCCCTATCATATTGGGTG  180

Query  219  CCCCAGATATAGCATTTCCACGAATAAATAATATAAGATTTTGACTTTTACCCCCCTCAT  278
            |||||||||||||||||||||||||||||||||||||||||||||||||||| || |||
Sbjct  181  CCCCAGATATAGCATTTCCACGAATAAATAATATAAGATTTTGACTTTTACCTCCTTCAC  240

Query  279  TATTTTTATTGATTAACTCTTCATTAATTGAATCCGGAGCTGGAACAGGATGAACAGTTT  338
            |||||||||| ||||||||||||||||||||||| |||||||| ||||||||||||||||
Sbjct  241  TATTTTTATTAATTAACTCTTCATTAATTGAATCTGGAGCTGGTACAGGATGAACAGTTT  300

Query  339  ATCCCCCTCTATCTTCAAATTTATCACATTATGGTCCATCAGTAGATTTAGCTATTTTTT  398
            | || |||||||||||||||||||||||||||||| || |||||||||||||||||||||
Sbjct  301  ACCCTCCTCTATCTTCAAATTTATCACATTATGGCCCTTCAGTAGATTTAGCTATTTTTT  360

Query  399  CTCTTCACCTTGCTGGTGCTTCTTCAATTTTAGGTGCAATTAACTTCATCACAACTATTG  458
            ||||||| |||||||||||||||||||||||||||||||||||||| || ||||||||||
Sbjct  361  CTCTTCATCTTGCTGGTGCTTCTTCAATTTTAGGTGCAATTAACTTTATTACAACTATTG  420

Query  459  TAAATATACGATCAATTGGAATAACAATAGAACGAATACCATTATTTGTTTGATCTGTTT  518
            |||| ||||||||||||||||||||||||||||| |||||||||||||||||||||||||
Sbjct  421  TAAACATACGATCAATTGGAATAACAATAGAACGTATACCATTATTTGTTTGATCTGTTT  480

Query  519  TAATTACAGCTATTTTACTACTTCTATCTTTACCTGTATTAGCAGGTGCCATTACAATAT  578
            ||||||||||||||||| ||||| ||||||||||||||||||||||||||||||||||||
Sbjct  481  TAATTACAGCTATTTTATTACTTTTATCTTTACCTGTATTAGCAGGTGCCATTACAATAT  540
```

```
Query  579  TATTAACTGATCGAAACTTTAACACATCATTTTTTGATCCTTCAGGAGGAGGAGATCCCA  638
            | |||||||||||||||||||| ||||||||||||||||||||||| |||||||| || |
Sbjct  541  TGTTAACTGATCGAAACTTTAATACATCATTTTTTGATCCTTCAGGGGGAGGAGACCCAA  600

Query  639  TTTTATATCAACACTTATTT  658
            ||||||||||||| ||||||
Sbjct  601  TTTTATATCAACATTTATTT  620
```

>Rhipicephalus sanguineus clone #2 cytochrome oxidase subunit I (COI) gene, partial cds; mitochondrial
Sequence ID: KX519707.1 Length: 626
Range 1: 1 to 616

Score:983 bits(532), Expect:0.0,
Identities:588/616(95%), Gaps:0/616(0%), Strand: Plus/Plus

```
Query  43   ACTTAGTATAAGAATATTAATTCGTATAGAATTAGGACAACCTGGAACTTTAATTGGAAA  102
            ||||||||||||||||||||||||||||||||||||||||||||||||||||||||||||
Sbjct  1    ACTTAGTATAAGAATATTAATTCGTATAGAATTAGGACAACCTGGAACTTTAATTGGAAA  60

Query  103  TGATCAGATTTATAATGTAATCGTAACAGCACATGCATTTATTATAATTTTCTTTATAGT  162
            ||||||||||||||||||||||||||||||||||||||||||||||||||| ||||||||
Sbjct  61   TGATCAGATTTATAATGTAATCGTAACAGCACATGCATTTATTATAATTTTTTTTATAGT  120

Query  163  TATACCAATCATAATCGGAGGGTTCGGAAATTGATTAGTTCCTATTATATTAGGTGCCCC  222
            ||||||||||||||||||||| || |||||||||||||| ||||| ||||| ||||||||
Sbjct  121  TATACCAATCATAATCGGAGGATTTGGAAATTGATTAGTCCCTATCATATTGGGTGCCCC  180

Query  223  AGATATAGCATTTCCACGAATAAATAATATAAGATTTTGACTTTTACCCCCCTCATTATT  282
            |||||||||||||||||||||||||||||||||||||||||||||||| || ||| ||||
Sbjct  181  AGATATAGCATTTCCACGAATAAATAATATAAGATTTTGACTTTTACCTCCTTCACTATT  240

Query  283  TTTATTGATTAACTCTTCATTAATTGAATCCGGAGCTGGAACAGGATGAACAGTTTATCC  342
            |||||| ||||||||||||||||||||||| |||||||| ||||||||||||||||| ||
Sbjct  241  TTTATTAATTAACTCTTCATTAATTGAATCTGGAGCTGGTACAGGATGAACAGTTTACCC  300

Query  343  CCCTCTATCTTCAAATTTATCACATTATGGTCCATCAGTAGATTTAGCTATTTTTTCTCT  402
             ||||||||||||||||||||||||||||| || ||||||||||||||||||||||||||
Sbjct  301  TCCTCTATCTTCAAATTTATCACATTATGGCCCTTCAGTAGATTTAGCTATTTTTTCTCT  360

Query  403  TCACCTTGCTGGTGCTTCTTCAATTTTAGGTGCAATTAACTTCATCACAACTATTGTAAA  462
            ||| |||||||||||||||||||||||||||||||||||||| || ||||||||||||||
Sbjct  361  TCATCTTGCTGGTGCTTCTTCAATTTTAGGTGCAATTAACTTTATTACAACTATTGTAAA  420

Query  463  TATACGATCAATTGGAATAACAATAGAACGAATACCATTATTTGTTTGATCTGTTTTAAT  522
             ||||||||||||||||||||||||||||| |||||||||||||||||||||||||||||
Sbjct  421  CATACGATCAATTGGAATAACAATAGAACGTATACCATTATTTGTTTGATCTGTTTTAAT  480

Query  523  TACAGCTATTTTACTACTTCTATCTTTACCTGTATTAGCAGGTGCCATTACAATATTATT  582
            ||||||||||||| ||||| ||||||||||||||||||||||||||||||||||||| ||
```

```
Sbjct  481   TACAGCTATTTTATTACTTTTATCTTTACCTGTATTAGCAGGTGCCATTACAATATTGTT  540

Query  583   AACTGATCGAAACTTTAACACATCATTTTTTGATCCTTCAGGAGGAGGAGATCCCATTTT  642
             |||||||||||||||||| |||||||||||||||||||||||| |||||||||| |||||
Sbjct  541   AACTGATCGAAACTTTAATACATCATTTTTTGATCCTTCAGGGGGAGGAGATCCAATTTT  600

Query  643   ATATCAACACTTATTT  658
             ||||||||| ||||||
Sbjct  601   ATATCAACATTTATTT  616
```

>Rhipicephalus sanguineus isolate Szekszard cytochrome oxidase subunit I (COI) gene, partial cds; mitochondrial
Sequence ID: KX757901.1 Length: 630
Range 1: 1 to 620

Score:979 bits(530), Expect:0.0,
Identities:590/620(95%), Gaps:0/620(0%), Strand: Plus/Plus

```
Query  39    TAGGACTTAGTATAAGAATATTAATTCGTATAGAATTAGGACAACCTGGAACTTTAATTG  98
             ||||||||||||||||||||||||||||||||||||||||||||||||||||||||||||
Sbjct  1     TAGGACTTAGTATAAGAATATTAATTCGTATAGAATTAGGACAACCTGGAACTTTAATTG  60

Query  99    GAAATGATCAGATTTATAATGTAATCGTAACAGCACATGCATTTATTATAATTTTCTTTA  158
             ||||||||||||||||||||||||||||||||||||||||||||||||||||||| ||||
Sbjct  61    GAAATGATCAGATTTATAATGTAATCGTAACAGCACATGCATTTATTATAATTTTTTTTA  120

Query  159   TAGTTATACCAATCATAATCGGAGGGTTCGGAAATTGATTAGTTCCTATTATATTAGGTG  218
             ||||||||||||||||||||||||| || |||||||||||||| ||||| ||||| ||||
Sbjct  121   TAGTTATACCAATCATAATCGGAGGATTTGGAAATTGATTAGTCCCTATCATATTGGGTG  180

Query  219   CCCCAGATATAGCATTTCCACGAATAAATAATATAAGATTTTGACTTTTACCCCCCTCAT  278
             |||||||||||||||||||||||||||||||||||||||||||||||||||| || |||
Sbjct  181   CCCCAGATATAGCATTTCCACGAATAAATAATATAAGATTTTGACTTTTACCTCCTTCAC  240

Query  279   TATTTTTATTGATTAACTCTTCATTAATTGAATCCGGAGCTGGAACAGGATGAACAGTTT  338
             |||||||||| ||||||||||||||||||||||| |||||||| ||||||||||||||||
Sbjct  241   TATTTTTATTAATTAACTCTTCATTAATTGAATCTGGAGCTGGTACAGGATGAACAGTTT  300

Query  339   ATCCCCCTCTATCTTCAAATTTATCACATTATGGTCCATCAGTAGATTTAGCTATTTTTT  398
             | || ||||||||||||||||||||||||||||| || ||||||||||||||||| ||||
Sbjct  301   ACCCTCCTCTATCTTCAAATTTATCACATTATGGCCCTTCAGTAGATTTAGCTATCTTTT  360

Query  399   CTCTTCACCTTGCTGGTGCTTCTTCAATTTTAGGTGCAATTAACTTCATCACAACTATTG  458
             ||||||| |||||||||||||||||||||||||||||||||||||| || ||||||||||
Sbjct  361   CTCTTCATCTTGCTGGTGCTTCTTCAATTTTAGGTGCAATTAACTTTATTACAACTATTG  420

Query  459   TAAATATACGATCAATTGGAATAACAATAGAACGAATACCATTATTTGTTTGATCTGTTT  518
             |||| ||||| ||||||||||||||||||||||| |||||||||||||||||||||||||
Sbjct  421   TAAACATACGGTCAATTGGAATAACAATAGAACGTATACCATTATTTGTTTGATCTGTTT  480

Query  519   TAATTACAGCTATTTTACTACTTCTATCTTTACCTGTATTAGCAGGTGCCATTACAATAT  578
```

```
            ||||||||||||||||| ||||| ||||||||||||||||||||||||||||||||||||
Sbjct  481  TAATTACAGCTATTTTATTACTTTTATCTTTACCTGTATTAGCAGGTGCCATTACAATAT  540

Query  579  TATTAACTGATCGAAACTTTAACACATCATTTTTTGATCCTTCAGGAGGAGGAGATCCCA  638
            | |||||||||||||||||||| ||||||||||||||||||||||| ||||||||||| |
Sbjct  541  TGTTAACTGATCGAAACTTTAATACATCATTTTTTGATCCTTCAGGGGGAGGAGATCCAA  600

Query  639  TTTTATATCAACACTTATTT  658
            ||||||||||||| ||||||
Sbjct  601  TTTTATATCAACATTTATTT  620
```

>Rhipicephalus sanguineus clone #4 cytochrome oxidase subunit I (COI) gene, partial cds; mitochondrial
Sequence ID: KX519709.1 Length: 635
Range 1: 2 to 621

Score:974 bits(527), Expect:0.0,
Identities:589/620(95%), Gaps:0/620(0%), Strand: Plus/Plus

```
Query  39   TAGGACTTAGTATAAGAATATTAATTCGTATAGAATTAGGACAACCTGGAACTTTAATTG  98
            ||||||||||||||||||||||||||||||||||||||||||||||||||||||||||||
Sbjct  2    TAGGACTTAGTATAAGAATATTAATTCGTATAGAATTAGGACAACCTGGAACTTTAATTG  61

Query  99   GAAATGATCAGATTTATAATGTAATCGTAACAGCACATGCATTTATTATAATTTTCTTTA  158
            |||||||||||||||||||||||||||||||||||||||| |||||||||||||| ||||
Sbjct  62   GAAATGATCAGATTTATAATGTAATCGTAACAGCACATGCTTTTATTATAATTTTTTTTA  121

Query  159  TAGTTATACCAATCATAATCGGAGGGTTCGGAAATTGATTAGTTCCTATTATATTAGGTG  218
            ||||||||||||||||||||||||| || |||||||||||||| ||||| ||||| ||||
Sbjct  122  TAGTTATACCAATCATAATCGGAGGATTTGGAAATTGATTAGTCCCTATCATATTGGGTG  181

Query  219  CCCCAGATATAGCATTTCCACGAATAAATAATATAAGATTTTGACTTTTACCCCCCTCAT  278
            |||| ||||||||||||||||||||||||||||||||||||||||||||||| || ||| 
Sbjct  182  CCCCGGATATAGCATTTCCACGAATAAATAATATAAGATTTTGACTTTTACCTCCTTCAC  241

Query  279  TATTTTTATTGATTAACTCTTCATTAATTGAATCCGGAGCTGGAACAGGATGAACAGTTT  338
            |||||||||| ||||||||||||||||||||||| |||||||| ||||||||||||||||
Sbjct  242  TATTTTTATTAATTAACTCTTCATTAATTGAATCTGGAGCTGGTACAGGATGAACAGTTT  301

Query  339  ATCCCCCTCTATCTTCAAATTTATCACATTATGGTCCATCAGTAGATTTAGCTATTTTTT  398
            | || ||||||||||||||||||||||||||||| || |||| |||||||||||||||||
Sbjct  302  ACCCTCCTCTATCTTCAAATTTATCACATTATGGCCCTTCAGGAGATTTAGCTATTTTTT  361

Query  399  CTCTTCACCTTGCTGGTGCTTCTTCAATTTTAGGTGCAATTAACTTCATCACAACTATTG  458
            ||||||| |||||||||||||||||||||||||||||||||||||| || ||||||||||
Sbjct  362  CTCTTCATCTTGCTGGTGCTTCTTCAATTTTAGGTGCAATTAACTTTATTACAACTATTG  421

Query  459  TAAATATACGATCAATTGGAATAACAATAGAACGAATACCATTATTTGTTTGATCTGTTT  518
            |||| ||||||||||||||||||||||||||||| |||||||||||||||||||||||||
Sbjct  422  TAAACATACGATCAATTGGAATAACAATAGAACGTATACCATTATTTGTTTGATCTGTTT  481
```

```
Query  519  TAATTACAGCTATTTTACTACTTCTATCTTTACCTGTATTAGCAGGTGCCATTACAATAT  578
            ||||||||||||||||| ||||| ||||||||||||||||||||||||||||||||||||
Sbjct  482  TAATTACAGCTATTTTATTACTTTTATCTTTACCTGTATTAGCAGGTGCCATTACAATAT  541

Query  579  TATTAACTGATCGAAACTTTAACACATCATTTTTTGATCCTTCAGGAGGAGGAGATCCCA  638
            | |||||||||||||||||||| |||||||||||||||||||||| |||||||||||| |
Sbjct  542  TGTTAACTGATCGAAACTTTAATACATCATTTTTTGATCCTTCAGGGGGAGGAGATCCAA  601

Query  639  TTTTATATCAACACTTATTT  658
            ||||||||||||| ||||||
Sbjct  602  TTTTATATCAACATTTATTT  621
```

>Rhipicephalus sanguineus isolate LIC4723B cytochrome oxidase subunit 1 (COI) gene, partial cds; mitochondrial
Sequence ID: KX714599.1 Length: 723
>Rhipicephalus sanguineus isolate LIC4750B cytochrome oxidase subunit 1 (COI) gene, partial cds; mitochondrial
Sequence ID: KX714600.1 Length: 723
Range 1: 1 to 597

Score:937 bits(507), Expect:0.0,
Identities:567/597(95%), Gaps:0/597(0%), Strand: Plus/Plus

```
Query  62   ATTCGTATAGAATTAGGACAACCTGGAACTTTAATTGGAAATGATCAGATTTATAATGTA  121
            ||||||||||||||||||||||||||||||||||||||||||||||||||||||||||||
Sbjct  1    ATTCGTATAGAATTAGGACAACCTGGAACTTTAATTGGAAATGATCAGATTTATAATGTA  60

Query  122  ATCGTAACAGCACATGCATTTATTATAATTTTCTTTATAGTTATACCAATCATAATCGGA  181
            |||||||||||||||||||||||||||||||| ||||||||||||||||||||||| |||
Sbjct  61   ATCGTAACAGCACATGCATTTATTATAATTTTTTTTATAGTTATACCAATCATAATTGGA  120

Query  182  GGGTTCGGAAATTGATTAGTTCCTATTATATTAGGTGCCCCAGATATAGCATTTCCACGA  241
            || || |||||||||||||| ||||| ||||| |||||||||||||||||||||||||||
Sbjct  121  GGATTTGGAAATTGATTAGTCCCTATCATATTGGGTGCCCCAGATATAGCATTTCCACGA  180

Query  242  ATAAATAATATAAGATTTTGACTTTTACCCCCCTCATTATTTTTATTGATTAACTCTTCA  301
            ||||||||||||||||||||||||||||| || ||| |||||||||| ||||||||||||
Sbjct  181  ATAAATAATATAAGATTTTGACTTTTACCTCCTTCACTATTTTTATTAATTAACTCTTCA  240

Query  302  TTAATTGAATCCGGAGCTGGAACAGGATGAACAGTTTATCCCCCTCTATCTTCAAATTTA  361
            ||||||||||| |||||||| ||||||||||||||||| || ||||||||||||||||||
Sbjct  241  TTAATTGAATCTGGAGCTGGTACAGGATGAACAGTTTACCCTCCTCTATCTTCAAATTTA  300

Query  362  TCACATTATGGTCCATCAGTAGATTTAGCTATTTTTTCTCTTCACCTTGCTGGTGCTTCT  421
            ||||||||||| || ||||||||||||||||| ||||||||||| |||||||||||||||
Sbjct  301  TCACATTATGGCCCTTCAGTAGATTTAGCTATCTTTTCTCTTCATCTTGCTGGTGCTTCT  360

Query  422  TCAATTTTAGGTGCAATTAACTTCATCACAACTATTGTAAATATACGATCAATTGGAATA  481
            ||||||||||||||||||||||| || |||||||||||||| ||||||||||||||||||
Sbjct  361  TCAATTTTAGGTGCAATTAACTTTATTACAACTATTGTAAACATACGATCAATTGGAATA  420
```

```
Query  482  ACAATAGAACGAATACCATTATTTGTTTGATCTGTTTTAATTACAGCTATTTTACTACTT  541
            ||||||||||| |||||||||||||||||||||||||||||||||||||||||| |||||
Sbjct  421  ACAATAGAACGTATACCATTATTTGTTTGATCTGTTTTAATTACAGCTATTTTATTACTT  480

Query  542  CTATCTTTACCTGTATTAGCAGGTGCCATTACAATATTATTAACTGATCGAAACTTTAAC  601
             ||||||||||||||||||||||||||||||||||||| ||||||||||||||||||||
Sbjct  481  TTATCTTTACCTGTATTAGCAGGTGCCATTACAATATTGTTAACTGATCGAAACTTTAAT  540

Query  602  ACATCATTTTTTGATCCTTCAGGAGGAGGAGATCCCATTTTATATCAACACTTATTT  658
            ||||||||||||||||||||||| ||||||||||| |||||||||||||| ||||||
Sbjct  541  ACATCATTTTTTGATCCTTCAGGGGGAGGAGATCCAATTTTATATCAACATTTATTT  597
```

>Rhipicephalus sanguineus clone #8 cytochrome oxidase subunit I (COI) gene, partial cds; mitochondrial
Sequence ID: KX519713.1 Length: 580
Range 1: 1 to 580

Score:933 bits(505), Expect:0.0,
Identities:555/580(96%), Gaps:0/580(0%), Strand: Plus/Plus

```
Query  43   ACTTAGTATAAGAATATTAATTCGTATAGAATTAGGACAACCTGGAACTTTAATTGGAAA  102
            ||||||||||||||||||||||||||||||||||||||||||||||||||||||||||||
Sbjct  1    ACTTAGTATAAGAATATTAATTCGTATAGAATTAGGACAACCTGGAACTTTAATTGGAAA  60

Query  103  TGATCAGATTTATAATGTAATCGTAACAGCACATGCATTTATTATAATTTTCTTTATAGT  162
            ||||||||||||||||||||||||||||||||||||||||||||||||||| ||||||||
Sbjct  61   TGATCAGATTTATAATGTAATCGTAACAGCACATGCATTTATTATAATTTTTTTTATAGT  120

Query  163  TATACCAATCATAATCGGAGGGTTCGGAAATTGATTAGTTCCTATTATATTAGGTGCCCC  222
            ||||||||||||||||||||| || |||||||||||||| ||||| ||||| ||||||||
Sbjct  121  TATACCAATCATAATCGGAGGATTTGGAAATTGATTAGTCCCTATCATATTGGGTGCCCC  180

Query  223  AGATATAGCATTTCCACGAATAAATAATATAAGATTTTGACTTTTACCCCCTCATTATT  282
            |||||||||||||||||||||||||||||||||||||||||||||||| || ||| ||||
Sbjct  181  AGATATAGCATTTCCACGAATAAATAATATAAGATTTTGACTTTTACCTCCTTCACTATT  240

Query  283  TTTATTGATTAACTCTTCATTAATTGAATCCGGAGCTGGAACAGGATGAACAGTTTATCC  342
            |||||| ||||||||||||||||||||||| |||||||| ||||||||||||||||| ||
Sbjct  241  TTTATTAATTAACTCTTCATTAATTGAATCTGGAGCTGGTACAGGATGAACAGTTTACCC  300

Query  343  CCCTCTATCTTCAAATTTATCACATTATGGTCCATCAGTAGATTTAGCTATTTTTTCTCT  402
             ||||||||||||||||||||||||||||| || ||||||||||||||||||||||||||
Sbjct  301  TCCTCTATCTTCAAATTTATCACATTATGGCCCTTCAGTAGATTTAGCTATTTTTTCTCT  360

Query  403  TCACCTTGCTGGTGCTTCTTCAATTTTAGGTGCAATTAACTTCATCACAACTATTGTAAA  462
            ||| |||||||||||||||||||||||||||||||||||||| || ||||||||||||||
Sbjct  361  TCATCTTGCTGGTGCTTCTTCAATTTTAGGTGCAATTAACTTTATTACAACTATTGTAAA  420

Query  463  TATACGATCAATTGGAATAACAATAGAACGAATACCATTATTTGTTTGATCTGTTTTAAT  522
             ||||||||||||||||||||||||||||| |||||||||||||||||||||||||||||
Sbjct  421  CATACGATCAATTGGAATAACAATAGAACGTATACCATTATTTGTTTGATCTGTTTTAAT  480
```

```
Query  523   TACAGCTATTTTACTACTTCTATCTTTACCTGTATTAGCAGGTGCCATTACAATATTATT  582
             ||||||||||||| ||||| ||||||||||||||||||||||||||||||||||||| ||
Sbjct  481   TACAGCTATTTTATTACTTTTATCTTTACCTGTATTAGCAGGTGCCATTACAATATTGTT  540

Query  583   AACTGATCGAAACTTTAACACATCATTTTTTGATCCTTCA  622
             |||||||||||||||||| |||||||||||||||||||||
Sbjct  541   AACTGATCGAAACTTTAATACATCATTTTTTGATCCTTCA  580
```

>Rhipicephalus sanguineus clone #1 cytochrome oxidase subunit I (COI) gene, partial cds; mitochondrial
Sequence ID: KX519706.1 Length: 595
Range 1: 1 to 570

Score:898 bits(486), Expect:0.0,
Identities:542/570(95%), Gaps:0/570(0%), Strand: Plus/Plus

```
Query  89    ACTTTAATTGGAAATGATCAGATTTATAATGTAATCGTAACAGCACATGCATTTATTATA  148
             ||||||||||||||||||||||||||||||||||||||||||||||||||||||||||||
Sbjct  1     ACTTTAATTGGAAATGATCAGATTTATAATGTAATCGTAACAGCACATGCATTTATTATA  60

Query  149   ATTTTCTTTATAGTTATACCAATCATAATCGGAGGGTTCGGAAATTGATTAGTTCCTATT  208
             ||||| ||||||||||||||||||||||||||||| || |||||||||||||| ||||| 
Sbjct  61    ATTTTTTTTATAGTTATACCAATCATAATCGGAGGATTTGGAAATTGATTAGTCCCTATC  120

Query  209   ATATTAGGTGCCCCAGATATAGCATTTCCACGAATAAATAATATAAGATTTTGACTTTTA  268
             ||||| ||||||||||||||||||||||||||||||||||||||||||||||||||||||
Sbjct  121   ATATTGGGTGCCCCAGATATAGCATTTCCACGAATAAATAATATAAGATTTTGACTTTTA  180

Query  269   CCCCCCTCATTATTTTTATTGATTAACTCTTCATTAATTGAATCCGGAGCTGGAACAGGA  328
             || || ||| |||||||||| ||||||||||||||||||||||| |||||||| ||||||
Sbjct  181   CCTCCTTCACTATTTTTATTAATTAACTCTTCATTAATTGAATCTGGAGCTGGTACAGGA  240

Query  329   TGAACAGTTTATCCCCCTCTATCTTCAAATTTATCACATTATGGTCCATCAGTAGATTTA  388
             ||||||||||| || ||||||||||||||||||||||||||||| || ||||||||||||
Sbjct  241   TGAACAGTTTACCCTCCTCTATCTTCAAATTTATCACATTATGGCCCTTCAGTAGATTTA  300

Query  389   GCTATTTTTTCTCTTCACCTTGCTGGTGCTTCTTCAATTTTAGGTGCAATTAACTTCATC  448
             ||||||||||||||||| ||||||||||||||||||||||||||||||||||||||| || 
Sbjct  301   GCTATTTTTTCTCTTCATCTTGCTGGTGCTTCTTCAATTTTAGGTGCAATTAACTTTATT  360

Query  449   ACAACTATTGTAAATATACGATCAATTGGAATAACAATAGAACGAATACCATTATTTGTT  508
             |||||||||||||| |||||||||||||||||||||||||||||| |||||||||||||||
Sbjct  361   ACAACTATTGTAAACATACGATCAATTGGAATAACAATAGAACGTATACCATTATTTGTT  420

Query  509   TGATCTGTTTTAATTACAGCTATTTTACTACTTCTATCTTTACCTGTATTAGCAGGTGCC  568
             |||||||||||||||||||||||||| |||||| ||||||||||||||||||||||||||
Sbjct  421   TGATCTGTTTTAATTACAGCTATTTTATTACTTTTATCTTTACCTGTATTAGCAGGTGCC  480

Query  569   ATTACAATATTATTAACTGATCGAAACTTTAACACATCATTTTTTGATCCTTCAGGAGGA  628
             ||||||||||| |||||||||||||||||||| ||||||||||||||||||||||| |||
```

```
Sbjct  481  ATTACAATATTGTTAACTGATCGAAACTTTAATACATCATTTTTTGATCCTTCAGGGGGA  540

Query  629  GGAGATCCCATTTTATATCAACACTTATTT  658
            |||||||| |||||||||||||| ||||||
Sbjct  541  GGAGATCCAATTTTATATCAACATTTATTT  570
```

>Rhipicephalus sanguineus isolate Rsang2 cytochrome oxidase subunit 1 gene, partial cds; mitochondrial
Sequence ID: KY678135.1 Length: 790
Range 1: 18 to 675

Score:883 bits(478), Expect:0.0,
Identities:599/659(91%), Gaps:2/659(0%), Strand: Plus/Plus

```
Query  1    AACAATATATTTAATTTTTGGAGCATGATCAGGTATATTAGGACTTAGTATAAGAATATT  60
            |||||||||||||||||||||||||||||| || ||||||||| | || ||||||||| |
Sbjct  18   AACAATATATTTAATTTTTGGAGCATGATCCGGAATATTAGGATTAAGAATAAGAATACT  77

Query  61   AATTCGTATAGAATTAGGACAACCTGGAACTTTAATTGGAAATGATCAGATTTATAATGT  120
            |||||||||||||||||| |||||||||||  ||||||| |||||||| |||||||||||
Sbjct  78   AATTCGTATAGAATTAGGCCAACCTGGAACCCTAATTGGTAATGATCAAATTTATAATGT  137

Query  121  AATCGTAACAGCACATGCATTTATTATAATTTTCTTTATAGTTATACCAATCATAATCGG  180
            ||| || ||||| |||||||||||||||||||| |||||||| |||||||||||||| ||
Sbjct  138  AATTGTTACAGCTCATGCATTTATTATAATTTTTTTTATAGTAATACCAATCATAATTGG  197

Query  181  AGGGTTCGGAAATTGATTAGTTCCTATTATATTAGGTGCCCCAGATATAGCATTTCCACG  240
             || || ||||| |||||||||||||||||| |||| || |||||||||||||| |||||
Sbjct  198  TGGATTTGGAAACTGATTAGTTCCTATTATACTAGGAGCTCCAGATATAGCATTCCCACG  257

Query  241  AATAAATAATATAAGATTTTGACTTTTACCCCCCTCATTATTTTTATTGATTAACTCTTC  300
            ||||||||||||||||||||||||| | || |||||||||||| |||| ||||| |||||
Sbjct  258  AATAAATAATATAAGATTTTGACTTCTTCCTCCCTCATTATTTATATTAATTAATTCTTC  317

Query  301  ATTAATTGAATCCGGAGCTGGAACAGGATGAACAGTTTATCC-CCCTCTATCTTCAAATT  359
            ||||||||| || ||||| || |||||||||||||||||||| |||| |||| |||||||
Sbjct  318  ATTAATTGAGTCAGGAGCAGGTACAGGATGAACAGTTTATCCTCCCT-TATCCTCAAATT  376

Query  360  TATCACATTATGGTCCATCAGTAGATTTAGCTATTTTTTCTCTTCACCTTGCTGGTGCTT  419
            ||||||||||||| |||||||||||||||||||||||||||||||| |||||||||||||
Sbjct  377  TATCACATTATGGGCCATCAGTAGATTTAGCTATTTTTTCTCTTCATCTTGCTGGTGCTT  436

Query  420  CTTCAATTTTAGGTGCAATTAACTTCATCACAACTATTGTAAATATACGATCAATTGGAA  479
            |||||||||||||||||||||| || || |||||||||||||||||||||||| |||||||
Sbjct  437  CTTCAATTTTAGGTGCAATTAATTTTATTACAACTATTGTAAATATACGATCTATTGGAA  496

Query  480  TAACAATAGAACGAATACCATTATTTGTTTGATCTGTTTTAATTACAGCTATTTTACTAC  539
            |||||||||||||||||||||||||||| ||||||||||||||||| || |||||| |||
Sbjct  497  TAACAATAGAACGAATACCATTATTTGTATGATCTGTTTTAATTACTGCAATTTTATTAC  556

Query  540  TTCTATCTTTACCTGTATTAGCAGGTGCCATTACAATATTATTAACTGATCGAAACTTTA  599
```

```
             |  ||||||||||||| ||||||||||||||||||||| ||||||| |||||||| ||||
Sbjct  557   TATTATCTTTACCTGTTTTAGCAGGTGCCATTACAATACTATTAACCGATCGAAATTTTA  616

Query  600   ACACATCATTTTTTGATCCTTCAGGAGGAGGAGATCCCATTTTATATCAACACTTATTT  658
             |||| ||||||||||| |||||||| ||||| ||||| |||||||||||||| ||||||
Sbjct  617   ACACTTCATTTTTTGACCCTTCAGGGGGAGGGGATCCAATTTTATATCAACATTTATTT  675
```

>Rhipicephalus turanicus isolate COX1-225-Goat cytochrome c oxidase subunit I (COX1) gene, partial cds; mitochondrial
Sequence ID: MT800313.1 Length: 673
Range 1: 8 to 665

Score:883 bits(478), Expect:0.0,
Identities:598/658(91%), Gaps:0/658(0%), Strand: Plus/Plus

```
Query  1     AACAATATATTTAATTTTTGGAGCATGATCAGGTATATTAGGACTTAGTATAAGAATATT  60
             ||||||||||||||||||||| ||||||||||| ||||||||||| || ||||||||| |
Sbjct  8     AACAATATATTTAATTTTTGGGGCATGATCAGGAATATTAGGACTGAGAATAAGAATACT  67

Query  61    AATTCGTATAGAATTAGGACAACCTGGAACTTTAATTGGAAATGATCAGATTTATAATGT  120
             |||||| ||||||||||||||||||||||||||||||||||||||||| |||||||| ||
Sbjct  68    AATTCGCATAGAATTAGGACAACCTGGAACTTTAATTGGAAATGATCAAATTTATAACGT  127

Query  121   AATCGTAACAGCACATGCATTTATTATAATTTTCTTTATAGTTATACCAATCATAATCGG  180
             ||| |||||||| |||||||||||||||||||| ||||| || ||||| || ||||| ||
Sbjct  128   AATTGTAACAGCTCATGCATTTATTATAATTTTTTTTATGGTAATACCTATTATAATTGG  187

Query  181   AGGGTTCGGAAATTGATTAGTTCCTATTATATTAGGTGCCCCAGATATAGCATTTCCACG  240
              || || ||||||||||||||||||||||||||||| || |||||||||||||| |||||
Sbjct  188   TGGATTTGGAAATTGATTAGTTCCTATTATATTAGGGGCTCCAGATATAGCATTCCCACG  247

Query  241   AATAAATAATATAAGATTTTGACTTTTACCCCCCTCATTATTTTTATTGATTAACTCTTC  300
              |||||||| |||||||||||| |  | || || ||||||||| |||| |||||||||||
Sbjct  248   TATAAATAACATAAGATTTTGATTACTTCCTCCTTCATTATTTCTATTAATTAACTCTTC  307

Query  301   ATTAATTGAATCCGGAGCTGGAACAGGATGAACAGTTTATCCCCCTCTATCTTCAAATTT  360
             |||||||||||| ||||| |||||||||||||| |||||||||||| |||| ||||||||
Sbjct  308   ATTAATTGAATCAGGAGCAGGAACAGGATGAACTGTTTATCCCCCTTTATCCTCAAATTT  367

Query  361   ATCACATTATGGTCCATCAGTAGATTTAGCTATTTTTCTCTTCACCTTGCTGGTGCTTC  420
             |||||||||||| |||||||||||||||||||||||||||||||| ||||||||||||||
Sbjct  368   ATCACATTATGGACCATCAGTAGATTTAGCTATTTTTCTCTTCATCTTGCTGGTGCTTC  427

Query  421   TTCAATTTTAGGTGCAATTAACTTCATCACAACTATTGTAAATATACGATCAATTGGAAT  480
             ||||||| ||||||||||||| || || ||||||||||||||||||||||| ||||||||
Sbjct  428   TTCAATTCTAGGTGCAATTAATTTTATTACAACTATTGTAAATATACGATCTATTGGAAT  487

Query  481   AACAATAGAACGAATACCATTATTTGTTTGATCTGTTTTAATTACAGCTATTTTACTACT  540
             ||||||||||||||||||||||||||||||||||||||||||||| || || ||||| ||
Sbjct  488   AACAATAGAACGAATACCATTATTTGTTTGATCTGTTTTAATTACCGCCATCTTACTTCT  547
```

```
Query  541  TCTATCTTTACCTGTATTAGCAGGTGCCATTACAATATTATTAACTGATCGAAACTTTAA  600
              ||||||||||||| ||||||||||| |||||||||||||||||||||||||| || ||
Sbjct  548  CTTATCTTTACCTGTTTTAGCAGGTGCTATTACAATATTATTAACTGATCGAAATTTCAA  607

Query  601  CACATCATTTTTTGATCCTTCAGGAGGAGGAGATCCCATTTTATATCAACACTTATTT  658
             || |||||||||||||||||||| ||||| ||||| |||||||| ||||||||||||
Sbjct  608  TACTTCATTTTTTGATCCTTCAGGGGGAGGTGATCCAATTTTATACCAACACTTATTT  665
```

>Rhipicephalus sanguineus sensu lato 'temperate lineage' isolate SC3005 cytochrome c oxidase subunit I (COI) gene, partial cds; mitochondrial
Sequence ID: MF425987.1 Length: 574
Range 1: 1 to 561

Score:881 bits(477), Expect:0.0,
Identities:533/561(95%), Gaps:0/561(0%), Strand: Plus/Plus

```
Query  98   GGAAATGATCAGATTTATAATGTAATCGTAACAGCACATGCATTTATTATAATTTTCTTT  157
            |||||||||||||||||||||||||||||||||||||| ||||||||||||||||| |||
Sbjct  1    GGAAATGATCAGATTTATAATGTAATCGTAACAGCACACGCATTTATTATAATTTTTTTT  60

Query  158  ATAGTTATACCAATCATAATCGGAGGGTTCGGAAATTGATTAGTTCCTATTATATTAGGT  217
            |||||||||||||||||||||||||| || |||||||||||||| |||||||||||||||
Sbjct  61   ATAGTTATACCAATCATAATCGGAGGATTTGGAAATTGATTAGTCCCTATTATATTAGGT  120

Query  218  GCCCCAGATATAGCATTTCCACGAATAAATAATATAAGATTTTGACTTTTACCCCCCTCA  277
            ||||||||||||||||||||||||||||||||||||||||||||||||||||| || |||
Sbjct  121  GCCCCAGATATAGCATTTCCACGAATAAATAATATAAGATTTTGACTTTTACCTCCTTCA  180

Query  278  TTATTTTTATTGATTAACTCTTCATTAATTGAATCCGGAGCTGGAACAGGATGAACAGTT  337
             |||||||||| ||||||||||||||||||||||| |||||||| |||||||||||||||
Sbjct  181  CTATTTTTATTAATTAACTCTTCATTAATTGAATCTGGAGCTGGTACAGGATGAACAGTT  240

Query  338  TATCCCCCTCTATCTTCAAATTTATCACATTATGGTCCATCAGTAGATTTAGCTATTTTT  397
            || || ||||||||||||||||||||||||||||| || ||||||||||||||||| |||
Sbjct  241  TACCCTCCTCTATCTTCAAATTTATCACATTATGGCCCTTCAGTAGATTTAGCTATCTTT  300

Query  398  TCTCTTCACCTTGCTGGTGCTTCTTCAATTTTAGGTGCAATTAACTTCATCACAACTATT  457
            |||||||| |||||||||||||||||||||||||||||||||||||| || |||||||||
Sbjct  301  TCTCTTCATCTTGCTGGTGCTTCTTCAATTTTAGGTGCAATTAACTTTATTACAACTATT  360

Query  458  GTAAATATACGATCAATTGGAATAACAATAGAACGAATACCATTATTTGTTTGATCTGTT  517
            ||||| ||||||||||||||||||||||||||||| ||||||||||||||||||||||||
Sbjct  361  GTAAACATACGATCAATTGGAATAACAATAGAACGTATACCATTATTTGTTTGATCTGTT  420

Query  518  TTAATTACAGCTATTTTACTACTTCTATCTTTACCTGTATTAGCAGGTGCCATTACAATA  577
            |||||||||||||||||| ||||| |||||||||||||||||||||||||||||||||||
Sbjct  421  TTAATTACAGCTATTTTATTACTTTTATCTTTACCTGTATTAGCAGGTGCCATTACAATA  480

Query  578  TTATTAACTGATCGAAACTTTAACACATCATTTTTTGATCCTTCAGGAGGAGGAGATCCC  637
            || |||||||||||||||||||| ||||||||||||||||||||||| |||||||||||
Sbjct  481  TTGTTAACTGATCGAAACTTTAATACATCATTTTTTGATCCTTCAGGGGGAGGAGATCCA  540
```

```
Query  638  ATTTTATATCAACACTTATTT  658
            |||||||||||||| ||||||
Sbjct  541  ATTTTATATCAACATTTATTT  561
```

>Rhipicephalus sanguineus cytochrome oxidase subunit 1 (CO1) gene, partial cds; mitochondrial
Sequence ID: KU214592.1 Length: 829
Range 1: 1 to 648

Score:876 bits(474), Expect:0.0,
Identities:590/648(91%), Gaps:0/648(0%), Strand: Plus/Plus

```
Query  11   TTAATTTTTGGAGCATGATCAGGTATATTAGGACTTAGTATAAGAATATTAATTCGTATA  70
            |||||||||||||||||||| || ||||||||| | || ||||||||| |||||||||||
Sbjct  1    TTAATTTTTGGAGCATGATCCGGAATATTAGGATTAAGAATAAGAATACTAATTCGTATA  60

Query  71   GAATTAGGACAACCTGGAACTTTAATTGGAAATGATCAGATTTATAATGTAATCGTAACA  130
            |||||||| |||||||||||| ||||||| |||||||| |||||||||||||| || |||
Sbjct  61   GAATTAGGCCAACCTGGAACTCTAATTGGTAATGATCAAATTTATAATGTAATTGTTACA  120

Query  131  GCACATGCATTTATTATAATTTTCTTTATAGTTATACCAATCATAATCGGAGGGTTCGGA  190
            || |||||||||||||||||||| |||||||| |||||||||||||| || || || |||
Sbjct  121  GCTCATGCATTTATTATAATTTTTTTTATAGTAATACCAATCATAATTGGTGGATTTGGA  180

Query  191  AATTGATTAGTTCCTATTATATTAGGTGCCCCAGATATAGCATTTCCACGAATAAATAAT  250
            || |||||||||||||||||| |||| || ||||||||||||||||||||||||||||||
Sbjct  181  AACTGATTAGTTCCTATTATACTAGGAGCTCCAGATATAGCATTTCCACGAATAAATAAT  240

Query  251  ATAAGATTTTGACTTTTACCCCCCTCATTATTTTTATTGATTAACTCTTCATTAATTGAA  310
            ||||||||||||||| | || |||||||||||| |||| ||||| ||||||||||||||
Sbjct  241  ATAAGATTTTGACTTCTTCCTCCCTCATTATTTATATTAATTAATTCTTCATTAATTGAG  300

Query  311  TCCGGAGCTGGAACAGGATGAACAGTTTATCCCCCTCTATCTTCAAATTTATCACATTAT  370
            || ||||| || |||||||| ||||||||||| || ||||| ||||||||||||||||||
Sbjct  301  TCAGGAGCAGGTACAGGATGGACAGTTTATCCTCCCCTATCCTCAAATTTATCACATTAT  360

Query  371  GGTCCATCAGTAGATTTAGCTATTTTTTCTCTTCACCTTGCTGGTGCTTCTTCAATTTTA  430
            || |||||||||||||||||||||||||||||||| ||||||||||||||||||||||||
Sbjct  361  GGGCCATCAGTAGATTTAGCTATTTTTTCTCTTCATCTTGCTGGTGCTTCTTCAATTTTA  420

Query  431  GGTGCAATTAACTTCATCACAACTATTGTAAATATACGATCAATTGGAATAACAATAGAA  490
            ||||||||||| || || ||||||||||||||||||||||| ||||||||||||||||||
Sbjct  421  GGTGCAATTAATTTTATTACAACTATTGTAAATATACGATCTATTGGAATAACAATAGAA  480

Query  491  CGAATACCATTATTTGTTTGATCTGTTTTAATTACAGCTATTTTACTACTTCTATCTTTA  550
            ||||||||||||||||| ||||||||||||||||| || |||||| ||||  ||||||||
Sbjct  481  CGAATACCATTATTTGTATGATCTGTTTTAATTACTGCAATTTTATTACTATTATCTTTA  540

Query  551  CCTGTATTAGCAGGTGCCATTACAATATTATTAACTGATCGAAACTTTAACACATCATTT  610
            ||||| ||||||||||||||||||||||||||||| |||||||| |||||||| ||||||
```

```
Sbjct  541  CCTGTTTTAGCAGGTGCCATTACAATATTATTAACCGATCGAAATTTTAACACTTCATTT  600

Query  611  TTTGATCCTTCAGGAGGAGGAGATCCCATTTTATATCAACACTTATTT  658
            ||||| |||||||| ||||| ||||| |||||||||||||| ||||||
Sbjct  601  TTTGACCCTTCAGGGGGAGGGGATCCAATTTTATATCAACATTTATTT  648
```

>Rhipicephalus turanicus isolate GY34-1 cytochrome c oxidase subunit I (COX1) gene, partial cds; mitochondrial
Sequence ID: MT079206.1 Length: 678
Range 1: 1 to 652

Score:872 bits(472), Expect:0.0,
Identities:592/652(91%), Gaps:0/652(0%), Strand: Plus/Plus

```
Query  7    ATATTTAATTTTTGGAGCATGATCAGGTATATTAGGACTTAGTATAAGAATATTAATTCG  66
            ||||||||||||||||||||||||||||||||||||| | || |||||||||||||||||
Sbjct  1    ATATTTAATTTTTGGAGCATGATCAGGTATATTAGGATTAAGAATAAGAATATTAATTCG  60

Query  67   TATAGAATTAGGACAACCTGGAACTTTAATTGGAAATGATCAGATTTATAATGTAATCGT  126
             ||||||||||||||||||||||||||||||||||||||||| |||||||||||||| ||
Sbjct  61   AATAGAATTAGGACAACCTGGAACTTTAATTGGAAATGATCAAATTTATAATGTAATTGT  120

Query  127  AACAGCACATGCATTTATTATAATTTTCTTTATAGTTATACCAATCATAATCGGAGGGTT  186
            |||||| |||||||||||||||||||| |||||||| |||||||||||||| ||||| ||
Sbjct  121  AACAGCTCATGCATTTATTATAATTTTTTTTATAGTAATACCAATCATAATTGGAGGATT  180

Query  187  CGGAAATTGATTAGTTCCTATTATATTAGGTGCCCCAGATATAGCATTTCCACGAATAAA  246
             ||||| || |||||||| ||||||||||| || ||||| ||||||||||||||||||||
Sbjct  181  TGGAAACTGGTTAGTTCCAATTATATTAGGAGCTCCAGACATAGCATTTCCACGAATAAA  240

Query  247  TAATATAAGATTTTGACTTTTACCCCCCTCATTATTTTTATTGATTAACTCTTCATTAAT  306
            |||||||||||||||| |  | |||||||||||||| ||||| ||||| |||||| | ||
Sbjct  241  TAATATAAGATTTTGATTACTTCCCCCCTCATTATTCTTATTAATTAATTCTTCACTGAT  300

Query  307  TGAATCCGGAGCTGGAACAGGATGAACAGTTTATCCCCCTCTATCTTCAAATTTATCACA  366
            |||||| || || || ||||| ||||| |||||||| ||| |||| ||||||||||| ||
Sbjct  301  TGAATCTGGGGCAGGGACAGGGTGAACTGTTTATCCTCCTTTATCCTCAAATTTATCCCA  360

Query  367  TTATGGTCCATCAGTAGATTTAGCTATTTTTTCTCTTCACCTTGCTGGTGCTTCTTCAAT  426
            |||||| |||||||||||||||||||| || |||||||| ||||||||||||||||||||
Sbjct  361  TTATGGACCATCAGTAGATTTAGCTATCTTCTCTCTTCATCTTGCTGGTGCTTCTTCAAT  420

Query  427  TTTAGGTGCAATTAACTTCATCACAACTATTGTAAATATACGATCAATTGGAATAACAAT  486
            |||||| |||||||| ||||| |||||||||||||| |||||||| ||||||||||||||
Sbjct  421  TTTAGGCGCAATTAATTTCATTACAACTATTGTAAACATACGATCTATTGGAATAACAAT  480

Query  487  AGAACGAATACCATTATTTGTTTGATCTGTTTTAATTACAGCTATTTTACTACTTCTATC  546
            |||||||||||||||||| ||||||||||||||||| || ||||||||| | ||||||||
Sbjct  481  AGAACGAATACCATTATTCGTTTGATCTGTTTTAATCACCGCTATTTTATTGCTTCTATC  540

Query  547  TTTACCTGTATTAGCAGGTGCCATTACAATATTATTAACTGATCGAAACTTTAACACATC  606
```

```
            ||||||||| ||||||||||||||||||||| |||||||||||||||| |||||||| ||
Sbjct  541  TTTACCTGTTTTAGCAGGTGCCATTACAATACTATTAACTGATCGAAATTTTAACACTTC  600

Query  607  ATTTTTTGATCCTTCAGGAGGAGGAGATCCCATTTTATATCAACACTTATTT  658
            ||||||||| |||||||||||||| || || |||||||||||||| ||||||
Sbjct  601  ATTTTTTGACCCTTCAGGAGGAGGTGACCCAATTTTATATCAACATTTATTT  652
```

>Rhipicephalus sanguineus isolate GY34 cytochrome c oxidase subunit I (COX1)
gene, partial cds; mitochondrial
Sequence ID: MN862754.1 Length: 678
Range 1: 1 to 652

Score:872 bits(472), Expect:0.0,
Identities:592/652(91%), Gaps:0/652(0%), Strand: Plus/Plus

```
Query  7    ATATTTAATTTTTGGAGCATGATCAGGTATATTAGGACTTAGTATAAGAATATTAATTCG  66
            ||||||||||||||||||||||||||||||||||||| | || |||||||||||||||||
Sbjct  1    ATATTTAATTTTTGGAGCATGATCAGGTATATTAGGATTAAGAATAAGAATATTAATTCG  60

Query  67   TATAGAATTAGGACAACCTGGAACTTTAATTGGAAATGATCAGATTTATAATGTAATCGT  126
             ||||||||||||||||||||||||||||||||||||||||| |||||||||||||| ||
Sbjct  61   AATAGAATTAGGACAACCTGGAACTTTAATTGGAAATGATCAAATTTATAATGTAATTGT  120

Query  127  AACAGCACATGCATTTATTATAATTTTCTTTATAGTTATACCAATCATAATCGGAGGGTT  186
            |||||| |||||||||||||||||||| |||||||| |||||||||||||| ||||| ||
Sbjct  121  AACAGCTCATGCATTTATTATAATTTTTTTTATAGTAATACCAATCATAATTGGAGGATT  180

Query  187  CGGAAATTGATTAGTTCCTATTATATTAGGTGCCCCAGATATAGCATTTCCACGAATAAA  246
             ||||| || |||||||| ||||||||||| || ||||| ||||||||||||||||||||
Sbjct  181  TGGAAACTGGTTAGTTCCAATTATATTAGGAGCTCCAGACATAGCATTTCCACGAATAAA  240

Query  247  TAATATAAGATTTTGACTTTTACCCCCCTCATTATTTTTATTGATTAACTCTTCATTAAT  306
            |||||||||||||||| |  | |||||||||||||| ||||| ||||| |||||| | ||
Sbjct  241  TAATATAAGATTTTGATTACTTCCCCCCTCATTATTCTTATTAATTAATTCTTCACTGAT  300

Query  307  TGAATCCGGAGCTGGAACAGGATGAACAGTTTATCCCCCTCTATCTTCAAATTTATCACA  366
            |||||| || || || ||||| ||||| |||||||| ||| |||| ||||||||||| ||
Sbjct  301  TGAATCTGGGGCAGGGACAGGGTGAACTGTTTATCCTCCTTTATCCTCAAATTTATCCCA  360

Query  367  TTATGGTCCATCAGTAGATTTAGCTATTTTTTCTCTTCACCTTGCTGGTGCTTCTTCAAT  426
            |||||| |||||||||||||||||||| || |||||||| ||||||||||||||||||||
Sbjct  361  TTATGGACCATCAGTAGATTTAGCTATCTTCTCTCTTCATCTTGCTGGTGCTTCTTCAAT  420

Query  427  TTTAGGTGCAATTAACTTCATCACAACTATTGTAAATATACGATCAATTGGAATAACAAT  486
            |||||| |||||||| ||||| |||||||||||||| |||||||| ||||||||||||||
Sbjct  421  TTTAGGCGCAATTAATTTCATTACAACTATTGTAAACATACGATCTATTGGAATAACAAT  480

Query  487  AGAACGAATACCATTATTTGTTTGATCTGTTTTAATTACAGCTATTTTACTACTTCTATC  546
            |||||||||||||||||| ||||||||||||||||| || ||||||||| | ||||||||
Sbjct  481  AGAACGAATACCATTATTCGTTTGATCTGTTTTAATCACCGCTATTTTATTGCTTCTATC  540
```

```
Query  547   TTTACCTGTATTAGCAGGTGCCATTACAATATTATTAACTGATCGAAACTTTAACACATC  606
             ||||||||| ||||||||||||||||||||| |||||||||||||||| |||||||| ||
Sbjct  541   TTTACCTGTTTTAGCAGGTGCCATTACAATACTATTAACTGATCGAAATTTTAACACTTC  600

Query  607   ATTTTTTGATCCTTCAGGAGGAGGAGATCCCATTTTATATCAACACTTATTT  658
             ||||||||| |||||||||||||| || || |||||||||||||| ||||||
Sbjct  601   ATTTTTTGACCCTTCAGGAGGAGGTGACCCAATTTTATATCAACATTTATTT  652
```

>Rhipicephalus sanguineus isolate Gilan-e Gharb cytochrome oxidase subunit 1 gene, complete cds; mitochondrial
Sequence ID: KM494916.1 Length: 1539
Range 1: 45 to 702

Score:872 bits(472), Expect:0.0,
Identities:596/658(91%), Gaps:0/658(0%), Strand: Plus/Plus

```
Query  1     AACAATATATTTAATTTTTGGAGCATGATCAGGTATATTAGGACTTAGTATAAGAATATT  60
             |||||||||||||||||||||||||||||| || ||||||||| | || ||||||||| |
Sbjct  45    AACAATATATTTAATTTTTGGAGCATGATCCGGAATATTAGGATTAAGAATAAGAATACT  104

Query  61    AATTCGTATAGAATTAGGACAACCTGGAACTTTAATTGGAAATGATCAGATTTATAATGT  120
             |||||||||||||||||| |||||||||||| ||||||| |||||||| |||||||||||
Sbjct  105   AATTCGTATAGAATTAGGTCAACCTGGAACTCTAATTGGTAATGATCAAATTTATAATGT  164

Query  121   AATCGTAACAGCACATGCATTTATTATAATTTTCTTTATAGTTATACCAATCATAATCGG  180
             ||| || ||||| |||||||||||||||||||| |||||||| |||||||| ||||| ||
Sbjct  165   AATTGTTACAGCTCATGCATTTATTATAATTTTTTTTATAGTAATACCAATTATAATTGG  224

Query  181   AGGGTTCGGAAATTGATTAGTTCCTATTATATTAGGTGCCCCAGATATAGCATTTCCACG  240
              || || ||||| |||||||| ||||||||| |||| || |||||||||||||| |||||
Sbjct  225   TGGATTTGGAAACTGATTAGTACCTATTATACTAGGAGCTCCAGATATAGCATTCCCACG  284

Query  241   AATAAATAATATAAGATTTTGACTTTTACCCCCCTCATTATTTTTATTGATTAACTCTTC  300
             ||||||||||||||||||||||||| | || |||||||||||| |||| ||||| |||||
Sbjct  285   AATAAATAATATAAGATTTTGACTTCTTCCTCCCTCATTATTTATATTAATTAATTCTTC  344

Query  301   ATTAATTGAATCCGGAGCTGGAACAGGATGAACAGTTTATCCCCCTCTATCTTCAAATTT  360
             ||||||||| || ||||| || |||||||||||||||||||| || ||||| ||||||||
Sbjct  345   ATTAATTGAGTCAGGAGCAGGTACAGGATGAACAGTTTATCCTCCCCTATCCTCAAATTT  404

Query  361   ATCACATTATGGTCCATCAGTAGATTTAGCTATTTTTTCTCTTCACCTTGCTGGTGCTTC  420
             |||||||||||| |||||||||||||||||||||||||||||||| ||||||||||||||
Sbjct  405   ATCACATTATGGGCCATCAGTAGATTTAGCTATTTTTTCTCTTCATCTTGCTGGTGCTTC  464

Query  421   TTCAATTTTAGGTGCAATTAACTTCATCACAACTATTGTAAATATACGATCAATTGGAAT  480
             ||||||||||||||||||||| || || ||||||||||| ||||||||||| ||||||||
Sbjct  465   TTCAATTTTAGGTGCAATTAATTTTATTACAACTATTGTGAATATACGATCTATTGGAAT  524

Query  481   AACAATAGAACGAATACCATTATTTGTTTGATCTGTTTTAATTACAGCTATTTTACTACT  540
             ||||||||||||||||||||||||||| ||||||||||||||||| || |||||| ||||
Sbjct  525   AACAATAGAACGAATACCATTATTTGTATGATCTGTTTTAATTACTGCAATTTTATTACT  584
```

```
Query  541  TCTATCTTTACCTGTATTAGCAGGTGCCATTACAATATTATTAACTGATCGAAACTTTAA  600
              ||||||||||||| ||||||||||| ||||||||| ||||||| |||||||| |||||
Sbjct  585  ATTATCTTTACCTGTTTTAGCAGGTGCTATTACAATACTATTAACCGATCGAAATTTTAA  644

Query  601  CACATCATTTTTTGATCCTTCAGGAGGAGGAGATCCCATTTTATATCAACACTTATTT  658
            ||| ||||||||||| |||||||||||||| ||||| |||||||||||||| ||||||
Sbjct  645  CACTTCATTTTTTGACCCTTCAGGAGGAGGGGATCCAATTTTATATCAACATTTATTT  702
```

>Rhipicephalus sanguineus isolate Tehran cytochrome oxidase subunit 1 gene, complete cds; mitochondrial
Sequence ID: KM494915.1 Length: 1539
Range 1: 45 to 702

Score:872 bits(472), Expect:0.0,
Identities:596/658(91%), Gaps:0/658(0%), Strand: Plus/Plus

```
Query  1    AACAATATATTTAATTTTTGGAGCATGATCAGGTATATTAGGACTTAGTATAAGAATATT  60
            |||||||||||||||||||||||||||||| || ||||||||| | || ||||||||| |
Sbjct  45   AACAATATATTTAATTTTTGGAGCATGATCCGGAATATTAGGATTAAGAATAAGAATACT  104

Query  61   AATTCGTATAGAATTAGGACAACCTGGAACTTTAATTGGAAATGATCAGATTTATAATGT  120
            |||||||||||||||||| |||||||||||| ||||||| |||||||| |||||||||||
Sbjct  105  AATTCGTATAGAATTAGGTCAACCTGGAACTCTAATTGGTAATGATCAAATTTATAATGT  164

Query  121  AATCGTAACAGCACATGCATTTATTATAATTTTCTTTATAGTTATACCAATCATAATCGG  180
            ||| || ||||| |||||||||||||||||||| |||||||| |||||||| ||||| ||
Sbjct  165  AATTGTTACAGCTCATGCATTTATTATAATTTTTTTTATAGTAATACCAATTATAATTGG  224

Query  181  AGGGTTCGGAAATTGATTAGTTCCTATTATATTAGGTGCCCCAGATATAGCATTTCCACG  240
             || || ||||| |||||||| ||||||||| |||| || |||||||||||||| |||||
Sbjct  225  TGGATTTGGAAACTGATTAGTACCTATTATACTAGGAGCTCCAGATATAGCATTCCCACG  284

Query  241  AATAAATAATATAAGATTTTGACTTTTACCCCCCTCATTATTTTTATTGATTAACTCTTC  300
            ||||||||||||||||||||||||| | || |||||||||||| |||| ||||| |||||
Sbjct  285  AATAAATAATATAAGATTTTGACTTCTTCCTCCCTCATTATTTATATTAATTAATTCTTC  344

Query  301  ATTAATTGAATCCGGAGCTGGAACAGGATGAACAGTTTATCCCCCTCTATCTTCAAATTT  360
            ||||||||| || ||||| || |||||||||||||||||||| || ||||| ||||||||
Sbjct  345  ATTAATTGAGTCAGGAGCAGGTACAGGATGAACAGTTTATCCTCCCCTATCCTCAAATTT  404

Query  361  ATCACATTATGGTCCATCAGTAGATTTAGCTATTTTTTCTCTTCACCTTGCTGGTGCTTC  420
            |||||||||||| |||||||||||||||||||||||||||||||| ||||||||||||||
Sbjct  405  ATCACATTATGGGCCATCAGTAGATTTAGCTATTTTTTCTCTTCATCTTGCTGGTGCTTC  464

Query  421  TTCAATTTTAGGTGCAATTAACTTCATCACAACTATTGTAAATATACGATCAATTGGAAT  480
            ||||||||||||||||||||| || || ||||||||||| ||||||||||| ||||||||
Sbjct  465  TTCAATTTTAGGTGCAATTAATTTTATTACAACTATTGTGAATATACGATCTATTGGAAT  524

Query  481  AACAATAGAACGAATACCATTATTTGTTTGATCTGTTTTAATTACAGCTATTTTACTACT  540
            ||||||||||||||||||||||||||| ||||||||||||||||| || |||||| ||||
```

```
Sbjct  525  AACAATAGAACGAATACCATTATTTGTATGATCTGTTTTAATTACTGCAATTTTATTACT  584

Query  541  TCTATCTTTACCTGTATTAGCAGGTGCCATTACAATATTATTAACTGATCGAAACTTTAA  600
              ||||||||||||| ||||||||||| ||||||||| ||||||| |||||||| |||||
Sbjct  585  ATTATCTTTACCTGTTTTAGCAGGTGCTATTACAATACTATTAACCGATCGAAATTTTAA  644

Query  601  CACATCATTTTTTGATCCTTCAGGAGGAGGAGATCCCATTTTATATCAACACTTATTT  658
            ||| ||||||||||| |||||||||||||| ||||| |||||||||||||| ||||||
Sbjct  645  CACTTCATTTTTTGACCCTTCAGGAGGAGGGGATCCAATTTTATATCAACATTTATTT  702
```

>Rhipicephalus turanicus isolate COX1-18-Sheep cytochrome c oxidase subunit I (COX1) gene, partial cds; mitochondrial
Sequence ID: MT800314.1 Length: 673
Range 1: 8 to 664

Score:870 bits(471), Expect:0.0,
Identities:595/657(91%), Gaps:0/657(0%), Strand: Plus/Plus

```
Query  1    AACAATATATTTAATTTTTGGAGCATGATCAGGTATATTAGGACTTAGTATAAGAATATT  60
            ||||||||||||||||||||| ||||||||||| ||||||||||| || ||||||||| |
Sbjct  8    AACAATATATTTAATTTTTGGGGCATGATCAGGAATATTAGGACTGAGAATAAGAATACT  67

Query  61   AATTCGTATAGAATTAGGACAACCTGGAACTTTAATTGGAAATGATCAGATTTATAATGT  120
            |||||| ||||||||||||||||||||||||||||||||||||||||| |||||||| ||
Sbjct  68   AATTCGCATAGAATTAGGACAACCTGGAACTTTAATTGGAAATGATCAAATTTATAACGT  127

Query  121  AATCGTAACAGCACATGCATTTATTATAATTTTCTTTATAGTTATACCAATCATAATCGG  180
            ||| |||||||| |||||||||||||||||||| ||||| || ||||| || ||||| ||
Sbjct  128  AATTGTAACAGCTCATGCATTTATTATAATTTTTTTTATGGTAATACCCATTATAATTGG  187

Query  181  AGGGTTCGGAAATTGATTAGTTCCTATTATATTAGGTGCCCCAGATATAGCATTTCCACG  240
             || || ||||||||||||||||||||||||||||| || |||||||||||||| |||||
Sbjct  188  TGGATTTGGAAATTGATTAGTTCCTATTATATTAGGGGCTCCAGATATAGCATTCCCACG  247

Query  241  AATAAATAATATAAGATTTTGACTTTTACCCCCCTCATTATTTTTATTGATTAACTCTTC  300
             |||||||| |||||||||||| |  | || || ||||||||| |||| |||||||| ||
Sbjct  248  TATAAATAACATAAGATTTTGATTACTTCCTCCTTCATTATTTCTATTAATTAACTCCTC  307

Query  301  ATTAATTGAATCCGGAGCTGGAACAGGATGAACAGTTTATCCCCCTCTATCTTCAAATTT  360
            |||||||||||| ||||| |||||||||||||| |||||||||||| |||| ||||||||
Sbjct  308  ATTAATTGAATCGGGAGCAGGAACAGGATGAACTGTTTATCCCCCTTTATCCTCAAATTT  367

Query  361  ATCACATTATGGTCCATCAGTAGATTTAGCTATTTTTTCTCTTCACCTTGCTGGTGCTTC  420
            |||||||||||| |||||||||||||||||||||||||||||||| ||||||||||||||
Sbjct  368  ATCACATTATGGACCATCAGTAGATTTAGCTATTTTTTCTCTTCATCTTGCTGGTGCTTC  427

Query  421  TTCAATTTTAGGTGCAATTAACTTCATCACAACTATTGTAAATATACGATCAATTGGAAT  480
            ||||||| ||||||||||||| || || |||||||||||||| |||||||| ||||||||
Sbjct  428  TTCAATTCTAGGTGCAATTAATTTTATTACAACTATTGTAAACATACGATCTATTGGAAT  487

Query  481  AACAATAGAACGAATACCATTATTTGTTTGATCTGTTTTAATTACAGCTATTTTACTACT  540
```

```
            |||||||||||||||||||||||||||||||||||||||||||||| || || ||||| ||
Sbjct  488  AACAATAGAACGAATACCATTATTTGTTTGATCTGTTTTAATTACCGCCATCTTACTTCT  547

Query  541  TCTATCTTTACCTGTATTAGCAGGTGCCATTACAATATTATTAACTGATCGAAACTTTAA  600
              ||||||||||||| ||||||||||| |||||||||||||||||||||||||| || ||
Sbjct  548  CTTATCTTTACCTGTTTTAGCAGGTGCTATTACAATATTATTAACTGATCGAAATTTCAA  607

Query  601  CACATCATTTTTTGATCCTTCAGGAGGAGGAGATCCCATTTTATATCAACACTTATT  657
             || |||||||||||||||||||| ||||| ||||| |||||||| |||||||||||
Sbjct  608  TACTTCATTTTTTGATCCTTCAGGGGGAGGTGATCCAATTTTATACCAACACTTATT  664
```

>Rhipicephalus turanicus isolate 31X cytochrome oxidase subunit 1 (COI) gene, partial cds; mitochondrial
Sequence ID: KY606303.1 Length: 873
Range 1: 66 to 723

Score:861 bits(466), Expect:0.0,
Identities:594/658(90%), Gaps:0/658(0%), Strand: Plus/Plus

```
Query  1    AACAATATATTTAATTTTTGGAGCATGATCAGGTATATTAGGACTTAGTATAAGAATATT  60
            |||||||||||||||||||||||||||||| || ||||||||| | || |||||||||||
Sbjct  66   AACAATATATTTAATTTTTGGAGCATGATCCGGGATATTAGGATTAAGAATAAGAATATT  125

Query  61   AATTCGTATAGAATTAGGACAACCTGGAACTTTAATTGGAAATGATCAGATTTATAATGT  120
            |||||| ||||| ||||||||||||||||||||||||||||||||||| |||||||||||
Sbjct  126  AATTCGAATAGAGTTAGGACAACCTGGAACTTTAATTGGAAATGATCAAATTTATAATGT  185

Query  121  AATCGTAACAGCACATGCATTTATTATAATTTTCTTTATAGTTATACCAATCATAATCGG  180
            ||| |||||||| |||||||||||||||||||| |||||||| |||||||||||||| ||
Sbjct  186  AATTGTAACAGCTCATGCATTTATTATAATTTTTTTTATAGTAATACCAATCATAATTGG  245

Query  181  AGGGTTCGGAAATTGATTAGTTCCTATTATATTAGGTGCCCCAGATATAGCATTTCCACG  240
            ||| || ||||| |||||||| || ||||| ||||| || ||||| ||||||||||||||
Sbjct  246  AGGATTTGGAAACTGATTAGTGCCCATTATGTTAGGAGCTCCAGACATAGCATTTCCACG  305

Query  241  AATAAATAATATAAGATTTTGACTTTTACCCCCCTCATTATTTTTATTGATTAACTCTTC  300
            |||||||||||||||||||||| |  | || ||||||||||| ||||| ||||| |||||
Sbjct  306  AATAAATAATATAAGATTTTGATTACTTCCTCCCTCATTATTCTTATTAATTAATTCTTC  365

Query  301  ATTAATTGAATCCGGAGCTGGAACAGGATGAACAGTTTATCCCCCTCTATCTTCAAATTT  360
            | | |||||||| ||||| || ||||||||||| || || || ||| |||||||||||||
Sbjct  366  ACTGATTGAATCTGGAGCAGGGACAGGATGAACTGTCTACCCTCCTTTATCTTCAAATTT  425

Query  361  ATCACATTATGGTCCATCAGTAGATTTAGCTATTTTTTCTCTTCACCTTGCTGGTGCTTC  420
            ||| |||||||| |||||||||||||||||||| || |||||||| || |||||||||||
Sbjct  426  ATCGCATTATGGACCATCAGTAGATTTAGCTATCTTCTCTCTTCATCTCGCTGGTGCTTC  485

Query  421  TTCAATTTTAGGTGCAATTAACTTCATCACAACTATTGTAAATATACGATCAATTGGAAT  480
            ||||||||||||||||||||| ||||| |||||||||||||| |||||||| ||||||||
Sbjct  486  TTCAATTTTAGGTGCAATTAATTTCATTACAACTATTGTAAACATACGATCTATTGGAAT  545
```

```
Query  481   AACAATAGAACGAATACCATTATTTGTTTGATCTGTTTTAATTACAGCTATTTTACTACT  540
             |||||||||||||||||||||||| ||||||||||||||||| || ||||||||| | ||
Sbjct  546   AACAATAGAACGAATACCATTATTCGTTTGATCTGTTTTAATCACCGCTATTTTATTGCT  605

Query  541   TCTATCTTTACCTGTATTAGCAGGTGCCATTACAATATTATTAACTGATCGAAACTTTAA  600
             ||||||||||||||| |||||||||||||||||||||||||||||||||||||| || ||
Sbjct  606   TCTATCTTTACCTGTTTTAGCAGGTGCCATTACAATATTATTAACTGATCGAAATTTCAA  665

Query  601   CACATCATTTTTTGATCCTTCAGGAGGAGGAGATCCCATTTTATATCAACACTTATTT  658
             ||| ||||||||||| |||||||||||||| || || |||||||||||||| ||||||
Sbjct  666   CACTTCATTTTTTGACCCTTCAGGAGGAGGTGACCCTATTTTATATCAACATTTATTT  723
```

>Rhipicephalus turanicus isolate 19X cytochrome oxidase subunit 1 (COI) gene, partial cds; mitochondrial
Sequence ID: KY606302.1 Length: 873
Range 1: 66 to 723

Score:861 bits(466), Expect:0.0,
Identities:594/658(90%), Gaps:0/658(0%), Strand: Plus/Plus

```
Query  1     AACAATATATTTAATTTTTGGAGCATGATCAGGTATATTAGGACTTAGTATAAGAATATT  60
             |||||||||||||||||||||||||||||| || ||||||||| | || |||||||||||
Sbjct  66    AACAATATATTTAATTTTTGGAGCATGATCCGGGATATTAGGATTAAGAATAAGAATATT  125

Query  61    AATTCGTATAGAATTAGGACAACCTGGAACTTTAATTGGAAATGATCAGATTTATAATGT  120
             |||||| ||||| ||||||||||||||||||||||||||||||||||| |||||||||||
Sbjct  126   AATTCGAATAGAGTTAGGACAACCTGGAACTTTAATTGGAAATGATCAAATTTATAATGT  185

Query  121   AATCGTAACAGCACATGCATTTATTATAATTTTCTTTATAGTTATACCAATCATAATCGG  180
             ||| |||||||| |||||||||||||||||||| |||||||| |||||||||||||| ||
Sbjct  186   AATTGTAACAGCTCATGCATTTATTATAATTTTTTTTATAGTAATACCAATCATAATTGG  245

Query  181   AGGGTTCGGAAATTGATTAGTTCCTATTATATTAGGTGCCCCAGATATAGCATTTCCACG  240
             ||| || ||||| |||||||| || ||||| ||||| || ||||| ||||||||||||||
Sbjct  246   AGGATTTGGAAACTGATTAGTGCCCATTATGTTAGGAGCTCCAGACATAGCATTTCCACG  305

Query  241   AATAAATAATATAAGATTTTGACTTTTACCCCCCTCATTATTTTTATTGATTAACTCTTC  300
             |||||||||||||||||||||| |  | || ||||||||||| ||||| ||||| |||||
Sbjct  306   AATAAATAATATAAGATTTTGATTACTTCCTCCCTCATTATTCTTATTAATTAATTCTTC  365

Query  301   ATTAATTGAATCCGGAGCTGGAACAGGATGAACAGTTTATCCCCCTCTATCTTCAAATTT  360
             | | |||||||| ||||| || ||||||||||| || || || ||| |||||||||||||
Sbjct  366   ACTGATTGAATCTGGAGCAGGGACAGGATGAACTGTCTACCCTCCTTTATCTTCAAATTT  425

Query  361   ATCACATTATGGTCCATCAGTAGATTTAGCTATTTTTTCTCTTCACCTTGCTGGTGCTTC  420
             ||| |||||||| |||||||||||||||||||| || |||||||| || |||||||||||
Sbjct  426   ATCGCATTATGGACCATCAGTAGATTTAGCTATCTTCTCTCTTCATCTCGCTGGTGCTTC  485

Query  421   TTCAATTTTAGGTGCAATTAACTTCATCACAACTATTGTAAATATACGATCAATTGGAAT  480
             ||||||||||||||||||||| ||||| |||||||||||||| |||||||| ||||||||
Sbjct  486   TTCAATTTTAGGTGCAATTAATTTCATTACAACTATTGTAAACATACGATCTATTGGAAT  545
```

```
Query  481   AACAATAGAACGAATACCATTATTTGTTTGATCTGTTTTAATTACAGCTATTTTACTACT  540
             |||||||||||||||||||||||| ||||||||||||||||| || ||||||||| | ||
Sbjct  546   AACAATAGAACGAATACCATTATTCGTTTGATCTGTTTTAATCACCGCTATTTTATTGCT  605

Query  541   TCTATCTTTACCTGTATTAGCAGGTGCCATTACAATATTATTAACTGATCGAAACTTTAA  600
             ||||||||||||||| |||||||||||||||||||||||||||||||||||||| || ||
Sbjct  606   TCTATCTTTACCTGTTTTAGCAGGTGCCATTACAATATTATTAACTGATCGAAATTTCAA  665

Query  601   CACATCATTTTTTGATCCTTCAGGAGGAGGAGATCCCATTTTATATCAACACTTATTT  658
             ||| ||||||||||| |||||||||||||| || || |||||||||||||| ||||||
Sbjct  666   CACTTCATTTTTTGACCCTTCAGGAGGAGGTGACCCTATTTTATATCAACATTTATTT  723
```

>Rhipicephalus turanicus isolate 19C cytochrome oxidase subunit 1 (COI) gene, partial cds; mitochondrial
Sequence ID: KY606301.1 Length: 873
Range 1: 66 to 723

Score:861 bits(466), Expect:0.0,
Identities:594/658(90%), Gaps:0/658(0%), Strand: Plus/Plus

```
Query  1     AACAATATATTTAATTTTTGGAGCATGATCAGGTATATTAGGACTTAGTATAAGAATATT  60
             |||||||||||||||||||||||||||||| || ||||||||| | || |||||||||||
Sbjct  66    AACAATATATTTAATTTTTGGAGCATGATCCGGGATATTAGGATTAAGAATAAGAATATT  125

Query  61    AATTCGTATAGAATTAGGACAACCTGGAACTTTAATTGGAAATGATCAGATTTATAATGT  120
             |||||| ||||| ||||||||||||||||||||||||||||||||||| |||||||||||
Sbjct  126   AATTCGAATAGAGTTAGGACAACCTGGAACTTTAATTGGAAATGATCAAATTTATAATGT  185

Query  121   AATCGTAACAGCACATGCATTTATTATAATTTTCTTTATAGTTATACCAATCATAATCGG  180
             ||| |||||||| |||||||||||||||||||| |||||||| |||||||||||||| ||
Sbjct  186   AATTGTAACAGCTCATGCATTTATTATAATTTTTTTTATAGTAATACCAATCATAATTGG  245

Query  181   AGGGTTCGGAAATTGATTAGTTCCTATTATATTAGGTGCCCCAGATATAGCATTTCCACG  240
             ||| || ||||| |||||||| || ||||| ||||| || ||||| ||||||||||||||
Sbjct  246   AGGATTTGGAAACTGATTAGTGCCCATTATGTTAGGAGCTCCAGACATAGCATTTCCACG  305

Query  241   AATAAATAATATAAGATTTTGACTTTTACCCCCCTCATTATTTTTATTGATTAACTCTTC  300
             |||||||||||||||||||||| |  | || ||||||||||| ||||| ||||| |||||
Sbjct  306   AATAAATAATATAAGATTTTGATTACTTCCTCCCTCATTATTCTTATTAATTAATTCTTC  365

Query  301   ATTAATTGAATCCGGAGCTGGAACAGGATGAACAGTTTATCCCCCTCTATCTTCAAATTT  360
             | | |||||||| ||||| || ||||||||||| || || || ||| |||||||||||||
Sbjct  366   ACTGATTGAATCTGGAGCAGGGACAGGATGAACTGTCTACCCTCCTTTATCTTCAAATTT  425

Query  361   ATCACATTATGGTCCATCAGTAGATTTAGCTATTTTTTCTCTTCACCTTGCTGGTGCTTC  420
             ||| |||||||| |||||||||||||||||||| || |||||||| || |||||||||||
Sbjct  426   ATCGCATTATGGACCATCAGTAGATTTAGCTATCTTCTCTCTTCATCTCGCTGGTGCTTC  485

Query  421   TTCAATTTTAGGTGCAATTAACTTCATCACAACTATTGTAAATATACGATCAATTGGAAT  480
             ||||||||||||||||||||| ||||| |||||||||||||| |||||||| ||||||||
```

```
Sbjct  486   TTCAATTTTAGGTGCAATTAATTTCATTACAACTATTGTAAACATACGATCTATTGGAAT  545
```
[truncated: 540,440 more chars]
