# Supplementary material for: Possible Association between Selected Tick-Borne Pathogen Prevalence and Rhipicephalus sanguineus sensu lato Infestation in Dogs from Juarez City (Chihuahua), Northwest Mexico–US Border
Source: Pathogens. 2022 May 7;11(5):552. doi: 10.3390/pathogens11050552 (PMC9145599; doi:10.3390/pathogens11050552)
Supplement: Supplementary file 1 [file pathogens-11-00552-s001.zip › pathogens-1665365-supplementary/Supplementary material (not include Table S1).pdf]

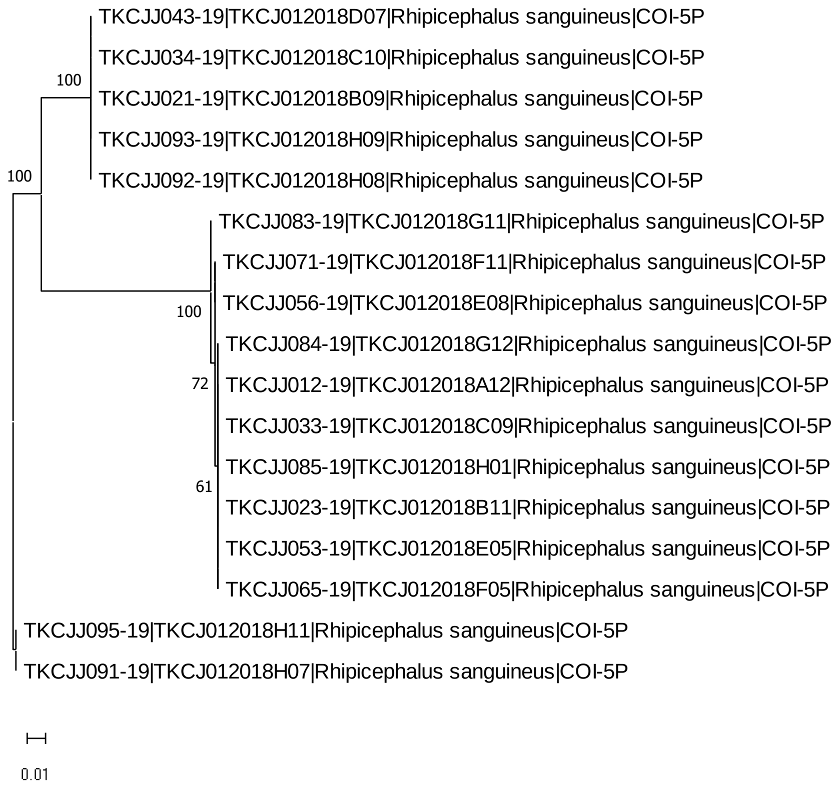

Figure S1. Distance genetic analyses Tree R. sanguineus. The evolutionary history was inferred using the Neighbor-Joining method. The optimal tree is shown. The percentage of replicate trees in which the associated taxa clustered together in the bootstrap test (1000 replicates) are shown next to the branches. The tree is drawn to scale, with branch lengths in the same units as those of the evolutionary distances used to infer the phylogenetic tree. The evolutionary distances were computed using the Kimura 2-parameter method and are in the units of the number of base substitutions per site. This analysis involved 17 nucleotide sequences. All ambiguous positions were removed for each sequence pair (pairwise deletion option). There were a total of 658 positions in the final dataset. Evolutionary analyses were conducted in MEGA11. Overal Mean distance: 0.06 +/- 0.01

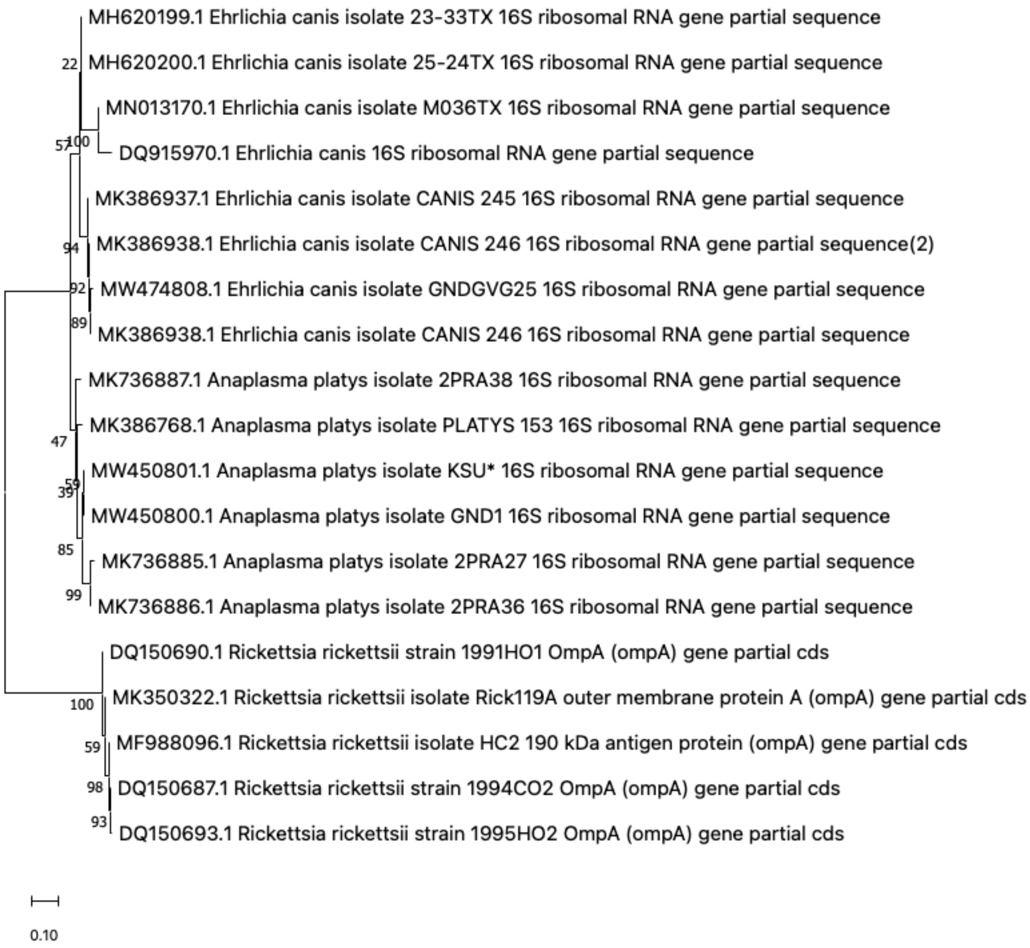
MH620199.1 Ehrlichia canis isolate 23-33TX 16S ribosomal RNA gene partial sequence
MH620200.1 Ehrlichia canis isolate 25-24TX 16S ribosomal RNA gene partial sequence
MN013170.1 Ehrlichia canis isolate M036TX 16S ribosomal RNA gene partial sequence
DQ915970.1 Ehrlichia canis 16S ribosomal RNA gene partial sequence
MK386937.1 Ehrlichia canis isolate CANIS 245 16S ribosomal RNA gene partial sequence
MK386938.1 Ehrlichia canis isolate CANIS 246 16S ribosomal RNA gene partial sequence(2)
MW474808.1 Ehrlichia canis isolate GNDGVG25 16S ribosomal RNA gene partial sequence
MK386938.1 Ehrlichia canis isolate CANIS 246 16S ribosomal RNA gene partial sequence
MK736887.1 Anaplasma platys isolate 2PRA38 16S ribosomal RNA gene partial sequence
MK386768.1 Anaplasma platys isolate PLATYS 153 16S ribosomal RNA gene partial sequence
MW450801.1 Anaplasma platys isolate KSU* 16S ribosomal RNA gene partial sequence
MW450800.1 Anaplasma platys isolate GND1 16S ribosomal RNA gene partial sequence
MK736885.1 Anaplasma platys isolate 2PRA27 16S ribosomal RNA gene partial sequence
MK736886.1 Anaplasma platys isolate 2PRA36 16S ribosomal RNA gene partial sequence
DQ150690.1 Rickettsia rickettsii strain 1991HO1 OmpA (ompA) gene partial cds
MK350322.1 Rickettsia rickettsii isolate Rick119A outer membrane protein A (ompA) gene partial cds
MF988096.1 Rickettsia rickettsii isolate HC2 190 kDa antigen protein (ompA) gene partial cds
DQ150687.1 Rickettsia rickettsii strain 1994CO2 OmpA (ompA) gene partial cds
DQ150693.1 Rickettsia rickettsii strain 1995HO2 OmpA (ompA) gene partial cds
22
57
100
94
92
89
47
59
39
85
99
100
59
98
93
0.10

[1] #MH620199.1_Ehrlichia_canis_isolate_23-33TX_16S_ribosomal_RNA_gene_partial_sequence
[2] #MH620200.1_Ehrlichia_canis_isolate_25-24TX_16S_ribosomal_RNA_gene_partial_sequence
[3] #MN013170.1_Ehrlichia_canis_isolate_M036TX_16S_ribosomal_RNA_gene_partial_sequence
[4] #MW474808.1_Ehrlichia_canis_isolate_GNDGVG25_16S_ribosomal_RNA_gene_partial_sequence
[5] #DQ915970.1_Ehrlichia_canis_16S_ribosomal_RNA_gene_partial_sequence
[6] #MK386937.1_Ehrlichia_canis_isolate_CANIS_245_16S_ribosomal_RNA_gene_partial_sequence
[7] #MK386938.1_Ehrlichia_canis_isolate_CANIS_246_16S_ribosomal_RNA_gene_partial_sequence(2)
[8] #MK386938.1_Ehrlichia_canis_isolate_CANIS_246_16S_ribosomal_RNA_gene_partial_sequence
[9] #MW450801.1_Anaplasma_platys_isolate_KSU*_16S_ribosomal_RNA_gene_partial_sequence
[10] #MW450800.1_Anaplasma_platys_isolate_GND1_16S_ribosomal_RNA_gene_partial_sequence
[11] #MK736885.1_Anaplasma_platys_isolate_2PRA27_16S_ribosomal_RNA_gene_partial_sequence
[12] #MK736886.1_Anaplasma_platys_isolate_2PRA36_16S_ribosomal_RNA_gene_partial_sequence
[13] #MK736887.1_Anaplasma_platys_isolate_2PRA38_16S_ribosomal_RNA_gene_partial_sequence
[14] #MK386768.1_Anaplasma_platys_isolate_PLATYS_153_16S_ribosomal_RNA_gene_partial_sequence
[15] #DQ150690.1_Rickettsia_rickettsii_strain_1991HO1_OmpA_(ompA)_gene_partial_cds
[16] #MF988096.1_Rickettsia_rickettsii_isolate_HC2_190_kDa_antigen_protein_(ompA)_gene_partial_cds
[17] #DQ150687.1_Rickettsia_rickettsii_strain_1994CO2_OmpA_(ompA)_gene_partial_cds
[18] #DQ150693.1_Rickettsia_rickettsii_strain_1995HO2_OmpA_(ompA)_gene_partial_cds
[19] #MK350322.1_Rickettsia_rickettsii_isolate_Rick119A_outer_membrane_protein_A_(ompA)_gene_partial_cds

[	1	2	3	4	5	6	7	8	9	10	11	12	13	14	15	16	17	18	19]
[1]																			
[2]	0.0000																		
[3]	0.0317	0.0317																	
[4]	0.0515	0.0515	0.0766																
[5]	0.1272	0.1272	0.0246	0.2306															
[6]	0.0328	0.0328	0.0724	0.0034	0.1536														
[7]	0.0327	0.0327	0.0724	0.0034	0.1579	0.0000													
[8]	0.0327	0.0327	0.0724	0.0034	0.1579	0.0000	0.0000												
[9]	0.0863	0.0863	0.0848	0.1606	0.1191	0.1291	0.1297	0.1297											
[10]	0.0899	0.0899	0.0879	0.1606	0.1223	0.1291	0.1297	0.1297	0.0026										
[11]	0.1823	0.1823	0.1418	0.1276	0.2191	0.1263	0.1268	0.1268	0.0645	0.0645									
[12]	0.1296	0.1296	0.1144	0.1609	0.1434	0.1563	0.1563	0.1563	0.0116	0.0116	0.0063								
[13]	0.1677	0.1677	0.1349	0.0699	0.2527	0.0544	0.0542	0.0542	0.0622	0.0622	0.0645	0.0882							
[14]	0.0820	0.0820	0.0805	0.1752	0.0911	0.1201	0.1206	0.1206	0.0510	0.0476	0.1125	0.0241	0.0473						
[15]	0.5831	0.5831	0.6788	0.6739	0.8036	0.6727	0.6771	0.6771	0.6588	0.6494	0.7222	0.6660	0.6229	0.6770					
[16]	0.6605	0.6605	0.6787	0.7340	0.7764	0.7220	0.7331	0.7331	0.6588	0.6494	0.7377	0.6950	0.7084	0.6270	0.0000				
[17]	0.6605	0.6605	0.6787	0.7340	0.8209	0.7220	0.7331	0.7331	0.6588	0.6494	0.7377	0.6950	0.7084	0.6787	0.0000	0.0000			
[18]	0.6605	0.6605	0.6787	0.7340	0.8864	0.7220	0.7331	0.7331	0.6588	0.6494	0.7377	0.6950	0.7084	0.7410	0.0000	0.0000	0.0000		
[19]	0.6121	0.6121	0.6787	0.6984	0.7095	0.7014	0.7059	0.7059	0.6588	0.6494	0.7386	0.6851	0.6528	0.5996	0.0023	0.0023	0.0023	0.0023	

Table. Estimates of Evolutionary Divergence between Sequences

Figure S2. Evolutionary relationships of taxa. The evolutionary history was inferred using the Neighbor-Joining method. The optimal tree with the sum of branch length = 0.98841046 is shown. The percentage of replicate trees in which the associated taxa clustered together in the bootstrap test (1000 replicates) are shown next to the branches. The tree is drawn to scale, with branch lengths in the same units as those of the evolutionary distances used to infer the phylogenetic tree. The evolutionary distances were computed using the Kimura 2-parameter method and are in the units of the number of base substitutions per site. This analysis involved 19 nucleotide sequences. Codon positions included were 1st+2nd+3rd+Noncoding. All ambiguous positions were removed for each sequence pair (pairwise deletion option). There were a total of 1434 positions in the final dataset. Evolutionary analyses were conducted in MEGA X.

Table S2. Regression analy

Effect of tick number, dog breed, dog age, infested site, dog sex on E. canis infection in individual dogs (infected and not): logistic regresion									
Efect	Breed	Anatomic site	STATUS	SEX	Estimator	Standard	DF	T value	Pr > ILI
Intercept					-0.5602	0.473	138	-1.18	0.283
Breed	Siberian Husky				0.1121	0.3527	138	0.32	0.7511
Breed	Labrador				0.1699	0.4223	138	0.4	0.6881
Breed	Mixed				-0.0238	0.2017	138	-0.12	0.9062
Breed	A. pitbull				0	-	-	-	-
Anatomic site		Abdomen			-2.7567	3.3401	138	-0.83	0.4106
Anatomic site		Armpit			0.4751	0	138	Infty	<.0001
Anatomic site		Head			0.2184	0	138	Infty	<.0001
Anatomic site		Neck			-0.2411	0.554	138	-0.44	0.6641
Anatomic site		Back			-0.1441	0.5013	138	-0.29	0.7741
Anatomic site		Ears			-0.1088	0.4788	138	-0.23	0.8207
Anatomic site		arms and legs			-0.257	0.6113	138	-0.42	0.6749
Anatomic site		Chest			0	-	-	-	-
Age			puppy		-0.00844	0.3665	138	-0.02	0.9817
Age			adult		0.2889	0.1741	138	1.66	0.0994
Age			old		0	-	-	-	-
Sex				0 female	-0.0079	0.1642	138	-0.05	0.9617
Sex				1 male	0	-	-	-	-
Total female ticks					0.04098	0.08339	138	0.49	0.6239
Total male ticks					-0.1319	0.07866	138	-1.68	0.0959
Engorged female					0.0271	0.09955	138	0.27	0.7858
